# Family-group names in Coleoptera (Insecta)

**DOI:** 10.3897/zookeys.88.807

**Published:** 2011-04-04

**Authors:** Patrice Bouchard, Yves Bousquet, Anthony E. Davies, Miguel A. Alonso-Zarazaga, John F. Lawrence, Chris H. C. Lyal, Alfred F. Newton, Chris A. M. Reid, Michael Schmitt, S. Adam Ślipiński, Andrew B. T. Smith

**Affiliations:** 1 Canadian National Collection of Insects, Arachnids and Nematodes, Agriculture and Agri-Food Canada, 960 Carling Avenue, Ottawa, Ontario, K1A 0C6, Canada; 2 Departamento de Biodiversidad y Biología Evolutiva, Museo Nacional de Ciencias Naturales, Jose Gutierrez Abascal, 2; E-28006, Madrid, Spain; 3 Australian National Insect Collection, CSIRO Entomology, GPO Box 1700, Canberra, ACT 2601, Australia; 4 Department of Entomology, The Natural History Museum, Cromwell Road, London SW7 5BD, United Kingdom; 5 Zoology Department, Field Museum of Natural History, 1400 South Lake Shore Drive Chicago, IL 60605, USA; 6 Australian Museum, 6 College Street, Sydney, NSW 2010, Australia; 7 Ernst-Moritz-Arndt-Universitaet, Allgemeine & Systematische Zoologie, Anklamer Str. 20, D-17489 Greifswald, Germany; 8 Canadian Museum of Nature, P. O. Box 3443, Station D, Ottawa, Ontario, K1P 6P4, Canada

**Keywords:** Beetles, nomenclature, classification, world fauna, family-group names, type genera, stem

## Abstract

We synthesize data on all known extant and fossil Coleoptera family-group names for the first time. A catalogue of 4887 family-group names (124 fossil, 4763 extant) based on 4707 distinct genera in Coleoptera is given. A total of 4492 names are available, 183 of which are permanently invalid because they are based on a preoccupied or a suppressed type genus. Names are listed in a classification framework. We recognize as valid 24 superfamilies, 211 families, 541 subfamilies, 1663 tribes and 740 subtribes. For each name, the original spelling, author, year of publication, page number, correct stem and type genus are included. The original spelling and availability of each name were checked from primary literature. A list of necessary changes due to Priority and Homonymy problems, and actions taken, is given. Current usage of names was conserved, whenever possible, to promote stability of the classification.

New synonymies (family-group names followed by genus-group names): Agronomina Gistel, 1848 syn. nov. of Amarina Zimmermann, 1832 (Carabidae), Hylepnigalioini Gistel, 1856 syn. nov. of Melandryini Leach, 1815 (Melandryidae), Polycystophoridae Gistel, 1856 syn. nov. of Malachiinae Fleming, 1821 (Melyridae), Sclerasteinae Gistel, 1856 syn. nov. of Ptilininae Shuckard, 1839 (Ptinidae), Phloeonomini Ádám, 2001 syn. nov. of Omaliini MacLeay, 1825 (Staphylinidae), Sepedophilini Ádám, 2001 syn. nov. of Tachyporini MacLeay, 1825 (Staphylinidae), Phibalini Gistel, 1856 syn. nov. of Cteniopodini Solier, 1835 (Tenebrionidae); *Agronoma* Gistel 1848 (type species *Carabus familiaris* Duftschmid, 1812, designated herein) syn. nov. of *Amara* Bonelli, 1810 (Carabidae), *Hylepnigalio* Gistel, 1856 (type species *Chrysomela caraboides* Linnaeus, 1760, by monotypy) syn. nov. of *Melandrya* Fabricius, 1801 (Melandryidae), *Polycystophorus* Gistel, 1856 (type species *Cantharis aeneus* Linnaeus, 1758, designated herein) syn. nov. of *Malachius* Fabricius, 1775 (Melyridae), *Sclerastes* Gistel, 1856 (type species *Ptilinus costatus* Gyllenhal, 1827, designated herein) syn. nov. of *Ptilinus* Geoffroy, 1762 (Ptinidae), *Paniscus* Gistel, 1848 (type species *Scarabaeus fasciatus* Linnaeus, 1758, designated herein) syn. nov. of *Trichius* Fabricius, 1775 (Scarabaeidae), *Phibalus* Gistel, 1856 (type species *Chrysomela pubescens* Linnaeus, 1758, by monotypy) syn. nov. of *Omophlus* Dejean, 1834 (Tenebrionidae). The following new replacement name is proposed: Gompeliina Bouchard, 2011 nom. nov. for Olotelina Báguena Corella, 1948 (Aderidae).

Reversal of Precedence (Article 23.9) is used to conserve usage of the following names (family-group names followed by genus-group names): Perigonini Horn, 1881 nom. protectum over Trechicini Bates, 1873 nom. oblitum (Carabidae), Anisodactylina Lacordaire, 1854 nom. protectum over Eurytrichina LeConte, 1848 nom. oblitum (Carabidae), Smicronychini Seidlitz, 1891 nom. protectum over Desmorini LeConte, 1876 nom. oblitum (Curculionidae), Bagoinae Thomson, 1859 nom. protectum over Lyprinae Gistel 1848 nom. oblitum (Curculionidae), Aterpina Lacordaire, 1863 nom. protectum over Heliomenina Gistel, 1848 nom. oblitum (Curculionidae), Naupactini Gistel, 1848 nom. protectum over Iphiini Schönherr, 1823 nom. oblitum (Curculionidae), Cleonini Schönherr, 1826 nom. protectum over Geomorini Schönherr, 1823 nom. oblitum (Curculionidae), Magdalidini Pascoe, 1870 nom. protectum over Scardamyctini Gistel, 1848 nom. oblitum (Curculionidae), Agrypninae/-ini Candèze, 1857 nom. protecta over Adelocerinae/-ini Gistel, 1848 nom. oblita and Pangaurinae/-ini Gistel, 1856 nom. oblita (Elateridae), Prosternini Gistel, 1856 nom. protectum over Diacanthini Gistel, 1848 nom. oblitum (Elateridae), Calopodinae Costa, 1852 nom. protectum over Sparedrinae Gistel, 1848 nom. oblitum (Oedemeridae), Adesmiini Lacordaire, 1859 nom. protectum over Macropodini Agassiz, 1846 nom. oblitum (Tenebrionidae), Bolitophagini Kirby, 1837 nom. protectum over Eledonini Billberg, 1820 nom. oblitum (Tenebrionidae), Throscidae Laporte, 1840 nom. protectum over Stereolidae Rafinesque, 1815 nom. oblitum (Throscidae) and Lophocaterini Crowson, 1964 over Lycoptini Casey, 1890 nom. oblitum (Trogossitidae); *Monotoma* Herbst, 1799 nom. protectum over *Monotoma* Panzer, 1792 nom. oblitum (Monotomidae); *Pediacus* Shuckard, 1839 nom. protectum over *Biophloeus* Dejean, 1835 nom. oblitum (Cucujidae), *Pachypus* Dejean, 1821 nom. protectum over *Pachypus* Billberg, 1820 nom. oblitum (Scarabaeidae), *Sparrmannia* Laporte, 1840 nom. protectum over *Leocaeta* Dejean, 1833 nom. oblitum and *Cephalotrichia* Hope, 1837 nom. oblitum (Scarabaeidae).

*Zoological nomenclature affects the work of all zoologists, yet only a minuscule fraction of one percent of [the craziest] zoologists deal directly with problems associated with scientific names of animals (Bock 1994)*.

## Introduction

The accurate use of scientific names in zoology is necessary to minimize nomenclatural instability and allow the maximal retrieval of scientific information from the ever increasing body of literature. Akin to other scientific names such as species- and genus-group names, family-group names are extremely important in the exchange of information about the world we live in. Rules about how to treat family-group names, as determined by the *International Commission on Zoological Nomenclature* [henceforth the Commission], have been an integral part of zoological nomenclature since the early 1900s (for a historical review of family-group names refer to Bock 1994).

The first family-group names based on the stem of their type genus appeared in zoological literature in the early 19th Century (see Sabrosky 1999). The first authors inconsistently used a variety of endings (–ides, –ites, –ida, –i, –ide, etc.) for suprageneric divisions at various levels. The ending –idae was apparently first suggested by Kirby (1813) as a characteristic ending for names at the rank of family. Although many ranks have been used in the past for suprageneric names, only the following suffixes are currently regulated by the *International Code of Zoological Nomenclature* [henceforth the Code] (ICZN 1999a): superfamily (–oidea), family (–idae), subfamily (–inae), tribe (–ini) and subtribe (–ina).

The last twenty five years has seen an increase in the number of studies on family-group names of entire groups in zoology (e.g., Štys and Jansson 1988 for Hemiptera: Nepomorpha; Bock 1994 for Aves; McKenna and Bell 1997 for Mammalia; Ferraris and de Pinna 1999 for Pisces: Siluriformes; Sabrosky 1999 for Diptera; Engel and Krishna 2004 for Isoptera; Speidel and Naumann 2004 for Lepidoptera: Noctuoidea; Bouchet and Rocroi 2005 for Gastropoda; Engel 2005 for Hymenoptera: Apoidea; Engel and Haas 2007 for Dermaptera; Bouchet and Rocroi 2010 for Bivalvia). The contents (i.e., whether they include fossils or not) and presentation of the data (i.e., in a classification scheme, in chronological order or in alphabetic order) in those works vary greatly. The most extensive treatments of animal family-group names to date are those of Diptera (Sabrosky 1999) and Gastropoda (Bouchet and Rocroi 2005) which covered names based on approximately 2000 and 2400 distinct type genera respectively. Some of the reasons given by authors of these works for providing comprehensive lists of family-group names include avoiding the unnecessary proposal of new names, facilitating decisions on priority and promoting long-term stability of the classification. We believe that such catalogues are especially important as the number of studies dealing with the higher relationships of major clades will continue to increase as new algorithms and data sets (e.g., molecular data) become available.

Given the importance of family-group names in the scientific literature, it is rather surprising that still many authors do not cite the author or year of publication of those names correctly, or at all. According to Recommendation 51A of the Code (ICZN 1999a) “The original author and date of a name should be cited at least once in each work dealing with the taxon denoted by that name.” In addition to satisfying Recommendation 51A of the Code, citation of authorities of scientific names can also have a positive impact on modern world taxonomists (Werner 2006; Agnarson and Kuntner 2007). Adding to further confusion and errors in the literature is the fact that the Principle of Coordination (Art. 36.1) is still overlooked by some authors.

Coleoptera are currently the most species-rich group of organisms on this planet with approximately 360 000 described species (Bouchard et al. 2009). The great morphological diversity of beetles has led to the proliferation of suprageneric taxa at various ranks. Latreille (1797) was apparently the first to introduce the concept of family-level taxa (see Bock 1994: 244) but it is only a few years later (Latreille 1802) that he proposed available names for these groupings, including several in the order Coleoptera. More recent evidence suggests that available family-group names in some groups of animals other than Coleoptera (e.g., Chordata: Sauropsida) appeared in the late 18th Century (see Dubois and Bour 2010), even earlier than in Latreille (1802).

The nomenclature of family-group names in Coleoptera did not receive much attention until the treatment of Geadephaga names by Madge (1989). Similar lists, based on rules from two different editions of the Code (ICZN 1985g, 1999a), have now been published in Dytiscidae (Nilsson et al. 1989), Staphyliniformia (Newton and Thayer 1992), Cucujoidea (Pakaluk et al. 1994), Curculionoidea (Alonso-Zarazaga and Lyal 1999), Buprestoidea (Bellamy 2003, 2008a-d, 2009), Tenebrionidae (Bouchard et al. 2005), Scarabaeoidea (Smith 2006) and Cerambycidae (Bousquet et al. 2009). Another important contribution was that of Lawrence and Newton (1995) which included a review of the nomenclature of all beetle family-group names for the rank of subfamily and above.

The vast body of scientific literature dealing with beetles has been a deterrent to producing a complete review of all Coleoptera family-group names in the past. This publication was only made possible by the collaboration of several coleopterists.

The specific objectives of this study are to: 1) establish the first comprehensive list of all Coleoptera family-group names with information on type genus, author(s), year of publication and complete bibliographical references; 2) assess the availability and validity of each name using rules laid out in the most recent *Code of Zoological Nomenclature* (ICZN 1999a), 3) summarize Priority and Homonymy problems with currently used names and, 4) propose or implement solutions to these problems in order to promote stability. We include family-group names that were published on or before December 31, 2010.

This publication should be seen as the starting point of what could eventually serve as the basis for a submission of Part of a *List of Available names in Zoology* (Art. 79). With this goal in mind, we ask all coleopterists to either send us, or to publish, corrections or differences of opinions in the months and years to come. It is our intention to update our list of family-group names as new data are published and to provide a second edition of this work in approximately five years.

## Methods

### Criteria of availability

Decisions about the availability and validity of each Coleoptera family-group name in our catalogue were made following the process outline in Figure 1. First we established whether the name was available or unavailable based on the criteria of availability summarized below. If a name first proposed on a particular type genus was determined to be unavailable then searches were conducted to establish if a family-group name based on the same type genus was made available subsequently. If so then this available name was entered in our catalogue. From the pool of available names, we removed those that are permanently invalid (Art. 39). All the remaining available names were then separated into those that are valid based on the classification used here (at any rank from subtribe to superfamily) and those that are invalid (i.e., synonyms).

**Figure:**
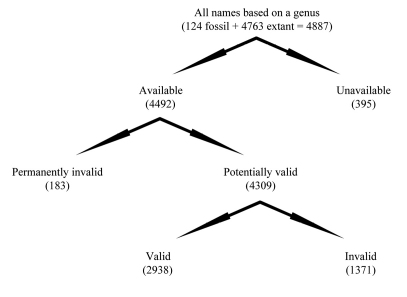
**Figure 1.** Overview of the process used to determine the availability and validity of family-group names in Coleoptera (modified from Bouchet and Rocroi 2005). The number of names for each category is given in parentheses.

In order to be available, a family-group name proposed before 1931 must to be a scientific name (i.e., in latinized form) in the nominative plural based on the stem of an available genus name then used as valid in the new suprageneric taxon (Art. 11.7).

In addition to the criteria of availability mentioned above, new family-group names proposed between 1931 and 1999 had to be described in words, or be associated with a bibliographic reference to such a description, in order to be considered available (Arts 13, 15). In works published after 1930 which contained more than one use of a new family-group name, we selected a page where a description was clearly associated with the new taxon for the catalogue. It should be noted that replacement names proposed in that time period are available without description (Art. 13.1.3). Additionally, “a family-group name first published after 1930 and before 1961 which does not satisfy the provisions of Article 13.1 is available from its original publication only if it was used as valid before 2000, and also was not rejected by an author who, after 1960 and before 2000, expressly applied Article 13 of the then current editions of the Code” (Art. 13.2.1). Names proposed between 1931 and 1960 can also be considered available if the description of the new family-group name and a single new genus-group name is combined (Art. 13.5). Finally, family-group names proposed since 2000 have to be explicitly indicated as new and the name of the type genus has to be clearly cited in order to be available (Art. 16). Based on our interpretation of Article 16.2, any new family-group name proposed after 1999 in a paper in which the name of the type genus is clearly cited in connection with it (although maybe not explicitly with a formula such as “Type genus = *Aus* Doe, 2010”) is available.

One of the most difficult tasks while working on this review was to decide on the most consistent and objective way to apply Article 11.7.2, which deals with the availability of names that were originally proposed in a vernacular form. Vernacular names are generally not treated as scientific names in zoological nomenclature (see Recommendation 11A). However, the International Commission on Zoological Nomenclature has made a single, but rather restricted, exception that applies to family-group names. According to the Code, a vernacular name “…is available with its original author and date only if it has been latinized by later authors and has been generally accepted as valid by authors interested in the group concerned and as dating from that first publication in vernacular form” (Art. 11.7.2).

We have interpreted names proposed in the following languages to be vernacular: all German names with the suffix “–en”, all Spanish names with the suffix “–os” or “–as” and all French names with the suffix “–iens”. The most important issue was to determine the correct status of names originally proposed with the suffix “–es.” We have used the principle that all family-group names proposed by non-French writers with the suffix “–es” were in fact latinized names with an ending that differs from those regulated by the Commission (these are mostly older names proposed before rules of zoological nomenclature became well-established). For each name with the suffix “–es” first proposed by French workers (e.g., Latreille, Lacordaire, Lameere) we went through the entire work containing those names and established if these authors consistently used either vernacular or latinized names in their work. French vernacular names often have accents in them (“é” or “è”) while latinized names do not. We have found that determining whether an author used vernacular or latinized names in a particular work was fairly straightforward (Bousquet et al. 2009). Latreille proposed new family-group names with the suffix “–es” in several of his works. We noticed that he most often used both the vernacular (listed first, with accents when required by French language) and latinized (listed second, always in italics) forms of each name together in the same heading. We have listed the latinized form of the name in each of those cases.

As in Bousquet et al. (2009), we have interpreted the requirements listed in Art. 11.7.2. as three separate conditions to be met. For each vernacular name we determined if it had 1) been subsequently latinized, 2) been generally accepted as valid by authors interested in the group and 3) been attributed to the author and date of original publication. Failure to fulfil any of the requirements resulted in the treatment of that name as unavailable. In those cases, we provide a comment explaining why this taxon was treated as unavailable (e.g., “original vernacular name unavailable (Art. 11.7.2): not subsequently latinized”). Many recent authors have treated family-group names first proposed in vernacular form as available if they were latinized by later authors but we do not believe that this practice is in line with the requirements of the Code. We have accepted as available all vernacular names published before 1900 that have subsequently been used in latinized form, while being used as valid, and credited to the publication in their vernacular form. For every vernacular name that meets the requirement of availability, we have added a comment in the format of the following example “original vernacular name available (Art. 11.7.2): first used in latinized form by J. L. LeConte (1862: 240, as Meracanthini), generally accepted as in Gebien (1911: 567, as Meracanthinae)”.

### Formation and treatment of family-group names

Article 29.2 includes a list of suffixes to be used for groups at the superfamily (-oidea), family (-idae), subfamily (-inae), tribe (-ini) and subtribe (-ina) names. The suffix of other categories is not regulated by the Code. The rank of supertribe (-itae) was used here for a small number of groups in which recent changes in classification required an additional rank between subfamily and tribe (e.g., Staphylinoidea).

The correct spelling of family-group names depends on the stem of its type genus (Art. 29.3). As pointed out by Newton and Thayer (1992) the family-group name stems of most generic names that can be regarded as Latin nouns are determined easily by dropping the following nominative case ending –*us*, –*um*, –*es* and –*a*. Examples of such straightforward case in Coleoptera include Carabidae (type genus *Carabus*), Adeliini (type genus *Adelium*), Trechodina (type genus *Trechodes*) and Anthiini (type genus *Anthia*). A summary of other commonly encountered generic endings, along with their corresponding stems, is given in Table 1. Note that generic names ending with -*gaster* (Greek for stomach) can have either -*gaster*- or -*gastr*- as their correct stem and therefore we have accepted the stem formation of the first author of the family-group name based on such genera as correct. Stems were reviewed for all type genera included in our catalogue.

**Table:** **Table 1.** List of common Coleoptera generic suffixes with their associated family-group name endings (based partly on Bouchet and Rocroi 2005, with additions by YB, AFN, MAAZ and M. K. Thayer).

*Generic ending*	*Meaning*	*Derived family-group name ending*	*Type genus example*	*Family-group name example*
-*apion*	pear (Greek)	-api-	*Aspidapion*	Aspidapiina
-*arthron*	joint (Greek)	-arthr-	*Decarthron*	Decarthrina
-*aspis*	shield (Greek)	-aspid-	*Anaspis*	Anaspidinae
-*baris*	flat-bottomed boat (Greek)	-barid-	*Baris*	Baridini
-*chlamys*	mantle (Greek)	-clamyd-	*Spodochlamys*	Spodochlamydini
-*celis*	spot (Greek)	-celid-	*Xiphoscelis*	Xiphoscelidini
-*ceras*	horn (Greek)	-cerat-	*Megaceras*	Megaceratini
-*cnema*	shin or tibia (Greek)	-cnem-	*Pachycnema*	Pachycnemina
-*cupes*	dainty (Latin)	-cuped-	*Cupes*	Cupedidae
-*dacne*	bite (Greek)	-dacn-	*Dacne*	Dacnini
-*deres*	neck, throat (Greek)	-der-	*Aglycyderes*	Aglycyderini
-*derma*	skin (Greek)	-dermat-	*Cryptoderma*	Cryptodermatinae
-*dytes*	diver (Greek)	-dyt-	*Aspidytes*	Aspidytidae
-*genys*	jaw (Greek)	-geny-	*Chaetogenys*	Chaetogenyini
-*hospes*	guest (Latin)	-hospit-	*Termitohospes*	Termitohospitini
-*ides*	similar to, derived from (Greek)	-id-	*Anaides*	Anaidinae
-*ifer*	carrier of (Latin)	-ifer-	*Undulifer*	Unduliferinae
-*iger*	carrier of (Latin)	-iger-	*Apoderiger*	Apoderigerina
-*ites*	like (Latin, Greek)	-it-	*Aegialites*	Aegialitinae
-*loma*	edge or fringe (Greek)	-lomat-	*Discoloma*	Discolomatinae
-*macer*	thin (Latin)	-macr-	*Rhynchitomacer*	Rhynchitomacrini
-*mycter*	nose (Greek)	-mycter-	*Eurymycter*	Eurymycterini
-*odes*	similar to (Greek)	-od-	*Agyrtodes*	Agyrtodini
-*odon*	tooth (Greek)	-odont-	*Pentodon*	Pentodontini
-*oides*	like (Greek)	-oid-	*Acmaeoderoides*	Acmaeoderoidina
-*omma*	eye (Greek)	-ommat-	*Omma*	Ommatidae
-*onyx*	yellow gem stone (Greek)	-onych-	*Trichonyx*	Trichonychini
-*ops*	eye (Greek)	-op-	*Achaenops*	Achaenopina
-*opsis*	appearance (Greek)	-opse-	*Brachyceropsis*	Brachyceropseini
-*otes*	quality, nature (Greek)	-ot-	*Agriotes*	Agriotini
-*pholis*	horny scale (Greek)	-pholid-	*Trachypholis*	Trachypholidini
-*pteryx*	wing (Greek)	-pteryg-	*Trichopteryx*	Trichopterygini
-*pus*	foot (Greek)	-pod-	*Baripus*	Baripodina
-*rhinus*	snout (Greek)	-rhin-	*Platyrhinus*	Platyrhinini
-*rhynchus*	snout (Greek)	-rhynch-	*Doydirhynchus*	Doydirhynchini
-*rhipis*	fan (Greek)	-rhipid-	*Xenorhipis*	Xenorhipidini
-*soma*	body (Greek)	-somat-	*Platysoma*	Platysomatini
-*stoma*	mouth (Greek)	-stomat-	*Stenostoma*	Stenostomatini
-*teles*	perfect (Greek)	-tel-	*Abroteles*	Abrotelina
-*termes*	wood-worm (Latin)	-termit-	*Philotermes*	Philotermitini
-*thorax*	chest (Greek)	-thorac-	*Mecyclothorax*	Mecyclothoracini
-*trox*	gnawer (Greek)	-trog-	*Trox*	Trogidae
-*trupes*	borer (Greek)	-trup-	*Ceratotrupes*	Ceratotrupini
-*typus*	shape (Greek)	-typ-	*Amarotypus*	Amarotypini

It should be noted that if a family-group name was not formed in accordance with Art. 29.3 but its original spelling is in prevailing usage then the current spelling is to be maintained (Art. 29.5). We have conserved the spelling of several family-group names currently used as valid however we did not do so for names that are listed as synonyms. For names based on incorrect stems proposed after 1999, we have considered that prevailing usage cannot be used to conserve the original spellings because too few references using these names could be found. We have therefore corrected the stems of such names unless the name of the type genus was an arbitrary combination of letters (Art. 29.4).

As stated in Art. 35.4.1 “A family-group name based upon an unjustified emendation … or an incorrect spelling of the name of the type genus must be corrected, unless it is preserved under Article 29.5 or unless the spelling of the genus-group name used to form the family-group name is preserved under Articles 33.2.3.1 or 33.3.1.” When an unjustified emendation or an incorrect subsequent spelling of the type genus is in prevailing usage and is attributed to the original author and date (Art. 33.2.3.1; 33.3.1), the correct spelling of the type genus is that in current usage.

In the glossary of the Code, a name in prevailing usage is defined as a “name which is adopted by at least a substantial majority of the most recent authors concerned with the relevant taxon, irrespective of how long ago their work was published”. The unfortunate subjectivity in this definition, as pointed out by Ferraris (2000), left us with no choice but determine prevailing usage in an *ad hoc* fashion throughout.

### Principle of Coordination

The Principle of Coordination (Art. 36.1) is, unfortunately, still overlooked by some authors. In some instances authors who propose new ranks for previously established suprageneric names are sometimes treated as the author of those names when in fact only a change of rank was presented. Based on the Code “A name established at any rank in the family-group is deemed to have been simultaneously established for nominal taxa at all ranks in the family-group; all these taxa have the same type genus, and their names are formed from the stem of the name of the type genus [Art. 29.3] with appropriate change of suffix [Art. 34.1]. The name has the same authorship and date at every rank.”

### Principle of Priority

As for species- and genus-group names, the oldest available name for a family-group taxon should be considered as valid (Art. 23). However four important exceptions need further discussion. Firstly, when a little-known family-group name was discovered to be older than a name currently used as valid for a particular taxon, we used the Reversal of Precedence to conserve usage of the younger name if the conditions of Art. 23.9.2 could be fulfilled. Younger names conserved using Reversal of Precedence are listed in Appendix 1. In some instances we could not fulfill all conditions of Art. 23.9.2 although we considered that using the newly discovered older name as valid would threaten stability or cause confusion. In those cases we maintained usage of the younger name as valid and either submitted an application to the Commission to conserve the younger name or made a recommendation that such submission should be submitted in the near future (Art. 23.9.3).

According to Art. 35.5 “If after 1999 a name in use for a family-group taxon (e.g., for a subfamily) is found to be older than a name in prevailing usage for a taxon at higher rank in the same family-group taxon (e.g., for the family within which the older name is the name of a subfamily) the older name is not to displace the younger name.” Indeed, we encountered a small number of cases in which the replacement of a name at the higher rank (e.g., Lymexyloidea Fleming, 1821) by the discovery of an older name for a taxon at a lower rank (e.g., Hylecoetoidea Germar, 1818) would not have served the stability of well-established names. In such cases usage of the younger name at the higher rank was conserved.

In cases where a family-group name was replaced before 1961 because of the synonymy of its type genus (e.g., Lepiceridae Hinton, 1936 instead of Cyathoceridae Sharp, 1882), the substitute name is to be maintained if it is in prevailing usage (Art. 40.2). In such cases, the valid family-group name retains its own author but takes the priority of the replaced name. Based on Recommendation 40A, we have cited those names with their original author and date, followed by the date of its priority enclosed in parentheses, as determined by Art. 40.2.1 (e.g., Lepiceridae Hinton, 1936 (1882)).

Lastly, if the stability and continuity of the meaning of a family-group name is threatened by the discovery that the type genus was originally misidentified, or that the type genus was based on a misidentified type species, or that an older type species of the type genus had been overlooked, then the case is to be referred to the Commission for a ruling (Art. 65.2.1). In cases where the oldest available name for a family-group taxon is based on misidentified type genus, or an altered concept of the type genus (e.g., see Scydmaenini Reitter, 1882), we have preferred to consider this name as invalid and to use instead the family-group name which is in prevailing usage (e.g., Cyrtoscydmini L. W. Schaufuss, 1889) until an application is submitted and a ruling is rendered by the Commission.

A list of problem cases based on the Principle of Priority, with comments on implementation of solutions or necessary actions to be taken in the future, is given in Appendix 2.

### Principle of Homonymy

Based on the Principle of Homonymy “when two or more names are homonyms, only the senior, as determined by the Principle of Priority…, may be used as the valid name…” (Art. 55.2). Here we report several instances in which family-group names in Coleoptera are identical to other family-group names in zoological nomenclature. These names are either based on identical type genera or on type genera that are similar but not identical. In the first instance the family-group name based on the preoccupied type genus is permanently invalid (Art. 39) but available.

When family-group names are homonyms because their type genera are similar but not identical, the case must be referred to the Commission for a ruling to remove homonymy (Art. 55.3.1). Such is the case with Adeliini Kirby, 1825 (type genus *Adelium* Kirby, 1819) and the hymenopteran name Adeliini Viereck, 1918 (type genus *Adelius* Haliday, 1833) which are both correctly formed from the stem of their type genus. More than thirty such cases were encountered during our research on Coleoptera family-group names.

Junior homonyms conserved using Reversal of Precedence are listed in Appendix 1. A list of problem cases based on the Principle of Homonymy, with comments on implementation of solutions or necessary actions to be taken in the future, is given in Appendix 3.

### Submissions to the Commission

As mentioned above, we have encountered several cases which require an application to the Commission because of problems with priority and/or homonymy. Some of these cases have been submitted recently (Engel and Bouchard 2009, Bousquet et al. 2010, Bousquet and Bouchard 2010) and others will be submitted in the near future (see summary in Appendices 2 and 3). However, it is not our intention to submit applications for all cases. Since we intend to submit this work as Part of the *List of Available Names in Zoology* in approximately five years, we hope the coleopterist community will take this opportunity to submit applications to the Commission in order to resolve some of the remaining problems outlined here.

### Bibliographic notes

Von Hayek (1973: 282, 290) and Stibick (1979: 157) reported that the important works on Elateridae classification by Candèze (1857) and Lacordaire (1857) were published in May and June of 1857 respectively. Since then, when the same new name appeared in both of these works, Candèze’s names have been given priority over Lacordaire’s. We have discovered that Lacordaire’s work was in fact published before 25 May 1857 (as recorded by the Académie des Sciences de France) which would make the names in his work the oldest based on strict adherence to the Code (Art. 21.3). In order to avoid unnecessary changes in authorship in the future and maintain prevailing usage, we have opted to continue attributing elaterid family-group names to Candèze, 1857.

Authorship of the three volumes on Coleoptera of the series *Encyclopédie d’histoire naturelle* is debated. The title pages on the volumes list Jean Charles Chenu as the author of the *Encyclopédie d’histoire naturelle* with the assistance of Eugène Desmarest for the Coleoptera section. However, the *Société Entomologique de France* recorded the livraisons received for 1851 in their *Bulletin* (p. cxxv) as “*Encyclopédie d’histoire naturelle, ou traité complet de cette science, sous la direction de M. le docteur Chenu. Coléoptères, par M.E. Desmarest*.” This was likely the title on the wrapper and clearly suggests that Desmarest was responsible alone for the three volume series on Coleoptera. This is also substained by Desmarest himself who in the *Bulletin de la Société Entomologique de France* for 1860 (p. lxiii) speaks of the three volumes series as being his work, published under the direction of Chenu. Based on the above fact, we credit the three-volume series to Desmarest alone (Desmarest 1851, 1857, 1860).

The articles presented by Mulsant (and Rey) in the “Opuscules Entomologiques” are considered here to be reprints (as a compilation) of original publications in various Annales, mostly printed at Lyon, for the following reasons: In the dedication to the first volume Mulsant wrote “ces Opuscules, publiés déjà ça et là dans nos Recueils académiques”. The dates of the dedications in the Opuscules are always later than the dates on which they were presented to the various Sociétés, frequently in the following year. Although published at Paris, the Opuscules (see verso of title pages) were printed at Lyon by F. Dumoulin and others, the same printers which published the *Mémoires de l’Académie... des Sciences, Belles-Lettres et Arts de Lyon*, etc. Rey himself, in later publications such as the continuation of the “Histoire Naturelle des Coléoptères de France” (e.g., *Annales de la Société Linnéenne de Lyon* (N. S.) 32[1885]: 1-186 + [4], pls. 1-2 [see p. 106]) cited the *Annales de la Société Linnéenne de Lyon* article before the *Opuscules* version. E. A. Fitch (1881: 46-47), in his obituary of Mulsant, indicated that both the Histoire Naturelle des Coléoptères de France and the Opuscules Entomologiques «appeared originally... in the Annals» and «later republished in separate form at Paris». These concepts were reiterated by Tottenham (1949: 352). Marseul (1882: 20) wrote, “Tous les ouvrages de Mulsant, sauf quelques-uns, ont été publiés dans les Annales des trois sociétés... et l’Académie des Sciences. Tous ont été reproduits séparément soit dans l’*Histoire Naturelle des Coléoptères et des Punaises de France*, soit dans les *Opuscules*.»

However, the first fascicles of the *Histoire Naturelle* series up to the “*Pectinipèdes*” were not reprinted in journals. It seems that most of the succeeding fascicles were submitted to the various Sociétés, were printed by Dumoulin, Barret, Pinier, etc. at Lyon, the dedications were then added and they were sent to Paris for separate publication with the same typesetting, and with changes only to the pagination and some titles. Since the issuing of the journal volumes was frequently delayed by the compilation of many papers (usually in the following year), the separate edition often appeared first, as shown by records of other journals and, in particular, the *Bibliographie de la France*. Therefore we have given date priority to the *Histoire Naturelle* versions in most instances, only those page numbers are cited with the scientific names in the catalogue unless evidence was found to the contrary, and the alternate versions are cited only in the bibliography, following each reference.

### Format of the catalogue and conventions used

This paper is organized into two main parts: 1) a synoptic classification containing only the names which we consider as valid herein and 2) a catalogue of all family-group names organized in the same order as in the synoptic classification. It should be noted that our publication is first and foremost a nomenclatural treatment of family-group names. We want to emphasize that the classification used here is not based on newly generated phylogenetic data. Furthermore, we do not necessarily endorse all parts of the classification presented, particularly those which were not based on phylogenetic approaches. For the most part, we follow currently accepted concepts in recent taxon-specific catalogues as well as comprehensive syntheses such as the *Handbook of Zoology* (Beutel and Leschen 2005, Leschen et al. 2010), *American Beetles* (Arnett and Thomas 2000; Arnett et al. 2002) and the *Catalogue of Palaearctic Coleoptera* (Löbl and Smetana 2003, 2004, 2006, 2007, 2008, 2010). Although we recognize that the classification we use will likely become outdated in the near future, we believe that this is the best way to present the assembled data.

Only family-group names that are based on genera are included in our catalogue. For each family-group name the original spelling, author, year of publication, page number, correct stem and type genus are given. Complete data and comments regarding a particular family-group name are presented with the lowest-rank name when the same stem is used in more than one rank, since the same criteria apply in accordance with the Principle of Coordination (Art. 36.1). The correct stem to be used, which is given in square brackets (e.g., [stem: *Uralocole-*]), is especially important for synonyms, since the status of a name may be changed from synonym to valid in the future. Subsequent alternative spellings of family-group names (i.e., simple changes in suffix depending on change in rank) are not given here. Both available and unavailable names are listed together in the catalogue in order to enhance information retrieval. Unavailable names are preceded by an asterisk “*” and are identified as such in the “Comments” section along with the reason for this status. As is common practice in publications of the International Commission on Zoological Nomenclature, we use small capital letters for family-group names. Names in the catalogue are organized in a “phylogenetic” framework, as accepted in recent publications on the group, down to subfamily level. Valid tribes and subtribes are listed in alphabetical order within each subfamily. Synonyms are listed in chronological order under each valid taxon.

Known works of Johannes Gistel (who also published under the name Johannes Gistl and as G. Tilesius; see Evenhuis 1997a) were included in this paper. Although Gistel’s works were largely ignored by contemporaries (e.g., Gemminger and Harold 1868a) and continue to be ignored by some workers (e.g., Cate 2007) they are nevertheless broadly available and represent a significant contribution to the nomenclature of family-group and genus-group names in Coleoptera. In order to promote stability, some names proposed for the first time by Gistel (but ignored until now) that threaten names in current usage are treated here as *nomina oblita* (when conditions of Art. 23.9 could be met) or are subject to appeals to the Commission. According to the glossary of the Code, a *nomen oblitum* is a “Latin term applied after 1 January 2000 to a name, unused since 1899, which as a result of an action taken under Article 23.9.2 does not take precedence over a younger synonym or homonym in prevailing usage. The term *nomen oblitum* was also applied to a disused senior synonym rejected between 6 November 1961 and 1 January 1973 under Art. 23b of the Code editions then in force (see Art. 23.12.2). *Nomina oblita* are available names; see Articles 23.9 and 23.12 for conditions controlling their use as valid names.”

Because of the importance of the Principle of Priority, we have tried to find the most accurate date of publication (given in square brackets in the References section) for works cited in the manuscript. The date of publication was determined either from the original publication itself, from the date of “stamps” when received in libraries of natural history institutions or from secondary literature sources. A list of natural history institutions and secondary literature sources is given at the beginning of the References section. Data on dates of publication were provided by AFN, PB, AED, MAAZ, CHCL, ABTS and YB. References for type genera are not included here to conserve space and because they are for the most part available in other recent publications (e.g., Löbl and Smetana 2003, 2004, 2006, 2007, 2008, 2010).

Names on the *Official List of Family-Group Names in Zoology* and type genera on the *Official List of Generic Names in Zoology* are included in our catalogue and are summarized in Appendix 4 and 5 respectively. Cases involving family-group names and/or their type genera that are awaiting a ruling by the Commission are summarized in Appendix 6.

## Results

### Number of family-group names

A total of 4887 family-group names are included in our catalogue (see Figure 1). Of the names recorded, 4492 are available, of which 183 are permanently invalid because they are based on a preoccupied type genus or a genus which has been suppressed by the Commission. The majority of names were proposed in the suborder Polyphaga (4314) followed by Adephaga (531), Myxophaga (16), Archostemata (17) and Protocoleoptera (9). Within Adephaga the family Carabidae contains the highest number of names by far (441) followed by Dytiscidae (47). The five superfamilies with the highest number of names in Polyphaga are the Curculionoidea (862), Chrysomeloidea (794), Staphylinoidea (594), Tenebrionoidea (579) and Scarabaeoidea (436). Overall, the five families with the highest number of family-group names proposed are Curculionidae (555), Staphylinidae (493), Cerambycidae (468), Carabidae (441) and Tenebrionidae (323).

The number of Coleoptera family-group names that appeared in the 19th and 20th Centuries are almost identical (2331 and 2556 respectively) while the last decade saw an increase of 5% (246 names) in the total number of names in the literature (Figure 2). A large number of names proposed in the middle of the 19th Century are unavailable. Those names were generally proposed in vernacular form and were not made available subsequently (Art. 11.7.2). Unavailable names proposed after 1930 generally lacked a description or bibliographic reference to such a description (Arts 13, 15). Several names proposed in the last decade were either not explicitly introduced as new taxa or did not include necessary information about the type genus and are therefore unavailable (Art. 16).

**Figure:**
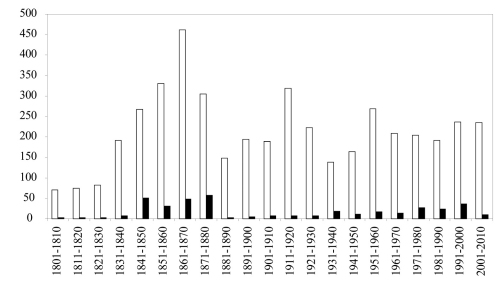
**Figure 2.** Number of family-group names proposed in Coleoptera by decade. White bars = available names. Black bars = unavailable names.

### Significant contributions

A summary of the most significant contributions, in terms of the total number of names proposed by author, is shown in Table 2. Eighteen authors proposed 50 or more family-group names in Coleoptera. Lacordaire was the most prolific with 353 new names. Although the majority of Lacordaire’s names were proposed in vernacular form, a large proportion of them (90%) were subsequently made available and many are used as valid today.

**Table:** **Table 2.** Summary of number of family-group names in Coleoptera by author. Only authors that have proposed fifty or more names are included. Authors listed in decreasing order by total number of names proposed. For each author we give the total number of name proposed, the percentage of those names that are available, the range of years in which those names were proposed and the number of families in which they proposed new family-group names in.

Rank	Author	Country of origin	Total family-group names	Percent available	Publication <br/> (year range)	Number of families
1	Lacordaire, Jean Théodore	France	353	90	1848–1872	34
2	Gistel, Johannes von Nepomuk Franz Xaver	Germany	135	99	1848–1856	42
3	LeConte, John Lawrence	USA	131	100	1847–1883	45
4	Thomson, James	USA	124	99	1857–1877	6
5	Mulsant, Étienne	France	122	76	1839–1880	25
6	Chapuis, Félicien	France	120	72	1869–1876	7
7	Jeannel, René	France	109	99	1910–1967	6
8	Reitter, Edmund	Germany	87	100	1875–1926	24
9	Legalov, Andrei Aleksandrovich	Russia	73	100	2001–2009	9
10	Voss, Eduard	Germany	66	97	1922–1972	6
11	Burmeister, Hermann Carl Conrad	Germany	66	98	1840–1873	2
12	Casey, Thomas Lincoln	USA	64	100	1884–1922	14
13	Horn, George Henry	USA	63	100	1867–1893	20
14	Latreille, Pierre André	France	62	98	1802–1834	30
15	Rey, Claudius	France	57	53	1853–1886	12
16	Thomson, Carl Gustaf	Sweden	55	100	1857–1867	27
17	Blanchard, Charles Émile	France	55	71	1845–1853	20
18	Laporte, François Louis Nompar de Caumont (Comte Castelnau)	France	51	100	1834–1840	21

The authors with the lowest percentage of available family-group names (Mulsant, Chapuis, Rey and Blanchard) originally proposed their names in vernacular form and a significant proportion of those names were not made available subsequently. All names proposed by LeConte, Reitter, Casey, Horn, Legalov, C. G. Thomson and Laporte are considered available.

Thirteen of the authors in Table 2 were active exclusively during the 19th Century while two authors, Reitter and Casey, published their works containing new family-group names in both the 19th and 20th Centuries. Two authors published family-group names exclusively during the 20th Century (Jeannel and Voss). Legalov is our only contemporary colleague in this list; he proposed more than 50 new family-group names in the last decade. It should be noted that the recent proliferation of new scientific names proposed by Legalov is treated by some authors as “extreme splitting” (Riedel 2006) and “based on numerous spurious characters of doubtful phylogenetic value” (Oberprieler et al. 2007).

The broad taxonomic interests and expertise of Lacordaire, Gistel, LeConte, Mulsant, Latreille and C. G. Thomson are exemplified by the fact that they introduced new family-group names in 25 or more families. This was done either in catalogues (e.g., Gistel, C. G. Thomson) and/or in large scale taxonomic treatments (e.g., Lacordaire, LeConte, Latreille, Mulsant). On the other hand, the taxonomic expertise of J. Thomson, Chapuis, Jeannel, Voss, Burmeister and Legalov is much more targeted towards a small number of families (fewer than 10).

### New nomenclatural acts

The following synonymies are recorded here for the first time: Agronomina Gistel, 1848 syn. nov. of Amarina Zimmermann, 1832 (Carabidae), Hylepnigalioini Gistel, 1856 syn. nov. of Melandryini Leach, 1815 (Melandryidae), Polycystophoridae Gistel, 1856 syn. nov. of Malachiinae Fleming, 1821 (Melyridae), Sclerasteinae Gistel, 1856 syn. nov. of Ptilininae Shuckard, 1839 (Ptinidae), Phloeonomini Ádám, 2001 syn. nov. of Omaliini MacLeay, 1825 (Staphylinidae), Sepedophilini Ádám, 2001 syn. nov. of Tachyporini MacLeay, 1825 (Staphylinidae), Phibalini Gistel, 1856 syn. nov. of Cteniopodini Solier, 1835 (Tenebrionidae); *Agronoma* Gistel 1848 (type species *Carabus familiaris* Duftschmid, 1812, designated herein) syn. nov. of *Amara* Bonelli, 1810 (Carabidae), *Hylepnigalio* Gistel, 1856 (type species *Chrysomela caraboides* Linnaeus, 1760, by monotypy) syn. nov. of *Melandrya* Fabricius, 1801 (Melandryidae), *Polycystophorus* Gistel, 1856 (type species *Cantharis aeneus* Linnaeus, 1758, designated herein) syn. nov. of *Malachius* Fabricius, 1775 (Melyridae), *Sclerastes* Gistel, 1856 (type species *Ptilinus costatus* Gyllenhal, 1827, designated herein) syn. nov. of *Ptilinus* Geoffroy, 1762 (Ptinidae), *Paniscus* Gistel, 1848 (type species *Scarabaeus fasciatus* Linnaeus, 1758, designated herein) syn. nov. of *Trichius* Fabricius, 1775 (Scarabaeidae), *Phibalus* Gistel, 1856 (type species *Chrysomela pubescens* Linnaeus, 1758, by monotypy) syn. nov. of *Omophlus* Dejean, 1834 (Tenebrionidae). The following replacement name is used here for the first time: Gompeliina Bouchard, 2011 nom. nov. for Olotelina Báguena Corella, 1948 (Aderidae).

Reversal of Precedence (Article 23.9) is used to conserve usage of the following names (family-group names followed by genus-group names): Perigonini Horn, 1881 nom. protectum over Trechicini Bates, 1873 nom. oblitum (Carabidae), Anisodactylina Lacordaire, 1854 nom. protectum over Eurytrichina LeConte, 1848 nom. oblitum (Carabidae), Smicronychini Seidlitz, 1891 nom. protectum over Desmorini LeConte, 1876 nom. oblitum (Curculionidae), Bagoinae Thomson, 1859 nom. protectum over Lyprinae Gistel 1848 nom. oblitum (Curculionidae), Aterpina Lacordaire, 1863 nom. protectum over Heliomenina Gistel, 1848 nom. oblitum (Curculionidae), Naupactini Gistel, 1848 nom. protectum over Iphiini Schönherr, 1823 nom. oblitum (Curculionidae), Cleonini Schönherr, 1826 nom. protectum over Geomorini Schönherr, 1823 nom. oblitum (Curculionidae), Magdalidini Pascoe, 1870 nom. protectum over Scardamyctini Gistel, 1848 nom. oblitum (Curculionidae), Agrypninae/-ini Candèze, 1857 nom. protecta over Adelocerinae/-ini Gistel, 1848 nom. oblita and Pangaurinae/-ini Gistel, 1856 nom. oblita (Elateridae), Prosternini Gistel, 1856 nom. protectum over Diacanthini Gistel, 1848 nom. oblitum (Elateridae), Calopodinae Costa, 1852 nom. protectum over Sparedrinae Gistel, 1848 nom. oblitum (Oedemeridae), Adesmiini Lacordaire, 1859 nom. protectum over Macropodini Agassiz, 1846 nom. oblitum (Tenebrionidae), Bolitophagini Kirby, 1837 nom. protectum over Eledonini Billberg, 1820 nom. oblitum (Tenebrionidae), Throscidae Laporte, 1840 nom. protectum over Stereolidae Rafinesque, 1815 nom. oblitum (Throscidae) and Lophocaterini Crowson, 1964 over Lycoptini Casey, 1890 nom. oblitum (Trogossitidae); *Monotoma* Herbst, 1799 nom. protectum over *Monotoma* Panzer, 1792 nom. oblitum (Monotomidae); *Pediacus* Shuckard, 1839 nom. protectum over *Biophloeus* Dejean, 1835 nom. oblitum (Cucujidae), *Pachypus* Dejean, 1821 nom. protectum over *Pachypus* Billberg, 1820 nom. oblitum (Scarabaeidae), *Sparrmannia* Laporte, 1840 nom. protectum over *Leocaeta* Dejean, 1833 nom. oblitum and *Cephalotrichia* Hope, 1837 nom. oblitum (Scarabaeidae).

## Synoptic classification of the world Coleoptera

**Order Coleoptera**

**†Suborder PROTOCOLEOPTERA**

**†Superfamily Tshekardocoleoidea Rohdendorf, 1944**

**†Family Tshekardocoleidae Rohdendorf, 1944**

**†Family Labradorocoleidae Ponomarenko, 1969**

**†Family Oborocoleidae Kukalová, 1969**

**†Superfamily Permocupedoidea Martynov, 1933**

**†Family Permocupedidae Martynov, 1933**

**†Family Taldycupedidae Rohdendorf, 1961**

**†Superfamily Permosynoidea Tillyard, 1924**

**†Family Ademosynidae Ponomarenko, 1968**

**†Family Permosynidae Tillyard, 1924**

**Suborder ARCHOSTEMATA**

**Family Crowsoniellidae Iablokoff-Khnzorian, 1983**

**Family Cupedidae Laporte, 1836**

**Subfamily Priacminae Crowson, 1962**

**†Subfamily Mesocupedinae Ponomarenko, 1969**

**Subfamily Cupedinae Laporte, 1836**

**Family Micromalthidae Barber, 1913**

**Family Ommatidae Sharp and Muir, 1912**

**†Subfamily Brochocoleinae Hong, 1982**

**Subfamily Tetraphalerinae Crowson, 1962**

**Subfamily Ommatinae Sharp and Muir, 1912**

†Tribe Lithocupedini Ponomarenko, 1969

†Tribe Notocupedini Ponomarenko, 1966

Tribe Ommatini Sharp and Muir, 1912

**Family Jurodidae Ponomarenko, 1985**

**†Family Triadocupedidae Ponomarenko, 1966**

**†Family Magnocoleidae Hong, 1998**

**†Family Obrieniidae Zherikhin and Gratshev, 1994**

**†Subfamily Kararhynchinae Zherikhin and Gratshev, 1994**

†Tribe Kararhynchini Zherikhin and Gratshev, 1994

†Tribe Kenderlykaini Legalov, 2009

**†Subfamily Obrieniinae Zherikhin and Gratshev, 1994**

**Suborder MYXOPHAGA**

**†Superfamily Asiocoleoidea Rohdendorf, 1961**

**†Family Asiocoleidae Rohdendorf, 1961**

**†Family Tricoleidae Ponomarenko, 1969**

**†Superfamily Rhombocoleoidea Rohdendorf, 1961**

**†Family Rhombocoleidae Rohdendorf, 1961**

**†Superfamily Schizophoroidea Ponomarenko, 1968**

**†Family Schizophoridae Ponomarenko, 1968**

**†Family Catiniidae Ponomarenko, 1968**

**†Family Schizocoleidae Rohdendorf, 1961**

**Superfamily Lepiceroidea Hinton, 1936 (1882)**

**Family Lepiceridae Hinton, 1936 (1882)**

**Superfamily Sphaeriusoidea Erichson, 1845**

**Family Torridincolidae Steffan, 1964**

**Subfamily Torridincolinae Steffan, 1964**

**Subfamily Deleveinae Endrödy-Younga, 1997**

**Family Hydroscaphidae LeConte, 1874**

**Family Sphaeriusidae Erichson, 1845**

**Suborder ADEPHAGA**

**†Family Tritarsidae Hong, 2002**

**Family Gyrinidae Latreille, 1810**

**Subfamily Spanglerogyrinae Folkerts, 1979**

**Subfamily Gyrininae Latreille, 1810**

Tribe Enhydrini Régimbart, 1882

Subtribe Dineutina Desmarest, 1851

Subtribe Enhydrina Régimbart, 1882

Tribe Gyrinini Latreille, 1810

Subtribe Gyrinina Latreille, 1810

Subtribe Heterogyrina Brinck, 1956

Tribe Orectochilini Régimbart, 1882

**Family Trachypachidae Thomson, 1857**

**†Subfamily Eodromeinae Ponomarenko, 1977**

**Subfamily Trachypachinae Thomson, 1857**

**Family Rhysodidae Laporte, 1840**

Tribe Leoglymmiini Bell and Bell, 1978

Tribe Dhysorini Bell and Bell, 1978

Tribe Medisorini Bell and Bell, 1987

Tribe Rhysodini Laporte, 1840

Tribe Clinidiini Bell and Bell, 1978

Tribe Omoglymmiini Bell and Bell, 1978

Tribe Sloanoglymmiini Bell and Bell, 1991

**Family Carabidae Latreille, 1802**

**†Subfamily Protorabinae Ponomarenko, 1977**

**†Subfamily Conjunctiinae Ponomarenko, 1977**

**Subfamily Nebriinae Laporte, 1834**

Tribe Nebriini Laporte, 1834

Tribe Notiokasiini Kavanaugh and Nègre, 1983

Tribe Notiophilini Motschulsky, 1850

Tribe Opisthiini Dupuis, 1912

Tribe Pelophilini Kavanaugh, 1996

**Subfamily Cicindinae Csiki, 1927**

**Subfamily Cicindelinae Latreille, 1802**

Tribe Amblycheilini Csiki, 1903

Tribe Cicindelini Latreille, 1802

Subtribe Apteroessina Rivalier, 1971

Subtribe Cicindelina Latreille, 1802

Subtribe Dromicina Thomson, 1859

Subtribe Iresiina Rivalier, 1971

Subtribe Theratina Horn, 1893

Tribe Collyridini Brullé, 1834

Subtribe Collyridina Brullé, 1834

Subtribe Tricondylina Naviaux, 1991

Tribe Ctenostomatini Laporte, 1834

Tribe Manticorini Laporte, 1834

Tribe Megacephalini Laporte, 1834

**Subfamily Carabinae Latreille, 1802**

Tribe Carabini Latreille, 1802

Tribe Ceroglossini Lapouge, 1927

Tribe Cychrini Perty, 1830

Tribe Pamborini Hope, 1838

**Subfamily Loricerinae Bonelli, 1810**

**Subfamily Omophroninae Bonelli, 1810**

**Subfamily Elaphrinae Latreille, 1802**

**Subfamily Migadopinae Chaudoir, 1861**

Tribe Amarotypini Erwin, 1985

Tribe Migadopini Chaudoir, 1861

Subtribe Aquilicina Moret, 2005

Subtribe Migadopina Chaudoir, 1861

**Subfamily Hiletinae Schiødte, 1848**

**Subfamily Scaritinae Bonelli, 1810**

Tribe Carenini MacLeay, 1887

Tribe Clivinini Rafinesque, 1815

Subtribe Ardistomina Putzeys, 1867

Subtribe Clivinina Rafinesque, 1815

Subtribe Forcipatorina Bänninger, 1938

Tribe Dalyatini Mateu, 2002

Tribe Dyschiriini Kolbe, 1880

†Tribe Palaeoaxinidiini McKay, 1991

Tribe Pasimachini Putzeys, 1867

Tribe Promecognathini LeConte, 1853

Tribe Salcediini Alluaud, 1930 (1929)

Subtribe Androzelmina Bell, 1998

Subtribe Salcediina Alluaud, 1930 (1929)

Subtribe Solenogenyina Bell, 1998

Tribe Scaritini Bonelli, 1810

Subtribe Acanthoscelina Csiki, 1927

Subtribe Corintascarina Basilewsky, 1973

Subtribe Dyscherina Basilewsky, 1973

Subtribe Ochyropina Basilewsky, 1973

Subtribe Oxylobina Andrewes, 1929

Subtribe Scapterina Putzeys, 1867

Subtribe Scaritina Bonelli, 1810

Subtribe Storthodontina Jeannel, 1946

**Subfamily Broscinae Hope, 1838**

Tribe Broscini Hope, 1838

Subtribe Axonyina Roig-Juñent, 2000

Subtribe Baripodina Jeannel, 1941

Subtribe Broscina Hope, 1838

Subtribe Creobiina Jeannel, 1941

Subtribe Nothobroscina Roig-Juñent, 2000

**Subfamily Apotominae LeConte, 1853**

**Subfamily Siagoninae Bonelli, 1813**

Tribe Enceladini Horn, 1881

Tribe Lupercini Lecordier, 1977

Tribe Siagonini Bonelli, 1813

**Subfamily Melaeninae Csiki, 1933**

**Subfamily Gehringiinae Darlington, 1933**

Tribe Gehringiini Darlington, 1933

Subtribe Gehringiina Darlington, 1933

Subtribe Helenaeina Deuve, 2007

**Subfamily Trechinae Bonelli, 1810**

Tribe Bembidiini Stephens, 1827

Subtribe Anillina Jeannel, 1937

Subtribe Bembidiina Stephens, 1827

Subtribe Tachyina Motschulsky, 1862

Subtribe Xystosomina Erwin, 1994

Tribe Horologionini Jeannel, 1949

Tribe Pogonini Laporte, 1834

Tribe Trechini Bonelli, 1810

Subtribe Aepina Fowler, 1887

Subtribe Cnidina Jeannel, 1958

Subtribe Perileptina Sloane, 1903

Subtribe Plocamotrechina Jeannel, 1960

Subtribe Trechina Bonelli, 1810

Subtribe Trechodina Jeannel, 1926

Tribe Zolini Sharp, 1886

Subtribe Chalteniina Roig-Juñent and Cicchino, 2001

Subtribe Sinozolina Deuve, 1997

Subtribe Zolina Sharp, 1886

**Subfamily Patrobinae Kirby, 1837**

Tribe Lissopogonini Zamotajlov, 2000

Tribe Patrobini Kirby, 1837

Subtribe Deltomerina Chaudoir, 1871

Subtribe Deltomerodina Zamotajlov, 2002

Subtribe Patrobina Kirby, 1837

Subtribe Platidiolina Zamotajlov and Lafer, 2001

**Subfamily Psydrinae LeConte, 1853**

Tribe Amblytelini Blackburn, 1892

Tribe Mecyclothoracini Jeannel, 1940

Tribe Meonini Sloane, 1898

Tribe Moriomorphini Sloane, 1890

Tribe Psydrini LeConte, 1853

Tribe Tropopterini Sloane, 1898

**Subfamily Nototylinae Bänninger, 1927**

**Subfamily Paussinae Latreille, 1806**

Tribe Metriini LeConte, 1853

Tribe Mystropomini Horn, 1881

Tribe Ozaenini Hope, 1838

Tribe Paussini Latreille, 1806

†Subtribe Arthropteritina Luna de Carvalho, 1961

Subtribe Carabidomemnina Wasmann, 1928

Subtribe Cerapterina Billberg, 1820

†Subtribe Eopaussina Luna de Carvalho, 1951

Subtribe Heteropaussina Janssens, 1950

Subtribe Homopterina Wasmann, 1920

Subtribe Paussina Latreille, 1806

Subtribe Pentaplatarthrina Jeannel, 1946

Tribe Protopaussini Gestro, 1892

**Subfamily Brachininae Bonelli, 1810**

Tribe Brachinini Bonelli, 1810

Subtribe Aptinina Gistel, 1848

Subtribe Brachinina Bonelli, 1810

Subtribe Mastacina Erwin, 1970

Subtribe Pheropsophina Jeannel, 1949

Tribe Crepidogastrini Jeannel, 1949

**Subfamily Harpalinae Bonelli, 1810**

Tribe Abacetini Chaudoir, 1873

Tribe Amorphomerini Sloane, 1923

Tribe Anthiini Bonelli, 1813

Tribe Atranini Horn, 1881

Tribe Bascanini Basilewsky, 1953

Tribe Calophaenini Jeannel, 1948

Tribe Catapieseini Bates, 1882

Tribe Chaetodactylini Tschitschérine, 1903

Tribe Chaetogenyini Emden, 1958

Tribe Chlaeniini Brullé, 1834

Subtribe Callistina Laporte, 1834

Subtribe Chlaeniina Brullé, 1834

Tribe Cnemalobini Germain, 1911

Tribe Cratocerini Lacordaire, 1854

Tribe Ctenodactylini Laporte, 1834

Tribe Cuneipectini Sloane, 1907

Tribe Cyclosomini Laporte, 1834

Subtribe Cyclosomina Laporte, 1834

Subtribe Masoreina Chaudoir, 1871

Tribe Dercylini Sloane, 1923

Tribe Drimostomatini Chaudoir, 1872

Tribe Dryptini Bonelli, 1810

Tribe Enoicini Basilewsky, 1985

Tribe Galeritini Kirby, 1825

Subtribe Galeritina Kirby, 1825

Subtribe Planetina Jedlička, 1941

Tribe Geobaenini Péringuey, 1896

Tribe Ginemini Ball and Shpeley, 2002

Tribe Glyptini Horn, 1881

Tribe Graphipterini Latreille, 1802

Tribe Harpalini Bonelli, 1810

Subtribe Anisodactylina Lacordaire, 1854 nomen protectum

Subtribe Harpalina Bonelli, 1810

Subtribe Pelmatellina Bates, 1882

Subtribe Stenolophina Kirby, 1837

Tribe Helluonini Hope, 1838

Subtribe Helluonina Hope, 1838

Subtribe Omphrina Jedlička, 1941

Tribe Hexagoniini Horn, 1881 (1834)

Tribe Idiomorphini Bates, 1891

Tribe Lachnophorini LeConte, 1853

Subtribe Lachnophorina LeConte, 1853

Subtribe Selinina Jeannel, 1948

Tribe Lebiini Bonelli, 1810

Subtribe Actenonycina Bates, 1871

Subtribe Agrina Kirby, 1837

Subtribe Apenina Ball, 1983

Subtribe Calleidina Chaudoir, 1873

Subtribe Celaenephina Habu, 1982

Subtribe Cymindidina Laporte, 1834

Subtribe Demetriadina Bates, 1886

Subtribe Dromiusina Bonelli, 1810

Subtribe Gallerucidiina Chaudoir, 1872

Subtribe Lebiina Bonelli, 1810

Subtribe Metallicina Basilewsky, 1984

Subtribe Nemotarsina Bates, 1883

Subtribe Pericalina Hope, 1838

Subtribe Pseudotrechina Basilewsky, 1984

Subtribe Sugimotoina Habu, 1975

Subtribe Trichina Basilewsky, 1984

Tribe Licinini Bonelli, 1810

Subtribe Dicaelina Laporte, 1834

Subtribe Dicrochilina Ball, 1992

Subtribe Lestignathina Ball, 1992

Subtribe Licinina Bonelli, 1810

Tribe Melanchitonini Jeannel, 1948

Tribe Microcheilini Jeannel, 1948

Tribe Morionini Brullé, 1835

Tribe Odacanthini Laporte, 1834

Tribe Omphreini Ganglbauer, 1891

Tribe Oodini LaFerté-Sénectère, 1851

Tribe Orthogoniini Schaum, 1857

Tribe Panagaeini Bonelli, 1810

Subtribe Brachygnathina Basilewsky, 1946

Subtribe Panagaeina Bonelli, 1810

Subtribe Tefflina Basilewsky, 1946

Tribe Peleciini Chaudoir, 1880

Subtribe Agonicina Sloane, 1920

Subtribe Peleciina Chaudoir, 1880

Tribe Pentagonicini Bates, 1873

Tribe Perigonini Horn, 1881 nomen protectum

Tribe Physocrotaphini Chaudoir, 1863

Tribe Platynini Bonelli, 1810

Tribe Pseudomorphini Hope, 1838

Tribe Pterostichini Bonelli, 1810

Subtribe Abacomorphina Tschitschérine, 1902

Subtribe Euchroina Chaudoir, 1874

Subtribe Metiina Straneo, 1951

Subtribe Microcephalina Tschitschérine, 1898

Subtribe Pterostichina Bonelli, 1810

Tribe Sphodrini Laporte, 1834

Subtribe Atranopsina Baehr, 1982

Subtribe Calathina Laporte, 1834

Subtribe Dolichina Brullé, 1834

Subtribe Pristosiina Lindroth, 1956

Subtribe Sphodrina Laporte, 1834

Subtribe Synuchina Lindroth, 1956

Tribe Xenaroswellianini Erwin, 2007

Tribe Zabrini Bonelli, 1810

Subtribe Amarina Zimmermann, 1832

Subtribe Zabrina Bonelli, 1810

Tribe Zuphiini Bonelli, 1810

Subtribe Dicrodontina Machado, 1992

Subtribe Leleupidiina Basilewsky, 1951

Subtribe Metazuphiina Mateu, 1992

Subtribe Mischocephalina Mateu, 1992

Subtribe Patriziina Basilewsky, 1953

Subtribe Zuphiina Bonelli, 1810

**Family Haliplidae Aubé, 1836**

**†Family Triaplidae Ponomarenko, 1977**

**†Family Colymbotethidae Ponomarenko, 1994**

**†Family Parahygrobiidae Ponomarenko, 1977**

**†Family Coptoclavidae Ponomarenko, 1961**

**†Subfamily Necronectinae Ponomarenko, 1977**

**†Subfamily Charonoscaphinae Ponomarenko, 1977**

**†Subfamily Coptoclavinae Ponomarenko, 1961**

**†Subfamily Coptoclaviscinae Soriano, Ponomarenko and Delclos, 2007**

**†Subfamily Hispanoclavinae Soriano, Ponomarenko and Delclos, 2007**

**†Family Liadytidae Ponomarenko, 1977**

**Family Meruidae Spangler and Steiner, 2005**

**Family Noteridae Thomson, 1860**

**Subfamily Noterinae Thomson, 1860**

Tribe Neohydrocoptini Zalat, Saleh, Angus and Kaschef, 2000

Tribe Noterini Thomson, 1860

Tribe Pronoterini Nilsson, 2005

Tribe Tonerini Miller, 2009

**Subfamily Notomicrinae Zimmermann, 1919**

**Subfamily Phreatodytinae Uéno, 1957**

**Family Amphizoidae LeConte, 1853**

**Family Aspidytidae Ribera, Beutel, Balke and Vogler, 2002**

**Family Hygrobiidae Régimbart, 1879 (1837)**

**Family Dytiscidae Leach, 1815**

**Subfamily Agabinae Thomson, 1867**

**Subfamily Colymbetinae Erichson, 1837**

Tribe Anisomeriini Brinck, 1948

Tribe Carabdytini Pederzani, 1995

Tribe Colymbetini Erichson, 1837

**Subfamily Copelatinae Branden, 1885**

**Subfamily Coptotominae Branden, 1885**

**Subfamily Dytiscinae Leach, 1815**

Tribe Aciliini Thomson, 1867

Tribe Aubehydrini Guignot, 1942

Tribe Cybisterini Sharp, 1880

Tribe Dytiscini Leach, 1815

Tribe Eretini Crotch, 1873

Tribe Hydaticini Sharp, 1880

Tribe Hyderodini Miller, 2000

**Subfamily Hydrodytinae Miller, 2001**

**Subfamily Hydroporinae Aubé, 1836**

Tribe Bidessini Sharp, 1880

Tribe Carabhydrini Watts, 1978

Tribe Hydroporini Aubé, 1836

Tribe Hydrovatini Sharp, 1880

Tribe Hygrotini Portevin, 1929

Tribe Hyphydrini Gistel, 1848

Tribe Laccornini Wolfe and Roughley, 1990

Tribe Methlini Branden, 1885

†Tribe Schistomerini Palmer, 1957

Tribe Vatellini Sharp, 1880

**Subfamily Laccophilinae Gistel, 1848**

Tribe Agabetini Branden, 1885

Tribe Laccophilini Gistel, 1848

**Subfamily Lancetinae Branden, 1885**

**Subfamily Matinae Branden, 1885**

**†Subfamily Palaeogyrininae Schlechtendal, 1894**

**†Subfamily Liadytiscinae Prokin and Ren, 2010**

**Suborder POLYPHAGA**

**Series STAPHYLINIFORMIA**

**Superfamily Hydrophiloidea Latreille, 1802**

**Family Hydrophilidae Latreille, 1802**

**Subfamily Helophorinae Leach, 1815**

**Subfamily Epimetopinae Zaitzev, 1908**

**Subfamily Georissinae Laporte, 1840**

**Subfamily Hydrochinae Thomson, 1859**

**Subfamily Spercheinae Erichson, 1837**

**Subfamily Horelophinae Hansen, 1991**

**Subfamily Horelophopsinae Hansen, 1997**

**Subfamily Hydrophilinae Latreille, 1802**

Tribe Anacaenini Hansen, 1991

Tribe Berosini Mulsant, 1844

Tribe Chaetarthriini Bedel, 1881

Tribe Hydrophilini Latreille, 1802

Subtribe Acidocerina Zaitzev, 1908

Subtribe Globuloseina García, 2001

Subtribe Hydrobiusina Mulsant, 1844

Subtribe Hydrophilina Latreille, 1802

Tribe Laccobiini Houlbert, 1922

Tribe Sperchopsini Hansen, 1991

**Subfamily Sphaeridiinae Latreille, 1802**

Tribe Andotypini Hansen, 1991

Tribe Borborophorini Hansen, 1991

Tribe Coelostomatini Heyden, 1891 (1890)

Tribe Megasternini Mulsant, 1844

Tribe Omicrini Smetana, 1975

Tribe Protosternini Hansen, 1991

Tribe Rygmodini Orchymont, 1916

Tribe Sphaeridiini Latreille, 1802

Tribe Tormissini Hansen, 1991

**Family Sphaeritidae Shuckard, 1839**

**Family Synteliidae Lewis, 1882**

**Family Histeridae Gyllenhal, 1808**

**Subfamily Niponiinae Fowler, 1912**

**Subfamily Abraeinae MacLeay, 1819**

Tribe Abraeini MacLeay, 1819

Tribe Acritini Wenzel, 1944

Tribe Acritomorphini Wenzel, 1944

Tribe Plegaderini Portevin, 1929

Tribe Teretriini Bickhardt, 1914

**Subfamily Trypeticinae Bickhardt, 1913**

**Subfamily Trypanaeinae Marseul, 1857**

**Subfamily Saprininae Blanchard, 1845**

**Subfamily Dendrophilinae Reitter, 1909**

Tribe Anapleini Olexa, 1982

Tribe Bacaniini Kryzhanovskij, 1976

Tribe Dendrophilini Reitter, 1909

Tribe Paromalini Reitter, 1909

**Subfamily Onthophilinae MacLeay, 1819**

**Subfamily Tribalinae Bickhardt, 1914**

**Subfamily Histerinae Gyllenhal, 1808**

Tribe Exosternini Bickhardt, 1914

Tribe Histerini Gyllenhal, 1808

Tribe Hololeptini Hope, 1840

Tribe Omalodini Kryzhanovskij, 1972

Tribe Platysomatini Bickhardt, 1914

**Subfamily Haeteriinae Marseul, 1857**

Tribe Haeteriini Marseul, 1857

Tribe Nymphistrini Tishechkin, 2007

Tribe Synoditulini Tishechkin, 2007

**Subfamily Chlamydopsinae Bickhardt, 1914**

**Superfamily Staphylinoidea Latreille, 1802**

**Family Hydraenidae Mulsant, 1844**

**Subfamily Orchymontiinae Perkins, 1997**

**Subfamily Prosthetopinae Perkins, 1994**

Tribe Coelometoponini Perkins, 2005

Tribe Nucleotopini Perkins, 1994

Tribe Parasthetopini Perkins, 1994

Tribe Prosthetopini Perkins, 1994

Tribe Protosthetopini Perkins, 1994

Tribe Pterosthetopini Perkins, 1994

**Subfamily Hydraeninae Mulsant, 1844**

Tribe Hydraenidini Perkins, 1980

Tribe Hydraenini Mulsant, 1844

Tribe Limnebiini Mulsant, 1844

Tribe Madagastrini Perkins, 1997

Tribe Parhydraenini Perkins, 1997

**Subfamily Ochthebiinae Thomson, 1859**

Tribe Ochthebiini Thomson, 1859

Subtribe Enicocerina Perkins, 1997

Subtribe Meropathina Perkins, 1997

Subtribe Neochthebiina Perkins, 1997

Subtribe Ochthebiina Thomson, 1859

Subtribe Protochthebiina Perkins, 1997

Tribe Ochtheosini Perkins, 1997

**Family Ptiliidae Erichson, 1845**

**Subfamily Ptiliinae Erichson, 1845**

Tribe Discheramocephalini Grebennikov, 2009

Tribe Nanosellini Barber, 1924

Tribe Ptenidiini Flach, 1889

Tribe Ptiliini Erichson, 1845

Tribe Ptinellini Reitter, 1906 (1891)

**Subfamily Cephaloplectinae Sharp, 1883**

**Subfamily Acrotrichinae Reitter, 1909 (1856)**

**Family Agyrtidae Thomson, 1859**

**Subfamily Agyrtinae Thomson, 1859**

**Subfamily Necrophilinae Newton, 1997**

**Subfamily Pterolomatinae Thomson, 1862**

**Family Leiodidae Fleming, 1821**

**Subfamily Camiarinae Jeannel, 1911**

Tribe Agyrtodini Jeannel, 1936

Tribe Camiarini Jeannel, 1911

Tribe Neopelatopini Jeannel, 1962

**Subfamily Catopocerinae Hatch, 1927 (1880)**

Tribe Catopocerini Hatch, 1927 (1880)

Tribe Glacicavicolini Westcott, 1968

**Subfamily Leiodinae Fleming, 1821**

Tribe Agathidiini Westwood, 1838

Tribe Estadiini Portevin, 1914

Tribe Leiodini Fleming, 1821

Tribe Pseudoliodini Portevin, 1926

Tribe Scotocryptini Reitter, 1884

Tribe Sogdini Lopatin, 1961

**Subfamily Coloninae Horn, 1880 (1859)**

**Subfamily Cholevinae Kirby, 1837**

Tribe Anemadini Hatch, 1928

Subtribe Anemadina Hatch, 1928

Subtribe Eocatopina Jeannel, 1936

Subtribe Eunemadina Newton, 1998

Subtribe Nemadina Jeannel, 1936

Subtribe Paracatopina Jeannel, 1936

Tribe Cholevini Kirby, 1837

Subtribe Catopina Chaudoir, 1845

Subtribe Cholevina Kirby, 1837

Tribe Eucatopini Jeannel, 1921

Tribe Leptodirini Lacordaire, 1854 (1849)

Subtribe Anthroherponina Jeannel, 1910

Subtribe Bathysciina Horn, 1880

Subtribe Bathysciotina Guéorguiev, 1974

Subtribe Leptodirina Lacordaire, 1854 (1849)

Subtribe Pholeuina Reitter, 1886

Subtribe Platycholeina Horn, 1880

Subtribe Spelaeobatina Guéorguiev, 1974

Tribe Oritocatopini Jeannel, 1936

Tribe Ptomaphagini Jeannel, 1911

Subtribe Baryodirina Perreau, 2000

Subtribe Ptomaphagina Jeannel, 1911

Subtribe Ptomaphaginina Szymczakowski, 1964

Tribe Sciaphyini Perreau, 2000

**Subfamily Platypsyllinae Ritsema, 1869**

**Family Silphidae Latreille, 1806**

**Subfamily Silphinae Latreille, 1806**

**Subfamily Nicrophorinae Kirby, 1837**

**Family Staphylinidae Latreille, 1802**

**Subfamily Glypholomatinae Jeannel, 1962**

**Subfamily Microsilphinae Crowson, 1950**

**Subfamily Omaliinae MacLeay, 1825**

Tribe Anthophagini Thomson, 1859

Tribe Aphaenostemmini Peyerimhoff, 1914

Tribe Corneolabiini Steel, 1950

Tribe Coryphiini Jakobson, 1908

Subtribe Boreaphilina Zerche, 1990

Subtribe Coryphiina Jakobson, 1908

Tribe Eusphalerini Hatch, 1957

Tribe Hadrognathini Portevin, 1929

Tribe Omaliini MacLeay, 1825

**Subfamily Empelinae Newton and Thayer, 1992**

**Subfamily Proteininae Erichson, 1839**

Tribe Anepiini Steel, 1966

Tribe Austrorhysini Newton and Thayer, 1995

Tribe Nesoneini Steel, 1966

Tribe Proteinini Erichson, 1839

Tribe Silphotelini Newton and Thayer, 1995

**Subfamily Micropeplinae Leach, 1815**

**Subfamily Neophoninae Fauvel, 1905**

**Subfamily Dasycerinae Reitter, 1887**

**Subfamily Protopselaphinae Newton and Thayer, 1995**

**Subfamily Pselaphinae Latreille, 1802**

**Supertribe Batrisitae Reitter, 1882**

Tribe Amauropini Jeannel, 1948

Tribe Batrisini Reitter, 1882

Subtribe Ambicocerina Leleup, 1970

Subtribe Batrisina Reitter, 1882

Subtribe Leupeliina Jeannel, 1954

Subtribe Stilipalpina Jeannel, 1954

Tribe Thaumastocephalini Poggi, Nonveiller, Colla, Pavićević and Rada, 2001

**Supertribe Clavigeritae Leach, 1815**

Tribe Clavigerini Leach, 1815

Subtribe Apoderigerina Jeannel, 1954

Subtribe Clavigerina Leach, 1815

Subtribe Clavigerodina Schaufuss, 1882

Subtribe Disarthricerina Jeannel, 1949

Subtribe Hoplitoxenina Célis, 1969

Subtribe Lunillina Célis, 1969

Subtribe Mastigerina Jeannel, 1954

Subtribe Miroclavigerina Jeannel, 1949

Subtribe Neocerina Jeannel, 1954

Subtribe Radamina Jeannel, 1954

Subtribe Thysdariina Jeannel, 1954

Tribe Colilodionini Besuchet, 1991

Tribe Tiracerini Besuchet, 1986

**Supertribe Euplectitae Streubel, 1839**

Tribe Bythinoplectini Schaufuss, 1890

Subtribe Bythinoplectina Schaufuss, 1890

Subtribe Pyxidicerina Raffray, 1904

Tribe Dimerini Raffray, 1908

Tribe Euplectini Streubel, 1839

Tribe Jubini Raffray, 1904

Tribe Mayetiini Winkler, 1925

Tribe Metopiasini Raffray, 1904

Subtribe Metopiasina Raffray, 1904

Subtribe Rhinoscepsina Bowman, 1934

Tribe Trichonychini Reitter, 1882

Subtribe Bibloporina Park, 1951

Subtribe Panaphantina Jeannel, 1950

Subtribe Trichonychina Reitter, 1882

Subtribe Trimiina Bowman, 1934

Tribe Trogastrini Jeannel, 1949

Subtribe Phtegnomina Park, 1951

Subtribe Rhexiina Park, 1951

Subtribe Trogastrina Jeannel, 1949

**Supertribe Faronitae Reitter, 1882**

**Supertribe Goniaceritae Reitter, 1882 (1872)**

Tribe Arnylliini Jeannel, 1952

Tribe Barrosellini Leleup, 1973

Tribe Brachyglutini Raffray, 1904

Subtribe Baradina Park, 1951

Subtribe Brachyglutina Raffray, 1904

Subtribe Decarthrina Park, 1951

Subtribe Eupseniina Park, 1951

Tribe Bythinini Raffray, 1890

Subtribe Bythinina Raffray, 1890

Subtribe Machaeritina Jeannel, 1950

Subtribe Xenobythina Jeannel, 1950

Tribe Cyathigerini Schaufuss, 1872

Tribe Goniacerini Reitter, 1882 (1872)

Tribe Imirini Jeannel, 1949

Tribe Iniocyphini Park, 1951

Subtribe Iniocyphina Park, 1951

Subtribe Natypleurina Newton and Thayer, 1992

Tribe Machadoini Jeannel, 1951

Tribe Proterini Jeannel, 1949

Tribe Pygoxyini Reitter, 1909

Tribe Speleobamini Park, 1951

Tribe Tychini Raffray, 1904

Tribe Valdini Park, 1953

**Supertribe Pselaphitae Latreille, 1802**

Tribe Arhytodini Raffray, 1890

Tribe Attapseniini Bruch, 1933

Tribe Ctenistini Blanchard, 1845

Tribe Hybocephalini Raffray, 1890

Tribe Odontalgini Jeannel, 1949

Tribe Pachygastrodini Leleup, 1969

Tribe Phalepsini Jeannel, 1949

Tribe Pselaphini Latreille, 1802

Tribe Schistodactylini Raffray, 1890

Tribe Tmesiphorini Jeannel, 1949

Tribe Tyrini Reitter, 1882

Subtribe Centrophthalmina Jeannel, 1949

Subtribe Janusculina Cerruti, 1970

Subtribe Somatipionina Jeannel, 1949

Subtribe Tyrina Reitter, 1882

**Subfamily Phloeocharinae Erichson, 1839**

**Subfamily Olisthaerinae Thomson, 1858**

**Subfamily Tachyporinae MacLeay, 1825**

Tribe Deropini Smetana, 1983

Tribe Megarthropsini Cameron, 1919

Tribe Mycetoporini Thomson, 1859

Tribe Tachyporini MacLeay, 1825

Tribe Vatesini Seevers, 1958

**Subfamily Trichophyinae Thomson, 1858**

**Subfamily Habrocerinae Mulsant and Rey, 1876**

**Subfamily Aleocharinae Fleming, 1821**

Tribe Actocharini Bernhauer and Schubert, 1911

Tribe Aenictoteratini Kistner, 1993

Tribe Akatastopsisini Pace, 2000

Tribe Aleocharini Fleming, 1821

Subtribe Aleocharina Fleming, 1821

Subtribe Compactopediina Kistner, 1970

Subtribe Hodoxenina Kistner, 1970

Tribe Athetini Casey, 1910

Subtribe Athetina Casey, 1910

Subtribe Coptotermoeciina Kistner and Pasteels, 1970

Subtribe Microceroxenina Kistner, 1970

Subtribe Nasutiphilina Kistner, 1970

Subtribe Schistogeniina Fenyes, 1918

Subtribe Taxicerina Lohse, 1989

Subtribe Termitotelina Kistner, 1970

Subtribe Thamiaraeina Fenyes, 1921

Tribe Autaliini Thomson, 1859

Tribe Cordobanini Bernhauer, 1910

Tribe Corotocini Fenyes, 1918

Subtribe Abrotelina Seevers, 1957

Subtribe Corotocina Fenyes, 1918

Subtribe Eburniogastrina Jacobson, Kistner and Pasteels, 1986

Subtribe Nasutitellina Jacobson, Kistner and Pasteels, 1986

Subtribe Sphuridaethina Pace, 1988

Subtribe Termitocharina Seevers, 1957

Subtribe Termitocupidina Jacobson, Kistner and Pasteels, 1986

Subtribe Termitogastrina Bernhauer and Scheerpeltz, 1926

Subtribe Termitoiceina Jacobson, Kistner and Pasteels, 1986

Subtribe Termitopithina Jacobson, Kistner and Pasteels, 1986

Subtribe Termitoptochina Fenyes, 1921

Subtribe Timeparthenina Fenyes, 1921

Tribe Crematoxenini Mann, 1921

Tribe Cryptonotopseini Pace, 2003

Tribe Deinopsini Sharp, 1883

Tribe Diestotini Mulsant and Rey, 1871

Tribe Diglottini Jakobson, 1909

Tribe Digrammini Fauvel, 1900

Tribe Dorylogastrini Wasmann, 1916

Tribe Dorylomimini Wasmann, 1916

Tribe Drepanoxenini Kistner and Watson, 1972

Tribe Ecitocharini Seevers, 1965

Tribe Ecitogastrini Fenyes, 1918

Tribe Eusteniamorphini Bernhauer and Scheerpeltz, 1926

Tribe Falagriini Mulsant and Rey, 1873

Tribe Feldini Kistner, 1972

Tribe Gymnusini Heer, 1839

Tribe Himalusini Klimaszewski, Pace and Center, 2010

Tribe Homalotini Heer, 1839

Subtribe Bolitocharina Thomson, 1859

Subtribe Dinardopsina Bernhauer and Scheerpeltz, 1926

Subtribe Gyrophaenina Kraatz, 1856

Subtribe Homalotina Heer, 1839

Subtribe Silusina Fenyes, 1918

Tribe Hoplandriini Casey, 1910

Subtribe Hoplandriina Casey, 1910

Subtribe Platandriina Hanley, 2002

Subtribe Pseudoplandriina Hanley, 2002

Tribe Hygronomini Thomson, 1859

Subtribe Hygronomina Thomson, 1859

Subtribe Saphoglossina Bernhauer and Scheerpeltz, 1926

Tribe Hypocyphtini Laporte, 1835

Tribe Leucocraspedini Fenyes, 1921

Tribe Liparocephalini Fenyes, 1918

Tribe Lomechusini Fleming, 1821

Subtribe Aenictobiina Kistner, 1997

Subtribe Lomechusina Fleming, 1821

Subtribe Myrmedoniina Thomson, 1867

Subtribe Termitozyrina Seevers, 1957

Tribe Masuriini Cameron, 1939

Tribe Mesoporini Cameron, 1959

Tribe Mimanommatini Wasmann, 1912

Subtribe Dorylophilina Fenyes, 1921

Subtribe Mimanommatina Wasmann, 1912

Tribe Mimecitini Wasmann, 1917

Subtribe Labidopullina Jacobson and Kistner, 1991

Subtribe Leptanillophilina Fenyes, 1918

Subtribe Mimecitina Wasmann, 1917

Subtribe Mimonillina Bernhauer and Scheerpeltz, 1926

Tribe Myllaenini Ganglbauer, 1895

Tribe Oxypodini Thomson, 1859

Subtribe Aphytopodina Bernhauer and Scheerpeltz, 1926

Subtribe Blepharhymenina Klimaszewski and Peck, 1986

Subtribe Dinardina Mulsant and Rey, 1873

Subtribe Meoticina Seevers, 1978

Subtribe Oxypodina Thomson, 1859

Subtribe Tachyusina Thomson, 1859

Tribe Oxypodinini Fenyes, 1921

Tribe Paglini Newton and Thayer, 1992

Tribe Paradoxenusini Bruch, 1937

Tribe Pediculotini Ádám, 1987

Tribe Philotermitini Seevers, 1957

Tribe Phyllodinardini Wasmann, 1916

Tribe Phytosini Thomson, 1867

Tribe Placusini Mulsant and Rey, 1871

Tribe Pronomaeini Mulsant and Rey, 1873

Tribe Pseudoperinthini Cameron, 1939

Tribe Pygostenini Fauvel, 1899

Tribe Sahlbergiini Kistner, 1993

Tribe Sceptobiini Seevers, 1978

Tribe Skatitoxenini Kistner and Pasteels, 1969

Tribe Termitodiscini Wasmann, 1904

Subtribe Athexeniina Pace, 2000

Subtribe Termitodiscina Wasmann, 1904

Tribe Termitohospitini Seevers, 1941

Subtribe Hetairotermitina Seevers, 1957

Subtribe Termitohospitina Seevers, 1941

Tribe Termitonannini Fenyes, 1918

Subtribe Perinthina Bernhauer and Scheerpeltz, 1926

Subtribe Termitonannina Fenyes, 1918

Tribe Termitopaediini Seevers, 1957

Tribe Termitusini Fenyes, 1918

Subtribe Termitospectrina Seevers, 1957

Subtribe Termitusina Fenyes, 1918

Tribe Trichopseniini LeConte and Horn, 1883

Tribe Trilobitideini Fauvel, 1899

**Subfamily Trigonurinae Reiche, 1866**

**Subfamily Apateticinae Fauvel, 1895**

**Subfamily Scaphidiinae Latreille, 1806**

Tribe Cypariini Achard, 1924

Tribe Scaphidiini Latreille, 1806

Tribe Scaphiini Achard, 1924

Tribe Scaphisomatini Casey, 1893

**Subfamily Piestinae Erichson, 1839**

**Subfamily Osoriinae Erichson, 1839**

Tribe Eleusinini Sharp, 1887

Tribe Leptochirini Sharp, 1887

Tribe Osoriini Erichson, 1839

Subtribe Osoriina Erichson, 1839

Subtribe Parosoriina Bernhauer and Schubert, 1911

Tribe Thoracophorini Reitter, 1909

Subtribe Clavilispinina Newton and Thayer, 1992

Subtribe Glyptomina Newton and Thayer, 1992

Subtribe Lispinina Bernhauer and Schubert, 1910

Subtribe Thoracophorina Reitter, 1909

**Subfamily Oxytelinae Fleming, 1821**

Tribe Blediini Ádám, 2001

Tribe Coprophilini Heer, 1839

Tribe EuphaniiniReitter, 1909

Tribe Oxytelini Fleming, 1821

Tribe Planeustomini Jacquelin du Val, 1857

**Subfamily Oxyporinae Fleming, 1821**

**Subfamily Megalopsidiinae Leng, 1920**

**Subfamily Scydmaeninae Leach, 1815**

**†Supertribe Hapsomelitae Poinar and Brown, 2004**

**Supertribe Mastigitae Fleming, 1821**

Tribe Clidicini Casey, 1897

Tribe Leptomastacini Casey, 1897

Tribe Mastigini Fleming, 1821

**Supertribe Scydmaenitae Leach, 1815**

Tribe Cephenniini Reitter, 1882

Tribe Chevrolatiini Reitter, 1882

Tribe Cyrtoscydmini Schaufuss, 1889

Tribe Eutheiini Casey, 1897

Tribe Leptoscydmini Casey, 1897

Tribe Plaumanniolini Costa Lima, 1962

Tribe Scydmaenini Leach, 1815

**Subfamily Steninae MacLeay, 1825**

**Subfamily Euaesthetinae Thomson, 1859**

Tribe Alzadaesthetini Scheerpeltz, 1974

Tribe Austroesthetini Cameron, 1944

Tribe Euaesthetini Thomson, 1859

Tribe Fenderiini Scheerpeltz, 1974

Tribe Nordenskioldiini Bernhauer and Schubert, 1911

Tribe Stenaesthetini Bernhauer and Schubert, 1911

**Subfamily Solieriinae Newton and Thayer, 1992**

**Subfamily Leptotyphlinae Fauvel, 1874**

Tribe Cephalotyphlini Coiffait, 1963

Tribe Entomoculiini Coiffait, 1957

Tribe Leptotyphlini Fauvel, 1874

Tribe Metrotyphlini Coiffait, 1963

Tribe Neotyphlini Coiffait, 1963

**Subfamily Pseudopsinae Ganglbauer, 1895**

**Subfamily Paederinae Fleming, 1821**

Tribe Paederini Fleming, 1821

Subtribe Astenina Hatch, 1957

Subtribe Cryptobiina Casey, 1905

Subtribe Cylindroxystina Bierig, 1943

Subtribe Dolicaonina Casey, 1905

Subtribe Echiasterina Casey, 1905

Subtribe Lathrobiina Laporte, 1835

Subtribe Lithocharina Casey, 1905

Subtribe Medonina Casey, 1905

Subtribe Paederina Fleming, 1821

Subtribe Scopaeina Mulsant and Rey, 1878

Subtribe Stilicina Casey, 1905

Subtribe Stilicopsina Casey, 1905

Tribe Pinophilini Nordmann, 1837

Subtribe Pinophilina Nordmann, 1837

Subtribe Procirrina Bernhauer and Schubert, 1912

**Subfamily Staphylininae Latreille, 1802**

Tribe Arrowinini Solodovnikov and Newton, 2005

Tribe Diochini Casey, 1906

Tribe Maorothiini Assing, 2000

Tribe Othiini Thomson, 1859

Tribe Platyprosopini Lynch Arribálzaga, 1884

Tribe Staphylinini Latreille, 1802

Subtribe Amblyopinina Seevers, 1944

Subtribe Anisolinina Hayashi, 1993

Subtribe Eucibdelina Sharp, 1889

Subtribe Hyptiomina Casey, 1906

Subtribe Philonthina Kirby, 1837

Subtribe Quediina Kraatz, 1857

Subtribe Staphylinina Latreille, 1802

Subtribe Tanygnathinina Reitter, 1909

Subtribe Xanthopygina Sharp, 1884

Tribe Xantholinini Erichson, 1839

**†Subfamily Protactinae Heer, 1847**

**Series SCARABAEIFORMIA**

**Superfamily Scarabaeoidea Latreille, 1802**

**Family Pleocomidae LeConte, 1861**

**Subfamily Pleocominae LeConte, 1861**

**†Subfamily Cretocominae Nikolajev, 2002**

**†Subfamily Archescarabaeinae Nikolajev, 2010**

**Family Geotrupidae Latreille, 1802**

**Subfamily Taurocerastinae Germain, 1897**

**Subfamily Bolboceratinae Mulsant, 1842**

Tribe Athyreini Lynch Arribálzaga, 1878

Tribe Bolbelasmini Nikolajev, 1996

Tribe Bolboceratini Mulsant, 1842

Tribe Bolbochromini Nikolajev, 1970

Tribe Eubolbitini Nikolajev, 1970

Tribe Eucanthini Nikolajev, 2003

Tribe Gilletinini Nikolajev, 1990

Tribe Odonteini Shokhin, 2007

Tribe Stenaspidiini Nikolajev, 2003

**Subfamily Geotrupinae Latreille, 1802**

Tribe Ceratotrupini Zunino, 1984

Tribe Enoplotrupini Paulian, 1945

†Tribe Cretogeotrupini Nikolajev, 1996

Tribe Geotrupini Latreille, 1802

Tribe Lethrini Oken, 1843

**Family Belohinidae Paulian, 1959**

**Family Passalidae Leach, 1815**

**Subfamily Aulacocyclinae Kaup, 1868**

Tribe Aulacocyclini Kaup, 1868

Tribe Ceracupedini Boucher, 2006

**Subfamily Passalinae Leach, 1815**

Tribe Leptaulacini Kaup, 1871

Tribe Macrolinini Kaup, 1871

Tribe Passalini Leach, 1815

Tribe Proculini Kaup, 1868

Tribe Solenocyclini Kaup, 1871

**Family Trogidae MacLeay, 1819**

**†Subfamily Avitortorinae Nikolajev, 2007**

**Subfamily Troginae MacLeay, 1819**

**Subfamily Omorginae Nikolajev, 2005**

**Family Glaresidae Kolbe, 1905**

**Family Diphyllostomatidae Holloway, 1972**

**Family Lucanidae Latreille, 1804**

**†Subfamily Protolucaninae Nikolajev, 2007**

**Subfamily Aesalinae MacLeay, 1819**

Tribe Aesalini MacLeay, 1819

Tribe Ceratognathini Sharp, 1899

Tribe Nicagini LeConte, 1861

**†Subfamily Ceruchitinae Nikolajev, 2006**

**Subfamily Syndesinae MacLeay, 1819**

**Subfamily Lampriminae MacLeay, 1819**

Tribe Lamprimini MacLeay, 1819

Tribe Streptocerini Kikuta, 1986

**Subfamily Lucaninae Latreille, 1804**

Tribe Chiasognathini Burmeister, 1847

Tribe Lucanini Latreille, 1804

Tribe Platycerini Mulsant, 1842

Tribe Platyceroidini Paulsen and Hawks, 2008

**†Subfamily Paralucaninae Nikolajev, 2000**

**Family Ochodaeidae Mulsant and Rey, 1871**

**†Subfamily Cretochodaeinae Nikolajev, 1995**

**Subfamily Ochodaeinae Mulsant and Rey, 1871**

Tribe Enodognathini Scholtz, 1988

Tribe Ochodaeini Mulsant and Rey, 1871

**Subfamily Chaetocanthinae Scholtz, 1988**

Tribe Chaetocanthini Scholtz, 1988

Tribe Pseudochodaeini Scholtz, 1988

Tribe Synochodaeini Scholtz, 1988

**Family Hybosoridae Erichson, 1847**

**†Subfamily Mimaphodiinae Nikolajev, 2007**

**Subfamily Anaidinae Nikolajev, 1996**

**Subfamily Ceratocanthinae Martínez, 1968**

Tribe Ceratocanthini Martínez, 1968

Tribe Ivieolini Howden and Gill, 2000

Tribe Scarabatermitini Nikolajev, 1999

**Subfamily Hybosorinae Erichson, 1847**

**Subfamily Liparochrinae Ocampo, 2006**

**Subfamily Pachyplectrinae Ocampo, 2006**

**Family Glaphyridae MacLeay, 1819**

**Subfamily Glaphyrinae MacLeay, 1819**

**Subfamily Amphicominae Blanchard, 1845**

**†Subfamily Cretoglaphyrinae Nikolajev, 2005**

**Family Scarabaeidae Latreille, 1802**

**†Subfamily Lithoscarabaeinae Nikolajev, 1992**

**Subfamily Chironinae Blanchard, 1845**

**Subfamily Aegialiinae Laporte, 1840**

**Subfamily Eremazinae Iablokoff-Khnzorian, 1977**

**Subfamily Aphodiinae Leach, 1815**

Tribe Aphodiini Leach, 1815

Subtribe Aphodiina Leach, 1815

Subtribe Didactyliina Pittino, 1985

Subtribe Proctophanina Stebnicka and Howden, 1995

Tribe Corythoderini Schmidt, 1910

Tribe Eupariini Schmidt, 1910 nomen protectum

Tribe Odontolochini Stebnicka and Howden, 1996

Tribe Odochilini Rakovič, 1987

Tribe Psammodiini Mulsant, 1842

Subtribe Phycocina Landin, 1960

Subtribe Psammodiina Mulsant, 1842

Subtribe Rhyssemina Pittino and Mariani, 1986

Tribe Rhyparini Schmidt, 1910

Tribe Stereomerini Howden and Storey, 1992

Tribe Termitoderini Tangelder and Krikken, 1982

**Subfamily Aulonocneminae Janssens, 1946**

**Subfamily Termitotroginae Wasmann, 1918**

**Subfamily Scarabaeinae Latreille, 1802**

Tribe Ateuchini Perty, 1830

Subtribe Ateuchina Perty, 1830

Subtribe Scatimina Vaz-de-Mello, 2008

Tribe Coprini Leach, 1815

Tribe Deltochilini Lacordaire, 1856

Tribe Eucraniini Burmeister, 1873

Tribe Gymnopleurini Lacordaire, 1856

Tribe Oniticellini Kolbe, 1905

Subtribe Drepanocerina van Lansberge, 1875

Subtribe Eurysternina Vulcano, Martínez and Pereira, 1961

Subtribe Helictopleurina Janssens, 1946

Subtribe Oniticellina Kolbe, 1905

Tribe Onitini Laporte, 1840

Tribe Onthophagini Burmeister, 1846

Tribe Phanaeini Hope, 1838

Tribe Scarabaeini Latreille, 1802

Tribe Sisyphini Mulsant, 1842

**†Subfamily Prototroginae Nikolajev, 2000**

**†Subfamily Cretoscarabaeinae Nikolajev, 1995**

**Subfamily Dynamopodinae Arrow, 1911**

Tribe Dynamopodini Arrow, 1911

Tribe Thinorycterini Semenov and Reichardt, 1925

**Subfamily Phaenomeridinae Erichson, 1847**

**Subfamily Orphninae Erichson, 1847**

Tribe Aegidiini Paulian, 1984

Tribe Orphnini Erichson, 1847

**Subfamily Allidiostomatinae Arrow, 1940**

**Subfamily Aclopinae Blanchard, 1850**

Tribe Aclopini Blanchard, 1850

†Tribe Holcorobeini Nikolajev, 1992

Tribe Phaenognathini Iablokoff-Khnzorian, 1977

**Subfamily Melolonthinae Leach, 1819**

Tribe Ablaberini Blanchard, 1850

Tribe Automoliini Britton, 1978

Tribe Chasmatopterini Lacordaire, 1856

Tribe Colymbomorphini Blanchard, 1850

Tribe Comophorinini Britton, 1957

†Tribe Cretomelolonthini Nikolajev, 1998

Tribe Dichelonychini Burmeister, 1855

Tribe Diphucephalini Laporte, 1840

Tribe Diphycerini Medvedev, 1952

Tribe Diplotaxini Kirby, 1837

Tribe Euchirini Hope, 1840

Tribe Heteronychini Lacordaire, 1856

Tribe Hopliini Latreille, 1829

Subtribe Hopliina Latreille, 1829

Subtribe Pachycnemina Laporte, 1840

Tribe Lichniini Burmeister, 1844

Tribe Liparetrini Burmeister, 1855

Tribe Macrodactylini Kirby, 1837

Tribe Maechidiini Burmeister, 1855

Tribe Melolonthini Leach, 1819

Subtribe Enariina Dewailly, 1950

Subtribe Heptophyllina Medvedev, 1951

Subtribe Leucopholina Burmeister, 1855

Subtribe Melolonthina Leach, 1819

Subtribe Pegylina Lacroix, 1989

Subtribe Rhizotrogina Burmeister, 1855

Subtribe Schizonychina Burmeister, 1855

Tribe Oncerini LeConte, 1861

Tribe Pachypodini Erichson, 1840

Tribe Pachytrichini Burmeister, 1855

Tribe Phyllotocidiini Britton, 1957

Tribe Podolasiini Howden, 1997

Tribe Scitalini Britton, 1957

Tribe Sericini Kirby, 1837

Subtribe Phyllotocina Burmeister, 1855

Subtribe Sericina Kirby, 1837

Subtribe Trochalina Brenske, 1898

Tribe Sericoidini Erichson, 1847

Tribe Systellopini Sharp, 1877

Tribe Tanyproctini Erichson, 1847

Subtribe Macrophyllina Burmeister, 1855

Subtribe Tanyproctina Erichson, 1847

**Subfamily Rutelinae MacLeay, 1819**

Tribe Adoretini Burmeister, 1844

Subtribe Adoretina Burmeister, 1844

Subtribe Adorrhinyptiina Arrow, 1917

Subtribe Pachyrhinadoretina Ohaus, 1912

Subtribe Prodoretina Ohaus, 1912

Subtribe Trigonostomusina Ohaus, 1912

Tribe Alvarengiini Frey, 1975

Tribe Anatistini Lacordaire, 1856

Tribe Anomalini Streubel, 1839 nomen protectum

Subtribe Anisopliina Burmeister, 1844

Subtribe Anomalina Streubel, 1839 nomen protectum

Subtribe Isopliina Péringuey, 1902

Subtribe Leptohopliina Potts, 1974

Subtribe Popilliina Ohaus, 1918

Tribe Anoplognathini MacLeay, 1819

Subtribe Anoplognathina MacLeay, 1819

Subtribe Brachysternina Burmeister, 1844

Subtribe Phalangogoniina Ohaus, 1918

Subtribe Platycoeliina Burmeister, 1844

Subtribe Schizognathina Ohaus, 1918

Tribe Geniatini Burmeister, 1844

Tribe Rutelini MacLeay, 1819

Subtribe Areodina Burmeister, 1844

Subtribe Desmonychina Arrow, 1917

Subtribe Didrepanephorina Ohaus, 1918

Subtribe Heterosternina Bates, 1888 nomen protectum

Subtribe Lasiocalina Ohaus, 1918

Subtribe Oryctomorphina Burmeister, 1847

Subtribe Parastasiina Burmeister, 1844

Subtribe Rutelina MacLeay, 1819

**Subfamily Dynastinae MacLeay, 1819**

Tribe Agaocephalini Burmeister, 1847

Tribe Cyclocephalini Laporte, 1840

Tribe Dynastini MacLeay, 1819

Tribe Hexodontini Lacordaire, 1856

Tribe Oryctini Mulsant, 1842

Tribe Oryctoderini Endrödi, 1966

Tribe Pentodontini Mulsant, 1842

Subtribe Cheiroplatina Carne, 1957

Subtribe Dipelicina Carne, 1957

Subtribe Pentodontina Mulsant, 1842

Subtribe Pseudoryctina Carne, 1957

Tribe Phileurini Burmeister, 1847

Subtribe Cryptodina Burmeister and Schaum, 1840

Subtribe Phileurina Burmeister, 1847

**Subfamily Cetoniinae Leach, 1815**

Tribe Cetoniini Leach, 1815

Subtribe Cetoniina Leach, 1815

Subtribe Euphoriina Horn, 1880

Subtribe Leucocelina Kraatz, 1882

Tribe Cremastocheilini Burmeister and Schaum, 1841

Subtribe Aspilina Krikken, 1984

Subtribe Coenochilina Burmeister, 1842

Subtribe Cremastocheilina Burmeister and Schaum, 1841

Subtribe Cymophorina Krikken, 1984

Subtribe Genuchina Krikken, 1984

Subtribe Goliathopsidina Krikken, 1984

Subtribe Heterogeniina Krikken, 1984

Subtribe Lissogeniina Krikken, 1984

Subtribe Macromina Burmeister and Schaum, 1840

Subtribe Nyassinina Krikken, 1984

Subtribe Oplostomina Krikken, 1984

Subtribe Pilinurgina Krikken, 1984

Subtribe Spilophorina Krikken, 1984

Subtribe Telochilina Krikken, 1984

Subtribe Trichoplina Krikken, 1984

Subtribe Trogodina Krikken, 1984

Tribe Diplognathini Burmeister, 1842

Tribe Goliathini Latreille, 1829

Subtribe Coryphocerina Burmeister, 1842

Subtribe Dicronocephalina Krikken, 1984

Subtribe Goliathina Latreille, 1829

Subtribe Ichnestomatina Burmeister, 1842

Tribe Gymnetini Kirby, 1827

Subtribe Blaesiina Schoch, 1895

Subtribe Gymnetina Kirby, 1827

Tribe Phaedimini Schoch, 1894

Tribe Schizorhinini Burmeister, 1842

Subtribe Lomapterina Burmeister, 1842

Subtribe Schizorhinina Burmeister, 1842

Tribe Stenotarsiini Kraatz, 1880

Subtribe Anochiliina Krikken, 1984

Subtribe Coptomiina Schenkling, 1921

Subtribe Chromoptiliina Krikken, 1984

Subtribe Doryscelina Schenkling, 1921

Subtribe Euchroeina Paulian and Descarpentries, 1982

Subtribe Heterophanina Schoch, 1894

Subtribe Heterosomatina Krikken, 1984

Subtribe Pantoliina Krikken, 1984

Subtribe Parachiliina Krikken, 1984

Subtribe Stenotarsiina Kraatz, 1880

Tribe Taenioderini Mikšić, 1976

Subtribe Chalcotheina Mikšić, 1976

Subtribe Taenioderina Mikšić, 1976

Tribe Trichiini Fleming, 1821

Subtribe Cryptodontina Lacordaire, 1856

Subtribe Incina Burmeister, 1842

Subtribe Osmodermatina Schenkling, 1922

Subtribe Platygeniina Krikken, 1984

Subtribe Trichiina Fleming, 1821

Tribe Valgini Mulsant, 1842

Subtribe Microvalgina Kolbe, 1904

Subtribe Valgina Mulsant, 1842

Tribe Xiphoscelidini Burmeister, 1842

**†Family Coprinisphaeridae Genise, 2004**

**†Family Pallichnidae Genise, 2004**

**Series ELATERIFORMIA**

**Superfamily Scirtoidea Fleming, 1821**

**Family Decliniidae Nikitsky, Lawrence, Kirejtshuk and Gratshev, 1994**

**Family Eucinetidae Lacordaire, 1857**

**Family Clambidae Fischer von Waldheim, 1821**

**Subfamily Calyptomerinae Crowson, 1955**

**Subfamily Acalyptomerinae Crowson, 1979**

**Subfamily Clambinae Fischer von Waldheim, 1821**

**Family Scirtidae Fleming, 1821**

**Subfamily Scirtinae Fleming, 1821**

**Subfamily Nipponocyphoninae Lawrence and Yoshitomi, 2007**

**Subfamily Stenocyphoninae Lawrence and Yoshitomi, 2007**

**†Family Elodophthalmidae Kirejtshuk and Azar, 2008**

**†Family Mesocinetida Kirejtshuk and Ponomarenko, 2010**

**Superfamily Dascilloidea Guérin-Méneville, 1843 (1834)**

**Family Dascillidae Guérin-Méneville, 1843 (1834)**

**Subfamily Dascillinae Guérin-Méneville, 1843 (1834)**

Tribe Cinnabariini Pic, 1914

Tribe Dascillini Guérin-Méneville, 1843 (1834)

**Subfamily Karumiinae Escalera, 1913**

Tribe Emmitini Escalera, 1914

Tribe Escalerinini Paulus, 1972

Tribe Genecerini Pic, 1914

Tribe Karumiini Escalera, 1913

**Family Rhipiceridae Latreille, 1834**

**Superfamily Buprestoidea Leach, 1815**

**Family Schizopodidae LeConte, 1859**

**Subfamily Schizopodinae LeConte, 1859**

Tribe Dystaxiini Théry, 1929

†Tribe Electrapatini Iablokoff-Khnzorian, 1962

Tribe Schizopodini LeConte, 1859

**Family Buprestidae Leach, 1815**

**Subfamily Julodinae Lacordaire, 1857**

**Subfamily Polycestinae Lacordaire, 1857**

Tribe Acmaeoderini Kerremans, 1893

Subtribe Acmaeoderina Kerremans, 1893

Subtribe Acmaeoderoidina Cobos, 1955

Subtribe Nothomorphina Cobos, 1955

Tribe Astraeini Cobos, 1980

Tribe Bulini Bellamy, 1995

Tribe Haplostethini LeConte, 1861

Tribe Paratracheini Cobos, 1980

Tribe Perucolini Cobos, 1980

Tribe Polycestini Lacordaire, 1857

Subtribe Polycestina Lacordaire, 1857

Subtribe Xenopseina Volkovitsh, 2008

Tribe Polyctesini Cobos, 1955

Tribe Prospherini Cobos, 1980

Tribe Ptosimini Kerremans, 1903

Tribe Thrincopygini LeConte, 1861

Tribe Tyndaridini Cobos, 1955

Subtribe Mimicoclytrinina Bellamy, 2003

Subtribe Pseudacherusiina Cobos, 1980

Subtribe Tylaucheniina Cobos, 1959

Subtribe Tyndaridina Cobos, 1955

Tribe Xyroscelidini Cobos, 1955

**Subfamily Galbellinae Reitter, 1911**

**Subfamily Chrysochroinae Laporte, 1835**

Tribe Chrysochroini Laporte, 1835

Subtribe Chalcophorina Lacordaire, 1857 (1848)

Subtribe Chrysochroina Laporte, 1835

Subtribe Eucallopistina Bellamy, 2003

Tribe Dicercini Gistel, 1848

Subtribe Dicercina Gistel, 1848

Subtribe Haplotrinchina Holyński, 1993

Subtribe Hippomelanina Holyński, 1993

Subtribe Pseudoperotina Tôyama, 1987

Tribe Evidini Tôyama, 1987

Tribe Paraleptodemini Cobos, 1975

Subtribe Euchromatina Holyński, 1993

Subtribe Euplectaleciina Holyński, 1993

Subtribe Hypoprasina Holyński, 1993

Subtribe Paraleptodemina Cobos, 1975

Subtribe Pristipterina Holyński, 1993

Tribe Paratassini Bílý and Volkovitsh, 1996

Tribe Poecilonotini Jakobson, 1913

Subtribe Poecilonotina Jakobson, 1913

Subtribe Nesotrinchina Bílý, Kubáň and Volkovitsh, 2009

Tribe Sphenopterini Lacordaire, 1857

Tribe Vadonaxiini Descarpentries, 1970

**Subfamily Buprestinae Leach, 1815**

Tribe Actenodini Gistel, 1848

Tribe Anthaxiini Gory and Laporte, 1839

Tribe Bubastini Obenberger, 1920

Tribe Buprestini Leach, 1815

Subtribe Agaeocerina Nelson, 1982

Subtribe Buprestina Leach, 1815

Subtribe Lamprocheilina Holyński, 1993

Subtribe Trachykelina Holyński, 1988

Tribe Chrysobothrini Gory and Laporte, 1836

Tribe Coomaniellini Bílý, 1974

Tribe Curidini Holyński, 1988

Subtribe Anilarina Bílý, 2000

Subtribe Curidina Holyński, 1988

Subtribe Neocuridina Holyński, 1988

Tribe Epistomentini Levey, 1978

Tribe Exagistini Tôyama, 1987

Tribe Julodimorphini Kerremans, 1903

Tribe Kisanthobiini Richter, 1949

Tribe Maoraxiini Holyński, 1984

Tribe Melanophilini Bedel, 1921

Tribe Melobaseini Bílý, 2000

Tribe Mendizabaliini Cobos, 1968

Tribe Nascionini Holyński, 1988

Tribe Phrixiini Cobos, 1975

Tribe Pterobothrini Volkovitsh, 2001

Tribe Stigmoderini Lacordaire, 1857

Tribe Thomassetiini Bellamy, 1987

Subtribe Philanthaxiina Holyński, 1988

Subtribe Thomassetiina Bellamy, 1987

Tribe Trigonogeniini Cobos, 1956

Tribe Xenorhipidini Cobos, 1986

Subtribe Trichinorhipidina Bellamy, 2006

Subtribe Xenorhipidina Cobos, 1986

**Subfamily Agrilinae Laporte, 1835**

Tribe Agrilini Laporte, 1835

Subtribe Agrilina Laporte, 1835

Subtribe Amorphosternina Cobos, 1974

Subtribe Amyiina Holyński, 1993

Subtribe Rhaeboscelidina Cobos, 1976

Tribe Aphanisticini Jacquelin du Val, 1859

Subtribe Anthaxomorphina Holyński, 1993

Subtribe Aphanisticina Jacquelin du Val, 1859

Subtribe Cylindromorphina Portevin, 1931

Subtribe Cylindromorphoidina Cobos, 1979

Subtribe Germaricina Cobos, 1979

Tribe Coraebini Bedel, 1921

Subtribe Amorphosomatina Majer, 2000

Subtribe Cisseina Majer, 2000

Subtribe Clematina Majer, 2000

Subtribe Coraebina Bedel, 1921

Subtribe Dismorphina Cobos, 1990

Subtribe Ethoniina Majer, 2000

Subtribe Geraliina Cobos, 1988

Subtribe Meliboeina Majer, 2000

Subtribe Synechocerina Majer, 2000

Subtribe Toxoscelina Majer, 2000

Tribe Tracheini Laporte, 1835

Subtribe Brachina LeConte, 1861

Subtribe Leiopleurina Holyński, 1993

Subtribe Pachyschelina Böving and Craighead, 1931

Subtribe Tracheina Laporte, 1835

**†Subfamily Parathyreinae Alexeev, 1994**

**Superfamily Byrrhoidea Latreille, 1804**

**Family Byrrhidae Latreille, 1804**

**Subfamily Byrrhinae Latreille, 1804**

Tribe Byrrhini Latreille, 1804

Tribe Exomellini Casey, 1914

Tribe Morychini El Moursy, 1961

Tribe Pedilophorini Casey, 1912

Tribe Simplocariini Mulsant and Rey, 1869

**Subfamily Syncalyptinae Mulsant and Rey, 1869**

Tribe Microchaetini Paulus, 1973

Tribe Syncalyptini Mulsant and Rey, 1869

**Subfamily Amphicyrtinae LeConte, 1861**

**Family Elmidae Curtis, 1830**

**Subfamily Larainae LeConte, 1861**

Tribe Laraini LeConte, 1861

Tribe Potamophilini Mulsant and Rey, 1872

**Subfamily Elminae Curtis, 1830**

Tribe Ancyronychini Ganglbauer, 1904

Tribe Elmini Curtis, 1830

Subtribe Elmina Curtis, 1830

Subtribe Stenelmina Mulsant and Rey, 1872

Tribe Macronychini Gistel, 1848

**Family Dryopidae Billberg, 1820 (1817)**

**Family Lutrochidae Kasap and Crowson, 1975**

**Family Limnichidae Erichson, 1846**

**Subfamily Hyphalinae Britton, 1971**

**Subfamily Limnichinae Erichson, 1846**

Tribe Limnichini Erichson, 1846

Tribe Wooldridgeini Spangler, 1999

**Subfamily Cephalobyrrhinae Champion, 1925**

**Subfamily Thaumastodinae Champion, 1924**

**Family Heteroceridae MacLeay, 1825**

**Subfamily Elythomerinae Pacheco, 1964**

**Subfamily Heterocerinae MacLeay, 1825**

Tribe Augylini Pacheco, 1964

Tribe Heterocerini MacLeay, 1825

Tribe Micilini Pacheco, 1964

Tribe Tropicini Pacheco, 1964

**Family Psephenidae Lacordaire, 1854**

**Subfamily Afroeubriinae Lee, Satô, Shepard and Jäch, 2007**

**Subfamily Eubriinae Lacordaire, 1857**

**Subfamily Eubrianacinae Jakobson, 1913**

**Subfamily Psephenoidinae Bollow, 1938**

**Subfamily Psepheninae Lacordaire, 1854**

**Family Cneoglossidae Champion, 1897**

**Family Ptilodactylidae Laporte, 1836**

**Subfamily Anchytarsinae Champion, 1897**

**Subfamily Cladotominae Pic, 1914**

**Subfamily Aploglossinae Champion, 1897**

**Subfamily Araeopidiinae Lawrence, 1991**

**Subfamily Ptilodactylinae Laporte, 1836**

**Family Podabrocephalidae Pic, 1930**

**Family Chelonariidae Blanchard, 1845**

**Family Eulichadidae Crowson, 1973**

**Family Callirhipidae Emden, 1924**

**Superfamily Elateroidea Leach, 1815**

**Family Rhinorhipidae Lawrence, 1988**

**Family Artematopodidae Lacordaire, 1857**

**Subfamily Electribiinae Crowson, 1975**

**Subfamily Allopogoniinae Crowson, 1973**

**Subfamily Artematopodinae Lacordaire, 1857**

Tribe Artematopodini Lacordaire, 1857

Tribe Ctesibiini Crowson, 1973

Tribe Macropogonini LeConte, 1861

**Family Brachypsectridae LeConte and Horn, 1883**

**Family Cerophytidae Latreille, 1834**

**Family Eucnemidae Eschscholtz, 1829**

**Subfamily Perothopinae Lacordaire, 1857**

**Subfamily Phyllocerinae Reitter, 1905**

Tribe Anelastini Reitter, 1911

Tribe Phyllocerini Reitter, 1905

**Subfamily Pseudomeninae Muona, 1993**

Tribe Pseudomenini Muona, 1993

Tribe Schizophilini Muona, 1993

**Subfamily Palaeoxeninae Muona, 1993**

**Subfamily Phlegoninae Muona, 1993**

**Subfamily Anischiinae Fleutiaux, 1936**

**Subfamily Melasinae Fleming, 1821**

Tribe Calyptocerini Muona, 1993

Tribe Ceballosmelasini Muona, 1993

Tribe Dirhagini Reitter, 1911

Tribe Epiphanini Muona, 1993

Tribe Hylocharini Jacquelin du Val, 1859

Tribe Melasini Fleming, 1821

Subtribe Compsocnemina Muona, 1993

Subtribe Melasina Fleming, 1821

Tribe Neocharini Muona, 1993

Tribe Xylobiini Reitter, 1911

**Subfamily Eucneminae Eschscholtz, 1829**

Tribe Dendrocharini Fleutiaux, 1920

Tribe Dyscharachthini Muona, 1993

Tribe Entomosatopini Muona, 1993

Tribe Eucnemini Eschscholtz, 1829

Tribe Galbitini Muona, 1991

Tribe Mesogenini Muona, 1993

Tribe Muonajini Özdikmen, 2008

Tribe Perrotiini Muona, 1993

Tribe Phaenocerini Muona, 1993

Tribe Proutianini Muona, 1993

**Subfamily Macraulacinae Fleutiaux, 1923**

Tribe Anelastidini Muona, 1993

Tribe Echthrogasterini Cobos, 1965

Tribe Euryptychini Mamaev, 1976

Tribe Jenibuntorini Muona, 1993

Tribe Macraulacini Fleutiaux, 1923

Tribe Nematodini Leiler, 1976

Tribe Oisocerini Muona, 1993

Tribe Orodotini Muona, 1993

†Tribe Throscogeniini Iablokoff-Khnzorian, 1962

**Family Throscidae Laporte, 1840** nomen protectum

**†Family Praelateriidae Dolin, 1973**

**Family Elateridae Leach, 1815**

**Subfamily Cebrioninae Latreille, 1802**

**Subfamily Agrypninae Candèze, 1857** nomen protectum

Tribe Agrypnini Candèze, 1857 nomen protectum

Tribe Anaissini Golbach, 1984

†Tribe Cryptocardiini Dolin, 1980

Tribe Euplinthini Costa, 1975

Subtribe Cleidecostina Johnson, 2002

Subtribe Compsoplinthina Costa, 1975

Subtribe Euplinthina Costa, 1975

Tribe Hemirhipini Candèze, 1857

Tribe Oophorini Gistel, 1848

Tribe Platycrepidiini Costa and Casari-Chen, 1993

Tribe Pseudomelanactini Arnett, 1967

Tribe Pyrophorini Candèze, 1863

Subtribe Hapsodrilina Costa, 1975

Subtribe Nyctophyxina Costa, 1975

Subtribe Pyrophorina Candèze, 1863

Tribe Tetralobini Laporte, 1840

**Subfamily Thylacosterninae Fleutiaux, 1920**

**Subfamily Lissominae Laporte, 1835**

**Subfamily Semiotinae Jakobson, 1913**

**Subfamily Campyloxeninae Costa, 1975**

**Subfamily Pityobiinae Hyslop, 1917**

**Subfamily Oxynopterinae Candèze, 1857**

**Subfamily Dendrometrinae Gistel, 1848**

Tribe Crepidomenini Candèze, 1863

Tribe Dendrometrini Gistel, 1848

Subtribe Dendrometrina Gistel, 1848

Subtribe Denticollina Stein and Weise, 1877 (1848)

Subtribe Hemicrepidiina Champion, 1896

Tribe Dimini Candèze, 1863

Tribe Hypnoidini Schwarz, 1906 (1860)

Tribe Pleonomini Semenov and Pjatakova, 1936

Tribe Prosternini Gistel, 1856 nomen protectum

Tribe Senodoniini Schenkling, 1927

**Subfamily Negastriinae Nakane and Kishii, 1956**

Tribe Negastriini Nakane and Kishii, 1956

Tribe Quasimusini Schimmel and Tarnawski, 2009

Subtribe Loebliquasimusina Schimmel and Tarnawski, 2009

Subtribe Quasimusina Schimmel and Tarnawski, 2009

Subtribe Striatoquasimusina Schimmel and Tarnawski, 2009

Subtribe Wittmeroquasimusina Schimmel and Tarnawski, 2009

**Subfamily Elaterinae Leach, 1815**

Tribe Agriotini Laporte, 1840

Subtribe Agriotina Laporte, 1840

Subtribe Cardiorhinina Candèze, 1863

Tribe Ampedini Gistel, 1848

Tribe Dicrepidiini Thomson, 1858

Tribe Elaterini Leach, 1815

Tribe Megapenthini Gurjeva, 1973

Tribe Melanotini Candèze, 1859 (1848)

Tribe Odontonychini Girard, 1973

Tribe Physorhinini Candèze, 1859

Tribe Pomachiliini Candèze, 1859

Tribe Synaptini Gistel, 1856

**Subfamily Cardiophorinae Candèze, 1859**

**Subfamily Hemiopinae Fleutiaux, 1941**

**Subfamily Physodactylinae Lacordaire, 1857**

**Subfamily Eudicronychinae Girard, 1971**

**Subfamily Subprotelaterinae Fleutiaux, 1920**

**Subfamily Morostomatinae Dolin, 2000**

**†Subfamily Protagrypninae Dolin, 1973**

†Tribe Desmatini Dolin, 1975

†Tribe Hypnomorphini Dolin, 1975

†Tribe Protagrypnini Dolin, 1973

**Family Plastoceridae Crowson, 1972**

**Family Drilidae Blanchard, 1845**

**Subfamily Drilinae Blanchard, 1845**

**Subfamily Thilmaninae Kazantsev, 2004**

Tribe Euanomini Kazantsev, 2010

Tribe Thilmanini Kazantsev, 2004

**Family Omalisidae Lacordaire, 1857**

**†Family Berendtimiridae Winkler, 1987**

**Family Lycidae Laporte, 1836**

**Subfamily Libnetinae Bocák and Bocáková, 1990**

**Subfamily Dictyopterinae Houlbert, 1922**

Tribe Dictyopterini Houlbert, 1922

Tribe Lycoprogenthini Bocák and Bocáková, 2008

Tribe Taphini Bocák and Bocáková, 1990

**Subfamily Lyropaeinae Bocák and Bocáková, 1989**

Tribe Alyculini Bocák and Bocáková, 2008

Tribe Antennolycini Bocák and Bocáková, 2008

Tribe Lyropaeini Bocák and Bocáková, 1989

Tribe Miniduliticolini Kazantsev, 2003

Tribe Platerodrilini Kazantsev, 2004

**Subfamily Ateliinae Kleine, 1929**

Tribe Ateliini Kleine, 1929

Tribe Dilophotini Kleine, 1929

**Subfamily Lycinae Laporte, 1836**

Tribe Calochromini Lacordaire, 1857

Tribe Calopterini Green, 1949

Subtribe Acroleptina Bocáková, 2005

Subtribe Calopterina Green, 1949

Tribe Conderini Bocák and Bocáková, 1990

Tribe Dihammatini Bocák and Bocáková, 2008

Tribe Erotini LeConte, 1881

Tribe Eurrhacini Bocáková, 2005

Tribe Leptolycini Leng and Mutchler, 1922

Tribe Lycini Laporte, 1836

Tribe Lyponiini Bocák and Bocáková, 1990

Tribe Macrolycini Kleine, 1929

Tribe Melanerotini Kazantsev, 2010

Tribe Metriorrhynchini Kleine, 1926

Subtribe Hemiconderinina Bocák and Bocáková, 1990

Subtribe Metriorrhynchina Kleine, 1926

Subtribe Trichalina Kleine, 1929

Tribe Platerodini Kleine, 1929

Tribe Slipinskiini Bocák and Bocáková, 1992

Tribe Thonalmini Kleine, 1933

**Subfamily Dexorinae Bocák and Bocáková, 1989**

**Family Telegeusidae Leng, 1920**

**Family Phengodidae LeConte, 1861**

**Subfamily Phengodinae LeConte, 1861**

**Subfamily Mastinocerinae LeConte, 1881**

**Subfamily Penicillophorinae Paulus, 1975**

**Family Rhagophthalmidae Olivier, 1907**

**Family Lampyridae Rafinesque, 1815**

**Subfamily Psilocladinae McDermott, 1964**

**Subfamily Amydetinae Olivier, 1907**

Tribe Amydetini Olivier, 1907

Tribe Vestini McDermott, 1964

**Subfamily Lampyrinae Rafinesque, 1815**

Tribe Cratomorphini Green, 1948

Tribe Lamprocerini Olivier, 1907

Tribe Lamprohizini Kazantsev, 2010

Tribe Lampyrini Rafinesque, 1815

Tribe Lucidotini Lacordaire, 1857

Subtribe Dadophorina Olivier, 1907

Subtribe Lamprigerina McDermott, 1964

Subtribe Lucidotina Lacordaire, 1857

Subtribe Photinina LeConte, 1881

Tribe Pleotomini Summers, 1874

**Subfamily Luciolinae Lacordaire, 1857**

Tribe Curtosini McDermott, 1964

Tribe Luciolini Lacordaire, 1857

**Subfamily Photurinae Lacordaire, 1857**

**Family Omethidae LeConte, 1861**

**Subfamily Omethinae LeConte, 1861**

**Subfamily Matheteinae LeConte, 1881**

**Subfamily Driloniinae Crowson, 1972**

**Family Cantharidae Imhoff, 1856 (1815)**

**Subfamily Cantharinae Imhoff, 1856 (1815)**

Tribe Cantharini Imhoff, 1856 (1815)

Tribe Podabrini Gistel, 1856

**Subfamily Silinae Mulsant, 1862**

Tribe Silini Mulsant, 1862

Tribe Tytthonyxini Arnett, 1962

**Subfamily Dysmorphocerinae Brancucci, 1980**

**Subfamily Malthininae Kiesenwetter, 1852**

Tribe Malchinini Brancucci, 1980

Tribe Malthinini Kiesenwetter, 1852

Tribe Malthodini Böving and Craighead, 1931

**Subfamily Chauliognathinae LeConte, 1861**

Tribe Chauliognathini LeConte, 1861

Tribe Ichthyurini Champion, 1915

**Subfamily Cydistinae Paulus, 1972**

**Subfamily Pterotinae LeConte, 1861**

**Subfamily Ototretinae McDermott, 1964**

**Subfamily Ototretadrilinae Crowson, 1972**

**†Subfamily Lasiosynidae Kirejtshuk, Chang, Ren and Kun, 2010**

**Series DERODONTIFORMIA**

**Superfamily Derodontoidea LeConte, 1861**

**Family Derodontidae LeConte, 1861**

**Subfamily Peltasticinae LeConte, 1861**

**Subfamily Derodontinae LeConte, 1861**

**Subfamily Laricobiinae Mulsant and Rey, 1864**

**Family Nosodendridae Erichson, 1846**

**Family Jacobsoniidae Heller, 1926**

**Series BOSTRICHIFORMIA**

**Superfamily Bostrichoidea Latreille, 1802**

**Family Dermestidae Latreille, 1804**

**Subfamily Dermestinae Latreille, 1804**

Tribe Dermestini Latreille, 1804

Tribe Marioutini Jakobson, 1913

**Subfamily Thorictinae Agassiz, 1846**

Tribe Thaumaphrastini Anderson, 1949

Tribe Thorictini Agassiz, 1846

**Subfamily Orphilinae LeConte, 1861**

**Subfamily Trinodinae Casey, 1900**

†Tribe Cretonodini Kirejtshuk and Azar, 2009

Tribe Thylodriini Semenov, 1909

Tribe Trinodini Casey, 1900

Tribe Trinoparvini Háva, 2010

**Subfamily Attageninae Laporte, 1840**

Tribe Attagenini Laporte, 1840

Tribe Egidyellini Semenov, 1914

**Subfamily Megatominae Leach, 1815**

Tribe Anthrenini Gistel, 1848

Tribe Megatomini Leach, 1815

**Family Endecatomidae LeConte, 1861**

**Family Bostrichidae Latreille, 1802**

**Subfamily Dysidinae Lesne, 1921**

**Subfamily Polycaoninae Lesne, 1896**

**Subfamily Bostrichinae Latreille, 1802**

Tribe Apatini Billberg, 1820

Tribe Bostrichini Latreille, 1802

Tribe Dinapatini Lesne, 1910

Tribe Sinoxylini Marseul, 1857

Tribe Xyloperthini Lesne, 1921

**Subfamily Psoinae Blanchard, 1851**

**Subfamily Dinoderinae Thomson, 1863**

**Subfamily Lyctinae Billberg, 1820**

Tribe Lyctini Billberg, 1820

Tribe Trogoxylini Lesne, 1921

**Subfamily Euderiinae Lesne, 1934**

**Family Ptinidae Latreille, 1802**

**Subfamily Eucradinae LeConte, 1861**

Tribe Eucradini LeConte, 1861

Tribe Hedobiini Mulsant and Rey, 1868

**Subfamily Ptininae Latreille, 1802**

Tribe Gibbiini Jacquelin du Val, 1860

Tribe Meziini Bellés, 1985

Tribe Ptinini Latreille, 1802

Tribe Sphaericini Portevin, 1931

**Subfamily Dryophilinae Gistel, 1848**

Tribe Dryophilini Gistel, 1848

Tribe Ptilineurini Böving, 1927

**Subfamily Ernobiinae Pic, 1912**

**Subfamily Anobiinae Fleming, 1821**

**Subfamily Ptilininae Shuckard, 1839**

**Subfamily Alvarenganiellinae Viana and Martínez, 1971**

**Subfamily Xyletininae Gistel, 1848**

Tribe Lasiodermini Böving, 1927

Tribe Metholcini Zahradník, 2009

Tribe Xyletinini Gistel, 1848

**Subfamily Dorcatominae Thomson, 1859**

**Subfamily Mesocoelopodinae Mulsant and Rey, 1864**

Tribe Tricorynini White, 1971

Tribe Mesocoelopodini Mulsant and Rey, 1864

**Series CUCUJIFORMIA**

**Superfamily Lymexyloidea Fleming, 1821**

**Family Lymexylidae Fleming, 1821**

**Subfamily Hylecoetinae Germar, 1818**

**Subfamily Lymexylinae Fleming, 1821**

**Subfamily Atractocerinae Laporte, 1840**

**Subfamily Melittommatinae Wheeler, 1986**

**Superfamily Cleroidea Latreille, 1802**

**Family Phloiophilidae Kiesenwetter, 1863**

**Family Trogossitidae Latreille, 1802**

**Subfamily Peltinae Latreille, 1806**

Tribe Ancyronini Kolibáč, 2006

Tribe Colydiopeltini Kolibáč, 2006

Tribe Decamerini Crowson, 1964

Tribe Lophocaterini Crowson, 1964 nomen protectum

Tribe Peltini Latreille, 1806

Tribe Thymalini Léveillé, 1888

**Subfamily Trogossitinae Latreille, 1802**

Tribe Calityini Reitter, 1922

Tribe Egoliini Lacordaire, 1854

Tribe Gymnochilini Lacordaire, 1854

Tribe Larinotini Ślipiński, 1992

†Tribe Lithostomatini Kolibáč and Huang, 2008

Tribe Trogossitini Latreille, 1802

**Family Chaetosomatidae Crowson, 1952**

**Family Metaxinidae Kolibáč, 2004**

**Family Thanerocleridae Chapin, 1924**

**Subfamily Zenodosinae Kolibáč, 1992**

**Subfamily Thaneroclerinae Chapin, 1924**

Tribe Isoclerini Kolibáč, 1992

Tribe Thaneroclerini Chapin, 1924

Tribe Viticlerini Winkler, 1982

**Family Cleridae Latreille, 1802**

**Subfamily Tillinae Fischer von Waldheim, 1813**

**Subfamily Hydnocerinae Spinola, 1844**

Tribe Callimerini Kolibáč, 1998

Tribe Hydnocerini Spinola, 1844

Tribe Lemidiini Kolibáč, 1998

**Subfamily Clerinae Latreille, 1802**

**Subfamily Korynetinae Laporte, 1836**

**Family Acanthocnemidae Crowson, 1964**

**Family Phycosecidae Crowson, 1952**

**Family Prionoceridae Lacordaire, 1857**

Tribe Lobonychini Majer, 1987

Tribe Prionocerini Lacordaire, 1857

**Family Mauroniscidae Majer, 1995**

**Family Melyridae Leach, 1815**

**Subfamily Rhadalinae LeConte, 1861**

**Subfamily Melyrinae Leach, 1815**

Tribe Arthrobrachini Majer, 1987

Tribe Astylini Pic, 1929

Tribe Cerallini Pic, 1929

Tribe Melyrini Leach, 1815

**Subfamily Dasytinae Laporte, 1840**

Tribe Chaetomalachiini Majer, 1987

Tribe Danaceini Thomson, 1859

Tribe Dasytini Laporte, 1840

Tribe Gietellini Constantin and Menier, 1987

Tribe Listrini Majer, 1990

**Subfamily Malachiinae Fleming, 1821**

Tribe Amalthocini Majer, 2002

Tribe Attalomimini Majer, 1995

Tribe Carphurini Champion, 1923

Tribe Lemphini Wittmer, 1976

Tribe Malachiini Fleming, 1821

Tribe Pagurodactylini Constantin, 2001

**Superfamily Cucujoidea Latreille, 1802**

**†Family Parandrexidae Kirejtshuk, 1994**

**†Family Sinisilvanidae Hong, 2002**

**Family Boganiidae Sen Gupta and Crowson, 1966**

**Subfamily Paracucujinae Endrödy-Younga and Crowson, 1986**

**Subfamily Boganiinae Sen Gupta and Crowson, 1966**

**Family Byturidae Gistel, 1848**

**Subfamily Platydascillinae Pic, 1914**

**Subfamily Byturinae Gistel, 1848**

**Family Helotidae Chapuis, 1876**

**Family Protocucujidae Crowson, 1954**

**Family Sphindidae Jacquelin du Val, 1860**

**Subfamily Protosphindinae Sen Gupta and Crowson, 1979**

**Subfamily Odontosphindinae Sen Gupta and Crowson, 1979**

**Subfamily Sphindiphorinae Sen Gupta and Crowson, 1979**

**Subfamily Sphindinae Jacquelin du Val, 1860**

**Family Biphyllidae LeConte, 1861**

**Family Erotylidae Latreille, 1802**

**Subfamily Xenoscelinae Ganglbauer, 1899**

**Subfamily Pharaxonothinae Crowson, 1952**

**Subfamily Loberinae Bruce, 1951**

**Subfamily Languriinae Hope, 1840**

Tribe Hapalipini Leschen, 2003

Tribe Languriini Hope, 1840

Tribe Thallisellini Sen Gupta, 1968

**Subfamily Cryptophilinae Casey, 1900**

Tribe Cryptophilini Casey, 1900

Tribe Empocryptini Leschen, 2003

Tribe Toramini Sen Gupta, 1967

**Subfamily Erotylinae Latreille, 1802**

Tribe Dacnini Gistel, 1848

Tribe Encaustini Crotch, 1876

Tribe Erotylini Latreille, 1802

Tribe Megalodacnini Sen Gupta, 1970

Tribe Tritomini Curtis, 1834

**Family Monotomidae Laporte, 1840**

**Subfamily Rhizophaginae Redtenbacher, 1845**

**Subfamily Monotominae Laporte, 1840**

Tribe Europini Sen Gupta, 1988

Tribe Lenacini Crowson, 1952

Tribe Monotomini Laporte, 1840

†Tribe Rhizophtomini Kirejtshuk and Azar, 2009

Tribe Thionini Crowson, 1952

**Family Hobartiidae Sen Gupta and Crowson, 1966**

**Family Cryptophagidae Kirby, 1826**

**Subfamily Cryptophaginae Kirby, 1826**

Tribe Caenoscelini Casey, 1900

Tribe Cryptophagini Kirby, 1826

Tribe Picrotini Crowson, 1980

**Subfamily Atomariinae LeConte, 1861**

Tribe Atomariini LeConte, 1861

Tribe Cryptafricini Leschen, 1996

Tribe Hypocoprini Reitter, 1879

**Family Agapythidae Sen Gupta and Crowson, 1969**

**Family Priasilphidae Crowson, 1973**

**Family Phloeostichidae Reitter, 1911**

**Family Silvanidae Kirby, 1837**

**Subfamily Brontinae Blanchard, 1845**

Tribe Brontini Blanchard, 1845

Tribe Telephanini LeConte, 1861

**Subfamily Silvaninae Kirby, 1837**

**Family Cucujidae Latreille, 1802**

**Family Myraboliidae Lawrence and Britton, 1991**

**Family Cavognathidae Sen Gupta and Crowson, 1966**

**Family Lamingtoniidae Sen Gupta and Crowson, 1969**

**Family Passandridae Blanchard, 1845**

**Family Phalacridae Leach, 1815**

**Subfamily Phaenocephalinae Matthews, 1899**

**Subfamily Phalacrinae Leach, 1815**

**Family Propalticidae Crowson, 1952**

**Family Laemophloeidae Ganglbauer, 1899**

**Family Tasmosalpingidae Lawrence and Britton, 1991**

**Family Cyclaxyridae Gimmel, Leschen and Ślipiński, 2009**

**Family Kateretida Kirby, 1837**

**Family Nitidulidae Latreille, 1802**

**Subfamily Calonecrinae Kirejtshuk, 1982**

**Subfamily Maynipeplinae Kirejtshuk, 1998**

**Subfamily Epuraeinae Kirejtshuk, 1986**

Tribe Epuraeini Kirejtshuk, 1986

Tribe Taenioncini Kirejtshuk, 1998

**Subfamily Carpophilinae Erichson, 1842**

**Subfamily Amphicrossinae Kirejtshuk, 1986**

**Subfamily Meligethinae Thomson, 1859**

**Subfamily Nitidulinae Latreille, 1802**

Tribe Cychramini Gistel, 1848

Tribe Cychramptodini Kirejtshuk and Lawrence, 1992

Tribe Cyllodini Everts, 1898

Tribe Lawrencerosini Kirejtshuk, 1991

Tribe Mystropini Murray, 1864

Tribe Nitidulini Latreille, 1802

**Subfamily Cillaeinae Kirejtshuk and Audisio, 1986**

**Subfamily Cryptarchinae Thomson, 1859**

Tribe Arhinini Kirejtshuk, 1987

Tribe Cryptarchini Thomson, 1859

Tribe Eucalosphaerini Kirejtshuk, 1987

Tribe Platyarchini Kirejtshuk, 1998

**Subfamily Cybocephalinae Jacquelin du Val, 1858**

**Family Smicripidae Horn, 1880**

**Family Bothrideridae Erichson, 1845**

**Subfamily Teredinae Seidlitz, 1888**

Tribe Sosylopsini Dajoz, 1980

Tribe Sysolini Ślipiński and Pal, 1985

Tribe Teredini Seidlitz, 1888

**Subfamily Xylariophilinae Pal and Lawrence, 1986**

**Subfamily Anommatinae Ganglbauer, 1899**

**Subfamily Bothriderinae Erichson, 1845**

**Family Cerylonidae Billberg, 1820**

**Subfamily Euxestinae Grouvelle, 1908**

**Subfamily Loeblioryloninae Ślipiński, 1990**

**Subfamily Ostomopsinae Sen Gupta and Crowson, 1973**

**Subfamily Murmidiinae Jacquelin du Val, 1858**

**Subfamily Ceryloninae Billberg, 1820**

**Family Alexiidae Imhoff, 1856**

**Family Discolomatidae Horn, 1878**

**Subfamily Notiophyginae Jakobson, 1915**

Tribe Dystheamonini John, 1954

Tribe Notiophygini Jakobson, 1915

Tribe Pachyplacini John, 1954

**Subfamily Discolomatinae Horn, 1878**

**Subfamily Aphanocephalinae Jakobson, 1904**

**Subfamily Cephalophaninae John, 1954**

**Subfamily Pondonatinae John, 1954**

**Family Endomychidae Leach, 1815**

**Subfamily Merophysiinae Seidlitz, 1872**

**Subfamily Pleganophorinae Jacquelin du Val, 1858**

**Subfamily Anamorphinae Strohecker, 1953**

**Subfamily Leiestinae Thomson, 1863**

**Subfamily Mycetaeinae Jacquelin du Val, 1857**

**Subfamily Eupsilobiinae Casey, 1895**

**Subfamily Xenomycetinae Strohecker, 1962**

**Subfamily Danascelinae Tomaszewska, 2000**

**Subfamily Endomychinae Leach, 1815**

**Subfamily Epipocinae Gorham, 1873**

**Subfamily Stenotarsinae Chapuis, 1876**

**Subfamily Lycoperdininae Bromhead, 1838**

**Family Coccinellidae Latreille, 1807**

**Subfamily Microweiseinae Leng, 1920**

Tribe Microweiseini Leng, 1920

Tribe Serangiini Pope, 1962

Tribe Sukunahikonini Kamiya, 1960

**Subfamily Coccinellinae Latreille, 1807**

Tribe Argentipilosini Gordon and de Almeida, 1991

Tribe Aspidimerini Mulsant, 1850

Tribe Azyini Mulsant, 1850

Tribe Brachiacanthini Mulsant, 1850

Tribe Carinodulini Gordon, Pakaluk and Ślipiński, 1989

Tribe Cephaloscymnini Gordon, 1985

Tribe Chilocorini Mulsant, 1846

Tribe Chnoodini Mulsant, 1850

Tribe Coccidulini Mulsant, 1846

Tribe Coccinellini Latreille, 1807

Tribe Cranophorini Mulsant, 1850

Tribe Cryptognathini Mulsant, 1850

Tribe Cynegetini Thomson, 1866

Tribe Diomini Gordon, 1999

Tribe Epilachnini Mulsant, 1846

Tribe Epivertini Pang and Mao, 1979

Tribe Eremochilini Gordon and Vanderberg, 1987

Tribe Hyperaspidini Mulsant, 1846

Tribe Limnichopharini Miyatake, 1994

Tribe Monocorynini Miyatake, 1988

Tribe Noviini Mulsant, 1846

Tribe Ortaliini Mulsant, 1850

Tribe Oryssomini Gordon, 1974

Tribe Platynaspini Mulsant, 1846

Tribe Plotinini Miyatake, 1994

Tribe Poriini Mulsant, 1850

Tribe Scymnillini Casey, 1899

Tribe Scymnini Mulsant, 1846

Tribe Selvadiini Gordon, 1985

Tribe Shirozuellini Sasaji, 1967

Tribe Stethorini Dobzhansky, 1924

Tribe Sticholotidini Weise, 1901

Tribe Telsimiini Casey, 1899

Tribe Tetrabrachini Kapur, 1948

**Family Corylophidae LeConte, 1852**

**Subfamily Periptyctinae Ślipiński, Lawrence and Tomaszewska, 2001**

**Subfamily Corylophinae LeConte, 1852**

Tribe Aenigmaticini Casey, 1900

Tribe Cleidostethini Bowestead, Booth, Ślipiński and Lawrence, 2001

Tribe Corylophini LeConte, 1852

Tribe Foadiini Ślipiński, Tomaszewska and Lawrence, 2009

Tribe Orthoperini Jacquelin du Val, 1857

Tribe Parmulini Poey, 1854

Tribe Peltinodini Paulian, 1950

Tribe Rypobiini Paulian, 1950

Tribe Sericoderini Matthews, 1888

Tribe Teplinini Pakaluk, Ślipiński and Lawrence, 1994

**Family Akalyptoischiidae Lord, Hartley, Lawrence, McHugh and Miller, 2010**

**Family Latridiidae Erichson, 1842**

**Subfamily Latridiinae Erichson, 1842**

**Subfamily Corticariinae Curtis, 1829**

**†Subfamily Tetrameropseinae Kirejtshuk and Azar, 2008**

**Superfamily Tenebrionoidea Latreille, 1802**

**Family Mycetophagidae Leach, 1815**

**Subfamily Esarcinae Reitter, 1882**

**Subfamily Mycetophaginae Leach, 1815**

Tribe Mycetophagini Leach, 1815

Tribe Typhaeini Thomson, 1863

**Subfamily Bergininae Leng, 1920**

**Family Archeocrypticidae Kaszab, 1964**

**Family Pterogeniidae Crowson, 1953**

**Family Ciidae Leach, 1819**

**Subfamily Sphindociinae Lawrence, 1974**

**Subfamily Ciinae Leach, 1819**

Tribe Ciini Leach, 1819

Tribe Orophiini Thomson, 1863

Tribe Xylographellini Kawanabe and Miyatake, 1996

Subtribe Syncosmetina Lopes-Andrade, 2008

Subtribe Xylographellina Kawanabe and Miyatake, 1996

**Family Tetratomidae Billberg, 1820**

**Subfamily Tetratominae Billberg, 1820**

**Subfamily Piseninae Miyatake, 1960**

**Subfamily Penthinae Lacordaire, 1859**

**Subfamily Hallomeninae Gistel, 1848**

**Subfamily Eustrophinae Gistel, 1848**

Tribe Eustrophini Gistel, 1848

Tribe Holostrophini Nikitsky, 1998

**Family Melandryidae Leach, 1815**

**Subfamily Melandryinae Leach, 1815**

Tribe Anisoxiellini Nikitsky, 2007

Tribe Dircaeini Kirby, 1837

Tribe Hypulini Gistel, 1848

Tribe Melandryini Leach, 1815

Tribe Orchesiini Mulsant, 1856

Tribe Serropalpini Latreille, 1829

Tribe Xylitini Thomson, 1864

Tribe Zilorini Desbrochers des Loges, 1900

**Subfamily Osphyinae Mulsant, 1856 (1839)**

**Family Mordellidae Latreille, 1802**

**Subfamily Ctenidiinae Franciscolo, 1951**

**Subfamily Mordellinae Latreille, 1802**

Tribe Conaliini Ermisch, 1956

Tribe Mordellini Latreille, 1802

Tribe Mordellistenini Ermisch, 1941

Tribe Reynoldsiellini Franciscolo, 1957

Tribe Stenaliini Franciscolo, 1955

**Family Ripiphoridae Gemminger, 1870 (1855)**

**Subfamily Ptilophorinae Gerstaecker, 1855**

**Subfamily Pelecotominae Seidlitz, 1875**

**Subfamily Hemirhipidiinae Heller, 1921**

**Subfamily Ripidiinae Gerstaecker, 1855**

Tribe Eorhipidiini Iablokoff-Khnzorian, 1986

Tribe Ripidiini Gerstaecker, 1855

**Subfamily Ripiphorinae Gemminger, 1870 (1855)**

Tribe Macrosiagonini Heyden, 1908

Tribe Ripiphorini Gemminger, 1870 (1855)

**Family Zopheridae Solier, 1834**

**Subfamily Colydiinae Billberg, 1820**

Tribe Acropini Sharp, 1894

Tribe Adimerini Sharp, 1894

Tribe Colydiini Billberg, 1820

Tribe Gempylodini Sharp, 1893

Tribe Nematidiini Horn, 1878

Tribe Orthocerini Blanchard, 1845

Tribe Rhagoderini Horn, 1878

Tribe Rhopalocerini Reitter, 1911

Tribe Synchitini Erichson, 1845

**Subfamily Zopherinae Solier, 1834**

Tribe Latometini Ślipiński and Lawrence, 1999

Tribe Monommatini Blanchard, 1845

Tribe Phellopsini Ślipiński and Lawrence, 1999

Tribe Pycnomerini Erichson, 1845

Tribe Usechini Horn, 1867

Tribe Zopherini Solier, 1834

**Family Ulodidae Pascoe, 1869**

**Family Promecheilidae Lacordaire, 1859**

**Family Chalcodryidae Watt, 1974**

**Family Trachelostenidae Lacordaire, 1859**

**Family Tenebrionidae Latreille, 1802**

**Subfamily Zolodininae Watt, 1975**

**Subfamily Lagriinae Latreille, 1825 (1820)**

Tribe Adeliini Kirby, 1828

Tribe Belopini Reitter, 1917

Tribe Chaerodini Doyen, Matthews and Lawrence, 1990

Tribe Cossyphini Latreille, 1802

Tribe Goniaderini Lacordaire, 1859

Tribe Laenini Seidlitz, 1895

Tribe Lagriini Latreille, 1825 (1820)

Subtribe Lagriina Latreille, 1825 (1820)

Subtribe Statirina Blanchard, 1845

Tribe Lupropini Ardoin, 1958

Tribe Pycnocerini Lacordaire, 1859 nomen protectum

**Subfamily Nilioninae Oken, 1843**

**Subfamily Phrenapatinae Solier, 1834**

Tribe Archaeoglenini Watt, 1975

Tribe Penetini Lacordaire, 1859

Tribe Phrenapatini Solier, 1834

**Subfamily Pimeliinae Latreille, 1802**

Tribe Adelostomini Solier, 1834

Tribe Adesmiini Lacordaire, 1859 nomen protectum

Tribe Akidini Billberg, 1820

Tribe Anepsiini LeConte, 1862

Tribe Asidini Fleming, 1821

Tribe Boromorphini Skopin, 1978

Tribe Branchini LeConte, 1862

Tribe Caenocrypticini Koch, 1958

Tribe Ceratanisini Gebien, 1937

Tribe Cnemeplatiini Jacquelin du Val, 1861

Subtribe Actizetina Watt, 1992

Subtribe Cnemeplatiina Jacquelin du Val, 1861

Subtribe Rondoniellina Ferrer and Moragues, 2000

Subtribe Thorictosomatina Watt, 1992

Tribe Cnemodinini Gebien, 1910

Tribe Coniontini Waterhouse, 1858

Tribe Cossyphodini Wasmann, 1899

Subtribe Cossyphodina Wasmann, 1899

Subtribe Cossyphoditina Basilewsky, 1950

Subtribe Esemephina Steiner, 1980

Subtribe Paramellonina Andreae, 1961

Tribe Cryptochilini Solier, 1841

Subtribe Calognathina Lacordaire, 1859

Subtribe Cryptochilina Solier, 1841

Subtribe Homebiina Endrödy-Younga, 1989

Subtribe Horatomina Koch, 1955

Subtribe Vansoniina Koch, 1955

Tribe Cryptoglossini LeConte, 1862 nomen protectum

Tribe Edrotini Lacordaire, 1859

Tribe Elenophorini Solier, 1837

Tribe Epitragini Blanchard, 1845 nomen protectum

Tribe Erodiini Billberg, 1820 nomen protectum

Tribe Evaniosomini Lacordaire, 1859

Tribe Falsomycterini Gebien, 1910

Tribe Idisiini Medvedev, 1973

Tribe Klewariini Gebien, 1910

Tribe Kuhitangiini Medvedev, 1962

Tribe Lachnogyini Seidlitz, 1894

Subtribe Lachnodactylina Reitter, 1904

Subtribe Lachnogyina Seidlitz, 1894

Subtribe Netuschiliina Ferrer and Yvinec, 2004

Tribe Leptodini Lacordaire, 1859

Tribe Nycteliini Solier, 1834

Tribe Nyctoporini Lacordaire, 1859

Tribe Phrynocarenini Gebien, 1928

Tribe Physogasterini Lacordaire, 1859

Tribe Pimeliini Latreille, 1802

Tribe Praociini Eschscholtz, 1829

Tribe Sepidiini Eschscholtz, 1829

Subtribe Hypomelina Koch, 1955

Subtribe Molurina Solier, 1834

Subtribe Oxurina Koch, 1955

Subtribe Phanerotomeina Koch, 1958

Subtribe Sepidiina Eschscholtz, 1829

Subtribe Trachynotina Koch, 1955

Tribe Stenosini Schaum, 1859 (1834)

Tribe Tentyriini Eschscholtz, 1831

Tribe Thinobatini Lacordaire, 1859

Tribe Trilobocarini Lacordaire, 1859

Tribe Vacronini Gebien, 1910

Tribe Zophosini Solier, 1834

**Subfamily Tenebrioninae Latreille, 1802**

Tribe Acropteronini Doyen, 1989

Tribe Alphitobiini Reitter, 1917

Tribe Amarygmini Gistel, 1848

Tribe Amphidorini LeConte, 1862

Tribe Apocryphini Lacordaire, 1859

Tribe Blaptini Leach, 1815

Subtribe Blaptina Leach, 1815

Subtribe Gnaptorina Medvedev, 2001

Subtribe Gnaptorinina Medvedev, 2001

Subtribe Prosodina Skopin, 1960

Subtribe Remipedellina Semenov, 1907

Tribe Bolitophagini Kirby, 1837 nomen protectum

Tribe Centronopini Doyen, 1989

Tribe Cerenopini Horn, 1870

Tribe Dissonomini Medvedev, 1968

Tribe Eulabini Horn, 1870

Tribe Falsocossyphini Ferrer, 2006

Tribe Heleini Fleming, 1821

Subtribe Asphalina Matthews and Lawrence, 2005

Subtribe Cyphaleina Lacordaire, 1859

Subtribe Heleina Fleming, 1821

Tribe Helopini Latreille, 1802

Subtribe Helopina Latreille, 1802

Subtribe Cylindrinotina Español, 1956

Tribe Helopinini Lacordaire, 1859

Subtribe Aptilina Koch, 1958

Subtribe Helopinina Lacordaire, 1859

Subtribe Micrantereina Reitter, 1917

Subtribe Oncosomina Koch, 1958

Tribe Melanimonini Seidlitz, 1894 (1854)

Tribe Opatrini Brullé, 1832

Subtribe Heterocheirina Koch, 1956

Subtribe Heterotarsina Blanchard, 1845

Subtribe Opatrina Brullé, 1832

Subtribe Neopachypterina Bouchard, Löbl and Merkl, 2007

Tribe Palorini Matthews, 2003

Tribe Pedinini Eschscholtz, 1829

Subtribe Dendarina Mulsant and Rey, 1854

Subtribe Eurynotina Mulsant and Rey, 1854

Subtribe Leichenina Mulsant, 1854

Subtribe Loensina Koch, 1956

Subtribe Melambiina Mulsant and Rey, 1854

Subtribe Pedinina Eschscholtz, 1829

Subtribe Platynotina Mulsant and Rey, 1853

Subtribe Pythiopina Koch, 1953

Tribe Platyscelidini Lacordaire, 1859

Tribe Praeugenini De Moor, 1970

Tribe Rhysopaussini Wasmann, 1896

Tribe Scaurini Billberg, 1820

Tribe Scotobiini Solier, 1838

Tribe Tenebrionini Latreille, 1802

Tribe Titaenini Fauvel, 1905

Tribe Toxicini Oken, 1843

Subtribe Eudysantina Bouchard, Lawrence, Davies and Newton, 2005

Subtribe Nycteropina Lacordaire, 1859

Subtribe Toxicina Oken, 1843

Tribe Triboliini Gistel, 1848

Tribe Ulomini Blanchard, 1845

**Subfamily Alleculinae Laporte, 1840**

Tribe Alleculini Laporte, 1840

Subtribe Alleculina Laporte, 1840

Subtribe Gonoderina Seidlitz, 1896

Subtribe Mycetocharina Gistel, 1848

Subtribe Xystropodina Solier, 1835

Tribe Cteniopodini Solier, 1835

**Subfamily Diaperinae Latreille, 1802**

Tribe Crypticini Brullé, 1832

Tribe Diaperini Latreille, 1802

Subtribe Adelinina LeConte, 1862

Subtribe Diaperina Latreille, 1802

Tribe Ectychini Doyen, Matthews and Lawrence, 1990

Tribe Gnathidiini Gebien, 1921

Subtribe Anopidiina Jeannel and Paulian, 1945

Subtribe Gnathidiina Gebien, 1921

Tribe Hyociini Medvedev and Lawrence, 1982

Subtribe Brittonina Medvedev and Lawrence, 1986

Subtribe Hyociina Medvedev and Lawrence, 1982

Subtribe Uptonina Medvedev and Lawrence, 1986

Tribe Hypophlaeini Billberg, 1820

Tribe Leiochrinini Lewis, 1894

Tribe Myrmechixenini Jacquelin du Val, 1858

Tribe Phaleriini Blanchard, 1845

Tribe Scaphidemini Reitter, 1922

Tribe Trachyscelini Blanchard, 1845

**Subfamily Stenochiinae Kirby, 1837**

Tribe Cnodalonini Oken, 1843

Tribe Stenochiini Kirby, 1837

Tribe Talanini Champion, 1887 (1883)

**Family Prostomidae Thomson, 1859**

**Family Synchroidae Lacordaire, 1859**

**Family Stenotrachelidae Thomson, 1859**

**Subfamily Stenotrachelinae Thomson, 1859**

**Subfamily Cephaloinae LeConte, 1862**

**Subfamily Nematoplinae LeConte, 1862**

**Subfamily Stoliinae Nikitsky, 1985**

**Family Oedemeridae Latreille, 1810**

**Subfamily Polypriinae Lawrence, 2005**

**Subfamily Calopodinae Costa, 1852** nomen protectum

**Subfamily Oedemerinae Latreille, 1810**

Tribe Asclerini Gistel, 1848

Tribe Ditylini Mulsant, 1858

Tribe Nacerdini Mulsant, 1858

Tribe Oedemerini Latreille, 1810

Tribe Stenostomatini Mulsant, 1858

**Family Meloidae Gyllenhal, 1810**

**Subfamily Eleticinae Wellman, 1910**

Tribe Derideini Wellman, 1910

Tribe Eleticini Wellman, 1910

Subtribe Eleticina Wellman, 1910

Subtribe Eospastina Selander, 1966

Tribe Ertlianini Selander, 1966

Tribe Spasticini Kaszab, 1959

Subtribe Anthicoxenina Selander, 1966

Subtribe Protomeloina Abdullah, 1965

Subtribe Spasticina Kaszab, 1959

Subtribe Xenospastina Selander, 1966

**Subfamily Meloinae Gyllenhal, 1810**

Tribe Cerocomini Leach, 1815

Tribe Epicautini Parker and Böving, 1924

Tribe Eupomphini LeConte, 1862

Tribe Lyttini Solier, 1851

Tribe Meloini Gyllenhal, 1810

Tribe Mylabrini Rafinesque, 1815

Tribe Pyrotini MacSwain, 1956

**Subfamily Tetraonycinae Böving and Craighead, 1931**

**Subfamily Nemognathinae Laporte, 1840**

Tribe Horiini Latreille, 1802

Tribe Nemognathini Laporte, 1840

Subtribe Nemognathina Laporte, 1840

Subtribe Zonitidina Mulsant, 1857

Subtribe Sitarina Mulsant, 1857

Tribe Stenoderini Selander, 1991

**Family Mycteridae Oken, 1843**

**Subfamily Mycterinae Oken, 1843**

**Subfamily Eurypinae Thomson, 1860**

**Subfamily Hemipeplinae Lacordaire, 1854**

**Family Boridae Thomson, 1859**

**Subfamily Borinae Thomson, 1859**

**Subfamily Synercticinae Lawrence and Pollock, 1994**

**Family Trictenotomidae Blanchard, 1845**

**Family Pythidae Solier, 1834**

**Family Pyrochroidae Latreille, 1806**

**Subfamily Tydessinae Nikitsky, 1986**

**Subfamily Pilipalpinae Abdullah, 1964**

**Subfamily Pedilinae Lacordaire, 1859**

**Subfamily Pyrochroinae Latreille, 1806**

**Subfamily Agnathinae Lacordaire, 1859**

**Family Salpingidae Leach, 1815**

**Subfamily Othniinae LeConte, 1861**

**Subfamily Prostominiinae Grouvelle, 1914**

**Subfamily Agleninae Horn, 1878**

**Subfamily Inopeplinae Grouvelle, 1908**

**Subfamily Dacoderinae LeConte, 1862**

**Subfamily Aegialitinae LeConte, 1862**

**Subfamily Salpinginae Leach, 1815**

**Family Anthicidae Latreille, 1819**

**Subfamily Eurygeniinae LeConte, 1862**

Tribe Eurygeniini LeConte, 1862

Tribe Ictistygnini Borchmann, 1936

Tribe Mitraelabrini Abdullah, 1969

**Subfamily Macratriinae LeConte, 1862**

Tribe Macratriini LeConte, 1862

†Tribe Camelomorphini Kirejtshuk and Azar, 2008

**Subfamily Steropinae Jacquelin du Val, 1863**

**Subfamily Copobaeninae Abdullah, 1969**

**Subfamily Lemodinae Lawrence and Britton, 1991**

**Subfamily Tomoderinae Bonadona, 1961**

**Subfamily Anthicinae Latreille, 1819**

Tribe Anthicini Latreille, 1819

Tribe Endomiini Kaszab, 1956

Tribe Formicomini Bonadona, 1974

Tribe Microhoriini Bonadona, 1974

**Subfamily Notoxinae Stephens, 1829**

**Family Aderidae Csiki, 1909**

Tribe Aderini Csiki, 1909

Subtribe Aderina Csiki, 1909

Subtribe Cnopina Báguena Corella, 1948

Subtribe Gompeliina Bouchard, 2011

Subtribe Syzetoninina Báguena Corella, 1948

Tribe Emelinini Báguena Corella, 1948

Tribe Euglenesini Seidlitz, 1875

Subtribe Euglenesina Seidlitz, 1875

Subtribe Pseudolotelina Báguena Corella, 1948

Tribe Phytobaenini Báguena Corella, 1948

**Family Scraptiidae Gistel, 1848**

**Subfamily Scraptiinae Gistel, 1848**

Tribe Allopodini Franciscolo, 1964

Tribe Scraptiini Gistel, 1848

**Subfamily Anaspidinae Mulsant, 1856**

Tribe Anaspidini Mulsant, 1856

Tribe Anaspimordini Franciscolo, 1954

Tribe Menuthianaspidini Franciscolo, 1972

Tribe Pentariini Franciscolo, 1954

**Subfamily Lagrioidinae Abdullah and Abdullah, 1968**

**Subfamily Afreminae Levey, 1985**

**Subfamily Ischaliinae Blair, 1920**

**Superfamily Chrysomeloidea Latreille, 1802**

**Family Oxypeltidae Lacordaire, 1868**

**Family Vesperidae Mulsant, 1839**

**Subfamily Philinae Thomson, 1861**

**Subfamily Vesperinae Mulsant, 1839**

**Subfamily Anoplodermatinae Guérin-Méneville, 1840**

Tribe Anoplodermatini Guérin-Méneville, 1840

Tribe Hypocephalini Blanchard, 1845

Tribe Mysteriini Prosen, 1960

**Family Disteniidae Thomson, 1861**

Tribe Cyrtonopini Gressitt, 1940

Tribe Disteniini Thomson, 1861

Tribe Dynamostini Lacordaire, 1868

Tribe Heteropalpini Villiers, 1961

**Family Cerambycidae Latreille, 1802**

**Subfamily Parandrinae Blanchard, 1845**

Tribe Erichsoniini Thomson, 1861

Tribe Parandrini Blanchard, 1845

**Subfamily Prioninae Latreille, 1802**

Tribe Acanthophorini Thomson, 1864

Tribe Aegosomatini Thomson, 1861

Tribe Anacolini Thomson, 1857

Tribe Cacoscelini Thomson, 1861

Tribe Callipogonini Thomson, 1861

Tribe Calocomini Galileo and Martins, 1993

Tribe Cantharocnemini Thomson, 1861

Tribe Ergatini Fairmaire, 1864

Tribe Eurypodini Gahan, 1906 (1868)

Tribe Hopliderini Thomson, 1864

Tribe Macrodontiini Thomson, 1861

Tribe Macrotomini Thomson, 1861

Subtribe Archetypina Lameere, 1912

Subtribe Basitoxina Lameere, 1912

Subtribe Macrotomina Thomson, 1861

Subtribe Platygnathina Gilmour, 1954

Subtribe Xixuthrina Lameere, 1912

Tribe Mallaspini Thomson, 1861

Tribe Mallodonini Thomson, 1861

Tribe Meroscelisini Thomson, 1861

Tribe Prionini Latreille, 1802

Tribe Remphanini Lacordaire, 1868

Tribe Solenopterini Lacordaire, 1868

Tribe Tereticini Lameere, 1913

Tribe Vesperoctenini Vives, 2005

**Subfamily Lepturinae Latreille, 1802**

Tribe Desmocerini Blanchard, 1845

Tribe Encyclopini LeConte, 1873

Tribe Lepturini Latreille, 1802

Tribe Oxymirini Danilevsky, 1997

Tribe Rhagiini Kirby, 1837

Tribe Rhamnusiini Sama, 2009

Tribe Teledapini Pascoe, 1871

Tribe Sachalinobiini Danilevsky, 2010

Tribe Xylosteini Reitter, 1913

**Subfamily Spondylidinae Audinet-Serville, 1832**

Tribe Anisarthrini Mamaev and Danilevsky, 1973

Tribe Asemini Thomson, 1861

Tribe Atimiini LeConte, 1873

Tribe Saphanini Gistel, 1848

Tribe Spondylidini Audinet-Serville, 1832

**Subfamily Necydalinae Latreille, 1825**

**Subfamily Dorcasominae Lacordaire, 1868**

**Subfamily Apatophyseinae Lacordaire, 1869**

**Subfamily Cerambycinae Latreille, 1802**

Tribe Acangassuini Galileo and Martins, 2001

Tribe Achrysonini Lacordaire, 1868

Tribe Agallissini LeConte, 1873

Tribe Alanizini Di Iorio, 2003

Tribe Anaglyptini Lacordaire, 1868 nomen protectum

Tribe Aphanasiini Lacordaire, 1868

Tribe Aphneopini Lacordaire, 1868

Tribe Auxesini Lepesme and Breuning, 1952

Tribe Basipterini Fragoso, Monné and Campos Seabra, 1987

Tribe Bimiini Lacordaire, 1868

Tribe Bothriospilini Lane, 1950

Tribe Brachypteromatini Sama, 2008

Tribe Callichromatini Swainson, 1840

Tribe Callidiini Kirby, 1837

Tribe Callidiopini Lacordaire, 1868

Tribe Cerambycini Latreille, 1802

Subtribe Cerambycina Latreille, 1802

Subtribe Sphallotrichina Martins and Monné, 2005

Tribe Certallini Fairmaire, 1864

Tribe Chlidonini Waterhouse, 1879

Tribe Cleomenini Lacordaire, 1868

Tribe Clytini Mulsant, 1839

Tribe Compsocerini Thomson, 1864

Tribe Coptommatini Lacordaire, 1869

Tribe Curiini LeConte, 1873

Tribe Deilini Fairmaire, 1864

Tribe Dejanirini Lacordaire, 1868

Tribe Diorini Lane, 1950

Tribe Distichocerini Pascoe, 1867

Tribe Dodecosini Aurivillius, 1912

Tribe Dryobiini Arnett, 1962 nomen protectum

Tribe Eburiini Blanchard, 1845

Tribe Ectenessini Martins, 1998

Tribe Elaphidiini Thomson, 1864

Tribe Eligmodermini Lacordaire, 1868

Tribe Erlandiini Aurivillius, 1912

Tribe Eroschemini Lacordaire, 1868

Tribe Eumichthini Linsley, 1940

Tribe Gahaniini Quentin and Villiers, 1969

Tribe Glaucytini Lacordaire, 1868

Tribe Graciliini Mulsant, 1839

Tribe Hesperophanini Mulsant, 1839

Subtribe Daramina Sama, 2008

Subtribe Hesperophanina Mulsant, 1839

Tribe Hesthesini Pascoe, 1867

Tribe Heteropsini Lacordaire, 1868 nomen protectum

Tribe Hexoplini Martins, 2006

Tribe Holopleurini Chemsak and Linsley, 1974

Tribe Holopterini Lacordaire, 1868

Tribe Hyboderini Linsley, 1940

Tribe Hylotrupini Zagajkevich, 1991

Tribe Ibidionini Thomson, 1861

Subtribe Compsina Martins and Galileo, 2007

Subtribe Ibidionina Thomson, 1861

Subtribe Tropidina Martins and Galileo, 2007

Tribe Ideratini Martins and Napp, 2009

Tribe Lissonotini Swainson, 1840

Tribe Luscosmodicini Martins, 2003

Tribe Lygrini Sama, 2008

Tribe Macronini Lacordaire, 1868

Tribe Megacoelini Quentin and Villiers, 1969

Tribe Methiini Thomson, 1860

Tribe Molorchini Gistel, 1848

Tribe Mythodini Lacordaire, 1868

Tribe Necydalopsini Lacordaire, 1868

Tribe Neocorini Martins, 2005

Tribe Neostenini Lacordaire, 1868

Tribe Obriini Mulsant, 1839

Tribe Ochyrini Pascoe, 1871

Tribe Oedenoderini Aurivillius, 1912

Tribe Oemini Lacordaire, 1868

Subtribe Methioidina Martins, 1997

Subtribe Oemina Lacordaire, 1868

Tribe Opsimini LeConte, 1873

Tribe Oxycoleini Martins and Galileo, 2003

Tribe Paraholopterini Martins, 1997

Tribe Phalotini Lacordaire, 1868

Tribe Phlyctaenodini Lacordaire, 1868

Tribe Phoracanthini Newman, 1840

Tribe Phyllarthriini Lepesme and Breuning, 1956

Tribe Piesarthriini McKeown, 1947

Tribe Piezocerini Lacordaire, 1868

Subtribe Haruspicina Martins, 1976

Subtribe Piezocerina Lacordaire, 1868

Tribe Platyarthrini Bates, 1870

Tribe Plectogasterini Quentin and Villiers, 1969

Tribe Plectromerini Nearns and Braham, 2008

Tribe Pleiarthrocerini Lane, 1950

Tribe Protaxini Gahan, 1906

Tribe Prothemini Lacordaire, 1868

Tribe Psebiini Lacordaire, 1868

Tribe Pseudocephalini Aurivillius, 1912 (1861)

Tribe Pseudolepturini Thomson, 1861

Tribe Psilomorphini Lacordaire, 1868

Tribe Pteroplatini Thomson, 1861

Tribe Pyrestini Lacordaire, 1868

Tribe Rhagiomorphini Newman, 1841

Tribe Rhinotragini Thomson, 1861

Tribe Rhopalophorini Blanchard, 1845

Tribe Rosaliini Fairmaire, 1864

Tribe Sestyrini Lacordaire, 1868

Tribe Smodicini Lacordaire, 1868

Tribe Spintheriini Lacordaire, 1869

Tribe Stenhomalini Miroshnikov, 1989

Tribe Stenoderini Pascoe, 1867

Tribe Stenopterini Gistel, 1848

Tribe Strongylurini Lacordaire, 1868

Tribe Tessarommatini Lacordaire, 1868

Tribe Thraniini Gahan, 1906

Tribe Thyrsiini Marinoni and Napp, 1984

Tribe Tillomorphini Lacordaire, 1868

Tribe Torneutini Thomson, 1861

Tribe Trachyderini Dupont, 1836

Subtribe Ancylocerina Thomson, 1864

Subtribe Trachyderina Dupont, 1836

Tribe Tragocerini Pascoe, 1867

Tribe Trichomesiini Aurivillius, 1912

Tribe Tropocalymmatini Lacordaire, 1868

Tribe Typhocesini Lacordaire, 1868

Tribe Unxiini Napp, 2007

Tribe Uracanthini Blanchard, 1853

Tribe Vesperellini Sama, 2008

Tribe Xystrocerini Blanchard, 1845

**Subfamily Lamiinae Latreille, 1825**

Tribe Acanthocinini Blanchard, 1845

Tribe Acanthoderini Thomson, 1860

Tribe Acmocerini Thomson, 1864

Tribe Acridocephalini Dillon and Dillon, 1959

Tribe Acrocinini Swainson, 1840

Tribe Aderpasini Breuning and Teocchi, 1978

Tribe Aerenicini Lacordaire, 1872

Tribe Agapanthiini Mulsant, 1839

Tribe Amphoecini Breuning, 1951

Tribe Ancitini Aurivillius, 1917

Tribe Ancylonotini Lacordaire, 1869

Tribe Anisocerini Thomson, 1860

Tribe Apomecynini Thomson, 1860

Tribe Astathini Thomson, 1864

Tribe Batocerini Thomson, 1864

Tribe Calliini Thomson, 1864

Tribe Ceroplesini Thomson, 1860

Subtribe Ceroplesina Thomson, 1860

Subtribe Crossotina Thomson, 1864

Tribe Cloniocerini Lacordaire, 1872

Tribe Colobotheini Thomson, 1860

Tribe Compsosomatini Thomson, 1857

Tribe Cyrtinini Thomson, 1864

Tribe Desmiphorini Thomson, 1860

Tribe Dorcadionini Swainson, 1840

Tribe Dorcaschematini Thomson, 1860

Tribe Elytracanthinini Bousquet, 2009

Tribe Enicodini Thomson, 1864

Tribe Eupromerini Galileo and Martins, 1995

Tribe Forsteriini Tippmann, 1960

Tribe Gnomini Thomson, 1860

Tribe Gyaritini Breuning, 1950

Tribe Heliolini Breuning, 1951

Tribe Hemilophini Thomson, 1868 nomen protectum

Tribe Homonoeini Thomson, 1864

Tribe Hyborhabdini Aurivillius, 1911

Tribe Lamiini Latreille, 1825

Tribe Laticraniini Lane, 1959

Tribe Mauesiini Lane, 1956

Tribe Megabasini Thomson, 1860

Tribe Mesosini Mulsant, 1839

Tribe Microcymaturini Breuning and Teocchi, 1985

Tribe Moneilemini Thomson, 1864

Tribe Monochamini Gistel, 1848

Tribe Morimonellini Lobanov, Danilevsky and Murzin, 1981

Tribe Morimopsini Lacordaire, 1869

Tribe Nyctimeniini Gressitt, 1951

Tribe Obereini Thomson, 1864

Tribe Oculariini Breuning, 1950

Tribe Onciderini Thomson, 1860

Tribe Oncideropsidini Aurivillius, 1922

Tribe Onocephalini Thomson, 1860

Tribe Onychogleneini Aurivillius, 1923

Tribe Parmenini Mulsant, 1839

Tribe Petrognathini Blanchard, 1845

Tribe Phacellini Lacordaire, 1872

Tribe Phantasini Kolbe, 1897

Tribe Phrynetini Thomson, 1864

Tribe Phymasternini Teocchi, 1989

Tribe Phytoeciini Mulsant, 1839

Tribe Pogonocherini Mulsant, 1839

Tribe Polyrhaphidini Thomson, 1860

Tribe Pretiliini Martins and Galileo, 1990

Tribe Proctocerini Aurivillius, 1922

Tribe Prosopocerini Thomson, 1864

Tribe Pteropliini Thomson, 1860

Tribe Rhodopinini Gressitt, 1951

Tribe Saperdini Mulsant, 1839

Tribe Stenobiini Breuning, 1950

Tribe Sternotomini Thomson, 1860

Tribe Tapeinini Thomson, 1857

Tribe Tetraopini Thomson, 1860

Tribe Tetraulaxini Breuning and Teocchi, 1977

Tribe Tetropini Portevin, 1927

Tribe Theocrini Lacordaire, 1872

Tribe Tmesisternini Blanchard, 1853

Tribe Tragocephalini Thomson, 1857

Tribe Xenicotelini Matsushita, 1933

Tribe Xenofreini Aurivillius, 1923

Tribe Xenoleini Lacordaire, 1872

Tribe Xylorhizini Lacordaire, 1872

Tribe Zygocerini Thomson, 1864

**Family Megalopodidae Latreille, 1802**

**Subfamily Megalopodinae Latreille, 1802**

**Subfamily Palophaginae Kuschel and May, 1990**

**Subfamily Zeugophorinae Böving and Craighead, 1931**

**Family Orsodacnidae Thomson, 1859**

**Subfamily Orsodacninae Thomson, 1859**

**Subfamily Aulacoscelidinae Chapuis, 1874**

**Family Chrysomelidae Latreille, 1802**

**Subfamily Sagrinae Leach, 1815**

Tribe Carpophagini Chapuis, 1874

Tribe Diaphanopsidini Monrós, 1958

Tribe Megamerini Chapuis, 1874

Tribe Sagrini Leach, 1815

**Subfamily Bruchinae Latreille, 1802**

Tribe Amblycerini Bridwell, 1932

Subtribe Amblycerina Bridwell, 1932

Subtribe Spermophagina Borowiec, 1987

Tribe Bruchini Latreille, 1802

Subtribe Acanthoscelidina Bridwell, 1946

Subtribe Bruchina Latreille, 1802

Subtribe Megacerina Bridwell, 1946

Tribe Eubaptini Bridwell, 1932

Tribe Kytorhinini Bridwell, 1932

Tribe Pachymerini Bridwell, 1929

Subtribe Caryedontina Bridwell, 1929

Subtribe Caryopemina Bridwell, 1929

Subtribe Pachymerina Bridwell, 1929

Tribe Rhaebini Blanchard, 1845

**Subfamily Donaciinae Kirby, 1837**

Tribe Donaciini Kirby, 1837

Tribe Haemoniini Chen, 1941

Tribe Plateumarini Böving, 1922

**Subfamily Criocerinae Latreille, 1804**

Tribe Criocerini Latreille, 1804

Tribe Lemini Gyllenhal, 1813

Tribe Pseudocriocerini Heinze, 1962

**Subfamily Cassidinae Gyllenhal, 1813**

Tribe Alurnini Chapuis, 1875

Tribe Anisoderini Chapuis, 1875

Tribe Aproidini Weise, 1911

Tribe Arescini Chapuis, 1875

Tribe Aspidimorphini Chapuis, 1875

Tribe Basiprionotini Gressitt, 1952 (1929)

Tribe Botryonopini Chapuis, 1875

Tribe Callispini Chapuis, 1875

Tribe Callohispini Uhmann, 1960

Tribe Cassidini Gyllenhal, 1813

Tribe Cephaloleiini Chapuis, 1875

Tribe Chalepini Weise, 1910

Tribe Coelaenomenoderini Weise, 1911

Tribe Cryptonychini Chapuis, 1875

Tribe Delocraniini Spaeth, 1929

Tribe Dorynotini Monrós and Viana, 1949 (1923)

Tribe Eugenysini Hincks, 1952

Tribe Eurispini Chapuis, 1875

Tribe Exothispini Weise, 1911

Tribe Goniocheniini Spaeth, 1942

Tribe Gonophorini Chapuis, 1875

Tribe Hemisphaerotini Monrós and Viana, 1951 (1929)

Tribe Hispini Gyllenhal, 1813

Tribe Hispoleptini Chapuis, 1875

Tribe Hybosispini Weise, 1910

Tribe Imatidiini Hope, 1840

Tribe Ischyrosonychini Chapuis, 1875

Tribe Leptispini Fairmaire, 1868

Tribe Mesomphaliini Hope, 1840

Tribe Nothosacanthini Gressitt, 1952 (1929)

Tribe Oediopalpini Monrós and Viana, 1947 (1910)

Tribe Omocerini Hincks, 1952 (1923)

Tribe Oncocephalini Chapuis, 1875

Tribe Promecothecini Chapuis, 1875

Tribe Prosopodontini Weise, 1910

Tribe Sceloenoplini Uhmann, 1930

Tribe Spilophorini Chapuis, 1875

Tribe Uroplatini Weise, 1910

**Subfamily Chrysomelinae Latreille, 1802**

Tribe Chrysomelini Latreille, 1802

Tribe Timarchini Motschulsky, 1860

**Subfamily Galerucinae Latreille, 1802**

Tribe Alticini Newman, 1834

Tribe Decarthrocerini Laboissière, 1937

Tribe Galerucini Latreille, 1802

Tribe Hylaspini Chapuis, 1875

Tribe Luperini Gistel, 1848

Tribe Metacyclini Chapuis, 1875

Tribe Oidini Laboissière, 1921 (1875)

**Subfamily Lamprosomatinae Lacordaire, 1848**

Tribe Lamprosomatini Lacordaire, 1848

Tribe Neochlamysini Monrós, 1959

Tribe Sphaerocharini Chapuis, 1874

**Subfamily Cryptocephalinae Gyllenhal, 1813**

Tribe Clytrini Kirby, 1837

Tribe Cryptocephalini Gyllenhal, 1813

Subtribe Achaenopina Chapuis, 1874

Subtribe Cryptocephalina Gyllenhal, 1813

Subtribe Monachulina Leng, 1920

Subtribe Pachybrachina Chapuis, 1874

Subtribe Stylosomina Chapuis, 1874

Tribe Fulcidacini Jakobson, 1924

**Subfamily Eumolpinae Hope, 1840**

Tribe Bromiini Baly, 1865 (1863)

Tribe Caryonodini Bechyné, 1951

Tribe Cubispini Monrós, 1954

Tribe Eumolpini Hope, 1840

Tribe Euryopini Chapuis, 1874

Tribe Habrophorini Bechyné and Špringlová de Bechyné, 1969

Tribe Hemydacnini Bechyné, 1951

Tribe Megascelidini Chapuis, 1874

Tribe Merodini Chapuis, 1874

Tribe Pygomolpini Bechyné, 1949

Tribe Rosiroiini Bechyné, 1950

Tribe Typophorini Baly, 1865

**Subfamily Spilopyrinae Chapuis, 1874**

**Subfamily Synetinae LeConte and Horn, 1883**

**†Subfamily Protoscelidinae Medvedev, 1968**

**Superfamily Curculionoidea Latreille, 1802**

**Family Nemonychidae Bedel, 1882**

**Subfamily Nemonychinae Bedel, 1882**

**Subfamily Cimberidinae Gozis, 1882**

Tribe Cimberidini Gozis, 1882

Tribe Doydirhynchini Pierce, 1916

†Tribe Kuschelomacrini Riedel, 2010

**Subfamily Rhinorhynchinae Voss, 1922**

Tribe Mecomacerini Kuschel, 1994

Subtribe Brarina Legalov, 2009

Subtribe Mecomacerina Kuschel, 1994

Tribe Rhinorhynchini Voss, 1922

**†Subfamily Slonikinae Zherikhin, 1977**

†Tribe Slonikini Zherikhin, 1977

†Tribe Ulyaniscini Legalov, 2009

**†Subfamily Eccoptarthrinae Arnoldi, 1977**

**†Subfamily Brenthorrhininae Arnoldi, 1977**

†Tribe Brenthorrhinini Arnoldi, 1977

†Tribe Brenthorrhinoidini Legalov, 2003

**†Subfamily Distenorrhininae Arnoldi, 1977**

**†Subfamily Eobelinae Arnoldi, 1977**

†Tribe Eobelini Arnoldi, 1977

†Subtribe Eobelina Arnoldi, 1977

†Subtribe Procurculionina Arnoldi, 1977

†Tribe Karataucarini Legalov, 2009

†Tribe Nanophydini Arnoldi, 1977

†Tribe Oxycorynoidini Arnoldi, 1977

†Tribe Probelini Legalov, 2009

**†Subfamily Paleocartinae Legalov, 2003**

†Tribe Nebrenthorrhinini Legalov, 2007

†Tribe Paleocartini Legalov, 2003

**†Subfamily Metrioxenoidinae Legalov, 2009**

**†Subfamily Cretonemonychinae Gratshev and Legalov, 2009**

**†Subfamily Selengarhynchinae Gratshev and Legalov, 2009**

**Family Anthribidae Billberg, 1820**

**Subfamily Anthribinae Billberg, 1820**

Tribe Anthribini Billberg, 1820

Tribe Basitropini Lacordaire, 1865

Tribe Corrhecerini Lacordaire, 1865

Tribe Cratoparini LeConte, 1876

†Tribe Cretanthribini Legalov, 2009

Tribe Decataphanini Lacordaire, 1865

Tribe Discotenini Lacordaire, 1865

Tribe Ecelonerini Lacordaire, 1865

Tribe Ischnocerini Lacordaire, 1865

Tribe Gymnognathini Valentine, 1960

Tribe Jordanthribini Morimoto, 1980

Tribe Mauiini Valentine, 1990

Tribe Mecocerini Lacordaire, 1865

Tribe Mycteini Morimoto, 1972

Tribe Ozotomerini Morimoto, 1972

Tribe Piesocorynini Valentine, 1960

Tribe Platyrhinini Bedel, 1882

Tribe Platystomini Pierce, 1916

Tribe Proscoporhinini Lacordaire, 1865

Tribe Ptychoderini Jekel, 1855

Tribe Sintorini Lacordaire, 1865

Tribe Stenocerini Kolbe, 1895

Tribe Tophoderini Lacordaire, 1865

Tribe Trigonorhinini Valentine, 1999

Tribe Tropiderini Lacordaire, 1865

Tribe Xenocerini Lacordaire, 1865

Tribe Xylinadini Lacordaire, 1865

Tribe Zygaenodini Lacordaire, 1865

**Subfamily Choraginae Kirby, 1819**

Tribe Apolectini Lacordaire, 1865

Tribe Araecerini Lacordaire, 1865

Tribe Cisanthribini Zimmerman, 1994

Tribe Choragini Kirby, 1819

Tribe Valenfriesiini Alonso-Zarazaga and Lyal, 1999

Tribe Xenorchestini Lacordaire, 1865

**Subfamily Urodontinae Thomson, 1859**

**†Family Ulyanidae Zherikhin, 1993**

**Family Belidae Schönherr, 1826**

**Subfamily Belinae Schönherr, 1826**

Tribe Agnesiotidini Zimmerman, 1994

Tribe Belini Schönherr, 1826

Subtribe Belina Schönherr, 1826

Subtribe Homalocerina Legalov, 2009

Tribe Pachyurini Kuschel, 1959

**Subfamily Oxycoryninae Schönherr, 1840**

Tribe Aglycyderini Wollaston, 1864

Tribe Alloxycorynini Legalov, 2009

Tribe Distenorrhinoidini Legalov, 2009

Tribe Metrioxenini Voss, 1953

Subtribe Afrocorynina Voss, 1957

Subtribe Metrioxenina Voss, 1953

Subtribe Zherichinixenina Legalov, 2009

Tribe Oxycorynini Schönherr, 1840

Subtribe Allocorynina Sharp, 1890

Subtribe Oxycorynina Schönherr, 1840

Subtribe Oxycraspedina Marvaldi and Oberprieler, 2006

**Family Caridae Thompson, 1992**

**Subfamily Carinae Thompson, 1992**

**Subfamily Chilecarinae Legalov, 2009**

Tribe Carodini Legalov, 2009

Tribe Chilecarini Legalov, 2009

**†Subfamily Baissorhynchinae Zherikhin, 1993**

**Family Attelabidae Billberg, 1820**

**Subfamily Attelabinae Billberg, 1820**

Tribe Attelabini Billberg, 1820

Subtribe Attelabina Billberg, 1820

Subtribe Euscelina Voss, 1925

Subtribe Euscelophilina Voss, 1925

Subtribe Henicolabina Legalov, 2007

Subtribe Himatolabina Legalov, 2003

Subtribe Hybolabina Voss, 1925

Subtribe Isolabina Legalov, 2007

Subtribe Lagenoderina Voss, 1925

Subtribe Lamprolabina Voss, 1925

Subtribe Metocalolabina Legalov, 2003

Subtribe Omolabina Legalov, 2003

Subtribe Paramecolabina Legalov, 2003

Subtribe Phialodina Legalov, 2003

Subtribe Phymatolabina Voss, 1925

Subtribe Phymatopsinina Legalov, 2003

Subtribe Pleurolabina Legalov, 2003

Tribe Euopini Voss, 1925

Tribe Pilolabini Voss, 1925

**Subfamily Apoderinae Jekel, 1860**

Tribe Apoderini Jekel, 1860

Tribe Clitostylini Voss, 1929

Subtribe Allapoderina Legalov, 2003

Subtribe Clitostylina Voss, 1929

Subtribe Pseudophrysina Legalov, 2003

Tribe Hoplapoderini Voss, 1926

Subtribe Afroapoderina Legalov, 2003

Subtribe Hoplapoderina Voss, 1926

Subtribe Paratomapoderina Legalov, 2003

Tribe Trachelophorini Voss, 1926

**Subfamily Rhynchitinae Gistel, 1848**

Tribe Auletini Desbrochers des Loges, 1908

Subtribe Auletina Desbrochers des Loges, 1908

Subtribe Auletobiina Legalov, 2001

Subtribe Guineauletina Legalov, 2003

Subtribe Mandelschtamiina Legalov, 2003

Subtribe Pseudauletina Voss, 1933

Subtribe Pseudomesauletina Legalov, 2003

Tribe Auletorhinini Voss, 1935

Tribe Byctiscini Voss, 1923

Subtribe Byctiscina Voss, 1923

Subtribe Listrobyctiscina Legalov, 2003

Subtribe Svetlanaebyctiscina Legalov, 2003

Tribe Cesauletini Legalov, 2003

Tribe Deporaini Voss, 1929

Subtribe Chonostropheina Morimoto, 1962

Subtribe Deporaina Voss, 1929

Tribe Minurini Legalov, 2003

Tribe Rhinocartini Voss, 1931

Tribe Rhynchitini Gistel, 1848

Subtribe Acritorrhynchitina Legalov, 2007

Subtribe Anisomerinina Legalov, 2003

Subtribe Eugnamptina Voss, 1930

Subtribe Lasiorhynchitina Legalov, 2003

Subtribe Perrhynchitina Legalov, 2003

Subtribe Rhynchitallina Legalov, 2003

Subtribe Rhynchitina Gistel, 1848

Subtribe Temnocerina Legalov, 2003

**Subfamily Isotheinae Scudder, 1893**

Tribe Isotheini Scudder, 1893

Subtribe Depasophilina Legalov, 2003

†Subtribe Isotheina Scudder, 1893

†Tribe Toxorhynchini Scudder, 1893

**Subfamily Pterocolinae Lacordaire, 1865**

**Family Brentidae Billberg, 1820**

**Subfamily Brentinae Billberg, 1820**

Tribe Brentini Billberg, 1820

Subtribe Arrhenodina Lacordaire, 1865

Subtribe Brentina Billberg, 1820

Subtribe Eremoxenina Semenov, 1892

Tribe Cyladini Schönherr, 1823

Tribe Cyphagogini Kolbe, 1892

Subtribe Atopobrentina Damoiseau, 1965

Subtribe Cyphagogina Kolbe, 1892

†Subtribe Dominibrentina Poinar, 2009

Subtribe Hoplopisthiina Senna and Calabresi, 1919

Subtribe Stereodermina Sharp, 1895

Tribe Pholidochlamydini Damoiseau, 1962

Tribe Taphroderini Lacordaire, 1865

Tribe Trachelizini Lacordaire, 1865

Subtribe Acratina Alonso-Zarazaga, Lyal, Bartolozzi and Sforzi, 1999

Subtribe Ithystenina Lacordaire, 1865

Subtribe Microtrachelizina Zimmerman, 1994

Subtribe Pseudoceocephalina Kleine, 1922

Subtribe Rhyticephalina Kleine, 1922

Subtribe Trachelizina Lacordaire, 1865

Subtribe Tychaeina Schoenfeldt, 1908

Tribe Ulocerini Schönherr, 1823

**Subfamily Eurhynchinae Lacordaire, 1863**

†Tribe Axelrodiellini Legalov, 2009

Tribe Eurhynchini Lacordaire, 1863

**Subfamily Apioninae Schönherr, 1823**

**Supertribe Apionitae Schönherr, 1823**

Tribe Apionini Schönherr, 1823

Subtribe Apionina Schönherr, 1823

Subtribe Aplemonina Kissinger, 1968

Subtribe Aspidapiina Alonso-Zarazaga, 1990

Subtribe Catapiina Alonso-Zarazaga, 1990

Subtribe Ceratapiina Alonso-Zarazaga, 1990

Subtribe Exapiina Alonso-Zarazaga, 1990

Subtribe Ixapiina Alonso-Zarazaga, 1990

Subtribe Kalcapiina Alonso-Zarazaga, 1990

Subtribe Malvapiina Alonso-Zarazaga, 1990

Subtribe Metapiina Alonso-Zarazaga, 1990

Subtribe Oxystomatina Alonso-Zarazaga, 1990

Subtribe Piezotrachelina Voss, 1959

Subtribe Prototrichapiina Wanat, 1995

Subtribe Synapiina Alonso-Zarazaga, 1990

Subtribe Trichapiina Alonso-Zarazaga, 1990

Tribe Chilapiini Wanat, 2001

Tribe Noterapiini Kissinger, 2004

Tribe Podapiini Wanat, 2001

Tribe Rhinorhynchidiini Zimmerman, 1994

**Supertribe Antliarhinitae Schönherr, 1823**

**Supertribe Cybebitae Lacordaire, 1863**

**Supertribe Mecolenitae Wanat, 2001**

**Supertribe Myrmacicelitae Zimmerman, 1994**

Tribe Lispotheriini Wanat, 2001

Tribe Myrmacicelini Zimmerman, 1994

**Supertribe Rhadinocybitae Alonso-Zarazaga, 1992**

Tribe Notapionini Zimmerman, 1994

Tribe Rhadinocybini Alonso-Zarazaga, 1992

**Supertribe Tanaitae Schönherr, 1839**

**Subfamily Ithycerinae Schönherr, 1823**

**Subfamily Microcerinae Lacordaire, 1863**

**Subfamily Nanophyinae Gistel, 1848**

Tribe Corimaliini Alonso-Zarazaga, 1989

Tribe Nanophyini Gistel, 1848

**Family Dryophthoridae Schönherr, 1825**

**Subfamily Dryophthorinae Schönherr, 1825**

**Subfamily Cryptodermatinae Bovie, 1908**

**Subfamily Orthognathinae Lacordaire, 1865**

Tribe Orthognathini Lacordaire, 1865

Tribe Rhinostomini LeConte, 1874

**Subfamily Rhynchophorinae Schönherr, 1833**

Tribe Diocalandrini Zimmerman, 1993

Tribe Litosomini Lacordaire, 1865

Tribe Ommatolampini Lacordaire, 1865

Tribe Polytini Zimmerman, 1993

Tribe Rhynchophorini Schönherr, 1833

Tribe Sphenophorini Lacordaire, 1865

**Subfamily Stromboscerinae Lacordaire, 1865**

**Family Brachyceridae Billberg, 1820**

**Subfamily Brachycerinae Billberg, 1820**

Tribe Brachycerini Billberg, 1820

Tribe Byrsopini Germar, 1829

**Subfamily Cryptolarynginae Schalkwyk, 1966**

**Subfamily Erirhininae Schönherr, 1825**

Tribe Aonychini Zimmerman, 1993

Tribe Arthrostenini Reitter, 1913

†Tribe Cretuliini Legalov, 2009

Tribe Erirhinini Schönherr, 1825

Tribe Himasthlophallini Zherikhin, 1991

Tribe Stenopelmini LeConte, 1876

Tribe Tadiini Zimmerman, 1993

Tribe Tanysphyrini Gistel, 1848

**Subfamily Ocladiinae Lacordaire, 1865**

Tribe Desmidophorini Morimoto, 1962

Tribe Ocladiini Lacordaire, 1865

**Subfamily Raymondionyminae Reitter, 1913**

Tribe Myrtonymini Kuschel, 1990

Tribe Raymondionymini Reitter, 1913

**Family Curculionidae Latreille, 1802**

**Subfamily Curculioninae Latreille, 1802**

Tribe Acalyptini Thomson, 1859

Subtribe Acalyptina Thomson, 1859

Subtribe Derelomina Lacordaire, 1865

Subtribe Notolomina Franz, 2006

Subtribe Phyllotrogina Franz, 2006

Subtribe Staminodeina Franz, 2006

Tribe Acentrusini Alonso-Zarazaga, 2005

Tribe Ancylocnemidini Voss, 1962

Tribe Anthonomini Thomson, 1859

Tribe Camarotini Schönherr, 1833

Subtribe Camarotina Schönherr, 1833

Subtribe Prionomerina Lacordaire, 1863

Tribe Ceratopodini Lacordaire, 1863

Tribe Cionini Schönherr, 1825

Tribe Cranopoeini Kuschel, 2009

Tribe Cryptoplini Lacordaire, 1863

Tribe Curculionini Latreille, 1802

Subtribe Curculionina Latreille, 1802

Subtribe Pseudobalaninina Heller, 1925

Subtribe Timolina Heller, 1925

Tribe Diabathrariini Lacordaire, 1863

Tribe Ellescini Thomson, 1859

Subtribe Dorytomina Bedel, 1886

Subtribe Ellescina Thomson, 1859

Tribe Erodiscini Lacordaire, 1863

Tribe Eugnomini Lacordaire, 1863

Subtribe Eugnomina Lacordaire, 1863

Subtribe Meriphina Marshall, 1937

Tribe Gonipterini Lacordaire, 1863

Tribe Mecinini Gistel, 1848

Tribe Nerthopini Lacordaire, 1865

Tribe Otidocephalini Lacordaire, 1863

Tribe Piazorhinini Lacordaire, 1863

Tribe Prionobrachiini Hustache, 1938

Tribe Pyropini Lacordaire, 1865

Tribe Rhamphini Rafinesque, 1815

Subtribe Dinorhopalina Voss, 1936

Subtribe Ixalmina Voss, 1936

Subtribe Rhamphina Rafinesque, 1815

Subtribe Tachygonina Lacordaire, 1865

Tribe Smicronychini Seidlitz, 1891 nomen protectum

Tribe Sphaeriopoeini Kuschel, 2003

Tribe Storeini Lacordaire, 1863

Tribe Styphlini Jekel, 1861

Tribe Tychiini Gistel, 1848

Subtribe Demimaeina Voss, 1937

Subtribe Lignyodina Bedel, 1883

Subtribe Ochyromerina Voss, 1935

Subtribe Tychiina Gistel, 1848

Tribe Ulomascini Lacordaire, 1865

Tribe Viticiini Morimoto, 1983

**Subfamily Bagoinae Thomson, 1859** nomen protectum

**Subfamily Baridinae Schönherr, 1836**

Tribe Ambatini Lacordaire, 1863

Tribe Anopsilini Bondar, 1942

Tribe Apostasimerini Schönherr, 1844

Subtribe Apostasimerina Schönherr, 1844

Subtribe Madopterina Lacordaire, 1865

Subtribe Thaliabaridina Bondar, 1943

Subtribe Torcina Bondar, 1943

Subtribe Zygobaridina Pierce, 1907

Tribe Baridini Schönherr, 1836

Subtribe Baridina Schönherr, 1836

Subtribe Coelonertina Casey, 1922

Subtribe Coleomerina Casey, 1922

Subtribe Diorymerina Jekel, 1865

Subtribe Eurhinina Lacordaire, 1865

Tribe Madarini Jekel, 1865

Subtribe Barymerina Lacordaire, 1865

Subtribe Eutoxina Champion, 1908

Subtribe Leptoschoinina Lacordaire, 1865

Subtribe Madarina Jekel, 1865

Subtribe Tonesiina Alonso-Zarazaga and Lyal, 1999

Tribe Neosharpiini Hoffmann, 1956

Tribe Nertinini Voss, 1954

Tribe Optatini Champion, 1907

Tribe Pantotelini Lacordaire, 1865

Subtribe Cyrionychina Casey, 1922

Subtribe Pantotelina Lacordaire, 1865

Tribe Peridinetini Lacordaire, 1865

**Subfamily Ceutorhynchinae Gistel, 1848**

Tribe Ceutorhynchini Gistel, 1848

Tribe Cnemogonini Colonnelli, 1979

Tribe Egriini Pajni and Kohli, 1982

Tribe Hypohypurini Colonnelli, 2004

Tribe Hypurini Schultze, 1902

Tribe Lioxyonychini Colonnelli, 1984

Tribe Mecysmoderini Wagner, 1938

Tribe Mononychini LeConte, 1876

Tribe Phytobiini Gistel, 1848

Tribe Scleropterini Schultze, 1902

**Subfamily Conoderinae Schönherr, 1833**

Tribe Arachnopodini Lacordaire, 1865

Tribe Campyloscelini Schönherr, 1845

Subtribe Campyloscelina Schönherr, 1845

Subtribe Corynemerina Hustache, 1929

Subtribe Phaenomerina Faust, 1898

Tribe Conoderini Schönherr, 1833

Tribe Coryssomerini Thomson, 1859

Tribe Coryssopodini Lacordaire, 1865

Tribe Lechriopini Lacordaire, 1865

Tribe Lobotrachelini Lacordaire, 1865

Tribe Mecopini Lacordaire, 1865

Tribe Menemachini Lacordaire, 1865

Tribe Othippiini Morimoto, 1962

Tribe Peloropodini Hustache, 1932

Tribe Piazurini Lacordaire, 1865

Tribe Sphadasmini Lacordaire, 1865

Tribe Trichodocerini Champion, 1906

Tribe Zygopini Lacordaire, 1865

**Subfamily Cossoninae Schönherr, 1825**

Tribe Acamptini LeConte, 1876

Tribe Acanthinomerini Voss, 1972

Tribe Allomorphini Folwaczny, 1973

Tribe Aphyllurini Voss, 1955

Tribe Araucariini Kuschel, 1966

Tribe Choerorhinini Folwaczny, 1973

Tribe Cossonini Schönherr, 1825

Tribe Cryptommatini Voss, 1972

Tribe Dryotribini LeConte, 1876

Tribe Microxylobiini Voss, 1972

Tribe Nesiobiini Alonso-Zarazaga and Lyal, 1999

Tribe Neumatorini Folwaczny, 1973

Tribe Onychiini Chapuis, 1869

Tribe Onycholipini Wollaston, 1873

Tribe Pentarthrini Lacordaire, 1865

Tribe Proecini Voss, 1956

Tribe Pseudapotrepini Champion, 1909

Tribe Rhyncolini Gistel, 1848

Subtribe Phloeophagina Voss, 1955

Subtribe Pseudomimina Voss, 1939

Subtribe Rhyncolina Gistel, 1848

Tribe Tapiromimini Voss, 1972

**Subfamily Cryptorhynchinae Schönherr, 1825**

Tribe Aedemonini Faust, 1898

Tribe Camptorhinini Lacordaire, 1865

Tribe Cryptorhynchini Schönherr, 1825

Subtribe Cryptorhynchina Schönherr, 1825

Subtribe Mecistostylina Lacordaire, 1865

Subtribe Tylodina Lacordaire, 1865

Tribe Gasterocercini Zherikhin, 1991

Tribe Psepholacini Lacordaire, 1865

Tribe Sophrorhinini Lacordaire, 1865

Tribe Torneumatini Bedel, 1884

**Subfamily Cyclominae Schönherr, 1826**

Tribe Amycterini Waterhouse, 1854

Tribe Aterpini Lacordaire, 1863 nomen protectum

Subtribe Aterpina Lacordaire, 1863 nomen protectum

Subtribe Rhadinosomina Lacordaire, 1863

Tribe Cyclomini Schönherr, 1826

Tribe Dichotrachelini Hoffmann, 1957

Tribe Hipporhinini Lacordaire, 1863

Tribe Listroderini LeConte, 1876

Tribe Notiomimetini Wollaston, 1873

Tribe Rhythirrinini Lacordaire, 1863

**Subfamily Entiminae Schönherr, 1823**

Tribe Agraphini Horn, 1876

Tribe Alophini LeConte, 1874

Tribe Anomophthalmini Morrone, 1998

Tribe Anypotactini Champion, 1911

Tribe Blosyrini Lacordaire, 1863

Tribe Brachyderini Schönherr, 1826

Tribe Celeuthetini Lacordaire, 1863

Subtribe Celeuthetina Lacordaire, 1863

Subtribe Isopterina Morimoto and Kojima, 2001

Tribe Cneorhinini Lacordaire, 1863

Tribe Cratopodini Hustache, 1919

Tribe Cylydrorhinini Lacordaire, 1863

Tribe Cyphicerini Lacordaire, 1863

Subtribe Acanthotrachelina Marshall, 1944

Subtribe Cyphicerina Lacordaire, 1863

Subtribe Mylacorrhinina Reitter, 1913

Subtribe Myllocerina Pierce, 1913

Subtribe Phytoscaphina Lacordaire, 1863

Tribe Ectemnorhinini Lacordaire, 1863

Tribe Elytrurini Marshall, 1956

Tribe Embrithini Marshall, 1942

Tribe Entimini Schönherr, 1823

Tribe Episomini Lacordaire, 1863

Tribe Eudiagogini LeConte, 1874

Tribe Eupholini Günther, 1943

Tribe Eustylini Lacordaire, 1863

Tribe Geonemini Gistel, 1848

Tribe Holcorhinini Desbrochers des Loges, 1898

Tribe Hormorini Horn, 1876

Tribe Laparocerini Lacordaire, 1863

Tribe Leptostethini Lacordaire, 1863

Tribe Lordopini Schönherr, 1823

Tribe Mesostylini Reitter, 1913

Tribe Myorhinini Marseul, 1863

Tribe Nastini Reitter, 1913

Tribe Naupactini Gistel, 1848 nomen protectum

Tribe Nothognathini Marshall, 1916

Tribe Omiini Shuckard, 1839

Tribe Oosomini Lacordaire, 1863

Tribe Ophryastini Lacordaire, 1863

Tribe Ophtalmorrhynchini Hoffmann, 1965

Tribe Otiorhynchini Schönherr, 1826

Tribe Ottistirini Heller, 1925

Tribe Pachyrhynchini Schönherr, 1826

Tribe Peritelini Lacordaire, 1863

Tribe Phyllobiini Schönherr, 1826

Tribe Polycatini Marshall, 1956

Tribe Polydrusini Schönherr, 1823

Tribe Premnotrypini Kuschel, 1956

†Tribe Pristorhynchini Heer, 1847

Tribe Prypnini Lacordaire, 1863

Tribe Psallidiini Lacordaire, 1863

Tribe Rhyncogonini Sharp, 1919

Tribe Sciaphilini Sharp, 1891

Tribe Sitonini Gistel, 1848

Tribe Tanymecini Lacordaire, 1863

Subtribe Piazomiina Reitter, 1913

Subtribe Tainophthalmina Desbrochers des Loges, 1873

Subtribe Tanymecina Lacordaire, 1863

Tribe Tanyrhynchini Schönherr, 1826

Tribe Thecesternini Lacordaire, 1863

Tribe Trachyphloeini Gistel, 1848

Subtribe Trachyphilina Voss, 1948

Subtribe Trachyphloeina Gistel, 1848

Tribe Tropiphorini Marseul, 1863

Tribe Typhlorhinini Kuschel, 1954

**Subfamily Hyperinae Marseul, 1863 (1848)**

Tribe Cepurini Capiomont, 1867

Tribe Hyperini Marseul, 1863 (1848)

**Subfamily Lixinae Schönherr, 1823**

Tribe Cleonini Schönherr, 1826 nomen protectum

Tribe Lixini Schönherr, 1823

Tribe Rhinocyllini Lacordaire, 1863

**Subfamily Mesoptiliinae Lacordaire, 1863**

Tribe Carciliini Pierce, 1916

Tribe Laemosaccini Lacordaire, 1865

Tribe Magdalidini Pascoe, 1870 nomen protectum

Tribe Mesoptiliini Lacordaire, 1863

**Subfamily Molytinae Schönherr, 1823**

Tribe Anoplini Bedel, 1883

Tribe Amalactini Lacordaire, 1863

Tribe Aminyopini Voss, 1956

Tribe Amorphocerini Voss, 1939

Tribe Anchonini Imhoff, 1856

Tribe Brachyceropseini Aurivillius, 1926

Tribe Cholini Schönherr, 1825

Subtribe Cholina Schönherr, 1825

Subtribe Cholomina Vaurie, 1974

Subtribe Rhinastina Vaurie, 1973

Tribe Cleogonini Gistel, 1848

Tribe Conotrachelini Jekel, 1865

Tribe Cycloterini Lacordaire, 1863

Subtribe Cycloterina Lacordaire, 1863

Subtribe Thrombosternina Voss, 1965

Tribe Dinomorphini Lacordaire, 1863

Tribe Emphyastini Lacordaire, 1863

Tribe Euderini Lacordaire, 1865

Tribe Galloisiini Morimoto, 1962

Tribe Guioperini Lacordaire, 1865

Tribe Hylobiini Kirby, 1837

Subtribe Epistrophina Marshall, 1932

Subtribe Hylobiina Kirby, 1837

Tribe Ithyporini Lacordaire, 1865

Subtribe Colobodina Voss, 1958

Subtribe Ithyporina Lacordaire, 1865

Subtribe Sclerocardiina Lacordaire, 1865

Tribe Itini Reitter, 1913

Tribe Juanorhinini Aurivillius, 1931

Tribe Lepyrini Kirby, 1837

Tribe Lithinini Lacordaire, 1863

Subtribe Lithinina Lacordaire, 1863

Subtribe Rhytidophloeina Voss, 1963

Tribe Lymantini Lacordaire, 1865

Tribe Mecysolobini Reitter, 1913

Tribe Metatygini Pascoe, 1888

Tribe Molytini Schönherr, 1823

Subtribe Leiosomatina Reitter, 1913

Subtribe Molytina Schönherr, 1823

Subtribe Plinthina Lacordaire, 1863

Subtribe Typoderina Voss, 1965

Tribe Nettarhinini Lacordaire, 1865

Tribe Pacholenini Lacordaire, 1863

Tribe Paipalesomini Marshall, 1932

Tribe Petalochilini Lacordaire, 1863

Tribe Phoenicobatini Champion, 1914

Tribe Phrynixini Kuschel, 1964

Tribe Pissodini Gistel, 1848

Subtribe Cotasteromimina Morimoto, 1962

Subtribe Orthorhinina Jekel, 1865

Subtribe Pissodina Gistel, 1848

Tribe Sternechini Lacordaire, 1863

Tribe Styanacini Chûjô and Voss, 1960

Tribe Trachodini Gistel, 1848

Tribe Trigonocolini Lacordaire, 1863

Tribe Trypetidini Lacordaire, 1865

**Subfamily Orobitidinae Thomson, 1859**

**Subfamily Xiphaspidinae Marshall, 1920**

**Subfamily Scolytinae Latreille, 1804**

Tribe Amphiscolytini Mandelshtam and Beaver, 2003

Tribe Bothrosternini Blandford, 1896

Tribe Cactopinini Chamberlin, 1939

Tribe Carphodicticini Wood, 1971

Tribe Coptonotini Chapuis, 1869

Tribe Corthylini LeConte, 1876

Subtribe Corthylina LeConte, 1876

Subtribe Pityophthorina Eichhoff, 1878

Tribe Cryphalini Lindemann, 1877

Tribe Crypturgini LeConte, 1876

†Tribe Cylindrobrotini Kirejtshuk, Azar, Beaver, Mandelshtam and Nel, 2009

Tribe Diamerini Hagedorn, 1909

Tribe Dryocoetini Lindemann, 1877

Tribe Hexacolini Eichhoff, 1878

Tribe Hylastini LeConte, 1876

Tribe Hylesinini Erichson, 1836

Tribe Hylurgini Gistel, 1848

Tribe Hyorrhynchini Hopkins, 1915

Tribe Hypoborini Nüsslin, 1911

Tribe Ipini Bedel, 1888

Tribe Micracidini LeConte, 1876

Tribe Phloeosinini Nüsslin, 1912

Tribe Phloeotribini Chapuis, 1869

Tribe Phrixosomatini Wood, 1978

Tribe Polygraphini Chapuis, 1869

Tribe Premnobiini Browne, 1962

Tribe Scolytini Latreille, 1804

Tribe Scolytoplatypodini Blandford, 1893

Tribe Xyleborini LeConte, 1876

Tribe Xyloctonini Eichhoff, 1878

Tribe Xyloterini LeConte, 1876

**Subfamily Platypodinae Shuckard, 1839**

Tribe Mecopelmini Thompson, 1992

Tribe Platypodini Shuckard, 1839

Tribe Schedlariini Wood and Bright, 1992

Tribe Tesserocerini Strohmeyer, 1914

Subtribe Diapodina Strohmeyer, 1914

Subtribe Tesserocerina Strohmeyer, 1914

## Catalogue of Coleoptera family-group names

### Order COLEOPTERA

### †Suborder PROTOCOLEOPTERA

### †Superfamily Tshekardocoleoidea

Rohdendorf, 1944

Tshekardocoleidae Rohdendorf, 1944: 252 [stem: *Tshekardocole-*]. Type genus: *Tshekardocoleus* Rohdendorf, 1944.

### †Family Tshekardocoleidae

Rohdendorf, 1944

Tshekardocoleidae Rohdendorf, 1944: 252 [stem: *Tshekardocole-*]. Type genus: *Tshekardocoleus* Rohdendorf, 1944.
Uralocoleidae Zalesskiy, 1947: 857 [stem: *Uralocole-*]. Type genus: *Uralocoleus* Zalessky, 1947.

### †Family Labradorocoleidae

Ponomarenko, 1969

Labradorocoleidae Ponomarenko, 1969b: 307 [stem: *Labradorocole-*]. Type genus: *Labradorocoleus* Ponomarenko, 1969.

### †Family Oborocoleidae

Kukalová, 1969

Oborocoleidae Kukalová, 1969: 155 [stem: *Oborocole-*]. Type genus: *Oborocoleus* Kukalová, 1969.

### †Superfamily Permocupedoidea

Martynov, 1933

Permocupidae Martynov, 1933: 85 [stem: *Permocuped-*]. Type genus: *Permocupes* Martynov, 1933.

### †Family Permocupedidae

Martynov, 1933

Permocupidae Martynov, 1933: 85 [stem: *Permocuped-*]. Type genus: *Permocupes* Martynov, 1933. Comment: incorrect original stem formation, not in prevailing usage.
Kaltanocoleidae Rohdendorf, 1961: 397 [stem: *Kaltanocole-*]. Type genus: *Kaltanocoleus* Rohdendorf, 1961.

### †Family Taldycupedidae

Rohdendorf, 1961

Taldycupidae Rohdendorf, 1961: 412 [stem: *Taldycuped-*]. Type genus: *Taldycupes* Rohdendorf, 1961. Comment: usage of Trachycupidae by Fujiyama (1973: 375) was in error for Taldycupidae (Ponomarenko pers. comm. August 2009); incorrect original stem formation, not in prevailing usage.

### †Superfamily Permosynoidea

Tillyard, 1924

Permosynidae Tillyard, 1924: 431 [stem: *Permosyn-*]. Type genus: *Permosyne* Tillyard, 1924.

### †Family Ademosynidae

Ponomarenko, 1968

Ademosynidae Ponomarenko, 1968: 128 [stem: *Ademosyn-*]. Type genus: *Ademosyne* Handlirsch, 1906.

### †Family Permosynidae

Tillyard, 1924

Permosynidae Tillyard, 1924: 431 [stem: *Permosyn-*]. Type genus: *Permosyne* Tillyard, 1924.

### Suborder ARCHOSTEMATA

### Family Crowsoniellidae

Iablokoff-Khnzorian, 1983

Crowsoniellidae Iablokoff-Khnzorian, 1983: 65 [stem: *Crowsoniell-*]. Type genus: *Crowsoniella* Pace, 1975.

### Family Cupedidae

Laporte, 1836

Cupesidae Laporte, 1836: 56 [stem: *Cuped-*]. Type genus: *Cupes* Fabricius, 1801.

### Subfamily Priacminae

Crowson, 1962

Priacmini Crowson, 1962: 152 [stem: *Priacm-*]. Type genus: *Priacma* J. L. LeConte, 1874.

### †Subfamily Mesocupedinae

Ponomarenko, 1969

Mesocupedini Ponomarenko, 1969a: 105 [stem: *Mesocuped-*]. Type genus: *Mesocupes* Martynov, 1926.

### Subfamily Cupedinae

Laporte, 1836

Cupesidae Laporte, 1836: 56 [stem: *Cuped-*]. Type genus: *Cupes* Fabricius, 1802. Comment: incorrect original stem formation, not in prevailing usage.

### Family Micromalthidae

Barber, 1913

Micromalthidae Barber, 1913: 185 [stem: *Micromalth-*]. Type genus: *Micromalthus* J. L. LeConte, 1878.

### Family Ommatidae

Sharp and Muir, 1912

Ommadidae Sharp and Muir, 1912: 521 [stem: *Ommat-*]. Type genus: *Omma* Newman, 1839.

### †Subfamily Brochocoleinae

Hong, 1982

Brochocoleidae Hong, 1982: 100 [stem: *Brochocole-*]. Type genus: *Brochocoleus* Hong, 1982.

### Subfamily Tetraphalerinae

Crowson, 1962

Tetraphalerini Crowson, 1962: 152 [stem: *Tetraphaler-*]. Type genus: *Tetraphalerus* C. O. Waterhouse, 1901.

### Subfamily Ommatinae

Sharp and Muir, 1912

Ommadidae Sharp and Muir, 1912: 521 [stem: *Ommat-*]. Type genus: *Omma* Newman, 1839.

### †Tribe Lithocupedini

Ponomarenko, 1969

Lithocupedini Ponomarenko, 1969a: 82 [stem: *Lithocuped-*]. Type genus: *Lithocupes* Ponomarenko, 1966.

### †Tribe Notocupedini

Ponomarenko, 1966

Notocupedini Ponomarenko, 1966: 60 [stem: *Notocup-*]. Type genus: *Notocupes* Ponomarenko, 1966.

### Tribe Ommatini

Sharp and Muir, 1912

Ommadidae Sharp and Muir, 1912: 521 [stem: *Ommat*-]. Type genus: *Omma* Newman, 1839. Comment: incorrect original stem formation, not in prevailing usage.

### Family Jurodidae

Ponomarenko, 1985

Jurodidae Ponomarenko, 1985: 53 [stem: *Jurod-*]. Type genus: *Jurodes* Ponomarenko, 1985.
Sikhotealiniidae Lafer, 1996: 390 [stem: *Sikhotealini-*]. Type genus: *Sikhotealinia* Lafer, 1996.

### †Family Triadocupedidae

Ponomarenko, 1966

Triadocupedinae Ponomarenko, 1966: 48 [stem: *Triadocuped-*]. Type genus: *Triadocupes* Ponomarenko, 1966.

### †Family Magnocoleidae

Hong, 1998

Magnocoleidae Hong, 1998: 41 [stem: *Magnocole-*]. Type genus: *Magnocoleus* Hong, 1998.

### †Family Obrieniidae

Zherikhin and Gratshev, 1994

Obrieniidae Zherikhin and Gratshev, 1994: 51 [stem: *Obrieni-*]. Type genus: *Obrienia* Zherikhin and Gratshev, 1994. Comment: precedence (Obrieniidae Zherikhin and Gratshev, 1994 vs Kararhynchidae Zherikhin and Gratshev, 1994) given to taxon originally proposed at the higher rank (Art. 24.1).

### †Subfamily Kararhynchinae

Zherikhin and Gratshev, 1994

Kararhynchinae Zherikhin and Gratshev, 1994: 58 [stem: *Kararhynch-*]. Type genus: *Kararhynchus* Zherikhin and Gratshev, 1994.

### †Tribe Kararhynchini

Zherikhin and Gratshev, 1994

Kararhynchinae Zherikhin and Gratshev, 1994: 58 [stem: *Kararhynch-*]. Type genus: *Kararhynchus* Zherikhin and Gratshev, 1994.

### †Tribe Kenderlykaini

Legalov, 2009

Kenderlykanini Legalov, 2009c: 285 [stem: *Kenderlyka*-]. Type genus: *Kenderlyka* Zherikhin and Gratshev, 1994. Comment: incorrect original stem formation, not in prevailing usage.

### †Subfamily Obrieniinae

Zherikhin and Gratshev, 1994

Obrieniidae Zherikhin and Gratshev, 1994: 51 [stem: *Obrieni-*]. Type genus: *Obrienia* Zherikhin and Gratshev, 1994.

### Suborder MYXOPHAGA

### †Superfamily Asiocoleoidea

Rohdendorf, 1961

Asiocoleidae Rohdendorf, 1961: 396 [stem: *Asiocole-*]. Type genus: *Asiocoleus* Rohdendorf, 1961.

### †Family Asiocoleidae

Rohdendorf, 1961

Asiocoleidae Rohdendorf, 1961: 396 [stem: *Asiocole-*]. Type genus: *Asiocoleus* Rohdendorf, 1961.

### †Family Tricoleidae

Ponomarenko, 1969

Tricoleidae Ponomarenko, 1969a: 138 [stem: *Tricole-*]. Type genus: *Tricoleus* Ponomarenko, 1969.

### †Superfamily Rhombocoleoidea

Rohdendorf, 1961

Rhombocoleidae Rohdendorf, 1961: 432 [stem: *Rhombocole-*]. Type genus: *Rhombocoleus* Rohdendorf, 1961.

### †Family Rhombocoleidae

Rohdendorf, 1961

Rhombocoleidae Rohdendorf, 1961: 432 [stem: *Rhombocole-*]. Type genus: *Rhombocoleus* Rohdendorf, 1961.

### †Superfamily Schizophoroidea

Ponomarenko, 1968

Schizophoridae Ponomarenko, 1968: 130 [stem: *Schizophor-*]. Type genus: *Schizophorus* Ponomarenko, 1968. Comment: usage of this name conserved over Schizocoleoidea Rohdendorf, 1961 (Art. 35.5).

### †Family Schizophoridae

Ponomarenko, 1968

Schizophoridae Ponomarenko, 1968: 130 [stem: *Schizophor-*]. Type genus: *Schizophorus* Ponomarenko, 1968.

### †Family Catiniidae

Ponomarenko, 1968

Catiniidae Ponomarenko, 1968: 137 [stem: *Catini-*]. Type genus: *Catinius* Ponomarenko, 1968.

### †Family Schizocoleidae

Rohdendorf, 1961

Curculiopsidae Martynov, 1937: 39 [stem: *Curculiopse-*]. Type genus: *Curculiopsis* Martynov, 1937 [preoccupied genus name, not *Curculiopsis* Handlirsch, 1907 [fossil Curculionoidea]; syn. of *Aenigmocoleus* Rohdendorf, 1961 or *Rossocoleus* Rohdendorf, 1961]. Comment: permanently invalid (Art. 39): based on preoccupied type genus; incorrect original stem formation, not in prevailing usage.
Schizocoleidae Rohdendorf, 1961: 438 [stem: *Schizocole-*]. Type genus: *Schizocoleus* Rohdendorf, 1961.

### Superfamily Lepiceroidea

Hinton, 1936 (1882)

Lepiceridae Hinton, 1936: 473 [stem: *Lepicer-*]. Type genus: *Lepicerus* Motschulsky, 1855. Comment: use of family-group name conserved over Cyathoceroidea Sharp, 1882 (Art. 40.2) (see Lawrence and Newton 1995: 804).

### Family Lepiceridae

Hinton, 1936 (1882)

Cyathoceridae Sharp, 1882: 141 [stem: *Cyathocer-*]. Type genus: *Cyathocerus* Sharp, 1882 [syn. of *Lepicerus* Motschulsky, 1855]. Comment: use of younger name Lepiceridae Hinton, 1936 conserved over this name (Art. 40.2) (see Lawrence and Newton 1995: 804).
Lepiceridae Hinton, 1936: 473 [stem: *Lepicer-*]. Type genus: *Lepicerus* Motschulsky, 1855. Comment: use of family-group name conserved over Cyathoceridae Sharp, 1882 (Art. 40.2) (see Lawrence and Newton 1995: 804).
†Haplochelidae Kirejtshuk and Poinar, 2006: 156 [stem: *Haplochel-*]. Type genus: *Haplochelus* Kirejtshuk and Poinar, 2006. Comment: synonymy with Lepiceridae by Ge et al. (2010: 336).

### Superfamily Sphaeriusoidea

Erichson, 1845

Sphaerina Erichson, 1845: 38 [stem: *Sphaerius-*]. Type genus: *Sphaerius* Waltl, 1838 [originally placed on the Official Index of Rejected and Invalid Generic Names in Zoology by the Commission (ICZN 1985d) but later placed on the Official List of Generic Names in Zoology (ICZN 2000)]. Comment: correct stem determined to be *Sphaerius*- and Sphaeriusidae Erichson, 1845 placed on the Official List of Family-Group Names in Zoology (ICZN 2000).

### Family Torridincolidae

Steffan, 1964

Torridincolidae Steffan, 1964: 199 [stem: *Torridincol-*]. Type genus: *Torridincola* Steffan, 1964.

### Subfamily Torridincolinae

Steffan, 1964

Torridincolidae Steffan, 1964: 199 [stem: *Torridincol-*]. Type genus: *Torridincola* Steffan, 1964.
Ptyopterinae M. Abdullah, 1974: 961 [stem: *Ptyopteryg-*]. Type genus: *Ptyopteryx* H. Reichardt and C. Costa, 1967 [preoccupied genus name, not *Ptyopteryx* Kolenati, 1848 [Trichoptera]; syn. of *Iapir* Py-Daniel et al., 1993]. Comment: permanently invalid (Art. 39): based on preoccupied type genus; incorrect original stem formation, not in prevailing usage.

### Subfamily Deleveinae

Endrödy-Younga, 1997

Deleveinae Endrödy-Younga, 1997: 317 [stem: *Deleve-*]. Type genus: *Delevea* H. Reichardt, 1976.

### Family Hydroscaphidae

LeConte, 1874

Hydroscaphidae J. L. LeConte, 1874a: 45 [stem: *Hydroscaph-*]. Type genus: *Hydroscapha* J. L. LeConte, 1874.

### Family Sphaeriusidae

Erichson, 1845

Sphaerina Erichson, 1845: 38 [stem: *Sphaerius-*]. Type genus: *Sphaerius* Waltl, 1838 [originally placed on the Official Index of Rejected and Invalid Generic Names in Zoology by the Commission (ICZN 1985d) but later placed on the Official List of Generic Names in Zoology (ICZN 2000)]. Comment: family-group name and its type genus were rejected by the Commission (ICZN 1985d) but rescinded afterwards (ICZN 2000); family-group name placed on the Official List of Family-Group Names in Zoology and correct stem ruled to be *Sphaerius*- (ICZN 2000).
Microsporidae Crotch, 1873a: 78 [stem: *Microspor-*]. Type genus: *Microsporus* Kolenati, 1846 [placed on the Official List of Generic Names in Zoology (ICZN 1985d)]. Comment: name originally placed on the Official List of Family-Group Names in Zoology as “Microsporidae H. Reichardt, 1976” (ICZN 1985d) but author and year subsequently corrected to Crotch, 1873 (ICZN 2000).

### Suborder ADEPHAGA

### †Family Tritarsidae

Hong, 2002

Tritarsusidae Hong, 2002: 102 [stem: *Tritars-*]. Type genus: *Tritarsus* Hong, 2002. Comment: incorrect original stem formation, not in prevailing usage.

### Family Gyrinidae

Latreille, 1810

Gyrinites Latreille, 1810: 141 [stem: *Gyrin-*]. Type genus: *Gyrinus* Geoffroy, 1762 [placed on the Official List of Generic Names in Zoology (ICZN 1994a)].

### Subfamily Spanglerogyrinae

Folkerts, 1979

Spanglerogyrinae Folkerts, 1979: 7 [stem: *Spanglerogyr-*]. Type genus: *Spanglerogyrus* Folkerts, 1979.

### Subfamily Gyrininae

Latreille, 1810

Gyrinites Latreille, 1810: 141 [stem: *Gyrin-*]. Type genus: *Gyrinus* Geoffroy, 1762 [placed on the Official List of Generic Names in Zoology (ICZN 1994a)].

### Tribe Enhydrini

Régimbart, 1882

Enhydrini Régimbart, 1882: 392 [stem: *Enhydr-*]. Type genus: *Enhydrus* Laporte, 1834 [placed on the Official List of Generic Names in Zoology (ICZN 1964)]. Comment: usage of this name conserved over Dineutini Desmarest, 1851 (Art. 35.5).

### Subtribe Dineutina

Desmarest, 1851

Dineutides Desmarest, 1851: 223 [stem: *Dineut-*]. Type genus: *Dineutes* W. S. MacLeay, 1825.

### Subtribe Enhydrina

Régimbart, 1882

Enhydrini Régimbart, 1882: 392 [stem: *Enhydr-*]. Type genus: *Enhydrus* Laporte, 1834 [placed on the Official List of Generic Names in Zoology (ICZN 1964)]. Comment: junior homonym of Enhydrini Gray, 1825 (type genus *Enhydra* Fleming, 1822) in Mammalia; an application was recently submitted to the Commission by Özdikmen and Darilmaz (2010; see Appendix 6) to remove the homonymy (Art. 55.3.1).

### Tribe Gyrinini

Latreille, 1810

Gyrinites Latreille, 1810: 141 [stem: *Gyrin-*]. Type genus: *Gyrinus* Geoffroy, 1762 [placed on the Official List of Generic Names in Zoology (ICZN 1994a)].

### Subtribe Gyrinina

Latreille, 1810

Gyrinites Latreille, 1810: 141 [stem: *Gyrin-*]. Type genus: *Gyrinus* Geoffroy, 1762 [placed on the Official List of Generic Names in Zoology (ICZN 1994a)].

### Subtribe Heterogyrina

Brinck, 1956

Heterogyrini P. Brinck, 1956: 37, in key [stem: *Heterogyr-*]. Type genus: *Heterogyrus* Legros, 1953.

### Tribe Orectochilini

Régimbart, 1882

Orectochilini Régimbart, 1882: 391 [stem: *Orectochil-*]. Type genus: *Orectochilus* Dejean, 1833.

### Family Trachypachidae

Thomson, 1857

Trachypachini C. G. Thomson, 1857: 5 [stem: *Trachypach-*]. Type genus: *Trachypachus* Motschulsky, 1844.

### †Subfamily Eodromeinae

Ponomarenko, 1977

Eodromeinae Ponomarenko, 1977: 46 [stem: *Eodrome-*]. Type genus: *Eodromeus* Ponomarenko, 1977. Comment: the type genus originally included four species: *antiquus*, *stenalis*, *dissectus* and *major*; Ponomarenko (1977: 66) designated “*fasciatus*” as the type species of the genus which was a *lapsuscalami* for *dissectus* (see Carpenter 1992: 291); this genus is considered here as being available in Ponomarenko (1977: 66) and not Ponomarenko (in Carpenter 1992: 291); this action enables us to treat the family-group name Eodromeinae as available from the original description in Ponomarenko (1977: 46).
Leptopodocoleidae Hong, 1982: 118 [stem: *Leptopodocole-*]. Type genus: *Leptopodocoleus* Hong, 1982.

### Subfamily Trachypachinae

Thomson, 1857

Trachypachini C. G. Thomson, 1857: 5 [stem: *Trachypach-*]. Type genus: *Trachypachus* Motschulsky, 1844.
*Systolosomini Erwin, 1985: 467 [stem: *Systolosomat-*]. Type genus: *Systolosoma* Solier, 1849. Comment: unavailable family-group name, proposed after 1930 without description or bibliographic reference to such a description (Art. 13.1); incorrect original stem formation, not in prevailing usage.
Systolosomini Erwin, 1991: 4, in key [stem: *Systolosomat-*]. Type genus: *Systolosoma* Solier, 1849. Comment: incorrect original stem formation, not in prevailing usage.

### Family Rhysodidae

Laporte, 1840

Rhysodites Laporte, 1840a: 291 [stem: *Rhysod-*]. Type genus: *Rhysodes* Germar, 1822.

### Tribe Leoglymmiini

Bell and Bell, 1978

Leoglymmiina R. T. Bell and J. R. Bell, 1978: 53 [stem: *Leoglymmi-*]. Type genus: *Leoglymmius* Bell and Bell, 1978.

### Tribe Dhysorini

Bell and Bell, 1978

Dhysorina R. T. Bell and J. R. Bell, 1978: 53 [stem: *Dhysor-*]. Type genus: *Dhysores* Grouvelle, 1903.

### Tribe Medisorini

Bell and Bell, 1987

Medisorina R. T. Bell and J. R. Bell, 1987: 287 [stem: *Medisor-*]. Type genus: *Medisores* Bell and Bell, 1987.

### Tribe Rhysodini

Laporte, 1840

Rhysodites Laporte, 1840a: 291 [stem: *Rhysod-*]. Type genus: *Rhysodes* Germar, 1822. Comment: original vernacular name available (Art. 11.7.2): first used in latinized form by Agassiz (1846b: 326, as Rhyssodeoidae [incorrect stem formation]), generally accepted as in R. T. Bell (2003: 78, as Rhysodidae).

### Tribe Clinidiini

Bell and Bell, 1978

Clinidiina R. T. Bell and J. R. Bell, 1978: 59 [stem: *Clinidi-*]. Type genus: *Clinidium* Kirby, 1830.

### Tribe Omoglymmiini

Bell and Bell, 1978

Omoglymmiina R. T. Bell and J. R. Bell, 1978: 66 [stem: *Omoglymmi-*]. Type genus: *Omoglymmius* Ganglbauer, 1891.

### Tribe Sloanoglymmiini

Bell and Bell, 1991

Sloanoglymmiina R. T. Bell and J. R. Bell, 1991: 183 [stem: *Sloanoglymmi-*]. Type genus: *Sloanoglymmius* Bell and Bell, 1991.

### Family Carabidae

Latreille, 1802

Carabici Latreille, 1802: 80 [stem: *Carab-*]. Type genus: *Carabus* Linnaeus, 1758 [placed on the Official List of Generic Names in Zoology (ICZN 1950)]. Comment: First Reviser (Carabidae Latreille, 1802 vs Cicindelidae Latreille, 1802 vs Elaphridae Latreille, 1802) not determined, current usage maintained.

### †Subfamily Protorabinae

Ponomarenko, 1977

Protorabinae Ponomarenko, 1977: 71 [stem: *Protorab-*]. Type genus: *Protorabus* Ponomarenko, 1977.

### †Subfamily Conjunctiinae

Ponomarenko, 1977

Conjunctiini Ponomarenko, 1977: 85 [stem: *Conjuncti-*]. Type genus: *Conjunctia* Ponomarenko, 1977.

### Subfamily Nebriinae

Laporte, 1834

Nebriidae Laporte, 1834b: 90 [stem: *Nebri-*]. Type genus: *Nebria* Latreille, 1802.

### Tribe Nebriini

Laporte, 1834

Nebriidae Laporte, 1834b: 90 [stem: *Nebri-*]. Type genus: *Nebria* Latreille, 1802.

### Tribe Notiokasiini

Kavanaugh and Nègre, 1983

*Notiokasiini Erwin, 1979: 578 [stem: *Notiokasi-*]. Type genus: *Notiokasis* Kavanaugh and Nègre, 1983. Comment: family-group name unavailable (Art. 11.7.1.1): not based on an available genus name at the time.
Notiokasiini Kavanaugh and Nègre, 1983: 551 [stem: *Notiokasi-*]. Type genus: *Notiokasis* Kavanaugh and Nègre, 1983.

### Tribe Notiophilini

Motschulsky, 1850

*Notiophiles Motschulsky, 1849: 54 [stem: *Notiophil-*]. Type genus: *Notiophilus* Duméril, 1805. Comment: original vernacular name unavailable (Art. 11.7.2): subsequently used in latinized form but not generally attributed to Motschulsky (1849).
Notiophili Motschulsky, 1850: 16 [stem: *Notiophil-*]. Type genus: *Notiophilus* Duméril, 1805.

### Tribe Opisthiini

Dupuis, 1912

Opisthiinae Dupuis, 1912: 1 [stem: *Opisthi-*]. Type genus: *Opisthius* Kirby, 1837.

### Tribe Pelophilini

Kavanaugh, 1996

Pelophilini Kavanaugh, 1996: 35 [stem: *Pelophil-*]. Type genus: *Pelophila* Dejean, 1821.

### Subfamily Cicindinae

Csiki, 1927

Cicindini Csiki, 1927: 425 [stem: *Cicind-*]. Type genus: *Cicindis* Bruch, 1908.

### Subfamily Cicindelinae

Latreille, 1802

Cicindeletae Latreille, 1802: 77 [stem: *Cicindel-*]. Type genus: *Cicindela* Linné, 1758.

### Tribe Amblycheilini

Csiki, 1903

Amblychilinae Csiki, 1903: 124 [stem: *Amblycheil-*]. Type genus: *Amblycheila* Say, 1830 [as *Amblychila*, unjustified emendation of type genus name by Agassiz (1846b), not in prevailing usage]. Comment: incorrect original stem formation, not in prevailing usage.
Omites W. Horn, 1907: 466 [stem: *Om-*]. Type genus: *Omus* Eschscholtz, 1829.

### Tribe Cicindelini

Latreille, 1802

Cicindeletae Latreille, 1802: 77 [stem: *Cicindel-*]. Type genus: *Cicindela* Linnaeus, 1758.

### Subtribe Apteroessina

Rivalier, 1971

Apteroessina Rivalier, 1971: 143 [stem: *Apteroess-*]. Type genus: *Apteroessa* Hope, 1838.

### Subtribe Cicindelina

Latreille, 1802

Cicindeletae Latreille, 1802: 77 [stem: *Cicindel-*]. Type genus: *Cicindela* Linnaeus, 1758.

### Subtribe Dromicina

Thomson, 1859

Dromicitae J. Thomson, 1859: 89 [stem: *Dromic-*]. Type genus: *Dromica* Dejean, 1826.
Caledonicini W. Horn, 1893: 324 [stem: *Caledonic-*]. Type genus: *Caledonica* Chaudoir, 1861.
Odontochilini W. Horn, 1899: 41 [stem: *Odontocheil-*]. Type genus: *Odontocheila* Laporte, 1834 [as *Odontochila*, unjustified emendation of type genus name by Agassiz (1846b), not in prevailing usage]. Comment: incorrect original stem formation, not in prevailing usage.
Prepusini W. Horn, 1899: 44 [stem: *Prepus-*]. Type genus: *Prepusa* Chaudoir, 1850 [syn. of *Eulampra* Chaudoir, 1848].
Euryodini W. Horn, 1899: 37 [stem: *Euryod-*]. Type genus: *Euryoda* Lacordaire, 1842 [syn. of *Prothyma* Hope, 1838].
Prothymini W. Horn, 1906: 86 [stem: *Prothym-*]. Type genus: *Prothyma* Hope, 1838.

### Subtribe Iresiina

Rivalier, 1971

Euprosopini W. Horn, 1893: 324 [stem: *Euprosop-*]. Type genus: *Euprosopus* Dejean, 1825.
Eucalliini W. Horn, 1893: 324 [stem: *Eucalli-*]. Type genus: *Eucallia* Guérin-Méneville, 1844 [syn. of *Callidema* Guérin-Méneville, 1843].
Distypsiderini W. Horn, 1893: 324 [stem: *Distipsider-*]. Type genus: *Distipsidera* Westwood, 1837 [as *Distypsidera*, unjustified emendation of type genus name by Gemminger and Harold (1868a: 32), not in prevailing usage]. Comment: incorrect original stem formation, not in prevailing usage.
Iresina Rivalier, 1971: 138 [stem: *Iresi-*]. Type genus: *Iresia* Dejean, 1829. Comment: although this is not the oldest name for the subtribe, we recommend that an application be sent to the Commission in order to conserve usage of Iresiina Rivalier, 1971 over the three older names proposed by W. Horn in 1893 which have not been used as valid recently to our knowledge; incorrect original stem formation, not in prevailing usage.

### Subtribe Theratina

Horn, 1893

Theratidae W. Horn, 1893: 324 [stem: *Therat-*]. Type genus: *Therates* Latreille, 1816.

### Tribe Collyridini

Brullé, 1834

Collyriens Brullé, 1834: 96 [stem: *Collyrid-*]. Type genus: *Collyris* Fabricius, 1801.

### Subtribe Collyridina

Brullé, 1834

Collyriens Brullé, 1834: 96 [stem: *Collyrid-*]. Type genus: *Collyris* Fabricius, 1801. Comment: original vernacular name available (Art. 11.7.2): first used in latinized form by Hope (1838a: 30, as Collyridae [incorrect stem formation]), generally accepted as in Puchkov and Matalin (2003: 116, as Collyridina); incorrect original stem formation, not in prevailing usage.
Tracheloniadae Gistel, 1850: 75 [stem: *Tracheloni-*]. Type genus: *Trachelonia* Gistel, 1850 [syn. of *Collyris* Fabricius, 1801].
*Colliurides Motschulsky, 1855: 34 [stem: *Colliur-*]. Type genus: *Colliuris* sensu Latreille, 1802 [not *Colliuris* DeGeer, 1774; syn. of *Collyris* Fabricius, 1801]. Comment: original vernacular name unavailable (Art. 11.7.2): not subsequently latinized.

### Subtribe Tricondylina

Naviaux, 1991

Tricondylina Naviaux, 1991: 219 [stem: *Tricondyl-*]. Type genus: *Tricondyla* Latreille, 1822.

### Tribe Ctenostomatini

Laporte, 1834

Ctenostomidae Laporte, 1834b: 38 [stem: *Ctenostomat-*]. Type genus: *Ctenostoma* Klug, 1821. Comment: incorrect original stem formation, not in prevailing usage.

### Tribe Manticorini

Laporte, 1834

Manticoridae Laporte, 1834b: 33 [stem: *Manticor-*]. Type genus: *Manticora* Fabricius, 1781.

### Tribe Megacephalini

Laporte, 1834

Megacephalidae Laporte, 1834b: 33 [stem: *Megacephal-*]. Type genus: *Megacephala* Latreille, 1802.
Oxycheilites J. Thomson, 1857a: 17 [stem: *Oxycheil-*]. Type genus: *Oxycheila* Dejean, 1825. Comment: original vernacular name available (Art. 11.7.2): first used in latinized form by Chaudoir (1861a: 326, as Oxychilini [incorrect stem formation]), generally accepted as in Fleutiaux (1892a: 13, as Oxychilini [incorrect stem formation]); the stem *Oxychil*- should not be used for this taxon in order to avoid homonymy with Oxychilini Hesse, 1927 (type genus *Oxychilus* Fitzinger 1833), currently being used as valid in Mollusca.
Platychilidae W. Horn, 1893: 325 [stem: *Platychil-*]. Type genus: *Platychile* W. S. MacLeay, 1825.
Tetrachae Leng and Mutchler, 1916: 683 [stem: *Tetrach-*]. Type genus: *Tetracha* Hope, 1838.

### Subfamily Carabinae

Latreille, 1802

Carabici Latreille, 1802: 80 [stem: *Carab-*]. Type genus: *Carabus* Linnaeus, 1758.

### Tribe Carabini

Latreille, 1802

Carabici Latreille, 1802: 80 [stem: *Carab-*]. Type genus: *Carabus* Linnaeus, 1758.
Calosomii Bonelli, 1810: Tabula Synoptica [stem: *Calosomat-*]. Type genus: *Calosoma* Weber, 1801. Comment: incorrect original stem formation, not in prevailing usage.
Proceridae Laporte, 1834b: 87 [stem: *Procer-*]. Type genus: *Procerus* Dejean, 1821.
Callisthenisidae Gistel, 1848: [2] [stem: *Callisthen-*]. Type genus: *Callisthenes* Fischer von Waldheim, 1820. Comment: incorrect original stem formation, not in prevailing usage.
*Cechenogenici Morawitz, 1889: 40 [stem: *Cechen-*]. Type genus: *Cechenus* Fischer von Waldheim, 1822. Comment: family-group name unavailable (Art. 11.7.1.1): original name not proposed as a noun.
*Tribacogenici Morawitz, 1889: 40 [stem: *Tribac-*]. Type genus: *Tribax* Fischer von Waldheim, 1817. Comment: family-group name unavailable (Art. 11.7.1.1): original name not proposed as a noun.
*Procrustogenici Morawitz, 1889: 40 [stem: *Procrust-*]. Type genus: *Procrustes* Bonelli, 1810. Comment: family-group name unavailable (Art. 11.7.1.1): original name not proposed as a noun.
Procrustogenici Roeschke, 1898: 285 [stem: *Procrust-*]. Type genus: *Procrustes* Bonelli, 1810.
Aplothoracina Lapouge, 1927: 45 [stem: *Aplothorac-*]. Type genus: *Aplothorax* G. R. Waterhouse, 1842.
*Callistheniens Lapouge, 1927: 47 [stem: *Callisthen-*]. Type genus: *Callisthenes* Fischer von Waldheim, 1820. Comment: original vernacular name unavailable (Art. 11.7.2): proposed after 1899; family-group name proposed as new without reference to Callisthenisidae Gistel, 1848.
Cechenogenici Csiki, 1927: 110 [stem: *Cechen-*]. Type genus: *Cechenus* Fischer von Waldheim, 1822.
Tribacogenici Csiki, 1927: 121 [stem: *Tribac-*]. Type genus: *Tribax* Fischer von Waldheim, 1817.
Megadontici Csiki, 1927: 60 [stem: *Megodont-*]. Type genus: *Megodontus* Solier, 1848 [as *Megadontus*, incorrect subsequent spelling of type genus name, not in prevailing usage]. Comment: incorrect original stem formation, not in prevailing usage.

### Tribe Ceroglossini

Lapouge, 1927

Ceroglossina Lapouge, 1927: 45 [stem: *Cerogloss-*]. Type genus: *Ceroglossus* Solier, 1848.

### Tribe Cychrini

Perty, 1830

Cychrii Perty, 1830: 6 [stem: *Cychr-*]. Type genus: *Cychrus* Fabricius, 1794. Comment: name previously attributed to Laporte (1834b: 86).

### Tribe Pamborini

Hope, 1838

Pamboridae Hope, 1838a: 47 [stem: *Pambor-*]. Type genus: *Pamborus* Latreille, 1812.

### Subfamily Loricerinae

Bonelli, 1810

Loricerides Bonelli, 1810: Tabula Synoptica [stem: *Loricer-*]. Type genus: *Loricera* Latreille, 1802.

### Subfamily Omophroninae

Bonelli, 1810

Omophronii Bonelli, 1810: Tabula Synoptica [stem: *Omophron-*]. Type genus: *Omophron* Latreille, 1802.
Scolyti Motschulsky, 1850: 91 [stem: *Scolyt-*]. Type genus: *Scolytus* Fabricius, 1790 [preoccupied genus name, not *Scolytus* Geoffroy, 1762 [Coleoptera: Curculionidae]; syn. of *Omophron* Latreille, 1802]. Comment: permanently invalid (Art. 39): based on preoccupied type genus; Scolytinae/-ini Latreille, 1804 are currently used as valid in Curculionidae.
Epactiini Fauvel, 1888: 1 [stem: *Epacti-*]. Type genus: *Epactius* Schneider, 1791 [syn. of *Omophron* Latreille, 1802].

### Subfamily Elaphrinae

Latreille, 1802

Elaphrii Latreille, 1802: 81 [stem: *Elaphr-*]. Type genus: *Elaphrus* Fabricius, 1775.

### Subfamily Migadopinae

Chaudoir, 1861

Migadopidae Chaudoir, 1861b: 510 [stem: *Migadop-*]. Type genus: *Migadops* G. R. Waterhouse, 1842.

### Tribe Amarotypini

Erwin, 1985

Amarotypini Erwin, 1985: 468 [stem: *Amarotyp-*]. Type genus: *Amarotypus* H. W. Bates, 1872.

### Tribe Migadopini

Chaudoir, 1861

Migadopidae Chaudoir, 1861b: 510 [stem: *Migadop-*]. Type genus: *Migadops* G. R. Waterhouse, 1842.

### Subtribe Aquilicina

Moret, 2005

Aquilicina Moret, 2005: 30 [stem: *Aquilic-*]. Type genus: *Aquilex* Moret, 1989.

### Subtribe Migadopina

Chaudoir, 1861

Migadopidae Chaudoir, 1861b: 510 [stem: *Migadop-*]. Type genus: *Migadops* G. R. Waterhouse, 1842.
Monolobinae Jeannel, 1938: 10, in key [stem: *Monolob-*]. Type genus: *Monolobus* Solier, 1849.
Loxomeriformes Erwin, 1985: 446 [stem: *Loxomer-*]. Type genus: *Loxomerus* Chaudoir, 1842.

### Subfamily Hiletinae

Schiødte, 1848

Hiletini Schiødte, 1848: 69 [stem: *Hilet-*]. Type genus: *Hiletus* Schiødte, 1848.
Camaragnathini Csiki, 1927: 341 [stem: *Camaragnath-*]. Type genus: *Camaragnathus* Bertrand-Bocandé, 1849 [syn. of *Hiletus* Schiødte, 1847].

### Subfamily Scaritinae

Bonelli, 1810

Scaritides Bonelli, 1810: Tabula Synoptica [stem: *Scarit-*]. Type genus: *Scarites* Fabricius, 1775.

### Tribe Carenini

MacLeay, 1887

Carenides W. J. MacLeay, 1887: 117 [stem: *Caren-*]. Type genus: *Carenum* Bonelli, 1813.

### Tribe Clivinini

Rafinesque, 1815

Clivinidia Rafinesque, 1815: 109 [stem: *Clivin-*]. Type genus: *Clivina* Latreille, 1802.

### Subtribe Ardistomina

Putzeys, 1867

Ardistomides Putzeys, 1867: 200 [stem: *Ardistom-*]. Type genus: *Ardistomis* Putzeys, 1846. Comment: original vernacular name available (Art. 11.7.2): first used in latinized form by Csiki (1927: 547, as Ardistomina), generally accepted as in Balkenohl (2003: 219, as Ardistomina); current spelling maintained (Art. 29.3.1.1): incorrect stem formation in prevailing usage (should be *Ardistomid*-).

### Subtribe Clivinina

Rafinesque, 1815

Clivinidia Rafinesque, 1815: 109 [stem: *Clivin-*]. Type genus: *Clivina* Latreille, 1802.
Reicheina Jeannel, 1957: 141, in key [stem: *Reichei-*]. Type genus: *Reicheia* Saulcy, 1862. Comment: incorrect original stem formation, not in prevailing usage.
Italodytina Jeannel, 1957: 141, in key [stem: *Italodyt-*]. Type genus: *Italodytes* Müller, 1938.
Reicheiina Basilewsky, 1980: 293 [stem: *Reichei-*]. Type genus: *Reicheia* Saulcy, 1862. Comment: family-group name proposed as new without reference to Reicheiina Jeannel, 1957.

### Subtribe Forcipatorina

Bänninger, 1938

*Oxystomides Putzeys, 1867: 12 [stem: *Oxystom-*]. Type genus: *Oxystomus* Dejean, 1825 [preoccupied genus name, not *Oxystomus* Fischer von Waldheim, 1803 [Mammalia]; syn. of *Forcipator* Maindron, 1904]. Comment: original vernacular name unavailable (Art. 11.7.2): subsequently used in latinized form but not generally attributed to Putzeys (1867).
Oxystomina Csiki, 1927: 491. Type genus: *Oxystomus* Dejean, 1825 [preoccupied genus name, not *Oxystomus* Fischer von Waldheim, 1803 [Mammalia]; syn. of *Forcipator* Maindron, 1904]. Comment: permanently invalid (Art. 39): based on preoccupied type genus.
Forcipatorina Bänninger, 1938: 83 [stem: *Forcipator-*]. Type genus: *Forcipator* Maindron, 1904.

### Tribe Dalyatini

Mateu, 2002

Dalyatinae Mateu, 2002: 67 [stem: *Dalyat-*]. Type genus: *Dalyat* Mateu, 2002.

### Tribe Dyschiriini

Kolbe, 1880

Dyschiriini Kolbe, 1880: 266 [stem: *Dyschiri-*]. Type genus: *Dyschirius* Bonelli, 1810.

### †Tribe Palaeoaxinidiini

McKay, 1991

Palaeoaxinidini McKay, 1991: 10 [stem: *Palaeoaxinidi-*]. Type genus: *Palaeoaxinidium* McKay, 1991. Comment: incorrect original stem formation, not in prevailing usage.

### Tribe Pasimachini

Putzeys, 1867

Pasimachides Putzeys, 1867: 3 [stem: *Pasimach-*]. Type genus: *Pasimachus* Bonelli, 1813. Comment: original vernacular name available (Art. 11.7.2): first used in latinized form and generally accepted as in Csiki (1927: 444, as Pasimachina).

### Tribe Promecognathini

LeConte, 1853

Promecognathi J. L. LeConte, 1853b: 394 [stem: *Promecognath-*]. Type genus: *Promecognathus* Chaudoir, 1846.
Axinidiini Basilewsky, 1963: 307 [stem: *Axinidi-*]. Type genus: *Axinidium* Sturm, 1843.

### Tribe Salcediini

Alluaud, 1930

Salcediini Alluaud, 1930a: 21 [stem: *Salcedi-*]. Type genus: *Salcedia* Fairmaire, 1899.

### Subtribe Androzelmina

Bell, 1998

Androzelmina R. T. Bell, 1998: 271 [stem: *Androzelm-*]. Type genus: *Androzelma* Dostal, 1993.

### Subtribe Salcediina

Alluaud, 1930 (1929)

Zelmides Andrewes, 1929: 209, 416 [stem: *Zelm-*]. Type genus: *Zelma* Andrewes, 1920 [syn. of *Salcedia* Fairmaire, 1899]. Comment: use of younger name Salcediina Alluaud, 1930 conserved over this name (Art. 40.2).
Salcediini Alluaud, 1930a: 21 [stem: *Salcedi-*]. Type genus: *Salcedia* Fairmaire, 1899. Comment: name proposed to replace Zelmini Andrewes, 1929 because of the synonymy of the type genus; use of Salcediina conserved over Zelmina Andrewes, 1929 (Art. 40.2).

### Subtribe Solenogenyina

Bell, 1998

Solenogenyina R. T. Bell, 1998: 271 [stem: *Solenogeny-*]. Type genus: *Solenogenys* Westwood, 1859.

### Tribe Scaritini

Bonelli, 1810

Scaritides Bonelli, 1810: Tabula Synoptica [stem: *Scarit-*]. Type genus: *Scarites* Fabricius, 1775.

### Subtribe Acanthoscelina

Csiki, 1927

Acanthoscelina Csiki, 1927: 489 [stem: *Acanthoscel-*]. Type genus: *Acanthoscelis* Dejean, 1825. Comment: the incorrect stem formation is in prevailing usage (should be *Acanthoscelid*-) and is maintained (Art. 29.3.1.1); usage of the stem *Acanthoscel*- avoids homonymy with Acanthoscelidina Bridwell, 1946 (type genus *Acanthoscelides* Schilsky, 1905) in Chrysomelidae.

### Subtribe Corintascarina

Basilewsky, 1973

Corintascarini Basilewsky, 1973a: 10 [stem: *Corintascar-*]. Type genus: *Corintascaris* Basilewsky, 1952. Comment: current spelling maintained (Art. 29.3.1.1): incorrect stem formation in prevailing usage (should be *Corintascarid*-).

### Subtribe Dyscherina

Basilewsky, 1973

Dyscherina Basilewsky, 1973a: 101 [stem: *Dyscher-*]. Type genus: *Dyscherus* Chaudoir, 1855.

### Subtribe Ochyropina

Basilewsky, 1973

Ochyropini Basilewsky, 1973a: 9 [stem: *Ochyrop-*]. Type genus: *Ochyropus* Schiødte, 1847. Comment: current spelling maintained (Art. 29.5): incorrect stem formation in prevailing usage (should be *Ochyropod*-).

### Subtribe Oxylobina

Andrewes, 1929

Oxylobides Andrewes, 1929: 209, 292 [stem: *Oxylob-*]. Type genus: *Oxylobus* Chaudoir, 1855.

### Subtribe Scapterina

Putzeys, 1867

Scaptérides Putzeys, 1867: 7 [stem: *Scapter-*]. Type genus: *Scapterus* Dejean, 1826. Comment: original vernacular name available (Art. 11.7.2): first used in latinized form by Csiki (1927: 490, as Scapterina), generally accepted as in Balkenohl (2003: 231, as Scapterina).
Passalidiina Csiki, 1927: 489 [stem: *Passalidi-*]. Type genus: *Passalidius* Chaudoir, 1863.

### Subtribe Scaritina

Bonelli, 1810

Scaritides Bonelli, 1810: Tabula Synoptica [stem: *Scarit-*]. Type genus: *Scarites* Fabricius, 1775.

### Subtribe Storthodontina

Jeannel, 1946

Storthodontini Jeannel, 1946: 237, in key [stem: *Storthodont-*]. Type genus: *Storthodontus* Chaudoir, 1855.

### Subfamily Broscinae

Hope, 1838

Broschidae Hope, 1838a: 80 [stem: *Brosc-*]. Type genus: *Broscus* Panzer, 1813.

### Tribe Broscini

Hope, 1838

Broschidae Hope, 1838a: 80 [stem: *Brosc-*]. Type genus: *Broscus* Panzer, 1813.

### Subtribe Axonyina

Roig-Juñent, 2000

Axonyina Roig-Juñent, 2000: 18 [stem: *Axony-*]. Type genus: *Axonya* Andrewes, 1923.

### Subtribe Baripodina

Jeannel, 1941

Barypitae Jeannel, 1941: 287, in key [stem: *Baripod-*]. Type genus: *Baripus* Dejean, 1828 [as *Barypus*, unjustified emendation of type genus name by Agassiz (1846b: 43), not in prevailing usage]. Comment: incorrect original stem formation, not in prevailing usage.

### Subtribe Broscina

Hope, 1838

Broschidae Hope, 1838a: 80 [stem: *Brosc-*]. Type genus: *Broscus* Panzer, 1813 [as *Broschus*, incorrect subsequent spelling of type genus name, not in prevailing usage]. Comment: incorrect original stem formation, not in prevailing usage.
Cephalotida Heer, 1838: 12 [stem: *Cephalot-*]. Type genus: *Cephalotes* Bonelli, 1810 [preoccupied genus name, not *Cephalotes* Latreille, 1802 [Hymenoptera]; syn. of *Broscus* Panzer, 1813]. Comment: permanently invalid (Art. 39): based on preoccupied type genus.
Zacotini G. H. Horn, 1881: 169 [stem: *Zacot-*]. Type genus: *Zacotus* J. L. LeConte, 1869.

### Subtribe Creobiina

Jeannel, 1941

Creobitae Jeannel, 1941: 287, in key [stem: *Creobi-*]. Type genus: *Creobius* Guérin-Méneville, 1838. Comment: incorrect original stem formation, not in prevailing usage.

### Subtribe Nothobroscina

Roig-Juñent, 2000

Nothobroscina Roig-Juñent, 2000: 32 [stem: *Nothobrosc-*]. Type genus: *Nothobroscus* Roig-Juñent and Ball, 1995.

### Subfamily Apotominae

LeConte, 1853

Apotomi J. L. LeConte, 1853b: 370 [stem: *Apotom-*]. Type genus: *Apotomus* Illiger, 1807.

### Subfamily Siagoninae

Bonelli, 1813

Siagones Bonelli, 1813: 24 [stem: *Siagon-*]. Type genus: *Siagona* Latreille, 1804.

### Tribe Enceladini

Horn, 1881

Enceladini G. H. Horn, 1881: 118 [stem: *Encelad-*]. Type genus: *Enceladus* Bonelli, 1813.

### Tribe Lupercini

Lecordier, 1977

Lupercini Lecordier, 1977: 628 [stem: *Luperc-*]. Type genus: *Luperca* Laporte, 1840.

### Tribe Siagonini

Bonelli, 1813

Siagones Bonelli, 1813: 24 [stem: *Siagon-*]. Type genus: *Siagona* Latreille, 1804.

### Subfamily Melaeninae

Csiki, 1933

Coscinides Chaudoir, 1876b: 115 [stem: *Coscini-*]. Type genus: *Coscinia* Dejean, 1831 [preoccupied genus name, not *Coscinia* Hübner, 1819 [Lepidoptera]; syn. of *Cymbionotum* Baudi, 1864]. Comment: permanently invalid (Art. 39): based on preoccupied type genus; incorrect original stem formation, not in prevailing usage.
Granigerini Bedel, 1900: 24 [stem: *Graniger-*]. Type genus: *Graniger* sensu Chaudoir, 1876 [not *Graniger* Motschulsky, 1864; syn. of *Cymbionotum* Baudi, 1864]. Comment: based on a misidentified type genus; an application will need to be submitted to the Commission to suppress this name for the Principles of Priority and Homonymy (Art. 65.2.1) if Granigerini Antoine, 1959 in Harpalinae: Harpalini: Harpalina is to be used as valid in the future.
Melaeninae Csiki, 1933: 1650 [stem: *Melaen-*]. Type genus: *Melaenus* Dejean, 1831. Comment: published 26 May 1933; name proposed after 1930 without description or bibliographic reference to such a description (Art. 13.1), however available because it was used as valid before 2000 as in Lorenz (1998b: 149) and was not rejected by an author who, between 1961 and 1999, applied Article 13 of the then current edition of the Code (see Art. 13.2.1).
Cymbionotini Andrewes, 1933: 3 [stem: *Cymbionot-*]. Type genus: *Cymbionotum* Baudi di Selve, 1864. Comment: published 30 June 1933.

### Subfamily Gehringiinae

Darlington, 1933

Gehringiini Darlington, 1933: 110 [stem: *Gehringi-*]. Type genus: *Gehringia* Darlington, 1933.

### Tribe Gehringiini

Darlington, 1933

Gehringiini Darlington, 1933: 110 [stem: *Gehringi-*]. Type genus: *Gehringia* Darlington, 1933.

### Subtribe Gehringiina

Darlington, 1933

Gehringiini Darlington, 1933: 110 [stem: *Gehringi-*]. Type genus: *Gehringia* Darlington, 1933.

### Subtribe Helenaeina

Deuve, 2007

Helenaeina Deuve, 2007: 217 [stem: *Helenae-*]. Type genus: *Helenaea* Schatzmayr and Koch, 1934.

### Subfamily Trechinae

Bonelli, 1810

Trechii Bonelli, 1810: Tabula Synoptica [stem: *Trech-*]. Type genus: *Trechus* Clairville, 1806.

### Tribe Bembidiini

Stephens, 1827

Bembidiidae Stephens, 1827: 5 [stem: *Bembidi-*]. Type genus: *Bembidion* Latreille, 1802 [as *Bembidium*, unjustified emendation of type genus name by Gyllenhal (1810: 12), not in prevailing usage].

### Subtribe Anillina

Jeannel, 1937

Anillini Jeannel, 1937: 244, in key [stem: *Anill-*]. Type genus: *Anillus* Jacquelin du Val, 1851. Comment: precedence (Anillina Jeannel, 1937 vs Scotodipnina Jeannel, 1937) given to taxon originally proposed at the higher rank (Art. 24.1).
Scotodipnina Jeannel, 1937: 265, in key [stem: *Scotodipn-*]. Type genus: *Scotodipnus* Schaum, 1860.
Typhlocharina Jeanne, 1973: 95, in key [stem: *Typhlocharit-*]. Type genus: *Typhlocharis* Dieck, 1869. Comment: incorrect original stem formation, not in prevailing usage.

### Subtribe Bembidiina

Stephens, 1827

Bembidiidae Stephens, 1827: 5 [stem: *Bembidi-*]. Type genus: *Bembidion* Latreille, 1802 [as *Bembidium*, unjustified emendation of type genus name by Gyllenhal (1810), not in prevailing usage].
Peryphidae Kirby, 1837: 52 [stem: *Peryph-*]. Type genus: *Peryphus* Dejean, 1821.

### Subtribe Tachyina

Motschulsky, 1862

Tachyaires Motschulsky, 1862: 24 [stem: *Tachy-*]. Type genus: *Tachys* Dejean, 1821 [placed on the Official List of Generic Names in Zoology (ICZN 1990c)]. Comment: original vernacular name available (Art. 11.7.2): first used in latinized form by Jeannel (1941: 401, 422, as Tachyini), generally accepted as in J. K. Park et al. (2006: 91, as Tachyini); current spelling maintained (Art. 29.5): incorrect stem formation in prevailing usage (should be *Tache*-, see Alonso-Zarazaga 2007).
Micratopini Casey, 1914a: 42 [stem: *Micratop-*]. Type genus: *Micratopus* Casey, 1914.
Limnastini Jeannel, 1937: 245, in key [stem: *Lymnast-*]. Type genus: *Lymnastis* Motschulsky, 1862 [as *Limnastis*, unjustified emendation of type genus name by Ganglbauer (1891a: 181), not in prevailing usage]. Comment: incorrect original stem formation, not in prevailing usage.

### Subtribe Xystosomina

Erwin, 1994

Xystosomina Erwin, 1994: 560 [stem: *Xystosom-*]. Type genus: *Xystosomus* Schaum, 1863.

### Tribe Horologionini

Jeannel, 1949

Horologionidae Jeannel, 1949b: 91 [stem: *Horologion-*]. Type genus: *Horologion* J. M. Valentine, 1932. Comment: current spelling maintained (Art. 29.5): incorrect stem formation in prevailing usage (should be *Horologi*-).

### Tribe Pogonini

Laporte, 1834

Pogonidae Laporte, 1834b: 70 [stem: *Pogon-*]. Type genus: *Pogonus* Dejean, 1821.
Pogonopsini Bedel, 1900: 20 [stem: *Pogonopse-*]. Type genus: *Pogonopsis* Bedel, 1898. Comment: incorrect original stem formation, not in prevailing usage.

### Tribe Trechini

Bonelli, 1810

Trechii Bonelli, 1810: Tabula Synoptica [stem: *Trech-*]. Type genus: *Trechus* Clairville, 1806.

### Subtribe Aepina

Fowler, 1887

Aëpyes Fowler, 1887: 123 [stem: *Aep-*]. Type genus: *Aepus* Samouelle, 1819.
Temnostegini Enderlein, 1909: 376 [stem: *Temnosteg-*]. Type genus: *Temnostega* Enderlein, 1905.

### Subtribe Cnidina

Jeannel, 1958

Cnidina Jeannel, 1958b: 733 [stem: *Cnid-*]. Type genus: *Cnides* Motschulsky, 1862. Comment: name proposed after 1930 without description or bibliographic reference to such a description (Art. 13.1), however available because it was used as valid before 2000 as in Casale and Laneyrie (1982: 9, as Cnidini) and was not rejected by an author who, between 1961 and 1999, applied Article 13 of the then current edition of the Code (see Art. 13.2.1).

### Subtribe Perileptina

Sloane, 1903

Perileptides Sloane, 1903: 583 [stem: *Perilept-*]. Type genus: *Perileptus* Schaum, 1860. Comment: Perileptides Sloane, 1903 was treated as Latin and available in Madge (1989: 466) but the valid name for this taxon was listed as Perilepti Jeannel, 1922 by Csiki (1928) and Perileptina Jeannel, 1922 by Lorenz (2005); we follow the usage of Madge (1989).
Ochthephilini Jeannel, 1922: 165 [stem: *Ochthephil-*]. Type genus: *Ochthephilus* Nietner, 1857 [preoccupied genus name, not *Ochthephilus* Mulsant and Rey, 1856 [Coleoptera: Staphylinidae]; syn. of *Perileptus* Schaum, 1860]. Comment: permanently invalid (Art. 39): based on preoccupied type genus.

### Subtribe Plocamotrechina

Jeannel, 1960

*Plocamotrechina Jeannel, 1958b: 733 [stem: *Plocamotrech-*]. Type genus: *Plocamotrechus* Jeannel, 1926 [syn. of *Pachydesus* Motschulsky, 1864]. Comment: unavailable family-group name, proposed after 1930 without description or bibliographic reference to such a description (Art. 13.1).
Plocamotrechina Jeannel, 1960: 53, in key [stem: *Plocamotrech-*]. Type genus: *Plocamotrechus* Jeannel, 1926 [syn. of *Pachydesus* Motschulsky, 1864].

### Subtribe Trechina

Bonelli, 1810

Trechii Bonelli, 1810: Tabula Synoptica [stem: *Trech-*]. Type genus: *Trechus* Clairville, 1806.
*Aphaenopses Fauvel, 1888: 7 [stem: *Aphoenop-*]. Type genus: *Aphoenops* Bonvouloir, 1861 [as *Aphaenops*, incorrect subsequent spelling of type genus name, not in prevailing usage]. Comment: original vernacular name unavailable (Art. 11.7.2): not subsequently latinized; incorrect original stem formation, not in prevailing usage.
Homaloderini Jeannel, 1926: 397, in key [stem: *Omaloder-*]. Type genus: *Omalodera* Blanchard, 1842 [as *Homalodera*, unjustified emendation of type genus name by Gemminger and Harold (1868a: 389), not in prevailing usage]. Comment: incorrect original stem formation, not in prevailing usage.

### Subtribe Trechodina

Jeannel, 1926

Trechodini Jeannel, 1926: 469 [stem: *Trechod-*]. Type genus: *Trechodes* Blackburn, 1901.
Thalassophili Csiki, 1928: 233 [stem: *Thalassophil-*]. Type genus: *Thalassophilus* Wollaston, 1854.

### Tribe Zolini

Sharp, 1886

Zolini Sharp, 1886a: 371 [stem: *Zol-*]. Type genus: *Zolus* Sharp, 1886.

### Subtribe Chalteniina

Roig-Juñent and Cicchino, 2001

Chalteniina Roig-Juñent and Cicchino, 2001: 658 [stem: *Chalteni-*]. Type genus: *Chaltenia* Roig-Juñent and Cicchino, 2001.

### Subtribe Sinozolina

Deuve, 1997

Sinozolini Deuve, 1997: 35 [stem: *Sinozol-*]. Type genus: *Sinozolus* Bedel, 1898.

### Subtribe Zolina

Sharp, 1886

Zolini Sharp, 1886a: 371 [stem: *Zol-*]. Type genus: *Zolus* Sharp, 1886.
Merizodini Sloane, 1920: 139 [stem: *Merizodont-*]. Type genus: *Merizodus* Solier, 1849. Comment: incorrect original stem formation, not in prevailing usage.
*Oopterini Jeannel, 1938: 45 [stem: *Oopter-*]. Type genus: *Oopterus* Guérin-Méneville, 1841. Comment: unavailable family-group name, proposed after 1930 without description or bibliographic reference to such a description (Art. 13.1).
Oopterini Jeannel, 1940: 93 [stem: *Oopter-*]. Type genus: *Oopterus* Guérin-Méneville, 1841.

### Subfamily Patrobinae

Kirby, 1837

Patrobidae Kirby, 1837: 50 [stem: *Patrob-*]. Type genus: *Patrobus* Dejean, 1821.

### Tribe Lissopogonini

Zamotajlov, 2000

Lissopogonini Zamotajlov, 2000: 266 [stem: *Lissopogon-*]. Type genus: *Lissopogonus* Andrewes, 1923.
Zolinopatrobina Deuve and Tian, 2001: 421 [stem: *Zolinopatrob-*]. Type genus: *Zolinopatrobus* Deuve and Tian, 2001 [syn. of *Lissopogonus* Andrewes, 1923].

### Tribe Patrobini

Kirby, 1837

Patrobidae Kirby, 1837: 50 [stem: *Patrob-*]. Type genus: *Patrobus* Dejean, 1821.

### Subtribe Deltomerina

Chaudoir, 1871

Deltomeridae Chaudoir, 1871a: 51 [stem: *Deltomer-*]. Type genus: *Deltomerus* Motschulsky, 1850.

### Subtribe Deltomerodina

Zamotajlov, 2002

Deltomerodina Zamotajlov, 2002: 83 [stem: *Deltomerod-*]. Type genus: *Deltomerodes* Deuve, 1992.

### Subtribe Patrobina

Kirby, 1837

Patrobidae Kirby, 1837: 50 [stem: *Patrob-*]. Type genus: *Patrobus* Dejean, 1821.

### Subtribe Platidiolina

Zamotajlov and Lafer, 2001

Platidiolini Zamotajlov and Lafer, 2001: 411 [stem: *Platidiol-*]. Type genus: *Platidiolus* Chaudoir, 1878.

### Subfamily Psydrinae

LeConte, 1853

Psydri J. L. LeConte, 1853b: 393 [stem: *Psydr-*]. Type genus: *Psydrus* J. L. LeConte, 1846.

### Tribe Amblytelini

Blackburn, 1892

Amblytelides Blackburn, 1892: 85 [stem: *Amblytel-*]. Type genus: *Amblytelus* Erichson, 1842. Comment: the junior homonym Amblytelinae Viereck, 1918 (type genus *Amblyteles* Wesmael, 1845) is available in Hymenoptera: Ichneumonidae, it is presently considered a synonym of Ichneumonini Latreille, 1802; both family-group names are based on the same stem; this case is to be referred to the Commission to remove the homonymy (Art. 55.3.1).

### Tribe Mecyclothoracini

Jeannel, 1940

Mecyclothoracitae Jeannel, 1940: 97 [stem: *Mecyclothorac-*]. Type genus: *Mecyclothorax* Sharp, 1903. Comment: name proposed after 1930 without description or bibliographic reference to such a description (Art. 13.1), however available because it was used as valid before 2000 as in Lorenz (1998a: 222) and was not rejected by an author who, between 1961 and 1999, applied Article 13 of the then current edition of the Code (see Art. 13.2.1).

### Tribe Meonini

Sloane, 1898

Meonides Sloane, 1898: 470 [stem: *Meon-*]. Type genus: *Meonis* Laporte, 1867. Comment: current spelling maintained (Art. 29.3.1.1): incorrect stem formation in prevailing usage (should be *Meonid*-).

### Tribe Moriomorphini

Sloane, 1890

Moriomorphini Sloane, 1890: 646 [stem: *Moriomorph-*]. Type genus: *Moriomorpha* Laporte, 1867.
Melisoderides Sloane, 1898: 470 [stem: *Melisoder-*]. Type genus: *Melisodera* Westwood, 1835.

### Tribe Psydrini

LeConte, 1853

Psydri J. L. LeConte, 1853b: 393 [stem: *Psydr-*]. Type genus: *Psydrus* J. L. LeConte, 1846.
Nomiidae Gozis, 1875: 3 [stem: *Nomi-*]. Type genus: *Nomius* Laporte, 1835. Comment: the younger name Nomiidae Robertson, 1904 (type genus *Nomia* Latreille, 1804) has been used in Hymenoptera and a Case was recently submitted to the Commission to emend the beetle family-group name to Nomiusidae (Art. 55.3) in order to remove the homonymy (Engel and Bouchard 2009).

### Tribe Tropopterini

Sloane, 1898

Tropopterides Sloane, 1898: 470 [stem: *Tropopter-*]. Type genus: *Tropopterus* Solier, 1849.

### Subfamily Nototylinae

Bänninger, 1927

Tylonotini Schaum, 1863: 74 [stem: *Tylonot-*]. Type genus: *Tylonotus* Schaum, 1863 [preoccupied genus name, not *Tylonotus* Haldeman, 1847 [Coleoptera: Cerambycidae], not *Tylonotus* Fieber, 1858 [Hemiptera]; syn. of *Nototylus* Gemminger and Harold, 1868]. Comment: permanently invalid (Art. 39): based on preoccupied type genus.
Nototylini Bänninger, 1927: 177 [stem: *Nototyl-*]. Type genus: *Nototylus* Gemminger and Harold, 1868.

### Subfamily Paussinae

Latreille, 1806

Paussili Latreille, 1806: 234 [stem: *Pauss-*]. Type genus: *Paussus* Linnaeus, 1775.

### Tribe Metriini

LeConte, 1853

Metrii J. L. LeConte, 1853b: 394 [stem: *Metri-*]. Type genus: *Metrius* Eschscholtz, 1829.

### Tribe Mystropomini

Horn, 1881

Mystropomini G. H. Horn, 1881: 116 [stem: *Mystropom-*]. Type genus: *Mystropomus* Chaudoir, 1848.

### Tribe Ozaenini

Hope, 1838

Ozaenidae Hope, 1838a: 107 [stem: *Ozaen-*]. Type genus: *Ozaena* A. G. Olivier, 1812.
*Tropopsitos Solier, 1849: 179 [stem: *Tropopse-*]. Type genus: *Tropopsis* Solier, 1849. Comment: original vernacular name unavailable (Art. 11.7.2): not subsequently latinized; incorrect original stem formation, not in prevailing usage.
Pseudozaenini Sloane, 1905: 704 [stem: *Pseudozaen-*]. Type genus: *Pseudozaena* Laporte, 1834.
Eustrini Jeannel, 1946: 48 [stem: *Eustr-*]. Type genus: *Eustra* Schmidt-Göbel, 1846.
Pachytelini Jeannel, 1946: 47, in key [stem: *Pachytel-*]. Type genus: *Pachyteles* Perty, 1830.
Physeitae Jeannel, 1946: 47, in key [stem: *Physe-*]. Type genus: *Physea* Brullé, 1834.

### Tribe Paussini

Latreille, 1806

Paussili Latreille, 1806: 234 [stem: *Pauss-*]. Type genus: *Paussus* Linnaeus, 1775.

### †Subtribe Arthropteritina

Luna de Carvalho, 1961

Arthropteritina Luna de Carvalho, 1961: 3 [stem: *Arthropterit-*]. Type genus: *Arthropterites* Wasmann, 1926.

### Subtribe Carabidomemnina

Wasmann, 1928

*Carabidomemninen Kolbe, 1927a: 178 [stem: *Carabidomemn-*]. Type genus: *Carabidomemnus* Kolbe, 1924. Comment: original vernacular name unavailable (Art. 11.7.2): proposed after 1899.
*Eohomopterinen Kolbe, 1927b: 214 [stem: *Eohomopter-*]. Type genus: *Eohomopterus* Wasmann, 1920. Comment: original vernacular name unavailable (Art. 11.7.2): proposed after 1899; the latinized form Eohomopterini was used by Nagel (1987: 33, 54, 55, 56) but it is unavailable because it was proposed after 1930 without description or bibliographic reference to such a description (Art. 13.1).
Carabidomemninae Wasmann, 1928: 271 [stem: *Carabidomemn-*]. Type genus: *Carabidomemnus* Kolbe, 1924.

### Subtribe Cerapterina

Billberg, 1820

Cerapterides Billberg, 1820a: 47 [stem: *Cerapter-*]. Type genus: *Cerapterus* Swederus, 1788.
Megalopaussinae Wasmann, 1920: 111 [stem: *Megalopauss-*]. Type genus: *Megalopaussus* Lea, 1906.
*Arthropterinen Kolbe, 1927b: 211 [stem: *Arthropter-*]. Type genus: *Arthropterus* W. S. MacLeay, 1838. Comment: original vernacular name unavailable (Art. 11.7.2): proposed after 1899.
Arthropterini Wasmann, 1928: 274 [stem: *Arthropter-*]. Type genus: *Arthropterus* W. S. MacLeay, 1838. Comment: Arthropteridae Fieber, 1861 has been used in Hemiptera but it is not based on a genus and is therefore unavailable; Arthropterini Wasmann, 1928 is a junior homonym of Arthropteridae Jordan, 1923 proposed in Pisces (type genus *Arthropterus* Agassiz, 1843, preoccupied genus name replaced by *Arthrobatis* Whitley 1940); the Pisces name is permanently invalid since its type genus is a junior homonym; an application to the Commission is needed to conserve the Coleoptera name if it is to be used as valid.
*Mesarthropterina Nagel, 1987: 60 [stem: *Mesarthropter-*]. Type genus: *Mesarthropterus* Wasmann, 1926. Comment: unavailable family-group name, proposed after 1930 without description or bibliographic reference to such a description (Art. 13.1).

### †Subtribe Eopaussina

Luna de Carvalho, 1951

*Eopaussina Darlington, 1950: 84 [stem: *Eopauss-*]. Type genus: *Eopaussus* Wasmann, 1926. Comment: unavailable family-group name, proposed after 1930 without description or bibliographic reference to such a description (Art. 13.1).
Eopaussinae Luna de Carvalho, 1951: 22 [stem: *Eopauss-*]. Type genus: *Eopaussus* Wasmann, 1926.

### Subtribe Heteropaussina

Janssens, 1950

Pleuropterini Wasmann, 1920: 111 [stem: *Pleuropter-*]. Type genus: *Pleuropterus* Westwood, 1841 [preoccupied genus name, not *Pleuropterus* Burnett, 1829 [Mammalia]; syn. of *Janssenius* Luna de Carvalho, 1951]. Comment: permanently invalid (Art. 39): based on preoccupied type genus.
Heteropaussines Janssens, 1950: 5 (footnote) [stem: *Heteropauss-*]. Type genus: *Heteropaussus* J. Thomson, 1860.
Janssenini Luna de Carvalho, 1951: 18 [stem: *Jansseni-*]. Type genus: *Janssenius* Luna de Carvalho, 1951. Comment: incorrect original stem formation, not in prevailing usage.

### Subtribe Homopterina

Wasmann, 1920

Homopterinae Wasmann, 1920: 111 [stem: *Homopter-*]. Type genus: *Homopterus* Westwood, 1841.

### Subtribe Paussina

Latreille, 1806

Paussili Latreille, 1806: 234 [stem: *Pauss-*]. Type genus: *Paussus* Linnaeus, 1775.
Platyrhopalini Jeannel, 1946: 65 [stem: *Platyrhopal-*]. Type genus: *Platyrhopalus* Westwood, 1833.
Enneapaussini Jeannel, 1946: 62 [stem: *Enneapauss-*]. Type genus: *Enneapaussus* Jeannel, 1946.
Ceratoderina Darlington, 1950: 107 [stem: *Ceratoder-*]. Type genus: *Ceratoderus* Westwood, 1841.
Hylotorini Luna de Carvalho, 1951: 50 [stem: *Hylotor-*]. Type genus: *Hylotorus* Dalman, 1823.
Leleupaussina Luna de Carvalho, 1989: 430 [stem: *Leleupauss-*]. Type genus: *Leleupaussus* Luna de Carvalho, 1962.

### Subtribe Pentaplatarthrina

Jeannel, 1946

*Pentaplatarthrinen Kolbe, 1927b: 214 [stem: *Pentaplatarthr-*]. Type genus: *Pentaplatarthrus* Westwood, 1833. Comment: original vernacular name unavailable (Art. 11.7.2): proposed after 1899.
Pentaplatarthrini Jeannel, 1946: 65 [stem: *Pentaplatarthr-*]. Type genus: *Pentaplatarthrus* Westwood, 1833.
Hexaplatarthrina Luna de Carvalho, 1961: 3 [stem: *Hexaplatarthr-*]. Type genus: *Hexaplatarthrus* Jeannel, 1955.

### Tribe Protopaussini

Gestro, 1892

Protopaussini Gestro, 1892: 707 [stem: *Protopauss-*]. Type genus: *Protopaussus* Gestro, 1892.

### Subfamily Brachininae

Bonelli, 1810

Brachinii Bonelli, 1810: Tabula Synoptica [stem: *Brachin-*]. Type genus: *Brachinus* Weber, 1801.

### Tribe Brachinini

Bonelli, 1810

Brachinii Bonelli, 1810: Tabula Synoptica [stem: *Brachin-*]. Type genus: *Brachinus* Weber, 1801.

### Subtribe Aptinina

Gistel, 1848

Aptinidae Gistel, 1848: [2] [stem: *Aptin-*]. Type genus: *Aptinus* Bonelli, 1810. Comment: Aptemidae Gistel (1856a: 356) is probably a misspelling of Aptinidae (see Madge 1989: 460).
Styphlomerini Habu, 1984: 133 [stem: *Styphlomer-*]. Type genus: *Styphlomerus* Chaudoir, 1875.

### Subtribe Brachinina

Bonelli, 1810

Brachinii Bonelli, 1810: Tabula Synoptica [stem: *Brachin-*]. Type genus: *Brachinus* Weber, 1801.

### Subtribe Mastacina

Erwin, 1970

Mastacina Erwin, 1970: 28, 32 [stem: *Mastac-*]. Type genus: *Mastax* Fischer von Waldheim, 1828.

### Subtribe Pheropsophina

Jeannel, 1949

Pheropsophini Jeannel, 1949c: 1083, in key [stem: *Pheropsoph-*]. Type genus: *Pheropsophus* Solier, 1833.

### Tribe Crepidogastrini

Jeannel, 1949

Crepidogastritae Jeannel, 1949c: 1080 [stem: *Crepidogastr-*]. Type genus: *Crepidogaster* Boheman, 1848.

### Subfamily Harpalinae

Bonelli, 1810

Harpalii Bonelli, 1810: Tabula Synoptica [stem: *Harpal-*]. Type genus: *Harpalus* Latreille, 1802. Comment: First Reviser (Harpalinae Bonelli, 1810 vs Dryptinae Bonelli, 1810 vs Lebiinae Bonelli, 1810 vs Licininae Bonelli, 1810 vs Panagaeinae Bonelli, 1810 vs Platyninae Bonelli, 1810 vs Pterostichinae Bonelli, 1810 vs Zabrinae Bonelli, 1810 vs Zuphiinae Bonelli, 1810) not determined, current usage maintained; Graphipterinae Latreille, 1802 has precedence over this taxon, however, Harpalinae Bonelli, 1810 is in prevailing usage and is maintained (Art. 35.5).

### Tribe Abacetini

Chaudoir, 1873

Abacétides Chaudoir, 1873a: 5 [stem: *Abacet-*]. Type genus: *Abacetus* Dejean, 1828. Comment: original vernacular name available (Art. 11.7.2): first used in latinized form by H. W. Bates (1873: 224, as Abacetinae), generally accepted as in Bousquet (2003: 346, as Abacetini).
Celioschesini Jeannel, 1948b: 442 [stem: *Celioschese-*]. Type genus: *Celioschesis* Tschitschérine, 1898 [syn. of *Aristopus* LaFerté-Sénectère, 1853]. Comment: incorrect original stem formation, not in prevailing usage.
Loxandrina Erwin and Sims, 1984: 383, in key [stem: *Loxandr-*]. Type genus: *Loxandrus* J. L. LeConte, 1852.
Loxandrini Bousquet and Larochelle, 1993: 31 [stem: *Loxandr-*]. Type genus: *Loxandrus* J. L. LeConte, 1852. Comment: family-group name proposed as new without reference to Loxandrina Erwin and Sims, 1984.

### Tribe Amorphomerini

Sloane, 1923

Trimerinae Alluaud, 1922: 500 [stem: *Trimer-*]. Type genus: *Trimerus* Chaudoir, 1878 [preoccupied genus name, not *Trimerus* Green, 1832 [Trilobita]; syn. of *Amorphomerus* Sloane, 1923]. Comment: permanently invalid (Art. 39): based on preoccupied type genus.
Amorphomerini Sloane, 1923a: 249 [stem: *Amorphomer-*]. Type genus: *Amorphomerus* Sloane, 1923.

### Tribe Anthiini

Bonelli, 1813

Anthies Bonelli, 1813: 18 [stem: *Anthi-*]. Type genus: *Anthia* Weber, 1801.
Polyhirmi Rousseau, 1905: 3, in key [stem: *Polyhirm-*]. Type genus: *Polyhirma* Chaudoir, 1850.
Cypholobini G. Strohmeyer, 1928: 287 [stem: *Cypholob-*]. Type genus: *Cypholoba* Chaudoir, 1850.

### Tribe Atranini

Horn, 1881

Atrani G. H. Horn, 1881: 145 [stem: *Atran-*]. Type genus: *Atranus* J. L. LeConte, 1848.

### Tribe Bascanini

Basilewsky, 1953

Bascanini Basilewsky, 1953a: 165 [stem: *Bascan-*]. Type genus: *Bascanus* Péringuey, 1896.

### Tribe Calophaenini

Jeannel, 1948

*Calophaenidae Jeannel, 1942: 1017 [stem: *Calophaen-*]. Type genus: *Calophaena* Klug, 1821. Comment: unavailable family-group name, proposed after 1930 without description or bibliographic reference to such a description (Art. 13.1).
Calophaenidae Jeannel, 1948b: 378 [stem: *Calophaen-*]. Type genus: *Calophaena* Klug, 1821.

### Tribe Catapieseini

Bates, 1882

Catapiesinae H. W. Bates, 1882: 90 [stem: *Catapiese-*]. Type genus: *Catapiesis* Solier, 1835. Comment: incorrect original stem formation, not in prevailing usage.

### Tribe Chaetodactylini

Tschitschérine, 1903

Chaetodactylini Tschitschérine, 1903: 157 [stem: *Chaetodactyl-*]. Type genus: *Chaetodactyla* Tschitschérine, 1897.

### Tribe Chaetogenyini

Emden, 1958

Chaetogenyina Emden, 1958: 24 [stem: *Chaetogeny-*]. Type genus: *Chaetogenys* Emden, 1958.

### Tribe Chlaeniini

Brullé, 1834

Chlaenides Brullé, 1834: 123 [stem: *Chlaeni-*]. Type genus: *Chlaenius* Bonelli, 1810.

### Subtribe Callistina

Laporte, 1834

Callistidae Laporte, 1834b: 80 [stem: *Callist-*]. Type genus: *Callistus* Bonelli, 1810. Comment: published before 9 August 1834.
Eusynetadae Gistel, 1856a: 356 [stem: *Eusynet-*]. Type genus: *Eusyneta* Gistel, 1856 [syn. of *Callistus* Bonelli, 1810]. Comment: incorrect original stem formation, not in prevailing usage.

### Subtribe Chlaeniina

Brullé, 1834

Chlaenides Brullé, 1834: 123 [stem: *Chlaeni-*]. Type genus: *Chlaenius* Bonelli, 1810. Comment: published before 2 August 1834; original vernacular name available (Art. 11.7.2): first used in latinized form by Erichson (1837: 96, as Chlaeniini), generally accepted as in Lorenz (2005: 328, as Chlaeniini); incorrect original stem formation, not in prevailing usage.
Lissaucheniidae Gistel, 1848: [2] [stem: *Lissaucheni-*]. Type genus: *Lissauchenius* W. S. MacLeay, 1825.
Rhopalomelini Alluaud, 1930b: 105 [stem: *Rhopalomel-*]. Type genus: *Rhopalomelus* Boheman, 1848.
Chlaeniodini Jeannel, 1949c: 777 [stem: *Chlaeniod-*]. Type genus: *Chlaeniodus* Jeannel, 1949.
Eccoptomenini Jeannel, 1949c: 821 [stem: *Eccoptomen-*]. Type genus: *Eccoptomenus* Chaudoir, 1850.
Chlaenionini Jeannel, 1949c: 776, in key [stem: *Chlaenion-*]. Type genus: *Chlaenionus* Kuntzen, 1913.
Procletini Basilewsky, 1950b: 49 [stem: *Proclet-*]. Type genus: *Procletus* Péringuey, 1896.
Pleroticini Basilewsky, 1950b: 50 [stem: *Plerotic-*]. Type genus: *Pleroticus* Péringuey, 1896.
Callistoidini Basilewsky, 1950b: 51 [stem: *Callistoid-*]. Type genus: *Callistoides* Motschulsky, 1865.
Harpaglossini Basilewsky, 1950b: 52 [stem: *Harpagloss-*]. Type genus: *Harpaglossus* Motschulsky, 1858.
Leptodinodini Basilewsky and Grundmann, 1955: 205 [stem: *Leptodinod-*]. Type genus: *Leptodinodes* Jeannel, 1949.
Chlaenioctenini Basilewsky and Grundmann, 1955: 204 [stem: *Chlaeniocten-*]. Type genus: *Chlaenioctenus* H. W. Bates, 1892.
Brachylobini Basilewsky and Grundmann, 1955: 204 [stem: *Brachylob-*]. Type genus: *Brachylobus* Chaudoir, 1876.

### Tribe Cnemalobini

Germain, 1911

Cnémacanthides Lacordaire, 1854a: 237 [stem: *Cnemacanth-*]. Type genus: *Cnemacanthus* sensu Brullé, 1834 [not *Cnemacanthus* Gray, 1832; syn. of *Cnemalobus* Guérin-Méneville, 1838]. Comment: original vernacular name available (Art. 11.7.2): first used in latinized form by Schaum (1860: 353, as Cnemacanthidae), generally accepted as in Broun (1880: 7, as Cnemacanthidae); based on a misidentified type genus, name treated here as invalid until an application is submitted to the Commission to suppress it for the Principle of Priority (Art. 65.2.1).
Cnemalobini Germain, 1911: 53 [stem: *Cnemalob-*]. Type genus: *Cnemalobus* Guérin-Méneville, 1838.
Cnemalobini Bousquet and Larochelle, 1993: 27 [stem: *Cnemalob-*]. Type genus: *Cnemalobus* Guérin-Méneville, 1838. Comment: proposed as a new without reference to Cnemalobini Germain, 1911.

### Tribe Cratocerini

Lacordaire, 1854

Cratocérides Lacordaire, 1854a: 257 [stem: *Cratocer-*]. Type genus: *Cratocerus* Dejean, 1829. Comment: original vernacular name available (Art. 11.7.2): first used in latinized form by Murray (1858: 343, as Cratoceridae), generally accepted as in Csiki (1929: 493, as Cratoceri).

### Tribe Ctenodactylini

Laporte, 1834

Ctenodactylidae Laporte, 1834b: 45 [stem: *Ctenodactyl-*]. Type genus: *Ctenodactyla* Dejean, 1825.
*Leptotrachélides Chaudoir, 1848: 52 [stem: *Leptotrachel-*]. Type genus: *Leptotrachelus* Latreille, 1829. Comment: original vernacular name unavailable (Art. 11.7.2): not subsequently latinized, also originally proposed in synonymy with Rhagocrepides (see Madge 1989: 464).
Rhagocrepides Chaudoir, 1848: 52 [stem: *Rhagocrepid-*]. Type genus: *Rhagocrepis* Eschscholtz, 1829 [syn. of *Leptotrachelus* Latreille, 1829]. Comment: original vernacular name available (Art. 11.7.2): first used in latinized form by and generally accepted as in Chaudoir (1861b: 528, as Rhagocrepidae); incorrect original stem formation, not in prevailing usage.

### Tribe Cuneipectini

Sloane, 1907

Cuneipectini Sloane, 1907a: 358 [stem: *Cuneipect-*]. Type genus: *Cuneipectus* Sloane, 1907. Comment: current spelling maintained (Art. 29.5): incorrect stem formation in prevailing usage (should be *Cuneipector*-).

### Tribe Cyclosomini

Laporte, 1834

Cyclosomidae Laporte, 1834b: 69 [stem: *Cyclosom-*]. Type genus: *Cyclosomus* Latreille, 1829.

### Subtribe Cyclosomina

Laporte, 1834

Cyclosomidae Laporte, 1834b: 69 [stem: *Cyclosom-*]. Type genus: *Cyclosomus* Latreille, 1829.
Tétragonodérides Chaudoir, 1871b: 111 [stem: *Tetragonoder-*]. Type genus: *Tetragonoderus* Dejean, 1829. Comment: original vernacular name available (Art. 11.7.2): first used in latinized form by and generally accepted as in Chaudoir (1876a: 2, as Tetragonoderidae).
Sarothrocrepidae Chaudoir, 1876a: 83 [stem: *Sarothrocrepid-*]. Type genus: *Sarothrocrepis* Chaudoir, 1850. Comment: incorrect original stem formation, not in prevailing usage; Chaudoir also used the spelling Sarothrocrépides in his original publication on page 80.

### Subtribe Masoreina

Chaudoir, 1871

*Somoplatides Chaudoir, 1846: 511 [stem: *Somoplat-*]. Type genus: *Somoplatus* Dejean, 1829. Comment: original vernacular name unavailable (Art. 11.7.2): subsequently used in latinized form but not generally attributed to Chaudoir (1846) and generally accepted as valid; this name was first used in latinized form by Carus and Engelmann (1861: 1806 [index], as Somoplatidae) referring to Chaudoir’s paper, but the name was not used as valid; this name was treated as “vernacular, not scientific” by Ball (1979: 77); Basilewsky (1984: 527, as Somoplatini) used this taxon as valid but did not refer to Chaudoir’s original vernacular name, Somoplatini Basilewsky, 1984 is also unavailable since it was proposed after 1930 without description or bibliographic reference to such a description (Art. 13.1); Lorenz (2005: 451) listed this name as as “Somoplatides Chaudoir 1846 [suppr.]”.
Mazoréides Chaudoir, 1871b: 111 [stem: *Masore-*]. Type genus: *Masoreus* Dejean, 1821 [implicit use of *Mazoreus* as the type genus, which is an incorrect subsequent spelling of the type genus name, not in prevailing usage]. Comment: original vernacular name available (Art. 11.7.2): first used in latinized form by Chaudoir (1876: 2, as Masoreidae), generally accepted as in Lorenz (2005: 451, as Masoreina); incorrect stem formation, not in prevailing usage.
Corsyrini Ganglbauer, 1891b: 53 [stem: *Corsyr-*]. Type genus: *Corsyra* Dejean, 1825.
Aephnidiina Jakobson, 1907: 390 [stem: *Aephnidi-*]. Type genus: *Aephnidius* W. S. MacLeay, 1825.
Anaulacini Csiki, 1932b: 1287 [stem: *Anaulac-*]. Type genus: *Anaulacus* W. S. MacLeay, 1825. Comment: name proposed after 1930 without description or bibliographic reference to such a description (Art. 13.1), however available because it was used as valid before 2000 as in Blackwelder (1944: 52) and was not rejected by an author who, between 1961 and 1999, applied Article 13 of the then current edition of the Code (see Art. 13.2.1).
Discopterini Jedlička, 1941: 6, in key [stem: *Discopter-*]. Type genus: *Discoptera* Semenov, 1889.

### Tribe Dercylini

Sloane, 1923

Dercylini Sloane, 1923b: 250a [stem: *Dercyl-*]. Type genus: *Dercylus* Laporte, 1832.
Dercylidae Jeannel, 1948b: 626 [stem: *Dercyl-*]. Type genus: *Dercylus* Laporte, 1832. Comment: family-group name proposed as new without reference to Dercylini Sloane, 1923.

### Tribe Drimostomatini

Chaudoir, 1872

Drimostomidas Chaudoir, 1872a: 283 [stem: *Drimostomat-*]. Type genus: *Drimostoma* Dejean, 1830 [syn. of *Caelostomus* W. S. MacLeay, 1825]. Comment: the original spelling Drimostomidas is the accusative (Latin) form of Drimostomidae and was recognized as such by Madge (1989: 463); original incorrect original stem formation, not in prevailing usage.
Caelostomina Burgeon, 1935: 194 [stem: *Caelostom-*]. Type genus: *Caelostomus* W. S. MacLeay, 1825.
Cyrtolaina Whitehead and Ball, 1975: 595 [stem: *Cyrtola-*]. Type genus: *Cyrtolaus* H. W. Bates, 1882.

### Tribe Dryptini

Bonelli, 1810

Dryptinae Bonelli, 1810: Tabula Synoptica [stem: *Drypt-*]. Type genus: *Drypta* Latreille, 1797.

### Tribe Enoicini

Basilewsky, 1985

Enoicini Basilewsky, 1985: 16 [stem: *Enoic-*]. Type genus: *Enoicus* Péringuey, 1896.

### Tribe Galeritini

Kirby, 1825

Galeritidae Kirby, 1825: 564 [stem: *Galerit-*]. Type genus: *Galerita* Fabricius, 1801 [*Galerita* Gouan, 1770 [Aves] has been suppressed both for the Principle of Priority and the Principle of Homonymy (ICZN 1968b); *Galerita* Fabricius, 1801 placed on the Official List of Generic Names in Zoology (ICZN 1968b)].

### Subtribe Galeritina

Kirby, 1825

Galeritidae Kirby, 1825: 564 [stem: *Galerit-*]. Type genus: *Galerita* Fabricius, 1801 [*Galerita* Gouan, 1770 [Aves] has been suppressed both for the Principle of Priority and the Principle of Homonymy (ICZN 1968b); *Galerita* Fabricius, 1801 placed on the Official List of Generic Names in Zoology (ICZN 1968b)]. Comment: family-group name previously attributed to J. L. LeConte (1853b) or Lacordaire (1854a); name placed on the Official List of Family-Group Names in Zoology (ICZN 1968b, as Galeritini Lacordaire, 1854).
Galeritinini Jeannel, 1949c: 1057 [stem: *Galeritin-*]. Type genus: *Galeritina* Jeannel, 1949 [syn. of *Galerita* Fabricius, 1801]. Comment: replacement name for “Galeritini Lacordaire, 1854” because of the homonymy of the type genus; name placed on the Official Index of Rejected and Invalid Family-Group Names in Zoology (ICZN 1968b).
Galeritulini Jedlička, 1963: 279, in key [stem: *Galeritul-*]. Type genus: *Galeritula* Strand, 1936 [syn. of *Galerita* Fabricius, 1801]. Comment: replacement name for “Galeritini Lacordaire, 1854” because of the homonymy of the type genus; name placed on the Official Index of Rejected and Invalid Family-Group Names in Zoology (ICZN 1968b).

### Subtribe Planetina

Jedlička, 1941

Planetini Jedlička, 1941: 7, in key [stem: *Planet-*]. Type genus: *Planetes* W. S. MacLeay, 1825.

### Tribe Geobaenini

Péringuey, 1896

Geobaenides Péringuey, 1896: 469 [stem: *Geobaen-*]. Type genus: *Geobaenus* Dejean, 1829.

### Tribe Ginemini

Ball and Shpeley, 2002

Ginemini Ball and Shpeley, 2002: 77 [stem: *Ginem-*]. Type genus: *Ginema* Ball and Shpeley, 2002.

### Tribe Glyptini

Horn, 1881

Glypti G. H. Horn, 1881: 179 [stem: *Glypt-*]. Type genus: *Glyptus* Brullé, 1835 [*Glyptus* Hoffmannsegg, 1818 placed on the Official Index of Rejected and Invalid Generic Names in Zoology and *Glyptus* Brullé, 1835 placed on the Official List of Generic Names in Zoology (ICZN 1985b)].

### Tribe Graphipterini

Latreille, 1802

Graphipterides Latreille, 1802: 83 [stem: *Graphipter-*]. Type genus: *Graphipterus* Latreille, 1802. Comment: usage of Harpalinae Bonelli, 1810 is maintained over Graphipterinae Latreille, 1802 (Art. 35.5).

### Tribe Harpalini

Bonelli, 1810

Harpalii Bonelli, 1810: Tabula Synoptica [stem: *Harpal-*]. Type genus: *Harpalus* Latreille, 1802.

### Subtribe Anisodactylina

Lacordaire, 1854 nomen protectum

Eurytrichini J. L. LeConte, 1847: 376 [stem: *Eurytrich-*]. Type genus: *Eurytrichus* J. L. LeConte, 1847 [syn. of *Anisotarsus* Chaudoir, 1837]. Comment: *nomen oblitum* (Appendix 1).
Anisodactylides Lacordaire, 1854a: 268 [stem: *Anisodactyl-*]. Type genus: *Anisodactylus* Dejean, 1829. Comment: *nomen protectum* (see Appendix 1); original vernacular name available (Art. 11.7.2): first used in latinized form by Murray (1858: 346, as Anisodactylidae), generally accepted as in Lorenz (2005: 348, as Anisodactylini).
Anisotarsi Csiki, 1932a: 1039 [stem: *Anisotars-*]. Type genus: *Anisotarsus* Chaudoir, 1837. Comment: name proposed after 1930 without description or bibliographic reference to such a description (Art. 13.1), however available because it was used as valid before 2000 as in Blackwelder (1944: 46) and was not rejected by an author who, between 1961 and 1999, applied Article 13 of the then current edition of the Code (see Art. 13.2.1).
*Geopini Csiki, 1932a: 1026 [stem: *Geopin-*]. Type genus: *Geopinus* J. L. LeConte, 1847. Comment: unavailable family-group name, proposed after 1930 without description or bibliographic reference to such a description (Art. 13.1).
*Gnathaphani Schauberger, 1934: 104, 106 [stem: *Gnathaphan-*]. Type genus: *Gnathaphanus* W. S. MacLeay, 1825. Comment: unavailable family-group name, proposed after 1930 without description or bibliographic reference to such a description (Art. 13.1).

### Subtribe Harpalina

Bonelli, 1810

Harpalii Bonelli, 1810: Tabula Synoptica [stem: *Harpal-*]. Type genus: *Harpalus* Latreille, 1802. Comment: First Reviser found (Harpalini Bonelli, 1810 vs Ditomini Bonelli, 1810) is Noonan (1976: 28).
Ditomici Bonelli, 1810: Tabula Synoptica [stem: *Ditom-*]. Type genus: *Ditomus* Bonelli, 1810.
Acinopidae Laporte, 1834b: 67 [stem: *Acinopod-*]. Type genus: *Acinopus* Dejean, 1821. Comment: incorrect original stem formation, not in prevailing usage.
Ophonidae Laporte, 1834b: 68 [stem: *Ophon-*]. Type genus: *Ophonus* Dejean, 1821 [placed on the Official List of Generic Names in Zoology (ICZN 1990c)].
Cratognathidae Laporte, 1834b: 70 [stem: *Cratognath-*]. Type genus: *Cratognathus* Dejean, 1829.
Stenomorphidae Laporte, 1834b: 71 [stem: *Stenomorph-*]. Type genus: *Stenomorphus* Dejean, 1831.
Daptini J. L. LeConte, 1847: 371 [stem: *Dapt-*]. Type genus: *Daptus* Fischer von Waldheim, 1823.
Amblystomini Fauvel, 1889: 17 [stem: *Amblystom-*]. Type genus: *Amblystomus* Erichson, 1837.
Trichopselaphini Tschitschérine, 1900: 351 [stem: *Trichopselaph-*]. Type genus: *Trichopselaphus* Chaudoir, 1843.
Selenophorini Casey, 1914b: 134 [stem: *Selenophor-*]. Type genus: *Selenophorus* Dejean, 1829.
Pachycarina Stichel, 1923: 81 [stem: *Pachycar-*]. Type genus: *Pachycarus* Solier, 1835.
*Dioctini Csiki, 1932a: 1023a [stem: *Dioct-*]. Type genus: *Dioctes* Ménétriés, 1849 [syn. of *Machozetus* Chaudoir, 1850]. Comment: family-group name unavailable (Art. 11.6): originally published as synonym and not made available subsequently.
Diorychi Csiki, 1932a: 1193 [stem: *Diorych-*]. Type genus: *Dioryche* W. S. MacLeay, 1825. Comment: name proposed after 1930 without description or bibliographic reference to such a description (Art. 13.1), however available because it was used as valid before 2000 as in Blackwelder (1944: 48) and was not rejected by an author who, between 1961 and 1999, applied Article 13 of the then current edition of the Code (see Art. 13.2.1).
*Machozeti Csiki, 1932a: 1023 [stem: *Machozet-*]. Type genus: *Machozetus* Chaudoir, 1850. Comment: unavailable family-group name, proposed after 1930 without description or bibliographic reference to such a description (Art. 13.1).
Bradybaeni Csiki, 1932a: 1187 [stem: *Bradybaen-*]. Type genus: *Bradybaenus* Dejean, 1829. Comment: name proposed after 1930 without description or bibliographic reference to such a description (Art. 13.1), however available because it was used as valid before 2000 as in Noonan (1976: 28) and was not rejected by an author who, between 1961 and 1999, applied Article 13 of the then current edition of the Code (see Art. 13.2.1); Bradybaenidae Pilsbry, 1939 (type genus *Bradybaena* Beck, 1837) is currently used as valid in Mollusca, both family-group names have a correct stem; this case is to be referred to the Commission to remove the homonymy (Art. 55.3.1).
*Euryderi Csiki, 1932a: 1081 [stem: *Euryder-*]. Type genus: *Euryderus* J. L. LeConte, 1846. Comment: unavailable family-group name, proposed after 1930 without description or bibliographic reference to such a description (Art. 13.1).
*Heteracanthi Csiki, 1932a: 1085 [stem: *Heteracanth-*]. Type genus: *Heteracantha* Brullé, 1834. Comment: unavailable family-group name, proposed after 1930 without description or bibliographic reference to such a description (Art. 13.1).
*Barysomi Csiki, 1932a: 1192 [stem: *Barysom-*]. Type genus: *Barysomus* Dejean, 1829. Comment: unavailable family-group name, proposed after 1930 without description or bibliographic reference to such a description (Art. 13.1).
Machozeti Schauberger, 1934: 99 [stem: *Machozet-*]. Type genus: *Machozetus* Chaudoir, 1850.
Trichotichnini Jeannel, 1942: 624 [stem: *Trichotichn-*]. Type genus: *Trichotichnus* Morawitz, 1863.
Bleusei Antoine, 1959: 386 [stem: *Bleuse-*]. Type genus: *Bleusea* Bedel, 1896.
Eriotomi Antoine, 1959: 354 [stem: *Eriotom-*]. Type genus: *Eriotomus* Piochard de la Brûlerie, 1873 [syn. of *Oedesis* Motschulsky, 1850].
Granigeri Antoine, 1959: 357 [stem: *Graniger-*]. Type genus: *Graniger* Motschulsky, 1864. Comment: an application will need to be submitted to the Commission to suppress Granigerini Bedel, 1900 (based on the misidentified type genus *Graniger* sensu Chaudoir, 1876) for the Principles of Priority and Homonymy (Art. 65.2.1) if this name is to be used as valid.
Cratacanthi Lindroth, 1968: 742 [stem: *Cratacanth-*]. Type genus: *Cratacanthus* Dejean, 1829.
*Carterophonini Jeanne, 1971: 11 [stem: *Carterophon-*]. Type genus: *Carterophonus* Ganglbauer, 1891 [syn. of *Graniger* Motschulsky, 1864]. Comment: unavailable family-group name, proposed after 1930 without description or bibliographic reference to such a description (Art. 13.1).

### Subtribe Pelmatellina

Bates, 1882

Pelmatellinae H. W. Bates, 1882: 67 [stem: *Pelmatell-*]. Type genus: *Pelmatellus* H. W. Bates, 1882.

### Subtribe Stenolophina

Kirby, 1837

Stenolophidae Kirby, 1837: 46 [stem: *Stelonoph-*]. Type genus: *Stenolophus* Dejean, 1821.
Polpochilinae H. W. Bates, 1891a: 10 [stem: *Polpochil-*]. Type genus: *Polpochila* Solier, 1849.
Acupalpini Tschitschérine, 1900: 351 [stem: *Acupalp-*]. Type genus: *Acupalpus* Latreille, 1829.
Cratocarini Casey, 1914b: 48 [stem: *Cratocar-*]. Type genus: *Cratocara* J. L. LeConte, 1863 [syn. of *Polpochila* Solier, 1849].
Pachytracheli Csiki, 1932a: 1082 [stem: *Pachytrachel-*]. Type genus: *Pachytrachelus* Chaudoir, 1852. Comment: name proposed after 1930 without description or bibliographic reference to such a description (Art. 13.1), however available because it was used as valid before 2000 as in Jeannel (1948b: 720, as Pachytrachelini) and was not rejected by an author who, between 1961 and 1999, applied Article 13 of the then current edition of the Code (see Art. 13.2.1).
*Agonoderi Csiki, 1932a: 1188 [stem: *Agonoder-*]. Type genus: *Agonoderus* Dejean, 1829. Comment: unavailable family-group name, proposed after 1930 without description or bibliographic reference to such a description (Art. 13.1).
*Bradycelli Csiki, 1932a: 1222 [stem: *Bradycell-*]. Type genus: *Bradycellus* Erichson, 1837. Comment: unavailable family-group name, proposed after 1930 without description or bibliographic reference to such a description (Art. 13.1).
Anoplogenii Schauberger, 1937: 272 [stem: *Anoplogeni-*]. Type genus: *Anoplogenius* Chaudoir, 1852 [syn. of *Loxoncus* Schmidt-Göbel, 1846].
*Dichirotrichi F. Burmeister, 1939: 186 [stem: *Dicheirotrich-*]. Type genus: *Dicheirotrichus* Jacquelin du Val, 1855 [as *Dichirotrichus*, unjustified emendation of type genus name by Gemminger and Harold (1868a: 262), not in prevailing usage]. Comment: unavailable family-group name, proposed after 1930 without description or bibliographic reference to such a description (Art. 13.1); incorrect original stem formation, not in prevailing usage (see Madge 1989: 463).
Bradycellini Jeannel, 1942: 700 [stem: *Bradycell-*]. Type genus: *Bradycellus* Erichson, 1837.
Hippolaetina Basilewsky, 1951b: 258 [stem: *Hippoloet-*]. Type genus: *Hippoloetis* Laporte, 1835 [as *Hippolaetis*, incorrect subsequent spelling of type genus name, not in prevailing usage]. Comment: incorrect original stem formation, not in prevailing usage.
Anthracini Schuler, 1970: 114 [stem: *Anthrac-*]. Type genus: *Anthracus* Motschulsky, 1850.

### Tribe Helluonini

Hope, 1838

Helluonidae Hope, 1838a: 110 [stem: *Helluon-*]. Type genus: *Helluo* Bonelli, 1813.

### Subtribe Helluonina

Hope, 1838

Helluonidae Hope, 1838a: 110 [stem: *Helluon-*]. Type genus: *Helluo* Bonelli, 1813.

### Subtribe Omphrina

Jedlička, 1941

Omphrini Jedlička, 1941: 6, in key [stem: *Omphr-*]. Type genus: *Omphra* Dejean, 1825.
Helluomorphina H. Reichardt, 1974: 226 [stem: *Helluomorph-*]. Type genus: *Helluomorpha* Laporte, 1834.

### Tribe Hexagoniini

Horn, 1881 (1834)

Trigonodactyliens Brullé, 1834: 127 [stem: *Trigonodactyl-*]. Type genus: *Trigonodactyla* Dejean, 1831 [syn. of *Hexagonia* Kirby, 1825]. Comment: original vernacular name available (Art. 11.7.2): first used in latinized form and generally accepted as in Desmarest (1851: 56, as Trigonodactylidae); use of the younger name Hexagoniini G. H. Horn, 1881 conserved over this name (Art. 40.2).
Hexagoniae G. H. Horn, 1881: 146 [stem: *Hexagoni-*]. Type genus: *Hexagonia* Kirby, 1825. Comment: use of family-group name conserved over Trigonodactylini Brullé, 1834 (Art. 40.2).

### Tribe Idiomorphini

Bates, 1891

Idiomorphinae H. W. Bates, 1891b: cccxxxiii [stem: *Idiomorph-*]. Type genus: *Idiomorphus* Chaudoir, 1846.
Perochnoristhinae Basilewsky, 1973b: 224 [stem: *Perochnoristh-*]. Type genus: *Perochnoristhus* Basilewsky, 1973.

### Tribe Lachnophorini

LeConte, 1853

Lachnophori J. L. LeConte, 1853b: 375 [stem: *Lachnophor-*]. Type genus: *Lachnophorus* Dejean, 1831.

### Subtribe Lachnophorina

LeConte, 1853

Lachnophori J. L. LeConte, 1853b: 375 [stem: *Lachnophor-*]. Type genus: *Lachnophorus* Dejean, 1831.
Anchonodérides Lacordaire, 1854a: 373 [stem: *Anchonoder-*]. Type genus: *Anchonoderus* Reiche, 1843. Comment: original vernacular name available (Art. 11.7.2): first used in latinized form and generally accepted as in H. W. Bates (1871: 30, as Anchonoderinae).
Eucaeri J. L. LeConte, 1861: 22 [stem: *Eucaer-*]. Type genus: *Eucaerus* J. L. LeConte, 1853.
Egini G. H. Horn, 1881: 152 [stem: *Eg-*]. Type genus: *Ega* Laporte, 1834.

### Subtribe Selinina

Jeannel, 1948

Selinini Jeannel, 1948b: 743, in key [stem: *Selin-*]. Type genus: *Selina* Motschulsky, 1858. Comment: the junior homonym Selinina Koch, 1956 (type genus *Selinus* Mulsant and Rey, 1853) is available in Tenebrionidae; this case is to be referred to the Commission to remove the homonymy (Art. 55.3.1).

### Tribe Lebiini

Bonelli, 1810

Lebiotae Bonelli, 1810: Tabula Synoptica [stem: *Lebi-*]. Type genus: *Lebia* Latreille, 1802. Comment: First Reviser (Lebiini Bonelli, 1810 vs Dromiusini Bonelli, 1810) not determined, current usage maintained.

### Subtribe Actenonycina

Bates, 1871

Actenonycinae H. W. Bates, 1871: 30 [stem: *Actenonyc-*]. Type genus: *Actenonyx* A. White, 1846. Comment: current spelling maintained (Art. 29.5): incorrect stem formation in prevailing usage (should be *Actenonych*-).

### Subtribe Agrina

Kirby, 1837

Agridae Kirby, 1837: 13 [stem: *Agr-*]. Type genus: *Agra* Fabricius, 1801.

### Subtribe Apenina

Ball, 1983

Apenina Ball, 1983: 516 [stem: *Apen-*]. Type genus: *Apenes* J. L. LeConte, 1851.
*Trymosternini Zaballos and Jeanne, 1994: 116 [stem: *Trymostern-*]. Type genus: *Trymosternus* Chaudoir, 1873. Comment: unavailable family-group name, proposed after 1930 without description or bibliographic reference to such a description (Art. 13.1).
*Platytarini Zaballos and Jeanne, 1994: 116 [stem: *Platytar-*]. Type genus: *Platytarus* Fairmaire, 1850. Comment: unavailable family-group name, proposed after 1930 without description or bibliographic reference to such a description (Art. 13.1).

### Subtribe Calleidina

Chaudoir, 1873

Callidides Chaudoir, 1873b: 97 [stem: *Calleid-*]. Type genus: *Calleida* Dejean, 1824 [as *Callida*, unjustified emendation of type genus name by Agassiz (1846b: 58), not in prevailing usage]. Comment: original vernacular name available (Art. 11.7.2): first used in latinized form by H. W. Bates (1873: 311, as Calleidinae), generally accepted as in Csiki (1932b: 1436, as Callidi [incorrect stem formation]); incorrect stem formation, not in prevailing usage.
Plochionidae Gozis, 1875: 3 [stem: *Plochion-*]. Type genus: *Plochionus* Dejean, 1821.
Anomotarina Habu, 1967: 117 [stem: *Anomotar-*]. Type genus: *Anomotarus* Chaudoir, 1875.

### Subtribe Celaenephina

Habu, 1982

Celaenephina Habu, 1982: 111 [stem: *Celaeneph-*]. Type genus: *Celaenephes* Schmidt-Göbel, 1846.

### Subtribe Cymindidina

Laporte, 1834

Cymindidae Laporte, 1834b: 46 [stem: *Cymindid-*]. Type genus: *Cymindis* Latreille, 1806. Comment: incorrect original stem formation, not in prevailing usage.
Taridae Gistel, 1848: [2] [stem: *Tar-*]. Type genus: *Tarus* Clairville, 1806 [syn. of *Cymindis* Latreille, 1806].
Pseudomasoreini Jeannel, 1942: 1039, in key [stem: *Pseudomasore-*]. Type genus: *Pseudomasoreus* Desbrochers des Loges, 1904.

### Subtribe Demetriadina

Bates, 1886

Demetriinae H. W. Bates, 1886: 207 [stem: *Demetriad-*]. Type genus: *Demetrias* Bonelli, 1810. Comment: incorrect original stem formation, not in prevailing usage.
Peliocypini Basilewsky, 1984: 553 [stem: *Peliocypad-*]. Type genus: *Peliocypas* Schmidt-Göbel, 1846. Comment: incorrect original stem formation, not in prevailing usage.

### Subtribe Dromiusina

Bonelli, 1810

Dromiei Bonelli, 1810: Tabula Synoptica [stem: *Dromius-*]. Type genus: *Dromius* Bonelli, 1810 [placed on the Official List of Generic Names in Zoology (ICZN 2006b)]. Comment: Dromiidae Bonelli, 1810 placed on the Official Index of Rejected and Invalid Family-Group Names in Zoology, correct stem ruled to be *Dromius*- and Dromiusidae Bonelli, 1810 placed on the Official List of Family-Group Names in Zoology (ICZN 2006b).
Lichnasthenitae J. Thomson, 1858: 35 [stem: *Lichnasthen-*]. Type genus: *Lichnasthenus* J. Thomson, 1858. Comment: J. Thomson (1858: 35) used the spelling Lichnastenitae but corrected the name to Lichnasthenitae in the same publication in the index (page 458) and in the errata (page [472, unn.]), the corrected spelling is considered a justified emendation (see Madge 1989: 464).
Lionychidae Jeannel, 1948b: 378, in key [stem: *Lionych-*]. Type genus: *Lionychus* Wissmann, 1846.
Singilini Jeannel, 1949c: 915 [stem: *Singil-*]. Type genus: *Singilis* Rambur, 1837.
*Syntomini Jeanne, 1972: 101 [stem: *Syntom-*]. Type genus: *Syntomus* Hope, 1838. Comment: unavailable family-group name, proposed after 1930 without description or bibliographic reference to such a description (Art. 13.1).
Metadromiina Basilewsky, 1984: 545 [stem: *Metadromi-*]. Type genus: *Metadromius* Bedel, 1907.
Metaxymorphina Basilewsky, 1984: 551 [stem: *Metaxymorph-*]. Type genus: *Metaxymorphus* Chaudoir, 1850.
Singiliomimina Basilewsky, 1984: 552 [stem: *Singiliomim-*]. Type genus: *Singiliomimus* Péringuey, 1896.

### Subtribe Gallerucidiina

Chaudoir, 1872

Gallerucidiae Chaudoir, 1872b: 416 [stem: *Gallerucidi-*]. Type genus: *Gallerucidia* Chaudoir, 1872.
Lebidiina Jakobson, 1907: 394 [stem: *Lebidi-*]. Type genus: *Lebidia* Morawitz, 1862.

### Subtribe Lebiina

Bonelli, 1810

Lebiotae Bonelli, 1810: Tabula Synoptica [stem: *Lebi-*]. Type genus: *Lebia* Latreille, 1802.
Encratidae Gistel, 1856a: 355 [stem: *Encrat-*]. Type genus: *Encrates* Gistel, 1848 [syn. of *Lebia* Latreille, 1802].
Lampriadae Chaudoir, 1871b: 115 [stem: *Lampri-*]. Type genus: *Lamprias* Bonelli, 1810. Comment: incorrect original stem formation, not in prevailing usage.
Physodérides Chaudoir, 1877: 213 [stem: *Physoder-*]. Type genus: *Physodera* Eschscholtz, 1829. Comment: original vernacular name available (Art. 11.7.2): first used in latinized form by H. W. Bates (1883: 207, as Physoderinae), generally accepted as in Lorenz (2005: 489, as Physoderina).

### Subtribe Metallicina

Basilewsky, 1984

Metallicini Basilewsky, 1984: 542 [stem: *Metallic-*]. Type genus: *Metallica* Chaudoir, 1872.

### Subtribe Nemotarsina

Bates, 1883

Nemotarsinae H. W. Bates, 1883: 173 [stem: *Nemotars-*]. Type genus: *Nemotarsus* J. L. LeConte, 1853.

### Subtribe Pericalina

Hope, 1838

Pericallidae Hope, 1838a: 105 [stem: *Perical-*]. Type genus: *Pericalus* W. S. MacLeay, 1825. Comment: incorrect original stem formation, not in prevailing usage.
*Mormolycites Blanchard, 1845a: 366 [stem: *Mormolyc-*]. Type genus: *Mormolyce* Hagenbach, 1825. Comment: original vernacular name unavailable (Art. 11.7.2): subsequently used in latinized form but not generally attributed to Blanchard (1845a).
Costodérides Chaudoir, 1848: 116 [stem: *Coptoder-*]. Type genus: *Coptodera* Dejean, 1825. Comment: original vernacular name available (Art. 11.7.2): first used in latinized form by H. W. Bates (1871: 30, as Coptoderinae), generally accepted as in Handlirsch (1925: 547, as Coptoderini); incorrect stem formation, not in prevailing usage.
Mormolycidae Desmarest, 1851: 62 [stem: *Mormolyc-*]. Type genus: *Mormolyce* Hagenbach, 1825.
Thyréoptérides Chaudoir, 1869: 113 [stem: *Thyreopter-*]. Type genus: *Thyreopterus* Dejean, 1831. Comment: original vernacular name available (Art. 11.7.2): first used in latinized form by Bertkau (1878: 417, as Thyreopterini), generally accepted as in Jeannel (1950b: 165, as Thyreopteridae).
Eucheilinae H. W. Bates, 1883: 168 [stem: *Eucheil-*]. Type genus: *Eucheila* Dejean, 1829 [*Eucheila* is an incorrect subsequent spelling of the original spelling *Eucheyla*, in prevailing usage and so deemed to be the correct original spelling (Art. 33.3.1)].
Miscelini Sloane, 1907b: 473 [stem: *Miscel-*]. Type genus: *Miscelus* Klug, 1834.
Periglossiinae Liebke, 1929: 247 [stem: *Periglossi-*]. Type genus: *Periglossium* Liebke, 1929 [syn. of *Inna* Putzeys, 1861].
Catascopi Csiki, 1932b: 1352 [stem: *Catascop-*]. Type genus: *Catascopus* Kirby, 1823. Comment: name proposed after 1930 without description or bibliographic reference to such a description (Art. 13.1), however available because it was used as valid before 2000 as in Blackwelder (1944: 57) and was not rejected by an author who, between 1961 and 1999, applied Article 13 of the then current edition of the Code (see Art. 13.2.1).
Miscelini Jeannel, 1949c: 1006 [stem: *Miscel-*]. Type genus: *Miscelus* Klug, 1834. Comment: family-group name proposed as new without reference to Miscelini Sloane, 1907.
Lobodontini Jeannel, 1949c: 1007, in key [stem: *Lobodont-*]. Type genus: *Lobodontus* Chaudoir, 1842.
Thysanotini Jeannel, 1949c: 947, in key [stem: *Thysanot-*]. Type genus: *Thysanotus* Chaudoir, 1848.
Somotrichini Mateu, 1963: 125, in key [stem: *Somotrich-*]. Type genus: *Somotrichus* Seidlitz, 1887.

### Subtribe Pseudotrechina

Basilewsky, 1984

Pseudotrechini Basilewsky, 1984: 555 [stem: *Pseudotrech-*]. Type genus: *Pseudotrechus* Rosenhauer, 1856.

### Subtribe Sugimotoina

Habu, 1975

Sugimotoina Habu, 1975: 77 [stem: *Sugimoto-*]. Type genus: *Sugimotoa* Habu, 1975.

### Subtribe Trichina

Basilewsky, 1984

Trichini Basilewsky, 1984: 549 [stem: *Trich-*]. Type genus: *Trichis* Klug, 1832.

### Tribe Licinini

Bonelli, 1810

Licinii Bonelli, 1810: Tabula Synoptica [stem: *Licin-*]. Type genus: *Licinus* Latreille, 1802.

### Subtribe Dicaelina

Laporte, 1834

Dicoelidae Laporte, 1834b: 83 [stem: *Dicael-*]. Type genus: *Dicaelus* Bonelli, 1813 [as *Dicoelus*, incorrect subsequent spelling of type genus name, not in prevailing usage]. Comment: incorrect original stem formation, not in prevailing usage.
Rembidae Gistel, 1848: [2] [stem: *Remb-*]. Type genus: *Rembus* W. S. MacLeay, 1825 [preoccupied genus name, not *Rembus* Germar, 1824 [Coleoptera: Curculionidae]; syn. of *Diplocheila* Brullé, 1834]. Comment: permanently invalid (Art. 39): based on preoccupied type genus.
*Submerini Lafer, 1989: 205 [stem: *Submer-*]. Type genus: *Submera* Habu, 1956. Comment: unavailable family-group name, proposed after 1930 without description or bibliographic reference to such a description (Art. 13.1).

### Subtribe Dicrochilina

Ball, 1992

Dicrochilina Ball, 1992: 347 [stem: *Dicrochil-*]. Type genus: *Dicrochile* Guérin-Méneville, 1846.

### Subtribe Lestignathina

Ball, 1992

Lestignathina Ball, 1992: 347 [stem: *Lestignath-*]. Type genus: *Lestignathus* Erichson, 1842.

### Subtribe Licinina

Bonelli, 1810

Licinii Bonelli, 1810: Tabula Synoptica [stem: *Licin-*]. Type genus: *Licinus* Latreille, 1802.
Badistidae Gistel, 1856a: 357 [stem: *Badister-*]. Type genus: *Badister* Clairville, 1806 [as *Badistes*, unjustified emendation of type genus name by Agassiz (1846b: 42), not in prevailing usage]. Comment: incorrect original stem formation, not in prevailing usage.
Badistritae Jeannel, 1942: 999 [stem: *Badister-*]. Type genus: *Badister* Clairville, 1806 [as *Badistes*, unjustified emendation of type genus name by Agassiz (1846b: 42), not in prevailing usage]. Comment: family-group name proposed as new without reference to Badistidae Gistel, 1856; incorrect original stem formation, not in prevailing usage.

### Tribe Melanchitonini

Jeannel, 1948

Melanodini Alluaud, 1916: 228 [stem: *Melanod-*]. Type genus: *Melanodes* Chaudoir, 1876 [preoccupied type genus, not *Melanodes* Guenée, 1857 [Lepidoptera]; syn. of *Melanchiton* Basilewsky, 1946]. Comment: permanently invalid (Art. 39): based on preoccupied type genus.
Melanchitonitae Jeannel, 1948b: 627 [stem: *Melanchiton-*]. Type genus: *Melanchiton* Basilewsky, 1946. Comment: replacement name for Melanodini Alluaud, 1916.

### Tribe Microcheilini

Jeannel, 1948

Microchilitae Jeannel, 1948b: 616 [stem: *Microcheil-*]. Type genus: *Microcheila* Brullé, 1834 [as *Microchila*, unjustified emendation of type genus name by Agassiz (1846b: 233), not in prevailing usage]. Comment: incorrect original stem formation, not in prevailing usage.

### Tribe Morionini

Brullé, 1835

Morioniens Brullé, 1835: 36 [stem: *Morion-*]. Type genus: *Morion* Latreille, 1810. Comment: original vernacular name available (Art. 11.7.2): first used in latinized form by Hope (1838a: 109, as Morionidae [based on “*Morio* Latreille”, an incorrect subsequent spellling of the type genus name, see Madge (1989: 465)], generally accepted as in Lorenz (2005: 247, as Morionini).

### Tribe Odacanthini

Laporte, 1834

Odacanthidae Laporte, 1834b: 40 [stem: *Odacanth-*]. Type genus: *Odacantha* Paykull, 1798.
Casnoniae J. L. LeConte, 1861: 21 [stem: *Cosnani-*]. Type genus: *Cosnania* Dejean, 1821 [as *Casnonia*, incorrect subsequent spelling of type genus name, not in prevailing usage]. Comment: incorrect original stem formation, not in prevailing usage.
Colliurini Bedel, 1910: 72 [stem: *Colliur-*]. Type genus: *Colliuris* DeGeer, 1774.
Lasiocerini Jeannel, 1948b: 747, in key [stem: *Lasiocer-*]. Type genus: *Lasiocera* Dejean, 1831.

### Tribe Omphreini

Ganglbauer, 1891

Omphreini Ganglbauer, 1891b: 26 [stem: *Omphre-*]. Type genus: *Omphreus* Dejean, 1828.

### Tribe Oodini

LaFerté-Sénectère, 1851

Oodites LaFerté-Sénectère, 1851: 266 [stem: *Ood-*]. Type genus: *Oodes* Bonelli, 1810. Comment: original vernacular name available (Art. 11.7.2): first used in latinized form by Bedel (1879: 52, as Oodini), generally accepted as in Lorenz (2005: 324, as Oodini).
Thryptocerini Jeannel, 1949c: 841 [stem: *Thryptocer-*]. Type genus: *Thryptocerus* Chaudoir, 1878.
Sphaerodini Jeannel, 1949c: 829, in key [stem: *Sphoerod-*]. Type genus: *Sphoerodes* Chaudoir, 1883 [as *Sphaerodes*, incorrect subsequent spelling of type genus name, not in prevailaing usage]. Comment: incorrect original stem formation, not in prevailing usage.
Simoini Basilewsky, 1953a: 153 [stem: *Simo-*]. Type genus: *Simous* Chaudoir, 1882.

### Tribe Orthogoniini

Schaum, 1857

Orthogoniden Schaum, 1857b: 308 [stem: *Orthogoni-*]. Type genus: *Orthogonius* W. S. MacLeay, 1825. Comment: original vernacular name available (Art. 11.7.2): first used in latinized form by Schaum (1860: 774, as Orthogonini [incorrect stem formation]), generally accepted as in Lorenz (2005: 391, as Orthogoniinae); incorrect original stem formation, not in prevailing usage.

### Tribe Panagaeini

Bonelli, 1810

Panagaeides Bonelli, 1810: Tabula Synoptica [stem: *Panagae-*]. Type genus: *Panagaeus* Latreille, 1802.

### Subtribe Brachygnathina

Basilewsky, 1946

Brachygnathini Basilewsky, 1946: 7 [stem: *Brachygnath-*]. Type genus: *Brachygnathus* Perty, 1830.

### Subtribe Panagaeina

Bonelli, 1810

Panagaeides Bonelli, 1810: Tabula Synoptica [stem: *Panagae-*]. Type genus: *Panagaeus* Latreille, 1802.

### Subtribe Tefflina

Basilewsky, 1946

Tefflini Basilewsky, 1946: 7 [stem: *Teffl-*]. Type genus: *Tefflus* Leach, 1819.

### Tribe Peleciini

Chaudoir, 1880

Pélécides Chaudoir, 1880: 317 [stem: *Peleci-*]. Type genus: *Pelecium* Kirby, 1819.

### Subtribe Agonicina

Sloane, 1920

Agonicini Sloane, 1920: 129 [stem: *Agonic-*]. Type genus: *Agonica* Sloane, 1920.

### Subtribe Peleciina

Chaudoir, 1880

Pélécides Chaudoir, 1880: 317 [stem: *Peleci-*]. Type genus: *Pelecium* Kirby, 1819. Comment: original vernacular name available (Art. 11.7.2): first used in latinized form by G. H. Horn (1881: 170, as Peleciini), generally accepted as in Csiki (1932b: 1285, as Peleciini); incorrect original stem formation, not in prevailing usage.
Disphaericini Sloane, 1923a: 248 [stem: *Dispheric-*]. Type genus: *Disphericus* G. R. Waterhouse, 1842 [as *Disphaericus*, unjustified emendation of type genus name by Agassiz (1846b: 127), not in prevailing usage]. Comment: incorrect original stem formation, not in prevailing usage.

### Tribe Pentagonicini

Bates, 1873

Pentagonicinae H. W. Bates, 1873: 320 [stem: *Pentagonic-*]. Type genus: *Pentagonica* Schmidt-Göbel, 1846.
Scopodinae H. W. Bates, 1874b: 275 [stem: *Scopod-*]. Type genus: *Scopodes* Erichson, 1842.

### Tribe Perigonini

Horn, 1881 nomen protectum

Trechichinae H. W. Bates, 1873: 281 [stem: *Trechic-*]. Type genus: *Trechicus* J. L. LeConte, 1853 [as *Trechichus*, incorrect subsequent spelling of type genus name, not in prevailing usage; subgenus of *Perigona* Laporte, 1835]. Comment: *nomen oblitum* (see Appendix 1); incorrect original stem formation, not in prevailing usage.
Perigonae G. H. Horn, 1881: 143 [stem: *Perigon-*]. Type genus: *Perigona* Laporte, 1835. Comment: *nomen protectum* (see Appendix 1).

### Tribe Physocrotaphini

Chaudoir, 1863

Physocrotaphides Chaudoir, 1863: 303 [stem: *Physocrotaph-*]. Type genus: *Physocrotaphus* Parry, 1849. Comment: original vernacular name available (Art. 11.7.2): first used in latinized form by H. W. Bates (1892: 388, as Physocrotaphinae), generally accepted as in B. P. Moore (1998: 369, as Physocrotaphini).
Helluodini Csiki, 1932b: 1571 [stem: *Helluod-*]. Type genus: *Helluodes* Westwood, 1847. Comment: name proposed after 1930 without description or bibliographic reference to such a description (Art. 13.1), however available because it was used as valid before 2000 as in Darlington (1968: 222) and was not rejected by an author who, between 1961 and 1999, applied Article 13 of the then current edition of the Code (see Art. 13.2.1).

### Tribe Platynini

Bonelli, 1810

Platynii Bonelli, 1810: Tabula Synoptica [stem: *Platyn-*]. Type genus: *Platynus* Bonelli, 1810. Comment: First Reviser found (Platynina Bonelli, 1810 vs Anchomenina Bonelli, 1810) is Jakobson (1906: 317).
Anchomenii Bonelli, 1810: Tabula Synoptica [stem: *Anchomen-*]. Type genus: *Anchomenus* Bonelli, 1810.
Sericodiadae Kirby, 1837: 14 [stem: *Sericod-*]. Type genus: *Sericoda* Kirby, 1837.
Agonidae Kirby, 1837: 23 [stem: *Agonum-*]. Type genus: *Agonum* Bonelli, 1810 [placed on the Official List of Generic Names in Zoology (ICZN 1996d)]. Comment: senior homonym of Agonidae Swainson, 1839 (type genus *Agonus* Bloch and Schneider, 1801) in Pisces; Agonidae Kirby, 1837 placed on the Official Index of Rejected and Invalid Family-Group Names in Zoology (ICZN 1996d), stem emended to *Agonum*- and Agonumidae Kirby, 1837 placed on the Official List of Family-Group Names in Zoology (ICZN 1996d).
Colpodidas Chaudoir, 1872a: 285 [stem: *Colpod-*]. Type genus: *Colpodes* W. S. MacLeay, 1825. Comment: the original spelling Colpodidas is the accusative (Latin) form of and Colpodidae was recognized as such by Madge (1989: 463); Colpodidae Poche, 1913 (type genus *Colpoda* Müller, 1773) has been used as valid in Protista as recently as in 2006; this case is to be referred to the Commission to remove the homonymy (Art. 55.3.1).
Agelaeina Jakobson, 1907: 334 [stem: *Agelae-*]. Type genus: *Agelaea* Gené, 1839.
Prosphodrini J. M. Valentine, 1987: 74 [stem: *Prosphodr-*]. Type genus: *Prosphodrus* Britton, 1959.
Meleagrosini Morvan, 2004: 2 [stem: *Meleagr-*]. Type genus: *Meleagros* Kirschenhofer, 1999. Comment: incorrect original stem formation, not in prevailing usage.

### Tribe Pseudomorphini

Hope, 1838

Heteromorphidae Hope, 1838a: 108 [stem: *Pseudomorph-*]. Type genus: *Pseudomorpha* Kirby, 1823 [as *Heteromorpha*, incorrect original spelling of type genus name, not in prevailing usage]. Comment: this family-group name is based on an incorrect original spelling of the name of its type genus (*Heteromorpha*) and must be corrected (ICZN 1999a: Article 35.4.1); Kirby (1823a) originally used two different names for the type genus, *Pseudomorpha* (in the description on p. 98) and *Heteromorpha* (in the explanation of the figures on p. 109). Both names are considered different original spellings of the same name. Kirby reissued the paper later the same year in another journal, without the plate, and so only *Pseudomorpha* was used; as such, Kirby (1823b) is the “First Reviser” and *Pseudomorpha* is the correct original spelling (ICZN 1999a: Article 24.2.4), therefore Heteromorphidae Hope, 1838 is corrected to Pseudomorphidae Hope, 1838.

### Tribe Pterostichini

Bonelli, 1810

Pterostichii Bonelli, 1810: Tabula Synoptica [stem: *Pterostich-*]. Type genus: *Pterostichus* Bonelli, 1810.

### Subtribe Abacomorphina

Tschitschérine, 1902

Abacomorphini Tschitschérine, 1902: 507 [stem: *Abacomorph-*]. Type genus: *Abacomorphus* Chaudoir, 1878. Comment: First Reviser (Abacomorphina Tschitschérine, 1902 vs Sphodrosomina Tschitschérine, 1902) not determined, current usage maintained.
Sphodrosomini Tschitschérine, 1902: 507 [stem: *Sphodrosom-*]. Type genus: *Sphodrosomus* Perroud and Montrouzier, 1864.

### Subtribe Euchroina

Chaudoir, 1874

Euchroides Chaudoir, 1874: 16 [stem: *Euchro-*]. Type genus: *Euchroa* Brullé, 1834. Comment: original vernacular name available (Art. 11.7.2): first used in latinized form by Tschitschérine (1899: 84, as Euchroini), generally accepted as in Frania and Ball (2007: 12, as Euchroina).

### Subtribe Metiina

Straneo, 1951

Antarctiides Lacordaire, 1854a: 336 [stem: *Antarcti-*]. Type genus: *Antarctia* Dejean, 1828 [preoccupied genus name, not *Antarctia* Hübner, 1820 [Lepidoptera]; syn. of *Metius* Curtis, 1838]. Comment: original vernacular name available (Art. 11.7.2): first used in latinized form by Reed (1874: 58, as Antarctiinae), generally accepted as in Jeannel (1942: 734, as Antarctiitae); permanently invalid (Art. 39): based on preoccupied type genus.
Metiini Straneo, 1951: 56 [stem: *Meti-*]. Type genus: *Metius* Curtis, 1838.
Kuschelinini Jeannel, 1967: 437 [stem: *Kuschelin-*]. Type genus: *Kuschelinus* Straneo, 1963.

### Subtribe Microcephalina

Tschitschérine, 1898

Microcephalides Tschitschérine, 1898: 46 [stem: *Microcephal-*]. Type genus: *Microcephalus* Dejean, 1828. Comment: the older name Microcephali Latreille, 1825: 345 is unavailable (Art. 11.7.1.1) because it is not based on an available generic name [descriptive name for a group of Staphylinidae including *Tachyporus* and other genera with relatively small heads].
Tichoniini Semenov, 1904a: 201 [stem: *Tichoni-*]. Type genus: *Tichonia* Semenov, 1904 [preoccupied genus name, not *Tichonia* Hübner, 1826 [Lepidoptera]; syn. of *Microcephalus* Dejean, 1828]. Comment: replacement name for Microcephalini Tschitschérine, 1898 because of the homonymy of the type genus; permanently invalid (Art. 39): based on preoccupied type genus.
Tichonillina Emden, 1958: 24 [stem: *Tichonill-*]. Type genus: *Tichonilla* Strand, 1942 [syn. of *Microcephalus* Dejean, 1828].

### Subtribe Pterostichina

Bonelli, 1810

Pterostichii Bonelli, 1810: Tabula Synoptica [stem: *Pterostich-*]. Type genus: *Pterostichus* Bonelli, 1810. Comment: First Reviser (Pterostichina Bonelli, 1810 vs Poecilina Bonelli, 1810 vs Molopina Bonelli, 1810) not determined, current usage maintained.
Poecilii Bonelli, 1810: Tabula Synoptica [stem: *Poecil-*]. Type genus: *Poecilus* Bonelli, 1810.
Molopides Bonelli, 1810: Tabula Synoptica [stem: *Molop-*]. Type genus: *Molops* Bonelli, 1810.
Féroniens Dejean, 1825: 3 [stem: *Feroni-*]. Type genus: *Feronia* Latreille, 1816 [syn. of *Poecilus* Bonelli, 1810]. Comment: original vernacular name available (Art. 11.7.2): first used in latinized form by Thon (1827: 15, as Feroniae), generally accepted as in Schaum (1857a: 136, as Feronidae).
Trogonotomidae Laporte, 1834b: 75 [stem: *Trigonotom-*]. Type genus: *Trigonotoma* Dejean, 1828. Comment: the original spelling was Trogonotomidae, but this was apparently a typographical error since both the vernacular spelling (Trigonotomites) and the genus name (*Trigonotoma*) were spelled with the stem “*Trigonotom*-” on the same page.
Catadromiens Brullé, 1834: 277, 328 [stem: *Catadrom-*]. Type genus: *Catadromus* W. S. MacLeay, 1825. Comment: original vernacular name available (Art. 11.7.2): first used in latinized form and generally accepted as in Desmarest (1851: 143, as Catadromidae).
Thaliadae Hope, 1838a: 71 [stem: *Thali-*]. Type genus: *Thalia* Hope, 1838 [preoccupied genus name, not *Thalia* Blumenbach, 1827 [Tunicata]; syn. of *Poecilus* Bonelli, 1810]. Comment: permanently invalid (Art. 39): based on preoccupied type genus.
Stomidae Chaudoir, 1846: 514 [stem: *Stomid-*]. Type genus: *Stomis* Clairville, 1806. Comment: incorrect original stem formation, not in prevailing usage.
Rhathyminae H. W. Bates, 1891b: cccxxxiv [stem: *Rathym-*]. Type genus: *Rathymus* Dejean, 1831 [as *Rhathymus*, unjustified emendation of type genus name by Agassiz (1846b: 321), not in prevailing usage]. Comment: incorrect original stem formation, not in prevailing usage.
Trigonognathides Tschitschérine, 1898: 66 [stem: *Trigonognath-*]. Type genus: *Trigonognatha* Motschulsky, 1858.
Platysmatini Tschitschérine, 1899: 84 [stem: *Platysmat-*]. Type genus: *Platysma* Bonelli, 1810.
Cyrtoderini Tschitschérine, 1902: 506 [stem: *Cyrtoder-*]. Type genus: *Cyrtoderus* Hope, 1842.
Darodiliini Tschitschérine, 1902: 506 [stem: *Darodili-*]. Type genus: *Darodilia* Laporte, 1867.
Deliniini Tschitschérine, 1902: 506 [stem: *Delini-*]. Type genus: *Delinius* Westwood, 1864.
Cyphosomatini Tschitschérine, 1902: 507 [stem: *Cyphosomat-*]. Type genus: *Cyphosoma* Hope, 1842 [preoccupied genus name, not *Cyphosoma* Mannerheim, 1837 [Coleoptera: Buprestidae]; syn. of *Cratogaster* Blanchard, 1853]. Comment: permanently invalid (Art. 39): based on preoccupied type genus.
Myadina Jakobson, 1907: 334 [stem: *Myad-*]. Type genus: *Myas* Sturm, 1826.
Cratogastri Csiki, 1929: 524 [stem: *Cratogastr-*]. Type genus: *Cratogaster* Blanchard, 1843.
Abaxini Schuler, 1970: 115 [stem: *Abac-*]. Type genus: *Abax* Bonelli, 1810. Comment: incorrect original stem formation, not in prevailing usage.
Aristochroodini Sciaky, 1996: 437 [stem: *Aristochrood-*]. Type genus: *Aristochroodes* Marcilhac, 1993.

### Tribe Sphodrini

Laporte, 1834

Sphodridae Laporte, 1834b: 78 [stem: *Sphodr-*]. Type genus: *Sphodrus* Clairville, 1806. Comment: published before 9 August 1834; First Reviser found (Sphodrini Laporte, 1834 vs Calathini Laporte, 1834) is Habu (1978: 296).

### Subtribe Atranopsina

Baehr, 1982

Atranopsina Baehr, 1982: 265 [stem: *Atranops-*]. Type genus: *Atranopsis* Baehr, 1982. Comment: current spelling maintained (Art. 29.5): incorrect stem formation in prevailing usage (should be *Atranopse*-).
*Platyderina Baehr, 1982: 265 [stem: *Platyder-*]. Type genus: *Platyderes* Stephens, 1827. Comment: unavailable family-group name, proposed after 1930 without description or bibliographic reference to such a description (Art. 13.1).

### Subtribe Calathina

Laporte, 1834

Calathidae Laporte, 1834b: 71 [stem: *Calath-*]. Type genus: *Calathus* Bonelli, 1810.

### Subtribe Dolichina

Brullé, 1834

Dolichiens Brullé, 1834: 295 [stem: *Dolich-*]. Type genus: *Dolichus* Bonelli, 1810. Comment: original vernacular name available (Art. 11.7.2): first used in latinized form by Hope (1838a: 72, as Dolichidae), generally accepted as in Lorenz (2005: 399, as Dolichina); published “31 Dec” 1834, therefore Sphodrini Laporte, 1834 and Calathini Laporte, 1834 have priority over this name for the tribe name.

### Subtribe Pristosiina

Lindroth, 1956

Pristosiae Lindroth, 1956: 489, in key [stem: *Pristosi-*]. Type genus: *Pristosia* Motschulsky, 1865.
*Eucalathi Lindroth, 1956: 489, in key [stem: *Eucalath-*]. Type genus: *Eucalathus* H. W. Bates, 1883 [syn. of *Pristosia* Motschulsky, 1865]. Comment: family-group name unavailable (Art. 11.6): originally published as synonym and not made available subsequently.

### Subtribe Sphodrina

Laporte, 1834

Sphodridae Laporte, 1834b: 78 [stem: *Sphodr-*]. Type genus: *Sphodrus* Clairville, 1806.
Pristonychidae Gistel, 1848: [2] [stem: *Pristonych-*]. Type genus: *Pristonychus* Dejean, 1828.
*Laemostenina Baehr, 1982: 265 [stem: *Laemosten-*]. Type genus: *Laemostenus* Bonelli, 1810. Comment: unavailable family-group name, proposed after 1930 without description or bibliographic reference to such a description (Art. 13.1).

### Subtribe Synuchina

Lindroth, 1956

Synuchi Lindroth, 1956: 489, in key [stem: *Synuch-*]. Type genus: *Synuchus* Gyllenhal, 1810.

### Tribe Xenaroswellianini

Erwin, 2007

Xenaroswellanini Erwin, 2007: 563 [stem: *Xenaroswellian-*]. Type genus: *Xenaroswelliana* Erwin, 2007. Comment: the original spelling Xenaroswellanini is considered a *lapsus calami* and the stem is corrected to *Xenaroswellian*- here.

### Tribe Zabrini

Bonelli, 1810

Zabrides Bonelli, 1810: Tabula Synoptica [stem: *Zabr-*]. Type genus: *Zabrus* Clairville, 1806.

### Subtribe Amarina

Zimmermann, 1832

Amaroiden C. Zimmermann, 1832: 6 [stem: *Amar-*]. Type genus: *Amara* Bonelli, 1810. Comment: original vernacular name available (Art. 11.7.2): first used in latinized form by Laporte (1834b: 78, as Amaridae), generally accepted as in Hieke (2003: 547, as Amarina).
Isopleuridae Kirby, 1837: 49 [stem: *Isopleur-*]. Type genus: *Isopleurus* Kirby, 1837 [syn. of *Celia* C. Zimmermann, 1832].
Agronomaeidae Gistel, 1848: [2] [stem: *Agronom-*]. Type genus: *Agronoma* Gistel, 1848 [*Carabus familiaris* Duftschmid, 1812, one of the species originally included, is here chosen as type species of *Agronoma* Gistel, 1848; **syn. nov.** of *Amara* Bonelli, 1810]. Comment: **syn. nov.**; incorrect original stem formation, not in prevailing usage.
Pangeteidae Gistel, 1856a: 358 [stem: *Panget-*]. Type genus: *Pangetes* Gistel, 1856 [syn. of *Amara* Bonelli, 1810]. Comment: incorrect original stem formation, not in prevailing usage.

### Subtribe Zabrina

Bonelli, 1810

Zabrides Bonelli, 1810: Tabula Synoptica [stem: *Zabr-*]. Type genus: *Zabrus* Clairville, 1806.

### Tribe Zuphiini

Bonelli, 1810

Zuphietae Bonelli, 1810: Tabula Synoptica [stem: *Zuphi-*]. Type genus: *Zuphium* Latreille, 1806.

### Subtribe Dicrodontina

Machado, 1992

Dicrodontini Machado, 1992: 569 [stem: *Dicrodont-*]. Type genus: *Dicrodontus* Chaudoir, 1872.

### Subtribe Leleupidiina

Basilewsky, 1951

Leleupidiini Basilewsky, 1951a: 178 [stem: *Leleupidi-*]. Type genus: *Leleupidia* Basilewsky, 1951.

### Subtribe Metazuphiina

Mateu, 1992

Metazuphiina Mateu, 1992: 198, in key [stem: *Metazuphi-*]. Type genus: *Metazuphium* Mateu, 1992.

### Subtribe Mischocephalina

Mateu, 1992

Mischocephalina Mateu, 1992: 198, in key [stem: *Mischocephal-*]. Type genus: *Mischocephalus* Chaudoir, 1863. Comment: this name was incorrectly spelled Mischocephaliina in the key on page 198 but correctly spelled Mischocephalina on p. 203 of the same publication.

### Subtribe Patriziina

Basilewsky, 1953

Patriziini Basilewsky, 1953b: 266 [stem: *Patrizi-*]. Type genus: *Patrizia* Alluaud, 1931.

### Subtribe Zuphiina

Bonelli, 1810

Zuphietae Bonelli, 1810: Tabula Synoptica [stem: *Zuphi-*]. Type genus: *Zuphium* Latreille, 1806.
*Polystichides Chaudoir, 1863: 308 [stem: *Polistich-*]. Type genus: *Polistichus* Bonelli, 1810 [as *Polystichus*, unjustified emendation of type genus name by Agassiz (1846b: 301), not in prevailing usage]. Comment: original vernacular name unavailable (Art. 11.7.2): subsequently used in latinized form but not generally attributed to Chaudoir (1863); incorrect original spelling of family-group name, not in prevailing usage.
Polystichinae H. W. Bates, 1886: 199 [stem: *Polistich-*]. Type genus: *Polistichus* Bonelli, 1810 [as *Polystichus*, unjustified emendation of type genus name by Agassiz (1846b: 301), not in prevailing usage]. Comment: incorrect original stem formation, not in prevailing usage.

### Family Haliplidae

Aubé, 1836

Haliplides Aubé, 1836: 15 [stem: *Halipl-*]. Type genus: *Haliplus* Latreille, 1802. Comment: original vernacular name available (Art. 11.7.2): first used in latinized form by Erichson (1837: 183, as Haliplini), generally accepted as in Lawrence and Newton (1995: 807, as Haliplidae).
Peltodytinae Böving and Craighead, 1931: 17 [stem: *Peltodyt-*]. Type genus: *Peltodytes* Régimbart, 1879.
Brychiini Ádám, 1996: 15 [stem: *Brychi-*]. Type genus: *Brychius* C. G. Thomson, 1859.

### †Family Triaplidae

Ponomarenko, 1977

Triaplidae Ponomarenko, 1977: 17 [stem: *Triapl-*]. Type genus: *Triaplus* Ponomarenko, 1977.

### †Family Colymbotethidae

Ponomarenko, 1994

Colymbotethidae Ponomarenko, 1994: 188 [stem: *Colymboteth-*]. Type genus: *Colymbotethis* Ponomarenko, 1994.

### †Family Parahygrobiidae

Ponomarenko, 1977

Parahygrobiidae Ponomarenko, 1977: 19 [stem: *Parahygrobi-*]. Type genus: *Parahygrobia* Ponomarenko, 1977.

### †Family Coptoclavidae

Ponomarenko, 1961

Coptoclavidae Ponomarenko, 1961: 68 [stem: *Coptoclav-*]. Type genus: *Coptoclava* Ping, 1928.

### †Subfamily Necronectinae

Ponomarenko, 1977

Necronectinae Ponomarenko, 1977: 22 [stem: *Necronect-*]. Type genus: *Necronectes* Ponomarenko, 1977 [syn. of *Timarchopsis* Brauer, Redtenbacher and Ganglbauer, 1889].

### †Subfamily Charonoscaphinae

Ponomarenko, 1977

Charonoscaphinae Ponomarenko, 1977: 32 [stem: *Charonoscaph-*]. Type genus: *Charonoscapha* Ponomarenko, 1977.

### †Subfamily Coptoclavinae

Ponomarenko, 1961

Coptoclavidae Ponomarenko, 1961: 68 [stem: *Coptoclav-*]. Type genus: *Coptoclava* Ping, 1928.

### †Subfamily Coptoclaviscinae

Soriano, Ponomarenko and Delclos, 2007

Coptoclaviscinae Soriano et al., 2007: 532 [stem: *Coptoclavisc-*]. Type genus: *Coptoclavisca* Ponomarenko, 1987.

### †Subfamily Hispanoclavinae

Soriano, Ponomarenko and Delclos, 2007

Hispanoclavinae Soriano et al., 2007: 527 [stem: *Hispanoclav-*]. Type genus: *Hispanoclava* Soriano, Ponomarenko and Delclos, 2007.

### †Family Liadytidae

Ponomarenko, 1977

Liadytidae Ponomarenko, 1977: 37 [stem: *Liadyt-*]. Type genus: *Liadytes* Ponomarenko, 1977.

### Family Meruidae

Spangler and Steiner, 2005

Meruidae Spangler and Steiner, 2005: 351 [stem: *Meru-*]. Type genus: *Meru* Spangler and Steiner, 2005.

### Family Noteridae

Thomson, 1860

Noterides C. G. Thomson, 1860: 34 [stem: *Noter-*]. Type genus: *Noterus* Clairville, 1806.

### Subfamily Noterinae

Thomson, 1860

Noterides C. G. Thomson, 1860: 34 [stem: *Noter-*]. Type genus: *Noterus* Clairville, 1806.

### Tribe Neohydrocoptini

Zalat, Saleh, Angus and Kaschef, 2000

Neohydrocoptini Zalat et al., 2000: 11 [stem: *Neohydrocopt-*]. Type genus: *Neohydrocoptus* Satô, 1972.

### Tribe Noterini

Thomson, 1860

Noterides C. G. Thomson, 1860: 34 [stem: *Noter-*]. Type genus: *Noterus* Clairville, 1806.
Hydrocanthini Sharp, 1880: cxlviii [stem: *Hydrocanth-*]. Type genus: *Hydrocanthus* Say, 1823.
Suphisini Sharp, 1880: cxlviii [stem: *Suphid-*]. Type genus: *Suphis* Aubé, 1836. Comment: incorrect original stem formation, not in prevailing usage.

### Tribe Pronoterini

Nilsson, 2005

Pronoterini Nilsson, 2005: 90 [stem: *Pronoter-*]. Type genus: *Pronoterus* Sharp, 1882.

### Tribe Tonerini

Miller, 2009

Tonerini K. B. Miller, 2009: 201 [stem: *Toner-*]. Type genus: *Tonerus* K. B. Miller, 2009.

### Subfamily Notomicrinae

Zimmermann, 1919

Notomicrini A. Zimmermann, 1919: 110 [stem: *Notomicr-*]. Type genus: *Notomicrus* Sharp, 1882.

### Subfamily Phreatodytinae

Uéno, 1957

Phreatodytidae Uéno, 1957: 251 [stem: *Phreatodyt-*]. Type genus: *Phreatodytes* Uéno, 1957.

### Family Amphizoidae

LeConte, 1853

Amphizoidae J. L. LeConte, 1853a: 227 [stem: *Amphizo-*]. Type genus: *Amphizoa* J. L. LeConte, 1853.

### Family Aspidytidae

Ribera, Beutel, Balke and Vogler, 2002

Aspidytidae Ribera et al., 2002: 2354 [stem: *Aspidyt-*]. Type genus: *Aspidytes* Ribera, Beutel, Balke and Vogler, 2002.

### Family Hygrobiidae

Régimbart, 1879 (1837)

Pelobiinae Erichson, 1837: 182 [stem: *Paelobi-*]. Type genus: *Paelobius* Schönherr, 1808 [as *Pelobius*, unjustified emendation of type genus name by Erichson (1832: 45); syn. of *Hygrobia* Latreille, 1804]. Comment: use of younger family-group name Hygrobiidae Régimbart, 1879 conserved (Art. 40.2) (see Lawrence and Newton 1995: 808); incorrect original stem formation, not in prevailing usage.
Hygrobiinae Régimbart, 1879: 449 [stem: *Hygrobi-*]. Type genus: *Hygrobia* Latreille, 1804 [the original spelling of the type genus, *Hygriobia*, has been corrected to *Hygrobia* and *Hygrobia* Latreille, 1804 placed on the Official List of Generic Names in Zoology (ICZN 1954)]. Comment: use of this name conserved over the older name Paelobiidae Erichson, 1837 (Art. 40.2) (see Lawrence and Newton 1995: 808).

### Family Dytiscidae

Leach, 1815

Dyticides Leach, 1815: 84 [stem: *Dytisc-*]. Type genus: *Dytiscus* Linnaeus, 1758 [placed on the Official List of Generic Names in Zoology (ICZN 1961e)]. Comment: name placed on the Official List of Family-Group Names in Zoology (ICZN 1961e, as Dytiscidae Leach, 1817).

### Subfamily Agabinae

Thomson, 1867

Agabides C. G. Thomson, 1867: 84 [stem: *Agab-*]. Type genus: *Agabus* Leach, 1817.
Agabinini Crotch, 1873d: 385 [stem: *Agabin-*]. Type genus: *Agabinus* Crotch, 1873 [syn. of *Platambus* C. G. Thomson, 1859].
Ilybii Acloque, 1896: 81 [stem: *Ilybi-*]. Type genus: *Ilybius* Erichson, 1832.
Hydronebriini Guignot, 1948: 168, in key [stem: *Hydronebri-*]. Type genus: *Hydronebrius* Jakovlev, 1897. Comment: printed in the issue of November 1948; this family-group name was also used in the same year by C. Brinck (1948 [December]: 112, as Hydronebriini).
Hydrotrupinae Roughley, 2000: 52 [stem: *Hydrotrup-*]. Type genus: *Hydrotrupes* Sharp, 1882.

### Subfamily Colymbetinae

Erichson, 1837

Colymbetini Erichson, 1837: 149 [stem: *Colymbet-*]. Type genus: *Colymbetes* Clairville, 1806.

### Tribe Anisomeriini

Brinck, 1948

Anisomeriini C. Brinck, 1948: 112 [stem: *Anisomeri-*]. Type genus: *Anisomeria* C. Brinck, 1943.

### Tribe Carabdytini

Pederzani, 1995

Carabdytini Pederzani, 1995: 45, in key [stem: *Carabdyt-*]. Type genus: *Carabdytes* Balke, Hendrich and Wewalka, 1992.

### Tribe Colymbetini

Erichson, 1837

Colymbetini Erichson, 1837: 149 [stem: *Colymbet-*]. Type genus: *Colymbetes* Clairville, 1806.
Cymatopterini Portevin, 1929: 198, in key [stem: *Cymatopter-*]. Type genus: *Cymatopterus* Dejean, 1833 [syn. of *Colymbetes* Clairville, 1806].

### Subfamily Copelatinae

Branden, 1885

Copelatini Branden, 1885: 82 [stem: *Copelat-*]. Type genus: *Copelatus* Erichson, 1832. Comment: First Reviser (Copelatini Branden, 1885 vs Aglymbini Branden, 1885 vs Lacconectini Branden, 1885) not determined, current usage maintained.
Aglymbini Branden, 1885: 87 [stem: *Aglymb-*]. Type genus: *Aglymbus* Sharp, 1882.
Lacconectini Branden, 1885: 87 [stem: *Lacconect-*]. Type genus: *Lacconectus* Motschulsky, 1855.

### Subfamily Coptotominae

Branden, 1885

Coptotomini Branden, 1885: 88 [stem: *Coptotom-*]. Type genus: *Coptotomus* Say, 1830.
Coptotomini C. Brinck, 1948: 112, 116 [stem: *Coptotom-*]. Type genus: *Coptotomus* Say, 1830. Comment: family-group name proposed as new without reference to Coptotomini Branden, 1885.

### Subfamily Dytiscinae

Leach, 1815

Dyticides Leach, 1815: 84 [stem: *Dytisc-*]. Type genus: *Dytiscus* Linnaeus, 1758 [placed on the Official List of Generic Names in Zoology (ICZN 1961e)]. Comment: name placed on the Official List of Family-Group Names in Zoology (ICZN 1961e, as Dytiscidae Leach, 1817).

### Tribe Aciliini

Thomson, 1867

Aciliides C. G. Thomson, 1867: 84 [stem: *Acili-*]. Type genus: *Acilius* Leach, 1817 [placed on the Official List of Generic Names in Zoology (ICZN 1961e)].
Thermonectini Sharp, 1880: cl [stem: *Thermonect-*]. Type genus: *Thermonectus* Dejean, 1833.
Graphoderini Houlbert, 1934: 126 [stem: *Graphoder-*]. Type genus: *Graphoderus* Dejean, 1833 [placed on the Official List of Generic Names in Zoology (ICZN 1961d)].

### Tribe Aubehydrini

Guignot, 1942

Aubehydrinae Guignot, 1942: 11 [stem: *Aubehydr-*]. Type genus: *Aubehydrus* Guignot, 1942 [syn. of *Notaticus* A. Zimmermann, 1928].

### Tribe Cybisterini

Sharp, 1880

Cybistrini Sharp, 1880: cl [stem: *Cybister-*]. Type genus: *Cybister* Curtis, 1827. Comment: incorrect original stem formation, not in prevailing usage.

### Tribe Dytiscini

Leach, 1815

Dyticides Leach, 1815: 84 [stem: *Dytisc-*]. Type genus: *Dytiscus* Linnaeus, 1758 [placed on the Official List of Generic Names in Zoology (ICZN 1961e)]. Comment: incorrect original stem formation, not in prevailing usage; name placed on the Official List of Family-Group Names in Zoology (ICZN 1961e, as Dytiscidae Leach, 1817).

### Tribe Eretini

Crotch, 1873

Eretini Crotch, 1873d: 385 [stem: *Eret-*]. Type genus: *Eretes* Laporte, 1833.
Eunectini Portevin, 1929: 208 [stem: *Eunect-*]. Type genus: *Eunectes* Erichson, 1832 [preoccupied genus name, not *Eunectes* Wagler, 1830 [Reptilia]; syn. of *Eretes* Laporte, 1833]. Comment: permanently invalid (Art. 39): based on preoccupied type genus.

### Tribe Hydaticini

Sharp, 1880

Hydaticini Sharp, 1880: cl [stem: *Hydatic-*]. Type genus: *Hydaticus* Leach, 1817.

### Tribe Hyderodini

Miller, 2000

Hyderodini K. B. Miller, 2000: 175 [stem: *Hyderod-*]. Type genus: *Hyderodes* Hope, 1838.

### Subfamily Hydrodytinae

Miller, 2001

Hydrodytinae K. B. Miller, 2001: 76 [stem: *Hydrodyt-*]. Type genus: *Hydrodytes* K. B. Miller, 2001.

### Subfamily Hydroporinae

Aubé, 1836

Hydroporides Aubé, 1836: 15 [stem: *Hydropor-*]. Type genus: *Hydroporus* Clairville, 1806.

### Tribe Bidessini

Sharp, 1880

Bidessini Sharp, 1880: cxlviii [stem: *Bidess-*]. Type genus: *Bidessus* Sharp, 1882.

### Tribe Carabhydrini

Watts, 1978

Carabhydrini Watts, 1978: 26 [stem: *Carabhydr-*]. Type genus: *Carabhydrus* Watts, 1978.

### Tribe Hydroporini

Aubé, 1836

Hydroporides Aubé, 1836: 15 [stem: *Hydropor-*]. Type genus: *Hydroporus* Clairville, 1806. Comment: original vernacular name available (Art. 11.7.2): first used in latinized form by Erichson (1837: 166, as Hydroporini), generally accepted as in Nilsson (2003: 54, as Hydroporinae).
Hydrocoptini Branden, 1885: 13 [stem: *Hydrocopt-*]. Type genus: *Hydrocoptus* Motschulsky, 1853 [syn. of *Hydroporus* Clairville, 1806].
Sternopriscini Branden, 1885: 38 [stem: *Sternoprisc-*]. Type genus: *Sternopriscus* Sharp, 1882.
Siettitiini Smrz, 1982: 289 [stem: *Siettiti-*]. Type genus: *Siettitia* Abeille de Perrin, 1904.
Deronectini Galewski, 1994: 98, in key [stem: *Deronect-*]. Type genus: *Deronectes* Sharp, 1882.

### Tribe Hydrovatini

Sharp, 1880

Hydrovatini Sharp, 1880: cxlviii [stem: *Hydrovat-*]. Type genus: *Hydrovatus* Motschulsky, 1853.

### Tribe Hygrotini

Portevin, 1929

Hygrotini Portevin, 1929: 180 [stem: *Hygrot-*]. Type genus: *Hygrotus* Stephens, 1828.

### Tribe Hyphydrini

Gistel, 1848

Hyphydriidae Gistel, 1848: [2] [stem: *Hyphydr-*]. Type genus: *Hyphydrus* Illiger, 1802. Comment: incorrect original stem formation, not in prevailing usage.
Actobaenidae Gistel, 1856a: 355 [stem: *Actobaen-*]. Type genus: *Actobaena* Gistel, 1856 [syn. of *Hyphydrus* Illiger, 1802].
Pachydrini Biström et al., 1997: 66 [stem: *Pachydr-*]. Type genus: *Pachydrus* Sharp, 1882.

### Tribe Laccornini

Wolfe and Roughley, 1990

Laccornini Wolfe and Roughley, 1990: 302 [stem: *Laccorn-*]. Type genus: *Laccornis* Gozis, 1914. Comment: current spelling maintained (Art. 29.5): incorrect stem formation in prevailing usage (should be *Laccornith*-).

### Tribe Methlini

Branden, 1885

Methlidae Branden, 1885: 65 [stem: *Methl-*]. Type genus: *Methles* Sharp, 1882. Comment: precedence (Methlini Branden, 1885 vs Celinini Branden, 1885) given to taxon originally proposed at the higher rank (Art. 24.1).
Celinini Branden, 1885: 65 [stem: *Celin-*]. Type genus: *Celina* Aubé, 1837.

### †Tribe Schistomerini

Palmer, 1957

Schistomerini Palmer, 1957: 259 [stem: *Schistomer-*]. Type genus: *Schistomerus* Palmer, 1957.

### Tribe Vatellini

Sharp, 1880

Vatellini Sharp, 1880: cxlviii [stem: *Vatell-*]. Type genus: *Vatellus* Aubé, 1837 [placed on the Official List of Generic Names in Zoology (ICZN 1992)].

### Subfamily Laccophilinae

Gistel, 1848

Laccophilidae Gistel, 1848: [1] [stem: *Laccophil-*]. Type genus: *Laccophilus* Leach, 1815.

### Tribe Agabetini

Branden, 1885

Agabetini Branden, 1885: 87 [stem: *Agabet-*]. Type genus: *Agabetes* Crotch, 1873.

### Tribe Laccophilini

Gistel, 1848

Laccophilidae Gistel, 1848: [1] [stem: *Laccophil-*]. Type genus: *Laccophilus* Leach, 1817.

### Subfamily Lancetinae

Branden, 1885

Lancetini Branden, 1885: 88 [stem: *Lancet-*]. Type genus: *Lancetes* Sharp, 1882.

### Subfamily Matinae

Branden, 1885

Matini Branden, 1885: 88 [stem: *Mat-*]. Type genus: *Matus* Aubé, 1836.

### †Subfamily Palaeogyrininae

Schlechtendal, 1894

Palaeogyrinidae Schlechtendal, 1894: 200 [stem: *Palaeogyrin-*]. Type genus: *Palaeogyrinus* Schlechtendal, 1894.

### †Subfamily Liadytiscinae

Prokin and Ren, 2010

Liadytiscinae Prokin and Ren, 2010: 48 [stem: *Liadytisc-*]. Type genus: *Liadytiscus* Prokin and Ren, 2010.

### Suborder POLYPHAGA

### Series STAPHYLINIFORMIA

### Superfamily Hydrophiloidea

Latreille, 1802

Hydrophilii Latreille, 1802: 136 [stem: *Hydrophil-*]. Type genus: *Hydrophilus* Geoffroy, 1762 [placed on the Official List of Generic Names in Zoology (ICZN 1994a)]. Comment: First Reviser (Hydrophiloidea Latreille, 1802 vs Sphaeridioidea Latreille, 1802) not determined, current usage maintained.

### Family Hydrophilidae

Latreille, 1802

Hydrophilii Latreille, 1802: 136 [stem: *Hydrophil-*]. Type genus: *Hydrophilus* Geoffroy, 1762 [placed on the Official List of Generic Names in Zoology (ICZN 1994a)]. Comment: First Reviser (Hydrophilidae Latreille, 1802 vs Sphaeridiidae Latreille, 1802) not determined, current usage maintained.

### Subfamily Helophorinae

Leach, 1815

Helopherida Leach, 1815: 95 [stem: *Helophor-*]. Type genus: *Helophorus* Fabricius, 1775 [the original spelling *Elophorus* Fabricius, 1775 was considered an incorrect original spelling and placed on the Official Index of Rejected and Invalid Generic Names in Zoology (ICZN 1993b); *Helophorus* Fabricius, 1775 was placed on the Official List of Generic Names in Zoology (ICZN 1993b)]. Comment: published in April 1815; incorrect original stem formation, not in prevailing usage; this family-group name was also used in the same year by Rafinesque (1815 [between April 1815 and 21 July 1815]: 112, as Elophoria).

### Subfamily Epimetopinae

Zaitzev, 1908

Epimetopina Zaitzev, 1908: 353 [stem: *Epimetop-*]. Type genus: *Epimetopus* Lacordaire, 1854.

### Subfamily Georissinae

Laporte, 1840

Géorissites Laporte, 1840b: 44 [stem: *Georiss-*]. Type genus: *Georissus* Latreille, 1809 [placed on the Official List of Generic Names in Zoology (ICZN 1998)]. Comment: original vernacular name available (Art. 11.7.2): first used in latinized form by Heer (1841a: 471, as Georissida), generally accepted as in Hansen (2004: 42, as Georissidae).

### Subfamily Hydrochinae

Thomson, 1859

Hydrochidae C. G. Thomson, 1859: 15 [stem: *Hydroch-*]. Type genus: *Hydrochus* Leach, 1817.

### Subfamily Spercheinae

Erichson, 1837

Spercheini Erichson, 1837: 193 [stem: *Sperche-*]. Type genus: *Spercheus* Kugelann, 1798.

### Subfamily Horelophinae

Hansen, 1991

Horelophinae Hansen, 1991: 104 [stem: *Horeloph-*]. Type genus: *Horelophus* Orchymont, 1913.

### Subfamily Horelophopsinae

Hansen, 1997

Horelophopsinae Hansen, 1997: 108 [stem: *Horelophops-*]. Type genus: *Horelophopsis* Hansen, 1997. Comment: current spelling maintained (Art. 29.5): incorrect stem formation in prevailing usage (should be *Horelophopse*-).

### Subfamily Hydrophilinae

Latreille, 1802

Hydrophilii Latreille, 1802: 136 [stem: *Hydrophil-*]. Type genus: *Hydrophilus* Geoffroy, 1762 [placed on the Official List of Generic Names in Zoology (ICZN 1994a)].

### Tribe Anacaenini

Hansen, 1991

Anacaenini Hansen, 1991: 129 [stem: *Anacaen-*]. Type genus: *Anacaena* C. G. Thomson, 1859.

### Tribe Berosini

Mulsant, 1844

Bérosaires Mulsant, 1844: 97 [stem: *Beros-*]. Type genus: *Berosus* Leach, 1817 [placed on the Official List of Generic Names in Zoology (ICZN 1990a)]. Comment: original vernacular name available (Art. 11.7.2): first used in latinized form by C. G. Thomson (1859: 17, as Berosina), generally accepted as in Hansen (2004: 46, as Berosini).
Derallini Bertrand, 1967: 86, in key [stem: *Derall-*]. Type genus: *Derallus* Sharp, 1882.

### Tribe Chaetarthriini

Bedel, 1881

*Cyllidiaires Mulsant, 1844: 143 [stem: *Cyllidi-*]. Type genus: *Cyllidium* Erichson, 1837 [syn. of *Chaetarthria* Stephens, 1835]. Comment: original vernacular name unavailable (Art. 11.7.2): not subsequently latinized.
Chaetarthriini Bedel, 1881: 314 [stem: *Chaetarthri-*]. Type genus: *Chaetarthria* Stephens, 1835.
Amphiopitae Kuwert, 1890: 120 [stem: *Amphiop-*]. Type genus: *Amphiops* Erichson, 1843.

### Tribe Hydrophilini

Latreille, 1802

Hydrophilii Latreille, 1802: 136 [stem: *Hydrophil-*]. Type genus: *Hydrophilus* Geoffroy, 1762 [placed on the Official List of Generic Names in Zoology (ICZN 1994a)].

### Subtribe Acidocerina

Zaitzev, 1908

*Philhydrates Mulsant, 1844: 131 [stem: *Philydr-*]. Type genus: *Philydrus* Solier, 1834 [as *Philhydrus*, incorrect subsequent spelling of type genus name, not in prevailing usage; preoccupied genus name, not *Philydrus* Duftschmid, 1805 [Coleoptera: Elmidae]; syn. of *Enochrus* C. G. Thomson, 1859]. Comment: original vernacular name unavailable (Art. 11.7.2): subsequently used in latinized form but not generally attributed to Mulsant (1844); incorrect original stem formation, not in prevailing usage.
Philhydrida J. E. LeConte, 1849: 27 [stem: *Philydr-*]. Type genus: *Philydrus* Solier, 1834 [as *Philhydrus*, incorrect subsequent spelling of type genus name, not in prevailing usage; preoccupied genus name, not *Philydrus* Duftschmid, 1805 [Coleoptera: Elmidae]; syn. of *Enochrus* C. G. Thomson, 1859]. Comment: incorrect original stem formation, not in prevailing usage; permanently invalid (Art. 39): based on preoccupied type genus.
Helopeltini G. H. Horn, 1873: 118 [stem: *Helopelt-*]. Type genus: *Helopeltis* G. H. Horn, 1873 [preoccupied genus name, not *Helopeltis* Signoret, 1858 [Hemiptera]; syn. of *Helobata* Bergroth, 1888]. Comment: permanently invalid (Art. 39): based on preoccupied type genus.
Acidocerini Zaitzev, 1908: 353 [stem: *Acidocer-*]. Type genus: *Acidocerus* Klug, 1855.
Helocharae Orchymont, 1919: 147 [stem: *Helochar-*]. Type genus: *Helochares* Mulsant, 1844 [placed on the Official List of Generic Names in Zoology (ICZN 1964)]. Comment: senior homonym of Helocharini Metcalf, 1965 in Homoptera (type genus *Helochara* Fitch, 1851), the homopteran name does not appear to be available (Art. 13.1).

### Subtribe Globuloseina

García, 2001

Globulina García, 2001: 153 [stem: *Globulose*-]. Type genus: *Globulosis* García, 2001. Comment: incorrect original stem formation, not in prevailing usage.

### Subtribe Hydrobiusina

Mulsant, 1844

Hydrobiaires Mulsant, 1844: 116 [stem: *Hydrobius-*]. Type genus: *Hydrobius* Leach, 1815 [placed on the Official List of Generic Names in Zoology (ICZN 1990a)]. Comment: spelling emended to *Hydrobius*- to avoid homonymy with Hydrobiidae Troschel, 1857 in Mollusca (type genus *Hydrobia* Hartmann, 1821) (ICZN 2003a); Hydrobiusina Mulsant, 1844 placed on the Official List of Family-Group Names in Zoology (ICZN 2003a); Hydrobiina Mulsant, 1844 placed on the Official Index of Rejected and Invalid Family-Group Names in Zoology (ICZN 2003a); the family-group name Hydrobia was used earlier by Rafinesque (1815: 110) but since the list of genera did not include *Hydrobius*, it was probably not based on that genus name.

### Subtribe Hydrophilina

Latreille, 1802

Hydrophilii Latreille, 1802: 136 [stem: *Hydrophil-*]. Type genus: *Hydrophilus* Geoffroy, 1762 [placed on the Official List of Generic Names in Zoology (ICZN 1994a)].
Hydatophilidae Gistel, 1856a: 353 [stem: *Hydatophil-*]. Type genus: *Hydatophilus* Gistel, 1856 [syn. of *Hydrophilus* Geoffroy, 1762]. Comment: published by 18 February 1856; this family-group name was also used in the same year by Gistel (1856b [“31 December”]: 95, as Hydatophilida).

### Tribe Laccobiini

Houlbert, 1922

Laccobiini Houlbert, 1922a: 154, in key [stem: *Laccobi-*]. Type genus: *Laccobius* Erichson, 1837. Comment: family-group name previously attributed to Bertrand (1954: 439; see Hansen 2004).
Oocyclini Hansen, 1991: 136 [stem: *Oocycl-*]. Type genus: *Oocyclus* Sharp, 1882.

### Tribe Sperchopsini

Hansen, 1991

Sperchopsini Hansen, 1991: 108 [stem: *Sperchops-*]. Type genus: *Sperchopsis* J. L. LeConte, 1861. Comment: current spelling maintained (Art. 29.5): incorrect stem formation in prevailing usage (should be *Sperchopse*-).

### Subfamily Sphaeridiinae

Latreille, 1802

Sphaeridiota Latreille, 1802: 135 [stem: *Sphaeridi-*]. Type genus: *Sphaeridium* Fabricius, 1775.

### Tribe Andotypini

Hansen, 1991

Andotypini Hansen, 1991: 186 [stem: *Andotyp-*]. Type genus: *Andotypus* Spangler, 1979.

### Tribe Borborophorini

Hansen, 1991

Borborophorini Hansen, 1991: 190 [stem: *Borborophor-*]. Type genus: *Borborophorus* Hansen, 1990.

### Tribe Coelostomatini

Heyden, 1891 (1890)

*Cyclonotides Motschulsky, 1857: 74 [stem: *Cyclonot-*]. Type genus: *Cyclonotum* Erichson, 1837 [syn. of *Coelostoma* Brullé, 1835]. Comment: original vernacular name unavailable (Art. 11.7.2): subsequently used in latinized form but not generally attributed to Motschulsky (1857).
Cyclonotides G. H. Horn, 1890: 281 [stem: *Cyclonot-*]. Type genus: *Cyclonotum* Erichson, 1837 [syn. of *Coelostoma* Brullé, 1835]. Comment: use of younger family-group name Coelostomatini L. Heyden, 1891 conserved (Art. 40.2).
Coelostomitae L. Heyden, 1891: 71 [stem: *Coelostomat-*]. Type genus: *Coelostoma* Brullé, 1835. Comment: incorrect original stem formation, not in prevailing usage; use of family-group name conserved over Cyclonotini G. H. Horn, 1890 (Art. 40.2) (see Hansen 1991); Hansen (1991: 282) mentions that “Cyclonotaires Rey, 1886” is the oldest name for this tribe but he used Art. 40b of the then Code to conserve usage of Coelostomatini L. Heyden, 1891; we could not find any instance where the requirements of Art. 11.7.2 were met and therefore treat the vernacular name Cyclonotaires as unavailable.
Cyllomina Zaitzev, 1908: 400 [stem: *Cylomat-*]. Type genus: *Cyloma* Sharp, 1872 [as *Cylloma*, unjustified emendation of type genus name, not in prevailing usage]. Comment: incorrect original stem formation, not in prevailing usage.

### Tribe Megasternini

Mulsant, 1844

Mégasternaires Mulsant, 1844: 186 [stem: *Megastern-*]. Type genus: *Megasternum* Mulsant, 1844 [placed on the Official List of Generic Names in Zoology (ICZN 1981b)]. Comment: original vernacular name available (Art. 11.7.2): first used in latinized form by G. H. Horn (1890: 307, as Megasterni), generally accepted as in Hansen (2004: 61, as Megasternini).
Cercyones G. H. Horn, 1890: 287 [stem: *Cercyon-*]. Type genus: *Cercyon* Leach, 1817.

### Tribe Omicrini

Smetana, 1975

Omicrini Smetana, 1975: 155 [stem: *Omicr-*]. Type genus: *Omicrus* Sharp, 1879.

### Tribe Protosternini

Hansen, 1991

Protosternini Hansen, 1991: 212 [stem: *Protostern-*]. Type genus: *Protosternum* Sharp, 1890.

### Tribe Rygmodini

Orchymont, 1916

Rygmodini Orchymont, 1916: 238 [stem: *Rygmod-*]. Type genus: *Rygmodus* A. White, 1846.
Rygmodini Orchymont, 1919: 105 [stem: *Rygmod-*]. Type genus: *Rygmodus* A. White, 1846. Comment: family-group name proposed as new without reference to Rygmodini Orchymont, 1916.

### Tribe Sphaeridiini

Latreille, 1802

Sphaeridiota Latreille, 1802: 135 [stem: *Sphaeridi-*]. Type genus: *Sphaeridium* Fabricius, 1775. Comment: Latreille also used the incorrect original spelling Sphéridiote on page 138 of the same work.

### Tribe Tormissini

Hansen, 1991

Tormissini Hansen, 1991: 181 [stem: *Tormiss-*]. Type genus: *Tormissus* Broun, 1893.

### Family Sphaeritidae

Shuckard, 1839

Sphaeritidae Shuckard, 1839a: 149 [stem: *Sphaerit-*]. Type genus: *Sphaerites* Duftschmid, 1805.

### Family Synteliidae

Lewis, 1882

Synteliidae Lewis, 1882: 137 [stem: *Synteli-*]. Type genus: *Syntelia* Westwood, 1864.

### Family Histeridae

Gyllenhal, 1808

Histeroides Gyllenhal, 1808: 74 [stem: *Hister-*]. Type genus: *Hister* Linnaeus, 1758.

### Subfamily Niponiinae

Fowler, 1912

Niponiidae Fowler, 1912: 93 [stem: *Niponi-*]. Type genus: *Niponius* Lewis, 1885.

### Subfamily Abraeinae

MacLeay, 1819

Abreidae W. S. MacLeay, 1819: 25 [stem: *Abrae-*]. Type genus: *Abraeus* Leach, 1817.

### Tribe Abraeini

MacLeay, 1819

Abreidae W. S. MacLeay, 1819: 25 [stem: *Abrae-*]. Type genus: *Abraeus* Leach, 1817.

### Tribe Acritini

Wenzel, 1944

Acritini Wenzel, 1944: 55, in key [stem: *Acrit-*]. Type genus: *Acritus* J. L. LeConte, 1853.

### Tribe Acritomorphini

Wenzel, 1944

Acritomorphini Wenzel, 1944: 55, in key [stem: *Acritomorph-*]. Type genus: *Acritomorphus* Wenzel, 1944.

### Tribe Plegaderini

Portevin, 1929

Plegaderini Portevin, 1929: 601, in key [stem: *Plegader-*]. Type genus: *Plegaderus* Erichson, 1834.

### Tribe Teretriini

Bickhardt, 1914

Teretriinae Bickhardt, 1914: 306 [stem: *Teretri-*]. Type genus: *Teretrius* Erichson, 1834.

### Subfamily Trypeticinae

Bickhardt, 1913

Trypeticinae Bickhardt, 1913: 166 [stem: *Trypetic-*]. Type genus: *Trypeticus* Marseul, 1864.

### Subfamily Trypanaeinae

Marseul, 1857

Trypanéens Marseul, 1857b: 148 [stem: *Trypanae-*]. Type genus: *Trypanaeus* Eschscholtz, 1829. Comment: original vernacular name available (Art. 11.7.2): first used in latinized form by Jakobson (1910: 638, as Trypanaeina), generally accepted as in Newton and Thayer (1992: 25, as Trypanaeinae); incorrect stem formation, not in prevailing usage.

### Subfamily Saprininae

Blanchard, 1845

Saprinites Blanchard, 1845a: 276 [stem: *Saprin-*]. Type genus: *Saprinus* Erichson, 1834. Comment: original vernacular name available (Art. 11.7.2): first used in latinized form by Fairmaire and Laboulbène (1855: 273, as Saprinii), generally accepted as in Mazur (2004: 90, as Saprininae).
Myrmetini Portevin, 1929: 593 [stem: *Myrmet-*]. Type genus: *Myrmetes* Marseul, 1862.

### Subfamily Dendrophilinae

Reitter, 1909

Dendrophilini Reitter, 1909: 288 [stem: *Dendrophil-*]. Type genus: *Dendrophilus* Leach, 1817. Comment: First Reviser (Dendrophilinae Reitter, 1909 vs Paromalinae Reitter, 1909) not determined, current usage maintained.

### Tribe Anapleini

Olexa, 1982

Anapleini Olexa, 1982: 38 [stem: *Anaple-*]. Type genus: *Anapleus* G. H. Horn, 1873.

### Tribe Bacaniini

Kryzhanovskij, 1976

*Bacaniini Vienna, 1974: 273 [stem: *Bacani-*]. Type genus: *Bacanius* J. L. LeConte, 1853. Comment: unavailable name (Art. 13.1): proposed after 1930 without description or bibliographic reference to such a description.
Bacaniini Kryzhanovskij, 1976: 266 [stem: *Bacani-*]. Type genus: *Bacanius* J. L. LeConte, 1853.

### Tribe Dendrophilini

Reitter, 1909

Dendrophilini Reitter, 1909: 288 [stem: *Dendrophil-*]. Type genus: *Dendrophilus* Leach, 1817.

### Tribe Paromalini

Reitter, 1909

Paromalini Reitter, 1909: 287 [stem: *Paromal-*]. Type genus: *Paromalus* Erichson, 1834.

### Subfamily Onthophilinae

MacLeay, 1819

Onthophilidae W. S. MacLeay, 1819: 25 [stem: *Onthophil-*]. Type genus: *Onthophilus* Leach, 1817.
Scolytini Jakobson, 1911a: 652 [stem: *Scolyt-*]. Type genus: *Scolytus* Müller, 1764 [preoccupied genus name, not *Scolytus* Geoffroy, 1762 [Coleoptera: Curculionidae]; syn. of *Onthophilus* Leach, 1817]. Comment: permanently invalid (Art. 39): based on preoccupied type genus.

### Subfamily Tribalinae

Bickhardt, 1914

Tribalini Bickhardt, 1914: 307 [stem: *Tribal-*]. Type genus: *Tribalus* Erichson, 1834.

### Subfamily Histerinae

Gyllenhal, 1808

Histeroides Gyllenhal, 1808: 74 [stem: *Hister-*]. Type genus: *Hister* Linnaeus, 1758.

### Tribe Exosternini

Bickhardt, 1914

Exosternini Bickhardt, 1914: 308 [stem: *Exostern-*]. Type genus: *Exosternus* Lewis, 1902.

### Tribe Histerini

Gyllenhal, 1808

Histeroides Gyllenhal, 1808: 74 [stem: *Hister-*]. Type genus: *Hister* Linnaeus, 1758. Comment: current spelling maintained (Art. 29.5): incorrect stem formation in prevailing usage (should be *Histr*-).

### Tribe Hololeptini

Hope, 1840

Hololeptidae Hope, 1840a: 106 [stem: *Hololept-*]. Type genus: *Hololepta* Paykull, 1811. Comment: W. S. MacLeay (1819: 25) published the name Omoleptidae, presumably as a *lapsus calami* for Hololeptidae, but he did not cite the type genus, nor did he use the family-group name subsequently; in the absence of validating evidence we treat Omoleptidae as a *nomen dubium*.

### Tribe Omalodini

Kryzhanovskij, 1972

*Omalodini A. N. Reichardt, 1941: 37 [stem: *Omalod-*]. Type genus: *Omalodes* Dejean, 1833. Comment: unavailable family-group name, proposed after 1930 without description or bibliographic reference to such a description (Art. 13.1).
Omalodini Kryzhanovskij, 1972: 19 [stem: *Omalod-*]. Type genus: *Omalodes* Dejean, 1833.

### Tribe Platysomatini

Bickhardt, 1914

Platysomini Bickhardt, 1914: 307 [stem: *Platysomat-*]. Type genus: *Platysoma* Leach, 1817. Comment: incorrect original stem formation, not in prevailing usage; correction of original spelling by Mazur (1973); the corrected spelling should be used in order to avoid homonymy with Platysomidae Young, 1866 (type genus *Platysomus* Agassiz, 1833) in Pisces.
Althanini Cooman, 1939: 138 [stem: *Althan-*]. Type genus: *Althanus* Lewis, 1903.

### Subfamily Haeteriinae

Marseul, 1857

Hétériens Marseul, 1857b: 148 [stem: *Haeteri-*]. Type genus: *Haeterius* Dejean, 1833 [as *Hetaerius*, incorrect subsequent spelling of type genus name, not in prevailing usage].

### Tribe Haeteriini

Marseul, 1857

Hétériens Marseul, 1857b: 148 [stem: *Haeteri-*]. Type genus: *Haeterius* Dejean, 1833 [as *Hetaerius*, incorrect subsequent spelling of type genus name, not in prevailing usage]. Comment: original vernacular name available (Art. 11.7.2): first used in latinized form by J. Schmidt (1885: 283, as Hetaeriini [incorrect stem formation]), generally accepted as in Mazur (2004: 75, as Haeteriinae); incorrect stem formation, not in prevailing usage, correction of original spelling by Bousquet and Laplante (1999: 104).

### Tribe Nymphistrini

Tishechkin, 2007

Nymphisterini Tishechkin, 2007: 51 [stem: *Nymphistr-*]. Type genus: *Nymphister* Reichensperger, 1933. Comment: incorrect original stem formation, not in prevailing usage.

### Tribe Synoditulini

Tishechkin, 2007

Synoditulini Tishechkin, 2007: 50 [stem: *Synoditul-*]. Type genus: *Synoditulus* Reichensperger, 1924.

### Subfamily Chlamydopsinae

Bickhardt, 1914

Chlamydopsini Bickhardt, 1914: 308 [stem: *Chlamydops-*]. Type genus: *Chlamydopsis* Westwood, 1869. Comment: current spelling maintained (Art. 29.5): incorrect stem formation in prevailing usage (should be *Chlamydopse*-).

### Superfamily Staphylinoidea

Latreille, 1802

Staphyliniae Latreille, 1802: 124 [stem: *Staphylin-*]. Type genus: *Staphylinus* Linnaeus, 1758 [placed on the Official List of Generic Names in Zoology (ICZN 1959a)]. Comment: First Reviser (Staphylinoidea Latreille, 1802 vs Pselaphoidea Latreille, 1802) not determined, current usage maintained.

### Family Hydraenidae

Mulsant, 1844

Hydraenaires Mulsant, 1844: 50 [stem: *Hydraen-*]. Type genus: *Hydraena* Kugelann, 1794.

### Subfamily Orchymontiinae

Perkins, 1997

Orchymontinae Perkins, 1997: 189 [stem: *Orchymonti-*]. Type genus: *Orchymontia* Broun, 1919. Comment: incorrect original stem formation, not in prevailing usage.

### Subfamily Prosthetopinae

Perkins, 1994

Prosthetopinae Perkins, 1994: 7 [stem: *Prosthetop-*]. Type genus: *Prosthetops* C. O. Waterhouse, 1879. Comment: precedence (Nucleotopinae Perkins, 1994 vs Parasthetopinae Perkins, 1994 vs Prosthetopinae Perkins, 1994 vs Protosthetopinae Perkins, 1994 vs Pterosthetopinae Perkins, 1994) given to taxon originally proposed at the higher rank (Art. 24.1).

### Tribe Coelometoponini

Perkins, 2005

Coelometoponini Perkins, 2005: 9 [stem: *Coelometopon-*]. Type genus: *Coelometopon* Janssens, 1972. Comment: current spelling maintained (Art. 29.5): incorrect stem formation in prevailing usage (should be *Coelometop*-).

### Tribe Nucleotopini

Perkins, 1994

Nucleotopini Perkins, 1994: 8, in key [stem: *Nucleotop-*]. Type genus: *Nucleotops* Perkins and Balfour-Browne, 1994.

### Tribe Parasthetopini

Perkins, 1994

Parasthetopini Perkins, 1994: 8, in key [stem: *Parasthetop-*]. Type genus: *Parasthetops* Perkins and Balfour-Browne, 1994.

### Tribe Prosthetopini

Perkins, 1994

Prosthetopinae Perkins, 1994: 7 [stem: *Prosthetop-*]. Type genus: *Prosthetops* C. O. Waterhouse, 1879.

### Tribe Protosthetopini

Perkins, 1994

Protosthetopini Perkins, 1994: 8, in key [stem: *Protosthetop-*]. Type genus: *Protosthetops* Perkins, 1994.

### Tribe Pterosthetopini

Perkins, 1994

Pterosthetopini Perkins, 1994: 9, in key [stem: *Pterosthetop-*]. Type genus: *Pterosthetops* Perkins, 1994.

### Subfamily Hydraeninae

Mulsant, 1844

Hydraenaires Mulsant, 1844: 50 [stem: *Hydraen-*]. Type genus: *Hydraena* Kugelann, 1794. Comment: First Reviser (Hydraeninae Mulsant, 1844 vs Limnebiinae Mulsant, 1844) not determined, current usage maintained.

### Tribe Hydraenidini

Perkins, 1980

Hydraenidini Perkins, 1980: 414 [stem: *Hydraenid-*]. Type genus: *Hydraenida* Germain, 1901.

### Tribe Hydraenini

Mulsant, 1844

Hydraenaires Mulsant, 1844: 50 [stem: *Hydraen-*]. Type genus: *Hydraena* Kugelann, 1794. Comment: original vernacular name available (Art. 11.7.2): first used in latinized form by Gistel (1848: [1], as Hydraenaeidae [incorrect stem formation]), generally accepted as in Jäch (2004: 102, as Hydraenini).

### Tribe Limnebiini

Mulsant, 1844

Limnébiaires Mulsant, 1844: 88 [stem: *Limnebi-*]. Type genus: *Limnebius* Leach, 1815. Comment: original vernacular name available (Art. 11.7.2): first used in latinized form by C. G. Thomson (1859: 14, as Limnebiidae), generally accepted as in Jäch (2004: 110, as Limnebiini).

### Tribe Madagastrini

Perkins, 1997

Madagastrini Perkins, 1997: 176 [stem: *Madagastr-*]. Type genus: *Madagaster* Perkins, 1997.

### Tribe Parhydraenini

Perkins, 1997

Parhydraenini Perkins, 1997: 170 [stem: *Parhydraen-*]. Type genus: *Parhydraena* Orchymont, 1937.

### Subfamily Ochthebiinae

Thomson, 1859

Ochtebiidae C. G. Thomson, 1859: 15 [stem: *Ochthebi-*]. Type genus: *Ochthebius* Leach, 1815 [placed on the Official List of Generic Names in Zoology (ICZN 1991a)].

### Tribe Ochthebiini

Thomson, 1859

Ochtebiidae C. G. Thomson, 1859: 15 [stem: *Ochthebi-*]. Type genus: *Ochthebius* Leach, 1815 [placed on the Official List of Generic Names in Zoology (ICZN 1991a)].

### Subtribe Enicocerina

Perkins, 1997

Enicocerina Perkins, 1997: 157 [stem: *Enicocer-*]. Type genus: *Enicocerus* Stephens, 1829.

### Subtribe Meropathina

Perkins, 1997

Meropathina Perkins, 1997: 143 [stem: *Meropath-*]. Type genus: *Meropathus* Enderlein, 1901.

### Subtribe Neochthebiina

Perkins, 1997

Neochthebina Perkins, 1997: 152 [stem: *Neochthebi-*]. Type genus: *Neochthebius* Orchymont, 1932. Comment: incorrect original stem formation, not in prevailing usage.

### Subtribe Ochthebiina

Thomson, 1859

Ochtebiidae C. G. Thomson, 1859: 15 [stem: *Ochthebi-*]. Type genus: *Ochthebius* Leach, 1815 [as *Ochtebius*, incorrect subsequent spelling of type genus name, not in prevailing usage; *Ochthebius* placed on the Official List of Generic Names in Zoology (ICZN 1991a)]. Comment: incorrect original stem formation, not in prevailing usage.
Ochthebiinae Perkins, 1980: 414 [stem: *Ochthebi-*]. Type genus: *Ochthebius* Leach, 1815 [placed on the Official List of Generic Names in Zoology (ICZN 1991a)]. Comment: family-group name proposed as new without reference to Ochtebiidae C. G. Thomson, 1859.

### Subtribe Protochthebiina

Perkins, 1997

Protochthebiina Perkins, 1997: 152 [stem: *Protochthebi-*]. Type genus: *Protochthebius* Perkins, 1997.

### Tribe Ochtheosini

Perkins, 1997

Ochtheosini Perkins, 1997: 123 [stem: *Ochtheos-*]. Type genus: *Ochtheosus* Perkins, 1997.

### Family Ptiliidae

Erichson, 1845

Ptilina Erichson, 1845: 15 [stem: *Ptili-*]. Type genus: *Ptilium* Gyllenhal, 1827 [placed on the Official List of Generic Names in Zoology (ICZN 1984b)].

### Subfamily Ptiliinae

Erichson, 1845

Ptilina Erichson, 1845: 15 [stem: *Ptili-*]. Type genus: *Ptilium* Gyllenhal, 1827 [placed on the Official List of Generic Names in Zoology (ICZN 1984b)].

### Tribe Discheramocephalini

Grebennikov, 2009

Discheramocephalini Grebennikov, 2009: 115 [stem: *Discheramocephal-*]. Type genus: *Discheramocephalus* C. Johnson, 2007.

### Tribe Nanosellini

Barber, 1924

Nanosellinae Barber, 1924: 170 [stem: *Nanosell-*]. Type genus: *Nanosella* Motschulsky, 1869.

### Tribe Ptenidiini

Flach, 1889

Ptenidiini Flach, 1889: 489 [stem: *Ptenidi-*]. Type genus: *Ptenidium* Erichson, 1845 [placed on the Official List of Generic Names in Zoology and given precedence over the generic name *Anisarthria* Stephens, 1830 whenever the two names are considered synonyms (ICZN 1984b)].

### Tribe Ptiliini

Erichson, 1845

*Ptilina Heer, 1843: 60 [stem: *Ptili-*]. Type genus: *Ptilium* Gyllenhal, 1827 [as a synonym of *Trichopteryx* Kirby, 1826; *Ptilium* Gyllenhal, 1827 placed on the Official List of Generic Names in Zoology (ICZN 1984b)]. Comment: family-group name unavailable (Art. 11.7.1.1): not based on a genus used as valid at the time; incorrect original stem formation, not in prevailing usage.
Ptilina Erichson, 1845: 15 [stem: *Ptili-*]. Type genus: *Ptilium* Gyllenhal, 1827 [placed on the Official List of Generic Names in Zoology (ICZN 1984b)]. Comment: published 28 May 1845; incorrect original stem formation, not in prevailing usage; this family-group name was also used in the same year by Motschulsky (1845 [after 6 August]: 504, as Ptilien); Motschulsky’s name, originally proposed in a vernacular form, was treated as available by Lawrence and Newton (1995: 818).
Ptilopteriidae Gistel, 1848: [4] [stem: *Ptilopteri-*]. Type genus: *Ptilopterium* Gistel, 1848 [syn. of *Ptilium* Gyllenhal, 1827].
Actidiini Portevin, 1929: 568, in key [stem: *Actidi-*]. Type genus: *Actidium* A. Matthews, 1868.

### Tribe Ptinellini

Reitter, 1906 (1891)

Neuglenini Reitter, 1891: 146 [stem: *Neuglen-*]. Type genus: *Neuglenes* C. G. Thomson, 1859 [syn. of *Ptinella* Motschulsky, 1844].
Ptinellini Reitter, 1906: 259 [stem: *Ptinell-*]. Type genus: *Ptinella* Motschulsky, 1844 [placed on the Official List of Generic Names in Zoology (ICZN 1985b)]. Comment: name proposed to replace Neuglenini Reitter, 1891 because of the synonymy of the type genus; use of younger family-group name Ptinellini Reitter, 1906 conserved (Art. 40.2).
*Pterycini Dybas, 1966: 16, 44 [stem: *Pteryg-*]. Type genus: *Pteryx* A. Matthews, 1858. Comment: incorrect original stem formation, not in prevailing usage; the earlier usage of “pterycine group” by Dybas (1955: 562) is unavailable because it is not a noun (Art. 11.7.1.1); also this name has been used subsequently by Hall (2003: 85) but the name Pterycini has not been made available yet (see Hall 2005: 257).

### Subfamily Cephaloplectinae

Sharp, 1883

Cephaloplectinae Sharp, 1883: 295 [stem: *Cephaloplect-*]. Type genus: *Cephaloplectus* Sharp, 1883.
Limulodinae Ganglbauer, 1898: 297 [stem: *Limulod-*]. Type genus: *Limulodes* A. Matthews, 1866.

### Subfamily Acrotrichinae

Reitter, 1909 (1856)

Trichopterygia Erichson, 1845: 13 [stem: *Trichopteryg-*]. Type genus: *Trichopteryx* Kirby, 1826 [preoccupied genus name, not *Trichopteryx* Hübner, 1825 [Lepidoptera]; syn. of *Acrotrichis* Motschulsky, 1848]. Comment: permanently invalid (Art. 39): based on preoccupied type genus.
Cleopteriidae Gistel, 1856a: 360 [stem: *Cleopteri-*]. Type genus: *Cleopterium* Gistel, 1856 [syn. of *Acrotrichis* Motschulsky, 1848]. Comment: use of younger family-group name Acrotrichinae Reitter, 1909 conserved (Art. 40.2) (see Newton and Thayer 1992: 44).
Acrotrichini Reitter, 1909: 272 [stem: *Acrotrich-*]. Type genus: *Acrotrichis* Motschulsky, 1848. Comment: use of family-group name conserved over Cleopteriinae Gistel, 1856 (Art. 40.2) (see Newton and Thayer 1992: 44).
Nephanini Portevin, 1929: 573, in key [stem: *Nephan-*]. Type genus: *Nephanes* C. G. Thomson, 1859 [placed on the Official List of Generic Names in Zoology (ICZN 1985b)].

### Family Agyrtidae

Thomson, 1859

Agyrtidae C. G. Thomson, 1859: 57 [stem: *Agyrt-*]. Type genus: *Agyrtes* Frölich, 1799.

### Subfamily Agyrtinae

Thomson, 1859

*Agyrtes Motschulsky, 1849: 57 [stem: *Agyrt-*]. Type genus: *Agyrtes* Frölich, 1799. Comment: original vernacular name unavailable (Art. 11.7.2): subsequently used in latinized form but not generally attributed to Motschulsky (1849).
Agyrtidae C. G. Thomson, 1859: 57 [stem: *Agyrt-*]. Type genus: *Agyrtes* Frölich, 1799.
Lyrosomini G. H. Horn, 1880b: 247 [stem: *Lyrosomat-*]. Type genus: *Lyrosoma* Mannerheim, 1853. Comment: incorrect original stem formation, not in prevailing usage; correction of stem by Newton and Thayer (1992: 33).
Lendomini Bradley, 1930: 159, in key [stem: *Lendom-*]. Type genus: *Lendomus* Casey, 1924 [syn. of *Agyrtes* Frölich, 1799].

### Subfamily Necrophilinae

Newton, 1997

*Necrophilini Jeannel, 1936: 10 [stem: *Necrophil-*]. Type genus: *Necrophilus* Latreille, 1829. Comment: unavailable family-group name, proposed after 1930 without description or bibliographic reference to such a description (Art. 13.1).
Necrophilinae Newton, 1997: 127 [stem: *Necrophil-*]. Type genus: *Necrophilus* Latreille, 1829. Comment: the type genus of the older names Necrophilidae Gistel, 1848: [4] and Necrophilidae Gistel, 1856a: 362 cannot be determined (either *Necrophilus* Latreille, 1829 [Agyrtidae] or *Necrophila* Kirby and Spence, 1828 [Silphidae]) and therefore those names are considered unavailable (also see comments in Newton and Thayer (1992: 23) and Newton (1997: 147)).

### Subfamily Pterolomatinae

Thomson, 1862

Pterolomina C. G. Thomson, 1862: 20 [stem: *Pterolomat-*]. Type genus: *Pteroloma* Gyllenhal, 1827. Comment: incorrect original stem formation, not in prevailing usage; correction of stem by Newton and Thayer (1992: 33).

### Family Leiodidae

Fleming, 1821

Leiodesidae Fleming, 1821: 51 [stem: *Leiod-*]. Type genus: *Leiodes* Latreille, 1797. Comment: incorrect original stem formation, not in prevailing usage.

### Subfamily Camiarinae

Jeannel, 1911

Camiarinae Jeannel, 1911: 192, in key [stem: *Camiar-*]. Type genus: *Camiarus* Sharp, 1878.

### Tribe Agyrtodini

Jeannel, 1936

Agyrtodini Jeannel, 1936: 99 [stem: *Agyrtod-*]. Type genus: *Agyrtodes* Portevin, 1907.
*†Guvanocoleina Perkovsky, 2002: 16 [stem: *Gurvanocole-*]. Type genus: *Gurvanocoleus* Ponomarenko, 1986. Comment: unavailable family-group name, proposed after 1930 without description or bibliographic reference to such a description (Art. 13.1); incorrect original stem formation, not in prevailing usage.

### Tribe Camiarini

Jeannel, 1911

Camiarinae Jeannel, 1911: 192, in key [stem: *Camiar-*]. Type genus: *Camiarus* Sharp, 1878.
*†Mesecanina Perkovsky, 2002: 15 [stem: *Mesecan-*]. Type genus: *Mesecanus* Newton, 1982. Comment: unavailable family-group name, proposed after 1930 without description or bibliographic reference to such a description (Art. 13.1).

### Tribe Neopelatopini

Jeannel, 1962

Neopelatopini Jeannel, 1962b: 487 [stem: *Neopelatop-*]. Type genus: *Neopelatops* Jeannel, 1936.

### Subfamily Catopocerinae

Hatch, 1927 (1880)

Catopocerini Hatch, 1927: 4, in key [stem: *Catopocer-*]. Type genus: *Catopocerus* Motschulsky, 1869. Comment: use of family-group name conserved over Pinodytinae G. H. Horn, 1880 (Art. 40.2) (see Newton and Thayer 1992: 34).

### Tribe Catopocerini

Hatch, 1927 (1880)

Pinodytini G. H. Horn, 1880b: 248 [stem: *Pinodyt-*]. Type genus: *Pinodytes* G. H. Horn, 1880 [syn. of *Catopocerus* Motschulsky, 1869]. Comment: use of younger family-group name Catopocerini Hatch, 1927 conserved (Art. 40.2) (see Newton and Thayer 1992: 34).
Catopocerini Hatch, 1927: 4, in key [stem: *Catopocer-*]. Type genus: *Catopocerus* Motschulsky, 1869. Comment: new name for Pinodytini G. H. Horn, 1880 because of synonymy of type genus; use of family-group name conserved over Pinodytini G. H. Horn, 1880 (Art. 40.2) (see Newton and Thayer 1992: 34).

### Tribe Glacicavicolini

Westcott, 1968

Glacicavicolinae Westcott, 1968: 1 [stem: *Glacicavicol-*]. Type genus: *Glacicavicola* Westcott, 1968.

### Subfamily Leiodinae

Fleming, 1821

Leiodesidae Fleming, 1821: 51 [stem: *Leiod-*]. Type genus: *Leiodes* Latreille, 1797.

### Tribe Agathidiini

Westwood, 1838

*Anisotomidae Stephens, 1828: 99 [stem: *Anisotom-*]. Type genus: *Anisotoma* Panzer, 1797. Comment: family-group name unavailable (Art. 11.7.1.1): not based on a genus used as valid at the time; see Newton and Thayer (1992: 20).
Agathidiidae Westwood, 1838: 10 [stem: *Agathidi-*]. Type genus: *Agathidium* Illiger, 1798.
Anisotomidae Reitter, 1884: 6 [stem: *Anisotom-*]. Type genus: *Anisotoma* Panzer, 1797. Comment: an application will need to be submitted to the Commission to suppress Anisotomidae Erichson, 1845 (based on the misidentified type genus *Anisotoma* sensu Schmidt, 1841) for the Principles of Priority and Homonymy (Art. 65.2.1) if this name is to be used as valid.

### Tribe Estadiini

Portevin, 1914

Estadiini Portevin, 1914: 199 [stem: *Estadi-*]. Type genus: *Estadia* Fairmaire, 1903 [syn. of *Dietta* Sharp, 1876].

### Tribe Leiodini

Fleming, 1821

Leiodesidae Fleming, 1821: 51 [stem: *Leiod-*]. Type genus: *Leiodes* Latreille, 1797. Comment: incorrect original stem formation, not in prevailing usage; correction of stem by Newton and Thayer (1992: 34).
Anisotomidae Erichson, 1845: 41 [stem: *Anisotom-*]. Type genus: *Anisotoma* sensu Schmidt, 1841 [not *Anisotoma* Panzer, 1797; syn. of *Leiodes* Latreille, 1797]. Comment: based on a misidentified type genus; an application will need to be submitted to the Commission to suppress this name for the Principles of Priority and Homonymy (Art. 65.2.1) if Anisotomidae Reitter, 1884 in Leiodinae: Agathidiini is to be used as valid in the future.
*Cyrtusina Perkovsky, 1997b: 168 [stem: *Cyrtus-*]. Type genus: *Cyrtusa* Erichson, 1842. Comment: unavailable family-group name, proposed after 1930 without description or bibliographic reference to such a description (Art. 13.1).
*Hypoliodina Perkovsky, 2002: 15 [stem: *Hypoliod-*]. Type genus: *Hypoliodes* Portevin, 1908. Comment: unavailable family-group name, proposed after 1930 without description or bibliographic reference to such a description (Art. 13.1).

### Tribe Pseudoliodini

Portevin, 1926

Pseudoliodini Portevin, 1926: 75 [stem: *Pseudoliod-*]. Type genus: *Pseudoliodes* Portevin, 1926 [syn. of *Pseudcolenis* Reitter, 1884].
Dermatohomoeini Hlisnikovský, 1963: 311 [stem: *Dermatohomoe-*]. Type genus: *Dermatohomoeus* Hlisnikovský, 1963.

### Tribe Scotocryptini

Reitter, 1884

Scotocryptini Reitter, 1884: 91 [stem: *Scotocrypt-*]. Type genus: *Scotocryptus* Girard, 1874.

### Tribe Sogdini

Lopatin, 1961

Sogdiidae Lopatin, 1961: 121 [stem: *Sogd-*]. Type genus: *Sogda* Lopatin, 1961. Comment: incorrect original stem formation, not in prevailing usage; correction of stem by Newton and Thayer (1992: 36).
Triarthrini Jeannel, 1962b: 486, in key [stem: *Triarthr-*]. Type genus: *Triarthron* Märkel, 1840. Comment: junior homonym of Triarthridae Ulrich, 1930 (type genus *Triarthrus* Green, 1832) in Trilobita; this case is to be referred to the Commission to remove the homonymy (Art. 55.3.1).
Hydnobiini Jeannel, 1962b: 492 [stem: *Hydnobi-*]. Type genus: *Hydnobius* Schmidt, 1841.
*Stereina Perkovsky, 2002: 15 [stem: *Stere-*]. Type genus: *Stereus* Wollaston, 1857 [preoccupied genus name, not *Stereus* Mannerheim, 1846 [Coleoptera: Curculionidae]; syn. of *Deltocnemis* Sahlberg, 1886]. Comment: unavailable family-group name, proposed after 1930 without description or bibliographic reference to such a description (Art. 13.1).

### Subfamily Coloninae

Horn, 1880 (1859)

Myloechina C. G. Thomson, 1859: 60 [stem: *Myloech-*]. Type genus: *Myloechus* Latreille, 1807 [syn. of *Colon* Herbst, 1797]. Comment: use of younger family-group name Coloninae G. H. Horn, 1880 conserved (Art. 40.2); see Newton and Thayer (1992: 36).
Colones G. H. Horn, 1880b: 266 [stem: *Colon-*]. Type genus: *Colon* Herbst, 1797 [the original spelling *Kolon* was placed on the Official Index of Rejected and Suppressed Generic Names in Zoology, *Colon* was considered the correct original spelling of the genus and placed on the Official List of Generic Names in Zoology (ICZN 1995b)]. Comment: use of family-group name conserved over Myloechinae C. G. Thomson, 1859 (Art. 40.2); see Newton and Thayer (1992: 36).

### Subfamily Cholevinae

Kirby, 1837

Cholevidae Kirby, 1837: 108 [stem: *Cholev-*]. Type genus: *Choleva* Latreille, 1797.

### Tribe Anemadini

Hatch, 1928

Anemadina Hatch, 1928: 159 [stem: *Anemad-*]. Type genus: *Anemadus* Reitter, 1884.

### Subtribe Anemadina

Hatch, 1928

Anemadina Hatch, 1928: 159 [stem: *Anemad-*]. Type genus: *Anemadus* Reitter, 1884.
Anemadinae Jeannel, 1936: 179 [stem: *Anemad-*]. Type genus: *Anemadus* Reitter, 1884. Comment: family-group name proposed as new without reference to Anemadina Hatch, 1928.

### Subtribe Eocatopina

Jeannel, 1936

Eocatopina Jeannel, 1936: 124, in key [stem: *Eocatop-*]. Type genus: *Eocatops* Peyerimhoff, 1924.

### Subtribe Eunemadina

Newton, 1998

Eunemadina Newton, 1998: 103 [stem: *Eunemad-*]. Type genus: *Eunemadus* Portevin, 1914.

### Subtribe Nemadina

Jeannel, 1936

Nemadinae Jeannel, 1936: 96 [stem: *Nemad-*]. Type genus: *Nemadus* C. G. Thomson, 1867.

### Subtribe Paracatopina

Jeannel, 1936

Paracatopini Jeannel, 1936: 181 [stem: *Paracatop-*]. Type genus: *Paracatops* Portevin, 1907.

### Tribe Cholevini

Kirby, 1837

Cholevidae Kirby, 1837: 108 [stem: *Cholev-*]. Type genus: *Choleva* Latreille, 1797.

### Subtribe Catopina

Chaudoir, 1845

Catopides Chaudoir, 1845: 195 [stem: *Catop-*]. Type genus: *Catops* Paykull, 1798. Comment: original vernacular name available (Art. 11.7.2): first used in latinized form by Agassiz (1846b: 68, as Catopidae), generally accepted as in Perreau (2004: 136, as Catopina).

### Subtribe Cholevina

Kirby, 1837

Cholevidae Kirby, 1837: 108 [stem: *Cholev-*]. Type genus: *Choleva* Latreille, 1797.

### Tribe Eucatopini

Jeannel, 1921

Eucatopini Jeannel, 1921: 233 [stem: *Eucatop-*]. Type genus: *Eucatops* Portevin, 1903.

### Tribe Leptodirini

Lacordaire, 1854 (1849)

Leptodérides Lacordaire, 1854b: 195 [stem: *Leptodir-*]. Type genus: *Leptodirus* Schmidt, 1832 [as *Leptoderus*, unjustified emendation of type genus name by F. Schmidt (1852), not in prevailing usage]. Comment: use of family-group name conserved over Stagobiini Schiødte, 1849 (Art. 40.2); see Newton and Thayer (1992: 35).

### Subtribe Anthroherponina

Jeannel, 1910

Antroherpona Jeannel, 1910: 25 [stem: *Antroherpon-*]. Type genus: *Anthroherpon* Reitter, 1889 [as *Antroherpon*, incorrect subsequent spelling of type genus name, not in prevailing usage]. Comment: incorrect original stem formation, not in prevailing usage.
Hadesiini Absolon, 1913: 108 [stem: *Hadesi-*]. Type genus: *Hadesia* J. Müller, 1911.
Antroherponina Guéorguiev, 1974: 841, in key [stem: *Antroherpon-*]. Type genus: *Anthroherpon* Reitter, 1889 [as *Antroherpon*, incorrect subsequent spelling of type genus name, not in prevailing usage]. Comment: family-group name proposed as new without reference to Antroherpona Jeannel, 1910.

### Subtribe Bathysciina

Horn, 1880

Bathysciae G. H. Horn, 1880b: 251 [stem: *Bathysci-*]. Type genus: *Bathyscia* Schiødte, 1847.
Oriotini Reitter, 1889b: 296 [stem: *Oryot-*]. Type genus: *Oryotus* Miller, 1856 [as *Oriotus*, incorrect subsequent spelling of type genus name, not in prevailing usage]. Comment: incorrect original stem formation, not in prevailing usage.
Bathysciina Guéorguiev, 1974: 841, in key [stem: *Bathysci-*]. Type genus: *Bathyscia* Schiødte, 1847. Comment: family-group name proposed as new without reference to Bathysciae G. H. Horn, 1880.
Neotropospeonellina Perkovsky, 1997a: 59 [stem: *Neotropospeonell-*]. Type genus: *Neotropospeonella* Pace, 1985 [syn. of *Oryotus* Miller, 1856].
*Aphaobiina Perkovsky, 2002: 16 [stem: *Aphaobi-*]. Type genus: *Aphaobius* Abeille de Perrin, 1878. Comment: unavailable family-group name, proposed after 1930 without description or bibliographic reference to such a description (Art. 13.1).

### Subtribe Bathysciotina

Guéorguiev, 1974

Bathysciotina Guéorguiev, 1974: 841, in key [stem: *Bathysciot-*]. Type genus: *Bathysciotes* Jeannel, 1910.

### Subtribe Leptodirina

Lacordaire, 1854 (1849)

Stagobiinae Schiødte, 1849: 16 [stem: *Stagobi-*]. Type genus: *Stagobius* Schiødte, 1847 [syn. of *Leptodirus* Schmidt, 1832]. Comment: use of younger family-group name Leptodirina Lacordaire, 1854 conserved (Art. 40.2); see Newton and Thayer (1992: 35).
Leptodérides Lacordaire, 1854b: 195 [stem: *Leptodir-*]. Type genus: *Leptodirus* Schmidt, 1832 [as *Leptoderus*, unjustified emendation of type genus name by Schmidt (1852), not in prevailing usage]. Comment: original vernacular name available (Art. 11.7.2): first used in latinized form by Kraatz (1859a: 35, as Leptoderidae), generally accepted as in Perreau (2004: 147, as Leptodirini); use of family-group name conserved over Stagobiina Schiødte, 1849 (Art. 40.2); see Newton and Thayer (1992: 35); incorrect original stem formation, not in prevailing usage.
Leptodirina Guéorguiev, 1974: 841, in key [stem: *Leptodir-*]. Type genus: *Leptodirus* Schmidt, 1832. Comment: family-group name proposed as new without reference to Leptodérides Lacordaire, 1854.
*Laneyriellina Perkovsky, 2002: 16 [stem: *Laneyriell-*]. Type genus: *Laneyriella* Guéorguiev, 1976. Comment: unavailable family-group name, proposed after 1930 without description or bibliographic reference to such a description (Art. 13.1).

### Subtribe Pholeuina

Reitter, 1886

Pholeuones Reitter, 1886: 314 [stem: *Pholeu-*]. Type genus: *Pholeuon* Hampe, 1856. Comment: incorrect original stem formation, not in prevailing usage; correction of stem by Newton and Thayer (1992: 35); current spelling maintained (Art. 29.5): corrected stem formation in prevailing usage (should be *Pholeuont*-).
Ghidiniina Guéorguiev, 1974: 841, in key [stem: *Ghidini-*]. Type genus: *Ghidinia* Pavan, 1938 [syn. of *Boldoria* Jeannel, 1924].
*Coiffaitiolina Perkovsky, 2002: 16 [stem: *Coiffaitiol-*]. Type genus: *Coiffaitiola* Jeannel, 1955. Comment: unavailable family-group name, proposed after 1930 without description or bibliographic reference to such a description (Art. 13.1).

### Subtribe Platycholeina

Horn, 1880

Platycholei G. H. Horn, 1880b: 251 [stem: *Platychole-*]. Type genus: *Platycholeus* G. H. Horn, 1880.

### Subtribe Spelaeobatina

Guéorguiev, 1974

Spelaeobatina Guéorguiev, 1974: 841, in key [stem: *Spelaeobat-*]. Type genus: *Spelaeobates* Müller, 1901.

### Tribe Oritocatopini

Jeannel, 1936

Oritocatopini Jeannel, 1936: 116 [stem: *Oritocatop-*]. Type genus: *Oritocatops* Jeannel, 1921.

### Tribe Ptomaphagini

Jeannel, 1911

Ptomaphagini Jeannel, 1911: 193 [stem: *Ptomaphag-*]. Type genus: *Ptomaphagus* Hellwig, 1795.

### Subtribe Baryodirina

Perreau, 2000

Baryodirina Perreau, 2000: 24 [stem: *Baryodir-*]. Type genus: *Baryodirus* Perreau, 2000.

### Subtribe Ptomaphagina

Jeannel, 1911

Ptomaphagini Jeannel, 1911: 193 [stem: *Ptomaphag-*]. Type genus: *Ptomaphagus* Hellwig, 1795.

### Subtribe Ptomaphaginina

Szymczakowski, 1964

Ptomaphaginini Szymczakowski, 1964: 66 [stem: *Ptomaphagin-*]. Type genus: *Ptomaphaginus* Portevin, 1914.

### Tribe Sciaphyini

Perreau, 2000

Sciaphyini Perreau, 2000: 23 [stem: *Sciaphy-*]. Type genus: *Sciaphyes* Jeannel, 1910.

### Subfamily Platypsyllinae

Ritsema, 1869

Platypsyllidae Ritsema, 1869: 38 [stem: *Platypsyll-*]. Type genus: *Platypsyllus* Ritsema, 1869.
Leptinidae J. L. LeConte, 1872: 802 [stem: *Leptin-*]. Type genus: *Leptinus* Müller, 1817. Comment: this family-group name has been attributed to J. L. LeConte (1866 [error for 1867]) in the literature, however J. L. LeConte (1867: 368) did not give a name to the new taxon and therefore it is not available; see Newton and Thayer (1992: 37).

### Family Silphidae

Latreille, 1806

Silphales Latreille, 1806: 1 [stem: *Silph-*]. Type genus: *Silpha* Linnaeus, 1758.

### Subfamily Silphinae

Latreille, 1806

Silphales Latreille, 1806: 1 [stem: *Silph-*]. Type genus: *Silpha* Linnaeus, 1758.
Necrodisidae Gistel, 1848: [4] [stem: *Necrod-*]. Type genus: *Necrodes* Leach, 1815. Comment: incorrect original stem formation, not in prevailing usage.

### Subfamily Nicrophorinae

Kirby, 1837

Necrophoridae Kirby, 1837: 95 [stem: *Nicrophor-*]. Type genus: *Nicrophorus* Fabricius, 1775 [as *Necrophorus*, unjustified emendation of type genus name by Thunberg (1789), not in prevailing usage]. Comment: incorrect original stem formation, not in prevailing usage.

### Family Staphylinidae

Latreille, 1802

Staphyliniae Latreille, 1802: 124 [stem: *Staphylin-*]. Type genus: *Staphylinus* Linnaeus, 1758 [placed on the Official List of Generic Names in Zoology (ICZN 1959a)]. Comment: placed on the Official List of Family-Group Names in Zoology (as Staphylinidae Latreille, [1803-1804]) and “Staphylinii Latreille, [1803-1804]” placed on the Official Index of Rejected and Invalid Family-Group Names in Zoology (ICZN 1959a); First Reviser (Staphylinidae Latreille, 1802 vs Pselaphidae Latreille, 1802) not determined, current usage maintained.

### Subfamily Glypholomatinae

Jeannel, 1962

Glypholomini Jeannel, 1962b: 482 [stem: *Glypholomat-*]. Type genus: *Glypholoma* Jeannel, 1962. Comment: originally proposed as a tribe of Silphidae; incorrect original stem formation, not in prevailing usage; correction of stem by Newton and Thayer (1992: 58).

### Subfamily Microsilphinae

Crowson, 1950

*Micragyrtini Blackwelder, 1944: 84 [stem: *Micragyrt-*]. Type genus: *Micragyrtes* Champion, 1918 [syn. of *Microsilpha* Broun, 1886]. Comment: unavailable family-group name, proposed after 1930 without description or bibliographic reference to such a description (Art. 13.1); originally proposed as a tribe of Leiodidae.
Microsilphinae Crowson, 1950: 279, in key [stem: *Microsilph-*]. Type genus: *Microsilpha* Broun, 1886. Comment: originally proposed as a subfamily of Silphidae.
Micragyrtini Jeannel, 1962b: 484 [stem: *Micragyrt-*]. Type genus: *Micragyrtes* Champion, 1918 [syn. of *Microsilpha* Broun, 1886]. Comment: originally proposed as a tribe of Silphidae.

### Subfamily Omaliinae

MacLeay, 1825

Omalidae W. S. MacLeay, 1825: 49 [stem: *Omali-*]. Type genus: *Omalium* Gravenhorst, 1802.

### Tribe Anthophagini

Thomson, 1859

Anthophagides C. G. Thomson, 1859: 48 [stem: *Anthophag-*]. Type genus: *Anthophagus* Gravenhorst, 1802 [placed on the Official List of Generic Names in Zoology (ICZN 2004d)].
Brathinidae J. L. LeConte, 1861: 48 [stem: *Brathin-*]. Type genus: *Brathinus* J. L. LeConte, 1852. Comment: originally proposed as a subfamily of Silphidae.
*Lestévates Mulsant and Rey, 1880: 8 [stem: *Lestev-*]. Type genus: *Lesteva* Latreille, 1797 [placed on the Official List of Generic Names in Zoology (ICZN 2004d)]. Comment: original vernacular name unavailable (Art. 11.7.2): subsequently used in latinized form but not generally attributed to Mulsant and Rey (1880).
Lestevina Jakobson, 1908: 450 [stem: *Lestev-*]. Type genus: *Lesteva* Latreille, 1797 [placed on the Official List of Generic Names in Zoology (ICZN 2004d)].

### Tribe Aphaenostemmini

Peyerimhoff, 1914

Aphaenostemmini Peyerimhoff, 1914: 248, in key [stem: *Aphaenostemm-*]. Type genus: *Aphaenostemmus* Peyerimhoff, 1914.

### Tribe Corneolabiini

Steel, 1950

Corneolabiini Steel, 1950: 54 [stem: *Corneolabi-*]. Type genus: *Corneolabium* Steel, 1950.

### Tribe Coryphiini

Jakobson, 1908

Coryphiina Jakobson, 1908: 452 [stem: *Coryphi-*]. Type genus: *Coryphium* Stephens, 1834 [placed on the Official List of Generic Names in Zoology (ICZN 1990b)].

### Subtribe Boreaphilina

Zerche, 1990

*Boréaphilaires Mulsant and Rey, 1880: 391 [stem: *Boreaphil-*]. Type genus: *Boreaphilus* Sahlberg, 1832. Comment: original vernacular name unavailable (Art. 11.7.2): subsequently used in latinized form but not generally attributed to Mulsant and Rey (1880).
Boreaphilina Zerche, 1990: 22, in key [stem: *Boreaphil-*]. Type genus: *Boreaphilus* Sahlberg, 1832. Comment: family-group name proposed as new without reference to Boréaphilaires Mulsant and Rey, 1880.

### Subtribe Coryphiina

Jakobson, 1908

Coryphiina Jakobson, 1908: 452 [stem: *Coryphi-*]. Type genus: *Coryphium* Stephens, 1834 [placed on the Official List of Generic Names in Zoology (ICZN 1990b)].

### Tribe Eusphalerini

Hatch, 1957

*Anthobiates Mulsant and Rey, 1880: 290 [stem: *Anthobi-*]. Type genus: *Anthobium* sensu Erichson, 1840 [not *Anthobium* Leach, 1819; syn. of *Eusphalerum* Kraatz, 1857]. Comment: original vernacular name unavailable (Art. 11.7.2): subsequently used in latinized form but not generally attributed to Mulsant and Rey (1880).
Anthobiini Portevin, 1929: 450 [stem: *Anthobi-*]. Type genus: *Anthobium* sensu Erichson, 1840 [not *Anthobium* Leach, 1819; syn. of *Eusphalerum* Kraatz, 1857]. Comment: based on a misidentified type genus, name treated here as invalid until an application is submitted to the Commission to suppress it for the Principle of Priority (Art. 65.2.1).
Eusphalerini Hatch, 1957: 82 [stem: *Eusphaler-*]. Type genus: *Eusphalerum* Kraatz, 1857. Comment: new name for Anthobiini; although this is not the oldest name for the tribe, we recommend that an application be submitted to the Commission to suppress the older name Anthobiini Poertevin, 1929 because it is based on a misidentified type genus (Art. 65.2.1).

### Tribe Hadrognathini

Portevin, 1929

*Eugnathates Mulsant and Rey, 1880: 386 [stem: *Eugnath-*]. Type genus: *Eugnathus* Mulsant and Rey, 1851 [preoccupied genus name, not *Eugnathus* Schönherr, 1833 [Coleoptera: Curculionidae] or *Eugnathus* Agassiz, 1836 [Pisces]; syn. of *Hadrognathus* Schaum, 1852]. Comment: original vernacular name unavailable (Art. 11.7.2): not subsequently latinized; if found to be available then permanently invalid (Art. 39): based on preoccupied type genus.
Hadrognathini Portevin, 1929: 431 [stem: *Hadrognath-*]. Type genus: *Hadrognathus* Schaum, 1852.

### Tribe Omaliini

MacLeay, 1825

Omalidae W. S. MacLeay, 1825: 49 [stem: *Omali-*]. Type genus: *Omalium* Gravenhorst, 1802. Comment: incorrect original stem formation, not in prevailing usage.
Micralymmates Mulsant and Rey, 1880: 3 [stem: *Micralymmat-*]. Type genus: *Micralymma* Westwood, 1838. Comment: original vernacular name available (Art. 11.7.2): first used in latinized form by Portevin (1929: 443, as Micralymmini), generally accepted as in Newton and Thayer (1992: 58, as Micralymmini); incorrect original stem formation, not in prevailing usage.
Tetradeli Fauvel, 1904: 90 [stem: *Tetradel-*]. Type genus: *Tetradelus* Fauvel, 1904.
Arpediopsini Cameron, 1917a: 123 [stem: *Arpediopse-*]. Type genus: *Arpediopsis* Cameron, 1917 [preoccupied genus name, not *Arpediopsis* Ganglbauer, 1895 [Coleoptera: Staphylinidae: Omaliinae: Anthophagini]; syn. of *Crymus* Fauvel, 1904]. Comment: permanently invalid (Art. 39): based on preoccupied type genus; incorrect original stem formation, not in prevailing usage.
Arpediomimi Cameron, 1917b: 277 [stem: *Arpediomim-*]. Type genus: *Arpediomimus* Cameron, 1917 [syn. of *Crymus* Fauvel, 1904]. Comment: replacement name for Arpediopsini Cameron, 1917; incorrect original stem formation, not in prevailing usage.
Phloeonomini Ádám, 2001: 231 [stem: *Phloeonom-*]. Type genus: *Phloeonomus* Heer, 1839. Comment: **syn. nov.**

### Subfamily Empelinae

Newton and Thayer, 1992

*Empelidae M. Abdullah, 1969b: 683 [stem: *Empel-*]. Type genus: *Empelus* J. L. LeConte, 1861. Comment: unavailable family-group name, proposed after 1930 without description or bibliographic reference to such a description (Art. 13.1).
Empelinae Newton and Thayer, 1992: 25 [stem: *Empel-*]. Type genus: *Empelus* J. L. LeConte, 1861.

### Subfamily Proteininae

Erichson, 1839

Proteinini Erichson, 1839a: 641 [stem: *Protein-*]. Type genus: *Proteinus* Latreille, 1797 [placed on the Official List of Generic Names in Zoology (ICZN 1969a)].

### Tribe Anepiini

Steel, 1966

Anepiini Steel, 1966: 306 [stem: *Anepi-*]. Type genus: *Anepius* Blackburn, 1902.

### Tribe Austrorhysini

Newton and Thayer, 1995

Austrorhysini Newton and Thayer, 1995: 298 [stem: *Austrorhys-*]. Type genus: *Austrorhysus* Steel, 1966.

### Tribe Nesoneini

Steel, 1966

Nesoneini Steel, 1966: 292 [stem: *Nesone-*]. Type genus: *Nesoneus* Bernhauer, 1939.

### Tribe Proteinini

Erichson, 1839

Proteinini Erichson, 1839a: 641 [stem: *Protein-*]. Type genus: *Proteinus* Latreille, 1797 [placed on the Official List of Generic Names in Zoology (ICZN 1969a)]. Comment: this family-group name was also proposed in the same year by Erichson (1839b: 31, as Proteinini) and Heer (1839b: 4, as Proteinina); for comments about the priority of these works see Newton and Thayer (1992: 24).
*Phléobiens Mulsant and Rey, 1876: 209 [stem: *Phloeobi-*]. Type genus: *Phloeobium* sensu Erichson, 1840 [not *Phloeobium* Dejean, 1833; syn. of *Metopsia* Wollaston, 1854]. Comment: original vernacular name unavailable (Art. 11.7.2): subsequently used in latinized form but not generally attributed to Mulsant and Rey, 1876; incorrect original stem formation, not in prevailing usage.
Phloeobiinae Fowler, 1888: 431 [stem: *Phloeobi-*]. Type genus: *Phloeobium* sensu Erichson, 1840 [not *Phloeobium* Dejean, 1833; syn. of *Metopsia* Wollaston, 1854]. Comment: based on a misidentified type genus.
Megarthrini Joy, 1932: 93, in key [stem: *Megarthr-*]. Type genus: *Megarthrus* Curtis, 1829.
Metopsiinae Tottenham, 1954: 8, in key [stem: *Metopsi-*]. Type genus: *Metopsia* Wollaston, 1854.
Pteronini Arnett, 1961: 238, in key [stem: *Pteroni-*]. Type genus: *Pteronius* Blackwelder, 1952 [syn. of *Proteinus* Latreille, 1797]. Comment: incorrect original stem formation, not in prevailing usage.
Pteroniinae I. Moore, 1964: 85, in key [stem: *Pteroni-*]. Type genus: *Pteronius* Blackwelder, 1952 [syn. of *Proteinus* Latreille, 1797]. Comment: proposed as new without reference to Pteronini Arnett, 1961.

### Tribe Silphotelini

Newton and Thayer, 1995

Silphotelini Newton and Thayer, 1995: 297 [stem: *Silphotel-*]. Type genus: *Silphotelus* Broun, 1895.

### Subfamily Micropeplinae

Leach, 1815

Micropeplida Leach, 1815: 90 [stem: *Micropepl-*]. Type genus: *Micropeplus* Latreille, 1809.
Micropeplida Heer, 1839a: 169 [stem: *Micropepl-*]. Type genus: *Micropeplus* Latreille, 1809. Comment: family-group name proposed as new without reference to Micropeplida Leach, 1815; this family-group name was also used in the same year by Heer (1839b: 4, as Micropeplida); for comments about the priority of these works see Newton and Thayer (1992: 24).

### Subfamily Neophoninae

Fauvel, 1905

Neophoni Fauvel, 1905a: 98 [stem: *Neophon-*]. Type genus: *Neophonus* Fauvel, 1905.

### Subfamily Dasycerinae

Reitter, 1887

Dasycerini Reitter, 1887: 8, in key [stem: *Dasycer-*]. Type genus: *Dasycerus* Brongniart, 1800. Comment: originally proposed as a new tribe of Latridiidae; the earlier usage of the Dasycerinae by Swainson (1840: 293) was not based on a type genus and is therefore unavailable.

### Subfamily Protopselaphinae

Newton and Thayer, 1995

Protopselaphinae Newton and Thayer, 1995: 227 [stem: *Protopselaph-*]. Type genus: *Protopselaphus* Newton and Thayer, 1995.

### Subfamily Pselaphinae

Latreille, 1802

Pselaphii Latreille, 1802: 239 [stem: *Pselaph-*]. Type genus: *Pselaphus* Herbst, 1791.

### Supertribe Batrisitae

Reitter, 1882

Batrisini Reitter, 1882b: 183 [stem: *Batris-*]. Type genus: *Batrisus* Aubé, 1833.

### Tribe Amauropini

Jeannel, 1948

Amauropsini Jeannel, 1948a: 1 [stem: *Amaurop-*]. Type genus: *Amaurops* Fairmaire, 1851. Comment: incorrect original stem formation, not in prevailing usage; correction of stem by Newton and Thayer (1992: 37).

### Tribe Batrisini

Reitter, 1882

Batrisini Reitter, 1882b: 183 [stem: *Batris-*]. Type genus: *Batrisus* Aubé, 1833.

### Subtribe Ambicocerina

Leleup, 1970

Ambicocerina Leleup, 1970: 309 [stem: *Ambicocer-*]. Type genus: *Ambicocerus* Leleup, 1970.

### Subtribe Batrisina

Reitter, 1882

Batrisini Reitter, 1882b: 183 [stem: *Batris-*]. Type genus: *Batrisus* Aubé, 1833.
Oropygiina Jeannel, 1949a: 113, in key [stem: *Orropygi-*]. Type genus: *Orropygia* Raffray, 1910 [as *Oropygia*, incorrect subsequent spelling of type genus name, not in prevailing usage]. Comment: incorrect original stem formation, not in prevailing usage; correction of stem by Newton and Thayer (1992: 37).
Trabisina Jeannel, 1949a: 114, in key [stem: *Trabis-*]. Type genus: *Trabisus* Raffray, 1890 [syn. of *Atheropterus* Raffray, 1882].

### Subtribe Leupeliina

Jeannel, 1954

Leupeliina Jeannel, 1954c: 106 [stem: *Leupeli-*]. Type genus: *Leupelia* Jeannel, 1954.

### Subtribe Stilipalpina

Jeannel, 1954

Stilipalpina Jeannel, 1954c: 118 [stem: *Stilipalp-*]. Type genus: *Stilipalpus* Jeannel, 1951.

### Tribe Thaumastocephalini

Poggi, Nonveiller, Colla, Pavićević and Rada, 2001

Thaumastocephalini Poggi et al., 2001: 5, in key [stem: *Thaumastocephal-*]. Type genus: *Thaumastocephalus* Poggi et al., 2001.

### Supertribe Clavigeritae

Leach, 1815

Clavigerides Leach, 1815: 117 [stem: *Claviger-*]. Type genus: *Claviger* Preyssler, 1790.

### Tribe Clavigerini

Leach, 1815

Clavigerides Leach, 1815: 117 [stem: *Claviger-*]. Type genus: *Claviger* Preyssler, 1790.

### Subtribe Apoderigerina

Jeannel, 1954

Apoderigerini Jeannel, 1954a: 310 [stem: *Apoderiger-*]. Type genus: *Apoderiger* Wasmann, 1897.

### Subtribe Clavigerina

Leach, 1815

Clavigerides Leach, 1815: 117 [stem: *Claviger-*]. Type genus: *Claviger* Preyssler, 1790.
Adranites Desmarest, 1857: 144 [stem: *Adran-*]. Type genus: *Adranes* J. L. LeConte, 1849.
Adraniini O. Park, 1951: 58, in key [stem: *Adran-*]. Type genus: *Adranes* J. L. LeConte, 1849. Comment: family-group name proposed as new without reference to Adranites Desmarest, 1857; incorrect original stem formation, not in prevailing usage.

### Subtribe Clavigerodina

Schaufuss, 1882

Clavigerodini L. W. Schaufuss, 1882a: 205 [stem: *Clavigerod-*]. Type genus: *Clavigerodes* Raffray, 1877.
Commatocerini L. W. Schaufuss, 1882b: 349 [stem: *Commatocer-*]. Type genus: *Commatocerus* Raffray, 1882 [syn. of *Fustiger* J. L. LeConte, 1866].
Clavigeropsini L. W. Schaufuss, 1890: in table [stem: *Clavigeropse-*]. Type genus: *Clavigeropsis* Raffray, 1882. Comment: incorrect original stem formation, not in prevailing usage.
Fustigerini Jeannel, 1949a: 31 [stem: *Fustiger-*]. Type genus: *Fustiger* J. L. LeConte, 1866.
Neoceratopsini Célis, 1970: 260 [stem: *Neoceratopse-*]. Type genus: *Neoceratopsis* Jeannel, 1956. Comment: incorrect original stem formation, not in prevailing usage.

### Subtribe Disarthricerina

Jeannel, 1949

Diarthricerini Jeannel, 1949a: 29, in key [stem: *Disarthricer-*]. Type genus: *Disarthricerus* Raffray, 1895 [as *Diarthricerus*, incorrect subsequent spelling of type genus name, not in prevailing usage]. Comment: incorrect original stem formation, not in prevailing usage.

### Subtribe Hoplitoxenina

Célis, 1969

Hoplitoxenini Célis, 1969: 418 [stem: *Hoplitoxen-*]. Type genus: *Hoplitoxenus* Jeannel, 1960.
Dimerometopini Célis, 1970: 244 [stem: *Dimerometop-*]. Type genus: *Dimerometopus* Célis, 1970.

### Subtribe Lunillina

Célis, 1969

Lunillini Célis, 1969: 416 [stem: *Lunill-*]. Type genus: *Lunilla* Jeannel, 1957.

### Subtribe Mastigerina

Jeannel, 1954

Mastigerini Jeannel, 1954a: 291, in key [stem: *Mastiger-*]. Type genus: *Mastiger* Motschulsky, 1851.

### Subtribe Miroclavigerina

Jeannel, 1949

Miroclavigerini Jeannel, 1949a: 29, in key [stem: *Miroclaviger-*]. Type genus: *Miroclaviger* Wasmann, 1893.

### Subtribe Neocerina

Jeannel, 1954

Neocerini Jeannel, 1954a: 316 [stem: *Neocer-*]. Type genus: *Neocerus* Wasmann, 1893. Comment: First Reviser (Neocerina Jeannel, 1954 vs Theocerina Jeannel, 1954) not determined, current usage maintained.
Theocerini Jeannel, 1954a: 314 [stem: *Theocer-*]. Type genus: *Theocerus* Raffray, 1897.

### Subtribe Radamina

Jeannel, 1954

Radamini Jeannel, 1954a: 319 [stem: *Radam-*]. Type genus: *Radama* Raffray, 1883.

### Subtribe Thysdariina

Jeannel, 1954

Thysdariini Jeannel, 1954a: 332 [stem: *Thysdari-*]. Type genus: *Thysdarius* Fairmaire, 1904.

### Tribe Colilodionini

Besuchet, 1991

Colilodionini Besuchet, 1991: 514 [stem: *Colilodion-*]. Type genus: *Colilodion* Besuchet, 1991.

### Tribe Tiracerini

Besuchet, 1986

Articerides Desmarest, 1857: 145 [stem: *Articer-*]. Type genus: *Articerus* sensu Hope, 1845 [not *Articerus* Dalman, 1826; syn. of *Tiracerus* Besuchet, 1986]. Comment: based on a misidentified type genus, name treated here as invalid until an application is submitted to the Commission to suppress it for the Principle of Priority (Art. 65.2.1).
Tiracerini Besuchet, 1986: 263 [stem: *Tiracer-*]. Type genus: *Tiracerus* Besuchet, 1986.

### Supertribe Euplectitae

Streubel, 1839

Euplectidae Streubel, 1839: 135 [stem: *Euplect-*]. Type genus: *Euplectus* Leach, 1817.

### Tribe Bythinoplectini

Schaufuss, 1890

Bythinoplectini L. W. Schaufuss, 1890: in table [stem: *Bythinoplect-*]. Type genus: *Bythinoplectus* Reitter, 1882.

### Subtribe Bythinoplectina

Schaufuss, 1890

Bythinoplectini L. W. Schaufuss, 1890: in table [stem: *Bythinoplect-*]. Type genus: *Bythinoplectus* Reitter, 1882.
Zethini L. W. Schaufuss, 1890: in table [stem: *Zeth-*]. Type genus: *Zethus* L. W. Schaufuss, 1872 [preoccupied genus name, not *Zethus* Fabricius, 1805 [Hymenoptera], not *Zethus* Pander, 1830 [Trilobita]; syn. of *Zethopsus* Reitter, 1880]. Comment: permanently invalid (Art. 39): based on preoccupied type genus.
Zethopsina Jeannel, 1952a: 51, in key [stem: *Zethops-*]. Type genus: *Zethopsus* Reitter, 1880.

### Subtribe Pyxidicerina

Raffray, 1904

Pyxidicerini Raffray, 1904: 489, in key [stem: *Pyxidicer-*]. Type genus: *Pyxidicerus* Motschulsky, 1863.

### Tribe Dimerini

Raffray, 1908

Dimerini Raffray, 1908: 412 [stem: *Dimer-*]. Type genus: *Dimerus* Fiori, 1899 [syn. of *Octomicrus* L. W. Schaufuss, 1877]. Comment: published before 17 March 1908; this family-group name was also used in the same year by Bernhauer (1908 [23 March]: 327, as Dimerini); use of family-group name Dimerini Raffray, 1908 (based on synonym) conserved (Art. 40.2); see Newton and Thayer (1992: 39).
Octomicrini Jeannel, 1952a: 43 [stem: *Octomicr-*]. Type genus: *Octomicrus* L. W. Schaufuss, 1877.

### Tribe Euplectini

Streubel, 1839

Euplectidae Streubel, 1839: 135 [stem: *Euplect-*]. Type genus: *Euplectus* Leach, 1817.
Mitracephalini O. Park, 1951: 64, in key [stem: *Mitracephal-*]. Type genus: *Mitracephala* Raffray, 1890 [preoccupied genus name, not *Mitracephala* J. Thomson, 1859 [Coleoptera: Scarabaeidae]; syn. of *Mitrametopus* Raffray, 1911]. Comment: permanently invalid (Art. 39): based on preoccupied type genus.
Mitrametopina O. Park, 1952: 87 [stem: *Mitrametop-*]. Type genus: *Mitrametopus* Raffray, 1911. Comment: replacement name for Mitracephalini O. Park, 1951 because of the homonymy of the type genus.

### Tribe Jubini

Raffray, 1904

Jubini Raffray, 1904: 489, in key [stem: *Jub-*]. Type genus: *Jubus* L. W. Schaufuss, 1872.
Auxenocerini Jeannel, 1962a: 319 [stem: *Auxenocer-*]. Type genus: *Auxenocerus* Jeannel, 1962.

### Tribe Mayetiini

Winkler, 1925

Mayetiini A. Winkler, 1925: 348 [stem: *Mayeti-*]. Type genus: *Mayetia* Mulsant and Rey, 1875.

### Tribe Metopiasini

Raffray, 1904

Metopiini Raffray, 1904: 490, in key [stem: *Metopias-*]. Type genus: *Metopias* Gory, 1832 [placed on the Official List of Generic Names in Zoology (ICZN 1994d)]. Comment: *Metopias*- established as the correct stem and Metopiasini Raffray, 1904 placed on the Official List of Family-Group Names in Zoology (ICZN 1994d).

### Subtribe Metopiasina

Raffray, 1904

Metopiini Raffray, 1904: 490, in key [stem: *Metopias-*]. Type genus: *Metopias* Gory, 1832 [placed on the Official List of Generic Names in Zoology (ICZN 1994d)]. Comment: correct original stem *Metopi*- modified to *Metopias*- and Metopiasini Raffray, 1904 placed on the Official List of Family-Group Names in Zoology (ICZN 1994d); Metopiini Raffray, 1904 placed on the Official Index of Rejected and Invalid Family-Group Names in Zoology (ICZN 1994d).

### Subtribe Rhinoscepsina

Bowman, 1934

Rhinoscepsii Bowman, 1934: 8 [stem: *Rhinosceps-*]. Type genus: *Rhinoscepsis* J. L. LeConte, 1878. Comment: current spelling maintained (Art. 29.5): incorrect stem formation in prevailing usage (should be *Rhinoscepse*-).

### Tribe Trichonychini

Reitter, 1882

Trichonyides Reitter, 1882b: 194 [stem: *Trichonych-*]. Type genus: *Trichonyx* Chaudoir, 1845.

### Subtribe Bibloporina

Park, 1951

Bibloporini O. Park, 1951: 64, in key [stem: *Biblopor-*]. Type genus: *Bibloporus* C. G. Thomson, 1859.
Bibloporellina Jeannel, 1952a: 92 [stem: *Bibloporell-*]. Type genus: *Bibloporellus* Jeannel, 1949.

### Subtribe Panaphantina

Jeannel, 1950

Panaphantina Jeannel, 1950a: 76 [stem: *Panaphant-*]. Type genus: *Panaphantus* Kiesenwetter, 1858.
Trisignina O. Park and Schuster, 1955: 1 [stem: *Trisign-*]. Type genus: *Trisignis* O. Park and Schuster, 1955.
Acetaliini Jeannel, 1958a: 81 [stem: *Acetali-*]. Type genus: *Acetalius* Sharp, 1883.
Bibloplectina Jeannel, 1959: 96, in key [stem: *Bibloplect-*]. Type genus: *Bibloplectus* Reitter, 1881.

### Subtribe Trichonychina

Reitter, 1882

Trichonyides Reitter, 1882b: 194 [stem: *Trichonych-*]. Type genus: *Trichonyx* Chaudoir, 1845. Comment: incorrect original stem formation, not in prevailing usage.
Raffrayina Jeannel, 1949a: 76 [stem: *Raffrayi-*]. Type genus: *Raffrayia* Reitter, 1882. Comment: incorrect original stem formation, not in prevailing usage; correction of stem by Jeannel (1964: 65, as Raffrayiini).
Ranavalini Jeannel, 1954a: 183 [stem: *Ranaval-*]. Type genus: *Ranavala* Raffray, 1898.
Chrestomerina Jeannel, 1962a: 344 [stem: *Chrestomer-*]. Type genus: *Chrestomera* Jeannel, 1962.
Pteracmini Jeannel, 1962a: 347 [stem: *Pteracmet-*]. Type genus: *Pteracmes* Raffray, 1890. Comment: incorrect original stem formation, not in prevailing usage.
Trimiodytina Jeannel, 1964: 39 [stem: *Trimiodyt-*]. Type genus: *Trimiodytes* Raffray, 1897.

### Subtribe Trimiina

Bowman, 1934

Trimii Bowman, 1934: 8 [stem: *Trimi-*]. Type genus: *Trimium* Aubé, 1833.
Trimiina Jeannel, 1950a: 139 [stem: *Trimi-*]. Type genus: *Trimium* Aubé, 1833. Comment: family-group name proposed as new without reference to Trimii Bowman, 1934.

### Tribe Trogastrini

Jeannel, 1949

Trogastrini Jeannel, 1949a: 41, in key [stem: *Trogastr-*]. Type genus: *Trogaster* Sharp, 1874.

### Subtribe Phtegnomina

Park, 1951

Phtegnomini O. Park, 1951: 64, in key [stem: *Phtegnom-*]. Type genus: *Phtegnomus* Raffray, 1890.

### Subtribe Rhexiina

Park, 1951

Rhexini O. Park, 1951: 63, in key [stem: *Rhexi-*]. Type genus: *Rhexius* J. L. LeConte, 1849. Comment: incorrect original stem formation, not in prevailing usage; correction of stem by Newton and Thayer (1992: 39).

### Subtribe Trogastrina

Jeannel, 1949

Trogastrini Jeannel, 1949a: 41, in key [stem: *Trogastr-*]. Type genus: *Trogaster* Sharp, 1874.

### Supertribe Faronitae

Reitter, 1882

Faronides Reitter, 1882b: 194 [stem: *Faron-*]. Type genus: *Faronus* Aubé, 1844.

### Supertribe Goniaceritae

Reitter, 1882 (1872)

Goniacerides Reitter, 1882b: 188 [stem: *Goniacer-*]. Type genus: *Goniacerus* Motschulsky, 1855. Comment: use of family-group name conserved over Goniastitae L. W. Schaufuss, 1872 (Art. 40.2) (see Newton and Thayer 1992: 39).

### Tribe Arnylliini

Jeannel, 1952

Arnylliini Jeannel, 1952b: 100 [stem: *Arnylli-*]. Type genus: *Arnyllium* Reitter, 1884.

### Tribe Barrosellini

Leleup, 1973

Barrosellini Leleup, 1973: 81 [stem: *Barrosell-*]. Type genus: *Barrosellus* Jeannel, 1951.

### Tribe Brachyglutini

Raffray, 1904

Brachyglutini Raffray, 1904: 490, in key [stem: *Brachyglut-*]. Type genus: *Brachygluta* C. G. Thomson, 1859.

### Subtribe Baradina

Park, 1951

Baradiini O. Park, 1951: 62, in key [stem: *Barad-*]. Type genus: *Barada* Raffray, 1891 [syn. of *Euphalepsus* Reitter, 1883]. Comment: incorrect original stem formation, not in prevailing usage; correction of stem by Newton and Thayer (1992: 40).

### Subtribe Brachyglutina

Raffray, 1904

Bryaxes J. L. LeConte, 1861: 57 [stem: *Bryaxe-*]. Type genus: *Bryaxis* Leach, 1817 [preoccupied genus name, not *Bryaxis* Kugelann, 1794 [Coleoptera: Staphylinidae: Pselaphinae: Bythinini]; placed on the Official Index of Rejected and Invalid Generic Names in Zoology (ICZN 1969b); syn. of *Rybaxis* Saulcy, 1876]. Comment: permanently invalid (Art. 39): based on preoccupied and suppressed type genus; incorrect original stem formation, not in prevailing usage.
Brachyglutini Raffray, 1904: 490, in key [stem: *Brachyglut-*]. Type genus: *Brachygluta* C. G. Thomson, 1859. Comment: replacement name for Bryaxes J. L. LeConte, 1861.
Reichenbachiina Jakobson, 1910: 577 [stem: *Reichenbachi-*]. Type genus: *Reichenbachia* Leach, 1826.
Globina Jeannel, 1959: 462, in key [stem: *Glob-*]. Type genus: *Globa* Raffray, 1887.
Halorabyxina Leleup, 1969a: 138 [stem: *Halorabyx-*]. Type genus: *Halorabyxis* Jeannel, 1954 [syn. of *Physoplectus* Reitter, 1882].
Pselaptina O. Park, 1976: 48 [stem: *Pselapt-*]. Type genus: *Pselaptus* J. L. LeConte, 1880.

### Subtribe Decarthrina

Park, 1951

Decarthronini O. Park, 1951: 61, in key [stem: *Decarthr-*]. Type genus: *Decarthron* Brendel, 1865. Comment: incorrect original stem formation, not in prevailing usage; correction of stem by Newton and Thayer (1992: 40).

### Subtribe Eupseniina

Park, 1951

Eupseniini O. Park, 1951: 61, in key [stem: *Eupseni-*]. Type genus: *Eupsenius* J. L. LeConte, 1849.

### Tribe Bythinini

Raffray, 1890

Bythinini Raffray, 1890: 83, in key [stem: *Bythin-*]. Type genus: *Bythinus* Leach, 1817 [placed on the Official List of Generic Names in Zoology (ICZN 1969b)].

### Subtribe Bythinina

Raffray, 1890

Bythinini Raffray, 1890: 83, in key [stem: *Bythin-*]. Type genus: *Bythinus* Leach, 1817 [placed on the Official List of Generic Names in Zoology (ICZN 1969b)].
Bryaxina Jakobson, 1910: 579 [stem: *Bryaxe-*]. Type genus: *Bryaxis* Kugelann, 1794 [placed on the Official List of Generic Names in Zoology (ICZN 1969b)]. Comment: incorrect original stem formation, not in prevailing usage.

### Subtribe Machaeritina

Jeannel, 1950

Machaeritina Jeannel, 1950a: 168 [stem: *Machaerit-*]. Type genus: *Machaerites* Miller, 1855.

### Subtribe Xenobythina

Jeannel, 1950

Xenobythina Jeannel, 1950a: 201 [stem: *Xenobyth-*]. Type genus: *Xenobythus* Peyerimhoff, 1901.

### Tribe Cyathigerini

Schaufuss, 1872

Cyathigerini L. W. Schaufuss, 1872: 245 [stem: *Cyathiger-*]. Type genus: *Cyathiger* King, 1865 [syn. of *Plagiophorus* Motschulsky, 1851].

### Tribe Goniacerini

Reitter, 1882 (1872)

Goniastini L. W. Schaufuss, 1872: 245 [stem: *Goniast-*]. Type genus: *Goniastes* Westwood, 1870. Comment: use of younger family-group name Goniacerini Reitter, 1882 conserved (Art. 40.2) (see Newton and Thayer 1992: 40).
Goniacerides Reitter, 1882b: 188 [stem: *Goniacer-*]. Type genus: *Goniacerus* Motschulsky, 1855. Comment: use of family-group name conserved over Goniastini L. W. Schaufuss, 1872 (Art. 40.2) (see Newton and Thayer 1992: 40).
Simini L. W. Schaufuss, 1890: in table [stem: *Sim-*]. Type genus: *Simus* Raffray, 1882 [preoccupied genus name, not *Simus* Bonaparte, 1838 [Reptilia] or *Simus* Hodgson, 1841 [Aves]; syn. of *Ipsimus* Reitter, 1885]. Comment: permanently invalid (Art. 39): based on preoccupied type genus.
Listriophorini L. W. Schaufuss, 1890: in table [stem: *Listriophor-*]. Type genus: *Listriophorus* L. W. Schaufuss, 1872.

### Tribe Imirini

Jeannel, 1949

Mirini Raffray, 1917: 110 [stem: *Mir-*]. Type genus: *Mirus* Saulcy, 1877 [preoccupied genus name, not *Mirus* Albers, 1850 [Mollusca]; syn. of *Imirus* Reitter, 1885]. Comment: permanently invalid (Art. 39): based on preoccupied type genus; junior homonym of Miridae Hahn, 1833 (type genus *Miris* Fabricius, 1794) in Hemiptera.
Imirini Jeannel, 1949a: 41, in key [stem: *Imir-*]. Type genus: *Imirus* Reitter, 1885.

### Tribe Iniocyphini

Park, 1951

Iniocyphini O. Park, 1951: 60, in key [stem: *Iniocyph-*]. Type genus: *Iniocyphus* Raffray, 1912.

### Subtribe Iniocyphina

Park, 1951

Iniocyphini O. Park, 1951: 60, in key [stem: *Iniocyph-*]. Type genus: *Iniocyphus* Raffray, 1912. Comment: First Reviser found (Iniocyphina Park, 1951 vs Dalmodiina Park, 1951) is Chandler (2001: 377).
Dalmodiini O. Park, 1951: 61, in key [stem: *Dalmod-*]. Type genus: *Dalmodes* Reitter, 1882. Comment: incorrect original stem formation, not in prevailing usage; correction of stem by Newton and Thayer (1992: 41).

### Subtribe Natypleurina

Newton and Thayer, 1992

Tanypleurini Jeannel, 1949a: 79 [stem: *Tanypleur-*]. Type genus: *Tanypleurus* Raffray, 1890 [preoccupied genus name, not *Tanypleurus* Steenstrup and Luetken, 1861 [Crustacea]; syn. of *Natypleurus* Newton and Thayer, 1992]. Comment: permanently invalid (Art. 39): based on preoccupied type genus.
Natypleurina Newton and Thayer, 1992: 41 [stem: *Natypleur-*]. Type genus: *Natypleurus* Newton and Thayer, 1992. Comment: replacement name for Tanypleurini Jeannel, 1949 because of the homonymy of the type genus.

### Tribe Machadoini

Jeannel, 1951

Machadoini Jeannel, 1951: 105 [stem: *Machado-*]. Type genus: *Machadous* Jeannel, 1951.

### Tribe Proterini

Jeannel, 1949

Proterini Jeannel, 1949a: 41, in key [stem: *Proter-*]. Type genus: *Proterus* Raffray, 1897.

### Tribe Pygoxyini

Reitter, 1909

Pygoxyini Reitter, 1909: 202 [stem: *Pygoxy-*]. Type genus: *Pygoxyon* Reitter, 1880.

### Tribe Speleobamini

Park, 1951

Speleobamini O. Park, 1951: 51 [stem: *Speleobam-*]. Type genus: *Speleobama* O. Park, 1951.

### Tribe Tychini

Raffray, 1904

Tychini Raffray, 1904: 490, in key [stem: *Tych-*]. Type genus: *Tychus* Leach, 1817.

### Tribe Valdini

Park, 1953

Valdiini O. Park, 1953: 261 [stem: *Vald-*]. Type genus: *Valda* Casey, 1894. Comment: incorrect original stem formation, not in prevailing usage; correction of stem by Newton and Thayer (1992: 41).

### Supertribe Pselaphitae

Latreille, 1802

Pselaphii Latreille, 1802: 239 [stem: *Pselaph-*]. Type genus: *Pselaphus* Herbst, 1791.

### Tribe Arhytodini

Raffray, 1890

Arhytodini Raffray, 1890: 84, in key [stem: *Arhytod-*]. Type genus: *Arhytodes* Reitter, 1882 [syn. of *Rhytus* Westwood, 1870].
Holozodini Raffray, 1900: 518 [stem: *Holozod-*]. Type genus: *Holozodus* Fairmaire, 1898.

### Tribe Attapseniini

Bruch, 1933

Attapseniini Bruch, 1933a: 26 [stem: *Attapseni-*]. Type genus: *Attapsenius* Bruch, 1933.

### Tribe Ctenistini

Blanchard, 1845

Cténistites Blanchard, 1845a: 306 [stem: *Ctenist-*]. Type genus: *Ctenistes* Reichenbach, 1816. Comment: original vernacular name available (Art. 11.7.2): first used in latinized form by Reitter (1882b: 183, as Ctenistini), generally accepted as in Chandler (2001: 484, as Ctenistini).
Chenniides Reitter, 1882b: 183 [stem: *Chenni-*]. Type genus: *Chennium* Latreille, 1807.

### Tribe Hybocephalini

Raffray, 1890

Hybocephalini Raffray, 1890: 83, in key [stem: *Hybocephal-*]. Type genus: *Hybocephalus* Motschulsky, 1851.
Mestogastrina Jakobson, 1910: 585 [stem: *Mestogastr-*]. Type genus: *Mestogaster* Schmidt-Göbel, 1838.

### Tribe Odontalgini

Jeannel, 1949

Odontalgini Jeannel, 1949a: 177 [stem: *Odontalg-*]. Type genus: *Odontalgus* Raffray, 1877.

### Tribe Pachygastrodini

Leleup, 1969

Pachygastrodini Leleup, 1969b: 282 [stem: *Pachygastrod-*]. Type genus: *Pachygastrodes* Leleup, 1969.

### Tribe Phalepsini

Jeannel, 1949

Phalepsini Jeannel, 1949a: 208 [stem: *Phaleps-*]. Type genus: *Phalepsus* Westwood, 1870.

### Tribe Pselaphini

Latreille, 1802

Pselaphii Latreille, 1802: 239 [stem: *Pselaph-*]. Type genus: *Pselaphus* Herbst, 1791.

### Tribe Schistodactylini

Raffray, 1890

Schistodactylini Raffray, 1890: 84, in key [stem: *Schistodactyl-*]. Type genus: *Schistodactylus* Raffray, 1883.

### Tribe Tmesiphorini

Jeannel, 1949

Tmesiphorini Jeannel, 1949a: 202 [stem: *Tmesiphor-*]. Type genus: *Tmesiphorus* J. L. LeConte, 1849.

### Tribe Tyrini

Reitter, 1882

Tyrides Reitter, 1882b: 184 [stem: *Tyr-*]. Type genus: *Tyrus* Aubé, 1833.

### Subtribe Centrophthalmina

Jeannel, 1949

Centrophthalmina Jeannel, 1949a: 209, in key [stem: *Centrophthalm-*]. Type genus: *Centrophthalmus* Schmidt-Göbel, 1838.
Petanopini Jeannel, 1954b: 102, in key [stem: *Petanop-*]. Type genus: *Petanops* Jeannel, 1954 [syn. of *Daveyia* Lea, 1912].

### Subtribe Janusculina

Cerruti, 1970

Janusculina Cerruti, 1970: 123 [stem: *Januscul-*]. Type genus: *Janusculus* Cerruti, 1970.

### Subtribe Somatipionina

Jeannel, 1949

Somatipionini Jeannel, 1949a: 208 [stem: *Somatipion-*]. Type genus: *Somatipion* L. W. Schaufuss, 1877.
Hamotini O. Park, 1951: 67, in key [stem: *Hamot-*]. Type genus: *Hamotus* Aubé, 1844.

### Subtribe Tyrina

Reitter, 1882

Tyrides Reitter, 1882b: 184 [stem: *Tyr-*]. Type genus: *Tyrus* Aubé, 1833.
Chalcoplectini Oke, 1925: 13 [stem: *Chalcoplect-*]. Type genus: *Chalcoplectus* Oke, 1925.
Ceophyllini O. Park, 1951: 67, in key [stem: *Ceophyll-*]. Type genus: *Ceophyllus* J. L. LeConte, 1849.

### Subfamily Phloeocharinae

Erichson, 1839

Phloeocharini Erichson, 1839a: 612 [stem: *Phloeochar-*]. Type genus: *Phloeocharis* Mannerheim, 1830. Comment: this family-group name was also used in the same year by Erichson (1839b: 31, as Phloeocharini) and Heer (1839b: 5, as Phloeocharina); for comments about the priority of these works see Newton and Thayer (1992: 24); current spelling maintained (Art. 29.5): incorrect stem formation in prevailing usage (should be *Phloeocharit*-).
Scotodytidae Saulcy, 1870: 90 [stem: *Scotodyt-*]. Type genus: *Scotodytes* Saulcy, 1865 [syn. of *Phloeocharis* Mannerheim, 1830].

### Subfamily Olisthaerinae

Thomson, 1858

Olisthaerini C. G. Thomson, 1858: 38 [stem: *Olisthaer-*]. Type genus: *Olisthaerus* Dejean, 1833.

### Subfamily Tachyporinae

MacLeay, 1825

Tachyporidae W. S. MacLeay, 1825: 49 [stem: *Tachypor-*]. Type genus: *Tachyporus* Gravenhorst, 1802.

### Tribe Deropini

Smetana, 1983

Deropsini Smetana, 1983: 272 [stem: *Derop-*]. Type genus: *Derops* Sharp, 1889. Comment: incorrect original stem formation, not in prevailing usage; correction of stem by Newton and Thayer (1992: 66).

### Tribe Megarthropsini

Cameron, 1919

Megarthropsini Cameron, 1919: 231 [stem: *Megarthrops-*]. Type genus: *Megarthropsis* Cameron, 1919. Comment: current spelling maintained (Art. 29.5): incorrect stem formation in prevailing usage (should be *Megarthropse*-).

### Tribe Mycetoporini

Thomson, 1859

Mycetoporides C. G. Thomson, 1859: 46 [stem: *Mycetopor-*]. Type genus: *Mycetoporus* Mannerheim, 1830 [placed on the Official List of Generic Names in Zoology (ICZN 1993c)].
Bolitobii G. H. Horn, 1877: 83 [stem: *Bolitobi-*]. Type genus: *Bolitobius* Leach, 1819.

### Tribe Tachyporini

MacLeay, 1825

Tachinidae Fleming, 1821: 49 [stem: *Tachinus-*]. Type genus: *Tachinus* Gravenhorst, 1802 [placed on the Official List of Generic Names in Zoology (ICZN 1993f)]. Comment: senior homonym of Tachinariae Robineau-Desvoidy, 1830 [Diptera: Tachinidae], stem emended to *Tachinus*- and Tachinusidae Fleming, 1821 placed on the Official List of Family-Group Names in Zoology (ICZN 1993f); Tachinidae Fleming, 1821 placed on the Official Index of Rejected and Invalid Family-Group Names in Zoology (ICZN 1993f).
Tachyporidae W. S. MacLeay, 1825: 49 [stem: *Tachypor-*]. Type genus: *Tachyporus* Gravenhorst, 1802 [placed on the Official List of Generic Names in Zoology (ICZN 1993f)]. Comment: family-group name given precedence over Tachinusidae Fleming, 1821 and placed on the Official List of Family-Group Names in Zoology (ICZN 1993f).
Symmixini Bernhauer, 1915: 56 [stem: *Symmix-*]. Type genus: *Symmixus* Bernhauer, 1915.
Euconosomini Cameron, 1918: 216 [stem: *Euconosomat-*]. Type genus: *Euconosoma* Cameron, 1918. Comment: incorrect original stem formation, not in prevailing usage.
Conosomini Jeannel and Jarrige, 1949: 335 [stem: *Conosomat-*]. Type genus: *Conosoma* Kraatz, 1857 [preoccupied genus name, not *Conosoma* Lenz, 1794 [Diptera]; syn. of *Tachinus* Gravenhorst, 1802]. Comment: permanently invalid (Art. 39): based on preoccupied type genus; incorrect original stem formation, not in prevailing usage.
Sepedophilini Ádám, 2001: 199 [stem: *Sepedophil-*]. Type genus: *Sepedophilus* Gistel, 1856. Comment: **syn. nov.**

### Tribe Vatesini

Seevers, 1958

Xenocephalini Wasmann, 1887: 411 [stem: *Xenocephal-*]. Type genus: *Xenocephalus* Wasmann, 1887 [preoccupied genus name, not *Xenocephalus* Kaup, 1858 [Pisces]; replaced by *Wasmannotherium* Bernhauer, 1921; syn. of *Vatesus* Sharp, 1876]. Comment: permanently invalid (Art. 39): based on preoccupied type genus.
Vatesini Seevers, 1958: 183 [stem: *Vates-*]. Type genus: *Vatesus* Sharp, 1876. Comment: replacement name for Xenocephalini Wasmann, 1887 because of the homonymy of the type genus.

### Subfamily Trichophyinae

Thomson, 1858

Trichophyini C. G. Thomson, 1858: 30 [stem: *Trichophy-*]. Type genus: *Trichophya* Mannerheim, 1830.

### Subfamily Habrocerinae

Mulsant and Rey, 1876

Habrocériens Mulsant and Rey, 1876: 210 [stem: *Habrocer-*]. Type genus: *Habrocerus* Erichson, 1839. Comment: original vernacular name available (Art. 11.7.2): first used in latinized form by G. H. Horn (1877: 83, as Habroceri), generally accepted as in Newton and Thayer (1992: 56, as Habrocerinae).

### Subfamily Aleocharinae

Fleming, 1821

Aleocharidae Fleming, 1821: 49 [stem: *Aleochar-*]. Type genus: *Aleochara* Gravenhorst, 1802. Comment: First Reviser (Aleocharinae Fleming, 1821 vs Lomechusinae Fleming, 1821) not determined, current usage maintained.

### Tribe Actocharini

Bernhauer and Schubert, 1911

Actochari Bernhauer and Schubert, 1911: 91 [stem: *Actochar-*]. Type genus: *Actocharis* Fauvel, 1869. Comment: originally proposed as a subtribe of Oxytelini Fleming, 1821; current spelling maintained (Art. 29.5): incorrect stem formation in prevailing usage (should be *Actocharit*-).

### Tribe Aenictoteratini

Kistner, 1993

Aenictoteratini Kistner, 1993: 242 [stem: *Aenictoterat-*]. Type genus: *Aenictoteras* W. M. Wheeler, 1932.

### Tribe Akatastopsisini

Pace, 2000

Akatastopsisini Pace, 2000a: 112 [stem: *Akatastopsis-*]. Type genus: *Akatastopsis* Pace, 2000.

### Tribe Aleocharini

Fleming, 1821

Aleocharidae Fleming, 1821: 49 [stem: *Aleochar-*]. Type genus: *Aleochara* Gravenhorst, 1802.

### Subtribe Aleocharina

Fleming, 1821

Aleocharidae Fleming, 1821: 49 [stem: *Aleochar-*]. Type genus: *Aleochara* Gravenhorst, 1802.
Piochardiae Fenyes, 1918: 20 [stem: *Piochardi-*]. Type genus: *Piochardia* Heyden, 1870.

### Subtribe Compactopediina

Kistner, 1970

Compactopedina Kistner, 1970b: 18 [stem: *Compactopedi-*]. Type genus: *Compactopedia* Kistner, 1970. Comment: incorrect original stem formation, not in prevailing usage; correction of stem by Newton and Thayer (1992: 47).

### Subtribe Hodoxenina

Kistner, 1970

Hodoxenina Kistner, 1970a: 12 [stem: *Hodoxen-*]. Type genus: *Hodoxenus* Kistner, 1970.

### Tribe Athetini

Casey, 1910

Athetae Casey, 1910: 2 [stem: *Athet-*]. Type genus: *Atheta* C. G. Thomson, 1858 [placed on the Official List of Generic Names in Zoology (ICZN 1961b)]. Comment: notice of a new application for the conservation of this name over the older name Callicerini Jakobson, 1908 by Gusarov was recently published in the Bulletin of Zoological Nomenclature (2010: 270; also see Appendix 6).

### Subtribe Athetina

Casey, 1910

Callicerina Jakobson, 1908: 448 [stem: *Callicer-*]. Type genus: *Callicerus* Gravenhorst, 1802. Comment: junior homonym of Callicerina Rondani, 1856 (type genus *Callicera* Panzer, 1806) in Diptera: Syrphidae; notice of a new application for the suppression of this name by Gusarov was recently published in the Bulletin of Zoological Nomenclature (2010: 270; also see Appendix 6).
Athetae Casey, 1910: 2 [stem: *Athet-*]. Type genus: *Atheta* C. G. Thomson, 1858 [placed on the Official List of Generic Names in Zoology (ICZN 1961b)]. Comment: notice of a new application for the conservation of this name over the older name Callicerina Jakobson, 1908 by Gusarov was recently published in the Bulletin of Zoological Nomenclature (2010: 270; also see Appendix 6).
Strigotae Casey, 1910: 176 [stem: *Strigot-*]. Type genus: *Strigota* Casey, 1910.
Plagiarthrini Cameron, 1926: 184 [stem: *Plagiarthrin-*]. Type genus: *Plagiarthrina* Keys, 1920 [syn. of *Atheta* C. G. Thomson, 1858]. Comment: incorrect original stem formation, not in prevailing usage.
Ischnopodini Hatch, 1957: 141 [stem: *Ischnopod-*]. Type genus: *Ischnopoda* sensu Westwood, 1838 [not *Ischnopoda* Stephens, 1835 (see ICZN 1961b); syn. of *Acrotona* C. G. Thomson, 1859]. Comment: based on misidentified type genus.
*Xenotae Seevers, 1978: 113 [stem: *Xenot-*]. Type genus: *Xenota* Mulsant and Rey, 1873 [subgenus of *Atheta* C. G. Thomson, 1858]. Comment: unavailable family-group name, proposed after 1930 without description or bibliographic reference to such a description (Art. 13.1).
Acrotonae Seevers, 1978: 97 [stem: *Acroton-*]. Type genus: *Acrotona* C. G. Thomson, 1859 [placed on the Official List of Generic Names in Zoology (ICZN 1961b)].
Geostibae Seevers, 1978: 126 [stem: *Geostib-*]. Type genus: *Geostiba* C. G. Thomson, 1858 [placed on the Official List of Generic Names in Zoology (ICZN 2005a)].
Dimetrotae Seevers, 1978: 102 [stem: *Dimetrot-*]. Type genus: *Dimetrota* Mulsant and Rey, 1873 [subgenus of *Atheta* C. G. Thomson, 1858].
*Hydrosmectina Muona, 1979: 23 [stem: *Hydrosmect-*]. Type genus: *Hydrosmecta* C. G. Thomson, 1858. Comment: unavailable family-group name, proposed after 1930 without description or bibliographic reference to such a description (Art. 13.1).
*Trichomicrina Muona, 1979: 23 [stem: *Trichomicr-*]. Type genus: *Trichomicra* Brundin, 1945. Comment: unavailable family-group name, proposed after 1930 without description or bibliographic reference to such a description (Art. 13.1).
*Dadobiina Muona, 1979: 23 [stem: *Dadobi-*]. Type genus: *Dadobia* C. G. Thomson, 1858. Comment: unavailable family-group name, proposed after 1930 without description or bibliographic reference to such a description (Art. 13.1).
*Amischina Muona, 1979: 25 [stem: *Amisch-*]. Type genus: *Amischa* C. G. Thomson, 1858. Comment: unavailable family-group name, proposed after 1930 without description or bibliographic reference to such a description (Art. 13.1).

### Subtribe Coptotermoeciina

Kistner and Pasteels, 1970

*Coptotermoeciina Seevers, 1957: 248 [stem: *Coptotermoeci-*]. Type genus: *Coptotermoecia* Oke, 1933. Comment: unavailable family-group name, proposed after 1930 without description or bibliographic reference to such a description (Art. 13.1).
Coptotermoeciina Kistner and Pasteels, 1970: 86 [stem: *Coptotermoeci-*]. Type genus: *Coptotermoecia* Oke, 1933.

### Subtribe Microceroxenina

Kistner, 1970

Microceroxenina Kistner, 1970c: 10 [stem: *Microceroxen-*]. Type genus: *Microceroxenus* Kistner, 1970.

### Subtribe Nasutiphilina

Kistner, 1970

Nasutiphilina Kistner, 1970d: 500 [stem: *Nasutiphil-*]. Type genus: *Nasutiphilus* Kistner, 1970.

### Subtribe Schistogeniina

Fenyes, 1918

Schistogeniae Fenyes, 1918: 18 [stem: *Schistogeni-*]. Type genus: *Schistogenia* Kraatz, 1857.

### Subtribe Taxicerina

Lohse, 1989

Taxicerina Lohse, 1989: 210 [stem: *Taxicer-*]. Type genus: *Taxicera* Mulsant and Rey, 1873.

### Subtribe Termitotelina

Kistner, 1970

*Termitotelina Seevers, 1957: 250 [stem: *Termitotel-*]. Type genus: *Termitotelus* Wasmann, 1908. Comment: unavailable family-group name, proposed after 1930 without description or bibliographic reference to such a description (Art. 13.1).
Termitotelina Kistner, 1970a: 4 [stem: *Termitotel-*]. Type genus: *Termitotelus* Wasmann, 1908.

### Subtribe Thamiaraeina

Fenyes, 1921

Thamiaraeini Fenyes, 1921: 34 [stem: *Thamiarae-*]. Type genus: *Thamiaraea* C. G. Thomson, 1858.

### Tribe Autaliini

Thomson, 1859

Autaliides C. G. Thomson, 1859: 30 [stem: *Autali-*]. Type genus: *Autalia* Leach, 1819.
Ophioglossae Fenyes, 1918: 18 [stem: *Ophiogloss-*]. Type genus: *Ophioglossa* Fauvel, 1866.
Rhopalogastra Fenyes, 1918: 17 [stem: *Rhopalogastr-*]. Type genus: *Rhopalogastrum* Bernhauer, 1912.

### Tribe Cordobanini

Bernhauer, 1910

Cordobanini Bernhauer, 1910: 386 [stem: *Cordoban-*]. Type genus: *Cordobanus* Bernhauer, 1910.

### Tribe Corotocini

Fenyes, 1918

Corotocini Fenyes, 1918: 61 [stem: *Corotoc-*]. Type genus: *Corotoca* Schiødte, 1847.

### Subtribe Abrotelina

Seevers, 1957

Abrotelina Seevers, 1957: 121 [stem: *Abrotel-*]. Type genus: *Abroteles* Casey, 1889.

### Subtribe Corotocina

Fenyes, 1918

Corotocini Fenyes, 1918: 61 [stem: *Corotoc-*]. Type genus: *Corotoca* Schiødte, 1847.
Termitomimini Fenyes, 1921: 33 [stem: *Termitomim-*]. Type genus: *Termitomimus* Trägårdh, 1907.

### Subtribe Eburniogastrina

Jacobson, Kistner and Pasteels, 1986

Eburniogastrina Jacobson et al., 1986: 27 [stem: *Eburniogastr-*]. Type genus: *Eburniogaster* Seevers, 1938.

### Subtribe Nasutitellina

Jacobson, Kistner and Pasteels, 1986

Nasutitellina Jacobson et al., 1986: 95 [stem: *Nasutitell-*]. Type genus: *Nasutitella* Pasteels, 1967.

### Subtribe Sphuridaethina

Pace, 1988

Sphuridaethina Pace, 1988: 980 [stem: *Sphuridaeth-*]. Type genus: *Sphuridaethes* Pace, 1988.

### Subtribe Termitocharina

Seevers, 1957

Termitocharina Seevers, 1957: 64, in key [stem: *Termitochar-*]. Type genus: *Termitochara* Wasmann, 1893.

### Subtribe Termitocupidina

Jacobson, Kistner and Pasteels, 1986

Termitocupidina Jacobson et al., 1986: 35 [stem: *Termitocupid-*]. Type genus: *Termitocupidus* Jacobson et al., 1986.

### Subtribe Termitogastrina

Bernhauer and Scheerpeltz, 1926

Termitogastri Bernhauer and Scheerpeltz, 1926: 734 [stem: *Termitogastr-*]. Type genus: *Termitogaster* Casey, 1889.
Termitellici Jacobson et al., 1986: 47 [stem: *Termitell-*]. Type genus: *Termitella* Wasmann, 1911. Comment: proposed as an infratribe, a rank not used here.
Termitogastrici Jacobson et al., 1986: 58 [stem: *Termitogastr-*]. Type genus: *Termitogaster* Casey, 1889. Comment: family-group name proposed as a new taxon, without reference to Termitogastri Bernhauer and Scheerpeltz, 1926; proposed as an infratribe, a rank not used here.

### Subtribe Termitoiceina

Jacobson, Kistner and Pasteels, 1986

Termitoiceina Jacobson et al., 1986: 84 [stem: *Termitoice-*]. Type genus: *Termitoiceus* Silvestri, 1901.

### Subtribe Termitopithina

Jacobson, Kistner and Pasteels, 1986

Termitopithina Jacobson et al., 1986: 80 [stem: *Termitopith-*]. Type genus: *Termitopithus* Seevers, 1957.

### Subtribe Termitoptochina

Fenyes, 1921

Termitoptochini Fenyes, 1921: 33 [stem: *Termitoptoch-*]. Type genus: *Termitoptochus* Silvestri, 1911.
Termitoptochina Jacobson et al., 1986: 96 [stem: *Termitoptoch-*]. Type genus: *Termitoptochus* Silvestri, 1911. Comment: family-group name proposed as new without reference to Termitoptochini Fenyes, 1921.

### Subtribe Timeparthenina

Fenyes, 1921

Timeparthenini Fenyes, 1921: 34 [stem: *Timeparthen-*]. Type genus: *Timeparthenus* Silvestri, 1901.

### Tribe Crematoxenini

Mann, 1921

Crematoxenini Mann, 1921: 547 [stem: *Crematoxen-*]. Type genus: *Crematoxenus* Mann, 1921.
Pulicomorphini Mann, 1924: 87 [stem: *Pulicomorph-*]. Type genus: *Pulicomorpha* Mann, 1924.
Philacamatini Bruch, 1933b: 206 [stem: *Philacamat-*]. Type genus: *Philacamatus* Bruch, 1933.

### Tribe Cryptonotopseini

Pace, 2003

Cryptonotopsisini Pace, 2003: 38 [stem: *Cryptonotopse*-]. Type genus: *Cryptonotopsis* Pace, 2003. Comment: incorrect original stem formation, not in prevailing usage.

### Tribe Deinopsini

Sharp, 1883

Deinopsini Sharp, 1883: 294 [stem: *Deinops-*]. Type genus: *Deinopsis* A. Matthews, 1838. Comment: current spelling maintained (Art. 29.5): incorrect original stem formation in prevailing usage (should be *Deinopse*-).
Adinopsini Cameron, 1919: 242 [stem: *Adinopse-*]. Type genus: *Adinopsis* Cameron, 1919. Comment: incorrect original stem formation, not in prevailing usage.

### Tribe Diestotini

Mulsant and Rey, 1871

Diestotates Mulsant and Rey, 1871c: 96 [stem: *Diestot-*]. Type genus: *Diestota* Mulsant and Rey, 1870. Comment: original vernacular name available (Art. 11.7.2): first used in latinized form by Lohse (1989: 186, as Diestotini), generally accepted as in Newton and Thayer (1992: 49, as Diestotini).
Elachistarthronini Notman, 1920: 714 [stem: *Elachistarthr-*]. Type genus: *Elachistarthron* Notman, 1920 [syn. of *Diestota* Mulsant and Rey, 1870]. Comment: incorrect original stem formation, not in prevailing usage; correction of stem by Newton and Thayer (1992: 49).

### Tribe Diglottini

Jakobson, 1909

Diglossaires Mulsant and Rey, 1873a: 73 [stem: *Digloss-*]. Type genus: *Diglossa* Haliday, 1837 [preoccupied genus name, not *Diglossa* Wagler, 1832 [Aves]; syn. of *Diglotta* Champion, 1899]. Comment: original vernacular name available (Art. 11.7.2): first used in latinized form and generally accepted as in Ganglbauer (1895: 313, as Diglossini); permanently invalid (Art. 39): based on preoccupied type genus.
Diglottina Jakobson, 1909: 529 [stem: *Diglott-*]. Type genus: *Diglotta* Champion, 1899. Comment: published 4 March 1909; this family-group name was also used in the same year by Eichelbaum (1909 [before 26 December]: 204, as Diglottini).

### Tribe Digrammini

Fauvel, 1900

Digrammini Fauvel, 1900: 123 [stem: *Digramm-*]. Type genus: *Digrammus* Fauvel, 1900.

### Tribe Dorylogastrini

Wasmann, 1916

Dorylogastrini Wasmann, 1916a: 103 [stem: *Dorylogastr-*]. Type genus: *Dorylogaster* Wasmann, 1904.

### Tribe Dorylomimini

Wasmann, 1916

Dorylomimini Wasmann, 1916a: 99 [stem: *Dorylomim-*]. Type genus: *Dorylomimus* Wasmann, 1902.

### Tribe Drepanoxenini

Kistner and Watson, 1972

Drepanoxenini Kistner and Watson, 1972: 2 [stem: *Drepanoxen-*]. Type genus: *Drepanoxenus* Kistner and Watson, 1972.

### Tribe Ecitocharini

Seevers, 1965

Ecitocharini Seevers, 1965: 287 [stem: *Ecitochar-*]. Type genus: *Ecitochara* Wasmann, 1887.

### Tribe Ecitogastrini

Fenyes, 1918

Ecitogastrini Fenyes, 1918: 74 [stem: *Ecitogastr-*]. Type genus: *Ecitogaster* Wasmann, 1899.

### Tribe Eusteniamorphini

Bernhauer and Scheerpeltz, 1926

Eusteniamorphini Bernhauer and Scheerpeltz, 1926: 517 [stem: *Eusteniamorph-*]. Type genus: *Eusteniamorpha* Cameron, 1920.

### Tribe Falagriini

Mulsant and Rey, 1873

Falagriates Mulsant and Rey, 1873b: 8 [stem: *Falagri-*]. Type genus: *Falagria* Leach, 1819. Comment: original vernacular name available (Art. 11.7.2): first used in latinized form by Seidlitz (1874 [Gatt.]: 71, as Falagriina), generally accepted as in Newton and Thayer (1992: 50, as Falagriini).

### Tribe Feldini

Kistner, 1972

*Feldina Seevers, 1957: 236 [stem: *Feld-*]. Type genus: *Felda* Blackwelder, 1952. Comment: unavailable family-group name, proposed after 1930 without description or bibliographic reference to such a description (Art. 13.1).
Feldini Kistner, 1972: 2 [stem: *Feld-*]. Type genus: *Felda* Blackwelder, 1952.

### Tribe Gymnusini

Heer, 1839

Gymnusida Heer, 1839a: 302 [stem: *Gymnus-*]. Type genus: *Gymnusa* Gravenhorst, 1806. Comment: this family-group name was also used in the same year by Heer (1839b: 49, as Gymnusida); for comments about the priority of these works see Newton and Thayer (1992: 24).

### Tribe Himalusini

Klimaszewski, Pace and Center, 2010

Himalusini Klimaszewski et al., 2010: 3 [stem: *Himalus-*]. Type genus: *Himalusa* Pace, 2006.

### Tribe Homalotini

Heer, 1839

Homalotida Heer, 1839a: 305 [stem: *Homalot-*]. Type genus: *Homalota* Mannerheim, 1830.

### Subtribe Bolitocharina

Thomson, 1859

Bolitocharides C. G. Thomson, 1859: 31 [stem: *Bolitochar-*]. Type genus: *Bolitochara* Mannerheim, 1830 [placed on the Official List of Generic Names in Zoology (ICZN 1961a)]. Comment: First Revisers found (Bolitocharini C. G. Thomson, 1859 vs Euryusini C. G. Thomson, 1859) are Newton and Thayer (1992: 50); Bolitocharini C. G. Thomson, 1859 placed on the Official List of Family-Group Names in Zoology (ICZN 1961a); the original spelling Bolitocharides C. G. Thomson, 1859 and several subsequent spellings of this name placed on the Official Index of Rejected and Invalid Family-Group Names in Zoology (ICZN 1961a).
Euryusides C. G. Thomson, 1859: 40 [stem: *Euryus-*]. Type genus: *Euryusa* Erichson, 1837.
Sipaliae Casey, 1910: 167 [stem: *Sipali-*]. Type genus: *Sipalia* Mulsant and Rey, 1853 [placed on the Official Index of Rejected and Invalid Generic Names in Zoology (ICZN 2005d)]. Comment: permanently invalid (Art. 39): based on suppressed type genus.
Heterotae Fenyes, 1918: 18 [stem: *Heterot-*]. Type genus: *Heterota* Mulsant and Rey, 1873.
Leptusae Fenyes, 1918: 18 [stem: *Leptus-*]. Type genus: *Leptusa* Kraatz, 1856 [placed on the Official List of Generic Names in Zoology (ICZN 2005d)].
Nanoglossae Fenyes, 1918: 20 [stem: *Nanogloss-*]. Type genus: *Nanoglossa* Fauvel, 1868 [subgenus of *Leptusa* Kraatz, 1856].
Ditropaliini Hatch, 1957: 134, in key, 147 [stem: *Ditropali-*]. Type genus: *Ditropalia* Casey, 1906 [syn. of *Bolitochara* Mannerheim, 1830]. Comment: this taxon was named correctly Ditropaliini in the text on p. 147 but incorrect as Bolitocharini in the key on p. 134.

### Subtribe Dinardopsina

Bernhauer and Scheerpeltz, 1926

Dinardopses Bernhauer and Scheerpeltz, 1926: 525, 804 [stem: *Dinardops-*]. Type genus: *Dinardopsis* Bruch, 1917. Comment: the erroneous spelling Dinardopsis was used in the catalogue on p. 525 but this was corrected to Dinardopses on page 804 of the same work; current spelling maintained (Art. 29.5): incorrect stem formation in prevailing usage (should be *Dinardopse*-).

### Subtribe Gyrophaenina

Kraatz, 1856

Gyrophaenini Kraatz, 1856: 351 [stem: *Gyrophaen-*]. Type genus: *Gyrophaena* Mannerheim, 1830.

### Subtribe Homalotina

Heer, 1839

Homalotida Heer, 1839a: 305 [stem: *Homalot-*]. Type genus: *Homalota* Mannerheim, 1830. Comment: this family-group name was also used in the same year by Heer (1839b: 50, as Homalotida); for comments about the priority of these works see Newton and Thayer (1992: 24).
Thecturotae Fenyes, 1918: 18 [stem: *Thecturot-*]. Type genus: *Thecturota* Casey, 1893.
*Cypheae Seevers, 1978: 272 [stem: *Cyphe-*]. Type genus: *Cyphea* Fauvel, 1863. Comment: unavailable family-group name, proposed after 1930 without description or bibliographic reference to such a description (Art. 13.1).

### Subtribe Silusina

Fenyes, 1918

Silusae Fenyes, 1918: 17 [stem: *Silus-*]. Type genus: *Silusa* Erichson, 1837.

### Tribe Hoplandriini

Casey, 1910

Hoplandriae Casey, 1910: 170 [stem: *Hoplandri-*]. Type genus: *Hoplandria* Kraatz, 1857.

### Subtribe Hoplandriina

Casey, 1910

Hoplandriae Casey, 1910: 170 [stem: *Hoplandri-*]. Type genus: *Hoplandria* Kraatz, 1857.

### Subtribe Platandriina

Hanley, 2002

Platandriina Hanley, 2002: 317 [stem: *Platandri-*]. Type genus: *Platandria* Casey, 1893.

### Subtribe Pseudoplandriina

Hanley, 2002

Pseudoplandriina Hanley, 2002: 317 [stem: *Pseudoplandri-*]. Type genus: *Pseudoplandria* Fenyes, 1921.

### Tribe Hygronomini

Thomson, 1859

Hygronomides C. G. Thomson, 1859: 31 [stem: *Hygronom-*]. Type genus: *Hygronoma* Erichson, 1837.

### Subtribe Hygronomina

Thomson, 1859

Hygronomides C. G. Thomson, 1859: 31 [stem: *Hygronom-*]. Type genus: *Hygronoma* Erichson, 1837.

### Subtribe Saphoglossina

Bernhauer and Scheerpeltz, 1926

Saphoglossae Bernhauer and Scheerpeltz, 1926: 521 [stem: *Saphogloss-*]. Type genus: *Saphoglossa* Sharp, 1883.

### Tribe Hypocyphtini

Laporte, 1835

Hypocyphtidae Laporte, 1835a: 135 [stem: *Hypocypht-*]. Type genus: *Hypocyphtus* Gyllenhal, 1827 [syn. of *Cypha* Leach, 1819].
Oligotides C. G. Thomson, 1859: 30 [stem: *Oligot-*]. Type genus: *Oligota* Mannerheim, 1830.
Nematoscelini Fenyes, 1921: 33 [stem: *Nematoscelid-*]. Type genus: *Nematoscelis* Wollaston, 1867. Comment: incorrect original stem formation, not in prevailing usage.
*Cyphinae Lohse, 1974: 7 [stem: *Cyph-*]. Type genus: *Cypha* Leach, 1819. Comment: family-group name unavailable (Art. 11.6): originally published as synonym and not made available subsequently; also see Cyphini Lacordaire, 1863 (type genus *Cyphus* Germar, 1824) in Coleoptera: Curculionidae.

### Tribe Leucocraspedini

Fenyes, 1921

Leucocraspedini Fenyes, 1921: 34 [stem: *Leucocrasped-*]. Type genus: *Leucocraspedum* Kraatz, 1859.

### Tribe Liparocephalini

Fenyes, 1918

Liparocephali Fenyes, 1918: 18 [stem: *Liparocephal-*]. Type genus: *Liparocephalus* Mäklin, 1853.

### Tribe Lomechusini

Fleming, 1821

Lomechusidae Fleming, 1821: 49 [stem: *Lomechus-*]. Type genus: *Lomechusa* Gravenhorst, 1806.

### Subtribe Aenictobiina

Kistner, 1997

Aenictobiina Kistner, 1997: 174 [stem: *Aenictobi-*]. Type genus: *Aenictobia* Seevers, 1953.

### Subtribe Lomechusina

Fleming, 1821

Lomechusidae Fleming, 1821: 49 [stem: *Lomechus-*]. Type genus: *Lomechusa* Gravenhorst, 1806.
Xenodusae Seevers, 1978: 155 [stem: *Xenodus-*]. Type genus: *Xenodusa* Wasmann, 1894.

### Subtribe Myrmedoniina

Thomson, 1867

Myrmedoniides C. G. Thomson, 1867: 209 [stem: *Myrmedoni-*]. Type genus: *Myrmedonia* Erichson, 1837 [syn. of *Zyras* Stephens, 1835].
*Myrméciates Mulsant and Rey, 1873b: 98 [stem: *Myrmoeci-*]. Type genus: *Myrmoecia* Mulsant and Rey, 1873. Comment: original vernacular name unavailable (Art. 11.7.2): not subsequently latinized; incorrect original stem formation, not in prevailing usage.
Zyrini Bradley, 1930: 83 [stem: *Zyr-*]. Type genus: *Zyras* Stephens, 1835 [placed on the Official List of Generic Names in Zoology (ICZN 1961a)].
*Ecitoporae Seevers, 1978: 13 [stem: *Ecitopor-*]. Type genus: *Ecitopora* Wasmann, 1887. Comment: unavailable family-group name, proposed after 1930 without description or bibliographic reference to such a description (Art. 13.1).
*Tetradoniae Seevers, 1978: 13 [stem: *Tetradoni-*]. Type genus: *Tetradonia* Wasmann, 1894. Comment: unavailable family-group name, proposed after 1930 without description or bibliographic reference to such a description (Art. 13.1).
*Dinocorynae Seevers, 1978: 13 [stem: *Dinocoryn-*]. Type genus: *Dinocoryna* Casey, 1893. Comment: unavailable family-group name, proposed after 1930 without description or bibliographic reference to such a description (Art. 13.1).

### Subtribe Termitozyrina

Seevers, 1957

Termitozyrina Seevers, 1957: 62, in key [stem: *Termitozyr-*]. Type genus: *Termitozyras* Seevers, 1957.

### Tribe Masuriini

Cameron, 1939

Masuriini Cameron, 1939: 24 [stem: *Masuri-*]. Type genus: *Masuria* Cameron, 1928.

### Tribe Mesoporini

Cameron, 1959

Mesoporinae Cameron, 1959: 119 [stem: *Mesopor-*]. Type genus: *Mesoporus* Cameron, 1959.

### Tribe Mimanommatini

Wasmann, 1912

Mimanommatinae Wasmann, 1912: 478 [stem: *Mimanommat-*]. Type genus: *Mimanomma* Wasmann, 1912.

### Subtribe Dorylophilina

Fenyes, 1921

Dorylophilini Fenyes, 1921: 34 [stem: *Dorylophil-*]. Type genus: *Dorylophila* Wasmann, 1904.
Deremini Seevers, 1965: 294 [stem: *Derem-*]. Type genus: *Derema* Fauvel, 1899.

### Subtribe Mimanommatina

Wasmann, 1912

Mimanommatinae Wasmann, 1912: 478 [stem: *Mimanommat-*]. Type genus: *Mimanomma* Wasmann, 1912.

### Tribe Mimecitini

Wasmann, 1917

Mimecitonini Wasmann, 1917: 325 [stem: *Mimecit-*]. Type genus: *Mimeciton* Wasmann, 1893.

### Subtribe Labidopullina

Jacobson and Kistner, 1991

Labidopullina Jacobson and Kistner, 1991: 7 [stem: *Labidopull-*]. Type genus: *Labidopullus* Borgmeier, 1958.

### Subtribe Leptanillophilina

Fenyes, 1918

Leptanillophilini Fenyes, 1918: 59 [stem: *Leptanillophil-*]. Type genus: *Leptanillophilus* Holmgren, 1908.

### Subtribe Mimecitina

Wasmann, 1917

Mimecitonini Wasmann, 1917: 325 [stem: *Mimecit-*]. Type genus: *Mimeciton* Wasmann, 1893. Comment: incorrect original stem formation, not in prevailing usage; correction of stem by Newton and Thayer (1992: 52).

### Subtribe Mimonillina

Bernhauer and Scheerpeltz, 1926

Mimonillae Bernhauer and Scheerpeltz, 1926: 518 [stem: *Mimonill-*]. Type genus: *Mimonilla* Wasmann, 1913.

### Tribe Myllaenini

Ganglbauer, 1895

Myllaenini Ganglbauer, 1895: 317 [stem: *Myllaen-*]. Type genus: *Myllaena* Erichson, 1837.
Dimonomerini Cameron, 1933: 103 [stem: *Dimonomer-*]. Type genus: *Dimonomera* Cameron, 1933.

### Tribe Oxypodini

Thomson, 1859

Oxypodides C. G. Thomson, 1859: 36 [stem: *Oxypod-*]. Type genus: *Oxypoda* Mannerheim, 1830 [placed on the Official List of Generic Names in Zoology (ICZN 1957)]. Comment: First Revisers found (Oxypodini C. G. Thomson, 1859 vs Tachyusini C. G. Thomson, 1859 vs Ocaleini C. G. Thomson, 1859 vs Phloeoporini C. G. Thomson, 1859) are Newton and Thayer (1992: 53); name placed on the Official List of Family-Group Names in Zoology (ICZN 1957).

### Subtribe Aphytopodina

Bernhauer and Scheerpeltz, 1926

Aphytopi Bernhauer and Scheerpeltz, 1926: 740 [stem: *Aphytopod-*]. Type genus: *Aphytopus* Sharp, 1886. Comment: incorrect original stem formation, not in prevailing usage; correction of stem by Newton and Thayer (1992: 53).

### Subtribe Blepharhymenina

Klimaszewski and Peck, 1986

*Blepharhymeni Seevers, 1978: 82 [stem: *Blepharhymen-*]. Type genus: *Blepharhymenus* Solier, 1849. Comment: unavailable family-group name, proposed after 1930 without description or bibliographic reference to such a description (Art. 13.1).
Blepharrhymeni Klimaszewski and Peck, 1986: 58 [stem: *Blepharhymen-*]. Type genus: *Blepharhymenus* Solier, 1849 [as *Blepharrhymenus*, unjustified emendation of genus name by Gemminger and Harold (1868b: 505), not in prevailing usage]. Comment: incorrect original stem formation, not in prevailing usage; correction of stem by Newton and Thayer (1992: 53).

### Subtribe Dinardina

Mulsant and Rey, 1873

Dinardaires Mulsant and Rey, 1873a: 6 [stem: *Dinard-*]. Type genus: *Dinarda* Leach, 1819. Comment: original vernacular name available (Art. 11.7.2): first used in latinized form by Wasmann (1904a: 218, as Dinardini), generally accepted as in Newton and Thayer (1992: 53, as Dinardina).
*Homéusates Mulsant and Rey, 1874: 286 [stem: *Homoeus-*]. Type genus: *Homoeusa* Kraatz, 1856. Comment: original vernacular name unavailable (Art. 11.7.2): not subsequently latinized; incorrect original stem formation, not in prevailing usage.
Decusini Fenyes, 1918: 19 [stem: *Decus-*]. Type genus: *Decusa* Casey, 1900.

### Subtribe Meoticina

Seevers, 1978

Meoticae Seevers, 1978: 78 [stem: *Meotic-*]. Type genus: *Meotica* Mulsant and Rey, 1873.

### Subtribe Oxypodina

Thomson, 1859

Oxypodides C. G. Thomson, 1859: 36 [stem: *Oxypod-*]. Type genus: *Oxypoda* Mannerheim, 1830 [placed on the Official List of Generic Names in Zoology (ICZN 1957)]. Comment: First Reviser (Oxypodina C. G. Thomson, 1859 vs Ocaleina C. G. Thomson, 1859 vs Phloeoporina C. G. Thomson, 1859) not determined, current usage maintained; placed on the Official List of Family-Group Names in Zoology (ICZN 1957).
Ocaleides C. G. Thomson, 1859: 38 [stem: *Ocale-*]. Type genus: *Ocalea* Erichson, 1837.
Phloeoporides C. G. Thomson, 1859: 33 [stem: *Phloeopor-*]. Type genus: *Phloeopora* Erichson, 1837.
Ocyusates Mulsant and Rey, 1874: 286 [stem: *Ocyus-*]. Type genus: *Ocyusa* Kraatz, 1856. Comment: original vernacular name available (Art. 11.7.2): first used in latinized form by Fenyes (1918: 20, as Ocyusae), generally accepted as in Newton and Thayer (1992: 53, as Ocyusina).
Calodérates Mulsant and Rey, 1874: 286 [stem: *Caloder-*]. Type genus: *Calodera* Mannerheim, 1830. Comment: original vernacular name available (Art. 11.7.2): first used in latinized form by Fenyes (1918: 20, as Caloderae), generally accepted as in Ádám (2001:142, as Caloderini).
Microglottae Fenyes, 1918: 20 [stem: *Microglott-*]. Type genus: *Microglotta* Kraatz, 1862 [syn. of *Haploglossa* Kraatz, 1856].
Phloeoporini Cameron, 1939: 562 [stem: *Phloeopor-*]. Type genus: *Phloeopora* Erichson, 1837. Comment: proposed as new without reference to Phloeoporides C. G. Thomson, 1859.

### Subtribe Tachyusina

Thomson, 1859

Tachyusides C. G. Thomson, 1859: 34 [stem: *Tachyus-*]. Type genus: *Tachyusa* Erichson, 1837 [placed on the Official List of Generic Names in Zoology (ICZN 1961b)].

### Tribe Oxypodinini

Fenyes, 1921

Oxypodinini Fenyes, 1918: 18 [stem: *Oxypodin-*]. Type genus: *Oxypodinus* Bernhauer, 1901.
Heterotaxini Fenyes, 1921: 33 [stem: *Heterotax-*]. Type genus: *Heterotaxus* Bernhauer, 1915.

### Tribe Paglini

Newton and Thayer, 1992

Pachyglossini Fenyes, 1918: 60 [stem: *Pachygloss-*]. Type genus: *Pachyglossa* Fauvel, 1868 [preoccupied genus name, not *Pachyglossa* Hodgson, 1843 [Aves]; syn. of *Pagla* Blackwelder, 1952]. Comment: permanently invalid (Art. 39): based on preoccupied type genus.
Paglini Newton and Thayer, 1992: 54 [stem: *Pagl-*]. Type genus: *Pagla* Blackwelder, 1952. Comment: replacement name for Pachyglossini Fenyes, 1918 because of the homonymy of the type genus.

### Tribe Paradoxenusini

Bruch, 1937

Paradoxenusini Bruch, 1937: 354 [stem: *Paradoxenus-*]. Type genus: *Paradoxenusa* Bruch, 1937.

### Tribe Pediculotini

Ádám, 1987

Pediculotini Ádám, 1987: 156 [stem: *Pediculot-*]. Type genus: *Pediculota* Ádám, 1987.

### Tribe Philotermitini

Seevers, 1957

Philotermitini Seevers, 1957: 63, in key [stem: *Philotermit-*]. Type genus: *Philotermes* Kraatz, 1857.

### Tribe Phyllodinardini

Wasmann, 1916

Phyllodinardini Wasmann, 1916a: 105 [stem: *Phyllodinard-*]. Type genus: *Phyllodinarda* Wasmann, 1916.

### Tribe Phytosini

Thomson, 1867

Phytosides C. G. Thomson, 1867: 206 [stem: *Phytos-*]. Type genus: *Phytosus* Curtis, 1838.

### Tribe Placusini

Mulsant and Rey, 1871

Placusates Mulsant and Rey, 1871c: 102 [stem: *Placus-*]. Type genus: *Placusa* Erichson, 1837. Comment: original vernacular name available (Art. 11.7.2): first used in latinized form by Fenyes (1918: 17, as Placusae), generally accepted as in Newton and Thayer (1992: 54, as Placusini).
*Euvirae Seevers, 1978: 272 [stem: *Euvir-*]. Type genus: *Euvira* Sharp, 1883. Comment: unavailable family-group name, proposed after 1930 without description or bibliographic reference to such a description (Art. 13.1).

### Tribe Pronomaeini

Mulsant and Rey, 1873

Pronoméates Mulsant and Rey, 1873b: 8 [stem: *Pronomae-*]. Type genus: *Pronomaea* Erichson, 1837. Comment: original vernacular name available (Art. 11.7.2): first used in latinized form by Ganglbauer (1895: 315, as Pronomaeini), generally accepted as in Newton and Thayer (1992: 54, as Pronomaeini); incorrect original stem formation, not in prevailing usage.

### Tribe Pseudoperinthini

Cameron, 1939

Pseudoperinthinae Cameron, 1939: 1 [stem: *Pseudoperinth-*]. Type genus: *Pseudoperinthus* Wasmann, 1916.

### Tribe Pygostenini

Fauvel, 1899

Pygostenini Fauvel, 1899: 5 [stem: *Pygosten-*]. Type genus: *Pygostenus* Kraatz, 1858.
Sympolemonini Fenyes, 1918: 51 [stem: *Sympolemont-*]. Type genus: *Sympolemon* Wasmann, 1900. Comment: incorrect original stem formation, not in prevailing usage.

### Tribe Sahlbergiini

Kistner, 1993

Sahlbergiini Kistner, 1993: 315 [stem: *Sahlbergi-*]. Type genus: *Sahlbergius* Bernhauer, 1927.

### Tribe Sceptobiini

Seevers, 1978

Sceptobiini Seevers, 1978: 148 [stem: *Sceptobi-*]. Type genus: *Sceptobius* Sharp, 1883.

### Tribe Skatitoxenini

Kistner and Pasteels, 1969

Skatitoxenini Kistner and Pasteels, 1969: 1190 [stem: *Skatitoxen-*]. Type genus: *Skatitoxenus* Kistner and Pasteels, 1969.

### Tribe Termitodiscini

Wasmann, 1904

Termitodiscini Wasmann, 1904b: 656 [stem: *Termitodisc-*]. Type genus: *Termitodiscus* Wasmann, 1899.

### Subtribe Athexeniina

Pace, 2000

Athexenina Pace, 2000b: 336, in key [stem: *Athexeni-*]. Type genus: *Athexenia* Pace, 1999. Comment: incorrect original stem formation, not in prevailing usage.

### Subtribe Termitodiscina

Wasmann, 1904

Termitodiscini Wasmann, 1904b: 656 [stem: *Termitodisc-*]. Type genus: *Termitodiscus* Wasmann, 1899.

### Tribe Termitohospitini

Seevers, 1941

Termitohospini Seevers, 1941: 331 [stem: *Termitohospit-*]. Type genus: *Termitohospes* Seevers, 1941.

### Subtribe Hetairotermitina

Seevers, 1957

Hetairotermitina Seevers, 1957: 191 [stem: *Hetairotermit-*]. Type genus: *Hetairotermes* Cameron, 1920.

### Subtribe Termitohospitina

Seevers, 1941

Termitohospini Seevers, 1941: 331 [stem: *Termitohospit-*]. Type genus: *Termitohospes* Seevers, 1941. Comment: incorrect original stem formation, not in prevailing usage; correction of stem by Seevers (1957: 191).

### Tribe Termitonannini

Fenyes, 1918

Termitonannini Fenyes, 1918: 75 [stem: *Termitonann-*]. Type genus: *Termitonannus* Wasmann, 1902.

### Subtribe Perinthina

Bernhauer and Scheerpeltz, 1926

Perinthi Bernhauer and Scheerpeltz, 1926: 521 [stem: *Perinth-*]. Type genus: *Perinthus* Casey, 1889.
*Poduroideae Scheerpeltz, 1934: 1537 [stem: *Poduroid-*]. Type genus: *Poduroides* Mann, 1926. Comment: unavailable family-group name, proposed after 1930 without description or bibliographic reference to such a description (Art. 13.1).

### Subtribe Termitonannina

Fenyes, 1918

Termitonannini Fenyes, 1918: 75 [stem: *Termitonann-*]. Type genus: *Termitonannus* Wasmann, 1902.

### Tribe Termitopaediini

Seevers, 1957

Termitopaediini Seevers, 1957: 214 [stem: *Termitopaedi-*]. Type genus: *Termitopaedia* Wasmann, 1911.
Termitondina Seevers, 1957: 63, in key [stem: *Termitond-*]. Type genus: *Termitonda* Seevers, 1957. Comment: as Termitendina, name spelled correctly on page 238.

### Tribe Termitusini

Fenyes, 1918

Termitusae Fenyes, 1918: 18 [stem: *Termitus-*]. Type genus: *Termitusa* Wasmann, 1905.

### Subtribe Termitospectrina

Seevers, 1957

Termitospectrina Seevers, 1957: 191 [stem: *Termitospectr-*]. Type genus: *Termitospectrum* Mann, 1926.

### Subtribe Termitusina

Fenyes, 1918

Termitusae Fenyes, 1918: 18 [stem: *Termitus-*]. Type genus: *Termitusa* Wasmann, 1905.

### Tribe Trichopseniini

LeConte and Horn, 1883

Trichopsenii J. L. LeConte and G. H. Horn, 1883: 100 [stem: *Trichopseni-*]. Type genus: *Trichopsenius* G. H. Horn, 1877.
Termitopsenini Wasmann, 1916b: 196 [stem: *Termitopseni-*]. Type genus: *Termitopsenius* Wasmann, 1902. Comment: incorrect original stem formation, not in prevailing usage.
Schizelythrinae Kemner, 1925: 122 [stem: *Schizelythr-*]. Type genus: *Schizelythron* Kemner, 1925.

### Tribe Trilobitideini

Fauvel, 1899

Trilobitideidae Fauvel, 1899: 3 [stem: *Trilobitide-*]. Type genus: *Trilobitideus* Raffray, 1898.

### Subfamily Trigonurinae

Reiche, 1866

Trigonurides Reiche, 1866: 642 [stem: *Trigonur-*]. Type genus: *Trigonurus* Mulsant, 1847.

### Subfamily Apateticinae

Fauvel, 1895

Apateticae Fauvel, 1895: 190 [stem: *Apatetic-*]. Type genus: *Apatetica* Westwood, 1848.

### Subfamily Scaphidiinae

Latreille, 1806

Scaphidilia Latreille, 1806: 3 [stem: *Scaphidi-*]. Type genus: *Scaphidium* A. G. Olivier, 1790.

### Tribe Cypariini

Achard, 1924

Cypariini Achard, 1924: 28 [stem: *Cypari-*]. Type genus: *Cyparium* Erichson, 1845.

### Tribe Scaphidiini

Latreille, 1806

Scaphidilia Latreille, 1806: 3 [stem: *Scaphidi-*]. Type genus: *Scaphidium* A. G. Olivier, 1790. Comment: incorrect original stem formation, not in prevailing usage.
Cerambyciscaphini Pic, 1915: 30 [stem: *Cerambyciscaph-*]. Type genus: *Cerambyciscapha* Pic, 1915.
Diateliitae Achard, 1924: 28 [stem: *Diateli-*]. Type genus: *Diatelium* Pascoe, 1863.

### Tribe Scaphiini

Achard, 1924

Scaphiitae Achard, 1924: 27 [stem: *Scaphi-*]. Type genus: *Scaphium* Kirby, 1837.

### Tribe Scaphisomatini

Casey, 1893

Scaphisomini Casey, 1893: 511 [stem: *Scaphisomat-*]. Type genus: *Scaphisoma* Leach, 1815. Comment: incorrect original stem formation, not in prevailing usage; correction of stem by Newton and Thayer (1992: 64).
Heteroscaphini Achard, 1914: 395 [stem: *Heteroscaph-*]. Type genus: *Heteroscapha* Achard, 1914 [syn. of *Bironium* Csiki, 1909].
Cyparellini Achard, 1924: 28 [stem: *Cyparell-*]. Type genus: *Cyparella* Achard, 1924 [syn. of *Baeocera* Erichson, 1845].
Baeoceritae Achard, 1924: 30 [stem: *Baeocer-*]. Type genus: *Baeocera* Erichson, 1845 [placed on the Official List of Generic Names in Zoology (ICZN 1982)].
Sciatrophitae Achard, 1924: 30 [stem: *Sciatroph-*]. Type genus: *Sciatrophes* Blackburn, 1903 [syn. of *Baeocera* Erichson, 1845].
Baeoceridiitae Achard, 1924: 30 [stem: *Baeoceridi-*]. Type genus: *Baeoceridium* Reitter, 1889.
Scaphicomitae Achard, 1924: 31 [stem: *Scaphicom-*]. Type genus: *Scaphicoma* Motschulsky, 1863.
Toxidiini Achard, 1924: 31 [stem: *Toxidi-*]. Type genus: *Toxidium* J. L. LeConte, 1860.

### Subfamily Piestinae

Erichson, 1839

Piestini Erichson, 1839b: 31 [stem: *Piest-*]. Type genus: *Piestus* Gravenhorst, 1806.
Prognathites Blanchard, 1845a: 290 [stem: *Prognath-*]. Type genus: *Prognathus* Berthold, 1827 [as *Prognata*, incorrect subsequent spelling of type genus name not in prevailing usage; syn. of *Siagonium* Kirby and Spence, 1815]. Comment: original vernacular name available (Art. 11.7.2): first used in latinized form and generally accepted as in Blanchard (1853: 54, as Prognathitae).
*Siagoniini Crowson, 1980: 289 [stem: *Siagoni-*]. Type genus: *Siagonium* Kirby and Spence, 1815. Comment: unavailable family-group name, proposed after 1930 without description or bibliographic reference to such a description (Art. 13.1).

### Subfamily Osoriinae

Erichson, 1839

Osorini Erichson, 1839b: 30 [stem: *Osori-*]. Type genus: *Osorius* Latreille, 1829.

### Tribe Eleusinini

Sharp, 1887

Eleusinina Sharp, 1887: 728 [stem: *Eleusin-*]. Type genus: *Eleusis* Laporte, 1835.

### Tribe Leptochirini

Sharp, 1887

Leptochirina Sharp, 1887: 733 [stem: *Leptochir-*]. Type genus: *Leptochirus* Germar, 1824.

### Tribe Osoriini

Erichson, 1839

Osorini Erichson, 1839b: 30 [stem: *Osori-*]. Type genus: *Osorius* Latreille, 1829.

### Subtribe Osoriina

Erichson, 1839

Osorini Erichson, 1839b: 30 [stem: *Osori-*]. Type genus: *Osorius* Latreille, 1829. Comment: incorrect original stem formation, not in prevailing usage; correction of stem by J. L. LeConte (1861: 68).

### Subtribe Parosoriina

Bernhauer and Schubert, 1911

Parosorii Bernhauer and Schubert, 1911: 146 [stem: *Parosori-*]. Type genus: *Parosorius* Bernhauer, 1904.

### Tribe Thoracophorini

Reitter, 1909

Thoracophorinae Reitter, 1909: 199 [stem: *Thoracophor-*]. Type genus: *Thoracophorus* Motschulsky, 1837 [unjustified emendation of original type genus name *Thoraxophorus* by Erichson (1840a: 908); unjustified emendation in prevailing usage, treated as justified emendation (Art. 33.2.3.1)].

### Subtribe Clavilispinina

Newton and Thayer, 1992

Paralispini Blackwelder, 1942: 79 [stem: *Paralispin-*]. Type genus: *Paralispinus* Bernhauer, 1921 [preoccupied genus name, not *Paralispinus* Eichelbaum, 1913 [Coleoptera: Staphylinidae]; syn. of *Clavilispinus* Bernhauer, 1926]. Comment: permanently invalid (Art. 39): based on preoccupied type genus.
Clavilispinina Newton and Thayer, 1992: 59 [stem: *Clavilispin-*]. Type genus: *Clavilispinus* Bernhauer, 1926. Comment: replacement name for Paralispini Blackwelder, 1942 because of the homonymy of the type genus.

### Subtribe Glyptomina

Newton and Thayer, 1992

Caloceri Blackwelder, 1942: 78 [stem: *Calocer-*]. Type genus: *Calocerus* Fauvel, 1891 [preoccupied genus name, not *Calocerus* J. L. LeConte, 1853 [Coleoptera: Elateridae]; syn. of *Glyptoma* Erichson, 1839]. Comment: permanently invalid (Art. 39): based on preoccupied type genus.
Glyptomina Newton and Thayer, 1992: 59 [stem: *Glyptom-*]. Type genus: *Glyptoma* Erichson, 1839. Comment: replacement name for Caloceri Blackwelder, 1942 because of the homonymy of the type genus; current spelling maintained (Art. 29.5): incorrect stem formation in prevailing usage (should be *Glyptomat*-).

### Subtribe Lispinina

Bernhauer and Schubert, 1910

Lispini Bernhauer and Schubert, 1910: 19 [stem: *Lispin-*]. Type genus: *Lispinus* Erichson, 1839.

### Subtribe Thoracophorina

Reitter, 1909

Thoracophorinae Reitter, 1909: 199 [stem: *Thoracophor-*]. Type genus: *Thoracophorus* Motschulsky, 1837 [unjustified emendation of original type genus name *Thoraxophorus* by Erichson (1840a: 908); unjustified emendation in prevailing usage, treated as justified emendation (Art. 33.2.3.1)]. Comment: based on corrected spelling of type genus.

### Subfamily Oxytelinae

Fleming, 1821

Oxytelidae Fleming, 1821: 49 [stem: *Oxytel-*]. Type genus: *Oxytelus* Gravenhorst, 1802.

### Tribe Blediini

Ádám, 2001

Blediini Ádám, 2001: 216 [stem: *Bledi-*]. Type genus: *Bledius* Leach, 1819.

### Tribe Coprophilini

Heer, 1839

Coprophilina Heer, 1839a: 198 [stem: *Coprophil-*]. Type genus: *Coprophilus* Latreille, 1829 [placed on the Official List of Generic Names in Zoology (ICZN 1993a)]. Comment: this family-group name was also used in the same year by Erichson (1839b: 30, as Coprophilini) and Heer (1839b: 13, as Coprophilida); for comments about the priority of these works see Newton and Thayer (1992: 24).
*Homalotriquitos Solier, 1849: 321 [stem: *Homalotrich-*]. Type genus: *Homalotrichus* Solier, 1849. Comment: original vernacular name unavailable (Art. 11.7.2): not subsequently latinized.
Toxoderi Bernhauer and Schubert, 1911: 91 [stem: *Toxoder-*]. Type genus: *Toxoderus* Fauvel, 1900 [syn. of *Homalotrichus* Solier, 1849]. Comment: junior homonym of Toxoderini Saussure, 1869 (type genus *Toxodera* Audinet-Serville, 1837) in Mantodea: Mantidae; this case is to be referred to the Commission to remove the homonymy (Art. 55.3.1).

### Tribe Euphaniini

Reitter, 1909

*Pholidiens Mulsant and Rey, 1876: 209 [stem: *Pholid-*]. Type genus: *Pholidus* Mulsant and Rey, 1856 [preoccupied genus name, not *Pholidus* Rafinesque, 1815 [Pisces], or *Pholidus* Gray, 1840 [Aves]; syn. of *Euphanias* Fairmaire and Laboulbène, 1856]. Comment: original vernacular name unavailable (Art. 11.7.2): subsequently used in latinized form but not generally attributed to Mulsant and Rey (1876).
Pholidini Acloque, 1896: 145 [stem: *Pholid-*]. Type genus: *Pholidus* Mulsant and Rey, 1856 [preoccupied genus name, not *Pholidus* Rafinesque, 1815 [Pisces], or *Pholidus* Gray, 1840 [Aves]; syn. of *Euphanias* Fairmaire and Laboulbène, 1856]. Comment: permanently invalid (Art. 39): based on preoccupied type genus.
Euphaniae Reitter, 1909: 16 [stem: *Euphani-*]. Type genus: *Euphanias* Fairmaire and Laboulbène, 1856. Comment: precedence (Euphaniini Reitter, 1909 vs Deleasterini Reitter, 1909) given to taxon originally proposed at the higher rank (Art. 24.1).
Deleasterini Reitter, 1909: 164 [stem: *Deleaster-*]. Type genus: *Deleaster* Erichson, 1839.
Syntomiinae Böving and Craighead, 1931: 28 [stem: *Syntomi-*]. Type genus: *Syntomium* Curtis, 1828.

### Tribe Oxytelini

Fleming, 1821

Oxytelidae Fleming, 1821: 49 [stem: *Oxytel-*]. Type genus: *Oxytelus* Gravenhorst, 1802.
Thinobiides J. Sahlberg, 1876: 242 [stem: *Thinobi-*]. Type genus: *Thinobius* Kiesenwetter, 1844.
Trogophléaires Mulsant and Rey, 1878b: 688 [stem: *Trogophloe-*]. Type genus: *Trogophloeus* Mannerheim, 1830 [syn. of *Carpelimus* Leach, 1819]. Comment: original vernacular name available (Art. 11.7.2): first used in latinized form by Seidlitz (1889 [Gatt.]: 90, as Trogophloeina), generally accepted as in Johansen (1914: 533, as Trogophloeina); incorrect original stem formation, not in prevailing usage.
Apocellaria Lynch Arribálzaga, 1884: 344 [stem: *Apocell-*]. Type genus: *Apocellus* Erichson, 1839.
Ecitoclimacini Borgmeier, 1934: 452 [stem: *Ecitoclimac-*]. Type genus: *Ecitoclimax* Borgmeier, 1934. Comment: originally proposed as a tribe of Aleocharinae.
Torrentomi Bierig, 1934: 213 [stem: *Torrentom-*]. Type genus: *Torrentomus* Bierig, 1934 [syn. of *Thinobius* Kiesenwetter, 1844].
Trigonobregmini Scheerpeltz, 1944: 170, in key [stem: *Trigonobregmat-*]. Type genus: *Trigonobregma* Scheerpeltz, 1944. Comment: incorrect original stem formation, not in prevailing usage; placement following Herman (2001).
Carpelimini Hatch, 1957: 85, in key [stem: *Carpelim-*]. Type genus: *Carpelimus* Leach, 1819.
*Thinodromini Gildenkov, 2000: 56 [stem: *Thinodrom-*]. Type genus: *Thinodromus* Kraatz, 1858. Comment: name unavailable (Art. 16.1): name not indicated as intentionally new.
Aploderini Ádám, 2001: 218 [stem: *Aploder-*]. Type genus: *Aploderus* Stephens, 1834.

### Tribe Planeustomini

Jacquelin du Val, 1857

Planeustomites Jacquelin du Val, 1857a: 58 [stem: *Planeustom-*]. Type genus: *Planeustomus* Jacquelin du Val, 1857. Comment: original vernacular name available (Art. 11.7.2): first used in latinized form and generally accepted as in Ádám (2001: 208, as Planeustomini).
Acrognathini Reitter, 1909: 164 [stem: *Acrognath-*]. Type genus: *Acrognathus* Erichson, 1839 [preoccupied genus name, not *Acrognathus* Agassiz, 1836 [Pisces]; syn. of *Manda* Blackwelder, 1952]. Comment: permanently invalid (Art. 39): based on preoccupied type genus.
Mandini Gildenkov, 2003: 35 [stem: *Mand-*]. Type genus: *Manda* Blackwelder, 1952. Comment: replacement name for Acrognathini Reitter, 1909 because of the homonymy of the type genus.

### Subfamily Oxyporinae

Fleming, 1821

Oxyporidae Fleming, 1821: 49 [stem: *Oxypor-*]. Type genus: *Oxyporus* Fabricius, 1775.

### Subfamily Megalopsidiinae

Leng, 1920

Megalopini Erichson, 1839b: 30 [stem: *Megalop-*]. Type genus: *Megalops* Erichson, 1839 [preoccupied genus name, not *Megalops* Lacepède, 1803 [Pisces], not *Megalops* Rafinesque, 1815 [Pisces]; syn. of *Megalopinus* Eichelbaum, 1915]. Comment: permanently invalid (Art. 39): based on preoccupied type genus.
Megalopsidiini Leng, 1920: 98 [stem: *Megalopsidi-*]. Type genus: *Megalopsidia* Leng, 1918 [syn. of *Megalopinus* Eichelbaum, 1915].
Aulacotrachelinae L. Benick, 1920: 1 [stem: *Aulacotrachel-*]. Type genus: *Aulacotrachelus* Benick, 1920 [syn. of *Megalopinus* Eichelbaum, 1915]. Comment: replacement name for Megalopinae Erichson, 1839 because of the homonymy of the type genus.
Stylopodinae Blackwelder, 1943: 202 [stem: *Stylopod-*]. Type genus: *Stylopodus* Benick, 1917 [syn. of *Megalopinus* Eichelbaum, 1915]. Comment: name proposed to replace Megalopinae Erichson, 1839 and Megalopsidiinae Leng, 1920 because of the synonymy of the type genus.
*Megalopininae Puthz, 1967: 192 [stem: *Megalopin-*]. Type genus: *Megalopinus* Eichelbaum, 1915. Comment: unavailable family-group name, proposed after 1930 without description or bibliographic reference to such a description (Art. 13.1).
Megalopininae Naomi, 1986: 344 [stem: *Megalopin-*]. Type genus: *Megalopinus* Eichelbaum, 1915. Comment: name proposed to replace Megalopinae Erichson, 1839 and Megalopsidiinae Leng, 1920 because of the synonymy of the type genus.

### Subfamily Scydmaeninae

Leach, 1815

Scydmaenides Leach, 1815: 92 [stem: *Scydmaen-*]. Type genus: *Scydmaenus* Latreille, 1802. Comment: placement follows Grebennikov and Newton (2009).

### †Supertribe Hapsomelitae

Poinar and Brown, 2004

Hapsomelinae Poinar and Brown, 2004: 790 [stem: *Hapsomel-*]. Type genus: *Hapsomela* Poinar and Brown, 2004.

### Supertribe Mastigitae

Fleming, 1821

Mastigoidae Fleming, 1821: 49 [stem: *Mastig-*]. Type genus: *Mastigus* Latreille, 1802. Comment: incorrect original stem formation, not in prevailing usage.

### Tribe Clidicini

Casey, 1897

Clidicini Casey, 1897: 541 [stem: *Clidic-*]. Type genus: *Clidicus* Laporte, 1833.

### Tribe Leptomastacini

Casey, 1897

Leptomastacini Casey, 1897: 541 [stem: *Leptomastac-*]. Type genus: *Leptomastax* Pirazzoli, 1855.

### Tribe Mastigini

Fleming, 1821

Mastigoidae Fleming, 1821: 49 [stem: *Mastig-*]. Type genus: *Mastigus* Latreille, 1802. Comment: incorrect original stem formation, not in prevailing usage.

### Supertribe Scydmaenitae

Leach, 1815

Scydmaenides Leach, 1815: 92 [stem: *Scydmaen-*]. Type genus: *Scydmaenus* Latreille, 1802.

### Tribe Cephenniini

Reitter, 1882

Cephenniini Reitter, 1882c: 142 [stem: *Cephenni-*]. Type genus: *Cephennium* Müller and Kunze, 1822.
Anisosphaeridae Tömösváry, 1883: 128 [stem: *Anisosphaer-*]. Type genus: *Anisosphaera* Tömösváry, 1883 [syn. of *Cephennium* Müller and Kunze, 1822]. Comment: family-group name originally based on larva only.

### Tribe Chevrolatiini

Reitter, 1882

Chevrolatini Reitter, 1882c: 142 [stem: *Chevrolati-*]. Type genus: *Chevrolatia* Jacquelin du Val, 1859. Comment: incorrect original stem formation, not in prevailing usage.

### Tribe Cyrtoscydmini

Schaufuss, 1889

Cyrtoscydmini L. W. Schaufuss, 1889: 2 [stem: *Cyrtoscydm-*]. Type genus: *Cyrtoscydmus* Motschulsky, 1869 [syn. of *Stenichnus* C. G. Thomson, 1859].
Glandulariidae L. W. Schaufuss, 1889: 3 [stem: *Glandulari-*]. Type genus: *Glandularia* L. W. Schaufuss, 1889 [syn. of *Euconnus* (*Napochus*) C. G. Thomson, 1859].
Euconnini Casey, 1897: 354 [stem: *Euconn-*]. Type genus: *Euconnus* C. G. Thomson, 1859.
Opresini Casey, 1897: 354 [stem: *Opres-*]. Type genus: *Opresus* Casey, 1897 [syn. of *Microscydmus* Saulcy and Croissandeau, 1893].
Lophioderini Casey, 1897: 356 [stem: *Lophioder-*]. Type genus: *Lophioderus* Casey, 1897.
Stenichnini Ganglbauer, 1898: 25 [stem: *Stenichn-*]. Type genus: *Stenichnus* C. G. Thomson, 1859.
Neuraphini Csiki, 1909b: 18 [stem: *Neuraph-*]. Type genus: *Neuraphes* C. G. Thomson, 1859 [this name is an incorrect subsequent spelling of *Nevraphes*, in prevailing usage and so deemed to be the correct original spelling (Art. 33.3.1)].
Syndicini Csiki, 1919: 17 [stem: *Syndic-*]. Type genus: *Syndicus* Motschulsky, 1851.
Sciacharini Csiki, 1919: 69 [stem: *Sciacharit-*]. Type genus: *Sciacharis* Broun, 1893. Comment: incorrect original stem formation, not in prevailing usage.
Siamitini H. Franz, 1989: 44 [stem: *Siamit-*]. Type genus: *Siamites* Franz, 1989.

### Tribe Eutheiini

Casey, 1897

Eutheiini Casey, 1897: 507 [stem: *Euthei-*]. Type genus: *Eutheia* Stephens, 1830.
Ascydmini Casey, 1897: 355 [stem: *Ascydm-*]. Type genus: *Ascydmus* Casey, 1897 [syn. of *Euthiconus* Reitter, 1882].

### Tribe Leptoscydmini

Casey, 1897

Leptoscydmini Casey, 1897: 355 [stem: *Leptoscydm-*]. Type genus: *Leptoscydmus* Casey, 1897.

### Tribe Plaumanniolini

Costa Lima, 1962

Plaumanniolinae Costa Lima, 1962: 415 [stem: *Plaumanniol-*]. Type genus: *Plaumanniola* Costa Lima, 1962. Comment: originally proposed as a subfamily of Ptinidae.

### Tribe Scydmaenini

Leach, 1815

Scydmaenides Leach, 1815: 92 [stem: *Scydmaen-*]. Type genus: *Scydmaenus* Latreille, 1802.
Eumicrini Reitter, 1882c: 192 [stem: *Eumicr-*]. Type genus: *Eumicrus* Laporte, 1833 [syn. of *Scydmaenus* Latreille, 1802].

### Subfamily Steninae

MacLeay, 1825

Stenidae W. S. MacLeay, 1825: 49 [stem: *Sten-*]. Type genus: *Stenus* Latreille, 1797. Comment: the younger name Steninae Fraser and Purves, 1960 (type genus *Steno* Gray, 1846) in Mammalia: Delphinidae is unavailable according to Newton and Thayer (1992: 66).

### Subfamily Euaesthetinae

Thomson, 1859

Euaesthetina C. G. Thomson, 1859: 42 [stem: *Euaesthet-*]. Type genus: *Euaesthetus* Gravenhorst, 1806.

### Tribe Alzadaesthetini

Scheerpeltz, 1974

Alzadaesthetini Scheerpeltz, 1974: 102, in key [stem: *Alzadaesthet-*]. Type genus: *Alzadaesthetus* Kistner, 1961.

### Tribe Austroesthetini

Cameron, 1944

Austroaesthetini Cameron, 1944: 69 [stem: *Austroesthet-*]. Type genus: *Austroesthetus* Oke, 1933 [as *Austroaesthetus*, unjustified emendation of genus name not in prevailing usage]. Comment: incorrect original stem formation, not in prevailing usage.

### Tribe Euaesthetini

Thomson, 1859

Euaesthetina C. G. Thomson, 1859: 42 [stem: *Euaesthet-*]. Type genus: *Euaesthetus* Gravenhorst, 1806.
Tamotini Coiffait, 1984: 353 [stem: *Tamot-*]. Type genus: *Tamotus* L. W. Schaufuss, 1872.

### Tribe Fenderiini

Scheerpeltz, 1974

Fenderiini Scheerpeltz, 1974: 103, in key [stem: *Fenderi-*]. Type genus: *Fenderia* Hatch, 1957.

### Tribe Nordenskioldiini

Bernhauer and Schubert, 1911

Nordenskioeldiini Bernhauer and Schubert, 1911: 186 [stem: *Nordenskioldi-*]. Type genus: *Nordenskioldia* Sahlberg, 1880 [as *Nordenskioeldia*, incorrect subsequent spelling of genus name not in prevailing usage]. Comment: incorrect original stem formation, not in prevailing usage (see Newton and Thayer 1992: 56).

### Tribe Stenaesthetini

Bernhauer and Schubert, 1911

Stenaesthetini Bernhauer and Schubert, 1911: 186 [stem: *Stenaesthet-*]. Type genus: *Stenaesthetus* Sharp, 1874.

### Subfamily Solieriinae

Newton and Thayer, 1992

Fisognatitos Solier, 1849: 303 [stem: *Physognath-*]. Type genus: *Physognathus* Solier, 1849 [preoccupied genus name, not *Physognathus* Agassiz, 1846 [Reptilia]; syn. of *Solierius* Bernhauer, 1921]. Comment: original vernacular name available (Art. 11.7.2): first used in latinized form and generally accepted as in Kraatz (1859b: 3, as Physognathites [treated as Latin]); permanently invalid (Art. 39): based on preoccupied type genus.
Solieriinae Newton and Thayer, 1992: 27 [stem: *Solieri-*]. Type genus: *Solierius* Bernhauer, 1921. Comment: replacement name for Physognathinae Solier, 1849 because of the homonymy of the type genus.

### Subfamily Leptotyphlinae

Fauvel, 1874

Leptotyphli Fauvel, 1874: 35 [stem: *Leptotyphl-*]. Type genus: *Leptotyphlus* Fauvel, 1874.

### Tribe Cephalotyphlini

Coiffait, 1963

Cephalotyphlini Coiffait, 1963: 380, in key [stem: *Cephalotyphl-*]. Type genus: *Cephalotyphlus* Coiffait, 1955.

### Tribe Entomoculiini

Coiffait, 1957

Entomoculini Coiffait, 1957: 61 [stem: *Entomoculi-*]. Type genus: *Entomoculia* Croissandeau, 1891. Comment: incorrect original stem formation, not in prevailing usage; correction of stem by Newton and Thayer (1992: 56).

### Tribe Leptotyphlini

Fauvel, 1874

Leptotyphli Fauvel, 1874: 35 [stem: *Leptotyphl-*]. Type genus: *Leptotyphlus* Fauvel, 1874.
Leptotyphlini Coiffait, 1957: 61 [stem: *Leptotyphl-*]. Type genus: *Leptotyphlus* Fauvel, 1874. Comment: family-group name proposed as new without reference to Leptotyphli Fauvel, 1874.

### Tribe Metrotyphlini

Coiffait, 1963

Metrotyphlini Coiffait, 1963: 381 [stem: *Metrotyphl-*]. Type genus: *Metrotyphlus* Coiffait, 1959.

### Tribe Neotyphlini

Coiffait, 1963

Neotyphlini Coiffait, 1963: 381, in key [stem: *Neotyphl-*]. Type genus: *Neotyphlus* Coiffait, 1959.

### Subfamily Pseudopsinae

Ganglbauer, 1895

Pseudopsini Ganglbauer, 1895: 690 [stem: *Pseudops-*]. Type genus: *Pseudopsis* Newman, 1834. Comment: current spelling maintained (Art. 29.5): incorrect stem formation in prevailing usage (should be *Pseudopse*-).

### Subfamily Paederinae

Fleming, 1821

Poederidae Fleming, 1821: 49 [stem: *Paeder-*]. Type genus: *Paederus* Fabricius, 1775.

### Tribe Paederini

Fleming, 1821

Poederidae Fleming, 1821: 49 [stem: *Paeder-*]. Type genus: *Paederus* Fabricius, 1775.

### Subtribe Astenina

Hatch, 1957

Suniina Sharp, 1886b: 591 [stem: *Suni-*]. Type genus: *Sunius* sensu Erichson, 1839 [not *Sunius* Stephens, 1829; syn. of *Astenus* Dejean, 1833]. Comment: based on a misidentified type genus, name treated here as invalid until an application is submitted to the Commission to suppress it for the Principle of Priority (Art. 65.2.1).
Astenina Hatch, 1957: 151, in key [stem: *Asten-*]. Type genus: *Astenus* Dejean, 1833. Comment: although this is not the oldest name for the subtribe, we recommend that an application be submitted to the Commission to suppress Suniina Sharp, 1886 because it is based on a misidentified type genus (Art. 65.2.1).

### Subtribe Cryptobiina

Casey, 1905

Cryptobia Casey, 1905: 21 [stem: *Cryptobi-*]. Type genus: *Cryptobium* Mannerheim, 1830 [syn. of *Ochthephilum* Stephens, 1829]. Comment: the younger name Cryptobiinae Hollande, 1952 (type genus *Cryptobia* Leidy, 1846) in Protozoa: Bodonidae is unavailable (see Newton and Thayer 1992: 61).
Cryptobiina Bordoni, 1975: 420 [stem: *Cryptobi-*]. Type genus: *Cryptobium* Mannerheim, 1830 [syn. of *Ochthephilum* Stephens, 1829]. Comment: family-group name proposed as new without reference to Cryptobia Casey, 1905.

### Subtribe Cylindroxystina

Bierig, 1943

Cylindroxystini Bierig, 1943: 158 [stem: *Cylindroxyst-*]. Type genus: *Cylindroxystus* Bierig, 1943.

### Subtribe Dolicaonina

Casey, 1905

Gnatimenitos Solier, 1849: 326 [stem: *Gnathymen-*]. Type genus: *Gnathymenus* Solier, 1849. Comment: original vernacular name available (Art. 11.7.2): first used in latinized form and generally accepted as in Ádám (2001: 115, as Gnathymenini); incorrect original stem formation, not in prevailing usage; this name was treated as unavailable by Newton and Thayer (1992: 61) which led to their use of Dolicaonina Casey, 1905 as the valid name for this subtribe; all subsequent authors have followed Newton and Thayer (1992: 61) except for the subsequent use of “Gnathymenini (Solier, 1849)” as valid by Ádám (2001: 115) which made Solier’s name available according to our criteria of availability for names originally proposed in vernacular form; here we continue to use Dolicaonina Casey, 1905 as the valid name for this subtribe and recommend that an application be submitted to the Commission to suppress the name Gnathymenina Solier, 1849.
Dolicaones Casey, 1905: 56 [stem: *Dolicaon-*]. Type genus: *Dolicaon* Laporte, 1835. Comment: see comments under the name Gnatimenitos Solier, 1849 above.
Leptobii Bordoni, 1980: 170 [stem: *Leptobi-*]. Type genus: *Leptobium* Casey, 1905. Comment: unnecessary replacement name for “Dolicaina Bordoni, 1975”.

### Subtribe Echiasterina

Casey, 1905

Echiasteres Casey, 1905: 245 [stem: *Echiaster-*]. Type genus: *Echiaster* Erichson, 1839.

### Subtribe Lathrobiina

Laporte, 1835

Lathrobidae Laporte, 1835a: 117 [stem: *Lathrobi-*]. Type genus: *Lathrobium* Gravenhorst, 1802. Comment: incorrect original stem formation, not in prevailing usage.
Sphaeronia Casey, 1905: 54 [stem: *Sphaeron-*]. Type genus: *Sphaeronum* Sharp, 1876 [as *Sphaeronium*, incorrect subsequent spelling of type genus name, not in prevailing usage]. Comment: incorrect original stem formation, not in prevailing usage.

### Subtribe Lithocharina

Casey, 1905

Lithochares Casey, 1905: 146 [stem: *Lithochar-*]. Type genus: *Lithocharis* Dejean, 1833. Comment: current spelling maintained (Art. 29.5): incorrect stem formation in prevailing usage (should be *Lithocharit*-).
Lithocharina Bordoni, 1974: 324 [stem: *Lithochar-*]. Type genus: *Lithocharis* Dejean, 1833. Comment: family-group name proposed as new without reference to Lithochares Casey, 1905.

### Subtribe Medonina

Casey, 1905

Medones Casey, 1905: 20 [stem: *Medon-*]. Type genus: *Medon* Stephens, 1833. Comment: current spelling maintained (Art. 29.5): incorrect stem formation in prevailing usage (should be *Medont*-).
Medina Bordoni, 1975: 420 [stem: *Medon-*]. Type genus: *Medon* Stephens, 1833. Comment: family-group name proposed as new without reference to Medones Casey, 1905; incorrect original stem formation, not in prevailing usage (should be *Medont*-).
Acanthoglossi Coiffait, 1982: 10 [stem: *Acanthogloss-*]. Type genus: *Acanthoglossa* Kraatz, 1859.

### Subtribe Paederina

Fleming, 1821

Poederidae Fleming, 1821: 49 [stem: *Paeder-*]. Type genus: *Paederus* Fabricius, 1775 [as *Poederus*, incorrect subsequent spelling of type genus name, not in prevailing usage]. Comment: incorrect original stem formation, not in prevailing usage, see comments in Newton and Thayer (1992: 60).
Geopaederidae Gistel, 1848: [13] [stem: *Geopaeder-*]. Type genus: *Geopaederus* Gistel, 1848 [syn. of *Paederus* Fabricius, 1775].

### Subtribe Scopaeina

Mulsant and Rey, 1878

*Poliodontidos Solier, 1849: 303 [stem: *Polyodont-*]. Type genus: *Polyodontus* Solier, 1849 [preoccupied genus name, not *Polyodontus* Eysenhardt, 1818 [Vermes]; syn. of *Scopaeus* Erichson, 1839]. Comment: original vernacular name unavailable (Art. 11.7.2): not subsequently latinized; if found to be available then permanently invalid (Art. 39): based on preoccupied type genus; Polyodontidae Bonaparte, 1838 (type genus *Polyodon* Lacepède, 1797) is available in Pisces; incorrect original stem formation, not in prevailing usage.
Scopéates Mulsant and Rey, 1878a: 178 [stem: *Scopae-*]. Type genus: *Scopaeus* Erichson, 1839. Comment: original vernacular name available (Art. 11.7.2): first used in latinized form by Seidlitz (1889 [Gatt.]: 92, as Scopaeina), generally accepted as in Newton and Thayer (1992: 62, as Scopaeina); incorrect original stem formation, not in prevailing usage.

### Subtribe Stilicina

Casey, 1905

Stilici Casey, 1905: 218 [stem: *Stilic-*]. Type genus: *Stilicus* Berthold, 1827 [syn. of *Rugilus* Leach, 1819].
Rugilina Hatch, 1957: 151, in key [stem: *Rugil-*]. Type genus: *Rugilus* Leach, 1819.

### Subtribe Stilicopsina

Casey, 1905

Stilicopses Casey, 1905: 230 [stem: *Stilicops-*]. Type genus: *Stilicopsis* Sachse, 1852. Comment: current spelling maintained (Art. 29.5): incorrect stem formation in prevailing usage (should be *Stilicopse*-).
Xenasteres Bierig, 1939: 179 [stem: *Xenaster-*]. Type genus: *Xenaster* Bierig, 1939 [preoccupied genus name, not *Xenaster* Simonwitsch, 1871 [Echinodermata]; the nomenclatural status of *Xenaster* will be addressed in the near future by L. Herman (pers. comm. 2010) therefore we refrain from proposing a new replacement name here]. Comment: permanently invalid (Art. 39): based on preoccupied type genus.
*Stamnoderes Blackwelder, 1944: 126 [stem: *Stamnoder-*]. Type genus: *Stamnoderus* Sharp, 1886. Comment: unavailable family-group name, proposed after 1930 without description or bibliographic reference to such a description (Art. 13.1).

### Tribe Pinophilini

Nordmann, 1837

Pinophiliniformes Nordmann, 1837: 6 [stem: *Pinophil-*]. Type genus: *Pinophilus* Gravenhorst, 1802.

### Subtribe Pinophilina

Nordmann, 1837

Pinophiliniformes Nordmann, 1837: 6 [stem: *Pinophil-*]. Type genus: *Pinophilus* Gravenhorst, 1802.

### Subtribe Procirrina

Bernhauer and Schubert, 1912

Procirri Bernhauer and Schubert, 1912: 197 [stem: *Procirr-*]. Type genus: *Procirrus* Latreille, 1829.

### Subfamily Staphylininae

Latreille, 1802

Staphyliniae Latreille, 1802: 124 [stem: *Staphylin-*]. Type genus: *Staphylinus* Linnaeus, 1758 [placed on the Official List of Generic Names in Zoology (ICZN 1959a)].

### Tribe Arrowinini

Solodovnikov and Newton, 2005

Arrowinini Solodovnikov and Newton, 2005: 420 [stem: *Arrowin-*]. Type genus: *Arrowinus* Bernhauer, 1935.

### Tribe Diochini

Casey, 1906

Diochi Casey, 1906: 429 [stem: *Dioch-*]. Type genus: *Diochus* Erichson, 1839.
Diochinae I. Moore, 1964: 86, in key [stem: *Dioch-*]. Type genus: *Diochus* Erichson, 1839. Comment: family-group name proposed as new without reference to Diochi Casey, 1906.

### Tribe Maorothiini

Assing, 2000

Maorothiini Assing, 2000: 16 [stem: *Maorothi-*]. Type genus: *Maorothius* Assing, 2000.

### Tribe Othiini

Thomson, 1859

Othiides C. G. Thomson, 1859: 26 [stem: *Othi-*]. Type genus: *Othius* Stephens, 1829 [placed on the Official List of Generic Names in Zoology (ICZN 1983c)].
Atrecini Hatch, 1957: 172, in key [stem: *Atrec-*]. Type genus: *Atrecus* Jacquelin du Val, 1856.

### Tribe Platyprosopini

Lynch Arribálzaga, 1884

Platyprosoparia Lynch Arribálzaga, 1884: 165 [stem: *Platyprosop-*]. Type genus: *Platyprosopus* Mannerheim, 1830.
Platyprosopinae I. Moore, 1964: 86, in key [stem: *Platyprosop-*]. Type genus: *Platyprosopus* Mannerheim, 1830. Comment: family-group name proposed as new without reference to Platyprosopinae Lynch Arribálzaga, 1884.

### Tribe Staphylinini

Latreille, 1802

Staphyliniae Latreille, 1802: 124 [stem: *Staphylin-*]. Type genus: *Staphylinus* Linnaeus, 1758 [placed on the Official List of Generic Names in Zoology (ICZN 1959a)]. Comment: placed on the Official List of Family-Group Names in Zoology (as Staphylinidae Latreille, [1803-1804]) (ICZN 1959a).

### Subtribe Amblyopinina

Seevers, 1944

Amblyopininae Seevers, 1944: 157 [stem: *Amblyopin-*]. Type genus: *Amblyopinus* Solsky, 1875.
Heterothopsi Coiffait, 1978: 300 [stem: *Heterothop-*]. Type genus: *Heterothops* Stephens, 1829. Comment: incorrect original stem formation, not in prevailing usage; correction of stem by Newton and Thayer (1992: 65); placement based on Chatzimanolis et al. (2010).

### Subtribe Anisolinina

Hayashi, 1993

Anisolinina Hayashi, 1993: 288 [stem: *Anisolin-*]. Type genus: *Anisolinus* Sharp, 1889. Comment: originally proposed as a subtribe of Philonthini Kirby, 1837.

### Subtribe Eucibdelina

Sharp, 1889

Eucibdelini Sharp, 1889: 112 [stem: *Eucibdel-*]. Type genus: *Eucibdelus* Kraatz, 1859.

### Subtribe Hyptiomina

Casey, 1906

Hyptiomae Casey, 1906: 361 [stem: *Hyptiom-*]. Type genus: *Hyptioma* Casey, 1906 [syn. of *Holisus* Erichson, 1839]. Comment: current spelling maintained (Art. 29.5): incorrect stem formation in prevailing usage (should be *Hyptiomat*-).
*Holisi Blackwelder, 1944: 143 [stem: *Holis-*]. Type genus: *Holisus* Erichson, 1839. Comment: unavailable family-group name, proposed after 1930 without description or bibliographic reference to such a description (Art. 13.1).
Holisina Newton, 1988: 259 [stem: *Holis-*]. Type genus: *Holisus* Erichson, 1839.

### Subtribe Philonthina

Kirby, 1837

Philonthidae Kirby, 1837: 91 [stem: *Philonth-*]. Type genus: *Philonthus* Curtis, 1829.
*Rémates Mulsant and Rey, 1876: 596 [stem: *Rem-*]. Type genus: *Remus* sensu C. G. Thomson, 1859 [not *Remus* Holme, 1837; syn. of *Erichsonius* Fauvel, 1874]. Comment: original vernacular name unavailable (Art. 11.7.2): not subsequently latinized; also based on a misidentified type genus.
Craspedomeri Bernhauer, 1911: 88 [stem: *Craspedomer-*]. Type genus: *Craspedomerus* Bernhauer, 1911.

### Subtribe Quediina

Kraatz, 1857

Quediiformes Kraatz, 1857: 473 [stem: *Quedi-*]. Type genus: *Quedius* Stephens, 1829 [placed on the Official List of Generic Names in Zoology (ICZN 1996c)]. Comment: use of family-group name given precedence over Platycnemini Nordmann 1837 and "Quediini Kraatz, [1857]" placed on the Official List of Family-Group Names in Zoology (ICZN 1996c).
*Acylophorini Scheerpeltz, 1968: 97 [stem: *Acylophor-*]. Type genus: *Acylophorus* Nordmann, 1837. Comment: unavailable family-group name, proposed after 1930 without description or bibliographic reference to such a description (Art. 13.1).
Acylophorini Outerelo and Gamarra, 1985: 48, in key [stem: *Acylophor-*]. Type genus: *Acylophorus* Nordmann, 1837.

### Subtribe Staphylinina

Latreille, 1802

Staphyliniae Latreille, 1802: 124 [stem: *Staphylin-*]. Type genus: *Staphylinus* Linnaeus, 1758 [placed on the Official List of Generic Names in Zoology (ICZN 1959a)]. Comment: placed on the Official List of Family-Group Names in Zoology (as Staphylinidae Latreille, [1803-1804]) and “Staphylinii Latreille, [1803-1804]” placed on the Official Index of Rejected and Invalid Family-Group Names in Zoology (ICZN 1959a).
Creophilidae Kirby, 1837: 95 [stem: *Creophil-*]. Type genus: *Creophilus* Leach, 1819 [*Creophilus* (attributed to Samouelle, 1819) placed on the Official List of Generic Names in Zoology (ICZN 1959a)].
Thinopininae Böving and Craighead, 1931: 30 [stem: *Thinopin-*]. Type genus: *Thinopinus* J. L. LeConte, 1852.
Ocypina Hatch, 1957: 173, in key [stem: *Ocypod-*]. Type genus: *Ocypus* Leach, 1819. Comment: incorrect original stem formation, not in prevailing usage; correction of stem by Newton and Thayer (1992: 65).

### Subtribe Tanygnathinina

Reitter, 1909

Tanygnathinini Reitter, 1909: 105 [stem: *Tanygnathin-*]. Type genus: *Tanygnathinus* Reitter, 1909 [syn. of *Atanygnathus* Jakobson, 1909].
Tanygnathini Casey, 1915b: 424 [stem: *Tanygnath-*]. Type genus: *Tanygnathus* Erichson, 1839 [preoccupied genus name, not *Tanygnathus* Wagler, 1832 [Aves]; syn. of *Atanygnathus* Jakobson, 1909]. Comment: permanently invalid (Art. 39): based on preoccupied type genus.
Atanygnathini Lohse, 1964: 220 [stem: *Atanygnath-*]. Type genus: *Atanygnathus* Jakobson, 1909.

### Subtribe Xanthopygina

Sharp, 1884

Platycnemidiformes Nordmann, 1837: 6 [stem: *Platycnem-*]. Type genus: *Platycnemus* Nordmann, 1837 [placed on the Official List of Generic Names in Zoology (ICZN 1996c); syn. of *Haematodes* Laporte, 1835]. Comment: younger name Xanthopygina Sharp, 1884 given precedence over this name (ICZN 1996c); placed on the Official List of Family-Group Names in Zoology (ICZN 1996c, as Platycnemini Nordmann, 1837).
Xanthopygina Sharp, 1884: 342 [stem: *Xanthopyg-*]. Type genus: *Xanthopygus* Kraatz, 1857. Comment: family-group name given precedence over Platycnemini Nordmann, 1837 and placed on the Official List of Family-Group Names in Zoology (ICZN 1996c).
Triacri Bernhauer, 1931: 84 [stem: *Triacr-*]. Type genus: *Triacrus* Nordmann, 1837.

### Tribe Xantholinini

Erichson, 1839

Agraeformes Nordmann, 1837: 7 [stem: *Agrod-*]. Type genus: *Agrodes* Nordmann, 1837 [placed on the Official List of Generic Names in Zoology (ICZN 1996c); syn. of *Plochionocerus* Dejean, 1833]. Comment: name placed on the Official List of Family-Group Names in Zoology (ICZN 1996c, as Agrodini Nordmann, 1837); younger name Xantholinini Erichson, 1839 given precedence over this name (ICZN 1996c).
Gyrohypnidae Kirby, 1837: 88 [stem: *Gyrohypn-*]. Type genus: *Gyrohypnus* Leach, 1819 [placed on the Official List of Generic Names in Zoology (ICZN 1983c, as *Gyrohypnus* Samouelle, 1819)]. Comment: placed on the Official List of Family-Group Names in Zoology (ICZN 1996c, as Gyrohypnini Kirby, 1837); younger name Xantholinini Erichson, 1839 given precedence over this name (ICZN 1996c).
Xantholinini Erichson, 1839b: 28 [stem: *Xantholin-*]. Type genus: *Xantholinus* Dejean, 1821 [placed on the Official List of Generic Names in Zoology (ICZN 1983c)]. Comment: family-group name given precedence over Agrodini Nordmann, 1837 and Gyrohypnini Kirby, 1837 and placed on the Official List of Family-Group Names in Zoology (ICZN 1996c).
Araeocnemes Casey, 1906: 359 [stem: *Araeocnem-*]. Type genus: *Araeocnemus* Nordmann, 1837 [as *Araeocnemis*, incorrect subsequent spelling of type genus name, not in prevailing usage; syn. of *Plochionocerus* Dejean, 1833].
Metoponci Casey, 1906: 360 [stem: *Metoponc-*]. Type genus: *Metoponcus* Kraatz, 1857 [syn. of *Zeteotomus* Jacquelin du Val, 1856].

### †Subfamily Protactinae

Heer, 1847

Protactiden Heer, 1847: 28 [stem: *Protact-*]. Type genus: *Protactus* Heer, 1847. Comment: original vernacular name available (Art. 11.7.2): first used in latinized form by Bronn (1848: 1045, as Protactidae), generally accepted as in Herman (2001: 3839, as Protactinae).

### Staphylinoidea incertae sedis

Hameedini M. Abdullah and Quadri, 1968: 310 [stem: *Hameedi-*]. Type genus: *Hameedia* M. Abdullah and Quadri, 1968. Comment: incorrect original stem formation, not in prevailing usage; originally proposed as a tribe of Staphylinidae: Oxyporinae; correct placement uncertain (see Newton and Thayer 1992: 67).

### Series SCARABAEIFORMIA

### Superfamily Scarabaeoidea

Latreille, 1802

Scarabaeïdes Latreille, 1802: 144 [stem: *Scarabae-*]. Type genus: *Scarabaeus* Linnaeus, 1758. Comment: First Reviser (Scarabaeoidea Latreille, 1802 vs Geotrupoidea Latreille, 1802) not determined, current usage maintained.

### Family Pleocomidae

LeConte, 1861

Pleocomini J. L. LeConte, 1861: 128 [stem: *Pleocom-*]. Type genus: *Pleocoma* J. L. LeConte, 1856.

### Subfamily Pleocominae

LeConte, 1861

Pleocomini J. L. LeConte, 1861: 128 [stem: *Pleocom-*]. Type genus: *Pleocoma* J. L. LeConte, 1856.

### †Subfamily Cretocominae

Nikolajev, 2002

Cretocomini Nikolajev, 2002: 53 [stem: *Cretocom-*]. Type genus: *Cretocoma* Nikolajev, 2002.

### †Subfamily Archescarabaeinae

Nikolajev, 2010

Archescarabaeinae Nikolajev, 2010: 69 [stem: *Archescarabae*-]. Type genus: *Archescarabaeus* Nikolajev, 2010.

### Family Geotrupidae

Latreille, 1802

Geotrupini Latreille, 1802: 142 [stem: *Geotrup-*]. Type genus: *Geotrupes* Latreille, 1797 [placed on the Official List of Generic Names in Zoology (ICZN 1955b)]. Comment: name placed on the Official List of Family-Group Names in Zoology (ICZN 1955c, as Geotrupini Latreille, 1806).

### Subfamily Taurocerastinae

Germain, 1897

Taurocerastidae Germain, 1897: 288 [stem: *Taurocerast-*]. Type genus: *Taurocerastes* Philippi, 1866.

### Subfamily Bolboceratinae

Mulsant, 1842

Bolbocéraires Mulsant, 1842: 347 [stem: *Bolbocerat-*]. Type genus: *Bolboceras* Kirby, 1819 [placed on the Official List of Generic Names in Zoology (ICZN 2006a)].

### Tribe Athyreini

Lynch Arribálzaga, 1878

*Athyréites Blanchard, 1845a: 221 [stem: *Athyre-*]. Type genus: *Athyreus* W. S. MacLeay, 1819 [placed on the Official List of Generic Names in Zoology (ICZN 1985a)]. Comment: original vernacular name unavailable (Art. 11.7.2): subsequently used in latinized form but not generally attributed to Blanchard (1845a) (see A. B. T. Smith 2006: 148).
Athyreitae Lynch Arribálzaga, 1878: 145 [stem: *Athyre-*]. Type genus: *Athyreus* W. S. MacLeay, 1819 [placed on the Official List of Generic Names in Zoology (ICZN 1985a)]. Comment: name previously attributed to Howden and Martínez (1963).
Athyreini Howden and Martínez, 1963: 346 [stem: *Athyre-*]. Type genus: *Athyreus* W. S. MacLeay, 1819 [placed on the Official List of Generic Names in Zoology (ICZN 1985a)]. Comment: family-group name proposed as new without reference to Athyréides Blanchard, 1845 or Athyreitae Lynch Arribálzaga, 1878.

### Tribe Bolbelasmini

Nikolajev, 1996

Bolbelasmini Nikolajev, 1996: 96 [stem: *Bolbelasm-*]. Type genus: *Bolbelasmus* Boucomont, 1911.

### Tribe Bolboceratini

Mulsant, 1842

Bolbocéraires Mulsant, 1842: 347 [stem: *Bolbocerat-*]. Type genus: *Bolboceras* Kirby, 1819 [placed on the Official List of Generic Names in Zoology (ICZN 2006a)]. Comment: original vernacular name available (Art. 11.7.2): first used in latinized form by Boucomont (1911: 333, as Bolbocerinae [incorrect stem formation]), generally accepted as in A. B. T. Smith (2006: 148, as Bolboceratini); concept of *Bolboceras* Kirby, 1819 fixed by the Commission (ICZN 2006a); incorrect original stem formation, not in prevailing usage.
Australobolbini Nikolajev, 1996: 96 [stem: *Australobolb-*]. Type genus: *Australobolbus* Howden and Cooper, 1977.

### Tribe Bolbochromini

Nikolajev, 1970

Bolbochromini Nikolajev, 1970: 34 [stem: *Bolbochrom-*]. Type genus: *Bolbochromus* Boucomont, 1909.

### Tribe Eubolbitini

Nikolajev, 1970

Eubolbitini Nikolajev, 1970: 33 [stem: *Eubolbit-*]. Type genus: *Eubolbitus* Reitter, 1892.

### Tribe Eucanthini

Nikolajev, 2003

Eucanthini Nikolajev, 2003b: 209 [stem: *Eucanth-*]. Type genus: *Eucanthus* Westwood, 1848.

### Tribe Gilletinini

Nikolajev, 1990

Gilletinini Nikolajev, 1990: 99 [stem: *Gilletin-*]. Type genus: *Gilletinus* Boucomont, 1932.

### Tribe Odonteini

Shokhin, 2007

Odonteini Shokhin, 2007: 111 [stem: *Odonte-*]. Type genus: *Odonteus* Samouelle, 1819 [placed on the Official List of Generic Names in Zoology (ICZN 2006a)]. Comment: replacement name for Bolboceratini of authors because the concept of *Bolboceras* Kirby, 1819 was fixed differently by the Commission (ICZN 2006a).

### Tribe Stenaspidiini

Nikolajev, 2003

Stenaspidiini Nikolajev, 2003a: 190, in key [stem: *Stenaspidi-*]. Type genus: *Stenaspidius* Westwood, 1848.

### Subfamily Geotrupinae

Latreille, 1802

Geotrupini Latreille, 1802: 142 [stem: *Geotrup-*]. Type genus: *Geotrupes* Latreille, 1797 [placed on the Official List of Generic Names in Zoology (ICZN 1955b)]. Comment: name placed on the Official List of Family-Group Names in Zoology (ICZN 1955c, as Geotrupini Latreille, 1806).

### Tribe Ceratotrupini

Zunino, 1984

Ceratotrupini Zunino, 1984: 89 [stem: *Ceratotrup-*]. Type genus: *Ceratotrupes* Jekel, 1866.

### Tribe Enoplotrupini

Paulian, 1945

Enoplotrupini Paulian, 1945: 40, in key [stem: *Enoplotrup-*]. Type genus: *Enoplotrupes* Lucas, 1869.
Chromogeotrupini Zunino, 1984: 30 [stem: *Chromogeotrup-*]. Type genus: *Chromogeotrupes* Bovo and Zunino, 1983.

### †Tribe Cretogeotrupini

Nikolajev, 1996

Cretogeotrupini Nikolajev, 1996: 97 [stem: *Cretogeotrup-*]. Type genus: *Cretogeotrupes* Nikolajev, 1992.

### Tribe Geotrupini

Latreille, 1802

Geotrupini Latreille, 1802: 142 [stem: *Geotrup-*]. Type genus: *Geotrupes* Latreille, 1797 [placed on the Official List of Generic Names in Zoology (ICZN 1955b)]. Comment: name placed on the Official List of Family-Group Names in Zoology (ICZN 1955c, as Geotrupini Latreille, 1806).

### Tribe Lethrini

Oken, 1843

Lethren Oken, 1843: 484 [stem: *Lethr-*]. Type genus: *Lethrus* Scopoli, 1777. Comment: original vernacular name available (Art. 11.7.2): first used in latinized form and generally accepted as in Tulk (1847: 614, as Lethri); name previously attributed to Mulsant and Rey (1871a).

### Family Belohinidae

Paulian, 1959

Belohininae Paulian, 1959: 40 [stem: *Belohin-*]. Type genus: *Belohina* Paulian, 1959.

### Family Passalidae

Leach, 1815

Passalida Leach, 1815: 100 [stem: *Passal-*]. Type genus: *Passalus* Fabricius, 1792.

### Subfamily Aulacocyclinae

Kaup, 1868

Aulacocyclinae Kaup, 1868a: 4 [stem: *Aulacocycl-*]. Type genus: *Aulacocyclus* Kaup, 1868.

### Tribe Aulacocyclini

Kaup, 1868

Aulacocyclinae Kaup, 1868a: 4 [stem: *Aulacocycl-*]. Type genus: *Aulacocyclus* Kaup, 1868.

### Tribe Ceracupedini

Boucher, 2006

Ceracupini Boucher, 2006: 319 [stem: *Ceracuped-*]. Type genus: *Ceracupes* Kaup, 1871. Comment: incorrect original stem formation, not in prevailing usage.

### Subfamily Passalinae

Leach, 1815

Passalida Leach, 1815: 100 [stem: *Passal-*]. Type genus: *Passalus* Fabricius, 1792.

### Tribe Leptaulacini

Kaup, 1871

Leptaulaceae Kaup, 1871: 28 [stem: *Leptaulac-*]. Type genus: *Leptaulax* Kaup, 1868.

### Tribe Macrolinini

Kaup, 1871

Macrolineae Kaup, 1871: 42 [stem: *Macrolin-*]. Type genus: *Macrolinus* Kaup, 1868. Comment: First Reviser found (Macrolinini Kaup, 1871 vs Aceraiini Kaup, 1871) is Gravely (1918: 76).
Aceraiae Kaup, 1871: 47 [stem: *Acerai-*]. Type genus: *Aceraius* Kaup, 1868.
Eriocnemiae Kaup, 1871: 35 [stem: *Eriocnemid-*]. Type genus: *Eriocnemis* Kaup, 1868 [preoccupied genus name, not *Eriocnemis* Reichenbach, 1849 [Aves]; syn. of *Pelopides* Kuwert, 1896]. Comment: permanently invalid (Art. 39): based on preoccupied type genus; incorrect original stem formation, not in prevailing usage.
Gonatinae Kuwert, 1891: 169 [stem: *Gonat-*]. Type genus: *Gonatas* Kaup, 1871.
Mastachilinae Kuwert, 1891: 167 [stem: *Mastachil-*]. Type genus: *Mastachilus* Kaup, 1868.
Pharochilinae Kuwert, 1891: 166 [stem: *Pharochil-*]. Type genus: *Pharochilus* Kaup, 1868.
Tarquininae Kuwert, 1891: 164 [stem: *Tarquini-*]. Type genus: *Tarquinius* Kuwert, 1891. Comment: incorrect original stem formation, not in prevailing usage.
Vellejinae Kuwert, 1891: 167 [stem: *Vellej-*]. Type genus: *Vellejus* Kaup, 1871 [preoccupied genus name, not *Vellejus* Mannerheim, 1830 [Coleoptera: Staphylinidae], not *Vellejus* Stål, 1865 [Hemiptera]; syn. of *Labienus* Kaup, 1871]. Comment: permanently invalid (Art. 39): based on preoccupied type genus.
Aureliinae Kuwert, 1896: 230 [stem: *Aureli-*]. Type genus: *Aurelius* Kuwert, 1891.
Lachinae Kuwert, 1896: 230 [stem: *Lach-*]. Type genus: *Laches* Kaup, 1871 [preoccupied genus name, not *Laches* Gistel, 1848 [Hymenoptera], not *Laches* Thorell, 1869 [Arachnida]; syn. of *Aceraius* Kaup, 1868]. Comment: permanently invalid (Art. 39): based on preoccupied type genus.
Pelopinae Kuwert, 1896: 229 [stem: *Pelopid-*]. Type genus: *Pelopides* Kuwert, 1896. Comment: incorrect original stem formation, not in prevailing usage.
Pleurariinae Kuwert, 1896: 224 [stem: *Pleurari-*]. Type genus: *Pleurarius* Kaup, 1868.
Gnaphalocneminae Gravely, 1914: 194 [stem: *Gnaphalocnemid-*]. Type genus: *Gnaphalocnemis* Heller, 1900 [syn. of *Pelopides* Kuwert, 1896]. Comment: incorrect original stem formation, not in prevailing usage.
Austropassalinae Mjöberg, 1917: 11 [stem: *Austropassal-*]. Type genus: *Austropassalus* Mjöberg, 1917.

### Tribe Passalini

Leach, 1815

Passalida Leach, 1815: 100 [stem: *Passal-*]. Type genus: *Passalus* Fabricius, 1792.
Neleinae Kaup, 1869: 28 [stem: *Nele-*]. Type genus: *Neleus* Kaup, 1869 [preoccupied genus name, not *Neleus* Desbonne and Schramm, 1867 [Crustacea]; syn. of *Passalus* Fabricius, 1792]. Comment: permanently invalid (Art. 39): based on preoccupied type genus.
Pertinaceae Kaup, 1871: 89 [stem: *Pertinac-*]. Type genus: *Pertinax* Kaup, 1869.
Phoroneae Kaup, 1871: 97 [stem: *Phorone-*]. Type genus: *Phoroneus* Kaup, 1869 [preoccupied genus name, not *Phoroneus* Stål, 1865 [Hemiptera]; syn. of *Passalus* Fabricius, 1792]. Comment: permanently invalid (Art. 39): based on preoccupied type genus.
Mitrorhinae Kuwert, 1891: 190 [stem: *Mitrorhin-*]. Type genus: *Mitrorhinus* Kaup, 1871.
Paxillinae Kuwert, 1891: 182 [stem: *Paxill-*]. Type genus: *Paxillus* W. S. MacLeay, 1819.
Petrejinae Kuwert, 1891: 176 [stem: *Petrej-*]. Type genus: *Petrejus* Kaup, 1869.
Nelidinae Kuwert, 1896: 222 [stem: *Neleid-*]. Type genus: *Neleides* Kaup, 1869. Comment: incorrect original stem formation, not in prevailing usage.
Ptichopinae Kuwert, 1896: 224 [stem: *Ptichopod-*]. Type genus: *Ptichopus* Kaup, 1869. Comment: incorrect original stem formation, not in prevailing usage.
Rhodacanthopinae Kuwert, 1896: 222 [stem: *Rhodocanthopod-*]. Type genus: *Rhodocanthopus* Kaup, 1871. Comment: incorrect original stem formation, not in prevailing usage.
Vatiniinae Kuwert, 1896: 226 [stem: *Vatini-*]. Type genus: *Vatinius* Kaup, 1869 [preoccupied genus name, not *Vatinius* Stål, 1865 [Hemiptera]; syn. of *Passalus* Fabricius, 1792]. Comment: permanently invalid (Art. 39): based on preoccupied type genus.

### Tribe Proculini

Kaup, 1868

Proculinae Kaup, 1868b: 8 [stem: *Procul-*]. Type genus: *Proculus* Kaup, 1868.
Pseudacantheae Kaup, 1871: 73 [stem: *Pseudacanth-*]. Type genus: *Pseudacanthus* Kaup, 1869.
Oileinae Kuwert, 1891: 192 [stem: *Oile-*]. Type genus: *Oileus* Kaup, 1869.
Sertorinae Kuwert, 1891: 175 [stem: *Sertori-*]. Type genus: *Sertorius* Kaup, 1871 [preoccupied genus name, not *Sertorius* Stål, 1866 [Hemiptera]; syn. of *Arrox* Zang, 1905]. Comment: permanently invalid (Art. 39): based on preoccupied type genus; incorrect original stem formation, not in prevailing usage.
Unduliferinae Kuwert, 1891: 176 [stem: *Undulifer-*]. Type genus: *Undulifer* Kaup, 1869.
Veturinae Kuwert, 1891: 173 [stem: *Veturi-*]. Type genus: *Veturius* Kaup, 1871. Comment: incorrect original stem formation, not in prevailing usage.
Popiliinae Kuwert, 1896: 221 [stem: *Popili-*]. Type genus: *Popilius* Kaup, 1871.
Proculejinae Kuwert, 1896: 221 [stem: *Proculej-*]. Type genus: *Proculejus* Kaup, 1868.
Spuriinae Kuwert, 1896: 221 [stem: *Spuri-*]. Type genus: *Spurius* Kaup, 1871.
Vindicinae Kuwert, 1896: 227 [stem: *Vindic-*]. Type genus: *Vindex* Kaup, 1871.

### Tribe Solenocyclini

Kaup, 1871

Solenocycleae Kaup, 1871: 24 [stem: *Solenocycl-*]. Type genus: *Solenocyclus* Kaup, 1868. Comment: First Reviser found (Solenocyclini Kaup, 1871 vs Stephanocephalini Kaup, 1871) is Boucher (2006).
Stephanocephaleae Kaup, 1871: 78 [stem: *Stephanocephal-*]. Type genus: *Stephanocephalus* Kaup, 1868.
Ciceroninae Kuwert, 1891: 183 [stem: *Ciceroni-*]. Type genus: *Ciceronius* Kaup, 1871. Comment: incorrect original stem formation, not in prevailing usage.
Erionominae Kuwert, 1891: 176 [stem: *Erionom-*]. Type genus: *Erionomus* Kaup, 1868.
Flamininae Kuwert, 1891: 185 [stem: *Flamini-*]. Type genus: *Flaminius* Kuwert, 1891. Comment: incorrect original stem formation, not in prevailing usage.
Semicyclinae Kuwert, 1891: 177 [stem: *Semicycl-*]. Type genus: *Semicyclus* Kaup, 1871.

### Family Trogidae

MacLeay, 1819

Trogidae W. S. MacLeay, 1819: 59 [stem: *Trog-*]. Type genus: *Trox* Fabricius, 1775.

### †Subfamily Avitortorinae

Nikolajev, 2007

Avitortorinae Nikolajev, 2007a: 110 [stem: *Avitortor-*]. Type genus: *Avitortor* Ponomarenko, 1977.

### Subfamily Troginae

MacLeay, 1819

Trogidae W. S. MacLeay, 1819: 59 [stem: *Trog-*]. Type genus: *Trox* Fabricius, 1775.
Phoberidae Gistel, 1848: [5] [stem: *Phober-*]. Type genus: *Phoberus* W. S. MacLeay, 1819.

### Subfamily Omorginae

Nikolajev, 2005

Omorgini Nikolajev, 2005a: 322 [stem: *Omorg-*]. Type genus: *Omorgus* Erichson, 1847.

### Family Glaresidae

Kolbe, 1905

Glaresini Kolbe, 1905: 543 [stem: *Glares-*]. Type genus: *Glaresis* Erichson, 1848.

### Family Diphyllostomatidae

Holloway, 1972

Diphyllostomatidae Holloway, 1972: 38 [stem: *Diphyllostomat-*]. Type genus: *Diphyllostoma* Fall, 1901.

### Family Lucanidae

Latreille, 1804

Lucanides Latreille, 1804c: 149 [stem: *Lucan-*]. Type genus: *Lucanus* Scopoli, 1763.

### †Subfamily Protolucaninae

Nikolajev, 2007

Protolucaninae Nikolajev, 2007a: 18 [stem: *Protolucan-*]. Type genus: *Protolucanus* Nikolajev, 2007.

### Subfamily Aesalinae

MacLeay, 1819

Aesalidae W. S. MacLeay, 1819: 102 [stem: *Aesal-*]. Type genus: *Aesalus* Fabricius, 1801.

### Tribe Aesalini

MacLeay, 1819

Aesalidae W. S. MacLeay, 1819: 102 [stem: *Aesal-*]. Type genus: *Aesalus* Fabricius, 1801.

### Tribe Ceratognathini

Sharp, 1899

Ceratognathini Sharp, 1899a: 194 [stem: *Ceratognath-*]. Type genus: *Ceratognathus* Westwood, 1838.

### Tribe Nicagini

LeConte, 1861

Nicagini J. L. LeConte, 1861: 130 [stem: *Nicag-*]. Type genus: *Nicagus* J. L. LeConte, 1861.

### †Subfamily Ceruchitinae

Nikolajev, 2006

Ceruchitinae Nikolajev, 2006: 133 [stem: *Ceruchit-*]. Type genus: *Ceruchites* Statz, 1952.

### Subfamily Syndesinae

MacLeay, 1819

Syndesidae W. S. MacLeay, 1819: 103 [stem: *Syndes-*]. Type genus: *Syndesus* W. S. MacLeay, 1819.
Sinodendriens Mulsant, 1842: 600 [stem: *Sinodendr-*]. Type genus: *Sinodendron* Hellwig, 1791. Comment: original vernacular name available (Art. 11.7.2): first used in latinized form by Imhoff (1856: xii, as Sinodendridae), generally accepted as in Ratcliffe (2002: 8, as Sinodendrini).
Ceruchites Jacquelin du Val, 1859: 4 [stem: *Ceruch-*]. Type genus: *Ceruchus* W. S. MacLeay, 1819. Comment: original vernacular name available (Art. 11.7.2): first used in latinized form by J. L. LeConte (1861: 121, as Ceruchini), generally accepted as in Ratcliffe (2002: 8, as Ceruchini).

### Subfamily Lampriminae

MacLeay, 1819

Lamprimidae W. S. MacLeay, 1819: 97 [stem: *Lamprim-*]. Type genus: *Lamprima* Latreille, 1804.

### Tribe Lamprimini

MacLeay, 1819

Lamprimidae W. S. MacLeay, 1819: 97 [stem: *Lamprim-*]. Type genus: *Lamprima* Latreille, 1804.

### Tribe Streptocerini

Kikuta, 1986

Streptocerini Kikuta, 1986: 131 [stem: *Streptocer-*]. Type genus: *Streptocerus* Fairmaire, 1850.

### Subfamily Lucaninae

Latreille, 1804

Lucanides Latreille, 1804c: 149 [stem: *Lucan-*]. Type genus: *Lucanus* Scopoli, 1763.

### Tribe Chiasognathini

Burmeister, 1847

Chiasognathidae H. C. C. Burmeister, 1847: 334 [stem: *Chiasognath-*]. Type genus: *Chiasognathus* Stephens, 1831.

### Tribe Lucanini

Latreille, 1804

Lucanides Latreille, 1804c: 149 [stem: *Lucan-*]. Type genus: *Lucanus* Scopoli, 1763. Comment: published 7 March 1804; this family-group name was also used in the same year by Latreille (1804b [between 19 August and 17 September]: 234, as Lucanides).
Figulidae H. C. C. Burmeister, 1847: 428 [stem: *Figul-*]. Type genus: *Figulus* W. S. MacLeay, 1819.
Corypticidae Gistel, 1848: [5] [stem: *Coryptic-*]. Type genus: *Corypticus* Sturm, 1843 [the genus was spelled *Coryptius* (Sturm 1843: 136) and *Corypticus* (Sturm 1843: 347) in the paper making the genus name available but the latter spelling is in prevailing usage and is the correct spelling].
Dorcidae Parry, 1864: 86 [stem: *Dorc-*]. Type genus: *Dorcus* W. S. MacLeay, 1819.
Cladognathidae Parry, 1870: 75 [stem: *Cladognath-*]. Type genus: *Cladognathus* H. C. C. Burmeister, 1847 [syn. of *Prosopocoilus* Hope, 1845].
Odontolabidae Parry, 1870: 106 [stem: *Odontolabid-*]. Type genus: *Odontolabis* Hope, 1842. Comment: incorrect original stem formation, not in prevailing usage.
Nigidiini Jakobson, 1911b: 142 [stem: *Nigidi-*]. Type genus: *Nigidius* W. S. MacLeay, 1819. Comment: Nigidiini was attributed to Benesh (1960) and treated as unavailable by A. B. T. Smith (2006: 154).
Leptinopterini Jakobson, 1911b: 142 [stem: *Leptinopter-*]. Type genus: *Leptinopterus* Hope, 1838 [unjustified emendation of *Leptynopterus* by Hope (1845: 5), in prevailing usage and so deemed to be a justified emendation (Article 33.2.3.1)].
Rhaetulinae Miwa, 1931: 323 [stem: *Rhaetul-*]. Type genus: *Rhaetulus* Westwood, 1871.
Penichrolucaninae Arrow, 1950: 233 [stem: *Penichrolucan-*]. Type genus: *Penichrolucanus* Deyrolle, 1863.
*Chalcodinae Didier and Séguy, 1953: 91 [stem: *Chalcod-*]. Type genus: *Chalcodes* H. C. C. Burmeister, 1847. Comment: unavailable family-group name, proposed after 1930 without description or bibliographic reference to such a description (Art. 13.1).
Dendeziini Benesh, 1955b: 72 [stem: *Dendezi-*]. Type genus: *Dendezia* Basilewsky, 1952.
Lissotini Benesh, 1955b: 73 [stem: *Lissotet-*]. Type genus: *Lissotes* Westwood, 1855. Comment: incorrect original stem formation, not in prevailing usage.
Sclerostomini Benesh, 1955a: 97 [stem: *Sclerostom-*]. Type genus: *Sclerostomus* H. C. C. Burmeister, 1847.
Scortizini Benesh, 1955a: 103 [stem: *Scortiz-*]. Type genus: *Scortizus* Westwood, 1834.
*Prosopocoilini Benesh, 1960: 50 [stem: *Prosopocoil-*]. Type genus: *Prosopocoilus* Hope, 1845. Comment: unavailable family-group name, proposed after 1930 without description or bibliographic reference to such a description (Art. 13.1).
*Pseudodorcini Benesh, 1960: 97 [stem: *Pseudodorc-*]. Type genus: *Pseudodorcus* Parry, 1870. Comment: unavailable family-group name, proposed after 1930 without description or bibliographic reference to such a description (Art. 13.1).
*Rhyssonotini Benesh, 1960: 148 [stem: *Ryssonot-*]. Type genus: *Ryssonotus* W. S. MacLeay, 1819 [as *Rhyssonotus*, unjustified emendation of type genus name by Agassiz (1846b: 329), not in prevailing usage]. Comment: unavailable family-group name, proposed after 1930 without description or bibliographic reference to such a description (Art. 13.1); incorrect original stem formation, not in prevailing usage.
*Chalcodinae J. P. Lacroix, 1979: 258 [stem: *Chalcod-*]. Type genus: *Chalcodes* H. C. C. Burmeister, 1847. Comment: unavailable family-group name, proposed after 1930 without description or bibliographic reference to such a description (Art. 13.1).
Pholidotini Kikuta, 1986: 131 [stem: *Pholidot-*]. Type genus: *Pholidotus* W. S. MacLeay, 1819 [preoccupied genus name, not *Pholidotus* Brisson, 1762 [Mammalia]; syn. of *Casignetus* W. S. MacLeay, 1819]. Comment: permanently invalid (Art. 39): based on preoccupied type genus.
*Aegini Maes, 1992b: 97 [stem: *Aeg-*]. Type genus: *Aegus* W. S. MacLeay, 1819. Comment: unavailable family-group name, proposed after 1930 without description or bibliographic reference to such a description (Art. 13.1); Aegidae A. White, 1850 (type genus *Aega* Leach, 1815) is currently used as valid in Isopoda and therefore anyone wishing to base a new family-group name on the genus *Aegus* W. S. MacLeay should change the stem in order to avoid homonymy with the isopod name.
*Allotopini Maes, 1992a: 56 [stem: *Allotop-*]. Type genus: *Allotopus* Albers, 1894. Comment: unavailable family-group name, proposed after 1930 without description or bibliographic reference to such a description (Art. 13.1).
*Casignetini Maes, 1992b: 61 [stem: *Casignet-*]. Type genus: *Casignetus* W. S. MacLeay, 1819. Comment: unavailable family-group name, proposed after 1930 without description or bibliographic reference to such a description (Art. 13.1); Casignetini Falkovitsh, 1972 (type genus *Casigneta* Wallengren, 1881) proposed in Lepidoptera is permanently invalid since it is based on a preoccupied type genus name.
*Colophonini Maes, 1992b: 107 [stem: *Colophon-*]. Type genus: *Colophon* Gray, 1832. Comment: unavailable family-group name, proposed after 1930 without description or bibliographic reference to such a description (Art. 13.1).
*Cyclommatini Maes, 1992a: 56 [stem: *Cyclommat-*]. Type genus: *Cyclommatus* Parry, 1863. Comment: unavailable family-group name, proposed after 1930 without description or bibliographic reference to such a description (Art. 13.1).
*Homoderini Maes, 1992b: 85 [stem: *Homoder-*]. Type genus: *Homoderus* Parry, 1863. Comment: unavailable family-group name, proposed after 1930 without description or bibliographic reference to such a description (Art. 13.1).
Casignetini Reid, 1999: 175 [stem: *Casignet-*]. Type genus: *Casignetus* W. S. MacLeay, 1819. Comment: replacement name for Pholidotini Kikuta, 1986 because of the homonymy of the type genus.
Brasilucanini Nikolajev, 1999a: 171 [stem: *Brasilucan-*]. Type genus: *Brasilucanus* Vulcano and Pereira, 1961.

### Tribe Platycerini

Mulsant, 1842

Platycéraires Mulsant, 1842: 593 [stem: *Platycer-*]. Type genus: *Platycerus* Geoffroy, 1762 [placed on the Official List of Generic Names in Zoology (ICZN 1994a)]. Comment: original vernacular name available (Art. 11.7.2): first used in latinized form by Gistel (1856a: 365, as Platyceridae), generally accepted as in Paulsen and Hawks (2008: 1, as Platycerini).
Systenocerini Portevin, 1931: 2, in key [stem: *Systenocer-*]. Type genus: *Systenocerus* Weise, 1883 [syn. of *Platycerus* Geoffroy, 1762].

### Tribe Platyceroidini

Paulsen and Hawks, 2008

Platyceroidini Paulsen and Hawks, 2008: 2 [stem: *Platyceroid-*]. Type genus: *Platyceroides* Benesh, 1946.

### †Subfamily Paralucaninae

Nikolajev, 2000

Paralucaninae Nikolajev, 2000b: S328 [stem: *Paralucan-*]. Type genus: *Paralucanus* Nikolajev, 2000.

### Family Ochodaeidae

Mulsant and Rey, 1871

Ochodéens Mulsant and Rey, 1871b: 493 [stem: *Ochodae-*]. Type genus: *Ochodaeus* Dejean, 1821.

### †Subfamily Cretochodaeinae

Nikolajev, 1995

Cretochodaeini Nikolajev, 1995a: 78 [stem: *Cretochodae-*]. Type genus: *Cretochodaeus* Nikolajev, 1995.

### Subfamily Ochodaeinae

Mulsant and Rey, 1871

Ochodéens Mulsant and Rey, 1871b: 493 [stem: *Ochodae-*]. Type genus: *Ochodaeus* Dejean, 1821.

### Tribe Enodognathini

Scholtz, 1988

Endognathini Scholtz, 1988: 228 [stem: *Enodognath-*]. Type genus: *Enodognathus* Benderitter, 1920 [as *Endognathus*, incorrect subsequent spelling of type genus name, not in prevailing usage]. Comment: incorrect original stem formation, not in prevailing usage; correction of stem by Scholtz (1991: 30).

### Tribe Ochodaeini

Mulsant and Rey, 1871

Ochodéens Mulsant and Rey, 1871b: 493 [stem: *Ochodae-*]. Type genus: *Ochodaeus* Dejean, 1821. Comment: original vernacular name available (Art. 11.7.2): first used in latinized form by Arrow (1904: 747, as Ochodaeinae), generally accepted as in A. B. T. Smith (2006: 155, as Ochodaeidae); incorrect original stem formation, not in prevailing usage.

### Subfamily Chaetocanthinae

Scholtz, 1988

Chaetocanthinae Scholtz, 1988: 231 [stem: *Chaetocanth-*]. Type genus: *Chaetocanthus* Péringuey, 1901. Comment: precedence (Chaetocanthinae Scholtz, 1988 vs Pseudochodaeinae Scholtz, 1988 vs Synochodaeinae Scholtz, 1988) given to taxon originally proposed at the higher rank (Art. 24.1).

### Tribe Chaetocanthini

Scholtz, 1988

Chaetocanthinae Scholtz, 1988: 231 [stem: *Chaetocanth-*]. Type genus: *Chaetocanthus* Péringuey, 1901.

### Tribe Pseudochodaeini

Scholtz, 1988

Pseudochodaeini Scholtz, 1988: 235 [stem: *Pseudochodae-*]. Type genus: *Pseudochodaeus* Carlson and Ritcher, 1974.

### Tribe Synochodaeini

Scholtz, 1988

Synochodaeini Scholtz, 1988: 237 [stem: *Synochodae-*]. Type genus: *Synochodaeus* Kolbe, 1907.

### Family Hybosoridae

Erichson, 1847

Hybosoridae Erichson, 1847a: 104 [stem: *Hybosor-*]. Type genus: *Hybosorus* W. S. MacLeay, 1819.

### †Subfamily Mimaphodiinae

Nikolajev, 2007

Mimaphodiinae Nikolajev, 2007b: 47 [stem: *Mimaphodi-*]. Type genus: *Mimaphodius* Nikolajev, 2007.

### Subfamily Anaidinae

Nikolajev, 1996

Anaidini Nikolajev, 1996: 94 [stem: *Anaid-*]. Type genus: *Anaides* Westwood, 1842.
Cryptogeniini Howden, 2001: 199 [stem: *Cryptogeni-*]. Type genus: *Cryptogenius* Westwood, 1842.

### Subfamily Ceratocanthinae

Martínez, 1968

Ceratocanthini Martínez, 1968: 14 [stem: *Ceratocanth-*]. Type genus: *Ceratocanthus* A. White, 1842.

### Tribe Ceratocanthini

Martínez, 1968

Acanthocérides Lacordaire, 1856: 155 [stem: *Acanthocer-*]. Type genus: *Acanthocerus* W. S. MacLeay, 1819 [preoccupied genus name, not *Acanthocerus* Palisot de Beauvois, 1818 [Hemiptera]; syn. of *Ceratocanthus* A. White, 1842]. Comment: original vernacular name available (Art. 11.7.2): first used in latinized form and generally accepted as in J. L. LeConte (1861: 129, as Acanthocerini); permanently invalid (Art. 39): based on preoccupied type genus.
Ceratocanthini Martínez, 1968: 14 [stem: *Ceratocanth-*]. Type genus: *Ceratocanthus* A. White, 1842. Comment: replacement name for Acanthocerini Lacordaire, 1856 because of the homonymy of the type genus.

### Tribe Ivieolini

Howden and Gill, 2000

Ivieolini Howden and Gill, 2000: 315 [stem: *Ivieol-*]. Type genus: *Ivieolus* Howden and Gill, 1988.

### Tribe Scarabatermitini

Nikolajev, 1999

Scarabatermitini Nikolajev, 1999b: 175 [stem: *Scarabatermit-*]. Type genus: *Scarabatermes* Howden, 1973.

### Subfamily Hybosorinae

Erichson, 1847

Hybosoridae Erichson, 1847a: 104 [stem: *Hybosor-*]. Type genus: *Hybosorus* W. S. MacLeay, 1819.

### Subfamily Liparochrinae

Ocampo, 2006

Liparochrinae Ocampo, 2006: 29 [stem: *Liparochr-*]. Type genus: *Liparochrus* Erichson, 1848.

### Subfamily Pachyplectrinae

Ocampo, 2006

Pachyplectrinae Ocampo, 2006: 30 [stem: *Pachyplectr-*]. Type genus: *Pachyplectrus* J. L. LeConte, 1874.

### Family Glaphyridae

MacLeay, 1819

Glaphyridae W. S. MacLeay, 1819: 76 [stem: *Glaphyr-*]. Type genus: *Glaphyrus* Latreille, 1802.

### Subfamily Glaphyrinae

MacLeay, 1819

Glaphyridae W. S. MacLeay, 1819: 76 [stem: *Glaphyr-*]. Type genus: *Glaphyrus* Latreille, 1802.

### Subfamily Amphicominae

Blanchard, 1845

Amphicomites Blanchard, 1845a: 211 [stem: *Amphicom-*]. Type genus: *Amphicoma* Latreille, 1807. Comment: original vernacular name available (Art. 11.7.2): first used in latinized form and generally accepted as in Blanchard (1850: 52, as Amphicomitae); name treated as unavailable by A. B. T. Smith (2006: 157).

### †Subfamily Cretoglaphyrinae

Nikolajev, 2005

Cretoglaphyrini Nikolajev, 2005b: 70 [stem: *Cretoglaphyr-*]. Type genus: *Cretoglaphyrus* Nikolajev, 2005.

### Family Scarabaeidae

Latreille, 1802

Scarabaeïdes Latreille, 1802: 144 [stem: *Scarabae-*]. Type genus: *Scarabaeus* Linnaeus, 1758.

### †Subfamily Lithoscarabaeinae

Nikolajev, 1992

Lithoscarabaeinae Nikolajev, 1992: 76 [stem: *Lithoscarabae-*]. Type genus: *Lithoscarabaeus* Nikolajev, 1992.

### Subfamily Chironinae

Blanchard, 1845

Chironites Blanchard, 1845a: 225 [stem: *Chiron-*]. Type genus: *Chiron* W. S. MacLeay, 1819. Comment: original vernacular name available (Art. 11.7.2): first used in latinized form by Harold (1868: 278, as Chironidae), generally accepted as in A. B. T. Smith (2006: 157, as Chironinae).

### Subfamily Aegialiinae

Laporte, 1840

Aegialites Laporte, 1840b: 99 [stem: *Aegiali-*]. Type genus: *Aegialia* Latreille, 1807. Comment: original vernacular name available (Art. 11.7.2): first used in latinized form by Harold (1868: 278, as Aegialidae [incorrect stem formation]), generally accepted as in A. B. T. Smith (2006: 157, as Aegialiinae); incorrect original stem formation, not in prevailing usage.
Silluviinae Landin, 1950: 3 [stem: *Silluvi-*]. Type genus: *Silluvia* Landin, 1950.
Saprini Nikolajev, 2008: 149 [stem: *Sapr-*]. Type genus: *Saprus* Blackburn, 1904 [preoccupied genus name, not *Saprus* Gistel, 1856 [Coleoptera: Sphaeriusidae]; syn. of *Sapriniana* Strand, 1917]. Comment: permanently invalid (Art. 39): based on preoccupied type genus.

### Subfamily Eremazinae

Iablokoff-Khnzorian, 1977

Eremazini Iablokoff-Khnzorian, 1977 [3 October]: 168 [stem: *Eremaz-*]. Type genus: *Eremazus* Mulsant, 1851.
Eremazina Stebnicka, 1977 [“31 December”]: 412 [stem: *Eremaz-*]. Type genus: *Eremazus* Mulsant, 1851. Comment: proposed as new, without reference to Eremazini Iablokoff-Khnzorian, 1977.

### Subfamily Aphodiinae

Leach, 1815

Aphodida Leach, 1815: 97 [stem: *Aphodi-*]. Type genus: *Aphodius* Illiger, 1798.

### Tribe Aphodiini

Leach, 1815

Aphodida Leach, 1815: 97 [stem: *Aphodi-*]. Type genus: *Aphodius* Illiger, 1798.

### Subtribe Aphodiina

Leach, 1815

Aphodida Leach, 1815: 97 [stem: *Aphodi-*]. Type genus: *Aphodius* Illiger, 1798.
*Ammoeciates Mulsant, 1842: 301 [stem: *Ammoeci-*]. Type genus: *Ammoecius* Mulsant, 1842. Comment: original vernacular name unavailable (Art. 11.7.2): not subsequently latinized.
*Plagiogonates Mulsant and Rey, 1871a: 609 [stem: *Plagiogon-*]. Type genus: *Plagiogonus* Mulsant, 1842. Comment: original vernacular name unavailable (Art. 11.7.2): not subsequently latinized.
*Heptaulacates Mulsant and Rey, 1871a: 585 [stem: *Heptaulac-*]. Type genus: *Heptaulacus* Mulsant, 1842. Comment: original vernacular name unavailable (Art. 11.7.2): not subsequently latinized.

### Subtribe Didactyliina

Pittino, 1985

Didactyliina Pittino, 1985: 270, in key [stem: *Didactyli-*]. Type genus: *Didactylia* d’Orbigny, 1896.

### Subtribe Proctophanina

Stebnicka and Howden, 1995

Proctophanini Stebnicka and Howden, 1995: 742 [stem: *Proctophan-*]. Type genus: *Proctophanes* Harold, 1861.

### Tribe Corythoderini

Schmidt, 1910

Corythoderina A. Schmidt, 1910a: 137 [stem: *Corythoder-*]. Type genus: *Corythoderus* Klug, 1845. Comment: the earliest known publication date of this work is 31 December 1910 (see Evenhuis, 1994: 55); although Corythoderina A. Schmidt (1910b: 93) was published on 30 September 1910, we use A. Schmidt, 1910a as the correct original work since “Corythoderina Schmidt 1910: 137” was used as the correct citation for this name by A. Schmidt himself (1910b: 93).

### Tribe Eupariini

Schmidt, 1910 nomen protectum

Ataenidae Harold, 1868: 278 [stem: *Ataeni-*]. Type genus: *Ataenius* Harold, 1867 [placed on the Official List of Generic Names in Zoology (ICZN 2010a)]. Comment: *nomen oblitum* (see A. B. T. Smith 2006: 158).
*Hexalates Mulsant and Rey, 1871a: 605 [stem: *Hexal-*]. Type genus: *Hexalus* Mulsant and Rey, 1871 [syn. of *Ataenius* Harold, 1867]. Comment: original vernacular name unavailable (Art. 11.7.2): not subsequently latinized.
Eupariina A. Schmidt, 1910a: 102 [stem: *Eupari-*]. Type genus: *Euparia* Lepeletier and Audinet-Serville, 1828. Comment: *nomen protectum* (see A. B. T. Smith 2006: 158); senior homonym of Eupariini B. D. Valentine, 1960 (type genus *Euparius* Schönherr, 1823) in Anthribidae; this case is to be referred to the Commission to remove the homonymy (Art. 55.3.1); the earliest known publication date of this work is 31 December 1910 (see Evenhuis, 1994: 55); although Eupariina A. Schmidt (1910b: 71) was published on 30 September 1910, we use A. Schmidt, 1910a as the correct original work since “Eupariina Schmidt 1910: 102” was used as the correct citation for this name by A. Schmidt himself (1910b: 71).
Lomanoxiini Stebnicka, 1999: 280 [stem: *Lomanoxi-*]. Type genus: *Lomanoxia* Martínez, 1951.

### Tribe Odontolochini

Stebnicka and Howden, 1996

Odontolochini Stebnicka and Howden, 1996: 99 [stem: *Odontoloch-*]. Type genus: *Odontolochus* Schmidt, 1916.

### Tribe Odochilini

Rakovič, 1987

Odochilini Rakovič, 1987: 29 [stem: *Odochil-*]. Type genus: *Odochilus* Harold, 1877.

### Tribe Psammodiini

Mulsant, 1842

Psammodiaires Mulsant, 1842: 317 [stem: *Psammodi-*]. Type genus: *Psammodius* Fallén, 1807.

### Subtribe Phycocina

Landin, 1960

Phycochi Landin, 1960: 59 [stem: *Phycoc-*]. Type genus: *Phycocus* Broun, 1886 [as *Phycochus*, incorrect subsequent spelling of type genus name, not in prevailing usage (see Stebnicka 2001)]. Comment: incorrect original stem formation, not in prevailing usage.
Phycochini Rakovič and Král, 1997: 59 [stem: *Phycoc-*]. Type genus: *Phycocus* Broun, 1886 [as *Phycochus*, incorrect subsequent spelling of type genus name, not in prevailing usage (see Stebnicka 2001)]. Comment: incorrect original stem formation, not in prevailing usage; family-group name proposed as new without reference to Phycochi Landin, 1960.

### Subtribe Psammodiina

Mulsant, 1842

Psammodiaires Mulsant, 1842: 317 [stem: *Psammodi-*]. Type genus: *Psammodius* Fallén, 1807. Comment: original vernacular name available (Art. 11.7.2): first used in latinized form by Harold (1868: 278, as Psammodidae [incorrect stem formation]), generally accepted as in A. B. T. Smith (2006: 159, as Psammodiini).
Pleurophorates Mulsant, 1842: 304 [stem: *Pleurophor-*]. Type genus: *Pleurophorus* Mulsant, 1842. Comment: original vernacular name available (Art. 11.7.2): first used in latinized form and generally accepted as in Ádám (1994: 15, as Pleurophorini).
Psammobiina Reitter, 1909: 303 [stem: *Psammobi-*]. Type genus: *Psammobius* Heer, 1841 [syn. of *Psammodius* Fallén, 1807].

### Subtribe Rhyssemina

Pittino and Mariani, 1986

Rhyssemina Pittino and Mariani, 1986: 17, in key [stem: *Rhyssem-*]. Type genus: *Rhyssemus* Mulsant, 1842.

### Tribe Rhyparini

Schmidt, 1910

Rhyparina A. Schmidt, 1910a: 130 [stem: *Rhypar-*]. Type genus: *Rhyparus* Westwood, 1845 [*Rhyparus* is an unjustified emendation of *Ryparus* Westwood, 1845 by Agassiz (1846b: 328), in prevailing usage, and so deemed to be a justified emendation (Article 33.2.3.1); the emended spelling avoids homonymy with *Ryparus* Spinola, 1844 [Coleoptera: Cleridae] (see A. B. T. Smith 2006: 159)]. Comment: the earliest known publication date of this work is 31 December 1910 (see Evenhuis, 1994: 55); although Rhyparina A. Schmidt (1910b: 91) was published on 30 September 1910, we use A. Schmidt, 1910a as the correct original work since “Rhyparina Schmidt 1910: 130” was used as the correct citation for this name by A. Schmidt himself (1910b: 91).

### Tribe Stereomerini

Howden and Storey, 1992

Stereomerini Howden and Storey, 1992: 1811 [stem: *Stereomer-*]. Type genus: *Stereomera* Arrow, 1905.

### Tribe Termitoderini

Tangelder and Krikken, 1982

Termitoderini Tangelder and Krikken, 1982: 10, in key [stem: *Termitoder-*]. Type genus: *Termitoderus* Mateu, 1966.

### Subfamily Aulonocneminae

Janssens, 1946

Aulonocneminae Janssens, 1946: 7, in key [stem: *Aulonocnem-*]. Type genus: *Aulonocnemis* Klug, 1837. Comment: current spelling maintained (Art. 29.3.1.1): incorrect stem formation in prevailing usage (should be *Aulonocnemid*-).

### Subfamily Termitotroginae

Wasmann, 1918

Termitotrogini Wasmann, 1918: 4 [stem: *Termitotrog-*]. Type genus: *Termitotrox* Reichensperger, 1915.

### Subfamily Scarabaeinae

Latreille, 1802

Scarabaeïdes Latreille, 1802: 144 [stem: *Scarabae-*]. Type genus: *Scarabaeus* Linnaeus, 1758.

### Tribe Ateuchini

Perty, 1830

Ateuchidae Perty, 1830: 38 [stem: *Ateuch-*]. Type genus: *Ateuchus* Weber, 1801.

### Subtribe Ateuchina

Perty, 1830

Ateuchidae Perty, 1830: 38 [stem: *Ateuch-*]. Type genus: *Ateuchus* Weber, 1801. Comment: name previously attributed to Laporte (1840b: 63); use of subtribes follow Vaz-de-Mello (2008).
Choerididae Harold, 1867: 9 [stem: *Choeridi-*]. Type genus: *Choeridium* Lepeletier and Audinet-Serville, 1828.
Pinotinae Kolbe, 1905: 548 [stem: *Pinot-*]. Type genus: *Pinotus* Erichson, 1847.
Dichotomiini Pereira, 1954: 55 [stem: *Dichotomi-*]. Type genus: *Dichotomius* Hope, 1838.

### Subtribe Scatimina

Vaz-de-Mello, 2008

Scatimina Vaz-de-Mello, 2008: 10 [stem: *Scatim-*]. Type genus: *Scatimus* Erichson, 1847.

### Ateuchini incertae sedis

Demarziellini Balthasar, 1961: 178 [stem: *Demarziell-*]. Type genus: *Demarziella* Balthasar, 1961. Comment: family-group taxon not originally described but available (Art. 13.5) (see Matthews and Stebnicka 1986); current placement follows Vaz-de-Mello (2008).

### Tribe Coprini

Leach, 1815

*Coprides Baudet-Lafarge, 1809: 44 [stem: *Copr-*]. Type genus: *Copris* Geoffroy, 1762 [placed on the Official List of Generic Names in Zoology (ICZN 1994a)]. Comment: original vernacular name unavailable (Art. 11.7.2): subsequently used in latinized form but not generally attributed to Baudet-Lafarge (1809).
Coprides Leach, 1815: 96 [stem: *Copr-*]. Type genus: *Copris* Geoffroy, 1762 [placed on the Official List of Generic Names in Zoology (ICZN 1994a)]. Comment: current spelling maintained (Art. 29.3.1.1): incorrect stem formation in prevailing usage (should be *Coprid*-).
Coptodactylini Paulian, 1933: 67 [stem: *Coptodactyl-*]. Type genus: *Coptodactyla* H. C. C. Burmeister, 1846.

### Tribe Deltochilini

Lacordaire, 1856

Deltochilides Lacordaire, 1856: 78 [stem: *Deltochil-*]. Type genus: *Deltochilum* Eschscholtz, 1822. Comment: original vernacular name available (Art. 11.7.2): first used in latinized form by J. L. LeConte (1861: 125, as Deltochila), generally accepted as in Germain (1903: 354, as Deltochilidae); First Reviser (Deltochilini Lacordaire, 1856 vs Mentophilini Lacordaire, 1856 vs Scatonomini Lacordaire, 1856) not determined, current usage maintained.
Minthophilides Lacordaire, 1856: 80 [stem: *Mentophil-*]. Type genus: *Mentophilus* Laporte, 1840 [as *Minthophilus*, both *Mentophilus* (p. 74) and *Minthophilus* (p. 63) were used in the original publication by Laporte (1840b), although *Mentophilus* has been used as the correct spelling in recent literature, we could not determine the First Reviser]. Comment: original vernacular name available (Art. 11.7.2): first used in latinized form and generally accepted as in Gerstaecker (1861: 464, as Minthophilidae); name treated as unavailable by A. B. T. Smith (2006: 160); incorrect original stem formation, not in prevailing usage.
Scatonomides Lacordaire, 1856: 87 [stem: *Scatonom-*]. Type genus: *Scatonomus* Erichson, 1835. Comment: original vernacular name available (Art. 11.7.2): first used in latinized form and generally accepted as in J. L. LeConte (1861: 125, as Scatonomi); transferred from Ateuchini by Vaz-de-Mello (2008).
Coprobiadae H. C. C. Burmeister, 1873: 407 [stem: *Coprobi-*]. Type genus: *Coprobius* Latreille, 1829.
Canthonides van Lansberge, 1875a: 184 [stem: *Canthon-*]. Type genus: *Canthon* Hoffmannsegg, 1817. Comment: original vernacular name available (Art. 11.7.2): first used in latinized form by Kolbe (1905: 530, as Canthoninae), generally accepted as in A. B. T. Smith (2006: 160, as Canthonini).
Epilissides van Lansberge, 1875a: 188 [stem: *Epiliss-*]. Type genus: *Epilissus* Dejean, 1836. Comment: original vernacular name available (Art. 11.7.2): first used in latinized form and generally accepted as in Shipp (1894: 255, as Epilissini).
Epirinides van Lansberge, 1875a: 189 [stem: *Epirin-*]. Type genus: *Epirinus* Dejean, 1833. Comment: original vernacular name available (Art. 11.7.2): first used in latinized form and generally accepted as in Bertkau (1875: 335, as Epirini); name treated as unavailable by A. B. T. Smith (2006: 160).
Panelini Arrow, 1931: 404 [stem: *Panel-*]. Type genus: *Panelus* Lewis, 1895.

### Tribe Eucraniini

Burmeister, 1873

Eucraniadae H. C. C. Burmeister, 1873: 405 [stem: *Eucrani-*]. Type genus: *Eucranium* Brullé, 1837.
Ennearabdini Pereira and Martínez, 1956: 238 [stem: *Ennearabd-*]. Type genus: *Ennearabdus* van Lansberge, 1874.

### Tribe Gymnopleurini

Lacordaire, 1856

Gymnopleurides Lacordaire, 1856: 72 [stem: *Gymnopleur-*]. Type genus: *Gymnopleurus* Illiger, 1803. Comment: original vernacular name available (Art. 11.7.2): first used in latinized form by J. L. LeConte (1861: 124, as Gymnopleuri), generally accepted as in A. B. T. Smith (2006: 160, as Gymnopleurini).

### Tribe Oniticellini

Kolbe, 1905

Oniticellini Kolbe, 1905: 547 [stem: *Oniticell-*]. Type genus: *Oniticellus* Dejean, 1821. Comment: usage of this name over Drepanocerini conserved (Art. 35.5) (see A. B. T. Smith 2006).

### Subtribe Drepanocerina

van Lansberge, 1875

Drèpanocérides van Lansberge, 1875b: 14 [stem: *Drepanocer-*]. Type genus: *Drepanocerus* Kirby, 1828. Comment: original vernacular name available (Art. 11.7.2): first used in latinized form by Kolbe (1905: 531, as Drepanocerini), generally accepted as in A. B. T. Smith (2006: 161, as Drepanocerini); although Drepanocerini has priority over Oniticellini, the latter is in prevailing usage at the tribal level and must not be displaced by the older name (Art. 35.5) (see A. B. T. Smith 2006).

### Subtribe Eurysternina

Vulcano, Martínez and Pereira, 1961

Eurysternini Vulcano et al., 1961: 268 [stem: *Eurystern-*]. Type genus: *Eurysternus* Dalman, 1824. Comment: current placement based on Génier (2009).

### Subtribe Helictopleurina

Janssens, 1946

Helictopleurides Janssens, 1946: 11, in key [stem: *Helictopleur-*]. Type genus: *Helictopleurus* d’Orbigny, 1915.

### Subtribe Oniticellina

Kolbe, 1905

Oniticellini Kolbe, 1905: 547 [stem: *Oniticell-*]. Type genus: *Oniticellus* Dejean, 1821.

### Tribe Onitini

Laporte, 1840

Onitides Laporte, 1840b: 88 [stem: *Onit-*]. Type genus: *Onitis* Fabricius, 1798. Comment: original vernacular name available (Art. 11.7.2): first used in latinized form by Shipp (1894: 255, as Onitidae), generally accepted as in A. B. T. Smith (2006: 161, as Onitini); although Fabricius originally treated the type genus name as masculine, it appears in Greek dictionaries as a feminine noun (with stem *Onitid*-); we recommend that an application be submitted to the Commission to establish the correct gender of *Onitis* and the correct stem based on that genus.

### Tribe Onthophagini

Burmeister, 1846

Onthophagidae H. C. C. Burmeister, 1846: [1] [stem: *Onthophag-*]. Type genus: *Onthophagus* Latreille, 1802.
Alloscelides Janssens, 1946: 10, in key [stem: *Alloscel-*]. Type genus: *Alloscelus* Boucomont, 1923.

### Tribe Phanaeini

Hope, 1838

Phanaeidae Hope, 1838a: 321 [stem: *Phanae-*]. Type genus: *Phanaeus* W. S. MacLeay, 1819.
Gromphina Zunino, 1985a: 22 [stem: *Gromphad-*]. Type genus: *Gromphas* Brullé, 1837. Comment: published 20 November 1985; this family-group name was also used in the same year by Zunino (1985b [20 December]: 107, as Gromphina); incorrect original stem formation, not in prevailing usage.

### Tribe Scarabaeini

Latreille, 1802

Scarabaeïdes Latreille, 1802: 144 [stem: *Scarabae-*]. Type genus: *Scarabaeus* Linnaeus, 1758.
Pachysomides Ferreira, 1953: 5, in key [stem: *Pachysomat-*]. Type genus: *Pachysoma* W. S. MacLeay, 1821. Comment: incorrect original stem formation, not in prevailing usage.
Actinophorini Ádám, 2003: 130 [stem: *Actinophor-*]. Type genus: *Actinophorus* Creutzer, 1799 [syn. of *Scarabaeus* Linnaeus, 1758].

### Tribe Sisyphini

Mulsant, 1842

Sisyphaires Mulsant, 1842: 41 [stem: *Sisyph-*]. Type genus: *Sisyphus* Latreille, 1807 [the original spelling of the type genus is *Sisyphe*, however, the incorrect subsequent spelling *Sisyphus* is in prevailing usage and should be considered the correct original spelling (Art. 33.3.1) (see A. B. T. Smith 2006)]. Comment: original vernacular name available (Art. 11.7.2): first used in latinized form by Shipp (1894: 255, as Sisyphinae), generally accepted as in A. B. T. Smith (2006: 162, as Sisyphini).

### †Subfamily Prototroginae

Nikolajev, 2000

Prototroginae Nikolajev, 2000a: 63 [stem: *Prototrog-*]. Type genus: *Prototrox* Nikolajev, 2000.

### †Subfamily Cretoscarabaeinae

Nikolajev, 1995

Cretoscarabaeinae Nikolajev, 1995b: 147 [stem: *Cretoscarabae-*]. Type genus: *Cretoscarabaeus* Nikolajev, 1995.

### Subfamily Dynamopodinae

Arrow, 1911

Dynamopinae Arrow, 1911: 611 [stem: *Dynamopod-*]. Type genus: *Dynamopus* Semenov, 1895.

### Tribe Dynamopodini

Arrow, 1911

Dynamopinae Arrow, 1911: 611 [stem: *Dynamopod-*]. Type genus: *Dynamopus* Semenov, 1895. Comment: incorrect original stem formation, not in prevailing usage; correction of stem by Lawrence and Newton (1995).

### Tribe Thinorycterini

Semenov and A. N. Reichardt, 1925

Thinorycterina Semenov and A. N. Reichardt, 1925: 86 [stem: *Thinorycter-*]. Type genus: *Thinorycter* Semenov and A. N. Reichardt, 1925.

### Subfamily Phaenomeridinae

Erichson, 1847

Phaenomerini Erichson, 1847b: 655 [stem: *Phaenomerid-*]. Type genus: *Phaenomeris* Hope, 1833 [placed on the Official List of Generic Names in Zoology (ICZN 1962)]. Comment: stem emended from *Phaenomer*- to *Phaenomerid*- thus removing it from homonymy with another family-group name in Curculionidae and placed on the Official List of Family-Group Names in Zoology (ICZN 1962, as Phaenomerididae Ohaus, 1913); in this ruling (ICZN 1962) ‘‘Ohaus, 1913’’ was erroneously given as the original author of this family-group name (see A. B. T. Smith 2006).

### Subfamily Orphninae

Erichson, 1847

Orphnidae Erichson, 1847a: 111 [stem: *Orphn-*]. Type genus: *Orphnus* W. S. MacLeay, 1819.

### Tribe Aegidiini

Paulian, 1984

Aegidiinae Paulian, 1984: 68 [stem: *Aegidi-*]. Type genus: *Aegidium* Westwood, 1845.

### Tribe Orphnini

Erichson, 1847

Orphnidae Erichson, 1847a: 111 [stem: *Orphn-*]. Type genus: *Orphnus* W. S. MacLeay, 1819.
Hybalidae Marseul, 1857a: 83 [stem: *Hybal-*]. Type genus: *Hybalus* Dejean, 1833. Comment: name previously attributed to Jacquelin du Val (1859: 31, as Hybalites) and treated as unavailable by A. B. T. Smith (2006: 163).

### Subfamily Allidiostomatinae

Arrow, 1940

Idiostominae Arrow, 1904: 747 [stem: *Idiostomat-*]. Type genus: *Idiostoma* Arrow, 1904 [preoccupied genus name, not *Idiostoma* Walsingham, 1882 [Lepidoptera]; syn. of *Allidiostoma* Arrow, 1940]. Comment: permanently invalid (Art. 39): based on preoccupied type genus; incorrect original stem formation, not in prevailing usage.
Allidiostomidae Arrow, 1940: 16 [stem: *Allidiostomat-*]. Type genus: *Allidiostoma* Arrow, 1940. Comment: replacement name for Idiostominae Arrow, 1904 because of the homonymy of the type genus; incorrect original stem formation, not in prevailing usage; correction of stem by Lawrence and Newton (1995).

### Subfamily Aclopinae

Blanchard, 1850

Aclopitae Blanchard, 1850: 96 [stem: *Aclop-*]. Type genus: *Aclopus* Erichson, 1835.

### Tribe Aclopini

Blanchard, 1850

Aclopitae Blanchard, 1850: 96 [stem: *Aclop-*]. Type genus: *Aclopus* Erichson, 1835.

### †Tribe Holcorobeini

Nikolajev, 1992

Holcorobeini Nikolajev, 1992: 81 [stem: *Holcorobe-*]. Type genus: *Holcorobeus* Nikritin, 1977.

### Tribe Phaenognathini

Iablokoff-Khnzorian, 1977

Phaenognathini Iablokoff-Khnzorian, 1977: 137, 172 [stem: *Phaenognath-*]. Type genus: *Phaenognatha* Hope, 1842.

### Subfamily Melolonthinae

Leach, 1819

Melolonthidae Leach, 1819: 189 [stem: *Melolonth-*]. Type genus: *Melolontha* Fabricius, 1775 [placed on the Official List of Generic Names in Zoology (ICZN 1994a)].

### Tribe Ablaberini

Blanchard, 1850

Ablaberitae Blanchard, 1850: 100 [stem: *Ablaber-*]. Type genus: *Ablabera* Dejean, 1833.
Camentini Machatschke, 1959: 738 [stem: *Cament-*]. Type genus: *Camenta* Erichson, 1847.

### Tribe Automoliini

Britton, 1978

Caulobiina H. C. C. Burmeister, 1855: 204 [stem: *Caulobi-*]. Type genus: *Caulobius* Le Guillou, 1844 [preoccupied genus name, not *Caulobius* Duponchel, 1838 [Lepidoptera]; syn. of *Deuterocaulobius* Dalla Torre, 1912]. Comment: permanently invalid (Art. 39): based on preoccupied type genus.
Automolini Britton, 1957: 72 [stem: *Automol-*]. Type genus: *Automolus* H. C. C. Burmeister, 1855 [preoccupied genus name, not *Automolus* Reichenbach, 1853 [Aves]; syn. of *Automolius* Britton, 1978]. Comment: permanently invalid (Art. 39): based on preoccupied type genus.
Automoliini Britton, 1978: 7 [stem: *Automoli-*]. Type genus: *Automolius* Britton, 1978. Comment: replacement name for Automolini Britton, 1957 because of the homonymy of the type genus.

### Tribe Chasmatopterini

Lacordaire, 1856

Chasmatoptérides Lacordaire, 1856: 220 [stem: *Chasmatopter-*]. Type genus: *Chasmatopterus* Dejean, 1821. Comment: original vernacular name available (Art. 11.7.2): first used in latinized form by H. W. Bates (1887: 130, as Chasmatopteridae), generally accepted as in A. B. T. Smith (2006: 164, as Chasmatopterini).

### Tribe Colymbomorphini

Blanchard, 1850

Colymbomorphitae Blanchard, 1850: 97 [stem: *Colymbomorph-*]. Type genus: *Colymbomorpha* Blanchard, 1850.
Stethaspididae H. C. C. Burmeister, 1855: 218 [stem: *Stethaspid-*]. Type genus: *Stethaspis* Hope, 1837 [placed on the Official List of Generic Names in Zoology (ICZN 1983b)]. Comment: A. B. T. Smith (2006) used “Stethaspini” as the valid name for this tribe, however, the fact that the genus *Colymbomorpha* is considered to be in this tribe, e.g., Houston and Weir (1992), brings the name Colymbomorphini into synonymy with Stethaspini and Xylonichini; Colymbomorphini has nomenclatural priority and should be considered the valid name for this tribe.
Xylonychini Britton, 1957: 9 [stem: *Xylonich-*]. Type genus: *Xylonichus* Boisduval, 1835 [as *Xylonychus*, incorrect subsequent spelling of type genus name, not in prevailing usage (see A. B. T. Smith 2006: 166)]. Comment: incorrect original stem formation, not in prevailing usage.

### Tribe Comophorinini

Britton, 1957

Comophorini Britton, 1957: 10 [stem: *Comophorin-*]. Type genus: *Comophorina* Strand, 1928. Comment: incorrect original stem formation, not in prevailing usage.

### †Tribe Cretomelolonthini

Nikolajev, 1998

Cretomelolonthini Nikolajev, 1998: 80 [stem: *Cretomelolonth-*]. Type genus: *Cretomelolontha* Nikolajev, 1998.

### Tribe Dichelonychini

Burmeister, 1855

Dichelonychidae H. C. C. Burmeister, 1855: 70 [stem: *Dichelonych-*]. Type genus: *Dichelonyx* Harris, 1827.

### Tribe Diphucephalini

Laporte, 1840

Diphucéphalites Laporte, 1840b: 145 [stem: *Diphucephal-*]. Type genus: *Diphucephala* Dejean, 1821. Comment: original vernacular name available (Art. 11.7.2): first used in latinized form by Agassiz (1846b: 125, as Diphycocephaloidae [incorrect stem formation]), generally accepted as in A. B. T. Smith (2006: 165, as Diphucephalini).

### Tribe Diphycerini

Medvedev, 1952

Diphycerini S. I. Medvedev, 1952: 186 [stem: *Diphycer-*]. Type genus: *Diphycerus* Deyrolle and Fairmaire, 1878.

### Tribe Diplotaxini

Kirby, 1837

Diplotaxidae Kirby, 1837: 129 [stem: *Diplotax-*]. Type genus: *Diplotaxis* Kirby, 1837. Comment: current spelling maintained (Art. 29.5): incorrect stem formation in prevailing usage (should be *Diplotaxe*-).
Apogoniitae Blanchard, 1851a: 228 [stem: *Apogoni-*]. Type genus: *Apogonia* Kirby, 1819.
Liogenitae Blanchard, 1851a: 166 [stem: *Liogeny-*]. Type genus: *Liogenys* Guérin-Méneville, 1831. Comment: incorrect original stem formation, not in prevailing usage.

### Tribe Euchirini

Hope, 1840

Eucheiridae Hope, 1840b: 300 [stem: *Euchir-*]. Type genus: *Euchirus* Kirby, 1828 [as *Eucheirus*, incorrect subsequent spelling of type genus name, not in prevailing usage]. Comment: incorrect original stem formation, not in prevailing usage.

### Tribe Heteronychini

Lacordaire, 1856

Hétéronycides Lacordaire, 1856: 225 [stem: *Heteronych-*]. Type genus: *Heteronyx* Guérin-Méneville, 1831. Comment: original vernacular name available (Art. 11.7.2): first used in latinized form by Bertkau (1890: 277, as Heteronychini), generally accepted as in A. B. T. Smith (2006: 167, as Heteronychini); incorrect original stem formation, not in prevailing usage.

### Tribe Hopliini

Latreille, 1829

Hoplides Latreille, 1829a: 563 [stem: *Hopli-*]. Type genus: *Hoplia* Illiger, 1803.

### Subtribe Hopliina

Latreille, 1829

Hoplides Latreille, 1829a: 563 [stem: *Hopli-*]. Type genus: *Hoplia* Illiger, 1803. Comment: incorrect original stem formation, not in prevailing usage.
Dicheliden Oken, 1843: 483 [stem: *Dichel-*]. Type genus: *Dichelus* Lepeletier and Audinet-Serville, 1828. Comment: original vernacular name available (Art. 11.7.2): first used in latinized form and generally accepted as in Tulk (1847: 614, as Dichelidae).
Gymnolomidae H. C. C. Burmeister, 1844: 138 [stem: *Gymnolomat-*]. Type genus: *Gymnoloma* Dejean, 1833. Comment: incorrect original stem formation, not in prevailing usage.
Heterochelidae H. C. C. Burmeister, 1844: 86 [stem: *Heterochel-*]. Type genus: *Heterochelus* H. C. C. Burmeister, 1844.
Lepisiidae H. C. C. Burmeister, 1844: 166 [stem: *Lepisi-*]. Type genus: *Lepisia* Lepeletier and Audinet-Serville, 1828.
Scelophysides Péringuey, 1902: 624 [stem: *Scelophys-*]. Type genus: *Scelophysa* H. C. C. Burmeister, 1844.
*Madahopliini M. Lacroix, 1997: 21 [stem: *Madahopli-*]. Type genus: *Madahoplia* Lacroix, 1998. Comment: unavailable family-group name, not based on available genus name at the time (see A. B. T. Smith 2006).

### Subtribe Pachycnemina

Laporte, 1840

Pachycnémides Laporte, 1840b: 155 [stem: *Pachycnem-*]. Type genus: *Pachycnema* Lepeletier and Audinet-Serville, 1828. Comment: original vernacular name available (Art. 11.7.2): first used in latinized form by Burmeister (1844: 53, as Pachycnemidae), generally accepted as in A. B. T. Smith (2006: 170, as Pachycnemina).
Lepitrichiden Oken, 1843: 483 [stem: *Lepitrich-*]. Type genus: *Lepitrix* Lepeletier and Audinet-Serville, 1828. Comment: original vernacular name available (Art. 11.7.2): first used in latinized form and generally accepted as in Tulk (1847: 614, as Lepitrichidae).
Anisonychidae H. C. C. Burmeister, 1844: 35 [stem: *Anisonych-*]. Type genus: *Anisonyx* Latreille, 1807. Comment: Lucas (1920: 3) listed “Anisochelidae H. C. C. Burmeister” in error for “Anisonychidae H. C. C. Burmeister”; this name is a senior homonym of Anisonychini Legalov, 2003 (type genus *Anisonychus* Voss, 1927) in Attelabidae; this case is to be referred to the Commission to remove the homonymy (Art. 55.3.1).

### Tribe Lichniini

Burmeister, 1844

Lichniadae H. C. C. Burmeister, 1844: 8 [stem: *Lichni-*]. Type genus: *Lichnia* Erichson, 1835.

### Tribe Liparetrini

Burmeister, 1855

Liparetridae H. C. C. Burmeister, 1855: 187 [stem: *Liparetr-*]. Type genus: *Liparetrus* Guérin-Méneville, 1831.
Haplonychidae H. C. C. Burmeister, 1855: 224 [stem: *Haplonych-*]. Type genus: *Haplonycha* Dejean, 1836 [unjustified emendation of *Aplonycha* by Agassiz (1846b: 29), in prevailing usage and so deemed to be a justified emendation (Article 33.2.3.1) (see A. B. T. Smith 2006: 166); syn. of *Colpochila* Erichson, 1843]. Comment: this name is a senior homonym of Haplonychini Lacordaire, 1865 (type genus *Haplonyx* Schönherr, 1836) proposed in Curculionidae and now a synonym of Cryptoplini Lacordaire, 1863; this case is to be referred to the Commission to remove the homonymy (Art. 55.3.1).
Allarini Britton, 1955: 125 [stem: *Allar-*]. Type genus: *Allara* Britton, 1955.
Colpochilini Britton, 1957: 10 [stem: *Colpochil-*]. Type genus: *Colpochila* Erichson, 1843.

### Tribe Macrodactylini

Kirby, 1837

Macrodactylidae Kirby, 1837: 133 [stem: *Macrodactyl-*]. Type genus: *Macrodactylus* Dejean, 1821.
Ceraspididae H. C. C. Burmeister, 1855: 91 [stem: *Ceraspid-*]. Type genus: *Ceraspis* Lepeletier and Audinet-Serville, 1828.
Dicraniadae H. C. C. Burmeister, 1855: 65 [stem: *Dicrani-*]. Type genus: *Dicrania* Lepeletier and Audinet-Serville, 1828.
Isonychidae H. C. C. Burmeister, 1855: 22 [stem: *Isonych-*]. Type genus: *Isonychus* Mannerheim, 1829.
Microcraniadae H. C. C. Burmeister, 1855: 75 [stem: *Microcrani-*]. Type genus: *Microcrania* H. C. C. Burmeister, 1855.
Plectridae H. C. C. Burmeister, 1855: 80 [stem: *Plectr-*]. Type genus: *Plectris* Lepeletier and Audinet-Serville, 1828.
*Philochlénides Lacordaire, 1856: 256 [stem: *Philochloeni-*]. Type genus: *Philochloenia* Dejean, 1833 [as *Philochlaenia*, incorrect subsequent spelling of type genus name, not in prevailing usage]. Comment: original vernacular name unavailable (Art. 11.7.2): not subsequently latinized; incorrect original stem formation, not in prevailing usage.
Clavipalpides Lacordaire, 1856: 267 [stem: *Clavipalp-*]. Type genus: *Clavipalpus* Laporte, 1832. Comment: published before 29 March 1856; original vernacular name available (Art. 11.7.2): first used in latinized form by Imhoff (1856 [before 25 December]: xi, as Clavipalpidae), generally accepted as in G. H. Horn (1880e: 147, as Clavipalpides [treated as Latin]).

### Tribe Maechidiini

Burmeister, 1855

Maechidiina H. C. C. Burmeister, 1855: 208 [stem: *Maechidi-*]. Type genus: *Maechidius* W. S. MacLeay, 1819.

### Tribe Melolonthini

Leach, 1819

Melolonthidae Leach, 1819: 189 [stem: *Melolonth-*]. Type genus: *Melolontha* Fabricius, 1775 [placed on the Official List of Generic Names in Zoology (ICZN 1994a)].

### Subtribe Enariina

Dewailly, 1950

Enarina Dewailly, 1950: 323 [stem: *Enari-*]. Type genus: *Enaria* Erichson, 1847. Comment: incorrect original stem formation, not in prevailing usage.

### Subtribe Heptophyllina

Medvedev, 1951

Heptophyllini S. I. Medvedev, 1951: 197 [stem: *Heptophyll-*]. Type genus: *Heptophylla* Motschulsky, 1858.

### Subtribe Leucopholina

Burmeister, 1855

Leucopholidae H. C. C. Burmeister, 1855: 285 [stem: *Leucophol-*]. Type genus: *Leucopholis* Dejean, 1833. Comment: current spelling maintained (Art. 29.3.1.1): incorrect stem formation in prevailing usage (should be *Leucopholid*-).

### Subtribe Melolonthina

Leach, 1819

*Mélolonthides Baudet-Lafarge, 1809: 12 [stem: *Melolonth-*]. Type genus: *Melolontha* Fabricius, 1775 [placed on the Official List of Generic Names in Zoology (ICZN 1994a)]. Comment: original vernacular name unavailable (Art. 11.7.2): subsequently used in latinized form but not generally attributed to Baudet-Lafarge (1809).
Melolonthidae Leach, 1819: 189 [stem: *Melolonth-*]. Type genus: *Melolontha* Fabricius, 1775 [placed on the Official List of Generic Names in Zoology (ICZN 1994a)]. Comment: published in June 1819; this family-group name was also used in the same year by W. S. MacLeay (1819 [November]: 79, as Melolonthidae).
Polyphyllidae H. C. C. Burmeister, 1855: 397 [stem: *Polyphyll-*]. Type genus: *Polyphylla* Harris, 1841.
Psilonychides Péringuey, 1904: 184 [stem: *Psilonych-*]. Type genus: *Psilonychus* H. C. C. Burmeister, 1855.

### Subtribe Pegylina

Lacroix, 1989

Pegylini M. Lacroix, 1989: 115 [stem: *Pegyl-*]. Type genus: *Pegylis* Erichson, 1847. Comment: current spelling maintained (Art. 29.3.1.1): incorrect stem formation in prevailing usage (should be *Pegylid*-).

### Subtribe Rhizotrogina

Burmeister, 1855

Rhizotrogidae H. C. C. Burmeister, 1855: 308 [stem: *Rhizotrog-*]. Type genus: *Rhizotrogus* Latreille, 1825.
Tostegopterae J. L. LeConte, 1861: 139 [stem: *Tostegopter-*]. Type genus: *Tostegoptera* Blanchard, 1851.

### Subtribe Schizonychina

Burmeister, 1855

Schizonychidae H. C. C. Burmeister, 1855: 265 [stem: *Schizonych-*]. Type genus: *Schizonycha* Dejean, 1833.

### Tribe Oncerini

LeConte, 1861

Oncerini J. L. LeConte, 1861: 133 [stem: *Oncer-*]. Type genus: *Oncerus* J. L. LeConte, 1856.

### Tribe Pachypodini

Erichson, 1840

Pachypoden Erichson, 1840b: 29 [stem: *Pachypod-*]. Type genus: *Pachypus* Dejean, 1821 [*nomen protectum*; this genus name is a junior homonym of *Pachypus* Billberg, 1820 *nomen oblitum*; we provide references to support the conservation of *Pachypus* Dejean, 1821 as the valid name for this genus (Art. 23.9.1) (see Appendix 1)]. Comment: original vernacular name available (Art. 11.7.2): first used in latinized form by Erichson (1847a: 100, as Pachypoda), generally accepted as in A. B. T. Smith (2006: 164, as Pachypodini).

### Tribe Pachytrichini

Burmeister, 1855

Pachytrichiadae H. C. C. Burmeister, 1855: 241 [stem: *Pachytrich-*]. Type genus: *Pachytricha* Hope, 1841.

### Tribe Phyllotocidiini

Britton, 1957

Phyllotocidiini Britton, 1957: 58 [stem: *Phyllotocidi-*]. Type genus: *Phyllotocidium* Blackburn, 1898.

### Tribe Podolasiini

Howden, 1997

Lasiopodes J. L. LeConte, 1856: 282 [stem: *Lasiopod-*]. Type genus: *Lasiopus* J. L. LeConte, 1856 [preoccupied genus name, not *Lasiopus* Schönherr, 1823 [Coleoptera: Curculionidae], not *Lasiopus* Geoffroy, 1835 [Mammalia]; syn. of *Podolasia* Harold, 1869]. Comment: permanently invalid (Art. 39): based on preoccupied type genus.
Podolasiini Howden, 1997: 224 [stem: *Podolasi-*]. Type genus: *Podolasia* Harold, 1869.

### Tribe Scitalini

Britton, 1957

Scitalini Britton, 1957: 10 [stem: *Scital-*]. Type genus: *Scitala* Erichson, 1842.

### Tribe Sericini

Kirby, 1837

Sericidae Kirby, 1837: 128 [stem: *Seric-*]. Type genus: *Serica* W. S. MacLeay, 1819.

### Subtribe Phyllotocina

Burmeister, 1855

Phyllotocidae H. C. C. Burmeister, 1855: 182 [stem: *Phyllotoc-*]. Type genus: *Phyllotocus* Fisher von Waldheim, 1823.

### Subtribe Sericina

Kirby, 1837

Sericidae Kirby, 1837: 128 [stem: *Seric-*]. Type genus: *Serica* W. S. MacLeay, 1819. Comment: published 23 October 1837; this family-group name was also proposed in the same year by Hope (1837 [“31 December”]: 73, 107, as Sericidae); Hope (1840a: 112) refers to the publication by Kirby (1837) which is further evidence that the publication by Kirby was published first (see A. B. T. Smith 2006: 165).
Omalopliites Blanchard, 1845a: 212 [stem: *Omalopli-*]. Type genus: *Omaloplia* Schönherr, 1817. Comment: original vernacular name available (Art. 11.7.2): first used in latinized form by Gistel (1848: [5], as Omalopliaeidae [incorrect stem formation]), generally accepted as in Blanchard (1853: 129, as Omalopliitae); Omalopliites Blanchard, 1845 treated as unavailable and the first available name based on this type genus listed as Homalopliina H. C. C. Burmeister, 1855 by A. B. T. Smith (2006: 165).
Astaenidae H. C. C. Burmeister, 1855: 123 [stem: *Astaen-*]. Type genus: *Astaena* Erichson, 1847.

### Subtribe Trochalina

Brenske, 1898

Trochalinae Brenske, 1898: 354 [stem: *Trochal-*]. Type genus: *Trochalus* Laporte, 1832.

### Tribe Sericoidini

Erichson, 1847

Sericoideae Erichson, 1847a: 102 [stem: *Sericoid-*]. Type genus: *Sericoides* Guérin-Méneville, 1840.

### Tribe Systellopini

Sharp, 1877

Systellopides Sharp, 1877: 311 [stem: *Systellop-*]. Type genus: *Systellopus* Sharp, 1877. Comment: current spelling maintained (Art. 29.5): incorrect stem formation in prevailing usage (should be *Systellopod*-).

### Tribe Tanyproctini

Erichson, 1847

Tanyproctini Erichson, 1847b: 653 [stem: *Tanyproct-*]. Type genus: *Tanyproctus* Ménétriés, 1832.

### Subtribe Macrophyllina

Burmeister, 1855

Macrophyllidae H. C. C. Burmeister, 1855: 447 [stem: *Macrophyll-*]. Type genus: *Macrophylla* Hope, 1837 [syn. of *Aegostheta* Dejean, 1833]. Comment: senior homonym of Macrophyllina Gray, 1866 (type genus *Macrophyllum* Gray, 1838) in Mammalia; this case is to be referred to the Commission to remove the homonymy (Art. 55.3.1).
Aegosthetini Lacroix, 2007: 203 [stem: *Aegosthet-*]. Type genus: *Aegostheta* Dejean, 1833.

### Subtribe Tanyproctina

Erichson, 1847

Tanyproctini Erichson, 1847b: 653 [stem: *Tanyproct-*]. Type genus: *Tanyproctus* Ménétriés, 1832.
Elaphoceritae Blanchard, 1851a: 164 [stem: *Elaphocer-*]. Type genus: *Elaphocera* Géné, 1836. Comment: Elaphocerini no longer has priority over Pachydemini through a reversal of precedence (A. B. T. Smith 2006: 168).
Pachydemidae H. C. C. Burmeister, 1855: 437 [stem: *Pachydem-*]. Type genus: *Pachydema* Laporte, 1832. Comment: name conserved over Elaphocerini Blanchard, 1851 as a *nomen protectum* and treated as valid by A. B. T. Smith (2006: 168).
Achloidae H. C. C. Burmeister, 1855: 465 [stem: *Achlo-*]. Type genus: *Achloa* Erichson, 1840.
Cephalotrichiadae H. C. C. Burmeister, 1855: 433 [stem: *Cephalotrichi-*]. Type genus: *Cephalotrichia* Hope, 1837.
*Leptopodidae H. C. C. Burmeister, 1855: 428 [stem: *Leptopod-*]. Type genus: *Leptopus* Waltl, 1838 [preoccupied genus name, not *Leptopus* Latreille, 1809 [Hemiptera], not *Leptopus* Rafinesque, 1814 [Pisces], not *Leptopus* Lamarck, 1818 [Crustacea], and not *Leptopus* Fallén, 1823 [Diptera]; syn. of *Elaphocera* Gené, 1836]. Comment: family-group name unavailable (Art. 11.7.1.1): not based on a genus used as valid at the time.
Sparrmannini Péringuey, 1904: 170 [stem: *Sparrmanni-*]. Type genus: *Sparrmannia* Laporte, 1840 [*nomen protectum*; this genus name is a junior synonym of *Leocaeta* Dejean, 1833 *nomen oblitum* and *Cephalotrichia* Hope, 1837 *nomen oblitum*; we provide references to support the conservation of *Sparrmannia* as the valid name for this genus (Art. 23.9.1) (see Appendix 1); *Sparrmannia* is an incorrect subsequent spelling of *Sparmannia* Laporte, 1840, in prevailing usage and so deemed to be the correct original spelling (see Evans 1989)]. Comment: incorrect original stem formation, not in prevailing usage.

### Subfamily Rutelinae

MacLeay, 1819

Rutelidae W. S. MacLeay, 1819: 69 [stem: *Rutel-*]. Type genus: *Rutela* Latreille, 1802. Comment: First Reviser (Rutelinae MacLeay, 1819 vs Anoplognathinae MacLeay, 1819) not determined, current usage maintained.

### Tribe Adoretini

Burmeister, 1844

Adoretidae H. C. C. Burmeister, 1844: 466 [stem: *Adoret-*]. Type genus: *Adoretus* Dejean, 1833.

### Subtribe Adoretina

Burmeister, 1844

Adoretidae H. C. C. Burmeister, 1844: 466 [stem: *Adoret-*]. Type genus: *Adoretus* Dejean, 1833.
Adorodociina Ohaus, 1912: 151 [stem: *Adorodoci-*]. Type genus: *Adorodocia* Brenske, 1893.
Adoroleptina Ohaus, 1912: 151 [stem: *Adorolept-*]. Type genus: *Adoroleptus* Brenske, 1893.
Pseudadoretina Ohaus, 1912: 151 [stem: *Pseudadoret-*]. Type genus: *Pseudadoretus* Semenov, 1889.
Scaphorhinadoretina Ohaus, 1912: 151 [stem: *Scaphorhinadoret-*]. Type genus: *Scaphorhinadoretus* Ohaus, 1912. Comment: erroneously cited as a new genus originally but correctly listed as a subtribe on p. 426 of the same work.

### Subtribe Adorrhinyptiina

Arrow, 1917

Adorrhinyptiini Arrow, 1917: 273 [stem: *Adorrhinypti-*]. Type genus: *Adorrhinyptia* Arrow, 1917.

### Subtribe Pachyrhinadoretina

Ohaus, 1912

Pachyrhinadoretina Ohaus, 1912: 151 [stem: *Pachyrhinadoret-*]. Type genus: *Pachyrhinadoretus* Ohaus, 1912.

### Subtribe Prodoretina

Ohaus, 1912

Prodoretina Ohaus, 1912: 151 [stem: *Prodoret-*]. Type genus: *Prodoretus* Brenske, 1893.

### Subtribe Trigonostomusina

Ohaus, 1912

Trigonostomina Ohaus, 1912: 151 [stem: *Trigonostomus-*]. Type genus: *Trigonostomus* Brenske, 1893 [placed on the Official List of Generic Names in Zoology (ICZN 2009b)]. Comment: *Trigonostomus*- determined to be the correct stem of this name to avoid homonymy with Trigonostomidae Graff, 1905 (type genus *Trigonostomum* Schmidt, 1852) in Platyhelminthes and Trigonostomusina Ohaus, 1912 placed on the Official List of Family-group Names in Zoology (ICZN 2009b).

### Tribe Alvarengiini

Frey, 1975

*Pachylides Lacordaire, 1856: 394 [stem: *Pachyl-*]. Type genus: *Pachylus* H. C. C. Burmeister, 1847 [preoccupied genus name, not *Pachylus* C. L. Koch, 1839 [Arachnida]; syn. of *Ottokelleria* d’Andretta and Martínez, 1957]. Comment: original vernacular name unavailable (Art. 11.7.2): not subsequently latinized; if discovered to be available then permanently invalid (Art. 39): based on preoccupied type genus; Pachylinae Sørensen, 1884 (type genus *Pachylus* C. L. Koch, 1839) is used as valid in Arachnida.
Alvarengiini Frey, 1975: 84 [stem: *Alvarengi-*]. Type genus: *Alvarengius* Frey, 1975.

### Tribe Anatistini

Lacordaire, 1856

Anatistides Lacordaire, 1856: 321 [stem: *Anatist-*]. Type genus: *Anatista* Brême, 1844. Comment: published before 29 March 1856; original vernacular name available (Art. 11.7.2): first used in latinized form and generally accepted as in Imhoff (1856 [before 25 December]: xi, as Anatistidae).
Spodochlamydini Ohaus, 1918: 166 [stem: *Spodochlamyd-*]. Type genus: *Spodochlamys* H. C. C. Burmeister, 1855.

### Tribe Anomalini

Streubel, 1839 nomen protectum

Anomalidae Streubel, 1839: 136 [stem: *Anomal-*]. Type genus: *Anomala* Samouelle, 1819 [placed on the Official List of Generic Names in Zoology (ICZN 1989d); *Anomala* von Block, 1799 was suppressed for the purposes of the Principle of Priority and the Principle of Homonymy (ICZN 1989d)]. Comment: *nomen protectum* (see A. B. T. Smith 2006: 173).

### Subtribe Anisopliina

Burmeister, 1844

Anisopliadae H. C. C. Burmeister, 1844: 208 [stem: *Anisopli-*]. Type genus: *Anisoplia* Schönherr, 1817. Comment: incorrect original stem formation, not in prevailing usage.

### Subtribe Anomalina

Streubel, 1839 nomen protectum

Euchloridae Hope, 1839: 67 [stem: *Euchlor-*]. Type genus: *Euchlora* W. S. MacLeay, 1819. Comment: *nomen oblitum* (see A. B. T. Smith 2006: 173).
Anomalidae Streubel, 1839: 136 [stem: *Anomal-*]. Type genus: *Anomala* Samouelle, 1819 [placed on the Official List of Generic Names in Zoology (ICZN 1989d); *Anomala* von Block, 1799 was suppressed for the purposes of the Principle of Priority and the Principle of Homonymy (ICZN 1989d)]. Comment: *nomen protectum* (see A. B. T. Smith 2006: 173).
Phyllurgaeidae Gistel, 1848: [5] [stem: *Phyllurg-*]. Type genus: *Phyllurga* Gistel, 1848 [syn. of *Euchlora* W. S. MacLeay, 1819].
Dilophochilina Ohaus, 1918: 166 [stem: *Dilophochil-*]. Type genus: *Dilophochila* H. W. Bates, 1888.

### Subtribe Isopliina

Péringuey, 1902

Isopliini Péringuey, 1902: 564 [stem: *Isopli-*]. Type genus: *Isoplia* H. C. C. Burmeister, 1855.

### Subtribe Leptohopliina

Potts, 1974

Lepothopliini Potts, 1974: 152 [stem: *Leptohopli-*]. Type genus: *Leptohoplia* Saylor, 1935. Comment: incorrect original stem formation, not in prevailing usage.

### Subtribe Popilliina

Ohaus, 1918

Popilliina Ohaus, 1918: 133 [stem: *Popilli-*]. Type genus: *Popillia* Dejean, 1821.

### Tribe Anoplognathini

MacLeay, 1819

Anoplognathidae W. S. MacLeay, 1819: 81 [stem: *Anoplognath-*]. Type genus: *Anoplognathus* Leach, 1815.

### Subtribe Anoplognathina

MacLeay, 1819

Anoplognathidae W. S. MacLeay, 1819: 81 [stem: *Anoplognath-*]. Type genus: *Anoplognathus* Leach, 1815.

### Subtribe Brachysternina

Burmeister, 1844

Brachysternidae H. C. C. Burmeister, 1844: 455 [stem: *Brachystern-*]. Type genus: *Brachysternus* Guérin-Méneville, 1831.

### Subtribe Phalangogoniina

Ohaus, 1918

Phalangogoniina Ohaus, 1918: 176 [stem: *Phalangogoni-*]. Type genus: *Phalangogonia* H. C. C. Burmeister, 1844.

### Subtribe Platycoeliina

Burmeister, 1844

Platycoeliidae H. C. C. Burmeister, 1844: 451 [stem: *Platycoeli-*]. Type genus: *Platycoelia* Dejean, 1833.

### Subtribe Schizognathina

Ohaus, 1918

Schizognathina Ohaus, 1918: 174 [stem: *Schizognath-*]. Type genus: *Schizognathus* Fischer von Waldheim, 1823.

### Tribe Geniatini

Burmeister, 1844

Geniatidae H. C. C. Burmeister, 1844: 478 [stem: *Geniat-*]. Type genus: *Geniates* Kirby, 1819. Comment: First Reviser (Geniatini H. C. C. Burmeister, 1844 vs Leucothyreini H. C. C. Burmeister, 1844) not determined, current usage maintained.
Leucothyreidae H. C. C. Burmeister, 1844: 485 [stem: *Leucothyre-*]. Type genus: *Leucothyreus* W. S. MacLeay, 1819.

### Tribe Rutelini

MacLeay, 1819

Rutelidae W. S. MacLeay, 1819: 69 [stem: *Rutel-*]. Type genus: *Rutela* Latreille, 1802.

### Subtribe Areodina

Burmeister, 1844

Areodidae H. C. C. Burmeister, 1844: 423 [stem: *Areod-*]. Type genus: *Areoda* W. S. MacLeay, 1819.

### Subtribe Desmonychina

Arrow, 1917

Desmonychinae Arrow, 1917: 359 [stem: *Desmonych-*]. Type genus: *Desmonyx* Arrow, 1907. Comment: spelled Desmonycinae pp. xiii, 359, 382, but spelled correctly Desmonychinae p. 3 [in key].

### Subtribe Didrepanephorina

Ohaus, 1918

Didrepanophorina Ohaus, 1918: 14 [stem: *Didrepanephor-*]. Type genus: *Didrepanephorus* Wood-Mason, 1878 [as *Didrepanophorus*, incorrect subsequent spelling of type genus name, not in prevailing usage]. Comment: incorrect original stem formation, not in prevailing usage.

### Subtribe Heterosternina

Bates, 1888 nomen protectum

Macropni G. H. Horn, 1867b: 398 [stem: *Macropn-*]. Type genus: *Macropnus* G. H. Horn, 1867. Comment: *nomen oblitum* (see A. B. T. Smith 2006: 171).
Heterosterninae H. W. Bates, 1888: 286 [stem: *Heterostern-*]. Type genus: *Heterosternus* Dupont, 1832. Comment: *nomen protectum* (see A. B. T. Smith 2006: 171).

### Subtribe Lasiocalina

Ohaus, 1918

Lasiocalina Ohaus, 1918: 30 [stem: *Lasiocal-*]. Type genus: *Lasiocala* Blanchard, 1851.

### Subtribe Oryctomorphina

Burmeister, 1847

Oryctomorphidae H. C. C. Burmeister, 1847: 28 [stem: *Oryctomorph-*]. Type genus: *Oryctomorphus* Guérin-Méneville, 1831.

### Subtribe Parastasiina

Burmeister, 1844

Parastasiidae H. C. C. Burmeister, 1844: 368 [stem: *Parastasi-*]. Type genus: *Parastasia* Westwood, 1841.

### Subtribe Rutelina

MacLeay, 1819

Rutelidae W. S. MacLeay, 1819: 69 [stem: *Rutel-*]. Type genus: *Rutela* Latreille, 1802.
Chasmodiidae H. C. C. Burmeister, 1844: 333 [stem: *Chasmodi-*]. Type genus: *Chasmodia* W. S. MacLeay, 1819.
Chrysophoridae H. C. C. Burmeister, 1844: 412 [stem: *Chrysophor-*]. Type genus: *Chrysophora* Dejean, 1821.
Macraspididae H. C. C. Burmeister, 1844: 343 [stem: *Macraspid-*]. Type genus: *Macraspis* W. S. MacLeay, 1819.
Pelidnotidae H. C. C. Burmeister, 1844: 388 [stem: *Pelidnot-*]. Type genus: *Pelidnota* W. S. MacLeay, 1819 [placed on the Official List of Generic Names in Zoology (ICZN 2003b)].
Antichirides Lacordaire, 1856: 341 [stem: *Anticheir-*]. Type genus: *Anticheira* Eschscholtz, 1818 [as *Antichira*, unjustified emendation of type genus name by Agassiz (1846b: 27), not in prevailing usage]. Comment: original vernacular name available (Art. 11.7.2): first used in latinized form and generally accepted as in H. W. Bates (1888: 262, as Antichirina); incorrect original stem formation, not in prevailing usage.
Plusiotina H. W. Bates, 1888: 276 [stem: *Plusiotid-*]. Type genus: *Plusiotis* H. C. C. Burmeister, 1844. Comment: incorrect original stem formation, not in prevailing usage.
Fruhstorferiina Ohaus, 1918: 43 [stem: *Fruhstorferi-*]. Type genus: *Fruhstorferia* Kolbe, 1894.

### Subfamily Dynastinae

MacLeay, 1819

Dynastidae W. S. MacLeay, 1819: 64 [stem: *Dynast-*]. Type genus: *Dynastes* W. S. MacLeay, 1819.

### Tribe Agaocephalini

Burmeister, 1847

Agaocephalidae H. C. C. Burmeister, 1847: 280 [stem: *Agaocephal-*]. Type genus: *Agaocephala* Lepeletier and Audinet-Serville, 1828 [as *Agacephala*, incorrect subsequent spelling for *Agacephala*, in prevailing usage and so deemed to be the correct original spelling (Art. 33.3.1) (see A. B. T. Smith 2006)].

### Tribe Cyclocephalini

Laporte, 1840

Cyclocephalites Laporte, 1840b: 124 [stem: *Cyclocephal-*]. Type genus: *Cyclocephala* Dejean, 1821. Comment: original vernacular name available (Art. 11.7.2): first used in latinized form by Imhoff (1856: xi, as Cyclocephalidae), generally accepted as in A. B. T. Smith (2006: 175, as Cyclocephalini).
Chalepidae H. C. C. Burmeister, 1847: 71 [stem: *Chalep-*]. Type genus: *Chalepus* W. S. MacLeay, 1819 [preoccupied genus name, not *Chalepus* Thunberg, 1805 [Coleoptera: Chrysomelidae]; syn. of *Dyscinetus* Harold, 1869]. Comment: permanently invalid (Art. 39): based on preoccupied type genus; this family-group name is a senior homonym of Chalepini Weise, 1910 (type genus *Chalepus* Thunberg, 1805) currently used as valid in Chrysomelidae.
Peltonotini Arrow, 1917: 27 [stem: *Peltonot-*]. Type genus: *Peltonotus* H. C. C. Burmeister, 1847.
Acrobolbiina Ohaus, 1918: 13 [stem: *Acrobolbi-*]. Type genus: *Acrobolbia* Ohaus, 1912.

### Tribe Dynastini

MacLeay, 1819

Dynastidae W. S. MacLeay, 1819: 64 [stem: *Dynast-*]. Type genus: *Dynastes* W. S. MacLeay, 1819.
Xylotrupidae Hope, 1838b: 319 [stem: *Xylotrup-*]. Type genus: *Xylotrupes* Hope, 1837.
Megasominae Swainson, 1840: 210 [stem: *Megasomat-*]. Type genus: *Megasoma* Kirby, 1825. Comment: family-group name attributed to Imhoff (1856) in A. B. T. Smith (2006: 175); incorrect original stem formation, not in prevailing usage.

### Tribe Hexodontini

Lacordaire, 1856

Hexodontides Lacordaire, 1856: 391 [stem: *Hexodont-*]. Type genus: *Hexodon* A. G. Olivier, 1789. Comment: published before 29 March 1856; original vernacular name available (Art. 11.7.2): first used in latinized form by Imhoff (1856 [before 25 December]: xi, as Hexodontidae), generally accepted as in Arrow (1937: 4, as Hexodontini).

### Tribe Oryctini

Mulsant, 1842

Oryctésaires Mulsant, 1842: 372 [stem: *Oryct-*]. Type genus: *Oryctes* Illiger, 1798. Comment: original vernacular name available (Art. 11.7.2): first used in latinized form by MacDonald (1845: 185, as Oryctesiae [incorrect stem formation]), generally accepted as in A. B. T. Smith (2006: 175, as Oryctini); incorrect original stem formation, not in prevailing usage.
Megaceridae H. C. C. Burmeister, 1847: 212 [stem: *Megacerat-*]. Type genus: *Megaceras* Hope, 1837. Comment: incorrect original stem formation, not in prevailing usage.
Strategidae H. C. C. Burmeister, 1847: 87 [stem: *Strateg-*]. Type genus: *Strategus* Kirby, 1828.

### Tribe Oryctoderini

Endrödi, 1966

Oryctoderini Endrödi, 1966: 25 [stem: *Oryctoder-*]. Type genus: *Oryctoderus* Boisduval, 1835.

### Tribe Pentodontini

Mulsant, 1842

Pentodonaires Mulsant, 1842: 381 [stem: *Pentodont-*]. Type genus: *Pentodon* Hope, 1837 [placed on the Official List of Generic Names in Zoology (ICZN 2003b)].

### Subtribe Cheiroplatina

Carne, 1957

Cheiroplatina Carne, 1957: 61 [stem: *Cheiroplat-*]. Type genus: *Cheiroplatys* Hope, 1837. Comment: Casey (1915a) cites “Cheiroplatids” several times within the Pentodontini without formally erecting a subtribe; current spelling maintained (Art. 29.5): incorrect stem formation in prevailing usage (should be *Cheiroplate*-).

### Subtribe Dipelicina

Carne, 1957

Dipelicina Carne, 1957: 117 [stem: *Dipelic-*]. Type genus: *Dipelicus* Hope, 1843.

### Subtribe Pentodontina

Mulsant, 1842

Pentodonaires Mulsant, 1842: 381 [stem: *Pentodont-*]. Type genus: *Pentodon* Hope, 1837 [placed on the Official List of Generic Names in Zoology (ICZN 2003b)]. Comment: original vernacular name available (Art. 11.7.2): first used in latinized form by H. W. Bates (1888: 314, as Pentodontinae), generally accepted as in A. B. T. Smith (2006: 176, as Pentodontina); incorrect original stem formation, not in prevailing usage.
Calicnémiens Mulsant, 1842: 386 [stem: *Calicnemid-*]. Type genus: *Calicnemis* Laporte, 1832. Comment: original vernacular name available (Art. 11.7.2): first used in latinized form by MacDonald (1845: 185, as Callicnemiae [incorrect stem formation]) and generally accepted as in Acloque (1896: 267, as Calicnemisii) and Houlbert (1922b: 225, as Callicnemini [incorrect stem formation]); name attributed to Blanchard (1845a) and treated as unavailable by A. B. T. Smith (2006: 168); incorrect original stem formation, not in prevailing usage.
Bothynidae H. C. C. Burmeister, 1847: 90 [stem: *Bothyn-*]. Type genus: *Bothynus* Hope, 1837 [placed on the Official List of Generic Names in Zoology (ICZN 2008a)].
Pimelopodea H. C. C. Burmeister, 1847: 172 [stem: *Pimelopod-*]. Type genus: *Pimelopus* Erichson, 1842.
Podalgidae H. C. C. Burmeister, 1847: 90 [stem: *Podalg-*]. Type genus: *Podalgus* H. C. C. Burmeister, 1847 [placed on the Official List of Generic Names in Zoology (ICZN 2004a)].
Metanastina Carne, 1957: 32 [stem: *Metanast-*]. Type genus: *Metanastes* Arrow, 1911.

### Subtribe Pseudoryctina

Carne, 1957

Pseudoryctina Carne, 1957: 121 [stem: *Pseudoryct-*]. Type genus: *Pseudoryctes* Sharp, 1873.

### Tribe Phileurini

Burmeister, 1847

Phileuridae H. C. C. Burmeister, 1847: 138 [stem: *Phileur-*]. Type genus: *Phileurus* Latreille, 1807. Comment: usage of this name conserved over Cryptodini H. C. C. Burmeister and Schaum, 1840 (Art. 35.5).

### Subtribe Cryptodina

Burmeister and Schaum, 1840

Cryptodinae H. C. C. Burmeister and Schaum, 1840: 360 [stem: *Cryptod-*]. Type genus: *Cryptodus* MacLeay, 1819. Comment: current spelling maintained (Art. 29.5): incorrect stem formation in prevailing usage (should be *Cryptodont*-); conservation of original stem avoids homonymy problems with Cryptodontidae Dall, 1895 (type genus *Cryptodon* Turton, 1822) in Mollusca: Bivalvia and Cryptodontina Lacordaire, 1856 (type genus *Cryptodontes* H. C. C. Burmeister, 1847) in Scarabaeidae: Cetoniinae: Trichiini.

### Subtribe Phileurina

Burmeister, 1847

Phileuridae H. C. C. Burmeister, 1847: 138 [stem: *Phileur-*]. Type genus: *Phileurus* Latreille, 1807.

### Subfamily Cetoniinae

Leach, 1815

Cetonida Leach, 1815: 99 [stem: *Cetoni-*]. Type genus: *Cetonia* Fabricius, 1775. Comment: A. B. T. Smith (2006: 177) noted that although Cetoniinae has priority over Melolonthinae, the latter is in prevailing usage at the family level and must not be displaced by the older name under Article 35.5 (only relevant in cases where authors consider Melolonthidae to be a family containing the subfamily Cetoniinae).

### Tribe Cetoniini

Leach, 1815

Cetonida Leach, 1815: 99 [stem: *Cetoni-*]. Type genus: *Cetonia* Fabricius, 1775.

### Subtribe Cetoniina

Leach, 1815

Cetonida Leach, 1815: 99 [stem: *Cetoni-*]. Type genus: *Cetonia* Fabricius, 1775.
Elaphini Schoch, 1894: 175 [stem: *Elaphin-*]. Type genus: *Elaphinis* H. C. C. Burmeister, 1842.
Glycyphanae Schoch, 1894: 175 [stem: *Glycyphan-*]. Type genus: *Glycyphana* H. C. C. Burmeister, 1842.
Pachnodii Péringuey, 1907: 371 [stem: *Pachnod-*]. Type genus: *Pachnoda* H. C. C. Burmeister, 1842.
Tephraeides Schenkling, 1921: 313 [stem: *Tephrae-*]. Type genus: *Tephraea* H. C. C. Burmeister, 1842.

### Subtribe Euphoriina

Horn, 1880

Euphoriae G. H. Horn, 1880c: 397 [stem: *Euphori-*]. Type genus: *Euphoria* H. C. C. Burmeister, 1842. Comment: name attributed to Schoch (1894) by A. B. T. Smith (2006: 182).

### Subtribe Leucocelina

Kraatz, 1882

Leucoceliden Kraatz, 1882b: 65 [stem: *Leucocel-*]. Type genus: *Leucocelis* H. C. C. Burmeister, 1842. Comment: original vernacular name available (Art. 11.7.2): first used in latinized form by Schoch (1894: 213, as Leucocelidae), generally accepted as in A. B. T. Smith (2006: 182, as Leucocelina); current spelling maintained (Art. 29.3.1.1): incorrect stem formation in prevailing usage (should be *Leucocelid*-).

### Tribe Cremastocheilini

Burmeister and Schaum, 1841

Cremastochilidae H. C. C. Burmeister and Schaum, 1841: 243 [stem: *Cremastocheil-*]. Type genus: *Cremastocheilus* Knoch, 1801. Comment: usage of this name conserved over Macromini H. C. C. Burmeister and Schaum, 1840 (Art. 35.5).

### Subtribe Aspilina

Krikken, 1984

Aspilina Krikken, 1984: 25, in key [stem: *Aspil-*]. Type genus: *Aspilus* Westwood in Schaum, 1848.

### Subtribe Coenochilina

Burmeister, 1842

Coenochilidae H. C. C. Burmeister, 1842: 148 [stem: *Coenochil-*]. Type genus: *Coenochilus* Schaum, 1841.

### Subtribe Cremastocheilina

Burmeister and Schaum, 1841

Cremastochilidae H. C. C. Burmeister and Schaum, 1841: 243 [stem: *Cremastocheil-*]. Type genus: *Cremastocheilus* Knoch, 1801. Comment: incorrect original stem formation, not in prevailing usage.

### Subtribe Cymophorina

Krikken, 1984

Cymophorina Krikken, 1984: 23, in key [stem: *Cymophor-*]. Type genus: *Cymophorus* Kirby, 1827.

### Subtribe Genuchina

Krikken, 1984

Genuchina Krikken, 1984: 21, in key [stem: *Genuch-*]. Type genus: *Genuchus* Kirby, 1825.

### Subtribe Goliathopsidina

Krikken, 1984

Goliathopsidina Krikken, 1984: 25, in key [stem: *Goliathopsid-*]. Type genus: *Goliathopsis* Janson, 1881. Comment: current spelling maintained (Art. 29.5): incorrect stem formation in prevailing usage (should be *Goliathopse*-).

### Subtribe Heterogeniina

Krikken, 1984

Heterogeniina Krikken, 1984: 25, in key [stem: *Heterogeni-*]. Type genus: *Heterogenius* Moser, 1911.

### Subtribe Lissogeniina

Krikken, 1984

Lissogeniina Krikken, 1984: 25, in key [stem: *Lissogeni-*]. Type genus: *Lissogenius* Schaum, 1845.

### Subtribe Macromina

Burmeister and Schaum, 1840

Macrominae H. C. C. Burmeister and Schaum, 1840: 360 [stem: *Macrom-*]. Type genus: *Macroma* Gory and Percheron, 1833 [syn. of *Campsiura* Hope, 1831]. Comment: current spelling maintained (Art. 29.5): incorrect stem formation in prevailing usage (should be *Macromat*-).

### Subtribe Nyassinina

Krikken, 1984

Nyassinina Krikken, 1984: 25, in key [stem: *Nyassin-*]. Type genus: *Nyassinus* Westwood, 1879.

### Subtribe Oplostomina

Krikken, 1984

Oplostomina Krikken, 1984: 23, in key [stem: *Oplostom-*]. Type genus: *Oplostomus* W. S. MacLeay, 1838.

### Subtribe Pilinurgina

Krikken, 1984

Pilinurgina Krikken, 1984: 25, in key [stem: *Pilinurg-*]. Type genus: *Pilinurgus* H. C. C. Burmeister, 1842.

### Subtribe Spilophorina

Krikken, 1984

Spilophorina Krikken, 1984: 25, in key [stem: *Spilophor-*]. Type genus: *Spilophorus* Westwood in Schaum, 1848. Comment: this family-group name is a junior homonym of Spilophorini Chapuis, 1875 (type genus *Spilophora* Boheman, 1850) currently used as valid in Chrysomelidae; this case is to be referred to the Commission to remove the homonymy (Art. 55.3.1).

### Subtribe Telochilina

Krikken, 1984

Telochilina Krikken, 1984: 21, in key [stem: *Telochil-*]. Type genus: *Telochilus* Krikken, 1975.

### Subtribe Trichoplina

Krikken, 1984

Trichoplina Krikken, 1984: 23, in key [stem: *Trichopl-*]. Type genus: *Trichoplus* H. C. C. Burmeister, 1842.

### Subtribe Trogodina

Krikken, 1984

Trogodina Krikken, 1984: 27, in key [stem: *Trogod-*]. Type genus: *Trogodes* Westwood, 1874.

### Tribe Diplognathini

Burmeister, 1842

Diplognathidae H. C. C. Burmeister, 1842: 617 [stem: *Diplognath-*]. Type genus: *Diplognatha* Gory and Percheron, 1833.
Porphyronotii Péringuey, 1907: 371 [stem: *Porphyronot-*]. Type genus: *Porphyronota* H. C. C. Burmeister, 1842.

### Tribe Goliathini

Latreille, 1829

Goliathides Latreille, 1829a: 571 [stem: *Goliath-*]. Type genus: *Goliathus* Lamarck, 1801. Comment: name attributed to Griffith and Pidgeon (1832: 492) in A. B. T. Smith (2006: 181).

### Subtribe Coryphocerina

Burmeister, 1842

Coryphoceridae H. C. C. Burmeister, 1842: 215 [stem: *Coryphocer-*]. Type genus: *Coryphocera* H. C. C. Burmeister, 1842.
Heterorrhinidae Kraatz, 1880a: 21 [stem: *Heterorhin-*]. Type genus: *Heterorhina* Westwood, 1842 [as *Heterorrhina*, incorrect subsequent spelling of type genus name, not in prevailing usage]. Comment: incorrect original stem formation, not in prevailing usage.
Ceratorrhinidae Kraatz, 1880a: 18 [stem: *Ceratorhin-*]. Type genus: *Ceratorhina* Westwood, 1843 [as *Ceratorrhina*, incorrect subsequent spelling of type genus name, not in prevailing usage]. Comment: incorrect original stem formation, not in prevailing usage.
Bothrorrhinae Schoch, 1894: 173 [stem: *Bothrorrhin-*]. Type genus: *Bothrorrhina* H. C. C. Burmeister, 1842.
Gnathoceridae Schoch, 1894: 170 [stem: *Gnathocer-*]. Type genus: *Gnathocera* Kirby, 1825.
Ischnosceli Schoch, 1894: 170 [stem: *Ischnoscelid-*]. Type genus: *Ischnoscelis* H. C. C. Burmeister, 1842. Comment: incorrect original stem formation, not in prevailing usage.
Rhomborrhinae Schoch, 1894: 171 [stem: *Rhomborhin-*]. Type genus: *Rhomborhina* Hope, 1837 [as *Rhomborrhina*, incorrect subsequent spelling of type genus name, not in prevailing usage]. Comment: incorrect original stem formation, not in prevailing usage.
Tmesorrhinae Schoch, 1894: 170 [stem: *Tmesorrhin-*]. Type genus: *Tmesorrhina* Westwood, 1842.
Coelorrhinae Schoch, 1895: iii [stem: *Coelorrhin-*]. Type genus: *Coelorrhina* Hope, 1841 [*Coelorrhina* is an incorrect subsequent spelling for *Caelorrhina* in prevailing usage, and so deemed to be the correct original spelling (Article 33.3.1) (see A. B. T. Smith 2006: 181)].
Mecynorrhinina Schenkling, 1921: 15 [stem: *Mecynorhin-*]. Type genus: *Mecynorhina* Hope, 1837 [as *Mecynorrhina*, incorrect subsequent spelling of type genus name, not in prevailing usage]. Comment: incorrect original stem formation, not in prevailing usage.
Stephanorrhinina Schenkling, 1921: 35 [stem: *Stephanorrhin-*]. Type genus: *Stephanorrhina* H. C. C. Burmeister, 1842.

### Subtribe Dicronocephalina

Krikken, 1984

Dicronocephalina Krikken, 1984: 37, in key [stem: *Dicronocephal-*]. Type genus: *Dicronocephalus* Hope, 1831 [*Dicronocephalus* is an incorrect subsequent spelling for *Dicranocephalus*, since the incorrect subsequent spelling is in prevailing usage, it is now considered the correct original spelling of the name under Article 33.3.1; the original spelling placed the name in homonymy with *Dicranocephalus* Hahn, 1826 (Hemiptera) but usage of the subsequent spelling as the correct spelling avoids this homonymy problem (see A. B. T. Smith 2006)].

### Subtribe Goliathina

Latreille, 1829

Goliathides Latreille, 1829a: 571 [stem: *Goliath-*]. Type genus: *Goliathus* Lamarck, 1801. Comment: name attributed to Griffith and Pidgeon (1832) in A. B. T. Smith (2006: 181).
Hypselogeniae Schoch, 1894: 169 [stem: *Hypselogeni-*]. Type genus: *Hypselogenia* H. C. C. Burmeister, 1840.

### Subtribe Ichnestomatina

Burmeister, 1842

Ischnostomidae H. C. C. Burmeister, 1842: 600 [stem: *Ichnestomat-*]. Type genus: *Ichnestoma* Gory and Percheron, 1833 [as *Ichnostoma*, incorrect subsequent spelling of type genus name, not in prevailing usage]. Comment: incorrect original stem formation, not in prevailing usage.

### Tribe Gymnetini

Kirby, 1827

Gymnetidae Kirby, 1827: 150 [stem: *Gymnet-*]. Type genus: *Gymnetis* W. S. MacLeay, 1819 [placed on the Official List of Generic Names in Zoology (ICZN 1967a)].

### Subtribe Blaesiina

Schoch, 1895

Blaesiae Schoch, 1895: iii [stem: *Blaesi-*]. Type genus: *Blaesia* H. C. C. Burmeister, 1842.

### Subtribe Gymnetina

Kirby, 1827

Gymnetidae Kirby, 1827: 150 [stem: *Gymnet-*]. Type genus: *Gymnetis* W. S. MacLeay, 1819. Comment: current spelling maintained (Art. 29.3.1.1): incorrect stem formation in prevailing usage (should be *Gymnetid*-).
Clinteriidae Kraatz, 1882a: 49 [stem: *Clinteri-*]. Type genus: *Clinteria* H. C. C. Burmeister, 1842.
Stethodesmae Schoch, 1894: 172 [stem: *Stethodesmat-*]. Type genus: *Stethodesma* Bainbridge, 1841. Comment: incorrect original stem formation, not in prevailing usage.

### Tribe Phaedimini

Schoch, 1894

Phaedimi Schoch, 1894: 169 [stem: *Phaedim-*]. Type genus: *Phaedimus* Westwood, 1841.

### Tribe Schizorhinini

Burmeister, 1842

Schizorrhinidae H. C. C. Burmeister, 1842: 530 [stem: *Schizorhin-*]. Type genus: *Schizorhina* Kirby, 1825. Comment: First Reviser (Lomapterini H. C. C. Burmeister, 1842 vs Macronotini H. C. C. Burmeister, 1842 vs Schizorrhinini H. C. C. Burmeister, 1842) not determined, current usage maintained.

### Subtribe Lomapterina

Burmeister, 1842

Lomapteridae H. C. C. Burmeister, 1842: 310 [stem: *Lomapter-*]. Type genus: *Lomaptera* Gory and Percheron, 1833. Comment: First Reviser (Lomapterina H. C. C. Burmeister, 1842 vs Macronotina H. C. C. Burmeister, 1842) not determined, current usage maintained.
Macronotidae H. C. C. Burmeister, 1842: 318 [stem: *Macronot-*]. Type genus: *Macronota* Hoffmannsegg, 1817.

### Subtribe Schizorhinina

Burmeister, 1842

Schizorrhinidae H. C. C. Burmeister, 1842: 530 [stem: *Schizorhin-*]. Type genus: *Schizorhina* Kirby, 1825 [as *Schizorrhina*, incorrect subsequent spelling of type genus name, not in prevailing usage]. Comment: incorrect original stem formation, not in prevailing usage.
Diaphoniadae Kraatz, 1880b: 195 [stem: *Diaphoni-*]. Type genus: *Diaphonia* Newman, 1840.
Eupoecilidae Kraatz, 1880b: 188 [stem: *Eupoecil-*]. Type genus: *Eupoecila* H. C. C. Burmeister, 1842.
Hemipharidae Kraatz, 1880b: 182 [stem: *Hemiphar-*]. Type genus: *Hemipharis* H. C. C. Burmeister, 1842.

### Tribe Stenotarsiini

Kraatz, 1880

Stenotarsiden Kraatz, 1880b: 182 [stem: *Stenotarsi-*]. Type genus: *Stenotarsia* H. C. C. Burmeister, 1842.

### Subtribe Anochiliina

Krikken, 1984

*Anochiliens Pouillaude, 1917: 54 [stem: *Anochili-*]. Type genus: *Anochilia* H. C. C. Burmeister, 1842. Comment: original vernacular name unavailable (Art. 11.7.2): proposed after 1899.
Anochiliina Krikken, 1984: 31, in key [stem: *Anochili-*]. Type genus: *Anochilia* H. C. C. Burmeister, 1842.

### Subtribe Coptomiina

Schenkling, 1921

Coptomiini Schenkling, 1921: 147 [stem: *Coptomi-*]. Type genus: *Coptomia* H. C. C. Burmeister, 1842.

### Subtribe Chromoptiliina

Krikken, 1984

Chromoptiliina Krikken, 1984: 31, in key [stem: *Chromoptili-*]. Type genus: *Chromoptilia* Westwood, 1842.

### Subtribe Doryscelina

Schenkling, 1921

*Dorysceliens Pouillaude, 1917: 53, in key [stem: *Doryscel-*]. Type genus: *Doryscelis* Dejean, 1836. Comment: original vernacular name unavailable (Art. 11.7.2): proposed after 1899.
Doryscelina Schenkling, 1921: 148 [stem: *Doryscel-*]. Type genus: *Doryscelis* Dejean, 1836. Comment: name attributed to Krikken (1984) by A. B. T. Smith (2006: 180); current spelling maintained (Art. 29.3.1.1): incorrect stem formation in prevailing usage (should be *Doryscelid*-).

### Subtribe Euchroeina

Paulian and Descarpentries, 1982

*Euchroeens Pouillaude, 1917: 64 [stem: *Euchroe-*]. Type genus: *Euchroea* H. C. C. Burmeister, 1842. Comment: original vernacular name unavailable (Art. 11.7.2): proposed after 1899; the author also used the incorrect spelling *Euchaeeus* in the key on page 54.
Euchroeina Paulian and Descarpentries, 1982: 5 [stem: *Euchroe-*]. Type genus: *Euchroea* H. C. C. Burmeister, 1842. Comment: the name Euchroeidae Dahlbom, 1854 (type genus *Euchroeus* Latreille, 1809) is available in Hymenoptera; this case is to be referred to the Commission to remove the homonymy (Art. 55.3.1).

### Subtribe Heterophanina

Schoch, 1894

Heterophanae Schoch, 1894: 173 [stem: *Heterophan-*]. Type genus: *Heterophana* H. C. C. Burmeister, 1842.

### Subtribe Heterosomatina

Krikken, 1984

Heterosomatina Krikken, 1984: 29, in key [stem: *Heterosomat-*]. Type genus: *Heterosoma* Schaum, 1845.

### Subtribe Pantoliina

Krikken, 1984

*Pantoliens Pouillaude, 1917: 54 [stem: *Pantoli-*]. Type genus: *Pantolia* H. C. C. Burmeister, 1842. Comment: original vernacular name unavailable (Art. 11.7.2): proposed after 1899.
Pantoliina Krikken, 1984: 33, in key [stem: *Pantoli-*]. Type genus: *Pantolia* H. C. C. Burmeister, 1842.

### Subtribe Parachiliina

Krikken, 1984

Parachiliina Krikken, 1984: 33, in key [stem: *Parachili-*]. Type genus: *Parachilia* H. C. C. Burmeister, 1842.

### Subtribe Stenotarsiina

Kraatz, 1880

Stenotarsiden Kraatz, 1880b: 182 [stem: *Stenotarsi-*]. Type genus: *Stenotarsia* H. C. C. Burmeister, 1842. Comment: original vernacular name available (Art. 11.7.2): first used in latinized form by Jakobson (1915: 961, as Stenotarsina [incorrect stem formation]), generally accepted as in A. B. T. Smith (2006: 179, as Stenotarsiini); incorrect original stem formation, not in prevailing usage.

### Tribe Taenioderini

Mikšić, 1976

Taenioderina Mikšić, 1976: 29 [stem: *Taenioder-*]. Type genus: *Taeniodera* H. C. C. Burmeister, 1842. Comment: First Reviser found (Taenioderini Mikšić, 1976 vs Chalcotheini Mikšić, 1976) is Krikken (1984: 63).

### Subtribe Chalcotheina

Mikšić, 1976

Chalcotheina Mikšić, 1976: 357 [stem: *Chalcothe-*]. Type genus: *Chalcothea* H. C. C. Burmeister, 1842.

### Subtribe Taenioderina

Mikšić, 1976

Taenioderina Mikšić, 1976: 29 [stem: *Taenioder-*]. Type genus: *Taeniodera* H. C. C. Burmeister, 1842.

### Tribe Trichiini

Fleming, 1821

Trichiadae Fleming, 1821: 50 [stem: *Trichi-*]. Type genus: *Trichius* Fabricius, 1775 [placed on the Official List of Generic Names in Zoology (ICZN 2004c)]. Comment: Trichiinae Lozek, 1956 (type genus *Trichia* Hartmann, 1840) proposed in Mollusca: Gastropoda is a junior homonym of this family-group name; Trichiinae Lozek, 1956 was recently placed on the Official index of Rejected and Invalid Family-Group Names in Zoology (ICZN 2004c).

### Subtribe Cryptodontina

Lacordaire, 1856

Cryptodontides Lacordaire, 1856: 462 [stem: *Cryptodont-*]. Type genus: *Cryptodontes* H. C. C. Burmeister, 1847. Comment: published before 29 March 1856; original vernacular name available (Art. 11.7.2): first used in latinized form by Imhoff (1856 [before 25 December]: xii, as Cryptodontidae), generally accepted as in A. B. T. Smith (2006: 177, as Cryptodontina); the name Cryptodontidae Dall, 1895 (type genus *Cryptodon* Turton, 1822) is available in Mollusca: Bivalvia while the name Cryptodontidae Owen, 1860 used in Therapsida is unavailable because it is not based on an available genus-group name; this case is to be referred to the Commission to remove the homonymy (Art. 55.3.1).

### Subtribe Incina

Burmeister, 1842

Incadae H. C. C. Burmeister, 1842: 704 [stem: *Inc-*]. Type genus: *Inca* Lepeletier and Audinet-Serville, 1828.

### Subtribe Osmodermatina

Schenkling, 1922

Osmodermini Schenkling, 1922: 3 [stem: *Osmodermat-*]. Type genus: *Osmoderma* Lepeletier and Audinet-Serville, 1828 [placed on the Official List of Generic Names in Zoology (ICZN 2007)]. Comment: incorrect original stem formation, not in prevailing usage.

### Subtribe Platygeniina

Krikken, 1984

Platygeniini Krikken, 1984: 18, in key [stem: *Platygeni-*]. Type genus: *Platygenia* W. S. MacLeay, 1819.

### Subtribe Trichiina

Fleming, 1821

*Trichides Baudet-Lafarge, 1809: 34 [stem: *Trichi-*]. Type genus: *Trichius* Fabricius, 1775 [placed on the Official List of Generic Names in Zoology (ICZN 2004c)]. Comment: original vernacular name unavailable (Art. 11.7.2): subsequently used in latinized form but not generally attributed to Baudet-Lafarge (1809); incorrect original stem formation, not in prevailing usage.
Trichiadae Fleming, 1821: 50 [stem: *Trichi-*]. Type genus: *Trichius* Fabricius, 1775 [placed on the Official List of Generic Names in Zoology (ICZN 2004c)].
Paniscidae Gistel, 1848: [5] [stem: *Panisc-*]. Type genus: *Paniscus* Gistel, 1848 [preoccupied genus name, not *Paniscus* Schrank, 1802 [Hymenoptera]; Gistel (1848: [5]) originally included *Scarabaeusfasciatus* Linnaeus, 1758 and *Trichiuszonatus* Germar, 1831 in his genus *Paniscus*, we hereby select *Scarabaeusfasciatus* Linnaeus, 1758 as the type species of *Paniscus* Gistel, 1848; **syn. nov.** of *Trichius* Fabricius, 1775]. Comment: permanently invalid (Art. 39): based on preoccupied type genus.
Elpidides Péringuey, 1907: 314 [stem: *Elpid-*]. Type genus: *Elpidus* Péringuey, 1907.
Myodermini Péringuey, 1907: 313 [stem: *Myoderm-*]. Type genus: *Myodermum* H. C. C. Burmeister and Schaum, 1840.

### Tribe Valgini

Mulsant, 1842

Valguaires Mulsant, 1842: 519 [stem: *Valg-*]. Type genus: *Valgus* Scriba, 1790.

### Subtribe Microvalgina

Kolbe, 1904

Microvalginae Kolbe, 1904: 10 [stem: *Microvalg-*]. Type genus: *Microvalgus* Kraatz, 1883.

### Subtribe Valgina

Mulsant, 1842

Valguaires Mulsant, 1842: 519 [stem: *Valg-*]. Type genus: *Valgus* Scriba, 1790. Comment: published before 6 August 1842; original vernacular name available (Art. 11.7.2): used in latinized form by several authors, generally accepted as in A. B. T. Smith (2006: 177, as Valgini); this family-group name was also used in the same year by Burmeister (1842 [before 28 December]: 718, as Valgidae); incorrect original stem formation, not in prevailing usage.
Acanthovalginae Kolbe, 1904: 11 [stem: *Acanthovalg-*]. Type genus: *Acanthovalgus* Kraatz, 1895.
Cosmovalginae Kolbe, 1904: 11 [stem: *Cosmovalg-*]. Type genus: *Cosmovalgus* Kolbe, 1897.
Dasyvalginae Kolbe, 1904: 11 [stem: *Dasyvalg-*]. Type genus: *Dasyvalgus* Kolbe, 1904.
Ischnovalginae Kolbe, 1904: 9 [stem: *Ischnovalg-*]. Type genus: *Ischnovalgus* Kolbe, 1897.
Sphinctovalginae Kolbe, 1904: 9 [stem: *Sphinctovalg-*]. Type genus: *Sphinctovalgus* Kolbe, 1904.

### Tribe Xiphoscelidini

Burmeister, 1842

Xiphoscelideae H. C. C. Burmeister, 1842: 613 [stem: *Xiphoscelid-*]. Type genus: *Xiphoscelis* H. C. C. Burmeister, 1842. Comment: incorrect original stem formation, not in prevailing usage.

### †Family Coprinisphaeridae

Genise, 2004

Coprinisphaeridae Genise, 2004: 426 [stem: *Coprinisphaer-*]. Type genus: *Coprinisphaera* Sauer, 1955 [placed on the Official List of Generic Names in Zoology and given precedence over *Fontanai* Roselli, 1939 (ICZN 2008b)]. Comment: ichnotaxon based on dung beetle burrows.

### †Family Pallichnidae

Genise, 2004

Pallichnidae Genise, 2004: 432 [stem: *Pallichn-*]. Type genus: *Pallichnus* Retallack, 1984. Comment: ichnotaxon based on dung beetle burrows.

### Series ELATERIFORMIA

### Superfamily Scirtoidea

Fleming, 1821

Scirtesidae Fleming, 1821: 50 [stem: *Scirt-*]. Type genus: *Scirtes* Illiger, 1807. Comment: as pointed out by Lawrence and Newton (1995: 838) the name Scirtoidea Fleming, 1821 has priority over Clamboidea Fischer von Waldheim, 1821.

### Family Decliniidae

Nikitsky, Lawrence, Kirejtshuk and Gratshev, 1994

Decliniidae Nikitsky et al., 1994: 7 [stem: *Declini-*]. Type genus: *Declinia* Nikitsky et al., 1994.

### Family Eucinetidae

Lacordaire, 1857

Eucinétides Lacordaire, 1857: 281 [stem: *Eucinet-*]. Type genus: *Eucinetus* Germar, 1818. Comment: original vernacular name available (Art. 11.7.2): first used in latinized form by J. L. LeConte (1861: 181, as Eucinetini), generally accepted as in Hansen (1996: 135, as Eucinetidae).
Cryptomeridae Broun, 1893: 1358 [stem: *Cryptomer-*]. Type genus: *Cryptomera* Broun, 1893 [syn. of *Eucinetus* Germar, 1818].

### Family Clambidae

Fischer von Waldheim, 1821

Clambini Fischer von Waldheim, 1821: 52 [stem: *Clamb-*]. Type genus: *Clambus* Fischer von Waldheim, 1821.

### Subfamily Calyptomerinae

Crowson, 1955

Calyptomeridae Crowson, 1955: 11 [stem: *Calyptomer-*]. Type genus: *Calyptomerus* Redtenbacher, 1849.

### Subfamily Acalyptomerinae

Crowson, 1979

Acalyptomerinae Crowson, 1979: 612, in key [stem: *Acalyptomer-*]. Type genus: *Acalyptomerus* Crowson, 1979.

### Subfamily Clambinae

Fischer von Waldheim, 1821

Clambini Fischer von Waldheim, 1821: 52 [stem: *Clamb-*]. Type genus: *Clambus* Fischer von Waldheim, 1821.

### Family Scirtidae

Fleming, 1821

Scirtesidae Fleming, 1821: 50 [stem: *Scirt-*]. Type genus: *Scirtes* Illiger, 1807.
†Sinodryopitidae Hong, 2002: 107 [stem: *Sinodryopit-*]. Type genus: *Sinodryopites* Hong, 2002. Comment: originally described in Byrrhoidea; synonymy with Scirtidae by Kirejtshuk and Azar (2008: 29).

### Subfamily Scirtinae

Fleming, 1821

Scirtesidae Fleming, 1821: 50 [stem: *Scirt-*]. Type genus: *Scirtes* Illiger, 1807. Comment: incorrect original stem formation, not in prevailing usage.
Cyphonidae Stephens, 1829a: 11 [stem: *Cyphon-*]. Type genus: *Cyphon* Paykull, 1799.
Elodiidae Shuckard, 1839b: 41 [stem: *Elod-*]. Type genus: *Elodes* Latreille, 1797. Comment: incorrect original stem formation, not in prevailing usage.
Atopidini Pic, 1914: 16 [stem: *Atopid-*]. Type genus: *Atopida* A. White, 1846.

### Subfamily Nipponocyphoninae

Lawrence and Yoshitomi, 2007

Nipponocyphoninae Lawrence and Yoshitomi, 2007: 522 [stem: *Nipponocyphon-*]. Type genus: *Nipponocyphon* Lawrence and Yoshitomi, 2007.

### Subfamily Stenocyphoninae

Lawrence and Yoshitomi, 2007

Stenocyphoninae Lawrence and Yoshitomi, 2007: 522 [stem: *Stenocyphon-*]. Type genus: *Stenocyphon* Lawrence, 2001.

### †Family Elodophthalmidae

Kirejtshuk and Azar, 2008

Elodophthalmidae Kirejtshuk and Azar, 2008: 24 [stem: *Elodophthalm-*]. Type genus: *Elodophthalmus* Kirejtshuk and Azar, 2008.

### †Famil y Mesocinetidae

Kirejtshuk and Ponomarenko, 2010

Mesocinetidae Kirejtshuk and Ponomarenko, 2010: 304 [stem: *Mesocinet*-]. Type genus: *Mesocinetus* Ponomarenko, 1986.

### Superfamily Dascilloidea

Guérin-Méneville, 1843 (1834)

Dascillidae Guérin-Méneville, 1843: 193 [stem: *Dascill-*]. Type genus: *Dascillus* Latreille, 1797. Comment: usage of younger name conserved over Atopoidea Laporte, 1834 (Art. 40.2); although Rhipiceroidea is also an older name for this superfamily, its use for a taxon including the families Dascilidae (including Karumiinae) and Rhipiceridae is likely to cause some confusion and we therefore continue to use Dascilloidea as valid; the concept of Rhipiceroidea has varied among authors, Crowson (1953) used Rhipiceroidea for the families Rhipiceridae and Callirhipidae, which are not now considered to form a monophyletic group, and Dascilloidea for Dascillidae plus those families now included in Scirtoidea; the family group name Sandalidae (which is a junior synonym of Rhipiceridae) was used by Craighead (1921) and Böving and Craighead (1931) for Rhipiceridae in the strict sense, while Emden (1924, 1931, 1933) used the same name for a family which also included those genera now placed in Callirhipidae; Crowson (1971, 1973b) proposed a reconstituted Dascilloidea for Dascillidae plus Rhipiceridae (sensu stricto) and excluded Callirhipidae from Rhipiceroidea and placed it in another superfamily Artematopoidea (along with Artematopidae (now Artematopodidae) and Brachypsectridae); finally, the evidence for placing Dascillidae and Rhipiceridae in the same superfamily is not convincing (especially when the larvae are taken into account) so it is quite possible that Dascilloidea in the sense of Crowson (1971) may cease to exist when more evidence is presented.

### Family Dascillidae

Guérin-Méneville, 1843 (1834)

Dascillidae Guérin-Méneville, 1843: 193 [stem: *Dascill-*]. Type genus: *Dascillus* Latreille, 1797.

### Subfamily Dascillinae

Guérin-Méneville, 1843 (1834)

Dascillidae Guérin-Méneville, 1843: 193 [stem: *Dascill-*]. Type genus: *Dascillus* Latreille, 1797.

### Tribe Cinnabariini

Pic, 1914

Cinnabariini Pic, 1914: 15 [stem: *Cinnabari-*]. Type genus: *Cinnabarium* Fairmaire, 1895 [syn. of *Coptocera* Murray, 1868].

### Tribe Dascillini

Guérin-Méneville, 1843 (1834)

Atopites Laporte, 1834a: 227 [stem: *Atop-*]. Type genus: *Atopa* Paykull, 1799 [syn. of *Dascillus* Latreille, 1797]. Comment: original vernacular name available (Art. 11.7.2): first used in latinized form and generally accepted as in Laporte (1836: 21, as Atopidae).
Dascillidae Guérin-Méneville, 1843: 193 [stem: *Dascill-*]. Type genus: *Dascillus* Latreille, 1797. Comment: name proposed to replace Atopidae Laporte, 1834 because of the synonymy of the type genus; usage of younger name conserved over Atopini Laporte, 1834 (Art. 40.2) (see Lawrence and Newton 1995).

### Subfamily Karumiinae

Escalera, 1913

Karuminae Escalera, 1913: 320 [stem: *Karumi-*]. Type genus: *Karumia* Escalera, 1913.

### Tribe Emmitini

Escalera, 1914

Emminae Escalera, 1913: 318 [stem: *Emm-*]. Type genus: *Emma* Escalera, 1913 [preoccupied genus name, not *Emma* Gray, 1843 [Bryozoa]; syn. of *Emmita* Escalera, 1914]. Comment: permanently invalid (Art. 39): based on preoccupied type genus.
Emmitinae Escalera, 1914a: 349 [stem: *Emmit-*]. Type genus: *Emmita* Escalera, 1914. Comment: replacement name for Emminae Escalera, 1913 because of the homonymy of the type genus.

### Tribe Escalerinini

Paulus, 1972

Escalerini Paulus, 1972a: 49, in key [stem: *Escalerin-*]. Type genus: *Escalerina* Bolívar, 1926. Comment: incorrect original stem formation, not in prevailing usage.

### Tribe Genecerini

Pic, 1914

Genecerini Pic, 1914: 16 [stem: *Genecer-*]. Type genus: *Genecerus* Walker, 1871.

### Tribe Karumiini

Escalera, 1913

Karuminae Escalera, 1913: 320 [stem: *Karumi-*]. Type genus: *Karumia* Escalera, 1913. Comment: incorrect original stem formation, not in prevailing usage.
Zarudniolidae Semenov and Martynov, 1925: 74 [stem: *Zarudniol-*]. Type genus: *Zarudniola* Semenov and Martynov, 1925 [syn. of *Karumia* Escalera, 1913].

### Family Rhipiceridae

Latreille, 1834

Rhipicerides Latreille, 1834: 167 [stem: *Rhipicer-*]. Type genus: *Rhipicera* Latreille, 1816. Comment: published in issue 1 of volume 3 of the Annales de la Société Entomologique de France; this family-group name was also used in the same year by Laporte (1834a: 226, as Rhipicérites) in issue 2 of volume 3 of the same journal.
Sandalidae Jakobson, 1913: 729 [stem: *Sandal-*]. Type genus: *Sandalus* Knoch, 1801.

### Superfamily Buprestoidea

Leach, 1815

Buprestides Leach, 1815: 85 [stem: *Buprest-*]. Type genus: *Buprestis* Linnaeus, 1758 [placed on the Official List of Generic Names in Zoology (ICZN 1994e)].

### Family Schizopodidae

LeConte, 1859

Schizopodidae J. L. LeConte, 1859b: 122 [stem: *Schizopod-*]. Type genus: *Schizopus* J. L. LeConte, 1858 [placed on the Official List of Generic Names in Zoology (ICZN 1993d)].

### Subfamily Schizopodinae

LeConte, 1859

Schizopodidae J. L. LeConte, 1859b: 122 [stem: *Schizopod-*]. Type genus: *Schizopus* J. L. LeConte, 1858 [placed on the Official List of Generic Names in Zoology (ICZN 1993d)].

### Tribe Dystaxiini

Théry, 1929

Dystaxini Théry, 1929: 60 [stem: *Dystaxi-*]. Type genus: *Dystaxia* J. L. LeConte, 1866. Comment: incorrect original stem formation, not in prevailing usage.
Glyptoscelimorphini Cobos, 1963: 354 [stem: *Glyptoscelimorph-*]. Type genus: *Glyptoscelimorpha* G. H. Horn, 1893.

### †Tribe Electrapatini

Iablokoff-Khnzorian, 1962

Electrapatidae Iablokoff-Khnzorian, 1962: 87 [stem: *Electrapat-*]. Type genus: *Electrapate* Iablokoff-Khnzorian, 1962.

### Tribe Schizopodini

LeConte, 1859

Schizopodidae J. L. LeConte, 1859b: 122 [stem: *Schizopod-*]. Type genus: *Schizopus* J. L. LeConte, 1858 [*Schizopus* J. L. LeConte, 1858 has precedence over *Schizopus* Claparéde et Lachman, 1858 and was placed on the Official List of Generic Names in Zoology (ICZN 1993d)].

### Family Buprestidae

Leach, 1815

Buprestides Leach, 1815: 85 [stem: *Buprest-*]. Type genus: *Buprestis* Linnaeus, 1758 [placed on the Official List of Generic Names in Zoology (ICZN 1994e)].

### Subfamily Julodinae

Lacordaire, 1857

Julodides Lacordaire, 1857: 10 [stem: *Julod-*]. Type genus: *Julodis* Eschscholtz, 1829. Comment: original vernacular name available (Art. 11.7.2): first used in latinized form by J. L. LeConte (1861: 154, as Julodini), generally accepted as in Bellamy (2008a: 23, as Julodinae).
Sternocerini Csiki, 1904: 132 [stem: *Sternocer-*]. Type genus: *Sternocera* Eschscholtz, 1829.
Amblysternini Cobos, 1955: 22, in key [stem: *Amblystern-*]. Type genus: *Amblysterna* Saunders, 1871.

### Subfamily Polycestinae

Lacordaire, 1857

Polycestides Lacordaire, 1857: 61 [stem: *Polycest-*]. Type genus: *Polycesta* Dejean, 1833.

### Tribe Acmaeoderini

Kerremans, 1893

Acmaeoderini Kerremans, 1893: 112 [stem: *Acmaeoder-*]. Type genus: *Acmaeodera* Eschscholtz, 1829 [placed on the Official List of Generic Names in Zoology (ICZN 2005b)].

### Subtribe Acmaeoderina

Kerremans, 1893

Acmaeoderini Kerremans, 1893: 112 [stem: *Acmaeoder-*]. Type genus: *Acmaeodera* Eschscholtz, 1829 [placed on the Official List of Generic Names in Zoology (ICZN 2005b)].

### Subtribe Acmaeoderoidina

Cobos, 1955

Acmaeoderoidini Cobos, 1955: 15 [stem: *Acmaeoderoid-*]. Type genus: *Acmaeoderoides* Van Dyke, 1942.

### Subtribe Nothomorphina

Cobos, 1955

Notomorphini Cobos, 1955: 17 [stem: *Nothomorph-*]. Type genus: *Nothomorpha* Saunders, 1871 [as *Notomorpha*, incorrect subsequent spelling of type genus name, not in prevailing usage]. Comment: Cobos (1955: 23) also used the spelling Nothomorphini in his original paper, this is considered a misspelling since he listed the type genus name as *Notomorpha*; incorrect original stem formation, not in prevailing usage.

### Acmaeoderini incertae sedis

*Odetteina Volkovitsh, 2001: 52, 91 [stem: *Odette-*]. Type genus: *Odettea* Baudon, 1966. Comment: unavailable family-group name, proposed after 1930 without description or bibliographic reference to such a description (Art. 13.1).

### Tribe Astraeini

Cobos, 1980

Astraeusini Cobos, 1980: 28 [stem: *Astrae-*]. Type genus: *Astraeus* Laporte and Gory, 1838 [placed on the Official List of Generic Names in Zoology (ICZN 1966)]. Comment: incorrect original stem formation, not in prevailing usage.

### Tribe Bulini

Bellamy, 1995

Bulisina Bellamy, 1995: 173 [stem: *Bul-*]. Type genus: *Bulis* Laporte and Gory, 1838. Comment: incorrect original stem formation, not in prevailing usage; stem correction by Bellamy (1996: 222).

### Tribe Haplostethini

LeConte, 1861

Haplostethini J. L. LeConte, 1861: 155 [stem: *Haplosteth-*]. Type genus: *Haplostethus* J. L. LeConte, 1860.
Mastogenini J. L. LeConte and G. H. Horn, 1883: 199 [stem: *Mastogeni-*]. Type genus: *Mastogenius* Solier, 1849. Comment: incorrect original stem formation, not in prevailing usage.

### Tribe Paratracheini

Cobos, 1980

Paratrachysae Cobos, 1980: 47 [stem: *Paratrache-*]. Type genus: *Paratrachys* Saunders, 1873. Comment: here we adopt the stem *Paratrache*- since the correct stem for the genus *Trachys*, with the same ending, was recently determined to be *Trache*- (ICZN 2009a).

### Tribe Perucolini

Cobos, 1980

Perucolini Cobos, 1980: 81 [stem: *Perucol-*]. Type genus: *Perucola* Théry, 1925.

### Tribe Polycestini

Lacordaire, 1857

Polycestides Lacordaire, 1857: 61 [stem: *Polycest-*]. Type genus: *Polycesta* Dejean, 1833.

### Subtribe Polycestina

Lacordaire, 1857

Polycestides Lacordaire, 1857: 61 [stem: *Polycest-*]. Type genus: *Polycesta* Dejean, 1833. Comment: original vernacular name available (Art. 11.7.2): first used in latinized form by Stein (1868: 62, as Polycestini), generally accepted as in Bellamy (2008a: 23, as Polycestini).

### Subtribe Xenopseina

Volkovitsh, 2008

Xenopsina Volkovitsh, 2008: 628 [stem: *Xenopse-*]. Type genus: *Xenopsis* Saunders, 1867. Comment: incorrect original stem formation, not in prevailing usage.

### Tribe Polyctesini

Cobos, 1955

Polyctesini Cobos, 1955: 6 [stem: *Polyctes-*]. Type genus: *Polyctesis* Marseul, 1865. Comment: current spelling maintained (Art. 29.5): incorrect stem formation in prevailing usage (should be *Polyctese*-).

### Tribe Prospherini

Cobos, 1980

Prospheresini Cobos, 1980: 84 [stem: *Prospher-*]. Type genus: *Prospheres* Saunders, 1868. Comment: incorrect original stem formation, not in prevailing usage.

### Tribe Ptosimini

Kerremans, 1903

Ptosimites Kerremans, 1903: 37 [stem: *Ptosim-*]. Type genus: *Ptosima* Dejean, 1833.

### Tribe Thrincopygini

LeConte, 1861

Thrincopygini J. L. LeConte, 1861: 154 [stem: *Thrincopyg-*]. Type genus: *Thrincopyge* J. L. LeConte, 1858.

### Tribe Tyndaridini

Cobos, 1955

Tyndarini Cobos, 1955: 11 [stem: *Tyndarid-*]. Type genus: *Tyndaris* J. Thomson, 1857.

### Subtribe Mimicoclytrinina

Bellamy, 2003

Acherusini Cobos, 1955: 24, in key [stem: *Acherusi-*]. Type genus: *Acherusia* Laporte and Gory, 1838 [preoccupied genus name, not *Acherusia* Costa, 1834 [Crustacea]; syn. of *Mimicoclytrina* Bellamy, 2003]. Comment: permanently invalid (Art. 39): based on preoccupied type genus.
Mimicoclytrinina Bellamy, 2003: 25 [stem: *Mimicoclytrin-*]. Type genus: *Mimicoclytrina* Bellamy, 2003. Comment: replacement name for Acherusiina Cobos, 1955 because of the homonymy of the type genus.

### Subtribe Pseudacherusiina

Cobos, 1980

Pseudoacherusini Cobos, 1980: 78 [stem: *Pseudacherusi-*]. Type genus: *Pseudacherusia* Kerremans, 1905. Comment: incorrect original stem formation, not in prevailing usage.

### Subtribe Tylaucheniina

Cobos, 1959

Tylacheniae Cobos, 1959: 4 [stem: *Tylaucheni-*]. Type genus: *Tylauchenia* H. C. C. Burmeister, 1872 [syn. of *Ocypetes* Saunders, 1871]. Comment: incorrect original stem formation, not in prevailing usage.

### Subtribe Tyndaridina

Cobos, 1955

Tyndarini Cobos, 1955: 11 [stem: *Tyndarid-*]. Type genus: *Tyndaris* J. Thomson, 1857. Comment: incorrect original stem formation, not in prevailing usage.

### Tribe Xyroscelidini

Cobos, 1955

Xiroscelini Cobos, 1955: 19 [stem: *Xyroscelid-*]. Type genus: *Xyroscelis* Saunders, 1868 [as *Xiroscelis*, incorrect subsequent spelling of type genus name, not in prevailing usage]. Comment: incorrect original stem formation, not in prevailing usage.

### Subfamily Galbellinae

Reitter, 1911

Galbellinae Reitter, 1911: 178 [stem: *Galbell-*]. Type genus: *Galbella* Westwood, 1848.

### Subfamily Chrysochroinae

Laporte, 1835

Chrysochroidae Laporte, 1835b: 158 [stem: *Chrysochro-*]. Type genus: *Chrysochroa* Dejean, 1833.

### Tribe Chrysochroini

Laporte, 1835

Chrysochroidae Laporte, 1835b: 158 [stem: *Chrysochro-*]. Type genus: *Chrysochroa* Dejean, 1833.

### Subtribe Chalcophorina

Lacordaire, 1857 (1848)

Anaglyptisidae Gistel, 1848: [5] [stem: *Anaglypt-*]. Type genus: *Anaglyptes* Gistel, 1848 [syn. of *Chalcophora* Dejean, 1833]. Comment: senior homonym of Anaglyptini Lacordaire, 1868 (type genus *Anaglyptus* Mulsant, 1839) in Cerambycidae; incorrect original stem formation, not in prevailing usage; the younger name Chalcophorina Lacordaire, 1857 is conserved over this name (Art. 40.2).
Chalcophorides Lacordaire, 1857: 14 [stem: *Chalcophor-*]. Type genus: *Chalcophora* Dejean, 1833. Comment: original vernacular name available (Art. 11.7.2): first used in latinized form by J. L. LeConte (1861: 151, as Chalcophorae), generally accepted as in Bellamy (2008a: 23, as Chalcophorina); name conserved over the older name Anaglyptina Gistel, 1848 (Art. 40.2).
*Chrysodemides H. Deyrolle, 1865: 11 [stem: *Chrysodem-*]. Type genus: *Chrysodema* Laporte and Gory, 1837 [placed on the Official List of Generic Names in Zoology (ICZN 2004b)]. Comment: original vernacular name unavailable (Art. 11.7.2): subsequently used in latinized form but not generally attributed to H. Deyrolle (1865).
Chrysodémides Kerremans, 1892: 49 [stem: *Chrysodem-*]. Type genus: *Chrysodema* Laporte and Gory, 1837 [placed on the Official List of Generic Names in Zoology (ICZN 2004b)]. Comment: original vernacular name available (Art. 11.7.2): first used in latinized form and generally accepted as in Skinner (1892: 124, as Chrysodemidae).
Chalcophorellini Tôyama, 1986: 189 [stem: *Chalcophorell-*]. Type genus: *Chalcophorella* Kerremans, 1903.
Iridotaenini Tôyama, 1987: 5 [stem: *Iridotaeni-*]. Type genus: *Iridotaenia* Deyrolle, 1864 [placed on the Official List of Generic Names in Zoology (ICZN 2004b)]. Comment: incorrect original stem formation, not in prevailing usage.
Lampropeplina Holyński, 1993: 22 [stem: *Lampropepl-*]. Type genus: *Lampropepla* Fairmaire, 1904 [syn. of *Madecassia* Kerremans, 1903].

### Subtribe Chrysochroina

Laporte, 1835

Chrysochroidae Laporte, 1835b: 158 [stem: *Chrysochro-*]. Type genus: *Chrysochroa* Dejean, 1833.
Catoxanthina Jakobson, 1913: 772 [stem: *Catoxanth-*]. Type genus: *Catoxantha* Solier, 1833.

### Subtribe Eucallopistina

Bellamy, 2003

Callopistina Kurosawa, 1990: 63 [stem: *Callopist-*]. Type genus: *Callopistus* Deyrolle, 1864 [preoccupied genus name, not *Callopistus* Say, 1831 [Coleoptera: Curculionidae]; syn. of *Eucallopistus* Bellamy, 2003]. Comment: permanently invalid (Art. 39): based on preoccupied type genus.
Eucallopistina Bellamy, 2003: 31 [stem: *Eucallopist-*]. Type genus: *Eucallopistus* Bellamy, 2003. Comment: replacement name for Callopistina Kurosawa, 1990 because of the homonymy of the type genus.

### Tribe Dicercini

Gistel, 1848

Dicercaeidae Gistel, 1848: [5] [stem: *Dicerc-*]. Type genus: *Dicerca* Eschscholtz, 1829 [placed on the Official List of Generic Names in Zoology (ICZN 1994e)]. Comment: First Reviser found (Dicercini Gistel, 1848 vs Polybothrisini Gistel, 1848) is Bellamy (2008b: 819).

### Subtribe Dicercina

Gistel, 1848

Dicercaeidae Gistel, 1848: [5] [stem: *Dicerc-*]. Type genus: *Dicerca* Eschscholtz, 1829 [placed on the Official List of Generic Names in Zoology (ICZN 1994e)]. Comment: incorrect original stem formation, not in prevailing usage.
Polybothrisidae Gistel, 1848: [5] [stem: *Polybothrid-*]. Type genus: *Polybothris* Spinola, 1837. Comment: incorrect original stem formation, not in prevailing usage.
Psiloptérides Lacordaire, 1857: 26 [stem: *Psilopter-*]. Type genus: *Psiloptera* Dejean, 1833. Comment: original vernacular name available (Art. 11.7.2): first used in latinized form by J. Thomson (1858: 72, as Psilopteritae), generally accepted as in Bellamy (2002: 61, as Psilopterini).
Capnodina Jakobson, 1913: 779 [stem: *Capnod-*]. Type genus: *Capnodis* Eschscholtz, 1829.

### Subtribe Haplotrinchina

Holyński, 1993

Haplotrinchina Holyński, 1993: 27 [stem: *Haplotrinch-*]. Type genus: *Haplotrinchus* Kerremans, 1903.

### Subtribe Hippomelanina

Holyński, 1993

Hippomelanina Holyński, 1993: 24 [stem: *Hippomelan-*]. Type genus: *Hippomelas* Gory and Laporte, 1837.

### Subtribe Pseudoperotina

Tôyama, 1987

Pseudoperotina Tôyama, 1987: 4 [stem: *Pseudoperot-*]. Type genus: *Pseudoperotis* Obenberger, 1936.

### Tribe Evidini

Tôyama, 1987

Evidini Tôyama, 1987: 6 [stem: *Evid-*]. Type genus: *Evides* Dejean, 1833.

### Tribe Paraleptodemini

Cobos, 1975

Paraleptodemini Cobos, 1975: 88 [stem: *Paraleptodem-*]. Type genus: *Paraleptodema* Obenberger, 1936 [syn. of *Cinyra* Laporte and Gory, 1837].

### Subtribe Euchromatina

Holyński, 1993

Euchromatina Holyński, 1993: 23 [stem: *Euchromat-*]. Type genus: *Euchroma* Dejean, 1833.

### Subtribe Euplectaleciina

Holyński, 1993

Euplectaleciina Holyński, 1993: 24 [stem: *Euplectaleci-*]. Type genus: *Euplectalecia* Obenberger, 1924.

### Subtribe Hypoprasina

Holyński, 1993

Hypoprasina Holyński, 1993: 23 [stem: *Hypopras-*]. Type genus: *Hypoprasis* Fairmaire and Germain, 1864. Comment: current spelling maintained (Art. 29.5): incorrect stem formation in prevailing usage (should be *Hypoprase*-).

### Subtribe Paraleptodemina

Cobos, 1975

Paraleptodemini Cobos, 1975: 88 [stem: *Paraleptodem-*]. Type genus: *Paraleptodema* Obenberger, 1936 [syn. of *Cinyra* Laporte and Gory, 1837].
Cinyrini Cobos, 1979a: 226 [stem: *Cinyr-*]. Type genus: *Cinyra* Gory and Laporte, 1837.

### Subtribe Pristipterina

Holyński, 1993

Pristipterina Holyński, 1993: 23 [stem: *Pristipter-*]. Type genus: *Pristiptera* Dejean, 1833 [syn. of *Pelecopselaphus* Solier, 1833].

### Tribe Paratassini

Bílý and Volkovitsh, 1996

Paratassini Bílý and Volkovitsh, 1996: 329 [stem: *Paratass-*]. Type genus: *Paratassa* Marseul, 1882.

### Tribe Poecilonotini

Jakobson, 1913

Poecilonotina Jakobson, 1913: 773 [stem: *Poecilonot-*]. Type genus: *Poecilonota* Eschscholtz, 1829 [placed on the Official List of Generic Names in Zoology (ICZN 1996a)].

### Subtribe Poecilonotina

Jakobson, 1913

Poecilonotina Jakobson, 1913: 773 [stem: *Poecilonot-*]. Type genus: *Poecilonota* Eschscholtz, 1829 [placed on the Official List of Generic Names in Zoology (ICZN 1996a)].

### Subtribe Nesotrinchina

Bílý, Kubáň and Volkovitsh, 2009

Nesotrinchina Bílý et al., 2009: 750 [stem: *Nesotrinch-*]. Type genus: *Nesotrinchus* Obenberger, 1924.

### Tribe Sphenopterini

Lacordaire, 1857

Sphénoptérides Lacordaire, 1857: 68 [stem: *Sphenopter-*]. Type genus: *Sphenoptera* Dejean, 1833. Comment: original vernacular name available (Art. 11.7.2): first used in latinized form by Stein (1868: 62, as Sphenopterini), generally accepted as in Bellamy (2008a: 23, as Sphenopterini).

### Tribe Vadonaxiini

Descarpentries, 1970

Vadonaxiini Descarpentries, 1970: 188 [stem: *Vadonaxi-*]. Type genus: *Vadonaxia* Descarpentries, 1970.

### Subfamily Buprestinae

Leach, 1815

Buprestides Leach, 1815: 85 [stem: *Buprest-*]. Type genus: *Buprestis* Linnaeus, 1758 [placed on the Official List of Generic Names in Zoology (ICZN 1994e)].

### Tribe Actenodini

Gistel, 1848

Actenodeidae Gistel, 1848: [5] [stem: *Actenod-*]. Type genus: *Actenodes* Dejean, 1833 [placed on the Official List of Generic Names in Zoology (ICZN 2002b)]. Comment: name previously attributed to Kerremans (1890) in the literature; incorrect original stem formation, not in prevailing usage.
Belionotina Jakobson, 1913: 793 [stem: *Belionot-*]. Type genus: *Belionota* Eschscholtz, 1829.

### Tribe Anthaxiini

Gory and Laporte, 1839

Anthaxidae Gory and Laporte, 1839: unnumbered page [stem: *Anthaxi-*]. Type genus: *Anthaxia* Eschscholtz, 1829 [placed on the Official List of Generic Names in Zoology (ICZN 2002c)]. Comment: incorrect original stem formation, not in prevailing usage.

### Tribe Bubastini

Obenberger, 1920

Bubastini Obenberger, 1920: 89 [stem: *Bubast-*]. Type genus: *Bubastes* Laporte and Gory, 1838.

### Tribe Buprestini

Leach, 1815

Buprestides Leach, 1815: 85 [stem: *Buprest-*]. Type genus: *Buprestis* Linnaeus, 1758 [placed on the Official List of Generic Names in Zoology (ICZN 1994e)].

### Subtribe Agaeocerina

Nelson, 1982

Agaeocerini Nelson, 1982: 440 [stem: *Agaeocer-*]. Type genus: *Agaeocera* Saunders, 1871.

### Subtribe Buprestina

Leach, 1815

Buprestides Leach, 1815: 85 [stem: *Buprest-*]. Type genus: *Buprestis* Linnaeus, 1758 [placed on the Official List of Generic Names in Zoology (ICZN 1994e)]. Comment: current spelling maintained (Art. 29.3.1.1): incorrect stem formation in prevailing usage (should be *Buprestid*-).
Ancylochirina Jakobson, 1913: 787 [stem: *Ancylochir-*]. Type genus: *Ancylochira* Eschscholtz, 1829 [subgenus of *Buprestis* Linnaeus, 1758].

### Subtribe Lamprocheilina

Holyński, 1993

Lamprocheilina Holyński, 1993: 13 [stem: *Lamprocheil-*]. Type genus: *Lamprocheila* Saunders, 1871.

### Subtribe Trachykelina

Holyński, 1988

Trachykelina Holyński, 1988: 51, in key [stem: *Trachykel-*]. Type genus: *Trachykele* Marseul, 1865.

### Tribe Chrysobothrini

Gory and Laporte, 1836

Chrysobothridae Gory and Laporte, 1836: [1] [stem: *Chrysobothr-*]. Type genus: *Chrysobothris* Eschscholtz, 1829 [placed on the Official List of Generic Names in Zoology (ICZN 1994e)]. Comment: current spelling maintained (Art. 29.3.1.1): incorrect stem formation in prevailing usage (should be *Chrysobothrid*-).

### Tribe Coomaniellini

Bílý, 1974

Coomaniellini Bílý, 1974: 41 [stem: *Coomaniell-*]. Type genus: *Coomaniella* Bourgoin, 1924.

### Tribe Curidini

Holyński, 1988

Curidina Holyński, 1988: 52, in key [stem: *Curid-*]. Type genus: *Curis* Gory and Laporte, 1837 [syn. of *Selagis* Dejean, 1836 (also see ICZN 2008c)]. Comment: First Reviser (Curidini Holyński, 1988 vs Neocuridini Holyński, 1988) not determined, current usage maintained.

### Subtribe Anilarina

Bílý, 2000

Anilarini Bílý, 2000: 113 [stem: *Anilar-*]. Type genus: *Anilara* Saunders, 1868.

### Subtribe Curidina

Holyński, 1988

Curidina Holyński, 1988: 52, in key [stem: *Curid-*]. Type genus: *Curis* Gory and Laporte, 1837 [syn. of *Selagis* Dejean, 1836 (also see ICZN 2008c)].

### Subtribe Neocuridina

Holyński, 1988

Neocuridina Holyński, 1988: 52, in key [stem: *Neocurid-*]. Type genus: *Neocuris* Saunders, 1868.

### Tribe Epistomentini

Levey, 1978

Epistomentini Levey, 1978: 155 [stem: *Epistoment-*]. Type genus: *Epistomentis* Solier, 1849.

### Tribe Exagistini

Tôyama, 1987

Exagistini Tôyama, 1987: 2 [stem: *Exagist-*]. Type genus: *Exagistus* Deyrolle, 1864.

### Tribe Julodimorphini

Kerremans, 1903

Julodimorphites Kerremans, 1903: 16 [stem: *Julodimorph-*]. Type genus: *Julodimorpha* Harold, 1869.

### Tribe Kisanthobiini

Richter, 1949

Kisanthobiini Richter, 1949: 215 [stem: *Kisanthobi-*]. Type genus: *Kisanthobia* Marseul, 1865.

### Tribe Maoraxiini

Holyński, 1984

Maoraxiini Holyński, 1984: 106 [stem: *Maoraxi-*]. Type genus: *Maoraxia* Obenberger, 1937.

### Tribe Melanophilini

Bedel, 1921

Melanophilini Bedel, 1921: 171 [stem: *Melanophil-*]. Type genus: *Melanophila* Eschscholtz, 1829 [placed on the Official List of Generic Names in Zoology (ICZN 1996b)].

### Tribe Melobaseini

Bílý, 2000

Melobasini Bílý, 2000: 113 [stem: *Melobase-*]. Type genus: *Melobasis* Gory and Laporte, 1837. Comment: incorrect original stem formation, not in prevailing usage.

### Tribe Mendizabaliini

Cobos, 1968

Mendizabaliini Cobos, 1968: 19 [stem: *Mendizabali-*]. Type genus: *Mendizabalia* Cobos, 1957.

### Tribe Nascionini

Holyński, 1988

Nascionina Holyński, 1988: 51, in key [stem: *Nascion-*]. Type genus: *Nascio* Laporte and Gory, 1838 [placed on the Official List of Generic Names in Zoology (ICZN 2002b)].

### Tribe Phrixiini

Cobos, 1975

Phrixiini Cobos, 1975: 102 [stem: *Phrixi-*]. Type genus: *Phrixia* Deyrolle, 1864.

### Tribe Pterobothrini

Volkovitsh, 2001

Pterobothrini Volkovitsh, 2001: 86, 103 [stem: *Pterobothr-*]. Type genus: *Pterobothris* Fairmaire and Germain, 1858. Comment: current spelling maintained (Art. 29.3.1.1): incorrect original stem formation in prevailing usage (should be *Pterobothrid*-).

### Tribe Stigmoderini

Lacordaire, 1857

Stigmodérides Lacordaire, 1857: 52 [stem: *Stigmoder-*]. Type genus: *Stigmodera* Eschscholtz, 1829. Comment: original vernacular name available (Art. 11.7.2): first used in latinized form by Wallace (1860: 183, as Stigmoderidae), generally accepted as in Bellamy (2008a: 23, as Stigmoderini).

### Tribe Thomassetiini

Bellamy, 1987

Thomassetiini Bellamy, 1987: 223 [stem: *Thomasseti-*]. Type genus: *Thomassetia* Théry, 1928.

### Subtribe Philanthaxiina

Holyński, 1988

Philanthaxiina Holyński, 1988: 51, in key [stem: *Philanthaxi-*]. Type genus: *Philanthaxia* Deyrolle, 1864.

### Subtribe Thomassetiina

Bellamy, 1987

Thomassetiini Bellamy, 1987: 223 [stem: *Thomasseti-*]. Type genus: *Thomassetia* Théry, 1928.

### Tribe Trigonogeniini

Cobos, 1956

Trigonogeniini Cobos, 1956: 72 [stem: *Trigonogeni-*]. Type genus: *Trigonogenium* Harold, 1869.

### Tribe Xenorhipidini

Cobos, 1986

Xenorhipini Cobos, 1986: 136 [stem: *Xenorhipid-*]. Type genus: *Xenorhipis* J. L. LeConte, 1866. Comment: incorrect original stem formation, not in prevailing usage.

### Subtribe Trichinorhipidina

Bellamy, 2006

Trichinorhipidina Bellamy, 2006: 139 [stem: *Trichinorhipid-*]. Type genus: *Trichinorhipis* Barr, 1948.

### Subtribe Xenorhipidina

Cobos, 1986

Xenorhipini Cobos, 1986: 136 [stem: *Xenorhipid-*]. Type genus: *Xenorhipis* J. L. LeConte, 1866. Comment: incorrect original stem formation, not in prevailing usage.

### Buprestinae incertae sedis

†Glaphyropteridae Pongrácz, 1935: 541 [stem: *Glaphyropter-*]. Type genus: *Glaphyroptera* Heer, 1852. Comment: the older name Glaphyropteridae Brauer, 1852 is a collective name for most Neuroptera in the present sense and it is not based on a genus name (Ponomarenko 2009 pers. comm.).

### Subfamily Agrilinae

Laporte, 1835

Agrilidae Laporte, 1835b: 165 [stem: *Agril-*]. Type genus: *Agrilus* Curtis, 1825. Comment: First Reviser (Agrilinae Laporte, 1835 vs Tracheinae Laporte, 1835) not determined, current usage maintained.

### Tribe Agrilini

Laporte, 1835

Agrilidae Laporte, 1835b: 165 [stem: *Agril-*]. Type genus: *Agrilus* Curtis, 1825.

### Subtribe Agrilina

Laporte, 1835

Agrilidae Laporte, 1835b: 165 [stem: *Agril-*]. Type genus: *Agrilus* Curtis, 1825.

### Subtribe Amorphosternina

Cobos, 1974

Amorphosternae Cobos, 1974: 69 [stem: *Amorphostern-*]. Type genus: *Amorphosternus* Deyrolle, 1864.

### Subtribe Amyiina

Holyński, 1993

Amyiina Holyński, 1993: 14 [stem: *Amyi-*]. Type genus: *Amyia* Saunders, 1871.

### Subtribe Rhaeboscelidina

Cobos, 1976

*Rhaeboscelini Böving and Craighead, 1931: 77 [stem: *Rhaeboscelid-*]. Type genus: *Rhaeboscelis* Chevrolat, 1838. Comment: unavailable family-group name, proposed after 1930 without description or bibliographic reference to such a description (Art. 13.1); incorrect original stem formation, not in prevailing usage.
Rhaeboscelidi Cobos, 1976: 20 [stem: *Rhaeboscelid-*]. Type genus: *Rhaeboscelis* Chevrolat, 1838.

### Tribe Aphanisticini

Jacquelin du Val, 1859

Aphanisticites Jacquelin du Val, 1859: 104 [stem: *Aphanistic-*]. Type genus: *Aphanisticus* Latreille, 1810.

### Subtribe Anthaxomorphina

Holyński, 1993

Anthaxomorphina Holyński, 1993: 32 [stem: *Anthaxomorph-*]. Type genus: *Anthaxomorphus* Deyrolle, 1864.

### Subtribe Aphanisticina

Jacquelin du Val, 1859

Aphanisticites Jacquelin du Val, 1859: 104 [stem: *Aphanistic-*]. Type genus: *Aphanisticus* Latreille, 1829. Comment: original vernacular name available (Art. 11.7.2): first used in latinized form by Acloque (1896: 280, as Aphanisticii), generally accepted as in Hansen (1996: 137, as Aphanisticini).

### Subtribe Cylindromorphina

Portevin, 1931

Cylindromorphini Portevin, 1931: 335 [stem: *Cylindromorph-*]. Type genus: *Cylindromorphus* Kiesenwetter, 1857.

### Subtribe Cylindromorphoidina

Cobos, 1979

Cylindromorphoidini Cobos, 1979b: 420, in key [stem: *Cylindromorphoid-*]. Type genus: *Cylindromorphoides* Kerremans, 1903.

### Subtribe Germaricina

Cobos, 1979

Germaricini Cobos, 1979b: 420, in key [stem: *Germaric-*]. Type genus: *Germarica* Blackburn, 1887.

### Tribe Coraebini

Bedel, 1921

Coraebini Bedel, 1921: 170 [stem: *Coraeb-*]. Type genus: *Coraebus* Gory and Laporte, 1839.

### Subtribe Amorphosomatina

Majer, 2000

Amorphosomina Majer, 2000: 210 [stem: *Amorphosomat-*]. Type genus: *Amorphosoma* Laporte, 1835. Comment: incorrect original stem formation, not in prevailing usage.

### Subtribe Cisseina

Majer, 2000

Cisseina Majer, 2000: 203 [stem: *Cisse-*]. Type genus: *Cisseis* Gory and Laporte, 1839 [syn. of *Diphucrania* Dejean, 1833 (see ICZN 2008c)].

### Subtribe Clematina

Majer, 2000

Clemina Majer, 2000: 215 [stem: *Clemat-*]. Type genus: *Clema* Semenov, 1900. Comment: incorrect original stem formation, not in prevailing usage.

### Subtribe Coraebina

Bedel, 1921

Coraebini Bedel, 1921: 170 [stem: *Coraeb-*]. Type genus: *Coraebus* Gory and Laporte, 1839.

### Subtribe Dismorphina

Cobos, 1990

Dismorphina Cobos, 1990: 542 [stem: *Dismorph-*]. Type genus: *Dismorpha* Gistel, 1848.

### Subtribe Ethoniina

Majer, 2000

Ethoniina Majer, 2000: 201 [stem: *Ethoni-*]. Type genus: *Ethonion* Kubáň, 2000.

### Subtribe Geraliina

Cobos, 1988

Geraliina Cobos, 1988: 10 [stem: *Gerali-*]. Type genus: *Geralius* Harold, 1869.

### Subtribe Meliboeina

Majer, 2000

Meliboeina Majer, 2000: 213 [stem: *Meliboe-*]. Type genus: *Meliboeus* Deyrolle, 1864.

### Subtribe Synechocerina

Majer, 2000

Synechocerina Majer, 2000: 214 [stem: *Synechocer-*]. Type genus: *Synechocera* Deyrolle, 1864.

### Subtribe Toxoscelina

Majer, 2000

Toxoscelina Majer, 2000: 207 [stem: *Toxoscel-*]. Type genus: *Toxoscelus* Deyrolle, 1864.

### Tribe Tracheini

Laporte, 1835

Trachisidae Laporte, 1835b: 166 [stem: *Trache-*]. Type genus: *Trachys* Fabricius, 1801 [placed on the Official List of Generic Names in Zoology (ICZN 2009a)]. Comment: correct stem ruled to be *Trache*- and Tracheidae Laporte, 1835 placed on the Official List of Family-Group Names in Zoology (ICZN 2009a).

### Subtribe Brachina

LeConte, 1861

Braches J. L. LeConte, 1861: 156 [stem: *Brach-*]. Type genus: *Brachys* Dejean, 1833. Comment: current spelling maintained (Art. 29.5): incorrect stem formation in prevailing usage (should be *Brache*-).
Brachyini Cobos, 1979b: 417, in key [stem: *Brach-*]. Type genus: *Brachys* Dejean, 1833. Comment: family-group name proposed as a new taxon, without reference to Braches J. L. LeConte, 1861; incorrect original stem formation, not in prevailing usage.

### Subtribe Leiopleurina

Holyński, 1993

Leiopleurina Holyński, 1993: 32 [stem: *Leiopleur-*]. Type genus: *Leiopleura* Deyrolle, 1864.

### Subtribe Pachyschelina

Böving and Craighead, 1931

Pachyschelinae Böving and Craighead, 1931: 49, in key [stem: *Pachyschel-*]. Type genus: *Pachyschelus* Solier, 1833.

### Subtribe Tracheina

Laporte, 1835

Trachisidae Laporte, 1835b: 166 [stem: *Trache-*]. Type genus: *Trachys* Fabricius, 1801 [placed on the Official List of Generic Names in Zoology (ICZN 2009a)]. Comment: published before 6 May 1835; correct stem ruled to be *Trache*- and Tracheidae Laporte, 1835 placed on the Official List of Family-Group Names in Zoology (ICZN 2009a); Trachisidae Laporte, 1835 deemed to be an incorrect original spelling and placed on the Official Index of Rejected and Invalid Family-Group Names in Zoology along with subsequent family-group names based on *Trachys* Fabricius (ICZN 2009a); the correct spelling Tracheidae was first used by Gistel (1848: [5]); this family-group name was also used in the same year by Solier (1835c [before 4 May]: c, as Trachysides).
Phytoteradae Gistel, 1856a: 366 [stem: *Phytoter-*]. Type genus: *Phytotera* Gistel, 1856 [syn. of *Trachys* Fabricius, 1801]. Comment: incorrect original stem formation, not in prevailing usage.

### †Subfamily Parathyreinae

Alexeev, 1994

Parathyreinae Alexeev, 1994: 10 [stem: *Parathyre-*]. Type genus: *Parathyrea* Alexeev, 1994.

### Superfamily Byrrhoidea

Latreille, 1804

Byrrhii Latreille, 1804c: 146 [stem: *Byrrh-*]. Type genus: *Byrrhus* Linnaeus, 1767 [placed on the Official List of Generic Names in Zoology (ICZN 1994a)].

### Family Byrrhidae

Latreille, 1804

Byrrhii Latreille, 1804c: 146 [stem: *Byrrh-*]. Type genus: *Byrrhus* Linnaeus, 1767 [placed on the Official List of Generic Names in Zoology (ICZN 1994a)].

### Subfamily Byrrhinae

Latreille, 1804

Byrrhii Latreille, 1804c: 146 [stem: *Byrrh-*]. Type genus: *Byrrhus* Linnaeus, 1767 [placed on the Official List of Generic Names in Zoology (ICZN 1994a)].

### Tribe Byrrhini

Latreille, 1804

Byrrhii Latreille, 1804c: 146 [stem: *Byrrh-*]. Type genus: *Byrrhus* Linnaeus, 1767 [placed on the Official List of Generic Names in Zoology (ICZN 1994a)]. Comment: published 7 March 1804; this family-group name was also used in the same year by Latreille (1804a [between 19 August and 17 September]: 190, as Byrrhii).
*Tylicini Johnson, 1991: 160 [stem: *Tylic*-]. Type genus: *Tylicus* Casey, 1912 [syn. of *Arctobyrrhus* Munster, 1902]. Comment: unavailable family-group name, proposed after 1930 without description or bibliographic reference to such a description (Art. 13.1).

### Tribe Exomellini

Casey, 1914

Exomellini Casey, 1914a: 378 [stem: *Exomell-*]. Type genus: *Exomella* Casey, 1914.

### Tribe Morychini

El Moursy, 1961

Morychini El Moursy, 1961: 11 [stem: *Morych-*]. Type genus: *Morychus* Erichson, 1847.

### Tribe Pedilophorini

Casey, 1912

Pedilophorini Casey, 1912: 4 [stem: *Pedilophor-*]. Type genus: *Pedilophorus* Steffahny, 1843.

### Tribe Simplocariini

Mulsant and Rey, 1869

Simplocariates Mulsant and Rey, 1869: 151 [stem: *Simplocari-*]. Type genus: *Simplocaria* Stephens, 1829 [placed on the Official List of Generic Names in Zoology (ICZN 1985c)]. Comment: original vernacular name available (Art. 11.7.2): first used in latinized form by Casey (1912: 14, as Simplocariini), generally accepted as in P. J. Johnson (2002b: 115, as Simplocariini).
Lioonini Leng, 1920: 193 [stem: *Lio-*]. Type genus: *Lioon* Casey, 1912. Comment: incorrect original stem formation, not in prevailing usage.

### Subfamily Syncalyptinae

Mulsant and Rey, 1869

Syncalyptaires Mulsant and Rey, 1869: 31 [stem: *Syncalypt-*]. Type genus: *Syncalypta* Dillwyn, 1829 [syn. of *Chaetophora* Kirby and Spence, 1817].

### Tribe Microchaetini

Paulus, 1973

Microchaetini Paulus, 1973: 353, in key [stem: *Microchaet-*]. Type genus: *Microchaetes* Hope, 1834.

### Tribe Syncalyptini

Mulsant and Rey, 1869

Syncalyptaires Mulsant and Rey, 1869: 31 [stem: *Syncalypt-*]. Type genus: *Syncalypta* Dillwyn, 1829 [syn. of *Chaetophora* Kirby and Spence, 1817]. Comment: original vernacular name available (Art. 11.7.2): first used in latinized form by Portevin (1931: 290, as Syncalyptini), generally accepted as in Hansen (1996: 138, as Syncalyptinae).

### Subfamily Amphicyrtinae

LeConte, 1861

Amphicyrtini J. L. LeConte, 1861: 111 [stem: *Amphicyrt-*]. Type genus: *Amphicyrta* Erichson, 1843.

### Family Elmidae

Curtis, 1830

Elmidae Curtis, 1830: pl. 294 [stem: *Elm-*]. Type genus: *Elmis* Latreille, 1802 [placed on the Official List of Generic Names in Zoology (ICZN 1995d)]. Comment: name placed on the Official List of Family-Group Names in Zoology and correct stem ruled to be *Elm*- (ICZN 1995d).

### Subfamily Larainae

LeConte, 1861

Larini J. L. LeConte, 1861: 116 [stem: *Lara-*]. Type genus: *Lara* J. L. LeConte, 1852 [placed on the Official List of Generic Names in Zoology (ICZN 1988g)].

### Tribe Laraini

LeConte, 1861

Larini J. L. LeConte, 1861: 116 [stem: *Lara-*]. Type genus: *Lara* J. L. LeConte, 1852 [placed on the Official List of Generic Names in Zoology (ICZN 1988g)]. Comment: Laraini J. L. LeConte, 1861 placed on the Official List of Family-Group Names in Zoology and correct stem ruled to be *Lara*- to avoid homonymy with Laridae Rafinesque-Schmaltz, 1815 (type genus *Larus* Linnaeus, 1758) in Aves (ICZN 1988g); Larini J. L. LeConte, 1861 placed on the Official Index of Rejected and Invalid Family-Group Names in Zoology (ICZN 1988g).

### Tribe Potamophilini

Mulsant and Rey, 1872

*Potamophiles Motschulsky, 1849: 54 [stem: *Potamophil-*]. Type genus: *Potamophilus* Germar, 1811. Comment: original vernacular name unavailable (Art. 11.7.2): subsequently used in latinized form but not generally attributed to Motschulsky (1849).
Potamophilaires Mulsant and Rey, 1872b: 11 [stem: *Potamophil-*]. Type genus: *Potamophilus* Germar, 1811. Comment: original vernacular name available (Art. 11.7.2): first used in latinized form by Ganglbauer (1904: 99, as Potamophilini), generally accepted as in Jäch et al. (2006: 432, as Potamophilini).

### Subfamily Elminae

Curtis, 1830

Elmidae Curtis, 1830: pl. 294 [stem: *Elm-*]. Type genus: *Elmis* Latreille, 1802 [placed on the Official List of Generic Names in Zoology (ICZN 1995d)]. Comment: placed on the Official List of Family-Group Names in Zoology and correct stem ruled to be *Elm*- (ICZN 1995d).

### Tribe Ancyronychini

Ganglbauer, 1904

Ancyronychini Ganglbauer, 1904: 108 [stem: *Ancyronych-*]. Type genus: *Ancyronyx* Erichson, 1847.

### Tribe Elmini

Curtis, 1830

Elmidae Curtis, 1830: pl. 294 [stem: *Elm-*]. Type genus: *Elmis* Latreille, 1802 [placed on the Official List of Generic Names in Zoology (ICZN 1995d)]. Comment: Elmidae Curtis, 1830 placed on the Official List of Family-Group Names in Zoology and correct stem ruled to be *Elm*- (ICZN 1995d).

### Subtribe Elmina

Curtis, 1830

*Limniidae Stephens, 1828: 104 [stem: *Limni-*]. Type genus: *Limnius* Illiger, 1802. Comment: unavailable family-group name, not based on genus used as valid at the time (see Jäch 1994).
*Limniidae Stephens, 1829a: 5 [stem: *Limni-*]. Type genus: *Limnius* Illiger, 1802. Comment: unavailable family-group name, not based on genus used as valid at the time.
Elmidae Curtis, 1830: pl. 294 [stem: *Elm-*]. Type genus: *Elmis* Latreille, 1802 [placed on the Official List of Generic Names in Zoology (ICZN 1995d)]. Comment: Elmidae Curtis, 1830 placed on the Official List of Family-Group Names in Zoology and correct stem ruled to be *Elm*- (ICZN 1995d).
Limniidae Hope, 1838a: 153 [stem: *Limni-*]. Type genus: *Limnius* Illiger, 1802. Comment: family-group name previously attributed to C. G. Thomson (1859) in the literature.

### Subtribe Stenelmina

Mulsant and Rey, 1872

Stenelmisates Mulsant and Rey, 1872a: 49 [stem: *Stenelm-*]. Type genus: *Stenelmis* Dufour, 1835. Comment: original vernacular name available (Art. 11.7.2): first used in latinized form by Bollow (1941: 3, as Stenelmini), generally accepted as in Jäch et al. (2006: 437, as Stenelmina); incorrect original stem formation, not in prevailing usage.

### Tribe Macronychini

Gistel, 1848

Macronychidae Gistel, 1848: [1] [stem: *Macronych-*]. Type genus: *Macronychus* P. W. J. Müller, 1806. Comment: family-group name previously attributed to Mulsant and Rey (1872) in the literature.

### Family Dryopidae

Billberg, 1820 (1817)

Parnidea Leach, 1817: 88 [stem: *Parn-*]. Type genus: *Parnus* Fabricius, 1792 [syn. of *Dryops* A. G. Olivier, 1791]. Comment: younger name Dryopidae Billberg, 1820 conserved (Art. 40.2) (see Lawrence and Newton 1995).
Dryopides Billberg, 1820a: 38 [stem: *Dryop-*]. Type genus: *Dryops* A. G. Olivier, 1791. Comment: younger name conserved over Parnidae Leach, 1817 (Art. 40.2) (see Lawrence and Newton 1995).
Pelonominae Böving and Craighead, 1931: 45, in key [stem: *Pelonom-*]. Type genus: *Pelonomus* Erichson, 1847.
Chiloeidae Dajoz, 1973: 179 [stem: *Chiloe-*]. Type genus: *Chiloea* Dajoz, 1973 [syn. of *Sosteamorphus* Hinton, 1936].

### Family Lutrochidae

Kasap and Crowson, 1975

*Lutrochidae Hinton, 1971: 297 [stem: *Lutroch-*]. Type genus: *Lutrochus* Erichson, 1847. Comment: unavailable family-group name, proposed after 1930 without description or bibliographic reference to such a description (Art. 13.1).
Lutrochidae Kasap and Crowson, 1975: 442 [stem: *Lutroch-*]. Type genus: *Lutrochus* Erichson, 1847.

### Family Limnichidae

Erichson, 1846

Limnichini Erichson, 1846: 465 [stem: *Limnich-*]. Type genus: *Limnichus* Dejean, 1821.

### Subfamily Hyphalinae

Britton, 1971

Hyphalinae Britton, 1971: 88 [stem: *Hyphal-*]. Type genus: *Hyphalus* Britton, 1971.

### Subfamily Limnichinae

Erichson, 1846

Limnichini Erichson, 1846: 465 [stem: *Limnich-*]. Type genus: *Limnichus* Dejean, 1821.

### Tribe Limnichini

Erichson, 1846

Limnichini Erichson, 1846: 465 [stem: *Limnich-*]. Type genus: *Limnichus* Dejean, 1821.
Botriophorates Mulsant and Rey, 1869: 160 [stem: *Bothriophor-*]. Type genus: *Bothriophorus* Mulsant and Rey, 1852 [as *Botriophorus*, incorrect subsequent spelling of type genus name, not in prevailing usage]. Comment: family-group name also spelled Botriaphorates in the original publication (p. 173); original vernacular name available (Art. 11.7.2): first used in latinized form by Ganglbauer (1904: 51, as Bothriophorini), generally accepted as in Shepard (2002: 125, as Bothriophorini); incorrect original stem formation, not in prevailing usage.

### Tribe Wooldridgeini

Spangler, 1999

Wooldridgeini Spangler, 1999: 181 [stem: *Wooldridge-*]. Type genus: *Wooldridgeus* Spangler, 1998.

### Subfamily Cephalobyrrhinae

Champion, 1925

Cephalobyrrhinae Champion, 1925: 174 [stem: *Cephalobyrrh-*]. Type genus: *Cephalobyrrhus* Pic, 1923.

### Subfamily Thaumastodinae

Champion, 1924

Thaumastodinae Champion, 1924: 25 [stem: *Thaumastod-*]. Type genus: *Thaumastodus* Champion, 1924 [syn. of *Pseudeucinetus* Heller, 1921].

### Family Heteroceridae

MacLeay, 1825

Heteroceridae W. S. MacLeay, 1825: 34 [stem: *Heterocer-*]. Type genus: *Heterocerus* Fabricius, 1792.

### Subfamily Elythomerinae

Pacheco, 1964

Elythomerini Pacheco, 1964: 120 [stem: *Elythomer-*]. Type genus: *Elythomerus* C. O. Waterhouse, 1874.

### Subfamily Heterocerinae

MacLeay, 1825

Heteroceridae W. S. MacLeay, 1825: 34 [stem: *Heterocer-*]. Type genus: *Heterocerus* Fabricius, 1792.

### Tribe Augylini

Pacheco, 1964

Augyliini Pacheco, 1964: 19 [stem: *Augyl-*]. Type genus: *Augyles* Schiødte, 1866. Comment: incorrect original stem formation, not in prevailing usage.

### Tribe Heterocerini

MacLeay, 1825

Heteroceridae W. S. MacLeay, 1825: 34 [stem: *Heterocer-*]. Type genus: *Heterocerus* Fabricius, 1792.

### Tribe Micilini

Pacheco, 1964

Micilini Pacheco, 1964: 32 [stem: *Micil-*]. Type genus: *Micilus* Mulsant and Rey, 1872.

### Tribe Tropicini

Pacheco, 1964

Tropicini Pacheco, 1964: 101 [stem: *Tropic-*]. Type genus: *Tropicus* Pacheco, 1964.

### Family Psephenidae

Lacordaire, 1854

Pséphénides Lacordaire, 1854b: 497 [stem: *Psephen-*]. Type genus: *Psephenus* Haldeman, 1853.

### Subfamily Afroeubriinae

Lee, Satô, Shepard and Jäch, 2007

Afroeubriinae Lee, Satô, Shepard and Jäch, 2007: 532 [stem: *Afroeubri-*]. Type genus: *Afroeubria* Villiers, 1961.

### Subfamily Eubriinae

Lacordaire, 1857

Eubriades Lacordaire, 1857: 283 [stem: *Eubri-*]. Type genus: *Eubria* Germar, 1818. Comment: original vernacular name available (Art. 11.7.2): first used in latinized form by J. L. LeConte (1861: 180, as Eubriini), generally accepted as in Hansen (1996: 139, as Eubriinae).

### Subfamily Eubrianacinae

Jakobson, 1913

Placonychini G. H. Horn, 1880a: 110 [stem: *Placonych-*]. Type genus: *Placonycha* G. H. Horn, 1880 [syn. of *Eubrianax* Kiesenwetter, 1874]. Comment: usage of the younger name Eubrianacinae Jakobson, 1913 conserved over this name (Art. 40.2) (see Lawrence and Newton 1995: 846).
Eubrianacini Jakobson, 1913: 723 [stem: *Eubrianac-*]. Type genus: *Eubrianax* Kiesenwetter, 1874. Comment: current spelling maintained (Art. 29.5): incorrect stem formation in prevailing usage (should be *Eubrianact*-); use of this name conserved over Placonychinae G. H. Horn, 1880 (Art. 40.2) (see Lawrence and Newton 1995: 846).

### Subfamily Psephenoidinae

Bollow, 1938

Psephenoidini Bollow, 1938: 156 [stem: *Psephenoid-*]. Type genus: *Psephenoides* Gahan, 1914.

### Subfamily Psepheninae

Lacordaire, 1854

Eurypalpini J. L. LeConte, 1852a: 41 [stem: *Eurypalp-*]. Type genus: *Eurypalpus* J. L. LeConte, 1852 [preoccupied genus name, not *Eurypalpus* Macquart, 1835 [Diptera]; syn. of *Psephenus* Haldeman, 1853]. Comment: permanently invalid (Art. 39): based on preoccupied type genus.
Pséphénides Lacordaire, 1854b: 497 [stem: *Psephen-*]. Type genus: *Psephenus* Haldeman, 1853. Comment: original vernacular name available (Art. 11.7.2): first used in latinized form by Imhoff (1856: xv, as Psephenidae), generally accepted as in Hansen (1996: 139, as Psephenidae).

### Family Cneoglossidae

Champion, 1897

Cneoglossini Champion, 1897: 594 [stem: *Cneogloss-*]. Type genus: *Cneoglossa* Guérin-Méneville, 1843.

### Family Ptilodactylidae

Laporte, 1836

Ptilodactylidae Laporte, 1836: 21 [stem: *Ptilodactyl-*]. Type genus: *Ptilodactyla* Illiger, 1807.

### Subfamily Anchytarsinae

Champion, 1897

Coloboderides Erichson, 1847a: 174 [stem: *Coloboder-*]. Type genus: *Colobodera* Klug, 1838 [syn. of *Daemon* Laporte, 1836].
Anchytarsini Champion, 1897: 593 [stem: *Anchytars-*]. Type genus: *Anchytarsus* Guérin-Méneville, 1843. Comment: Coloboderinae Erichson, 1847 is the oldest name for this tribe, however since Coloboderinae has not been used as valid after 1899 to our knowledge, we believe that usage of Anchytarsinae Champion, 1897 should be conserved and an application be submitted to the Commission to suppress the older name.
*Epilichadinae Lawrence and Stribling, 1992: 19 [stem: *Epilichad-*]. Type genus: *Epilichas* A. White, 1859. Comment: although Ivie (2002: 136) treated this name as available we do not believe that requirements of Art. 13.1 were met and we therefore treat this name here as unavailable.

### Subfamily Cladotominae

Pic, 1914

Cladotomini Pic, 1914: 45 [stem: *Cladotom-*]. Type genus: *Cladotoma* Westwood, 1836.

### Subfamily Aploglossinae

Champion, 1897

Aploglossinae Champion, 1897: 623 [stem: *Aplogloss-*]. Type genus: *Aploglossa* Guérin-Méneville, 1849.

### Subfamily Araeopidiinae

Lawrence, 1991

Araeopidiinae Lawrence, 1991: 250, in key [stem: *Araeopidi-*]. Type genus: *Araeopidius* Cockerell, 1906.

### Subfamily Ptilodactylinae

Laporte, 1836

Ptilodactylidae Laporte, 1836: 21 [stem: *Ptilodactyl-*]. Type genus: *Ptilodactyla* Illiger, 1807.

### Family Podabrocephalidae

Pic, 1930

Podabrocephalidae Pic, 1930: 314 [stem: *Podabrocephal-*]. Type genus: *Podabrocephalus* Pic, 1913.

### Family Chelonariidae

Blanchard, 1845

Chelonariites Blanchard, 1845b: 70 [stem: *Chelonari-*]. Type genus: *Chelonarium* Fabricius, 1801. Comment: original vernacular name available (Art. 11.7.2): first used in latinized form by Imhoff (1856: 140, as Chelonarii), generally accepted as in Satô (2006: 454, as Chelonariidae).

### Family Eulichadidae

Crowson, 1973

*Lichadiden Kolbe, 1908: 249 [stem: *Lichad-*]. Type genus: *Lichas* Westwood, 1853 [preoccupied genus name, not *Lichas* Dalman, 1827 [Trilobita]; syn. of *Eulichas* Jakobson, 1913]. Comment: original vernacular name unavailable (Art. 11.7.2): proposed after 1899.
Lichadidae Forbes, 1926: 102 [stem: *Lichad-*]. Type genus: *Lichas* Westwood, 1853 [preoccupied genus name, not *Lichas* Dalman, 1827 [Trilobita]; syn. of *Eulichas* Jakobson, 1913]. Comment: permanently invalid (Art. 39): based on preoccupied type genus.
Eulichadidae Crowson, 1973b: 237 [stem: *Eulichad-*]. Type genus: *Eulichas* Jakobson, 1913.

### Family Callirhipidae

Emden, 1924

Zenoini J. L. LeConte, 1866a: 50 [stem: *Zeno-*]. Type genus: *Zenoa* Say, 1835. Comment: this name is older than Callirhipidae Emden, 1924 however we recommend that an application be submitted to conserve the younger name.
Callirhipini Emden, 1924: 87 [stem: *Callirhip-*]. Type genus: *Callirhipis* Latreille, 1829. Comment: the oldest name for this family is Zenoidae LeConte, 1866, however, as pointed out by Lawrence and Newton (1995: 849) an application to the Commission is needed in order to preserve the broadly accepted younger name Callirhipidae Emden, 1924; current spelling maintained (Art. 29.3.1.1): incorrect stem formation in prevailing usage (should be *Callirhipid*-).

### Superfamily Elateroidea

Leach, 1815

Elaterides Leach, 1815: 85 [stem: *Elater-*]. Type genus: *Elater* Linnaeus, 1758. Comment: the oldest available name for this superfamily is Cebrionoidea Latreille, 1802, however Elateroidea Leach, 1815 is maintained here pending resolution of an application to the ICZN being prepared by P. J. Johnson (see Lawrence and Newton 1995: 849; P. J. Johnson pers. comm. 2009).

### Family Rhinorhipidae

Lawrence, 1988

Rhinorhipidae Lawrence, 1988: 3 [stem: *Rhinorhip-*]. Type genus: *Rhinorhipus* Lawrence, 1988.

### Family Artematopodidae

Lacordaire, 1857

Artématopides Lacordaire, 1857: 260 [stem: *Artematopod-*]. Type genus: *Artematopus* Perty, 1832.

### Subfamily Electribiinae

Crowson, 1975

Electropogonini Crowson, 1975: 77 [stem: *Electribi-*]. Type genus: *Electribius* Crowson, 1973. Comment: incorrect original stem formation, not in prevailing usage; Electropogonini (based on *Electropogon*) in Crowson’s work has been treated as a *lapsus calami* for Electribiidae based on *Electribius* (see Lawrence and Newton 1995: 850).

### Subfamily Allopogoniinae

Crowson, 1973

Allopogonini Crowson, 1973b: 231, in key [stem: *Allopogoni-*]. Type genus: *Allopogonia* Cockerell, 1906. Comment: incorrect original stem formation, not in prevailing usage.

### Subfamily Artematopodinae

Lacordaire, 1857

Artématopides Lacordaire, 1857: 260 [stem: *Artematopod-*]. Type genus: *Artematopus* Perty, 1832.

### Tribe Artematopodini

Lacordaire, 1857

Artématopides Lacordaire, 1857: 260 [stem: *Artematopod-*]. Type genus: *Artematopus* Perty, 1832. Comment: original vernacular name available (Art. 11.7.2): first used in latinized form by Champion (1897: 586, as Artematopinae [incorrect stem formation]), generally accepted as in Lawrence and Newton (1995: 849, as Artematopodidae); incorrect original stem formation, not in prevailing usage.

### Tribe Ctesibiini

Crowson, 1973

Ctesibiinae Crowson, 1973b: 228, in key [stem: *Ctesibi-*]. Type genus: *Ctesibius* Champion, 1897.

### Tribe Macropogonini

LeConte, 1861

Macropogonini J. L. LeConte, 1861: 178 [stem: *Macropogon-*]. Type genus: *Macropogon* Motschulsky, 1845.
Eurypogonidae Böving and Craighead, 1931: 45, in key [stem: *Eurypogon-*]. Type genus: *Eurypogon* Motschulsky, 1859.

### Family Brachypsectridae

LeConte and Horn, 1883

Brachypsectrini J. L. LeConte and G. H. Horn, 1883: 170 [stem: *Brachypsectr-*]. Type genus: *Brachypsectra* J. L. LeConte, 1874.

### Family Cerophytidae

Latreille, 1834

Cerophytides Latreille, 1834: 133 [stem: *Cerophyt-*]. Type genus: *Cerophytum* Latreille, 1809.

### Family Eucnemidae

Eschscholtz, 1829

Eucnemides Eschscholtz, 1829a: 10 [stem: *Eucnem-*]. Type genus: *Eucnemis* Ahrens, 1812. Comment: the name Melasidae Fleming, 1821 has priority over this name, however Muona and Alaruikka (2007: 32) mentioned that an application has been sent to the Commission to conserve usage of Eucnemidae over the older name Melasidae for reasons of stability, we follow current usage until the case is resolved (also see Appendix 6).

### Subfamily Perothopinae

Lacordaire, 1857

Pérothopides Lacordaire, 1857: 128 [stem: *Perothop-*]. Type genus: *Perothops* Eschscholtz, 1836. Comment: original vernacular name available (Art. 11.7.2): first used in latinized form by J. L. LeConte (1861: 162, as Perothopini), generally accepted as in Lawrence and Newton (1995: 851, as Perothopinae).

### Subfamily Phyllocerinae

Reitter, 1905

Phylloceridae Reitter, 1905: 4 [stem: *Phyllocer-*]. Type genus: *Phyllocerus* Lepeletier and Audinet-Serville, 1825.

### Tribe Anelastini

Reitter, 1911

Anelastini Reitter, 1911: 202 [stem: *Anelast-*]. Type genus: *Anelastes* Kirby, 1819.

### Tribe Phyllocerini

Reitter, 1905

Phylloceridae Reitter, 1905: 4 [stem: *Phyllocer-*]. Type genus: *Phyllocerus* Lepeletier and Audinet-Serville, 1825.

### Subfamily Pseudomeninae

Muona, 1993

Pseudomeninae Muona, 1993: 41 [stem: *Pseudomen-*]. Type genus: *Pseudomenes* Fleutiaux, 1902. Comment: precedence (Pseudomeninae Muona, 1993 vs Schizophilinae Muona, 1993) given to taxon originally proposed at the higher rank (Art. 24.1).

### Tribe Pseudomenini

Muona, 1993

* Pseudomeninae Muona, 1991a: 167 [stem: *Pseudomen-*]. Type genus: *Pseudomenes* Fleutiaux, 1902. Comment: unavailable family-group name, proposed after 1930 without description or bibliographic reference to such a description (Art. 13.1).
Pseudomeninae Muona, 1993: 41 [stem: *Pseudomen-*]. Type genus: *Pseudomenes* Fleutiaux, 1902.

### Tribe Schizophilini

Muona, 1993

*Schizophilini Muona, 1991a: 167 [stem: *Schizophil-*]. Type genus: *Schizophilus* Bonvouloir, 1871. Comment: unavailable family-group name, proposed after 1930 without description or bibliographic reference to such a description (Art. 13.1).
Schizophilini Muona, 1993: 42 [stem: *Schizophil-*]. Type genus: *Schizophilus* Bonvouloir, 1871.

### Subfamily Palaeoxeninae

Muona, 1993

Palaeoxeninae Muona, 1993: 42 [stem: *Palaeoxen-*]. Type genus: *Palaeoxenus* G. H. Horn, 1891.

### Subfamily Phlegoninae

Muona, 1993

Phlegoninae Muona, 1993: 42 [stem: *Phlegon-*]. Type genus: *Phlegon* Laporte, 1840. Comment: current spelling maintained (Art. 29.5): incorrect stem formation in prevailing usage (should be *Phlegont*-).

### Subfamily Anischiinae

Fleutiaux, 1936

Anischinae Fleutiaux, 1936: 292 [stem: *Anischi-*]. Type genus: *Anischia* Fleutiaux, 1896. Comment: incorrect original stem formation, not in prevailing usage.

### Subfamily Melasinae

Fleming, 1821

Melasidae Fleming, 1821: 49 [stem: *Melas-*]. Type genus: *Melasis* A. G. Olivier, 1790.

### Tribe Calyptocerini

Muona, 1993

*Calyptocerini Muona, 1991a: 167 [stem: *Calyptocer-*]. Type genus: *Calyptocerus* Guérin-Méneville, 1843. Comment: unavailable family-group name, proposed after 1930 without description or bibliographic reference to such a description (Art. 13.1).
Calyptocerini Muona, 1993: 43 [stem: *Calyptocer-*]. Type genus: *Calyptocerus* Guérin-Méneville, 1843.

### Tribe Ceballosmelasini

Muona, 1993

*Ceballosmelasini Muona, 1991a: 167 [stem: *Ceballosmelas-*]. Type genus: *Ceballosmelasis* Cobos, 1964. Comment: unavailable family-group name, proposed after 1930 without description or bibliographic reference to such a description (Art. 13.1).
Ceballosmelasini Muona, 1993: 42 [stem: *Ceballosmelas-*]. Type genus: *Ceballosmelasis* Cobos, 1964.

### Tribe Dirhagini

Reitter, 1911

Dirrhagini Reitter, 1911: 202 [stem: *Dirhag-*]. Type genus: *Dirhagus* Latreille, 1834 [as *Dirrhagus*, incorrect subsequent spelling of type genus name, not in prevailing usage; syn. of *Microrhagus* Dejean, 1833]. Comment: incorrect original stem formation, not in prevailing usage.
Microrhaginae Fleutiaux, 1919: 112 [stem: *Microrhag-*]. Type genus: *Microrhagus* Dejean, 1833.
Arhipini Cobos, 1965: 396 [stem: *Arrhipid-*]. Type genus: *Arrhipis* Bonvouloir, 1871 [as *Arhipis*, incorrect subsequent spelling type genus name, not in prevailing usage]. Comment: incorrect original stem formation, not in prevailing usage.

### Tribe Epiphanini

Muona, 1993

*Epiphanini Muona, 1991a: 167 [stem: *Epiphan-*]. Type genus: *Epiphanis* Eschscholtz, 1829. Comment: unavailable family-group name, proposed after 1930 without description or bibliographic reference to such a description (Art. 13.1).
Epiphanini Muona, 1993: 45 [stem: *Epiphan-*]. Type genus: *Epiphanis* Eschscholtz, 1829.

### Tribe Hylocharini

Jacquelin du Val, 1859

Hylocharites Jacquelin du Val, 1859: 119 [stem: *Hylochar-*]. Type genus: *Hylochares* Latreille, 1834. Comment: original vernacular name available (Art. 11.7.2): generally accepted as in Muona (2007: 84, as Hylocharini); family-group names with the incorrectly formed stem *Hylochar*- have been used in Aves (type genus *Hylocharis* Boie, 1831), the correct stem for the bird family-group name is *Hylocharit*- (see Bock 1994: 143).
Hylocharini Cobos, 1965: 369 [stem: *Hylochar-*]. Type genus: *Hylochares* Latreille, 1834. Comment: proposed as new without reference to Hylocharini Jacquelin du Val, 1859.

### Tribe Melasini

Fleming, 1821

Melasidae Fleming, 1821: 49 [stem: *Melas-*]. Type genus: *Melasis* A. G. Olivier, 1790.

### Subtribe Compsocnemina

Muona, 1993

Compsocnemina Muona, 1993: 43 [stem: *Compsocnem-*]. Type genus: *Compsocnemis* Bonvouloir, 1871. Comment: current spelling maintained (Art. 29.3.1.1): incorrect stem formation in prevailing usage (should be *Compsocnemid*-).

### Subtribe Melasina

Fleming, 1821

Melasidae Fleming, 1821: 49 [stem: *Melas-*]. Type genus: *Melasis* A. G. Olivier, 1790.

### Tribe Neocharini

Muona, 1993

*Neocharini Muona, 1991a: 167 [stem: *Neochar-*]. Type genus: *Neocharis* Sharp, 1887. Comment: unavailable family-group name, proposed after 1930 without description or bibliographic reference to such a description (Art. 13.1).
Neocharini Muona, 1993: 44 [stem: *Neochar-*]. Type genus: *Neocharis* Sharp, 1887. Comment: current spelling maintained (Art. 29.5): incorrect stem formation in prevailing usage (should be *Neocharit*-).

### Tribe Xylobiini

Reitter, 1911

Xylobiini Reitter, 1911: 203 [stem: *Xylobi-*]. Type genus: *Xylobius* Latreille, 1834 [syn. of *Xylophilus* Mannerheim, 1823].

### Subfamily Eucneminae

Eschscholtz, 1829

Eucnemides Eschscholtz, 1829a: 10 [stem: *Eucnem-*]. Type genus: *Eucnemis* Ahrens, 1812.

### Tribe Dendrocharini

Fleutiaux, 1920

Dendrocharini Fleutiaux, 1920: 100, in key [stem: *Dendrochar-*]. Type genus: *Dendrocharis* Guérin-Méneville, 1843. Comment: current spelling maintained (Art. 29.5): incorrect stem formation in prevailing usage (should be *Dendrocharit*-).

### Tribe Dyscharachthini

Muona, 1993

*Dyscharachthini Muona, 1991a: 167 [stem: *Dyscharachth-*]. Type genus: *Dyscharachthis* Blackburn, 1900. Comment: unavailable family-group name, proposed after 1930 without description or bibliographic reference to such a description (Art. 13.1).
Dyscharachthini Muona, 1993: 49 [stem: *Dyscharachth-*]. Type genus: *Dyscharachthis* Blackburn, 1900. Comment: current spelling maintained (Art. 29.5): incorrect stem formation in prevailing usage (should be *Dyscharachthent*-).

### Tribe Entomosatopini

Muona, 1993

*Entomosatopini Muona, 1991a: 167 [stem: *Entomosatop-*]. Type genus: *Entomosatopus* Bonvouloir, 1871. Comment: unavailable family-group name, proposed after 1930 without description or bibliographic reference to such a description (Art. 13.1).
Entomosatopini Muona, 1993: 48 [stem: *Entomosatop-*]. Type genus: *Entomosatopus* Bonvouloir, 1871.

### Tribe Eucnemini

Eschscholtz, 1829

Eucnemides Eschscholtz, 1829a: 10 [stem: *Eucnem-*]. Type genus: *Eucnemis* Ahrens, 1812. Comment: current spelling maintained (Art. 29.3.1.1): incorrect stem formation in prevailing usage (should be *Eucnemid*-).
Gastraulaci Fleutiaux, 1902: 648 [stem: *Gastraulac-*]. Type genus: *Gastraulacus* Guérin-Méneville, 1843.

### Tribe Galbitini

Muona, 1991

*Galbites Blanchard, 1845b: 71 [stem: *Galb-*]. Type genus: *Galba* Latreille, 1829 [preoccupied genus name, not *Galba* Schrank, 1803 [Mollusca]; syn. of *Galbites* Fleutiaux, 1918]. Comment: original vernacular name unavailable (Art. 11.7.2): not subsequently latinized; if found to be availabe in the future then permanently invalid (Art. 39): based on preoccupied type genus.
Pterotarsini Cobos, 1965: 294 [stem: *Pterotars-*]. Type genus: *Pterotarsus* sensu Guérin-Méneville, 1838 [syn. of *Galbites* Fleutiaux, 1918]. Comment: proposed as new without reference to Pterotarsini Fleutiaux, 1902 in Elateridae; based on misidentified type genus; name treated here as invalid until an application is submitted to the Commission to suppress it for the Principle of Priority (Art. 65.2.1).
*Galbitini Muona, 1991a: 167 [stem: *Galbit-*]. Type genus: *Galbites* Fleutiaux, 1918. Comment: unavailable family-group name, proposed after 1930 without description or bibliographic reference to such a description (Art. 13.1).
Galbitini Muona, 1991b: 19 [stem: *Galbit-*]. Type genus: *Galbites* Fleutiaux, 1918.

### Tribe Mesogenini

Muona, 1993

*Mesogenini Muona, 1991a: 167 [stem: *Mesogen-*]. Type genus: *Mesogenus* Bonvouloir, 1871. Comment: unavailable family-group name, proposed after 1930 without description or bibliographic reference to such a description (Art. 13.1).
Mesogenini Muona, 1993: 49 [stem: *Mesogen-*]. Type genus: *Mesogenus* Bonvouloir, 1871.

### Tribe Muonajini

Özdikmen, 2008

*Yangini Muona, 1991a: 167 [stem: *Yang-*]. Type genus: *Yanga* Muona, 1993 [preoccupied genus name, not *Yanga* Distant, 1904 [Hemiptera]; syn. of *Muonaja* Özdikmen, 2008]. Comment: unavailable family-group name, proposed after 1930 without description or bibliographic reference to such a description (Art. 13.1).
Yangini Muona, 1993: 48 [stem: *Yang-*]. Type genus: *Yanga* Muona, 1993 [preoccupied genus name, not *Yanga* Distant, 1904 [Hemiptera]; syn. of *Muonaja* Özdikmen, 2008]. Comment: permanently invalid (Art. 39): based on preoccupied type genus.
Muonajini Özdikmen, 2008: 675 [stem: *Muonaj-*]. Type genus: *Muonaja* Özdikmen, 2008. Comment: replacement name for Yangini Muona, 1993 because of the homonymy of the type genus.

### Tribe Perrotiini

Muona, 1993

*Perrotini Muona, 1991a: 167 [stem: *Perroti-*]. Type genus: *Perrotius* Fleutiaux, 1938. Comment: unavailable family-group name, proposed after 1930 without description or bibliographic reference to such a description (Art. 13.1).
Perrotini Muona, 1993: 47 [stem: *Perroti-*]. Type genus: *Perrotius* Fleutiaux, 1938. Comment: incorrect original stem formation, not in prevailing usage.

### Tribe Phaenocerini

Muona, 1993

* Phaenocerini Muona, 1991a: 167 [stem: *Phaenocer-*]. Type genus: *Phaenocerus* Bonvouloir, 1871. Comment: unavailable family-group name, proposed after 1930 without description or bibliographic reference to such a description (Art. 13.1).
Phaenocerini Muona, 1993: 47 [stem: *Phaenocer-*]. Type genus: *Phaenocerus* Bonvouloir, 1871.

### Tribe Proutianini

Muona, 1993

*Proutianini Muona, 1991a: 167 [stem: *Proutian-*]. Type genus: *Proutianus* Muona, 1993. Comment: unavailable family-group name, proposed after 1930 without description or bibliographic reference to such a description (Art. 13.1).
Proutianini Muona, 1993: 47 [stem: *Proutian-*]. Type genus: *Proutianus* Muona, 1993.

### Subfamily Macraulacinae

Fleutiaux, 1923

Macraulacinae Fleutiaux, 1923: 304 [stem: *Micraulac-*]. Type genus: *Macraulacus* Bonvouloir, 1872.

### Tribe Anelastidini

Muona, 1993

Anelastidini Muona, 1993: 52 [stem: *Anelastid-*]. Type genus: *Anelastidius* Jacquelin du Val, 1863. Comment: current spelling maintained (Art. 29.5): incorrect original stem formation in prevailing usage (should be *Anelastidi*-).

### Tribe Echthrogasterini

Cobos, 1965

Echthrogasterini Cobos, 1965: 369 [stem: *Echthrogaster-*]. Type genus: *Echthrogaster* Blackburn, 1900.

### Tribe Euryptychini

Mamaev, 1976

Euryptychini Mamaev, 1976: 154 [stem: *Euryptych-*]. Type genus: *Euryptychus* J. L. LeConte, 1852.

### Tribe Jenibuntorini

Muona, 1993

*Jenibuntorini Muona, 1991a: 167 [stem: *Jenibuntor-*]. Type genus: *Jenibuntor* Muona, 1993. Comment: unavailable family-group name, proposed after 1930 without description or bibliographic reference to such a description (Art. 13.1).
Jenibuntorini Muona, 1993: 51 [stem: *Jenibuntor-*]. Type genus: *Jenibuntor* Muona, 1993.

### Tribe Macraulacini

Fleutiaux, 1923

Macraulacinae Fleutiaux, 1923: 304 [stem: *Macraulac-*]. Type genus: *Macraulacus* Bonvouloir, 1872.
Fornaxini Cobos, 1965: 294 [stem: *Fornac-*]. Type genus: *Fornax* Laporte, 1835. Comment: incorrect original stem formation, not in prevailing usage.
Dromaeolini Leiler, 1976: 48 [stem: *Dromaeol-*]. Type genus: *Dromaeolus* Kiesenwetter, 1858.

### Tribe Nematodini

Leiler, 1976

Nematodini Leiler, 1976: 48 [stem: *Nematod-*]. Type genus: *Nematodes* Berthold, 1827.

### Tribe Oisocerini

Muona, 1993

Oisocerini Muona, 1993: 51 [stem: *Oisocer-*]. Type genus: *Oisocerus* Bonvouloir, 1868.

### Tribe Orodotini

Muona, 1993

Cryptostomidae Laporte, 1835b: 181 [stem: *Cryptostomat-*]. Type genus: *Cryptostoma* Dejean, 1821 [preoccupied genus name, not *Cryptostoma* Blainville, 1818 [Mollusca]; syn. of *Ceratogonys* Perty, 1830]. Comment: permanently invalid (Art. 39): based on preoccupied type genus; incorrect original stem formation, not in prevailing usage.
*Orodotini Muona, 1991a: 167 [stem: *Orodot-*]. Type genus: *Orodotes* Bonvouloir, 1871. Comment: unavailable family-group name, proposed after 1930 without description or bibliographic reference to such a description (Art. 13.1).
Orodotini Muona, 1993: 52 [stem: *Orodot-*]. Type genus: *Orodotes* Bonvouloir, 1871.

### †Tribe Throscogeniini

Iablokoff-Khnzorian, 1962

Throscogeniinae Iablokoff-Khnzorian, 1962: 81 [stem: *Throscogeni-*]. Type genus: *Throscogenius* Iablokoff-Khnzorian, 1962.

### Family Throscidae

Laporte, 1840 nomen protectum

Stereolia Rafinesque, 1815: 112 [stem: *Stereol-*]. Type genus: *Stereolus* Rafinesque, 1815 [unjustified emendation of *Throscus* Latreille, 1797 not in prevailing usage; syn. of *Trixagus* Kugelann, 1794]. Comment: *nomen oblitum* (see Appendix 1).
Throscites Laporte, 1840a: 228 [stem: *Throsc-*]. Type genus: *Throscus* Latreille, 1797 [syn. of *Trixagus* Kugelann, 1794]. Comment: *nomen protectum* (see Appendix 1); original vernacular name available (Art. 11.7.2): first used in latinized form by Agassiz (1846b: 369, as Throscoidae), generally accepted as in P. J. Johnson (2002b: 158, as Throscidae).
Trixagidae Gistel, 1848: [4] [stem: *Trixag-*]. Type genus: *Trixagus* Kugelann, 1794.
Potergini Cobos, 1961: 5 [stem: *Poterg-*]. Type genus: *Potergus* Bonvouloir, 1871.

### †Family Praelateriidae

Dolin, 1973

Praelateriidae Dolin, 1973: 78 [stem: *Praelateri-*]. Type genus: *Praelaterium* Dolin, 1973.

### Family Elateridae

Leach, 1815

Elaterides Leach, 1815: 85 [stem: *Elater-*]. Type genus: *Elater* Linnaeus, 1758. Comment: the oldest available name for this family is Cebrionidae Latreille, 1802, however Elateridae Leach, 1815 is maintained here pending resolution of an application to the ICZN being prepared by P. J. Johnson (see Lawrence and Newton 1995: 852; P. J. Johnson pers. comm. 2009).

### Subfamily Cebrioninae

Latreille, 1802

Cebrionates Latreille, 1802: 97 [stem: *Cebrion-*]. Type genus: *Cebrio* A. G. Olivier, 1790.
Plastocerini J. L. LeConte, 1861: 172 [stem: *Plastocer-*]. Type genus: *Plastocerus* sensu J. L. LeConte, 1853 [not *Plastocerus* Schaum, 1852; syn. of *Octinodes* Candèze, 1863]. Comment: based on a misidentified type genus; an application should be submitted to the Commission to suppress this name for the Principles of Priority and Homonymy (Art. 65.2.1) since Plastoceridae Crowson, 1972 is currently used as valid in Elateroidea.
Aplastinae Stibick, 1979: 175 [stem: *Aplast-*]. Type genus: *Aplastus* J. L. LeConte, 1859.
Cebriognathinae Paulus, 1981: 264, in key [stem: *Cebriognath-*]. Type genus: *Cebriognathus* Chobaut, 1899.

### Subfamily Agrypninae

Candèze, 1857 nomen protectum

Agrypnides Candèze, 1857: 17 [stem: *Agrypn-*]. Type genus: *Agrypnus* Eschscholtz, 1829. Comment: *nomen protectum* (see Appendix 1); Agrypninae Candèze, 1857 given precedence for subfamily name over Oophorinae Gistel, 1848 (Art. 35.5).

### Tribe Agrypnini

Candèze, 1857 nomen protectum

Adeloceraeidae Gistel, 1848: [5] [stem: *Adelocer-*]. Type genus: *Adelocera* Latreille, 1829. Comment: *nomen oblitum* (see Appendix 1); incorrect original stem formation, not in prevailing usage.
Pangauradae Gistel, 1856a: 366 [stem: *Pangaur-*]. Type genus: *Pangaura* Gistel, 1856 [syn. of *Lacon* Laporte, 1838]. Comment: *nomen oblitum* (see Appendix 1); incorrect original stem formation, not in prevailing usage.
Agrypnides Candèze, 1857: 17 [stem: *Agrypn-*]. Type genus: *Agrypnus* Eschscholtz, 1829. Comment: published before 29 June 1857; *nomen protectum* (see Appendix 1); original vernacular name available (Art. 11.7.2): first used in latinized form by Kiesenwetter (1858: 230, as Agrypnini), generally accepted as in Lawrence and Newton (1995: 854, as Agrypninae); this family-group name was also proposed in the same year by Lacordaire (1857 [before 25 May]: 138, as Agrypnides) (see *Bibliographic notes* in Introduction).
*Octocryptites Candèze, 1892: 486 [stem: *Octocrypt-*]. Type genus: *Octocryptus* Candèze, 1892. Comment: original vernacular name unavailable (Art. 11.7.2): subsequently used in latinized form but not generally attributed to Candèze (1892).
Adelocerini Buysson, 1893: 15 [stem: *Adelocer-*]. Type genus: *Adelocera* Latreille, 1829. Comment: family-group name proposed as new without reference to Adeloceridae Gistel, 1848.
Octocryptini Schwarz, 1906: 31 [stem: *Octocrypt-*]. Type genus: *Octocryptus* Candèze, 1892.
Cavicoxumidae Pic, 1928: 21 [stem: *Cavicox-*]. Type genus: *Cavicoxum* Pic, 1928 [syn. of *Agraeus* Candèze, 1857]. Comment: incorrect original stem formation, not in prevailing usage.
Laconini Dajoz, 1964: 60 [stem: *Lacon-*]. Type genus: *Lacon* Laporte, 1838.

### Tribe Anaissini

Golbach, 1984

Alampina C. Costa, 1975: 53 [stem: *Alamp-*]. Type genus: *Alampes* Champion, 1895 [preoccupied genus name, not *Alampes* Horváth, 1884 [Hemiptera]; syn. of *Peralampes* P. J. Johnson, 2002]. Comment: permanently invalid (Art. 39): based on preoccupied type genus.
Anaissini Golbach, 1984: 81 [stem: *Anaiss-*]. Type genus: *Anaissus* Candèze, 1857.

### †Tribe Cryptocardiini

Dolin, 1980

Cryptocardiini Dolin, 1980: 74 [stem: *Cryptocardi-*]. Type genus: *Cryptocardius* Dolin, 1980.

### Tribe Euplinthini

Costa, 1975

Euplinthina C. Costa, 1975: 66 [stem: *Euplinth-*]. Type genus: *Euplinthus* C. Costa, 1975. Comment: we act as First Revisers (Compsoplinthini C. Costa, 1975 vs Euplinthini C. Costa, 1975).

### Subtribe Cleidecostina

Johnson, 2002

Heligmini C. Costa, 1975: 53 [stem: *Heligm-*]. Type genus: *Heligmus* Candèze, 1865 [preoccupied genus name, not *Heligmus* Dujardin, 1845 [Nematoda]; syn. of *Cleidecosta* P. J. Johnson, 2002]. Comment: permanently invalid (Art. 39): based on preoccupied type genus.
Cleidecostini P. J. Johnson, 2002a: 16 [stem: *Cleidecost-*]. Type genus: *Cleidecosta* P. J. Johnson, 2002. Comment: replacement name for Heligmini C. Costa, 1975 because of the homonymy of the type genus.

### Subtribe Compsoplinthina

Costa, 1975

Compsoplinthina C. Costa, 1975: 71 [stem: *Compsoplinth-*]. Type genus: *Compsoplinthus* C. Costa, 1975.

### Subtribe Euplinthina

Costa, 1975

Euplinthina C. Costa, 1975: 66 [stem: *Euplinth-*]. Type genus: *Euplinthus* C. Costa, 1975.

### Tribe Hemirhipini

Candèze, 1857

Hémirhipides Candèze, 1857: 199 [stem: *Hemirhip-*]. Type genus: *Hemirhipus* Berthold, 1827. Comment: published before 29 June; original vernacular name available (Art. 11.7.2): first used in latinized form by J. Thomson (1858: 75, as Hemirhipitae), generally accepted as in P. J. Johnson (2002b: 169, as Hemirhipini); this family-group name was also proposed in the same year by Lacordaire (1857 [before 25 May]: 148, as Hémirhipides) (see *Bibliographic notes* in Introduction); First Reviser (Hemirhipini Candèze, 1857 vs Chalcolepidiini Candèze, 1857) not determined, current usage maintained.
Chalcolépidiides Candèze, 1857: 257 [stem: *Chalcolepidi-*]. Type genus: *Chalcolepidius* Eschscholtz, 1829. Comment: published before 29 June 1857; original vernacular name available (Art. 11.7.2): first used in latinized form by J. L. LeConte (1861: 164, as Chalcolepidiini), generally accepted as in Hyslop (1917: 252, as Chalcolepidina [incorrect stem formation]); this family-group name was also proposed in the same year by Lacordaire (1857 [before 25 May]: 153, as Chalcolépidiides) (see *Bibliographic notes* in Introduction).
Alaites Candèze, 1874: 112 [stem: *Ala-*]. Type genus: *Alaus* Eschscholtz, 1829. Comment: original vernacular name available (Art. 11.7.2): first used in latinized form by Champion (1894: 269, as Alaini), generally accepted as in Heyne and Taschenberg (1905: 152, as Alaini).
Ludioctenina Jakobson, 1913: 755 [stem: *Ludiocten-*]. Type genus: *Ludioctenus* Fairmaire, 1893.
Alauinae Laurent, 1974: 16 [stem: *Ala-*]. Type genus: *Alaus* Eschscholtz, 1829. Comment: family-group name proposed as new without reference to Alaites Candèze, 1874; incorrect original stem formation, not in prevailing usage.

### Tribe Oophorini

Gistel, 1848

Oophoridae Gistel, 1848: [5] [stem: *Oophor-*]. Type genus: *Oophorus* Eschscholtz, 1833 [syn. of *Aeolus* Eschscholtz, 1829]. Comment: family-group name attributed to Gistel (1856a: 367) in recent literature.
Monocrépidiites Candèze, 1859: 176 [stem: *Monocrepidi-*]. Type genus: *Monocrepidius* Eschscholtz, 1829 [syn. of *Conoderus* Eschscholtz, 1829]. Comment: original vernacular name available (Art. 11.7.2): first used in latinized form by J. L. LeConte (1861: 167, as Monocrepidii), generally accepted as in Burakowski et al. (1985: 147, as Monocrepidiinae).
Drasteriini Houlbert, 1912: 184 [stem: *Drasteri-*]. Type genus: *Drasterius* Eschscholtz, 1829 [placed on the Official List of Generic Names in Zoology (ICZN 1987b)]. Comment: the junior homonym Drasteriini Wiltshire, 1976 (type genus *Drasteria* Hübner, 1818) is available in Lepidoptera: Noctuidae; this case is to be referred to the Commission to remove the homonymy (Art. 55.3.1).
*Aeolina Jakobson, 1913: 747 [stem: *Aeol-*]. Type genus: *Aeolus* Eschscholtz, 1829. Comment: family-group name unavailable (Art. 11.6): originally published as synonym and not made available subsequently.
Conoderinae Fleutiaux, 1919: 58 [stem: *Conoder-*]. Type genus: *Conoderus* Eschscholtz, 1829. Comment: the older name Conoderinae Schönherr, 1833 (type genus *Conoderes* Schönherr, 1833) is curently used as valid in Coleoptera: Curculionidae; this case is to be referred to the Commission to remove the homonymy (Art. 55.3.1).
Pachyderinae Fleutiaux, 1919: 57 [stem: *Pachyder-*]. Type genus: *Pachyderes* Guérin-Méneville, 1830.

### Tribe Platycrepidiini

Costa and Casari-Chen, 1993

Eudactylites Candèze, 1859: 153 [stem: *Eudactyl-*]. Type genus: *Eudactylus* Sallé, 1855 [preoccupied genus name, not *Eudactylus* Fitzinger, 1843 [Reptilia]; syn. of *Platycrepidius* Candèze, 1859]. Comment: original vernacular name available (Art. 11.7.2): first used in latinized form by Champion (1895: 337, as Eudactylini), generally accepted as in Hyslop (1917: 259, as Eudactylini); permanently invalid (Art. 39): based on preoccupied type genus.
Platycrepidiini C. Costa and Casari-Chen, 1993: 62 [stem: *Platycrepidi-*]. Type genus: *Platycrepidius* Candèze, 1859.

### Tribe Pseudomelanactini

Arnett, 1967

Pseudomelanactini Arnett, 1967: 111 [stem: *Pseudomelanact-*]. Type genus: *Pseudomelanactes* Mathieu, 1961 [syn. of *Anthracalaus* Fairmaire, 1889].

### Tribe Pyrophorini

Candèze, 1863

Pyrophorites Candèze, 1863: 3 [stem: *Pyrophor-*]. Type genus: *Pyrophorus* Billberg, 1820.

### Subtribe Hapsodrilina

Costa, 1975

Hapsodrilina C. Costa, 1975: 88 [stem: *Hapsodril-*]. Type genus: *Hapsodrilus* C. Costa, 1975.

### Subtribe Nyctophyxina

Costa, 1975

Nyctophyxina C. Costa, 1975: 85 [stem: *Nyctophyx-*]. Type genus: *Nyctophysis* C. Costa, 1975. Comment: current spelling maintained (Art. 29.5): incorrect stem formation in prevailing usage (should be *Nyctophyse*-).

### Subtribe Pyrophorina

Candèze, 1863

Pyrophorites Candèze, 1863: 3 [stem: *Pyrophor-*]. Type genus: *Pyrophorus* Billberg, 1820. Comment: original vernacular name available (Art. 11.7.2): first used in latinized form by Champion (1896: 463, as Pyrophorini), generally accepted as in P. J. Johnson (2002b: 169, as Pyrophorini).

### Tribe Tetralobini

Laporte, 1840

Tetralobites Laporte, 1840a: 230 [stem: *Tetralob-*]. Type genus: *Tetralobus* Lepeletier and Audinet-Serville, 1828. Comment: original vernacular name available (Art. 11.7.2): first used in latinized form by Blanchard (1853: 84, as Tetralobitae), generally accepted as in Lawrence and Newton (1995: 853, as Tetralobinae).
Phyllophoridae Hope, 1842: 73 [stem: *Phyllophor-*]. Type genus: *Phyllophorus* Hope, 1842 [preoccupied genus name, not *Phyllophorus* Grube, 1840 [Echinodermata]; syn. of *Tetralobus* Lepeletier and Audinet-Serville, 1828]. Comment: permanently invalid (Art. 39): based on preoccupied type genus.
Piezophyllini Laurent, 1967: 85 [stem: *Piezophyll-*]. Type genus: *Piezophyllus* Hope, 1842.

### Subfamily Thylacosterninae

Fleutiaux, 1920

Soleniscinae Lameere, 1900: 377 [stem: *Solenisc-*]. Type genus: *Soleniscus* Bonvouloir, 1875 [preoccupied genus name, not *Soleniscus* Meek and Worthen, 1860 [Mollusca]; syn. of *Cussolenis* Fleutiaux, 1918]. Comment: permanently invalid (Art. 39): based on preoccupied type genus.
Pterotarsini Fleutiaux, 1902: 648 [stem: *Pterotars-*]. Type genus: *Pterotarsus* Guérin-Méneville, 1829. Comment: although this name has priority over Thylacosterninae Fleutiaux, 1920, an application will be submitted to the Commission by J. Muona and H. Silfverberg to conserve the well-established younger name.
Thylacosterninae Fleutiaux, 1920: 94 [stem: *Thylacostern-*]. Type genus: *Thylacosternus* Bonvouloir, 1875. Comment: an application will be submitted to the Commission by J. Muona and H. Silfverberg (pers. comm. 2010) to conserve prevailing usage of Thylacosterninae Fleutiaux, 1920 over the older name Pterotarsinae Fleutiaux, 1902.
Balginae Fleutiaux, 1926: 30 [stem: *Balg-*]. Type genus: *Balgus* Fleutiaux, 1920.
Cussolenitae Cobos, 1961: 3 [stem: *Cussolen-*]. Type genus: *Cussolenis* Fleutiaux, 1918.

### Subfamily Lissominae

Laporte, 1835

Lissomidae Laporte, 1835b: 178 [stem: *Lissom-*]. Type genus: *Lissomus* Dalman, 1824.
Drapetini J. L. LeConte, 1863: 44 [stem: *Drapet-*]. Type genus: *Drapetes* Dejean, 1821. Comment: Drapetini Collin, 1961 (type genus *Drapetis* Meigen, 1822) has been used as valid to this day in Diptera although Sabrosky (1999: 118) pointed out that the correct stem for the Diptera name is *Drapetid*-.
Protelateridae Schwarz, 1902: 365 [stem: *Protelater-*]. Type genus: *Protelater* Sharp, 1877.
Oestodini Hyslop, 1917: 251 [stem: *Oestod-*]. Type genus: *Oestodes* J. L. LeConte, 1853.
Athoomorphinae Laurent, 1966: 818 [stem: *Athoomorph-*]. Type genus: *Athoomorphus* Schwarz, 1898.
Drapetini Dolin, 1975b: 1627, in key [stem: *Drapet-*]. Type genus: *Drapetes* Dejean, 1821. Comment: proposed as new without reference to Drapetini J. L. LeConte, 1863.
Sphaenelaterini Stibick, 1979: 179 [stem: *Sphaenelater-*]. Type genus: *Sphaenelater* Schwarz, 1902.

### Subfamily Semiotinae

Jakobson, 1913

Semiotina Jakobson, 1913: 736 [stem: *Semiot-*]. Type genus: *Semiotus* Eschscholtz, 1829.
Semiotinae Golbach, 1970: 320 [stem: *Semiot-*]. Type genus: *Semiotus* Eschscholtz, 1829. Comment: family-group name proposed as new without reference to Semiotina Jakobson, 1913.

### Subfamily Campyloxeninae

Costa, 1975

Campyloxeninae C. Costa, 1975: 114 [stem: *Campyloxen-*]. Type genus: *Campyloxenus* Fairmaire, 1860.

### Subfamily Pityobiinae

Hyslop, 1917

Pityobini Hyslop, 1917: 249 [stem: *Pityobi-*]. Type genus: *Pityobius* J. L. LeConte, 1853. Comment: incorrect original stem formation, not in prevailing usage.
Rostricephalinae Fleutiaux, 1947: 238, in key [stem: *Rostricephal-*]. Type genus: *Rostricephalus* Fleutiaux, 1918.
Adzusini Kishii, 1989: 2 [stem: *Adzus-*]. Type genus: *Adzusa* Kishii, 1957.

### Subfamily Oxynopterinae

Candèze, 1857

Oxynoptérides Candèze, 1857: 355 [stem: *Oxynopter-*]. Type genus: *Oxynopterus* Hope, 1842. Comment: published before 29 June 1857; original vernacular name available (Art. 11.7.2): first used in latinized form by Heyne and Taschenberg (1905: 155, as Oxynopterini), generally accepted as in Johnson (2002b: 169, as Oxynopterini); this family-group name was also proposed in the same year by Lacordaire (1857 [before 25 May]: 158, as Oxynoptérides) (see *Bibliographic notes* in Introduction); First Revisor (Melanactinae Candèze, 1857 vs Oxynopterinae Candèze, 1857) not determined, current usage maintained.
Mélanactides Candèze, 1857: 182 [stem: *Melanact-*]. Type genus: *Melanactes* J. L. LeConte, 1853. Comment: published before 29 June 1857; original vernacular name available (Art. 11.7.2): first used in latinized form by Holmgren (1899: 199, as Melanactidae), generally accepted as in Stibick (1979: 164, as Melanactinae); this family-group name was also proposed in the same year by Lacordaire (1857 [before 25 May]: 144, as Mélanactides) (see *Bibliographic notes* in Introduction).
Asaphites Candèze, 1863: 207 [stem: *Asaph-*]. Type genus: *Asaphes* Kirby, 1837 [preoccupied genus name, not *Asaphes* Walker, 1834 [Hymenoptera]; syn. of *Hemicrepidius* Germar, 1839]. Comment: original vernacular name available (Art. 11.7.2): first used in latinized form and generally accepted as in Heyne and Taschenberg (1905: 163, as Asaphini); permanently invalid (Art. 39): based on preoccupied type genus; the older name Asaphidae H. C. C. Burmeister, 1843 (type genus *Asaphus* Brongniart 1822) is currently used as valid in Trilobita; the younger name Asaphinae Ashmead, 1904 (type genus *Asaphes* Walker, 1834) is currently used as valid in Hymenoptera: Pteromalidae.
Campsosterninae Fleutiaux, 1927a: 104 [stem: *Campsostern-*]. Type genus: *Campsosternus* Latreille, 1834. Comment: published 25 April 1927; this family-group name was also used in the same year by Fleutiaux (1927b [“31 December”]: 108, as Campsosterninae).
Pectocerini Gurjeva, 1974: 107, in key [stem: *Pectocer-*]. Type genus: *Pectocera* Hope, 1842.

### Subfamily Dendrometrinae

Gistel, 1848

Dendrometridae Gistel, 1848: [5] [stem: *Dendrometr-*]. Type genus: *Dendrometrus* Gistel, 1848 [syn. of *Limonius* Eschscholtz, 1829]. Comment: First Reviser found (Denticollinae Stein and Weise, 1877 (1848) vs Dendrometrinae Gistel, 1848) is Sánchez-Ruiz (1996: 74).

### Tribe Crepidomenini

Candèze, 1863

Crépidoménites Candèze, 1863: 190 [stem: *Crepidomen-*]. Type genus: *Crepidomenus* Erichson, 1842. Comment: original vernacular name available (Art. 11.7.2): first used in latinized form by Heyne and Taschenberg (1905: 163, as Crepidomenini), generally accepted as in Calder (1978: 295, as Crepidomeninae).

### Tribe Dendrometrini

Gistel, 1848

Dendrometridae Gistel, 1848: [5] [stem: *Dendrometr-*]. Type genus: *Dendrometrus* Gistel, 1848 [syn. of *Limonius* Eschscholtz, 1829]. Comment: First Reviser found (Denticollini Stein and Weise, 1877 (1848) vs Dendrometrini Gistel, 1848) is Sánchez-Ruiz (1996: 75).

### Subtribe Dendrometrina

Gistel, 1848

Dendrometridae Gistel, 1848: [5] [stem: *Dendrometr-*]. Type genus: *Dendrometrus* Gistel, 1848 [syn. of *Limonius* Eschscholtz, 1829].
Athoites Candèze, 1859: 4 [stem: *Atho-*]. Type genus: *Athous* Eschscholtz, 1829. Comment: original vernacular name available (Art. 11.7.2): first used in latinized form by J. L. LeConte (1861: 170, as Athoi), generally accepted as in Burakowski et al. (1985: 189, as Athoinae).
Limoniina Jakobson, 1913: 755 [stem: *Limoni-*]. Type genus: *Limonius* Eschscholtz, 1829.

### Subtribe Denticollina

Stein and Weise, 1877 (1848)

Campylidae Gistel, 1848: [5] [stem: *Campyl-*]. Type genus: *Campylus* Fischer von Waldheim, 1824 [syn. of *Denticollis* Piller and Mitterpacher, 1783]. Comment: usage of the younger name Denticollina Stein and Weise, 1877 conserved over this name (Art. 40.2).
Denticollini Stein and Weise, 1877: 96 [stem: *Denticoll-*]. Type genus: *Denticollis* Piller and Mitterpacher, 1783. Comment: younger name conserved over Campylina Gistel, 1848 (Art. 40.2).
Lepturoidini Schwarz, 1906: 3, in key [stem: *Lepturoid-*]. Type genus: *Lepturoides* Herbst, 1784 [syn. of *Denticollis* Piller and Mitterpacher, 1783].

### Subtribe Hemicrepidiina

Champion, 1896

Hemicrepidiini Champion, 1896: 477 [stem: *Hemicrepidi-*]. Type genus: *Hemicrepidius* Germar, 1839.

### Tribe Dimini

Candèze, 1863

Dimites Candèze, 1863: 237 [stem: *Dim-*]. Type genus: *Dima* Charpentier, 1825. Comment: original vernacular name available (Art. 11.7.2): first used in latinized form by Champion (1896: 476, as Dimini), generally accepted as in Gurjeva (1974: 107, as Diminae); current spelling maintained (Art. 29.5): incorrect stem formation in prevailing usage (should be *Dimat*-).
Beliophorina Jakobson, 1913: 737 [stem: *Beliophor-*]. Type genus: *Beliophorus* Eschscholtz, 1829.
Peniini Dolin, 1990: 17 [stem: *Peni-*]. Type genus: *Penia* Laporte, 1836.

### Tribe Hypnoidini

Schwarz, 1906 (1860)

Cryptohypnites Candèze, 1860: 50 [stem: *Cryptohypn-*]. Type genus: *Cryptohypnus* Eschscholtz, 1830 [syn. of *Hypnoidus* Dillwyn, 1829]. Comment: original vernacular name available (Art. 11.7.2): first used in latinized form by J. L. LeConte (1861: 166, as Cryptohypni), generally accepted as in Burakowski et al. (1985: 214, as Cryptohypninae); Hypnoidini Schwarz, 1906 conserved over this name (Art. 40.2).
Hypnoidini Schwarz, 1906: 150 [stem: *Hypnoid-*]. Type genus: *Hypnoidus* Dillwyn, 1829. Comment: name proposed to replace Cryptohypnites Candèze, 1860 because of the synonymy of the type genus; younger name conserved over Cryptohypnini Candèze, 1860 (Art. 40.2).
Hypolithinae Fleutiaux, 1928: 252 [stem: *Hypolith-*]. Type genus: *Hypolithus* Eschscholtz, 1829. Comment: name proposed to replace Cryptohypnites Candèze, 1860 because of the synonymy of the type genus.
*Prisahypini Stibick, 1976: 197 [stem: *Prisahypn-*]. Type genus: *Prisahypnus* Stibick, 1979. Comment: unavailable family-group name, not based on available genus name; incorrect original stem formation, not in prevailing usage.
Prisahypini Stibick, 1979: 166 [stem: *Prisahypn-*]. Type genus: *Prisahypnus* Stibick, 1979. Comment: incorrect original stem formation, not in prevailing usage.

### Tribe Pleonomini

Semenov and Pjatakova, 1936

Pleonomini Semenov and Pjatakova, 1936: 103 [stem: *Pleonom-*]. Type genus: *Pleonomus* Ménétriés, 1849. Comment: name proposed after 1930 without description or bibliographic reference to such a description (Art. 13.1), however available because it was used as valid before 2000 as in Stibick (1979: 176) and was not rejected by an author who, between 1961 and 1999, applied Article 13 of the then current edition of the Code (see Art. 13.2.1).

### Tribe Prosternini

Gistel, 1856 nomen protectum

Diacanthidae Gistel, 1848: [5] [stem: *Diacanth-*]. Type genus: *Diacanthus* Latreille, 1834 [syn. of *Selatosomus* Stephens, 1830]. Comment: *nomen oblitum* (see Appendix 1).
Prosternidae Gistel, 1856a: 367 [stem: *Prostern-*]. Type genus: *Prosternon* Latreille, 1834. Comment: *nomen protectum* (see Appendix 1).
Corymbitini J. L. LeConte, 1861: 169 [stem: *Corymbit-*]. Type genus: *Corymbites* Latreille, 1834 [syn. of *Ctenicera* Latreille, 1829].
Ctenicerina Jakobson, 1913: 736 [stem: *Ctenicer-*]. Type genus: *Ctenicera* Latreille, 1829. Comment: family-group name previously attributed to Fleutiaux (1936) in the literature; “Ctenicerini Fleutiaux, 1936” used as valid instead of Prosternini Gistel, 1856 by Cate (2007: 33, 173).
Ctenicerinae Neboiss, 1956: 47 [stem: *Ctenicer-*]. Type genus: *Ctenicera* Latreille, 1829. Comment: name proposed to replace Corymbitinae J. L. LeConte, 1861 because of the synonymy of the type genus; family-group name proposed as new without reference to Ctenicerina Jakobson, 1913 or Ctenicerinae Fleutiaux, 1936.

### Tribe Senodoniini

Schenkling, 1927

Allotriites Candèze, 1863: 225 [stem: *Allotri-*]. Type genus: *Allotrius* Laporte, 1840 [preoccupied genus name, not *Allotrius* Temminck, 1835 [Aves]; syn. of *Senodonia* Laporte, 1838]. Comment: original vernacular name available (Art. 11.7.2): first used in latinized form by Champion (1896: 489, as Allotriini), generally accepted as in Heyne and Taschenberg (1905: 163, as Allotriini); permanently invalid (Art. 39): based on preoccupied type genus.
Senodoniinae Schenkling, 1927: 417 [stem: *Senodoni-*]. Type genus: *Senodonia* Laporte, 1838.
Senodoniini Dolin, 2000: 19 [stem: *Senodoni-*]. Type genus: *Senodonia* Laporte, 1838. Comment: family-group name proposed as new without reference to Senodoniinae Schenkling, 1927.

### Subfamily Negastriinae

Nakane and Kishii, 1956

Negastriinae Nakane and Kishii, 1956: 203 [stem: *Negastri-*]. Type genus: *Negastrius* C. G. Thomson, 1859.

### Tribe Negastriini

Nakane and Kishii, 1956

Negastriinae Nakane and Kishii, 1956: 203 [stem: *Negastri-*]. Type genus: *Negastrius* C. G. Thomson, 1859.

### Tribe Quasimusini

Schimmel and Tarnawski, 2009

Quasimusini Schimmel and Tarnawski, 2009: 17 [stem: *Quasimus*-]. Type genus: *Quasimus* Gozis, 1886.

### Subtribe Loebliquasimusina

Schimmel and Tarnawski, 2009

Loebliquasina Schimmel and Tarnawski, 2009: 18 [stem: *Loebliquas*-]. Type genus: *Loebliquasis* Dolin, 1997. Comment: incorrect original stem formation, not in prevailing usage.

### Subtribe Quasimusina

Schimmel and Tarnawski, 2009

Quasimusina Schimmel and Tarnawski, 2009: 19 [stem: *Quasimus*-]. Type genus: *Quasimus* Gozis, 1886.

### Subtribe Striatoquasimusina

Schimmel and Tarnawski, 2009

Striatoquasina Schimmel and Tarnawski, 2009: 20 [stem: *Striatoquasimus*-]. Type genus: *Striatoquasimus* Schimmel and Tarnawski, 2009. Comment: incorrect original stem formation, not in prevailing usage.

### Subtribe Wittmeroquasimusina

Schimmel and Tarnawski, 2009

Wittmeroquasina Schimmel and Tarnawski, 2009: 20 [stem: *Wittmeroquasimus*-]. Type genus: *Wittmeroquasimus* Dolin, 1997. Comment: incorrect original stem formation, not in prevailing usage.

### Subfamily Elaterinae

Leach, 1815

Elaterides Leach, 1815: 85 [stem: *Elater-*]. Type genus: *Elater* Linnaeus, 1758.

### Tribe Agriotini

Laporte, 1840

Agriotites Laporte, 1840a: 233 [stem: *Agriot-*]. Type genus: *Agriotes* Eschscholtz, 1829.

### Subtribe Agriotina

Laporte, 1840

Agriotites Laporte, 1840a: 233 [stem: *Agriot-*]. Type genus: *Agriotes* Eschscholtz, 1829. Comment: original vernacular name available (Art. 11.7.2): first used in latinized form by Champion (1896: 511, as Agriotini), generally accepted as in Hansen (1996: 143, as Agriotini); current spelling maintained (Art. 29.5): incorrect stem formation in prevailing usage (should be *Agriotet*-).

### Subtribe Cardiorhinina

Candèze, 1863

Cardiorhinites Candèze, 1863: 247 [stem: *Cardiorhin-*]. Type genus: *Cardiorhinus* Eschscholtz, 1829. Comment: original vernacular name available (Art. 11.7.2): first used in latinized form by Champion (1896: 495, as Cardiorhini), generally accepted as in Heyne and Taschenberg (1905: 164, as Cardiorhinini).

### Tribe Ampedini

Gistel, 1848

Ampedidae Gistel, 1848: [5] [stem: *Amped-*]. Type genus: *Ampedus* Dejean, 1833. Comment: family-group name previously attributed to Gistel (1856a: 367), e.g., P. J. Johnson (2002b: 168).

### Tribe Dicrepidiini

Thomson, 1858

Dicrepidiitae J. Thomson, 1858: 75 [stem: *Dicrepidi-*]. Type genus: *Dicrepidius* Eschscholtz, 1829.

### Tribe Elaterini

Leach, 1815

Elaterides Leach, 1815: 85 [stem: *Elater-*]. Type genus: *Elater* Linnaeus, 1758.
Arneidae Gistel, 1848: [6] [stem: *Arne-*]. Type genus: *Arneus* Gistel, 1848 [syn. of *Sericus* Eschscholtz, 1829].
Steatoderidae Gistel, 1848: [5] [stem: *Steatoder-*]. Type genus: *Steatoderus* Dejean, 1833 [syn. of *Elater* Linnaeus, 1758].
Amphilabridae Gistel, 1856a: 367 [stem: *Amphilabr-*]. Type genus: *Amphilabris* Gistel, 1834 [syn. of *Sericus* Eschscholtz, 1829].
Ludiides Lacordaire, 1857: 197 [stem: *Ludi-*]. Type genus: *Ludius* Berthold, 1827 [syn. of *Elater* Linnaeus, 1758]. Comment: original vernacular name available (Art. 11.7.2): first used in latinized form and generally accepted as in J. L. LeConte (1861: 168, as Ludii); the junior homonym Ludiini Aurivillius, 1904 (type genus *Ludia* Wallengren, 1865) in Lepidoptera: Saturniidae was replaced by Micragonini Cockerell, 1914 (see Oberprieler 1997: 146).
Hypodésites Candèze, 1863: 242 [stem: *Hypodese-*]. Type genus: *Hypodesis* Latreille, 1834. Comment: original vernacular name available (Art. 11.7.2): first used in latinized form by Champion (1896: 490, as Hypodesini), generally accepted as in Heyne and Taschenberg (1905: 164, as Hypodesini); incorrect original stem formation, not in prevailing usage.
Sericosomina Hyslop, 1917: 258 [stem: *Sericosom-*]. Type genus: *Sericosomus* Dejean, 1833 [syn. of *Sericus* Eschscholtz, 1829].
*Dolerosomini Dolin, 1975b: 1632 [stem: *Dolerosom-*]. Type genus: *Dolerosomus* Motschulsky, 1859. Comment: unavailable family-group name, proposed after 1930 without description or bibliographic reference to such a description (Art. 13.1).

### Tribe Megapenthini

Gurjeva, 1973

Megapenthini Gurjeva, 1973: 448 [stem: *Megapenth-*]. Type genus: *Megapenthes* Kiesenwetter, 1858.

### Tribe Melanotini

Candèze, 1859 (1848)

Cratonychidae Gistel, 1848: [5] [stem: *Cratonych-*]. Type genus: *Cratonychus* Dejean, 1833 [syn. of *Melanotus* Eschscholtz, 1829]. Comment: use of Melanotini Candèze, 1859 converved over this name (Art. 40.2).
Mélanotites Candèze, 1859: 4 [stem: *Melanot-*]. Type genus: *Melanotus* Eschscholtz, 1829. Comment: original vernacular name available (Art. 11.7.2): first used in latinized form by J. L. LeConte (1861: 169, as Melanoti), generally accepted as in Hansen (1996: 143, as Melanotini); name conserved over Cratonychini Gistel, 1848 (Art. 40.2) (see Sánchez-Ruiz 1996: 167).

### Tribe Odontonychini

Girard, 1973

Odontonychini Girard, 1973: 276 [stem: *Odontonych-*]. Type genus: *Odontonychus* Candèze, 1897.

### Tribe Physorhinini

Candèze, 1859

Physorhinites Candèze, 1859: 384 [stem: *Physorhin-*]. Type genus: *Physorhinus* Germar, 1840. Comment: original vernacular name available (Art. 11.7.2): first used in latinized form by J. L. LeConte (1861: 167, as Physorhini), generally accepted as in Burakowski et al. (1985: 114, as Physorhininae).

### Tribe Pomachiliini

Candèze, 1859

Pomachiliites Candèze, 1859: 4 [stem: *Pomachili-*]. Type genus: *Pomachilius* Eschscholtz, 1829. Comment: original vernacular name available (Art. 11.7.2): first used in latinized form by Champion (1895: 402, as Pomachiliini), generally accepted as in Burakowski et al. (1985: 131, as Pomachiliinae).

### Tribe Synaptini

Gistel, 1856

Dairaeidae Gistel, 1848: [5] [stem: *Dair-*]. Type genus: *Daira* Gistel, 1848 [preoccupied genus name, not *Daira* Milne-Edwards, 1830 [Crustacea]; syn. of *Synaptus* Eschscholtz, 1829]. Comment: permanently invalid (Art. 39): based on preoccupied type genus; incorrect original stem formation, not in prevailing usage.
Synaptidae Gistel, 1856a: 366 [stem: *Synapt-*]. Type genus: *Synaptus* Eschscholtz, 1829.
Adrastites Candèze, 1863: 448 [stem: *Adrast-*]. Type genus: *Adrastus* Eschscholtz, 1829. Comment: original vernacular name available (Art. 11.7.2): first used in latinized form by Champion (1896: 533, as Adrastini), generally accepted as in Burakowski et al. (1985: 133, as Adrastinae).

### Subfamily Cardiophorinae

Candèze, 1859

Cardiophorites Candèze, 1859: 4 [stem: *Cardiophor-*]. Type genus: *Cardiophorus* Eschscholtz, 1829. Comment: original vernacular name available (Art. 11.7.2): first used in latinized form by J. L. LeConte (1861: 166, as Cardiophori), generally accepted as in Burakowski et al. (1985: 227, as Cardiophorinae).
Aphrici J. L. LeConte, 1861: 173 [stem: *Aphric-*]. Type genus: *Aphricus* J. L. LeConte, 1854.
Aptopina Jakobson, 1913: 760 [stem: *Aptopod-*]. Type genus: *Aptopus* Eschscholtz, 1829. Comment: incorrect original stem formation, not in prevailing usage.
Esthesopinae Fleutiaux, 1919: 76 [stem: *Esthesopod-*]. Type genus: *Esthesopus* Eschscholtz, 1829. Comment: incorrect original stem formation, not in prevailing usage.
Nyctorini Semenov and Pjatakova, 1936: 102 [stem: *Nyctor-*]. Type genus: *Nyctor* Semenov and Pjatakova, 1936 [syn. of *Cardiophorus* Eschscholtz, 1829].

### Subfamily Hemiopinae

Fleutiaux, 1941

Hemiopinae Fleutiaux, 1941: 31 [stem: *Hemiop-*]. Type genus: *Hemiops* Laporte, 1838.

### Subfamily Physodactylinae

Lacordaire, 1857

Physodactylides Lacordaire, 1857: 236 [stem: *Physodactyl-*]. Type genus: *Physodactylus* Fischer von Waldheim, 1823. Comment: original vernacular name available (Art. 11.7.2): first used in latinized form by Fleutiaux (1892b: 404, as Physodactylini), generally accepted as in Lawrence and Newton (1995: 855, as Physodactylinae).
Toxognathinae Fleutiaux, 1941: 34 [stem: *Toxognath-*]. Type genus: *Toxognathus* Fairmaire, 1878.

### Subfamily Eudicronychinae

Girard, 1971

Dicronychidae Schwarz, 1897: 11 [stem: *Dicronych-*]. Type genus: *Dicronychus* Laporte, 1840 [preoccupied genus name, not *Dicronychus* Brullé, 1832 [Coleoptera: Elateridae: Cardiophorinae]; syn. of *Eudicronychus* Méquignon, 1931]. Comment: permanently invalid (Art. 39): based on preoccupied type genus.
Eudicronychinae Girard, 1971: 645 [stem: *Eudicronych-*]. Type genus: *Eudicronychus* Méquignon, 1931. Comment: description by indication (distinguishing characters given in Schwarz (1907: 2, as Dicronychidae)).

### Subfamily Subprotelaterinae

Fleutiaux, 1920

Subprotelaterinae Fleutiaux, 1920: 99 [stem: *Subprotelater-*]. Type genus: *Subprotelater* Fleutiaux, 1916.

### Subfamily Morostomatinae

Dolin, 2000

Morostominae Dolin, 2000: 18 [stem: *Morostomat-*]. Type genus: *Morostoma* Candèze, 1879. Comment: incorrect original stem formation, not in prevailing usage.

### †Subfamily Protagrypninae

Dolin, 1973

Protagrypnini Dolin, 1973: 74 [stem: *Protagrypn-*]. Type genus: *Protagrypnus* Dolin, 1973.

### †Tribe Desmatini

Dolin, 1975

Desmatini Dolin, 1975a: 60 [stem: *Desmat-*]. Type genus: *Desmatus* Dolin, 1975.

### †Tribe Hypnomorphini

Dolin, 1975

Hypnomorphini Dolin, 1975a: 54 [stem: *Hypnomorph-*]. Type genus: *Hypnomorphus* Dolin, 1975.

### †Tribe Protagrypnini

Dolin, 1973

Protagrypnini Dolin, 1973: 74 [stem: *Protagrypn-*]. Type genus: *Protagrypnus* Dolin, 1973.

### Family Plastoceridae

Crowson, 1972

Plastoceridae Crowson, 1972: 37, in key [stem: *Plastocer-*]. Type genus: *Plastocerus* Schaum, 1852. Comment: an application needs to be submitted to the Commission to suppress Plastoceri J. L. LeConte, 1861 (based on the misidentified type genus *Plastocerus* sensu J. L. LeConte, 1853) for the Principles of Priority and Homonymy (Art. 65.2.1) to conserve this name as valid.

### Family Drilidae

Blanchard, 1845

Drilites Blanchard, 1845b: 53 [stem: *Dril-*]. Type genus: *Drilus* A. G. Olivier, 1790.

### Subfamily Drilinae

Blanchard, 1845

Drilites Blanchard, 1845b: 53 [stem: *Dril-*]. Type genus: *Drilus* A. G. Olivier, 1790. Comment: original vernacular name available (Art. 11.7.2): first used in latinized form by Gistel (1848: [11], as Drilidae), generally accepted as in Hansen (1996: 144, as Drilidae).

### Subfamily Thilmaninae

Kazantsev, 2004

Thilmaninae Kazantsev, 2004b: 240 [stem: *Thilman-*]. Type genus: *Thilmanus* Baudi di Selve, 1871. Comment: transferred from Lycidae by Bocák and Brlik (2008).

### Tribe Euanomini

Kazantsev, 2010

Euanomini Kazantsev, 2010a: 55 [stem: *Euanom-*]. Type genus: *Euanoma* Reitter, 1889.

### Tribe Thilmanini

Kazantsev, 2004

Thilmaninae Kazantsev, 2004b: 240 [stem: *Thilman-*]. Type genus: *Thilmanus* Baudi di Selve, 1871.

### Family Omalisidae

Lacordaire, 1857

*Homalises Motschulsky, 1849: 55 [stem: *Omalis-*]. Type genus: *Omalisus* Geoffroy, 1762 [as *Homalisus*, unjustified emendation of type genus name by Illiger (1801: 139), not in prevailing usage; placed on the Official List of Generic Names in Zoology (ICZN 1994a)]. Comment: original vernacular name unavailable (Art. 11.7.2): subsequently used in latinized form but not generally attributed to Motschulsky (1849).
Homalisides Lacordaire, 1857: 303 [stem: *Omalis-*]. Type genus: *Omalisus* Geoffroy, 1762 [as *Homalisus*, unjustified emendation of type genus name by Illiger (1801: 139), not in prevailing usage; placed on the Official List of Generic Names in Zoology (ICZN 1994a)]. Comment: original vernacular name available (Art. 11.7.2): first used in latinized form by Kiesenwetter (1860: 442, as Homalisidae [incorrect stem formation]), generally accepted as in Lawrence and Newton (1995: 856, as Omalisidae); incorrect original stem formation, not in prevailing usage.

### †Family Berendtimiridae

Winkler, 1987

Berendtimiridae J. R. Winkler, 1987: 52 [stem: *Berendtimir-*]. Type genus: *Berendtimirus* J. R. Winkler, 1987.

### Family Lycidae

Laporte, 1836

Lycusidae Laporte, 1836: 25 [stem: *Lyc-*]. Type genus: *Lycus* Fabricius, 1787.

### Subfamily Libnetinae

Bocák and Bocáková, 1990

Libnetinina Bocák and Bocáková, 1990: 652 [stem: *Libnet-*]. Type genus: *Libnetis* C. O. Waterhouse, 1878. Comment: incorrect original stem formation, not in prevailing usage.

### Subfamily Dictyopterinae

Houlbert, 1922

Dictyopterini Houlbert, 1922a: 338 [stem: *Dictyopter-*]. Type genus: *Dictyoptera* Latreille, 1829.

### Tribe Dictyopterini

Houlbert, 1922

Dictyopterini Houlbert, 1922a: 338 [stem: *Dictyopter-*]. Type genus: *Dictyoptera* Latreille, 1829. Comment: family-group name previously attributed to Kleine (1929: 226); although Houlbert (1922a) uses the tribe name Lycini for a group of genera that includes *Dictyoptera* Latreille in the main text (page 240), this was certainly a mistake since the tribe name Dictyopterini is correctly used in the “Index alphabétique” (page 319) and in the “Table systématique” (page 338).

### Tribe Lycoprogenthini

Bocák and Bocáková, 2008

Lycoprogenthini Bocák and Bocáková, 2008: 709 [stem: *Lycoprogenth-*]. Type genus: *Lycoprogenthes* Pic, 1915.

### Tribe Taphini

Bocák and Bocáková, 1990

Taphinina Bocák and Bocáková, 1990: 650 [stem: *Taph-*]. Type genus: *Taphes* C. O. Waterhouse, 1878. Comment: incorrect original stem formation, not in prevailing usage.

### Subfamily Lyropaeinae

Bocák and Bocáková, 1989

Lyropaeini Bocák and Bocáková, 1989: 718 [stem: *Lyropae-*]. Type genus: *Lyropaeus* C. O. Waterhouse, 1878.

### Tribe Alyculini

Bocák and Bocáková, 2008

Alyculini Bocák and Bocáková, 2008: 710 [stem: *Alycul-*]. Type genus: *Alyculus* Kazantsev, 1999.

### Tribe Antennolycini

Bocák and Bocáková, 2008

Antennolycini Bocák and Bocáková, 2008: 710 [stem: *Antennolyc-*]. Type genus: *Antennolycus* Bocák and Bocáková, 1999.

### Tribe Lyropaeini

Bocák and Bocáková, 1989

Lyropaeini Bocák and Bocáková, 1989: 718 [stem: *Lyropae-*]. Type genus: *Lyropaeus* C. O. Waterhouse, 1878.
Paralycinae L. N. Medvedev and Kazantsev, 1992: 59 [stem: *Paralyc-*]. Type genus: *Paralycus* L. N. Medvedev and Kazantsev, 1992 [syn. of *Lyropaeus* C. O. Waterhouse, 1878].

### Tribe Miniduliticolini

Kazantsev, 2003

Miniduliticolini Kazantsev, 2003: 20 [stem: *Miniduliticol-*]. Type genus: *Miniduliticola* Kazantsev, 2003.

### Tribe Platerodrilini

Kazantsev, 2004

*Duliticolinae Mjöberg, 1925: 140 [stem: *Duliticol-*]. Type genus: *Duliticola* Mjöberg, 1925 [syn. of *Platerodrilus* Pic, 1921]. Comment: unavailable family-group name (Art. 8.3): nomenclatural act disclaimed.
*Duliticolinae Kazantsev, 2003: 19 [stem: *Duliticol-*]. Type genus: *Duliticola* Mjöberg, 1925 [syn. of *Platerodrilus* Pic, 1921]. Comment: family-group name proposed as a new taxon but unavailable (Art. 11.7.1.1) because it was based on a type genus which was considered a synonym of *Platerodrilus* Pic, 1921 by the author.
Platerodrilini Kazantsev, 2004b: 241 [stem: *Platerodril-*]. Type genus: *Platerodrilus* Pic, 1921.

### Subfamily Ateliinae

Kleine, 1929

Atelinae Kleine, 1929: 222 [stem: *Ateli-*]. Type genus: *Atelius* C. O. Waterhouse, 1878. Comment: First Revisers found (Ateliinae Kleine, 1928 vs Dilophotinae Kleine, 1928) are Bocák and Bocáková (2008: 712).

### Tribe Ateliini

Kleine, 1929

Atelinae Kleine, 1929: 222 [stem: *Ateli-*]. Type genus: *Atelius* C. O. Waterhouse, 1878. Comment: incorrect original stem formation, not in prevailing usage.

### Tribe Dilophotini

Kleine, 1929

Dilophotinae Kleine, 1929: 222 [stem: *Dilophot-*]. Type genus: *Dilophotes* C. O. Waterhouse, 1879. Comment: current spelling maintained (Art. 29.5): incorrect stem formation in prevailing usage (should be *Dilophotet*-).

### Subfamily Lycinae

Laporte, 1836

Lycusidae Laporte, 1836: 25 [stem: *Lyc-*]. Type genus: *Lycus* Fabricius, 1787.

### Tribe Calochromini

Lacordaire, 1857

Calochromides Lacordaire, 1857: 301 [stem: *Calochrom-*]. Type genus: *Calochromus* Guérin-Méneville, 1833. Comment: original vernacular name available (Art. 11.7.2): first used in latinized form by Gorham (1880: 1, as Calochrominae), generally accepted as in Hansen (1996: 144, as Calochrominae).
Lygistopteri J. L. LeConte, 1881: 27 [stem: *Lygistopter-*]. Type genus: *Lygistopterus* Dejean, 1833.

### Tribe Calopterini

Green, 1949

Calopterini Green, 1949: 56, in key [stem: *Calopter-*]. Type genus: *Calopteron* Laporte, 1838.

### Subtribe Acroleptina

Bocáková, 2005

Acroleptina Bocáková, 2005: 445 [stem: *Acrolept-*]. Type genus: *Acroleptus* Bourgeois, 1886.

### Subtribe Calopterina

Green, 1949

*Calopterini Kleine, 1933: 20 [stem: *Calopter-*]. Type genus: *Calopteron* Laporte, 1838. Comment: unavailable family-group name, proposed after 1930 without description or bibliographic reference to such a description (Art. 13.1).
Calopterini Green, 1949: 56, in key [stem: *Calopter-*]. Type genus: *Calopteron* Laporte, 1838.

### Tribe Conderini

Bocák and Bocáková, 1990

Conderini Bocák and Bocáková, 1990: 643 [stem: *Conder-*]. Type genus: *Conderis* C. O. Waterhouse, 1879. Comment: current spelling maintained (Art. 29.5): incorrect stem formation in prevailing usage (should be *Conderid*-).

### Tribe Dihammatini

Bocák and Bocáková, 2008

Dihammatini Bocák and Bocáková, 2008: 716 [stem: *Dihammat-*]. Type genus: *Dihammatus* C. O. Waterhouse, 1879.

### Tribe Erotini

LeConte, 1881

Erotes J. L. LeConte, 1881: 23 [stem: *Erot-*]. Type genus: *Eros* Newman, 1838.
Flagraxina Kazantsev, 2004a: 35, in key [stem: *Flagrax-*]. Type genus: *Flagrax* Kazantsev, 1992.
Aferotini Kazantsev, 2004a: 4 [stem: *Aferot-*]. Type genus: *Aferos* Kazantsev, 1992.

### Tribe Eurrhacini

Bocáková, 2005

Eurrhacina Bocáková, 2005: 444 [stem: *Eurrhac-*]. Type genus: *Eurrhacus* C. O. Waterhouse, 1879.

### Tribe Leptolycini

Leng and Mutchler, 1922

Leptolycini Leng and Mutchler, 1922: 430 [stem: *Leptolyc-*]. Type genus: *Leptolycus* Leng and Mutchler, 1922.

### Tribe Lycini

Laporte, 1836

Lycusidae Laporte, 1836: 25 [stem: *Lyc-*]. Type genus: *Lycus* Fabricius, 1787. Comment: incorrect original stem formation, not in prevailing usage.

### Tribe Lyponiini

Bocák and Bocáková, 1990

Lyponiinina Bocák and Bocáková, 1990: 652 [stem: *Lyponi-*]. Type genus: *Lyponia* C. O. Waterhouse, 1878. Comment: incorrect original stem formation, not in prevailing usage.

### Tribe Macrolycini

Kleine, 1929

Macrolycinae Kleine, 1929: 222 [stem: *Macrolyc-*]. Type genus: *Macrolycus* C. O. Waterhouse, 1878.

### Tribe Melanerotini

Kazantsev, 2010

Melanerotini Kazantsev, 2010b: 195 [stem: *Melanerot-*]. Type genus: *Melaneros* Fairmaire, 1877.

### Tribe Metriorrhynchini

Kleine, 1926

Metriorrhynchinae Kleine, 1926: 97 [stem: *Metriorrhynch-*]. Type genus: *Metriorrhynchus* Guérin-Méneville, 1838.

### Subtribe Hemiconderinina

Bocák and Bocáková, 1990

Hemiconderinina Bocák and Bocáková, 1990: 645 [stem: *Hemiconderin-*]. Type genus: *Hemiconderis* Kleine, 1926. Comment: current spelling maintained (Art. 29.5): incorrect stem formation in prevailing usage (should be *Hemiconderid*-).

### Subtribe Metriorrhynchina

Kleine, 1926

Metriorrhynchinae Kleine, 1926: 97 [stem: *Metriorrhynch-*]. Type genus: *Metriorrhynchus* Guérin-Méneville, 1838 [*Metriorrhynchus* is an incorrect subsequent spelling by Gemminger (1869: 1629) of the original name *Metriorhynchus* in prevailing usage and so deemed to be the correct original spelling (Art. 33.3.1)]. Comment: First Reviser (Metriorrhynchina Kleine, 1926 vs Dilolycina Kleine, 1926) not determined, current usage maintained.
Dilolycinae Kleine, 1926: 186 [stem: *Dilolyc-*]. Type genus: *Dilolycus* Kleine, 1926. Comment: one of the original spellings of this family-group name was Haplothoracinae on page 95 (based on the new genus *Haplothorax* Kleine), the spelling of the family-group and genus-group names were changed to Dilolycinae and *Dilolycus* on page 186 and in the “Corrigenda” on page 188 of the same work because *Haplothorax* Agassiz, 1846 (unjustified emendation of *Aplothorax* G. R. Waterhouse, 1841) was already available in Carabidae; Dilolycinae is therefore treated as the correct original spelling of this family-group name (Art. 19.2).
Cladophorinae Kleine, 1929: 222 [stem: *Cladophor-*]. Type genus: *Cladophorus* Guérin-Méneville, 1830.

### Subtribe Trichalina

Kleine, 1929

Trichalinae Kleine, 1929: 222 [stem: *Trichal-*]. Type genus: *Trichalus* C. O. Waterhouse, 1877.

### Tribe Platerodini

Kleine, 1929

Platerodinae Kleine, 1929: 222 [stem: *Platerod-*]. Type genus: *Plateros* Bourgeois, 1879. Comment: current spelling maintained (Art. 29.5): incorrect stem formation in prevailing usage (should be *Platerot*-) (see Kazantsev 2010c: 278).

### Tribe Slipinskiini

Bocák and Bocáková, 1992

Slipinskiinina Bocák and Bocáková, 1992: 256 [stem: *Slipinski-*]. Type genus: *Slipinskia* Bocák and Bocáková, 1992. Comment: incorrect original stem formation, not in prevailing usage.

### Tribe Thonalmini

Kleine, 1933

Thonalmini Kleine, 1933: 18 [stem: *Thonalm-*]. Type genus: *Thonalmus* Bourgeois, 1883. Comment: name proposed after 1930 without description or bibliographic reference to such a description (Art. 13.1), however available because it was used as valid before 2000 as in Blackwelder (1945: 343, as Thonalmini) and was not rejected by an author who, between 1961 and 1999, applied Article 13 of the then current edition of the Code (see Art. 13.2.1) (see Bocák and Bocáková 2008: 717).

### Subfamily Dexorinae

Bocák and Bocáková, 1989

*Dexorini Kleine, 1933: 113 [stem: *Dexor-*]. Type genus: *Dexoris* C. O. Waterhouse, 1878. Comment: unavailable family-group name, proposed after 1930 without description or bibliographic reference to such a description (Art. 13.1).
Dexorinae Bocák and Bocáková, 1989: 718 [stem: *Dexor-*]. Type genus: *Dexoris* C. O. Waterhouse, 1878.

### Family Telegeusidae

Leng, 1920

Telegeusidae Leng, 1920: 152 [stem: *Telegeus-*]. Type genus: *Telegeusis* G. H. Horn, 1895. Comment: current spelling maintained (Art. 29.5): incorrect stem formation in prevailing usage (should be *Telegeuse*-).

### Family Phengodidae

LeConte, 1861

Phengodini J. L. LeConte, 1861: 185 [stem: *Phengod-*]. Type genus: *Phengodes* Illiger, 1807.

### Subfamily Phengodinae

LeConte, 1861

Phengodini J. L. LeConte, 1861: 185 [stem: *Phengod-*]. Type genus: *Phengodes* Illiger, 1807.
Pseudophengodidae Pic, 1930: 319 [stem: *Pseudophengod-*]. Type genus: *Pseudophengodes* Pic, 1930.

### Subfamily Mastinocerinae

LeConte, 1881

Mastinocerini J. L. LeConte, 1881: 40 [stem: *Mastinocer-*]. Type genus: *Mastinocerus* Solier, 1849.

### Subfamily Penicillophorinae

Paulus, 1975

Penicillophorini Paulus, 1975: 80 [stem: *Penicillophor-*]. Type genus: *Penicillophorus* Paulus, 1975.

### Family Rhagophthalmidae

Olivier, 1907

Rhagophthalmidae E. Olivier, 1907: 63 [stem: *Rhagophthalm-*]. Type genus: *Rhagophthalmus* Motschulsky, 1853.

### Family Lampyridae

Rafinesque, 1815

Lampyria Rafinesque, 1815: 110 [stem: *Lampyr-*]. Type genus: *Lampyris* Geoffroy, 1762 [placed on the Official List of Generic Names in Zoology (ICZN 1984a)].

### Subfamily Psilocladinae

McDermott, 1964

Psilocladina McDermott, 1964: 12, in key [stem: *Psiloclad-*]. Type genus: *Psilocladus* Blanchard, 1846.
Cyphonocerinae Crowson, 1972: 55, in key [stem: *Cyphonocer-*]. Type genus: *Cyphonocerus* Kiesenwetter, 1879.

### Subfamily Amydetinae

Olivier, 1907

Amydetini E. Olivier, 1907: 48 [stem: *Amydet-*]. Type genus: *Amydetes* Hoffmansegg, 1807.

### Tribe Amydetini

Olivier, 1907

Amydetini E. Olivier, 1907: 48 [stem: *Amydet-*]. Type genus: *Amydetes* Hoffmansegg, 1807.
Megalophthalmini E. Olivier, 1907: 46 [stem: *Megalophthalm-*]. Type genus: *Megalophthalmus* Gray, 1832 [preoccupied genus name, not *Megalophthalmus* Leach, 1830 [Crustacea]; syn. of *Magnoculus* McDermott, 1964]. Comment: permanently invalid (Art. 39): based on preoccupied type genus.

### Tribe Vestini

McDermott, 1964

Vestina McDermott, 1964: 12, in key [stem: *Vest-*]. Type genus: *Vesta* Laporte, 1833.

### Subfamily Lampyrinae

Rafinesque, 1815

Lampyria Rafinesque, 1815: 110 [stem: *Lampyr-*]. Type genus: *Lampyris* Geoffroy, 1762 [placed on the Official List of Generic Names in Zoology (ICZN 1984a)].

### Tribe Cratomorphini

Green, 1948

Cratomorphi Green, 1948: 68, in key [stem: *Cratomorph-*]. Type genus: *Cratomorphus* Motschulsky, 1853.

### Tribe Lamprocerini

Olivier, 1907

Lamprocerini E. Olivier, 1907: 7 [stem: *Lamprocer-*]. Type genus: *Lamprocera* Laporte, 1833.

### Tribe Lamprohizini

Kazantsev, 2010

Lamprohizini Kazantsev, 2010d: 189 [stem: *Lamprohiz-*]. Type genus: *Lamprohiza* Motschulsky, 1853.

### Tribe Lampyrini

Rafinesque, 1815

Lampyria Rafinesque, 1815: 110 [stem: *Lampyr-*]. Type genus: *Lampyris* Geoffroy, 1762 [placed on the Official List of Generic Names in Zoology (ICZN 1984a)]. Comment: family-group name previously attributed to Latreille (1816: 236); current spelling maintained (Art. 29.3.1.1): incorrect stem formation in prevailing usage (should be *Lampyrid*-).

### Tribe Lucidotini

Lacordaire, 1857

Lucidotides Lacordaire, 1857: 310 [stem: *Lucidot-*]. Type genus: *Lucidota* Laporte, 1833.

### Subtribe Dadophorina

Olivier, 1907

Dadophorini E. Olivier, 1907: 26 [stem: *Dadophor-*]. Type genus: *Dadophora* E. Olivier, 1907.

### Subtribe Lamprigerina

McDermott, 1964

Lamprigerina McDermott, 1964: 12, in key [stem: *Lampriger-*]. Type genus: *Lamprigera* Motschulsky, 1853.

### Subtribe Lucidotina

Lacordaire, 1857

Lucidotides Lacordaire, 1857: 310 [stem: *Lucidot-*]. Type genus: *Lucidota* Laporte, 1833. Comment: original vernacular name available (Art. 11.7.2): first used in latinized form by J. L. LeConte (1861: 184, as Lucidotae), generally accepted as in Gorham (1881: 29, as Lucidotides [treated as Latin]).
Pristolycini J. R. Winkler, 1953: 409 [stem: *Pristolyc-*]. Type genus: *Pristolycus* Gorham, 1883.
Pristolycini Kazantsev, 2010d: 205 [stem: *Pristolyc-*]. Type genus: *Pristolycus* Gorham, 1883. Comment: name proposed as new without reference to Pristolycini J. R. Winkler, 1953.

### Subtribe Photinina

LeConte, 1881

*Phosphaenaires Mulsant, 1862: 116 [stem: *Phosphaen-*]. Type genus: *Phosphaenus* Laporte, 1833. Comment: original vernacular name unavailable (Art. 11.7.2): subsequently used in latinized form but not generally attributed to Mulsant (1862).
Photini J. L. LeConte, 1881: 30 [stem: *Photin-*]. Type genus: *Photinus* Laporte, 1833. Comment: incorrect original stem formation, not in prevailing usage; this name is a senior homonym of Photininae Giglio-Tos, 1915 (type genus *Photina* Burmeister, 1838) in Mantodea; this case was submitted to the Commission to remove the homonymy by emending the stem of the mantid name (see Svenson and Branham 2007: 243).
Phosphaenides E. Olivier, 1884: 4 [stem: *Phosphaen-*]. Type genus: *Phosphaenus* Laporte, 1833. Comment: original vernacular name available (Art. 11.7.2): first used in latinized form and generally accepted as in Ganglbauer (1886: 280, as Phosphaenini).

### Tribe Pleotomini

Summers, 1874

Pleotomini Summers, 1874: 91 [stem: *Pleotom-*]. Type genus: *Pleotomus* J. L. LeConte, 1861.
Calyptocephalina Jakobson, 1911a: 667 [stem: *Calyptocephal-*]. Type genus: *Calyptocephalus* Gray, 1832.
Pleotomi Green, 1948: 68, in key [stem: *Pleotom-*]. Type genus: *Pleotomus* J. L. LeConte, 1861. Comment: family-group name proposed as new without reference to Pleotomini Summers, 1874.

### Subfamily Luciolinae

Lacordaire, 1857

Luciolides Lacordaire, 1857: 333 [stem: *Luciol-*]. Type genus: *Luciola* Laporte, 1833.

### Tribe Curtosini

McDermott, 1964

Curtosini McDermott, 1964: 47 [stem: *Curtos-*]. Type genus: *Curtos* Motschulsky, 1845. Comment: current spelling maintained (Art. 29.5): incorrect stem formation in prevailing usage (should be *Curt*-).

### Tribe Luciolini

Lacordaire, 1857

Luciolides Lacordaire, 1857: 333 [stem: *Luciol-*]. Type genus: *Luciola* Laporte, 1833. Comment: original vernacular name available (Art. 11.7.2): first used in latinized form by J. L. LeConte (1861: 184, as Luciolini), generally accepted as in Lawrence and Newton (1995: 859, as Luciolinae).

### Subfamily Photurinae

Lacordaire, 1857

Photurides Lacordaire, 1857: 338 [stem: *Photur-*]. Type genus: *Photuris* J. L. LeConte, 1851. Comment: original vernacular name available (Art. 11.7.2): first used in latinized form by Heyne and Taschenberg (1906: 177, as Photurini), generally accepted as in Lawrence and Newton (1995: 859, as Photurinae); current spelling maintained (Art. 29.3.1.1): incorrect stem formation in prevailing usage (should be *Photurid*-).

### Lampyridae incertae sedis

Cheguevariini Kazantsev, 2007: 370 [stem: *Cheguevari-*]. Type genus: *Cheguevaria* Kazantsev, 2007.

### Family Omethidae

LeConte, 1861

Omethes J. L. LeConte, 1861: 187 [stem: *Ometh-*]. Type genus: *Omethes* J. L. LeConte, 1861.

### Subfamily Omethinae

LeConte, 1861

Omethes J. L. LeConte, 1861: 187 [stem: *Ometh-*]. Type genus: *Omethes* J. L. LeConte, 1861.

### Subfamily Matheteinae

LeConte, 1881

Mathetei J. L. LeConte, 1881: 29 [stem: *Mathete-*]. Type genus: *Matheteus* J. L. LeConte, 1874.

### Subfamily Driloniinae

Crowson, 1972

Driloniinae Crowson, 1972: 58, in key [stem: *Driloni-*]. Type genus: *Drilonius* Kiesenwetter, 1874.

### Family Cantharidae

Imhoff, 1856 (1815)

Cantharidae Imhoff, 1856: [2] 69 [stem: *Canthar-*]. Type genus: *Cantharis* Linnaeus, 1758. Comment: this family-group name was used by many authors prior to Imhoff’s usage, these were based on a the misidentified type genus *Cantharis* (syn. of *Lytta* Fabricius, 1775) and are therefore not used as valid for this group (see Lawrence and Newton 1995:860); this case should be referred to the Commission to suppress any use of Cantharidae prior to Imhoff (1856); usage of the younger name Cantharidae Imhoff, 1856 over Telephoridae Leach, 1815 is conserved (Art. 40.2) (see Lawrence and Newton 1995: 860).

### Subfamily Cantharinae

Imhoff, 1856 (1815)

Cantharidae Imhoff, 1856: [2] 69 [stem: *Canthar-*]. Type genus: *Cantharis* Linnaeus, 1758. Comment: usage of the younger name over Telephorinae Leach, 1815 is conserved (Art. 40.2) (see Lawrence and Newton 1995: 860).

### Tribe Cantharini

Imhoff, 1856 (1815)

Telephorides Leach, 1815: 85 [stem: *Telephor-*]. Type genus: *Telephorus* Schaeffer, 1766 [syn. of *Cantharis* Linnaeus, 1758]. Comment: usage of Cantharidae/-inae/-ini over this name is conserved (Art. 40.2) (see Lawrence and Newton 1995: 860).
Cantharidae Imhoff, 1856: [2] 69 [stem: *Canthar-*]. Type genus: *Cantharis* Linnaeus, 1758. Comment: usage of the younger name over Telephorini Leach, 1815 is conserved (Art. 40.2) (see Lawrence and Newton 1995: 860); current spelling maintained (Art. 29.3.1.1): incorrect stem formation in prevailing usage (should be *Cantharid*-).

### Tribe Podabrini

Gistel, 1856

Podabridae Gistel, 1856a: 385 [stem: *Podabr-*]. Type genus: *Podabrus* Dejean, 1833. Comment: family-group name attributed to J. L. LeConte (1881: 45) in recent literature although the same author had used the family-group name Podabri twenty years earlier (J. L. LeConte, 1861: 188).

### Subfamily Silinae

Mulsant, 1862

Siliaires Mulsant, 1862: 342 [stem: *Sil-*]. Type genus: *Silis* Charpentier, 1825.

### Tribe Silini

Mulsant, 1862

Siliaires Mulsant, 1862: 342 [stem: *Sil-*]. Type genus: *Silis* Charpentier, 1825. Comment: original vernacular name available (Art. 11.7.2): first used in latinized form by Gorham (1881: 91, as Silini), generally accepted as in Hansen (1996: 145, as Silinae).

### Tribe Tytthonyxini

Arnett, 1962

Tytthonyini Arnett, 1962a: 537 [stem: *Tytthonyx-*]. Type genus: *Tytthonyx* J. L. LeConte, 1851. Comment: spelling changed to Tytthonyxini by Wittmer (1970: 42); current spelling maintained (Art. 29.5): incorrect stem formation in prevailing usage (should be *Tytthonych*-).

### Subfamily Dysmorphocerinae

Brancucci, 1980

Dysmorphocerinae Brancucci, 1980: 292 [stem: *Dysmorphocer-*]. Type genus: *Dysmorphocerus* Solier, 1849.

### Subfamily Malthininae

Kiesenwetter, 1852

Malthinen Kiesenwetter, 1852: 239 [stem: *Malthin-*]. Type genus: *Malthinus* Latreille, 1806.

### Tribe Malchinini

Brancucci, 1980

Malchinini Brancucci, 1980: 313 [stem: *Malchin-*]. Type genus: *Malchinus* Kiesenwetter, 1863.

### Tribe Malthinini

Kiesenwetter, 1852

Malthinen Kiesenwetter, 1852: 239 [stem: *Malthin-*]. Type genus: *Malthinus* Latreille, 1806. Comment: original vernacular name available (Art. 11.7.2): first used in latinized form by J. L. LeConte (1861: 187, as Malthini), generally accepted as in Lawrence and Newton (1995: 860, as Malthininae); this family-group name was also used in the same year by Motschulsky (1852: 1, as Malthinides) but this vernacular name is unavailable (Art. 11.7.2) because it is not generally attributed to Motschulsky (1852).

### Tribe Malthodini

Böving and Craighead, 1931

Malthodinae Böving and Craighead, 1931: 48, in key [stem: *Malthod-*]. Type genus: *Malthodes* Kiesenwetter, 1852.
Malthodini Brancucci, 1980: 307 [stem: *Malthod-*]. Type genus: *Malthodes* Kiesenwetter, 1852. Comment: family-group name proposed as new without reference to Malthodinae Böving and Craighead, 1931.

### Subfamily Chauliognathinae

LeConte, 1861

Chauliognathini J. L. LeConte, 1861: 186 [stem: *Chauliognath-*]. Type genus: *Chauliognathus* Hentz, 1829.

### Tribe Chauliognathini

LeConte, 1861

Chauliognathini J. L. LeConte, 1861: 186 [stem: *Chauliognath-*]. Type genus: *Chauliognathus* Hentz, 1829.

### Tribe Ichthyurini

Champion, 1915

Ichthyurini Champion, 1915: 128 [stem: *Ichthyur-*]. Type genus: *Ichthyurus* Westwood, 1848.

### Subfamily Cydistinae

Paulus, 1972

Cydistinae Paulus, 1972a: 48, in key [stem: *Cydist-*]. Type genus: *Cydistus* Bourgeois, 1885.

### Subfamily Pterotinae

LeConte, 1861

Pterotini J. L. LeConte, 1861: 185 [stem: *Pterot-*]. Type genus: *Pterotus* J. L. LeConte, 1859.

### Subfamily Ototretinae

McDermott, 1964

Ototretinae McDermott, 1964: 11, in key [stem: *Ototret-*]. Type genus: *Ototreta* E. Olivier, 1900 [syn. of *Drilaster* Kiesenwetter, 1879].

### Subfamily Ototretadrilinae

Crowson, 1972

Ototretadrilinae Crowson, 1972: 55, in key [stem: *Ototretadril-*]. Type genus: *Ototretadrilus* Pic, 1921.

### †Subfamily Lasiosynidae

Kirejtshuk, Chang, Ren and Kun, 2010

Lasiosynidae Kirejtshuk et al., 2010: 68 [stem: *Lasiosyn-*]. Type genus: *Lasiosyne* Tan et al., 2007.

### Series DERODONTIFORMIA

### Superfamily Derodontoidea

LeConte, 1861

Derodontidae J. L. LeConte, 1861: 100 [stem: *Derodont-*]. Type genus: *Derodontus* J. L. LeConte, 1861. Comment: name conserved over Nosodendroidea Erichson, 1846 (Art. 35.5).

### Family Derodontidae

LeConte, 1861

Derodontidae J. L. LeConte, 1861: 100 [stem: *Derodont-*]. Type genus: *Derodontus* J. L. LeConte, 1861. Comment: First Reviser found (Derodontidae J. L. LeConte, 1861 vs Peltasticidae J. L. LeConte, 1861) is Schenkling (1915: 3).

### Subfamily Peltasticinae

LeConte, 1861

Peltasticidae J. L. LeConte, 1861: 88 [stem: *Peltastic-*]. Type genus: *Peltastica* Mannerheim, 1852.

### Subfamily Derodontinae

LeConte, 1861

Derodontidae J. L. LeConte, 1861: 100 [stem: *Derodont-*]. Type genus: *Derodontus* J. L. LeConte, 1861.

### Subfamily Laricobiinae

Mulsant and Rey, 1864

Laricobiens Mulsant and Rey, 1864a: 374 [stem: *Laricobi-*]. Type genus: *Laricobius* Rosenhauer, 1846. Comment: original vernacular name available (Art. 11.7.2): first used in latinized form by Ganglbauer (1899: 766, as Laricobiidae), generally accepted as in Hansen (1996: 146, as Laricobiinae).

### Family Nosodendridae

Erichson, 1846

Nosodendrini Erichson, 1846: 465 [stem: *Nosodendr-*]. Type genus: *Nosodendron* Latreille, 1804.

### Family Jacobsoniidae

Heller, 1926

Jacobsoniidae Heller, 1926: 127 [stem: *Jacobsoni-*]. Type genus: *Jacobsonium* Heller, 1926 [syn. of *Sarothrias* Grouvelle, 1918].
Sarothriidae Crowson, 1955: 75 [stem: *Sarothri-*]. Type genus: *Sarothrias* Grouvelle, 1918. Comment: replacement name for Jacobsoniidae Heller, 1926 because of synonymy of the type genus.
Derolathriinae Sen Gupta, 1979: 692 [stem: *Derolathr-*]. Type genus: *Derolathrus* Sharp, 1900. Comment: incorrect original stem formation, not in prevailing usage.

### Series BOSTRICHIFORMIA

### Superfamily Bostrichoidea

Latreille, 1802

Bostrichini Latreille, 1802: 202 [stem: *Bostrich-*]. Type genus: *Bostrichus* Geoffroy, 1762 [placed on the Official List of Generic Names in Zoology (ICZN 1994a)]. Comment: First Reviser (Bostrichoidea Latreille, 1802 vs Ptinoidea Latreille, 1802) not determined, current usage maintained.

### Family Dermestidae

Latreille, 1804

Dermestini Latreille, 1804c: 142 [stem: *Dermest-*]. Type genus: *Dermestes* Linnaeus, 1758.

### Subfamily Dermestinae

Latreille, 1804

Dermestini Latreille, 1804c: 142 [stem: *Dermest-*]. Type genus: *Dermestes* Linnaeus, 1758.

### Tribe Dermestini

Latreille, 1804

Dermestini Latreille, 1804c: 142 [stem: *Dermest-*]. Type genus: *Dermestes* Linnaeus, 1758. Comment: published 7 March 1804; this family-group name was also used in the same year by Latreille (1804a [between 19 August and 17 September]: 233, as Dermestini).

### Tribe Marioutini

Jakobson, 1913

Marioutini Jakobson, 1913: 826 [stem: *Mariout-*]. Type genus: *Mariouta* Pic, 1899.
Rhopalosilphinae Arrow, 1929: 98 [stem: *Rhopalosilph-*]. Type genus: *Rhopalosilpha* Arrow, 1929. Comment: originally proposed as a subfamily of Silphidae.

### Subfamily Thorictinae

Agassiz, 1846

Thorictides Agassiz, 1846a: 162 [stem: *Thorict-*]. Type genus: *Thorictus* Germar, 1834.

### Tribe Thaumaphrastini

Anderson, 1949

Thaumaphrastinae W. H. Anderson, 1949: 127 [stem: *Thaumaphrast-*]. Type genus: *Thaumaphrastus* Blaisdell, 1927 [syn. of *Thorictodes* Reitter, 1875].

### Tribe Thorictini

Agassiz, 1846

Thorictides Agassiz, 1846a: 162 [stem: *Thorict-*]. Type genus: *Thorictus* Germar, 1834.

### Subfamily Orphilinae

LeConte, 1861

Orphili J. L. LeConte, 1861: 109 [stem: *Orphil-*]. Type genus: *Orphilus* Erichson, 1846.

### Subfamily Trinodinae

Casey, 1900

Trinodini Casey, 1900: 139 [stem: *Trinod-*]. Type genus: *Trinodes* Dejean, 1821.

### †Tribe Cretonodini

Kirejtshuk and Azar, 2009

Cretonodini Kirejtshuk and Azar, 2009: 121 [stem: *Cretonod-*]. Type genus: *Cretonodes* Kirejtshuk and Azar, 2009.

### Tribe Thylodriini

Semenov, 1909

Thelydriini Semenov, 1909: xxv [stem: *Thylodri-*]. Type genus: *Thylodrias* Motschulsky, 1839 [as *Thelydrias*, unjustified emendation of type genus name by Agassiz (1846b: 370), not in prevailing usage]. Comment: incorrect original stem formation, not in prevailing usage.
Trichelodini Peacock, 1978: 344 [stem: *Trichelod-*]. Type genus: *Trichelodes* Carter, 1935.

### Tribe Trinodini

Casey, 1900

Trinodini Casey, 1900: 139 [stem: *Trinod-*]. Type genus: *Trinodes* Dejean, 1821.

### Tribe Trinoparvini

Háva, 2010

Trinoparvini Háva, 2010: 56 [stem: *Trinoparv-*]. Type genus: *Trinoparvus* Háva, 2004.

### Subfamily Attageninae

Laporte, 1840

Attagénites Laporte, 1840b: 35 [stem: *Attagen-*]. Type genus: *Attagenus* Latreille, 1802.

### Tribe Attagenini

Laporte, 1840

Attagénites Laporte, 1840b: 35 [stem: *Attagen-*]. Type genus: *Attagenus* Latreille, 1802. Comment: original vernacular name available (Art. 11.7.2): first used in latinized form by J. L. LeConte (1861: 108, as Attageni), generally accepted as in Hansen (1996: 147, as Attageninae).

### Tribe Egidyellini

Semenov, 1914

Epidyellini Semenov, 1914: 15 [stem: *Egidyell-*]. Type genus: *Egidyella* Reitter, 1899 [as *Epidyella*, incorrect subsequent spelling of type genus name, not in prevailing usage]. Comment: incorrect original stem formation, not in prevailing usage.

### Subfamily Megatominae

Leach, 1815

Megatomida Leach, 1815: 94 [stem: *Megatom-*]. Type genus: *Megatoma* Herbst, 1791.

### Tribe Anthrenini

Gistel, 1848

Anthrenidae Gistel, 1848: [5] [stem: *Anthren-*]. Type genus: *Anthrenus* Geoffroy, 1762 [placed on the Official List of Generic Names in Zoology (ICZN 1994a)].
*Trogodermates Mulsant and Rey, 1867a: 120 [stem: *Trogodermat-*]. Type genus: *Trogoderma* Dejean, 1821. Comment: original vernacular name unavailable (Art. 11.7.2): not subsequently latinized; incorrect original stem formation, not in prevailing usage.

### Tribe Megatomini

Leach, 1815

Megatomida Leach, 1815: 94 [stem: *Megatom-*]. Type genus: *Megatoma* Herbst, 1791.
Ctesiini Rees, 1943: 12 [stem: *Ctesi-*]. Type genus: *Ctesias* Stephens, 1830.

### Family Endecatomidae

LeConte, 1861

Endecatomini J. L. LeConte, 1861: 207 [stem: *Endecatom-*]. Type genus: *Endecatomus* Mellié, 1847.

### Family Bostrichidae

Latreille, 1802

Bostrichini Latreille, 1802: 202 [stem: *Bostrich-*]. Type genus: *Bostrichus* Geoffroy, 1762 [placed on the Official List of Generic Names in Zoology (ICZN 1994a)].

### Subfamily Dysidinae

Lesne, 1921

Dysididae Lesne, 1921b: 286 [stem: *Dysid-*]. Type genus: *Dysides* Perty, 1832.
Apoleoninae Gardner, 1933: 3 [stem: *Apoleont-*]. Type genus: *Apoleon* Gorham, 1885. Comment: incorrect original stem formation, not in prevailing usage.

### Subfamily Polycaoninae

Lesne, 1896

Polycaoninae Lesne, 1896: 96 [stem: *Polycaon-*]. Type genus: *Polycaon* Laporte, 1836.

### Subfamily Bostrichinae

Latreille, 1802

Bostrichini Latreille, 1802: 202 [stem: *Bostrich-*]. Type genus: *Bostrichus* Geoffroy, 1762 [placed on the Official List of Generic Names in Zoology (ICZN 1994a)].

### Tribe Apatini

Billberg, 1820

Apatides Billberg, 1820a: 47 [stem: *Apat-*]. Type genus: *Apate* Fabricius, 1775 [*nomen protectum* (see Borowski and Węgrzynowicz 2009)]. Comment: this family-group name was also used in the same year by Billberg (1820b: 394, as Apatides).
Ligniperdidae Jacobi, 1906: 139 [stem: *Ligniperd-*]. Type genus: *Ligniperda* Pallas, 1772 [*nomen oblitum* (see Borowski and Węgrzynowicz 2009); syn. of *Apate* Fabricius, 1775].
Chileniidae Lesne, 1921b: 287 [stem: *Chileni-*]. Type genus: *Chilenius* Lesne, 1921.
Bostrychopsini Lesne, 1921b: 288 [stem: *Bostrychopse-*]. Type genus: *Bostrychopsis* Lesne, 1899 [syn. of *Apate* Fabricius, 1775]. Comment: incorrect original stem formation, not in prevailing usage.

### Tribe Bostrichini

Latreille, 1802

Bostrichini Latreille, 1802: 202 [stem: *Bostrich-*]. Type genus: *Bostrichus* Geoffroy, 1762 [placed on the Official List of Generic Names in Zoology (ICZN 1994a)].
Apatidini Bradley, 1930: 207, in key [stem: *Apatid-*]. Type genus: *Apatides* Casey, 1898.
Lichenophanini Portevin, 1931: 470, in key [stem: *Lichenophan-*]. Type genus: *Lichenophanes* Lesne, 1899.

### Tribe Dinapatini

Lesne, 1910

Dinapatinae Lesne, 1910: 471 [stem: *Dinapat-*]. Type genus: *Dinapate* G. H. Horn, 1886.

### Tribe Sinoxylini

Marseul, 1857

Sinoxylidae Marseul, 1857a: 107 [stem: *Sinoxyl-*]. Type genus: *Sinoxylon* Duftschmid, 1825.

### Tribe Xyloperthini

Lesne, 1921

Xyloperthini Lesne, 1921b: 288 [stem: *Xyloperth-*]. Type genus: *Xylopertha* Guérin-Méneville, 1845.

### Subfamily Psoinae

Blanchard, 1851

Psoitas Blanchard, 1851b: 434 [stem: *Pso-*]. Type genus: *Psoa* Herbst, 1797. Comment: original vernacular name available (Art. 11.7.2): first used in latinized form by J. L. LeConte (1861: 208, as Psoini), generally accepted as in Ivie (2002: 241, as Psoinae).

### Subfamily Dinoderinae

Thomson, 1863

Dinoderina C. G. Thomson, 1863: 201 [stem: *Dinoder-*]. Type genus: *Dinoderus* Stephens, 1830.

### Subfamily Lyctinae

Billberg, 1820

Lyctides Billberg, 1820a: 48 [stem: *Lyct-*]. Type genus: *Lyctus* Fabricius, 1792.

### Tribe Lyctini

Billberg, 1820

Lyctides Billberg, 1820a: 48 [stem: *Lyct-*]. Type genus: *Lyctus* Fabricius, 1792.

### Tribe Trogoxylini

Lesne, 1921

Trogoxylini Lesne, 1921a: 231 [stem: *Trogoxyl-*]. Type genus: *Trogoxylon* J. L. LeConte, 1862.
Tristariini Lesne, 1921b: 287 [stem: *Tristari-*]. Type genus: *Tristaria* Reitter, 1878.

### Subfamily Euderiinae

Lesne, 1934

Euderiitae Lesne, 1934: 392 [stem: *Euderi-*]. Type genus: *Euderia* Broun, 1880.

### Family Ptinidae

Latreille, 1802

Ptiniores Latreille, 1802: 112 [stem: *Ptin-*]. Type genus: *Ptinus* Linnaeus, 1767 [placed on the Official List of Generic Names in Zoology (ICZN 1995a)]. Comment: Ptinidae Latreille, 1802 placed on the Official List of Family-Group Names in Zoology (ICZN 1995a); several subfamilies in Ptinidae contain tribes introduced in the literature by R. E. White (1982), those tribal names were not described originally and have not been made available subsequently; although some of White’s tribes have been used as available and valid names recently, e.g., Zahradník (2007), they are not available; in subfamilies containing tribes first proposed by R. E. White (1982), we have decided not to use tribal names at all in order to avoid adding to the confusion, the necessary phylogenetic work needed to establish a meaningful tribal classification in those subfamilies is currently under way (K. Philips pers. comm. 2009).

### Subfamily Eucradinae

LeConte, 1861

Eucradini J. L. LeConte, 1861: 202 [stem: *Eucrad-*]. Type genus: *Eucrada* J. L. LeConte, 1861.

### Tribe Eucradini

LeConte, 1861

Eucradini J. L. LeConte, 1861: 202 [stem: *Eucrad-*]. Type genus: *Eucrada* J. L. LeConte, 1861.

### Tribe Hedobiini

Mulsant and Rey, 1868

Hédobiaires Mulsant and Rey, 1868b: 23 [stem: *Hedobi-*]. Type genus: *Hedobia* Dejean, 1821. Comment: original vernacular name available (Art. 11.7.2): first used in latinized form by Seidlitz (1889 [Gatt.]: 116, as Hedobiini), generally accepted as in Philips (2002: 254, as Hedobiini).

### Subfamily Ptininae

Latreille, 1802

Ptiniores Latreille, 1802: 112 [stem: *Ptin-*]. Type genus: *Ptinus* Linnaeus, 1767 [placed on the Official List of Generic Names in Zoology (ICZN 1995a)]. Comment: Ptinidae Latreille, 1802 placed on the Official List of Family-Group Names in Zoology (ICZN 1995a).

### Tribe Gibbiini

Jacquelin du Val, 1860

Gibbiites Jacquelin du Val, 1860: 211 [stem: *Gibbi-*]. Type genus: *Gibbium* Scopoli, 1777. Comment: original vernacular name available (Art. 11.7.2): first used in latinized form by Kiesenwetter (1877: 44, as Gibbiini), generally accepted as in Bellés (1985: 13, as Gibbiinae).

### Tribe Meziini

Bellés, 1985

Meziini Bellés, 1985: 37, in key [stem: *Mezi-*]. Type genus: *Mezium* Curtis, 1828.

### Tribe Ptinini

Latreille, 1802

Ptiniores Latreille, 1802: 112 [stem: *Ptin-*]. Type genus: *Ptinus* Linnaeus, 1767 [placed on the Official List of Generic Names in Zoology (ICZN 1995a)]. Comment: Ptinidae Latreille, 1802 placed on the Official List of Family-Group Names in Zoology (ICZN 1995a).

### Tribe Sphaericini

Portevin, 1931

Sphaericini Portevin, 1931: 494 [stem: *Sphaeric-*]. Type genus: *Sphaericus* Wollaston, 1854.

### Ptininae incertae sedis

Gnostidae Gemminger and Harold, 1868: 700 [stem: *Gnost-*]. Type genus: *Gnostus* Westwood, 1855.
Ectrephidae Wasmann, 1894: 121 [stem: *Ectreph-*]. Type genus: *Ectrephes* Pascoe, 1866.

### Subfamily Dryophilinae

Gistel, 1848

Dryophilidae Gistel, 1848: [6] [stem: *Dryophil-*]. Type genus: *Dryophilus* Chevrolat, 1832.

### Tribe Dryophilini

Gistel, 1848

Dryophilidae Gistel, 1848: [6] [stem: *Dryophil-*]. Type genus: *Dryophilus* Chevrolat, 1832. Comment: family-group name previously attributed to J. L. LeConte (1861: 205) in the literature.
Dryobiadae Gistel, 1856a: 368 [stem: *Dryobi-*]. Type genus: *Dryobia* Gistel, 1856 [syn. of *Dryophilus* Chevrolat, 1832]. Comment: senior homonym of Dryobiini Arnett, 1962 (type genus *Dryobius* J. L. LeConte, 1850) used as valid in Cerambycidae; *nomen oblitum* (see Bousquet et al. 2009: 45); incorrect original stem formation, not in prevailing usage.

### Tribe Ptilineurini

Böving, 1927

Ptilineurini Böving, 1927a: 56 [stem: *Ptilineur-*]. Type genus: *Ptilineurus* Reitter, 1901.

### Subfamily Ernobiinae

Pic, 1912

Cosmoceroideos Solier, 1849: 476 [stem: *Cosmocer-*]. Type genus: *Cosmocerus* Solier, 1849 [preoccupied genus name, not *Cosmocerus* Guérin-Méneville, 1844 [Coleoptera: Cerambycidae]; syn. of *Cerocosmus* Gemminger, 1873]. Comment: permanently invalid (Art. 39): based on preoccupied type genus; Lawrence and Newton (1995: 864) used the latinized form Cosmocerinae and treated Solier’s name as available therefore we treat this name as available but permanently invalid.
Ernobiinae Pic, 1912: 12 [stem: *Ernobi-*]. Type genus: *Ernobius* C. G. Thomson, 1859. Comment: First Reviser found (Ernobiinae Pic, 1912 vs Cerocosminae Pic, 1912) is White (1974: 419).
Cerocosminae Pic, 1912: 45 [stem: *Cerocosm-*]. Type genus: *Cerocosmus* Gemminger, 1873.
Xestobiini Böving, 1927a: 56 [stem: *Xestobi-*]. Type genus: *Xestobium* Motschulsky, 1845.
*Ozognathini R. E. White, 1982: 2 [stem: *Ozognath-*]. Type genus: *Ozognathus* J. L. LeConte, 1861. Comment: unavailable family-group name, proposed after 1930 without description or bibliographic reference to such a description (Art. 13.1).

### Subfamily Anobiinae

Fleming, 1821

Anobiumedae Fleming, 1821: 50 [stem: *Anobi-*]. Type genus: *Anobium* Fabricius, 1775 [placed on the Official List of Generic Names in Zoology (ICZN 1976)]. Comment: incorrect original stem formation, not in prevailing usage.
*Euceratocerini R. E. White, 1982: 7 [stem: *Euceratocer-*]. Type genus: *Euceratocerus* J. L. LeConte, 1874. Comment: unavailable family-group name, proposed after 1930 without description or bibliographic reference to such a description (Art. 13.1).
*Colposternini R. E. White, 1982: 9 [stem: *Colpostern-*]. Type genus: *Colposternus* Fall, 1905. Comment: unavailable family-group name, proposed after 1930 without description or bibliographic reference to such a description (Art. 13.1).
*Gastrallini R. E. White, 1982: 9 [stem: *Gastrall-*]. Type genus: *Gastrallus* Jacquelin du Val, 1860. Comment: unavailable family-group name, proposed after 1930 without description or bibliographic reference to such a description (Art. 13.1).
*Hadrobregmini R. E. White, 1982: 15 [stem: *Hadrobregm-*]. Type genus: *Hadrobregmus* C. G. Thomson, 1859. Comment: unavailable family-group name, proposed after 1930 without description or bibliographic reference to such a description (Art. 13.1).
*Nicobiini R. E. White, 1982: 10 [stem: *Nicobi-*]. Type genus: *Nicobium* J. L. LeConte, 1861. Comment: unavailable family-group name, proposed after 1930 without description or bibliographic reference to such a description (Art. 13.1).
*Stegobiini R. E. White, 1982: 11 [stem: *Stegobi-*]. Type genus: *Stegobium* Motschulsky, 1860. Comment: unavailable family-group name, proposed after 1930 without description or bibliographic reference to such a description (Art. 13.1).

### Subfamily Ptilininae

Shuckard, 1839

Ptilinidae Shuckard, 1839b: 45 [stem: *Ptilin-*]. Type genus: *Ptilinus* Geoffroy, 1762 [placed on the Official List of Generic Names in Zoology (ICZN 1994a)].
Sclerasteidae Gistel, 1856a: 368 [stem: *Sclerast-*]. Type genus: *Sclerastes* Gistel, 1856 [this genus originally included “*pectinicornis*” and “*costatus* Gy”, we here chose *Ptilinuscostatus* Gyllenhal, 1827 as the type of *Sclerastes*; **syn. nov.** of *Ptilinus* Geoffroy, 1762]. Comment: **syn. nov.**; incorrect original stem formation, not in prevailing usage.

### Subfamily Alvarenganiellinae

Viana and Martínez, 1971

Alvarenganiellinae Viana and Martínez, 1971: 121 [stem: *Alvarenganiell-*]. Type genus: *Alvarenganiella* Viana and Martínez, 1971.

### Subfamily Xyletininae

Gistel, 1848

Xyletinidae Gistel, 1848: [6] [stem: *Xyletin-*]. Type genus: *Xyletinus* Latreille, 1810 [placed on the Official List of Generic Names in Zoology (ICZN 1971)].

### Tribe Lasiodermini

Böving, 1927

Lasiodermini Böving, 1927a: 56 [stem: *Lasioderm-*]. Type genus: *Lasioderma* Stephens, 1835. Comment: current spelling maintained (Art. 29.5): incorrect stem formation in prevailing usage (should be *Lasiodermat*-).

### Tribe Metholcini

Zahradník, 2009

Metholcini Zahradník, 2009: 180 [stem: *Metholc-*]. Type genus: *Metholcus* Jacquelin du Val, 1860.

### Tribe Xyletinini

Gistel, 1848

Xyletinidae Gistel, 1848: [6] [stem: *Xyletin-*]. Type genus: *Xyletinus* Latreille, 1810 [placed on the Official List of Generic Names in Zoology (ICZN 1971)].
Vrilettini Böving, 1927a: 56 [stem: *Vrillett-*]. Type genus: *Vrilletta* J. L. LeConte, 1874. Comment: incorrect original stem formation, not in prevailing usage.

### Subfamily Dorcatominae

Thomson, 1859

Dorcatomina C. G. Thomson, 1859: 90 [stem: *Dorcatom-*]. Type genus: *Dorcatoma* Herbst, 1791 [the original spelling *Dorkatoma* was placed on the Official Index of Rejected and Suppressed Generic Names in Zoology, *Dorcatoma* was chosen as correct original spelling and placed on the Official List of Generic Names in Zoology (ICZN 1995b)].
Caenocarini Böving, 1927a: 57 [stem: *Caenocar-*]. Type genus: *Caenocara* C. G. Thomson, 1859. Comment: also misspelled as Coenecarini (page 56) in the original publication.
*Calymmaderini R. E. White, 1982: 22 [stem: *Calymmader-*]. Type genus: *Calymmaderus* Solier, 1849. Comment: unavailable family-group name, proposed after 1930 without description or bibliographic reference to such a description (Art. 13.1).
*Cryptoramorphini R. E. White, 1982: 25 [stem: *Cryptoramorph-*]. Type genus: *Cryptoramorphus* R. E. White, 1966. Comment: unavailable family-group name, proposed after 1930 without description or bibliographic reference to such a description (Art. 13.1).
*Petaliini R. E. White, 1982: 23 [stem: *Petali-*]. Type genus: *Petalium* J. L. LeConte, 1861. Comment: unavailable family-group name, proposed after 1930 without description or bibliographic reference to such a description (Art. 13.1); the tribe Petaliini Tillyard, 1917 in Odonata (type genus *Petalia* Hagen, 1854, a junior homonym of *Petalia* Gray, 1838 [Mammalia]) is available but permanently invalid because it is based on a preoccupied type genus.
*Prothecini R. E. White, 1982: 25 [stem: *Prothec-*]. Type genus: *Protheca* J. L. LeConte, 1865. Comment: unavailable family-group name, proposed after 1930 without description or bibliographic reference to such a description (Art. 13.1).

### Subfamily Mesocoelopodinae

Mulsant and Rey, 1864

Mésocoelopaires Mulsant and Rey, 1864b: 311 [stem: *Mesocoelopod-*]. Type genus: *Mesocoelopus* Jacquelin du Val, 1860.

### Tribe Tricorynini

White, 1971

Tricoryninae R. E. White, 1971: 1301 [stem: *Tricoryn-*]. Type genus: *Tricorynus* G. R. Waterhouse, 1849.

### Tribe Mesocoelopodini

Mulsant and Rey, 1864

Mésocoelopaires Mulsant and Rey, 1864b: 311 [stem: *Mesocoelopod-*]. Type genus: *Mesocoelopus* Jacquelin du Val, 1860. Comment: original vernacular name available (Art. 11.7.2): first used in latinized form by Kiesenwetter (1877: 153, as Mesocoelopini [incorrect stem formation]), generally accepted as in Philips (2002: 258, as Mesocoelopodinae); incorrect original stem formation, not in prevailing usage.
Mesothini Portevin, 1931: 489 [stem: *Mesothet-*]. Type genus: *Mesothes* Mulsant and Rey, 1864. Comment: incorrect original stem formation, not in prevailing usage.

### Ptinidae incertae sedis

Fabiinae Martínez and Viana, 1964: 7 [stem: *Fabi-*]. Type genus: *Fabia* Martínez and Viana, 1964.

### Series CUCUJIFORMIA

### Superfamily Lymexyloidea

Fleming, 1821

Lymoxylonidae Fleming, 1821: 49 [stem: *Lymexyl-*]. Type genus: *Lymexylon* Fabricius, 1775. Comment: Lymexyloidea given precedence for superfamily name over Hylecoetoidea Germar, 1818 (Art. 35.5).

### Family Lymexylidae

Fleming, 1821

Lymoxylonidae Fleming, 1821: 49 [stem: *Lymexyl-*]. Type genus: *Lymexylon* Fabricius, 1775. Comment: Lymexylidae given precedence for family name over Hylecoetidae Germar, 1818 (Art. 35.5).

### Subfamily Hylecoetinae

Germar, 1818

Hylecoeti Germar, 1818: 344 [stem: *Hylecoet-*]. Type genus: *Hylecoetus* Latreille, 1806. Comment: name previously attributed to Gistel (1856a).

### Subfamily Lymexylinae

Fleming, 1821

Lymoxylonidae Fleming, 1821: 49 [stem: *Lymexyl-*]. Type genus: *Lymexylon* Fabricius, 1775 [as *Lymoxylon*, incorrect subsequent spelling of type genus name, not in prevailing usage]. Comment: published in July 1821; incorrect original stem formation, not in prevailing usage; this family-group name was also used in the same year by Fischer von Waldheim (1821 [“31 December”]: 37, as Lymexyla).

### Subfamily Atractocerinae

Laporte, 1840

Atractocérites Laporte, 1840a: 290 [stem: *Atractocer-*]. Type genus: *Atractocerus* Palisot de Beauvois, 1802. Comment: original vernacular name available (Art. 11.7.2): first used in latinized form by Brues and Melander (1932: 23, as Atractoceridae), generally accepted as in Cuccodoro (2007: 363, as Atractocerinae).

### Subfamily Melittommatinae

Wheeler, 1986

Melittomminae Q. D. Wheeler, 1986: 160 [stem: *Melittommat-*]. Type genus: *Melittomma* Murray, 1867. Comment: incorrect original stem formation, not in prevailing usage.

### Superfamily Cleroidea

Latreille, 1802

Clerii Latreille, 1802: 110 [stem: *Cler-*]. Type genus: *Clerus* Geoffroy, 1762 [placed on the Official List of Generic Names in Zoology (ICZN 1984a)]. Comment: First Reviser (Cleroidea Latreille, 1802 vs Trogossitoidea Latreille, 1802) not determined, current usage maintained.

### Family Phloiophilidae

Kiesenwetter, 1863

Phloeophilini Kiesenwetter, 1863: 626, in key [stem: *Phloiophil-*]. Type genus: *Phloiophilus* Stephens, 1830 [as *Phloeophilus*, unjustified emendation of type genus name by Agassiz (1846b: 286), not in prevailing usage; *Phloeophilus* Agassiz, 1846 is a junior homonym of *Phloeophilus* Schönherr, 1833 [Coleoptera: Anthribidae]]. Comment: incorrect original stem formation, not in prevailing usage; using the original spelling would place Phloeophilini Lacordaire, 1865 [Coleoptera: Anthribidae] into homonymy with this name.

### Family Trogossitidae

Latreille, 1802

Trogossitarii Latreille, 1802: 159 [stem: *Trogossit-*]. Type genus: *Trogossita* A. G. Olivier, 1790 [syn. of *Tenebroides* Piller and Mitterpacher, 1783].

### Subfamily Peltinae

Latreille, 1806

Peltides Latreille, 1806: 8 [stem: *Pelt-*]. Type genus: *Peltis* Kugelann, 1792 [placed on the Official List of Generic Names in Zoology (ICZN 1994a)].

### Tribe Ancyronini

Kolibáč, 2006

Ancyronini Kolibáč, 2006: 127 [stem: *Ancyron-*]. Type genus: *Ancyrona* Reitter, 1876.

### Tribe Colydiopeltini

Kolibáč, 2006

Colydiopeltini Kolibáč, 2006: 126 [stem: *Colydiopelt-*]. Type genus: *Colydiopeltis* Ślipiński, 1992.

### Tribe Decamerini

Crowson, 1964

Decamerinae Crowson, 1964: 287 [stem: *Decamer-*]. Type genus: *Decamerus* Solier, 1849.

### Tribe Lophocaterini

Crowson, 1964 nomen protectum

Lycoptini Casey, 1890: 311 [stem: *Lycopt-*]. Type genus: *Lycoptis* Casey, 1890. Comment: *nomen oblitum* (see Appendix 1).
Lophocaterinae Crowson, 1964: 297 [stem: *Lophocater-*]. Type genus: *Lophocateres* Olliff, 1883. Comment: *nomen protectum* (see Appendix 1).

### Tribe Peltini

Latreille, 1806

Peltides Latreille, 1806: 8 [stem: *Pelt-*]. Type genus: *Peltis* Kugelann, 1792 [placed on the Official List of Generic Names in Zoology (ICZN 1994a)]. Comment: name previously attributed to Kirby (1837).
Ostomini Harold, 1876: 169 [stem: *Ostomat-*]. Type genus: *Ostoma* Laicharting, 1781. Comment: incorrect original stem formation, not in prevailing usage.

### Tribe Thymalini

Léveillé, 1888

*Thymalites Blanchard, 1845a: 277 [stem: *Thymal-*]. Type genus: *Thymalus* Latreille, 1802. Comment: original vernacular name unavailable (Art. 11.7.2): subsequently used in latinized form but not generally attributed to Blanchard (1845a).
Thymalini Léveillé, 1888: 444 [stem: *Thymal-*]. Type genus: *Thymalus* Latreille, 1802.
Rentoniinae Crowson, 1966: 120 [stem: *Rentoni-*]. Type genus: *Rentonium* Crowson, 1966.
Protopeltini Crowson, 1966: 120 [stem: *Protopelt-*]. Type genus: *Protopeltis* Crowson, 1964.
†Meligethiellinae Kirejtshuk and Ponomarenko, 1990: 79 [stem: *Meligethiell-*]. Type genus: *Meligethiella* L. N. Medvedev, 1969.

### Subfamily Trogossitinae

Latreille, 1802

Trogossitarii Latreille, 1802: 159 [stem: *Trogossit-*]. Type genus: *Trogossita* A. G. Olivier, 1790 [syn. of *Tenebroides* Piller and Mitterpacher, 1783].

### Tribe Calityini

Reitter, 1922

Calityni Reitter, 1922a: 66 [stem: *Cality-*]. Type genus: *Calitys* C. G. Thomson, 1859. Comment: incorrect original stem formation, not in prevailing usage.

### Tribe Egoliini

Lacordaire, 1854

Égoliides Lacordaire, 1854b: 334 [stem: *Egoli-*]. Type genus: *Egolia* Erichson, 1842. Comment: original vernacular name available (Art. 11.7.2): first used in latinized form by Ganglbauer (1899: 419, as Egoliides [treated as Latin]), generally accepted as in Kolibáč (2006: 119, as Egoliini).

### Tribe Gymnochilini

Lacordaire, 1854

Gymnochilides Lacordaire, 1854b: 344 [stem: *Gymnochil-*]. Type genus: *Gymnochila* Klug, 1844. Comment: original vernacular name available (Art. 11.7.2): first used in latinized form by J. Thomson (1858: 43, as Gymnochilitae), generally accepted as in Burakowski et al. (1986: 118, as Gymnochilinae).
Leperini Reitter, 1876a: 55 [stem: *Leperin-*]. Type genus: *Leperina* Erichson, 1844. Comment: incorrect original stem formation, not in prevailing usage.

### Tribe Larinotini

Ślipiński, 1992

Larinotinae Ślipiński, 1992: 443 [stem: *Larinot-*]. Type genus: *Larinotus* Carter and Zeck, 1937.

### †Tribe Lithostomatini

Kolibáč and Huang, 2008

Lithostomini Kolibáč and Huang, 2008: 142 [stem: *Lithostomat-*]. Type genus: *Lithostoma* Martynov, 1926. Comment: incorrect original stem formation, not in prevailing usage.

### Tribe Trogossitini

Latreille, 1802

Trogossitarii Latreille, 1802: 159 [stem: *Trogossit-*]. Type genus: *Trogossita* A. G. Olivier, 1790 [syn. of *Tenebroides* Piller and Mitterpacher, 1783].
Nemosomida Leach, 1815: 110 [stem: *Nemozomat-*]. Type genus: *Nemozoma* Latreille, 1804 [as *Nemosoma*, incorrect subsequent spelling of type genus name, not in prevailing usage]. Comment: incorrect original stem formation, not in prevailing usage.
Temnochilini Léveillé, 1888: 432 [stem: *Temnoscheil-*]. Type genus: *Temnoscheila* Westwood, 1830 [as *Temnochila*, unjustified emendation of type genus name by Erichson (1844: 449), not in prevailing usage]. Comment: incorrect original stem formation, not in prevailing usage.
Tenebrioidini Ganglbauer, 1899: 420 [stem: *Tenebroid-*]. Type genus: *Tenebroides* Piller and Mitterpacher, 1783 [as *Tenebrioides*, incorrect subsequent spelling of type genus name, not in prevailing usage]. Comment: incorrect original stem formation, not in prevailing usage.

### Family Chaetosomatidae

Crowson, 1952

Chaetosomatidae Crowson, 1952: 66 [stem: *Chaetosomat-*]. Type genus: *Chaetosoma* Westwood, 1851 [this genus is a junior homonym of *Chaetosoma* Chevrolat, 1843 in Cerambycidae; *Chaetosoma* Chevrolat, 1843 is also a senior objective synonym of the well-established name *Apodasya* Pascoe, 1863]. Comment: this name is a junior homonym of Chaetosomatidae Claus, 1872 (type genus *Chaetosoma* Claparède, 1863, which is a junior homonym of *Chaetosoma* Westwood, 1851) in Nematoda; the nematode family-group name is permanently invalid (Art. 39) because it is based on a junior homonym, the valid name for this nematode family is Draconematidae Filipjev, 1918; furthermore, the type genus of Chaetosomatidae Crowson is a junior homonym of *Chaetosoma* Chevrolat, 1843 in Cerambycidae; an application was recently submitted by Bousquet and Bouchard (2010) to suppress *Chaetosoma* Chevrolat, 1843 for the Principles of Priority and Homonymy and Chaetosomatidae Claus, 1872 for the Principle of Priority, therefore conserving the names Chaetosomatidae Crowson and *Chaetosoma* Westwood (see Appendix 6).

### Family Metaxinidae

Kolibáč, 2004

Metaxinidae Kolibáč, 2004: 247 [stem: *Metaxin-*]. Type genus: *Metaxina* Broun, 1909.

### Family Thanerocleridae

Chapin, 1924

Thaneroclerinae Chapin, 1924: 251 [stem: *Thanerocler-*]. Type genus: *Thaneroclerus* Westwood, 1838.

### Subfamily Zenodosinae

Kolibáč, 1992

Zenodosini Kolibáč, 1992: 307 [stem: *Zenodos-*]. Type genus: *Zenodosus* Wolcott, 1910.

### Subfamily Thaneroclerinae

Chapin, 1924

Thaneroclerinae Chapin, 1924: 251 [stem: *Thanerocler-*]. Type genus: *Thaneroclerus* Westwood, 1838.

### Tribe Isoclerini

Kolibáč, 1992

Isoclerina Kolibáč, 1992: 315 [stem: *Isocler-*]. Type genus: *Isoclerus* Lewis, 1892.

### Tribe Thaneroclerini

Chapin, 1924

Thaneroclerinae Chapin, 1924: 251 [stem: *Thanerocler-*]. Type genus: *Thaneroclerus* Westwood, 1838.

### Tribe Viticlerini

Winkler, 1982

Viticlerini J. R. Winkler, 1982: 529, in key [stem: *Viticler-*]. Type genus: *Viticlerus* Miyatake, 1977.

### Family Cleridae

Latreille, 1802

Clerii Latreille, 1802: 110 [stem: *Cler-*]. Type genus: *Clerus* Geoffroy, 1762 [placed on the Official List of Generic Names in Zoology (ICZN 1984a)]. Comment: see Opitz (2010) for an alternative subfamilial classification within this family.

### Subfamily Tillinae

Fischer von Waldheim, 1813

Tillii Fischer von Waldheim, 1813: 232 [stem: *Till-*]. Type genus: *Tillus* A. G. Olivier, 1790. Comment: family-group name previously attributed to Leach (1815).
Cylidrina Reitter, 1894: 38 [stem: *Cylidr-*]. Type genus: *Cylidrus* Latreille, 1829.
*Monophyllini Böving and Craighead, 1931: 78 [stem: *Monophyll-*]. Type genus: *Monophylla* Spinola, 1841. Comment: unavailable family-group name, proposed after 1930 without description or bibliographic reference to such a description (Art. 13.1).

### Subfamily Hydnocerinae

Spinola, 1844

Hydnocéroïdes Spinola, 1844: Tableau générique (3) [stem: *Hydnocer-*]. Type genus: *Hydnocera* Newman, 1838 [syn. of *Phyllobaenus* Dejean, 1833].

### Tribe Callimerini

Kolibáč, 1998

Callimerini Kolibáč, 1998: 165 [stem: *Callimer-*]. Type genus: *Callimerus* Gorham, 1876.

### Tribe Hydnocerini

Spinola, 1844

Hydnocéroïdes Spinola, 1844: Tableau générique (3) [stem: *Hydnocer-*]. Type genus: *Hydnocera* Newman, 1838 [syn. of *Phyllobaenus* Dejean, 1833]. Comment: original vernacular name available (Art. 11.7.2): first used in latinized form by Desmarest (1857: 263, as Hydnoceridae), generally accepted as in Kolibáč (1998: 127, as Hydnocerinae).
Phyllobénides Lacordaire, 1857: 466 [stem: *Phyllobaen-*]. Type genus: *Phyllobaenus* Dejean, 1833. Comment: original vernacular name available (Art. 11.7.2): first used in latinized form by and generally accepted as in Gorham (1876: 59, as Phyllobenides [treated as Latin]); incorrect original stem formation, not in prevailing usage.

### Tribe Lemidiini

Kolibáč, 1998

Lemidiini Kolibáč, 1998: 183 [stem: *Lemidi-*]. Type genus: *Lemidia* Spinola, 1841.

### Subfamily Clerinae

Latreille, 1802

Clerii Latreille, 1802: 110 [stem: *Cler-*]. Type genus: *Clerus* Geoffroy, 1762 [placed on the Official List of Generic Names in Zoology (ICZN 1984a)].
Notoxii Sturm, 1826: 38 [stem: *Notox-*]. Type genus: *Notoxus* sensu Fabricius, 1775 [not *Notoxus* Geoffroy, 1762; syn. of *Opilo* Latreille, 1802]. Comment: based on a misidentified type genus; an application should be submitted to the Commission to suppress this name for the Principles of Priority and Homonymy (Art. 65.2.1) since Notoxinae Stephens, 1829 is currently used as valid in Anthicidae.
Prioceridae Laporte, 1836: 33 [stem: *Priocer-*]. Type genus: *Priocera* Kirby, 1819.
*Trichodites Blanchard, 1845b: 84 [stem: *Trichod-*]. Type genus: *Trichodes* Herbst, 1792 [placed on the Official List of Generic Names in Zoology (ICZN 1984a)]. Comment: original vernacular name unavailable (Art. 11.7.2): subsequently used in latinized form but not generally attributed to Blanchard (1845b).
Opilonidae Gistel, 1848: [6] [stem: *Opilon-*]. Type genus: *Opilo* Latreille, 1802.
Thanasimiidae Gistel, 1848: [6] [stem: *Thanasim-*]. Type genus: *Thanasimus* Latreille, 1806. Comment: incorrect original stem formation, not in prevailing usage.
Dendroplaneteidae Gistel, 1856a: 395 [stem: *Dendroplanet-*]. Type genus: *Dendroplanetes* Gistel, 1856 [syn. of *Opilo* Latreille, 1802]. Comment: also spelled Dondroplaneteidae in the same publication on page 368; incorrect original stem formation, not in prevailing usage.
Trichodini Portevin, 1931: 457, in key [stem: *Trichod-*]. Type genus: *Trichodes* Herbst, 1792 [placed on the Official List of Generic Names in Zoology (ICZN 1984a)]. Comment: the older name originally proposed as Trichodina Maitland, 1851 (type genus *Trichoda* Müller, 1773) is available in Protozoa: Ciliophora; this case is to be referred to the Commission to remove the homonymy (Art. 55.3.1).
Dieropsinae J. R. Winkler, 1964: 317 [stem: *Dieropse-*]. Type genus: *Dieropsis* Gahan, 1908. Comment: incorrect original stem formation, not in prevailing usage.
Cleropiestinae J. R. Winkler, 1980: 437 [stem: *Cleropiest-*]. Type genus: *Cleropiestus* Fairmaire, 1889.
Anthicoclerinae Opitz, 2010: 58 [stem: *Anthicocler*-]. Type genus: *Anthicoclerus* Schenkling, 1906.

### Subfamily Korynetinae

Laporte, 1836

Corynetidae Laporte, 1836: 34 [stem: *Korynet-*]. Type genus: *Korynetes* Herbst, 1792 [as *Corynetes*, unjustified emendation of type genus name by Paykull (1798: 274), not in prevailing usage; *Korynetes* Herbst, 1792 placed on the Official List of Generic Names in Zoology and *Corynetes* Paykull, 1798 placed on the Official Index of Rejected and Invalid Generic Names in Zoology (ICZN 1961c)]. Comment: incorrect original stem formation, not in prevailing usage.
Ichnoïdes Spinola, 1841: 71 [stem: *Ichne-*]. Type genus: *Ichnea* Laporte, 1836. Comment: original vernacular name available (Art. 11.7.2): first used in latinized form by Agassiz (1846b: 193, as Ichneoidae), treated as available by Opitz and Herman (2009: 183, as Ichneinae); *nomen oblitum*, Epiphloeinae Kuwert, 1893 conserved as valid subfamily over this name by Opitz and Herman (2009).
Platynoptéroïdes Spinola, 1844: Tableau générique (4) [stem: *Platynopter-*]. Type genus: *Platynoptera* Chevrolat, 1834. Comment: original vernacular name available (Art. 11.7.2): first used in latinized form and generally accepted as in Desmarest (1857: 269, as Platynopteridae).
Necrobiaeidae Gistel, 1848: [6] [stem: *Necrobi-*]. Type genus: *Necrobia* A. G. Olivier, 1795 [placed on the Official List of Generic Names in Zoology (ICZN 1961c)]. Comment: incorrect original stem formation, not in prevailing usage.
Enopliidae Gistel, 1848: [6] [stem: *Enopli-*]. Type genus: *Enoplium* Latreille, 1802.
Tarsosténites Jacquelin du Val, 1860: 198 [stem: *Tarsosten-*]. Type genus: *Tarsostenus* Spinola, 1844. Comment: original vernacular name available (Art. 11.7.2): first used in latinized form by Böving and Craighead (1931: 57, as Tarsosteninae), generally accepted as in Opitz (2002: 279, as Tarsostenninae [incorrect stem formation]).
Epiphlöinen Kuwert, 1893: 492 [stem: *Epiphloe-*]. Type genus: *Epiphloeus* Spinola, 1841. Comment: original vernacular name available (Art. 11.7.2): first used in latinized form by Schenkling (1910: 113, as Epiphloeini), generally accepted as in Opitz and Herman (2009: 183, as Epiphloeinae); *nomen protectum* (see Opitz and Herman (2009: 184)); although Kuwert apparently originally published his name as a synonym of Phyllobaenides Lacordaire, 1857, the name Epiphloeini was treated as valid by several authors, e.g., Chapin (1924: 165), before 1961 which made the name available (Art. 11.6.1) (see Opitz and Herman 2009: 184).
Dermestoidini Jakobson, 1911a: 719 [stem: *Dermestoid-*]. Type genus: *Dermestoides* Schaeffer, 1771.
Orthopleurinae Böving and Craighead, 1931: 56, in key [stem: *Orthopleur-*]. Type genus: *Orthopleura* Spinola, 1844.
Neorthopleurinae Opitz, 2009: 138 [stem: *Neorthopleur-*]. Type genus: *Neorthopleura* Barr, 1976.
Peloniinae Opitz, 2010: 97 [stem: *Peloni*-]. Type genus: *Pelonium* Spinola, 1844.

### Family Acanthocnemidae

Crowson, 1964

Acanthocneminae Crowson, 1964: 317, in key [stem: *Acanthocnem-*]. Type genus: *Acanthocnemus* Perris, 1866.

### Family Phycosecidae

Crowson, 1952

Phycosecidae Crowson, 1952: 117 [stem: *Phycosec-*]. Type genus: *Phycosecis* Pascoe, 1875. Comment: current spelling maintained (Art. 29.3.1.1): incorrect stem formation in prevailing usage (should be *Phycosecid*-).

### Family Prionoceridae

Lacordaire, 1857

Prionocérides Lacordaire, 1857: 411 [stem: *Prionocer-*]. Type genus: *Prionocerus* Perty, 1831. Comment: Prionocérites was used earlier by Laporte (1840a: 275) but this name was treated as a *lapsus calami* for Priocérites Laporte (1834b: 33) by Lawrence and Newton (1995: 871).

### Tribe Lobonychini

Majer, 1987

Lobonychini Majer, 1987: 790, in key [stem: *Lobonych-*]. Type genus: *Lobonyx* Jacquelin du Val, 1859.

### Tribe Prionocerini

Lacordaire, 1857

Prionocérides Lacordaire, 1857: 411 [stem: *Prionocer-*]. Type genus: *Prionocerus* Perty, 1831. Comment: original vernacular name available (Art. 11.7.2): first used in latinized form by Ritsema (1884: 494, as Prionocerini), generally accepted as in M. C. Thomas (2008: 445, as Prionoceridae); the earlier usage of Prionocerites by Laporte (1840a: 285) was in error for Priocerites in Cleridae: Clerinae (see Lawrence and Newton 1995: 871); Prionocerini Savchenko, 1966 (type genus *Prionocera* Loew, 1844) is available in Diptera; this case is to be referred to the Commission to remove the homonymy (Art. 55.3.1).

### Family Mauroniscidae

Majer, 1995

Mauroniscidae Majer, 1995a: 340 [stem: *Mauronisc-*]. Type genus: *Mauroniscus* Bourgeois, 1911. Comment: published 30 April 1995; this family-group name was also used in the same year by Majer (1995b [1 December]: 57, as Mauroniscidae).

### Family Melyridae

Leach, 1815

Melyrides Leach, 1815: 87 [stem: *Melyr-*]. Type genus: *Melyris* Fabricius, 1775.

### Subfamily Rhadalinae

LeConte, 1861

Rhadalini J. L. LeConte, 1861: 194 [stem: *Rhadal-*]. Type genus: *Rhadalus* J. L. LeConte, 1852.
Haplocnémates Mulsant and Rey, 1868a: 181 [stem: *Aplocnem-*]. Type genus: *Aplocnemus* Stephens, 1830 [as *Haplocnemus*, unjustified emendation of type genus name by Agassiz (1846b: 29), not in prevailing usage]. Comment: original vernacular name available (Art. 11.7.2): first used in latinized form by Crowson (1964: 316, as Haplocneminae [incorrect stem formation]), generally accepted as in Majer (1987: 800, as Aplocnemini); incorrect original stem formation, not in prevailing usage.
Microjulistini Majer, 1987: 797, in key [stem: *Microjulist-*]. Type genus: *Microjulistus* Reitter, 1889.
Pelecophorini Majer, 1987: 797, in key [stem: *Pelecophor-*]. Type genus: *Pelecophora* Lepeletier and Audinet-Serville, 1825.

### Subfamily Melyrinae

Leach, 1815

Melyrides Leach, 1815: 87 [stem: *Melyr-*]. Type genus: *Melyris* Fabricius, 1775.

### Tribe Arthrobrachini

Majer, 1987

Arthrobrachini Majer, 1987: 793, in key [stem: *Arthrobrach-*]. Type genus: *Arthrobrachus* Solier, 1849.

### Tribe Astylini

Pic, 1929

Astylini Pic, 1929: 1 [stem: *Astyl-*]. Type genus: *Astylus* Laporte, 1836.

### Tribe Cerallini

Pic, 1929

Cerallini Pic, 1929: 13 [stem: *Cerall-*]. Type genus: *Cerallus* Jacquelin du Val, 1859.

### Tribe Melyrini

Leach, 1815

Melyrides Leach, 1815: 87 [stem: *Melyr-*]. Type genus: *Melyris* Fabricius, 1775. Comment: published April 1815; this family-group name was also used in the same year by Rafinesque (1815 [between April and 21 July]: 110, as Melyria).
Zygiidae Jakobson, 1911a: 687 [stem: *Zygi-*]. Type genus: *Zygia* Fabricius, 1775 [syn. of *Melyris* Fabricius, 1775].

### Subfamily Dasytinae

Laporte, 1840

Dasydites Laporte, 1840a: 280 [stem: *Dasyt-*]. Type genus: *Dasytes* Paykull, 1799.

### Tribe Chaetomalachiini

Majer, 1987

Chaetomalachiini Majer, 1987: 799, in key [stem: *Chaetomalachi-*]. Type genus: *Chaetomalachius* Kraatz, 1882.

### Tribe Danaceini

Thomson, 1859

Danacaeina C. G. Thomson, 1859: 108 [stem: *Danace-*]. Type genus: *Danacea* Laporte, 1838 [as *Danacaea*, incorrect subsequent spelling of type genus name, not in prevailing usage]. Comment: incorrect original stem formation, not in prevailing usage.
Amauronoinini Majer, 1987: 799, in key [stem: *Amauronioid-*]. Type genus: *Amauronioides* Champion, 1923 [syn. of *Pseudamauronia* Pic, 1915]. Comment: incorrect original stem formation, not in prevailing usage.
Danacaeomimini Majer, 1987: 799, in key [stem: *Danacaeomim-*]. Type genus: *Danacaeomimus* Champion, 1922.

### Tribe Dasytini

Laporte, 1840

Dasydites Laporte, 1840a: 280 [stem: *Dasyt-*]. Type genus: *Dasytes* Paykull, 1799. Comment: original vernacular name available (Art. 11.7.2): first used in latinized form by Agassiz (1846b: 117, as Dasytetoidae [incorrect stem formation]), generally accepted as in Pic (1937: 1, as Dasytinae); current spelling maintained (Art. 29.5): incorrect stem formation in prevailing usage (should be *Dasytet*-).
Colpothisidae Gistel, 1848: [11] [stem: *Colpoth-*]. Type genus: *Colpothis* Gistel, 1848 [syn. of *Dasytes* Paykull, 1799]. Comment: incorrect original stem formation, not in prevailing usage.
*Hénicopaires Mulsant and Rey, 1868a: 264 [stem: *Enicopod-*]. Type genus: *Enicopus* Stephens, 1830 [as *Henicopus*, unjustified emendation of type genus name by Agassiz (1846b: 138), not in prevailing usage]. Comment: original vernacular name unavailable (Art. 11.7.2): subsequently used in latinized form but not generally attributed to Mulsant and Rey (1868a); incorrect original stem formation, not in prevailing usage.
Henicopini Escalera, 1927: 7 [stem: *Enicopod-*]. Type genus: *Enicopus* Stephens, 1830 [as *Henicopus*, unjustified emendation of type genus name by Agassiz (1846b: 138), not in prevailing usage]. Comment: incorrect original stem formation, not in prevailing usage.

### Tribe Gietellini

Constantin and Menier, 1987

Gietellinae Constantin and Menier, 1987: 62 [stem: *Gietell-*]. Type genus: *Gietella* Constantin and Menier, 1987.

### Tribe Listrini

Majer, 1990

Listrini Majer, 1990: 371 [stem: *Listr-*]. Type genus: *Listrus* Motschulsky, 1859.

### Subfamily Malachiinae

Fleming, 1821

Malachiusidae Fleming, 1821: 50 [stem: *Malachi-*]. Type genus: *Malachius* Fabricius, 1775.

### Tribe Amalthocini

Majer, 2002

Amalthocinae Majer, 2002: 186 [stem: *Amalthoc-*]. Type genus: *Amalthocus* Fairmaire, 1886.

### Tribe Attalomimini

Majer, 1995

Attalomimidae Majer, 1995a: 378 [stem: *Attalomim-*]. Type genus: *Attalomimus* Wittmer, 1976.

### Tribe Carphurini

Champion, 1923

Carphurinae Champion, 1923: 2 [stem: *Carphur-*]. Type genus: *Carphurus* Erichson, 1840.

### Tribe Lemphini

Wittmer, 1976

Lemphini Wittmer, 1976: 427 [stem: *Lemph-*]. Type genus: *Lemphus* Erichson, 1840.

### Tribe Malachiini

Fleming, 1821

Malachiusidae Fleming, 1821: 50 [stem: *Malachi-*]. Type genus: *Malachius* Fabricius, 1775. Comment: incorrect original stem formation, not in prevailing usage.
Tamulidae Gistel, 1848: [11] [stem: *Tamul-*]. Type genus: *Tamulus* Gistel, 1848 [syn. of *Malachius* Fabricius, 1775].
Polycystophoridae Gistel, 1856a: 385 [stem: *Polycystophor-*]. Type genus: *Polycystophorus* Gistel, 1856 [this genus was included by Gistel in his new family Polycystophoridae along with one genus now classified in Cantharidae (*Malthinus* Latreille) and four genera of Malachiinae (*Anthocomus* Erichson, *Ebaeus* Erichson, *Charopus* Erichson and *Troglops* Erichson); several available species names were originally included in *Polycystophorus* (Gistel, 1856a: 385), we hereby select *Cantharis aeneus* Linnaeus, 1758 as the type species; **syn. nov.** of *Malachius* Fabricius, 1775]. Comment: **syn. nov.**
Apalochraires Mulsant and Rey, 1867b: 19 [stem: *Apalochr-*]. Type genus: *Apalochrus* Erichson, 1840. Comment: original vernacular name available (Art. 11.7.2): first used in latinized form and generally accepted as in Houlbert (1922a: 250, as Apalochrini).
*Anthocomates Mulsant and Rey, 1867b: 127 [stem: *Anthocom-*]. Type genus: *Anthocomus* Erichson, 1840. Comment: original vernacular name unavailable (Art. 11.7.2): not subsequently latinized.
*Troglopates Mulsant and Rey, 1867b: 281 [stem: *Troglop-*]. Type genus: *Troglops* Erichson, 1840. Comment: original vernacular name unavailable (Art. 11.7.2): not subsequently latinized.
Caulautaires Abeille de Perrin, 1890: 244 [stem: *Colot-*]. Type genus: *Colotes* Erichson, 1840 [as *Caulautes*, unjustified emendation of type genus name by Abeille de Perrin (1890), not in prevailing usage]. Comment: original vernacular name available (Art. 11.7.2): first used in latinized form by Bertkau (1891: 304, as Colotini), generally accepted as in Hansen (1996: 152, as Colotini); incorrect original stem formation, not in prevailing usage; Colotini Larsen, 1983 (type genus *Colotis* Hübner, 1819) does not appear to be available in Lepidoptera: Pieridae; if the Lepidoptera name is found to be available then this case is to be referred to the Commission to remove the homonymy (Art. 55.3.1).
Attalaires Abeille de Perrin, 1891: 364 [stem: *Attal-*]. Type genus: *Attalus* Erichson, 1840. Comment: original vernacular name available (Art. 11.7.2): first used in latinized form by Hatch (1962: 87, as Attalina), generally accepted as in Mayor (2007: 419, as Attalini).
Laiina Jakobson, 1911a: 688 [stem: *Lai-*]. Type genus: *Laius* Guérin-Méneville, 1838.
Illopina Jakobson, 1911a: 688 [stem: *Illop-*]. Type genus: *Illops* Erichson, 1840.
Paratini Portevin, 1931: 426, in key [stem: *Paratin-*]. Type genus: *Paratinus* Abeille de Perrin, 1891 [syn. of *Apalochrus* Erichson, 1840]. Comment: the correct spelling Paratinini was used in the same work on page 442.
Ebaeini Portevin, 1931: 426, in key [stem: *Ebae-*]. Type genus: *Ebaeus* Erichson, 1840.
Collopina Hatch, 1962: 88, in key [stem: *Collop-*]. Type genus: *Collops* Erichson, 1840.

### Tribe Pagurodactylini

Constantin, 2001

Pagurodactylinae Constantin, 2001: 6 [stem: *Pagurodactyl-*]. Type genus: *Pagurodactylus* Gorham, 1900.

### Melyridae incertae sedis

Elosomatidae Jakobson, 1915: 993 [stem: *Elosomat-*]. Type genus: *Elosoma* Motschulsky, 1845. Comment: transferred from Salpingidae by Zerche (2004), correct placement within the family uncertain.

### Superfamily Cucujoidea

Latreille, 1802

Cucujipes Latreille, 1802: 210 [stem: *Cucuj-*]. Type genus: *Cucujus* Fabricius, 1775 [placed on the Official List of Generic Names in Zoology (ICZN 1994a)]. Comment: First Reviser (Cucujoidea Latreille, 1802 vs Erotyloidea Latreille, 1802 vs Nitiduloidea Latreille, 1802) not determined, current usage maintained.

### †Family Parandrexidae

Kirejtshuk, 1994

Parandrexidae Kirejtshuk, 1994: 57 [stem: *Parandrex-*]. Type genus: *Parandrexis* Martynov, 1926. Comment: current spelling maintained (Art. 29.5): incorrect stem formation in prevailing usage (should be *Parandrexe*-).

### †Family Sinisilvanidae

Hong, 2002

Sinisilvanidae Hong, 2002: 132 [stem: *Sinisilvan-*]. Type genus: *Sinisilvana* Hong, 2002.

### Family Boganiidae

Sen Gupta and Crowson, 1966

Boganiidae Sen Gupta and Crowson, 1966: 63 [stem: *Bogani-*]. Type genus: *Boganium* Sen Gupta and Crowson, 1966.

### Subfamily Paracucujinae

Endrödy-Younga and Crowson, 1986

Paracucujinae Endrödy-Younga and Crowson, 1986: 255 [stem: *Paracucuj-*]. Type genus: *Paracucujus* Sen Gupta and Crowson, 1966.
Athertoniini Crowson, 1990: 91 [stem: *Athertoni-*]. Type genus: *Athertonium* Crowson, 1990.

### Subfamily Boganiinae

Sen Gupta and Crowson, 1966

Boganiidae Sen Gupta and Crowson, 1966: 63 [stem: *Bogani-*]. Type genus: *Boganium* Sen Gupta and Crowson, 1966.

### Family Byturidae

Gistel, 1848

Byturidae Gistel, 1848: [3] [stem: *Bytur-*]. Type genus: *Byturus* Latreille, 1797.

### Subfamily Platydascillinae

Pic, 1914

Platydascillidae Pic, 1914: 20 [stem: *Platydascill-*]. Type genus: *Platydascillus* Everts, 1909.

### Subfamily Byturinae

Gistel, 1848

Byturidae Gistel, 1848: [3] [stem: *Bytur-*]. Type genus: *Byturus* Latreille, 1797. Comment: family-group name previously attributed to Jacquelin du Val (1858: 211).

### Family Helotidae

Chapuis, 1876

Hélotides Chapuis, 1876: 15 [stem: *Helot-*]. Type genus: *Helota* W. S. MacLeay, 1825. Comment: published before 1 May 1876; original vernacular name available (Art. 11.7.2): used in latinized form by several authors subsequently, generally accepted as in Lawrence and Newton (1995: 875, as “Helotidae Reitter, 1876/Chapuis, 1876”); this family-group name was also used in the same year by Reitter (1876a [before 15 August]: 5, as Helotidae); this is a junior homonym of Helotidae Adams et al., 1854 (type genus *Helotes* Cuvier, 1829) in Pisces, this case is to be referred to the Commission to remove the homonymy (Art. 55.3.1).

### Family Protocucujidae

Crowson, 1954

Protocucujidae Crowson, 1954: 60 [stem: *Protocucuj-*]. Type genus: *Protocucujus* Crowson, 1954.

### Family Sphindidae

Jacquelin du Val, 1860

Sphindides Jacquelin du Val, 1860: 224 [stem: *Sphind-*]. Type genus: *Sphindus* Dejean, 1821 [placed on the Official List of Generic Names in Zoology (ICZN 1997)]. Comment: this name (as Sphindidae Jacquelin du Val, [1861]) was placed on the Official List of Family-Group Names in Zoology and ruled under the plenary power that this and other family-group names based on *Sphindus* Dejean, 1821 are to be given precedence over Aspidiphoridae Kiesenwetter, 1877 (1859) and other family-group names based on *Aspidiphorus* Dejean, 1821 whenever their type genera are placed in the same family-group taxon (ICZN 1997).

### Subfamily Protosphindinae

Sen Gupta and Crowson, 1979

Protosphindinae Sen Gupta and Crowson, 1979: 179, in key [stem: *Protosphind-*]. Type genus: *Protosphindus* Sen Gupta and Crowson, 1979.

### Subfamily Odontosphindinae

Sen Gupta and Crowson, 1979

Odontosphindini Sen Gupta and Crowson, 1979: 180, in key [stem: *Odontosphind-*]. Type genus: *Odontosphindus* Sen Gupta and Crowson, 1979.

### Subfamily Sphindiphorinae

Sen Gupta and Crowson, 1979

Sphindiphorini Sen Gupta and Crowson, 1979: 180, in key [stem: *Sphindiphor-*]. Type genus: *Sphindiphorus* Sen Gupta and Crowson, 1979.

### Subfamily Sphindinae

Jacquelin du Val, 1860

Coniporina C. G. Thomson, 1859: 90 [stem: *Conipor-*]. Type genus: *Coniporus* C. G. Thomson, 1859 [placed on the Official Index of Rejected and Invalid Generic Names in Zoology (ICZN 1997); syn. of *Aspidiphorus* Dejean, 1821]. Comment: permanently invalid (Art. 39): based on suppressed type genus.
Sphindides Jacquelin du Val, 1860: 224 [stem: *Sphind-*]. Type genus: *Sphindus* Dejean, 1821 [placed on the Official List of Generic Names in Zoology (ICZN 1997)]. Comment: original vernacular name available (Art. 11.7.2): first used in latinized form by Kiesenwetter (1877: 7, as Sphindini), generally accepted as in Lawrence and Newton (1995: 872, as Sphindidae).
Aspidiphoridae Kiesenwetter, 1877: 198 [stem: *Aspidiphor-*]. Type genus: *Aspidiphorus* Dejean, 1821 [*Arpidiphorus*, the original spelling of the type genus, as well as *Aspidophorus* Agassiz, 1846 were placed on the Official Index of Rejected and Invalid Generic Names in Zoology and *Aspidiphorus* was placed on the Official List of Generic Names in Zoology (ICZN 1997)]. Comment: this name (as Aspidiphoridae Kiesenwetter, 1877 (1859)) was placed on the Official List of Family-Group Names in Zoology and ruled under the plenary power that this and other family-group names based on *Aspidiphorus* are not to be given priority over Sphindidae and other family-group names based on *Sphindus* Dejean, 1821 whenever their type genera are placed in the same family-group taxon (ICZN 1997).
Eurysphindinae Sen Gupta and Crowson, 1979: 180, in key [stem: *Eurysphind-*]. Type genus: *Eurysphindus* J. L. LeConte, 1878.

### Family Biphyllidae

LeConte, 1861

Diphyllidae J. L. LeConte, 1861: 105 [stem: *Biphyll-*]. Type genus: *Biphyllus* Dejean, 1821 [as *Diphyllus*, unjustified emendation of type genus name by Agassiz (1846b: 47), not in prevailing usage]. Comment: incorrect original stem formation, not in prevailing usage (see Cline and Shockley 2010).

### Family Erotylidae

Latreille, 1802

Erotilenae Latreille, 1802: 233 [stem: *Erotyl-*]. Type genus: *Erotylus* Fabricius, 1775.

### Subfamily Xenoscelinae

Ganglbauer, 1899

Xenoscelini Ganglbauer, 1899: 649 [stem: *Xenoscel-*]. Type genus: *Xenoscelis* Wollaston, 1864. Comment: current spelling maintained (Art. 29.3.1.1): incorrect stem formation in prevailing usage (should be *Xenoscelid*-).
Eicolyctini Vogt, 1967: 103 [stem: *Eicolyct-*]. Type genus: *Eicolyctus* J. R. Sahlberg, 1919 [syn. of *Zavaljus* Reitter, 1880].
Loberonothini Sen Gupta and Crowson, 1969a: 127 [stem: *Loberonoth-*]. Type genus: *Loberonotha* Sen Gupta and Crowson, 1969.

### Subfamily Pharaxonothinae

Crowson, 1952

Setariini Casey, 1900: 77 [stem: *Setari-*]. Type genus: *Setaria* Mulsant and Rey, 1863 [preoccupied genus name, not *Setaria* Viborg, 1795 [Vermes], not *Setaria* Oken, 1815 [Vermes], not *Setaria* Blyth, 1844 [Aves]; syn. of *Setariola* Jakobson, 1915]. Comment: permanently invalid (Art. 39): based on preoccupied type genus.
Pharaxonothinae Crowson, 1952: 127 [stem: *Pharaxonoth-*]. Type genus: *Pharaxonotha* Reitter, 1875. Comment: First Reviser found (Pharaxonothinae Crowson, 1952 vs Setariolinae Crowson, 1952) is Leschen (2003: 35).
Setariolinae Crowson, 1952: 127 [stem: *Setariol-*]. Type genus: *Setariola* Jakobson, 1915.
Xenoscelinini Sen Gupta and Crowson, 1971: 5, in key [stem: *Xenoscelin-*]. Type genus: *Xenoscelinus* Grouvelle, 1910 [syn. of *Cathartocryptus* Sharp, 1886].

### Subfamily Loberinae

Bruce, 1951

Loberinae Bruce, 1951: 4 [stem: *Lober-*]. Type genus: *Loberus* J. L. LeConte, 1861. Comment: name proposed after 1930 without description or bibliographic reference to such a description (Art. 13.1), however available because it was used as valid before 2000 as in Sen Gupta (1968a: 1, as Loberini) and was not rejected by an author who, between 1961 and 1999, applied Article 13 of the then current edition of the Code (see Art. 13.2.1).

### Subfamily Languriinae

Hope, 1840

Languiridae Hope, 1840a: 190 [stem: *Languri-*]. Type genus: *Languria* Latreille, 1802 [as *Languiria*, incorrect subsequent spelling of type genus name, not in prevailing usage].

### Tribe Hapalipini

Leschen, 2003

Hapalipini Leschen, 2003: 38 [stem: *Hapalip-*]. Type genus: *Hapalips* Reitter, 1877.

### Tribe Languriini

Hope, 1840

Languiridae Hope, 1840a: 190 [stem: *Languri-*]. Type genus: *Languria* Latreille, 1802 [as *Languiria*, incorrect subsequent spelling of type genus name, not in prevailing usage]. Comment: incorrect original stem formation, not in prevailing usage; family-group name previously attributed to Crotch (1873c) by Pakaluk et al. (1994) and subsequent authors; usage of «Wiedeman, 1823» as the correct author and year for this family-group name in the literature is incorrect (see Pakaluk et al. 1994).
Cladoxeninae Arrow, 1925: 166 [stem: *Cladoxen-*]. Type genus: *Cladoxena* Motschulsky, 1866.

### Tribe Thallisellini

Sen Gupta, 1968

Thallisellini Sen Gupta, 1968b: 470 [stem: *Thallisell-*]. Type genus: *Thallisella* Crotch, 1876.

### Subfamily Cryptophilinae

Casey, 1900

Cryptophilini Casey, 1900: 77 [stem: *Cryptophil-*]. Type genus: *Cryptophilus* Reitter, 1874.

### Tribe Cryptophilini

Casey, 1900

Cryptophilini Casey, 1900: 77 [stem: *Cryptophil-*]. Type genus: *Cryptophilus* Reitter, 1874.

### Tribe Empocryptini

Leschen, 2003

Empocryptini Leschen, 2003: 41 [stem: *Empocrypt-*]. Type genus: *Empocryptus* Sharp, 1900.

### Tribe Toramini

Sen Gupta, 1967

Toraminae Sen Gupta, 1967: 168 [stem: *Toram-*]. Type genus: *Toramus* Grouvelle, 1916.

### Subfamily Erotylinae

Latreille, 1802

Erotilenae Latreille, 1802: 233 [stem: *Erotyl-*]. Type genus: *Erotylus* Fabricius, 1775.

### Tribe Dacnini

Gistel, 1848

*Engidites Latreille, 1829a: 506 [stem: *Eng-*]. Type genus: *Engis* Paykull, 1800 [syn. of *Dacne* Latreille, 1797]. Comment: family-group name unavailable (Art. 11.7.1.1): not based on a genus used as valid at the time.
Dacneidae Gistel, 1848: [3] [stem: *Dacn-*]. Type genus: *Dacne* Latreille, 1797. Comment: incorrect original stem formation, not in prevailing usage.
Cryptodacnini Sen Gupta, 1970: 101, in key [stem: *Cryptodacn-*]. Type genus: *Cryptodacne* Sharp, 1878.

### Tribe Encaustini

Crotch, 1876

Engidae W. S. MacLeay, 1825: 40 [stem: *Engid-*]. Type genus: *Engis* sensu W. S. MacLeay, 1825 [not *Engis* Paykull, 1800; syn. of *Encaustes* Lacordaire, 1842]. Comment: based on a misidentified type genus, name treated here as invalid until an application is submitted to the Commission to suppress it for the Principle of Priority (Art. 65.2.1).
Encaustini Crotch, 1876: 476 [stem: *Encaust-*]. Type genus: *Encaustes* Lacordaire, 1842. Comment: published after February 1876; this family-group name was also used in the same year by Chapuis (1876 [before 1 May]: 46, as Encaustites).

### Tribe Erotylini

Latreille, 1802

Erotilenae Latreille, 1802: 233 [stem: *Erotyl-*]. Type genus: *Erotylus* Fabricius, 1775. Comment: incorrect original stem formation, not in prevailing usage.

### Tribe Megalodacnini

Sen Gupta, 1970

Megalodacnini Sen Gupta, 1970: 100, in key [stem: *Megalodacn-*]. Type genus: *Megalodacne* Crotch, 1873.

### Tribe Tritomini

Curtis, 1834

Tritomidae Curtis, 1834: plate 498 [stem: *Tritom-*]. Type genus: *Tritoma* Fabricius, 1775 [placed on the Official List of Generic Names in Zoology (ICZN 1994a)].
Triplacinae Erichson, 1847a: 179 [stem: *Triplac-*]. Type genus: *Triplax* Herbst, 1793.
Renaniinae Chûjô, 1941: 10 [stem: *Renani-*]. Type genus: *Renania* Lewis, 1887.
Cyrtotriplacina Chûjô, 1969: 201 [stem: *Cyrtotriplac-*]. Type genus: *Cyrtotriplax* Crotch, 1873 [syn. of *Tritoma* Fabricius, 1775].

### Family Monotomidae

Laporte, 1840

Monotomites Laporte, 1840b: 377 [stem: *Monotom-*]. Type genus: *Monotoma* Herbst, 1793.

### Subfamily Rhizophaginae

Redtenbacher, 1845

Rhizophagi L. Redtenbacher, 1845: 125 [stem: *Rhizophag-*]. Type genus: *Rhizophagus* Herbst, 1793 [the original spelling *Ryzophagus* was placed on the Official Index of Rejected and Suppressed Generic Names in Zoology, *Rhizophagus* was considered the correct original spelling of the genus and placed on the Official List of Generic Names in Zoology (ICZN 1995b)].

### Subfamily Monotominae

Laporte, 1840

Monotomites Laporte, 1840b: 377 [stem: *Monotom-*]. Type genus: *Monotoma* Herbst, 1793.

### Tribe Europini

Sen Gupta, 1988

Europini Sen Gupta, 1988: 14, in key [stem: *Europ-*]. Type genus: *Europs* Wollaston, 1854.

### Tribe Lenacini

Crowson, 1952

Lenacinae Crowson, 1952: 121 [stem: *Lenac-*]. Type genus: *Lenax* Sharp, 1877.

### Tribe Monotomini

Laporte, 1840

Monotomites Laporte, 1840b: 377 [stem: *Monotom-*]. Type genus: *Monotoma* Herbst, 1793 [*nomen protectum*; this genus name is a junior homonym of *Monotoma* Panzer, 1792 *nomen oblitum*; we provide references to support the conservation of *Monotoma* Herbst, 1793 as the valid name for this genus (Art. 23.9.1) (see Appendix 1)]. Comment: original vernacular name available (Art. 11.7.2): first used in latinized form by Agassiz (1846b: 238, as Monotomoidae), generally accepted as in Pakaluk et al. (1994: 230, as Monotomidae).

### †Tribe Rhizophtomini

Kirejtshuk and Azar, 2009

Rhizophtominae Kirejtshuk and Azar, 2009: 123 [stem: *Rhizophtom-*]. Type genus: *Rhizophtoma* Kirejtshuk and Azar, 2009.

### Tribe Thionini

Crowson, 1952

Thioninae Crowson, 1952: 121 [stem: *Thion-*]. Type genus: *Thione* Sharp, 1899.

### Family Hobartiidae

Sen Gupta and Crowson, 1966

Hobartiini Sen Gupta and Crowson, 1966: 65, in key [stem: *Hobarti-*]. Type genus: *Hobartius* Sen Gupta and Crowson, 1966.

### Family Cryptophagidae

Kirby, 1826

Cryptophagidae Kirby, 1826: 504 [stem: *Cryptophag-*]. Type genus: *Cryptophagus* Herbst, 1792.

### Subfamily Cryptophaginae

Kirby, 1826

Cryptophagidae Kirby, 1826: 504 [stem: *Cryptophag-*]. Type genus: *Cryptophagus* Herbst, 1792.

### Tribe Caenoscelini

Casey, 1900

Caenoscelini Casey, 1900: 103 [stem: *Caenoscel-*]. Type genus: *Caenoscelis* C. G. Thomson, 1863. Comment: First Reviser found (Caenoscelini Casey, 1900 vs Sternodeini Casey, 1900) is Leschen (1996: 607); current spelling maintained (Art. 29.3.1.1): incorrect stem formation in prevailing usage (should be *Caenoscelid*-).
Sternodeini Casey, 1900: 103 [stem: *Sternode-*]. Type genus: *Sternodea* Reitter, 1875.

### Tribe Cryptophagini

Kirby, 1826

Ips Latreille, 1802: 129 [stem: *Ip-*]. Type genus: *Ips* sensu Latreille, 1802 [not *Ips* DeGeer, 1775; syn. of *Cryptophagus* Herbst, 1792]. Comment: although the spelling of the family-group names is identical to the spelling of the type genus, it is clear, based on other evidence from the same work, that Latreille was using it as a family-group name (see Lawrence and Newton 1995: 913); based on a misidentified type genus; name treated here as invalid until an application is submitted to the Commission to suppress it for the Principles of Priority and Homonymy (Art. 65.2.1); also see Ipini Bedel, 1888 in Curculionidae: Scolytinae.
Cryptophagidae Kirby, 1826: 504 [stem: *Cryptophag-*]. Type genus: *Cryptophagus* Herbst, 1792 [the original spelling *Kryptophagus* was placed on the Official Index of Rejected and Suppressed Generic Names in Zoology, *Cryptophagus* was considered the correct original spelling of the genus and placed on the Official List of Generic Names in Zoology (ICZN 1995b)].
Telmatophilides Jacquelin du Val, 1858: 209 [stem: *Telmatophil-*]. Type genus: *Telmatophilus* Heer, 1841. Comment: original vernacular name available (Art. 11.7.2): first used in latinized form by J. L. LeConte (1861: 98, as Telmatophilini), generally accepted as in Telnov (2004: 73, as Telmatophilini).
Antherophagi J. L. LeConte, 1861: 98 [stem: *Antherophag-*]. Type genus: *Antherophagus* Dejean, 1821.
Paramecosomina Reitter, 1875: 4 [stem: *Paramecosomat-*]. Type genus: *Paramecosoma* Curtis, 1833. Comment: incorrect original stem formation, not in prevailing usage.
Catopochrotidae Reitter, 1889a: 289 [stem: *Catopochrot-*]. Type genus: *Catopochrotus* Reitter, 1889.
Emphyli Casey, 1900: 86 [stem: *Emphyl-*]. Type genus: *Emphylus* Erichson, 1846 [syn. of *Spavius* Motschulsky, 1844].
Spaniophaeni Casey, 1900: 86 [stem: *Spaniophaen-*]. Type genus: *Spaniophaenus* Reitter, 1875.

### Tribe Picrotini

Crowson, 1980

Picrotini Crowson, 1980: 283 [stem: *Picrot-*]. Type genus: *Picrotus* Sharp, 1886.
*Cryptosomatulini Crowson, 1980: 284 [stem: *Cryptosomatul-*]. Type genus: *Cryptosomatula* Bruce, 1940. Comment: unavailable family-group name, proposed after 1930 without description or bibliographic reference to such a description (Art. 13.1).
Cryptosomatulini Leschen, 1996: 605 [stem: *Cryptosomatul-*]. Type genus: *Cryptosomatula* Bruce, 1940.

### Subfamily Atomariinae

LeConte, 1861

Atomariini J. L. LeConte, 1861: 99 [stem: *Atomari-*]. Type genus: *Atomaria* Stephens, 1829.

### Tribe Atomariini

LeConte, 1861

Atomariini J. L. LeConte, 1861: 99 [stem: *Atomari-*]. Type genus: *Atomaria* Stephens, 1829.
Ephistemini Casey, 1900: 104 [stem: *Ephistem-*]. Type genus: *Ephistemus* Stephens, 1829.
*Salltiini Crowson, 1980: 284 [stem: *Sallti-*]. Type genus: *Salltius* Broun, 1893. Comment: unavailable family-group name, proposed after 1930 without description or bibliographic reference to such a description (Art. 13.1).
*Atomaroidini Lyubarsky, 1998: 71 [stem: *Atomaroid-*]. Type genus: *Atomaroides* Lyubarsky, 1989. Comment: unavailable family-group name, proposed after 1930 without description or bibliographic reference to such a description (Art. 13.1).

### Tribe Cryptafricini

Leschen, 1996

Cryptafricini Leschen, 1996: 623 [stem: *Cryptafric-*]. Type genus: *Cryptafricus* Leschen, 1996.
*Microphagini Lyubarsky, 1998: 39, in key [stem: *Microphag-*]. Type genus: *Microphagus* Leschen, 1996. Comment: unavailable family-group name, proposed after 1930 without description or bibliographic reference to such a description (Art. 13.1).
*Scytomariini Lyubarsky, 1998: 71 [stem: *Scytomari-*]. Type genus: *Scytomaria* Lyubarsky, 1998 [syn. of *Anitamaria* Leschen, 1996]. Comment: unavailable family-group name, proposed after 1930 without description or bibliographic reference to such a description (Art. 13.1).

### Tribe Hypocoprini

Reitter, 1879

Hypocoprini Reitter, 1879: 74 [stem: *Hypocopr-*]. Type genus: *Hypocoprus* Motschulsky, 1839.
*Alfieriellinae Crowson, 1980: 282 [stem: *Alfieriell-*]. Type genus: *Alfieriella* Wittmer, 1935. Comment: unavailable family-group name, proposed after 1930 without description or bibliographic reference to such a description (Art. 13.1).
*Hypophagina Lyubarsky, 1998: 71 [stem: *Hypophag-*]. Type genus: *Hypophagus* Lyubarsky, 1989. Comment: unavailable family-group name, proposed after 1930 without description or bibliographic reference to such a description (Art. 13.1).

### Family Agapythidae

Sen Gupta and Crowson, 1969

Agapythinae Sen Gupta and Crowson, 1969b: 579, in key [stem: *Agapyth-*]. Type genus: *Agapytho* Broun, 1921.

### Family Priasilphidae

Crowson, 1973

Priasilphinae Crowson, 1973a: 56 [stem: *Priasilph-*]. Type genus: *Priasilpha* Broun, 1893.

### Family Phloeostichidae

Reitter, 1911

Phloeostichini Reitter, 1911: 44 [stem: *Phloeostich-*]. Type genus: *Phloeostichus* W. Redtenbacher, 1842.
Hymaeinae Sen Gupta and Crowson, 1966: 65, in key [stem: *Hymae-*]. Type genus: *Hymaea* Pascoe, 1869.

### Family Silvanidae

Kirby, 1837

Sylvanidae Kirby, 1837: 110 [stem: *Silvan-*]. Type genus: *Silvanus* Latreille, 1804.

### Subfamily Brontinae

Blanchard, 1845

Brontites Blanchard, 1845b: 134 [stem: *Bront-*]. Type genus: *Brontes* Fabricius, 1801.

### Tribe Brontini

Blanchard, 1845

Brontites Blanchard, 1845b: 134 [stem: *Bront-*]. Type genus: *Brontes* Fabricius, 1801. Comment: published before 11 June 1845; original vernacular name available (Art. 11.7.2): first used in latinized form by Erichson (1845 [before 31 June]: 304, as Brontini), treated as available by Lawrence and Newton (1995: 876).
Dendrophagidae Gistel, 1848: [8] [stem: *Dendrophag-*]. Type genus: *Dendrophagus* Schönherr, 1809.
Uleiotaeidae Gistel, 1848: [10] [stem: *Uleiot-*]. Type genus: *Uleiota* Latreille, 1797. Comment: incorrect original stem formation, not in prevailing usage.

### Tribe Telephanini

LeConte, 1861

Telephanidae J. L. LeConte, 1861: 96 [stem: *Telephan-*]. Type genus: *Telephanus* Erichson, 1845.
Pseudophanini J. L. LeConte, 1861: 96 [stem: *Pseudophan-*]. Type genus: *Pseudophanus* J. L. LeConte, 1859 [syn. of *Cryptamorpha* Wollaston, 1854].
Psammoecini Reitter, 1879: 74 [stem: *Psammoec-*]. Type genus: *Psammoecus* Latreille, 1829.
Cryptamorphini Casey, 1884: 102 [stem: *Cryptamorph-*]. Type genus: *Cryptamorpha* Wollaston, 1854.

### Subfamily Silvaninae

Kirby, 1837

Sylvanidae Kirby, 1837: 110 [stem: *Silvan-*]. Type genus: *Silvanus* Latreille, 1804. Comment: incorrect original stem formation, not in prevailing usage.

### Family Cucujidae

Latreille, 1802

Cucujipes Latreille, 1802: 210 [stem: *Cucuj-*]. Type genus: *Cucujus* Fabricius, 1775 [placed on the Official List of Generic Names in Zoology (ICZN 1994a)].
*Biophloces Motschulsky, 1849: 60 [stem: *Biophloe-*]. Type genus: *Biophloeus* Dejean, 1835 [*nomen oblitum*; this genus name is a senior synonym of the well-established name *Pediacus* Shuckard, 1839 *nomen protectum*; however since *Biophloeus* Dejean has not been used as valid after 1899 to our knowledge, we provide references to support the conservation of *Pediacus* as the valid name for this genus (Art. 23.9.1) (see Appendix 1)]. Comment: original vernacular name unavailable (Art. 11.7.2): not subsequently latinized.
Earophilidae Gistel, 1856a: 375 [stem: *Earophil-*]. Type genus: *Earophilus* Gistel, 1856 [syn. of *Cucujus* Fabricius, 1775].

### Family Myraboliidae

Lawrence and Britton, 1991

Myraboliinae Lawrence and Britton, 1991: 650 [stem: *Myraboli-*]. Type genus: *Myrabolia* Reitter, 1876.

### Family Cavognathidae

Sen Gupta and Crowson, 1966

Cavognathinae Sen Gupta and Crowson, 1966: 65, in key [stem: *Cavognath-*]. Type genus: *Cavognatha* Crowson, 1964 [syn. of *Taphropiestes* Reitter, 1875].

### Family Lamingtoniidae

Sen Gupta and Crowson, 1969

Lamingtoniidae Sen Gupta and Crowson, 1969a: 125 [stem: *Lamingtoni-*]. Type genus: *Lamingtonium* Sen Gupta and Crowson, 1969.

### Family Passandridae

Blanchard, 1845

Passandrites Blanchard, 1845b: 134 [stem: *Passandr-*]. Type genus: *Passandra* Dalman, 1817. Comment: published before 11 June 1845; original vernacular name available (Art. 11.7.2): first used in latinized form by Erichson (1845 [before June 31]: 304, as Passandrini), treated as available by Lawrence and Newton (1995: 876).
Ancistriinae Sharp, 1899b: 541 [stem: *Ancistri-*]. Type genus: *Ancistria* Erichson, 1845.
Catogenini Grouvelle, 1916: 6 [stem: *Catogen-*]. Type genus: *Catogenus* Westwood, 1830.
Passandrellini Grouvelle, 1916: 6 [stem: *Passandrell-*]. Type genus: *Passandrella* Grouvelle, 1916.
Scalidiini Grouvelle, 1916: 6 [stem: *Scalidi-*]. Type genus: *Scalidia* Erichson, 1845.
Laemotmetini Kessel, 1921: 35 [stem: *Laemotmet-*]. Type genus: *Laemotmetus* Gerstaecker, 1871 [syn. of *Aulonosoma* Motschulsky, 1858].

### Family Phalacridae

Leach, 1815

Phalacrurida Leach, 1815: 116 [stem: *Phalacr-*]. Type genus: *Phalacrus* Paykull, 1800.

### Subfamily Phaenocephalinae

Matthews, 1899

Phaenocephalidae A. Matthews, 1899: 205 [stem: *Phaenocephal-*]. Type genus: *Phaenocephalus* Wollaston, 1873.

### Subfamily Phalacrinae

Leach, 1815

Phalacrurida Leach, 1815: 116 [stem: *Phalacr-*]. Type genus: *Phalacrus* Paykull, 1800. Comment: incorrect original stem formation, not in prevailing usage.
Idiobiidae Gistel, 1856a: 383 [stem: *Idiobi-*]. Type genus: *Idiobius* Gistel, 1856 [syn. of *Olibrus* Erichson, 1845].
Eustilbini Guillebeau, 1892: 149 [stem: *Eustilb-*]. Type genus: *Eustilbus* Sharp, 1888.
Olibrini Guillebeau, 1892: 147 [stem: *Olibr-*]. Type genus: *Olibrus* Erichson, 1845.
Tolyphini Guillebeau, 1892: 147 [stem: *Tolyph-*]. Type genus: *Tolyphus* Erichson, 1845.
Biophytini Guillebeau, 1894: 276, in key [stem: *Biophyt-*]. Type genus: *Biophytus* Guillebeau, 1894.
Megapalpini Guillebeau, 1894: 278, in key [stem: *Megapalp-*]. Type genus: *Megapalpus* Guillebeau, 1893 [preoccupied genus name, not *Megapalpus* Macquart, 1834 [Diptera]; syn. of *Megistopalpus* Guillebeau, 1895]. Comment: permanently invalid (Art. 39): based on preoccupied type genus.
Ochrolitini Guillebeau, 1894: 278, in key [stem: *Ochrolit-*]. Type genus: *Ochrolitus* Sharp, 1889.
Stilbini Jakobson, 1915: 948 [stem: *Stilb-*]. Type genus: *Stilbus* Seidlitz, 1872.

### Family Propalticidae

Crowson, 1952

Propalticidae Crowson, 1952: 117 [stem: *Propaltic-*]. Type genus: *Propalticus* Sharp, 1879.

### Family Laemophloeidae

Ganglbauer, 1899

Laemophloeini Ganglbauer, 1899: 606 [stem: *Laemophloe-*]. Type genus: *Laemophloeus* Dejean, 1835.
Nartheciinae Grouvelle, 1908: 453 [stem: *Nartheci-*]. Type genus: *Narthecius* J. L. LeConte, 1861.

### Family Tasmosalpingidae

Lawrence and Britton, 1991

Tasmosalpinginae Lawrence and Britton, 1991: 651 [stem: *Tasmosalping-*]. Type genus: *Tasmosalpingus* Lea, 1918.

### Family Cyclaxyridae

Gimmel, Leschen and Ślipiński, 2009

*Cyclaxyrinae Crowson, 1984: 259 [stem: *Cyclaxyr-*]. Type genus: *Cyclaxyra* Broun, 1893 [placed on the Official List of Generic Names in Zoology (ICZN 1988a)]. Comment: unavailable family-group name, proposed after 1930 without description or bibliographic reference to such a description (Art. 13.1).
Cyclaxyridae Gimmel et al., 2009: 513 [stem: *Cyclaxyr-*]. Type genus: *Cyclaxyra* Broun, 1893 [placed on the Official List of Generic Names in Zoology (ICZN 1988a)]. Comment: although this family-group name was used several times before 2009, all previous uses of the name were unavailable (see Gimmel et al. 2009: 512, 513).

### Family Kateretidae

Kirby, 1837

Catheretidae Kirby, 1837: 107 [stem: *Kateret-*]. Type genus: *Kateretes* Herbst, 1793 [as *Catheretes*, incorrect subsequent spelling of type genus name, not in prevailing usage; *Kateretes* Herbst, 1793 placed on Official List of Generic Names in Zoology (ICZN 1999b)]. Comment: incorrect original stem formation, not in prevailing usage; Kateretidae chosen as correct spelling of the family-group name, given precedence over Brachypteridae Erichson, 1845 and placed on Official List of Family-Group Names in Zoology (ICZN 1999b, as Kateretidae Erichson in Agassiz, 1846); discovery of the older available name proposed by Kirby (1837) removes priority problems with Brachypterinae.
Brachypterinae Erichson, 1845: 125 [stem: *Brachypter-*]. Type genus: *Brachypterus* Kugelann, 1794 [placed on the Official List of Generic Names in Zoology (ICZN 1999b)]. Comment: placed on Official List of Family-Group Names in Zoology (ICZN 1999b).
Cercidae Desmarest, 1851: 291 [stem: *Cerc-*]. Type genus: *Cercus* Latreille, 1797 [syn. of *Kateretes* Herbst, 1793].

### Family Nitidulidae

Latreille, 1802

Nitidulariae Latreille, 1802: 131 [stem: *Nitidul-*]. Type genus: *Nitidula* Fabricius, 1775. Comment: the classification of Nitidulidae follows Kirejtshuk (2008).

### Subfamily Calonecrinae

Kirejtshuk, 1982

Calonecrinae Kirejtshuk, 1982: 117 [stem: *Calonecr-*]. Type genus: *Calonecrus* J. Thomson, 1857.

### Subfamily Maynipeplinae

Kirejtshuk, 1998

Maynipeplinae Kirejtshuk, 1998b: 540 [stem: *Maynipepl-*]. Type genus: *Maynipeplus* Kirejtshuk, 1998.

### Subfamily Epuraeinae

Kirejtshuk, 1986

Epuraeini Kirejtshuk, 1986: 27 [stem: *Epurae-*]. Type genus: *Epuraea* Erichson, 1843.

### Tribe Epuraeini

Kirejtshuk, 1986

Epuraeini Kirejtshuk, 1986: 27 [stem: *Epurae-*]. Type genus: *Epuraea* Erichson, 1843.

### Tribe Taenioncini

Kirejtshuk, 1998

Taenioncini Kirejtshuk, 1998a: 322 [stem: *Taenionc-*]. Type genus: *Taenioncus* Kirejtshuk, 1984.

### Subfamily Carpophilinae

Erichson, 1842

Carpophilinae Erichson, 1842: 148 [stem: *Carpophil-*]. Type genus: *Carpophilus* Stephens, 1829.

### Subfamily Amphicrossinae

Kirejtshuk, 1986

Amphicrossini Kirejtshuk, 1986: 28 [stem: *Amphicross-*]. Type genus: *Amphicrossus* Erichson, 1843.

### Subfamily Meligethinae

Thomson, 1859

Meligethina C. G. Thomson, 1859: 67 [stem: *Meligeth-*]. Type genus: *Meligethes* Stephens, 1830.

### Subfamily Nitidulinae

Latreille, 1802

Nitidulariae Latreille, 1802: 131 [stem: *Nitidul-*]. Type genus: *Nitidula* Fabricius, 1775.

### Tribe Cychramini

Gistel, 1848

Cychramidae Gistel, 1848: [4] [stem: *Cychram-*]. Type genus: *Cychramus* Kugelann, 1794.

### Tribe Cychramptodini

Kirejtshuk and Lawrence, 1992

Cychramptodini Kirejtshuk and Lawrence, 1992: 29, in key [stem: *Cychramptod-*]. Type genus: *Cychramptodes* Reitter, 1878.

### Tribe Cyllodini

Everts, 1898

Strongylinae Erichson, 1843: 227 [stem: *Strongyl-*]. Type genus: *Strongylus* Herbst, 1792 [preoccupied genus name, not *Strongylus* O. F. Müller, 1780 [Nematoda]; syn. of *Cyllodes* Erichson, 1843]. Comment: permanently invalid (Art. 39): based on preoccupied type genus; Strongylidae Baird, 1853 (type genus *Strongylus* O. F. Müller, 1780) is available in Nematoda.
Cyllodini Everts, 1898: 469, in key [stem: *Cyllod-*]. Type genus: *Cyllodes* Erichson, 1843.
Amborotubini Leschen and Carlton, 2004: 450 [stem: *Amborotub-*]. Type genus: *Amborotubus* Leschen and Carlton, 2004.

### Tribe Lawrencerosini

Kirejtshuk, 1991

Lawrencerosini Kirejtshuk, 1991: 863 [stem: *Lawrenceros-*]. Type genus: *Lawrencerosus* Kirejtshuk, 1991.

### Tribe Mystropini

Murray, 1864

Mystropidae Murray, 1864: 411 [stem: *Mystrop-*]. Type genus: *Mystrops* Erichson, 1843.

### Tribe Nitidulini

Latreille, 1802

Nitidulariae Latreille, 1802: 131 [stem: *Nitidul-*]. Type genus: *Nitidula* Fabricius, 1775.
Thalycrina C. G. Thomson, 1859: 68 [stem: *Thalycr-*]. Type genus: *Thalycra* Erichson, 1843.
Pocadiini Seidlitz, 1872 [Gatt.]: 32 [stem: *Pocadi-*]. Type genus: *Pocadius* Erichson, 1843.
Prometopinae Böving and Craighead, 1931: 37, in key [stem: *Prometopi-*]. Type genus: *Prometopia* Erichson, 1843. Comment: the spelling Prometopinae in the key on page 37 is considered a *lapsus calami* since the correct spelling Prometopiinae was used in the same publication in the conspectus on page 74 and in the caption for plate 35.
Orvoenini Dajoz, 1980b: 191 [stem: *Orvoeni-*]. Type genus: *Orvoenia* Dajoz, 1980 [syn. of *Megauchenia* W. S. MacLeay, 1825]. Comment: incorrect original stem formation, not in prevailing usage.

### Subfamily Cillaeinae

Kirejtshuk and Audisio, 1986

Cillaeinae Kirejtshuk and Audisio, 1986: 219 [stem: *Cillae-*]. Type genus: *Cillaeus* Laporte, 1835.

### Subfamily Cryptarchinae

Thomson, 1859

Cryptarchina C. G. Thomson, 1859: 69 [stem: *Cryptarch-*]. Type genus: *Cryptarcha* Shuckard, 1839.

### Tribe Arhinini

Kirejtshuk, 1987

Arhinini Kirejtshuk, 1987: 63 [stem: *Arhin-*]. Type genus: *Arhina* Murray, 1876.

### Tribe Cryptarchini

Thomson, 1859

Cryptarchina C. G. Thomson, 1859: 69 [stem: *Cryptarch-*]. Type genus: *Cryptarcha* Shuckard, 1839.
Pityophagini Reitter, 1891: 163 [stem: *Pityophag-*]. Type genus: *Pityophagus* Shuckard, 1839.
Glischrochilini Iablokoff-Khnzorian, 1966: 314 [stem: *Glischrochil-*]. Type genus: *Glischrochilus* Reitter, 1873.

### Tribe Eucalosphaerini

Kirejtshuk, 1987

Eucalosphaerini Kirejtshuk, 1987: 80 [stem: *Eucalosphaer-*]. Type genus: *Eucalosphaera* Jelinek, 1978.

### Tribe Platyarchini

Kirejtshuk, 1998

Platyarchini Kirejtshuk, 1998a: 41, in key [stem: *Platyarch-*]. Type genus: *Platyarcha* Kirejtshuk, 1987.

### Subfamily Cybocephalinae

Jacquelin du Val, 1858

Cybocéphalites Jacquelin du Val, 1858: 151 [stem: *Cybocephal-*]. Type genus: *Cybocephalus* Erichson, 1844. Comment: original vernacular name available (Art. 11.7.2): first used in latinized form by C. G. Thomson (1862: 123, as Cybocephalidae), generally accepted as in Pakaluk et al. (1994: 230, as Cybocephalinae).

### Family Smicripidae

Horn, 1880

Smicripini G. H. Horn, 1880d: 268 [stem: *Smicrip-*]. Type genus: *Smicrips* J. L. LeConte, 1878.
Tisiphoninae Sharp, 1900a: 578 [stem: *Tisiphon-*]. Type genus: *Tisiphone* Reitter, 1876 [preoccupied genus name, not *Tisiphone* Hübner, 1816 [Lepidoptera]; syn. of *Smicrips* J. L. LeConte, 1878]. Comment: permanently invalid (Art. 39): based on preoccupied type genus.

### Family Bothrideridae

Erichson, 1845

Bothriderini Erichson, 1845: 287 [stem: *Bothrider-*]. Type genus: *Bothrideres* Dejean, 1835.

### Subfamily Teredinae

Seidlitz, 1888

Teredini Seidlitz, 1888 [Gatt.]: 57 [stem: *Tered-*]. Type genus: *Teredus* Dejean, 1835.

### Tribe Sosylopsini

Dajoz, 1980

Sosylopsini Dajoz, 1980a: 158 [stem: *Sosylops-*]. Type genus: *Sosylopsis* Grouvelle, 1910. Comment: current spelling maintained (Art. 29.5): incorrect stem formation in prevailing usage (should be *Sosylopse*-).

### Tribe Sysolini

Ślipiński and Pal, 1985

Sysolini Ślipiński and Pal, 1985: 40 [stem: *Sysol-*]. Type genus: *Sysolus* Grouvelle, 1908.

### Tribe Teredini

Seidlitz, 1888

Teredini Seidlitz, 1888 [Gatt.]: 57 [stem: *Tered-*]. Type genus: *Teredus* Dejean, 1835.

### Subfamily Xylariophilinae

Pal and Lawrence, 1986

Xylariophilinae Pal and Lawrence, 1986: 208, in key [stem: *Xylariophil-*]. Type genus: *Xylariophilus* Pal and Lawrence, 1986.

### Subfamily Anommatinae

Ganglbauer, 1899

Anommatini Ganglbauer, 1899: 894 [stem: *Anommat-*]. Type genus: *Anommatus* Wesmael, 1835.

### Subfamily Bothriderinae

Erichson, 1845

Bothriderini Erichson, 1845: 287 [stem: *Bothrider-*]. Type genus: *Bothrideres* Dejean, 1835.
Deretaphrini G. H. Horn, 1878: 578 [stem: *Deretaphr-*]. Type genus: *Deretaphrus* Newman, 1842.
Dastarcini Reitter, 1922a: 39 [stem: *Dastarc-*]. Type genus: *Dastarcus* Walker, 1858.

### Family Cerylonidae

Billberg, 1820

Cerylonides Billberg, 1820a: 47 [stem: *Cerylon-*]. Type genus: *Cerylon* Latreille, 1802 [placed on the Official List of Generic Names in Zoology (ICZN 1995c)]. Comment: Cerylonidae Billberg, 1820 placed on the Official List of Family-Group Names in Zoology (ICZN 1995c).

### Subfamily Euxestinae

Grouvelle, 1908

Euxestinae Grouvelle, 1908: 397, in key [stem: *Euxest-*]. Type genus: *Euxestus* Wollaston, 1858.
Pachyochthesinae Reitter, 1911: 105 [stem: *Pachyochth-*]. Type genus: *Pachyochthes* Reitter, 1897 [syn. of *Hypodacne* J. L. LeConte, 1875]. Comment: incorrect original stem formation, not in prevailing usage.
Metacerylini Heinze, 1944: 21 [stem: *Metacerylon-*]. Type genus: *Metacerylon* Grouvelle, 1906. Comment: incorrect original stem formation, not in prevailing usage.
Cycloxenini Jeannel and Paulian, 1945: 57, in key [stem: *Cycloxen-*]. Type genus: *Cycloxenus* Arrow, 1925.
Tachyoryctidiini Jeannel and Paulian, 1945: 57, in key [stem: *Tachyoryctidi-*]. Type genus: *Tachyoryctidium* Jeannel and Paulian, 1945.

### Subfamily Loeblioryloninae

Ślipiński, 1990

Loebliorylininae Ślipiński, 1990: 81 [stem: *Loebliorylon-*]. Type genus: *Loebliorylon* Ślipiński, 1990. Comment: incorrect original stem formation, not in prevailing usage.

### Subfamily Ostomopsinae

Sen Gupta and Crowson, 1973

Ostomopsini Sen Gupta and Crowson, 1973: 400 [stem: *Ostomops-*]. Type genus: *Ostomopsis* Scott, 1922. Comment: current spelling maintained (Art. 29.5): incorrect stem formation in prevailing usage (should be *Ostomopse*-).

### Subfamily Murmidiinae

Jacquelin du Val, 1858

Murmidiides Jacquelin du Val, 1858: 227 [stem: *Murmidi-*]. Type genus: *Murmidius* Leach, 1822. Comment: original vernacular name available (Art. 11.7.2): first used in latinized form by J. L. LeConte (1861: 78, as Murmidiidae), generally accepted as in Pakaluk et al. (1994: 232, as Murmidiinae).

### Subfamily Ceryloninae

Billberg, 1820

Cerylonides Billberg, 1820a: 47 [stem: *Cerylon-*]. Type genus: *Cerylon* Latreille, 1802 [placed on the Official List of Generic Names in Zoology (ICZN 1995c)]. Comment: Cerylonidae Billberg, 1820a placed on the Official List of Family-Group Names in Zoology (ICZN 1995c); this family-group name was also used in the same year by Billberg (1820b: 394, as Cerylonides).
Pleosomides Fauvel, 1891: 162 [stem: *Ploeosomat-*]. Type genus: *Ploeosoma* Wollaston, 1854. Comment: incorrect original stem formation, not in prevailing usage.
Lapethinae Sharp, 1894: 445 [stem: *Lapeth-*]. Type genus: *Lapethus* Casey, 1890 [syn. of *Mychocerus* Erichson, 1845].
Mychocerinae Sharp, 1895b: 494 [stem: *Mychocer-*]. Type genus: *Mychocerus* sensu J. L. LeConte, 1869 [syn. of *Mychocerinus* Ślipiński, 1990]. Comment: based on a misidentified type genus.
Aculagnathidae Oke, 1932: 22 [stem: *Aculagnath-*]. Type genus: *Aculagnathus* Oke, 1932.
Dolosidae Dajoz, 1963: 96 [stem: *Dolos-*]. Type genus: *Dolosus* Dajoz, 1963 [subgenus of *Thyroderus* Sharp, 1885].

### Family Alexiidae

Imhoff, 1856

Alexiadae Imhoff, 1856: [2] 151 [stem: *Alexi-*]. Type genus: *Alexia* Stephens, 1833 [syn. of *Sphaerosoma* Stephens, 1832].
Sphaerosominae Ganglbauer, 1899: 913 [stem: *Sphaerosomat-*]. Type genus: *Sphaerosoma* Stephens, 1832. Comment: incorrect original stem formation, not in prevailing usage.

### Family Discolomatidae

Horn, 1878

Discolomidae G. H. Horn, 1878: 557 [stem: *Discolomat-*]. Type genus: *Discoloma* Erichson, 1845. Comment: incorrect original stem formation, not in prevailing usage.

### Subfamily Notiophyginae

Jakobson, 1915

Notiophygidae Jakobson, 1915: 920 [stem: *Notiophyg-*]. Type genus: *Notiophygus* Gory, 1834.

### Tribe Dystheamonini

John, 1954

Dystheamonini John, 1954: 42 [stem: *Dystheamon-*]. Type genus: *Dystheamon* Grouvelle, 1927.

### Tribe Notiophygini

Jakobson, 1915

Notiophygidae Jakobson, 1915: 920 [stem: *Notiophyg-*]. Type genus: *Notiophygus* Gory, 1834.

### Tribe Pachyplacini

John, 1954

Pachyplacini John, 1954: 38 [stem: *Pachyplac-*]. Type genus: *Pachyplacus* John, 1935.

### Subfamily Discolomatinae

Horn, 1878

Discolomidae G. H. Horn, 1878: 557 [stem: *Discolomat-*]. Type genus: *Discoloma* Erichson, 1845. Comment: incorrect original stem formation, not in prevailing usage.

### Subfamily Aphanocephalinae

Jakobson, 1904

Aphanocephalidae Jakobson, 1904: 273 [stem: *Aphanocephal-*]. Type genus: *Aphanocephalus* Wollaston, 1873. Comment: name previously attributed to Grouvelle (1912: 198); usage of Aphaenocephalidae by Ganglbauer (1903: 316) in Staphylinoidea is a *lapsus calami* for Phaenocephalidae (now in Phalacridae) according to Lawrence and Newton (1995).

### Subfamily Cephalophaninae

John, 1954

Cephalophaninae John, 1954: 64 [stem: *Cephalophan-*]. Type genus: *Cephalophanus* John, 1940.

### Subfamily Pondonatinae

John, 1954

Pondonatinae John, 1954: 19 [stem: *Pondonat-*]. Type genus: *Pondonatus* John, 1954.

### Family Endomychidae

Leach, 1815

Endomychides Leach, 1815: 116 [stem: *Endomych-*]. Type genus: *Endomychus* Panzer, 1795.

### Subfamily Merophysiinae

Seidlitz, 1872

Merophysiini Seidlitz, 1872 [Gatt.]: 39 [stem: *Merophysi-*]. Type genus: *Merophysia* Lucas, 1852.
Holoparamecini Seidlitz, 1888 [Gatt.]: 57 [stem: *Holoparamec-*]. Type genus: *Holoparamecus* Curtis, 1833.

### Subfamily Pleganophorinae

Jacquelin du Val, 1858

Pléganophorides Jacquelin du Val, 1858: 186 [stem: *Pleganophor-*]. Type genus: *Pleganophorus* Hampe, 1855. Comment: original vernacular name available (Art. 11.7.2): first used in latinized form by Gerstaecker (1861: 419, as Pleganophoridae), generally accepted as in Pakaluk et al. (1994: 233, as Pleganophorinae).
Trochoidéites Chapuis, 1876: 146 [stem: *Trochoide-*]. Type genus: *Trochoideus* Westwood, 1833. Comment: original vernacular name available (Art. 11.7.2): first used in latinized form and generally accepted as in Ganglbauer (1899: 926, as Trochoideinae); the earlier use of Trochoïdeidae by Gorham (1874: 185) is not considered to be a proposal for a new family-group taxon but was rather used as a hypothetical example addressed to the editors of the journal.

### Subfamily Anamorphinae

Strohecker, 1953

Anamorphini Strohecker, 1953: 15, in key [stem: *Anamorph-*]. Type genus: *Anamorphus* J. L. LeConte, 1878.
Mychotheninae Sasaji, 1978: 8 [stem: *Mychothen-*]. Type genus: *Mychothenus* Strohecker, 1953.
Acritosomatinae Pakaluk and Ślipiński, 1995: 328 [stem: *Acritosomat-*]. Type genus: *Acritosoma* Pakaluk and Ślipiński, 1995.

### Subfamily Leiestinae

Thomson, 1863

Leiestina C. G. Thomson, 1863: 306 [stem: *Leiest-*]. Type genus: *Leiestes* Chevrolat, 1836.
Rhanes J. L. LeConte and G. H. Horn, 1883: 120 [stem: *Rhanid-*]. Type genus: *Rhanis* J. L. LeConte, 1854 [preoccupied genus name, not *Rhanis* C. L. Koch, 1846 [Arachnida]; syn. of *Rhanidea* Strohecker, 1953]. Comment: permanently invalid (Art. 39): based on preoccupied type genus; incorrect original stem formation, not in prevailing usage.
Phymaphorina Jakobson, 1915: 961 [stem: *Phymaphor-*]. Type genus: *Phymaphora* Newman, 1838.

### Subfamily Mycetaeinae

Jacquelin du Val, 1857

Mycetéides Jacquelin du Val, 1857b: 102 [stem: *Mycetae-*]. Type genus: *Mycetaea* Stephens, 1829. Comment: original vernacular name available (Art. 11.7.2): first used in latinized form by Gerstaecker (1861: 419, as Mycetaeidae), generally accepted as in Pakaluk et al. (1994: 233, as Mycetaeinae); incorrect original stem formation, not in prevailing usage.

### Subfamily Eupsilobiinae

Casey, 1895

Eupsilobiini Casey, 1895: 452 [stem: *Eupsilobi-*]. Type genus: *Eupsilobius* Casey, 1895 [syn. of *Eidoreus* Sharp, 1885].
Cerasommatidiidae Brèthes, 1925: 199 [stem: *Cerasommatidi-*]. Type genus: *Cerasommatidia* Brèthes, 1925.
Eidoreinae Sasaji, 1986: 235 [stem: *Eidore-*]. Type genus: *Eidoreus* Sharp, 1885.

### Subfamily Xenomycetinae

Strohecker, 1962

Xenomycetinae Strohecker, 1962: 801, in key [stem: *Xenomycet-*]. Type genus: *Xenomycetes* G. H. Horn, 1880.

### Subfamily Danascelinae

Tomaszewska, 2000

Danascelinae Tomaszewska, 2000: 494, in key [stem: *Danascel-*]. Type genus: *Danascelis* Tomaszewska, 1999. Comment: current spelling maintained (Art. 29.3.1.1): incorrect original stem formation in prevailing usage (should be *Danascelid*-).

### Subfamily Endomychinae

Leach, 1815

Endomychides Leach, 1815: 116 [stem: *Endomych-*]. Type genus: *Endomychus* Panzer, 1795.
*Agaricophiles Motschulsky, 1849: 58 [stem: *Agaricophil-*]. Type genus: *Agaricophilus* Motschulsky, 1837. Comment: original vernacular name unavailable (Art. 11.7.2): not subsequently latinized.
Cyclotomidae Imhoff, 1856: [2] 151 [stem: *Cyclotom-*]. Type genus: *Cyclotoma* Mulsant, 1851.
*Agaricophilinae Lawrence, 1991: 484 [stem: *Agaricophil-*]. Type genus: *Agaricophilus* Motschulsky, 1837. Comment: unavailable family-group name, proposed after 1930 without description or bibliographic reference to such a description (Art. 13.1); proposed as new without reference to Agaricophiles Motschulsky, 1849.

### Subfamily Epipocinae

Gorham, 1873

Epipocidae Gorham, 1873: 20 [stem: *Epipoc-*]. Type genus: *Epipocus* Germar, 1843.

### Subfamily Stenotarsinae

Chapuis, 1876

Sténotarsites Chapuis, 1876: 125 [stem: *Stenotars-*]. Type genus: *Stenotarsus* Perty, 1832. Comment: original vernacular name available (Art. 11.7.2): first used in latinized form by Csiki (1901: 37, as Stenotarsini), generally accepted as in Shockley et al. (2009: 73, as Stenotarsinae).

### Subfamily Lycoperdininae

Bromhead, 1838

Lycoperdinadae Bromhead, 1838: 419 [stem: *Lycoperdin-*]. Type genus: *Lycoperdina* Latreille, 1807. Comment: name previously attributed to L. Redtenbacher (1844); incorrect original stem formation, not in prevailing usage.
Eumorphidae Gistel, 1848: [10] [stem: *Eumorph-*]. Type genus: *Eumorphus* Weber, 1801.
Dapsini Gerstaecker, 1858: 170 [stem: *Daps-*]. Type genus: *Dapsa* Latreille, 1829.
Corynomalidae Gorham, 1873: 14 [stem: *Corynomal-*]. Type genus: *Corynomalus* Gerstaecker, 1857.
Amphicini Csiki, 1910: 25 [stem: *Amphic-*]. Type genus: *Amphix* Laporte, 1840.
Beccariini Arrow, 1925: 278 [stem: *Beccari-*]. Type genus: *Beccaria* Gorham, 1885 [preoccupied genus name, not *Beccaria* Bourguignat, 1883 [Mollusca]; syn. of *Beccariola* Arrow, 1943]. Comment: permanently invalid (Art. 39): based on preoccupied type genus.
Amphisternini Strohecker, 1964: 319 [stem: *Amphistern-*]. Type genus: *Amphisternus* Germar, 1843.

### Family Coccinellidae

Latreille, 1807

Coccinellidae Latreille, 1807: 70 [stem: *Coccinell-*]. Type genus: *Coccinella* Linnaeus, 1758.

### Subfamily Microweiseinae

Leng, 1920

Microweiseini Leng, 1920: 213 [stem: *Microweise-*]. Type genus: *Microweisea* Cockerell, 1903.

### Tribe Microweiseini

Leng, 1920

Microweiseini Leng, 1920: 213 [stem: *Microweise-*]. Type genus: *Microweisea* Cockerell, 1903.

### Tribe Serangiini

Pope, 1962

*Serangiini Blackwelder, 1945: 450 [stem: *Serangi-*]. Type genus: *Serangium* Blackburn, 1889. Comment: unavailable family-group name, proposed after 1930 without description or bibliographic reference to such a description (Art. 13.1).
Serangiini Pope, 1962: 627 [stem: *Serangi-*]. Type genus: *Serangium* Blackburn, 1889.

### Tribe Sukunahikonini

Kamiya, 1960

Sukunahikonini Kamiya, 1960: 24 [stem: *Sukunahikon-*]. Type genus: *Sukunahikona* Kamiya, 1960 [syn. of *Scymnomorphus* Weise, 1897].
Scotoscymninae Duverger, 2003: 63 [stem: *Scotoscymn-*]. Type genus: *Scotoscymnus* Weise, 1901. Comment: name proposed to replace Sukunahikonini Kamiya, 1960 because of the synonymy of the type genus.

### Subfamily Coccinellinae

Latreille, 1807

Coccinellidae Latreille, 1807: 70 [stem: *Coccinell-*]. Type genus: *Coccinella* Linnaeus, 1758.

### Tribe Argentipilosini

Gordon and de Almeida, 1991

Argentipilosini Gordon and de Almeida, 1991: 150 [stem: *Argentipilos-*]. Type genus: *Argentipilosa* Gordon and de Almeida, 1991.

### Tribe Aspidimerini

Mulsant, 1850

Aspidiméraires Mulsant, 1850: 943 [stem: *Aspidimer-*]. Type genus: *Aspidimerus* Mulsant, 1850. Comment: original vernacular name available (Art. 11.7.2): first used in latinized form by Jakobson (1915: 969, as Aspidimerina), generally accepted as in Pakaluk et al. (1994: 234, as Aspidimerini).

### Tribe Azyini

Mulsant, 1850

Azyaires Mulsant, 1850: 927 [stem: *Azy-*]. Type genus: *Azya* Mulsant, 1850. Comment: original vernacular name available (Art. 11.7.2): first used in latinized form by Crotch (1874: 279, as Azyae), generally accepted as in Pakaluk et al. (1994: 233, as Azyini).
*Bucolites Chapuis, 1876: 237 [stem: *Bucol-*]. Type genus: *Bucolus* Mulsant, 1850. Comment: original vernacular name unavailable (Art. 11.7.2): not subsequently latinized.

### Tribe Brachiacanthini

Mulsant, 1850

Brachyacanthaires Mulsant, 1850: 520 [stem: *Brachiacanth-*]. Type genus: *Brachiacantha* Chevrolat, 1836 [as *Brachyacantha*, incorrect subsequent spelling of type genus name, not in prevailing usage]. Comment: original vernacular name available (Art. 11.7.2): first used in latinized form by Duverger (1990: 143, as Brachiacanthadini [incorrect stem formation]), generally accepted as in Pakaluk et al. (1994: 249, as Brachiacanthini); incorrect original stem formation, not in prevailing usage.

### Tribe Carinodulini

Gordon, Pakaluk and Ślipiński, 1989

Carinodulini Gordon et al., 1989: 360 [stem: *Carinodul-*]. Type genus: *Carinodula* Gordon et al., 1989.

### Tribe Cephaloscymnini

Gordon, 1985

Cephaloscymnini Gordon, 1985: 66 [stem: *Cephaloscymn-*]. Type genus: *Cephaloscymnus* Crotch, 1872.

### Tribe Chilocorini

Mulsant, 1846

Chilocoriens Mulsant, 1846: 166 [stem: *Chilocor-*]. Type genus: *Chilocorus* Leach, 1815. Comment: original vernacular name available (Art. 11.7.2): first used in latinized form by Weise (1885: 239, as Chilocorini), generally accepted as in Pakaluk et al. (1994: 234, as Chilocorini).
*Exochomaires Mulsant, 1850: 465 [stem: *Exochom-*]. Type genus: *Exochomus* Redtenbacher, 1843. Comment: original vernacular name unavailable (Art. 11.7.2): not subsequently latinized.

### Tribe Chnoodini

Mulsant, 1850

Chnoodiens Mulsant, 1850: 907 [stem: *Chnood-*]. Type genus: *Chnoodes* Chevrolat, 1843. Comment: original vernacular name available (Art. 11.7.2): first used in latinized form and generally accepted as in Sicard (1909: 103, as Chnoodini); name incorrectly treated as unavailable by Pakaluk et al. (1994: 248).
*Siolaires Mulsant, 1850: 931 [stem: *Siol-*]. Type genus: *Siola* Mulsant, 1850. Comment: original vernacular name unavailable (Art. 11.7.2): not subsequently latinized.
Exoplectrides Crotch, 1874: xiii [stem: *Exoplectr-*]. Type genus: *Exoplectra* Chevrolat, 1836.
Sumniini Hoang, 1982: 138 [stem: *Sumni-*]. Type genus: *Sumnius* Weise, 1892.

### Tribe Coccidulini

Mulsant, 1846

Cocciduliens Mulsant, 1846: 266 [stem: *Coccidul-*]. Type genus: *Coccidula* Kugelann, 1798. Comment: original vernacular name available (Art. 11.7.2): first used in latinized form by Gistel (1848: [10], as Coccidulaeidae [incorrect stem formation]), generally accepted as in Pakaluk et al. (1994: 233, as Coccidulini).
*Rhizobiates Mulsant, 1846: 261 [stem: *Rhyzobi-*]. Type genus: *Rhyzobius* Stephens, 1829 [as *Rhizobius*, unjustified emendation of type genus name by Agassiz (1846b: 327), not in prevailing usage]. Comment: original vernacular name unavailable (Art. 11.7.2): subsequently used in latinized form but not generally attributed to Mulsant (1846); incorrect original stem formation, not in prevailing usage.
Rhizobiina C. G. Thomson, 1866: 328 [stem: *Rhyzobi-*]. Type genus: *Rhyzobius* Stephens, 1829 [as *Rhizobius*, unjustified emendation of type genus name by Agassiz (1846b: 327), not in prevailing usage]. Comment: incorrect original stem formation, not in prevailing usage; the name Rhizobiinae Buckton, 1883 (type genus *Rhizobius* Burmeister, 1835) is available in Hemiptera.

### Tribe Coccinellini

Latreille, 1807

Coccinellidae Latreille, 1807: 70 [stem: *Coccinell-*]. Type genus: *Coccinella* Linnaeus, 1758.
*Adoniates Mulsant, 1846: 35 [stem: *Adoni-*]. Type genus: *Adonia* Mulsant, 1846 [syn. of *Hippodamia* Chevrolat, 1836]. Comment: original vernacular name unavailable (Art. 11.7.2): not subsequently latinized.
*Hippodamiaires Mulsant, 1846: 30 [stem: *Hippodami-*]. Type genus: *Hippodamia* Chevrolat, 1836. Comment: original vernacular name unavailable (Art. 11.7.2): not subsequently latinized.
*Micraspiaires Mulsant, 1846: 162 [stem: *Micraspid-*]. Type genus: *Micraspis* Chevrolat, 1836. Comment: original vernacular name unavailable (Art. 11.7.2): not subsequently latinized; incorrect original stem formation, not in prevailing usage.
*Mysiates Mulsant, 1846: 125 [stem: *Myzi-*]. Type genus: *Myzia* Mulsant, 1846 [as *Mysia*, alternative original spelling of type genus name; we follow Kovář (2007: 620) in using *Myzia* as the correct spelling for this genus]. Comment: original vernacular name unavailable (Art. 11.7.2): not subsequently latinized; incorrect original stem formation, not in prevailing usage.
Halyziaires Mulsant, 1846: 123 [stem: *Halyzi-*]. Type genus: *Halyzia* Mulsant, 1846. Comment: original vernacular name available (Art. 11.7.2): first used in latinized form and generally accepted as in Pakaluk et al. (1994: 234, as Halyziini).
Alesiaires Mulsant, 1850: 343 [stem: *Alesi-*]. Type genus: *Alesia* Mulsant, 1850 [syn. of *Micraspis* Chevrolat, 1836]. Comment: original vernacular name available (Art. 11.7.2): first used in latinized form by Mader (1934: 297, as Alesiini), generally accepted as in Mader (1954: 93, as Alesiina).
*Cariaires Mulsant, 1850: 228 [stem: *Cari-*]. Type genus: *Caria* Mulsant, 1850 [preoccupied genus name, not *Caria* Hübner, 1823[ Lepidoptera]; syn. of *Megalocaria* Crotch, 1874]. Comment: original vernacular name unavailable (Art. 11.7.2): subsequently used in latinized form, e.g., Casey (1899: 72, as Cariini), but not generally accepted as valid; if name found to be available then permanently invalid (Art. 39): based on preoccupied type genus.
Coelophoraires Mulsant, 1850: 374 [stem: *Coelophor-*]. Type genus: *Coelophora* Mulsant, 1850. Comment: original vernacular name available (Art. 11.7.2): first used in latinized form and generally accepted as in Mader (1954: 93, as Coelophorina).
Cydoniaires Mulsant, 1850: 429 [stem: *Cydoni-*]. Type genus: *Cydonia* Mulsant, 1850 [syn. of *Cheilomenes* Chevrolat, 1836]. Comment: original vernacular name available (Art. 11.7.2): first used in latinized form and generally accepted as in Mader (1954: 93, as Cydoniina).
Discotomaires Mulsant, 1850: 214 [stem: *Discotom-*]. Type genus: *Discotoma* Mulsant, 1850. Comment: original vernacular name available (Art. 11.7.2): first used in latinized form by Korschevsky (1932: 577, as Discotomini), generally accepted as in Pakaluk et al. (1994: 234, as Discotomini).
Tytthaspides Crotch, 1874: xiii [stem: *Tytthaspid-*]. Type genus: *Tytthaspis* Crotch, 1874.
Synonychini Weise, 1885: 7 [stem: *Synonych-*]. Type genus: *Synonycha* Chevrolat, 1836.
Psylloborini Casey, 1899: 73 [stem: *Psyllobor-*]. Type genus: *Psyllobora* Chevrolat, 1836.
Anisostictina Jakobson, 1915: 969 [stem: *Anisostict-*]. Type genus: *Anisosticta* Chevrolat, 1836.
Anisolemniina Mader, 1954: 93, in key [stem: *Anisolemni-*]. Type genus: *Anisolemnia* Crotch, 1874.
Bulaeini Savoyskaya, 1969: 102 [stem: *Bulae-*]. Type genus: *Bulaea* Mulsant, 1850.
Singhikaliini Miyatake, 1972: 96 [stem: *Singhikali-*]. Type genus: *Singhikalia* Kapur, 1963.

### Tribe Cranophorini

Mulsant, 1850

Cranophoraires Mulsant, 1850: 939 [stem: *Cranophor-*]. Type genus: *Cranophorus* Mulsant, 1850. Comment: original vernacular name available (Art. 11.7.2): first used in latinized form by Casey (1899: 74, as Cranophorini), generally accepted as in Pakaluk et al. (1994: 233, as Cranophorini).

### Tribe Cryptognathini

Mulsant, 1850

Cryptognathaires Mulsant, 1850: 496 [stem: *Cryptognath-*]. Type genus: *Cryptognatha* Mulsant, 1850. Comment: original vernacular name available (Art. 11.7.2): first used in latinized form by Gordon (1971: 181, as Cryptognathini), generally accepted as in Pakaluk et al. (1994: 249, as Cryptognathini).
*Pentiliaires Mulsant, 1850: 501 [stem: *Pentili-*]. Type genus: *Pentilia* Mulsant, 1850. Comment: original vernacular name unavailable (Art. 11.7.2): subsequently used in latinized form but not generally attributed to Mulsant (1846).
Pentiliini Casey, 1899: 74 [stem: *Pentili-*]. Type genus: *Pentilia* Mulsant, 1850.
Oeneini Casey, 1899: 74 [stem: *Oene-*]. Type genus: *Oeneis* Mulsant, 1850 [preoccupied genus name, not *Oeneis* Hübner, 1819 [Lepidoptera]; syn. of *Delphastus* Casey, 1899]. Comment: permanently invalid (Art. 39): based on preoccupied type genus; the taxon Oeneini Wheeler, 1903 (type genus *Oeneis* Hübner, 1819) is available in Lepidoptera.

### Tribe Cynegetini

Thomson, 1866

Cynegetides C. G. Thomson, 1866: 374 [stem: *Cyneget-*]. Type genus: *Cynegetis* Chevrolat, 1836. Comment: current spelling maintained (Art. 29.3.1.1): incorrect stem formation in prevailing usage (should be *Cynegetid*-).
Madaini Gordon, 1975: 206 [stem: *Mad-*]. Type genus: *Mada* Mulsant, 1850. Comment: incorrect original stem formation, not in prevailing usage.

### Tribe Diomini

Gordon, 1999

Diomini Gordon, 1999: 4 [stem: *Diom-*]. Type genus: *Diomus* Mulsant, 1850.

### Tribe Epilachnini

Mulsant, 1846

Epilachniens Mulsant, 1846: 190 [stem: *Epilachn-*]. Type genus: *Epilachna* Chevrolat, 1836. Comment: original vernacular name available (Art. 11.7.2): first used in latinized form by Ganglbauer (1898: 947, as Epilachninae), generally accepted as in Pakaluk et al. (1994: 234, as Epilachnini).
*Chnootribaires Mulsant, 1850: 697 [stem: *Chnootrib-*]. Type genus: *Chnootriba* Chevrolat, 1836. Comment: original vernacular name unavailable (Art. 11.7.2): not subsequently latinized.
Subcoccinellini Jakobson, 1915: 968 [stem: *Subcoccinell-*]. Type genus: *Subcoccinella* Huber, 1841.

### Tribe Epivertini

Pang and Mao, 1979

Epivertini Pang and Mao, 1979: 158 [stem: *Epivert-*]. Type genus: *Epiverta* Dieke, 1947.

### Tribe Eremochilini

Gordon and Vanderberg, 1987

Eremochilini Gordon and Vanderberg, 1987: 6 [stem: *Eremochil-*]. Type genus: *Eremochilus* Weise, 1912.

### Tribe Hyperaspidini

Mulsant, 1846

Hypéraspiens Mulsant, 1846: 177 [stem: *Hyperaspid-*]. Type genus: *Hyperaspis* Chevrolat, 1836. Comment: original vernacular name available (Art. 11.7.2): first used in latinized form by Weise (1885: 5, as Hyperaspini), generally accepted as in Pakaluk et al. (1994: 233, as Hyperaspini); incorrect original stem formation, not in prevailing usage.
*Thalassaires Mulsant, 1850: 505 [stem: *Thalass-*]. Type genus: *Thalassa* Mulsant, 1850. Comment: original vernacular name unavailable (Art. 11.7.2): not subsequently latinized.
*Tiphysaires Mulsant, 1850: 516 [stem: *Tiphys-*]. Type genus: *Tiphysa* Mulsant, 1850. Comment: original vernacular name unavailable (Art. 11.7.2): not subsequently latinized.

### Tribe Limnichopharini

Miyatake, 1994

Limnichopharini Miyatake, 1994: 269 [stem: *Limnichophar-*]. Type genus: *Limnichopharus* Miyatake, 1994.

### Tribe Monocorynini

Miyatake, 1988

Monocorynini Miyatake, 1988: 28 [stem: *Monocoryn-*]. Type genus: *Monocoryna* Gorham, 1885.

### Tribe Noviini

Mulsant, 1846

Noviaires Mulsant, 1846: 213 [stem: *Novi-*]. Type genus: *Novius* Mulsant, 1846 [the original spelling used by Mulsant was *Nomius* (p. 213), however, the spelling was changed to *Novius* by Mulsant in the “Addenda et errata” of the same work and is therefore considered a justified emendation (Art. 19.2)]. Comment: the original spelling of this family-group name was Nomiaires (based on the new genus *Nomius* Mulsant, 1846), the spelling of the family-group and genus-group names were changed to Noviaires and *Novius* in the “Addenda et errata” of the same work, Noviaires is therefore treated as the correct original spelling of this family-group name (Art. 19.2); original vernacular name available (Art. 11.7.2): first used in latinized form by Ganglbauer (1899: 977, as Noviini), generally accepted as in Pakaluk et al. (1994: 233, as Noviini).
*Rodoliaires Mulsant, 1850: 901 [stem: *Rodoli-*]. Type genus: *Rodolia* Mulsant, 1850. Comment: original vernacular name unavailable (Art. 11.7.2): not subsequently latinized.

### Tribe Ortaliini

Mulsant, 1850

Ortaliens Mulsant, 1850: 892 [stem: *Ortali-*]. Type genus: *Ortalia* Mulsant, 1850. Comment: original vernacular name available (Art. 11.7.2): first used in latinized form by Crotch (1874: 274, as Ortaliae), generally accepted as in Pakaluk et al. (1994: 234, as Ortaliini).

### Tribe Oryssomini

Gordon, 1974

Oryssomini Gordon, 1974: 146 [stem: *Oryssom-*]. Type genus: *Oryssomus* Mulsant, 1850.

### Tribe Platynaspini

Mulsant, 1846

Platynaspiaires Mulsant, 1846: 215 [stem: *Platynasp-*]. Type genus: *Platynaspis* Redtenbacher, 1843. Comment: original vernacular name available (Art. 11.7.2): first used in latinized form by Casey (1899: 109, as Platynaspini), generally accepted as in Pakaluk et al. (1994: 234, as Platynaspini); current spelling maintained (Art. 29.3.1.1): incorrect stem formation in prevailing usage (should be *Platynaspid*-).

### Tribe Plotinini

Miyatake, 1994

Plotinini Miyatake, 1994: 279 [stem: *Plotin-*]. Type genus: *Plotina* Lewis, 1896.

### Tribe Poriini

Mulsant, 1850

Poriens Mulsant, 1850: 884 [stem: *Pori-*]. Type genus: *Poria* Mulsant, 1850. Comment: original vernacular name available (Art. 11.7.2): first used in latinized form and generally accepted as in Gorham (1894: 206, as Poriides [treated as Latin]).

### Tribe Scymnillini

Casey, 1899

Scymnillini Casey, 1899: 74 [stem: *Scymnill-*]. Type genus: *Scymnillus* G. H. Horn, 1895.
Zilini Gordon, 1985: 74 [stem: *Zil-*]. Type genus: *Zilus* Mulsant, 1850. Comment: name proposed to replace Scymnillini Casey, 1899 because of the synonymy of the type genus at that time.

### Tribe Scymnini

Mulsant, 1846

Scymniaires Mulsant, 1846: 189 [stem: *Scymn-*]. Type genus: *Scymnus* Kugelann, 1794. Comment: original vernacular name available (Art. 11.7.2): first used in latinized form by Weise (1885: 5, as Scymnini), generally accepted as in Pakaluk et al. (1994: 233, as Scymnini); incorrect original stem formation, not in prevailing usage.
*Cryptolaemaires Mulsant, 1853: 267 [stem: *Cryptolaem-*]. Type genus: *Cryptolaemus* Mulsant, 1853. Comment: original vernacular name unavailable (Art. 11.7.2): not subsequently latinized.

### Tribe Selvadiini

Gordon, 1985

Selvadiini Gordon, 1985: 347 [stem: *Selvadi-*]. Type genus: *Selvadius* Casey, 1899.

### Tribe Shirozuellini

Sasaji, 1967

Shirozuellini Sasaji, 1967: 23 [stem: *Shirozuell-*]. Type genus: *Shirozuella* Sasaji, 1967.
Ghaniini Ahmad, 1973: 449 [stem: *Ghani-*]. Type genus: *Ghanius* Ahmad, 1973.

### Tribe Stethorini

Dobzhansky, 1924

Stethorini Dobzhansky, 1924: 20 [stem: *Stethor-*]. Type genus: *Stethorus* Weise, 1885.

### Tribe Sticholotidini

Weise, 1901

Pharini Casey, 1899: 74 [stem: *Phar-*]. Type genus: *Pharus* Mulsant, 1850 [preoccupied genus name, not *Pharus* Gray, 1840 [Mollusca]; syn. of *Pharoscymnus* Bedel, 1906]. Comment: permanently invalid (Art. 39): based on preoccupied type genus; this family-group name was also proposed in the same year by Ganglbauer (1899: 954, as Pharini), priority could not be established between those two works; the name Pharinae Adams and Adams, 1856 in Mollusca (type genus *Pharus* Gray, 1840) is available.
Sticholotini Weise, 1901: 430 [stem: *Sticholotid-*]. Type genus: *Sticholotis* Crotch, 1874. Comment: incorrect original stem formation, not in prevailing usage; correction of stem by Gordon (1977: 186).
Clanini Weise, 1901: 430 [stem: *Clan-*]. Type genus: *Clanis* Mulsant, 1850 [preoccupied genus name, not *Clanis* Hübner, 1819 [Lepidoptera]; syn. of *Jauravia* Motschulsky, 1858]. Comment: permanently invalid (Art. 39): based on preoccupied type genus.
Coelopterini Weise, 1906: column 369 [stem: *Coelopter-*]. Type genus: *Coelopterus* Mulsant and Rey, 1852. Comment: name previously attributed to Della Beffa (1912: 171, as Coleopterini [incorrect stem formation]) in the literature.

### Tribe Telsimiini

Casey, 1899

Telsimiini Casey, 1899: 74 [stem: *Telsimi-*]. Type genus: *Telsimia* Casey, 1899.

### Tribe Tetrabrachini

Kapur, 1948

Lithophilidae Imhoff, 1856: [2] 151 [stem: *Lithophil-*]. Type genus: *Lithophilus* Fröhlich, 1799 [preoccupied genus name, not *Lithophilus* Schneider, 1791 [Coleoptera: Carabidae]; syn. of *Tetrabrachys* Kapur, 1948]. Comment: permanently invalid (Art. 39): based on preoccupied type genus.
Tetrabrachinae Kapur, 1948: 320 [stem: *Tetrabrach-*]. Type genus: *Tetrabrachys* Kapur, 1948. Comment: replacement name for Lithophilinae Imhoff, 1856 because of the homonymy of the type genus; current spelling maintained (Art. 29.5): incorrect stem formation in prevailing usage (should be *Tetrabrache*-).

### Family Corylophidae

LeConte, 1852

Corylophi J. L. LeConte, 1852b: 141 [stem: *Coryloph-*]. Type genus: *Corylophus* Stephens, 1833.

### Subfamily Periptyctinae

Ślipiński, Lawrence and Tomaszewska, 2001

Periptyctinae Ślipiński et al., 2001: 312 [stem: *Periptyct-*]. Type genus: *Periptyctus* Blackburn, 1825.

### Subfamily Corylophinae

LeConte, 1852

Corylophi J. L. LeConte, 1852b: 141 [stem: *Coryloph-*]. Type genus: *Corylophus* Stephens, 1833.

### Tribe Aenigmaticini

Casey, 1900

Aenigmaticini Casey, 1900: 61 [stem: *Aenigmatic-*]. Type genus: *Aenigmaticum* A. Matthews, 1888.

### Tribe Cleidostethini

Bowestead, Booth, Ślipiński and Lawrence, 2001

Cleidostethini Bowestead et al., 2001: 323 [stem: *Cleidosteth-*]. Type genus: *Cleidostethus* Arrow, 1929.

### Tribe Corylophini

LeConte, 1852

Corylophi J. L. LeConte, 1852b: 141 [stem: *Coryloph-*]. Type genus: *Corylophus* Stephens, 1833.

### Tribe Foadiini

Ślipiński, Tomaszewska and Lawrence, 2009

Foadiini Ślipiński et al., 2009: 422 [stem: *Foadi-*]. Type genus: *Foadia* Pakaluk, 1985.

### Tribe Orthoperini

Jacquelin du Val, 1857

Orthopérites Jacquelin du Val, 1857b: 100 [stem: *Orthoper-*]. Type genus: *Orthoperus* Stephens, 1829. Comment: original vernacular name available (Art. 11.7.2): first used in latinized form by C. G. Thomson (1859: 63, as Orthoperidae), generally accepted as in Ślipiński et al. (2009: 428, as Orthoperini).

### Tribe Parmulini

Poey, 1854

Clypeastres L. Redtenbacher, 1845: 122 [stem: *Clypeaster-*]. Type genus: *Clypeaster* Dejean, 1821 [preoccupied genus name, not *Clypeaster* Lamarck, 1801 [Echinodermata]; syn. of *Clypastraea* Haldeman, 1842]. Comment: permanently invalid (Art. 39): based on preoccupied type genus; incorrect original stem formation, not in prevailing usage; Clypeasteridae L. Agassiz, 1835 (type genus *Clypeaster* Lamarck, 1801) is available in Echinodermata.
Parmulini Poey, 1854: 323 [stem: *Parmul-*]. Type genus: *Parmulus* Gundlach, 1854 [syn. of *Clypastraea* Haldeman, 1842].
Saciina A. Matthews, 1888: 103 [stem: *Saci-*]. Type genus: *Sacium* J. L. LeConte, 1852 [syn. of *Clypastraea* Haldeman, 1842].
Arthrolipinae Böving and Craighead, 1931: 36, in key [stem: *Arthrolip-*]. Type genus: *Arthrolips* Wollaston, 1854.

### Tribe Peltinodini

Paulian, 1950

Peltinoditae Paulian, 1950: 19 [stem: *Peltinod-*]. Type genus: *Peltinodes* Paulian, 1950 [syn. of *Holopsis* Broun, 1883]. Comment: precedence (Peltinodini Paulian, 1950 vs Corylophodini Paulian, 1950) given to taxon originally proposed at the higher rank (Art. 24.1).
Corylophodini Paulian, 1950: 21, in key [stem: *Corylophod-*]. Type genus: *Corylophodes* A. Matthews, 1885 [syn. of *Holopsis* Broun, 1883].

### Tribe Rypobiini

Paulian, 1950

Rhypobiini Paulian, 1950: 48 [stem: *Rypobi-*]. Type genus: *Rypobius* J. L. LeConte, 1852 [as *Rhypobius*, unjustified emendation of type genus name by Gemminger and Harold (1876: 3818), not in prevailing usage]. Comment: incorrect original stem formation, not in prevailing usage.
Gloeosomatini Bowestead, 1999: 128 [stem: *Gloeosomat-*]. Type genus: *Gloeosoma* Wollaston, 1854.

### Tribe Sericoderini

Matthews, 1888

Sericoderina A. Matthews, 1888: 103 [stem: *Sericoder-*]. Type genus: *Sericoderus* Stephens, 1829.

### Tribe Teplinini

Pakaluk, Ślipiński and Lawrence, 1994

Peltinini Paulian, 1950: 21, in key [stem: *Peltin-*]. Type genus: *Peltinus* Mulsant, 1861 [preoccupied genus name, not *Peltinus* Rafinesque, 1815 [Coleoptera: Trogossitidae]; syn. of *Teplinus* Pakaluk et al., 1994]. Comment: permanently invalid (Art. 39): based on preoccupied type genus.
Teplinini Pakaluk et al., 1994: 251 [stem: *Teplin-*]. Type genus: *Teplinus* Pakaluk et al., 1994. Comment: replacement name for Peltinini Paulian, 1950 because of the homonymy of the type genus.

### Family Akalyptoischiidae

Lord, Hartley, Lawrence, McHugh and Miller, 2010

Akalyptoischiidae Lord et al., 2010: 761 [stem: *Akalyptoischi-*]. Type genus: *Akalyptoischion* Andrews, 1976.

### Family Latridiidae

Erichson, 1842

Lathridien Erichson, 1842: 122 [stem: *Latridi-*]. Type genus: *Latridius* Herbst, 1793 [as *Lathridius*, unjustified emendation of type genus name by Illiger (1802), not in prevailing usage]. Comment: the younger name Latridiidae has been used widely for this family in recent literature although Pakaluk et al. (1994: 252) and a small number of subsequent authors, e.g., Míka (2000), Lassau et al. (2005), have used the older name Corticariidae; an application was recently submitted to the Commission in order to conserve usage of the well-established name Latridiidae (Bousquet et al. 2010; see Appendix 6).

### Subfamily Latridiinae

Erichson, 1842

Lathridien Erichson, 1842: 122 [stem: *Latridi-*]. Type genus: *Latridius* Herbst, 1793 [as *Lathridius*, unjustified emendation of type genus name by Illiger (1802), not in prevailing usage]. Comment: original vernacular name available (Art. 11.7.2): first used in latinized form by L. Redtenbacher (1845: 123, as Lathridii), generally accepted as in Lawrence and Newton (1995: 886, as Latridiidae); incorrect original stem formation, not in prevailing usage.

### Subfamily Corticariinae

Curtis, 1829

Corticaridae Curtis, 1829: pl. 283 [stem: *Corticari-*]. Type genus: *Corticaria* Marsham, 1802. Comment: incorrect original stem formation, not in prevailing usage; although this is the oldest name for the family, an application was recently submitted by Bousquet et al. (2010) to conserve usage of the well-established name Latridiidae (see Appendix 6).
*Melanophthalmidae Arnett, 1962b: 835 [stem: *Melanophthalm-*]. Type genus: *Melanophthalma* Motschulsky, 1866. Comment: family-group name unavailable (Art. 11.6): originally published as synonym and not made available subsequently; name listed by Arnett as a synonym of Latridiidae and attributed to “auct.”, we could find an earlier usage of this name.

### †Subfamily Tetrameropseinae

Kirejtshuk and Azar, 2008

Tetrameropsinae Kirejtshuk and Azar, 2008: 36 [stem: *Tetrameropse-*]. Type genus: *Tetrameropsis* Kirejtshuk and Azar, 2008. Comment: incorrect original stem formation, not in prevailing usage.

### Superfamily Tenebrionoidea

Latreille, 1802

Tenebrionites Latreille, 1802: 165 [stem: *Tenebrion-*]. Type genus: *Tenebrio* Linnaeus, 1758. Comment: First Revisers found (Tenebrionoidea Latreille, 1802 vs Mordelloidea Latreille, 1802) are Alonso-Zarazaga and Mansilla-Castrillo (1988: 19).

### Family Mycetophagidae

Leach, 1815

Mycetophagida Leach, 1815: 110 [stem: *Mycetophag-*]. Type genus: *Mycetophagus* Fabricius, 1792.

### Subfamily Esarcinae

Reitter, 1882

Esarcini Reitter, 1882a: 115 [stem: *Esarc-*]. Type genus: *Esarcus* Reiche, 1864.

### Subfamily Mycetophaginae

Leach, 1815

Mycetophagida Leach, 1815: 110 [stem: *Mycetophag-*]. Type genus: *Mycetophagus* Fabricius, 1792.

### Tribe Mycetophagini

Leach, 1815

Mycetophagida Leach, 1815: 110 [stem: *Mycetophag-*]. Type genus: *Mycetophagus* Fabricius, 1792.
Tritomidae Crotch, 1873a: 78 [stem: *Tritom-*]. Type genus: *Tritoma* Geoffroy, 1762 [senior homonym of *Tritoma* Fabricius, 1775 [Coleoptera: Erotylidae] but placed on the Official Index of Rejected and Invalid Generic Names in Zoology (ICZN 1994a); syn. of *Mycetophagus* Fabricius, 1792]. Comment: permanently invalid (Art. 39): based on suppressed type genus; Tritomidae was also used the same year by Crotch (1873b: 42).
Triphyllini Harold, 1880: 757 [stem: *Triphyll-*]. Type genus: *Triphyllus* Dejean, 1821.

### Tribe Typhaeini

Thomson, 1863

Typhaeina C. G. Thomson, 1863: 241 [stem: *Typhae-*]. Type genus: *Typhaea* Stephens, 1829.
Typhaeini Nikitsky, 1993: 165 [stem: *Typhae-*]. Type genus: *Typhaea* Stephens, 1829. Comment: family-group name proposed as new without reference to Typhaeina C. G. Thomson, 1863.

### Subfamily Bergininae

Leng, 1920

Bergini Leng, 1920: 246 [stem: *Bergin-*]. Type genus: *Berginus* Erichson, 1846. Comment: incorrect original stem formation, not in prevailing usage.

### Family Archeocrypticidae

Kaszab, 1964

Archeocrypticini Kaszab, 1964: 361 [stem: *Archeocryptic-*]. Type genus: *Archeocrypticus* Kaszab, 1964.

### Family Pterogeniidae

Crowson, 1953

Pterogeniidae Crowson, 1953: 38 [stem: *Pterogeni-*]. Type genus: *Pterogenius* Candèze, 1861.

### Family Ciidae

Leach, 1819

Cisidae Leach, 1819: 206 [stem: *Ci-*]. Type genus: *Cis* Latreille, 1797. Comment: incorrect original stem formation, not in prevailing usage; Gistel (1848: [6], 1856a: 368) used the name Microtrocteidae for a family that included only the genus *Cis* Latreille, we could not find any genus name on which Microtrocteidae could be based on therefore this family-group name is unavailable.

### Subfamily Sphindociinae

Lawrence, 1974

Sphindociinae Lawrence, 1974b: 9 [stem: *Sphindoci-*]. Type genus: *Sphindocis* Fall, 1917. Comment: published 28 June 1974; this family-group name was also used in the same year by Lawrence (1974a [22 January]: 24, as Sphindociinae) but the older name is unavailable because it was proposed after 1930 without description or bibliographic reference to such a description (Art. 13.1).

### Subfamily Ciinae

Leach, 1819

Cisidae Leach, 1819: 206 [stem: *Ci-*]. Type genus: *Cis* Latreille, 1797.

### Tribe Ciini

Leach, 1819

Cisidae Leach, 1819: 206 [stem: *Ci-*]. Type genus: *Cis* Latreille, 1797. Comment: incorrect original stem formation, not in prevailing usage.

### Tribe Orophiini

Thomson, 1863

Orophiina C. G. Thomson, 1863: 195 [stem: *Orophi-*]. Type genus: *Orophius* L. Redtenbacher, 1848 [syn. of *Octotemnus* Mellié, 1847].
Octotemnidae Reitter, 1878: 21 [stem: *Octotemn-*]. Type genus: *Octotemnus* Mellié, 1847.
Rhopalodontini Everts, 1898: 517, in key [stem: *Ropalodont-*]. Type genus: *Ropalodontus* Mellié, 1847 [as *Rhopalodontus*, incorrect subsequent spelling of type genus name, not in prevailing usage]. Comment: the fossil Reptilia name Rhopalodontidae Seeley, 1894 (type genus *Rhopalodon* Fischer von Waldheim, 1841) is available, therefore we recomment using the spelling *Ropalodont*- for the ciid name in order to avoid homonymy problems; incorrect original stem formation, not in prevailing usage.

### Tribe Xylographellini

Kawanabe and Miyatake, 1996

Xylographellini Kawanabe and Miyatake, 1996: 125 [stem: *Xylographell-*]. Type genus: *Xylographella* Miyatake, 1985.

### Subtribe Syncosmetina

Lopes-Andrade, 2008

Syncosmetina Lopes-Andrade, 2008: 40 [stem: *Syncosmet-*]. Type genus: *Syncosmetus* Sharp, 1891.

### Subtribe Xylographellina

Kawanabe and Miyatake, 1996

Xylographellini Kawanabe and Miyatake, 1996: 125 [stem: *Xylographell-*]. Type genus: *Xylographella* Miyatake, 1985.

### Family Tetratomidae

Billberg, 1820

Tetratomaedes Billberg, 1820a: 34 [stem: *Tetratom-*]. Type genus: *Tetratoma* Fabricius, 1790.

### Subfamily Tetratominae

Billberg, 1820

Tetratomaedes Billberg, 1820a: 34 [stem: *Tetratom-*]. Type genus: *Tetratoma* Fabricius, 1790.

### Subfamily Piseninae

Miyatake, 1960

Pisenini Miyatake, 1960: 124 [stem: *Pisen-*]. Type genus: *Pisenus* Casey, 1900.

### Subfamily Penthinae

Lacordaire, 1859

Penthides Lacordaire, 1859: 456 [stem: *Penth-*]. Type genus: *Penthe* Newman, 1838. Comment: original vernacular name available (Art. 11.7.2): first used in latinized form by G. H. Horn (1888: 43, as Penthini), generally accepted as in Young and Pollock (2002: 415, as Penthinae).

### Subfamily Hallomeninae

Gistel, 1848

Hallomenidae Gistel, 1848: [11] [stem: *Hallomen-*]. Type genus: *Hallomenus* Panzer, 1793.
*Dryalates Mulsant, 1856b: 44 [stem: *Dryal-*]. Type genus: *Dryala* Mulsant, 1856 [syn. of *Hallomenus* Panzer, 1793]. Comment: original vernacular name unavailable (Art. 11.7.2): not subsequently latinized.
Mycétomiens Mulsant, 1856b: 103 [stem: *Mycetomat-*]. Type genus: *Mycetoma* Dejean, 1834. Comment: original vernacular name available (Art. 11.7.2): first used in latinized form and generally accepted as in Desbrochers des Loges (1900: 16, as Mycetomini); incorrect original stem formation, not in prevailing usage.

### Subfamily Eustrophinae

Gistel, 1848

Eustrophidae Gistel, 1848: [10] [stem: *Eustroph-*]. Type genus: *Eustrophus* Illiger, 1802.

### Tribe Eustrophini

Gistel, 1848

Eustrophidae Gistel, 1848: [10] [stem: *Eustroph-*]. Type genus: *Eustrophus* Illiger, 1802.

### Tribe Holostrophini

Nikitsky, 1998

Holostrophini Nikitsky, 1998: 39 [stem: *Holostroph-*]. Type genus: *Holostrophus* G. H. Horn, 1888.

### Family Melandryidae

Leach, 1815

Melyandrida Leach, 1815: 104 [stem: *Melandry-*]. Type genus: *Melandrya* Fabricius, 1801.

### Subfamily Melandryinae

Leach, 1815

Melyandrida Leach, 1815: 104 [stem: *Melandry-*]. Type genus: *Melandrya* Fabricius, 1801.

### Tribe Anisoxiellini

Nikitsky, 2007

Anisoxiellini Nikitsky, 2007: 58 [stem: *Anisoxiell-*]. Type genus: *Anisoxiella* Nikitsky, 1989.

### Tribe Dircaeini

Kirby, 1837

Dircaeidae Kirby, 1837: 240 [stem: *Dircae-*]. Type genus: *Dircaea* Fabricius, 1798.

### Tribe Hypulini

Gistel, 1848

Hypulidae Gistel, 1848: [11] [stem: *Hypul-*]. Type genus: *Hypulus* Paykull, 1798.
Maroliini Portevin, 1934: 57, in key [stem: *Maroli-*]. Type genus: *Marolia* Mulsant, 1856.

### Tribe Melandryini

Leach, 1815

Melyandrida Leach, 1815: 104 [stem: *Melandry-*]. Type genus: *Melandrya* Fabricius, 1801. Comment: incorrect original stem formation, not in prevailing usage.
*Phryganophiles Motschulsky, 1849: 58 [stem: *Phryganophil-*]. Type genus: *Phryganophilus* Sahlberg, 1833. Comment: original vernacular name unavailable (Art. 11.7.2): not subsequently latinized.
Hylepnigalionidae Gistel, 1856a: 384 [stem: *Hylepnigalion-*]. Type genus: *Hylepnigalio* Gistel, 1856 [this genus originally included the species *caraboides* Linnaeus and *caniculatus* (without author name); the type species of *Hylepnigalio* Gistel, 1856 is here considered to be *Chrysomela caraboides* Linnaeus, 1760 by monotypy; **syn. nov.** of *Melandrya* Fabricius, 1801]. Comment: **syn. nov.**

### Tribe Orchesiini

Mulsant, 1856

Orchésiens Mulsant, 1856b: 27 [stem: *Orchesi-*]. Type genus: *Orchesia* Latreille, 1807. Comment: original vernacular name available (Art. 11.7.2): first used in latinized form by C. G. Thomson (1859: 119, as Orchesiina), generally accepted as in Nikitsky and Pollock (2008: 68, as Orchesiini).

### Tribe Serropalpini

Latreille, 1829

Serropalpides Latreille, 1829b: 43 [stem: *Serropalp-*]. Type genus: *Serropalpus* Hellenius, 1786.

### Tribe Xylitini

Thomson, 1864

Xylitina C. G. Thomson, 1864: 316 [stem: *Xylit-*]. Type genus: *Xylita* Paykull, 1798.

### Tribe Zilorini

Desbrochers des Loges, 1900

Zilorini Desbrochers des Loges, 1900: 2, in key [stem: *Zilor-*]. Type genus: *Zilora* Mulsant, 1856. Comment: spelled Ziloriini in description on page 2 (in key), but correctly spelled Zilorini on p. 17 of the same work.
Zilorini Nikitsky, 2007: 60 [stem: *Zilor-*]. Type genus: *Zilora* Mulsant, 1856. Comment: family-group name proposed as new without reference to Zilorini Desbrochers des Loges, 1900.

### Subfamily Osphyinae

Mulsant, 1856 (1839)

Nothidae Shuckard, 1839b: 51 [stem: *Noth-*]. Type genus: *Nothus* A. G. Olivier, 1811 [syn. of *Osphya* Illiger, 1807]. Comment: Osphyinae Mulsant, 1856 conserved over this name (Art. 40.2) (see Lawrence and Newton 1995: 888).
*Osphies Motschulsky, 1849: 59 [stem: *Osphy-*]. Type genus: *Osphya* Illiger, 1807. Comment: original vernacular name unavailable (Art. 11.7.2): subsequently used in latinized form but not generally attributed to Motschulsky (1849); incorrect original stem formation, not in prevailing usage.
Osphyens Mulsant, 1856b: 108 [stem: *Osphy-*]. Type genus: *Osphya* Illiger, 1807. Comment: original vernacular name available (Art. 11.7.2): first used in latinized form by Seidlitz (1875 [Gatt.]: 103, as Osphyini), generally accepted as in Pollock (2002: 421, as Osphyinae); name conserved over the older Nothinae Shuckard, 1839 (Art. 40.2) (see Lawrence and Newton 1995: 888).
Conopalpiens Mulsant, 1856b: 105 [stem: *Conopalp-*]. Type genus: *Conopalpus* Gyllenhal, 1810. Comment: original vernacular name available (Art. 11.7.2): first used in latinized form by C. G. Thomson (1864: 313, as Conopalpina), generally accepted as in Hansen (1996: 171, as Conopalpini).

### Family Mordellidae

Latreille, 1802

Mordellonae Latreille, 1802: 183 [stem: *Mordell-*]. Type genus: *Mordella* Linnaeus, 1758.
†Praemordellinae Ščegoleva-Barovskaya, 1929: 27 [stem: *Praemordell-*]. Type genus: *Praemordella* Ščegoleva-Barovskaya, 1929.
†Liaoximordellidae Wang, 1993: 87 [stem: *Liaoximordell-*]. Type genus: *Liaoximordella* Wang, 1993.

### Subfamily Ctenidiinae

Franciscolo, 1951

Ctenidiinae Franciscolo, 1951: 56 [stem: *Ctenidi-*]. Type genus: *Ctenidia* Laporte, 1840.

### Subfamily Mordellinae

Latreille, 1802

Mordellonae Latreille, 1802: 183 [stem: *Mordell-*]. Type genus: *Mordella* Linnaeus, 1758.

### Tribe Conaliini

Ermisch, 1956

Conaliini Ermisch, 1956: 273 [stem: *Conali-*]. Type genus: *Conalia* Mulsant and Rey, 1858.

### Tribe Mordellini

Latreille, 1802

Mordellonae Latreille, 1802: 183 [stem: *Mordell-*]. Type genus: *Mordella* Linnaeus, 1758.

### Tribe Mordellistenini

Ermisch, 1941

Mordellistenini Ermisch, 1941: 714 [stem: *Mordellisten-*]. Type genus: *Mordellistena* A. Costa, 1854.

### Tribe Reynoldsiellini

Franciscolo, 1957

Reynoldsiellini Franciscolo, 1957: 237 [stem: *Reynoldsiell-*]. Type genus: *Reynoldsiella* Ray, 1930.

### Tribe Stenaliini

Franciscolo, 1955

Stenaliini Franciscolo, 1955: 177 [stem: *Stenali-*]. Type genus: *Stenalia* Mulsant, 1856.

### Family Ripiphoridae

Gemminger, 1870 (1855)

Rhipidophoridae Gemminger, 1870: 2117 [stem: *Ripiphor-*]. Type genus: *Ripiphorus* Bosc, 1791 [as *Rhipidophorus*, unjustified emendation of *Ripiphorus* Bosc, 1791 by Perty (1831: xix), not in prevailing usage]. Comment: name conserved over the older Myoditidae Gerstaecker, 1855 (Art. 40.2) (see Lawrence and Newton 1995: 889, as “Myoditini Costa, 1853”); First Reviser (Ripiphoridae Gemminger, 1870 (1855) vs Ptilophoridae Gerstaecker, 1855 vs Ripidiidae Gerstaecker, 1855) not determined, prevailing usage maintained.

### Subfamily Ptilophorinae

Gerstaecker, 1855

*Ptilophores Motschulsky, 1849: 59 [stem: *Ptilophor-*]. Type genus: *Ptilophorus* Dejean, 1834. Comment: original vernacular name unavailable (Art. 11.7.2): subsequently used in latinized form but not generally attributed to Motschulsky (1849).
Ptilophorini Gerstaecker, 1855: 2 [stem: *Ptilophor-*]. Type genus: *Ptilophorus* Dejean, 1834.
Évaniocérides Lacordaire, 1859: 618 [stem: *Evaniocer-*]. Type genus: *Evaniocera* Guérin-Méneville, 1835 [syn. of *Ptilophorus* Dejean, 1834]. Comment: original vernacular name available (Art. 11.7.2): first used in latinized form by J. L. LeConte (1862: 276, as Evaniocerini), generally accepted as in J. L. LeConte and G. H. Horn (1883: xxxvii, as Evaniocerini).

### Subfamily Pelecotominae

Seidlitz, 1875

Pelecotomini Seidlitz, 1875 [Gatt.]: 104 [stem: *Pelecotom-*]. Type genus: *Pelecotoma* Fischer von Waldheim, 1808.
Micholaeminae Viana, 1971: 69 [stem: *Micholaem-*]. Type genus: *Micholaemus* Viana, 1971.

### Subfamily Hemirhipidiinae

Heller, 1921

Hemirhipidiini Heller, 1921: 168 [stem: *Hemirhipidi-*]. Type genus: *Hemirhipidius* Heller, 1921 [syn. of *Nephrites* Shuckard, 1838].
Nephritinae Selander, 1957: 101 [stem: *Nephrit-*]. Type genus: *Nephrites* Shuckard, 1838. Comment: name proposed to replace Hemirhipidiini Heller because of the synonymy of the type genus, Hemirhipidiinae is in prevailing usage (see Lawrence and Newton, 1995: 889).

### Subfamily Ripidiinae

Gerstaecker, 1855

Rhipidiini Gerstaecker, 1855: 14 [stem: *Ripidi-*]. Type genus: *Ripidius* Thunberg, 1806 [as *Rhipidius*, unjustified emendation of type genus name by Agassiz (1846b: 327), not in prevailing usage].

### Tribe Eorhipidiini

Iablokoff-Khnzorian, 1986

Eorhipidiini Iablokoff-Khnzorian, 1986: 92 [stem: *Eorhipidi-*]. Type genus: *Eorhipidius* Iablokoff-Khnzorian, 1986.

### Tribe Ripidiini

Gerstaecker, 1855

Rhipidiini Gerstaecker, 1855: 14 [stem: *Ripidi-*]. Type genus: *Ripidius* Thunberg, 1806 [as *Rhipidius*, unjustified emendation of type genus name by Agassiz (1846b: 327), not in prevailing usage]. Comment: incorrect original stem formation, not in prevailing usage; spelling of family-group and type genus names based on Krell (1996).

### Subfamily Ripiphorinae

Gemminger, 1870 (1855)

Rhipidophoridae Gemminger, 1870: 2117 [stem: *Ripiphor-*]. Type genus: *Ripiphorus* Bosc, 1791 [as *Rhipidophorus*, unjustified emendation of *Ripiphorus* Bosc, 1791 by Perty (1831: xix), not in prevailing usage]. Comment: name conserved over the older Myoditinae Gerstaecker, 1855 (Art. 40.2) (see Lawrence and Newton 1995: 889, as “Myoditini Costa, 1853”).

### Tribe Macrosiagonini

Heyden, 1908

Rhipiphorites Laporte, 1840b: 261 [stem: *Ripiphor-*]. Type genus: *Ripiphorus* sensu Fabricius, 1792 [as *Rhipiphorus*, incorrect subsequent spelling of the type genus name, not in prevailing usage; not *Ripiphorus* Bosc, 1791; syn. of *Metoecus* Dejean, 1834]. Comment: original vernacular name available (Art. 11.7.2): first used in latinized form and generally accepted as in Gerstaecker (1855: 17, as Rhipiphorini [incorrect stem formation]); based on a misidentified type genus; name treated here as invalid until an application is submitted to the Commission to suppress it for the Principles of Priority and Homonymy (Art. 65.2.1); also see Ripiphorini Gemminger, 1870.
Macrosiagonini L. Heyden, 1908: 45 [stem: *Macrosiagon-*]. Type genus: *Macrosiagon* Hentz, 1830. Comment: although this is not the oldest name for the tribe, we recommend that an application be sent to the Commission to suppress Ripiphorini Laporte, 1840 because it is based on a misidentified type genus (Art. 65.2.1).

### Tribe Ripiphorini

Gemminger, 1870 (1855)

*Mioditini A. Costa, 1853: 2 [stem: *Myodit-*]. Type genus: *Myodites* Latreille, 1829 [syn. of *Ripiphorus* Bosc, 1791]. Comment: original vernacular name unavailable (Art. 11.7.2): subsequently used in latinized form but not generally treated as valid attributed to A. Costa, 1853; incorrect original stem formation, not in prevailing usage.
Myoditini Gerstaecker, 1855: 15 [stem: *Myodit-*]. Type genus: *Myodites* Latreille, 1829 [syn. of *Ripiphorus* Bosc, 1791]. Comment : usage of younger name Ripiphorini Gemminger, 1870 conserved over this name (Art. 40.2) (see Lawrence and Newton 1995: 889, as “Myoditini Costa, 1853”).
Rhipidophoridae Gemminger, 1870: 2117 [stem: *Ripiphor-*]. Type genus: *Ripiphorus* Bosc, 1791 [as *Rhipidophorus*, unjustified emendation of type genus name by Perty (1831: xix), not in prevailing usage]. Comment: name conserved over the older Myoditini Gerstaecker, 1855 (Art. 40.2) (see Lawrence and Newton 1995: 889, as “Myoditini Costa, 1853”); incorrect original stem formation, not in prevailing usage; as mentioned by Lawrence and Newton (1995: 889) a number of authors used Ripiphoridae before Gemminger, however, their family-group names were based on *Ripiphorus* sensu Fabricius, 1792, which is a synonym of *Metoecus* Dejean, 1834; spelling of family-group and type genus names based on Krell (1996); we recommend that an application be submitted to the Commission to suppress Ripiphorites Laporte, 1840 for the Principles of Priority and Homonymy (Art. 65.2.1).

### Family Zopheridae

Solier, 1834

Zophérites Solier, 1834: 505 [stem: *Zopher-*]. Type genus: *Zopherus* Gray, 1832. Comment: usage of this name conserved over Colydiidae Billberg, 1820 (Art. 35.5).

### Subfamily Colydiinae

Billberg, 1820

Colydiides Billberg, 1820b: 394 [stem: *Colydi-*]. Type genus: *Colydium* Fabricius, 1792 [placed on the Official List of Generic Names in Zoology (ICZN 1995c)]. Comment: name placed on Official List of Family-Group Names in Zoology (as Colydiidae Erichson, 1842) and given precedence over Orthocerinae Blanchard, 1845 and Cerylonidae Billberg, 1820 whenever their type genera are placed in the same family-group taxon (ICZN 1995c); usage of the younger name Zopheridae Solier, 1834 conserved over this name (Art. 35.5).

### Tribe Acropini

Sharp, 1894

Acropinae Sharp, 1894: 444 [stem: *Acrop-*]. Type genus: *Acropis* H. C. C. Burmeister, 1840. Comment: current spelling maintained (Art. 29.3.1.1): incorrect stem formation in prevailing usage (should be *Acropid*-).

### Tribe Adimerini

Sharp, 1894

Adimeridae Sharp, 1894: 441 [stem: *Adimer-*]. Type genus: *Adimerus* Sharp, 1894.
Monoedidae Schaeffer, 1911: 114 [stem: *Monoed-*]. Type genus: *Monoedus* G. H. Horn, 1882.

### Tribe Colydiini

Billberg, 1820

Colydiides Billberg, 1820b: 394 [stem: *Colydi-*]. Type genus: *Colydium* Fabricius, 1792 [placed on the Official List of Generic Names in Zoology (ICZN 1995c)].

### Tribe Gempylodini

Sharp, 1893

Gempylodini Sharp, 1893: 256 [stem: *Gempylod-*]. Type genus: *Gempylodes* Pascoe, 1863.

### Tribe Nematidiini

Horn, 1878

Nematidii G. H. Horn, 1878: 573 [stem: *Nematidi-*]. Type genus: *Nematidium* Erichson, 1845.

### Tribe Orthocerini

Blanchard, 1845

Sarrotriides Billberg, 1820a: 9 [stem: *Sarrotri-*]. Type genus: *Sarrotrium* Illiger, 1798 [syn. of *Orthocerus* Latreille, 1797]. Comment: the same name was also published in the same year by Billberg (1820b: 390); Sarrotriidae Billberg, 1820 placed on the Official Index of Rejected and Invalid Family-Group Names in Zoology (ICZN 1995c).
Orthocérites Blanchard, 1845b: 29 [stem: *Orthocer-*]. Type genus: *Orthocerus* Latreille, 1797 [placed on the Official List of Generic Names in Zoology (ICZN 1995c)]. Comment: Orthocerini Blanchard, 1845 placed on Official List of Family-group names in Zoology (ICZN 1995c).
Euglochidae Gistel, 1856a: 382 [stem: *Euglochin-*]. Type genus: *Euglochis* Gistel, 1856 [syn. of *Orthocerus* Latreille, 1797]. Comment: published 18 February 1856; this family-group name was also used in the same year by Gistel (1856b [“31 December”]: 180, as Euglochida); incorrect original stem formation, not in prevailing usage.

### Tribe Rhagoderini

Horn, 1878

Rhagoderini G. H. Horn, 1878: 557 [stem: *Rhagoder-*]. Type genus: *Rhagodera* Mannerheim, 1843.

### Tribe Rhopalocerini

Reitter, 1911

Apistini Ganglbauer, 1899: 873 [stem: *Apist-*]. Type genus: *Apistus* Motschulsky, 1840 [*Apistus* is an unjustified emendation of type genus name, originally spelled *Apeistus*, by Agassiz (1846b: 28), in prevailing usage; the emended name is a junior homonym of *Apistus* Cuvier, 1829 [Pisces]; *Apeistus* Motschulsky, 1840 and *Apistus* Agassiz, 1846 were placed on Official Index of Rejected and Invalid Generic Names in Zoology (ICZN 1986b); syn. of *Rhopalocerus* Redtenbacher, 1842]. Comment: permanently invalid (Art. 39): based on preoccupied type genus; placed on Official Index of Rejected and Invalid Family-Group Names in Zoology (ICZN 1986b).
Rhopalocerini Reitter, 1911: 108 [stem: *Rhopalocer-*]. Type genus: *Rhopalocerus* Redtenbacher, 1842 [placed on the Official List of Generic Names in Zoology (ICZN 1986b)]. Comment: placed on the Official List of Family-Group Names in Zoology (ICZN 1986b).

### Tribe Synchitini

Erichson, 1845

Synchitini Erichson, 1845: 254 [stem: *Synchit-*]. Type genus: *Synchita* Hellwig, 1792. Comment: published before 31 June 1845; this family-group name was also used in the same year by L. Redtenbacher (1845 [before September]: 123, as Synchitae).
Ditomidae Imhoff, 1856: [2] 159 [stem: *Bitom-*]. Type genus: *Bitoma* Herbst, 1793 [as *Ditoma*, unjustified emendation of type genus name by Illiger (1807), not in prevailing usage]. Comment: incorrect original stem formation, not in prevailing usage; stem of the family-group name corrected according to Art. 35.4.1 and also to remove from potential homonymy with the older family-group name originally proposed as Ditomici Bonelli, 1810 (type genus *Ditomus* Bonelli, 1810) in the family Carabidae.
Coxelini Seidlitz, 1872 [Gatt.]: 38 [stem: *Coxel-*]. Type genus: *Coxelus* Dejean, 1821.
Langelandiina Fowler, 1889: 192 [stem: *Langelandi-*]. Type genus: *Langelandia* Aubé, 1843.
Megataphrini Casey, 1890: 309 [stem: *Megataphr-*]. Type genus: *Megataphrus* Casey, 1890.
Tarphiinae Sharp, 1894: 444 [stem: *Tarphi-*]. Type genus: *Tarphius* Erichson, 1845.
Corticini Ganglbauer, 1899: 870 [stem: *Cortic-*]. Type genus: *Corticus* Latreille, 1829.
Diodesmini Reitter, 1911: 110 [stem: *Diodesmat-*]. Type genus: *Diodesma* Latreille, 1829. Comment: incorrect original stem formation, not in prevailing usage.
Trachypholini Grouvelle, 1911: 121 [stem: *Trachypholid-*]. Type genus: *Trachypholis* Erichson, 1845. Comment: incorrect original stem formation, not in prevailing usage.
Endophloeini Reitter, 1922a: 17 [stem: *Endophloe-*]. Type genus: *Endophloeus* Dejean, 1834.
Priolomini Dajoz, 1980a: 127 [stem: *Priolom-*]. Type genus: *Priolomus* Erichson, 1845.

### Subfamily Zopherinae

Solier, 1834

Zophérites Solier, 1834: 505 [stem: *Zopher-*]. Type genus: *Zopherus* Gray, 1832.

### Tribe Latometini

Ślipiński and Lawrence, 1999

Latometini Ślipiński and Lawrence, 1999: 9, in key [stem: *Latomet-*]. Type genus: *Latometus* Erichson, 1842.

### Tribe Monommatini

Blanchard, 1845

Monommites Blanchard, 1845b: 16 [stem: *Monommat-*]. Type genus: *Monomma* Klug, 1833. Comment: original vernacular name available (Art. 11.7.2): first used in latinized form by J. L. LeConte (1862: 246, as Monommidae [incorrect stem formation]), generally accepted as in Lawrence and Newton (1995: 890, as Monommatidae); incorrect original stem formation, not in prevailing usage.

### Tribe Phellopsini

Ślipiński and Lawrence, 1999

Phellopsini Ślipiński and Lawrence, 1999: 10, in key [stem: *Phellops-*]. Type genus: *Phellopsis* J. L. LeConte, 1862. Comment: current spelling maintained (Art. 29.5): incorrect stem formation in prevailing usage (should be *Phellopse*-).

### Tribe Pycnomerini

Erichson, 1845

Pycnomerini Erichson, 1845: 290 [stem: *Pycnomer-*]. Type genus: *Pycnomerus* Erichson, 1842.

### Tribe Usechini

Horn, 1867

Usechini G. H. Horn, 1867a: 294 [stem: *Usech-*]. Type genus: *Usechus* Motschulsky, 1845.

### Tribe Zopherini

Solier, 1834

Zophérites Solier, 1834: 505 [stem: *Zopher-*]. Type genus: *Zopherus* Gray, 1833. Comment: original vernacular name available (Art. 11.7.2): first used in latinized form by Agassiz (1846b: 392, as Zopheroidae), generally accepted as in Lawrence and Newton (1995: 891, as Zopheridae).
Nosodermini Casey, 1907: 280 [stem: *Nosodermat-*]. Type genus: *Nosoderma* Solier, 1841 [preoccupied genus name, not *Nosoderma* Guérin-Méneville, 1838 [Coleoptera: Zopheridae: Zopherinae: Zopherini]; syn. of *Verodes* Casey, 1907; see Foley and Ivie (2007) for comments on type genus]. Comment: permanently invalid (Art. 39): based on preoccupied type genus; all subsequent uses of Nosodermini were based on the junior homonym *Nosoderma* Solier, 1841 (see Foley and Ivie 2007); incorrect original stem formation, not in prevailing usage.
Zopherosini Casey, 1907: 522 [stem: *Zopherose-*]. Type genus: *Zopherosis* A. White, 1859. Comment: incorrect original stem formation, not in prevailing usage.

### Family Ulodidae

Pascoe, 1869

Ulodinae Pascoe, 1869a: 31 [stem: *Ulod-*]. Type genus: *Ulodes* Erichson, 1842.
Merycidae Crowson, 1953: 37 [stem: *Meryc-*]. Type genus: *Meryx* Latreille, 1802.

### Family Promecheilidae

Lacordaire, 1859

Proméchilides Lacordaire, 1859: 698 [stem: *Promecheil-*]. Type genus: *Promecheilus* Solier, 1851 [as *Promechilus*, unjustified emendation of type genus name by Lacordaire (1859: 700), not in prevailing usage]. Comment: original vernacular name available (Art. 11.7.2): first used in latinized form by Handlirsch (1925: 594, as Promecheilinae), generally accepted as in Lawrence et al. (2010: 563, as Promecheilidae); incorrect original stem formation, not in prevailing usage.
Perimylopidae St. George, 1939: 212 [stem: *Perimylop-*]. Type genus: *Perimylops* Müller, 1884.
*Parahelopinae Watt, 1975: 424 [stem: *Parahelop-*]. Type genus: *Parahelops* C. O. Waterhouse, 1876. Comment: unavailable family-group name, proposed after 1930 without description or bibliographic reference to such description (Art. 13.1); this name was used several times subsequently, e.g., Elgueta and Arriagada (1989: 37), Lawrence and Britton (1991: 665), Lawrence (1994: 339), but this taxon name remains unavailable (see Lawrence and Newton 1995: 892).

### Family Chalcodryidae

Watt, 1974

Chalcodryidae Watt, 1974: 24 [stem: *Chalcodry-*]. Type genus: *Chalcodrya* Redtenbacher, 1868.

### Family Trachelostenidae

Lacordaire, 1859

Trachélosténides Lacordaire, 1859: 567 [stem: *Trachelosten-*]. Type genus: *Trachelostenus* Solier, 1851. Comment: original vernacular name available (Art. 11.7.2): first used in latinized form by Seidlitz (1898: 319, as Trachelostenini), generally accepted as in Lawrence and Newton (1995: 892 as Trachelostenidae).

### Family Tenebrionidae

Latreille, 1802

Tenebrionites Latreille, 1802: 165 [stem: *Tenebrion-*]. Type genus: *Tenebrio* Linnaeus, 1758. Comment: First Reviser (Tenebrionidae Latreille, 1802 vs Pimeliidae Latreille, 1802 vs Diaperidae Latreille, 1802) not determined, current usage maintained.

### Subfamily Zolodininae

Watt, 1975

Zolodininae Watt, 1975: 401 [stem: *Zolodin-*]. Type genus: *Zolodinus* Blanchard, 1853.

### Subfamily Lagriinae

Latreille, 1825 (1820)

Lagriariae Latreille, 1825: 381 [stem: *Lagri-*]. Type genus: *Lagria* Fabricius, 1775. Comment: use of younger family-group name conserved over Lachninae Billberg, 1820 (Art. 40.2) (see Bouchard et al. 2005); the older name Cossyphinae Latreille, 1802 is not given precedence over this name because the current placement of Cossyphini in this subfamily is uncertain (see Bouchard et al. 2005).

### Tribe Adeliini

Kirby, 1828

Adeliadae Kirby, 1828: 525 [stem: *Adeli-*]. Type genus: *Adelium* Kirby, 1819. Comment: family-group name attributed to Hope (1840a: 188) in recent literature; this is a senior homonym of Adeliini Viereck, 1918 (type genus *Adelius* Haliday, 1833) which is considered a valid tribe in the Hymenoptera: Braconidae: Cheloninae; this case is to be referred to the Commission to remove the homonymy (Art. 55.3.1).
*Apatèlates Mulsant and Rey, 1859: 87 [stem: *Apatel-*]. Type genus: *Apatelus* Mulsant and Rey, 1859. Comment: original vernacular name unavailable (Art. 11.7.2): subsequently used in latinized form, e.g., Heyne and Taschenberg (1907: 208, as Apatelini), but not generally accepted as valid; if evidence is found in the future that would lead to the treatment of Mulsant and Rey’s name as available, this would threaten the younger name originally proposed as Apatelinae Grote, 1883 (type genus *Apatele* Hübner, 1822) in Lepidoptera which has been used as valid in recent literature.

### Tribe Belopini

Reitter, 1917

Calcariens Mulsant, 1854: 268 [stem: *Calcar-*]. Type genus: *Calcar* Dejean, 1821 [preoccupied genus name, not *Calcar* de Montfort, 1810 [Mollusca]; syn. of *Centorus* Mulsant, 1854]. Comment: original vernacular name available (Art. 11.7.2): first used in latinized form and generally accepted as in Seidlitz (1895: 647, as Calcarina); permanently invalid (Art. 39): based on preoccupied type genus.
Belopinae Reitter, 1917: 59 [stem: *Belop-*]. Type genus: *Belopus* Gebien, 1911. Comment: current spelling maintained (Art. 29.5): incorrect stem formation in prevailing usage (should be *Belopod*-).

### Tribe Chaerodini

Doyen, Matthews and Lawrence, 1990

Chaerodini Doyen et al., 1990: 239 [stem: *Chaerod-*]. Type genus: *Chaerodes* A. White, 1846.

### Tribe Cossyphini

Latreille, 1802

Cossyphores Latreille, 1802: 164 [stem: *Cossyph-*]. Type genus: *Cossyphus* A. G. Olivier, 1791. Comment: not given precedence over Lagriinae Latreille, 1825 because its current placement is uncertain (see Bouchard et al. 2005).

### Tribe Goniaderini

Lacordaire, 1859

Goniadérides Lacordaire, 1859: 390 [stem: *Goniader-*]. Type genus: *Goniadera* Perty, 1832. Comment: original vernacular name available (Art. 11.7.2): first used in latinized form by Seidlitz (1895: 614, as Goniaderina), generally accepted as in Gebien (1911: 467, as Goniaderinae).
*Phobéliides Lacordaire, 1859: 393 [stem: *Phobeli-*]. Type genus: *Phobelius* Blanchard, 1845. Comment: original vernacular name unavailable (Art. 11.7.2): subsequently used in latinized form, e.g., Heyne and Taschenberg (1907: 212, as Phobeliini), but not generally accepted as valid.
Eschatoporini Blaisdell, 1906: 78 [stem: *Eschatopori-*]. Type genus: *Eschatoporis* Blaisdell, 1906. Comment: incorrect original stem formation, not in prevailing usage.
Phobeliina Ardoin, 1961: 33 [stem: *Phobeli-*]. Type genus: *Phobelius* Blanchard, 1845.
*Anaedini Skopin, 1964: 7 [stem: *Anaed-*]. Type genus: *Anaedus* Blanchard, 1845. Comment: unavailable family-group name, proposed after 1930 without description or bibliographic reference to such a description (Art. 13.1).

### Tribe Laenini

Seidlitz, 1895

Laenina Seidlitz, 1895: 669 [stem: *Laen-*]. Type genus: *Laena* Dejean, 1821.

### Tribe Lagriini

Latreille, 1825 (1820)

Lagriariae Latreille, 1825: 381 [stem: *Lagri-*]. Type genus: *Lagria* Fabricius, 1775. Comment: use of younger family-group name conserved over Lachnini Billberg, 1820 (Art. 40.2) (see Bouchard et al. 2005).

### Subtribe Lagriina

Latreille, 1825 (1820)

*Lagrien Oken, 1817: 1180 [stem: *Lagri-*]. Type genus: *Lagria* Fabricius, 1775. Comment: original vernacular name unavailable (Art. 11.7.2): subsequently used in latinized form but not generally attributed to Oken (1817).
Lachnaedes Billberg, 1820a: 34 [stem: *Lachn-*]. Type genus: *Lachna* Billberg, 1820 [syn. of *Lagria* Fabricius, 1775]. Comment: the family-group names Lachnidae/-inae/-ini Herrich-Schaeffer 1854 (based on *Lachnus* H. C. C. Burmeister 1835) are currently used as valid in Hemiptera, Lachninae (incorrectly attributed to Passerini (1863)) was placed on the Official List of Family-Group Names in Zoology (ICZN 1956); this case is to be referred to the Commission to remove the homonymy (Art. 55.3.1).
Lagriariae Latreille, 1825: 381 [stem: *Lagri-*]. Type genus: *Lagria* Fabricius, 1775. Comment: use of younger family-group name conserved over Lachnina Billberg, 1820 (Art. 40.2) (see Bouchard et al. 2005).
Loubacantini Bonadona, 1959: 1034 [stem: *Loubacant-*]. Type genus: *Loubacantus* Bonadona, 1959 [syn. of *Entypodera* Gerstaecker, 1871]. Comment: originally proposed in Anthicidae, transfer to Lagriinae by Bonadona (1984).

### Subtribe Statirina

Blanchard, 1845

Statyrites Blanchard, 1845b: 39 [stem: *Statir-*]. Type genus: *Statira* Lepeletier and Audinet-Serville, 1828 [as *Statyra*, incorrect subsequent spelling of type genus name, not in prevailing usage]. Comment: original vernacular name available (Art. 11.7.2): first used in latinized form by J. L. LeConte (1862: 246, as Statyrini), generally accepted as in Bouchard et al. (2005: 501, as Statyrina); incorrect original stem formation, not in prevailing usage.
*Hystérarthrides Lacordaire, 1869: 231 [stem: *Hysterarthr-*]. Type genus: *Hysterarthron* J. Thomson, 1864. Comment: original vernacular name unavailable (Art. 11.7.2): subsequently used in latinized form, e.g., Heyne and Taschenberg (1907: 240, as Hysterarthrini), but not generally accepted as valid; type genus transferred from Cerambycidae by Ritsema (1892: 54).

### Tribe Lupropini

Ardoin, 1958

Lupropsini Ardoin, 1958: 59 [stem: *Luprop-*]. Type genus: *Luprops* Hope, 1833. Comment: incorrect original stem formation, not in prevailing usage.

### Tribe Pycnocerini

Lacordaire, 1859 nomen protectum

Chiroscelidae Hope, 1840a: 127 [stem: *Chiroscelid-*]. Type genus: *Chiroscelis* Lamarck, 1804. Comment: published before 30 September 1840; Bouchard et al. (2005: 524) treated this name and Chiroscelites Laporte (1840b [before 26 December]: 216) as *nomina oblita*; incorrect original stem formation, not in prevailing usage.
Pycnocérides Lacordaire, 1859: 399 [stem: *Pycnocer-*]. Type genus: *Pycnocerus* Westwood, 1841. Comment: *nomen protectum* (see Bouchard et al. 2005: 524); original vernacular name available (Art. 11.7.2): first used in latinized form by Kolbe (1887: 50, as Pycnoceridae), generally accepted as in Bouchard et al. (2005: 501, as Pycnocerini).
Prioscelina Skopin, 1964: 30, in key [stem: *Prioscelid-*]. Type genus: *Prioscelis* Hope, 1840. Comment: incorrect original stem formation, not in prevailing usage.

### Subfamily Nilioninae

Oken, 1843

Nilioniden Oken, 1843: 484 [stem: *Nilion-*]. Type genus: *Nilio* Latreille, 1802 [incorrect subsequent spelling of *Nilion* by Latreille (1804b: 333), incorrect subsequent spelling in prevailing usage, treated as correct original spelling (Art. 33.3.1)]. Comment: original vernacular name available (Art. 11.7.2): first used in latinized form and generally accepted as in Tulk (1847: 614, as Niliondae [incorrect stem formation]); family-group name previously attributed to Lacordaire (1859: 518).

### Subfamily Phrenapatinae

Solier, 1834

Phrépatides Solier, 1834: 488 [stem: *Phrenapat-*]. Type genus: *Phrenapates* Gray, 1832.

### Tribe Archaeoglenini

Watt, 1975

Archaeoglenini Watt, 1975: 412 [stem: *Archaeoglen-*]. Type genus: *Archaeoglenes* Broun, 1893.

### Tribe Penetini

Lacordaire, 1859

Pénétides Lacordaire, 1859: 318 [stem: *Penet-*]. Type genus: *Peneta* Lacordaire, 1859. Comment: original vernacular name available (Art. 11.7.2): first used in latinized form by Seidlitz (1894: 545, as Penetina), generally accepted as in Bouchard et al. (2005: 501, as Penetini).
Phthorini Boddy, 1965: 144 [stem: *Phtor-*]. Type genus: *Phtora* Mulsant, 1854 [as *Phthora*, incorrect subsequent spelling of type genus name, not in prevailing usage; *Phtora* is a junior homonym of *Phtora* Germar, 1836 [Coleoptera: Tenebrionidae: Diaperinae]; syn. of *Clamoris* Gozis, 1886]. Comment: permanently invalid (Art. 39): based on preoccupied type genus.

### Tribe Phrenapatini

Solier, 1834

Phrépatides Solier, 1834: 488 [stem: *Phrenapat-*]. Type genus: *Phrenapates* Gray, 1832 [as *Phrepates*, incorrect subsequent spelling of type genus name, not in prevailing usage]. Comment: original vernacular name available (Art. 11.7.2): first used in latinized form by Agassiz (1846b: 287, as Phrenapatoidae), generally accepted as in Bouchard et al. (2005: 501, as Phrenapatinae/-ini); incorrect original stem formation, not in prevailing usage.

### Subfamily Pimeliinae

Latreille, 1802

Pimeliariae Latreille, 1802: 166 [stem: *Pimeli-*]. Type genus: *Pimelia* Fabricius, 1775.

### Tribe Adelostomini

Solier, 1834

Adélostomites Solier, 1834: 502 [stem: *Adelostom-*]. Type genus: *Adelostoma* Duponchel, 1827. Comment: original vernacular name available (Art. 11.7.2): first used in latinized form by Agassiz (1846b: 8, as Adelostomoidae), generally accepted as in Bouchard et al. (2005: 501, as Adelostomini); current spelling maintained (Art. 29.5): incorrect stem formation in prevailing usage (should be *Adelostomat*-).
*Eurychorites Solier, 1837b: 153 [stem: *Eurychor-*]. Type genus: *Eurychora* Thunberg, 1791. Comment: original vernacular name unavailable (Art. 11.7.2): subsequently used in latinized form but not generally attributed to Solier (1837).
Eurychoridae Hope, 1840a: 121 [stem: *Eurychor-*]. Type genus: *Eurychora* Thunberg, 1791.

### Tribe Adesmiini

Lacordaire, 1859 nomen protectum

*Macropodites Solier, 1834: 501 [stem: *Macropod-*]. Type genus: *Macropoda* Solier, 1835. Comment: unavailable family-group name, not based on an available genus name.
*Macropodites Solier, 1835b: 509 [stem: *Macropod-*]. Type genus: *Macropoda* Solier, 1835. Comment: original vernacular name unavailable (Art. 11.7.2): subsequently used in latinized form but not generally accepted as valid.
*Adesmiites Blanchard, 1845b: 4 [stem: *Adesmi-*]. Type genus: *Adesmia* Fischer von Waldheim, 1822. Comment: original vernacular name unavailable (Art. 11.7.2): subsequently used in latinized form but not generally attributed to Blanchard (1845b).
Macropodoidae Agassiz, 1846b: 221 [stem: *Macropod-*]. Type genus: *Macropoda* Solier, 1835. Comment: *nomen oblitum* (Appendix 1); this name is a junior homonym of Macropodidae Gray, 1821 in Mammalia (type genus *Macropus* Shaw and Nodder, 1790) which is on the Official List of Family-Group Names in Zoology.
Megagenianos Solier, 1851: 124 [stem: *Megageni-*]. Type genus: *Megagenius* Solier, 1835. Comment: original vernacular name available (Art. 11.7.2): first used in latinized form by Kolbe (1887: 50, as Megageniidae), generally accepted as in Bouchard et al. (2007: 386, as Megageniini); *nomen oblitum*, Adesmiini Lacordaire, 1859 conserved over this name by Bouchard et al. (2007: 386).
Adesmiides Lacordaire, 1859: 22 [stem: *Adesmi-*]. Type genus: *Adesmia* Fischer von Waldheim, 1822. Comment: *nomen protectum* (Appendix 1); original vernacular name available (Art. 11.7.2): first used in latinized form by Baudi di Selve (1875: 22, as Adesmidae [incorrect stem formation]), generally accepted as in Bouchard et al. (2005: 502); also Adesmiini Lacordaire, 1859 conserved over Megageniini Solier, 1851 by Bouchard et al. (2007: 386).
Épiphysides Lacordaire, 1859: 29 [stem: *Epiphys-*]. Type genus: *Epiphysa* Dejean, 1834. Comment: original vernacular name available (Art. 11.7.2): first used in latinized form by J. L. LeConte (1862: 213, as Epiphysini), generally accepted as in Gebien (1910a: 82, as Epiphysinae).

### Tribe Akidini

Billberg, 1820

Acides Billberg, 1820a: 32 [stem: *Akid-*]. Type genus: *Akis* Herbst, 1799 [as *Acis*, unjustified emendation of type genus by Billberg (1820a), not in prevailing usage]. Comment: incorrect original stem formation, not in prevailing usage.

### Tribe Anepsiini

LeConte, 1862

Anepsiini J. L. LeConte, 1862: 215 [stem: *Anepsi-*]. Type genus: *Anepsius* J. L. LeConte, 1851.
Batuliini G. H. Horn, 1870: 270 [stem: *Batuli-*]. Type genus: *Batulius* J. L. LeConte, 1851.
Anchommini G. H. Horn, 1878: 558 [stem: *Anchommat-*]. Type genus: *Anchomma* J. L. LeConte, 1858 [genus originally proposed in Colydiidae]. Comment: incorrect original stem formation, not in prevailing usage.

### Tribe Asidini

Fleming, 1821

Asidadae Fleming, 1821: 51 [stem: *Asid-*]. Type genus: *Asida* Latreille, 1802.
*Machlides Lacordaire, 1859: 155 [stem: *Machl-*]. Type genus: *Machla* Herbst, 1799 [earlier usage of the type genus name by Lichtenstein (1796) was suppressed for nomenclatural purposes (ICZN 1995e); this genus is currently known under the name *Pseudomachla* Wilke, 1921]. Comment: original vernacular name unavailable (Art. 11.7.2): subsequently used in latinized form and attributed to the original author but not used as valid then (Bouchard et al. 2005: 506).
Astroti G. H. Horn, 1870: 289 [stem: *Astrot-*]. Type genus: *Astrotus* J. L. LeConte, 1858.
Craniotini J. L. LeConte and G. H. Horn, 1883: 361 [stem: *Craniot-*]. Type genus: *Craniotus* J. L. LeConte, 1851.
Machlini Chatanay, 1914: 1 [stem: *Machl-*]. Type genus: *Machla* Herbst, 1799 [earlier usage of the type genus name by Lichtenstein (1796) was suppressed for nomenclatural purposes (ICZN 1995e); this genus is currently known under the name *Pseudomachla* Wilke, 1921].
Parecatini Chatanay, 1914: 2 [stem: *Parecat-*]. Type genus: *Parecatus* Fairmaire, 1900.

### Tribe Boromorphini

Skopin, 1978

Boromorphini Skopin, 1978: 228, in key [stem: *Boromorph-*]. Type genus: *Boromorphus* Wollaston, 1854.

### Tribe Branchini

LeConte, 1862

Branchini J. L. LeConte, 1862: 222 [stem: *Branch-*]. Type genus: *Branchus* J. L. LeConte, 1862.

### Tribe Caenocrypticini

Koch, 1958

Caenocrypticini Koch, 1958: 121 [stem: *Caenocryptic-*]. Type genus: *Caenocrypticus* Gebien, 1920.

### Tribe Ceratanisini

Gebien, 1937

Apolitina Seidlitz, 1895: 666 [stem: *Apolit-*]. Type genus: *Apolites* Jacquelin du Val, 1861 [preoccupied genus name, not *Apolites* Sundevall, 1835 [Aves]; syn. of *Idastrandiella* Strand, 1929]. Comment: permanently invalid (Art. 39): based on preoccupied type genus.
Anisocerini Reitter, 1906: 477 [stem: *Anisocer-*]. Type genus: *Anisocerus* Faldermann, 1837 [preoccupied genus name, not *Anisocerus* Audinet-Serville, 1835 [Coleoptera: Cerambycidae]; syn. of *Ceratanisus* Gemminger, 1870]. Comment: junior homonym of Anisocerini J. Thomson, 1860 [Coleoptera: Cerambycidae]; permanently invalid (Art. 39): based on preoccupied type genus.
Ceratanisini Gebien, 1937: 791 [stem: *Ceratanis-*]. Type genus: *Ceratanisus* Gemminger, 1870.

### Tribe Cnemeplatiini

Jacquelin du Val, 1861

Cnéméplatiites Jacquelin du Val, 1861: 286 [stem: *Cnemeplati-*]. Type genus: *Cnemeplatia* A. Costa, 1847.

### Subtribe Actizetina

Watt, 1992

Actizetina Watt, 1992: 297 [stem: *Actizet-*]. Type genus: *Actizeta* Pascoe, 1875.

### Subtribe Cnemeplatiina

Jacquelin du Val, 1861

*Autocérides Lacordaire, 1859: 279 [stem: *Autocer-*]. Type genus: *Autocera* Wollaston, 1857 [syn. of *Cnemeplatia* A. Costa, 1847]. Comment: original vernacular name unavailable (Art. 11.7.2): subsequently used in latinized form, e.g., Heyne and Taschenberg (1907: 208, as Autocerini), but not generally accepted as valid.
Cnéméplatiites Jacquelin du Val, 1861: 286 [stem: *Cnemeplati-*]. Type genus: *Cnemeplatia* A. Costa, 1847. Comment: Bouchard et al. (2005: 504) considered Jacquelin du Val’s name as unavailable based on the requirements of Art. 11.7.2, however, the recent usage of «Cnemeplatiini Jacquelin du Val, 1861” as a valid taxon by Löbl et al. (2008: 140) made this name available.
Cnemeplatiini Csiki, 1953: 117 [stem: *Cnemeplati-*]. Type genus: *Cnemeplatia* A. Costa, 1847. Comment: proposed as new without reference to Cnéméplatiites Jacquelin du Val, 1861.

### Subtribe Rondoniellina

Ferrer and Moragues, 2000

Rondoniellina Ferrer and Moragues, 2000: 100 [stem: *Rondoniell-*]. Type genus: *Rondoniella* Kaszab, 1970.

### Subtribe Thorictosomatina

Watt, 1992

Thorictosomatina Watt, 1992: 296 [stem: *Thorictosomat-*]. Type genus: *Thorictosoma* Lea, 1919.

### Tribe Cnemodinini

Gebien, 1910

Cnemodini G. H. Horn, 1870: 266 [stem: *Cnemodont-*]. Type genus: *Cnemodus* G. H. Horn, 1870 [preoccupied genus name, not *Cnemodus* Herrich-Schaeffer, 1850 [Hemiptera]; syn. of *Cnemodinus* Cockerell, 1906]. Comment: permanently invalid (Art. 39): based on preoccupied type genus; incorrect original stem formation, not in prevailing usage.
Cnemodininae Gebien, 1910a: 4 [stem: *Cnemodin-*]. Type genus: *Cnemodinus* Cockerell, 1906.

### Tribe Coniontini

Waterhouse, 1858

Coniontidae G. R. Waterhouse, 1858: 59 [stem: *Coniont-*]. Type genus: *Coniontis* Eschscholtz, 1829. Comment: family-group name previously attributed to Lacordaire (1859)/Schaum (1859) (see Bouchard et al. 2005: 502).
Coelini Casey, 1907: 500 [stem: *Coel-*]. Type genus: *Coelus* Eschscholtz, 1829.
Eusatti Doyen, 1984: 11 [stem: *Eusatt-*]. Type genus: *Eusattus* J. L. LeConte, 1851.

### Tribe Cossyphodini

Wasmann, 1899

Cossyphodidae Wasmann, 1899: 161 [stem: *Cossyphod-*]. Type genus: *Cossyphodes* Westwood, 1851. Comment: downgraded from a subfamily of Tenebrionidae to a tribe of Pimeliinae by Matthews et al. (2010).

### Subtribe Cossyphodina

Wasmann, 1899

Cossyphodidae Wasmann, 1899: 161 [stem: *Cossyphod-*]. Type genus: *Cossyphodes* Westwood, 1851.

### Subtribe Cossyphoditina

Basilewsky, 1950

Cossyphoditinae Basilewsky, 1950a: 187 [stem: *Cossyphodit-*]. Type genus: *Cossyphodites* Brauns, 1901.

### Subtribe Esemephina

Steiner, 1980

Esemephini Steiner, 1980: 391 [stem: *Esemeph-*]. Type genus: *Esemephe* Steiner, 1980.

### Subtribe Paramellonina

Andreae, 1961

Paramelloninae Andreae, 1961: 200 [stem: *Paramellon-*]. Type genus: *Paramellon* C. O. Waterhouse, 1882. Comment: current spelling maintained (Art. 29.5): incorrect stem formation in prevailing usage (should be *Paramellont*-).

### Tribe Cryptochilini

Solier, 1841

Cryptochilites Solier, 1841: 248 [stem: *Cryptochil-*]. Type genus: *Cryptochile* Latreille, 1829.

### Subtribe Calognathina

Lacordaire, 1859

Calognathides Lacordaire, 1859: 85 [stem: *Calognath-*]. Type genus: *Calognathus* Guérin-Méneville, 1836. Comment: original vernacular name available (Art. 11.7.2): first used in latinized form by Kolbe (1887: 50, as Calognathidae), generally accepted as in Gebien (1910a: 117, as Calognathinae).

### Subtribe Cryptochilina

Solier, 1841

Cryptochilites Solier, 1841: 248 [stem: *Cryptochil-*]. Type genus: *Cryptochile* Latreille, 1829. Comment: original vernacular name available (Art. 11.7.2): first used in latinized form by Kolbe (1887: 50, as Cryptochilidae), generally accepted as in Bouchard et al. (2005: 502, as Cryptochilini).

### Subtribe Homebiina

Endrödy-Younga, 1989

Homebiina Endrödy-Younga, 1989: 124 [stem: *Homebi-*]. Type genus: *Homebius* Endrödy-Younga, 1989.

### Subtribe Horatomina

Koch, 1955

Horatomina Koch, 1955: 14 [stem: *Horatom-*]. Type genus: *Horatoma* Solier, 1840.

### Subtribe Vansoniina

Koch, 1955

Vansonini Koch, 1955: 12 [stem: *Vansoni-*]. Type genus: *Vansonium* Koch, 1950. Comment: incorrect original stem formation, not in prevailing usage.

### Tribe Cryptoglossini

LeConte, 1862 nomen protectum

Centrioptérides Lacordaire, 1859: 134 [stem: *Centriopter-*]. Type genus: *Centrioptera* Mannerheim, 1843. Comment: original vernacular name available (Art. 11.7.2): first used in latinized form by J. L. LeConte (1862: 220, as Centriopterae), generally accepted as in Bouchard et al. (2005: 502, as Centriopterini); *nomen oblitum* (see Aalbu 2006: 57).
Cryptoglossini J. L. LeConte, 1862: 220 [stem: *Cryptogloss-*]. Type genus: *Cryptoglossa* Solier, 1837. Comment: *nomen protectum* (see Aalbu 2006: 57).

### Tribe Edrotini

Lacordaire, 1859

Édrotides Lacordaire, 1859: 31 [stem: *Edrot-*]. Type genus: *Edrotes* J. L. LeConte, 1851. Comment: original vernacular name available (Art. 11.7.2): first used in latinized form by Casey (1907: 279, as Edrotini), generally accepted as in Bouchard et al. (2005: 502, as Edrotini).
Triorophi J. L. LeConte and G. H. Horn, 1883: 362 [stem: *Trioroph-*]. Type genus: *Triorophus* J. L. LeConte, 1851.
Auchmobii J. L. LeConte and G. H. Horn, 1883: 362 [stem: *Auchmobi-*]. Type genus: *Auchmobius* J. L. LeConte, 1851.
Trimytini Casey, 1907: 278 [stem: *Trimytid-*]. Type genus: *Trimytis* Leconte, 1851. Comment: incorrect original stem formation, not in prevailing usage.
Eurymetoponini Casey, 1907: 278 [stem: *Eurymetop-*]. Type genus: *Eurymetopon* Eschscholtz, 1831. Comment: incorrect original stem formation, not in prevailing usage.
Trientomini Casey, 1907: 278 [stem: *Trientom-*]. Type genus: *Trientoma* Solier, 1835.

### Tribe Elenophorini

Solier, 1837

Elénophorites Solier, 1837a: 638 [stem: *Elenophor-*]. Type genus: *Elenophorus* Dejean, 1821 [syn. of *Leptoderis* Billberg, 1820]. Comment: original vernacular name available (Art. 11.7.2): first used in latinized form by Schaum (1859: 66, as Elenophoridae), generally accepted as in Bouchard et al. (2005: 502, as Elenophorini).

### Tribe Epitragini

Blanchard, 1845 nomen protectum

Lygophilia Rafinesque, 1815: 113 [stem: *Lygophil-*]. Type genus: *Lygophilus* Rafinesque, 1815 [syn. of *Epitragus* Latreille, 1802]. Comment: *nomen oblitum* (see Bouchard et al. 2007: 386).
*Épitragites Solier, 1834: 490 [stem: *Epitrag-*]. Type genus: *Epitragus* Latreille, 1802. Comment: original vernacular name unavailable (Art. 11.7.2): subsequently used in latinized form but not generally attributed to Solier (1834).
Épitragites Blanchard, 1845b: 16 [stem: *Epitrag-*]. Type genus: *Epitragus* Latreille, 1802. Comment: original vernacular name available (Art. 11.7.2): first used in latinized form by Erichson (1847a: 117, as Epitragii), generally accepted as in Bouchard et al. (2005: 502, as Epitragini); *nomen protectum* (see Bouchard et al. 2007: 386).

### Tribe Erodiini

Billberg, 1820 nomen protectum

Cephaceria Rafinesque, 1815: 113 [stem: *Cephacer-*]. Type genus: *Cephacerus* Rafinesque, 1815 [syn. of *Erodius* Fabricius, 1775]. Comment: *nomen oblitum* (see Bouchard et al. 2007: 386).
Erodiides Billberg, 1820a: 32 [stem: *Erodi-*]. Type genus: *Erodius* Fabricius, 1775. Comment: *nomen protectum* (see Bouchard et al. 2007: 386); this family-group name was also used in the same year by Billberg (1820b: 392, as Erodiides).
*Estenogenianos Solier, 1851: 138 [stem: *Stenogeni-*]. Type genus: *Stenogenius* Solier, 1834 [unavailable genus name, proposed in synonymy and not subsequently made available]. Comment: family-group name unavailable (Art. 11.7.1.1): not based on an available genus name at the time; incorrect original stem formation, not in prevailing usage.
*Arthrodeiden Koch, 1943: 483 [stem: *Arthrode-*]. Type genus: *Arthrodeis* Solier, 1834. Comment: original vernacular name unavailable (Art. 11.7.2): proposed after 1899.

### Tribe Evaniosomini

Lacordaire, 1859

Évaniosomides Lacordaire, 1859: 73 [stem: *Evaniosom-*]. Type genus: *Evaniosomus* Guérin-Méneville, 1834. Comment: original vernacular name available (Art. 11.7.2): first used in latinized form and generally accepted as in Gebien (1910a: 22, as Evaniosominae).

### Tribe Falsomycterini

Gebien, 1910

Falsomycterinae Gebien, 1910b: 177 [stem: *Falsomycter-*]. Type genus: *Falsomycterus* Pic, 1907.

### Tribe Idisiini

Medvedev, 1973

Idisiini G. S. Medvedev, 1973: 644 [stem: *Idisi-*]. Type genus: *Idisia* Pascoe, 1866.

### Tribe Klewariini

Gebien, 1910

Klewariinae Gebien, 1910a: 36 [stem: *Klewari-*]. Type genus: *Klewaria* Reitter, 1910.

### Tribe Kuhitangiini

Medvedev, 1962

Kuhitangiinae G. S. Medvedev, 1962: 1184 [stem: *Kuhitangi-*]. Type genus: *Kuhitangia* G. S. Medvedev, 1962.

### Tribe Lachnogyini

Seidlitz, 1894

Lachnogyini Seidlitz, 1894: 490 [stem: *Lachnogy-*]. Type genus: *Lachnogya* Ménétriés, 1849. Comment: subtribal classification according to G. S. Medvedev (2006).

### Subtribe Lachnodactylina

Reitter, 1904

Lachnodactylina Reitter, 1904: 182 [stem: *Lachnodactyl-*]. Type genus: *Lachnodactylus* Seidlitz, 1898.

### Subtribe Lachnogyina

Seidlitz, 1894

Lachnogyini Seidlitz, 1894: 490 [stem: *Lachnogy-*]. Type genus: *Lachnogya* Ménétriés, 1849.

### Subtribe Netuschiliina

Ferrer and Yvinec, 2004

Netuschiliina Ferrer and Yvinec, 2004: 48 [stem: *Netuschili-*]. Type genus: *Netuschilia* Reitter, 1904. Comment: also incorrectly spelled as Netuschilina in the original publication on page 48 (First Revisers are Bouchard et al. 2005: 504).

### Tribe Leptodini

Lacordaire, 1859

Leptodides Lacordaire, 1859: 108 [stem: *Leptod-*]. Type genus: *Leptodes* Dejean, 1834. Comment: original vernacular name available (Art. 11.7.2): first used in latinized form by Baudi di Selve (1875: 74, as Leptodidae), generally accepted as in Gebien (1910a: 91, as Leptodinae).

### Tribe Nycteliini

Solier, 1834

Nyctélites Solier, 1834: 502 [stem: *Nycteli-*]. Type genus: *Nyctelia* Latreille, 1825. Comment: original vernacular name available (Art. 11.7.2): first used in latinized form by Hope (1840a: 127, as Nyctelidae [incorrect stem formation]), generally accepted as in Bouchard et al. (2005: 502, as Nycteliini); incorrect original stem formation, not in prevailing usage.

### Tribe Nyctoporini

Lacordaire, 1859

Nyctoporides Lacordaire, 1859: 130 [stem: *Nyctopor-*]. Type genus: *Nyctoporis* Eschscholtz, 1831. Comment: original vernacular name available (Art. 11.7.2): first used in latinized form by J. L. LeConte (1862: 219, as Nyctoporini), generally accepted as in Bouchard et al. (2005: 502, as Nyctoporini); current spelling maintained (Art. 29.5): incorrect stem formation in prevailing usage (should be *Nyctoporid*-).

### Tribe Phrynocarenini

Gebien, 1928

Phrynocareninae Gebien, 1928: 105 [stem: *Phrynocaren-*]. Type genus: *Phrynocarenum* Gebien, 1928.

### Tribe Physogasterini

Lacordaire, 1859

Physogastérides Lacordaire, 1859: 206 [stem: *Physogaster-*]. Type genus: *Physogaster* Lacordaire, 1830 [this genus name has been credited to Guérin-Méneville (1834: 2) in the literature, however the description of *Physogaster mendocinus* by Lacordaire (1830a: 276) makes the genus name available from that author and year]. Comment: original vernacular name available (Art. 11.7.2): first used in latinized form by H. C. C. Burmeister (1875: 488, as Physogasteridae), generally accepted as in Bouchard et al. (2005: 502, as Physogasterini).

### Tribe Pimeliini

Latreille, 1802

Pimeliariae Latreille, 1802: 166 [stem: *Pimeli-*]. Type genus: *Pimelia* Fabricius, 1775.
Pimidinia Rafinesque, 1815: 113 [stem: *Pimidi-*]. Type genus: *Pimidia* Rafinesque, 1815 [syn. of *Pimelia* Fabricius, 1775]. Comment: incorrect original stem formation, not in prevailing usage.
*Platyopes Motschulsky, 1849: 58 [stem: *Platyop-*]. Type genus: *Platyope* Fischer von Waldheim, 1820. Comment: original vernacular name unavailable (Art. 11.7.2): subsequently used in latinized form but not generally attributed to Motschulsky (1849).
Platyopidae Semenov, 1893a: 260 [stem: *Platyop-*]. Type genus: *Platyope* Fischer von Waldheim, 1820. Comment: family-group name proposed as new without reference to *Platyopes* Motschulsky, 1849; Platyopidae Huene, 1931 has been used in amphibians (type genus *Platyops* Twelvetrees, 1880) but this name is permanently invalid since it is based on a preoccupied genus name.
Leucolaephusini Pierre, 1961: 558 [stem: *Leucolaeph-*]. Type genus: *Leucolaephus* Lucas, 1859. Comment: incorrect original stem formation, not in prevailing usage.

### Tribe Praociini

Eschscholtz, 1829

Praocidae Eschscholtz, 1829b: 5 [stem: *Praoci-*]. Type genus: *Praocis* Eschscholtz, 1829. Comment: incorrect original stem formation, not in prevailing usage.

### Tribe Sepidiini

Eschscholtz, 1829

Sepidiae Eschscholtz, 1829b: 4 [stem: *Sepidi-*]. Type genus: *Sepidium* Fabricius, 1775.

### Subtribe Hypomelina

Koch, 1955

Hypomelina Koch, 1955: 36 [stem: *Hypomel-*]. Type genus: *Hypomelus* Solier, 1843.

### Subtribe Molurina

Solier, 1834

Molurites Solier, 1834: 505 [stem: *Molur-*]. Type genus: *Moluris* Latreille, 1802. Comment: original vernacular name available (Art. 11.7.2): first used in latinized form by Schaum (1859: 68, as Moluridae), generally accepted as in Bouchard et al. (2005: 502, as Molurina); current spelling maintained (Art. 29.3.1.1): incorrect stem formation in prevailing usage (should be *Molurid*-).
*Psammodoiden Koch, 1953a: 138 [stem: *Psammod-*]. Type genus: *Psammodes* Kirby, 1819. Comment: original vernacular name unavailable (Art. 11.7.2): proposed after 1899; Psammodidae has been used in the Scarabaeidae literature but this is based on an incorrect stem formation (type genus *Psammodius* Fallén, 1807, stem *Psammodi*-).
*Phrynocoloiden Koch, 1953a: 138 [stem: *Phrynocol-*]. Type genus: *Phrynocolus* Lacordaire, 1859. Comment: original vernacular name unavailable (Art. 11.7.2): proposed after 1899.

### Subtribe Oxurina

Koch, 1955

Oxurina Koch, 1955: 34 [stem: *Oxur-*]. Type genus: *Oxura* Kirby, 1819.

### Subtribe Phanerotomeina

Koch, 1958

*Phanerotomoide Koch, 1953a: 138 [stem: *Phanerotom-*]. Type genus: *Phanerotoma* Solier, 1843 [preoccupied genus name, not *Phanerotoma* Wesmael, 1838 [Hymenoptera]; syn. of *Phanerotomea* Koch, 1957]. Comment: family-group name unavailable (Art. 11.7.1.1): original name not proposed as a noun.
Phanerotomina Koch, 1955: 37 [stem: *Phanerotom-*]. Type genus: *Phanerotoma* Solier, 1843 [preoccupied genus name, not *Phanerotoma* Wesmael, 1838 [Hymenoptera]; syn. of *Phanerotomea* Koch, 1957]. Comment: permanently invalid (Art. 39): based on preoccupied type genus; Phanerotomini Baker, 1925 (type genus *Phanerotoma* Wesmael, 1838) is available in Hymenoptera: Braconidae.
Phanerotomeina Koch, 1958: 58 [stem: *Phanerotome-*]. Type genus: *Phanerotomea* Koch, 1958. Comment: replacement name for Phanerotomina Koch, 1955 because of the homonymy of the type genus.

### Subtribe Sepidiina

Eschscholtz, 1829

Sepidiae Eschscholtz, 1829b: 4 [stem: *Sepidi-*]. Type genus: *Sepidium* Fabricius, 1775.

### Subtribe Trachynotina

Koch, 1955

*Trachynotides Brullé, 1832: 189 [stem: *Trachynot-*]. Type genus: *Trachynotus* Latreille, 1828. Comment: original vernacular name unavailable (Art. 11.7.2): subsequently used in latinized form but not generally attributed to Brullé (1832).
Trachynotina Koch, 1955: 34, in key [stem: *Trachynot-*]. Type genus: *Trachynotus* Latreille, 1828. Comment: Trachynotidae Schrammen, 1924 in Porifera is permanently invalid because it was based on the junior homonym *Trachynotus* Schrammen, 1924; the fish name Trachynotinae Gill, 1861 (type genus *Trachynotus* Agassiz, 1846, an unjustified emendation of *Trachinotus* Lacepède, 1801 and also a junior homonym of *Trachynotus* Latreille, 1828) was later corrected to Trachinotinae, which is used as valid today; Trachynotoidae Förster, 1869 has also been used in ichneumonid literature but this name is permanently invalid since it was based on the junior homonym *Trachynotus* Gravenhorst, 1829.

### Tribe Stenosini

Schaum, 1859 (1834)

Tagénites Solier, 1834: 503 [stem: *Tageni-*]. Type genus: *Tagenia* Latreille, 1802 [syn. of *Stenosis* Herbst, 1799]. Comment: original vernacular name available (Art. 11.7.2): first used in latinized form by Hope (1840a: 127, as Tagenidae), generally accepted as in G. R. Waterhouse (1845: 30, as Tageniidae [incorrect stem formation]; use of younger name Stenosini conserved (Art. 40.2) (see Bouchard et al. 2005: 523); incorrect original stem formation, not in prevailing usage.
Stenosidae Schaum, 1859: 66 [stem: *Stenos-*]. Type genus: *Stenosis* Herbst, 1799. Comment: published before end of February 1859; this family-group name was also used in the same year by Lacordaire (1859 [before 27 June]: 101, as Sténosides); use of family-group name conserved over Tagenini Solier, 1834 (Art. 40.2) (see Bouchard et al. 2005: 523); current spelling maintained (Art. 29.5): incorrect stem formation in prevailing usage (should be *Stenose*-).
Platamodina Reitter, 1900: 82 [stem: *Platamod-*]. Type genus: *Platamodes* Ménétriés, 1849.
Typhlusechini Casey, 1907: 281 [stem: *Typhlusech-*]. Type genus: *Typhlusechus* Linell, 1897.
Araeoschizini Casey, 1907: 484 [stem: *Araeoschiz-*]. Type genus: *Araeoschizus* J. L. LeConte, 1851.
Dichillina Reitter, 1916: 137, in key [stem: *Dichill-*]. Type genus: *Dichillus* Jacquelin du Val, 1860.
Harvengina Ferrer, 2004: 370 [stem: *Harvengi-*]. Type genus: *Harvengia* Ferrer, 2004. Comment: incorrect original stem formation, not in prevailing usage.

### Tribe Tentyriini

Eschscholtz, 1831

Tentyridae Eschscholtz, 1831: 4 [stem: *Tentyri-*]. Type genus: *Tentyria* Latreille, 1802 [placed on the Official List of Generic Names in Zoology (ICZN 2010c)]. Comment: incorrect original stem formation, not in prevailing usage.
Gnathosiides Lacordaire, 1859: 33 [stem: *Gnathosi-*]. Type genus: *Gnathosia* Fischer von Waldheim, 1821. Comment: original vernacular name available (Art. 11.7.2): first used in latinized form by J. L. LeConte (1862: 213, as Gnathosiini), generally accepted as in Skopin (1979: 170, as Gnathosiina).
*Hypéropides Lacordaire, 1859: 60 [stem: *Hyperop-*]. Type genus: *Hyperops* Eschscholtz, 1831. Comment: original vernacular name unavailable (Art. 11.7.2): not subsequently latinized.
Capnisini Casey, 1907: 279 [stem: *Capnis-*]. Type genus: *Capnisa* Dejean, 1836 [syn. of *Gnathosia* Fisher von Waldheim, 1821].
Pachycerina Skopin, 1979: 170, in key [stem: *Pachycer-*]. Type genus: *Pachycera* Eschscholtz, 1831 [preoccupied genus name, not *Pachycera* Billberg, 1820 [Hemiptera]; syn. of *Oedenocera* Reiche, 1862]. Comment: permanently invalid (Art. 39): based on preoccupied type genus.
Himatismina Skopin, 1979: 170, in key [stem: *Himatism-*]. Type genus: *Himatismus* Erichson, 1843.

### Tribe Thinobatini

Lacordaire, 1859

Thinobatides Lacordaire, 1859: 63 [stem: *Thinobat-*]. Type genus: *Thinobatis* Eschscholtz, 1831. Comment: original vernacular name available (Art. 11.7.2): first used in latinized form by J. L. LeConte (1862: 214, as Thinobatini), generally accepted as in Bouchard et al. (2005: 502, as Thinobatini); current spelling maintained (Art. 29.3.1.1): incorrect stem formation in prevailing usage (should be *Thinobatid*-).

### Tribe Trilobocarini

Lacordaire, 1859

Tribolocarides Lacordaire, 1859: 69 [stem: *Trilobocar-*]. Type genus: *Trilobocara* Solier, 1851 [as *Tribolocara*, incorrect subsequent spelling of type genus name, not in prevailing usage]. Comment: original vernacular name available (Art. 11.7.2): first used in latinized form and generally accepted as in Kolbe (1887: 51, as Tribolocarini); incorrect stem formation, not in prevailing usage.
Salaxini Casey, 1907: 282 [stem: *Salac-*]. Type genus: *Salax* Guérin-Méneville, 1834. Comment: incorrect original stem formation, not in prevailing usage.

### Tribe Vacronini

Gebien, 1910

Vacroninae Gebien, 1910a: 118 [stem: *Vacron-*]. Type genus: *Vacronus* Casey, 1907 [syn. of *Alaephus* G. H. Horn, 1870].
*Eupsophulites Kwieton, 1982: 96 [stem: *Eupsophul-*]. Type genus: *Eupsophulus* Cockerell, 1906. Comment: unavailable family-group name, proposed after 1930 without description or bibliographic reference to such a description (Art. 13.1).

### Tribe Zophosini

Solier, 1834

Zophosites Solier, 1834: 597 [stem: *Zophos-*]. Type genus: *Zophosis* Latreille, 1802. Comment: original vernacular name available (Art. 11.7.2): first used in latinized form by Stein (1868: 78, as Zophosini), generally accepted as in Bouchard et al. (2005: 501, as Zophosini); current spelling maintained (Art. 29.5): incorrect stem formation in prevailing usage (should be *Zophose*-).
*Onychosites A. Deyrolle, 1867: 79 [stem: *Onychose-*]. Type genus: *Onychosis* Deyrolle, 1867. Comment: original vernacular name unavailable (Art. 11.7.2): not subsequently latinized; incorrect original stem formation, not in prevailing usage.
*Cardiosites A. Deyrolle, 1867: 79 [stem: *Cardiose-*]. Type genus: *Cardiosis* Deyrolle, 1867. Comment: original vernacular name unavailable (Art. 11.7.2): not subsequently latinized; incorrect original stem formation, not in prevailing usage.
Dactylocalcarini Gebien, 1938a: 46 [stem: *Dactylocalcar-*]. Type genus: *Dactylocalcar* Gebien, 1938.

### Subfamily Tenebrioninae

Latreille, 1802

Tenebrionites Latreille, 1802: 165 [stem: *Tenebrion-*]. Type genus: *Tenebrio* Linnaeus, 1758. Comment: First Reviser (Tenebrioninae Latreille, 1802 vs Helopinae Latreille, 1802) not determined, current usage maintained

### Tribe Acropteronini

Doyen, 1989

Acropteronini Doyen, 1989: 288 [stem: *Acropteron-*]. Type genus: *Acropteron* Perty, 1832. Comment: current spelling maintained (Art. 29.5): incorrect stem formation in prevailing usage (should be *Acropter-*).

### Tribe Alphitobiini

Reitter, 1917

Alphitobiini Reitter, 1917: 58 [stem: *Alphitobi-*]. Type genus: *Alphitobius* Stephens, 1829 [placed on the Official List of Generic Names in Zoology (ICZN 1975)].

### Tribe Amarygmini

Gistel, 1848

Amarygmiidae Gistel, 1848: [10] [stem: *Amarygm-*]. Type genus: *Amarygmus* Dalman, 1823. Comment: incorrect original stem formation, not in prevailing usage.
Mégacanthides Lacordaire, 1859: 467 [stem: *Megacanth-*]. Type genus: *Megacantha* Westwood, 1843. Comment: original vernacular name available (Art. 11.7.2): first used in latinized form and generally accepted as in Quedenfeldt (1885: 20, as Megacanthidae).
Méracanthides Lacordaire, 1859: 464 [stem: *Meracanth-*]. Type genus: *Meracantha* Kirby, 1837. Comment: original vernacular name available (Art. 11.7.2): first used in latinized form by J. L. LeConte (1862: 240, as Meracanthini), generally accepted as in Gebien (1911: 567, as Meracanthinae).
Megacanthina Ardoin, 1962: 960 [stem: *Megacanth-*]. Type genus: *Megacantha* Westwood, 1843. Comment: family-group name proposed as new without reference to Mégacanthides Lacordaire, 1859.

### Tribe Amphidorini

LeConte, 1862

*Nycterinoides Solier, 1851: 210 [stem: *Nycterin-*]. Type genus: *Nycterinus* Eschscholtz, 1829. Comment: original vernacular name unavailable (Art. 11.7.2): subsequently used in latinized form but not generally attributed to Solier (1851).
*Embaphionides Lacordaire, 1859: 151 [stem: *Embaphi-*]. Type genus: *Embaphion* Say, 1824. Comment: original vernacular name unavailable (Art. 11.7.2): subsequently used in latinized form, e.g., D. B. Thomas (2005: 549, as Embaphionini), but not generally attributed to Lacordaire (1859); Embaphionini, as used by D. B. Thomas (2005) is unavailable because it was proposed after 1930 without description or bibliographic reference to such a description (Art. 13.1); incorrect original stem formation, not in prevailing usage.
Amphidorae J. L. LeConte, 1862: 239 [stem: *Amphidor-*]. Type genus: *Amphidora* Eschscholtz, 1829.
Eleodiini Blaisdell, 1909: 27 [stem: *Eleod-*]. Type genus: *Eleodes* Eschscholtz, 1829. Comment: incorrect original stem formation, not in prevailing usage.
Eleodopsinae Blaisdell, 1939: 51 [stem: *Eleodopse-*]. Type genus: *Eleodopsis* Blaisdell, 1939 [syn. of *Eleodes* Eschscholtz, 1829]. Comment: incorrect original stem formation, not in prevailing usage.
Lariversiina La Rivers, 1948: 98 [stem: *Lariversi-*]. Type genus: *Lariversius* Blaisdell, 1947.
Trogloderina La Rivers, 1948: 98 [stem: *Trogloder-*]. Type genus: *Trogloderus* J. L. LeConte, 1879.
*Nycterini Doyen et al., 1990: 244 [stem: *Nycterin-*]. Type genus: *Nycterinus* Eschscholtz, 1829. Comment: unavailable family-group name, proposed after 1930 without description or bibliographic reference to such a description (Art. 13.1); incorrect original stem formation, not in prevailing usage.

### Tribe Apocryphini

Lacordaire, 1859

Apocryphides Lacordaire, 1859: 432 [stem: *Apocryph-*]. Type genus: *Apocrypha* Eschscholtz, 1831. Comment: original vernacular name available (Art. 11.7.2): first used in latinized form by J. L. LeConte (1862: 217, as Apocryphini), generally accepted as in Gebien (1911: 503, as Apocryphinae).
Diplocyrthini Escalera, 1914b: 355 [stem: *Diplocyrt-*]. Type genus: *Diplocyrtus* Quendenfeldt, 1887 [as *Diplocyrthus*, incorrect subsequent spelling of type genus name, not in prevailing usage]. Comment: incorrect original stem formation, not in prevailing usage.

### Tribe Blaptini

Leach, 1815

Blapsida Leach, 1815: 101 [stem: *Blapt-*]. Type genus: *Blaps* Fabricius, 1775.

### Subtribe Blaptina

Leach, 1815

Blapsida Leach, 1815: 101 [stem: *Blapt-*]. Type genus: *Blaps* Fabricius, 1775. Comment: although the original stem formation (*Blaps*-) was correct, the stem *Blapt*- has been used since it was first used by C. G. Thomson (1859: 114, as Blaptidae) and is also used here; an application to the Commission is needed to conserve the current spelling.

### Subtribe Gnaptorina

Medvedev, 2001

Gnaptorina G. S. Medvedev, 2001: 29 [stem: *Gnaptor-*]. Type genus: *Gnaptor* Brullé, 1832.

### Subtribe Gnaptorinina

Medvedev, 2001

Gnaptorinina G. S. Medvedev, 2001: 31 [stem: *Gnaptorin-*]. Type genus: *Gnaptorina* Reitter, 1887.

### Subtribe Prosodina

Skopin, 1960

Prosodina Skopin, 1960: 48 [stem: *Prosod-*]. Type genus: *Prosodes* Eschscholtz, 1829.

### Subtribe Remipedellina

Semenov, 1907

Remipedellini Semenov, 1907a: 259 [stem: *Remipedell-*]. Type genus: *Remipedella* Semenov, 1907. Comment: this family-group name was also used in the same year by Semenov (1907b: 176, as Remipedellini); priority for the two publications could not be established although Remipedellini Semenov, 1907b could not be considered available since the type genus *Remipedella* was made available only in Semenov (1907a).

### Tribe Bolitophagini

Kirby, 1837 nomen protectum

Eledonaedes Billberg, 1820b: 392 [stem: *Eledon-*]. Type genus: *Eledona* Latreille, 1797. Comment: *nomen oblitum* (see Appendix 1); the name Eledoninae (type genus *Eledone* Leach, 1817) is used as valid in cephalopods (earliest usage in cephalopods found is Eledonidae Rochebrune, 1884).
Bolitophagidae Kirby, 1837: 236 [stem: *Bolitophag-*]. Type genus: *Bolitophagus* Illiger, 1798. Comment: *nomen protectum* (see Appendix 1).
Rhipidandri J. L. LeConte, 1862: 236 [stem: *Rhipidandr-*]. Type genus: *Rhipidandrus* J. L. LeConte, 1862.
Eutomides Lacordaire, 1865: 369 [stem: *Eutom-*]. Type genus: *Eutomus* Lacordaire, 1865 [syn. of *Rhipidandrus* J. L. LeConte, 1862]. Comment: original vernacular name available (Art. 11.7.2): first used in latinized form and generally accepted as in Ferrari (1867: 3, as Eutomides [treated as Latin]); name treated as unavailable by Bouchard et al. (2005: 508) but recently considered available by Alonso-Zarazaga and Lyal (2009: 7); taxon originally described in Curculionidae: Scolytinae.

### Tribe Centronopini

Doyen, 1989

Centronopini Doyen, 1989: 284 [stem: *Centronop-*]. Type genus: *Centronopus* Solier, 1848. Comment: current spelling maintained (Art. 29.5): incorrect stem formation in prevailing usage (should be *Centronopod*-).

### Tribe Cerenopini

Horn, 1870

Cerenopi G. H. Horn, 1870: 325 [stem: *Cerenop-*]. Type genus: *Cerenopus* J. L. LeConte, 1851. Comment: current spelling maintained (Art. 29.5): incorrect stem formation in prevailing usage (should be *Cerenopod*-).

### Tribe Dissonomini

Medvedev, 1968

*Hétérophilates Mulsant and Rey, 1859: 6 [stem: *Heterophyl-*]. Type genus: *Heterophylus* Mulsant and Rey, 1859 [preoccupied genus name, not *Heterophylus* Klug, 1833 [Coleoptera: Tenebrionidae: Diaperinae]; syn. of *Dissonomus* Jacquelin du Val, 1861]. Comment: original vernacular name unavailable (Art. 11.7.2): not subsequently latinized; if found to be available then permanently invalid (Art. 39): based on preoccupied type genus; incorrect original stem formation, not in prevailing usage.
*Dissonomites Jacquelin du Val, 1861: 280 [stem: *Dissonom-*]. Type genus: *Dissonomus* Jacquelin du Val, 1861. Comment: original vernacular name unavailable (Art. 11.7.2): subsequently used in latinized form but not generally attributed to Jacquelin du Val (1861).
Dissonomini G. S. Medvedev, 1968: 211 [stem: *Dissonom-*]. Type genus: *Dissonomus* Jacquelin du Val, 1861.

### Tribe Eulabini

Horn, 1870

Eulabes G. H. Horn, 1870: 323 [stem: *Eulab-*]. Type genus: *Eulabis* Eschscholtz, 1829.

### Tribe Falsocossyphini

Ferrer, 2006

Falsocossyphini Ferrer, 2006: 77 [stem: *Falsocossyph-*]. Type genus: *Falsocossyphus* Pic, 1916.

### Tribe Heleini

Fleming, 1821

Heleadae Fleming, 1821: 51 [stem: *Hele-*]. Type genus: *Helea* Latreille, 1816.

### Subtribe Asphalina

Matthews and Lawrence, 2005

Asphalina E. G. Matthews and Lawrence, 2005: 544 [stem: *Asphal-*]. Type genus: *Asphalus* Pascoe, 1868.

### Subtribe Cyphaleina

Lacordaire, 1859

Cyphaléides Lacordaire, 1859: 407 [stem: *Cyphale-*]. Type genus: *Cyphaleus* Westwood, 1841. Comment: original vernacular name available (Art. 11.7.2): first used in latinized form by Pascoe (1866b: 470, as Cyphaleinae), generally accepted as in Bouchard et al. (2005: 502, as Cyphaleina); precedence (Cyphaleini Lacordaire, 1859 vs Nyctozoilini Lacordaire, 1859) given to taxon originally proposed at the higher rank (Art. 24.1).
Nyctozoïlides Lacordaire, 1859: 349 [stem: *Nyctozoil-*]. Type genus: *Nyctozoilus* Guérin-Méneville, 1830. Comment: original vernacular name available (Art. 11.7.2): first used in latinized form by Bates (1872b: 98, as Nyctozoilides [treated as Latin]), generally accepted as in Carter (1911: 138, as Nyctozoilides [treated as Latin]).

### Subtribe Heleina

Fleming, 1821

Heleadae Fleming, 1821: 51 [stem: *Hele-*]. Type genus: *Helea* Latreille, 1804. Comment: incorrect original stem formation, not in prevailing usage.
Briseinae Carter, 1924: 33 [stem: *Bris-*]. Type genus: *Brises* Pascoe, 1869. Comment: incorrect original stem formation, not in prevailing usage.

### Tribe Helopini

Latreille, 1802

Helopii Latreille, 1802: 176 [stem: *Helop-*]. Type genus: *Helops* Fabricius, 1775 [placed on the Official List of Generic Names in Zoology (ICZN 2009d)].

### Subtribe Helopina

Latreille, 1802

Helopii Latreille, 1802: 176 [stem: *Helop-*]. Type genus: *Helops* Fabricius, 1775 [placed on the Official List of Generic Names in Zoology (ICZN 2009d)].
Hypulia Rafinesque, 1815: 114 [stem: *Hypul-*]. Type genus: *Hypulus* Rafinesque, 1815 [syn. of *Helops* Fabricius, 1775].
*Enoplopites Solier, 1848: 155 [stem: *Enoplopod-*]. Type genus: *Enoplopus* Solier, 1848. Comment: original vernacular name unavailable (Art. 11.7.2): subsequently used in latinized form but not generally attributed to Solier (1848); incorrect original stem formation, not in prevailing usage.
*Hédyphanes Motschulsky, 1849: 57 [stem: *Hedyphan-*]. Type genus: *Hedyphanes* Fischer von Waldheim, 1820. Comment: original vernacular name unavailable (Art. 11.7.2): subsequently used in latinized form but not generally attributed to Motschulsky (1849).
Enoplopini Reitter, 1917: 62 [stem: *Enoplopod-*]. Type genus: *Enoplopus* Solier, 1848. Comment: incorrect original stem formation, not in prevailing usage.
Nephodini Reitter, 1917: 63 [stem: *Nephod-*]. Type genus: *Nephodes* Blanchard, 1845.
Hedyphanina Reitter, 1922b: 6 [stem: *Hedyphan-*]. Type genus: *Hedyphanes* Fischer von Waldheim, 1820.
*Stenotrichini Blaisdell, 1939: 50 [stem: *Stenotrich-*]. Type genus: *Stenotrichus* J. L. LeConte, 1862 [syn. of *Helops* Fabricius, 1775]. Comment: unavailable family-group name, proposed after 1930 without description or bibliographic reference to such a description (Art. 13.1).

### Subtribe Cylindrinotina

Español, 1956

*Xanthomini Antoine, 1949: 162 [stem: *Xanthom-*]. Type genus: *Xanthomus* Mulsant, 1854. Comment: unavailable family-group name, proposed after 1930 without description or bibliographic reference to such a description (Art. 13.1).
*Ectromopsini Antoine, 1949: 162 [stem: *Ectromopse-*]. Type genus: *Ectromopsis* Antoine, 1949. Comment: unavailable family-group name, proposed after 1930 without description or bibliographic reference to such a description (Art. 13.1); incorrect original stem formation, not in prevailing usage.
Cylindronotini Español, 1956: 84 [stem: *Cylindrinot-*]. Type genus: *Cylindrinotus* Faldermann, 1837 [as *Cylindronotus*, unjustified emendation of type genus name by Agassiz (1846b: 111), not in prevailing usage]. Comment: incorrect original stem formation, not in prevailing usage; correction of stem by Bouchard et al. (2005: 509).

### Tribe Helopinini

Lacordaire, 1859

Hélopinides Lacordaire, 1859: 457 [stem: *Helopin-*]. Type genus: *Helopinus* Solier, 1848.

### Subtribe Aptilina

Koch, 1958

Aptilina Koch, 1958: 139 [stem: *Aptil-*]. Type genus: *Aptila* Fåhraeus, 1870.

### Subtribe Helopinina

Lacordaire, 1859

Hélopinides Lacordaire, 1859: 457 [stem: *Helopin-*]. Type genus: *Helopinus* Solier, 1848. Comment: original vernacular name available (Art. 11.7.2): first used in latinized form by Bertkau (1875: 356, as Helopinini), generally accepted as in Gebien (1911: 563, as Helopininae).
Drosochrini Koch, 1958: 133 [stem: *Drosochr-*]. Type genus: *Drosochrus* Erichson, 1843. Comment: unnecessary replacement name for Helopinini Lacordaire, 1859.

### Subtribe Micrantereina

Reitter, 1917

Micrantereini Reitter, 1917: 60 [stem: *Micrantere-*]. Type genus: *Micrantereus* Solier, 1848.

### Subtribe Oncosomina

Koch, 1958

Oncosomina Koch, 1958: 134 [stem: *Oncosom-*]. Type genus: *Oncosoma* Westwood, 1843 [unjustified emendation of *Ogcosoma* by Agassiz (1846b: 257), in prevailing usage, treated as justified emendation (Art. 33.2.3.1)]. Comment: current spelling maintained (Art. 29.5): incorrect original stem formation in prevailing usage (should be *Oncosomat*-).

### Tribe Melanimonini

Seidlitz, 1894 (1854)

Microzoumates Mulsant, 1854: 176 [stem: *Microzo-*]. Type genus: *Microzoum* Dejean, 1834 [syn. of *Melanimon* Steven 1829]. Comment: original vernacular name available (Art. 11.7.2): first used in latinized form by Seidlitz (1889 [Gatt.]: 128, as Microzoina), generally accepted as in Bouchard et al. (2005: 523, as Microzoini); use of Melanimonini Seidlitz, 1894 conserved (Art. 40.2) (see Bouchard et al. 2005: 523); incorrect original stem formation, not in prevailing usage.
Melanimonina Seidlitz, 1894: 449 [stem: *Melanimon-*]. Type genus: *Melanimon* Steven, 1829. Comment: use of younger family-group name conserved (Art. 40.2) (see Bouchard et al. 2005).

### Tribe Opatrini

Brullé, 1832

Opatrites Brullé, 1832: 213 [stem: *Opatr-*]. Type genus: *Opatrum* Fabricius, 1775.

### Subtribe Heterocheirina

Koch, 1956

Heterocheirini Koch, 1956: 43 [stem: *Heterocheir-*]. Type genus: *Heterocheira* Lacordaire, 1859.

### Subtribe Heterotarsina

Blanchard, 1845

Hétérotarsites Blanchard, 1845b: 14 [stem: *Heterotars-*]. Type genus: *Heterotarsus* Latreille, 1829. Comment: original vernacular name available (Art. 11.7.2): first used in latinized form by J. L. LeConte (1862: 231, as Heterotarsini), generally accepted as in Bouchard et al. (2005: 502, as Heterotarsina).

### Subtribe Opatrina

Brullé, 1832

Opatrites Brullé, 1832: 213 [stem: *Opatr-*]. Type genus: *Opatrum* Fabricius, 1775. Comment: original vernacular name available (Art. 11.7.2): first used in latinized form by Shuckard (1839b: 49, as Opatridae), generally accepted as in Bouchard et al. (2005: 502, as Opatrini).
*Gonocéphalites Solier, 1834: 498 [stem: *Gonocephal-*]. Type genus: *Gonocephalum* Solier, 1834. Comment: original vernacular name unavailable (Art. 11.7.2): subsequently used in latinized form but not generally attributed to Solier (1834).
*Blapstinoides Solier, 1851: 231 [stem: *Blapstin-*]. Type genus: *Blapstinus* Dejean, 1821. Comment: original vernacular name unavailable (Art. 11.7.2): subsequently used in latinized form but not generally attributed to Solier (1851).
Blapstinites Mulsant and Rey, 1853: 258 [stem: *Blapstin-*]. Type genus: *Blapstinus* Dejean, 1821. Comment: original vernacular name available (Art. 11.7.2): first used in latinized form and generally accepted as in J. L. LeConte (1862: 227, as Blapstini).
Stizopides Lacordaire, 1859: 258 [stem: *Stizopod-*]. Type genus: *Stizopus* Erichson, 1843. Comment: original vernacular name available (Art. 11.7.2): first used in latinized form by Heyne and Taschenberg (1907: 207, as Stizopini), generally accepted as in Gebien (1938b: 72 [393], as Stizopini); incorrect original stem formation, not in prevailing usage.
Sclérides Lacordaire, 1859: 263 [stem: *Scler-*]. Type genus: *Scleron* Hope, 1840 [syn. of *Sclerum* Dejean, 1834]. Comment: original vernacular name available (Art. 11.7.2): first used in latinized form by Seidlitz (1890 [Gatt.]: 129, as Sclerina), generally accepted as in Seidlitz (1894: 415, as Sclerina).
*Penthicaires Mulsant and Rey, 1859: 5 [stem: *Penthic-*]. Type genus: *Penthicus* Faldermann, 1836. Comment: original vernacular name unavailable (Art. 11.7.2): subsequently used in latinized form, e.g., Heyne and Taschenberg (1907: 208, as Penthicini, under the valid tribe name Opatrini), but not generally accepted as valid.
*Caediaires Mulsant and Rey, 1859: 124 [stem: *Caedi-*]. Type genus: *Caedius* Blanchard, 1845. Comment: original vernacular name unavailable (Art. 11.7.2): subsequently used in latinized form, e.g., Heyne and Taschenberg (1907: 208, as Caediini, under the valid tribe name Opatrini), but not generally accepted as valid.
*Blacodaires Mulsant and Rey, 1859: 93 [stem: *Blacod-*]. Type genus: *Blacodes* Blanchard, 1845 [syn. of *Blenosia* Laporte, 1840]. Comment: original vernacular name unavailable (Art. 11.7.2): subsequently used in latinized form, e.g., Heyne and Taschenberg (1907: 208, as Blacodini, under the valid tribe name Opatrini), but not generally accepted as valid.
*Clitobiates Mulsant and Rey, 1859: 141 [stem: *Clitobi-*]. Type genus: *Clitobius* Mulsant and Rey, 1859. Comment: original vernacular name unavailable (Art. 11.7.2): subsequently used in latinized form, e.g., Heyne and Taschenberg (1907: 208, as Clitobiini, under the valid tribe name Opatrini), but not generally accepted as valid.
Gonocéphalates Mulsant and Revelière, 1861: 154 [stem: *Gonocephal-*]. Type genus: *Gonocephalum* Solier, 1834. Comment: original vernacular name available (Art. 11.7.2): first used in latinized form and generally accepted as in Gerstaecker (1861: 483, as Gonocephalidae).
*Dilamites Jacquelin du Val, 1861: 279 [stem: *Dilam-*]. Type genus: *Dilamus* Jacquelin du Val, 1861. Comment: original vernacular name unavailable (Art. 11.7.2): not subsequently latinized.
Ammobiini Desbrochers des Loges, 1902b: 1 [stem: *Ammobi-*]. Type genus: *Ammobius* Guérin-Méneville, 1844.
Emmallina Koch, 1956: 51 [stem: *Emmal-*]. Type genus: *Emmalus* Erichson, 1843 [as *Emmallus*, unjustified emendation of type genus name, not in prevailing usage]. Comment: incorrect original stem formation, not in prevailing usage.
Stenolamina Koch, 1956: 49 [stem: *Stenolam-*]. Type genus: *Stenolamus* Gebien, 1920.

### Subtribe Neopachypterina

Bouchard, Löbl and Merkl, 2007

Pachyptérates Mulsant and Rey, 1859: 83 [stem: *Pachypter-*]. Type genus: *Pachypterus* Lucas, 1846 [preoccupied genus name, not *Pachypterus* Swainson, 1839 [Pisces]; syn. of *Neopachypterus* Bouchard et al., 2007]. Comment: original vernacular name available (Art. 11.7.2): first used in latinized form and generally accepted as in Desbrochers des Loges (1901: 146, as Pachypterini [actually spelled Pachnephorini but this name was corrected to Pachypterini later by Desbroches des Loges (1902a: 85)]; permanently invalid (Art. 39): based on preoccupied type genus.
Pachypterini G. S. Medvedev, 1968: 247 [stem: *Pachypter-*]. Type genus: *Pachypterus* Lucas, 1846 [preoccupied genus name, not *Pachypterus* Swainson, 1839 [Pisces]; syn. of *Neopachypterus* Bouchard et al., 2007]. Comment: family-group name proposed as new without reference to Pachyptérates Mulsant and Rey, 1859; permanently invalid (Art. 39): based on preoccupied type genus.
Neopachypterina Bouchard et al., 2007: 386 [stem: *Neopachypter-*]. Type genus: *Neopachypterus* Bouchard et al., 2007. Comment: replacement name for Pachyptérates Mulsant and Rey, 1859 and Pachypterini G. S. Medvedev, 1968 because of the homonymy of the type genus.

### Tribe Palorini

Matthews, 2003

*Palorinae E. G. Matthews, 2003a: 50 [stem: *Palor-*]. Type genus: *Palorus* Mulsant, 1854. Comment: unavailable name (Art. 16): not explicitly indicated as new.
Palorinae E. G. Matthews, 2003b: 7 [stem: *Palor-*]. Type genus: *Palorus* Mulsant, 1854. Comment: validation of Palorinae Matthews (2003a).

### Tribe Pedinini

Eschscholtz, 1829

Pediniden Eschscholtz, 1829b: 4 [stem: *Pedin-*]. Type genus: *Pedinus* Latreille, 1797.

### Subtribe Dendarina

Mulsant and Rey, 1854

Pandarites Mulsant and Rey, 1854: 153 [stem: *Pandar-*]. Type genus: *Dendarus* Dejean, 1821 [as *Pandarus*, unjustified emendation of type genus name, not in prevailing usage]. Comment: original vernacular name available (Art. 11.7.2): first used in latinized form by Gerstaecker (1854: 226, as Pandaridae [incorrect stem formation]), and generally accepted as in Seidlitz (1889[Gatt.]: 126, as Dendarina).
*Omocratates Mulsant and Rey, 1854: 266, in key [stem: *Omocrat-*]. Type genus: *Omocrates* Mulsant, 1854 [preoccupied genus name, not *Omocrates* H. C. C. Burmeister, 1844 [Coleoptera: Scarabaeidae]; syn. of *Phylan* Dejean, 1821]. Comment: original vernacular name unavailable (Art. 11.7.2): not subsequently latinized; if found to be available then permanently invalid (Art. 39): based on preoccupied type genus.
*Micrositates Mulsant and Rey, 1854: 274 [stem: *Microsit-*]. Type genus: *Micrositus* Mulsant and Rey, 1854. Comment: original vernacular name unavailable (Art. 11.7.2): not subsequently latinized.
*Isocérates Mulsant and Rey, 1854: 188 [stem: *Isocer-*]. Type genus: *Isocerus* Dejean, 1821 [preoccupied genus name, not *Isocerus* Illiger, 1802 [Coleoptera: Cerambycidae]; syn. of *Neoisocerus* Bouchard et al., 2005]. Comment: original vernacular name unavailable (Art. 11.7.2): not subsequently latinized; if found to be available then permanently invalid (Art. 39): based on preoccupied type genus.
*Héliopathaires Mulsant and Rey, 1854: 265 [stem: *Heliopat-*]. Type genus: *Heliopates* Dejean, 1834 [as *Heliopathes*, incorrect subsequent spelling of type genus name, not in prevailing usage]. Comment: original vernacular name unavailable (Art. 11.7.2): not subsequently latinized; incorrect original stem formation, not in prevailing usage.
Phylacides Lacordaire, 1859: 270 [stem: *Phylac-*]. Type genus: *Phylax* Brullé, 1832 [syn. of *Dendarus* Dejean, 1821]. Comment: original vernacular name available (Art. 11.7.2): first used in latinized form and generally accepted as in Seidlitz (1893: 220, as Phylacina).
Bioplanina A. N. Reichardt, 1936: 24 [stem: *Bioplanet-*]. Type genus: *Bioplanes* Mulsant, 1854. Comment: incorrect original stem formation, not in prevailing usage.

### Subtribe Eurynotina

Mulsant and Rey, 1854

Eurynotaires Mulsant and Rey, 1854: 156 [stem: *Eurynot-*]. Type genus: *Eurynotus* Kirby, 1819. Comment: original vernacular name available (Art. 11.7.2): first used in latinized form by Koch (1956: 25, as Eurynotina), generally accepted as in Bouchard et al. (2005: 502, as Eurynotina).
Psectropini Kaszab, 1941: 33 [stem: *Psectropod-*]. Type genus: *Psectropus* sensu Kaszab, 1941 [not *Psectropus* Solier, 1848; syn. of *Schyzochelus* Koch, 1954]. Comment: based on misidentified type genus; incorrect original stem formation, not in prevailing usage.
Oncotini Koch, 1953c: 267, in key [stem: *Oncot-*]. Type genus: *Oncotus* Blanchard, 1845.
Schyzoschelina Koch, 1956: 25 [stem: *Schyzoschel-*]. Type genus: *Schyzoschelus* Koch, 1954.

### Subtribe Leichenina

Mulsant, 1854

Leichenaires Mulsant, 1854: 179 [stem: *Leichen-*]. Type genus: *Leichenum* Blanchard, 1845. Comment: original vernacular name available (Art. 11.7.2): first used in latinized form by Casey (1890: 391, as Leichenini), generally accepted as in Bouchard et al. (2005: 502, as Leichenina).

### Subtribe Loensina

Koch, 1956

*Loensini Koch, 1955: 1 [stem: *Loens-*]. Type genus: *Loensus* Lucas, 1920. Comment: unavailable family-group name, proposed after 1930 without description or bibliographic reference to such a description (Art. 13.1).
Loensini Koch, 1956: 402 [stem: *Loens-*]. Type genus: *Loensus* Lucas, 1920.

### Subtribe Melambiina

Mulsant and Rey, 1854

Melambiates Mulsant and Rey, 1854: 267 [stem: *Melambi-*]. Type genus: *Melambius* Mulsant and Rey, 1854. Comment: original vernacular name available (Art. 11.7.2): first used in latinized form by Español (1945: 226, as Melambina [incorrect stem formation]), generally accepted as in Bouchard et al. (2005: 502, as Melambiina).
Litoborinae Antoine, 1941: 19-21 [stem: *Litobor-*]. Type genus: *Litoborus* Mulsant and Rey, 1854.
Zadenina Koch, 1956: 279, in key [stem: *Zaden-*]. Type genus: *Zadenos* Laporte, 1840.

### Subtribe Pedinina

Eschscholtz, 1829

Pediniden Eschscholtz, 1829b: 4 [stem: *Pedin-*]. Type genus: *Pedinus* Latreille, 1797. Comment: original vernacular name available (Art. 11.7.2): first used in latinized form by G. R. Waterhouse (1845: 32, as Pedinidae), generally accepted as in Bouchard et al. (2005: 502, as Pedinini).

### Subtribe Platynotina

Mulsant and Rey, 1853

*Hétéroscélites Solier, 1836: 502 [stem: *Heteroscelid-*]. Type genus: *Heteroscelis* Latreille, 1829 [preoccupied genus name, not *Heteroscelis* Latreille, 1829 [Hemiptera]; syn. of *Anomalipus* Guérin-Méneville, 1831]. Comment: original vernacular name unavailable (Art. 11.7.2): not subsequently latinized; if found to be available then permanently invalid (Art. 39): based on preoccupied type genus; incorrect original stem formation, not in prevailing usage.
Platynotaires Mulsant and Rey, 1853: 263 [stem: *Platynot-*]. Type genus: *Platynotus* Fabricius, 1801. Comment: original vernacular name available (Art. 11.7.2): first used in latinized form by J. L. LeConte (1862: 226, as Platynotini), generally accepted as in Bouchard et al. (2005: 502, as Platynotina); First Reviser (Platynotina Mulsant and Rey, 1853 vs Trigonopodina Mulsant and Rey, 1853) not determined, current usage maintained.
Trigonopaires Mulsant and Rey, 1853: 104 [stem: *Trigonopod-*]. Type genus: *Trigonopus* Mulsant and Rey, 1853. Comment: original vernacular name available (Art. 11.7.2): first used in latinized form and generally accepted as in Aalbu (2006: 70, as Trigonopina); incorrect original stem formation, not in prevailing usage.
Gonopides Lacordaire, 1859: 255 [stem: *Gonopod-*]. Type genus: *Gonopus* Latreille, 1829. Comment: original vernacular name available (Art. 11.7.2): first used in latinized form by Heyne and Taschenberg (1907: 207, as Gonopini), generally accepted as in Gebien (1938a: 90, as Gonopini); incorrect original stem formation, not in prevailing usage.
Platynotini Koch, 1953c: 268, in key [stem: *Platynot-*]. Type genus: *Platynotus* Fabricius, 1801. Comment: family-group name proposed as new without reference to Platynotaires Mulsant and Rey, 1853.
Anomalipina Koch, 1954: 427 [stem: *Anomalipod-*]. Type genus: *Anomalipus* Guérin-Méneville, 1831 [unjustified emendation of *Anomalipes* Guérin-Méneville, 1831 by Lacordaire, 1859; unjustified emendation in prevailing usage, treated as justified emendation (Art. 33.2.3.1)]. Comment: incorrect original stem formation, not in prevailing usage.
*anchophthalmoid Koch, 1956: 71, in key [stem: *Anchophthalm-*]. Type genus: *Anchophthalmus* Gerstaecker, 1854. Comment: family-group name unavailable (Art. 11.7.1.1): original name not proposed as a noun.
*selinoid Koch, 1956: 70, in key [stem: *Selin-*]. Type genus: *Selinus* Mulsant and Rey, 1853. Comment: family-group name unavailable (Art. 11.7.1.1): original name not proposed as a noun; Selinini Jeannel, 1948 (type genus *Selina* Motschulsky, 1858) is currently used as a valid in Carabidae.
*melanocratoid Iwan, 1996: 379, 380-381 [stem: *Melanocrat-*]. Type genus: *Melanocratus* Fairmaire, 1895. Comment: family-group name unavailable (Art. 11.7.1.1): original name not proposed as a noun.

### Subtribe Pythiopina

Koch, 1953

Pythiopini Koch, 1953b: 245 [stem: *Pythiop-*]. Type genus: *Pythiopus* Koch, 1953. Comment: current spelling maintained (Art. 29.5): incorrect stem formation in prevailing usage (should be *Pythiopod*-).

### Tribe Platyscelidini

Lacordaire, 1859

Platyscélides Lacordaire, 1859: 229 [stem: *Platyscelid-*]. Type genus: *Platyscelis* Latreille, 1818 [placed on the Official List of Generic Names in Zoology (ICZN 1993e)]. Comment: original vernacular name available (Art. 11.7.2): first used in latinized form by Seidlitz (1889 [Gatt.]: 126, as Platyscelina [incorrect stem formation]), generally accepted as in Bouchard et al. (2005: 502, as Platyscelidini); incorrect original stem formation, not in prevailing usage; correction of stem by Egorov (1990: 401) in order to avoid homonymy with Platyscelidae Bate, 1862 in Crustacea.

### Tribe Praeugenini

De Moor, 1970

*Praogenini Ferreira, 1965: 312 [stem: *Praeugen-*]. Type genus: *Praeugena* Laporte, 1840 [as *Praogena*, unjustified emendation of type genus name, not in prevailing usage]. Comment: unavailable family-group name, proposed after 1930 without description or bibliographic reference to such a description (Art. 13.1); incorrect original stem formation, not in prevailing usage.
Praeugenina De Moor, 1970: 4 [stem: *Praeugen-*]. Type genus: *Praeugena* Laporte, 1840.

### Tribe Rhysopaussini

Wasmann, 1896

Rhysopaussidae Wasmann, 1896: 613 [stem: *Rhysopauss-*]. Type genus: *Rhysopaussus* Wasmann, 1896.

### Tribe Scaurini

Billberg, 1820

Scaurides Billberg, 1820a: 31 [stem: *Scaur-*]. Type genus: *Scaurus* Fabricius, 1775. Comment: this family-group name was also used in the same year by Billberg (1820b: 392, as Scaurides).

### Tribe Scotobiini

Solier, 1838

Scotobites Solier, 1838: 39 [stem: *Scotobi-*]. Type genus: *Scotobius* Germar, 1824. Comment: original vernacular name available (Art. 11.7.2): first used in latinized form by Agassiz (1846b: 336, as Scotobioidae), generally accepted as in Bouchard et al. (2005: 502, as Scotobiini); incorrect original stem formation, not in prevailing usage.

### Tribe Tenebrionini

Latreille, 1802

Tenebrionites Latreille, 1802: 165 [stem: *Tenebrion-*]. Type genus: *Tenebrio* Linnaeus, 1758.
Biuini Skopin, 1978: 224, in key [stem: *Bi-*]. Type genus: *Bius* Dejean, 1834. Comment: incorrect original stem formation, not in prevailing usage.

### Tribe Titaenini

Fauvel, 1905

Titaenini Fauvel, 1905b: 209 [stem: *Titaen-*]. Type genus: *Titaena* Erichson, 1842.

### Tribe Toxicini

Oken, 1843

Toxiciden Oken, 1843: 484 [stem: *Toxic-*]. Type genus: *Toxicum* Latreille, 1802.

### Subtribe Eudysantina

Bouchard, Lawrence, Davies and Newton, 2005

Dysantinae Gebien, 1922: 289 [stem: *Dysant-*]. Type genus: *Dysantes* Pascoe, 1871 [preoccupied genus name, not *Dysantes* Foerster, 1868 [Hymenoptera]; syn. of *Eudysantes* Bouchard et al., 2005]. Comment: permanently invalid (Art. 39): based on preoccupied type genus.
Eudysantina Bouchard et al., 2005: 508 [stem: *Eudysant-*]. Type genus: *Eudysantes* Bouchard et al., 2005. Comment: replacement name for Dysantina Gebien, 1922 because of the homonymy of the type genus.

### Subtribe Nycteropina

Lacordaire, 1859

Nyctéropides Lacordaire, 1859: 388 [stem: *Nycterop-*]. Type genus: *Nycteropus* Klug, 1833. Comment: original vernacular name available (Art. 11.7.2): first used in latinized form by Heyne and Taschenberg (1907: 211, as Nycteropini), generally accepted as in Bouchard et al. (2005: 502, as Nycteropina).

### Subtribe Toxicina

Oken, 1843

Toxiciden Oken, 1843: 484 [stem: *Toxic-*]. Type genus: *Toxicum* Latreille, 1802. Comment: original vernacular name available (Art. 11.7.2): first used in latinized form and generally accepted as in Tulk (1847: 614, as Toxicidae); family-group name previously attributed to Lacordaire (1859).
Anthraciini Houlbert, 1922b: 185, in key [stem: *Anthraci-*]. Type genus: *Anthracias* Dejean, 1834 [syn. of *Cryphaeus* Klug, 1833].

### Tribe Triboliini

Gistel, 1848

Triboliidae Gistel, 1848: [4] [stem: *Triboli-*]. Type genus: *Tribolium* W. S. MacLeay, 1825 [placed on the Official List of Generic Names in Zoology (ICZN 1988e)].

### Tribe Ulomini

Blanchard, 1845

Ulomites Blanchard, 1845b: 16 [stem: *Ulom-*]. Type genus: *Uloma* Dejean, 1821 [placed on the Official List of Generic Names in Zoology (ICZN 1975)]. Comment: original vernacular name available (Art. 11.7.2): first used in latinized form by Blanchard (1853: 164, as Ulomitae), generally accepted as in Bouchard et al. (2005: 502, as Ulomini); current spelling maintained (Art. 29.5): incorrect stem formation in prevailing usage (should be *Ulomat*-).
*Oligocaroides Solier, 1851: 225 [stem: *Oligocar-*]. Type genus: *Oligocara* Solier, 1848. Comment: original vernacular name unavailable (Art. 11.7.2): not subsequently latinized.
Alégoriides Lacordaire, 1859: 325 [stem: *Alegori-*]. Type genus: *Alegoria* Laporte, 1840. Comment: original vernacular name available (Art. 11.7.2): first used in latinized form and generally accepted as in Seidlitz (1894: 545, as Alegoriina).
Neopsectropinae Kaszab, 1941: 30 [stem: *Neopsectropod-*]. Type genus: *Neopsectropus* Kaszab, 1941. Comment: incorrect original stem formation, not in prevailing usage.

### Subfamily Alleculinae

Laporte, 1840

Alléculites Laporte, 1840b: 242 [stem: *Allecul-*]. Type genus: *Allecula* Fabricius, 1801. Comment: given precedence over the older names Xystropodinae Solier, 1835 and Cteniopodinae Solier, 1835 (Art. 35.5) (see Bouchard et al. 2005).

### Tribe Alleculini

Laporte, 1840

Alléculites Laporte, 1840b: 242 [stem: *Allecul-*]. Type genus: *Allecula* Fabricius, 1801. Comment: given precedence over the older name Xystropodini Solier, 1835 (Art. 35.5) (see Bouchard et al. 2005).

### Subtribe Alleculina

Laporte, 1840

Alléculites Laporte, 1840b: 242 [stem: *Allecul-*]. Type genus: *Allecula* Fabricius, 1801. Comment: original vernacular name available (Art. 11.7.2): first used in latinized form by Packard (1874: iii, as Alleculidae), generally accepted as in Bouchard et al. (2005: 502, as Alleculina).
*Cylindrothorides Lacordaire, 1859: 494 [stem: *Cylindrothor-*]. Type genus: *Cylindrothorus* Solier, 1844. Comment: original vernacular name unavailable (Art. 11.7.2): not subsequently latinized.
Upinellae J. L. LeConte, 1866b: 137 [stem: *Upinell-*]. Type genus: *Upinella* Mulsant, 1856.

### Subtribe Gonoderina

Seidlitz, 1896

Gonoderina Seidlitz, 1896: 83 [stem: *Gonoder-*]. Type genus: *Gonodera* Mulsant, 1856.
Pseudocistelini Portevin, 1934: 39, in key [stem: *Pseudocistel-*]. Type genus: *Pseudocistela* Crotch, 1873.

### Subtribe Mycetocharina

Gistel, 1848

Mycetocharisidae Gistel, 1848: [10] [stem: *Mycetochar-*]. Type genus: *Mycetochara* Berthold, 1827 [as *Mycetochares*, incorrect subsequent spelling not in prevailing usage]. Comment: incorrect original stem formation, not in prevailing usage.

### Subtribe Xystropodina

Solier, 1835

Xystropides Solier, 1835a: 229 [stem: *Xystropod-*]. Type genus: *Xystropus* Solier, 1835. Comment: original vernacular name available (Art. 11.7.2): first used in latinized form by Agassiz (1846b: 390, as Xystropodidae), generally accepted as in Bouchard et al. (2005: 502, as Xystropodina); incorrect original stem formation, not in prevailing usage.
Lystronychides Lacordaire, 1859: 512 [stem: *Lystronych-*]. Type genus: *Lystronychus* Latreille, 1829 [incorrect subsequent spelling of *Lystronichus*, incorrect spelling in current usage, treated as correct original spelling (Art. 33.3.1)]. Comment: original vernacular name available (Art. 11.7.2): first used in latinized form and generally accepted as in J. L. LeConte and G. H. Horn (1883: 390, as Lystronychi).

### Tribe Cteniopodini

Solier, 1835

Cisteleniae Latreille, 1802: 188 [stem: *Cistel-*]. Type genus: *Cistela* Fabricius, 1775 [preoccupied genus name, not *Cistela* Geoffroy, 1762 [Coleoptera: Byrrhidae]; syn. of *Cteniopus* Solier, 1835; Geoffroy’s generic name was suppressed for the purposes of the Principle of Priority but not for the Principle of Homonymy (ICZN 1994a)]. Comment: permanently invalid (Art. 39): based on preoccupied type genus.
Cténiopites Solier, 1835a: 245 [stem: *Cteniopod-*]. Type genus: *Cteniopus* Solier, 1835. Comment: original vernacular name available (Art. 11.7.2): first used in latinized form by Agassiz (1846b: 107, as Cteniopodidae), generally accepted as in Bouchard et al. (2005: 502, as Cteniopodini); incorrect original stem formation, not in prevailing usage.
Omophliens Mulsant, 1856a: 65 [stem: *Omophl-*]. Type genus: *Omophlus* Dejean, 1834. Comment: original vernacular name available (Art. 11.7.2): first used in latinized form by Seidlitz (1896: 28, as Omophlini), generally accepted as in Ogloblin and Znojko (1950: 9, as Omophlinae).
Phibalidae Gistel, 1856a: 384 [stem: *Phibal-*]. Type genus: *Phibalus* Gistel, 1856 [the genus *Phibalus* Gistel, 1856 originally included only one species therefore the type species is *Chrysomela pubescens* Linnaeus, 1758 by monotypy; this genus has not been used subsequently to our knowledge; **syn. nov.** of *Omophlus* Dejean, 1834]. Comment: **syn. nov.**
Petriidae Semenov, 1893b: 359 [stem: *Petri-*]. Type genus: *Petria* Semenov, 1894.
Podontini Portevin, 1934: 45, in key [stem: *Podont-*]. Type genus: *Podonta* Solier, 1835.

### Subfamily Diaperinae

Latreille, 1802

Diaperialae Latreille, 1802: 161 [stem: *Diaper-*]. Type genus: *Diaperis* Geoffroy, 1762 [placed on the Official List of Generic Names in Zoology (ICZN 1994a)].

### Tribe Crypticini

Brullé, 1832

Crypticites Brullé, 1832: 190 [stem: *Cryptic-*]. Type genus: *Crypticus* Latreille, 1816. Comment: original vernacular name available (Art. 11.7.2): first used in latinized form by C. G. Thomson (1859: 114, as Crypticina), generally accepted as in Bouchard et al. (2005: 503, as Crypticini).
Oochrotini Desbrochers des Loges, 1901: 143, in key [stem: *Oochrot-*]. Type genus: *Oochrotus* Lucas, 1852.

### Tribe Diaperini

Latreille, 1802

Diaperialae Latreille, 1802: 161 [stem: *Diaper-*]. Type genus: *Diaperis* Geoffroy, 1762 [placed on the Official List of Generic Names in Zoology (ICZN 1994a)].

### Subtribe Adelinina

LeConte, 1862

*Sitophagiens Mulsant, 1854: 263 [stem: *Sitophag-*]. Type genus: *Sitophagus* Mulsant, 1854. Comment: original vernacular name unavailable (Art. 11.7.2): not subsequently latinized.
Alphitophagida Gistel, 1856b: 185 [stem: *Alphitophag-*]. Type genus: *Alphitophagus* Stephens, 1832. Comment: although this is the oldest name for the subtribe, we recommend that an application be submitted to the Commission to conserve usage of the name Adelinina J. L. LeConte, 1862.
*Gnathocérites Jacquelin du Val, 1861: 304 [stem: *Gnatocer-*]. Type genus: *Gnatocerus* Thunberg, 1814 [as *Gnathocerus*, incorrect subsequent spelling of type genus name, not in prevailing usage; *Gnatocerus* Thunberg, 1814 placed on the Official List of Generic Names in Zoology (ICZN 1975)]. Comment: original vernacular name unavailable (Art. 11.7.2): subsequently used in latinized form but not generally attributed to Jacquelin du Val (1861); incorrect original stem formation, not in prevailing usage; Gnathoceridae Schoch, 1894 is available in Scarabaeidae.
Adelinini J. L. LeConte, 1862: 237 [stem: *Adelin-*]. Type genus: *Adelina* Dejean, 1835. Comment: this well-established family-group name is threatened by the discovery of the older name Alphitophagida Gistel, 1856; although using Reversal of Precedence (Art. 23.9.1) to conserve usage of Adelinina J. L. LeConte, 1862 would be preferable, this can not be done because we cannot find 25 references in which this name has been used in the last 50 years; current usage is maintained here and an application should be submitted to the Commission in order to conserve Adelinina J. L. LeConte, 1862.
Schedarosini Reitter, 1876b: 42 [stem: *Schedaros-*]. Type genus: *Schedarosus* Reitter, 1876 [syn. of *Adelina* Dejean, 1835]. Comment: originally placed in the family Cucujidae.
Doliemini Reitter, 1917: 58 [stem: *Doliem-*]. Type genus: *Doliema* Pascoe, 1860 [syn. of *Adelina* Dejean, 1835].
Gnathocerini Skopin, 1978: 228, in key [stem: *Gnatocer-*]. Type genus: *Gnatocerus* Thunberg, 1814 [as *Gnathocerus*, incorrect subsequent spelling of type genus name, not in prevailing usage; *Gnatocerus* Thunberg, 1814 placed on the Official List of Generic Names in Zoology (ICZN 1975)]. Comment: incorrect original stem formation, not in prevailing usage; Gnathoceridae Schoch, 1894 is available in Scarabaeidae.

### Subtribe Diaperina

Latreille, 1802

Diaperialae Latreille, 1802: 161 [stem: *Diaper-*]. Type genus: *Diaperis* Geoffroy, 1762 [placed on the on Official List of Generic Names in Zoology (ICZN 1994a)].
Pentaphyllaires Mulsant, 1854: 196 [stem: *Pentaphyll-*]. Type genus: *Pentaphyllus* Dejean, 1821. Comment: original vernacular name available (Art. 11.7.2): first used in latinized form by J. L. LeConte (1862: 236, as Pentaphylli), generally accepted as in Silfverberg (1992: 61, as Pentaphyllini); the name Pentaphyllinae Schindewolf, 1942 (type genus *Pentaphyllum* de Koninck, 1872) is used as valid in Anthozoa; this case is to be referred to the Commission to remove the homonymy (Art. 55.3.1).
Platydeminae Reitter, 1917: 61 [stem: *Platydem-*]. Type genus: *Platydema* Laporte and Brullé, 1831.
*Hoplocephalini Kwieton, 1982: 98 [stem: *Hoplocephal-*]. Type genus: *Hoplocephala* Laporte and Brullé, 1831 [based on unjustified emendation of type genus name; originally proposed as *Oplocephala*; emended name in prevailing usage, treated as a justified emendation (Art. 33.2.3.1); syn. of *Neomida* Latreille, 1829]. Comment: unavailable family-group name, proposed after 1930 without description or bibliographic reference to such a description (Art. 13.1).

### Tribe Ectychini

Doyen, Matthews and Lawrence, 1990

Ectychini Doyen et al., 1990: 247 [stem: *Ectych-*]. Type genus: *Ectyche* Pascoe, 1869.

### Tribe Gnathidiini

Gebien, 1921

Gnathidiinae Gebien, 1921: 41 [stem: *Gnathidi-*]. Type genus: *Gnathidium* Gebien, 1921.

### Subtribe Anopidiina

Jeannel and Paulian, 1945

Anopidiini Jeannel and Paulian, 1945: 62 [stem: *Anopidi-*]. Type genus: *Anopidium* Jeannel and Paulian, 1945.

### Subtribe Gnathidiina

Gebien, 1921

Gnathidiinae Gebien, 1921: 41 [stem: *Gnathidi-*]. Type genus: *Gnathidium* Gebien, 1921.

### Tribe Hyociini

Medvedev and Lawrence, 1982

Hyocini G. S. Medvedev and Lawrence, 1982: 548 [stem: *Hyoci-*]. Type genus: *Hyocis* Pascoe, 1866.

### Subtribe Brittonina

Medvedev and Lawrence, 1986

Brittonina G. S. Medvedev and Lawrence, 1986: 574 [stem: *Britton-*]. Type genus: *Brittona* G. S. Medvedev and Lawrence, 1986.

### Subtribe Hyociina

Medvedev and Lawrence, 1982

Hyocini G. S. Medvedev and Lawrence, 1982: 548 [stem: *Hyoci-*]. Type genus: *Hyocis* Pascoe, 1866. Comment: incorrect original stem formation, not in prevailing usage.

### Subtribe Uptonina

Medvedev and Lawrence, 1986

Uptonina G. S. Medvedev and Lawrence, 1986: 581 [stem: *Upton-*]. Type genus: *Uptona* G. S. Medvedev and Lawrence, 1986.

### Tribe Hypophlaeini

Billberg, 1820

Hypophlaeides Billberg, 1820a: 33 [stem: *Hypophlae-*]. Type genus: *Hypophlaeus* Fabricius, 1790 [syn. of *Corticeus* Piller and Mitterpacher, 1783].
Corticeini Boddy, 1965: 144 [stem: *Cortice-*]. Type genus: *Corticeus* Piller and Mitterpacher, 1783.

### Tribe Leiochrinini

Lewis, 1894

Leiochrininae Lewis, 1894: 390 [stem: *Leiochrin-*]. Type genus: *Leiochrinus* Westwood, 1883.

### Tribe Myrmechixenini

Jacquelin du Val, 1858

Myrméchixénites Jacquelin du Val, 1858: 223 [stem: *Myrmechixen-*]. Type genus: *Myrmechixenus* Chevrolat, 1835. Comment: original vernacular name available (Art. 11.7.2): first used in latinized form by Marseul (1863: 111, as Myrmekixeni [incorrect stem formation]), generally accepted as in Bouchard et al. (2005: 503, as Myrmechixenini); originally placed in the family Mycetaeidae (syn. of Endomychidae).

### Tribe Phaleriini

Blanchard, 1845

Phalériides Blanchard, 1845b: 29 [stem: *Phaleri-*]. Type genus: *Phaleria* Latreille, 1802 [placed on the Official List of Generic Names in Zoology (ICZN 1975)]. Comment: original vernacular name available (Art. 11.7.2): first used in latinized form by Gistel (1848: [10], as Phaleriadae [incorrect stem formation]), generally accepted as in Bouchard et al. (2005: 503, as Phaleriini).
Sepedonastidae Gistel, 1856a: 382 [stem: *Sepedonast-*]. Type genus: *Sepedonastes* Gistel, 1856 [syn. of *Phaleria* Latreille, 1802].
Cataphronetini Reitter, 1917: 57 [stem: *Cataphronet-*]. Type genus: *Cataphronetis* Lucas, 1846 [syn. of *Phtora* Germar, 1836].

### Tribe Scaphidemini

Reitter, 1922

Scaphidemini Reitter, 1922b: 2 [stem: *Scaphidem-*]. Type genus: *Scaphidema* Redtenbacher, 1849.

### Tribe Trachyscelini

Blanchard, 1845

Trachyscélides Blanchard, 1845b: 28 [stem: *Trachyscel-*]. Type genus: *Trachyscelis* Latreille, 1809. Comment: original vernacular name available (Art. 11.7.2): first used in latinized form by G. R. Waterhouse (1858: 60, as Trachyscelidae), generally accepted as in Bouchard et al. (2005: 503, as Trachyscelini); current spelling maintained (Art. 29.5): incorrect stem formation in prevailing usage (should be *Trachyscelid*-); conservation of the original stem avoids potential homonymy with family-group names based on the type genus *Trachyscelida* G. H. Horn, 1893 in Chrysomelidae.

### Subfamily Stenochiinae

Kirby, 1837

Stenochiadae Kirby, 1837: 238 [stem: *Stenochi-*]. Type genus: *Stenochia* Kirby, 1819.

### Tribe Cnodalonini

Oken, 1843

Cnodaliden Oken, 1843: 484 [stem: *Cnodalon-*]. Type genus: *Cnodalon* Latreille, 1797. Comment: original vernacular name available (Art. 11.7.2): first used in latinized form and generally accepted as in Tulk (1847: 614, as Cnodalidae); family-group name previously attributed to Gistel (1856a: 382); the correct stem based on *Cnodalon* is *Cnodal*-, therefore the original stem was correctly formed; however, since this family-group name has not been attributed to Oken (1843) in the past and the accepted stem has been *Cnodalon*- to this date, we have kept the currently accepted stem; an application to the Commission is necessary to conserve *Cnodalon*- as the correct stem for this taxon.
Coelometopidae Schaum, 1859: 71 [stem: *Coelometop-*]. Type genus: *Coelometopus* Solier, 1848. Comment: published before February 31, 1859; this family-group name was also used in the same year by Lacordaire (1859 [before 27 June]: 358, as Coelométopides).
Upidae C. G. Thomson, 1859: 116 [stem: *Upid-*]. Type genus: *Upis* Fabricius, 1792. Comment: incorrect original stem formation, not in prevailing usage.
Eutélides Lacordaire, 1859: 354 [stem: *Eutel-*]. Type genus: *Eutelus* Solier, 1843 [preoccupied genus name, not *Eutelus* Walker, 1834 [Hymenoptera]; syn. of *Nodotelus* Koch, 1950]. Comment: original vernacular name available (Art. 11.7.2): first used in latinized form by Pascoe (1866b: 469, as Eutelinae), generally accepted as in Gebien (1911: 431, as Eutelinae); permanently invalid (Art. 39): based on preoccupied type genus; Eutelini Ashmead, 1904 (type genus *Eutelus* Walker, 1834) is available in Hymenoptera.
Misolampides Lacordaire, 1859: 440 [stem: *Misolamp-*]. Type genus: *Misolampus* Latreille, 1807. Comment: original vernacular name available (Art. 11.7.2): first used in latinized form by and generally accepted as in F. Bates (1873: 373, as Misolampides [treated as Latin]).
Catapiestides Lacordaire, 1859: 381 [stem: *Catapiest-*]. Type genus: *Catapiestus* Perty, 1831. Comment: original vernacular name available (Art. 11.7.2): first used in latinized form and generally accepted as in Seidlitz (1895: 614, as Catapiestina).
*Camarides Desmarest, 1860: 165 [stem: *Camari-*]. Type genus: *Camaria* Lepeletier and Audinet-Serville, 1828. Comment: original vernacular name unavailable (Art. 11.7.2): subsequently used in latinized form but not generally attributed to Desmarest (1860).
Polypleuri J. L. LeConte, 1862: 229 [stem: *Polypleur-*]. Type genus: *Polypleurus* Eschscholtz, 1831.
Misolampidiini Reitter, 1917: 60, in key [stem: *Misolampidi-*]. Type genus: *Misolampidius* Solsky, 1876.
Menephilini Reitter, 1920: 15 [stem: *Menephil-*]. Type genus: *Menephilus* Mulsant, 1854.
Stenophanini Reitter, 1922b: 2, 3 [stem: *Stenophan-*]. Type genus: *Stenophanes* Solsky, 1876.
Hegemonini Reitter, 1922b: 3 [stem: *Hegemon-*]. Type genus: *Hegemona* Laporte, 1840.
Camariinae Anonymous, 1924: 164 [stem: *Camari-*]. Type genus: *Camaria* Lepeletier and Audinet-Serville, 1828. Comment: this was a translation of the vernacular name “Camariinen” used by Gebien (1919); Gebien’s family-group name is not available since it was proposed in a vernacular form after 1899 (Art. 11.7.2); for comments about authorship of Camariinae see Evenhuis et al. (2008: 4).
Nodotelini Koch, 1950: 67 [stem: *Nodotel-*]. Type genus: *Nodotelus* Koch, 1950. Comment: replacement name for Eutelini Lacordaire, 1859 because of the homonymy of the type genus.
*Thesileini Kaszab, 1982: 29 [stem: *Thesile-*]. Type genus: *Thesilea* Haag-Rutenberg, 1878. Comment: unavailable family-group name, proposed after 1930 without description or bibliographic reference to such a description (Art. 13.1).

### Tribe Stenochiini

Kirby, 1837

Stenochiadae Kirby, 1837: 238 [stem: *Stenochi-*]. Type genus: *Stenochia* Kirby, 1819. Comment: incorrect original stem formation, not in prevailing usage.
Strongyliides Lacordaire, 1859: 478 [stem: *Strongyli-*]. Type genus: *Strongylium* Kirby, 1819 [name conserved over *Strongylium* Ditmar, 1809 [Myxomycota] (see Bouchard et al. 2005)]. Comment: original vernacular name available (Art. 11.7.2): first used in latinized form by J. L. LeConte (1862: 241, as Strongyliini), generally accepted as in Aalbu et al. (2002: 483, as Strongyliini).

### Tribe Talanini

Champion, 1887 (1883)

Dignamptini J. L. LeConte and G. H. Horn, 1883: 385 [stem: *Dignampt-*]. Type genus: *Dignamptus* J. L. LeConte, 1878 [syn. of *Talanus* Jacquelin du Val, 1857].
Talanides Champion, 1887: 321 [stem: *Talan-*]. Type genus: *Talanus* Jacquelin du Val, 1857. Comment: family-group name conserved over Dignamptini J. L. LeConte and G. H. Horn, 1883 (Art. 40.2) (see Bouchard et al. 2005).

### Tenebrionidae incertae sedis

Betschiini Dajoz, 1980a: 135 [stem: *Betschi-*]. Type genus: *Betschia* Dajoz, 1980. Comment: originally placed in the family Colydiidae, the type genus was transferred to Tenebrionidae by Ivie and Ślipiński (1990: 18); placement uncertain although probably close to Gnathidiini (Ivie pers. comm. 2008).

### Family Prostomidae

Thomson, 1859

*Mégagnathes Motschulsky, 1849: 60 [stem: *Megagnath-*]. Type genus: *Megagnathus* Dejean, 1821 [preoccupied genus name, not *Megagnathus* Billberg, 1820 [Coleoptera: Cerambycidae]; syn. of *Prostomis* Latreille, 1819]. Comment: original vernacular name unavailable (Art. 11.7.2): not subsequently latinized; if found to be available then permanently invalid (Art. 39): based on preoccupied type genus.
Prostomidae C. G. Thomson, 1859: 84 [stem: *Prostom-*]. Type genus: *Prostomis* Latreille, 1819. Comment: current spelling maintained (Art. 29.3.1.1): incorrect stem formation in prevailing usage (should be *Prostomid*-).

### Family Synchroidae

Lacordaire, 1859

Synchroïdes Lacordaire, 1859: 544 [stem: *Synchro-*]. Type genus: *Synchroa* Newman, 1838. Comment: original vernacular name available (Art. 11.7.2): first used in latinized form by J. L. LeConte (1862: 249, as Synchroae), generally accepted as in Lawrence and Newton (1995: 894, as Synchroidae).
Mallodryini G. H. Horn, 1888: 43 [stem: *Mallodry-*]. Type genus: *Mallodrya* G. H. Horn, 1888.

### Family Stenotrachelidae

Thomson, 1859

Stenotrachelidae C. G. Thomson, 1859: 124 [stem: *Stenotrachel-*]. Type genus: *Stenotrachelus* Berthold, 1827.

### Subfamily Stenotrachelinae

Thomson, 1859

Stenotrachelidae C. G. Thomson, 1859: 124 [stem: *Stenotrachel-*]. Type genus: *Stenotrachelus* Berthold, 1827.

### Subfamily Cephaloinae

LeConte, 1862

*Cephalaonides Motschulsky, 1860: 140 [stem: *Cephalo-*]. Type genus: *Cephaloon* Newman, 1838 [as *Cephalaon*, incorrect subsequent spelling of type genus name corrected by Motschulsky to *Cephaloon* on pp. 140 in same work]. Comment: original vernacular name unavailable (Art. 11.7.2): subsequently used in latinized form but not generally attributed to Motschulsky (1860).
Cephaloidae J. L. LeConte, 1862: 259 [stem: *Cephalo-*]. Type genus: *Cephaloon* Newman, 1838.

### Subfamily Nematoplinae

LeConte, 1862

Nematopli J. L. LeConte, 1862: 264 [stem: *Nematopl-*]. Type genus: *Nematoplus* J. L. LeConte, 1855.

### Subfamily Stoliinae

Nikitsky, 1985

Stoliinae Nikitsky, 1985: 32, in key [stem: *Stoli-*]. Type genus: *Stolius* Lewis, 1895.

### Family Oedemeridae

Latreille, 1810

Oedemerites Latreille, 1810: 216 [stem: *Oedemer-*]. Type genus: *Oedemera* A. G. Olivier, 1789.

### Subfamily Polypriinae

Lawrence, 2005

*Polypriidae Triplehorn and N. F. Johnson, 2005: 441 [stem: *Polypri-*]. Type genus: *Polypria* Chevrolat, 1874. Comment: unavailable family-group name, name published after 1999 and not explicitly indicated as new (Art. 16.1).
Polypriinae Lawrence, 2005: 671, in key [stem: *Polypri-*]. Type genus: *Polypria* Chevrolat, 1874.

### Subfamily Calopodinae

Costa, 1852 nomen protectum

Sparedriidae Gistel, 1848: [11] [stem: *Sparedr-*]. Type genus: *Sparedrus* Dejean, 1821. Comment: *nomen oblitum* (see Appendix 1); incorrect original stem formation, not in prevailing usage.
Calopini A. Costa, 1852: 4 [stem: *Calopod-*]. Type genus: *Calopus* Fabricius, 1775. Comment: original vernacular name available (Art. 11.7.2): first used in latinized form by Semenov (1894: 449, as Calopodidae), generally accepted as in Švihla (2008: 353, as Calopodinae); *nomen protectum* (see Appendix 1); incorrect original stem formation, not in prevailing usage.
Catachirotidae Gistel, 1856a: 384 [stem: *Catachirotet-*]. Type genus: *Catachirotes* Gistel, 1856 [syn. of *Calopus* Fabricius, 1775]. Comment: incorrect original stem formation, not in prevailing usage.

### Subfamily Oedemerinae

Latreille, 1810

Oedemerites Latreille, 1810: 216 [stem: *Oedemer-*]. Type genus: *Oedemera* A. G. Olivier, 1789.

### Tribe Asclerini

Gistel, 1848

Ascleraeidae Gistel, 1848: [11] [stem: *Ascler-*]. Type genus: *Asclera* Stephens, 1839. Comment: incorrect original stem formation, not in prevailing usage.
Ganglbaueriidae Semenov, 1894: 450 [stem: *Ganglebaueri-*]. Type genus: *Ganglbaueria* Semenov, 1891.
*Hypasclerini Macnamara, 1971: 164 [stem: *Hypascler-*]. Type genus: *Hypasclera* Kirsch, 1866. Comment: unavailable family-group name, proposed after 1930 without description or bibliographic reference to such a description (Art. 13.1).
*Hypasclerini Švihla, 1986: 161 [stem: *Hypascler-*]. Type genus: *Hypasclera* Kirsch, 1866. Comment: family-group name unavailable (Art. 11.6): originally published as synonym and not made available subsequently.
*Danacerinae Švihla, 1986: 161 [stem: *Danerc-*]. Type genus: *Danerces* Westwood, 1875. Comment: family-group name unavailable (Art. 11.6): originally published as synonym and not made available subsequently; incorrect original stem formation, not in prevailing usage.
*Oxacini Švihla, 1986: 161 [stem: *Oxacid-*]. Type genus: *Oxacis* J. L. LeConte, 1866. Comment: family-group name unavailable (Art. 11.6): originally published as synonym and not made available subsequently; incorrect original stem formation, not in prevailing usage.

### Tribe Ditylini

Mulsant, 1858

*Dityles Motschulsky, 1849: 59 [stem: *Dityl-*]. Type genus: *Ditylus* Fischer von Waldheim, 1817. Comment: original vernacular name unavailable (Art. 11.7.2): subsequently used in latinized form but not generally attributed to Motschulsky (1849).
Ditylates Mulsant, 1858: 100 [stem: *Dityl-*]. Type genus: *Ditylus* Fischer von Waldheim, 1817. Comment: original vernacular name available (Art. 11.7.2): first used in latinized form by Semenov (1894: 450, as Ditylidae), generally accepted as in Kriska (2002: 517, as Ditylini).

### Tribe Nacerdini

Mulsant, 1858

Nacerdates Mulsant, 1858: 104 [stem: *Nacerd-*]. Type genus: *Nacerdes* Dejean, 1834. Comment: original vernacular name available (Art. 11.7.2): first used in latinized form by Semenov (1894: 450, as Nacerdidae), generally accepted as in Hansen (1996: 175, as Nacerdini).

### Tribe Oedemerini

Latreille, 1810

Oedemerites Latreille, 1810: 216 [stem: *Oedemer-*]. Type genus: *Oedemera* A. G. Olivier, 1789.
Oncomeradae Gistel, 1856a: 384 [stem: *Oncomer-*]. Type genus: *Oncomera* Stephens, 1829 [subgenus of *Oedemera* A. G. Olivier, 1789; placed on the Official List of Generic Names in Zoology (ICZN 1988f)]. Comment: incorrect original stem formation, not in prevailing usage.
*Oncomerinini Švihla, 1986: 161 [stem: *Oncomerin-*]. Type genus: *Oncomerina* Seidlitz, 1899 [syn. of *Oncomera* Stephens, 1829]. Comment: family-group name unavailable (Art. 11.6): originally published as synonym and not made available subsequently.

### Tribe Stenostomatini

Mulsant, 1858

Sténostomates Mulsant, 1858: 228 [stem: *Stenostomat-*]. Type genus: *Stenostoma* Latreille, 1810. Comment: original vernacular name available (Art. 11.7.2): first used in latinized form by Semenov (1894: 450, as Stenostomatidae), generally accepted as in Švihla (2008: 369, as Stenostomini [incorrect stem formation]); incorrect original stem formation, not in prevailing usage.

### Family Meloidae

Gyllenhal, 1810

Melooides Gyllenhal, 1810: 481 [stem: *Melo-*]. Type genus: *Meloe* Linnaeus, 1758 [placed on the Official List of Generic Names in Zoology (ICZN 1999c)]. Comment: given precedence over the older name Horiidae Latreille, 1802 and placed on the Official List of Family-Group Names in Zoology (ICZN 1999c).

### Subfamily Eleticinae

Wellman, 1910

Eleticides Wellman, 1910: 221 [stem: *Eletic-*]. Type genus: *Eletica* Dejean, 1834. Comment: First Reviser (Eleticinae Wellman, 1910 vs Derideinae Wellman, 1910) not determined, current usage maintained.

### Tribe Derideini

Wellman, 1910

Derideides Wellman, 1910: 222 [stem: *Deride-*]. Type genus: *Deridea* Westwood, 1875.

### Tribe Eleticini

Wellman, 1910

Eleticides Wellman, 1910: 221 [stem: *Eletic-*]. Type genus: *Eletica* Dejean, 1834.

### Subtribe Eleticina

Wellman, 1910

Eleticides Wellman, 1910: 221 [stem: *Eletic-*]. Type genus: *Eletica* Dejean, 1834.

### Subtribe Eospastina

Selander, 1966

Eospastina Selander, 1966: 474 [stem: *Eospast-*]. Type genus: *Eospasta* Selander, 1966.

### Tribe Ertlianini

Selander, 1966

Ertliini Kaszab, 1959: 71 [stem: *Ertli-*]. Type genus: *Ertlia* Borchmann, 1942 [preoccupied genus name, not *Ertlia* Aurivillius, 1907 [Coleoptera: Cerambycidae]; syn. of *Morphozonitis* Pic, 1922]. Comment: permanently invalid (Art. 39): based on preoccupied type genus.
Ertlianina Selander, 1966: 478 [stem: *Ertlian-*]. Type genus: *Ertliana* Selander, 1964 [syn. of *Morphozonitis* Pic, 1922]. Comment: replacement name for Ertliini Kaszab, 1959 because of the homonymy of the type genus; although Selander (1991: 78) used Morphozonitina Kaszab, 1969 as the valid name for this taxon (because of the synonymy of *Ertliana* Selander with *Morphozonitis* Pic) the Principle of Priority applies here and therefore the oldest available family-group name Ertlianini should be used as valid instead.
Morphozonitini Kaszab, 1969: 242 [stem: *Morphozonitid-*]. Type genus: *Morphozonitis* Pic, 1922. Comment: replacement name for Ertliini Kaszab, 1959 because of the homonymy of the type genus and Ertlianina Selander, 1966 because of the synonymy of the type genus; incorrect original stem formation, not in prevailing usage.

### Tribe Spasticini

Kaszab, 1959

Spasticina Kaszab, 1959: 72 [stem: *Spastic-*]. Type genus: *Spastica* Lacordaire, 1859.

### Subtribe Anthicoxenina

Selander, 1966

Anthicoxenina Selander, 1966: 470 [stem: *Anthicoxen-*]. Type genus: *Anthicoxenus* Fairmaire and Germain, 1860.

### Subtribe Protomeloina

Abdullah, 1965

Protomeloini M. Abdullah, 1965b: 43 [stem: *Protomelo-*]. Type genus: *Protomeloe* M. Abdullah, 1965.

### Subtribe Spasticina

Kaszab, 1959

Spasticina Kaszab, 1959: 72 [stem: *Spastic-*]. Type genus: *Spastica* Lacordaire, 1859.

### Subtribe Xenospastina

Selander, 1966

Xenospastina Selander, 1966: 460, in key [stem: *Xenospast-*]. Type genus: *Xenospasta* Selander, 1966.

### Subfamily Meloinae

Gyllenhal, 1810

Melooides Gyllenhal, 1810: 481 [stem: *Melo-*]. Type genus: *Meloe* Linnaeus, 1758 [placed on the Official List of Generic Names in Zoology (ICZN 1999c)].

### Tribe Cerocomini

Leach, 1815

Cerocomatida Leach, 1815: 105 [stem: *Cerocom-*]. Type genus: *Cerocoma* Geoffroy, 1762 [placed on the Official List of Generic Names in Zoology (ICZN 1994a)].

### Tribe Epicautini

Parker and Böving, 1924

Macrobases J. L. LeConte, 1862: 272 [stem: *Macrobase-*]. Type genus: *Macrobasis* J. L. LeConte, 1862 [subgenus of *Epicauta* Dejean, 1834]. Comment: incorrect original stem formation, not in prevailing usage.
Apterospastides Wellman, 1910: 221 [stem: *Apterospast-*]. Type genus: *Apterospasta* J. L. LeConte, 1862 [syn. of *Macrobasis* J. L. LeConte, 1862].
Epicautini Parker and Böving, 1924: 25 [stem: *Epicaut-*]. Type genus: *Epicauta* Dejean, 1834. Comment: family-group name previously attributed to Denier (1935); this name is threatened by the two older names Macrobaseini J. L. LeConte, 1862 and Apterospastini Wellman, 1910; to maintain nomenclatural stability, we believe that an application should be submitted to the Commission to conserve usage of the well-established name Epicautini.
*Henoini Böving and Craighead, 1931: 79 [stem: *Heno-*]. Type genus: *Henous* Haldeman, 1852 [syn. of *Epicauta* Dejean, 1834]. Comment: unavailable family-group name, proposed after 1930 without description or bibliographic reference to such a description (Art. 13.1).

### Tribe Eupomphini

LeConte, 1862

Eupomphae J. L. LeConte, 1862: 274 [stem: *Eupomph-*]. Type genus: *Eupompha* J. L. LeConte, 1858. Comment: First Reviser found (Eupomphini J. L. LeConte, 1862 vs Phodagini J. L. LeConte, 1862) is Selander (1991: 78, as Eupomphina).
Phodagae J. L. LeConte, 1862: 274 [stem: *Phodag-*]. Type genus: *Phodaga* J. L. LeConte, 1858.
Calospastides Wellman, 1910: 221 [stem: *Calospast-*]. Type genus: *Calospasta* J. L. LeConte, 1862 [syn. of *Eupompha* J. L. LeConte, 1858].
Gynaecomelodides Wellman, 1910: 221 [stem: *Gynaecomelo-*]. Type genus: *Gynaecomeloe* Wellman, 1910 [syn. of *Cordylospasta* G. H. Horn, 1875]. Comment: incorrect original stem formation, not in prevailing usage.
Cordylospastides Wellman, 1910: 221 [stem: *Cordylospast-*]. Type genus: *Cordylospasta* G. H. Horn, 1875.
Cysteodemides Wellman, 1910: 221 [stem: *Cysteodem-*]. Type genus: *Cysteodemus* J. L. LeConte, 1851.
Tegroderini L. S. Dillon, 1952: 373 [stem: *Tegroder-*]. Type genus: *Tegrodera* J. L. LeConte, 1851.
Megetrina Kaszab, 1959: 80 [stem: *Megetr-*]. Type genus: *Megetra* J. L. LeConte, 1859.

### Tribe Lyttini

Solier, 1851

Cantharidiae Latreille, 1802: 185 [stem: *Canthar-*]. Type genus: *Cantharis* sensu Latreille, 1802 [not *Cantharis* Linnaeus, 1758; syn. of *Lytta* Fabricius, 1775]. Comment: based on a misidentified type genus, name treated here as invalid until an application is submitted to the Commission to suppress it for the Principles of Priority and Homonymy (Art. 65.2.1); also see Cantharidae Imhoff, 1856.
*Lyttes Motschulsky, 1849: 59 [stem: *Lytt-*]. Type genus: *Lytta* Fabricius, 1775. Comment: original vernacular name unavailable (Art. 11.7.2): subsequently used in latinized form but not generally attributed to Motschulsky (1849).
Lyttoides Solier, 1851: 278 [stem: *Lytt-*]. Type genus: *Lytta* Fabricius, 1775. Comment: original vernacular name available (Art. 11.7.2): first used in latinized form by Gistel (1856a: 385, as Lyttadae [incorrect stem formation]), generally accepted as in Selander (1991: 78, as Lyttina); although this is not the oldest name for the tribe, we recommend that an application be submitted to the Commission to suppress Cantharini Latreille, 1802 because it is based on a misidentified type genus (Art. 65.2.1).
*Alosimates Mulsant, 1857: 358 [stem: *Alosim-*]. Type genus: *Alosimus* Mulsant, 1857. Comment: original vernacular name unavailable (Art. 11.7.2): subsequently used in latinized form, e.g., Selander (1991: 65, as Alosimina), but not generally accepted as valid; Selander’s name is also unavailable, it was proposed after 1930 without description or bibliographic reference to such a description (Art. 13.1).
*Lydini Wellman, 1908: 424 [stem: *Lyd-*]. Type genus: *Lydus* Dejean, 1821. Comment: family-group name unavailable (Art. 11.6): originally published as synonym and not made available subsequently; Wellman correctly pointed out that the family-group for a taxon based on *Lydus* should be Lydini, however he did not want to use this family-group name since the name Lydidae Newman, 1834 (type genus *Lyda* Fabricius, 1805) is available for a group of Hymenoptera: Symphyta.
Sybarides Wellman, 1910: 221 [stem: *Sybare-*]. Type genus: *Sybaris* Stephens, 1832. Comment: incorrect original stem formation, not in prevailing usage.
Lydina Kaszab, 1959: 82 [stem: *Lyd-*]. Type genus: *Lydus* Dejean, 1821. Comment: Kaszab proposed the subtribe name Lydina without reference to the comment by Wellman (1908) and his family-group name has been used as valid in Meloidae since (e.g., Turco et al. 2006); the name Lydidae Newman, 1834 (type genus *Lyda* Fabricius, 1805) is available for a group of Hymenoptera: Symphyta; this case is to be referred to the Commission to remove the homonymy (Art. 55.3.1).
Prolyttini Selander, 1960: 25 [stem: *Prolytt-*]. Type genus: *Prolytta* Kaszab, 1959.

### Tribe Meloini

Gyllenhal, 1810

Melooides Gyllenhal, 1810: 481 [stem: *Melo-*]. Type genus: *Meloe* Linnaeus, 1758 [placed on the Official List of Generic Names in Zoology (ICZN 1999c)].
Proscarabaeidae Gistel, 1848: [11] [stem: *Proscarabae-*]. Type genus: *Proscarabaeus* Schrank, 1781 [syn. of *Meloe* Linnaeus, 1758].

### Tribe Mylabrini

Rafinesque, 1815

Mylabria Rafinesque, 1815: 114 [stem: *Mylabr-*]. Type genus: *Mylabris* Fabricius, 1775 [placed on the Official List of Generic Names in Zoology (ICZN 1995a)]. Comment: family-group name previously attributed to Billberg (1820a); Brullé (1832: 190) uses the family-group name “Corynites” for a group containing the genera *Sarrotrium* Illiger, 1798, *Orthocerus* Latreille, 1797, *Chiroscelis* Lamarck, 1804, *Toxicum* Latreille, 1802 and *Boros* Herbst, 1797, although Brullé could have based his family-group name on *Coryna* Billberg, 1813 (which is a junior homonym and a synonym of *Hycleus* Latreille, 1817 in Mylabrini) we cannot be sure and therefore we have not considered this a separate family-group name.
Zonabrini Wellman, 1908: 424 [stem: *Zonabr-*]. Type genus: *Zonabris* Harold, 1879 [syn. of *Mylabris* Fabricius, 1775].
Calydina Kaszab, 1960: 126 [stem: *Calyd-*]. Type genus: *Calydus* Reitter, 1896.

### Tribe Pyrotini

MacSwain, 1956

Pyrotini MacSwain, 1956: 67 [stem: *Pyrot-*]. Type genus: *Pyrota* Dejean, 1834.

### Subfamily Tetraonycinae

Böving and Craighead, 1931

Tetraonycidae Böving and Craighead, 1931: 59, in key [stem: *Tetraonyc-*]. Type genus: *Tetraonyx* Latreille, 1809. Comment: current spelling maintained (Art. 29.5): incorrect stem formation in prevailing usage (should be *Tetraonych*-).
Meloetyphlini Borgmeier, 1937: 248 [stem: *Meloetyphl-*]. Type genus: *Meloetyphlus* C. O. Waterhouse, 1872.

### Subfamily Nemognathinae

Laporte, 1840

Nemognathites Laporte, 1840b: 280 [stem: *Nemognath-*]. Type genus: *Nemognatha* Illiger, 1807 [as *Nemognathus*, unjustified emendation of type genus name by Latreille (1829b), not in prevailing usage; *Nemognatha* Illiger, 1807 placed on the Official List of Generic Names in Zoology (ICZN 1999c)]. Comment: Nemognathinae Laporte, 1840 placed on the Official List of Family-Group Names in Zoology and given precedence over Horiinae Latreille, 1802 (ICZN 1999c).

### Tribe Horiini

Latreille, 1802

Horiales Latreille, 1802: 182 [stem: *Hori-*]. Type genus: *Horia* Fabricius, 1787 [placed on the Official List of Generic Names in Zoology (ICZN 1999c)]. Comment: Horiidae Latreille, 1802 placed on the Official List of Family-Group Names in Zoology (ICZN 1999c); Meloidae Gyllenhal, 1810 and Nemognathinae Laporte, 1840 given precedence over this name (ICZN 1999c).
Cissitini Kaszab, 1966: 187 [stem: *Cissit-*]. Type genus: *Cissites* Latreille, 1804. Comment: we accept the character about parameres given by Kaszab (1966: 187) to characterize this tribe as sufficient to fulfill the requirements of Art. 13.1; Selander (1991: 66) treated this name as unavailable.

### Tribe Nemognathini

Laporte, 1840

Nemognathites Laporte, 1840b: 280 [stem: *Nemognath-*]. Type genus: *Nemognatha* Illiger, 1807 [as *Nemognathus*, unjustified emendation of type genus name by Latreille (1829b), not in prevailing usage; *Nemognatha* Illiger, 1807 placed on the Official List of Generic Names in Zoology (ICZN 1999c)].

### Subtribe Nemognathina

Laporte, 1840

Nemognathites Laporte, 1840b: 280 [stem: *Nemognath-*]. Type genus: *Nemognatha* Illiger, 1807 [as *Nemognathus*, unjustified emendation of type genus name by Latreille (1829b), not in prevailing usage; *Nemognatha* Illiger, 1807 placed on the Official List of Generic Names in Zoology (ICZN 1999c)]. Comment: original vernacular name available (Art. 11.7.2): first used in latinized form by J. L. LeConte (1862: 270, as Nemognathini), generally accepted as in Hansen (1996: 176, as Nemognathinae).
Hornii Dugès, 1889: 213 [stem: *Horni-*]. Type genus: *Hornia* Riley, 1877.
Tricraniides Wellman, 1910: 222 [stem: *Tricrani-*]. Type genus: *Tricrania* J. L. LeConte, 1860.

### Subtribe Zonitidina

Mulsant, 1857

*Zonites Motschulsky, 1849: 59 [stem: *Zonitid-*]. Type genus: *Zonitis* Fabricius, 1775 [placed on the Official List of Generic Names in Zoology (ICZN 1999c)]. Comment: original vernacular name unavailable (Art. 11.7.2): subsequently used in latinized form but not generally attributed to Motschulsky (1849); incorrect original stem formation, not in prevailing usage.
Zonitaires Mulsant, 1857: 322 [stem: *Zonitid-*]. Type genus: *Zonitis* Fabricius, 1775 [placed on the Official List of Generic Names in Zoology (ICZN 1999c)]. Comment: incorrect original stem formation, not in prevailing usage; Zonitidinae Mulsant, 1857 placed on the Official List of Family-Group Names in Zoology (ICZN 1999c); this family-group name is a senior homonym of Zonitidae Mörch, 1864 in Mollusca (based on *Zonites* Montfort, 1810) which is used as valid in recent literature (see Bologna and Pinto 1997); stem of family-group name changed to *Zonitid*- by the Commission (ICZN 1999c) to remove from homonymy with Mörch’s name.
*Leptopalpina Kaszab, 1959: 102, Fig. 97 [stem: *Leptopalp-*]. Type genus: *Leptopalpus* Guérin-Méneville, 1834. Comment: unavailable family-group name, proposed after 1930 without description or bibliographic reference to such a description (Art. 13.1).
Leptopalpina Kaszab, 1963: 138 [stem: *Leptopalp-*]. Type genus: *Leptopalpus* Guérin-Méneville, 1834.
*Palaestrina Kaszab, 1969: 244 [stem: *Palaestr-*]. Type genus: *Palaestra* Laporte, 1840. Comment: unavailable family-group name, proposed after 1930 without description or bibliographic reference to such a description (Art. 13.1).

### Subtribe Sitarina

Mulsant, 1857

*Apales Motschulsky, 1849: 59 [stem: *Apal-*]. Type genus: *Apalus* Fabricius, 1775. Comment: original vernacular name unavailable (Art. 11.7.2): subsequently used in latinized form but not generally attributed to Motschulsky (1849).
Sitarates Mulsant, 1857: 393 [stem: *Sitar-*]. Type genus: *Sitaris* Latreille, 1802. Comment: original vernacular name available (Art. 11.7.2): first used in latinized form by Dugès (1886: 580, as Sitarini), generally accepted as in Selander (1991: 79, as Sitarina).
Apalides Parker and Böving, 1924: 33 [stem: *Apal-*]. Type genus: *Apalus* Fabricius, 1775.
Stenoriides Parker and Böving, 1924: 32 [stem: *Stenori-*]. Type genus: *Stenoria* Mulsant, 1857.

### Tribe Stenoderini

Selander, 1991

*Stenoderini Selander, 1964: 1057 [stem: *Stenoder-*]. Type genus: *Stenodera* Eschscholtz, 1818. Comment: unavailable family-group name, proposed after 1930 without description or bibliographic reference to such a description (Art. 13.1).
Stenoderini Selander, 1991: 77 [stem: *Stenoder-*]. Type genus: *Stenodera* Eschscholtz, 1818. Comment: junior homonym of Stenoderini Pascoe, 1867 (type genus *Stenoderus* Dejean, 1821) currently used as valid in Cerambycidae; this case is to be referred to the Commission to remove the homonymy (Art. 55.3.1).

### Family Mycteridae

Oken, 1843

Mycteriden Oken, 1843: 484 [stem: *Mycter-*]. Type genus: *Mycterus* Clairville, 1798.

### Subfamily Mycterinae

Oken, 1843

Rhinomaceridae Fleming, 1821: 50 [stem: *Rhinomacr-*]. Type genus: *Rhinomacer* Fabricius, 1781 [placed on the Official Index of Rejected and Invalid Generic Names in Zoology (ICZN 2005c); syn. of *Mycterus* Clairville, 1798]. Comment: permanently invalid (Art. 39): based on suppressed type genus; incorrect original stem formation, not in prevailing usage.
Mycteriden Oken, 1843: 484 [stem: *Mycter-*]. Type genus: *Mycterus* Clairville, 1798. Comment: original vernacular name available (Art. 11.7.2): first used in latinized form and generally accepted as in Tulk (1847: 614, as Mycteridae); name previously attributed to Blanchard (1845b: 97).
Artaxidae Gistel, 1848: [8] [stem: *Artax-*]. Type genus: *Artaxus* Gistel, 1848 [syn. of *Mycterus* Clairville, 1798].

### Subfamily Eurypinae

Thomson, 1860

Eurypitae J. Thomson, 1860b: 63 [stem: *Euryp-*]. Type genus: *Eurypus* Kirby, 1819. Comment: the correct stem based on the type genus *Eurypus* is *Eurypod*-, however, we prefer to conserve the incorrect original stem formation for this name (Art. 29.5) to avoid homoymy with Eurypodini Gahan, 1906 (type genus *Eurypoda* W. Saunders, 1853) currently used as valid in Cerambycidae.
Lacconotini J. L. LeConte, 1862: 254 [stem: *Lacconot-*]. Type genus: *Lacconotus* J. L. LeConte, 1862.
Batobiina Seidlitz, 1917a: 88 [stem: *Batobi-*]. Type genus: *Batobius* Fairmaire and Germar, 1863.
Thisiina Seidlitz, 1917a: 91 [stem: *Thisi-*]. Type genus: *Thisias* Champion, 1889.
*Stilpnonotinae Blackwelder, 1945: 503 [stem: *Stilpnonot-*]. Type genus: *Stilpnonotus* Gray, 1832. Comment: unavailable family-group name, proposed after 1930 without description or bibliographic reference to such a description (Art. 13.1).

### Subfamily Hemipeplinae

Lacordaire, 1854

Hémipéplides Lacordaire, 1854b: 404 [stem: *Hemipepl-*]. Type genus: *Hemipeplus* Latreille, 1829. Comment: original vernacular name available (Art. 11.7.2): first used in latinized form by J. L. LeConte (1861: 96, as Hemipeplidae), generally accepted as in Pollock (2002: 532, as Hemipeplinae).

### Family Boridae

Thomson, 1859

Boridae C. G. Thomson, 1859: 117 [stem: *Bor-*]. Type genus: *Boros* Herbst, 1797.

### Subfamily Borinae

Thomson, 1859

Boridae C. G. Thomson, 1859: 117 [stem: *Bor-*]. Type genus: *Boros* Herbst, 1797.

### Subfamily Synercticinae

Lawrence and Pollock, 1994

Synercticinae Lawrence and Pollock, 1994: 36, in key [stem: *Synerctic-*]. Type genus: *Synercticus* Newman, 1842.

### Family Trictenotomidae

Blanchard, 1845

Tricténotomides Blanchard, 1845b: 137 [stem: *Trictenotom-*]. Type genus: *Trictenotoma* Gray, 1832. Comment: original vernacular name available (Art. 11.7.2): first used in latinized form by F. Smith (1851: 18, as Trictenotomidae), generally accepted as in Lawrence and Newton (1995: 897, as Trictenotomidae).

### Family Pythidae

Solier, 1834

Pythites Solier, 1834: 496 [stem: *Pyth-*]. Type genus: *Pytho* Latreille, 1797. Comment: original vernacular name available (Art. 11.7.2): first used in latinized form by C. G. Thomson (1859: 123, as Pythonidae [incorrect stem formation]), generally accepted as in Hansen (1996: 176, as Pythidae).
Enoptisidae Gistel, 1848: [11] [stem: *Enopt-*]. Type genus: *Enoptes* Gistel, 1848 [syn. of *Pytho* Latreille, 1797]. Comment: incorrect original stem formation, not in prevailing usage.
Ischyomiides Champion, 1886: 258 [stem: *Ischyomi-*]. Type genus: *Ischyomius* Chevrolat, 1878.
Osphyoplesiini Reitter, 1917: 59, in key [stem: *Osphyoplesi-*]. Type genus: *Osphyoplesius* A. Winkler, 1915.
Anaplopinae M. Abdullah, 1966: 147 [stem: *Anaplopod-*]. Type genus: *Anaplopus* Blackburn, 1890. Comment: incorrect original stem formation, not in prevailing usage.

### Family Pyrochroidae

Latreille, 1806

Pyrochroides Latreille, 1806: 199 [stem: *Pyrochro-*]. Type genus: *Pyrochroa* Geoffroy, 1762 [placed on the Official List of Generic Names in Zoology (ICZN 1994a)].

### Subfamily Tydessinae

Nikitsky, 1986

Tydessini Nikitsky, 1986: 1188 [stem: *Tydess-*]. Type genus: *Tydessa* Peacock, 1982.

### Subfamily Pilipalpinae

Abdullah, 1964

Pilipalpini M. Abdullah, 1964: 4 [stem: *Pilipalp-*]. Type genus: *Pilipalpus* Fairmaire, 1876.
Techmessinae Paulus, 1972b: 84, in key [stem: *Techmess-*]. Type genus: *Techmessa* F. Bates, 1874.

### Subfamily Pedilinae

Lacordaire, 1859

Pédilides Lacordaire, 1859: 574 [stem: *Pedil-*]. Type genus: *Pedilus* Fischer von Waldheim, 1820. Comment: published before 27 June 1859; original vernacular name available (Art. 11.7.2): first used in latinized form by J. L. LeConte (1859a [December]: 46, as Pedilidae), generally accepted as in Lawrence and Newton (1995: 898, as Pedilinae).
Neopedilini M. Abdullah, 1969a: 354 [stem: *Neopedil-*]. Type genus: *Neopedilus* M. Abdullah, 1969 [syn. of *Pedilus* Fischer von Waldheim, 1820].

### Subfamily Pyrochroinae

Latreille, 1806

Pyrochroides Latreille, 1806: 199 [stem: *Pyrochro-*]. Type genus: *Pyrochroa* Geoffroy, 1762 [placed on the Official List of Generic Names in Zoology (ICZN 1994a)].
Anthomanisidae Gistel, 1848: [11] [stem: *Anthoman-*]. Type genus: *Anthomanes* Gistel, 1848 [syn. of *Pyrochroa* Geoffroy, 1762]. Comment: incorrect original stem formation, not in prevailing usage.

### Subfamily Agnathinae

Lacordaire, 1859

Agnathides Lacordaire, 1859: 531 [stem: *Agnath-*]. Type genus: *Agnathus* Germar, 1818. Comment: original vernacular name available (Art. 11.7.2): first used in latinized form by Seidlitz (1875 [Gatt.]: 100, as Agnathini), generally accepted as in Young (2002: 542, as Agnathinae).
Cononotini J. L. LeConte, 1862: 256 [stem: *Cononot-*]. Type genus: *Cononotus* J. L. LeConte, 1851.

### Family Salpingidae

Leach, 1815

Salpingides Leach, 1815: 106 [stem: *Salping-*]. Type genus: *Salpingus* Illiger, 1802.

### Subfamily Othniinae

LeConte, 1861

Othniidae J. L. LeConte, 1861: 102 [stem: *Othni-*]. Type genus: *Othnius* J. L. LeConte, 1861 [syn. of *Elacatis* Pascoe, 1860].
Elacatidae Cockerell, 1906: 242 [stem: *Elacat-*]. Type genus: *Elacatis* Pascoe, 1860. Comment: junior homonym of Elacatinae Gill, 1861 (type genus *Elacate* Cuvier, 1831) proposed in Pisces; this case is to be referred to the Commission to remove the homonymy (Art. 55.3.1).

### Subfamily Prostominiinae

Grouvelle, 1914

Prostominini Grouvelle, 1914: 152 [stem: *Prostomini-*]. Type genus: *Prostominia* Reitter, 1889. Comment: incorrect original stem formation, not in prevailing usage.
*Trogocryptinae Crowson, 1953: 51 [stem: *Trogocrypt-*]. Type genus: *Trogocryptus* Sharp, 1900. Comment: unavailable family-group name, proposed after 1930 without description or bibliographic reference to such a description (Art. 13.1); this name has been used subsequently, e.g., Lawrence (1977: 43, 1980: 307), Lawrence and Newton (1995: 900), but it has not been made available.

### Subfamily Agleninae

Horn, 1878

Agleni G. H. Horn, 1878: 573 [stem: *Aglen-*]. Type genus: *Aglenus* Erichson, 1845.

### Subfamily Inopeplinae

Grouvelle, 1908

Inopeplini Grouvelle, 1908: 459, in key [stem: *Inopepl-*]. Type genus: *Inopeplus* F. Smith, 1851.

### Subfamily Dacoderinae

LeConte, 1862

Dacoderini J. L. LeConte, 1862: 216 [stem: *Dacoder-*]. Type genus: *Dacoderus* J. L. LeConte, 1858.
Tretothoracidae Lea, 1910: 210 [stem: *Tretothorac-*]. Type genus: *Tretothorax* Lea, 1910.

### Subfamily Aegialitinae

LeConte, 1862

Aegialitidae J. L. LeConte, 1862: 241 [stem: *Aegialit-*]. Type genus: *Aegialites* Mannerheim, 1853.
Eurystethidae Seidlitz, 1916: 127 [stem: *Eurysteth-*]. Type genus: *Eurystethes* Seidlitz, 1916 [syn. of *Aegialites* Mannerheim, 1853].

### Subfamily Salpinginae

Leach, 1815

Salpingides Leach, 1815: 106 [stem: *Salping-*]. Type genus: *Salpingus* Illiger, 1802.
*Rhinosimites Solier, 1834: 496 [stem: *Rhinosim-*]. Type genus: *Rhinosimus* Latreille, 1802. Comment: original vernacular name unavailable (Art. 11.7.2): subsequently used in latinized form but not generally attributed to Solier (1834).
Rhinosimidae Hope, 1840a: 134 [stem: *Rhinosim-*]. Type genus: *Rhinosimus* Latreille, 1802.
Lissodemina Seidlitz, 1917b: 422 [stem: *Lissodem-*]. Type genus: *Lissodema* Curtis, 1833.
Istrisiini Nikitsky, 1992: 483, in key [stem: *Istrisi-*]. Type genus: *Istrisia* Lewis, 1895.

### Family Anthicidae

Latreille, 1819

Anthicites Latreille, 1819: 363 [stem: *Anthic-*]. Type genus: *Anthicus* Paykull, 1798.

### Subfamily Eurygeniinae

LeConte, 1862

Eurygenii J. L. LeConte, 1862: 264 [stem: *Eurygeni-*]. Type genus: *Eurygenius* LaFerté-Sénectère, 1849.

### Tribe Eurygeniini

LeConte, 1862

Eurygenii J. L. LeConte, 1862: 264 [stem: *Eurygeni-*]. Type genus: *Eurygenius* LaFerté-Sénectère, 1849.

### Tribe Ictistygnini

Borchmann, 1936

Ictistygninae Borchmann, 1936: 534 [stem: *Ictistygn-*]. Type genus: *Ictistygna* Pascoe, 1866.

### Tribe Mitraelabrini

Abdullah, 1969

Mitraelabrini M. Abdullah, 1969a: 350 [stem: *Mitraelabr-*]. Type genus: *Mitraelabrus* Solier, 1851.

### Subfamily Macratriinae

LeConte, 1862

Macratriini J. L. LeConte, 1862: 265 [stem: *Macratri-*]. Type genus: *Macratria* Newman, 1838.

### Tribe Macratriini

LeConte, 1862

Macratriini J. L. LeConte, 1862: 265 [stem: *Macratri-*]. Type genus: *Macratria* Newman, 1838.

### †Tribe Camelomorphini

Kirejtshuk and Azar, 2008

Camelomorphini Kirejtshuk and Azar, 2008: 40 [stem: *Camelomorph-*]. Type genus: *Camelomorpha* Kirejtshuk and Azar, 2008.

### Subfamily Steropinae

Jacquelin du Val, 1863

Stéropites Jacquelin du Val, 1863: 365 [stem: *Sterop-*]. Type genus: *Steropes* Steven, 1806. Comment: original vernacular name available (Art. 11.7.2): first used in latinized form by Jakobson (1915: 1022, as Steropina), generally accepted as in Lawrence and Newton (1995: 901, as Steropinae); the older name Steropinae Dana, 1854 is available in copepods (type genus *Sterope* Goodsir, 1845) although it was recently treated as a *nomen oblitum* by Huys (2009: 28); this case is to be referred to the Commission to remove the homonymy (Art. 55.3.1).

### Subfamily Copobaeninae

Abdullah, 1969

Copobaeninae M. Abdullah, 1969a: 334 [stem: *Copobaen-*]. Type genus: *Copobaenus* Fairmaire and Germain, 1863.

### Subfamily Lemodinae

Lawrence and Britton, 1991

*Lemodinae Lawrence, 1977: 43 [stem: *Lemod-*]. Type genus: *Lemodes* Boheman, 1858. Comment: unavailable family-group name, proposed after 1930 without description or bibliographic reference to such a description (Art. 13.1).
Lemodinae Lawrence and Britton, 1991: 603, in key [stem: *Lemod-*]. Type genus: *Lemodes* Boheman, 1858.

### Subfamily Tomoderinae

Bonadona, 1961

Tomoderini Bonadona, 1961: 11 [stem: *Tomoder-*]. Type genus: *Tomoderus* LaFerté-Sénectère, 1849.

### Subfamily Anthicinae

Latreille, 1819

Anthicites Latreille, 1819: 363 [stem: *Anthic-*]. Type genus: *Anthicus* Paykull, 1798.

### Tribe Anthicini

Latreille, 1819

Anthicites Latreille, 1819: 363 [stem: *Anthic-*]. Type genus: *Anthicus* Paykull, 1798.

### Tribe Endomiini

Kaszab, 1956

Endomiini Kaszab, 1956: 45, in key [stem: *Endomi-*]. Type genus: *Endomia* Laporte, 1840.

### Tribe Formicomini

Bonadona, 1974

Formicomini Bonadona, 1974: 110, in key [stem: *Formicom-*]. Type genus: *Formicomus* LaFerté-Sénectère, 1849.

### Tribe Microhoriini

Bonadona, 1974

Microhorini Bonadona, 1974: 110, in key [stem: *Microhori-*]. Type genus: *Microhoria* Chevrolat, 1877. Comment: incorrect original stem formation, not in prevailing usage.
Liparoderini Bonadona, 1990: 20 [stem: *Liparoder-*]. Type genus: *Liparoderus* LaFerté-Sénectère, 1849.

### Subfamily Notoxinae

Stephens, 1829

Notoxidae Stephens, 1829b: 254 [stem: *Notox-*]. Type genus: *Notoxus* Geoffroy, 1762 [placed on the Official List of Generic Names in Zoology (ICZN 1994a)]. Comment: an application needs to be submitted to the Commission to suppress Notoxii Sturm, 1826 (based on the misidentified type genus *Notoxus* sensu Fabricius, 1775) for the Principles of Priority and Homonymy (Art. 65.2.1) to conserve this name as valid.

### Family Aderidae

Csiki, 1909

Aderidae Csiki, 1909a: 6 [stem: *Ader-*]. Type genus: *Aderus* Stephens, 1829 [placed on the Official List of Generic Names in Zoology (ICZN 1989e)]. Comment: name given precedence over Euglenesidae Seidlitz, 1875 (ICZN 1989e).
†Circaeidae Iablokoff-Khnzorian, 1961: 209 [stem: *Circae-*]. Type genus: *Circaeus* Iablokoff-Khnzorian, 1961.

### Tribe Aderini

Csiki, 1909

Aderidae Csiki, 1909a: 6 [stem: *Ader-*]. Type genus: *Aderus* Stephens, 1829 [placed on the Official List of Generic Names in Zoology (ICZN 1989e)]. Comment: name given precedence over Euglenesidae Seidlitz, 1875 and placed on the Official List of Family-Group Names in Zoology (ICZN 1989e, as Aderidae A. Winkler (1927)).

### Subtribe Aderina

Csiki, 1909

Xylophilidae Shuckard, 1839b: 47 [stem: *Xylophil-*]. Type genus: *Xylophilus* Latreille, 1825 [preoccupied genus name, not *Xylophilus* Mannerheim, 1823 [Coleoptera: Eucnemidae]; syn. of *Aderus* Stephen, 1829]. Comment: permanently invalid (Art. 39): based on preoccupied type genus.
Hylophilidae Pic, 1900: 754 [stem: *Hylophil-*]. Type genus: *Hylophilus* Berthold, 1827 [preoccupied genus name, not *Hylophilus* Temminck, 1822 [Aves]; syn. of *Aderus* Stephens, 1829]. Comment: permanently invalid (Art. 39): based on preoccupied type genus.
Aderidae Csiki, 1909a: 6 [stem: *Ader-*]. Type genus: *Aderus* Stephens, 1829 [placed on the Official List of Generic Names in Zoology (ICZN 1989e)]. Comment: placed on the Official List of Family-Group Names in Zoology (ICZN 1989e, as Aderidae A. Winkler (1927)).

### Subtribe Cnopina

Báguena Corella, 1948

Cnopina Báguena Corella, 1948: 57 [stem: *Cnop-*]. Type genus: *Cnopus* Champion, 1893.

### Subtribe Gompeliina

Bouchard, 2011

Olotelina Báguena Corella, 1948: 58 [stem: *Olotel-*]. Type genus: *Olotelus* Mulsant and Rey, 1866 [preoccupied genus name, not *Olotelus* Solier, 1851 [Coleoptera: Elateridae]; syn. of *Gompelia* Alonso-Zarazaga, 2010]. Comment: permanently invalid (Art. 39): based on preoccupied type genus.
Gompeliina Bouchard, 2011 **nom. nov.** [stem: *Gompeli-*]. Type genus: *Gompelia* Alonso-Zarazaga, 2010. Comment: replacement name for Olotelina Báguena Corella, 1948 because of the homonymy of the type genus.

### Subtribe Syzetoninina

Báguena Corella, 1948

Syzetoninini Báguena Corella, 1948: 41 [stem: *Syzetonin-*]. Type genus: *Syzetoninus* Blackburn, 1891.

### Tribe Emelinini

Báguena Corella, 1948

Emelinini Báguena Corella, 1948: 36 [stem: *Emelin-*]. Type genus: *Emelinus* Casey, 1895.

### Tribe Euglenesini

Seidlitz, 1875

Euglenini Seidlitz, 1875 [Gatt.]: 106 [stem: *Euglenes-*]. Type genus: *Euglenes* Westwood, 1830 [placed on the Official List of Generic Names in Zoology (ICZN 1989e)].

### Subtribe Euglenesina

Seidlitz, 1875

Euglenini Seidlitz, 1875 [Gatt.]: 106 [stem: *Euglenes-*]. Type genus: *Euglenes* Westwood, 1830 [placed on the Official List of Generic Names in Zoology (ICZN 1989e)]. Comment: the younger name Aderidae Csiki, 1909 (listed as Aderidae A. Winkler, 1927) was given precedence for the family name, *Euglenes*- was ruled to be the correct stem of this family-group name and Euglenesidae Seidlitz, 1875 was placed on the Official List of Family-Group Names in Zoology (ICZN 1989e); Euglenidae Seidlitz, 1875 was placed on the Official Index of Rejected and Invalid Family-Group Names in Zoology (ICZN 1989e).

### Subtribe Pseudolotelina

Báguena Corella, 1948

Pseudolotelina Báguena Corella, 1948: 40 [stem: *Pseudolotel-*]. Type genus: *Pseudolotelus* Pic, 1901.

### Tribe Phytobaenini

Báguena Corella, 1948

Phytobaenini Báguena Corella, 1948: 30 [stem: *Phytobaen-*]. Type genus: *Phytobaenus* Sahlberg, 1834.

### Family Scraptiidae

Gistel, 1848

Scraptiaeidae Gistel, 1848: [11] [stem: *Scrapti-*]. Type genus: *Scraptia* Latreille, 1807.

### Subfamily Scraptiinae

Gistel, 1848

Scraptiaeidae Gistel, 1848: [11] [stem: *Scrapti-*]. Type genus: *Scraptia* Latreille, 1807. Comment: incorrect original stem formation, not in prevailing usage.

### Tribe Allopodini

Franciscolo, 1964

Allopodini Franciscolo, 1964: 176 [stem: *Allopod-*]. Type genus: *Allopoda* J. L. LeConte, 1866.

### Tribe Scraptiini

Gistel, 1848

Scraptiaeidae Gistel, 1848: [11] [stem: *Scrapti-*]. Type genus: *Scraptia* Latreille, 1807. Comment: family-group name previously attributed to Mulsant (1856)/Gistel (1856); incorrect original stem formation, not in prevailing usage.

### Subfamily Anaspidinae

Mulsant, 1856

Anaspiens Mulsant, 1856c: 85 [stem: *Anaspid-*]. Type genus: *Anaspis* Geoffroy, 1762 [placed on the Official List of Generic Names in Zoology (ICZN 1984a)].

### Tribe Anaspidini

Mulsant, 1856

Anaspiens Mulsant, 1856c: 85 [stem: *Anaspid-*]. Type genus: *Anaspis* Geoffroy, 1762 [placed on the Official List of Generic Names in Zoology (ICZN 1984a)]. Comment: original vernacular name available (Art. 11.7.2): first used in latinized form by C. G. Thomson (1859: 121, as Anaspina [incorrect stem formation]), generally accepted as in Lawrence and Newton (1995: 903, as Anaspidinae); incorrect original stem formation, not in prevailing usage.

### Tribe Anaspimordini

Franciscolo, 1954

Anaspimordini Franciscolo, 1954: 65, in key [stem: *Anaspimord-*]. Type genus: *Anaspimorda* Ermisch, 1950.

### Tribe Menuthianaspidini

Franciscolo, 1972

*Cyrtoscraptiini Franciscolo, 1964: 176 [stem: *Cyrtoscrapti-*]. Type genus: *Cyrtoscraptia* Franciscolo, 1964 [unavailable genus name, proposed after 1930 without description or bibliographic reference to such a description (Art. 13.1); syn. of *Menuthianaspis* Franciscolo, 1972]. Comment: unavailable family-group name, proposed after 1930 without description or bibliographic reference to such a description (Art. 13.1), based on unavailable type genus (Art. 12.2.4).
Menuthianaspidini Franciscolo, 1972: 123 [stem: *Menuthianaspid-*]. Type genus: *Menuthianaspis* Franciscolo, 1972.

### Tribe Pentariini

Franciscolo, 1954

Pentariini Franciscolo, 1954: 64, in key [stem: *Pentari-*]. Type genus: *Pentaria* Mulsant, 1856.

### Subfamily Lagrioidinae

Abdullah and Abdullah, 1968

Lagrioidini M. Abdullah and A. Abdullah, 1968: 73 [stem: *Lagrioid-*]. Type genus: *Lagrioida* Fairmaire and Germain, 1860.

### Subfamily Afreminae

Levey, 1985

Afreminae Levey, 1985: 420 [stem: *Afrem-*]. Type genus: *Afremus* Levey, 1985.

### Subfamily Ischaliinae

Blair, 1920

Ischaliinae Blair, 1920: 134 [stem: *Ischali-*]. Type genus: *Ischalia* Pascoe, 1860.

### Superfamily Chrysomeloidea

Latreille, 1802

Chrysomelinae Latreille, 1802: 220 [stem: *Chrysomel-*]. Type genus: *Chrysomela* Linnaeus, 1758 [placed on the Official List of Generic Names in Zoology (ICZN 1984c)]. Comment: Švácha and Danilevsky (1987: 17) recognized Vesperinae, Oxypeltinae and Disteniinae at the family level, and later Švácha et al. (1997: 361) placed Anoplodermatinae and Philinae as subfamilies of Vesperidae; although the classification is based primarily on larval characters, it is also supported by adult features, as pointed out by Švácha et al. (1997), as well as by some earlier workers, e.g., Gahan (1906), Linsley (1961), Crowson (1981), Saito (1990); this classification will also be used in the upcoming volume 3 of the “Handbook of Zoology: Coleoptera, beetles” series; First Reviser (Chrysomeloidea Latreille, 1802 vs Megalopodoidea Latreille, 1802 vs Cerambycoidea Latreille, 1802) not determined, current usage maintained.

### Family Oxypeltidae

Lacordaire, 1868

Oxypeltides Lacordaire, 1868: 461 [stem: *Oxypelt-*]. Type genus: *Oxypeltus* Blanchard, 1851. Comment: original vernacular name available (Art. 11.7.2): first used in latinized form and generally accepted as in Aurivillius (1912: 254, as Oxypeltini).
*Cheloderidos Germain, 1900: 86 [stem: *Cheloder-*]. Type genus: *Cheloderus* Gray, 1832. Comment: original vernacular name unavailable (Art. 11.7.2): proposed after 1899.

### Family Vesperidae

Mulsant, 1839

Vespéraires Mulsant, 1839: 214 [stem: *Vesper-*]. Type genus: *Vesperus* Dejean, 1821.

### Subfamily Philinae

Thomson, 1861

Philitae J. Thomson, 1861: 297 [stem: *Phil-*]. Type genus: *Philus* Saunders, 1853.

### Subfamily Vesperinae

Mulsant, 1839

Vespéraires Mulsant, 1839: 214 [stem: *Vesper-*]. Type genus: *Vesperus* Dejean, 1821. Comment: original vernacular name available (Art. 11.7.2): first used in latinized form by Heyne and Taschenberg (1907: 240, as Vesperini), generally accepted as in Villiers (1978: 67, as Vesperini).

### Subfamily Anoplodermatinae

Guérin-Méneville, 1840

Anoplodermiens Guérin-Méneville, 1840: 276 [stem: *Anoplodermat-*]. Type genus: *Anoploderma* Guérin-Méneville, 1840.

### Tribe Anoplodermatini

Guérin-Méneville, 1840

Anoplodermiens Guérin-Méneville, 1840: 276 [stem: *Anoplodermat-*]. Type genus: *Anoploderma* Guérin-Méneville, 1840. Comment: original vernacular name available (Art. 11.7.2): first used in latinized form by J. Thomson (1861: 277, as Anoplodermitae [incorrect stem formation]), generally accepted as in Monné (1994c: 9, as Anoplodermatinae); incorrect original stem formation, not in prevailing usage.
Cherrocriinae Prosen, 1960: 90 [stem: *Cherrocri-*]. Type genus: *Cherrocrius* Berg, 1898.

### Tribe Hypocephalini

Blanchard, 1845

Hypocéphaliens Blanchard, 1845b: 135 [stem: *Hypocephal-*]. Type genus: *Hypocephalus* Desmarest, 1832. Comment: original vernacular name available (Art. 11.7.2): first used in latinized form by Imhoff (1856 [2]: 170, as Hypocephalidae), generally accepted as in Lameere (1913: 94, as Hypocephali).

### Tribe Mysteriini

Prosen, 1960

Mysterinae Prosen, 1960: 90 [stem: *Mysteri-*]. Type genus: *Mysteria* J. Thomson, 1860. Comment: incorrect original stem formation, not in prevailing usage.

### Family Disteniidae

Thomson, 1861

Distenitae J. Thomson, 1861: 181 [stem: *Disteni-*]. Type genus: *Distenia* Lepeletier and Audinet-Serville, 1828.

### Tribe Cyrtonopini

Gressitt, 1940

Cyrtonopini Gressitt, 1940: 27 [stem: *Cyrtonop-*]. Type genus: *Cyrtonops* A. White, 1853.

### Tribe Disteniini

Thomson, 1861

*Cométites Blanchard, 1845b: 163 [stem: *Comet-*]. Type genus: *Cometes* Lepeletier and Audinet-Serville, 1828. Comment: original vernacular name unavailable (Art. 11.7.2): not subsequently latinized; Monné and Santos-Silva (2008: 261) considered this name a *nomen oblitum*, this action was unnecessary since Cométites Blanchard is not an available name.
Distenitae J. Thomson, 1861: 181 [stem: *Disteni-*]. Type genus: *Distenia* Lepeletier and Audinet-Serville, 1828. Comment: incorrect original stem formation, not in prevailing usage.

### Tribe Dynamostini

Lacordaire, 1868

Dynamostides Lacordaire, 1868: 196 [stem: *Dynamost-*]. Type genus: *Dynamostes* Pascoe, 1857. Comment: original vernacular name available (Art. 11.7.2): first used in latinized form by Heyne and Taschenberg (1907: 238, as Dynamostini), generally accepted as in Santos-Silva and Martins (2004: 145, as Dynamostini).

### Tribe Heteropalpini

Villiers, 1961

Heteropalpini Villiers, 1961: 385, in key [stem: *Heteropalp-*]. Type genus: *Heteropalpus* Buquet, 1843.

### Family Cerambycidae

Latreille, 1802

Cerambicini Latreille, 1802: 211 [stem: *Cerambyc-*]. Type genus: *Cerambyx* Linnaeus, 1758. Comment: First Reviser (Cerambycidae Latreille, 1802 vs Prionidae Latreille, 1802 vs Lepturidae Latreille, 1802) not determined, current usage maintained.

### Subfamily Parandrinae

Blanchard, 1845

Parandrides Blanchard, 1845b: 134 [stem: *Parandr-*]. Type genus: *Parandra* Latreille, 1802.

### Tribe Erichsoniini

Thomson, 1861

Erichsonitae J. Thomson, 1861: 274 [stem: *Erichsoni-*]. Type genus: *Erichsonia* Westwood, 1849. Comment: incorrect original stem formation, not in prevailing usage.

### Tribe Parandrini

Blanchard, 1845

Parandrides Blanchard, 1845b: 134 [stem: *Parandr-*]. Type genus: *Parandra* Latreille, 1802. Comment: original vernacular name available (Art. 11.7.2): first used in latinized form by Gistel (1848: [8], as Parandraeidae [incorrect stem formation]), generally accepted as in Lameere (1913: 4, as Parandrae).

### Subfamily Prioninae

Latreille, 1802

Prionii Latreille, 1802: 212 [stem: *Prion-*]. Type genus: *Prionus* Geoffroy, 1762 [placed on the Official List of Generic Names in Zoology (ICZN 1994a)].

### Tribe Acanthophorini

Thomson, 1864

Acanthophoritae J. Thomson, 1864: 289 [stem: *Acanthophor-*]. Type genus: *Acanthophorus* Audinet-Serville, 1832.

### Tribe Aegosomatini

Thomson, 1861

Aegosomitae J. Thomson, 1861: 308 [stem: *Aegosomat-*]. Type genus: *Aegosoma* Audinet-Serville, 1832. Comment: incorrect original stem formation, not in prevailing usage.
*Catypnides Lacordaire, 1868: 62 [stem: *Catypn-*]. Type genus: *Catypnes* Pascoe, 1864. Comment: original vernacular name unavailable (Art. 11.7.2): not subsequently latinized.
Jamwoninae Kolbe, 1897: 294 [stem: *Jamwon-*]. Type genus: *Jamwonus* Harold, 1879.
Megopides Lameere, 1912: 181 [stem: *Megopid-*]. Type genus: *Megopis* Audinet-Serville, 1832. Comment: incorrect original stem formation, not in prevailing usage.

### Tribe Anacolini

Thomson, 1857

Anacolites J. Thomson, 1857b: 10 [stem: *Anacol-*]. Type genus: *Anacolus* Berthold, 1827. Comment: original vernacular name available (Art. 11.7.2): first used in latinized form by J. Thomson (1861: 302, as Anacolitae), generally accepted as in Galileo (1987: 482, as Anacolini).
*Poecilosomides Lacordaire, 1868: 185 [stem: *Poekilosomat-*]. Type genus: *Poekilosoma* Audinet-Serville, 1832 [as *Poecilosoma*, unjustified emendation of type genus name by Agassiz (1846b: 301), not in prevailing usage; unjustified emendation also junior homonym of *Poecilosoma* Hübner, 1819]. Comment: original vernacular name unavailable (Art. 11.7.2): subsequently used in latinized form but not generally attributed to Lacordaire (1868); incorrect original stem formation, not in prevailing usage.
Poecilosomi H. W. Bates, 1869: 49 [stem: *Poekilosomat-*]. Type genus: *Poekilosoma* Audinet-Serville, 1832 [as *Poecilosoma*, unjustified emendation of type genus name by Agassiz (1846b: 301), not in prevailing usage; unjustified emendation also junior homonym of *Poecilosoma* Hübner, 1819]. Comment: incorrect original stem formation, not in prevailing usage.
Erythraeninae H. W. Bates, 1875: 52 [stem: *Erythraen-*]. Type genus: *Erythraenus* H. W. Bates, 1875.
*Sobarines Lameere, 1901: 320 [stem: *Sobar-*]. Type genus: *Sobarus* Harold, 1879 [preoccupied genus name, not *Sobarus* Loew, 1855 [Diptera]; a replacement name is needed]. Comment: original vernacular name unavailable (Art. 11.7.2): proposed after 1899.
*Délochiliens Lameere, 1912: 57 [stem: *Delocheil-*]. Type genus: *Delocheilus* J. Thomson, 1860 [as *Delochilus*, unjustified emendation of type genus name by Gemminger (1872: 2777), not in prevailing usage]. Comment: original vernacular name unavailable (Art. 11.7.2): proposed after 1899.
Sobari Lameere, 1913: 85 [stem: *Sobar-*]. Type genus: *Sobarus* Harold, 1879 [preoccupied genus name, not *Sobarus* Loew, 1855 [Diptera]; a replacement name is needed]. Comment: permanently invalid (Art. 39): based on preoccupied type genus.
Delochili Lameere, 1913: 85 [stem: *Delocheil-*]. Type genus: *Delocheilus* J. Thomson, 1860 [as *Delochilus*, unjustified emendation of type genus name by Gemminger and Harold (1872), not in prevailing usage]. Comment: incorrect original stem formation, not in prevailing usage.

### Tribe Cacoscelini

Thomson, 1861

*Notophysites Blanchard, 1845b: 138 [stem: *Notophyse-*]. Type genus: *Notophysis* Audinet-Serville, 1832. Comment: original vernacular name unavailable (Art. 11.7.2): subsequently used in latinized form but not generally attributed to Blanchard (1845b); incorrect original stem formation, not in prevailing usage.
Cacoscelitae J. Thomson, 1861: 325 [stem: *Cacoscel-*]. Type genus: *Cacosceles* Newman, 1838.
Colpodérides Lacordaire, 1868: 133 [stem: *Colpoder-*]. Type genus: *Colpoderus* Audinet-Serville, 1832 [syn. of *Notophysis* Audinet-Serville, 1832]. Comment: original vernacular name available (Art. 11.7.2): first used in latinized form and generally accepted as in Pascoe (1869b: 673, as Colpoderinae); this name was incorrectly credited to Pascoe, 1869 by Bousquet et al. (2009: 115).
Nothophysini Lameere, 1903a: 4, in key [stem: *Notophyse-*]. Type genus: *Notophysis* Audinet-Serville, 1832 [as *Nothophysis*, unjustified emendation of type genus name by Scudder (1882: 226), not in prevailing usage]. Comment: incorrect original stem formation, not in prevailing usage.

### Tribe Callipogonini

Thomson, 1861

Callipogonitae J. Thomson, 1861: 323 [stem: *Callipogon-*]. Type genus: *Callipogon* Audinet-Serville, 1832.
Anacanthitae J. Thomson, 1864: 285 [stem: *Anacanth-*]. Type genus: *Anacanthus* Audinet-Serville, 1832 [preoccupied genus name, not *Anacanthus* Gray, 1831 [Pisces]; syn. of *Chorenta* Gistel, 1848]. Comment: permanently invalid (Art. 39): based on preoccupied type genus.
Enoploceritae J. Thomson, 1864: 290 [stem: *Enoplocer-*]. Type genus: *Enoplocerus* Audinet-Serville, 1832.
Orthomegitae J. Thomson, 1864: 294 [stem: *Orthomegal-*]. Type genus: *Orthomegas* Audinet-Serville, 1832. Comment: incorrect original stem formation, not in prevailing usage.
Ctenoscelitae J. Thomson, 1864: 295 [stem: *Ctenoscelid-*]. Type genus: *Ctenoscelis* Audinet-Serville, 1832. Comment: incorrect original stem formation, not in prevailing usage.

### Tribe Calocomini

Galileo and Martins, 1993

Calocomini Galileo and Martins, 1993: 81 [stem: *Calocom-*]. Type genus: *Calocomus* Audinet-Serville, 1832.

### Tribe Cantharocnemini

Thomson, 1861

Cantharocnemitae J. Thomson, 1861: 274 [stem: *Cantharocnem-*]. Type genus: *Cantharocnemis* Audinet-Serville, 1832. Comment: current spelling maintained (Art. 29.3.1.1): incorrect original stem formation in prevailing usage (should be *Cantharocnemid*-).
*Scéléocanthides Lacordaire, 1868: 34 [stem: *Sceleocanth-*]. Type genus: *Sceleocantha* Newman, 1840. Comment: original vernacular name unavailable (Art. 11.7.2): subsequently used in latinized form, e.g., Heyne and Taschenberg (1907: 236, as Sceleocanthini), but not generally accepted as valid.

### Tribe Ergatini

Fairmaire, 1864

Ergatites Fairmaire, 1864: 117 [stem: *Ergat-*]. Type genus: *Ergates* Audinet-Serville, 1832. Comment: original vernacular name available (Art. 11.7.2): first used in latinized form by J. L. LeConte (1873: 286, as Ergatini), generally accepted as in Linsley (1962: 24, as Ergatini).

### Tribe Eurypodini

Gahan, 1906 (1868)

Zaracides Lacordaire, 1868: 131 [stem: *Zarac-*]. Type genus: *Zarax* Pascoe, 1867 [syn. of *Eurypoda* W. Saunders, 1853]. Comment: original vernacular name available (Art. 11.7.2): first used in latinized form and generally accepted as in Pascoe (1869b: 672, as Zaracinae); usage of the younger name Eurypodini Gahan, 1906 conserved over this name (Art. 40.2).
Eurypodini Gahan, 1906: 27 [stem: *Eurypod-*]. Type genus: *Eurypoda* W. Saunders, 1853. Comment: name proposed to replace Zaracini Lacordaire, 1868 because of the synonymy of the type genus; use of family-group name conserved over Zaracini Lacordaire, 1868 (Art. 40.2); also see comments under Eurypinae Thomson, 1860 in Mycteridae.

### Tribe Hopliderini

Thomson, 1864

Hoplideritae J. Thomson, 1864: 290 [stem: *Hoplider-*]. Type genus: *Hoplideres* Audinet-Serville, 1832.

### Tribe Macrodontiini

Thomson, 1861

Macrodontitae J. Thomson, 1861: 324 [stem: *Macrodonti-*]. Type genus: *Macrodontia* Lacordaire, 1830. Comment: incorrect original stem formation, not in prevailing usage.
Acanthinoderitae J. Thomson, 1864: 294 [stem: *Acanthinoder-*]. Type genus: *Acanthinodera* Hope, 1833.
*Ancistrotides Lacordaire, 1868: 81 [stem: *Ancistrot-*]. Type genus: *Ancistrotus* Audinet-Serville, 1832. Comment: original vernacular name unavailable (Art. 11.7.2): subsequently used in latinized form but not generally attributed to Lacordaire (1868).
*Acalodegmites J. Thomson, 1877: 261 [stem: *Acalodegm-*]. Type genus: *Acalodegma* Thomson, 1877. Comment: original vernacular name unavailable (Art. 11.7.2): not subsequently latinized.
Ancistrotini Lameere, 1919: 90 [stem: *Ancistrot-*]. Type genus: *Ancistrotus* Audinet-Serville, 1832.

### Tribe Macrotomini

Thomson, 1861

Macrotomitae J. Thomson, 1861: 312 [stem: *Macrotom-*]. Type genus: *Macrotoma* Audinet-Serville, 1832.

### Subtribe Archetypina

Lameere, 1912

Archetypi Lameere, 1912: 180 [stem: *Archetyp-*]. Type genus: *Archetypus* J. Thomson, 1861.

### Subtribe Basitoxina

Lameere, 1912

*Mécosarthrines Lameere, 1903b: 307 [stem: *Mecosarthr-*]. Type genus: *Mecosarthron* Buquet, 1840. Comment: original vernacular name unavailable (Art. 11.7.2): proposed after 1899.
Basitoxi Lameere, 1912: 180 [stem: *Basitox-*]. Type genus: *Basitoxus* Audinet-Serville, 1832.
Mecosarthrini Melzer, 1919: 35 [stem: *Mecosarthr-*]. Type genus: *Mecosarthron* Buquet, 1840.

### Subtribe Macrotomina

Thomson, 1861

Macrotomitae J. Thomson, 1861: 312 [stem: *Macrotom-*]. Type genus: *Macrotoma* Audinet-Serville, 1832 [*Macrotoma* Audinet-Serville, 1832 [July] is a junior homonym of *Macrotoma* Laporte, 1832 [April], which is a synonym of *Longina* Wiedemann, 1830 in Diptera; Heffern et al. (2006) applied the Reversal of Precedence (Art. 23.9) to qualify *Macrotoma* Audinet-Serville as *nomen protectum*].
*Aulacopides Lacordaire, 1868: 101 [stem: *Aulacopod-*]. Type genus: *Aulacopus* Audinet-Serville, 1832. Comment: original vernacular name unavailable (Art. 11.7.2): subsequently used in latinized form but not generally attributed to Lacordaire (1868); incorrect original stem formation, not in prevailing usage.
Aulacopinae Kolbe, 1897: 295 [stem: *Aulacopod-*]. Type genus: *Aulacopus* Audinet-Serville, 1832. Comment: incorrect original stem formation, not in prevailing usage.
*Cnémoplitiens Lameere, 1904: 1 [stem: *Cnemoplit-*]. Type genus: *Cnemoplites* Newman, 1842. Comment: original vernacular name unavailable (Art. 11.7.2): proposed after 1899.
Cnemoplitinae Schröder, 1905: vii [stem: *Cnemoplit-*]. Type genus: *Cnemoplites* Newman, 1842. Comment: see Bousquet et al. (2009: 17) for comments about the authorship of this name.
Prinobiini Vives, 2000: 84 [stem: *Prinobi-*]. Type genus: *Prinobius* Mulsant, 1842. Comment: replacement name for Macrotomini Thomson, 1861 because of the homonymy of the type genus.

### Subtribe Platygnathina

Gilmour, 1954

Platygnathina Gilmour, 1954: 33 [stem: *Platygnath-*]. Type genus: *Platygnathus* Audinet-Serville, 1832. Comment: as pointed out by Bousquet et al. (2009: 18) Gilmour (1954: 33) did not provide a description of his new taxon but his new name is available because it was used as valid before 2000, e.g., Ferreira and Veiga Ferreira (1959a: 34, as "Platygnathina Gilmour, 1954") and was not rejected by an author who, after 1960 and before 2000, expressly applied Article 13 of the then current editions of the Code (Article 13.2.1).
Platygnathini Quentin and Villiers, 1975: 25 [stem: *Platygnath-*]. Type genus: *Platygnathus* Audinet-Serville, 1832. Comment: family-group name proposed as new without reference to Platygnathina Gilmour, 1954.

### Subtribe Xixuthrina

Lameere, 1912

Xixuthri Lameere, 1912: 181 [stem: *Xixuthr-*]. Type genus: *Xixuthrus* J. Thomson, 1864.

### Tribe Mallaspini

Thomson, 1861

Mallaspitae J. Thomson, 1861: 302 [stem: *Mallasp-*]. Type genus: *Mallaspis* Audinet-Serville, 1832. Comment: current spelling maintained (Art. 29.3.1.1): incorrect original stem formation in prevailing usage (should be *Mallaspid*-).
*Pyrodides Lacordaire, 1868: 174 [stem: *Pyrod-*]. Type genus: *Pyrodes* Audinet-Serville, 1832. Comment: original vernacular name unavailable (Art. 11.7.2): subsequently used in latinized form but not generally attributed to Lacordaire (1868).
Pyrodini Harold, 1879: 165 [stem: *Pyrod-*]. Type genus: *Pyrodes* Audinet-Serville, 1832.

### Tribe Mallodonini

Thomson, 1861

Mallodonitae J. Thomson, 1861: 318 [stem: *Mallodon-*]. Type genus: *Mallodon* Lacordaire, 1830. Comment: current spelling maintained (Art. 29.5): incorrect original stem formation in prevailing usage (should be *Mallodont*-); we treat this group as a valid tribe as in Drumont and Komiya (2010: 91).
*Sténodontines Lameere, 1902: 66 [stem: *Stenodont-*]. Type genus: *Stenodontes* Audinet-Serville, 1832. Comment: original vernacular name unavailable (Art. 11.7.2): proposed after 1899.
Stenodontini Lameere, 1903a: 54 [stem: *Stenodont-*]. Type genus: *Stenodontes* Audinet-Serville, 1832. Comment: homonym of Stenodontina Schmiedeknecht, 1903 (type genus *Stenodontus* Berthoumieu, 1897) in Hymenoptera; this case is to be referred to the Commission to remove the homonymy (Art. 55.3.1).

### Tribe Meroscelisini

Thomson, 1861

Meroscelisitae J. Thomson, 1861: 299 [stem: *Meroscelis-*]. Type genus: *Meroscelisus* Audinet-Serville, 1832.
*Tragosomites Fairmaire, 1864: 119 [stem: *Tragosomat-*]. Type genus: *Tragosoma* Audinet-Serville, 1832. Comment: original vernacular name unavailable (Art. 11.7.2): subsequently used in latinized form but not generally attributed to Fairmaire (1864); incorrect original stem formation, not in prevailing usage.
Tragosomitae J. Thomson, 1864: 286 [stem: *Tragosomat-*]. Type genus: *Tragosoma* Audinet-Serville, 1832. Comment: incorrect original stem formation, not in prevailing usage.
Clostérides Lacordaire, 1868: 149 [stem: *Closter-*]. Type genus: *Closterus* Audinet-Serville, 1832. Comment: original vernacular name available (Art. 11.7.2): first used in latinized form by Pascoe (1869b: 676, as Closterinae), generally accepted as in Lameere (1913: 81, as Closteri).
*Monodesmides Lacordaire, 1868: 157 [stem: *Monodesm-*]. Type genus: *Monodesmus* Audinet-Serville, 1832. Comment: original vernacular name unavailable (Art. 11.7.2): subsequently used in latinized form but not generally attributed to Lacordaire (1868).
Monodesminae Gahan, 1890: 299 [stem: *Monodesm-*]. Type genus: *Monodesmus* Audinet-Serville, 1832.
Luluina Gilmour, 1956: 222 [stem: *Lulu-*]. Type genus: *Lulua* Burgeon, 1931.

### Tribe Prionini

Latreille, 1802

Prionii Latreille, 1802: 212 [stem: *Prion-*]. Type genus: *Prionus* Geoffroy, 1762 [placed on the Official List of Generic Names in Zoology (ICZN 1994a)].
*Prioceria Rafinesque, 1815: 116 [stem: *Priocerat-*]. Type genus: *Prioceras* Rafinesque, 1815 [as pointed out by Bousquet et al. (2009: 19) Rafinesque (1815: 116) listed the genus “*Prioceras* R. sp. do.” following the genus “*Prionus* Fabr.” in his subfamily Prioceria, the abbreviations after the genus name *Prioceras* mean that this is a new genus by the author “R[afinesque].” and that the new genus includes some species that were included in *Prionus* previously “sp. do.”; Rafinesque did not list which species he included in his new genus *Prioceras* and we are not aware of any subsequent validation of this name; *Prioceras* Rafinesque is also listed as a *nomen nudum* in Neave (1940: 889) and Sherborn (1929: 5148)]. Comment: family-group name unavailable (Art. 11.7.1.1): not based on an available genus name at the time; incorrect original stem formation, not in prevailing usage.
*Cyrtognathites Blanchard, 1845b: 138 [stem: *Cyrtognath-*]. Type genus: *Cyrtognathus* Faldermann, 1835 [subgenus of *Dorysthenes* Vigors, 1826]. Comment: original vernacular name unavailable (Art. 11.7.2): subsequently used in latinized form but not generally attributed to Blanchard (1845b).
*Psalidognathites Blanchard, 1845b: 138 [stem: *Psalidognath-*]. Type genus: *Psalidognathus* Gray, 1832. Comment: original vernacular name unavailable (Art. 11.7.2): subsequently used in latinized form but not generally attributed to Blanchard (1845b).
Cyrthognathitae J. Thomson, 1861: 328 [stem: *Cyrtognath-*]. Type genus: *Cyrtognathus* Faldermann, 1835 [as *Cyrthognathus*, incorrect subsequent spelling of type genus name, not in prevailing usage; subgenus of *Dorysthenes* Vigors, 1826]. Comment: incorrect original stem formation, not in prevailing usage.
Psalidognathitae J. Thomson, 1861: 331 [stem: *Psalidognath-*]. Type genus: *Psalidognathus* Gray, 1832.
Prionommitae J. Thomson, 1861: 327 [stem: *Prionommat-*]. Type genus: *Prionomma* A. White, 1853. Comment: incorrect original stem formation, not in prevailing usage.
Orthosomitae J. Thomson, 1864: 284 [stem: *Orthosomat-*]. Type genus: *Orthosoma* Audinet-Serville, 1832. Comment: incorrect original stem formation, not in prevailing usage.
Pithoclitae J. Thomson, 1864: 291 [stem: *Pithocl-*]. Type genus: *Pithocles* J. Thomson, 1864 [syn. of *Derobrachus* Audinet-Serville, 1832].
Derobrachitae J. Thomson, 1864: 291 [stem: *Derobrach-*]. Type genus: *Derobrachus* Audinet-Serville, 1832.
Titanitae J. Thomson, 1864: 292 [stem: *Titan-*]. Type genus: *Titanus* Audinet-Serville, 1832.
Aulacoceritae J. Thomson, 1864: 292 [stem: *Aulacocer-*]. Type genus: *Aulacocerus* A. White, 1853.
*Psalidocoptides Lacordaire, 1868: 38 [stem: *Psalidocopt-*]. Type genus: *Psalidocoptus* A. White, 1856. Comment: original vernacular name unavailable (Art. 11.7.2): not subsequently latinized.
*Polyarthrides Lacordaire, 1868: 44 [stem: *Polyarthr-*]. Type genus: *Polyarthron* Audinet-Serville, 1832. Comment: original vernacular name unavailable (Art. 11.7.2): subsequently used in latinized form but not generally attributed to Lacordaire (1868).
*Micropsalides Lacordaire, 1868: 42 [stem: *Micropsalid-*]. Type genus: *Micropsalis* Burmeister, 1865 [preoccupied genus name, not *Micropsalis* Meyer, 1859 [Crustacea]; syn. of *Apterocaulus* Fairmaire, 1864]. Comment: original vernacular name unavailable (Art. 11.7.2): subsequently used in latinized form but not generally attributed to Lacordaire (1868); incorrect original stem formation, not in prevailing usage.
Polyarthrini Gounelle, 1911: 326 [stem: *Polyarthr-*]. Type genus: *Polyarthron* Audinet-Serville, 1832. Comment: Polyarthridae Daday, 1893 (type genus *Polyarthra* Ehrenberg, 1834) is available in Rotifera; this case is to be referred to the Commission to remove the homonymy (Art. 55.3.1).
Micropsalini Gounelle, 1911: 326 [stem: *Micropsalid-*]. Type genus: *Micropsalis* Burmeister, 1865 [preoccupied genus name, not *Micropsalis* Meyer, 1859 [Crustacea]; syn. of *Apterocaulus* Fairmaire, 1864]. Comment: permanently invalid (Art. 39): based on preoccupied type genus; incorrect original stem formation, not in prevailing usage.

### Tribe Remphanini

Lacordaire, 1868

Remphanides Lacordaire, 1868: 103 [stem: *Remphan-*]. Type genus: *Remphan* G. R. Waterhouse, 1835. Comment: original vernacular name available (Art. 11.7.2): first used in latinized form by Pascoe (1869b: 667, as Remphaninae), generally accepted as in Drumont and Komiya (2010: 95, as Remphanini); this name was incorrectly credited to Pascoe, 1869 by Bousquet et al. (2009: 18); we treat this group as a valid tribe as in Drumont and Komiya (2010: 95).
Rhaphipodi Lameere, 1912: 181 [stem: *Rhaphipod-*]. Type genus: *Rhaphipodus* Audinet-Serville, 1832.

### Tribe Solenopterini

Lacordaire, 1868

Solénoptérides Lacordaire, 1868: 180 [stem: *Solenopter-*]. Type genus: *Solenoptera* Audinet-Serville, 1832. Comment: original vernacular name available (Art. 11.7.2): first used in latinized form by J. L. LeConte (1873: 286, as Solenopterini), generally accepted as in Monné (1995c: 37, as Solenopterini).
*Dérancistrines Lameere, 1909: 1 [stem: *Derancistr-*]. Type genus: *Derancistrus* Audinet-Serville, 1832. Comment: original vernacular name unavailable (Art. 11.7.2): proposed after 1899.
Derancistrini Lameere, 1912: 181 [stem: *Derancistr-*]. Type genus: *Derancistrus* Audinet-Serville, 1832.

### Tribe Tereticini

Lameere, 1913

*Téréticiens Lameere, 1912: 72 [stem: *Teretic-*]. Type genus: *Tereticus* C. O. Waterhouse, 1879. Comment: original vernacular name unavailable (Art. 11.7.2): proposed after 1899.
Teretici Lameere, 1913: 87 [stem: *Teretic-*]. Type genus: *Tereticus* C. O. Waterhouse, 1879.

### Tribe Vesperoctenini

Vives, 2005

Vesperoctenini Vives, 2005: 438 [stem: *Vesperocten-*]. Type genus: *Vesperoctenus* H. W. Bates, 1891.

### Subfamily Lepturinae

Latreille, 1802

Lepturetae Latreille, 1802: 218 [stem: *Leptur-*]. Type genus: *Leptura* Linnaeus, 1758.

### Tribe Desmocerini

Blanchard, 1845

Desmocérites Blanchard, 1845b: 163 [stem: *Desmocer-*]. Type genus: *Desmocerus* Dejean, 1821. Comment: original vernacular name available (Art. 11.7.2): first used in latinized form by J. Thomson (1861: 159, as Desmoceritae), generally accepted as in Monné (1995b: 1, as Desmocerini).

### Tribe Encyclopini

LeConte, 1873

Encyclopini J. L. LeConte, 1873: 326 [stem: *Enclyclop-*]. Type genus: *Encyclops* Newman, 1838.

### Tribe Lepturini

Latreille, 1802

Lepturetae Latreille, 1802: 218 [stem: *Leptur-*]. Type genus: *Leptura* Linnaeus, 1758.
*Grammoptérates Mulsant, 1863b: 569 [stem: *Grammopter-*]. Type genus: *Grammoptera* Audinet-Serville, 1835. Comment: original vernacular name unavailable (Art. 11.7.2): not subsequently latinized.
*Strangalini Zagajkevich, 1991: 96 [stem: *Strangali-*]. Type genus: *Strangalia* Audinet-Serville, 1835. Comment: family-group name unavailable (Art. 11.6): originally published as synonym and not made available subsequently; incorrect original stem formation, not in prevailing usage.

### Tribe Oxymirini

Danilevsky, 1997

Oxymirini Danilevsky, 1997: 8 [stem: *Oxymir-*]. Type genus: *Oxymirus* Mulsant, 1862.

### Tribe Rhagiini

Kirby, 1837

Rhagiadae Kirby, 1837: 178 [stem: *Rhagi-*]. Type genus: *Rhagium* Fabricius, 1775.
*Toxotaires Mulsant, 1839: 230 [stem: *Toxot-*]. Type genus: *Toxotus* Dejean, 1821 [syn. of *Stenocorus* Geoffroy, 1762]. Comment: original vernacular name unavailable (Art. 11.7.2): subsequently used in latinized form but not generally attributed to Mulsant (1839).
*Pachytes Motschulsky, 1849: 60 [stem: *Pachyt-*]. Type genus: *Pachyta* Dejean, 1821. Comment: original vernacular name unavailable (Art. 11.7.2): subsequently used in latinized form but not generally attributed to Motschulsky (1849).
Stenocoritae J. Thomson, 1861: 156 [stem: *Stenocor-*]. Type genus: *Stenocorus* Geoffroy, 1762 [placed on the Official List of Generic Names in Zoology (ICZN 1994a)]. Comment: an application will need to be submitted to the Commission to suppress Stenocoridae Hope, 1834 (based on the misidentified type genus *Stenocorus* sensu Hope, 1834) for the Principles of Priority and Homonymy (Art. 65.2.1) if this name is to be used as valid.
Toxoti J. L. LeConte and G. H. Horn, 1883: 313 [stem: *Toxot-*]. Type genus: *Toxotus* Dejean, 1821 [syn. of *Stenocorus* Geoffroy, 1762]. Comment: junior homonym of Toxotidae Günther, 1860 (type genus *Toxotes* Cuvier and Cloquet, 1816) currently used as valid in Pisces; this case is to be referred to the Commission to remove the homonymy (Art. 55.3.1).
Pachytini Portevin, 1934: 119, in key [stem: *Pachyt-*]. Type genus: *Pachyta* Dejean, 1821.
*Enoploderini Danilevsky, 1997: 9 [stem: *Enoploder-*]. Type genus: *Enoploderes* Faldermann, 1837. Comment: unavailable family-group name, proposed after 1930 without description or bibliographic reference to such a description (Art. 13.1).

### Tribe Rhamnusiini

Sama, 2009

*Rhamnusiini Danilevsky, 1997: 9 [stem: *Rhamnusi-*]. Type genus: *Rhamnusium* Latreille, 1829. Comment: unavailable family-group name, proposed after 1930 without description or bibliographic reference to such a description (Art. 13.1).
Rhamnusiini Sama, 2009b: 383 [stem: *Rhamnusi-*]. Type genus: *Rhamnusium* Latreille, 1829.

### Tribe Teledapini

Pascoe, 1871

Teledapinae Pascoe, 1871: 268 [stem: *Teledap-*]. Type genus: *Teledapus* Pascoe, 1871.

### Tribe Sachalinobiini

Danilevsky, 2010

Sachalinobiini Danilevsky, 2010: 43 [stem: *Sachalinobi-*]. Type genus: *Sachalinobia* Jakobson, 1899.

### Tribe Xylosteini

Reitter, 1913

Xylosteina Reitter, 1913a: 5 [stem: *Xyloste-*]. Type genus: *Xylosteus* Frivaldszky, 1838.

### Subfamily Spondylidinae

Audinet-Serville, 1832

Spondylii Audinet-Serville, 1832: 123 [stem: *Spondylid-*]. Type genus: *Spondylis* Fabricius, 1775.

### Tribe Anisarthrini

Mamaev and Danilevsky, 1973

*Anisarthrites Fairmaire, 1864: 124 [stem: *Anisarthr-*]. Type genus: *Anisarthron* Dejean, 1835. Comment: original vernacular name unavailable (Art. 11.7.2): subsequently used in latinized form but not generally attributed to Fairmaire (1864).
Anisarthronini Mamaev and Danilevsky, 1973: 1260, in key [stem: *Anisarthr-*]. Type genus: *Anisarthron* Dejean, 1835. Comment: family-group name proposed as new without reference to Anisarthrites Fairmaire, 1864; incorrect original stem formation, not in prevailing usage.

### Tribe Asemini

Thomson, 1861

Asemitae J. Thomson, 1861: 259 [stem: *Asem-*]. Type genus: *Asemum* Eschscholtz, 1830.
*Criomorphates Mulsant, 1863a: 421 [stem: *Criomorph-*]. Type genus: *Criomorphus* Mulsant, 1839 [preoccupied genus name, not *Criomorphus* Curtis, 1831 [Hemiptera]; placed on the Official Index of Rejected and Invalid Generic Names in Zoology (ICZN 1988b); syn. of *Tetropium* Kirby, 1837]. Comment: original vernacular name unavailable (Art. 11.7.2): subsequently used in latinized form but not generally attributed to Mulsant (1863).
*Criocéphalites Fairmaire, 1864: 125 [stem: *Criocephal-*]. Type genus: *Criocephalus* Mulsant, 1839 [syn. of *Arhopalus* Audinet-Serville, 1834]. Comment: original vernacular name unavailable (Art. 11.7.2): subsequently used in latinized form but not generally attributed to Fairmaire (1864).
Tetropiina Seidlitz, 1891 [Gatt.]: 179 [stem: *Tetropi-*]. Type genus: *Tetropium* Kirby, 1837 [placed on the Official List of Generic Names in Zoology (ICZN 1988b)].
Criocephalinae Sharp, 1905: 147 [stem: *Criocephal-*]. Type genus: *Criocephalus* Mulsant, 1839 [syn. of *Arhopalus* Audinet-Serville, 1834].
Criomorphini Portevin, 1927: 20, in key [stem: *Criomorph-*]. Type genus: *Criomorphus* Mulsant, 1839 [preoccupied genus name, not *Criomorphus* Curtis, 1831 [Hemiptera]; placed on the Official Index of Rejected and Invalid Generic Names in Zoology (ICZN 1988b); syn. of *Tetropium* Kirby, 1837]. Comment: permanently invalid (Art. 39): based on preoccupied type genus.
Nothorhinini Zagajkevich, 1991: 110 [stem: *Nothorhin-*]. Type genus: *Nothorhina* Redtenbacher, 1845. Comment: placed in synonymy with Asemini by Vives and Alonso-Zarazaga (2000: 569).

### Tribe Atimiini

LeConte, 1873

Atimiini J. L. LeConte, 1873: 322 [stem: *Atimi-*]. Type genus: *Atimia* Haldeman, 1847.

### Tribe Saphanini

Gistel, 1848

Saphanidae Gistel, 1848: [8] [stem: *Saphan-*]. Type genus: *Saphanus* Audinet-Serville, 1834.
Michthysomini J. L. LeConte, 1873: 332 [stem: *Michthisomat-*]. Type genus: *Michthisoma* J. L. LeConte, 1850 [as *Michthysoma*, incorrect subsequent spelling of type genus name, not in prevailing usage]. Comment: incorrect original stem formation, not in prevailing usage.

### Tribe Spondylidini

Audinet-Serville, 1832

Spondylii Audinet-Serville, 1832: 123 [stem: *Spondylid-*]. Type genus: *Spondylis* Fabricius, 1775. Comment: incorrect original stem formation, not in prevailing usage.

### Subfamily Necydalinae

Latreille, 1825

Necydalides Latreille, 1825: 401 [stem: *Necydal-*]. Type genus: *Necydalis* Linnaeus, 1758.

### Subfamily Dorcasominae

Lacordaire, 1868

Dorcasomides Lacordaire, 1868: 456 [stem: *Dorcasom-*]. Type genus: *Dorcasomus* Audinet-Serville, 1834. Comment: original vernacular name available (Art. 11.7.2): first used in latinized form by Kolbe (1897: 299, as Dorcasominae), generally accepted as in Aurivillius (1912: 251, as Dorcasomini).

### Subfamily Apatophyseinae

Lacordaire, 1869

Apatophysides Lacordaire, 1869: 234 [stem: *Apatophyse-*]. Type genus: *Apatophysis* Chevrolat, 1860. Comment: original vernacular name available (Art. 11.7.2): first used in latinized form by Heyne and Taschenberg (1907: 240, as Apatophysini), generally accepted as in Švácha and Danilevsky (1988: 125, as Apatophyseinae); incorrect original stem formation, not in prevailing usage; valid status given here as in Danilevsky (2010: 48).

### Subfamily Cerambycinae

Latreille, 1802

Cerambicini Latreille, 1802: 211 [stem: *Cerambyc-*]. Type genus: *Cerambyx* Linnaeus, 1758.

### Tribe Acangassuini

Galileo and Martins, 2001

Acangassuini Galileo and Martins, 2001: 95 [stem: *Acangassu-*]. Type genus: *Acangassu* Galileo and Martins, 2001. Comment: this name was incorrectly treated as unavailable by Bousquet et al. (2009).

### Tribe Achrysonini

Lacordaire, 1868

Achrysonides Lacordaire, 1868: 231 [stem: *Achryson-*]. Type genus: *Achryson* Audinet-Serville, 1833. Comment: original vernacular name available (Art. 11.7.2): first used in latinized form by Murray (1870: 169, as Achrysonidae), generally accepted as in Aurivillius (1912: 39, as Achrysonini); current spelling maintained (Art. 29.5): incorrect original stem formation in prevailing usage (should be *Achrys*-).

### Tribe Agallissini

LeConte, 1873

Agallissini J. L. LeConte, 1873: 321 [stem: *Agalliss-*]. Type genus: *Agallissus* Dalman, 1823.

### Tribe Alanizini

Di Iorio, 2003

Alanizini Di Iorio, 2003: 1 [stem: *Alaniz-*]. Type genus: *Alanizus* Di Iorio, 2003.

### Tribe Anaglyptini

Lacordaire, 1868 nomen protectum

Anaglyptides Lacordaire, 1868: 404 [stem: *Anaglypt-*]. Type genus: *Anaglyptus* Mulsant, 1839. Comment: original vernacular name available (Art. 11.7.2): first used in latinized form by J. L. LeConte (1873: 319, as Anaglypti), generally accepted as in Linsley (1964: 173, as Anaglyptini); junior homonym of Anaglyptidae Gistel, 1848 (type genus *Anaglyptes* Gistel, 1848; syn. of *Chalcophora* Dejean, 1833) in Coleoptera: Buprestidae; this name was treated as a *nomen protectum* by Bousquet et al. (2009: 41).

### Tribe Aphanasiini

Lacordaire, 1868

Aphanasiides Lacordaire, 1868: 367 [stem: *Aphanasi-*]. Type genus: *Aphanasium* Dejean, 1835. Comment: original vernacular name available (Art. 11.7.2): first used in latinized form and generally accepted as in Aurivillius (1912: 139, as Aphanasiini).

### Tribe Aphneopini

Lacordaire, 1868

Aphnéopides Lacordaire, 1868: 421 [stem: *Aphneop-*]. Type genus: *Aphneope* Pascoe, 1863. Comment: original vernacular name available (Art. 11.7.2): first used in latinized form and generally accepted as in Aurivillius (1912: 155, as Aphneopini).

### Tribe Auxesini

Lepesme and Breuning, 1952

*Auxésides Lacordaire, 1872: 463 [stem: *Auxes-*]. Type genus: *Auxesis* J. Thomson, 1858. Comment: original vernacular name unavailable (Art. 11.7.2): subsequently used in latinized form but not generally attributed to Lacordaire (1872).
Auxesina Lepesme and Breuning, 1952: 140 [stem: *Auxes-*]. Type genus: *Auxesis* J. Thomson, 1858. Comment: current spelling maintained (Art. 29.5): incorrect original stem formation in prevailing usage (should be *Auxese*-).
Psathyrini Quentin, 1954: 103, in key [stem: *Psathyr-*]. Type genus: *Psathyrus* J. Thomson, 1857.

### Tribe Basipterini

Fragoso, Monné and Campos Seabra, 1987

Basipterini Fragoso et al., 1987: 201 [stem: *Basipter-*]. Type genus: *Basiptera* J. Thomson, 1864.

### Tribe Bimiini

Lacordaire, 1868

Bimiides Lacordaire, 1868: 464 [stem: *Bimi-*]. Type genus: *Bimia* A. White, 1850. Comment: original vernacular name available (Art. 11.7.2): first used in latinized form and generally accepted as in Aurivillius (1912: 254, as Bimiini).
Sibyllini Cerda, 1973: 115 [stem: *Sibyll-*]. Type genus: *Sibylla* J. Thomson, 1858 [preoccupied genus name, not *Sibylla* Stål, 1856 [Orthoptera]; the valid name for this genus has recently been given as *Sybilla* J. Thomson, 1864 by Monné and Bezark (2009: 30) which is an incorrect subsequent spelling of *Sibylla* J. Thomson, 1858; syn. of *Zehra* Özdikmen, 2008]. Comment: permanently invalid (Art. 39): based on preoccupied type genus.

### Tribe Bothriospilini

Lane, 1950

Bothriospilinae Lane, 1950: 370 [stem: *Bothriospil-*]. Type genus: *Bothriospila* Aurivillius, 1923.

### Tribe Brachypteromatini

Sama, 2008

Brachypteromini Sama, 2008: 229 [stem: *Brachypteromat-*]. Type genus: *Brachypteroma* Heyden, 1863. Comment: incorrect original stem formation, not in prevailing usage.

### Tribe Callichromatini

Swainson, 1840

Callichrominae Swainson, 1840: 293 [stem: *Callichromat-*]. Type genus: *Callichroma* Latreille, 1816. Comment: incorrect original stem formation, not in prevailing usage; this family-group name was incorrectly credited to Swainson and Shuckard (1840) by Bousquet et al. (2009: 42).
Terambidae Gistel, 1848: [8] [stem: *Teramb-*]. Type genus: *Terambus* Gistel, 1848 [syn. of *Aromia* Audinet-Serville, 1834].

### Tribe Callidiini

Kirby, 1837

Callidiadae Kirby, 1837: 170 [stem: *Callidi-*]. Type genus: *Callidium* Fabricius, 1775 [for comments on problems with the authorship and type species of this type genus see Bousquet et al. (2009: 42)].
*Phymatodates Mulsant, 1863a: 397 [stem: *Phymatod-*]. Type genus: *Phymatodes* Mulsant, 1839 [placed on the Official List of Generic Names in Zoology (ICZN 1989a)]. Comment: original vernacular name unavailable (Art. 11.7.2): not subsequently latinized.

### Tribe Callidiopini

Lacordaire, 1868

Callidiopsides Lacordaire, 1868: 340 [stem: *Callidiop-*]. Type genus: *Callidiopis* A. White, 1855 [as *Callidiopsis*, unjustified emendation of type-genus by Lacordaire (1868: 356), not in prevailing usage]. Comment: original vernacular name available (Art. 11.7.2): first used in latinized form by Pascoe (1869b: 535, as Callidiopsinae [incorrect stem formation]), generally accepted as in Aurivillius (1912: 115, as Callidiopini); current spelling maintained (Art. 29.3.1.1): incorrect stem formation in prevailing usage (should be *Callidiopid*-).

### Tribe Cerambycini

Latreille, 1802

Cerambicini Latreille, 1802: 211 [stem: *Cerambyc-*]. Type genus: *Cerambyx* Linnaeus, 1758.

### Subtribe Cerambycina

Latreille, 1802

Cerambicini Latreille, 1802: 211 [stem: *Cerambyc-*]. Type genus: *Cerambyx* Linnaeus, 1758. Comment: Gistel (1856a: 375) used the name Ceratambycidae but this was based on the incorrect subsequent spelling of the type genus name (incorrectly given as *Caratambyx* in the same work on page 375 but later corrected to *Ceratambyx* by Gistel (1856b: 13)) and was therefore not proposed as a new family-group name.

### Subtribe Sphallotrichina

Martins and Monné, 2005

Sphallotrichina Martins and Monné, 2005: 2 [stem: *Sphallotrich-*]. Type genus: *Sphallotrichus* Fragoso, 1982. Comment: this name was incorrectly treated as unavailable by Bousquet et al. (2009).

### Tribe Certallini

Fairmaire, 1864

Cartallites Fairmaire, 1864: 149 [stem: *Certall-*]. Type genus: *Certallum* Dejean, 1821 [as *Cartallum*, unjustified emendation of type genus name by Audinet-Serville (1834: 94), not in prevailing usage]. Comment: published early September 1864; original vernacular name available (Art. 11.7.2): first used in a latinized form by Sama (1990: 287, as Certallini), generally accepted as in Vives (2000: 155, as Certallini); incorrect original stem formation, not in prevailing usage.
Pytheitae J. Thomson, 1864: 153 [stem: *Pythe-*]. Type genus: *Pytheus* Newman, 1840. Comment: published before October 1864.
*Erionispites Chapuis, 1875: 301 [stem: *Erionisp-*]. Type genus: *Erionispa* Chapuis, 1875 [syn. of *Pytheus* Newman, 1840]. Comment: original vernacular name unavailable (Art. 11.7.2): subsequently used in latinized form but not generally attributed to Chapuis (1875); Erionispidae was used as valid by Ienistea (1986: 31) but it was not attributed to Chapuis (1875); Ienistea’s name is also unavailable, it was proposed after 1930 without description or bibliographic reference to such a description (Art. 13.1); name written with an accent as Érionispites on page 301 of the original publication, but without accent in the key and for the description; type genus transferred from Chrysomelidae by Lameere (1885) and treated as a synonym of *Pytheus* Newman, 1840.

### Tribe Chlidonini

Waterhouse, 1879

Chlidoninae C. O. Waterhouse, 1879b: 320 [stem: *Chlidon-*]. Type genus: *Chlidones* C. O. Waterhouse, 1879.

### Tribe Cleomenini

Lacordaire, 1868

Cléoménides Lacordaire, 1868: 405 [stem: *Cleomen-*]. Type genus: *Cleomenes* J. Thomson, 1864. Comment: original vernacular name available (Art. 11.7.2): first used in latinized form by Pascoe (1869b: 554, as Cleomeninae), generally accepted as in Adlbauer et al. (2010: 163, as Cleomenini); this name was incorrectly credited to Pascoe (1869) by Bousquet et al. (2009: 55); we treat this group as a valid tribe as in Adlbauer et al. (2010: 163).

### Tribe Clytini

Mulsant, 1839

Clytaires Mulsant, 1839: 70 [stem: *Clyt-*]. Type genus: *Clytus* Laicharting, 1784. Comment: original vernacular name available (Art. 11.7.2): first used in latinized form by Gistel (1848: [8], as Clytiidae [incorrect stem formation]), generally accepted as in Aurivillius (1912: 358, as Clytini).
Neoclytitae J. Thomson, 1861: 219 [stem: *Neoclyt-*]. Type genus: *Neoclytus* J. Thomson, 1861.
Cyllenitae J. Thomson, 1864: 184 [stem: *Cyllen-*]. Type genus: *Cyllene* Newman, 1840 [preoccupied genus name, not *Cyllene* Gray, 1834 [Mollusca]; syn. of *Megacyllene* Casey, 1912]. Comment: permanently invalid (Art. 39): based on preoccupied type genus.

### Tribe Compsocerini

Thomson, 1864

Compsoceritae J. Thomson, 1864: 260 [stem: *Compsocer-*]. Type genus: *Compsocerus* Audinet-Serville, 1834 [for comments on problems with the authorship and type species of this type genus see Bousquet et al. (2009: 44)].

### Tribe Coptommatini

Lacordaire, 1869

Coptommides Lacordaire, 1869: 221 [stem: *Coptommat-*]. Type genus: *Coptomma* Newman, 1840. Comment: original vernacular name available (Art. 11.7.2): first used in latinized form and generally accepted as in Aurivillius (1912: 488, as Coptommatini); First Reviser (Coptommatini Lacordaire, 1869 vs Navomorphini Lacordaire, 1869) not determined, current usage maintained.
Navomorphides Lacordaire, 1869: 223 [stem: *Navomorph-*]. Type genus: *Navomorpha* A. White, 1855 [syn. of *Coptomma* Newman, 1840]. Comment: original vernacular name available (Art. 11.7.2): first used in latinized form and generally accepted as in Aurivillius (1912: 488, as Navomorphini).

### Tribe Curiini

LeConte, 1873

Curii J. L. LeConte, 1873: 304 [stem: *Curi-*]. Type genus: *Curius* Newman, 1840.

### Tribe Deilini

Fairmaire, 1864

*Déilates Mulsant, 1863b: 190 [stem: *Deil-*]. Type genus: *Deilus* Audinet-Serville, 1834. Comment: original vernacular name unavailable (Art. 11.7.2): subsequently used in latinized form but not generally attributed to Mulsant (1863).
Déilates Fairmaire, 1864: 154 [stem: *Deil-*]. Type genus: *Deilus* Audinet-Serville, 1834. Comment: original vernacular name available (Art. 11.7.2): first used in latinized form and generally accepted as in Aurivillius (1912: 294, as Deilini).

### Tribe Dejanirini

Lacordaire, 1868

Déjanirides Lacordaire, 1868: 460 [stem: *Dejanir-*]. Type genus: *Dejanira* J. Thomson, 1864. Comment: original vernacular name available (Art. 11.7.2): first used in latinized form by Pascoe (1869b: 561, as Dejanirinae), generally accepted as in Aurivillius (1912: 253, as Dejanirini).

### Tribe Diorini

Lane, 1950

Diorinae Lane, 1950: 373 [stem: *Dior-*]. Type genus: *Diorus* A. White, 1853.

### Tribe Distichocerini

Pascoe, 1867

*Distichocérites Blanchard, 1845b: 144 [stem: *Distichocer-*]. Type genus: *Distichocera* Kirby, 1819. Comment: original vernacular name unavailable (Art. 11.7.2): subsequently used in latinized form but not generally attributed to Blanchard (1845b).
Distichocerinae Pascoe, 1867a: 125 [stem: *Distichocer-*]. Type genus: *Distichocera* Kirby, 1819.

### Tribe Dodecosini

Aurivillius, 1912

Dodecosini Aurivillius, 1912: 132 [stem: *Dodecos-*]. Type genus: *Dodecosis* H. W. Bates, 1867. Comment: current spelling maintained (Art. 29.5): incorrect original stem formation in prevailing usage (should be *Dodecose*-).
Olexandrellaeini Zajciw, 1960: 605 [stem: *Olexandrell-*]. Type genus: *Olexandrella* Zajciw, 1959. Comment: incorrect original stem formation, not in prevailing usage.

### Tribe Dryobiini

Arnett, 1962 nomen protectum

Dryobiini Arnett, 1962c: 861 [stem: *Dryobi-*]. Type genus: *Dryobius* J. L. LeConte, 1850. Comment: *nomen protectum* (see Bousquet et al. 2009: 45); this is a junior homonym of Dryobiadae Gistel, 1856a: 368 (type genus *Dryobia* Gistel, 1856) which is a junior synonym of Dryophilidae Gistel, 1848.

### Tribe Eburiini

Blanchard, 1845

Éburiites Blanchard, 1845b: 145 [stem: *Eburi-*]. Type genus: *Eburia* Lacordaire, 1830. Comment: original vernacular name available (Art. 11.7.2): first used in latinized form by J. Thomson (1861: 237, as Eburitae), generally accepted as in Monné (1993a: 20, as Eburiini).

### Tribe Ectenessini

Martins, 1998

Ectenessini Martins, 1998: 82 [stem: *Ecteness-*]. Type genus: *Ectenessa* H. W. Bates, 1885.

### Tribe Elaphidiini

Thomson, 1864

Elaphidionitae J. Thomson, 1864: 235 [stem: *Elaphidi-*]. Type genus: *Elaphidion* Audinet-Serville, 1834. Comment: incorrect original stem formation, not in prevailing usage; Ivie (1985: 303) pointed out that the correct stem based on *Elaphidion* is *Elaphidi*-; both Elaphidiini and Elaphidionini have been used in recent literature, we prefer to use the correct spelling of the stem here.
Sphérionides Lacordaire, 1868: 312 [stem: *Sphaeri-*]. Type genus: *Sphaerion* Audinet-Serville, 1834. Comment: original vernacular name available (Art. 11.7.2): first used in latinized form by Heyne and Taschenberg (1907: 239, as Sphaerionini), generally accepted as in Aurivillius (1912: 96, as Sphaerionini); incorrect original stem formation, not in prevailing usage; junior homonym of Sphaeriidae Deshayes, 1855 (type genus *Sphaerium* Scopoli, 1877) in Mollusca and Sphaerina Erichson, 1845 [incorrect original spelling] (type genus *Sphaerius* Waltl, 1838) in Myxophaga, the stem of the beetle family-group name was recently emended to *Sphaerius*- (ICZN 2000); this case is to be referred to the Commission to remove the homonymy (Art. 55.3.1).
Stenosphenini J. L. LeConte, 1873: 316 [stem: *Stenosphen-*]. Type genus: *Stenosphenus* Haldeman, 1847.

### Tribe Eligmodermini

Lacordaire, 1868

Éligmodermides Lacordaire, 1868: 337 [stem: *Eligmoderm-*]. Type genus: *Eligmoderma* J. Thomson, 1864. Comment: original vernacular name available (Art. 11.7.2): first used in latinized form and generally accepted as in Aurivillius (1912: 114, as Eligmodermini); current spelling maintained (Art. 29.5): incorrect original stem formation in prevailing usage (should be *Eligmodermat*-).

### Tribe Erlandiini

Aurivillius, 1912

Erlandiini Aurivillius, 1912: 12 [stem: *Erlandi-*]. Type genus: *Erlandia* Aurivillius, 1904.

### Tribe Eroschemini

Lacordaire, 1868

Éroschémides Lacordaire, 1868: 515 [stem: *Eroschem-*]. Type genus: *Eroschema* Pascoe, 1859. Comment: original vernacular name available (Art. 11.7.2): first used in latinized form by H. W. Bates (1872a: 185, as Eroscheminae), generally accepted as in Aurivillius (1912: 287, as Eroschemini); current spelling maintained (Art. 29.5): incorrect original stem formation in prevailing usage (should be *Eroschemat*-).

### Tribe Eumichthini

Linsley, 1940

Eumichthini Linsley, 1940: 368 [stem: *Eumichth-*]. Type genus: *Eumichthus* J. L. LeConte, 1873.

### Tribe Gahaniini

Quentin and Villiers, 1969

Gahaniini Quentin and Villiers, 1969: 615, in key [stem: *Gahani-*]. Type genus: *Gahania* Distant, 1907.

### Tribe Glaucytini

Lacordaire, 1868

Glaucytides Lacordaire, 1868: 405 [stem: *Glaucyt-*]. Type genus: *Glaucytes* J. Thomson, 1857. Comment: original vernacular name available (Art. 11.7.2): first used in latinized form by Pascoe (1869b: 650, as Glaucytinae), generally accepted as in Aurivillius (1912: 435, as Glaucytini); current spelling maintained (Art. 29.5): incorrect stem formation in prevailing usage (should be *Glaucytet*-).

### Tribe Graciliini

Mulsant, 1839

Graciliaires Mulsant, 1839: 99 [stem: *Gracili-*]. Type genus: *Gracilia* Audinet-Serville, 1834. Comment: original vernacular name available (Art. 11.7.2): first used in latinized form by J. L. LeConte (1873: 300, as Graciliinae), generally accepted as in Monné (1993b: 9, as Graciliini).

### Tribe Hesperophanini

Mulsant, 1839

Hespérophanaires Mulsant, 1839: 61 [stem: *Hesperophan-*]. Type genus: *Hesperophanes* Dejean, 1835.

### Subtribe Daramina

Sama, 2008

Daramina Sama, 2008: 224 [stem: *Daram-*]. Type genus: *Daramus* Fairmaire, 1892.

### Subtribe Hesperophanina

Mulsant, 1839

Hespérophanaires Mulsant, 1839: 61 [stem: *Hesperophan-*]. Type genus: *Hesperophanes* Dejean, 1835 [see Vives and Alonso-Zarazaga (2000: 657) and Bousquet et al. (2009: 47) for a discussion of problems with the type species of this genus]. Comment: original vernacular name available (Art. 11.7.2): first used in latinized form by Pascoe (1869: 523, as Hesperophaninae), generally accepted as in Monné (1993a: 1, as Hesperophanini).
Cerasphoritae J. Thomson, 1861: 234 [stem: *Cerasphor-*]. Type genus: *Cerasphorus* Audinet-Serville, 1834.

### Tribe Hesthesini

Pascoe, 1867

Hesthesinae Pascoe, 1867a: 127 [stem: *Hesthes-*]. Type genus: *Hesthesis* Newman, 1840. Comment: current spelling maintained (Art. 29.5): incorrect original stem formation in prevailing usage (should be *Hesthese*-).

### Tribe Heteropsini

Lacordaire, 1868 nomen protectum

Dichophyiaeidae Gistel, 1848: [8] [stem: *Dichophyi-*]. Type genus: *Dichophyia* Gistel, 1848. Comment: *nomen oblitum* (see Bousquet et al. 2009: 48). Comment: incorrect original stem formation, not in prevailing usage.
Hétéropsides Lacordaire, 1868: 405 [stem: *Heterops-*]. Type genus: *Heterops* Blanchard, 1842. Comment: *nomen protectum* (see Bousquet et al. 2009: 48); original vernacular name available (Art. 11.7.2): first used in latinized form by H. W. Bates (1872a: 179, as Heteropsinae), generally accepted as in Aurivillius (1912: 438, as Heteropsini); current spelling maintained (Art. 29.5): incorrect stem formation in prevailing usage (should be *Heterop*-); this name was incorrectly attributed to Lacordaire (1869) by Bousquet et al. (2009: 48).

### Tribe Hexoplini

Martins, 2006

Hexoplonini Martins, 2006: 22 [stem: *Hexopl-*]. Type genus: *Hexoplon* J. Thomson, 1864. Comment: incorrect original stem formation, not in prevailing usage; this name was incorrectly treated as unavailable by Bousquet et al. (2009).

### Tribe Holopleurini

Chemsak and Linsley, 1974

Holopleurini Chemsak and Linsley, 1974: 183 [stem: *Holopleur-*]. Type genus: *Holopleura* J. L. LeConte, 1873.

### Tribe Holopterini

Lacordaire, 1868

Holoptérides Lacordaire, 1868: 393 [stem: *Holopter-*]. Type genus: *Holopterus* Blanchard, 1851. Comment: original vernacular name available (Art. 11.7.2): first used in latinized form by Lucas (1920: xxiii, as Holopterini), generally accepted as in Aurivillius (1912: 148, as Holopterini); this tribe was transferred to the subfamily Lepturinae by Vitali (2002: 32) but placed back into the Cerambycinae by Monné (2005a: 301).

### Tribe Hyboderini

Linsley, 1940

Hyboderini Linsley, 1940: 371 [stem: *Hyboder-*]. Type genus: *Hybodera* J. L. LeConte, 1873.

### Tribe Hylotrupini

Zagajkevich, 1991

Hylotrupini Zagajkevich, 1991: 67 [stem: *Hylotrup-*]. Type genus: *Hylotrupes* Audinet-Serville, 1834.

### Tribe Ibidionini

Thomson, 1861

Ibidionitae J. Thomson, 1861: 199 [stem: *Ibidion-*]. Type genus: *Ibidion* Gory, 1833.

### Subtribe Compsina

Martins and Galileo, 2007

Compsina Martins and Galileo, 2007: 6, in key [stem: *Comps-*]. Type genus: *Compsa* Perty, 1832. Comment: this name was incorrectly treated as unavailable by Bousquet et al. (2009); junior homonym of Compsini Pierce, 1913 (type genus *Compsus* Schönherr, 1823) in Curculionidae; this case is to be referred to the Commission to remove the homonymy (Art. 55.3.1).

### Subtribe Ibidionina

Thomson, 1861

Ibidionitae J. Thomson, 1861: 199 [stem: *Ibidion-*]. Type genus: *Ibidion* Gory, 1833. Comment: current spelling maintained (Art. 29.5): incorrect stem formation in prevailing usage (should be *Ibidi*-).
*Sydacini Martins, 2003a: 204 [stem: *Sydac-*]. Type genus: *Sydax* Lacordaire, 1868. Comment: name unavailable (Art. 16.1): name not indicated as intentionally new; this taxon was originally described by Martins (1997a: 8-9) but not named.

### Subtribe Tropidina

Martins and Galileo, 2007

Tropidina Martins and Galileo, 2007: 7 [stem: *Tropid-*]. Type genus: *Tropidion* J. Thomson, 1867. Comment: this name was incorrectly treated as unavailable by Bousquet et al. (2009); incorrect original spelling maintained (should be *Tropidi*-) in order to avoid homonymy with Tropidiini Hull, 1949 (type genus *Tropidia* Meigen, 1822) available in Diptera: Syrphidae.

### Tribe Ideratini

Martins and Napp, 2009

Ideratini Martins and Napp, 2009: 216 [stem: *Iderat-*]. Type genus: *Ideratus* J. Thomson, 1864.

### Tribe Lissonotini

Swainson, 1840

Lissonotinae Swainson, 1840: 289 [stem: *Lissonot-*]. Type genus: *Lissonotus* Dalman, 1817. Comment: this family-group name was incorrectly credited to Swainson and Shuckard (1840) by Bousquet et al. (2009: 49); the junior homonym Lissonotini Förster, 1869 (type genus *Lissonota* Gravenhorst, 1829) is available in Hymenoptera: Ichneumonidae; this case is to be referred to the Commission to remove the homonymy (Art. 55.3.1).

### Tribe Luscosmodicini

Martins, 2003

Luscosmodicini Martins, 2003b: 30 [stem: *Luscosmodic-*]. Type genus: *Luscosmodicum* Martins, 1970. Comment: this name was incorrectly treated as unavailable by Bousquet et al. (2009).

### Tribe Lygrini

Sama, 2008

Lygrini Sama, 2008: 222 [stem: *Lygr-*]. Type genus: *Lygrus* Fåhraeus, 1872.

### Tribe Macronini

Lacordaire, 1868

Enchapteritae J. Thomson, 1861: 151 [stem: *Enchopter-*]. Type genus: *Enchoptera* Saunders, 1850 [as *Enchaptera*, incorrect subsequent spelling type genus name, not in prevailing usage]. Comment: incorrect original stem formation, not in prevailing usage; this name has precedence over Macronini Lacordaire, 1868 but has not been used as a valid name after 1899 to our knowledge, although we were unable to find 25 references to conserve usage of Macronini (Art. 23.9.2), we believe the name Macronini should be conserved for this group and an application should be submitted to the Commission (Art. 23.9.3).
Macronides Lacordaire, 1868: 414 [stem: *Macron-*]. Type genus: *Macrones* Newman, 1841. Comment: original vernacular name available (Art. 11.7.2): first used in latinized form by Pascoe (1871: 268, as Macroninae), generally accepted as in Aurivillius (1912: 153, as Macronini); although this is not the oldest name for the tribe, we recommend that an application be submitted to the Commission to conserve usage of the well-established name Macronini Lacordaire, 1868.

### Tribe Megacoelini

Quentin and Villiers, 1969

Megacoelini Quentin and Villiers, 1969: 615, in key [stem: *Megacoel-*]. Type genus: *Megacoelus* Lacordaire, 1868.

### Tribe Methiini

Thomson, 1860

Methiitae J. Thomson, 1860a: 127 [stem: *Methi-*]. Type genus: *Methia* Newman, 1842.

### Tribe Molorchini

Gistel, 1848

Molorchidae Gistel, 1848: [9] [stem: *Molorch-*]. Type genus: *Molorchus* Fabricius, 1792 [see Bousquet (2008: 620) for a discussion of the type species]. Comment: name previously attributed to Mulsant (1862) but was also used by Marseul (1857a: 166, as Molorchidae) prior to Mulsant’s name.

### Tribe Mythodini

Lacordaire, 1868

Mythodides Lacordaire, 1868: 418 [stem: *Mythod-*]. Type genus: *Mythodes* J. Thomson, 1864. Comment: original vernacular name available (Art. 11.7.2): first used in latinized form by Pascoe (1871: 268, as Mythodinae), generally accepted as in Aurivillius (1912: 154, as Mythodini).

### Tribe Necydalopsini

Lacordaire, 1868

Nécydalopsides Lacordaire, 1868: 493 [stem: *Necydalops-*]. Type genus: *Necydalopsis* Blanchard, 1851. Comment: original vernacular name available (Art. 11.7.2): first used in latinized form and generally accepted as in Aurivillius (1912: 275, as Necydalopsini); current spelling maintained (Art. 29.5): incorrect original stem formation in prevailing usage (should be *Necydalopse*-).

### Tribe Neocorini

Martins, 2005

Neocorini Martins, 2005: 240 [stem: *Neocor-*]. Type genus: *Neocorus* J. Thomson, 1864. Comment: this name was incorrectly treated as unavailable by Bousquet et al. (2009).

### Tribe Neostenini

Lacordaire, 1868

Néosténides Lacordaire, 1868: 363 [stem: *Neosten-*]. Type genus: *Neostenus* Pascoe, 1857. Comment: original vernacular name available (Art. 11.7.2): first used in latinized form by Pascoe (1871: 268, as Neosteninae), generally accepted as in Aurivillius (1912: 138, as Neostenini).

### Tribe Obriini

Mulsant, 1839

Obriaires Mulsant, 1839: 95 [stem: *Obri-*]. Type genus: *Obrium* Dejean, 1821. Comment: original vernacular name available (Art. 11.7.2): first used in latinized form by Gistel (1848: [9], as Obriidae), generally accepted as in Monné (1993a: 11, as Obriini).

### Tribe Ochyrini

Pascoe, 1871

Ochyrinae Pascoe, 1871: 273 [stem: *Ochyr-*]. Type genus: *Ochyra* Pascoe, 1871.

### Tribe Oedenoderini

Aurivillius, 1912

Oedenoderini Aurivillius, 1912: 358 [stem: *Oedenoder-*]. Type genus: *Oedenoderus* Chevrolat, 1858.

### Tribe Oemini

Lacordaire, 1868

Oemides Lacordaire, 1868: 216 [stem: *Oem-*]. Type genus: *Oeme* Newman, 1840.

### Subtribe Methioidina

Martins, 1997

Methioidina Martins, 1997a: 119 [stem: *Methioid-*]. Type genus: *Methioides* Chemsak and Linsley, 1967.

### Subtribe Oemina

Lacordaire, 1868

*Malacoptérites Blanchard, 1845b: 147 [stem: *Malacopter-*]. Type genus: *Malacopterus* Audinet-Serville, 1833. Comment: original vernacular name unavailable (Art. 11.7.2): not subsequently latinized.
Oemides Lacordaire, 1868: 216 [stem: *Oem-*]. Type genus: *Oeme* Newman, 1840. Comment: original vernacular name available (Art. 11.7.2): first used in latinized form by Pascoe (1869b: 498, as Oeminae), generally accepted as in Aurivillius (1912: 26, as Oemini).

### Tribe Opsimini

LeConte, 1873

Opsimi J. L. LeConte, 1873: 294 [stem: *Opsim-*]. Type genus: *Opsimus* Mannerheim, 1843.

### Tribe Oxycoleini

Martins and Galileo, 2003

Oxycoleini Martins and Galileo, 2003: 52 [stem: *Oxycole-*]. Type genus: *Oxycoleus* Lacordaire, 1868. Comment: this name was incorrectly treated as unavailable by Bousquet et al. (2009).

### Tribe Paraholopterini

Martins, 1997

Paraholopterini Martins, 1997b: 201 [stem: *Paraholopter-*]. Type genus: *Paraholopterus* Cerda and Cekalovic, 1987.

### Tribe Phalotini

Lacordaire, 1868

Phalotides Lacordaire, 1868: 495 [stem: *Phalot-*]. Type genus: *Phalota* Pascoe, 1863. Comment: original vernacular name available (Art. 11.7.2): first used in latinized form and generally accepted as in Aurivillius (1912: 276, as Phalotini).

### Tribe Phlyctaenodini

Lacordaire, 1868

Phlycténodides Lacordaire, 1868: 370 [stem: *Phlyctaenod-*]. Type genus: *Phlyctaenodes* Newman, 1840. Comment: original vernacular name available (Art. 11.7.2): first used in latinized form by Aurivillius (1912: 140, as Phlyctaenodini), generally accepted as in Monné (1993b: 20, as Phlyctaenodini); incorrect original stem formation, not in prevailing usage.

### Tribe Phoracanthini

Newman, 1840

Stenocoridae Hope, 1834: 106 [stem: *Stenocor-*]. Type genus: *Stenocorus* sensu Hope, 1835 [not *Stenocorus* Geoffroy, 1762; syn. of *Phoracantha* Newman, 1840]. Comment: based on a misidentified type genus, name treated here as invalid until an application is submitted to the Commission to suppress it for the Principles of Priority and Homonymy (Art. 65.2.1); also see Stenocoritae J. Thomson, 1861 in Lepturinae: Rhagiini.
Phoracanthidae Newman, 1840: 2 [stem: *Phoracanth-*]. Type genus: *Phoracantha* Newman, 1840.

### Tribe Phyllarthriini

Lepesme and Breuning, 1956

Phyllarthriini Lepesme and Breuning, 1956: 287 [stem: *Phyllarthri-*]. Type genus: *Phyllarthrius* Hope, 1843.

### Tribe Piesarthriini

McKeown, 1947

Piesarthrini McKeown, 1947: 55 [stem: *Piesarthri-*]. Type genus: *Piesarthrius* Hope, 1834. Comment: name proposed after 1930 without description or bibliographic reference to such a description (Art. 13.1), however available because it was used as valid before 2000 as in Gressitt (1959: 84, as Piesarthini) and was not rejected by an author who, between 1961 and 1999, applied Article 13 of the then current edition of the Code (see Art. 13.2.1); incorrect original stem formation, not in prevailing usage.

### Tribe Piezocerini

Lacordaire, 1868

Piézocérides Lacordaire, 1868: 324 [stem: *Piezocer-*]. Type genus: *Piezocera* Audinet-Serville, 1834.

### Subtribe Haruspicina

Martins, 1976

Haruspicina Martins, 1976: 199 [stem: *Haruspic-*]. Type genus: *Haruspex* J. Thomson, 1864.

### Subtribe Piezocerina

Lacordaire, 1868

Piézocérides Lacordaire, 1868: 324 [stem: *Piezocer-*]. Type genus: *Piezocera* Audinet-Serville, 1834. Comment: original vernacular name available (Art. 11.7.2): first used in latinized form and generally accepted as in Aurivillius (1912: 102, as Piezocerini).
Zelliboriinae Lane, 1951: 5 [stem: *Zellibori-*]. Type genus: *Zelliboria* Lane, 1951.

### Tribe Platyarthrini

Bates, 1870

*Coelarthrides Lacordaire, 1868: 405 [stem: *Caelomarth-*]. Type genus: *Caelomarthron* J. Thomson, 1860 [as *Coelarthron*, unjustified emendation of type genus name by Lacordaire (1869: 142), not in prevailing usage; syn. of *Platyarthron* Guérin-Méneville, 1844]. Comment: original vernacular name unavailable (Art. 11.7.2): subsequently used in latinized form but not generally accepted as valid; subsequent usage of Coelarthrinae by Lucas (1920: 17) and Caelarthrinae by Ferreira and Veiga Ferreira (1959b: 331) did not validate this name because Lacordaire’s taxon was listed as a synonym of Platyarthrini H. W. Bates; Coelarthridae was used as valid by Ienistea (1986: 30) but it was not attributed to Lacordaire (1868); Ienistea’s name is also unavailable, it was proposed after 1930 without description or bibliographic reference to such a description (Art. 13.1); incorrect original stem formation, not in prevailing usage.
Platyarthrinae H. W. Bates, 1870: 419 [stem: *Platyarthr-*]. Type genus: *Platyarthron* Guérin-Méneville, 1844.

### Tribe Plectogasterini

Quentin and Villiers, 1969

Plectogasterini Quentin and Villiers, 1969: 615, in key [stem: *Plectogaster-*]. Type genus: *Plectogaster* C. O. Waterhouse, 1881. Comment: Bousquet et al. (2009: 52) erroneously listed Plectogastrini as the original spelling.

### Tribe Plectromerini

Nearns and Braham, 2008

Plectromerini Nearns and Braham, 2008: 19 [stem: *Plectromer-*]. Type genus: *Plectromerus* Haldeman, 1847 [see Bousquet et al. (2009: 53) about problems with the type species of this genus].

### Tribe Pleiarthrocerini

Lane, 1950

Pleiarthrocerinae Lane, 1950: 371 [stem: *Pleiarthrocer-*]. Type genus: *Pleiarthrocerus* Bruch, 1915.

### Tribe Protaxini

Gahan, 1906

Protaxini Gahan, 1906: 92 [stem: *Protax-*]. Type genus: *Protaxis* Gahan, 1906. Comment: current spelling maintained (Art. 29.5): incorrect original stem formation in prevailing usage (should be *Protaxe*-).

### Tribe Prothemini

Lacordaire, 1868

Prothémides Lacordaire, 1868: 524 [stem: *Prothem-*]. Type genus: *Prothema* Pascoe, 1856. Comment: original vernacular name available (Art. 11.7.2): first used in latinized form by Pascoe (1869b: 578, as Protheminae), generally accepted as in Aurivillius (1912: 291, as Prothemini).

### Tribe Psebiini

Lacordaire, 1868

*Leptidéites Fairmaire, 1864: 148 [stem: *Leptide-*]. Type genus: *Leptidea* Mulsant, 1839 [preoccupied genus name, not *Leptidea* Billberg, 1820 [Lepidoptera]; syn. of *Nathrius* Bréthes, 1916]. Comment: original vernacular name unavailable (Art. 11.7.2): subsequently used in latinized form but not generally attributed to Fairmaire (1864).
Psébiides Lacordaire, 1868: 479 [stem: *Psebi-*]. Type genus: *Psebium* Pascoe, 1864. Comment: original vernacular name available (Art. 11.7.2): first used in latinized form by Kolbe (1897: 299, as Psebiinae), generally accepted as in Aurivillius (1912: 261, as Psebiini).
Leptideina Reitter, 1913a: 24 [stem: *Leptide-*]. Type genus: *Leptidea* Mulsant, 1839 [preoccupied genus name, not *Leptidea* Billberg, 1820 [Lepidoptera]; syn. of *Nathrius* Bréthes, 1916]. Comment: permanently invalid (Art. 39): based on preoccupied type genus; Leptideini Verity, 1947 (type genus *Leptidea* Billberg, 1820) is available in Lepidoptera.
Cambaiinae Lane, 1951: 12 [stem: *Cambai-*]. Type genus: *Cambaia* Lane, 1951 [syn. of *Paraleptidea* Gounelle, 1913].
Nathriini Arnett, 1962c: 860 [stem: *Nathri-*]. Type genus: *Nathrius* Brèthes, 1916.

### Tribe Pseudocephalini

Aurivillius, 1912 (1861)

Ametrocephalitae J. Thomson, 1861: 256 [stem: *Ametrocephal-*]. Type genus: *Ametrocephala* Blanchard, 1851 [syn. of *Pseudocephalus* Newman, 1842]. Comment: use of younger name Pseudocephalini Aurivillius, 1912 conserved over this name (Art. 40.2).
Pseudocephalini Aurivillius, 1912: 154 [stem: *Pseudocephal-*]. Type genus: *Pseudocephalus* Newman, 1842. Comment: name proposed to replace Ametrocephalini J. Thomson, 1861 because of the synonymy of the type genus; usage of this name conserved over Ametrocephalini Thomson, 1861 (Art. 40.2).

### Tribe Pseudolepturini

Thomson, 1861

Pseudolepturitae J. Thomson, 1861: 146 [stem: *Pseudoleptur-*]. Type genus: *Pseudoleptura* J. Thomson, 1861. Comment: we treat this group as a valid tribe as in Adlbauer et al. (2010: 200).
Erythrinae Pascoe, 1866a: 227 [stem: *Erythr-*]. Type genus: *Erythrus* A. White, 1853.

### Tribe Psilomorphini

Lacordaire, 1868

Psilomorphides Lacordaire, 1868: 392 [stem: *Psilomorph-*]. Type genus: *Psilomorpha* Saunders, 1850. Comment: original vernacular name available (Art. 11.7.2): first used in latinized form and generally accepted as in Aurivillius (1912: 148, as Psilomorphini).

### Tribe Pteroplatini

Thomson, 1861

Pteroplatitae J. Thomson, 1861: 254 [stem: *Pteroplat-*]. Type genus: *Pteroplatus* Buquet, 1840.

### Tribe Pyrestini

Lacordaire, 1868

Pyresthides Lacordaire, 1868: 518 [stem: *Pyrest-*]. Type genus: *Pyrestes* Pascoe, 1857 [as *Pyresthes*, incorrect subsequent spelling of type genus name, not in prevailing usage]. Comment: original vernacular name available (Art. 11.7.2): first used in latinized form by Aurivillius (1912: 288, as Pyrestini), generally accepted as in Bousquet et al. (2009: 54, as Pyrestini); incorrect original stem formation, not in prevailing usage.

### Tribe Rhagiomorphini

Newman, 1841

Rhagiomorphidae Newman, 1841: 34 [stem: *Rhagiomorph-*]. Type genus: *Rhagiomorpha* Newman, 1840.

### Tribe Rhinotragini

Thomson, 1861

Rhinotragitae J. Thomson, 1861: 177 [stem: *Rhinotrag-*]. Type genus: *Rhinotragus* Germar, 1824.

### Tribe Rhopalophorini

Blanchard, 1845

Rhopalophorites Blanchard, 1845b: 152 [stem: *Rhopalophor-*]. Type genus: *Rhopalophora* Audinet-Serville, 1834. Comment: original vernacular name available (Art. 11.7.2): first used in latinized form by Blanchard (1853: 268, as Rhopalophoritae), generally accepted as in Monné (1994a: 1, as Rhopalophorini).

### Tribe Rosaliini

Fairmaire, 1864

Rosaliites Fairmaire, 1864: 137 [stem: *Rosali-*]. Type genus: *Rosalia* Audinet-Serville, 1834. Comment: original vernacular name available (Art. 11.7.2): first used in latinized form by J. L. LeConte (1873: 310, as Rosaliini), generally accepted as in Linsley (1964: 4, as Rosaliini).

### Tribe Sestyrini

Lacordaire, 1868

Sestyrides Lacordaire, 1868: 405 [stem: *Sestyr-*]. Type genus: *Sestyra* Pascoe, 1867. Comment: original vernacular name available (Art. 11.7.2): first used in latinized form by Pascoe (1869b: 643, as Sestyrinae), generally accepted as in Aurivillius (1912: 424, as Sestyrini).

### Tribe Smodicini

Lacordaire, 1868

Smodicides Lacordaire, 1868: 405 [stem: *Smodic-*]. Type genus: *Smodicum* Haldeman, 1847. Comment: original vernacular name available (Art. 11.7.2): first used in latinized form by J. L. LeConte (1873: 294, as Smodici), generally accepted as in Aurivillius (1912: 12, as Smodicini); this name was incorrectly attributed to Lacordaire (1869) by Bousquet et al. (2009: 55).

### Tribe Spintheriini

Lacordaire, 1869

Spinthériides Lacordaire, 1869: 219 [stem: *Spintheri-*]. Type genus: *Spintheria* J. Thomson, 1861. Comment: original vernacular name available (Art. 11.7.2): first used in latinized form and generally accepted as in Aurivillius (1912: 487, as Spintheriini).

### Tribe Stenhomalini

Miroshnikov, 1989

Stenhomalini Miroshnikov, 1989: 742 [stem: *Stenhomal-*]. Type genus: *Stenhomalus* A. White, 1855.

### Tribe Stenoderini

Pascoe, 1867

*Sténodérites Blanchard, 1845b: 163 [stem: *Stenoder-*]. Type genus: *Stenoderus* Dejean, 1821. Comment: original vernacular name unavailable (Art. 11.7.2): subsequently used in latinized form but not generally attributed to Blanchard (1845b).
Syllitae J. Thomson, 1864: 138 [stem: *Syllit-*]. Type genus: *Syllitus* Pascoe, 1859. Comment: this name has precedence over Stenoderini Pascoe, 1867 but has not been used as a valid name after 1899 to our knowledge, unfortunately, we were unable to provide 25 references to conserve usage of Stenoderini (Art. 23.9.2) although we believe the name Stenoderini should be conserved for this group until an application to the ICZN is submitted (Art. 23.9.3).
Stenoderinae Pascoe, 1867b: 311 [stem: *Stenoder-*]. Type genus: *Stenoderus* Dejean, 1821. Comment: senior homonym of Stenoderini Selander, 1991 (type genus *Stenodera* Eschscholtz, 1818) currently used as valid in Meloidae; this case is to be referred to the Commission to remove the homonymy (Art. 55.3.1); also, this is not the oldest available name for this tribe, see comments under Syllitae Thomson, 1864.
*Ptérosténides Lacordaire, 1868: 410 [stem: *Pterosten-*]. Type genus: *Pterostenus* Laporte, 1840 [syn. of *Stenoderus* Dejean, 1821]. Comment: original vernacular name unavailable (Art. 11.7.2): subsequently used in latinized form but not generally accepted as valid; subsequent usage of Pterosteninae by Aurivillius (1912: 150) and Lucas (1920: 53) did not validate this name because Lacordaire’s taxon was listed as a synonym of Stenoderini.
Calliprasonini McKeown, 1947: 71 [stem: *Callipras-*]. Type genus: *Calliprason* A. White, 1843. Comment: name proposed after 1930 without description or bibliographic reference to such a description (Art. 13.1), however available because it was used as valid before 2000 as in Gressitt (1959: 148, as Calliprasonini) and was not rejected by an author who, between 1961 and 1999, applied Article 13 of the then current edition of the Code (see Art. 13.2.1); incorrect original stem formation, not in prevailing usage.

### Tribe Stenopterini

Gistel, 1848

Stenopteridae Gistel, 1848: [9] [stem: *Stenopter-*]. Type genus: *Stenopterus* Illiger, 1804.

### Tribe Strongylurini

Lacordaire, 1868

Strongylurides Lacordaire, 1868: 379 [stem: *Strongylur-*]. Type genus: *Strongylurus* Hope, 1834. Comment: original vernacular name available (Art. 11.7.2): first used in latinized form by Pascoe (1869b: 548, as Strongylurinae), generally accepted as in Aurivillius (1912: 144, as Strongylurini).

### Tribe Tessarommatini

Lacordaire, 1868

Tessarommides Lacordaire, 1868: 378 [stem: *Tessarommat-*]. Type genus: *Tessaromma* Newman, 1840. Comment: original vernacular name available (Art. 11.7.2): first used in latinized form and generally accepted as in Aurivillius (1912: 143, as Tessarommatini); name also spelled Tessérommides in original publication on page 204 (key); incorrect original stem formation, not in prevailing usage.

### Tribe Thraniini

Gahan, 1906

Thraniini Gahan, 1906: 236 [stem: *Thrani-*]. Type genus: *Thranius* Pascoe, 1859.

### Tribe Thyrsiini

Marinoni and Napp, 1984

Thyrsiini Marinoni and Napp, 1984: 44 [stem: *Thyrsi-*]. Type genus: *Thyrsia* Dalman, 1819.

### Tribe Tillomorphini

Lacordaire, 1868

Tillomorphides Lacordaire, 1868: 405 [stem: *Tillomorph-*]. Type genus: *Tillomorpha* Blanchard, 1851. Comment: original vernacular name available (Art. 11.7.2): first used in latinized form by Pascoe (1869b: 554, as Tillomorphinae), generally accepted as in Adlbauer et al. (2010: 206, as Tillomorphini); this name was incorrectly credited to Pascoe, 1869 by Bousquet et al. (2009: 56).
Epipedocerini Gahan, 1906: 305 [stem: *Epipedocer-*]. Type genus: *Epipedocera* Chevrolat, 1863.

### Tribe Torneutini

Thomson, 1861

Torneutitae J. Thomson, 1861: 272 [stem: *Torneut-*]. Type genus: *Torneutes* Reich, 1838.
Thaumasidae J. Thomson, 1864: 313 [stem: *Thaumas-*]. Type genus: *Thaumasus* Reiche, 1853.

### Tribe Trachyderini

Dupont, 1836

Trachydérides Dupont, 1836: 1 [stem: *Trachyder-*]. Type genus: *Trachyderes* Dalman, 1817.

### Subtribe Ancylocerina

Thomson, 1864

Ancyloceritae J. Thomson, 1864: 210 [stem: *Ancylocer-*]. Type genus: *Ancylocera* Audinet-Serville, 1834.

### Subtribe Trachyderina

Dupont, 1836

Trachydérides Dupont, 1836: 1 [stem: *Trachyder-*]. Type genus: *Trachyderes* Dalman, 1817. Comment: original vernacular name available (Art. 11.7.2): first used in latinized form by J. Thomson (1861: 209, as Trachyderitae), generally accepted as in Monné (1994b: 16, as Trachyderini).
Purpuricenitae J. Thomson, 1861: 203 [stem: *Purpuricen-*]. Type genus: *Purpuricenus* Dejean, 1821.
Tylositae J. Thomson, 1861: 205 [stem: *Tylose-*]. Type genus: *Tylosis* J. L. LeConte, 1850. Comment: incorrect original stem formation, not in prevailing usage.
Sphaenothecitae J. Thomson, 1861: 212 [stem: *Sphaenothec-*]. Type genus: *Sphaenothecus* Dupont, 1838.
Megaderitae J. Thomson, 1861: 213 [stem: *Megader-*]. Type genus: *Megaderus* Dejean, 1821.
Eriphitae J. Thomson, 1864: 200 [stem: *Eriph-*]. Type genus: *Eriphus* Audinet-Serville, 1834.
Pteracanthitae J. Thomson, 1864: 255 [stem: *Pteracanth-*]. Type genus: *Pteracantha* Newman, 1838.
Metopocoïlitae J. Thomson, 1864: 255 [stem: *Metopocoil-*]. Type genus: *Metopocoilus* Audinet-Serville, 1832.
Sternacanthitae J. Thomson, 1864: 259 [stem: *Sternacanth-*]. Type genus: *Sternacanthus* Audinet-Serville, 1832.
Tropidosomitae J. Thomson, 1864: 256 [stem: *Tropidosomat-*]. Type genus: *Tropidosoma* Perty, 1832. Comment: incorrect original stem formation, not in prevailing usage.
Poecilopéplides Lacordaire, 1868: 404 [stem: *Poecilopepl-*]. Type genus: *Poecilopeplus* Dejean, 1835. Comment: original vernacular name available (Art. 11.7.2): first used in latinized form and generally accepted as in Aurivillius (1912: 449, as Poecilopeplini).
*Dorcacérides Lacordaire, 1868: 404 [stem: *Dorcacer-*]. Type genus: *Dorcacerus* Dejean, 1821. Comment: original vernacular name unavailable (Art. 11.7.2): subsequently used in latinized form but not generally attributed to Lacordaire (1868).
Sténaspides Lacordaire, 1868: 404 [stem: *Stenaspid-*]. Type genus: *Stenaspis* Audinet-Serville, 1834. Comment: original vernacular name available (Art. 11.7.2): first used in latinized form by Pascoe (1869b: 653, as Stenaspidinae), generally accepted as in Aurivillius (1912: 457, as Stenaspini); incorrect original stem formation, not in prevailing usage.
Paristémiides Lacordaire, 1868: 404 [stem: *Paristemi-*]. Type genus: *Paristemia* Westwood, 1841. Comment: original vernacular name available (Art. 11.7.2): first used in latinized form and generally accepted as in J. L. LeConte (1873: 309, as Paristemiini).
Dorcacerinae H. W. Bates, 1870: 430 [stem: *Dorcacer-*]. Type genus: *Dorcacerus* Dejean, 1821. Comment: Aurivillius (1912: 476) used the spelling Dorcadocerini but this was based on *Docadocerus*, which is an incorrect subsequent spelling of the type genus name.

### Tribe Tragocerini

Pascoe, 1867

Tragocerinae Pascoe, 1867a: 125 [stem: *Tragocer-*]. Type genus: *Tragocerus* Latreille, 1829.

### Tribe Trichomesiini

Aurivillius, 1912

Trichomesiini Aurivillius, 1912: 276 [stem: *Trichomesi-*]. Type genus: *Trichomesia* Pascoe, 1859.

### Tribe Tropocalymmatini

Lacordaire, 1868

Tropocalymmides Lacordaire, 1868: 408 [stem: *Tropocalymmat-*]. Type genus: *Tropocalymma* J. Thomson, 1864. Comment: original vernacular name available (Art. 11.7.2): first used in latinized form and generally accepted as in Aurivillius (1912: 150, as Tropocalymmatini); incorrect original stem formation, not in prevailing usage.

### Tribe Typhocesini

Lacordaire, 1868

Typhocésides Lacordaire, 1868: 539 [stem: *Typhoces-*]. Type genus: *Typhocesis* Pascoe, 1863. Comment: original vernacular name available (Art. 11.7.2): first used in latinized form and generally accepted as in Aurivillius (1912: 296, as Typhocesini).

### Tribe Unxiini

Napp, 2007

Unxiini Napp, 2007: 312 [stem: *Unxi-*]. Type genus: *Unxia* J. Thomson, 1861.

### Tribe Uracanthini

Blanchard, 1853

*Uracantitas Blanchard, 1851b: 475 [stem: *Uracanth-*]. Type genus: *Uracanthus* Hope, 1833 [*Uracanthus* is an incorrect subsequent spelling of *Uracantha* Hope, 1833 (p. 64), first used by Hope (1834: 108), in prevailing usage and so deemed to be the correct original spelling (Art. 33.3.1)]. Comment: original vernacular name unavailable (Art. 11.7.2): subsequently used in latinized form but not generally attributed to Blanchard (1851b); incorrect original stem formation, not in prevailing usage.
Uracanthitae Blanchard, 1853: 264 [stem: *Uracanth-*]. Type genus: *Uracanthus* Hope, 1833 [*Uracanthus* is an incorrect subsequent spelling of *Uracantha* Hope, 1833 (p. 64), first used by Hope (1834: 108), in prevailing usage and so deemed to be the correct original spelling (Art. 33.3.1)].
Rhinophthalmitae J. Thomson, 1861: 152 [stem: *Rhinophthalm-*]. Type genus: *Rhinophthalmus* J. Thomson, 1861.

### Tribe Vesperellini

Sama, 2008

Vesperellini Sama, 2008: 227 [stem: *Vesperell-*]. Type genus: *Vesperella* Dayrem, 1933.

### Tribe Xystrocerini

Blanchard, 1845

Xystrocérites Blanchard, 1845b: 147 [stem: *Xystrocer-*]. Type genus: *Xystrocera* Audinet-Serville, 1834. Comment: original vernacular name available (Art. 11.7.2): first used in latinized form by J. Thomson (1861: 249, as Xystroceritae), generally accepted as in Martins and Carvalho (1984: 214, as Xystrocerini).

### Subfamily Lamiinae

Latreille, 1825

Lamiariae Latreille, 1825: 401 [stem: *Lami-*]. Type genus: *Lamia* Fabricius, 1775.

### Tribe Acanthocinini

Blanchard, 1845

*Aedilaires Mulsant, 1839: 142 [stem: *Aedil-*]. Type genus: *Aedilis* Audinet-Serville, 1835. Comment: original vernacular name unavailable (Art. 11.7.2): not subsequently latinized.
Acanthocinites Blanchard, 1845b: 154 [stem: *Acanthocin-*]. Type genus: *Acanthocinus* Dejean, 1821. Comment: original vernacular name available (Art. 11.7.2): first used in latinized form by Blanchard (1853: 289, as Acanthocinitae), generally accepted as in Monné (1995a: 1, as Acanthocinini).
Trypanidiitae J. Thomson, 1860a: 7 [stem: *Trypanidi-*]. Type genus: *Trypanidius* Blanchard, 1846.
Dectitae J. Thomson, 1860a: 127 [stem: *Dect-*]. Type genus: *Dectes* J. L. LeConte, 1852.
*Astynomaires Mulsant, 1863b: 286 [stem: *Astynom-*]. Type genus: *Astynomus* Dejean, 1835. Comment: original vernacular name unavailable (Art. 11.7.2): subsequently used in latinized form but not generally attributed to Mulsant (1863b).
Lagocheirinae H. W. Bates, 1863: 100 [stem: *Lagocheir-*]. Type genus: *Lagocheirus* Dejean, 1835.
Liopi J. L. LeConte, 1873: 338 [stem: *Leiopod-*]. Type genus: *Leiopus* Audinet-Serville, 1835 [as *Liopus*, unjustified emendation of type genus name by Agassiz (1846b: 204), not in prevailing usage]. Comment: incorrect original stem formation, not in prevailing usage.
Graphisurini Leng, 1920: 283 [stem: *Graphisur-*]. Type genus: *Graphisurus* Kirby, 1837.
Astynomini Portevin, 1927: 39 [stem: *Astynom-*]. Type genus: *Astynomus* Dejean, 1835.

### Tribe Acanthoderini

Thomson, 1860

Acanthoderitae J. Thomson, 1860a: 5 [stem: *Acanthoder-*]. Type genus: *Acanthoderes* Audinet-Serville, 1835. Comment: First Reviser (Acanthoderini J. Thomson, 1860 vs Dryoctenini J. Thomson, 1860 vs Oreoderini J. Thomson, 1860) not determined, current usage maintained.
Dryoctenitae J. Thomson, 1860a: 29 [stem: *Dryocten-*]. Type genus: *Dryoctenes* Audinet-Serville, 1835.
Oreoderitae J. Thomson, 1860a: 29 [stem: *Oreoder-*]. Type genus: *Oreodera* Audinet-Serville, 1835.
Hoplosiae J. L. LeConte and G. H. Horn, 1883: 326 [stem: *Oplosi-*]. Type genus: *Oplosia* Mulsant, 1863 [as *Hoplosia*, unjustified emendation of type genus name by Fairmaire (1864), not in prevailing usage]. Comment: incorrect original stem formation, not in prevailing usage.

### Tribe Acmocerini

Thomson, 1864

Acmoceritae J. Thomson, 1864: 57 [stem: *Acmocer-*]. Type genus: *Acmocera* Dejean, 1835.

### Tribe Acridocephalini

Dillon and Dillon, 1959

Acridocephalidi E. S. Dillon and L. S. Dillon, 1959a: 49 [stem: *Acridocephal-*]. Type genus: *Acridocephala* Chevrolat, 1855.

### Tribe Acrocinini

Swainson, 1840

Acrocininae Swainson, 1840: 287 [stem: *Acrocin-*]. Type genus: *Acrocinus* Illiger, 1806. Comment: this family-group name was incorrectly credited to Swainson and Shuckard (1840) by Bousquet et al. (2009: 24).

### Tribe Aderpasini

Breuning and Teocchi, 1978

Aderpasini Breuning and Teocchi, 1978: 142 [stem: *Aderpas-*]. Type genus: *Aderpas* J. Thomson, 1864.

### Tribe Aerenicini

Lacordaire, 1872

Aerénicides Lacordaire, 1872: 897 [stem: *Aerenic-*]. Type genus: *Aerenica* Dejean, 1835. Comment: original vernacular name available (Art. 11.7.2): first used in latinized form by Harold (1872: 248, as Aerenicinae), generally accepted as in Aurivillius (1923: 596, as Aerenicini).

### Tribe Agapanthiini

Mulsant, 1839

Agapanthaires Mulsant, 1839: 172 [stem: *Agapanthi-*]. Type genus: *Agapanthia* Audinet-Serville, 1835. Comment: original vernacular name available (Art. 11.7.2): first used in latinized form by H. W. Bates (1884: 255, as Agapanthinae [incorrect stem formation]), generally accepted as in Aurivillius (1923: 458, as Agapanthiini); incorrect original stem formation, not in prevailing usage.
Hippopsitae J. Thomson, 1860a: 123 [stem: *Hippopse-*]. Type genus: *Hippopsis* Lepeletier and Audinet-Serville, 1828. Comment: incorrect original stem formation, not in prevailing usage.
Nemotragitae J. Thomson, 1864: 93 [stem: *Nemotrag-*]. Type genus: *Nemotragus* Westwood, 1843.
Anauxesitae J. Thomson, 1864: 94 [stem: *Anauxese-*]. Type genus: *Anauxesis* J. Thomson, 1857. Comment: incorrect original stem formation, not in prevailing usage.
Aprosopitae J. Thomson, 1864: 95 [stem: *Aprosop-*]. Type genus: *Aprosopus* Guérin-Méneville, 1844.
Aegoprepinae Pascoe, 1871: 277 [stem: *Aegoprep-*]. Type genus: *Aegoprepes* Pascoe, 1871.
Pachypézides Lacordaire, 1872: 691 [stem: *Pachypez-*]. Type genus: *Pachypeza* Audinet-Serville, 1835. Comment: original vernacular name available (Art. 11.7.2): first used in latinized form and generally accepted as in L. S. Dillon and E. S. Dillon (1945: 12, as Pachypezini).
Spalacopsides Lacordaire, 1872: 701 [stem: *Spalacopse-*]. Type genus: *Spalacopsis* Newman, 1842. Comment: original vernacular name available (Art. 11.7.2): first used in latinized form by Gahan (1890: 325, as Spalacopsinae), generally accepted as in Aurivillius (1923: 360, as Spalacopsini); incorrect original stem formation, not in prevailing usage.
Didymonychini Aurivillius, 1922b: 31 [stem: *Didymonych-*]. Type genus: *Didymonycha* Aurivillius, 1922 [syn. of *Amillarus* J. Thomson, 1857].
*Amillarinen Aurivillius, 1926a: 22 [stem: *Amillar-*]. Type genus: *Amillarus* J. Thomson, 1857. Comment: original vernacular name unavailable (Art. 11.7.2): proposed after 1899.
Hippopsiconini L. S. Dillon and E. S. Dillon, 1945: 11 [stem: *Hippopsicon-*]. Type genus: *Hippopsicon* J. Thomson, 1858.

### Tribe Amphoecini

Breuning, 1951

Amphoecini Breuning, 1951a: 5 [stem: *Amphoec-*]. Type genus: *Amphoecus* Montrouzier, 1861.

### Tribe Ancitini

Aurivillius, 1917

Ancitini Aurivillius, 1917: 28 [stem: *Ancit-*]. Type genus: *Ancita* J. Thomson, 1864.

### Tribe Ancylonotini

Lacordaire, 1869

Ancylonotides Lacordaire, 1869: 391 [stem: *Ancylonot-*]. Type genus: *Ancylonotus* Dejean, 1835. Comment: original vernacular name available (Art. 11.7.2): first used in latinized form by Pascoe (1871: 268, as Ancylonotinae), generally accepted as in Aurivillius (1922a: 152, as Ancylonotini).

### Tribe Anisocerini

Thomson, 1860

Anisoceritae J. Thomson, 1860a: 31 [stem: *Anisocer-*]. Type genus: *Anisocerus* Lacordaire, 1830.
Onychoceritae J. Thomson, 1864: 19 [stem: *Onychocer-*]. Type genus: *Onychocerus* Lacordaire, 1830.
Platysternides Lacordaire, 1872: 729 [stem: *Platystern-*]. Type genus: *Platysternus* Dejean, 1835. Comment: original vernacular name available (Art. 11.7.2): first used in latinized form and generally accepted as in Aurivillius (1923: 371, as Platysternini); junior homonym of the turtle family Platysternidae Gray, 1869 (type genus *Platysternon* Gray, 1831) which is currently used as valid; this case is to be referred to the Commission to remove the homonymy (Art. 55.3.1).

### Tribe Apomecynini

Thomson, 1860

Apomecynitae J. Thomson, 1860a: 66 [stem: *Apomecyn-*]. Type genus: *Apomecyna* Dejean, 1821. Comment: name incorrectly spelled Apomecinitae on page 3 but correct spelling used on pages 42, 66 and 68 of the original publication.
Adétides Lacordaire, 1872: 592 [stem: *Adet-*]. Type genus: *Adetus* J. L. LeConte, 1852. Comment: original vernacular name available (Art. 11.7.2): first used in latinized form and generally accepted as in Aurivillius (1922a: 288, as Adetini).
*Agennopsides Lacordaire, 1872: 592 [stem: *Agennopse-*]. Type genus: *Agennopsis* J. Thomson, 1857 [syn. of *Adetus* J. L. LeConte, 1852]. Comment: original vernacular name unavailable (Art. 11.7.2): not subsequently latinized; incorrect original stem formation, not in prevailing usage.
Ptéricoptides Lacordaire, 1872: 601 [stem: *Ptericopt-*]. Type genus: *Ptericoptus* Lacordaire, 1830. Comment: original vernacular name available (Art. 11.7.2): first used in latinized form by Gestro (1876: 140, as Ptericoptini), generally accepted as in Aurivillius (1922a: 294, as Ptericoptini).
Ectatosiides Lacordaire, 1872: 708 [stem: *Ectatosi-*]. Type genus: *Ectatosia* Pascoe, 1857. Comment: original vernacular name available (Art. 11.7.2): first used in latinized form and generally accepted as in Aurivillius (1923: 363, as Ectatosiini).
Ischiolonchides Lacordaire, 1872: 709 [stem: *Ischiolonch-*]. Type genus: *Ischioloncha* J. Thomson, 1860. Comment: original vernacular name available (Art. 11.7.2): first used in latinized form and generally accepted as in Aurivillius (1923: 364, as Ischiolonchini).

### Tribe Astathini

Thomson, 1864

*Tétraophthalmites Blanchard, 1845b: 160 [stem: *Tetraophthalm-*]. Type genus: *Tetraophthalmus* Dejean, 1835. Comment: original vernacular name unavailable (Art. 11.7.2): subsequently used in latinized form, e.g., Sama (2010: 56, as Tetraophthalmini) but not generally accepted as valid.
Astathitae J. Thomson, 1864: 117 [stem: *Astath-*]. Type genus: *Astathes* Newman, 1842 [syn. of *Tetraophthalmus* Dejean, 1835]. Comment: published before October 1864; this family-group name was also used in the same year by Pascoe (1864 [before 3 October]: 8, as Asthateinae [incorrect stem formation]); Pascoe (1864: 81, 85) refers to the publication by J. Thomson (1864) which is further evidence that the publication by Thomson was published first; this tribe name was incorrectly credited to Pascoe (1864) by Bousquet et al. (2009: 26).

### Tribe Batocerini

Thomson, 1864

Batoceritae J. Thomson, 1864: 74 [stem: *Batocer-*]. Type genus: *Batocera* Dejean, 1835.

### Tribe Calliini

Thomson, 1864

Callitae J. Thomson, 1864: 123 [stem: *Calli-*]. Type genus: *Callia* Audinet-Serville, 1835. Comment: incorrect original stem formation, not in prevailing usage.
Gryllicides Lacordaire, 1872: 902 [stem: *Gryllic-*]. Type genus: *Gryllica* J. Thomson, 1860. Comment: original vernacular name available (Art. 11.7.2): first used in latinized form by H. W. Bates (1874a: 234, as Gryllicinae), generally accepted as in Aurivillius (1923: 604, as Gryllicini).

### Tribe Ceroplesini

Thomson, 1860

Ceroplesitae J. Thomson, 1860a: 95 [stem: *Ceroples-*]. Type genus: *Ceroplesis* Audinet-Serville, 1835 [placed on the Official List of Generic Names in Zoology (ICZN 1986c)].

### Subtribe Ceroplesina

Thomson, 1860

Ceroplesitae J. Thomson, 1860a: 95 [stem: *Ceroples-*]. Type genus: *Ceroplesis* Audinet-Serville, 1835 [placed on the Official List of Generic Names in Zoology (ICZN 1986c)].

### Subtribe Crossotina

Thomson, 1864

Crossotitae J. Thomson, 1864: 64 [stem: *Crossot-*]. Type genus: *Crossotus* Audinet-Serville, 1835. Comment: downgraded to subtribe by Sama (2008: 235).
Écyroschémides Lacordaire, 1872: 503 [stem: *Ecyroschemat-*]. Type genus: *Ecyroschema* J. Thomson, 1864. Comment: original vernacular name available (Art. 11.7.2): first used in latinized form and generally accepted as in Aurivillius (1922a: 241, as Ecyroschemini); incorrect original stem formation, not in prevailing usage.
Hécyridides Lacordaire, 1872: 517 [stem: *Hecyrid-*]. Type genus: *Hecyrida* J. Thomson, 1860 [syn. of *Hecyra* J. Thomson, 1857]. Comment: original vernacular name available (Art. 11.7.2): first used in latinized form by Kolbe (1897: 318, as Hecyridinae), generally accepted as in Aurivillius (1922a: 243, as Hecyrini).
Corynofreinae Aurivillius, 1911a: 37 [stem: *Corynofre-*]. Type genus: *Corynofrea* Aurivillius, 1911.

### Tribe Cloniocerini

Lacordaire, 1872

Cloniocérides Lacordaire, 1872: 590 [stem: *Cloniocer-*]. Type genus: *Cloniocerus* Dejean, 1835. Comment: original vernacular name available (Art. 11.7.2): first used in latinized form and generally accepted as in Aurivillius (1922a: 287, as Cloniocerini).

### Tribe Colobotheini

Thomson, 1860

Colobotheitae J. Thomson, 1860a: 18 [stem: *Colobothe-*]. Type genus: *Colobothea* Lepeletier and Audinet-Serville, 1825 [*Colobothea* is an incorrect subsequent spelling of the original spelling *Colobotea*, in prevailing usage and so deemed to be the correct original spelling (Art. 33.3.1)].

### Tribe Compsosomatini

Thomson, 1857

Compsosomites J. Thomson, 1857b: 70 [stem: *Compsosomat-*]. Type genus: *Compsosoma* Lacordaire, 1830. Comment: original vernacular name available (Art. 11.7.2): first used in latinized form by J. Thomson (1860a: 34, as Compsosomitae), generally accepted as in Aurivillius (1923: 336, as Compsosomatini); incorrect original stem formation, not in prevailing usage.
Aereneites J. Thomson, 1868: 92 [stem: *Aerene-*]. Type genus: *Aerenea* J. Thomson, 1857 [*Aerenea* is an incorrect subsequent spelling of the original spelling *Aerenaea*, introduced by J. Thomson (1860a: 34), in prevailing usage and so deemed to be the correct original spelling (Art. 33.3.1)]. Comment: original vernacular name available (Art. 11.7.2): first used in latinized form by Gounelle (1908: 7, as Aereneinae), generally accepted as in Aurivillius (1923: 338, as Aereneini).

### Tribe Cyrtinini

Thomson, 1864

Cyrtinitae J. Thomson, 1864: 41 [stem: *Cyrtin-*]. Type genus: *Cyrtinus* J. L. LeConte, 1852.
Acanthomerosternoplonini Tippmann, 1956: 10 [stem: *Acanthomerosternopl-*]. Type genus: *Acanthomerosternoplon* Tippmann, 1955 [syn. of *Omosarotes* Pascoe, 1860]. Comment: incorrect original stem formation, not in prevailing usage.
Scopadini Villiers, 1980: 587 [stem: *Scopad-*]. Type genus: *Scopadus* Pascoe, 1857.

### Tribe Desmiphorini

Thomson, 1860

Desmiphoritae J. Thomson, 1860a: 74 [stem: *Desmiphor-*]. Type genus: *Desmiphora* Audinet-Serville, 1835.
*Anaesthétites Fairmaire, 1864: 166 [stem: *Anaesthet-*]. Type genus: *Anaesthetis* Dejean, 1835. Comment: original vernacular name unavailable (Art. 11.7.2): not subsequently latinized.
Métonides Lacordaire, 1869: 387 [stem: *Metont-*]. Type genus: *Meton* Pascoe, 1859. Comment: original vernacular name available (Art. 11.7.2): first used in latinized form and generally accepted as in Aurivillius (1922a: 150, as Metonini); incorrect original stem formation, not in prevailing usage.
Hebesecinae Pascoe, 1871: 277 [stem: *Hebesecid-*]. Type genus: *Hebesecis* Pascoe, 1865. Comment: incorrect original stem formation, not in prevailing usage.
Amymomides Lacordaire, 1872: 468 [stem: *Amymom-*]. Type genus: *Amymoma* Pascoe, 1866 [preoccupied genus name, not *Amymoma* Latreille, 1797 [Crustacea]; syn. of *Neoamymoma* Marinoni, 1977]. Comment: original vernacular name available (Art. 11.7.2): first used in latinized form and generally accepted as in Aurivillius (1922a: 218, as Amymomini); permanently invalid (Art. 39): based on preoccupied type genus.
Crinotarsides Lacordaire, 1872: 475 [stem: *Crinotars-*]. Type genus: *Crinotarsus* Blanchard, 1853. Comment: original vernacular name available (Art. 11.7.2): first used in latinized form and generally accepted as in Aurivillius (1922a: 229, as Crinotarsini).
Épicastides Lacordaire, 1872: 490 [stem: *Epicast-*]. Type genus: *Epicasta* J. Thomson, 1864. Comment: original vernacular name available (Art. 11.7.2): first used in latinized form and generally accepted as in Aurivillius (1922a: 237, as Epicastini).
Apodasyides Lacordaire, 1872: 623 [stem: *Apodasy-*]. Type genus: *Apodasya* Pascoe, 1863 [the genus name *Chaetosoma* Chevrolat, 1843: 367 is a senior objective synonym of *Apodasya* Pascoe and should be used as valid based on the Principle of Priority; *Chaetosoma* Chevrolat, 1843 is also a senior homonym of the well-established *Chaetosoma* Westwood, 1851, which is the type genus of Chaetosomatidae Crowson, 1952 used as valid in Cucujoidea; because the discovery of the available name *Chaetosoma* Chevrolat, 1843 causes problems for well-established names in Cucujoidea and Cerambycidae, an application was recently submitted by Bousquet and Bouchard (2010) to suppress it for the Principles of Priority and Homonymy (see Appendix 6)]. Comment: original vernacular name available (Art. 11.7.2): first used in latinized form by Kolbe (1897: 321, as Apodasinae [incorrect stem formation]), generally accepted as in Aurivillius (1922a: 305, as Apodasyini).
Nédinides Lacordaire, 1872: 635 [stem: *Nedin-*]. Type genus: *Nedine* J. Thomson, 1864. Comment: original vernacular name available (Art. 11.7.2): first used in latinized form and generally accepted as in Aurivillius (1922a: 317, as Nedinini).
Estolides Lacordaire, 1872: 636 [stem: *Estol-*]. Type genus: *Estola* Fairmaire and Germain, 1859. Comment: original vernacular name available (Art. 11.7.2): first used in latinized form by J. L. LeConte (1873: 340, as Estolae), generally accepted as in Aurivillius (1922a: 317, as Estolini).
Psenocerini J. L. LeConte, 1873: 333 [stem: *Psenocer-*]. Type genus: *Psenocerus* J. L. LeConte, 1852.
Eupogonii J. L. LeConte, 1873: 342 [stem: *Eupogoni-*]. Type genus: *Eupogonius* J. L. LeConte, 1852.
Essisini Aurivillius, 1917: 44 [stem: *Essis-*]. Type genus: *Essisus* Pascoe, 1866.
Velorini Aurivillius, 1917: 32 [stem: *Velor-*]. Type genus: *Velora* J. Thomson, 1864.

### Tribe Dorcadionini

Swainson, 1840

Dorcadioninae Swainson, 1840: 290 [stem: *Dorcadi-*]. Type genus: *Dorcadion* Dalman, 1817. Comment: this family-group name was incorrectly credited to Swainson and Shuckard (1840) by Bousquet et al. (2009: 29); current spelling maintained (Art. 29.5): incorrect original stem formation in prevailing usage (should be *Dorcadi*-; see Vives and Alonso-Zarazaga 2000: 659); tribe placed in synonymy with Lamiini by Sama (2008: 233).
Dorcadodiidae Gistel, 1856a: 376 [stem: *Dorcadodi-*]. Type genus: *Dorcadodium* Gistel, 1856 [this name is a senior synonym of *Carinatodorcadion* Breuning, 1943 (see Vives and Alonso-Zarazaga 2000: 659 for type species designation of *Dorcadodium* Gistel); we could not find enough references to treat *Dorcadodium* Gistel, 1856 as a *nomen oblitum*, however we believe that an application should be submitted to the Commission to preserve usage of *Carinatodorcadion* Breuning, 1943].

### Tribe Dorcaschematini

Thomson, 1860

Dorcaschemitae J. Thomson, 1860a: 107 [stem: *Dorcaschemat-*]. Type genus: *Dorcaschema* Haldeman, 1847. Comment: incorrect original stem formation, not in prevailing usage; name spelled Dorchaschemitae on page 104 but spelled Dorcaschemitae on pages 4 and 107 of the original publication.
Protonarthronitae J. Thomson, 1864: 57 [stem: *Protonarthr-*]. Type genus: *Protonarthron* J. Thomson, 1858. Comment: incorrect original stem formation, not in prevailing usage.

### Tribe Elytracanthinini

Bousquet, 2009

Elytracanthinae Lane, 1955: 281 [stem: *Elytracanth-*]. Type genus: *Elytracantha* Lane, 1955 [preoccupied type genus, not *Elytracantha* Kleine, 1915 [Coleoptera: Brentidae]; syn. of *Elytracanthina* Monné, 2005]. Comment: permanently invalid (Art. 39): based on preoccupied type genus.
Elytracanthinini Bousquet, 2009: 30 [stem: *Elytracanthin-*]. Type genus: *Elytracanthina* Monné, 2005. Comment: replacement name for Elytracanthinae Lane, 1955 because of the homonymy of the type genus.

### Tribe Enicodini

Thomson, 1864

Enicoditae J. Thomson, 1864: 36 [stem: *Enicod-*]. Type genus: *Enicodes* Gray, 1832. Comment: First Reviser (Enicodini J. Thomson, 1864 vs Nemaschematini J. Thomson, 1864 vs Leptonotini J. Thomson, 1864) not determined, current usage maintained.
Nemaschemitae J. Thomson, 1864: 36 [stem: *Nemaschemat-*]. Type genus: *Nemaschema* J. Thomson, 1861. Comment: incorrect original stem formation, not in prevailing usage.
Leptonotitae J. Thomson, 1864: 36 [stem: *Leptonot-*]. Type genus: *Leptonota* J. Thomson, 1861.
*Énotides Lacordaire, 1872: 487 [stem: *Enotet-*]. Type genus: *Enotes* J. Thomson, 1864. Comment: original vernacular name unavailable (Art. 11.7.2): not subsequently latinized; incorrect original stem formation, not in prevailing usage.

### Tribe Eupromerini

Galileo and Martins, 1995

Eupromerini Galileo and Martins, 1995: 132 [stem: *Eupromer-*]. Type genus: *Eupromera* Westwood, 1845.

### Tribe Forsteriini

Tippmann, 1960

Hebestolitae J. Thomson, 1864: 107 [stem: *Hebestol-*]. Type genus: *Hebestola* Blanchard, 1851 [preoccupied genus name, not *Hebestola* Haldeman, 1847 [Coleoptera: Cerambycidae: Lamiinae: Monochamini]; syn. of *Neohebestola* Marinoni, 1977]. Comment: permanently invalid (Art. 39): based on preoccupied type genus.
Forsteriini Tippmann, 1960: 210 [stem: *Forsteri-*]. Type genus: *Forsteria* Tippmann, 1960 [syn. of *Falsamblesthis* Breuning, 1959].
Falsamblesthiini Gilmour, 1961: 131 [stem: *Falsamblesth-*]. Type genus: *Falsamblesthis* Breuning, 1959. Comment: name proposed to replace Forsteriini Tippmann, 1960 because of the synonymy of the type genus; however, because the name was proposed after 1960, it cannot be maintained (Art. 40.2); incorrect original stem formation, not in prevailing usage.

### Tribe Gnomini

Thomson, 1860

Gnomitae J. Thomson, 1860a: 105 [stem: *Gnom-*]. Type genus: *Gnoma* Fabricius, 1801.

### Tribe Gyaritini

Breuning, 1950

Gyaritini Breuning, 1950c: 27 [stem: *Gyarit-*]. Type genus: *Gyaritus* Pascoe, 1858.

### Tribe Heliolini

Breuning, 1951

Heliolini Breuning, 1951a: 8 [stem: *Heliol-*]. Type genus: *Heliolus* Fauvel, 1907.

### Tribe Hemilophini

Thomson, 1868 nomen protectum

Amphionychitae J. Thomson, 1860a: 63 [stem: *Amphionych-*]. Type genus: *Amphionycha* Dejean, 1835 [syn. of *Adesmus* Lepeletier and Audinet-Serville, 1825]. Comment: *nomen oblitum* (see Bousquet et al. 2009: 31).
Hemilophitae J. Thomson, 1868: 189 [stem: *Hemiloph-*]. Type genus: *Hemilophus* Audinet-Serville, 1835. Comment: *nomen protectum* (see Bousquet et al. 2009: 31).
Itesini Lepesme, 1943: 137 [stem: *It-*]. Type genus: *Ites* C. O. Waterhouse, 1880. Comment: incorrect original stem formation, not in prevailing usage; the tribe Itini Reitter, 1913 (type genus*Ita* Tournier, 1878), which is based on the same stem, is currently used as valid in Coleoptera: Curculionidae.

### Tribe Homonoeini

Thomson, 1864

Homonaeitae J. Thomson, 1864: 35 [stem: *Homonoe-*]. Type genus: *Homonoea* Newman, 1842 [as *Homonaea*, incorrect subsequent spelling of type genus name, not in prevailing usage]. Comment: incorrect original stem formation, not in prevailing usage.
Bumétopides Lacordaire, 1872: 477 [stem: *Bumetopi-*]. Type genus: *Bumetopia* Pascoe, 1858. Comment: original vernacular name available (Art. 11.7.2): first used in latinized form by Gestro (1876: 140, as Bumetopini [incorrect stem formation]), generally accepted as in Aurivillius (1922a: 231, as Bumetopini [incorrect stem formation]); incorrect original stem formation, not in prevailing usage.

### Tribe Hyborhabdini

Aurivillius, 1911

Hyborhabdinae Aurivillius, 1911b: 22 [stem: *Hyborhabd-*]. Type genus: *Hyborhabdus* Aurivillius, 1911.

### Tribe Lamiini

Latreille, 1825

Lamiariae Latreille, 1825: 401 [stem: *Lami-*]. Type genus: *Lamia* Fabricius, 1775.
Pachystolaeidae Gistel, 1848: [9] [stem: *Pachystol-*]. Type genus: *Pachystola* Dejean, 1835 [syn. of *Lamia* Fabricius, 1775]. Comment: incorrect original stem formation, not in prevailing usage.
Phrissomitae J. Thomson, 1860a: 22 [stem: *Phrissomat-*]. Type genus: *Phrissoma* Dejean, 1835. Comment: incorrect original stem formation, not in prevailing usage; J. Thomson originally spelled the family-group name Phryssomitae on page 25, however the spelling Phrissomitae was used on pages 2 and 22 in the same publication; tribe placed in synonymy with Lamiini by Sama (2008: 233).
Morimitae J. Thomson, 1864: 77 [stem: *Morim-*]. Type genus: *Morimus* Brullé, 1832.
Potemnemini Aurivillius, 1922a: 117 [stem: *Potemnem-*]. Type genus: *Potemnemus* J. Thomson, 1864.

### Tribe Laticraniini

Lane, 1959

Laticraniinae Lane, 1959: 312 [stem: *Laticrani-*]. Type genus: *Laticranium* Lane, 1959.

### Tribe Mauesiini

Lane, 1956

Mauesinae Lane, 1956: 19 [stem: *Mauesi-*]. Type genus: *Mauesia* Lane, 1956. Comment: incorrect original stem formation, not in prevailing usage.

### Tribe Megabasini

Thomson, 1860

Megabasitae J. Thomson, 1860a: 30 [stem: *Megabas-*]. Type genus: *Megabasis* Audinet-Serville, 1835. Comment: current spelling maintained (Art. 29.5): incorrect original stem formation in prevailing usage (should be *Megabase*-).

### Tribe Mesosini

Mulsant, 1839

Mésosaires Mulsant, 1839: 165 [stem: *Mesos-*]. Type genus: *Mesosa* Latreille, 1829. Comment: original vernacular name available (Art. 11.7.2): first used in latinized form by Gistel (1848: [9], as Mesosaeidae [incorrect stem formation]), generally accepted as in López-Pérez (2006: 59, as Mesosini).

### Tribe Microcymaturini

Breuning and Teocchi, 1985

Microcymaturini Breuning and Teocchi, 1985: 155 [stem: *Microcymatur-*]. Type genus: *Microcymatura* Breuning, 1950.

### Tribe Moneilemini

Thomson, 1864

Moneilemitae J. Thomson, 1864: 43 [stem: *Moneilem-*]. Type genus: *Moneilema* Say, 1824. Comment: current spelling maintained (Art. 29.5): incorrect original stem formation in prevailing usage (should be *Moneilemat*-).

### Tribe Monochamini

Gistel, 1848

Monohammidae Gistel, 1848: [9] [stem: *Monocham-*]. Type genus: *Monochamus* Dejean, 1821 [as *Monohammus*, unjustified emendation of type genus name by Dejean (1835: 340), not in prevailing usage]. Comment: incorrect original stem formation, not in prevailing usage.
Taeniotitae J. Thomson, 1864: 76 [stem: *Taeniot-*]. Type genus: *Taeniotes* Audinet-Serville, 1835.
Agnitae J. Thomson, 1864: 83 [stem: *Agni-*]. Type genus: *Agnia* Newman, 1842. Comment: incorrect original stem formation, not in prevailing usage.
Geranitae J. Thomson, 1864: 93 [stem: *Gerani-*]. Type genus: *Gerania* Audinet-Serville, 1835. Comment: incorrect original stem formation, not in prevailing usage.
Ptychodes J. L. LeConte, 1873: 335 [stem: *Ptychod-*]. Type genus: *Ptychodes* Audinet-Serville, 1835.
Goes J. L. LeConte, 1873: 335 [stem: *Goet-*]. Type genus: *Goes* J. L. LeConte, 1852. Comment: incorrect original stem formation, not in prevailing usage.
Docohammidi E. S. Dillon and L. S. Dillon, 1959b: 7 [stem: *Docohamm-*]. Type genus: *Docohammus* Aurivillius, 1908.

### Tribe Morimonellini

Lobanov, Danilevsky and Murzin, 1981

Morimonellini Lobanov et al., 1981: 790 [stem: *Morimonell-*]. Type genus: *Morimonella* Podaný, 1979. Comment: description by indication (distinguishing characters given in Podany (1979: 43, as Oligorchini)).

### Tribe Morimopsini

Lacordaire, 1869

Morimopsides Lacordaire, 1869: 289 [stem: *Morimops-*]. Type genus: *Morimopsis* J. Thomson, 1857. Comment: original vernacular name available (Art. 11.7.2): first used in latinized form and generally accepted as in Aurivillius (1922a: 64, as Morimopsini); current spelling maintained (Art. 29.5): incorrect original stem formation in prevailing usage (should be *Morimopse*-).

### Tribe Nyctimeniini

Gressitt, 1951

Nyctimenitae J. Thomson, 1864: 94 [stem: *Nyctimen-*]. Type genus: *Nyctimene* J. Thomson, 1857 [preoccupied genus name, not *Nyctimene* Borkenhausen, 1797 [Mammalia]; syn. of *Nyctimenius* Gressitt, 1951]. Comment: permanently invalid (Art. 39): based on preoccupied type genus.
Nyctimeniini Gressitt, 1951: 629 [stem: *Nyctimeni-*]. Type genus: *Nyctimenius* Gressitt, 1951. Comment: replacement name for Nyctimenini J. Thomson, 1864 because of the homonymy of the type genus.

### Tribe Obereini

Thomson, 1864

Obereitae J. Thomson, 1864: 117 [stem: *Obere-*]. Type genus: *Oberea* Dejean, 1835. Comment: published before October 1864; this family-group name was also used in the same year by Pascoe (1864 [before 3 October]: 8, as Obereinae); Pascoe (1864: 81, 85) refers to the publication by J. Thomson (1864) which is further evidence that the publication by Thomson was published first; this tribe name was incorrectly credited to Pascoe (1864) by Bousquet et al. (2009: 33).

### Tribe Oculariini

Breuning, 1950

Oculariini Breuning, 1950a: 263 [stem: *Oculari-*]. Type genus: *Ocularia* Jordan, 1894.

### Tribe Onciderini

Thomson, 1860

Oncideritae J. Thomson, 1860a: 38 [stem: *Oncider-*]. Type genus: *Oncideres* Lacordaire, 1830 [*Oncideres* is an incorrect subsequent spelling of *Oncyderes* Lacordaire, 1830, introduced by Audinet-Serville (1835: 67), in prevailing usage and attributed to Lacordaire (1830b), e.g., Monné (2005b: 571), and so deemed to be the correct original spelling (Art. 33.3.1)]. Comment: First Reviser (Onciderini J. Thomson, 1860 vs Hypsiomatini J. Thomson, 1860) not determined, current usage maintained.
Hypsiomitae J. Thomson, 1860a: 109 [stem: *Hypsiomat-*]. Type genus: *Hypsioma* Audinet-Serville, 1835. Comment: incorrect original stem formation, not in prevailing usage.
Hypselominae Pascoe, 1864: 7 [stem: *Hypselom-*]. Type genus: *Hypselomus* Perty, 1832.

### Tribe Oncideropsidini

Aurivillius, 1922

Oncideropsidini Aurivillius, 1922c: 165 [stem: *Oncideropsid-*]. Type genus: *Oncideropsis* Aurivillius, 1922. Comment: current spelling maintained (Art. 29.5): incorrect original stem formation in prevailing usage (should be *Oncideropse*-).

### Tribe Onocephalini

Thomson, 1860

Onocephalitae J. Thomson, 1860a: 120 [stem: *Onocephal-*]. Type genus: *Onocephala* J. Thomson, 1857.

### Tribe Onychogleneini

Aurivillius, 1923

Onychogleneini Aurivillius, 1923: 513 [stem: *Onychoglene-*]. Type genus: *Onychoglenea* Aurivillius, 1922.

### Tribe Parmenini

Mulsant, 1839

Parménaires Mulsant, 1839: 118 [stem: *Parmen-*]. Type genus: *Parmena* Dejean, 1821. Comment: original vernacular name available (Art. 11.7.2): first used in latinized form by J. Thomson (1864: 38, as Parmenitae), generally accepted as in Villiers (1978: 449, as Parmenini).
Hexathricitae J. Thomson, 1864: 38 [stem: *Hexatrich-*]. Type genus: *Hexatricha* A. White, 1846 [as *Hexathrica*, incorrect subsequent spelling of type genus name, not in prevailing usage]. Comment: the spelling Hexarthricitae (page 38) was corrected to Hexathricitae on page 339 and in the errata (page 483) of the same work; the corrected spelling represents an incorrect original stem formation, not in prevailing usage.
*Dorcadidides Lacordaire, 1869: 257 [stem: *Dorcadid-*]. Type genus: *Dorcadida* A. White, 1846. Comment: original vernacular name unavailable (Art. 11.7.2): subsequently used in latinized form, e.g., Heyne and Taschenberg (1907: 241, as Dorcadidini), but not generally accepted as valid.

### Tribe Petrognathini

Blanchard, 1845

Pétrognathites Blanchard, 1845b: 160 [stem: *Petrognath-*]. Type genus: *Petrognatha* Leach, 1819. Comment: original vernacular name available (Art. 11.7.2): first used in latinized form by Kolbe (1897: 316, as Petrognathinae), generally accepted as in Aurivillius (1922a: 205, as Petrognathini).
*Omacanthides Lacordaire, 1872: 447 [stem: *Omacanth-*]. Type genus: *Omacantha* Audinet-Serville, 1835 [syn. of *Petrognatha* Leach, 1819]. Comment: original vernacular name unavailable (Art. 11.7.2): subsequently used in latinized form, e.g., Heyne and Taschenberg (1907: 243, as Omacanthini), but not generally accepted as valid; Omacanthidae was used as valid by Ienistea (1986: 30) but it was not attributed to Lacordaire (1872); Ienistea’s name is also unavailable, it was proposed after 1930 without description or bibliographic reference to such a description (Art. 13.1).

### Tribe Phacellini

Lacordaire, 1872

Phacellides Lacordaire, 1872: 664 [stem: *Phacell-*]. Type genus: *Phacellus* Dejean, 1835. Comment: original vernacular name available (Art. 11.7.2): first used in latinized form by Gounelle (1908: 7, as Phacellinae), generally accepted as in Aurivillius (1923: 339, as Phacellini).

### Tribe Phantasini

Kolbe, 1897

*Phantasides Lacordaire, 1869: 285 [stem: *Phantas-*]. Type genus: *Phantasis* J. Thomson, 1860. Comment: original vernacular name unavailable (Art. 11.7.2): subsequently used in latinized form but not generally attributed to Lacordaire (1869).
Phantasinae Kolbe, 1897: 306 [stem: *Phantas-*]. Type genus: *Phantasis* J. Thomson, 1860. Comment: current spelling maintained (Art. 29.5): incorrect original stem formation in prevailing usage (should be *Phantase*-).
Phantasini Hunt and Breuning, 1957: 51 [stem: *Phantas-*]. Type genus: *Phantasis* J. Thomson, 1860. Comment: family group name proposed as new without reference to Phantasinae Kolbe, 1897; current spelling maintained (Art. 29.5): incorrect original stem formation in prevailing usage (should be *Phantase*-).

### Tribe Phrynetini

Thomson, 1864

Phrynetitae J. Thomson, 1864: 71 [stem: *Phrynet-*]. Type genus: *Phryneta* Dejean, 1835.

### Tribe Phymasternini

Teocchi, 1989

Phymasternini Teocchi, 1989: 4 [stem: *Phymastern-*]. Type genus: *Phymasterna* Laporte, 1840 [for comments on problems with the authorship and type species of this type genus see Bousquet et al. (2009: 35)].

### Tribe Phytoeciini

Mulsant, 1839

Phytoeciaires Mulsant, 1839: 191 [stem: *Phytoeci-*]. Type genus: *Phytoecia* Dejean, 1835. Comment: original vernacular name available (Art. 11.7.2): first used in latinized form by Pascoe (1864: 8, as Phytoeciinae), generally accepted as in Villiers (1978: 521, as Phytoeciini).

### Tribe Pogonocherini

Mulsant, 1839

Pogonochéraires Mulsant, 1839: 151 [stem: *Pogonocher-*]. Type genus: *Pogonocherus* Dejean, 1821. Comment: original vernacular name available (Art. 11.7.2): first used in latinized form by J. L. LeConte (1873: 340, as Pogonocherini), generally accepted as in Villiers (1978: 465, as Pogonocherini).
*Exocentrites Fairmaire, 1864: 157 [stem: *Exocentr-*]. Type genus: *Exocentrus* Dejean, 1835. Comment: published early September 1864; original vernacular name unavailable (Art. 11.7.2): subsequently used in latinized form but not generally attributed to Fairmaire (1864).
Exocentrinae Pascoe, 1864: 7 [stem: *Exocentr-*]. Type genus: *Exocentrus* Dejean, 1835. Comment: published 3 October 1864.
Zaploi J. L. LeConte and G. H. Horn, 1883: 327 [stem: *Zaplo-*]. Type genus: *Zaplous* J. L. LeConte, 1878.

### Tribe Polyrhaphidini

Thomson, 1860

Polyrhaphitae J. Thomson, 1860a: 30 [stem: *Polyrhaphid-*]. Type genus: *Polyrhaphis* Audinet-Serville, 1835. Comment: incorrect original stem formation, not in prevailing usage.

### Tribe Pretiliini

Martins and Galileo, 1990

Pretiliini Martins and Galileo, 1990: 705, in key [stem: *Pretili-*]. Type genus: *Pretilia* H. W. Bates, 1866.

### Tribe Proctocerini

Aurivillius, 1922

*Cliniides Lacordaire, 1872: 424 [stem: *Clini-*]. Type genus: *Clinia* J. Thomson, 1857 [syn. of *Proctocera* Chevrolat, 1855]. Comment: original vernacular name unavailable (Art. 11.7.2): subsequently used in latinized form, e.g., Breuning (1950d: 411, as Cliniini), but not generally accepted as valid; Cliniidae was used as valid by Ienistea (1986: 30) but it was not attributed to Lacordaire (1872); Ienistea’s name is also unavailable, it was proposed after 1930 without description or bibliographic reference to such a description (Art. 13.1).
Proctocerini Aurivillius, 1922a: 182 [stem: *Proctocer-*]. Type genus: *Proctocera* Chevrolat, 1855.

### Tribe Prosopocerini

Thomson, 1864

Prosopoceritae J. Thomson, 1864: 72 [stem: *Prosopocer-*]. Type genus: *Prosopocera* Blanchard, 1845.

### Tribe Pteropliini

Thomson, 1860

Pteropliitae J. Thomson, 1860a: 73 [stem: *Pteropli-*]. Type genus: *Pteroplius* Lacordaire, 1830 [as *Pteroplia*, incorrect subsequent spelling of type genus name, not in prevailing usage; *Pteroplius* is an incorrect subsequent spelling of *Pterhoplius* Lacordaire, 1830, introduced by Audinet-Serville (1835: 65), in prevailing usage and attributed to Lacordaire (1830b), e.g., Monné (2005b: 295), and so deemed to be the correct original spelling (Art. 33.3.1)].
Abrynitae J. Thomson, 1864: 44 [stem: *Abryn-*]. Type genus: *Abryna* Newman, 1842.
Protorhopalitae J. Thomson, 1864: 69 [stem: *Protorhopal-*]. Type genus: *Protorhopala* J. Thomson, 1860.
Niphoninae Pascoe, 1864: 56 [stem: *Niphon-*]. Type genus: *Niphona* Mulsant, 1839.
Ataxiides Lacordaire, 1872: 597 [stem: *Ataxi-*]. Type genus: *Ataxia* Haldeman, 1847. Comment: original vernacular name available (Art. 11.7.2): first used in latinized form by J. L. LeConte (1873: 344, as Ataxiini), generally accepted as in Aurivillius (1922a: 291, as Ataxiini).
Emphytoeciides Lacordaire, 1872: 713 [stem: *Emphytoeci-*]. Type genus: *Emphytoecia* Fairmaire and Germain, 1860. Comment: original vernacular name available (Art. 11.7.2): first used in latinized form and generally accepted as in Aurivillius (1923: 365, as Emphytoeciini).
Baroeides Lacordaire, 1872: 439 [stem: *Barae-*]. Type genus: *Baraeus* J. Thomson, 1858 [as *Baroeus*, incorrect subsequent spelling of type genus name, not in prevailing usage]. Comment: also spelled Baréides on page 414 of the same work; original vernacular name available (Art. 11.7.2): first used in latinized form and generally accepted as in Aurivillius (1922a: 206, as Baraeini).
Atossides Lacordaire, 1872: 496 [stem: *Atoss-*]. Type genus: *Atossa* J. Thomson, 1864 [syn. of *Grammoechus* J. Thomson, 1864]. Comment: original vernacular name available (Art. 11.7.2): first used in latinized form and generally accepted as in Aurivillius (1922a: 149, as Atossini).
Metagnomini Aurivillius, 1925: 13 [stem: *Metagnom-*]. Type genus: *Metagnoma* Aurivillius, 1925.

### Tribe Rhodopinini

Gressitt, 1951

Rhodopides Lacordaire, 1872: 450 [stem: *Rhodopid-*]. Type genus: *Rhodopis* J. Thomson, 1857 [preoccupied genus name, not *Rhodopis* Reichenbach, 1854 [Aves]; syn. of *Rhodopina* Gressit, 1951]. Comment: original vernacular name available (Art. 11.7.2): first used in latinized form and generally accepted as in Aurivillius (1922a: 210, as Rhodopini); incorrect original stem formation, not in prevailing usage; permanently invalid (Art. 39): based on preoccupied type genus.
Rhodopinini Gressitt, 1951: 439 [stem: *Rhodopin-*]. Type genus: *Rhodopina* Gressitt, 1951. Comment: replacement name for Rhodopini Lacordaire, 1872 because of the homonymy of the type genus.

### Tribe Saperdini

Mulsant, 1839

Saperdaires Mulsant, 1839: 181 [stem: *Saperd-*]. Type genus: *Saperda* Fabricius, 1775 [placed on the Official List of Generic Names in Zoology (ICZN 1980)]. Comment: original vernacular name available (Art. 11.7.2): first used in latinized form by Blanchard (1853: 299, as Saperditae), generally accepted as in Aurivillius (1923: 468, as Saperdini).
Gleneïtae J. Thomson, 1864: 123 [stem: *Glene-*]. Type genus: *Glenea* Newman, 1842 [for comments on problems with the authorship and type species of this type genus see Bousquet et al. (2009: 37)].

### Tribe Stenobiini

Breuning, 1950

Stenobiini Breuning, 1950b: 305 [stem: *Stenobi-*]. Type genus: *Stenobia* Lacordaire, 1872.

### Tribe Sternotomini

Thomson, 1860

*Stellognathites Blanchard, 1845b: 158 [stem: *Stellognath-*]. Type genus: *Stellognatha* Dejean, 1835. Comment: original vernacular name unavailable (Art. 11.7.2): not subsequently latinized.
Sternotomitae J. Thomson, 1860a: 87 [stem: *Sternotom-*]. Type genus: *Sternotomis* Percheron, 1836 [*Sternodonta* Dejean, 1835, a senior synonym of *Sternotomis* Percheron, 1836, is a *nomen oblitum* and *Sternotomis* is a *nomen protectum* following Sama (2009a: 24)]. Comment: current spelling maintained (Art. 29.3.1.1): incorrect original stem formation in prevailing usage (should be *Sternotomid*-).

### Tribe Tapeinini

Thomson, 1857

Tapeinites J. Thomson, 1857b: 41 [stem: *Tapein-*]. Type genus: *Tapeina* Lepeletier and Audinet-Serville, 1828. Comment: original vernacular name available (Art. 11.7.2): first used in latinized form by J. Thomson (1860a: 73, as Tapeinitae), generally accepted as in Aurivillius (1922a: 236, as Tapeinini).

### Tribe Tetraopini

Thomson, 1860

Tetraopesitae J. Thomson, 1860a: 66 [stem: *Tetraop-*]. Type genus: *Tetraopes* Schönherr, 1817. Comment: incorrect original stem formation, not in prevailing usage.

### Tribe Tetraulaxini

Breuning and Teocchi, 1977

Tetraulaxini Breuning and Teocchi, 1977: 881 [stem: *Tetraulax-*]. Type genus: *Tetraulax* Jordan, 1903. Comment: current spelling maintained (Art. 29.5): incorrect original stem formation in prevailing usage (should be *Tetraulac*-).

### Tribe Tetropini

Portevin, 1927

*Polyopsiates Mulsant, 1863b: 340 [stem: *Polyopsi-*]. Type genus: *Polyopsia* Mulsant, 1839 [syn. of *Tetrops* Stephens, 1829]. Comment: original vernacular name unavailable (Art. 11.7.2): not subsequently latinized.
*Tétropides Planet, 1924: 326 [stem: *Tetrop-*]. Type genus: *Tetrops* Stephens, 1829 [for comments on problems with the authorship and type species of this type genus see Bousquet et al. (2009: 38)]. Comment: original vernacular name unavailable (Art. 11.7.2): proposed after 1899; the argument of Sama (2008: 240) that Planet’s name is available, even if published after 1900 in a vernacular form, because it was used as valid in a latinized form and credited to Planet (1924) by Vives (2000: 508) is incorrect (see Bousquet et al. 2009: 38).
Tetropini Portevin, 1927: 39, in key [stem: *Tetrop-*]. Type genus: *Tetrops* Stephens, 1829 [for comments on problems with the authorship and type species of this type genus see Bousquet et al. (2009: 38)].

### Tribe Theocrini

Lacordaire, 1872

Théocrides Lacordaire, 1872: 494 [stem: *Theocr-*]. Type genus: *Theocris* J. Thomson, 1858. Comment: original vernacular name available (Art. 11.7.2): first used in latinized form by Gahan (1890: 322, as Theocrinae), generally accepted as in Aurivillius (1922a: 238, as Theocridini [incorrect stem formation]).

### Tribe Tmesisternini

Blanchard, 1853

Tmesisternitae Blanchard, 1853: 274 [stem: *Tmesistern-*]. Type genus: *Tmesisternus* Latreille, 1829.
Spingnothitae J. Thomson, 1864: 31 [stem: *Sphingnot-*]. Type genus: *Sphingnotus* Perroud, 1855 [as *Spingnothus*, incorrect subsequent spelling of type genus name, not in prevailing usage]. Comment: incorrect original stem formation, not in prevailing usage.
Ichthyosomitae J. Thomson, 1864: 33 [stem: *Ichthyosom-*]. Type genus: *Ichthyosomus* Boisduval, 1835.
*Arsysiides Lacordaire, 1872: 479 [stem: *Arsysi-*]. Type genus: *Arsysia* Pascoe, 1867 [syn. of *Trigonoptera* Perroud, 1855]. Comment: original vernacular name unavailable (Art. 11.7.2): subsequently used in latinized form, e.g., Gestro (1876: 140, as Arsysiini), but not generally accepted as valid; Arsysiidae was used as valid by Ienistea (1986: 30) but it was not attributed to Lacordaire (1872); Ienistea’s name is also unavailable, it was proposed after 1930 without description or bibliographic reference to such a description (Art. 13.1).
Trigonopterini Aurivillius, 1922a: 229 [stem: *Trigonopter-*]. Type genus: *Trigonoptera* Perroud, 1855. Comment: replacement name for Arsysiides Lacordaire, 1872 because of the synonymy of the type genus.

### Tribe Tragocephalini

Thomson, 1857

Tragocephalites J. Thomson, 1857b: 26 [stem: *Tragocephal-*]. Type genus: *Tragocephala* Dejean, 1835. Comment: original vernacular name available (Art. 11.7.2): first used in latinized form by J. Thomson (1860a: 88, as Tragocephalitae), generally accepted as in Aurivillius (1922a: 171, as Tragocephalini).

### Tribe Xenicotelini

Matsushita, 1933

Xenicotelini Matsushita, 1933: 346 [stem: *Xenicotel-*]. Type genus: *Xenicotela* H. W. Bates, 1884.

### Tribe Xenofreini

Aurivillius, 1923

Xenofreini Aurivillius, 1923: 375 [stem: *Xenofre-*]. Type genus: *Xenofrea* H. W. Bates, 1885. Comment: this name has been attributed to H. W. Bates (1885: 373) in the literature but we did not find such a name in the Biologia Centrali-Americana or other publications by the same author.

### Tribe Xenoleini

Lacordaire, 1872

Xénoléides Lacordaire, 1872: 460 [stem: *Xenole-*]. Type genus: *Xenolea* J. Thomson, 1864. Comment: original vernacular name available (Art. 11.7.2): first used in latinized form and generally accepted as in Aurivillius (1922a: 216, as Xenoleini).

### Tribe Xylorhizini

Lacordaire, 1872

Xylorhizides Lacordaire, 1872: 443 [stem: *Xylorhiz-*]. Type genus: *Xylorhiza* Dejean, 1835. Comment: original vernacular name available (Art. 11.7.2): first used in latinized form by Kolbe (1897: 316, as Xylorrhizinae [incorrect stem formation]), generally accepted as in Aurivillius (1922a: 208, as Xylorhizini).

### Tribe Zygocerini

Thomson, 1864

Zygoceritae J. Thomson, 1864: 87 [stem: *Zygocer-*]. Type genus: *Zygocera* Erichson, 1842.
Disterninae Pascoe, 1871: 268 [stem: *Distern-*]. Type genus: *Disterna* J. Thomson, 1864.

### Family Megalopodidae

Latreille, 1802

Megalopides Latreille, 1802: 227 [stem: *Megalopod-*]. Type genus: *Megalopus* Fabricius, 1801.

### Subfamily Megalopodinae

Latreille, 1802

Megalopides Latreille, 1802: 227 [stem: *Megalopod-*]. Type genus: *Megalopus* Fabricius, 1801. Comment: incorrect original stem formation, not in prevailing usage.

### Subfamily Palophaginae

Kuschel and May, 1990

Palophaginae Kuschel and May, 1990: 699 [stem: *Palophag-*]. Type genus: *Palophagus* Kuschel, 1990.

### Subfamily Zeugophorinae

Böving and Craighead, 1931

Zeugophorinae Böving and Craighead, 1931: 63, in key [stem: *Zeugophor-*]. Type genus: *Zeugophora* Kunze, 1818 [placed on the Official List of Generic Names in Zoology (ICZN 1986a)].

### Family Orsodacnidae

Thomson, 1859

Orsodachnidae C. G. Thomson, 1859: 154 [stem: *Orsodacn-*]. Type genus: *Orsodacne* Latreille, 1802 [placed on the Official List of Generic Names in Zoology (ICZN 2002a)].

### Subfamily Orsodacninae

Thomson, 1859

Orsodachnidae C. G. Thomson, 1859: 154 [stem: *Orsodacn-*]. Type genus: *Orsodacne* Latreille, 1802 [placed on the Official List of Generic Names in Zoology (ICZN 2002a)]. Comment: incorrect original stem formation, not in prevailing usage.

### Subfamily Aulacoscelidinae

Chapuis, 1874

Aulacoscélites Chapuis, 1874: 54 [stem: *Aulacoscelid-*]. Type genus: *Aulacoscelis* Duponchel et Chevrolat, 1842. Comment: original vernacular name available (Art. 11.7.2): first used in latinized form by Heyne and Taschenberg (1907: 245, as Aulacoscelinae), generally accepted as in Monrós (1959a: 18, as Aulacoscelidinae); incorrect original stem formation, not in prevailing usage; a recent application by Santiago-Blay (2008) to conserve usage of the stem *Aulacoscel*- was not approved by the Commission (ICZN 2010b).

### Family Chrysomelidae

Latreille, 1802

Chrysomelinae Latreille, 1802: 220 [stem: *Chrysomel-*]. Type genus: *Chrysomela* Linnaeus, 1758 [placed on the Official List of Generic Names in Zoology (ICZN 1984c)]. Comment: First Reviser (Chrysomelidae Latreille, 1802 vs Bruchidae Latreille, 1802 vs Galerucidae Latreille, 1802) not determined, current usage maintained.

### Subfamily Sagrinae

Leach, 1815

Sagrida Leach, 1815: 113 [stem: *Sagr-*]. Type genus: *Sagra* Fabricius, 1792.

### Tribe Carpophagini

Chapuis, 1874

Carpophagites Chapuis, 1874: 36 [stem: *Carpophag-*]. Type genus: *Carpophagus* W. S. MacLeay, 1827. Comment: original vernacular name available (Art. 11.7.2): first used in latinized form by Jacoby (1903: 4, as Carpophaginae), generally accepted as in Clavareau (1913a: 6, as Carpophagini); the older name Carpophaginae Selby, 1835 (type genus *Carpophaga* Selby, 1835) is available in Aves (see Bock 1994: 139); this case is to be referred to the Commission to remove the homonymy (Art. 55.3.1).

### Tribe Diaphanopsidini

Monrós, 1958

Diaphanopsidini Monrós, 1958a: 7 [stem: *Diaphanopsid-*]. Type genus: *Diaphanops* Schönherr, 1845. Comment: name proposed after 1930 without description or bibliographic reference to such a description (Art. 13.1), however available because it was used as valid before 2000 as in Seeno and Wilcox (1982: 17, 18) and was not rejected by an author who, between 1961 and 1999, applied Article 13 of the then current edition of the Code (see Art. 13.2.1); current spelling maintained (Art. 29.5): incorrect stem formation in prevailing usage (should be *Diaphanop*-).

### Tribe Megamerini

Chapuis, 1874

Mégamérites Chapuis, 1874: 30 [stem: *Megamer-*]. Type genus: *Megamerus* W. S. MacLeay, 1827. Comment: original vernacular name available (Art. 11.7.2): first used in latinized form by Jacoby (1903: 2, as Megamerinae), generally accepted as in Clavareau (1913a: 4, as Megamerini); First Reviser (Megamerini Chapuis, 1874 vs Ametallini Chapuis, 1874 vs Mecynoderini Chapuis, 1874) not determined, current usage maintained.
Amétallites Chapuis, 1874: 46 [stem: *Ametall-*]. Type genus: *Ametalla* Hope, 1840. Comment: original vernacular name available (Art. 11.7.2): first used in latinized form by Jacoby (1903: 1, as Ametallinae), generally accepted as in Clavareau (1913a: 12, as Ametallini).
Mécynodérites Chapuis, 1874: 44 [stem: *Mecynoder-*]. Type genus: *Mecynodera* Hope, 1840. Comment: original vernacular name available (Art. 11.7.2): first used in latinized form by Jacoby (1903: 1, as Mecynoderinae), generally accepted as in Clavareau (1913a: 11, as Mecynoderini).

### Tribe Sagrini

Leach, 1815

Sagrida Leach, 1815: 113 [stem: *Sagr-*]. Type genus: *Sagra* Fabricius, 1792.

### Subfamily Bruchinae

Latreille, 1802

Bruchelae Latreille, 1802: 192 [stem: *Bruch-*]. Type genus: *Bruchus* Linnaeus, 1767 [placed on the Official List of Generic Names in Zoology and older name *Bruchus* Geoffroy, 1762 placed on the Official Index of Rejected and Invalid Generic Names in Zoology (ICZN 1995a)]. Comment: Bruchidae Latreille, 1802 placed on the Official List of Family-Group Names in Zoology (ICZN 1995a).

### Tribe Amblycerini

Bridwell, 1932

Amblycerinae Bridwell, 1932: 103, in key [stem: *Amblycer-*]. Type genus: *Amblycerus* Thunberg, 1815.

### Subtribe Amblycerina

Bridwell, 1932

Spermophagidae Crotch, 1873b: 93 [stem: *Spermophag-*]. Type genus: *Spermophagus* sensu G. H. Horn, 1873 [not *Spermophagus* Schönherr, 1833; syn. of *Amblycerus* Thunberg, 1815]. Comment: based on a misidentified type genus, name treated here as invalid until an application is submitted to the Commission to suppress it for the Principles of Priority and Homonymy (Art. 65.2.1); also see Spermophagini Borowiec, 1987 below.
Amblycerinae Bridwell, 1932: 103, in key [stem: *Amblycer-*]. Type genus: *Amblycerus* Thunberg, 1815. Comment: although this is the oldest name for the tribe, we recommend that an application be sent to the Commission in order to conserve usage of Amblycerina Bridwell, 1932 because the older name Spermophagina Crotch, 1873 is based on a misidentified type genus (Art. 65.2.1); MAAZ and CHCL will submit an application to the Commission in order to conserve the current concept of *Amblycerus* Thunberg, 1815 following the discovery of an overlooked type species designation that would alter the concept of the genus (also see Alonso-Zarazaga and Lyal (1999: 8, 23)).

### Subtribe Spermophagina

Borowiec, 1987

Spermophagini Borowiec, 1987: 27 [stem: *Spermophag-*]. Type genus: *Spermophagus* Schönherr, 1833. Comment: family-group name proposed as new without reference to Spermophagidae Crotch, 1873; an application needs to be submitted to the Commission to suppress Spermophagidae Crotch, 1873 (based on the misidentified type genus *Spermophagus* sensu Horn, 1873) for the Principles of Priority and Homonymy (Art. 65.2.1) to conserve this name as valid.

### Tribe Bruchini

Latreille, 1802

Bruchelae Latreille, 1802: 192 [stem: *Bruch-*]. Type genus: *Bruchus* Linnaeus, 1767 [placed on the Official List of Generic Names in Zoology and older name *Bruchus* Geoffroy, 1762 placed on the Official Index of Rejected and Invalid Generic Names in Zoology (ICZN 1995a)]. Comment: Bruchidae Latreille, 1802 placed on the Official List of Family-Group Names in Zoology (ICZN 1995a).

### Subtribe Acanthoscelidina

Bridwell, 1946

Acanthoscelidini Bridwell, 1946: 54, in key [stem: *Acanthoscelid-*]. Type genus: *Acanthoscelides* Schilsky, 1905. Comment: First Reviser (Acanthoscelidina Bridwell, 1946 vs Bruchidiina Bridwell, 1946) not determined, current usage maintained; description of family-group name not unequivocal (Art. 13.1.1) but name treated as available (Art. 13.2.1); Bridwell (1946: 53) mentioned that he wanted to establish the two new tribes Bruchidiini and Acanthoscelidini but he followed by saying that it was “premature to attempt a diagnosis” of these tribes; both tribal names were included in his key (p. 54) but their identification was not unequivocal; Bradley (1947: 37) noted that the new tribes Bruchidiini and Acanthoscelidini were not diagnosed adequately by Bridwell (1946), however he did not cite Article 13 of the then current edition of the Code of Zoological nomenclature to treat those names as unavailable; since Bruchidiini and Acanthoscelidini have been used as valid and attributed to Bridwell (1946) by subsequent authors, e.g., Bottimer (1968: 1028 and 1015 respectively), we have also treated those names as available here.
Bruchidiini Bridwell, 1946: 54, in key [stem: *Bruchidi-*]. Type genus: *Bruchidius* Schilsky, 1905. Comment: description of family-group name not unequivocal (Art. 13.1.1) but name treated as available (Art. 13.2.1); see comment under Acanthoscelidini Bridwell, 1946.

### Subtribe Bruchina

Latreille, 1802

Bruchelae Latreille, 1802: 192 [stem: *Bruch-*]. Type genus: *Bruchus* Linnaeus, 1767 [placed on the Official List of Generic Names in Zoology and older name *Bruchus* Geoffroy, 1762 placed on the Official Index of Rejected and Invalid Generic Names in Zoology (ICZN 1995a)]. Comment: Bruchidae Latreille, 1802 placed on the Official List of Family-Group Names in Zoology (ICZN 1995a).
Lariidae Bedel, 1901: 341 [stem: *Lari-*]. Type genus: *Laria* Scopoli, 1763 [placed on the Official Index of Rejected and Invalid Generic Names in Zoology (ICZN 1995a); syn. of *Bruchus* Linnaeus, 1767]. Comment: permanently invalid (Art. 39): based on suppressed type genus.

### Subtribe Megacerina

Bridwell, 1946

Megacerini Bridwell, 1946: 54, in key [stem: *Megacer-*]. Type genus: *Megacerus* Fahraeus, 1839. Comment: Megacerini Viret, 1961 (type genus *Megaceros* Owen, 1844) is used as valid in Mammalia: Cervidae; this case is to be referred to the Commission to remove the homonymy (Art. 55.3.1).

### Tribe Eubaptini

Bridwell, 1932

Eubaptinae Bridwell, 1932: 103, in key [stem: *Eubapt-*]. Type genus: *Eubaptus* Lacordaire, 1845.

### Tribe Kytorhinini

Bridwell, 1932

Kytorhininae Bridwell, 1932: 103, in key [stem: *Kytorhin-*]. Type genus: *Kytorhinus* Fischer von Waldheim, 1809.

### Tribe Pachymerini

Bridwell, 1929

Pachymerinae Bridwell, 1929: 142 [stem: *Pachymer-*]. Type genus: *Pachymerus* Thunberg, 1805. Comment: precedence (Pachymerini Bridwell, 1929 vs Caryedini Brimwell, 1929 vs Caryopemini Brimwell, 1929) given to taxon originally proposed at the higher rank (Art. 24.1).

### Subtribe Caryedontina

Bridwell, 1929

Caryedini Bridwell, 1929: 143 [stem: *Caryedont-*]. Type genus: *Caryedon* Schönherr, 1823. Comment: incorrect original stem formation, not in prevailing usage; correction of stem by Decelle (1966).

### Subtribe Caryopemina

Bridwell, 1929

Caryopemini Bridwell, 1929: 143 [stem: *Caryopem-*]. Type genus: *Caryopemon* Jekel, 1855. Comment: current spelling maintained (Art. 29.5): incorrect stem formation in prevailing usage (should be *Caryopemon*-).

### Subtribe Pachymerina

Bridwell, 1929

Pachymerinae Bridwell, 1929: 142 [stem: *Pachymer-*]. Type genus: *Pachymerus* Thunberg, 1805.

### Tribe Rhaebini

Blanchard, 1845

Rhaebites Blanchard, 1845b: 180 [stem: *Rhaeb-*]. Type genus: *Rhaebus* Fischer von Waldheim, 1824. Comment: original vernacular name available (Art. 11.7.2): first used in latinized form by Kraatz (1879: 278, as Rhaebini), generally accepted as in Anton (2010: 353, as Rhaebini); Blanchard (1845b: 193) also used the spelling Rhoebites.

### Subfamily Donaciinae

Kirby, 1837

Donaciadae Kirby, 1837: 222 [stem: *Donaci-*]. Type genus: *Donacia* Fabricius, 1775.

### Tribe Donaciini

Kirby, 1837

Donaciadae Kirby, 1837: 222 [stem: *Donaci-*]. Type genus: *Donacia* Fabricius, 1775.
Donacociadae Gistel, 1856a: 377 [stem: *Donacoci-*]. Type genus: *Donacocia* Gistel, 1856 [although this genus has been considered a senior syn. of *Plateumaris* C. G. Thomson, 1859 by some authors, Löbl and Silfverberg (2010: 64) recently chose “*brevicornis* Ahrens, 1810” as the type species for this genus; syn. of *Donacia* Fabricius, 1775]. Comment: incorrect original stem formation, not in prevailing usage.

### Tribe Haemoniini

Chen, 1941

Haemoniini Chen, 1941: 8 [stem: *Haemoni-*]. Type genus: *Haemonia* Dejean, 1821 [syn. of *Macroplea* Smouelle, 1819].

### Tribe Plateumarini

Böving, 1922

Plateumarini Böving, 1922: 50 [stem: *Plateumar-*]. Type genus: *Plateumaris* C. G. Thomson, 1859. Comment: current spelling maintained (Art. 29.3.1.1): incorrect stem formation in prevailing usage (should be *Plateumarid*-).
Plateumarini Askevold, 1990: 633 [stem: *Plateumar-*]. Type genus: *Plateumaris* C. G. Thomson, 1859. Comment: family-group name proposed as new without reference to Plateumarini Böving, 1922; current spelling maintained (Art. 29.3.1.1): incorrect stem formation in prevailing usage (should be *Plateumarid*-).

### Subfamily Criocerinae

Latreille, 1804

Criocerides Latreille, 1804c: 159 [stem: *Criocer-*]. Type genus: *Crioceris* Geoffroy, 1762 [placed on the Official List of Generic Names in Zoology (ICZN 1994a)].

### Tribe Criocerini

Latreille, 1804

Criocerides Latreille, 1804c: 159 [stem: *Criocer-*]. Type genus: *Crioceris* Geoffroy, 1762 [placed on the Official List of Generic Names in Zoology (ICZN 1994a)]. Comment: published 7 March 1804; this family-group name was also used in the same year by Latreille (1804d [between 19 August and 17 September]: 324, as Criocerides); current spelling maintained (Art. 29.3.1.1): incorrect stem formation in prevailing usage (should be *Criocerid*-).

### Tribe Lemini

Gyllenhal, 1813

Lemoideae Gyllenhal, 1813: 632 [stem: *Lem-*]. Type genus: *Lema* Fabricius, 1798 [placed on the Official List of Generic Names in Zoology (ICZN 1970a)].

### Tribe Pseudocriocerini

Heinze, 1962

Pseudocriocerini Heinze, 1962: 198, in key [stem: *Pseudocriocer-*]. Type genus: *Pseudocrioceris* Pic, 1916. Comment: current spelling maintained (Art. 29.3.1.1): incorrect stem formation in prevailing usage (should be *Pseudocriocerid*-).

### Subfamily Cassidinae

Gyllenhal, 1813

Cassideae Gyllenhal, 1813: 434 [stem: *Cassid-*]. Type genus: *Cassida* Linnaeus, 1758 [placed on the Official List of Generic Names in Zoology (ICZN 1974b)]. Comment: name placed on the Official List of Family-Group Names in Zoology (ICZN 1974b, as Cassidinae Stephens, 1831); First Reviser found (Cassidinae Gyllenhal, 1813 vs Hispinae Gyllenhal, 1813) is Shuckard (1839b: 68).

### Tribe Alurnini

Chapuis, 1875

Alurnites Chapuis, 1875: 292 [stem: *Alurn-*]. Type genus: *Alurnus* Fabricius, 1775. Comment: original vernacular name available (Art. 11.7.2): first used in latinized form by Weise (1910: 69, as Alurnini), generally accepted as in Staines (2002: 742, as Alurnini).
*Sphaeropalpites Chapuis, 1875: 359 [stem: *Sphaeropalp-*]. Type genus: *Sphaeropalpus* Guérin-Méneville, 1844 [syn. of *Platyauchenia* Sturm, 1843]. Comment: original vernacular name unavailable (Art. 11.7.2): not subsequently latinized.
Platyaucheniitae Spaeth, 1929b: 113 [stem: *Platyaucheni-*]. Type genus: *Platyauchenia* Sturm, 1843.

### Tribe Anisoderini

Chapuis, 1875

Anisodérites Chapuis, 1875: 294 [stem: *Anisoder-*]. Type genus: *Anisodera* Chevrolat, 1836. Comment: original vernacular name available (Art. 11.7.2): first used in latinized form by Weise (1911: 40, as Anisoderini), generally accepted as in Würmli (1975: 10, as Anisoderini).
*Lasiochilini Gressitt, 1950: 62 [stem: *Lasiochil-*]. Type genus: *Lasiochila* Weise, 1916. Comment: unavailable family-group name, proposed after 1930 without description or bibliographic reference to such a description (Art. 13.1); the author apparently used this family-group name in error for Anisoderini (see Gressitt 1953: 126); Lasiochilinae Carayon, 1972 (type genus *Lasiochilus* Reuter, 1871) has been used as valid in Hemiptera.

### Tribe Aproidini

Weise, 1911

Aprioidini Weise, 1911: 41 [stem: *Aproid-*]. Type genus: *Aproida* Pascoe, 1863.

### Tribe Arescini

Chapuis, 1875

Arescites Chapuis, 1875: 298 [stem: *Aresc-*]. Type genus: *Arescus* Perty, 1832. Comment: original vernacular name available (Art. 11.7.2): first used in latinized form by Weise (1910: 69, as Arescini), generally accepted as in Staines (2002: 741, as Arescini).

### Tribe Aspidimorphini

Chapuis, 1875

Aspidimorphites Chapuis, 1875: 406 [stem: *Aspidimorph-*]. Type genus: *Aspidimorpha* Hope, 1840. Comment: original vernacular name available (Art. 11.7.2): first used in latinized form by Spaeth (1914: (129), as Aspidormophitae [incorrect stem formation]), generally accepted as in Hincks (1952: 336, as Aspidomorphini).

### Tribe Basiprionotini

Gressitt, 1952 (1914)

*Prioptérites Chapuis, 1875: 367 [stem: *Priopter-*]. Type genus: *Prioptera* Hope, 1840 [syn. of *Basiprionota* Chevrolat, 1836]. Comment: original vernacular name unavailable (Art. 11.7.2): subsequently used in latinized form but not generally attributed to Chapuis (1875).
Priopteritae Spaeth, 1914: (129) [stem: *Priopter-*]. Type genus: *Prioptera* Hope, 1840 [syn. of *Basiprionota* Chevrolat, 1836]. Comment: usage of Basiprionotini Gressitt, 1952 conserved over this name (Art. 40.2).
Basiprionotini Gressitt, 1952: 444 [stem: *Basiprionot-*]. Type genus: *Basiprionota* Chevrolat, 1836. Comment: published 8 December 1952; name proposed to replace Priopterini because of synonymy of the type genus; this family-group name was also proposed in the same year by Hincks (1952 [31 December]: 329, in key, as Basiprionotini); usage of this name over the older name Priopterini Spaeth, 1929 conserved (Art. 40.2).
Epistictinini Hincks, 1952: 329, in key [stem: *Epistictin-*]. Type genus: *Epistictina* Hincks, 1950.

### Tribe Botryonopini

Chapuis, 1875

Botryonopites Chapuis, 1875: 291 [stem: *Botryonop-*]. Type genus: *Botryonopa* Guérin-Méneville, 1840 [*Botryonopa* is a incorrect subsequent spelling of the original name *Bothryonopa*, in prevailing usage and so deemed to be the correct original spelling (Art. 33.3.1); for correct author and year of type genus see Staines (2010: 172)]. Comment: original vernacular name available (Art. 11.7.2): first used in latinized form by Weise (1911: 39, as Botryonopini), generally accepted as in Würmli (1975: 8, as Botryonopini).

### Tribe Callispini

Chapuis, 1875

Callispites Chapuis, 1875: 269 [stem: *Callisp-*]. Type genus: *Callispa* Baly, 1859. Comment: original vernacular name available (Art. 11.7.2): first used in latinized form by Weise (1911: 41, as Callispini), generally accepted as in Würmli (1975: 12, as Callispini).
*Hispodontites Chapuis, 1875: 284 [stem: *Hispodont-*]. Type genus: *Hispodonta* Baly, 1859. Comment: original vernacular name unavailable (Art. 11.7.2): not subsequently latinized.

### Tribe Callohispini

Uhmann, 1960

Callohispini Uhmann, 1960: 60 [stem: *Callohisp-*]. Type genus: *Callohispa* Uhmann, 1960.

### Tribe Cassidini

Gyllenhal, 1813

Cassideae Gyllenhal, 1813: 434 [stem: *Cassid-*]. Type genus: *Cassida* Linnaeus, 1758 [placed on the Official List of Generic Names in Zoology (ICZN 1974b)]. Comment: name placed on the Official List of Family-Group Names in Zoology (ICZN 1974b, as Cassidinae Stephens, 1831).
Evaspidotidae Gistel, 1856a: 381 [stem: *Evaspist-*]. Type genus: *Evaspistes* Gistel, 1856 [syn. of *Cassida* Linnaeus, 1758]. Comment: incorrect original stem formation, not in prevailing usage.
*Chiridites Chapuis, 1875: 405 [stem: *Chirid-*]. Type genus: *Chirida* Chapuis, 1875. Comment: original vernacular name unavailable (Art. 11.7.2): not subsequently latinized.
*Hybosites Chapuis, 1875: 380 [stem: *Hybos-*]. Type genus: *Hybosa* Boheman, 1855 [*Hybosa* has been credited to Duponchel and Chevrolat, 1842, e.g., Seeno and Wilcox (1982: 178), but the name is not available from that reference since there is no description nor available species included; this genus was first made available by Boheman (1855: 1)]. Comment: original vernacular name unavailable (Art. 11.7.2): not subsequently latinized.
Basiptites Chapuis, 1875: 379 [stem: *Basipt-*]. Type genus: *Basipta* Chevrolat, 1842. Comment: original vernacular name available (Art. 11.7.2): first used in latinized form and generally accepted as in Hincks (1952: 337, as Basiptini).
Coptocyclitae Spaeth and Reitter, 1926: 7, in key [stem: *Coptocycl-*]. Type genus: *Coptocycla* Chevrolat, 1836. Comment: this family-group name was also used in the same year by Spaeth (1926: 1, as Coptocyclitae) but we could not establish priority.
Charidotitae Spaeth, 1942: 40 [stem: *Charidotid-*]. Type genus: *Charidotis* Boheman, 1854. Comment: name proposed after 1930 without description or bibliographic reference to such a description (Art. 13.1), however available because it was used as valid before 2000 as in Hincks (1952: 343, as Charidotini) and was not rejected by an author who, between 1961 and 1999, applied Article 13 of the then current edition of the Code (see Art. 13.2.1).

### Tribe Cephaloleiini

Chapuis, 1875

Céphaloléites Chapuis, 1875: 277 [stem: *Cephalolei-*]. Type genus: *Cephaloleia* Chevrolat, 1836. Comment: original vernacular name available (Art. 11.7.2): first used in latinized form by Weise (1910: 69, as Cephaloleiini), generally accepted as in Borowiec (1995: 553, as Cephaloleiini); incorrect original stem formation, not in prevailing usage.

### Tribe Chalepini

Weise, 1910

Chalepini Weise, 1910: 69 [stem: *Chalep-*]. Type genus: *Chalepus* Thunberg, 1805. Comment: Chalepini Weise, 1910 is a junior homonym of Chalepidae H. C. C. Burmeister, 1847 (type genus *Chalepus* W. S. MacLeay, 1819) in Scarabaeidae; the older scarab family-group name is permanently invalid because it is based on a preoccupied type genus; an application to the Commission is needed to conserve usage of Chalepini Weise, 1910.

### Tribe Coelaenomenoderini

Weise, 1911

Coelaenomenoderini Weise, 1911: 51 [stem: *Coelaenomenoder-*]. Type genus: *Coelaenomenodera* Blanchard, 1845.
Pharangispini Uhmann, 1940: 122 [stem: *Pharangisp-*]. Type genus: *Pharangispa* Maulik, 1929. Comment: proposed after 1930 without description but available because accompanied by a bibliographic reference (Maulik 1929: 233) to a description (Art. 13.1).

### Tribe Cryptonychini

Chapuis, 1875

Cryptonychites Chapuis, 1875: 286 [stem: *Cryptonych-*]. Type genus: *Cryptonychus* Gyllenhal, 1817. Comment: original vernacular name available (Art. 11.7.2): first used in latinized form by Weise (1911: 45, as Cryptonychini), generally accepted as in Würmli (1975: 21, as Cryptonychini).

### Tribe Delocraniini

Spaeth, 1929

Delocraniitae Spaeth, 1929b: 113 [stem: *Delocrani-*]. Type genus: *Delocrania* Guérin-Méneville, 1844.

### Tribe Dorynotini

Monrós and Viana, 1949 (1923)

*Batonotites Chapuis, 1875: 377 [stem: *Batonot-*]. Type genus: *Batonota* Hope, 1839 [syn. of *Dorynota* Chevrolat, 1836]. Comment: original vernacular name unavailable (Art. 11.7.2): subsequently used in latinized form but not generally attributed to Chapuis (1875).
Batonotitae Spaeth, 1923: 66 [stem: *Batonot-*]. Type genus: *Batonota* Hope, 1839 [syn. of *Dorynota* Chevrolat, 1836]. Comment: usage of Dorynotini Monrós and Viana, 1949 conserved over this name (Art. 40.2).
Dorynotini Monrós and Viana, 1949: 392 [stem: *Dorynot-*]. Type genus: *Dorynota* Chevrolat, 1836. Comment: name proposed to replace “Batonotites” because of synonymy of the type genus; usage of younger name conserved over Batonotini Spaeth, 1923 (Art. 40.2).

### Tribe Eugenysini

Hincks, 1952

*Calaspideitae Spaeth, 1942: 18 [stem: *Calaspide-*]. Type genus: *Calaspidea* Hope, 1840 [syn. of *Eugenysa* Chevrolat, 1836]. Comment: unavailable family-group name, proposed after 1930 without description or bibliographic reference to such a description (Art. 13.1).
Eugenysini Hincks, 1952: 329, in key [stem: *Eugenys-*]. Type genus: *Eugenysa* Chevrolat, 1836.

### Tribe Eurispini

Chapuis, 1875

Eurispites Chapuis, 1875: 264 [stem: *Eurisp-*]. Type genus: *Eurispa* Baly, 1859. Comment: original vernacular name available (Art. 11.7.2): first used in latinized form by Weise (1911: 45, as Eurispini), generally accepted as in Uhmann (1960: 61, as Eurispini).

### Tribe Exothispini

Weise, 1911

Exothispini Weise, 1911: 51 [stem: *Exothisp-*]. Type genus: *Exothispa* Kolbe, 1897.

### Tribe Goniocheniini

Spaeth, 1942

Goniocheniitae Spaeth, 1942: 17 [stem: *Goniocheni-*]. Type genus: *Goniochenia* Weise, 1896. Comment: name proposed after 1930 without description or bibliographic reference to such a description (Art. 13.1), however available because it was used as valid before 2000 as in Viana (1964: 217, as Goniocheniici) and was not rejected by an author who, between 1961 and 1999, applied Article 13 of the then current edition of the Code (see Art. 13.2.1).

### Tribe Gonophorini

Chapuis, 1875

Gonophorites Chapuis, 1875: 303 [stem: *Gonophor-*]. Type genus: *Gonophora* Chevrolat, 1836. Comment: original vernacular name available (Art. 11.7.2): first used in latinized form by Weise (1911: 54, as Gonophorini), generally accepted as in Würmli (1975: 47, as Gonophorini).
*Wallacéites Chapuis, 1875: 281 [stem: *Wallace-*]. Type genus: *Wallacea* Baly, 1859 [this genus is a senior homonym of *Wallacea* Doleschall, 1859 (see Woodley 2002: 410)]. Comment: original vernacular name unavailable (Art. 11.7.2): subsequently used in latinized form but not generally attributed to Chapuis (1875); Wallaceiad[a]e [incorrect stem formation] was used as valid by Ienistea (1986: 31) but it was not attributed to Chapuis (1875); Ienistea’s name is also unavailable, it was proposed after 1930 without description or bibliographic reference to such a description (Art. 13.1).

### Tribe Hemisphaerotini

Monrós and Viana, 1951 (1929)

Porphyraspitae Spaeth, 1929a: 29 [stem: *Porphyraspid-*]. Type genus: *Porphyraspis* Hope, 1840 [syn. of *Hemisphaerota* Chevrolat, 1836]. Comment: this family-group name was also used in the same year by Spaeth (1929b: 113); usage of younger name Hemisphaerotini Monrós and Viana, 1951 conserved over this name (Art. 40.2); incorrect original stem formation, not in prevailing usage.
Hemisphaerotini Monrós and Viana, 1951: 370 [stem: *Hemisphaerot-*]. Type genus: *Hemisphaerota* Chevrolat, 1836. Comment: usage of this name conserved over Porphyraspidini Spaeth, 1929 (Art. 40.2).

### Tribe Hispini

Gyllenhal, 1813

Hispoideae Gyllenhal, 1813: 448 [stem: *Hisp-*]. Type genus: *Hispa* Linnaeus, 1767.
*Monochirites Chapuis, 1875: 330 [stem: *Monochir-*]. Type genus: *Monochirus* Chapuis, 1875 [preoccupied genus name, not *Monochirus* Rafinesque, 1814 [Pisces]; syn. of *Hispellinus* Weise, 1898]. Comment: original vernacular name unavailable (Art. 11.7.2): subsequently used in latinized form but not generally attributed to Chapuis (1875).
*Trichispites Chapuis, 1875: 331 [stem: *Trichisp-*]. Type genus: *Trichispa* Chapuis, 1875. Comment: original vernacular name unavailable (Art. 11.7.2): not subsequently latinized.
Monochirini Weise, 1905: 317 [stem: *Monochir-*]. Type genus: *Monochirus* Chapuis, 1875 [preoccupied genus name, not *Monochirus* Rafinesque, 1814 [Pisces]; syn. of *Hispellinus* Weise, 1897]. Comment: permanently invalid (Art. 39): based on preoccupied type genus.

### Tribe Hispoleptini

Chapuis, 1875

Hispoleptites Chapuis, 1875: 283 [stem: *Hispolept-*]. Type genus: *Hispopleptis* Baly, 1864. Comment: original vernacular name available (Art. 11.7.2): first used in latinized form by Uhmann (1940: 121, as Hispoleptini), generally accepted as in Staines (2002: 777, as Hispoleptini).

### Tribe Hybosispini

Weise, 1910

Hybosispini Weise, 1910: 69 [stem: *Hybosisp-*]. Type genus: *Hybosispa* Weise, 1910.

### Tribe Imatidiini

Hope, 1840

Imatidiidae Hope, 1840a: 152 [stem: *Imatidi-*]. Type genus: *Imatidium* Fabricius, 1801. Comment: name previously attributed to Chapuis (1875).

### Tribe Ischyrosonychini

Chapuis, 1875

Ischyrosonychites Chapuis, 1875: 382 [stem: *Ischyrosonych-*]. Type genus: *Ischyrosonyx* Sturm, 1843 [syn. of *Eurypedus* Gistel, 1834]. Comment: original vernacular name available (Art. 11.7.2): first used in latinized form by Spaeth (1942: 31, as Ischyronycitae [incorrect stem formation]), generally accepted as in Borowiec (1995: 556, as Ischyrosonychini).
*Physonotitae Spaeth, 1942: 32 [stem: *Physonot-*]. Type genus: *Physonota* Boheman, 1854. Comment: unavailable family-group name, proposed after 1930 without description or bibliographic reference to such a description (Art. 13.1).
Asterizini Hincks, 1952: 330, in key [stem: *Asteriz-*]. Type genus: *Asteriza* Chevrolat, 1836. Comment: Hincks (1952: 336) and Seeno and Wilcox (1982: 171) mention “Asterizitae Spaeth”, we could not find any earlier usage of Asterizitae by Spaeth.

### Tribe Leptispini

Fairmaire, 1868

Leptispites Fairmaire, 1868: 258 [stem: *Leptisp-*]. Type genus: *Leptispa* Baly, 1859. Comment: original vernacular name available (Art. 11.7.2): first used in latinized form by Weise (1911: 44, as Lepthispini [incorrect stem formation]), generally accepted as in Würmli (1975: 18, as Leptispini).

### Tribe Mesomphaliini

Hope, 1840

Mesomphalidae Hope, 1840a: 160 [stem: *Mesomphali-*]. Type genus: *Mesomphalia* Hope, 1839. Comment: incorrect original stem formation, not in prevailing usage.
*Chélymorphites Chapuis, 1875: 402 [stem: *Chelymorph-*]. Type genus: *Chelymorpha* Chevrolat, 1836. Comment: original vernacular name unavailable (Art. 11.7.2): not subsequently latinized.
*Elytrogonites Chapuis, 1875: 403 [stem: *Elytrogon-*]. Type genus: *Elytrogona* Chevrolat, 1836. Comment: original vernacular name unavailable (Art. 11.7.2): not subsequently latinized.
*Omoplatites Chapuis, 1875: 397 [stem: *Omoplat-*]. Type genus: *Omoplata* Hope, 1840 [syn. of *Echoma* Chevrolat, 1836]. Comment: original vernacular name unavailable (Art. 11.7.2): not subsequently latinized.
Stolaini Hincks, 1952: 329, in key [stem: *Stolad-*]. Type genus: *Stolas* Billberg, 1820. Comment: incorrect original stem formation, not in prevailing usage.

### Tribe Nothosacanthini

Gressitt, 1952 (1929)

*Hoplionotites Chapuis, 1875: 357 [stem: *Hoplionot-*]. Type genus: *Hoplionota* Hope, 1840 [syn. of *Notosacantha* Chevrolat, 1836]. Comment: original vernacular name unavailable (Art. 11.7.2): subsequently used in latinized form but not generally attributed to Chapuis (1875).
Hoplionotitae Spaeth, 1929b: 113 [stem: *Hoplionot-*]. Type genus: *Hoplionota* Hope, 1840 [syn. of *Notosacantha* Chevrolat, 1836]. Comment: usage of younger name Nothosacanthini Gressitt, 1952 conserved over this name (Art. 40.2).
Notosacanthina Gressitt, 1952: 444 [stem: *Notosacanth-*]. Type genus: *Notosacantha* Chevrolat, 1836. Comment: published 8 December 1952; name proposed to replace Hoplionotini Spaeth, 1929 because of the synonymy of the type genus; this family-group name was also proposed in the same year by Hincks (1952 [31 December]: 328, in key, as Notosacanthini); usage of this name conserved over Hoplionotini Spaeth, 1929 (Art. 40.2).

### Tribe Oediopalpini

Monrós and Viana, 1947 (1910)

Amplipalpini Weise, 1910: 69 [stem: *Amplipalp-*]. Type genus: *Amplipalpa* Harold, 1875 [syn. of *Oediopalpa* Baly, 1859]. Comment: younger name Oediopalpini Monrós and Viana, 1947 conserved over this name (Art. 40.2).
Oediopalpini Monrós and Viana, 1947: 150, in key [stem: *Oediopalp-*]. Type genus: *Oediopalpa* Baly, 1859. Comment: name proposed to replace Amplipalpini Weise, 1910 because of synonymy of the type genus; usage of this name conserved over the older name Amplipalpini Weise, 1910 (Art. 40.2).

### Tribe Omocerini

Hincks, 1952 (1923)

*Tauromites Chapuis, 1875: 372 [stem: *Taurom-*]. Type genus: *Tauroma* Hope, 1839 [syn. of *Omocerus* Chevrolat, 1835]. Comment: original vernacular name unavailable (Art. 11.7.2): subsequently used in latinized form but not generally attributed to Chapuis (1875).
Tauromitae Spaeth, 1923: 66 [stem: *Taurom-*]. Type genus: *Tauroma* Hope, 1839 [syn. of *Omocerus* Chevrolat, 1835]. Comment: younger name Omocerini Hincks, 1952 conserved over this name (Art. 40.2).
Omocerini Hincks, 1952: 329, in key [stem: *Omocer-*]. Type genus: *Omocerus* Chevrolat, 1835. Comment: name proposed to replace Tauromini Spaeth, 1923 because of synonymy of the type genus; usage of younger name conserved over Tauromini Spaeth, 1923 (Art. 40.2).

### Tribe Oncocephalini

Chapuis, 1875

Oncocéphalites Chapuis, 1875: 308 [stem: *Oncocephal-*]. Type genus: *Onchocephala* Guérin-Méneville, 1844 [this genus name was first made available by Guérin-Méneville (1844: 280-281) although most authors credit Chevrolat (1847: 110, spelled *Oncocephalus*) as the correct author and date of publication of this genus]. Comment: original vernacular name available (Art. 11.7.2): first used in latinized form by Weise (1911: 50, as Oncocephalini), generally accepted as in Świętojańska et al. (2006: 49, as Oncocephalini); current spelling maintained (Art. 29.5): incorrect stem formation in prevailing usage (should be *Onchocephal*-).
Choeridionini Weise, 1911: 49 [stem: *Chaeridion-*]. Type genus: *Chaeridiona* Baly, 1869 [as *Choeridiona*, incorrect subsequent spelling of type genus name, not in prevailing usage]. Comment: incorrect original stem formation, not in prevailing usage.

### Tribe Promecothecini

Chapuis, 1875

Promecothécites Chapuis, 1875: 300 [stem: *Promecothec-*]. Type genus: *Promecotheca* Chevrolat, 1847. Comment: original vernacular name available (Art. 11.7.2): first used in latinized form by Weise (1911: 53, as Promecothecini), generally accepted as in Gressitt (1950: 81, as Promecothecini).

### Tribe Prosopodontini

Weise, 1910

Prosopodontini Weise, 1910: 69 [stem: *Prosopodont-*]. Type genus: *Prosopodonta* Baly, 1885.

### Tribe Sceloenoplini

Uhmann, 1930

*Céphalodontites Chapuis, 1875: 313 [stem: *Cephalodont-*]. Type genus: *Cephalodonta* Chevrolat, 1842 [this name was recently attributed to “Chevrolat, 1843” (e.g., Staines (2002: 748)) but it was made available for the first time by Chevrolat (1842: 272); syn. of *Sceloenopla* Chevrolat, 1836]. Comment: original vernacular name unavailable (Art. 11.7.2): subsequently used in latinized form but not generally attributed to Chapuis (1875).
Cephalodontinae Gestro, 1906: 548 [stem: *Cephalodont-*]. Type genus: *Cephalodonta* Chevrolat, 1842 [this name was recently attributed to “Chevrolat, 1843” (e.g., Staines (2002: 748)) but it was made available for the first time by Chevrolat (1842: 272); syn. of *Sceloenopla* Chevrolat, 1836]. Comment: usage of the younger name Sceloenoplini Uhmann, 1930 conserved over this name (Art. 40.2).
Sceloenoplini Uhmann, 1930: 238 [stem: *Sceloenopl-*]. Type genus: *Sceloenopla* Chevrolat, 1836. Comment: although this is not the oldest name for the tribe, we recommend that an application be sent to the Commission in order to conserve usage of Sceloenoplini Uhmann, 1930.

### Tribe Spilophorini

Chapuis, 1875

Spilophorites Chapuis, 1875: 364 [stem: *Spilophor-*]. Type genus: *Spilophora* Boheman, 1850. Comment: original vernacular name available (Art. 11.7.2): first used in latinized form by Spaeth (1929a: 29, as Spilophoritae), generally accepted as in Borowiec (1995: 553, as Spilophorini); this name is a senior homonym of Spilophorina Krikken, 1984 (type genus *Spilophorus* Schaum, 1848) currently used as valid in Scarabaeidae; this case is to be referred to the Commission to remove the homonymy (Art. 55.3.1).

### Tribe Uroplatini

Weise, 1910

*Octotomites Chapuis, 1875: 310 [stem: *Octotom-*]. Type genus: *Octotoma* Dejean, 1836. Comment: original vernacular name unavailable (Art. 11.7.2): subsequently used in latinized form but not generally attributed to Chapuis (1875); Octotomidae was used as valid by Ienistea (1986: 31) but it was not attributed to Chapuis (1875); Ienistea’s name is also unavailable, it was proposed after 1930 without description or bibliographic reference to such a description (Art. 13.1).
Uroplatini Weise, 1910: 69 [stem: *Uroplat-*]. Type genus: *Uroplata* Chevrolat, 1836 [“*Uroplata* Chevrolat, 1835” placed on the Official List of Generic Names in Zoology (ICZN 1985f)]. Comment: name placed on the Official List of Family-Group Names in Zoology (ICZN 1985f, as Uroplatini Leng, 1920).

### Subfamily Chrysomelinae

Latreille, 1802

Chrysomelinae Latreille, 1802: 220 [stem: *Chrysomel-*]. Type genus: *Chrysomela* Linnaeus, 1758 [placed on the Official List of Generic Names in Zoology (ICZN 1984c)]. Comment: see Kippenberg (2010b) for an alternative tribal classification within this subfamily.

### Tribe Chrysomelini

Latreille, 1802

Chrysomelinae Latreille, 1802: 220 [stem: *Chrysomel-*]. Type genus: *Chrysomela* Linnaeus, 1758 [placed on the Official List of Generic Names in Zoology (ICZN 1984c)].
Eleiaeidae Gistel, 1848: [9] [stem: *Elei-*]. Type genus: *Eleia* Gistel, 1848 [syn. of *Chrysomela* Linnaeus, 1758]. Comment: incorrect original stem formation, not in prevailing usage.
Prasocurisidae Gistel, 1848: [10] [stem: *Prasocur-*]. Type genus: *Prasocuris* Latreille, 1802. Comment: incorrect original stem formation, not in prevailing usage.
Chloëmeladae Gistel, 1856a: 379 [stem: *Chloemel-*]. Type genus: *Chloemela* Gistel, 1856 [syn. of *Chrysomela* Linnaeus, 1758]. Comment: incorrect original stem formation, not in prevailing usage.
Doryphores Motschulsky, 1860: 181 [stem: *Doryphor-*]. Type genus: *Doryphora* Illiger, 1807. Comment: original vernacular name available (Art. 11.7.2): first used in latinized form by Gerstaecker (1862: 405, as Doryphorae), generally accepted as in Riley et al. (2002: 639, as Doryphorina).
Gonioctènes Motschulsky, 1860: 179 [stem: *Goniocten-*]. Type genus: *Gonioctena* Chevrolat, 1836. Comment: original vernacular name available (Art. 11.7.2): first used in latinized form by Gerstaecker (1862: 405, as Gonioctenae), generally accepted as in Warchalowski (1994: 100, as Gonioctenina).
Linaeines Motschulsky, 1860: 196 [stem: *Lin-*]. Type genus: *Lina* Latreille, 1829 [syn. of *Chrysomela* Linnaeus, 1758]. Comment: original vernacular name available (Art. 11.7.2): first used in latinized form and generally accepted as in Gerstaecker (1862: 406, as Linae).
Ovosomes Motschulsky, 1860: 211 [stem: *Ovosomat-*]. Type genus: *Ovosoma* Motschulsky, 1860. Comment: original vernacular name available (Art. 11.7.2): first used in latinized form and generally accepted as in Gerstaecker (1862: 406, as Ovosomae); incorrect original stem formation, not in prevailing usage.
Paropsines Motschulsky, 1860: 192 [stem: *Paropse-*]. Type genus: *Paropsis* A. G. Olivier, 1807. Comment: original vernacular name available (Art. 11.7.2): first used in latinized form by Gerstaecker (1862: 405, as Paropsinae), generally accepted as in Riley et al. (2002: 639, as Paropsina); incorrect original stem formation, not in prevailing usage.
Phrathorines Motschulsky, 1860: 216 [stem: *Phrator-*]. Type genus: *Phratora* Chevrolat, 1836. Comment: original vernacular name available (Art. 11.7.2): first used in latinized form by Gerstaecker (1862: 406, as Phratorinae), generally accepted as in Warchalowski (1994: 139, as Phratorina); incorrect original stem formation, not in prevailing usage.
*Australicites Chapuis, 1874: 428 [stem: *Australic-*]. Type genus: *Australica* Chevrolat, 1836 [this name is sometimes treated as a *nomen nudum* (Reid 2006: 55), however it is available since four available species names were included by Chevrolat (1836)]. Comment: original vernacular name unavailable (Art. 11.7.2): not subsequently latinized.
*Clidonotites Chapuis, 1874: 414 [stem: *Clidonot-*]. Type genus: *Clidonotus* Chapuis, 1874. Comment: original vernacular name unavailable (Art. 11.7.2): subsequently used in latinized form but not generally attributed to Chapuis (1874); Clidonotidae was used as valid by Ienistea (1986: 31) but it was not attributed to Chapuis (1874); Ienistea’s name is also unavailable, it was proposed after 1930 without description or bibliographic reference to such a description (Art. 13.1).
*Colaspidémites Chapuis, 1874: 364 [stem: *Colaspidem-*]. Type genus: *Colaspidema* Laporte, 1833. Comment: original vernacular name unavailable (Art. 11.7.2): subsequently used in latinized form but not generally attributed to Chapuis (1874); Colaspidemidae was used as valid by Ienistea (1986: 31) but it was not attributed to Chapuis (1874); Ienistea’s name is also unavailable, it was proposed after 1930 without description or bibliographic reference to such a description (Art. 13.1).
*Cyrtonites Chapuis, 1874: 364 [stem: *Cyrton-*]. Type genus: *Cyrtonus* Latreille, 1829. Comment: original vernacular name unavailable (Art. 11.7.2): not subsequently latinized.
*Elytrosphoerites Chapuis, 1874: 406 [stem: *Elytrosphoer-*]. Type genus: *Elytrosphoera* Blanchard, 1845. Comment: original vernacular name unavailable (Art. 11.7.2): not subsequently latinized; name incorrectly spelled Elytrosphaerites in the index of the same work (page 449).
Entomoscélites Chapuis, 1874: 418 [stem: *Entomoscelid-*]. Type genus: *Entomoscelis* Chevrolat, 1836. Comment: original vernacular name available (Art. 11.7.2): first used in latinized form by J. L. LeConte and G. H. Horn (1883: 344, as Entomoscelides [treated as Latin]), generally accepted as in Warchalowski (1994: 154, as Entomoscelina); incorrect original stem formation, not in prevailing usage.
*Lycariites Chapuis, 1874: 420 [stem: *Lycari-*]. Type genus: *Lycaria* Stål, 1857. Comment: original vernacular name unavailable (Art. 11.7.2): not subsequently latinized.
Phyllocharites Chapuis, 1874: 422 [stem: *Phyllocharit-*]. Type genus: *Phyllocharis* Dalman, 1824. Comment: original vernacular name available (Art. 11.7.2): first used in latinized form and generally accepted as in Weise (1915: 436, as Phyllocharini); incorrect original stem formation, not in prevailing usage.
*Pyxites Chapuis, 1874: 438 [stem: *Pyxid-*]. Type genus: *Pyxis* Chevrolat, 1843. Comment: original vernacular name unavailable (Art. 11.7.2): not subsequently latinized; incorrect original stem formation, not in prevailing usage.
Phyllodectae J. L. LeConte and G. H. Horn, 1883: 344 [stem: *Phyllodect-*]. Type genus: *Phyllodecta* Kirby, 1837 [subgenus of *Phratora* Chevrolat, 1836].
Dicranosternini Weise, 1915: 436 [stem: *Dicranostern-*]. Type genus: *Dicranosterna* Motschulsky, 1860.
Phaedonini Weise, 1915: 435 [stem: *Phaedon-*]. Type genus: *Phaedon* Latreille, 1829.
Zygogrammini Weise, 1915: 435 [stem: *Zygogrammat-*]. Type genus: *Zygogramma* Chevrolat, 1836. Comment: incorrect original stem formation, not in prevailing usage.
Chrysolinina Chen, 1936: 64, in key [stem: *Chrysolin-*]. Type genus: *Chrysolina* Motschulsky, 1860 [placed on the Official List of Generic Names in Zoology (ICZN 1984c)].
Barymelini Bechyné, 1948: 295 [stem: *Barymel-*]. Type genus: *Barymela* Weise, 1910.
Doryphorini Bechyné, 1950a: 115, in key [stem: *Doryphor-*]. Type genus: *Doryphora* Illiger, 1807. Comment: family-group name proposed as new without reference to Doryphores Motschulsky, 1860.
Monarditini Bechyné, 1953: 85 [stem: *Monardit-*]. Type genus: *Monardita* Bechyné, 1948.
Oreinini Bechyné, 1958: 218, nota [stem: *Orein-*]. Type genus: *Oreina* Chevrolat, 1836. Comment: the older name Oreinini Bleeker, 1863 (type genus *Oreinus* M’Clelland, 1839) is available in Pisces; this case is to be referred to the Commission to remove the homonymy (Art. 55.3.1).
*Phytodectini Bechyné, 1980: 58 [stem: *Phytodect-*]. Type genus: *Phytodecta* Kirby, 1837 [syn. of *Gonioctena* Chevrolat, 1836]. Comment: unavailable family-group name, proposed after 1930 without description or bibliographic reference to such a description (Art. 13.1).
Hispostomini Daccordi, 1981: 181, in key [stem: *Hispostomat-*]. Type genus: *Hispostoma* Weise, 1907. Comment: incorrect original stem formation, not in prevailing usage.
Sphaeratrixini Daccordi, 1981: 181, in key [stem: *Sphaeratrix-*]. Type genus: *Sphaeratrix* Gistel, 1848. Comment: the correct stem based on *Sphaeratrix* is unclear since Gistel did not specify the etymology of his genus therefore we accept the original stem as correct.
*Gastrophysina Steinhausen, 2001: 47 [stem: *Gastrophys-*]. Type genus: *Gastrophysa* Chevrolat, 1836. Comment: unavailable family-group name, proposed after 1999 without explicit intention (Art. 16.1).
Gastrophysina Kippenberg, 2010a: 68 [stem: *Gastrophys-*]. Type genus: *Gastrophysa* Chevrolat, 1836.

### Tribe Timarchini

Motschulsky, 1860

Timarchaeines Motschulsky, 1860: 187 [stem: *Timarch-*]. Type genus: *Timarcha* Samouelle, 1819. Comment: original vernacular name available (Art. 11.7.2): first used in latinized form by Gerstaecker (1862: 405, as Timarchae), generally accepted as in Burakowski et al. (1990: 115, as Timarchini); incorrect original stem formation, not in prevailing usage.

### Subfamily Galerucinae

Latreille, 1802

Galerucae Latreille, 1802: 228 [stem: *Galeruc-*]. Type genus: *Galeruca* Geoffroy, 1762 [placed on the Official List of Generic Names in Zoology (ICZN 1994a)].

### Tribe Alticini

Newman, 1834

Halticites Newman, 1834: 421 [stem: *Altic-*]. Type genus: *Altica* Geoffroy, 1762 [as *Haltica*, unjustified emendation of type genus name by Illiger (1801), not in prevailing usage; *Altica* placed on the Official List of Generic Names in Zoology (ICZN 1994a)]. Comment: incorrect original stem formation, not in prevailing usage.
Hesperidae Swainson, 1840: 310 [stem: *Hesper-*]. Type genus: *Hespera* Weise, 1889.
Longitarses Maehler, 1850: 7 [stem: *Longitars-*]. Type genus: *Longitarsus* Latreille, 1829.
Plectroscelides C. G. Thomson, 1866: 213 [stem: *Plectroscelid-*]. Type genus: *Plectroscelis* Chevrolat, 1836. Comment: incorrect original stem formation, not in prevailing usage.
*Acrocryptites Chapuis, 1875: 36 [stem: *Acrocrypt-*]. Type genus: *Acrocrypta* Baly, 1862. Comment: original vernacular name unavailable (Art. 11.7.2): subsequently used in latinized form but not generally attributed to Chapuis (1875); Acrocryptidae was used as valid by Ienistea (1986: 31) but it was not attributed to Chapuis (1875); Ienistea’s name is also unavailable, it was proposed after 1930 without description or bibliographic reference to such a description (Art. 13.1).
*Amphimélites Chapuis, 1875: 34 [stem: *Amphimel-*]. Type genus: *Amphimela* Chapuis, 1875. Comment: original vernacular name unavailable (Art. 11.7.2): not subsequently used in latinized form and treated as valid.
Aphthonites Chapuis, 1875: 69 [stem: *Aphthon-*]. Type genus: *Aphthona* Chevrolat, 1836. Comment: original vernacular name available (Art. 11.7.2): first used in latinized form by J. L. LeConte and G. H. Horn (1883: 352, as Aphthonae), generally accepted as in Bechyné (1968: 1708, as Aphthonini).
Arsipodites Chapuis, 1875: 37 [stem: *Arsipod-*]. Type genus: *Arsipoda* Erichson, 1842. Comment: original vernacular name available (Art. 11.7.2): first used in latinized form by J. L. LeConte and G. H. Horn (1883: 353, as Arsipodes [treated as Latin]), generally accepted as in Bechyné and Špringlová de Bechyné (1973: 26, as Arsipodini).
Aspicélites Chapuis, 1875: 75 [stem: *Aspicel-*]. Type genus: *Aspicela* Dejean, 1836. Comment: original vernacular name available (Art. 11.7.2): first used in latinized form and generally accepted as in G. H. Horn (1889: 166, as Aspicelae).
Blépharidites Chapuis, 1875: 26 [stem: *Blepharid-*]. Type genus: *Blepharida* Chevrolat, 1836. Comment: original vernacular name available (Art. 11.7.2): first used in latinized form by J. L. LeConte and G. H. Horn (1883: 350, as Blepharidae [incorrect stem formation]), generally accepted as in Bechyné (1968: 1724, as Blepharidini).
Crépidodérites Chapuis, 1875: 51 [stem: *Crepidoder-*]. Type genus: *Crepidodera* Chevrolat, 1836. Comment: original vernacular name available (Art. 11.7.2): first used in latinized form and generally accepted as in J. L. LeConte and G. H. Horn (1883: 350, as Crepidoderae).
*Diamphidiites Chapuis, 1875: 24 [stem: *Diamphidi-*]. Type genus: *Diamphidia* Gerstaecker, 1855. Comment: original vernacular name unavailable (Art. 11.7.2): subsequently used in latinized form but not generally attributed to Chapuis (1875); Diamphidiini and Diamphidiidae were subsequently used as valid by Bechyné (1980: 58) and Ienistea (1986: 31) but those names were not attributed to Chapuis (1875); Bechyné’s and Ienistea’s names are also unavailable, they were proposed after 1930 without description or bibliographic reference to such a description (Art. 13.1).
Diboliites Chapuis, 1875: 137 [stem: *Diboli-*]. Type genus: *Dibolia* Latreille, 1829. Comment: original vernacular name available (Art. 11.7.2): first used in latinized form by J. L. LeConte and G. H. Horn (1883: 350, as Diboliae), generally accepted as in Bechyné (1997: 206, as Diboliini).
*Élithiites Chapuis, 1875: 21 [stem: *Elithi-*]. Type genus: *Elithia* Chapuis, 1875. Comment: original vernacular name unavailable (Art. 11.7.2): subsequently used in latinized form but not generally attributed to Chapuis (1875); this name was originally spelled Elithiites in the key but Élithiides in description (both p. 21); Elithiidae was used as valid by Ienistea (1986: 31) but it was not attributed to Chapuis (1875); Ienistea’s name is also unavailable, it was proposed after 1930 without description or bibliographic reference to such a description (Art. 13.1).
Lacticites Chapuis, 1875: 123 [stem: *Lactic-*]. Type genus: *Lactica* Erichson, 1847. Comment: original vernacular name available (Art. 11.7.2): first used in latinized form and generally accepted as in J. L. LeConte and G. H. Horn (1883: 350, as Lacticae).
Mniophilites Chapuis, 1875: 129 [stem: *Mniophil-*]. Type genus: *Mniophila* Stephens, 1831. Comment: original vernacular name available (Art. 11.7.2): first used in latinized form and generally accepted as in J. L. LeConte and G. H. Horn (1883: 350, as Mniophilae).
Monoplatites Chapuis, 1875: 91 [stem: *Monoplat-*]. Type genus: *Monoplatus* Chevrolat, 1846 [this genus is usually credited to Clark, 1860, e.g., Seeno and Wilcox (1982: 141) and Linzmeier and Konstantinov (2009), but the name was first made available with a short description by Chevrolat (1846: 333); syn. of *Sphaeronychus* Dejean, 1836]. Comment: original vernacular name available (Art. 11.7.2): first used in latinized form by J. L. LeConte and G. H. Horn (1883: 350, as Monoplati), generally accepted as in Bechyné and Špringlová de Bechyné (1975: 132, as Monoplatini); treated as a valid tribe and given precedence over Sphaeronychini (Art. 40.1) by Linzmeier and Konstantinov (2009: 657).
*Nonarthrites Chapuis, 1875: 141 [stem: *Nonarthr-*]. Type genus: *Nonarthra* Baly, 1862. Comment: original vernacular name unavailable (Art. 11.7.2): subsequently used in latinized form but not generally attributed to Chapuis (1875); Nonarthridae was used as valid by Ienistea (1986: 31) but it was not attributed to Chapuis (1875); Ienistea’s name is also unavailable, it was proposed after 1930 without description or bibliographic reference to such a description (Art. 13.1).
Oedionychites Chapuis, 1875: 81 [stem: *Oedionych-*]. Type genus: *Oedionychis* Latreille, 1829. Comment: original vernacular name available (Art. 11.7.2): first used in latinized form by J. L. LeConte and G. H. Horn (1883: 349, as Oedionyches [treated as Latin]), generally accepted as in Bechyné (1968: 1703, as Oedionychini).
Oxygonites Chapuis, 1875: 43 [stem: *Oxygon-*]. Type genus: *Oxygona* Chevrolat, 1847 [this genus has been attributed to “Chevrolat, 1837” (see Seeno and Wilcox 1982: 132) but the name is not available from Dejean’s second and third editions of his catalogue; it was first made available by Chevrolat (1847: 368)]. Comment: original vernacular name available (Art. 11.7.2): first used in latinized form and generally accepted as in Jacoby (1884a: 34, as Oxygoninae).
*Procalites Chapuis, 1875: 175 [stem: *Procal-*]. Type genus: *Procalus* Clark, 1865. Comment: original vernacular name unavailable (Art. 11.7.2): subsequently used in latinized form but not generally attributed to Chapuis (1875); Procalidae was used as valid by Ienistea (1986: 31) but it was not attributed to Chapuis (1875); Ienistea’s name is also unavailable, it was proposed after 1930 without description or bibliographic reference to such a description (Art. 13.1).
Psylliodites Chapuis, 1875: 140 [stem: *Psylliod-*]. Type genus: *Psylliodes* Latreille, 1829. Comment: original vernacular name available (Art. 11.7.2): first used in latinized form and generally accepted as in J. L. LeConte and G. H. Horn (1883: 349, as Psylliodes [treated as Latin]).
Chaetocnemae J. L. LeConte and G. H. Horn, 1883: 354 [stem: *Chaetocnem-*]. Type genus: *Chaetocnema* Stephens, 1831.
Disonychae J. L. LeConte and G. H. Horn, 1883: 351 [stem: *Disonych-*]. Type genus: *Disonycha* Chevrolat, 1836.
Euplectrosceles G. H. Horn, 1889: 167, in key [stem: *Euplectroscelid-*]. Type genus: *Euplectroscelis* Crotch, 1873. Comment: incorrect original stem formation, not in prevailing usage.
Pseudolampses G. H. Horn, 1889: 166, in key [stem: *Pseudolampse-*]. Type genus: *Pseudolampsis* Horn, 1889. Comment: incorrect original stem formation, not in prevailing usage.
Systenae G. H. Horn, 1889: 167, in key [stem: *Systen-*]. Type genus: *Systena* Chevrolat, 1836.
Luperalticini Leng, 1920: 301 [stem: *Luperaltic-*]. Type genus: *Luperaltica* Crotch, 1873.
Octogonotini Weise, 1921: 151 [stem: *Octogonot-*]. Type genus: *Octogonotes* Drapiez, 1820.
Serraticollini B. E. White, 1942: 17 [stem: *Serraticoll-*]. Type genus: *Serraticollis* B. E. White, 1942.
Sphaeronychini Bechyné and Špringlová de Bechyné, 1960: 7 [stem: *Sphaeronych-*]. Type genus: *Sphaeronychus* Dejean, 1836 [*Sphaeronychus* is an incorrect subsequent spelling of *Sphraeronychus* Dejean, 1836, in prevailing usage, treated as correct original spelling (Art. 33.3.1)]. Comment: this taxon was treated as a valid tribe (which included Monoplatini) by Furth (2007: 90).
Disonychina Bechyné and Špringlová de Bechyné, 1966: 142, in key [stem: *Disonych-*]. Type genus: *Disonycha* Chevrolat, 1836. Comment: family-group name proposed as new without reference to Disonychae J. L. LeConte and G. H. Horn, 1883.
Cacoscelini Bechyné, 1968: 1715 [stem: *Cacoscelid-*]. Type genus: *Cacoscelis* Chevrolat, 1836. Comment: Cacoscelini J. Thomson, 1861 (type genus *Cacosceles* Newman, 1838) is available in Cerambycidae and used as valid; the correct stem *Cacoscelid*- should be used for the alticine name in the future in order to avoid homonymy with the cerambycid name.
*Hermaeophagina Bechyné, 1968: 1698 [stem: *Hermaeophag-*]. Type genus: *Hermaeophaga* Foudras, 1859. Comment: unavailable family-group name, proposed after 1930 without description or bibliographic reference to such a description (Art. 13.1).
Longitarsini Bechyné, 1968: 1708 [stem: *Longitars-*]. Type genus: *Longitarsus* Latreille, 1829. Comment: family-group name proposed as new without reference to Longitarses Maehler, 1850.
Lypneina Bechyné, 1968: 1717 [stem: *Lypne-*]. Type genus: *Lypnea* Baly, 1876.
Podagricini Bechyné, 1968: 1718 [stem: *Podagric-*]. Type genus: *Podagrica* Chevrolat, 1836.
*Sphaerodermini Bechyné, 1968: 1702 [stem: *Sphaerodermat-*]. Type genus: *Sphaeroderma* Stephens, 1831. Comment: unavailable family-group name, proposed after 1930 without description or bibliographic reference to such a description (Art. 13.1); incorrect original stem formation, not in prevailing usage.
Wittmeralticina Bechyné, 1968: 1717 [stem: *Wittmeraltic-*]. Type genus: *Wittmeraltica* Bechyné, 1956.
Psilaphina Bechyné and Špringlová de Bechyné, 1973: 27, in key [stem: *Psilaph-*]. Type genus: *Psilapha* Clark, 1865.
Diphaulacini Bechyné and Špringlová de Bechyné, 1975: 116 [stem: *Diphaulac-*]. Type genus: *Diphaulaca* Chevrolat, 1836.
Manobiini Bechyné and Špringlová de Bechyné, 1975: 132, in key [stem: *Manobi-*]. Type genus: *Manobia* Jacoby, 1885.
Marcapatiini Bechyné and Špringlová de Bechyné, 1975: 131 [stem: *Marcapati-*]. Type genus: *Marcapatia* Bechyné, 1958.
Monomacrina Bechyné and Špringlová de Bechyné, 1975: 27 [stem: *Monomacr-*]. Type genus: *Monomacra* Chevrolat, 1836.
Systenini Bechyné and Špringlová de Bechyné, 1975: 132, in key [stem: *Systen-*]. Type genus: *Systena* Chevrolat, 1836. Comment: family-group name proposed as new without reference to Systenae G. H. Horn, 1889.
*Phygasiini Bechyné, 1980: 58 [stem: *Phygasi-*]. Type genus: *Phygasia* Dejean, 1836. Comment: unavailable family-group name, proposed after 1930 without description or bibliographic reference to such a description (Art. 13.1).
*Ocnoscelina Seeno and Wilcox, 1982: 127 [stem: *Ocnoscelid-*]. Type genus: *Ocnoscelis* Erichson, 1847. Comment: unavailable family-group name, proposed after 1930 without description or bibliographic reference to such a description (Art. 13.1); incorrect original stem formation, not in prevailing usage.
Hermaeophagina Bechyné, 1997: 116 [stem: *Hermaeophag-*]. Type genus: *Hermaeophaga* Foudras, 1859.
Ocnoscelina Bechyné, 1997: 124 [stem: *Ocnoscelid-*]. Type genus: *Ocnoscelis* Erichson, 1847. Comment: incorrect original stem formation, not in prevailing usage.

### Tribe Decarthrocerini

Laboissière, 1937

Decarthrocerina Laboissière, 1937: 29 [stem: *Decarthrocer-*]. Type genus: *Decarthrocera* Laboissière, 1937.

### Tribe Galerucini

Latreille, 1802

Galerucae Latreille, 1802: 228 [stem: *Galeruc-*]. Type genus: *Galeruca* Geoffroy, 1762 [placed on the Official List of Generic Names in Zoology (ICZN 1994a)].
Coelomérites Chapuis, 1875: 196 [stem: *Coelomer-*]. Type genus: *Coelomera* Chevrolat, 1836. Comment: original vernacular name available (Art. 11.7.2): first used in latinized form by Leng (1920: 296, as Coelomerini), generally accepted as in Riley et al. (2002: 639, as Coelomerites [treated as Latin]).
Atysites Chapuis, 1875: 192 [stem: *Atys-*]. Type genus: *Atysa* Baly, 1864. Comment: original vernacular name available (Art. 11.7.2): first used in latinized form by Leng (1920: 296, as Atysini), generally accepted as in Riley et al. (2002: 639, as Atysites [treated as Latin]).
Apophyliites Chapuis, 1875: 182 [stem: *Apophyli-*]. Type genus: *Apophylia* Duponchel and Chevrolat, 1841 [this genus name has been attributed to “J. Thomson, 1858”, e.g., Bezdĕk (2003: 71), however it was made available for the first time with a description by Duponchel and Chevrolat (1841: 31)]. Comment: original vernacular name available (Art. 11.7.2): first used in latinized form by Weise (1923: 124, as Apophydiini [incorrect stem formation]), generally accepted as in Seeno and Wilcox (1982: 96, as Apophyliites [treated as Latin]).
Rupiliites Chapuis, 1875: 213 [stem: *Rupili-*]. Type genus: *Rupilia* Clark, 1864. Comment: original vernacular name available (Art. 11.7.2): first used in latinized form and generally accepted as in Jacoby (1899: 83, as Rupilinae [incorrect stem formation]).
Schematizites Chapuis, 1875: 195 [stem: *Schematiz-*]. Type genus: *Schematiza* Chevrolat, 1836. Comment: original vernacular name available (Art. 11.7.2): first used in latinized form by Wilcox (1971: 101, as Schematizites [treated as Latin]), generally accepted as in Riley et al. (2002: 639, as Schematizites [treated as Latin]).
Mombasicites Laboissière, 1922: 230 [stem: *Mombasic-*]. Type genus: *Mombasica* Fairmaire, 1888.
Chorini Weise, 1923: 124 [stem: *Chorin-*]. Type genus: *Chorina* Baly, 1866. Comment: incorrect original stem formation, not in prevailing usage; correction of stem by Weise (1924: 179).
Leptosonychini Weise, 1924: 180 [stem: *Leptosonych-*]. Type genus: *Leptosonyx* Weise, 1885.
Theonina Laboissière, 1934: 7, in key [stem: *Theon-*]. Type genus: *Theone* Gistel, 1857.

### Tribe Hylaspini

Chapuis, 1875

Hylaspites Chapuis, 1875: 237 [stem: *Hylasp-*]. Type genus: *Hylaspes* Baly, 1865. Comment: original vernacular name available (Art. 11.7.2): first used in latinized form by Laboissière (1934: 8, as Hylaspina), generally accepted as in Silfverberg (1990: 120, as Hylaspini); First Reviser found (Hylaspini Chapuis, 1875 vs Agelasticini Chapuis, 1875) is Silfverberg (1990: 120).
Sermylites Chapuis, 1875: 224 [stem: *Sermyl-*]. Type genus: *Sermyla* Chapuis, 1875 [preoccupied genus name, not *Sermyla* Walker 1854 [Lepidoptera], not *Sermyla* Adams, 1854 [Mollusca]; syn. of *Sermylassa* Reitter, 1912]. Comment: original vernacular name available (Art. 11.7.2): first used in latinized form by Jacoby (1884a: 64, as Sermylinae), generally accepted as in Seeno and Wilcox (2002: 104, as Sermylites [treated as Latin]); permanently invalid (Art. 39): based on preoccupied type genus.
Antiphites Chapuis, 1875: 232 [stem: *Antiph-*]. Type genus: *Antipha* Baly, 1865 [preoccupied genus name, not *Antipha* Walker, 1855 [Lepidoptera]; syn. of *Dercetina* Gressitt and Kimoto, 1963]. Comment: original vernacular name available (Art. 11.7.2): first used in latinized form and generally accepted as in Seeno and Wilcox (1982: 103, as Antiphites [treated as Latin]); permanently invalid (Art. 39): based on preoccupied type genus.
Agelasticites Chapuis, 1875: 167 [stem: *Agelastic-*]. Type genus: *Agelastica* Chevrolat, 1836. Comment: original vernacular name available (Art. 11.7.2): first used in latinized form by Weise (1903a: 209, as Agelasticites [treated as Latin]), generally accepted as in Burakowski et al. (1991: 23, as Agelasticini).
Agelasini Leng, 1920: 298 [stem: *Agelas-*]. Type genus: *Agelasa* sensu Horn, 1893 [not *Agelasa* Motschulsky, 1860; syn. of *Sermylassa* Reitter, 1912]. Comment: based on a misidentified type genus.
Bonesiites Laboissière, 1926: 91 [stem: *Bonesi-*]. Type genus: *Bonesia* Baly, 1865.
Capulini Ogloblin, 1936: 341 [stem: *Capul-*]. Type genus: *Capula* Jakobson, 1925.
Gallerucidini Gressitt and Kimoto, 1963: 390, in key [stem: *Gallerucid-*]. Type genus: *Gallerucida* Motschulsky, 1861.
Sermylassini Mroczkowski, 1991: 22 [stem: *Sermylass-*]. Type genus: *Sermylassa* Reitter, 1912. Comment: replacement name for Sermylites Chapuis, 1875 and Agelasini Leng, 1920 because of the homonymy of the type genus.

### Tribe Luperini

Gistel, 1848

Luperiidae Gistel, 1848: [9] [stem: *Luper-*]. Type genus: *Luperus* Geoffroy, 1762 [placed on the Official List of Generic Names in Zoology (ICZN 1984a)]. Comment: name previously attributed to Chapuis (1875) in the literature; also see Beenen (2010: 464) for an alternative classification within this tribe; incorrect original stem formation, not in prevailing usage.
Aulacophorites Chapuis, 1875: 158 [stem: *Aulacophor-*]. Type genus: *Aulacophora* Chevrolat, 1836. Comment: original vernacular name available (Art. 11.7.2): first used in latinized form by Laboissière (1934: 8, as Aulacophorina), generally accepted as in Seeno and Wilcox (1982: 97, as Aulacophorites [treated as Latin]).
Diabroticites Chapuis, 1875: 165 [stem: *Diabrotic-*]. Type genus: *Diabrotica* Chevrolat, 1836. Comment: original vernacular name available (Art. 11.7.2): first used in latinized form by Leng (1920: 297, as Diabroticini), generally accepted as in Riley et al. (2002: 639, as Diabroticina).
Cérotomites Chapuis, 1875: 229 [stem: *Cerotom-*]. Type genus: *Cerotoma* Chevrolat, 1836. Comment: original vernacular name available (Art. 11.7.2): first used in latinized form by Leng (1920: 298, as Cerotomini), generally accepted as in Riley et al. (2002: 639, as Cerotomites [treated as Latin]).
Platyxanthites Chapuis, 1875: 243 [stem: *Platyxanth-*]. Type genus: *Platyxantha* Baly, 1864. Comment: original vernacular name available (Art. 11.7.2): first used in latinized form and generally accepted as in Jacoby (1884b: 226, as Platyxanthinae).
*Théopéites Chapuis, 1875: 241 [stem: *Theope-*]. Type genus: *Theopea* Baly, 1864. Comment: original vernacular name unavailable (Art. 11.7.2): subsequently used in latinized form but not generally attributed to Chapuis (1875); Theopeidae was used as valid by Ienistea (1986: 31) but it was not attributed to Chapuis (1875); Ienistea’s name is also unavailable, it was proposed after 1930 without description or bibliographic reference to such a description (Art. 13.1); Theopeina Clench, 1955 (type genus *Theope* Doubleday, 1847) is available in Lepidoptera.
Scelidites Chapuis, 1875: 184 [stem: *Scelid-*]. Type genus: *Scelida* Chapuis, 1875. Comment: original vernacular name available (Art. 11.7.2): first used in latinized form by Jacoby (1884a: 62, as Scelidinae), generally accepted as in Riley et al. (2002: 639, as Scelidites [treated as Latin]).
Phyllobroticites Chapuis, 1875: 163 [stem: *Phyllobrotic-*]. Type genus: *Phyllobrotica* Chevrolat, 1836. Comment: original vernacular name available (Art. 11.7.2): first used in latinized form by Leng (1920: 297, as Phyllobroticini), generally accepted as in Riley et al. (2002: 639, as Phyllobroticites [treated as Latin]).
*Agetocérites Chapuis, 1875: 177 [stem: *Agetocer-*]. Type genus: *Agetocera* Hope, 1831 [*Agetocera* is an unjustified emendation of the original genus name *Aegelocerus* Hope, 1831 by Hope (1840a: 170); because *Agetocera* is in prevailing usage and has been attributed to the original author and date, e.g. Bezdĕk (2010a: 73, 2010c: 464) it is treated here as a justified emendation (Art. 33.2.3.1)]. Comment: original vernacular name unavailable (Art. 11.7.2): subsequently used in latinized form but not generally attributed to Chapuis (1875); Agetoceridae was used as valid by Ienistea (1986: 31) but it was not attributed to Chapuis (1875); Ienistea’s name is also unavailable, it was proposed after 1930 without description or bibliographic reference to such a description (Art. 13.1).
Mimastrites Chapuis, 1875: 178 [stem: *Mimastr-*]. Type genus: *Mimastra* Baly, 1865. Comment: original vernacular name available (Art. 11.7.2): first used in latinized form by Jacoby (1884a: 67, as Mimastrinae), generally accepted as in Weise (1903b: 334, as Mimastrites [treated as Latin]).
Ornithognathites Chapuis, 1875: 176 [stem: *Ornithognath-*]. Type genus: *Ornithognathus* J. Thomson, 1858. Comment: original vernacular name available (Art. 11.7.2): first used in latinized form by Harold (1877: 110, as Ornithognathinae), generally accepted as in Seeno and Wilcox (1982: 97, as Ornithognathites [treated as Latin]).
Monoleptites Chapuis, 1875: 234 [stem: *Monolept-*]. Type genus: *Monolepta* Chevrolat, 1836. Comment: original vernacular name available (Art. 11.7.2): first used in latinized form by Jacoby (1884a: 61, as Monoleptinae), generally accepted as in Riley et al. (2002: 639, as Monoleptites [treated as Latin]).
Cerophysites Chapuis, 1875: 181 [stem: *Cerophys-*]. Type genus: *Cerophysa* Chevrolat, 1836. Comment: original vernacular name available (Art. 11.7.2): first used in latinized form and generally accepted as in Seeno and Wilcox (1982: 97, as Cerophysides [treated as Latin]).
Phyllecthrites G. H. Horn, 1893: 60, in key [stem: *Phyllecthr-*]. Type genus: *Phyllecthris* Dejean, 1836.
Rhaphidopalpini Weise, 1902: 140 [stem: *Raphidopalp-*]. Type genus: *Raphidopalpa* Chevrolat, 1836 [as *Rhaphidopalpa*, unjustified emendation of type genus name by Rosenhauer (1856: 325), not in prevailing usage; syn. of *Aulacophora* Chevrolat, 1836]. Comment: incorrect original stem formation, not in prevailing usage.
Androlyperini Leng, 1920: 298 [stem: *Androlyper-*]. Type genus: *Androlyperus* Crotch, 1873.
Idacanthites Laboissière, 1921: 63 [stem: *Idacanth-*]. Type genus: *Idacantha* Fairmaire, 1869.
Hyperacanthites Laboissière, 1924: 142 [stem: *Hyperacanth-*]. Type genus: *Hyperacantha* Chapuis, 1879.
Megalognathites Laboissière, 1926: 185 [stem: *Megalognath-*]. Type genus: *Megalognatha* Baly, 1878.
*Exosomites Wilcox, 1965: 93 [stem: *Exosomat-*]. Type genus: *Exosoma* Jacoby, 1903. Comment: unavailable family-group name, proposed after 1930 without description or bibliographic reference to such a description (Art. 13.1); incorrect original stem formation, not in prevailing usage.
*Trachyscelidites Wilcox, 1972: 430 [stem: *Trachyscelid-*]. Type genus: *Trachyscelida* G. H. Horn, 1893. Comment: unavailable family-group name, proposed after 1930 without description or bibliographic reference to such a description (Art. 13.1).
*Adoxiites Wilcox, 1973: 433 [stem: *Adoxi-*]. Type genus: *Adoxia* Broun, 1880. Comment: unavailable family-group name, proposed after 1930 without description or bibliographic reference to such a description (Art. 13.1).
*Eumeleptites Wilcox, 1973: 447 [stem: *Eumelept-*]. Type genus: *Eumelepta* Jacoby, 1892. Comment: unavailable family-group name, proposed after 1930 without description or bibliographic reference to such a description (Art. 13.1).
*Xenodites Wilcox, 1973: 604 [stem: *Xenod-*]. Type genus: *Xenoda* Baly, 1877. Comment: unavailable family-group name, proposed after 1930 without description or bibliographic reference to such a description (Art. 13.1).
*Doryscites Wilcox, 1973: 606 [stem: *Dorysc-*]. Type genus: *Doryscus* Jacoby, 1887. Comment: unavailable family-group name, proposed after 1930 without description or bibliographic reference to such a description (Art. 13.1).

### Tribe Metacyclini

Chapuis, 1875

Métacyclites Chapuis, 1875: 212 [stem: *Metacycl-*]. Type genus: *Metacycla* Baly, 1861. Comment: original vernacular name available (Art. 11.7.2): first used in latinized form by Leng (1920: 298, as Metacyclini), generally accepted as in Riley et al. (2002: 639, as Metacyclini).
Stictocemites Laboissière, 1922: 220 [stem: *Stictocem-*]. Type genus: *Stictocema* Jacoby, 1906.
*Exorini Wilcox, 1965: 179 [stem: *Exor-*]. Type genus: *Exora* Chevrolat, 1836. Comment: unavailable family-group name, proposed after 1930 without description or bibliographic reference to such a description (Art. 13.1).

### Tribe Oidini

Laboissière, 1921 (1875)

Adoriites Chapuis, 1875: 155 [stem: *Adori-*]. Type genus: *Adorium* Fabricius, 1801 [syn. of *Oides* Weber, 1801]. Comment: original vernacular name available (Art. 11.7.2): first used in latinized form and generally accepted as in Lea (1925: 16, as Adoriites [treated as Latin]); usage of younger name Oidini Laboissière, 1921 conserved (Art. 40.2).
Oïdites Laboissière, 1921: 38 [stem: *Oid-*]. Type genus: *Oides* Weber, 1801. Comment: usage of this name conserved over Adoriini Chapuis, 1875 (Art. 40.2).

### Subfamily Lamprosomatinae

Lacordaire, 1848

Lamprosomideae Lacordaire, 1848: 559 [stem: *Lamprosomat-*]. Type genus: *Lamprosoma* Kirby, 1819.

### Tribe Lamprosomatini

Lacordaire, 1848

Lamprosomideae Lacordaire, 1848: 559 [stem: *Lamprosomat-*]. Type genus: *Lamprosoma* Kirby, 1819. Comment: incorrect original stem formation, not in prevailing usage.

### Tribe Neochlamysini

Monrós, 1959

Neochlamysini Monrós, 1959b: 29 [stem: *Neochlamys-*]. Type genus: *Neochlamys* Jacoby, 1882. Comment: current spelling maintained (Art. 29.5): incorrect stem formation in prevailing usage (should be *Neochlamyd*-).

### Tribe Sphaerocharini

Chapuis, 1874

Sphoerocharides Chapuis, 1874: 206 [stem: *Sphaerochar-*]. Type genus: *Sphaerocharis* Lacordaire, 1848 [as *Sphoerocharis*, incorrect subsequent spelling of type genus name, not in prevailing usage]. Comment: original vernacular name available (Art. 11.7.2): first used in latinized form by Heyne and Taschenberg (1907: 248, as Sphaerocharini), generally accepted as in Monrós (1956: 32, as Sphaerocharini); current spelling maintained (Art. 29.5): incorrect stem formation in prevailing usage (should be *Sphaerocharit*-).

### Subfamily Cryptocephalinae

Gyllenhal, 1813

Cryptocephaloideae Gyllenhal, 1813: 582 [stem: *Cryptocephal-*]. Type genus: *Cryptocephalus* Geoffroy, 1762 [placed on the Official List of Generic Names in Zoology (ICZN 1994a)].

### Tribe Clytrini

Kirby, 1837

Clythridae Kirby, 1837: 207 [stem: *Clytr-*]. Type genus: *Clytra* Laicharting, 1781 [as *Clythra*, incorrect subsequent spelling of type genus name, not in prevailing usage]. Comment: incorrect original stem formation, not in prevailing usage.
Megalostomideae Lacordaire, 1848: 486 [stem: *Megalostomid-*]. Type genus: *Megalostomis* Chevrolat, 1836. Comment: incorrect original stem formation, not in prevailing usage.
Babideae Lacordaire, 1848: 394 [stem: *Babi-*]. Type genus: *Babia* Chevrolat, 1836. Comment: incorrect original stem formation, not in prevailing usage.
Ischiopachites Chapuis, 1874: 151 [stem: *Ischiopache-*]. Type genus: *Ischiopachys* Chevrolat, 1836. Comment: original vernacular name available (Art. 11.7.2): first used in latinized form by Jacoby and Clavareau (1906: 75, as Ischiopachitae), generally accepted as in Riley et al. (2002: 639, as Ischiopachina); incorrect original stem formation, not in prevailing usage.
Melolonthini Bedel, 1891: 106 [stem: *Melolonth-*]. Type genus: *Melolontha* Geoffroy, 1762 [preoccupied genus name, not *Melolontha* Fabricius, 1775 [Coleoptera: Scarabaeidae]; placed on the Official Index of Rejected and Invalid Generic Names in Zoology (ICZN 1994a); syn. of *Clytra* Laicharting, 1781]. Comment: permanently invalid (Art. 39): based on preoccupied type genus.
Eoclytrini Monrós, 1958b: 35 [stem: *Eoclytr-*]. Type genus: *Eoclytra* Monrós, 1958.
Arateini Moldenke, 1981: 85, in key [stem: *Arate-*]. Type genus: *Aratea* Lacordaire, 1848.

### Tribe Cryptocephalini

Gyllenhal, 1813

Cryptocephaloideae Gyllenhal, 1813: 582 [stem: *Cryptocephal-*]. Type genus: *Cryptocephalus* Geoffroy, 1762 [placed on the Official List of Generic Names in Zoology (ICZN 1994a)].

### Subtribe Achaenopina

Chapuis, 1874

Achaenopites Chapuis, 1874: 171 [stem: *Achaenop-*]. Type genus: *Achaenops* Suffrian, 1857. Comment: original vernacular name available (Art. 11.7.2): first used in latinized form and generally accepted as in Clavareau (1913b: 109, as Achaenopini).

### Subtribe Cryptocephalina

Gyllenhal, 1813

Cryptocephaloideae Gyllenhal, 1813: 582 [stem: *Cryptocephal-*]. Type genus: *Cryptocephalus* Geoffroy, 1762 [placed on the Official List of Generic Names in Zoology (ICZN 1994a)].

### Subtribe Monachulina

Leng, 1920

*Monachiden Suffrian, 1863: 79 [stem: *Monach-*]. Type genus: *Monachus* Chevrolat, 1836 [preoccupied genus name, not *Monachus* Fleming, 1822 [Mammalia], not *Monachus* Kaup, 1829 [Aves]; syn. of *Lexiphanes* Gistel, 1848]. Comment: original vernacular name unavailable (Art. 11.7.2): subsequently used in latinized form but not generally attributed to Suffrian (1863).
Monachites Chapuis, 1874: 172 [stem: *Monach-*]. Type genus: *Monachus* Chevrolat, 1836 [preoccupied genus name, not *Monachus* Fleming, 1822 [Mammalia], not *Monachus* Kaup, 1829 [Aves]; syn. of *Lexiphanes* Gistel, 1848]. Comment: original vernacular name available (Art. 11.7.2): first used in latinized form by J. L. LeConte and G. H. Horn (1883: 342, as Monachi), generally accepted as in Liu (1935: 286, as Monachini); permanently invalid (Art. 39): based on preoccupied type genus; Monachinae Trouessart, 1897 (type genus *Monachus* Fleming, 1822) is available in Mammalia.
Monachulini Leng, 1920: 290 [stem: *Monachul-*]. Type genus: *Monachulus* Leng, 1918 [syn. of *Lexiphanes* Gistel, 1848].

### Subtribe Pachybrachina

Chapuis, 1874

*Pachybrachiden Suffrian, 1863: 79 [stem: *Pachybrach-*]. Type genus: *Pachybrachis* Chevrolat, 1836. Comment: original vernacular name unavailable (Art. 11.7.2): subsequently used in latinized form but not generally attributed to Suffrian (1863).
Pachybrachites Chapuis, 1874: 163 [stem: *Pachybrach-*]. Type genus: *Pachybrachis* Chevrolat, 1836. Comment: original vernacular name available (Art. 11.7.2): first used in latinized form by J. L. LeConte and G. H. Horn (1883: 342, as Pachybrachi), generally accepted as in Burakowski et al. (1990: 63, as Pachybrachini).

### Subtribe Stylosomina

Chapuis, 1874

Stylosomites Chapuis, 1874: 162 [stem: *Stylosom-*]. Type genus: *Stylosomus* Suffrian, 1848. Comment: original vernacular name available (Art. 11.7.2): first used in latinized form by Sharp (1876: 99, as Stylosomites [treated as Latin]), generally accepted as in Liu (1935: 292, as Stylosomini).

### Tribe Fulcidacini

Jakobson, 1924

*Chlamytes Blanchard, 1845b: 186 [stem: *Chlamyd-*]. Type genus: *Chlamys* Knoch, 1801 [preoccupied genus name, not *Chlamys* Bolten, 1798 [Mollusca]; syn. of *Chlamisus* Rafinesque, 1815]. Comment: original vernacular name unavailable (Art. 11.7.2): subsequently used in latinized form but not generally attributed to Blanchard (1845b); incorrect original stem formation, not in prevailing usage.
Chlamydeae Lacordaire, 1848: 636 [stem: *Chlamyd-*]. Type genus: *Chlamys* Knoch, 1801 [preoccupied genus name, not *Chlamys* Bolten, 1798 [Mollusca]; syn. of *Chlamisus* Rafinesque, 1815]. Comment: incorrect original stem formation, not in prevailing usage; permanently invalid (Art. 39): based on preoccupied type genus.
Fulcidacina Jakobson, 1924: 239 [stem: *Fulcidac-*]. Type genus: *Fulcidax* Clavareau, 1913 [for comments on the authorship of this genus see Bezdĕk (2010b: 76); syn. of *Poropleura* Lacordaire, 1848]. Comment: we use Fulcidacini Jakobson, 1924 as valid for this taxon as in Bezdĕk (2010b: 76).
Chlamisinae Gressitt, 1946: 84 [stem: *Chlamis-*]. Type genus: *Chlamisus* Rafinesque, 1815. Comment: replacement name for Chlamydinae Lacordaire, 1848 because of the homonymy of the type genus.

### Subfamily Eumolpinae

Hope, 1840

Eumolpidae Hope, 1840a: 162 [stem: *Eumolp-*]. Type genus: *Eumolpus* Kugelann, 1798 [an application to suppress *Eumolpus* Kugelann, 1798 and conserve *Eumolpus* Weber, 1801 was recently submitted to the Commission by Moseyko et al. (2010)].

### Tribe Bromiini

Baly, 1865 (1863)

Adoxinae Baly, 1863: 146 [stem: *Adox-*]. Type genus: *Adoxus* Kirby, 1837 [syn. of *Bromius* Chevrolat, 1836]. Comment: use of younger name Bromiini Baly, 1865 conserved over this name (Art. 40.2).
Heteraspinae Baly, 1863: 147 [stem: *Heteraspid-*]. Type genus: *Heteraspis* Chevrolat, 1836 [this genus (incorrectly attributed to “Chevrolat, 1837”) has been treated as a synonym of *Scelodonta* Westwood, 1837 in the literature; *Heteraspis* was in fact made available by Chevrolat (1836: 413) and is a senior synonym of *Scelodonta* Westwood, 1837 (also see Löbl 2010: 83)]. Comment: although this is the oldest name for the tribe, we recommend that an application be sent to the Commission in order to conserve usage of Bromiini Baly, 1865; incorrect original stem formation, not in prevailing usage.
Bromiinae Baly, 1865: 438 [stem: *Bromi-*]. Type genus: *Bromius* Chevrolat, 1836 [an application to conserve *Bromius* Chevrolat, 1836, threatened by the older name *Eumolpus* Kugelann, 1798, was recently submitted to the Commission by Moseyko et al. (2010)]. Comment: use of family-group name conserved over Adoxiini Baly, 1863 (Art. 40.2).
Myochroinae Baly, 1865: 433 [stem: *Myochro-*]. Type genus: *Myochrous* Erichson, 1847.
Leprotites Chapuis, 1874: 268 [stem: *Leprotet-*]. Type genus: *Leprotes* Baly, 1863 [syn. of *Fidia* Motschulsky, 1860]. Comment: original vernacular name available (Art. 11.7.2): first used in latinized form by Lefèvre (1885: 71, as Leprotitae), generally accepted as in Riley et al. (2002: 639, as Leprotites [treated as Latin]); incorrect original stem formation, not in prevailing usage.
Pseudocolaspites Chapuis, 1874: 287 [stem: *Pseudocolaspid-*]. Type genus: *Pseudocolaspis* Laporte, 1833. Comment: original vernacular name available (Art. 11.7.2): first used in latinized form and generally accepted as in Lefèvre (1885: 84, as Pseudocolaspitae); incorrect original stem formation, not in prevailing usage.
Scelodontites Chapuis, 1874: 266 [stem: *Scelodont-*]. Type genus: *Scelodonta* Westwood, 1838 [syn. of *Heteraspis* Chevrolat, 1836]. Comment: original vernacular name available (Art. 11.7.2): first used in latinized form by Lefèvre (1885: 67, as Scelodontitae), generally accepted as in Riley et al. (2002: 639, as Scelodontites [treated as Latin]).
Tomyrites Chapuis, 1874: 264 [stem: *Tomyri-*]. Type genus: *Tomyris* Chapuis, 1874 [preoccupied genus name, not *Tomyris* Eichald, 1831 [Reptilia]; syn. of *Eboo* Reid, 1993]. Comment: original vernacular name available (Art. 11.7.2): first used in latinized form and generally accepted as in Lefèvre (1885: 64, as Tomyritae); permanently invalid (Art. 39): based on preoccupied type genus; incorrect original stem formation, not in prevailing usage.
*Goniopleurites Chapuis, 1875: 247 [stem: *Goniopleur-*]. Type genus: *Goniopleura* Westwood, 1832. Comment: original vernacular name unavailable (Art. 11.7.2): subsequently used in latinized form but not generally attributed to Chapuis (1875); Goniopleuridae was used as valid by Ienistea (1986: 31) but it was not attributed to Chapuis (1875); Ienistea’s name is also unavailable, it was proposed after 1930 without description or bibliographic reference to such a description (Art. 13.1); usage of the name Goniopleuridae dates back at least to the 1850’s in Trilobita, however it is based on the junior homonym *Goniopleura* Hawle and Corda 1847 and the trilobite family-group name is therefore permanently invalid (Art. 39).
Eubrachini Jacoby, 1908: 432 [stem: *Eubrach-*]. Type genus: *Eubrachis* sensu Baly, 1878 [not *Eubrachis* Dejean, 1836; syn. of *Macrocoma* Chapuis, 1874]. Comment: based on misidentified type genus.
Nerissini Kuntzen, 1912: 42 [stem: *Neriss-*]. Type genus: *Nerissus* Chapuis, 1874.
Cynoini Clavareau, 1914: 109 [stem: *Cynoi-*]. Type genus: *Cyno* T. A. Marshall, 1865. Comment: incorrect original stem formation, not in prevailing usage.
Odontionopini Clavareau, 1914: 63 [stem: *Odontionop-*]. Type genus: *Odontionopa* Erichson, 1842 [preoccupied genus name, not *Odontionopa* Chevrolat, 1836 [Coleoptera: Chrysomelidae: Eumolpinae: Euryopini]; syn. of *Eboo* Reid, 1993]. Comment: permanently invalid (Art. 39): based on preoccupied type genus.
Trichochryseini Clavareau, 1914: 82 [stem: *Trichochryse-*]. Type genus: *Trichochrysea* Baly, 1861.
Lypesthini Chûjô, 1956: 87 [stem: *Lypesthet-*]. Type genus: *Lypesthes* Baly, 1863 [conservation of usage of this name over *Fidia* Motschulsky, 1860 rejected by Commission (ICZN 2009c); syn. of *Fidia* Motschulsky, 1860]. Comment: name proposed to replace Leprotini Chapuis, 1874 because of the synonymy of the type genus; incorrect original stem formation, not in prevailing usage.
Ebooina Reid, 1993: 61 [stem: *Eboo*-]. Type genus: *Eboo* Reid, 1993. Comment: replacement name for Tomyrites Chapuis, 1874 because of the homonymy of the type genus.

### Tribe Caryonodini

Bechyné, 1951

Caryonodini Bechyné, 1951: 265 [stem: *Caryonod-*]. Type genus: *Caryonoda* Bechyné, 1951.

### Tribe Cubispini

Monrós, 1954

Cubispini Monrós, 1954: 26 [stem: *Cubisp-*]. Type genus: *Cubispa* Barber, 1946.

### Tribe Eumolpini

Hope, 1840

Eumolpidae Hope, 1840a: 162 [stem: *Eumolp-*]. Type genus: *Eumolpus* Kugelann, 1798 [an application to suppress *Eumolpus* Kugelann, 1798 and conserve *Eumolpus* Weber, 1801 was recently submitted to the Commission by Moseyko et al. (2010)]. Comment: First Reviser (Eumolpini Hope, 1840 vs Colaspidini Hope, 1840) not determined, current usage maintained.
Colaspidae Hope, 1840a: 163 [stem: *Colaspid-*]. Type genus: *Colaspis* Fabricius, 1801. Comment: incorrect original stem formation, not in prevailing usage.
Corynodina T. A. Marshall, 1865: 29 [stem: *Corynod-*]. Type genus: *Corynodes* Hope, 1840 [syn. of *Platycorynus* Chevrolat, 1836].
Chalcophanites Chapuis, 1874: 256 [stem: *Chalcophan-*]. Type genus: *Chalcophana* Chevrolat, 1836. Comment: original vernacular name available (Art. 11.7.2): first used in latinized form and generally accepted as in Lefèvre (1885: 50, as Chalcophanitae).
Edusites Chapuis, 1874: 306 [stem: *Edus-*]. Type genus: *Edusa* Chapuis, 1874 [preoccupied genus name, not *Edusa* Gistel, 1848 [Tunicata], not *Edusa* Albers, 1860 [Gastropoda] and not *Edusa* Martens 1860 [Mollusca]; syn. of *Edusella* Chapuis, 1874]. Comment: original vernacular name available (Art. 11.7.2): first used in latinized form and generally accepted as in Lefèvre (1885: 111, as Edusitae); permanently invalid (Art. 39): based on preoccupied type genus.
Endocéphalites Chapuis, 1874: 343 [stem: *Endocephal-*]. Type genus: *Endocephalus* Chevrolat, 1836. Comment: original vernacular name available (Art. 11.7.2): first used in latinized form and generally accepted as in Lefèvre (1885: 154, as Endocephalitae).
Iphiméites Chapuis, 1874: 230 [stem: *Iphime-*]. Type genus: *Iphimeis* Baly, 1864. Comment: original vernacular name available (Art. 11.7.2): first used in latinized form by Lefèvre (1885: 12, as Iphimeitae), generally accepted as in Riley et al. (2002: 639, as Iphimeites [treated as Latin]).
Chrysodinitae Lefèvre, 1885: 5 [stem: *Chrysodin-*]. Type genus: *Chrysodina* Baly, 1864 [syn. of *Spintherophyta* Dejean, 1836].
Edusellini Clavareau, 1914: 121 [stem: *Edusell-*]. Type genus: *Edusella* Chapuis, 1874.
Colaspoidini Chen, 1940: 488 [stem: *Colaspoid-*]. Type genus: *Colaspoides* Laporte, 1833.

### Tribe Euryopini

Chapuis, 1874

Euryopites Chapuis, 1874: 302 [stem: *Euryop-*]. Type genus: *Euryope* Dalman, 1824. Comment: original vernacular name available (Art. 11.7.2): first used in latinized form and generally accepted as in Lefèvre (1885: 108, as Euryopitae).
Odontionopites Lefèvre, 1876: 301 [stem: *Odontionop-*]. Type genus: *Odontionopa* Chevrolat, 1836. Comment: original vernacular name available (Art. 11.7.2): first used in latinized form and generally accepted as in Lefèvre (1885: 65, as Odontionopitae).
Prasoideini Clavareau, 1914: 65 [stem: *Prasoide-*]. Type genus: *Prasoidea* Weise, 1907 [syn. of *Odontionopa* Chevrolat, 1836].
*Colasposomini Bechyné, 1957: 7, 8 [stem: *Colasposomat-*]. Type genus: *Colasposoma* Laporte, 1833. Comment: unavailable family-group name, proposed after 1930 without description or bibliographic reference to such a description (Art. 13.1); incorrect original stem formation, not in prevailing usage.
Colasposomini Špringlová de Bechyné, 1960: 3 [stem: *Colasposomat-*]. Type genus: *Colasposoma* Laporte, 1833. Comment: incorrect original stem formation, not in prevailing usage.

### Tribe Habrophorini

Bechyné and Špringlová de Bechyné, 1969

Habrophorini Bechyné and Špringlová de Bechyné, 1969: 75 [stem: *Habrophor-*]. Type genus: *Habrophora* Erichson, 1847.

### Tribe Hemydacnini

Bechyné, 1951

Hemydacnini Bechyné, 1951: 91 [stem: *Hemydacn-*]. Type genus: *Hemydacne* Jacoby, 1897.

### Tribe Megascelidini

Chapuis, 1874

Mégascélides Chapuis, 1874: 82 [stem: *Megascelid-*]. Type genus: *Megascelis* Sturm, 1826. Comment: original vernacular name available (Art. 11.7.2): first used in latinized form by Jacoby and Clavareau (1905: 1, as Megascelidae [incorrect stem formation]), generally accepted as in Riley et al. (2002: 639, as Megascelidini); incorrect original stem formation, not in prevailing usage.

### Tribe Merodini

Chapuis, 1874

Merodites Chapuis, 1874: 327 [stem: *Merod-*]. Type genus: *Meroda* Baly, 1860. Comment: original vernacular name available (Art. 11.7.2): first used in latinized form and generally accepted as in Lefèvre (1885: 128, as Meroditae).

### Tribe Pygomolpini

Bechyné, 1949

Pygomolpini Bechyné, 1949: 532 [stem: *Pygomolp-*]. Type genus: *Pygomolpus* Bechyné, 1949.

### Tribe Rosiroiini

Bechyné, 1950

Rosiroiini Bechyné, 1950b: 148 [stem: *Rosiroi-*]. Type genus: *Rosiroia* Bechyné, 1950.

### Tribe Typophorini

Baly, 1865

Typophorinae Baly, 1865: 433 [stem: *Typophor-*]. Type genus: *Typophorus* Chevrolat, 1836.
Callisinites Chapuis, 1874: 263 [stem: *Callisin-*]. Type genus: *Callisina* Baly, 1860. Comment: original vernacular name available (Art. 11.7.2): first used in latinized form and generally accepted as in Lefèvre (1885: 63, as Callisinitae).
Métachromites Chapuis, 1874: 295 [stem: *Metachromat-*]. Type genus: *Metachroma* Chevrolat, 1836. Comment: original vernacular name available (Art. 11.7.2): first used in latinized form by Lefèvre (1885: 92, as Metachromitae), generally accepted as in Riley et al. (2002: 639, as Metachromites [treated as Latin]); incorrect original stem formation, not in prevailing usage.
Nodostomites Chapuis, 1874: 261 [stem: *Nodostomat-*]. Type genus: *Nodostoma* Motschulsky, 1860 [syn. of *Basilepta* Baly, 1860]. Comment: original vernacular name available (Art. 11.7.2): first used in latinized form and generally accepted as in Lefèvre (1885: 56, as Nodostomitae); incorrect original stem formation, not in prevailing usage.
Pagriites Lefèvre, 1884: lxvii [stem: *Pagri-*]. Type genus: *Pagria* Lefèvre, 1884. Comment: original vernacular name available (Art. 11.7.2): first used in latinized form and generally accepted as in Lefèvre (1885: 62, as Pagriitae).
Cheirideitae Lefèvre, 1885: 67 [stem: *Cheiride-*]. Type genus: *Cheiridea* Baly, 1878.
Nodinini Chen, 1940: 487 [stem: *Nodin-*]. Type genus: *Nodina* Motschulsky, 1858.
Basileptini Chûjô, 1956: 9 [stem: *Basilept-*]. Type genus: *Basilepta* Baly, 1860. Comment: name proposed to replace Nodostomini Chapuis, 1874 because of the synonymy of the type genus.
Nodini Selman, 1965: 145 [stem: *Nod-*]. Type genus: *Noda* Chevrolat, 1836 [preoccupied genus name, not *Noda* Schellenberg 1803 [Diptera]; syn. of *Brachypnoea* Gistel, 1848]. Comment: permanently invalid (Art. 39): based on preoccupied type genus.

### Eumolpinae incertae sedis

*Eupalini Verma et al., 2005: 164 [stem: *Eupal-*]. Type genus: *Eupales* Lefèvre, 1885 [although *Floricola* Gistel, 1848 is an older available name for this genus, almost all authors have used *Eupales* Lefèvre, 1885 in the last 125 years; we use *Eupales* Lefèvre, 1885 as valid here pending a vote on its conservation following a recent submission to the Commission by Jolivet and Verma (Bulletin of Zoological Nomenclature 2009: 204; also see Appendix 6)]. Comment: unavailable family-group name, proposed after 1999 without explicit intention (Art. 16.1); included here as “*incertae sedis*” as was done in the most recent volume of the Catalogue of Palaearctic Coleoptera (see Löbl 2010: 83); notice of a new application for the conservation of *Eupales* Lefèvre, 1885 and Eupalini Verma et al. submitted by Jolivet and Verma was recently published in the Bulletin of Zoological Nomenclature (2009: 204; also see Appendix 6).

### Subfamily Spilopyrinae

Chapuis, 1874

Spilopyrites Chapuis, 1874: 259 [stem: *Spilopyr-*]. Type genus: *Spilopyra* Baly, 1860. Comment: original vernacular name available (Art. 11.7.2): first used in latinized form by Lefèvre (1885: 56, as Spilopyritae), generally accepted as in Reid (2000: 852, as Spilopyrinae); First Reviser found (Spilopyrinae Chapuis, 1874 vs Stenomelinae Chapuis, 1874) is Reid (2000: 852).
Stenomèlites Chapuis, 1874: 421 [stem: *Stenomel-*]. Type genus: *Stenomela* Erichson, 1847. Comment: original vernacular name available (Art. 11.7.2): first used in latinized form and generally accepted as in Monrós (1958c: 146, as Stenomelini).
Hornibiini Crowson, 1946: 79, in key [stem: *Hornibi-*]. Type genus: *Hornibius* Fairmaire, 1888 [syn. of *Hornius* Fairmaire, 1885].

### Subfamily Synetinae

LeConte and Horn, 1883

Synetae J. L. LeConte and G. H. Horn, 1883: 338 [stem: *Synet-*]. Type genus: *Syneta* Dejean, 1835.
Synetidae Edwards, 1953: 29 [stem: *Synet-*]. Type genus: *Syneta* Dejean, 1835. Comment: family-group name proposed as new without reference to Synetae J. L. LeConte and G. H. Horn, 1883.

### †Subfamily Protoscelidinae

Medvedev, 1968

Protoscelinae L. N. Medvedev, 1968: 155 [stem: *Protoscelid-*]. Type genus: *Protoscelis* L. N. Medvedev, 1968. Comment: incorrect original stem formation, not in prevailing usage.

### Superfamily Curculionoidea

Latreille, 1802

Curculionites Latreille, 1802: 195 [stem: *Curculion-*]. Type genus: *Curculio* Linnaeus, 1758.

### Family Nemonychidae

Bedel, 1882

Nemonychidae Bedel, 1882: 3, in key [stem: *Nemonych-*]. Type genus: *Nemonyx* Redtenbacher, 1845 [placed on the Official List of Generic Names in Zoology (ICZN 2005c)]. Comment: given precedence over Cimberididae Gozis, 1882 and placed on the Official List of Family-Group Names in Zoology (ICZN 2005c).

### Subfamily Nemonychinae

Bedel, 1882

Nemonychidae Bedel, 1882: 3, in key [stem: *Nemonych-*]. Type genus: *Nemonyx* Redtenbacher, 1845 [placed on the Official List of Generic Names in Zoology (ICZN 2005c)]. Comment: placed on the Official List of Family-Group Names in Zoology (ICZN 2005c).

### Subfamily Cimberidinae

Gozis, 1882

Cimberidae Gozis, 1882: 58 [stem: *Cimberid-*]. Type genus: *Cimberis* Gozis, 1881 [placed on the Official List of Generic Names in Zoology (ICZN 2005c)]. Comment: Cimberidae Gozis, 1882 placed on the Official Index of Rejected and Invalid Family-Group Names in Zoology and Cimberididae Gozis, 1882 placed on the Official List of Family-Group Names in Zoology (ICZN 2005c).

### Tribe Cimberidini

Gozis, 1882

Rhinomacerides Schönherr, 1823: column 1136 [stem: *Rhinomacr-*]. Type genus: *Rhinomacer* sensu A. G. Olivier, 1807 [not *Rhinomacer* Fabricius, 1781; syn. of *Cimberis* Gozis, 1881]. Comment: placed on the Official Index of Rejected and Invalid Family-Group Names (ICZN 2005c).
Cimberidae Gozis, 1882: 58 [stem: *Cimberid-*]. Type genus: *Cimberis* Gozis, 1881 [placed on the Official List of Generic Names in Zoology (ICZN 2005c)]. Comment: Cimberidae Gozis, 1882 placed on the Official Index of Rejected and Invalid Family-Group Names in Zoology and Cimberididae Gozis, 1882 placed on the Official List of Family-Group Names in Zoology (ICZN 2005c); incorrect original stem formation, not in prevailing usage.
*Neocimberini O’Brien and Wibmer, 1982: 3 [stem: *Neocimberid-*]. Type genus: *Neocimberis* O’Brien and Wibmer, 1982 [placed on the Official Index of Rejected and Invalid Generic Names in Zoology (ICZN 2005c)]. Comment: unavailable family-group name, proposed after 1930 without description or bibliographic reference to such a description (Art. 13.1); incorrect original stem formation, not in prevailing usage.

### Tribe Doydirhynchini

Pierce, 1916

Doydirhynchoidea Pierce, 1916: 463, in key [stem: *Doydirhynch-*]. Type genus: *Doydirhynchus* Dejean, 1821.

### †Tribe Kuschelomacrini

Riedel, 2010

Kuschelomacerini Riedel, 2010: 31 [stem: *Kuschelomacr-*]. Type genus: *Kuschelomacer* Riedel, 2010. Comment: incorrect original stem formation, not in prevailing usage.

### Subfamily Rhinorhynchinae

Voss, 1922

Rhinorhynchini Voss, 1922: 17 [stem: *Rhinorhynch-*]. Type genus: *Rhinorhynchus* Sharp, 1882.

### Tribe Mecomacerini

Kuschel, 1994

Mecomacerini Kuschel, 1994: 576, in key [stem: *Mecomacer-*]. Type genus: *Mecomacer* Kuschel, 1954. Comment: current spelling maintained (Art. 29.5): incorrect original stem formation in prevailing usage (should be *Mecomacr*-).

### Subtribe Brarina

Legalov, 2009

Brarina Legalov, 2009b: 208 [stem: *Brar-*]. Type genus: *Brarus* Kuschel, 1997.

### Subtribe Mecomacerina

Kuschel, 1994

*Mecomacerini May, 1993: 15, in key [stem: *Mecomacer-*]. Type genus: *Mecomacer* Kuschel, 1954. Comment: unavailable family-group name, description shared with Rhynchitoplesiini, not unequivocal (Art. 13.1.1) (see Alonso-Zarazaga and Lyal 1999: 27).
*Rhynchitoplesiini May, 1993: 15, in key [stem: *Rhynchitoplesi-*]. Type genus: *Rhynchitoplesius* Voss, 1952. Comment: unavailable family-group name, description shared with Mecomacerini, not unequivocal (Art. 13.1.1) (see Alonso-Zarazaga and Lyal 1999: 27).
Mecomacerini Kuschel, 1994: 576, in key [stem: *Mecomacer-*]. Type genus: *Mecomacer* Kuschel, 1954. Comment: current spelling maintained (Art. 29.5): incorrect original stem formation in prevailing usage (should be *Mecomacr*-).

### Tribe Rhinorhynchini

Voss, 1922

Rhinorhynchini Voss, 1922: 17 [stem: *Rhinorhynch-*]. Type genus: *Rhinorhynchus* Sharp, 1882.
Rhynchitomacerini May, 1993: 15, in key [stem: *Rhynchitomacr-*]. Type genus: *Rhynchitomacer* Voss, 1937. Comment: incorrect original stem formation, not in prevailing usage.

### †Subfamily Slonikinae

Zherikhin, 1977

Slonikinae Zherikhin, 1977: 179 [stem: *Slonik-*]. Type genus: *Slonik* Zherikhin, 1977.

### †Tribe Slonikini

Zherikhin, 1977

Slonikinae Zherikhin, 1977: 179 [stem: *Slonik-*]. Type genus: *Slonik* Zherikhin, 1977.

### †Tribe Ulyaniscini

Legalov, 2009

Ulyaniscini Legalov, 2009a: 128 [stem: *Ulyanisc-*]. Type genus: *Ulyanisca* Gratschev, 1998.

### †Subfamily Eccoptarthrinae

Arnoldi, 1977

Eccoptarthrini Arnoldi, 1977: 169 [stem: *Eccoptarthr-*]. Type genus: *Eccoptarthrus* Arnoldi, 1977.
Mesophyletinae Poinar, 2006: 879 [stem: *Mesophylet-*]. Type genus: *Mesophyletis* Poinar, 2006. Comment: Poinar (2008: 262) unnecessarily revalidated this name on the basis of Art. 16.2, the criteria of availability were met in the original description in 2006 where the name of the type genus was cited.

### †Subfamily Brenthorrhininae

Arnoldi, 1977

Brenthorrhininae Arnoldi, 1977: 172 [stem: *Brenthorrhin-*]. Type genus: *Brenthorrhinus* Arnoldi, 1977. Comment: placement according to Legalov (2009c: 289).

### †Tribe Brenthorrhinini

Arnoldi, 1977

Brenthorrhininae Arnoldi, 1977: 172 [stem: *Brenthorrhin-*]. Type genus: *Brenthorrhinus* Arnoldi, 1977.

### †Tribe Brenthorrhinoidini

Legalov, 2003

Brenthorrhinoidini Legalov, 2003: 88 [stem: *Brenthorrhinoid-*]. Type genus: *Brenthorrhinoides* Gratshev and Zherikhin, 1996.

### †Subfamily Distenorrhininae

Arnoldi, 1977

Distenorrhinini Arnoldi, 1977: 170 [stem: *Distenorrhin-*]. Type genus: *Distenorrhinus* Arnoldi, 1977. Comment: placement according to Legalov (2009c: 290).

### †Subfamily Eobelinae

Arnoldi, 1977

Eobelidae Arnoldi, 1977: 144 [stem: *Eobel-*]. Type genus: *Eobelus* Arnoldi, 1977. Comment: precedence (Oxycorynoidinae Arnoldi, 1977 vs Nanophydinae Arnoldi, 1977 vs Eobelinae Arnoldi, 1977) given to taxon originally proposed at the higher rank (Art. 24.1).

### †Tribe Eobelini

Arnoldi, 1977

Eobelidae Arnoldi, 1977: 144 [stem: *Eobel-*]. Type genus: *Eobelus* Arnoldi, 1977. Comment: precedence (Eobelini Arnoldi, 1977 vs Procurculionini Arnoldi, 1977) given to taxon originally proposed at the higher rank (Art. 24.1).

### †Subtribe Eobelina

Arnoldi, 1977

Eobelidae Arnoldi, 1977: 144 [stem: *Eobel-*]. Type genus: *Eobelus* Arnoldi, 1977.

### †Subtribe Procurculionina

Arnoldi, 1977

Procurculionini Arnoldi, 1977: 157 [stem: *Procurculion-*]. Type genus: *Procurculio* Arnoldi, 1977. Comment: First Reviser (Eccoptothoracina Arnoldi, 1977 vs Procurculionina Arnoldi, 1977) not determined, current usage maintained.
Eccoptothoracini Arnoldi, 1977: 158 [stem: *Eccoptothorac-*]. Type genus: *Eccoptothorax* Arnoldi, 1977.

### †Tribe Karataucarini

Legalov, 2009

Karataucarini Legalov, 2009c: 288 [stem: *Karataucar-*]. Type genus: *Karataucar* Legalov, 2009.

### †Tribe Nanophydini

Arnoldi, 1977

Nanophydinae Arnoldi, 1977: 173 [stem: *Nanophyd-*]. Type genus: *Nanophydes* Arnoldi, 1977.

### †Tribe Oxycorynoidini

Arnoldi, 1977

Oxycorynoidinae Arnoldi, 1977: 159 [stem: *Oxycorynoid-*]. Type genus: *Oxycorynoides* Arnoldi, 1977.

### †Tribe Probelini

Legalov, 2009

Probelini Legalov, 2009c: 287 [stem: *Probel-*]. Type genus: *Probelus* Arnoldi, 1977.

### †Subfamily Paleocartinae

Legalov, 2003

Paleocartini Legalov, 2003: 78 [stem: *Paleocart-*]. Type genus: *Paleocartus* Legalov, 2003.

### †Tribe Nebrenthorrhinini

Legalov, 2007

Nebrenthorrhinina Legalov, 2007: 34 [stem: *Nebrenthorrhin-*]. Type genus: *Nebrenthorrhinus* Legalov, 2003. Comment: placement according to Legalov (2009c: 288).

### †Tribe Paleocartini

Legalov, 2003

Paleocartini Legalov, 2003: 78 [stem: *Paleocart-*]. Type genus: *Paleocartus* Legalov, 2003. Comment: placement according to Legalov (2009c: 288).

### †Subfamily Metrioxenoidinae

Legalov, 2009

Metrioxenoidinae Legalov, 2009c: 288 [stem: *Metrioxenoid-*]. Type genus: *Metrioxenoides* Gratshev et al., 1998.

### †Subfamily Cretonemonychinae

Gratshev and Legalov, 2009

Cretonemonychinae Gratshev and Legalov, 2009: 412 [stem: *Cretonemonych-*]. Type genus: *Cretonemonyx* Gratshev and Legalov, 2009.

### †Subfamily Selengarhynchinae

Gratshev and Legalov, 2009

Selengarhynchinae Gratshev and Legalov, 2009: 414 [stem: *Selengarhynch-*]. Type genus: *Selengarhynchus* Gratshev and Legalov, 2009.

### Family Anthribidae

Billberg, 1820

Anthribides Billberg, 1820a: 39 [stem: *Anthrib-*]. Type genus: *Anthribus* Geoffroy, 1762 [placed on the Official List of Generic Names in Zoology (ICZN 1994a)]. Comment: placed on the Official List of Family-Group Names in Zoology and given priority over Choragidae Kirby, 1819 (ICZN 1994b).

### Subfamily Anthribinae

Billberg, 1820

Anthribides Billberg, 1820a: 39 [stem: *Anthrib-*]. Type genus: *Anthribus* Geoffroy, 1762 [placed on the Official List of Generic Names in Zoology (ICZN 1994a)]. Comment: placed on the Official List of Family-Group Names in Zoology and given priority over Choragidae Kirby, 1819 (ICZN 1994b).

### Tribe Anthribini

Billberg, 1820

Anthribides Billberg, 1820a: 39 [stem: *Anthrib-*]. Type genus: *Anthribus* Geoffroy, 1762 [placed on the Official List of Generic Names in Zoology (ICZN 1994a)]. Comment: placed on the Official List of Family-Group Names in Zoology and given priority over Choragidae Kirby, 1819 (ICZN 1994b).
Brachytarsina C. G. Thomson, 1859: 128 [stem: *Brachytars-*]. Type genus: *Brachytarsus* Schönherr, 1823 [syn. of *Anthribus* Geoffroy, 1762].
Brachytarsini Pierce, 1930: 22, in key [stem: *Brachytars-*]. Type genus: *Brachytarsus* Schönherr, 1823 [syn. of *Anthribus* Geoffroy, 1762]. Comment: family-group name proposed as new without reference to Brachytarsina C. G. Thomson, 1859.

### Tribe Basitropini

Lacordaire, 1865

Basitropides Lacordaire, 1865: 566 [stem: *Basitrop-*]. Type genus: *Basitropis* Jekel, 1855. Comment: original vernacular name available (Art. 11.7.2): first used in latinized form by Stein (1868: 115, as Basitropini), generally accepted as in Alonso-Zarazaga and Lyal (1999: 28, as Basitropidini [incorrect stem formation]); Alonso-Zarazaga and Lyal (1999: 28) used the spelling Basitropidini but the same authors subsequently (2002: 4) reverted to Basitropini; precedence (Basitropini Lacordaire, 1865 vs Eugonini Lacordaire, 1865) given to taxon originally proposed at the higher rank (Art. 24.1).
Eugonides Lacordaire, 1865: 569 [stem: *Eugon-*]. Type genus: *Eugonus* Schönherr, 1833. Comment: original vernacular name available (Art. 11.7.2): first used in latinized form and generally accepted as in Sharp (1873: 32, as Eugonides [treated as Latin]); synonymy with Basitropini by Alonso-Zarazaga and Lyal (2002: 4).
Phaenithonini Pierce, 1930: 4, in key [stem: *Phaenithon-*]. Type genus: *Phaenithon* Schönherr, 1826.

### Tribe Corrhecerini

Lacordaire, 1865

Corrhécérides Lacordaire, 1865: 547 [stem: *Corrhecer-*]. Type genus: *Corrhecerus* Schönherr, 1826. Comment: original vernacular name available (Art. 11.7.2): first used in latinized form by Morimoto (1981: 78, as Corrhecerini), generally accepted as in Alonso-Zarazaga and Lyal (1999: 29, as Corrhecerini).
Nessiarini Morimoto, 1972: 38, in key [stem: *Nessiar-*]. Type genus: *Nessiara* Pascoe, 1860.

### Tribe Cratoparini

LeConte, 1876

Cratopares J. L. LeConte, 1876: 403 [stem: *Cratopar-*]. Type genus: *Cratoparis* Dejean, 1834 [syn. of *Euparius* Schönherr, 1823]. Comment: current spelling maintained (Art. 29.5): incorrect stem formation in prevailing usage (should be *Cratoparent*-).
Eupariini B. D. Valentine, 1960: 49, in key [stem: *Eupari-*]. Type genus: *Euparius* Schönherr, 1823. Comment: junior homonym of Eupariini A. Schmidt, 1910 (type genus *Euparia* Lepeletier and Serville, 1828) in Scarabaeidae.

### †Tribe Cretanthribini

Legalov, 2009

Cretanthribini Legalov, 2009c: 291 [stem: *Cretanthrib-*]. Type genus: *Cretanthribus* Legalov, 2009.

### Tribe Decataphanini

Lacordaire, 1865

Décataphanides Lacordaire, 1865: 556 [stem: *Decataphan-*]. Type genus: *Decataphanes* Labram and Imhoff, 1840. Comment: original vernacular name available (Art. 11.7.2): first used in latinized form by Kolbe (1897: 289, as Decatophaninae [incorrect stem formation]), generally accepted as in Alonso-Zarazaga and Lyal (1999: 30, as Decataphanini).

### Tribe Discotenini

Lacordaire, 1865

Discoténides Lacordaire, 1865: 500 [stem: *Discoten-*]. Type genus: *Discotenes* Labram and Imhoff, 1841. Comment: original vernacular name available (Art. 11.7.2): first used in latinized form by Pierce (1930: 4, as Discotenini), generally accepted as in Alonso-Zarazaga and Lyal (1999: 30, as Discotenini).

### Tribe Ecelonerini

Lacordaire, 1865

Ecélonérides Lacordaire, 1865: 562 [stem: *Eceloner-*]. Type genus: *Ecelonerus* Schönherr, 1839. Comment: original vernacular name available (Art. 11.7.2): first used in latinized form by Morimoto (1972: 38, as Ecelonerini), generally accepted as in Alonso-Zarazaga and Lyal (1999: 30, as Ecelonerini).

### Tribe Ischnocerini

Lacordaire, 1865

Ischnocérides Lacordaire, 1865: 504 [stem: *Ischnocer-*]. Type genus: *Ischnocerus* Schönherr, 1839. Comment: original vernacular name available (Art. 11.7.2): first used in latinized form by J. L. LeConte (1876: 393, as Ischnoceri), generally accepted as in Alonso-Zarazaga and Lyal (2002: 5, as Ischnocerini); the Hymenoptera name Ischnocerini Clement, 1938 (type genus *Ischnoceros* Gravenhorst, 1829) is apparently unavailable.
Meconemini Pierce, 1930: 4, in key [stem: *Meconem-*]. Type genus: *Meconemus* Labram and Imhoff, 1838.

### Tribe Gymnognathini

Valentine, 1960

Gymnognathini B. D. Valentine, 1960: 48, in key [stem: *Gymnognath-*]. Type genus: *Gymnognathus* Schönherr, 1826.

### Tribe Jordanthribini

Morimoto, 1980

Jordanthribini Morimoto, 1980: 16 [stem: *Jordanthrib-*]. Type genus: *Jordanthribus* Zimmerman, 1938.

### Tribe Mauiini

Valentine, 1990

Mauiini B. D. Valentine, 1990: 235, in key [stem: *Maui-*]. Type genus: *Mauia* Blackburn, 1885.

### Tribe Mecocerini

Lacordaire, 1865

Mécocérides Lacordaire, 1865: 493 [stem: *Mecocer-*]. Type genus: *Mecocerus* Schönherr, 1833 [placed on the Official List of Generic Names in Zoology (ICZN 1972); syn. of *Acanthothorax* Gaede, 1832]. Comment: original vernacular name available (Art. 11.7.2): first used in latinized form by Kolbe (1897: 288, as Mecocerinae), generally accepted as in Alonso-Zarazaga and Lyal (1999: 31, as Mecocerini).
Phloeophilides Lacordaire, 1865: 517 [stem: *Phloeophil-*]. Type genus: *Phloeophilus* Schönherr, 1833. Comment: original vernacular name available (Art. 11.7.2): first used in latinized form and generally accepted as in Olliff (1891: 75, as Phloeophilides [treated as Latin]); the older name Phloeophilini Kiesenwetter, 1863 [Cleroidea] was based on an unjustified emendation, the correct stem for the cleroid family-group name is *Phloiophil*-.
Cappadocini Alonso-Zarazaga and Lyal, 1999: 28 [stem: *Cappadoc-*]. Type genus: *Cappadox* Alonso-Zarazaga and Lyal, 1999 [syn. of *Phloeophilus* Schönherr, 1833].

### Tribe Mycteini

Morimoto, 1972

Mycteini Morimoto, 1972: 38, in key [stem: *Mycte-*]. Type genus: *Mycteis* Pascoe, 1860.

### Tribe Ozotomerini

Morimoto, 1972

Ozotomerini Morimoto, 1972: 37, in key [stem: *Ozotomer-*]. Type genus: *Ozotomerus* Perroud, 1853.

### Tribe Piesocorynini

Valentine, 1960

Piesocorynini B. D. Valentine, 1960: 49, in key [stem: *Piesocoryn-*]. Type genus: *Piesocorynus* Dejean, 1834.

### Tribe Platyrhinini

Bedel, 1882

Platyrrhinidae Bedel, 1882: 3, in key [stem: *Platyrhin-*]. Type genus: *Platyrhinus* Clairville, 1798. Comment: incorrect original stem formation, not in prevailing usage.

### Tribe Platystomini

Pierce, 1916

Anthotribidae Gistel, 1856a: 375 [stem: *Anthotrib-*]. Type genus: *Anthotribus* Gistel, 1856 [preoccupied genus name, not *Anthotribus* Hoffmann, 1803 [Coleoptera: Anthribidae: Anthribinae: Anthribini]; syn. of *Platystomos* Schneider, 1791]. Comment: permanently invalid (Art. 39): based on preoccupied type genus.
Platystomoidea Pierce, 1916: 463, in key [stem: *Platystom-*]. Type genus: *Platystomos* Schneider, 1791. Comment: the type genus for this family-group name was in fact proposed as a replacement name for *Anthribus* sensu Fabricius, 1790, however it was never used in that sense subsequently (Alonso-Zarazaga and Lyal 1999: 33); an application will be submitted by MAAZ and CHCL to conserve the current concept of *Platystomos* Schneider, 1791 and its associated family-group name.

### Tribe Proscoporhinini

Lacordaire, 1865

Proscoporhinides Lacordaire, 1865: 544 [stem: *Proscoporhin-*]. Type genus: *Proscoporhinus* Montrouzier, 1861. Comment: original vernacular name available (Art. 11.7.2): first used in latinized form by Ienistea (1986: 32, as Prosoporhinidae [incorrect stem formation]), generally accepted as in Alonso-Zarazaga and Lyal (1999: 33, as Proscoporhinini).

### Tribe Ptychoderini

Jekel, 1855

Ptychoderidae Jekel, 1855: 70 [stem: *Ptychoder-*]. Type genus: *Ptychoderes* Schönherr, 1823.
*Phloeotragides Lacordaire, 1865: 486 [stem: *Phloeotrag-*]. Type genus: *Phloeotragus* Schönherr, 1823. Comment: original vernacular name unavailable (Art. 11.7.2): subsequently used in latinized form but not generally attributed to Lacordaire (1865) and used as valid.
Phloeotraginae Kolbe, 1897: 288 [stem: *Phloeotrag-*]. Type genus: *Phloeotragus* Schönherr, 1823.

### Tribe Sintorini

Lacordaire, 1865

Sintorides Lacordaire, 1865: 510 [stem: *Sintor-*]. Type genus: *Sintor* Schönherr, 1839. Comment: original vernacular name available (Art. 11.7.2): first used in latinized form by Morimoto (1972: 38, as Sintorini), generally accepted as in Alonso-Zarazaga and Lyal (1999: 34, as Sintorini).

### Tribe Stenocerini

Kolbe, 1895

Stenocerinarum Kolbe, 1895: 381 [stem: *Stenocer-*]. Type genus: *Stenocerus* Schönherr, 1826. Comment: Stenocerinarum is thegenitive form of Stenocerinae and was recognized as such by Alonso-Zarazaga and Lyal (1999: 34).
Allandrini Pierce, 1930: 18 [stem: *Allandr-*]. Type genus: *Allandrus* J. L. LeConte, 1876.

### Tribe Tophoderini

Lacordaire, 1865

Tophodérides Lacordaire, 1865: 499 [stem: *Tophoder-*]. Type genus: *Tophoderes* Dejean, 1834. Comment: original vernacular name available (Art. 11.7.2): first used in latinized form by Kolbe (1897: 288, as Tophoderinae), generally accepted as in Alonso-Zarazaga and Lyal (1999: 34, as Tophoderini).

### Tribe Trigonorhinini

Valentine, 1999

Trigonorhinini B. D. Valentine, 1999: 287, in key [stem: *Trigonorhin-*]. Type genus: *Trigonorhinus* Wollaston, 1861.

### Tribe Tropiderini

Lacordaire, 1865

Tropidérides Lacordaire, 1865: 484 [stem: *Tropider-*]. Type genus: *Tropideres* Schönherr, 1823. Comment: original vernacular name available (Art. 11.7.2): first used in latinized form by Stein (1868: 115, as Tropiderini), generally accepted as in Alonso-Zarazaga and Lyal (1999: 35, as Tropiderini); precedence (Tropiderini Lacordaire, 1865 vs Acorynini Lacordaire, 1865) given to taxon originally proposed at the higher rank (Art. 24.1).
Acorynides Lacordaire, 1865: 512 [stem: *Acoryn-*]. Type genus: *Acorynus* Schönherr, 1833. Comment: original vernacular name available (Art. 11.7.2): first used in latinized form by Kolbe (1897: 289, as Acoryninae), generally accepted as in Morimoto (1980: 22, as Acorynini).
Eurymycterini Pierce, 1930: 15 [stem: *Eurymycter-*]. Type genus: *Eurymycter* J. L. LeConte, 1876.

### Tribe Xenocerini

Lacordaire, 1865

Xénocerides Lacordaire, 1865: 558 [stem: *Xenocer-*]. Type genus: *Xenocerus* Schönherr, 1833. Comment: original vernacular name available (Art. 11.7.2): first used in latinized form by Heyne and Taschenberg (1907: 235, as Xenocerini), generally accepted as in Alonso-Zarazaga and Lyal (1999: 35, as Xenocerini).

### Tribe Xylinadini

Lacordaire, 1865

Xylinadides Lacordaire, 1865: 560 [stem: *Xylinad-*]. Type genus: *Xylinades* Latreille, 1828 [syn. of *Xylinada* Berthold, 1827]. Comment: original vernacular name available (Art. 11.7.2): first used in latinized form by Kolbe (1897: 289, as Xylinadinae), generally accepted as in Alonso-Zarazaga and Lyal (1999: 36, as Xylinadini).

### Tribe Zygaenodini

Lacordaire, 1865

Zygénodides Lacordaire, 1865: 542 [stem: *Zygaenod-*]. Type genus: *Zygaenodes* Pascoe, 1859 [syn. of *Exechesops* Schönherr, 1847]. Comment: original vernacular name available (Art. 11.7.2): first used in latinized form by Kolbe (1897: 289, as Zygaenodinae), generally accepted as in Alonso-Zarazaga and Lyal (1999: 36, as Zygaenodini); incorrect original stem formation, not in prevailing usage.
Hormisci J. L. LeConte, 1876: 396 [stem: *Ormisc-*]. Type genus: *Ormiscus* G. R. Waterhouse, 1845 [as *Hormiscus*, unjustified emendation of type genus name by Agassiz (1846b: 263), not in prevailing usage]. Comment: incorrect original stem formation, not in prevailing usage.

### Subfamily Choraginae

Kirby, 1819

Choragidae Kirby, 1819: 447 [stem: *Chorag-*]. Type genus: *Choragus* Kirby, 1819 [placed on the Official List of Generic Names in Zoology (ICZN 1994b)]. Comment: placed on the Official List of Family-Group Names in Zoology and not given priority over Anthribidae Billberg, 1820 (ICZN 1994b).

### Tribe Apolectini

Lacordaire, 1865

Apolectides Lacordaire, 1865: 554 [stem: *Apolect-*]. Type genus: *Apolecta* Pascoe, 1859. Comment: original vernacular name available (Art. 11.7.2): first used in latinized form by Kolbe (1897: 289, as Apolectinae), generally accepted as in Alonso-Zarazaga and Lyal (1999: 28, as Apolectini); Apolectidae Jordan, 1923 (type genus *Apolectus* Cuvier, 1832) is available in Pisces although it is based on a preoccupied genus name and therefore permanently invalid; this case is to be referred to the Commission to remove the homonymy (Art. 55.3.1).

### Tribe Araecerini

Lacordaire, 1865

Araeocérides Lacordaire, 1865: 588 [stem: *Araecer-*]. Type genus: *Araecerus* Schönherr, 1823 [as *Araeocerus*, unjustified emendation of type genus name by Schönherr (1839), not in prevailing usage]. Comment: original vernacular name available (Art. 11.7.2): first used in latinized form by Stein (1868: 115, as Araeocerini), generally accepted as in Alonso-Zarazaga and Lyal (1999: 37, as Araecerini); incorrect original stem formation, not in prevailing usage.

### Tribe Cisanthribini

Zimmerman, 1994

Cisanthribini Zimmerman, 1994a: 232 [stem: *Cisanthrib-*]. Type genus: *Cisanthribus* Zimmerman, 1938.

### Tribe Choragini

Kirby, 1819

Choragidae Kirby, 1819: 447 [stem: *Chorag-*]. Type genus: *Choragus* Kirby, 1819 [placed on the Official List of Generic Names in Zoology (ICZN 1994b)]. Comment: not given priority over Anthribidae Billberg, 1820, placed on the Official List of Family-Group Names in Zoology (ICZN 1994b).

### Tribe Valenfriesiini

Alonso-Zarazaga and Lyal, 1999

Notioxénides Lacordaire, 1865: 593 [stem: *Notioxen-*]. Type genus: *Notioxenus* Wollaston, 1861 [preoccupied genus name, not *Notioxenus* Motschulsky, 1858 [Coleoptera: Carabidae]; syn. of *Valenfriesia* Alonso-Zarazaga and Lyal, 1999]. Comment: original vernacular name available (Art. 11.7.2): first used in latinized form by Pierce (1930: 31, as Notioxenini), generally accepted as in Morimoto (1978a: 18, as Notioxenini); permanently invalid (Art. 39): based on preoccupied type genus.
Valenfriesiini Alonso-Zarazaga and Lyal, 1999: 38 [stem: *Valenfriesi-*]. Type genus: *Valenfriesia* Alonso-Zarazaga and Lyal, 1999. Comment: replacement name for Notioxenini Lacordaire, 1865 because of the homonymy of the type genus.

### Tribe Xenorchestini

Lacordaire, 1865

Xénorchestides Lacordaire, 1865: 595 [stem: *Xenorchest-*]. Type genus: *Xenorchestes* Wollaston, 1854. Comment: original vernacular name available (Art. 11.7.2): first used in latinized form by J. L. LeConte (1876: 408, as Xenorchestini), generally accepted as in Alonso-Zarazaga and Lyal (1999: 38, as Xenorchestini).
Homoeoderides Wollaston, 1870: 23 [stem: *Homoeoder-*]. Type genus: *Homoeodera* Wollaston, 1870.

### Subfamily Urodontinae

Thomson, 1859

Urodontides C. G. Thomson, 1859: 128 [stem: *Urodont-*]. Type genus: *Urodon* Schönherr, 1823 [syn. of *Bruchela* Dejean, 1821].
Bruchelidae Pierce, 1916: 463 [stem: *Bruchel-*]. Type genus: *Bruchela* Dejean, 1821.

### †Family Ulyanidae

Zherikhin, 1993

Ulyanidae Zherikhin, 1993: 26 [stem: *Ulyan-*]. Type genus: *Ulyana* Zherikhin, 1993.

### Family Belidae

Schönherr, 1826

Belides Schönherr, 1826: 73 [stem: *Bel-*]. Type genus: *Belus* Schönherr, 1823 [syn. of *Rhinotia* Kirby, 1819].

### Subfamily Belinae

Schönherr, 1826

Belides Schönherr, 1826: 73 [stem: *Bel-*]. Type genus: *Belus* Schönherr, 1823 [syn. of *Rhinotia* Kirby, 1819].

### Tribe Agnesiotidini

Zimmerman, 1994

Agnesiotidini Zimmerman, 1994a: 258 [stem: *Agnesiotid-*]. Type genus: *Agnesiotis* Pascoe, 1870.

### Tribe Belini

Schönherr, 1826

Belides Schönherr, 1826: 73 [stem: *Bel-*]. Type genus: *Belus* Schönherr, 1823 [syn. of *Rhinotia* Kirby, 1819].

### Subtribe Belina

Schönherr, 1826

Belides Schönherr, 1826: 73 [stem: *Bel-*]. Type genus: *Belus* Schönherr, 1823 [syn. of *Rhinotia* Kirby, 1819].

### Subtribe Homalocerina

Legalov, 2009

Homalocerina Legalov, 2009d: 308 [stem: *Homalocer-*]. Type genus: *Homalocerus* Schönherr, 1839.

### Tribe Pachyurini

Kuschel, 1959

Pachyurini Kuschel, 1959a: 253, in key [stem: *Pachyur-*]. Type genus: *Pachyura* Hope, 1834.

### Subfamily Oxycoryninae

Schönherr, 1840

Oxycorynides Schönherr, 1840: 581 [stem: *Oxycoryn-*]. Type genus: *Oxycorynus* Chevrolat, 1832.

### Tribe Aglycyderini

Wollaston, 1864

Aglycyderidae Wollaston, 1864: 384 [stem: *Aglycyder-*]. Type genus: *Aglycyderes* Westwood, 1864.
Proterhinides Fauvel, 1891: 154 [stem: *Proterhin-*]. Type genus: *Proterhinus* Sharp, 1878. Comment: original vernacular name available (Art. 11.7.2): first used in latinized form and generally accepted as in Sharp (1899a: 298, as Proterhinidae).
Platycephalitae Paulian, 1944: 118 [stem: *Platycephal-*]. Type genus: *Platycephala* Montrouzier, 1861 [preoccupied genus name, not *Platycephala* Fallén, 1820 [Diptera]; syn. of *Aralius* Kuschel, 1990]. Comment: permanently invalid (Art. 39): based on preoccupied type genus; also Platycephalidae Gill, 1872 (type genus *Platycephalus* Bloch, 1795) is available in Pisces.

### Tribe Alloxycorynini

Legalov, 2009

Alloxycorynini Legalov, 2009d: 313 [stem: *Alloxycoryn-*]. Type genus: *Alloxycorynus* Voss, 1957.

### Tribe Distenorrhinoidini

Legalov, 2009

Distenorrhinoidini Legalov, 2009d: 313 [stem: *Distenorrhinoid-*]. Type genus: *Distenorrhinoides* Gratshev and Zherikhin, 2000.

### Tribe Metrioxenini

Voss, 1953

Metrioxenini Voss, 1953: 124, in key [stem: *Metrioxen-*]. Type genus: *Metrioxena* Pascoe, 1870.

### Subtribe Afrocorynina

Voss, 1957

Afrocorynini Voss, 1957: 102, in key [stem: *Afrocoryn-*]. Type genus: *Afrocorynus* G. A. K. Marshall, 1955.
Hispodini Voss, 1957: 102, in key [stem: *Hispod-*]. Type genus: *Hispodes* G. A. K. Marshall, 1955.

### Subtribe Metrioxenina

Voss, 1953

Metrioxenini Voss, 1953: 124, in key [stem: *Metrioxen-*]. Type genus: *Metrioxena* Pascoe, 1870.

### Subtribe Zherichinixenina

Legalov, 2009

Zherichinixenina Legalov, 2009d: 310 [stem: *Zherichinixen-*]. Type genus: *Zherichinixena* Legalov, 2009.

### Tribe Oxycorynini

Schönherr, 1840

Oxycorynides Schönherr, 1840: 581 [stem: *Oxycoryn-*]. Type genus: *Oxycorynus* Chevrolat, 1832.

### Subtribe Allocorynina

Sharp, 1890

Allocoryninae Sharp, 1890: 45 [stem: *Allocoryn-*]. Type genus: *Allocorynus* Sharp, 1890 [syn. of *Rhopalotria* Chevrolat, 1878].

### Subtribe Oxycorynina

Schönherr, 1840

Oxycorynides Schönherr, 1840: 581 [stem: *Oxycoryn-*]. Type genus: *Oxycorynus* Chevrolat, 1832.

### Subtribe Oxycraspedina

Marvaldi and Oberprieler, 2006

Oxycraspedina Marvaldi and Oberprieler, 2006: 460 [stem: *Oxycrasped-*]. Type genus: *Oxycraspedus* Kuschel, 1955.

### Family Caridae

Thompson, 1992

Carinae Thompson, 1992: 882 [stem: *Car-*]. Type genus: *Car* Blackburn, 1897.

### Subfamily Carinae

Thompson, 1992

Carinae Thompson, 1992: 882 [stem: *Car-*]. Type genus: *Car* Blackburn, 1897.
Caridae Zimmerman, 1994a: 499 [stem: *Car-*]. Type genus: *Car* Blackburn, 1897. Comment: family-group name proposed as new without reference to Carinae Thompson, 1992.
Carinae Kuschel, 1995: 18 [stem: *Car-*]. Type genus: *Car* Blackburn, 1897. Comment: family-group name proposed as new without reference to Carinae Thompson, 1992 or Caridae Zimmerman, 1994.

### Subfamily Chilecarinae

Legalov, 2009

Chilecarini Legalov, 2009a: 125 [stem: *Chilecar-*]. Type genus: *Chilecar* Kuschel, 1992.

### Tribe Carodini

Legalov, 2009

Carodesina Legalov, 2009a: 126 [stem: *Carod-*]. Type genus: *Carodes* Zimmerman, 1994. Comment: incorrect original stem formation, not in prevailing usage.

### Tribe Chilecarini

Legalov, 2009

Chilecarini Legalov, 2009a: 125 [stem: *Chilecar-*]. Type genus: *Chilecar* Kuschel, 1992.

### †Subfamily Baissorhynchinae

Zherikhin, 1993

Baissorhynchini Zherikhin, 1993: 30 [stem: *Baissorhynch-*]. Type genus: *Baissorhynchus* Zherikhin, 1977.
†Abrocarina Legalov, 2009c: 291 [stem: *Abrocar-*]. Type genus: *Abrocar* Liu and Ren, 2006.

### Family Attelabidae

Billberg, 1820

Attelabides Billberg, 1820a: 39 [stem: *Attelab-*]. Type genus: *Attelabus* Linnaeus, 1758 [placed on the Official List of Generic Names in Zoology (ICZN 1983a)].

### Subfamily Attelabinae

Billberg, 1820

Attelabides Billberg, 1820a: 39 [stem: *Attelab-*]. Type genus: *Attelabus* Linnaeus, 1758 [placed on the Official List of Generic Names in Zoology (ICZN 1983a)].

### Tribe Attelabini

Billberg, 1820

Attelabides Billberg, 1820a: 39 [stem: *Attelab-*]. Type genus: *Attelabus* Linnaeus, 1758 [placed on the Official List of Generic Names in Zoology (ICZN 1983a)].

### Subtribe Attelabina

Billberg, 1820

Attelabides Billberg, 1820a: 39 [stem: *Attelab-*]. Type genus: *Attelabus* Linnaeus, 1758 [placed on the Official List of Generic Names in Zoology (ICZN 1983a)]. Comment: this family-group name was also used in the same year by Billberg (1820b: 393, as Attelabides).

### Subtribe Euscelina

Voss, 1925

Euscelina Voss, 1925: 32 [stem: *Euscel-*]. Type genus: *Euscelus* Schönherr, 1833.
Alleuscelina Legalov, 2003: 411 [stem: *Alleuscel-*]. Type genus: *Alleuscelus* Voss, 1937. Comment: proposed as a subtribe of Euscelini.
Clinolabina Legalov, 2003: 410 [stem: *Clinolab-*]. Type genus: *Clinolabus* Jekel, 1860. Comment: proposed as a subtribe of Euscelini.

### Subtribe Euscelophilina

Voss, 1925

Euscelophilina Voss, 1925: 29 [stem: *Euscelophil-*]. Type genus: *Euscelophilus* Voss, 1925.

### Subtribe Henicolabina

Legalov, 2007

Henicolabina Legalov, 2007: 282 [stem: *Henicolab-*]. Type genus: *Henicolabus* Voss, 1925.

### Subtribe Himatolabina

Legalov, 2003

Himatolabina Legalov, 2003: 424 [stem: *Himatolab-*]. Type genus: *Himatolabus* Jekel, 1860. Comment: proposed as a subtribe of Hybolabini.

### Subtribe Hybolabina

Voss, 1925

Hybolabina Voss, 1925: 191 [stem: *Hybolab-*]. Type genus: *Hybolabus* Jekel, 1860.

### Subtribe Isolabina

Legalov, 2007

Isolabina Legalov, 2007: 282 [stem: *Isolab-*]. Type genus: *Isolabus* Voss, 1925.

### Subtribe Lagenoderina

Voss, 1925

Lagenoderina Voss, 1925: 206 [stem: *Lagenoder-*]. Type genus: *Lagenoderus* A. White, 1841.

### Subtribe Lamprolabina

Voss, 1925

Lamprolabina Voss, 1925: 213 [stem: *Lamprolab-*]. Type genus: *Lamprolabus* Jekel, 1860.

### Subtribe Metocalolabina

Legalov, 2003

Metocalolabina Legalov, 2003: 433 [stem: *Metocalolab-*]. Type genus: *Metocalolabus* Legalov, 2003.

### Subtribe Omolabina

Legalov, 2003

Omolabina Legalov, 2003: 426 [stem: *Omolab-*]. Type genus: *Omolabus* Jekel, 1860. Comment: proposed as a subtribe of Hybolabini.

### Subtribe Paramecolabina

Legalov, 2003

Paramecolabina Legalov, 2003: 439 [stem: *Paramecolab-*]. Type genus: *Paramecolabus* Jekel, 1860.

### Subtribe Phialodina

Legalov, 2003

Phialodina Legalov, 2003: 437 [stem: *Phialod-*]. Type genus: *Phialodes* Roelofs, 1874.

### Subtribe Phymatolabina

Voss, 1925

Phymatolabina Voss, 1925: 199 [stem: *Phymatolab-*]. Type genus: *Phymatolabus* Jekel, 1860.

### Subtribe Phymatopsinina

Legalov, 2003

Phymatopsinina Legalov, 2003: 456 [stem: *Phymatopsin-*]. Type genus: *Phymatopsinus* Voss, 1925. Comment: proposed as a subtribe of Lagenoderini.

### Subtribe Pleurolabina

Legalov, 2003

Pleurolabina Legalov, 2003: 460 [stem: *Pleurolab-*]. Type genus: *Pleurolabus* Jekel, 1860. Comment: proposed as a subtribe of Lagenoderini.

### Tribe Euopini

Voss, 1925

Euopsini Voss, 1925: 291 [stem: *Euop-*]. Type genus: *Euops* Schönherr, 1839. Comment: incorrect original stem formation, not in prevailing usage.
Archeuopsina Legalov, 2003: 359 [stem: *Archeuop-*]. Type genus: *Archeuops* Legalov, 2003. Comment: proposed as a subtribe of Euopini; incorrect original stem formation, not in prevailing usage.
Suniopsina Legalov, 2003: 364 [stem: *Suniop-*]. Type genus: *Suniops* Voss, 1928. Comment: proposed as a subtribe of Euopini; incorrect original stem formation, not in prevailing usage.
Synaptopsina Legalov, 2003: 368 [stem: *Synaptop-*]. Type genus: *Synaptops* Jekel, 1860. Comment: proposed as a subtribe of Euopini; incorrect original stem formation, not in prevailing usage.
Ljudmilinina Legalov, 2007: 219 [stem: *Ljudmilini-*]. Type genus: *Ljudmilinius* Legalov, 2003. Comment: proposed as a subtribe of Euopini; incorrect original stem formation, not in prevailing usage.
Parasynaptopsisina Legalov, 2007: 227 [stem: *Parasynaptopse-*]. Type genus: *Parasynaptopsis* Legalov, 2003. Comment: proposed as a subtribe of Euopini; incorrect original stem formation, not in prevailing usage.
Riedelinina Legalov, 2007: 218 [stem: *Riedelini-*]. Type genus: *Riedelinius* Legalov, 2003. Comment: proposed as a subtribe of Euopini; incorrect original stem formation, not in prevailing usage.
Sawadaeuopsina Legalov, 2007: 241 [stem: *Sawadaeuop-*]. Type genus: *Sawadaeuops* Legalov, 2003. Comment: proposed as a subtribe of Euopini; incorrect original stem formation, not in prevailing usage.

### Tribe Pilolabini

Voss, 1925

Pilolabini Voss, 1925: 19 [stem: *Pilolab-*]. Type genus: *Pilolabus* Jekel, 1860.

### Subfamily Apoderinae

Jekel, 1860

Apoderidae Jekel, 1860: 180 [stem: *Apoder-*]. Type genus: *Apoderus* A. G. Olivier, 1807.

### Tribe Apoderini

Jekel, 1860

Apoderidae Jekel, 1860: 180 [stem: *Apoder-*]. Type genus: *Apoderus* A. G. Olivier, 1807.
Anisonychina Legalov, 2003: 555 [stem: *Anisonych-*]. Type genus: *Anisonychus* Voss, 1927. Comment: junior homonym of Anisonychidae H. C. C. Burmeister, 1844 (type genus *Anisonyx* Latreille, 1807) available in Scarabaeidae; this case is to be referred to the Commission to remove the homonymy (Art. 55.3.1).
Centrocorynina Legalov, 2003: 557 [stem: *Centrocoryn-*]. Type genus: *Centrocorynus* Jekel, 1860.
Cycnotrachelina Legalov, 2003: 568 [stem: *Cycnotrachel-*]. Type genus: *Cycnotrachelus* Jekel, 1860.
Opanassenkoviina Legalov, 2003: 554 [stem: *Opanassenkovi-*]. Type genus: *Opanassenkovius* Legalov, 2003.
Pseudocycnotrachelina Legalov, 2003: 525 [stem: *Pseudocycnotrachel-*]. Type genus: *Pseudocycnotrachelus* Legalov, 2003.

### Tribe Clitostylini

Voss, 1929

Clitostylini Voss, 1929a: 192 [stem: *Clitostyl-*]. Type genus: *Clitostylus* Voss, 1929 [syn. of *Trachelismus* Motschulsky, 1870].

### Subtribe Allapoderina

Legalov, 2003

Allapoderina Legalov, 2003: 467 [stem: *Allapoder-*]. Type genus: *Allapoderus* Voss, 1927.

### Subtribe Clitostylina

Voss, 1929

Clitostylini Voss, 1929a: 192 [stem: *Clitostyl-*]. Type genus: *Clitostylus* Voss, 1929 [syn. of *Trachelismus* Motschulsky, 1870].

### Subtribe Pseudophrysina

Legalov, 2003

Pseudophrysina Legalov, 2003: 476 [stem: *Pseudophrys-*]. Type genus: *Pseudophrysus* Legalov, 2003.

### Tribe Hoplapoderini

Voss, 1926

Hoplapoderini Voss, 1926: 14 [stem: *Hoplapoder-*]. Type genus: *Hoplapoderus* Jekel, 1860.

### Subtribe Afroapoderina

Legalov, 2003

Afroapoderina Legalov, 2003: 482 [stem: *Afroapoder-*]. Type genus: *Afroapoderus* Legalov, 2003.

### Subtribe Hoplapoderina

Voss, 1926

Hoplapoderini Voss, 1926: 14 [stem: *Hoplapoder-*]. Type genus: *Hoplapoderus* Jekel, 1860.

### Subtribe Paratomapoderina

Legalov, 2003

Paratomapoderina Legalov, 2003: 488 [stem: *Paratomapoder-*]. Type genus: *Paratomapoderus* Voss, 1926.

### Tribe Trachelophorini

Voss, 1926

Trachelophorini Voss, 1926: 14, in key [stem: *Trachelophor-*]. Type genus: *Trachelophorus* Jekel, 1860.

### Subfamily Rhynchitinae

Gistel, 1848

Rhynchitisidae Gistel, 1848: [8] [stem: *Rhynchit-*]. Type genus: *Rhynchites* Schneider, 1791.

### Tribe Auletini

Desbrochers des Loges, 1908

Auletinidae Desbrochers des Loges, 1908: 10, in key [stem: *Aulet-*]. Type genus: *Auletes* Schönherr, 1826. Comment: incorrect original stem formation, not in prevailing usage.

### Subtribe Auletina

Desbrochers des Loges, 1908

Auletinidae Desbrochers des Loges, 1908: 10, in key [stem: *Aulet-*]. Type genus: *Auletes* Schönherr, 1826. Comment: incorrect original stem formation, not in prevailing usage.
Auletini Pierce, 1913: 365, in key [stem: *Aulet-*]. Type genus: *Auletes* Schönherr, 1826. Comment: family-group name proposed as new without reference to Auletinidae Desbrochers des Loges, 1908.

### Subtribe Auletobiina

Legalov, 2001

Auletobiina Legalov, 2001: 37 [stem: *Auletobi-*]. Type genus: *Auletobius* Desbrochers des Loges, 1869.

### Subtribe Guineauletina

Legalov, 2003

Guineauletina Legalov, 2003: 108 [stem: *Guineaulet-*]. Type genus: *Guineauletes* Legalov, 2003.

### Subtribe Mandelschtamiina

Legalov, 2003

Mandelschtamiina Legalov, 2003: 105 [stem: *Mandelschtami-*]. Type genus: *Mandelschtamius* Legalov, 2003.

### Subtribe Pseudauletina

Voss, 1933

Pseudauletina Voss, 1933b: 110, in key [stem: *Pseudaulet-*]. Type genus: *Pseudauletes* Voss, 1922.

### Subtribe Pseudomesauletina

Legalov, 2003

Pseudomesauletina Legalov, 2003: 113 [stem: *Pseudomesaulet-*]. Type genus: *Pseudomesauletes* Legalov, 2001.

### Tribe Auletorhinini

Voss, 1935

Auletorhinini Voss, 1935b: 509 [stem: *Auletorhin-*]. Type genus: *Auletorhinus* Voss, 1935.

### Tribe Byctiscini

Voss, 1923

Byctiscini Voss, 1923: 510 [stem: *Byctisc-*]. Type genus: *Byctiscus* C. G. Thomson, 1859.

### Subtribe Byctiscina

Voss, 1923

Byctiscini Voss, 1923: 510 [stem: *Byctisc-*]. Type genus: *Byctiscus* C. G. Thomson, 1859.

### Subtribe Listrobyctiscina

Legalov, 2003

Listrobyctiscina Legalov, 2003: 337 [stem: *Listrobyctisc-*]. Type genus: *Listrobyctiscus* Voss, 1923.

### Subtribe Svetlanaebyctiscina

Legalov, 2003

Svetlanaebyctiscina Legalov, 2003: 323 [stem: *Svetlanaebyctisc-*]. Type genus: *Svetlanaebyctiscus* Legalov, 2001.

### Tribe Cesauletini

Legalov, 2003

Cesauletini Legalov, 2003: 134 [stem: *Cesaulet-*]. Type genus: *Cesauletes* Hamilton, 1983. Comment: proposed as a tribe of the supertribe Rhynchititae.

### Tribe Deporaini

Voss, 1929

Deporaini Voss, 1929b: 28 [stem: *Depora-*]. Type genus: *Deporaus* Samouelle, 1819.

### Subtribe Chonostropheina

Morimoto, 1962

Chonostropheina Morimoto, 1962a: 30, in key [stem: *Chonostrophe-*]. Type genus: *Chonostropheus* Prell, 1924.

### Subtribe Deporaina

Voss, 1929

Deporaini Voss, 1929b: 28 [stem: *Depora-*]. Type genus: *Deporaus* Samouelle, 1819.

### Tribe Minurini

Legalov, 2003

Minurini Legalov, 2003: 133 [stem: *Minur-*]. Type genus: *Minurus* G. R. Waterhouse, 1842. Comment: proposed as a tribe of the supertribe Rhynchititae.

### Tribe Rhinocartini

Voss, 1931

Rhinocartini Voss, 1931: 162, in key [stem: *Rhinocart-*]. Type genus: *Rhinocartus* Voss, 1922.
*Rhynchitallini Voss, 1960: 415 [stem: *Rhynchitall-*]. Type genus: *Rhynchitallus* Voss, 1960. Comment: unavailable family-group name, proposed after 1930 without description or bibliographic reference to such a description (Art. 13.1).
Proteugnamptini Legalov, 2003: 80 [stem: *Proteugnamptini*]. Type genus: *Proteugnamptus* Voss, 1939. Comment: proposed as a tribe in supertribe Rhinocartitae.
Auletanini Legalov, 2003: 84 [stem: *Auletan-*]. Type genus: *Auletanus* Voss, 1922. Comment: proposed as a tribe in the supertribe Rhinocartitae.
†Sanyrevilleina Legalov, 2003: 85 [stem: *Sanyreville-*]. Type genus: *Sanyrevilleus* Gratshev and Zherikhin, 2000. Comment: proposed as a subtribe of Auletanini; raised to tribal level by Legalov (2007).
Proteugnamptini Legalov, 2003: 80 [stem: *Proteugnampt-*]. Type genus: *Proteugnamptus* Voss, 1939. Comment: proposed as a tribe in the supertribe Rhinocartitae.
Vossicartini Legalov, 2003: 79 [stem: *Vossicart-*]. Type genus: *Vossicartus* Legalov, 2003.
Eosalacina Legalov, 2007: 30 [stem: *Eosalac-*]. Type genus: *Eosalacus* Legalov, 2007. Comment: proposed as a subtribe of Proteugnamptini.
Parauletanina Legalov, 2007: 31 [stem: *Parauletan-*]. Type genus: *Parauletanus* Legalov, 2007. Comment: proposed as a subtribe of Sanyrevilleini.

### Tribe Rhynchitini

Gistel, 1848

Rhynchitisidae Gistel, 1848: [8] [stem: *Rhynchit-*]. Type genus: *Rhynchites* Schneider, 1791. Comment: incorrect original stem formation, not in prevailing usage.

### Subtribe Acritorrhynchitina

Legalov, 2007

Acritorrhynchitina Legalov, 2007: 70 [stem: *Acritorrhynchit-*]. Type genus: *Acritorrhynchites* Voss, 1941.

### Subtribe Anisomerinina

Legalov, 2003

Anisomerinina Legalov, 2003: 223 [stem: *Anisomerin-*]. Type genus: *Anisomerinus* Voss, 1933.

### Subtribe Eugnamptina

Voss, 1930

Eugnamptina Voss, 1930: 67 [stem: *Eugnampt-*]. Type genus: *Eugnamptus* Schönherr, 1839.

### Subtribe Lasiorhynchitina

Legalov, 2003

Lasiorhynchitina Legalov, 2003: 202 [stem: *Lasiorhynchit-*]. Type genus: *Lasiorhynchites* Jekel, 1860.

### Subtribe Perrhynchitina

Legalov, 2003

Perrhynchitina Legalov, 2003: 218 [stem: *Perrhynchit-*]. Type genus: *Perrhynchites* Voss, 1953.

### Subtribe Rhynchitallina

Legalov, 2003

Rhynchitallina Legalov, 2003: 226 [stem: *Rhynchitall-*]. Type genus: *Rhynchitallus* Voss, 1960.

### Subtribe Rhynchitina

Gistel, 1848

Rhynchitisidae Gistel, 1848: [8] [stem: *Rhynchit-*]. Type genus: *Rhynchites* Schneider, 1791. Comment: incorrect original stem formation, not in prevailing usage.
Rhynchitini Pierce, 1913: 365, in key [stem: *Rhynchit-*]. Type genus: *Rhynchites* Schneider, 1791. Comment: family-group name proposed as new without reference to Rhynchitisidae Gistel, 1848.

### Subtribe Temnocerina

Legalov, 2003

Temnocerina Legalov, 2003: 207 [stem: *Temnocer-*]. Type genus: *Temnocerus* Thunberg, 1815.

### Subfamily Isotheinae

Scudder, 1893

Isotheinae Scudder, 1893: 16 [stem: *Isothe-*]. Type genus: *Isothea* Scudder, 1893. Comment: precedence (Isotheinae Scudder, 1893 vs Toxorhynchinae Scudder, 1893) given to taxon originally proposed at the higher rank (Art. 24.1).

### Tribe Isotheini

Scudder, 1893

Isotheinae Scudder, 1893: 16 [stem: *Isothe-*]. Type genus: *Isothea* Scudder, 1893.

### Subtribe Depasophilina

Legalov, 2003

Depasophilina Legalov, 2003: 166 [stem: *Depasophil*-]. Type genus: *Depasophilus* Voss, 1922.

### †Subtribe Isotheina

Scudder, 1893

Isotheinae Scudder, 1893: 16 [stem: *Isothe-*]. Type genus: *Isothea* Scudder, 1893.

### †Tribe Toxorhynchini

Scudder, 1893

Toxorhynchini Scudder, 1893: 23 [stem: *Toxorhynch-*]. Type genus: *Toxorhynchus* Scudder, 1893.

### Subfamily Pterocolinae

Lacordaire, 1865

Ptérocolides Lacordaire, 1865: 190 [stem: *Pterocol-*]. Type genus: *Pterocolus* Schönherr, 1833 [preoccupied genus name, not *Pterocolus* Say, 1831 [Coleoptera: Attelabidae: Pterocolinae]; syn. of *Pterocolus* Say, 1831]. Comment: original vernacular name available (Art. 11.7.2): first used in latinized form by Pascoe (1870b: 437, as Pterocolinae), generally accepted as in Alonso-Zarazaga and Lyal (1999: 44, as Pterocolinae); permanently invalid (Art. 39): based on preoccupied type genus; although strictly speaking the type genus is a junior homonym, and thus cannot be used as valid, to preserve stability, we are considering this as a valid name here; an application to the Commission is necessary to officially conserve usage of this family-group name.

### Family Brentidae

Billberg, 1820

Brenthides Billberg, 1820a: 40 [stem: *Brent-*]. Type genus: *Brentus* Fabricius, 1787 [as *Brenthus*, unjustified emendation of type genus name by Illiger (1801), not in prevailing usage].

### Subfamily Brentinae

Billberg, 1820

Brenthides Billberg, 1820a: 40 [stem: *Brent-*]. Type genus: *Brentus* Fabricius, 1787 [as *Brenthus*, unjustified emendation of type genus name by Illiger (1801), not in prevailing usage].

### Tribe Brentini

Billberg, 1820

Brenthides Billberg, 1820a: 40 [stem: *Brent-*]. Type genus: *Brentus* Fabricius, 1787 [as *Brenthus*, unjustified emendation of type genus name by Illiger (1801), not in prevailing usage].

### Subtribe Arrhenodina

Lacordaire, 1865

Arrhénodides Lacordaire, 1865: 425 [stem: *Arrhenod-*]. Type genus: *Arrenodes* sensu Lacordaire, 1865 [as *Arrhenodes*, incorrect subsequent spelling of type genus name, not in prevailing usage; not *Arrenodes* Schönherr 1823; syn. of *Estenorhinus* Lacordaire, 1865]. Comment: based on misidentified type genus; MAAZ and CHCL will submit an application to designate as type genus *Arrenodes* Schönherr, 1823 and correct the name of the tribe to Arrenodini; First Reviser (Arrhenodina Lacordaire, 1865 vs Belopherina Lacordaire, 1865 vs Belorhynchina Lacordaire, 1865 vs Eutrachelina Lacordaire, 1865) not determined, current usage maintained.
Bélophérides Lacordaire, 1865: 433 [stem: *Belopher-*]. Type genus: *Belopherus* Schönherr, 1833 [syn. of *Belorhynchus* Berthold, 1827]. Comment: original vernacular name available (Art. 11.7.2): first used in latinized form by Pascoe (1872: 317, as Belopherinae), generally accepted as in Sharp (1895a: 53, as Belopherina).
Bélorhynchides Lacordaire, 1865: 437 [stem: *Belorhynch-*]. Type genus: *Belorhynchus* Berthold, 1827. Comment: original vernacular name available (Art. 11.7.2): first used in latinized form generally accepted as in Sharp (1895a: 63, as Belorhynchina); placed in synonymy with Arrhenodini by Alonso-Zarazaga and Lyal (2002: 10).
Eutrachélides Lacordaire, 1865: 438 [stem: *Eutrachel-*]. Type genus: *Eutrachelus* Berthold, 1827. Comment: original vernacular name available (Art. 11.7.2): first used in latinized form by Senna (1892: 179, as Eutrachelinae), generally accepted as in Sharp (1900b: 385, as Eutrachelides [treated as Latin]).
Orychodi Senna, 1895: 213 [stem: *Orychod-*]. Type genus: *Orychodes* Pascoe, 1862.
Eupsalini Muizon, 1960: 170 [stem: *Eupsalid-*]. Type genus: *Eupsalis* sensu Kleine, 1927 [not *Eupsalis* Lacordaire, 1865; syn. of *Orfilaia* Haedo Rosii, 1955]. Comment: based on a misidentified type genus; incorrect original stem formation, not in prevailing usage.

### Subtribe Brentina

Billberg, 1820

Brenthides Billberg, 1820a: 40 [stem: *Brent-*]. Type genus: *Brentus* Fabricius, 1787 [as *Brenthus*, unjustified emendation of type genus name by Illiger (1801), not in prevailing usage].

### Subtribe Eremoxenina

Semenov, 1892

Amorphocephalides Power, 1879: 478 [stem: *Amorphocephal-*]. Type genus: *Amorphocephalus* Schönherr, 1840 [preoccupied genus name, not *Amorphocephalus* Bowdich, 1825 [Pisces]; syn. of *Amorphocephala* Damoiseau, 1966]. Comment: permanently invalid (Art. 39): based on preoccupied type genus.
Eremoxenidae Semenov, 1892: 440 [stem: *Eremoxen-*]. Type genus: *Eremoxenus* Semenov, 1892.
Paussobrenthini Gestro, 1919: 270 [stem: *Paussobrenth-*]. Type genus: *Paussobrenthus* Gestro, 1919.

### Tribe Cyladini

Schönherr, 1823

Cylades Schönherr, 1823: column 1137 [stem: *Cylad-*]. Type genus: *Cylas* Latreille, 1802.

### Tribe Cyphagogini

Kolbe, 1892

Cyphagoginae Kolbe, 1892: 162 [stem: *Cyphagog-*]. Type genus: *Cyphagogus* Parry, 1849.

### Subtribe Atopobrentina

Damoiseau, 1965

Atopobrentini Damoiseau, 1965: 1 [stem: *Atopobrent-*]. Type genus: *Atopobrentus* Damoiseau, 1965.

### Subtribe Cyphagogina

Kolbe, 1892

Cyphagoginae Kolbe, 1892: 162 [stem: *Cyphagog-*]. Type genus: *Cyphagogus* Parry, 1849.
*Calodrominen Kolbe, 1916: 50 [stem: *Calodrom-*]. Type genus: *Calodromus* Guérin-Méneville, 1832. Comment: original vernacular name unavailable (Art. 11.7.2): proposed after 1899.
Calodromini Kleine, 1922a: 148 [stem: *Calodrom-*]. Type genus: *Calodromus* Guérin-Méneville, 1832.

### †Subtribe Dominibrentina

Poinar, 2009

Dominibrentini Poinar, 2009: 51 [stem: *Dominibrent*-]. Type genus: *Dominibrentus* Poinar, 2009.

### Subtribe Hoplopisthiina

Senna and Calabresi, 1919

Hoplopisthi Senna and Calabresi, 1919: 65 [stem: *Hoplopisthi-*]. Type genus: *Hoplopisthius* Senna, 1892. Comment: incorrect original stem formation, not in prevailing usage.

### Subtribe Stereodermina

Sharp, 1895

Stereodermina Sharp, 1895a: 7 [stem: *Stereoderm-*]. Type genus: *Stereodermus* Lacordaire, 1865.

### Tribe Pholidochlamydini

Damoiseau, 1962

Pholidochlamydinae Damoiseau, 1962: 26 [stem: *Pholidochlamyd-*]. Type genus: *Pholidochlamys* Lacordaire, 1865. Comment: the original spelling Pholidochlomydinae on page 26 is considered a *lapsus calami* since the the type genus is spelled correctly throughout and the family-group name is spelled correctly on page 18.

### Tribe Taphroderini

Lacordaire, 1865

Taphrodérides Lacordaire, 1865: 406 [stem: *Taphroder-*]. Type genus: *Taphroderes* Schönherr, 1823. Comment: original vernacular name available (Art. 11.7.2): first used in latinized form by Pascoe (1872: 317, as Taphroderinae), generally accepted as in Alonso-Zarazaga and Lyal (1999: 52, as Taphroderinae).
Ischnomérides Lacordaire, 1865: 414 [stem: *Ischnomer-*]. Type genus: *Ischnomerus* Schönherr, 1840 [preoccupied genus name, not *Ischnomerus* Labram and Imhoff, 1838 [Coleoptera: Curculionidae: Trachelizini]; syn. of *Aulacoderes* Chevrolat, 1839]. Comment: original vernacular name available (Art. 11.7.2): first used in latinized form and generally accepted as in Schoenfeldt (1908: 2, as Ischnomeridae); permanently invalid (Art. 39): based on preoccupied type genus.

### Tribe Trachelizini

Lacordaire, 1865

Trachélizides Lacordaire, 1865: 417 [stem: *Tracheliz-*]. Type genus: *Trachelizus* Dejean, 1834. Comment: First Reviser (Trachelizini Lacordaire, 1865 vs Ithystenini Lacordaire, 1865) not determined, current usage maintained.

### Subtribe Acratina

Alonso-Zarazaga, Lyal, Bartolozzi and Sforzi, 1999

Némocéphalides Lacordaire, 1865: 459 [stem: *Nemocephal-*]. Type genus: *Nemocephalus* sensu Lacordaire, 1865 [not *Nemocephalus* Guérin-Méneville, 1827; syn. of *Neacratus* Alonso-Zarazaga et al., 1999]. Comment: original vernacular name available (Art. 11.7.2): first used in latinized form and generally accepted as in Kolbe (1883: 79, as Nemocephali); based on a misidentified type genus, name treated here as invalid until an application is submitted to the Commission to suppress it for the Principle of Priority (Art. 65.2.1).
Acratini Alonso-Zarazaga et al., 1999: 53 [stem: *Acrat-*]. Type genus: *Acratus* Lacordaire, 1865.

### Subtribe Ithystenina

Lacordaire, 1865

Ithysténides Lacordaire, 1865: 464 [stem: *Ithysten-*]. Type genus: *Ithystenus* Pascoe, 1862. Comment: original vernacular name available (Art. 11.7.2): first used in latinized form by Pascoe (1872: 317, as Ithysteninae), generally accepted as in Alonso-Zarazaga and Lyal (1999: 54, as Ithystenini).
Ischnomeri Kolbe, 1883: 74 [stem: *Ischnomer-*]. Type genus: *Ischnomerus* Labram and Imhoff, 1838.
Leptorrhynchidae Schoenfeldt, 1908: 69 [stem: *Leptorhynch-*]. Type genus: *Leptorhynchus* Guérin-Méneville, 1838 [as *Leptorrhynchus*, incorrect subsequent spelling of type genus name, not in prevailing usage; preoccupied genus name, not *Leptorhynchus* Clift, 1828 [Reptilia]; syn. of *Ithystenus* Pascoe, 1862]. Comment: permanently invalid (Art. 39): based on preoccupied type genus; incorrect original stem formation, not in prevailing usage.
Ozodocerini Jakobson, 1911b: 142 [stem: *Ozodecer-*]. Type genus: *Ozodecerus* Chevrolat, 1839. Comment: incorrect original stem formation, not in prevailing usage.

### Subtribe Microtrachelizina

Zimmerman, 1994

Microtrachelizina Zimmerman, 1994b: 182 [stem: *Microtracheliz-*]. Type genus: *Microtrachelizus* Senna, 1893.

### Subtribe Pseudoceocephalina

Kleine, 1922

Céocéphalides Lacordaire, 1865: 444 [stem: *Ceocephal-*]. Type genus: *Ceocephalus* sensu Lacordaire, 1865 [not *Ceocephalus* Guérin-Méneville, 1833; *Ceocephalus* sensu Lacordaire, 1865 is a mixture of *Orphanobrentus* Damoiseau, 1962 and *Pseudoceocephalus* Kleine, 1920 (see Alonso-Zarazaga and Lyal 1999: 55)]. Comment: original vernacular name available (Art. 11.7.2): first used in latinized form and generally accepted as in Sharp (1900b: 385, as Ceocephalides [treated as Latin]); based on a misidentified type genus, name treated here as invalid until an application is submitted to the Commission to suppress it for the Principle of Priority (Art. 65.2.1).
Uropterini Jakobson, 1911b: 142 [stem: *Uropter-*]. Type genus: *Uroptera* Berthold, 1827.
Pseudoceocephalidae Kleine, 1922b: 225 [stem: *Pseudoceocephal-*]. Type genus: *Pseudoceocephalus* Kleine, 1920. Comment: this family-group name is preceeded by two older names: Ceocephalina Lacordaire, 1865, which is based on a misidentified type genus, and the newly discovered name Uropterina Jakobson, 1911; we recommend that an application be submitted to the Commission to conserve usage of the well-established name Pseudoceocephalina Kleine, 1922.

### Subtribe Rhyticephalina

Kleine, 1922

Rhyticephalini Kleine, 1922c: 163 [stem: *Rhyticephal-*]. Type genus: *Rhyticephalus* Chevrolat, 1839.

### Subtribe Trachelizina

Lacordaire, 1865

Trachélizides Lacordaire, 1865: 417 [stem: *Tracheliz-*]. Type genus: *Trachelizus* Dejean, 1834. Comment: original vernacular name available (Art. 11.7.2): first used in latinized form by Pascoe (1872: 317, as Trachelizinae), generally accepted as in Alonso-Zarazaga and Lyal (1999: 53, as Trachelizini); First Reviser (Trachelizina Lacordaire, 1865 vs Hephebocerina Lacordaire, 1865) not determined, current usage maintained.
Héphébocérides Lacordaire, 1865: 415 [stem: *Hephebocer-*]. Type genus: *Hephebocerus* Schönherr, 1840. Comment: original vernacular name available (Art. 11.7.2): first used in latinized form and generally accepted as in Pascoe (1872: 317, as Ephebocerinae [incorrect stem formation]).
Anchistenini Schedl, 1961: 198 [stem: *Anchiste-*]. Type genus: *Anchisteus* Kolbe, 1883. Comment: incorrect original stem formation, not in prevailing usage.

### Subtribe Tychaeina

Schoenfeldt, 1908

Tychaeidae Schoenfeldt, 1908: 48 [stem: *Tychae-*]. Type genus: *Tychaeus* Fischer von Waldheim, 1823 [syn. of *Nemorhinus* Schönherr, 1823].

### Tribe Ulocerini

Schönherr, 1823

Ulocerides Schönherr, 1823: column 1137 [stem: *Ulocer-*]. Type genus: *Ulocerus* Schönherr, 1823.

### Subfamily Eurhynchinae

Lacordaire, 1863

Eurhynchides Lacordaire, 1863: 527 [stem: *Eurhynch-*]. Type genus: *Eurhynchus* Kirby, 1828 [placed on the Official List of Generic Names in Zoology (ICZN 1985e)].

### †Tribe Axelrodiellini

Legalov, 2009

Axeirodiellini Legalov, 2009c: 292 [stem: *Axelrodiell-*]. Type genus: *Axelrodiellus* Zherikhin and Gratshev, 2004. Comment: incorrect original stem formation, not in prevailing usage.

### Tribe Eurhynchini

Lacordaire, 1863

Eurhynchides Lacordaire, 1863: 527 [stem: *Eurhynch-*]. Type genus: *Eurhynchus* Kirby, 1828 [placed on the Official List of Generic Names in Zoology (ICZN 1985e)]. Comment: original vernacular name available (Art. 11.7.2): first used in latinized form by Pascoe (1870b: 436, as Eurhynchinae), generally accepted as in Alonso-Zarazaga and Lyal (1999: 56, as Eurhynchidae); Eurhynchinae Lacordaire, 1863 placed on the Official List of Family-Group Names in Zoology (ICZN 1985e).
Eurhinini Kissinger, 1968: 10 [stem: *Eurhin-*]. Type genus: *Eurhinus* Kirby, 1819 [preoccupied genus name, not *Eurhinus* Illiger, 1807 [Coleoptera: Curculionidae: Baridini]; syn. of *Eurhynchus* Kirby, 1828]. Comment: replacement name for Eurhynchides Lacordaire, 1863 because of the homonymy of the type genus; junior homonym of Eurhinina Lacordaire, 1865 [Curculionidae: Baridinae]; permanently invalid (Art. 39): based on preoccupied type genus.

### Subfamily Apioninae

Schönherr, 1823

Apionides Schönherr, 1823: column 1136 [stem: *Apion-*]. Type genus: *Apion* Herbst, 1797. Comment: First Reviser (Apioninae Schönherr, 1823 vs Antliarhininae Schönherr, 1823) not determined, current usage maintained.

### Supertribe Apionitae

Schönherr, 1823

Apionides Schönherr, 1823: column 1136 [stem: *Apion-*]. Type genus: *Apion* Herbst, 1797.

### Tribe Apionini

Schönherr, 1823

Apionides Schönherr, 1823: column 1136 [stem: *Apion-*]. Type genus: *Apion* Herbst, 1797.

### Subtribe Apionina

Schönherr, 1823

Apionides Schönherr, 1823: column 1136 [stem: *Apion-*]. Type genus: *Apion* Herbst, 1797. Comment: current spelling maintained (Art. 29.5): incorrect stem formation in prevailing usage (should be *Api*-).
Oxeostomatidae Gistel, 1856a: 374 [stem: *Oxeostom-*]. Type genus: *Oxeostomum* Gistel, 1856 [syn. of *Apion* Herbst, 1797]. Comment: incorrect original stem formation, not in prevailing usage.

### Subtribe Aplemonina

Kissinger, 1968

Aplemonini Kissinger, 1968: 16 [stem: *Aplemon-*]. Type genus: *Aplemonus* Schönherr, 1847.

### Subtribe Aspidapiina

Alonso-Zarazaga, 1990

Aspidapiini Alonso-Zarazaga, 1990: 52 [stem: *Aspidapi-*]. Type genus: *Aspidapion* Schilsky, 1901.

### Subtribe Catapiina

Alonso-Zarazaga, 1990

Catapiina Alonso-Zarazaga, 1990: 114 [stem: *Catapi-*]. Type genus: *Catapion* Schilsky, 1906.

### Subtribe Ceratapiina

Alonso-Zarazaga, 1990

Ceratapiini Alonso-Zarazaga, 1990: 42 [stem: *Ceratapi-*]. Type genus: *Ceratapion* Schilsky, 1901.

### Subtribe Exapiina

Alonso-Zarazaga, 1990

Exapiini Alonso-Zarazaga, 1990: 75 [stem: *Exapi-*]. Type genus: *Exapion* Bedel, 1887.

### Subtribe Ixapiina

Alonso-Zarazaga, 1990

Ixapiini Alonso-Zarazaga, 1990: 71 [stem: *Ixapi-*]. Type genus: *Ixapion* Roudier and Tempère, 1973.

### Subtribe Kalcapiina

Alonso-Zarazaga, 1990

Kalcapiini Alonso-Zarazaga, 1990: 55 [stem: *Kalcapi-*]. Type genus: *Kalcapion* Schilsky, 1906.

### Subtribe Malvapiina

Alonso-Zarazaga, 1990

Malvapiini Alonso-Zarazaga, 1990: 65 [stem: *Malvapi-*]. Type genus: *Malvapion* Hoffmann, 1958.

### Subtribe Metapiina

Alonso-Zarazaga, 1990

Metapiini Alonso-Zarazaga, 1990: 62 [stem: *Metapi-*]. Type genus: *Metapion* Schilsky, 1906.

### Subtribe Oxystomatina

Alonso-Zarazaga, 1990

Oxystomatini Alonso-Zarazaga, 1990: 111 [stem: *Oxystomat-*]. Type genus: *Oxystoma* Duméril, 1805.

### Subtribe Piezotrachelina

Voss, 1959

Piezotrachelini Voss, 1959: 51 [stem: *Piezotrachel-*]. Type genus: *Piezotrachelus* Schönherr, 1839.

### Subtribe Prototrichapiina

Wanat, 1995

Prototrichapiini Wanat, 1995: 19 [stem: *Prototrichapi-*]. Type genus: *Prototrichapion* Voss, 1959.

### Subtribe Synapiina

Alonso-Zarazaga, 1990

Synapiina Alonso-Zarazaga, 1990: 118 [stem: *Synapi-*]. Type genus: *Synapion* Schilsky, 1902.

### Subtribe Trichapiina

Alonso-Zarazaga, 1990

Trichapiina Alonso-Zarazaga, 1990: 116 [stem: *Trichapi-*]. Type genus: *Trichapion* Wagner, 1912.

### Tribe Chilapiini

Wanat, 2001

Chilapiitae Wanat, 2001: 366 [stem: *Chilapi-*]. Type genus: *Chilapion* Kissinger, 1968.

### Tribe Noterapiini

Kissinger, 2004

Noterapionini Kissinger, 2004: 243 [stem: *Noterapi-*]. Type genus: *Noterapion* Kissinger, 2002. Comment: incorrect original stem formation, not in prevailing usage.

### Tribe Podapiini

Wanat, 2001

Podapiitae Wanat, 2001: 366 [stem: *Podapi-*]. Type genus: *Podapion* Riley, 1883.

### Tribe Rhinorhynchidiini

Zimmerman, 1994

Rhinorhynchidiinae Zimmerman, 1994b: 337 [stem: *Rhinorhynchidi-*]. Type genus: *Rhinorhynchidius* Voss, 1922.

### Supertribe Antliarhinitae

Schönherr, 1823

Antliarhinides Schönherr, 1823: column 1137 [stem: *Antliarhin-*]. Type genus: *Antliarhis* Billberg, 1820 [as *Antliarhinus*, incorrect subsequent spelling of type genus name, not in prevailing usage].

### Supertribe Cybebitae

Lacordaire, 1863

Cybébides Lacordaire, 1863: 539 [stem: *Cybeb-*]. Type genus: *Cybebus* Schönherr, 1839. Comment: original vernacular name available (Art. 11.7.2): first used in latinized form by Pascoe (1870b: 436, as Cybebinae), generally accepted as in Alonso-Zarazaga and Lyal (1999: 58, as Cybebini).

### Supertribe Mecolenitae

Wanat, 2001

Mecoleninae Wanat, 2001: 363 [stem: *Mecolen-*]. Type genus: *Mecolenus* Schönherr, 1847.

### Supertribe Myrmacicelitae

Zimmerman, 1994

Myrmacicelinae Zimmerman, 1994b: 349 [stem: *Myrmacicel-*]. Type genus: *Myrmacicelus* Chevrolat, 1833.

### Tribe Lispotheriini

Wanat, 2001

Lispotheriini Wanat, 2001: 365 [stem: *Lispotheri-*]. Type genus: *Lispotherium* Faust, 1891.

### Tribe Myrmacicelini

Zimmerman, 1994

Myrmacicelinae Zimmerman, 1994b: 349 [stem: *Myrmacicel-*]. Type genus: *Myrmacicelus* Chevrolat, 1833.

### Supertribe Rhadinocybitae

Alonso-Zarazaga, 1992

Rhadinocybini Alonso-Zarazaga, 1992: 193 [stem: *Rhadinocyb-*]. Type genus: *Rhadinocyba* Faust, 1889.

### Tribe Notapionini

Zimmerman, 1994

Notapionini Zimmerman, 1994b: 317 [stem: *Notapion-*]. Type genus: *Notapion* Zimmerman, 1994. Comment: current spelling maintained (Art. 29.5): incorrect stem formation in prevailing usage (should be *Notapi*-).

### Tribe Rhadinocybini

Alonso-Zarazaga, 1992

Rhadinocybini Alonso-Zarazaga, 1992: 193 [stem: *Rhadinocyb-*]. Type genus: *Rhadinocyba* Faust, 1889.

### Supertribe Tanaitae

Schönherr, 1839

Tanaonides Schönherr, 1839: 447 [stem: *Tana-*]. Type genus: *Tanaos* Schönherr, 1826. Comment: incorrect original stem formation, not in prevailing usage.

### Subfamily Ithycerinae

Schönherr, 1823

Ithycerides Schönherr, 1823: column 1136 [stem: *Ithycer-*]. Type genus: *Ithycerus* Schönherr, 1823.
Pachyrhinchidae Kirby, 1837: 203 [stem: *Pachyrhynch-*]. Type genus: *Pachyrhynchus* Kirby, 1837 [preoccupied genus name, not *Pachyrhynchus* Germar, 1824 [Coleoptera: Curculionidae: Otiorhynchini]; syn. of *Ithycerus* Schönherr, 1823]. Comment: permanently invalid (Art. 39): based on preoccupied type genus; incorrect original stem formation, not in prevailing usage.
†Gobicarini Legalov, 2009c: 291 [stem: *Gobicar-*]. Type genus: *Gobicar* Gratshev and Zherikhin, 1999.

### Subfamily Microcerinae

Lacordaire, 1863

Microcérides Lacordaire, 1863: 20 [stem: *Microcer-*]. Type genus: *Microcerus* Schönherr, 1833. Comment: original vernacular name available (Art. 11.7.2): first used in latinized form by Pascoe (1870b: 436, as Microcerinae), generally accepted as in Alonso-Zarazaga and Lyal (1999: 63, as Microcerinae).
*Épisides Lacordaire, 1863: 22 [stem: *Epis-*]. Type genus: *Episus* Billberg, 1820. Comment: original vernacular name unavailable (Art. 11.7.2): subsequently used in latinized form, e.g., Heyne and Taschenberg (1907: 224, as Episini), but not generally accepted as valid.

### Subfamily Nanophyinae

Gistel, 1848

Nanophyeidae Gistel, 1848: [7] [stem: *Nanophy-*]. Type genus: *Nanophyes* Schönherr, 1837 [placed on the Official List of Generic Names in Zoology (ICZN 1989b)].

### Tribe Corimaliini

Alonso-Zarazaga, 1989

Corimaliini Alonso-Zarazaga, 1989: 225 [stem: *Corimali-*]. Type genus: *Corimalia* Gozis, 1885.

### Tribe Nanophyini

Gistel, 1848

Nanophyeidae Gistel, 1848: [7] [stem: *Nanophy-*]. Type genus: *Nanophyes* Schönherr, 1837 [placed on the Official List of Generic Names in Zoology (ICZN 1989b)]. Comment: incorrect original stem formation, not in prevailing usage.

### Family Dryophthoridae

Schönherr, 1825

Dryophthorides Schönherr, 1825: column 588 [stem: *Dryophthor-*]. Type genus: *Dryophthorus* Germar, 1824 [placed on the Official List of Generic Names in Zoology (ICZN 1987c)].

### Subfamily Dryophthorinae

Schönherr, 1825

Dryophthorides Schönherr, 1825: column 588 [stem: *Dryophthor-*]. Type genus: *Dryophthorus* Germar, 1824 [placed on the Official List of Generic Names in Zoology (ICZN 1987c)].

### Subfamily Cryptodermatinae

Bovie, 1908

Oxyrhynchides Schönherr, 1823: column 1137 [stem: *Oxyrhynch-*]. Type genus: *Oxyrhynchus* Schönherr, 1823 [preoccupied genus name, not *Oxyrhynchus* Leach, 1818 [Pisces]; syn. of *Cryptoderma* Ritsema, 1885]. Comment: permanently invalid (Art. 39): based on preoccupied type genus.
Cryptoderminae Bovie, 1908: 1 [stem: *Cryptodermat-*]. Type genus: *Cryptoderma* Ritsema, 1885. Comment: replacement name for Oxyrhynchinae Schönherr, 1823 because of the homonymy of the type genus; incorrect original stem formation, not in prevailing usage.

### Subfamily Orthognathinae

Lacordaire, 1865

Orthognathides Lacordaire, 1865: 311 [stem: *Orthognath-*]. Type genus: *Orthognathus* Schönherr, 1837.

### Tribe Orthognathini

Lacordaire, 1865

Orthognathides Lacordaire, 1865: 311 [stem: *Orthognath-*]. Type genus: *Orthognathus* Schönherr, 1837. Comment: original vernacular name available (Art. 11.7.2): first used in latinized form by Heyne and Taschenberg (1907: 233, as Orthognathini), generally accepted as in Alonso-Zarazaga and Lyal (1999: 64, as Orthognathini).
Sipalides Lacordaire, 1865: 310 [stem: *Sipal-*]. Type genus: *Sipalus* Schönherr, 1825 [preoccupied genus name, not *Sipalus* Fischer, 1813 [Mammalia]; syn. of *Sipalinus* G. A. K. Marshall, 1943]. Comment: original vernacular name available (Art. 11.7.2): first used in latinized form by Pascoe (1870b: 437, as Sipalinae), generally accepted as in Heyne and Taschenberg (1907: 233, as Sipalini); permanently invalid (Art. 39): based on preoccupied type genus.
*Sipalininae G. A. K. Marshall, 1953: 117 [stem: *Sipalin-*]. Type genus: *Sipalinus* G. A. K. Marshall, 1943. Comment: unavailable family-group name, proposed after 1930 without description or bibliographic reference to such a description (Art. 13.1).
Sipalininae Zimmerman, 1993: 47, in key [stem: *Sipalin-*]. Type genus: *Sipalinus* G. A. K. Marshall, 1943.

### Tribe Rhinostomini

LeConte, 1874

Rhinidae J. L. LeConte, 1874b: 466 [stem: *Rhin-*]. Type genus: *Rhina* Latreille, 1806 [preoccupied genus name, not *Rhina* Schneider, 1801 [Pisces]; syn. of *Rhinostomus* Rafinesque, 1815]. Comment: permanently invalid (Art. 39): based on preoccupied type genus.
Rhinostomini Kuschel, 1995: 24, in key [stem: *Rhinostom-*]. Type genus: *Rhinostomus* Rafinesque, 1815 [placed on the Official List of Generic Names in Zoology (ICZN 1955a)].

### Subfamily Rhynchophorinae

Schönherr, 1833

Rhynchophorides Schönherr, 1833: 26 [stem: *Rhynchophor-*]. Type genus: *Rhynchophorus* Herbst, 1795.

### Tribe Diocalandrini

Zimmerman, 1993

Diocalandrini Zimmerman, 1993: 99 [stem: *Diocalandr-*]. Type genus: *Diocalandra* Faust, 1894.

### Tribe Litosomini

Lacordaire, 1865

Calandrina C. G. Thomson, 1859: 145 [stem: *Calandr-*]. Type genus: *Calandra* Gistel, 1848 [placed on the Official Index of Invalid and Rejected Generic Names in Zoology (ICZN 1959b); preoccupied genus name, not *Calandra* Clairville, 1798 [Coleoptera: Curculionidae: Rhynchophorinae: Sphenophorini]; syn. of *Sitophilus* Schönherr, 1837]. Comment: permanently invalid (Art. 39): based on suppressed type genus.
Litosomides Lacordaire, 1865: 303 [stem: *Litosom-*]. Type genus: *Litosomus* Lacordaire, 1865 [syn. of *Toxorhinus* Lacordaire, 1865]. Comment: original vernacular name available (Art. 11.7.2): first used in latinized form by Faust (1895: 224, as Litosominorum), generally accepted as in Alonso-Zarazaga and Lyal (1999: 65, as Litosomini).
Sitophili Csiki, 1936: 68 [stem: *Sitophil-*]. Type genus: *Sitophilus* Schönherr, 1837 [placed on the Official List of Generic Names in Zoology (ICZN 1959b)]. Comment: name proposed to replace Calandrina C. G. Thomson, 1865 because of the synonymy of the type genus (*Calandra* auctorum is a synonym of *Sitophilus* Schönherr, 1837).

### Tribe Ommatolampini

Lacordaire, 1865

Ommatolampides Lacordaire, 1865: 276 [stem: *Ommatolamp-*]. Type genus: *Ommatolampes* Schönherr, 1837. Comment: original vernacular name available (Art. 11.7.2): first used in latinized form by Heyne and Taschenberg (1907: 233, as Ommatolampini), generally accepted as in Alonso-Zarazaga and Lyal (1999: 66, as Ommatolampini).

### Tribe Polytini

Zimmerman, 1993

Polytini Zimmerman, 1993: 94 [stem: *Polyt-*]. Type genus: *Polytus* Faust, 1894.

### Tribe Rhynchophorini

Schönherr, 1833

Rhynchophorides Schönherr, 1833: 26 [stem: *Rhynchophor-*]. Type genus: *Rhynchophorus* Herbst, 1795.

### Tribe Sphenophorini

Lacordaire, 1865

Calandraedes Billberg, 1820a: 40 [stem: *Calandr-*]. Type genus: *Calandra* Clairville, 1798 [placed on the Official Index of Invalid and Rejected Generic Names in Zoology (ICZN 1959b)]. Comment: permanently invalid (Art. 39): based on suppressed type genus; several family-group names based on *Calandra* Clairville, 1798 were placed on the Official Index of Rejected and Invalid Family-Group Names in Zoology (ICZN 1959b).
Sphénophorides Lacordaire, 1865: 286 [stem: *Sphenophor-*]. Type genus: *Sphenophorus* Schönherr, 1837 [placed on the Official List of Generic Names in Zoology (ICZN 1959b)]. Comment: original vernacular name available (Art. 11.7.2): first used in latinized form by J. L. LeConte (1876: 330, as Sphenophorini), generally accepted as in Alonso-Zarazaga and Lyal (1999: 66, as Sphenophorini); First Reviser (Sphenophorini Lacordaire, 1865 vs Sphenocorynini Lacordaire, 1865) not determined, current usage maintained.
Sphénocorynides Lacordaire, 1865: 279 [stem: *Sphenocoryn-*]. Type genus: *Sphenocorynes* Schönherr, 1837. Comment: original vernacular name available (Art. 11.7.2): first used in latinized form by Aurivillius (1886: 97, as Sphenocoryninae), generally accepted as in Csiki (1936: 20, as Sphenocoryni).
Oxyopisthinae Kolbe, 1899: 5 [stem: *Oxyopisth-*]. Type genus: *Oxyopisthen* sensu Lacordaire, 1865 [not *Oxyopisthen* J. Thomson, 1858; syn. of *Korotyaevius* Alonso-Zarazaga and Lyal, 1999]. Comment: based on misidentified type genus.

### Subfamily Stromboscerinae

Lacordaire, 1865

Stromboscérides Lacordaire, 1865: 306 [stem: *Stromboscer-*]. Type genus: *Stromboscerus* Schönherr, 1837. Comment: original vernacular name available (Art. 11.7.2): first used in latinized form by Pascoe (1870b: 437, as Strombocerinae [incorrect stem formation]), generally accepted as in Alonso-Zarazaga and Lyal (1999: 68, as Stromboscerinae).

### Family Brachyceridae

Billberg, 1820

Brachycerides Billberg, 1820a: 39 [stem: *Brachycer-*]. Type genus: *Brachycerus* A. G. Olivier, 1789.

### Subfamily Brachycerinae

Billberg, 1820

Brachycerides Billberg, 1820a: 39 [stem: *Brachycer-*]. Type genus: *Brachycerus* A. G. Olivier, 1789. Comment: the tribal classification used here follows Oberprieler (2010).

### Tribe Brachycerini

Billberg, 1820

Brachycerides Billberg, 1820a: 39 [stem: *Brachycer-*]. Type genus: *Brachycerus* A. G. Olivier, 1789.
Protomantiinae Aurivillius, 1886: 21, in key [stem: *Protomanti-*]. Type genus: *Protomantis* Schönherr, 1840.

### Tribe Byrsopini

Germar, 1829

Cryptopsides Schönherr, 1826: 65 [stem: *Cryptop-*]. Type genus: *Cryptops* Schönherr, 1823 [preoccupied genus name, not *Cryptops* Leach, 1814 [Chilopoda]; syn. of *Byrsops* Germar, 1829]. Comment: permanently invalid (Art. 39): based on preoccupied type genus.
Byrsoptides Germar, 1829: 358 [stem: *Byrsop-*]. Type genus: *Byrsops* Germar, 1829. Comment: incorrect original stem formation, not in prevailing usage.
Byrsopsides Schönherr, 1833: 14 [stem: *Byrsop-*]. Type genus: *Byrsops* Schönherr, 1833 [preoccupied genus name, not *Byrsops* Germar, 1829 [Coleoptera: Curculionidae: Byrsopini]; syn. of *Byrsops* Germar, 1829]. Comment: permanently invalid (Art. 39): based on preoccupied type genus.
Brotheinae G. A. K. Marshall, 1907: 89 [stem: *Brothe-*]. Type genus: *Brotheus* Stephens, 1829. Comment: junior homonym of Brotheini Simon, 1879 in Scorpiones (type genus *Brotheas* C. L. Koch, 1837); this case is to be referred to the Commission to remove the homonymy (Art. 55.3.1).
Brotheusini Alonso-Zarazaga and Lyal, 2006: 24 [stem: *Brotheus-*]. Type genus: *Brotheus* Stephens, 1829. Comment: unnecessary replacement name for Brotheinae Marshall, 1907 because of the homonymy of the family-group name; Brotheinae/-ini/-ina Simon, 1879 (type genus *Brotheas* C. L. Koch, 1837) has been used as valid in Scorpiones in recent literature, e.g., Prendini and W. C. Wheeler (2005); the stem used by Alonso-Zarazaga and Lyal (2006) was chosen in accordance with Art. 29.6.

### Subfamily Cryptolarynginae

Schalkwyk, 1966

Cryptopharynginae G. A. K. Marshall, 1957: 18 [stem: *Cryptopharyng-*]. Type genus: *Cryptopharynx* G. A. K. Marshall, 1957 [preoccupied genus name, not *Cryptopharynx* Kahl, 1928 [Protozoa]; syn. of *Cryptolarynx* Schalkwyk, 1966]. Comment: permanently invalid (Art. 39): based on preoccupied type genus.
Cryptolarynginae Schalkwyk, 1966: 745 [stem: *Cryptolaryng-*]. Type genus: *Cryptolarynx* Schalkwyk, 1966. Comment: replacement name for Cryptopharynginae G. A. K. Marshall, 1957 because of the homonymy of the type genus.
Periegini Legalov, 2003: 68 [stem: *Perieg-*]. Type genus: *Perieges* Schönherr, 1842.

### Subfamily Erirhininae

Schönherr, 1825

Erirhinides Schönherr, 1825: column 582 [stem: *Erirhin-*]. Type genus: *Erirhinus* Schönherr, 1825 [syn. of *Notaris* Germar, 1817].

### Tribe Aonychini

Zimmerman, 1993

Aonychini Zimmerman, 1993: 136, in key [stem: *Aonych-*]. Type genus: *Aonychus* Schönherr, 1844. Comment: junior homonym of Aonychini Davis, 1978 in Mammalia (type genus *Aonyx* Lesson, 1827); this case is to be referred to the Commission to remove the homonymy (Art. 55.3.1).
Aonychusini Zimmerman, 1999: 72 [stem: *Aonychus-*]. Type genus: *Aonychus* Schönherr, 1844. Comment: unnecessary replacement name for Aonychini Zimmerman, 1993 because of the homonymy of the family-group name; the stem used by Zimmerman (1999) was chosen in accordance with Art. 29.6.

### Tribe Arthrostenini

Reitter, 1913

Arthrostenini Reitter, 1913b: 57, in key [stem: *Arthrosten-*]. Type genus: *Arthrostenus* Schönherr, 1826.

### †Tribe Cretuliini

Legalov, 2009

Cretuliini Legalov, 2009c: 293 [stem: *Cretuli-*]. Type genus: *Cretulio* Zherikhin, 1993.

### Tribe Erirhinini

Schönherr, 1825

Erirhinides Schönherr, 1825: column 582 [stem: *Erirhin-*]. Type genus: *Erirhinus* Schönherr, 1825 [syn. of *Notaris* Germar, 1817].
Rhynchaenides Latreille, 1828: 599 [stem: *Rhynchaen-*]. Type genus: *Rhynchaenus* sensu Latreille, 1828 [not *Rhynchaenus* Clairville, 1798; syn. of *Notaris* Germar, 1817]. Comment: based on a misidentified type genus; an application will need to be submitted to the Commission to suppress this name for the Principles of Priority and Homonymy (Art. 65.2.1) if Rhyncahenidae Blanchard, 1853 in Curculioninae: Rhamphini: Rhamphina is to be used as valid in the future.
Notarini Zumpt, 1929: 216 [stem: *Notar-*]. Type genus: *Notaris* sensu Tournier, 1874 [not *Notaris* Germar, 1817; syn. of *Tournotaris* Alonso-Zarazaga and Lyal, 1999]. Comment: based on a misidentified type genus.
Notodermina Voss, 1952: 196, in note [stem: *Notoderm-*]. Type genus: *Notodermus* Desbrochers des Loges, 1875 [syn. of *Procas* Stephens, 1831].

### Tribe Himasthlophallini

Zherikhin, 1991

Himasthlophallini Zherikhin, 1991: 31 [stem: *Himasthlophall-*]. Type genus: *Himasthlophallus* Egorov and Zherikhin, 1991.

### Tribe Stenopelmini

LeConte, 1876

Stenopelmi J. L. LeConte, 1876: 179 [stem: *Stenopelm-*]. Type genus: *Stenopelmus* Schönherr, 1835.
Lissorhoptrinae Böving and Craighead, 1931: 67, in key [stem: *Lissorhoptr-*]. Type genus: *Lissorhoptrus* J. L. LeConte, 1876.

### Tribe Tadiini

Zimmerman, 1993

Tadiini Zimmerman, 1993: 136, in key [stem: *Tadi-*]. Type genus: *Tadius* Pascoe, 1885.

### Tribe Tanysphyrini

Gistel, 1848

Tanysphyridae Gistel, 1848: [8] [stem: *Tanysphyr-*]. Type genus: *Tanysphyrus* Germar, 1817.
Brachypi J. L. LeConte, 1876: 180 [stem: *Brachypod-*]. Type genus: *Brachypus* Schönherr, 1826 [preoccupied genus name, not *Brachypus* Meyer, 1814 [Aves]; syn. of *Brachygyius* G. A. K. Marshall, 1939]. Comment: permanently invalid (Art. 39): based on preoccupied type genus; incorrect original stem formation, not in prevailing usage.

### Subfamily Ocladiinae

Lacordaire, 1865

Ocladiides Lacordaire, 1865: 79 [stem: *Ocladi-*]. Type genus: *Ocladius* Schönherr, 1825.

### Tribe Desmidophorini

Morimoto, 1962

Desmidophorinae Morimoto, 1962a: 32, in key [stem: *Desmidophor-*]. Type genus: *Desmidophorus* Dejean, 1835.

### Tribe Ocladiini

Lacordaire, 1865

Ocladiides Lacordaire, 1865: 79 [stem: *Ocladi-*]. Type genus: *Ocladius* Schönherr, 1825. Comment: original vernacular name available (Art. 11.7.2): first used in latinized form by Hustache (1925: 9, as Ocladiini), generally accepted as in Alonso-Zarazaga and Lyal (1999: 63, as Ocladiini).

### Subfamily Raymondionyminae

Reitter, 1913

Raymondionymini Reitter, 1913b: 58, in key [stem: *Raymondionym-*]. Type genus: *Raymondionymus* Wollaston, 1873.

### Tribe Myrtonymini

Kuschel, 1990

Myrtonymina Kuschel, 1990: 80 [stem: *Myrtonym-*]. Type genus: *Myrtonymus* Kuschel, 1990.

### Tribe Raymondionymini

Reitter, 1913

Raymondionymini Reitter, 1913b: 58, in key [stem: *Raymondionym-*]. Type genus: *Raymondionymus* Wollaston, 1873.

### Family Curculionidae

Latreille, 1802

Curculionites Latreille, 1802: 195 [stem: *Curculion-*]. Type genus: *Curculio* Linnaeus, 1758.

### Subfamily Curculioninae

Latreille, 1802

Curculionites Latreille, 1802: 195 [stem: *Curculion-*]. Type genus: *Curculio* Linnaeus, 1758.

### Tribe Acalyptini

Thomson, 1859

Acalyptina C. G. Thomson, 1859: 143 [stem: *Acalypt-*]. Type genus: *Acalyptus* Schönherr, 1833.

### Subtribe Acalyptina

Thomson, 1859

Acalyptina C. G. Thomson, 1859: 143 [stem: *Acalypt-*]. Type genus: *Acalyptus* Schönherr, 1833.

### Subtribe Derelomina

Lacordaire, 1865

Dérélomides Lacordaire, 1865: 9 [stem: *Derelom-*]. Type genus: *Derelomus* Schönherr, 1825. Comment: original vernacular name available (Art. 11.7.2): first used in latinized form by Marseul (1866: 109, as Derelomidae), generally accepted as in Alonso-Zarazaga and Lyal (1999: 77, as Derelomini).
Hoplorrhinina Champion, 1903: 277 [stem: *Hoplorhin-*]. Type genus: *Hoplorhinus* Chevrolat, 1878 [as *Hoplorrhinus*, incorrect subsequent spelling of type genus name, not in prevailing usage; syn. of *Celetes* Schönherr, 1836]. Comment: incorrect original stem formation, not in prevailing usage.
Celetini Voss, 1954: 344 [stem: *Celet-*]. Type genus: *Celetes* Schönherr, 1836. Comment: description by indication (distinguishing characters given in Champion (1903: 277, as Hoplorrhinina)); name proposed to replace Hoplorhinini Champion, 1903 because of synonymy of the type genus; Celetini is not in prevailing usage (Art. 40.2.1).

### Subtribe Notolomina

Franz, 2006

Notolomina N. M. Franz, 2006: 276 [stem: *Notolom-*]. Type genus: *Notolomus* J. L. LeConte, 1876.

### Subtribe Phyllotrogina

Franz, 2006

Phyllotrogina N. M. Franz, 2006: 276 [stem: *Phyllotrog-*]. Type genus: *Phyllotrox* Schönherr, 1843.

### Subtribe Staminodeina

Franz, 2006

Staminodeina N. M. Franz, 2006: 277 [stem: *Staminode-*]. Type genus: *Staminodeus* Franz, 2001.

### Tribe Acentrusini

Alonso-Zarazaga, 2005

Acentrina Seidlitz, 1890: 608 [stem: *Acentr-*]. Type genus: *Acentrus* Schönherr, 1845 [preoccupied genus name, not *Acentrus* Duponchel, 1839 [Coleoptera: Curculionidae: Curculioninae: Acentrusini]; syn. of *Acentrus* Duponchel, 1839]. Comment: permanently invalid (Art. 39): based on preoccupied type genus.
Acentrusini Alonso-Zarazaga, 2005: 92 [stem: *Acentrus-*]. Type genus: *Acentrus* Duponchel, 1839. Comment: replacement name for Acentrini Seidlitz, 1890 because of the homonymy of the family-group name; the stem used by Alonso-Zarazaga (2005) was chosen in accordance with Art. 29.6.

### Tribe Ancylocnemidini

Voss, 1962

Ancylocnemini Voss, 1962: 339 [stem: *Ancylocnemid-*]. Type genus: *Ancylocnemis* G. A. K. Marshall, 1920. Comment: incorrect original stem formation, not in prevailing usage.

### Tribe Anthonomini

Thomson, 1859

Anthonomina C. G. Thomson, 1859: 144 [stem: *Anthonom-*]. Type genus: *Anthonomus* Germar, 1817.
*Brachonydes Tournier, 1874: 68, in key [stem: *Brachonych-*]. Type genus: *Brachonyx* Schönherr, 1825. Comment: original vernacular name unavailable (Art. 11.7.2): subsequently used in latinized form but not generally attributed to Tournier (1874); Alonso-Zarazaga and Lyal (1999: 74) attributed this name to the correct author but did not use it as a valid taxon; incorrect original stem formation, not in prevailing usage.
Loncophorini Pierce, 1916: 467 [stem: *Loncophor-*]. Type genus: *Loncophorus* Chevrolat, 1832.
Bradybatini Pierce, 1919: 26, in key [stem: *Bradybat-*]. Type genus: *Bradybatus* Germar, 1824.
Brachonychina Voss, 1944: 38, in key [stem: *Brachonych-*]. Type genus: *Brachonyx* Schönherr, 1825.
Tachypterellina Voss, 1944: 38, in key [stem: *Tachypterell-*]. Type genus: *Tachypterellus* Fall and Cockerell, 1907 [syn. of *Anthonomus* Germar, 1817].

### Tribe Camarotini

Schönherr, 1833

Camarotides Schönherr, 1833: 4 [stem: *Camarot-*]. Type genus: *Camarotus* Germar, 1817.

### Subtribe Camarotina

Schönherr, 1833

Camarotides Schönherr, 1833: 4 [stem: *Camarot-*]. Type genus: *Camarotus* Germar, 1817.

### Subtribe Prionomerina

Lacordaire, 1863

Prionomérides Lacordaire, 1863: 598 [stem: *Prionomer-*]. Type genus: *Prionomerus* Schönherr, 1835 [syn. of *Odontopus* Say, 1831]. Comment: original vernacular name available (Art. 11.7.2): first used in latinized form by Pascoe (1870b: 436, as Prionomerinae), generally accepted as in Alonso-Zarazaga and Lyal (1999: 75, as Prionomerina).

### Tribe Ceratopodini

Lacordaire, 1863

Cératopides Lacordaire, 1863: 589 [stem: *Ceratopod-*]. Type genus: *Ceratopus* Schönherr, 1843. Comment: original vernacular name available (Art. 11.7.2): first used in latinized form by Pascoe (1870b: 436, as Ceratopinae [incorrect stem formation]), generally accepted as in Alonso-Zarazaga and Lyal (1999: 76, as Ceratopodini); incorrect original stem formation, not in prevailing usage.

### Tribe Cionini

Schönherr, 1825

Cionides Schönherr, 1825: column 587 [stem: *Cion-*]. Type genus: *Cionus* Clairville, 1798.

### Tribe Cranopoeini

Kuschel, 2009

Cranopoeini Kuschel, 2009: 44 [stem: *Cranopoe-*]. Type genus: *Cranopoeus* G. A. K. Marshall, 1931.

### Tribe Cryptoplini

Lacordaire, 1863

Cryptoplides Lacordaire, 1863: 486 [stem: *Cryptopl-*]. Type genus: *Cryptoplus* Erichson, 1842. Comment: original vernacular name available (Art. 11.7.2): first used in latinized form by J. L. LeConte (1876: 175, as Cryptopli), generally accepted as in Alonso-Zarazaga and Lyal (1999: 76, as Cryptoplini).
Haplonycides Lacordaire, 1865: 16 [stem: *Haplonych-*]. Type genus: *Haplonyx* Schönherr, 1836. Comment: original vernacular name available (Art. 11.7.2): first used in latinized form by Pascoe (1870b: 437, as Haplonychinae), generally accepted as in Schenkling and G. A. K. Marshall (1936: 1, as Haplonychinae); incorrect original stem formation, not in prevailing usage; this name is a junior homonym of Haplonychini Burmeister, 1855 (type genus *Haplonycha* Dejean, 1836) proposed in Scarabaeidae and now a synonym of Liparetrini H. C. C. Burmeister, 1855; this case is to be referred to the Commission to remove the homonymy (Art. 55.3.1) if either of those names is to be used as valid in the future.

### Tribe Curculionini

Latreille, 1802

Curculionites Latreille, 1802: 195 [stem: *Curculion-*]. Type genus: *Curculio* Linnaeus, 1758.

### Subtribe Curculionina

Latreille, 1802

Curculionites Latreille, 1802: 195 [stem: *Curculion-*]. Type genus: *Curculio* Linnaeus, 1758.
Balaninidae Gistel, 1848: [7] [stem: *Balanin-*]. Type genus: *Balaninus* Germar, 1817 [syn. of *Curculio* Linnaeus, 1758].

### Subtribe Pseudobalaninina

Heller, 1925

Pseudobalaninina Heller, 1925b: 94, in key [stem: *Pseudobalanin-*]. Type genus: *Pseudobalaninus* Faust, 1889.

### Subtribe Timolina

Heller, 1925

Timolina Heller, 1925b: 94, in key [stem: *Timol-*]. Type genus: *Timola* Pascoe, 1886.

### Tribe Diabathrariini

Lacordaire, 1863

Diabathrariides Lacordaire, 1863: 407 [stem: *Diabathrari-*]. Type genus: *Diabathrarius* Schönherr, 1840. Comment: original vernacular name available (Art. 11.7.2): first used in latinized form by Pascoe (1870b: 436, as Diabathrariinae), generally accepted as in Alonso-Zarazaga and Lyal (1999: 141, as Diabathrariini); transfer from Cyclominae by Oberprieler (2010).

### Tribe Ellescini

Thomson, 1859

Elleschina C. G. Thomson, 1859: 143 [stem: *Ellesc-*]. Type genus: *Ellescus* Dejean, 1821 [as *Elleschus*, unjustified emendation of type genus name by Schönherr (1837), not in prevailing usage].

### Subtribe Dorytomina

Bedel, 1886

Dorytomini Bedel, 1886: 280 [stem: *Dorytom-*]. Type genus: *Dorytomus* Germar, 1817.

### Subtribe Ellescina

Thomson, 1859

Elleschina C. G. Thomson, 1859: 143 [stem: *Ellesc-*]. Type genus: *Ellescus* Dejean, 1821 [as *Elleschus*, unjustified emendation of type genus name by Schönherr (1837), not in prevailing usage]. Comment: incorrect original stem formation, not in prevailing usage.

### Tribe Erodiscini

Lacordaire, 1863

Erodiscides Lacordaire, 1863: 566 [stem: *Erodisc-*]. Type genus: *Erodiscus* sensu Lacordaire, 1863 [Lacordaire excluded the type species of *Erodiscus* Schönherr, 1825 to place it in *Toxeutes* Schönherr, 1843; the five species that Lacordaire included in *Erodiscus* are now placed in *Sicoderus* Vanin, 1986, *Prosicoderus* Vanin, 1986 and *Pimelerodius* Vanin, 1986]. Comment: based on a misidentified type genus; MAAZ and CHCL will submit an application for the conservation of this name by designating *Erodiscus* Schönherr, 1825 as its type genus.

### Tribe Eugnomini

Lacordaire, 1863

Eugnomides Lacordaire, 1863: 499 [stem: *Eugnom-*]. Type genus: *Eugnomus* Schönherr, 1847.

### Subtribe Eugnomina

Lacordaire, 1863

Eugnomides Lacordaire, 1863: 499 [stem: *Eugnom-*]. Type genus: *Eugnomus* Schönherr, 1847. Comment: original vernacular name available (Art. 11.7.2): first used in latinized form by J. L. LeConte (1876: 174, as Eugnomi), generally accepted as in Alonso-Zarazaga and Lyal (1999: 79, as Eugnomini); First Reviser found (Eugnomina Lacordaire, 1863 vs Scolopterina Lacordaire, 1863) is Voss (1937a: 37).
Scoloptérides Lacordaire, 1863: 565 [stem: *Scolopter-*]. Type genus: *Scolopterus* A. White, 1846. Comment: original vernacular name available (Art. 11.7.2): first used in latinized form by Pascoe (1870b: 436, as Scolopterinae), generally accepted as in Broun (1880: 472 as Scolopteridae).
Pactolina Voss, 1936: 112, in key [stem: *Pactol-*]. Type genus: *Pactola* Pascoe, 1876.
Stephanorhynchina Voss, 1936: 112 [stem: *Stephanorhynch-*]. Type genus: *Stephanorhynchus* A. White, 1846.

### Subtribe Meriphina

Marshall, 1937

Meriphinae G. A. K. Marshall, 1937a: 329 [stem: *Meriph-*]. Type genus: *Meriphus* Erichson, 1842.

### Tribe Gonipterini

Lacordaire, 1863

Goniptérides Lacordaire, 1863: 391 [stem: *Gonipter-*]. Type genus: *Gonipterus* Schönherr, 1833. Comment: original vernacular name available (Art. 11.7.2): first used in latinized form by Pascoe (1870b: 436, as Gonipterinae), generally accepted as in Alonso-Zarazaga and Lyal (1999: 141, as Gonipterini); transfer from Cyclominae by Oberprieler (2010).

### Tribe Mecinini

Gistel, 1848

Mecinidae Gistel, 1848: [7] [stem: *Mecin-*]. Type genus: *Mecinus* Germar, 1821.
Gymnetrina C. G. Thomson, 1859: 143 [stem: *Gymnetr-*]. Type genus: *Gymnetron* Schönherr, 1825. Comment: junior homonym of Gymnetrinae Swainson, 1839 in Pisces (type genus *Gymnetrus* Bloch, 1795).
Miarides Tournier, 1874: 66 [stem: *Miar-*]. Type genus: *Miarus* Schönherr, 1826. Comment: original vernacular name available (Art. 11.7.2): first used in latinized form and generally accepted as in Pierce (1919: 30, as Miarinae).
Miarini Zherikhin, 1991: 123 [stem: *Miar-*]. Type genus: *Miarus* Schönherr, 1826. Comment: family-group name proposed as new without reference to Miarides Tournier, 1874.

### Tribe Nerthopini

Lacordaire, 1865

Nerthopides Lacordaire, 1865: 19 [stem: *Nerthop-*]. Type genus: *Nerthops* Schönherr, 1826. Comment: original vernacular name available (Art. 11.7.2): first used in latinized form by Pascoe (1870b: 437, as Nerthopinae), generally accepted as in Alonso-Zarazaga and Lyal (1999: 80, as Nerthopini); precedence (Nerthopini Lacordaire, 1865 vs Acallopistini Lacordaire, 1865) given to taxon originally proposed at the higher rank (Art. 24.1).
*Microstylides Lacordaire, 1865: 20 [stem: *Microstyl-*]. Type genus: *Microstylus* Schönherr, 1847. Comment: original vernacular name unavailable (Art. 11.7.2): subsequently used in latinized form but not generally attributed to Lacordaire (1865); subsequent usage of Microstylinae by Hoffmann (1968: 27) and Microstylidae by Ienistea (1986: 33) did not validate this name because these authors did not attribute their names to Lacordaire, 1865; Microstylinae Hoffmann, 1968: 27 and Microstylidae Ienistea, 1986: 33 are also unavailable because they were proposed after 1930 without description or bibliographic reference to such a description (Art. 13.1).
Acallopistides Lacordaire, 1865: 22 [stem: *Acallopist-*]. Type genus: *Acallopistus* Schönherr, 1826. Comment: original vernacular name available (Art. 11.7.2): first used in latinized form by Hustache (1932: 371, as Acallopistinae), generally accepted as in Hustache (1934: 3, as Acallopistini).
Archolabinae Voss, 1929a: 200 [stem: *Archolab-*]. Type genus: *Archolabus* Voss, 1929.

### Tribe Otidocephalini

Lacordaire, 1863

Otidocéphalides Lacordaire, 1863: 568 [stem: *Otidocephal-*]. Type genus: *Otidocephalus* Chevrolat, 1832. Comment: original vernacular name available (Art. 11.7.2): first used in latinized form by Pascoe (1870b: 436, as Otidocephalinae), generally accepted as in Alonso-Zarazaga and Lyal (1999: 80, as Otidocephalini).

### Tribe Piazorhinini

Lacordaire, 1863

Piazorhinides Lacordaire, 1863: 601 [stem: *Piazorhin-*]. Type genus: *Piazorhinus* Schönherr, 1835. Comment: original vernacular name available (Art. 11.7.2): first used in latinized form by Kirsch (1875: 162, as Piazorhinidae), generally accepted as in Alonso-Zarazaga and Lyal (1999: 81, as Piazorhinini).

### Tribe Prionobrachiini

Hustache, 1938

Prionobrachiina Hustache, 1938: 90, in key [stem: *Prionobrachi-*]. Type genus: *Prionobrachium* Faust, 1894.

### Tribe Pyropini

Lacordaire, 1865

Pyropides Lacordaire, 1865: 187 [stem: *Pyrop-*]. Type genus: *Pyropus* Schönherr, 1836. Comment: original vernacular name available (Art. 11.7.2): first used in latinized form by Pascoe (1870b: 437, as Pyropinae), generally accepted as in Alonso-Zarazaga and Lyal (1999: 81, as Pyropini).

### Tribe Rhamphini

Rafinesque, 1815

Ramphoria Rafinesque, 1815: 115 [stem: *Rhamph-*]. Type genus: *Rhamphus* Clairville, 1798 [as *Ramphorus*, incorrect subsequent spelling of type genus name, not in prevailing usage]. Comment: incorrect original stem formation, not in prevailing usage.

### Subtribe Dinorhopalina

Voss, 1936

Dinorrhopalina Voss, 1936: 112, in key [stem: *Dinorhopal-*]. Type genus: *Dinorhopala* Pascoe, 1860 [as *Dinorrhopala*, incorrect subsequent spelling of type genus name, not in prevailing usage]. Comment: incorrect original stem formation, not in prevailing usage.

### Subtribe Ixalmina

Voss, 1936

Ixalmina Voss, 1936: 112, in key [stem: *Ixalm-*]. Type genus: *Ixalma* Pascoe, 1871.

### Subtribe Rhamphina

Rafinesque, 1815

Ramphoria Rafinesque, 1815: 115 [stem: *Rhamph-*]. Type genus: *Rhamphus* Clairville, 1798 [as *Ramphorus*, incorrect subsequent spelling of type genus name, not in prevailing usage]. Comment: incorrect original stem formation, not in prevailing usage.
Rhamphides Schönherr, 1823: column 1136 [stem: *Rhamph-*]. Type genus: *Rhamphus* Clairville, 1798. Comment: family-group name proposed as new without reference to Ramphoria Rafinesque, 1815.
Orchestides Latreille, 1828: 601 [stem: *Orchest-*]. Type genus: *Orchestes* Illiger, 1798.
Rhynchenidae Blanchard, 1853: 240 [stem: *Rhynchaen-*]. Type genus: *Rhynchaenus* Clairville, 1798. Comment: incorrect original stem formation, not in prevailing usage; an application will need to be submitted to the Commission to suppress Rhynchaenides Latreille, 1828 (based on the misidentified type genus *Rhynchaenus* sensu Latreille, 1828) for the Principles of Priority and Homonymy (Art. 65.2.1) if this name is to be used as valid.

### Subtribe Tachygonina

Lacordaire, 1865

Tachygonides Lacordaire, 1865: 167 [stem: *Tachygon-*]. Type genus: *Tachygonus* Schönherr, 1833 [syn. of *Tachygonus* Guérin-Méneville, 1833]. Comment: original vernacular name available (Art. 11.7.2): first used in latinized form by Pascoe (1870b: 437, as Tachygoninae), generally accepted as in Alonso-Zarazaga and Lyal (1999: 82, as Tachygonina); the type genus for Lacordaire’s Tachygonides is *Tachygonus* Schönherr, 1833; an application will be submitted by MAAZ and CHCL to the Commission to designate *Tachygonus* Guérin-Méneville, [1833] as the type genus of this family-group name.

### Tribe Smicronychini

Seidlitz, 1891 nomen protectum

Desmorhines J. L. LeConte, 1876: 167 [stem: *Desmorin-*]. Type genus: *Desmoris* J. L. LeConte, 1876 [syn. of *Smicronyx* Schönherr, 1843]. Comment: *nomen oblitum* (see Appendix 1); incorrect original stem formation, not in prevailing usage.
Smicronycina Seidlitz, 1891 [Gatt.]: 162 [stem: *Smicronych-*]. Type genus: *Smicronyx* Schönherr, 1843. Comment: *nomen protectum* (see Appendix 1); incorrect original stem formation, not in prevailing usage.

### Tribe Sphaeriopoeini

Kuschel, 2003

Sphaeriopoeini Kuschel, 2003: 63 [stem: *Sphaeriopoe-*]. Type genus: *Sphaeriopoeus* Kuschel, 2003.

### Tribe Storeini

Lacordaire, 1863

Storéides Lacordaire, 1863: 494 [stem: *Store-*]. Type genus: *Storeus* Schönherr, 1843. Comment: original vernacular name available (Art. 11.7.2): first used in latinized form by Kirsch (1877: 170, as Storeidae), generally accepted as in Alonso-Zarazaga and Lyal (1999: 82, as Storeini).
Hybomorphides Lacordaire, 1865: 141 [stem: *Hybomorph-*]. Type genus: *Hybomorphus* Saunders and Jekel, 1855. Comment: original vernacular name available (Art. 11.7.2): first used in latinized form and generally accepted as in Hustache (1936: 261, as Hybomorphina).

### Tribe Styphlini

Jekel, 1861

Styphlidae Jekel, 1861: 274 [stem: *Styphl-*]. Type genus: *Styphlus* Schönherr, 1826.
*Orthochaetini A. Winkler, 1932: 1544 [stem: *Orthochaet-*]. Type genus: *Orthochaetes* Germar, 1824. Comment: unavailable family-group name, proposed after 1930 without description or bibliographic reference to such a description (Art. 13.1).
Orthocaetina Morimoto, 1962a: 56, in key [stem: *Orthochaet-*]. Type genus: *Orthochaetes* Germar, 1824. Comment: incorrect original stem formation, not in prevailing usage.

### Tribe Tychiini

Gistel, 1848

Tychiidae Gistel, 1848: [7] [stem: *Tychi-*]. Type genus: *Tychius* Germar, 1817.

### Subtribe Demimaeina

Voss, 1937

Demimaeini Voss, 1937b: 144 [stem: *Demimae-*]. Type genus: *Demimaea* Pascoe, 1870.

### Subtribe Lignyodina

Bedel, 1884

Lignyodini Bedel, 1884a: 66, in key [stem: *Lignyod-*]. Type genus: *Lignyodes* Dejean, 1835.

### Subtribe Ochyromerina

Voss, 1935

Ochyromerina Voss, 1935a: 228 [stem: *Ochyromer-*]. Type genus: *Ochyromera* Pascoe, 1874.
Endaeini Voss, 1958: 101, in key [stem: *Endae-*]. Type genus: *Endaeus* Schönherr, 1826.

### Subtribe Tychiina

Gistel, 1848

Tychiidae Gistel, 1848: [7] [stem: *Tychi-*]. Type genus: *Tychius* Germar, 1817.
Miccotrogidae Gistel, 1856a: 371 [stem: *Miccotrog-*]. Type genus: *Miccotrogus* Schönherr, 1825 [syn. of *Tychius* Germar, 1817].
Sibynidae Marseul, 1863: 239 [stem: *Sibini-*]. Type genus: *Sibinia* Germar, 1817 [as *Sibynes*, unjustified emendation of type genus name by Schönherr (1825), not in prevailing usage]. Comment: incorrect original stem formation, not in prevailing usage.

### Tribe Ulomascini

Lacordaire, 1865

Ulomascides Lacordaire, 1865: 184 [stem: *Ulomasc-*]. Type genus: *Ulomascus* Fairmaire, 1848. Comment: original vernacular name available (Art. 11.7.2): first used in latinized form by Pascoe (1870b: 437, as Ulomascinae), generally accepted as in Alonso-Zarazaga and Lyal (1999: 86, as Ulomascini).

### Tribe Viticiini

Morimoto, 1983

Viticiinae Morimoto, 1983: 55 [stem: *Vitici-*]. Type genus: *Viticis* Lea, 1930. Comment: transfer from Cyclominae by Oberprieler (2010).

### Subfamily Bagoinae

Thomson, 1859 nomen protectum

Lypriidae Gistel, 1848: [7] [stem: *Lypr-*]. Type genus: *Lyprus* Schönherr, 1826 [syn. of *Bagous* Germar, 1817]. Comment: as pointed out by Alonso-Zarazaga and Lyal (2002: 17) this is the oldest available name for the subfamily; Lyprinae Gistel, 1848 was recently considered a *nomen oblitum* by Colonnelli (2003: 7) however the necessary supporting references were not provided; we hereby consider Lyprinae Gistel 1848 as a *nomen oblitum* (see supporting references in Appendix 1); incorrect original stem formation, not in prevailing usage.
Bagoina C. G. Thomson, 1859: 135 [stem: *Bago-*]. Type genus: *Bagous* Germar, 1817. Comment: *nomen protectum* (see Appendix 1).
Hydronomides Lacordaire, 1863: 483 [stem: *Hydronom-*]. Type genus: *Hydronomus* Schönherr, 1825. Comment: original vernacular name available (Art. 11.7.2): first used in latinized form by J. L. LeConte (1876: 182, as Hydronomi), generally accepted as in Zimmerman (1993: 150, as Hydronomini).
Pseudobagoini Sharp, 1917: 26 [stem: *Pseudobago-*]. Type genus: *Pseudobagous* Sharp, 1917.

### Subfamily Baridinae

Schönherr, 1836

Baridides Schönherr, 1836: 636 [stem: *Barid-*]. Type genus: *Baris* Germar, 1817 [as *Baridius*, unjustified emendation of type genus name by Schönherr (1825), not in prevailing usage].

### Tribe Ambatini

Lacordaire, 1863

Ambatides Lacordaire, 1863: 512 [stem: *Ambat-*]. Type genus: *Ambates* Schönherr, 1836. Comment: original vernacular name available (Art. 11.7.2): first used in latinized form by Pascoe (1870b: 436, as Ambatinae), generally accepted as in Alonso-Zarazaga and Lyal (1999: 93, as Ambatini).
*Pteracanthides Chevrolat, 1878: 341 [stem: *Pteracanth-*]. Type genus: *Pteracanthus* Dejean, 1835. Comment: original vernacular name unavailable (Art. 11.7.2): not subsequently latinized; a family-group name with the same stem, Pteracanthina J. Thomson, 1864 (type genus *Pteracantha* Newman, 1838), is available in Coleoptera: Cerambycidae.

### Tribe Anopsilini

Bondar, 1942

Anopsilini Bondar, 1942: 253 [stem: *Anopsil-*]. Type genus: *Anopsilus* Kirsch, 1870.

### Tribe Apostasimerini

Schönherr, 1844

Apostasimerides Schönherr, 1844: 1 [stem: *Apostasimer-*]. Type genus: *Apostasimerus* Schönherr, 1844. Comment: see Prena (2009: 33) for details on authorship and taxa included in this tribe.

### Subtribe Apostasimerina

Schönherr, 1844

*Apostasimerides Schönherr, 1836: 557 [stem: *Apostasimer-*]. Type genus: *Apostasimerus* Schönherr, 1844. Comment: family-group name unavailable (Art. 11.7.1.1): not based on an available genus name at the time.
Apostasimerides Schönherr, 1844: 1 [stem: *Apostasimer-*]. Type genus: *Apostasimerus* Schönherr, 1844.

### Subtribe Madopterina

Lacordaire, 1865

Madoptérides Lacordaire, 1865: 243 [stem: *Madopter-*]. Type genus: *Madopterus* Schönherr, 1836. Comment: original vernacular name available (Art. 11.7.2): first used in latinized form by Kirsch (1870: 218, as Madopteridae), generally accepted as in Alonso-Zarazaga and Lyal (1999: 96, as Madopterini).

### Subtribe Thaliabaridina

Bondar, 1943

Thaliabarini Bondar, 1943b: 131 [stem: *Thaliabarid-*]. Type genus: *Thaliabaris* Bondar, 1943. Comment: incorrect original stem formation, not in prevailing usage.

### Subtribe Torcina

Bondar, 1943

Torcocina Bondar, 1943a: 53 [stem: *Torc-*]. Type genus: *Torcus* Casey, 1922. Comment: incorrect original stem formation, not in prevailing usage.

### Subtribe Zygobaridina

Pierce, 1907

Centrinides Jekel, 1865: 548 [stem: *Centrin-*]. Type genus: *Centrinus* Schönherr, 1825. Comment: junior homonym of Centrininae Swainson, 1839 in Pisces (type genus *Centrina* Cuvier 1817); this case is to be referred to the Commission to remove the homonymy (Art. 55.3.1).
Zygobari Pierce, 1907: 381 [stem: *Zygobarid-*]. Type genus: *Zygobaris* J. L. LeConte, 1876. Comment: incorrect original stem formation, not in prevailing usage; Alonso-Zarazaga and Lyal (1999: 97) and subsequent workers have used this name as valid instead of the junior homonym Centrinina Jekel, 1865, we continue to use Zygobaridina as valid here.
Limnobarini Casey, 1922: 269 [stem: *Limnobarid-*]. Type genus: *Limnobaris* Bedel, 1885. Comment: incorrect original stem formation, not in prevailing usage.

### Tribe Baridini

Schönherr, 1836

Baridides Schönherr, 1836: 636 [stem: *Barid-*]. Type genus: *Baris* Germar, 1817 [as *Baridius*, unjustified emendation of type genus name by Schönherr (1825), not in prevailing usage].

### Subtribe Baridina

Schönherr, 1836

Baridides Schönherr, 1836: 636 [stem: *Barid-*]. Type genus: *Baris* Germar, 1817 [as *Baridius*, unjustified emendation of type genus name by Schönherr (1825), not in prevailing usage].

### Subtribe Coelonertina

Casey, 1922

Coelonertini Casey, 1922: 94 [stem: *Coelonert-*]. Type genus: *Coelonertus* Solari and Solari, 1906.

### Subtribe Coleomerina

Casey, 1922

Coleomerini Casey, 1922: 84 [stem: *Coleomer-*]. Type genus: *Coleomerus* Schönherr, 1836.

### Subtribe Diorymerina

Jekel, 1865

Diorymerides Jekel, 1865: 548 [stem: *Diorymer-*]. Type genus: *Diorymerus* Schönherr, 1825.

### Subtribe Eurhinina

Lacordaire, 1865

Eurhinides Lacordaire, 1865: 220 [stem: *Eurhin-*]. Type genus: *Eurhinus* Illiger, 1807 [placed on the Official List of Generic Names in Zoology (ICZN 1985e)]. Comment: original vernacular name available (Art. 11.7.2): first used in latinized form by Pierce (1915: 15, as Eurhininae), generally accepted as in Alonso-Zarazaga and Lyal (1999: 92, as Eurhinina); Eurhinini Lacordaire, 1865 placed on the Official List of Family-Group Names in Zoology (ICZN 1985e).

### Tribe Madarini

Jekel, 1865

Madarides Jekel, 1865: 550 [stem: *Madar-*]. Type genus: *Madarus* Schönherr, 1825. Comment: Madarini, published on 25 January 1865, has priority over Barymerini and Leptoschoinini both published by Lacordaire “before 12 December 1865”.

### Subtribe Barymerina

Lacordaire, 1865

Barymérides Lacordaire, 1865: 259 [stem: *Barymer-*]. Type genus: *Barymerus* Lacordaire, 1865. Comment: original vernacular name available (Art. 11.7.2): first used in latinized form by Faust (1896: 127, as Barymerinorum), generally accepted as in Alonso-Zarazaga and Lyal (1999: 94, as Barymerina).
Sonnetiini Casey, 1922: 321 [stem: *Sonneti-*]. Type genus: *Sonnetius* Casey, 1922.

### Subtribe Eutoxina

Champion, 1908

Eutoxides Champion, 1908: 360 [stem: *Eutox-*]. Type genus: *Eutoxus* Schönherr, 1844.

### Subtribe Leptoschoinina

Lacordaire, 1865

Leptoschoïnides Lacordaire, 1865: 236 [stem: *Leptoschoin-*]. Type genus: *Leptoschoinus* Dejean, 1836. Comment: original vernacular name available (Art. 11.7.2): first used in latinized form by Faust (1896: 126, as Leptoschoenorum [incorrect stem formation]), generally accepted as in Alonso-Zarazaga and Lyal (1999: 94, as Leptoschoinina).

### Subtribe Madarina

Jekel, 1865

Madarides Jekel, 1865: 550 [stem: *Madar-*]. Type genus: *Madarus* Schönherr, 1825.

### Subtribe Tonesiina

Alonso-Zarazaga and Lyal, 1999

Lytériides Lacordaire, 1865: 249 [stem: *Lyteri-*]. Type genus: *Lyterius* sensu Lacordaire, 1865 [not *Lyterius* Schönherr, 1844; syn. of *Tonesia* Casey, 1922]. Comment: based on a misidentified type genus, name treated here as invalid until an application is submitted to the Commission to suppress it for the Principle of Priority (Art. 65.2.1).
Tonesiina Alonso-Zarazaga and Lyal, 1999: 95 [stem: *Tonesi-*]. Type genus: *Tonesia* Casey, 1922. Comment: although this is not the oldest name for the subtribe, we recommend that an application be submitted to the Commission to suppress Lyteriini Lacordaire, 1865 because it is based on a misidentified type genus (Art. 65.2.1).

### Tribe Neosharpiini

Hoffmann, 1956

Neosharpini Hoffmann, 1956: 246 [stem: *Neosharpi-*]. Type genus: *Neosharpia* Hoffmann, 1956. Comment: incorrect original stem formation, not in prevailing usage.

### Tribe Nertinini

Voss, 1954

*Nertides Lacordaire, 1865: 238 [stem: *Nert-*]. Type genus: *Nertus* Schönherr, 1844 [preoccupied genus name, not *Nertus* Boie, 1828 [Aves]; syn. of *Nertinus* G. A. K. Marshall, 1943]. Comment: original vernacular name unavailable (Art. 11.7.2): not subsequently latinized and attributed to Lacordaire (1865); if found to be available then permanently invalid (Art. 39): based on preoccupied type genus.
Nertini Bondar, 1949: 223 [stem: *Nert-*]. Type genus: *Nertus* Schönherr, 1844 [preoccupied genus name, not *Nertus* Boie 1828 [Aves]; syn. of *Nertinus* G. A. K. Marshall, 1943]. Comment: family-group name proposed as new without reference to Nertides Lacordaire, 1865; permanently invalid (Art. 39): based on preoccupied type genus.
Nertinina Voss, 1954: 323 [stem: *Nertin-*]. Type genus: *Nertinus* G. A. K. Marshall, 1943.

### Tribe Optatini

Champion, 1907

Optatides Champion, 1907: 185 [stem: *Optat-*]. Type genus: *Optatus* Pascoe, 1889.

### Tribe Pantotelini

Lacordaire, 1865

Pantotélides Lacordaire, 1865: 212 [stem: *Pantotel-*]. Type genus: *Pantoteles* Schönherr, 1845.

### Subtribe Cyrionychina

Casey, 1922

Cyrionichini Casey, 1922: 10 [stem: *Cyrionych-*]. Type genus: *Cyrionyx* Faust, 1896. Comment: incorrect original stem formation, not in prevailing usage.

### Subtribe Pantotelina

Lacordaire, 1865

Pantotélides Lacordaire, 1865: 212 [stem: *Pantotel-*]. Type genus: *Pantoteles* Schönherr, 1845. Comment: original vernacular name available (Art. 11.7.2): first used in latinized form by Pascoe (1870b: 437, as Pantotelinae), generally accepted as in Alonso-Zarazaga and Lyal (1999: 102, as Pantotelini).

### Tribe Peridinetini

Lacordaire, 1865

Péridinétides Lacordaire, 1865: 209 [stem: *Peridinet-*]. Type genus: *Peridinetus* Schönherr, 1837. Comment: original vernacular name available (Art. 11.7.2): first used in latinized form by Pascoe (1886: cliii, as Peridinetinae), generally accepted as in Alonso-Zarazaga and Lyal (1999: 102, as Peridinetini).

### Subfamily Ceutorhynchinae

Gistel, 1848

Centorhynchidae Gistel, 1848: [7] [stem: *Ceutorhynch-*]. Type genus: *Ceutorhynchus* Germar, 1824 [as *Centorhynchus*, incorrect subsequent spelling of type genus name, not in prevailing usage; placed on the Official List of Generic Names in Zoology (ICZN 1989c)]. Comment: incorrect original stem formation, not in prevailing usage; First Revisers found (Ceutorhynchinae Gistel, 1848 vs Phytobiinae Gistel, 1848) are Alonso-Zarazaga and Lyal (2002).

### Tribe Ceutorhynchini

Gistel, 1848

Centorhynchidae Gistel, 1848: [7] [stem: *Ceutorhynch-*]. Type genus: *Ceutorhynchus* Germar, 1824 [as *Centorhynchus*, incorrect subsequent spelling of type genus name, not in prevailing usage; placed on the Official List of Generic Names in Zoology (ICZN 1989c)]. Comment: incorrect original stem formation, not in prevailing usage.
Isorhynchides Lacordaire, 1865: 172 [stem: *Isorhynch-*]. Type genus: *Isorhynchus* Schönherr, 1833. Comment: original vernacular name available (Art. 11.7.2): first used in latinized form by Pascoe (1870b: 437, as Isorhynchinae), generally accepted as in Hustache (1934: 72, as Isorhynchini).
Coeliodes J. L. LeConte, 1876: 268 [stem: *Coeliod-*]. Type genus: *Coeliodes* Schönherr, 1837 [placed on the Official List of Generic Names in Zoology (ICZN 1989c)].
Poophagidae Schultze, 1902: 226 [stem: *Poophag-*]. Type genus: *Poophagus* Schönherr, 1837.
Amalina Wagner, 1936: 166 [stem: *Amal-*]. Type genus: *Amalus* Schönherr, 1825.
Phrydiuchina Wagner, 1938: 163 [stem: *Phrydiuch-*]. Type genus: *Phrydiuchus* Gozis, 1885.
Oxyonyxina Hoffmann, 1957b: 220 [stem: *Oxyonych-*]. Type genus: *Oxyonyx* Faust, 1885. Comment: incorrect original stem formation, not in prevailing usage.

### Tribe Cnemogonini

Colonnelli, 1979

Cnemogonini Colonnelli, 1979: 2 [stem: *Cnemogon-*]. Type genus: *Cnemogonus* J. L. LeConte, 1876.

### Tribe Egriini

Pajni and Kohli, 1982

Egriini Pajni and Kohli, 1982: 339, in key [stem: *Egri-*]. Type genus: *Egrius* Pascoe, 1865.
Egriini Colonnelli, 1984: 209 [stem: *Egri-*]. Type genus: *Egrius* Pascoe, 1865. Comment: family-group name proposed as new without reference to Egriini Pajni and Kohli, 1982.

### Tribe Hypohypurini

Colonnelli, 2004

Hypohypurini Colonnelli, 2004: 10 [stem: *Hypohypur-*]. Type genus: *Hypohypurus* Hustache, 1920.

### Tribe Hypurini

Schultze, 1902

Hypuridae Schultze, 1902: 209 [stem: *Hypur-*]. Type genus: *Hypurus* Rey, 1882.

### Tribe Lioxyonychini

Colonnelli, 1984

Lioxyonyxini Colonnelli, 1984: 209 [stem: *Lioxyonych-*]. Type genus: *Lioxyonyx* Hustache, 1933. Comment: incorrect original stem formation, not in prevailing usage.

### Tribe Mecysmoderini

Wagner, 1938

Mecysmoderina Wagner, 1938: 170 [stem: *Mecysmoder-*]. Type genus: *Mecysmoderes* Schönherr, 1837.

### Tribe Mononychini

LeConte, 1876

Mononychi J. L. LeConte, 1876: 267 [stem: *Mononych-*]. Type genus: *Mononychus* Germar, 1824 [placed on the Official List of Generic Names in Zoology (ICZN 1989c)].

### Tribe Phytobiini

Gistel, 1848

Phytobiidae Gistel, 1848: [7] [stem: *Phytobi-*]. Type genus: *Phytobius* Schönherr, 1833 [placed on the Official List of Generic Names in Zoology (ICZN 2001)].
Rhinoncides C. G. Thomson, 1865: 231 [stem: *Rhinonc-*]. Type genus: *Rhinoncus* Schönherr, 1825 [placed on the Official List of Generic Names in Zoology (ICZN 1989c)].

### Tribe Scleropterini

Schultze, 1902

Scleropteridae Schultze, 1902: 209 [stem: *Scleropter-*]. Type genus: *Scleropterus* Schönherr, 1825. Comment: the family-group name Scléropterides was used earlier by Solier (1834) in the Heteromera, however, Solier’s name was not based on a genus name and is therefore unavailable; Agassiz (1846a: 146) incorrectly mentions *Scleropterus* as the type genus of Solier’s name but this is impossible because the family-group and genus-group names belonged to different orders; Agassiz (1846b: 335) also latinized Solier’s name as Scleropteroidae.

### Subfamily Conoderinae

Schönherr, 1833

Conoderides Schönherr, 1833: 26 [stem: *Conoder-*]. Type genus: *Conoderes* Schönherr, 1833.

### Tribe Arachnopodini

Lacordaire, 1865

Arachnopides Lacordaire, 1865: 159 [stem: *Arachnopod-*]. Type genus: *Arachnopus* Guérin-Méneville, 1838 [syn. of *Arachnobas* Boisduval, 1835]. Comment: original vernacular name available (Art. 11.7.2): first used in latinized form by Heyne and Taschenberg (1907: 232, as Arachnopini [incorrect stem formation]), generally accepted as in Alonso-Zarazaga and Lyal (1999: 109, as Arachnopodini); incorrect original stem formation, not in prevailing usage.

### Tribe Campyloscelini

Schönherr, 1845

Campyloscelides Schönherr, 1845: 197 [stem: *Campyloscel-*]. Type genus: *Campyloscelus* Schönherr, 1845.

### Subtribe Campyloscelina

Schönherr, 1845

Campyloscelides Schönherr, 1845: 197 [stem: *Campyloscel-*]. Type genus: *Campyloscelus* Schönherr, 1845.

### Subtribe Corynemerina

Hustache, 1929

Corynemerinae Hustache, 1929: 531 [stem: *Corynemer-*]. Type genus: *Corynemerus* Fåhraeus, 1871.

### Subtribe Phaenomerina

Faust, 1898

Phaenomerina Faust, 1898: 76, in key [stem: *Phaenomer-*]. Type genus: *Phaenomerus* Schönherr, 1836 [placed on the Official List of Generic Names in Zoology (ICZN 1962)]. Comment: the stem of the senior homonym based on the Scarabaeidae genus *Phaenomeris* was emended to *Phaeromerid*- to remove the homonymy (ICZN 1962); available for family-group taxon below subfamily level and placed on the Official List of Family-Group Names in Zoology (ICZN 1962).

### Tribe Conoderini

Schönherr, 1833

Conoderides Schönherr, 1833: 26 [stem: *Conoder-*]. Type genus: *Conoderes* Schönherr, 1833. Comment: the name Conoderinae Fleutiaux, 1919 (type genus *Conoderus* Eschscholtz, 1829) is available in Elateridae; this case is to be referred to the Commission to remove the homonymy (Art. 55.3.1).
Conophorides Schönherr, 1837: 719 [stem: *Conophor-*]. Type genus: *Conophorus* Schönherr, 1837 [preoccupied genus name, not *Conophorus* Meigen, 1803 [Diptera]; syn. of *Conoderes* Schönherr, 1833]. Comment: permanently invalid (Art. 39): based on preoccupied type genus.

### Tribe Coryssomerini

Thomson, 1859

Coryssomerina C. G. Thomson, 1859: 137 [stem: *Coryssomer-*]. Type genus: *Coryssomerus* Schönherr, 1825.
Synophthalmides Lacordaire, 1863: 514 [stem: *Synophthalm-*]. Type genus: *Synophthalmus* Lacordaire, 1863. Comment: original vernacular name available (Art. 11.7.2): first used in latinized form by Hustache (1932: 373, as Synophthalmini), generally accepted as in Hustache (1934: 49, as Synophthalmini).
Metialmini Hustache, 1932: 374, in key [stem: *Metialm-*]. Type genus: *Metialma* Pascoe, 1871.

### Tribe Coryssopodini

Lacordaire, 1865

Coryssopides Lacordaire, 1865: 163 [stem: *Coryssopod-*]. Type genus: *Coryssopus* Schönherr, 1826. Comment: original vernacular name available (Art. 11.7.2): first used in latinized form by Heyne and Taschenberg (1907: 232, as Coryssopini [incorrect stem formation]), generally accepted as in Alonso-Zarazaga and Lyal (1999: 110, as Coryssopodini); incorrect original stem formation, not in prevailing usage.
*Sympiézopides Lacordaire, 1865: 166 [stem: *Sympiezopod-*]. Type genus: *Sympiezopus* Schönherr, 1837. Comment: original vernacular name unavailable (Art. 11.7.2): subsequently used in latinized form, e.g. Faust (1886b: 367, as Sympiezopinorum [incorrect stem formation]), but not generally attributed to Lacordaire (1865); incorrect original stem formation, not in prevailing usage.

### Tribe Lechriopini

Lacordaire, 1865

Léchriopides Lacordaire, 1865: 149 [stem: *Lechriop-*]. Type genus: *Lechriops* Schönherr, 1825. Comment: original vernacular name available (Art. 11.7.2): first used in latinized form by Heyne and Taschenberg (1907: 232, as Lechriopini), generally accepted as in Alonso-Zarazaga and Lyal (1999: 111, as Lechriopini).
Copturidae Desbrochers des Loges, 1892: 68 [stem: *Coptur-*]. Type genus: *Copturus* Schönherr, 1825.

### Tribe Lobotrachelini

Lacordaire, 1865

Lobotrachélides Lacordaire, 1865: 172 [stem: *Lobotrachel-*]. Type genus: *Lobotrachelus* Schönherr, 1837. Comment: original vernacular name available (Art. 11.7.2): first used in latinized form by Aurivillius (1910: 433, as Lobotrachelinae), generally accepted as in Alonso-Zarazaga and Lyal (1999: 111, as Lobotrachelini).

### Tribe Mecopini

Lacordaire, 1865

Mécopides Lacordaire, 1865: 156 [stem: *Mecop-*]. Type genus: *Mecopus* Schönherr, 1825. Comment: original vernacular name available (Art. 11.7.2): first used in latinized form by Heyne and Taschenberg (1907: 232, as Mecopini), generally accepted as in Alonso-Zarazaga and Lyal (1999: 111, as Mecopini); current spelling maintained (Art. 29.5): incorrect original stem formation in prevailing usage (should be *Mecopod*-); if the stem is corrected, it would threaten stability of a widely used name in Orthoptera (see Alonso-Zarazaga and Lyal 1999: 111).

### Tribe Menemachini

Lacordaire, 1865

Ménémachides Lacordaire, 1865: 27 [stem: *Menemach-*]. Type genus: *Menemachus* Schönherr, 1843. Comment: original vernacular name available (Art. 11.7.2): first used in latinized form by Pascoe (1870b: 437, as Mnemachinae [incorrect stem formation]), generally accepted as in Alonso-Zarazaga and Lyal (1999: 112, as Menemachini).

### Tribe Othippiini

Morimoto, 1962

Othippiini Morimoto, 1962a: 47, in key [stem: *Othippi-*]. Type genus: *Othippia* Pascoe, 1874.

### Tribe Peloropodini

Hustache, 1932

Peleropini Hustache, 1932: 372, in key [stem: *Peloropod-*]. Type genus: *Peloropus* Schönherr, 1836. Comment: incorrect original stem formation, not in prevailing usage.

### Tribe Piazurini

Lacordaire, 1865

Piazurides Lacordaire, 1865: 144 [stem: *Piazur-*]. Type genus: *Piazurus* Schönherr, 1825. Comment: original vernacular name available (Art. 11.7.2): first used in latinized form by Faust (1899: 100, as Piazurinorum), generally accepted as in Alonso-Zarazaga and Lyal (1999: 113, as Piazurini).

### Tribe Sphadasmini

Lacordaire, 1865

Sphadasmides Lacordaire, 1865: 161 [stem: *Sphadasm-*]. Type genus: *Sphadasmus* Schönherr, 1844. Comment: original vernacular name available (Art. 11.7.2): first used in latinized form by Faust (1899: 102, as Sphadasminorum), generally accepted as in Alonso-Zarazaga and Lyal (1999: 114, as Sphadasmini).

### Tribe Trichodocerini

Champion, 1906

Trichodocerides Champion, 1906: 713 [stem: *Trichodocer-*]. Type genus: *Trichodocerus* Chevrolat, 1879.

### Tribe Zygopini

Lacordaire, 1865

Zygopides Lacordaire, 1865: 142 [stem: *Zygop-*]. Type genus: *Zygops* Schönherr, 1825 [placed on the Official List of Generic Names in Zoology (ICZN 1987e)]. Comment: original vernacular name available (Art. 11.7.2): first used in latinized form by Pascoe (1870b: 437, as Zygopinae), generally accepted as in Alonso-Zarazaga and Lyal (1999: 114, as Zygopini).
Eccoptinae Pierce, 1919: 30, in key [stem: *Eccopt-*]. Type genus: *Eccoptus* Dejean, 1821 [placed on the Official Index of Rejected and Invalid Generic Names in Zoology (ICZN 1987e); syn. of *Zygops* Schönherr, 1825]. Comment: permanently invalid (Art. 39): based on suppressed type genus.
Cylindrocopturini Böving, 1927b: 156, in key [stem: *Cylindrocoptur-*]. Type genus: *Cylindrocopturus* Heller, 1895.

### Subfamily Cossoninae

Schönherr, 1825

Cossonides Schönherr, 1825: column 587 [stem: *Cosson-*]. Type genus: *Cossonus* Clairville, 1798.

### Tribe Acamptini

LeConte, 1876

Acampti J. L. LeConte, 1876: 238 [stem: *Acampt-*]. Type genus: *Acamptus* J. L. LeConte, 1876.

### Tribe Acanthinomerini

Voss, 1972

Acanthinomerini Voss, 1972: 382 [stem: *Acanthinomer-*]. Type genus: *Acanthinomerus* Boheman, 1859.

### Tribe Allomorphini

Folwaczny, 1973

Allomorphini Folwaczny, 1973: 68, in key [stem: *Allomorph-*]. Type genus: *Allomorphus* Folwaczny, 1968.

### Tribe Aphyllurini

Voss, 1955

Aphyllurina Voss, 1955a: 276 [stem: *Aphyllur-*]. Type genus: *Aphyllura* Reitter, 1884.

### Tribe Araucariini

Kuschel, 1966

Araucariini Kuschel, 1966: 4 [stem: *Araucari-*]. Type genus: *Araucarius* Kuschel, 1966.

### Tribe Choerorhinini

Folwaczny, 1973

Choerorhini Folwaczny, 1973: 69, in key [stem: *Choerorhin-*]. Type genus: *Choerorhinus* Fairmaire, 1858. Comment: incorrect original stem formation, not in prevailing usage.

### Tribe Cossonini

Schönherr, 1825

Cossonides Schönherr, 1825: column 587 [stem: *Cosson-*]. Type genus: *Cossonus* Clairville, 1798.

### Tribe Cryptommatini

Voss, 1972

Cryptommatini Voss, 1972: 373 [stem: *Cryptommat-*]. Type genus: *Cryptommata* Wollaston, 1877.

### Tribe Dryotribini

LeConte, 1876

Dryotribi J. L. LeConte, 1876: 335 [stem: *Dryotrib-*]. Type genus: *Dryotribus* G. H. Horn, 1873.
Cotasterinen Faust, 1886a: 28 [stem: *Cotaster-*]. Type genus: *Cotaster* Motschulsky, 1851. Comment: also as Cotasteriden on p. 31; original vernacular name available (Art. 11.7.2): first used in latinized form by Sainte-Claire Deville (1923: 63, as Cotastrini [incorrect stem formation]), generally accepted as in Hansen (1996: 209, as Cotasterini).

### Tribe Microxylobiini

Voss, 1972

Microxylobiini Voss, 1972: 431 [stem: *Microxylobi-*]. Type genus: *Microxylobius* Chevrolat, 1836.

### Tribe Nesiobiini

Alonso-Zarazaga and Lyal, 1999

Nesiotinae Voss, 1972: 338 [stem: *Nesiot-*]. Type genus: *Nesiotes* Wollaston, 1861 [preoccupied genus name, not *Nesiotes* Martens, 1860 [Mollusca]; syn. of *Nesiobius* G. A. K. Marshall, 1943]. Comment: permanently invalid (Art. 39): based on preoccupied type genus.
Nesiobiini Alonso-Zarazaga and Lyal, 1999: 119 [stem: *Nesiobi-*]. Type genus: *Nesiobius* G. A. K. Marshall, 1943. Comment: replacement name for Nesiotinae Voss, 1872 because of the homonymy of the type genus.

### Tribe Neumatorini

Folwaczny, 1973

Neumatorini Folwaczny, 1973: 69, in key [stem: *Neumator-*]. Type genus: *Neumatora* Normand, 1920.

### Tribe Onychiini

Chapuis, 1869

Onychiidae Chapuis, 1869: 48 [stem: *Onychi-*]. Type genus: *Onychius* Chapuis, 1869. Comment: see Alonso-Zarazaga and Lyal (2009: 9) for comments on placement.

### Tribe Onycholipini

Wollaston, 1873

Onycholipides Wollaston, 1873: 454 [stem: *Onycholip-*]. Type genus: *Onycholips* Wollaston, 1861.
Stenoscelides Wollaston, 1877: 82, in key [stem: *Stenoscelid-*]. Type genus: *Stenoscelis* Wollaston, 1861.
Stereocorynina Voss, 1955b: 232 [stem: *Stereocoryn-*]. Type genus: *Stereocorynes* Wollaston, 1873.
Stereocorynini Roudier, 1958: 212 [stem: *Stereocoryn-*]. Type genus: *Stereocorynes* Wollaston, 1873. Comment: family-group name proposed as new without reference to Stereocorynina Voss, 1955.

### Tribe Pentarthrini

Lacordaire, 1865

Pentarthrides Lacordaire, 1865: 323 [stem: *Pentarthr-*]. Type genus: *Pentarthrum* Wollaston, 1854. Comment: original vernacular name available (Art. 11.7.2): first used in latinized form by Heyne and Taschenberg (1907: 234, as Pentarthrini), generally accepted as in Alonso-Zarazaga and Lyal (1999: 120, as Pentarthrini).

### Tribe Proecini

Voss, 1956

Proecina Voss, 1956a: 139 [stem: *Proec-*]. Type genus: *Proeces* Schönherr, 1837.
Phylloplatypodini Kato, 1998: 77 [stem: *Phylloplatypod-*]. Type genus: *Phylloplatypus* Kato, 1998.

### Tribe Pseudapotrepini

Champion, 1909

Pseudapotrepides Champion, 1909: 21 [stem: *Pseudapotrep-*]. Type genus: *Pseudapotrepus* Champion, 1909.

### Tribe Rhyncolini

Gistel, 1848

Rhynchalidae Gistel, 1848: [6] [stem: *Rhyncol-*]. Type genus: *Rhyncolus* Germar, 1817 [as *Rhyncholus*, incorrect subsequent spelling of type genus name, not in prevailing usage; *Rhyncolus* Germar, 1817 placed on the Official List of Generic Names in Zoology (ICZN 1991b)].

### Subtribe Phloeophagina

Voss, 1955

Phloeophagina Voss, 1955b: 228 [stem: *Phloeophag-*]. Type genus: *Phloeophagus* Schönherr, 1837.

### Subtribe Pseudomimina

Voss, 1939

Pseudomimini Voss, 1939: 65 [stem: *Pseudomim-*]. Type genus: *Pseudomimus* Hartmann, 1904.

### Subtribe Rhyncolina

Gistel, 1848

Rhynchalidae Gistel, 1848: [6] [stem: *Rhyncol-*]. Type genus: *Rhyncolus* Germar, 1817 [as *Rhyncholus*, incorrect subsequent spelling of type genus name, not in prevailing usage; *Rhyncolus* Germar, 1817 placed on the Official List of Generic Names in Zoology (ICZN 1991b)]. Comment: incorrect original stem formation, not in prevailing usage.
Eremotini Voss, 1939: 65 [stem: *Eremot-*]. Type genus: *Eremotes* Wollaston, 1861.
Cotasterosomini Konishi, 1962: 2 [stem: *Cotasterosomat-*]. Type genus: *Cotasterosoma* Konishi, 1962. Comment: incorrect original stem formation, not in prevailing usage.
Himatinini Konishi, 1962: 7 [stem: *Himatin-*]. Type genus: *Himatinum* Cockerell, 1906 [syn. of *Himatium* Wollaston, 1873].

### Tribe Tapiromimini

Voss, 1972

Tapiromimini Voss, 1972: 380 [stem: *Tapiromim-*]. Type genus: *Tapiromimus* Wollaston, 1877.

### Subfamily Cryptorhynchinae

Schönherr, 1825

Cryptorhynchides Schönherr, 1825: column 585 [stem: *Cryptorhynch-*]. Type genus: *Cryptorhynchus* Illiger, 1807 [placed on the Official List of Generic Names in Zoology (ICZN 1967b)]. Comment: Cryptorhynchinae Schönherr, 1825 placed on the Official List of Family-Group Names in Zoology (ICZN 1967b).

### Tribe Aedemonini

Faust, 1898

Acdemonini Faust, 1898: 34 [stem: *Aedemon-*]. Type genus: *Aedemonus* Schönherr, 1837 [as *Acdemonus*, incorrect subsequent spelling of type genus name, not in prevailing usage]. Comment: incorrect original stem formation, not in prevailing usage.
Mecistocerini Morimoto, 1978b: 122 [stem: *Mechistocer-*]. Type genus: *Mechistocerus* Fauvel, 1863 [as *Mecistocerus*, unjustified emendation of type genus name by Pascoe (1870b), not in prevailing usage]. Comment: incorrect original stem formation, not in prevailing usage; correction of stem by Morimoto (1987).
Derbyiellina Zimmerman, 1994a: 645, in key [stem: *Derbyiell-*]. Type genus: *Derbyiella* Lea, 1907.

### Tribe Camptorhinini

Lacordaire, 1865

Camptorhinides Lacordaire, 1865: 86 [stem: *Camptorhin-*]. Type genus: *Camptorhinus* Schönherr, 1825. Comment: original vernacular name available (Art. 11.7.2): first used in latinized form by Pascoe (1885: 254, as Camptorhininae), generally accepted as in Alonso-Zarazaga and Lyal (1999: 136, as Camptorhinini).

### Tribe Cryptorhynchini

Schönherr, 1825

Cryptorhynchides Schönherr, 1825: column 585 [stem: *Cryptorhynch-*]. Type genus: *Cryptorhynchus* Illiger, 1807 [placed on the Official List of Generic Names in Zoology (ICZN 1967b)]. Comment: Cryptorhynchinae Schönherr, 1825 placed on the Official List of Family-Group Names in Zoology (ICZN 1967b).

### Subtribe Cryptorhynchina

Schönherr, 1825

Cryptorhynchides Schönherr, 1825: column 585 [stem: *Cryptorhynch-*]. Type genus: *Cryptorhynchus* Illiger, 1807 [placed on the Official List of Generic Names in Zoology (ICZN 1967b)]. Comment: Cryptorhynchinae Schönherr, 1825 placed on the Official List of Family-Group Names in Zoology (ICZN 1967b).
Cryptorhynchidiini Bradley, 1930: 277, in key [stem: *Cryptorhynchidi-*]. Type genus: *Cryptorhynchidius* Pierce, 1919 [syn. of *Cryptorhynchus* Illiger, 1807].

### Subtribe Mecistostylina

Lacordaire, 1865

Mécistostylides Lacordaire, 1865: 131 [stem: *Mecistostyl-*]. Type genus: *Mecistostylus* Lacordaire, 1865. Comment: original vernacular name available (Art. 11.7.2): first used in latinized form by G. A. K. Marshall (1916: 10, as Mecistostylini), generally accepted as in Alonso-Zarazaga and Lyal (1999: 129, as Mecistostylina).
Cylindrocosyninae Pascoe, 1875: 55 [stem: *Cylindrocoryn-*]. Type genus: *Cylindrocorynus* Schönherr, 1837. Comment: incorrect original stem formation, not in prevailing usage.

### Subtribe Tylodina

Lacordaire, 1865

Tylodides Lacordaire, 1865: 90 [stem: *Tylod-*]. Type genus: *Tylodes* Sahlberg, 1823. Comment: original vernacular name available (Art. 11.7.2): first used in latinized form by Pascoe (1885: 255, as Tylodinae), generally accepted as in Alonso-Zarazaga and Lyal (1999: 129, as Tylodina).

### Tribe Gasterocercini

Zherikhin, 1991

Gasterocercini Zherikhin, 1991: 92 [stem: *Gasterocerc-*]. Type genus: *Gasterocercus* Laporte and Brullé, 1828.

### Tribe Psepholacini

Lacordaire, 1865

Psépholacides Lacordaire, 1865: 72 [stem: *Psepholac-*]. Type genus: *Psepholax* Lacordaire, 1865. Comment: original vernacular name available (Art. 11.7.2): first used in latinized form by Hustache (1936: 56, as Psepholacina), generally accepted as in Alonso-Zarazaga and Lyal (1999: 136, as Psepholacini); First Reviser (Psepholacini Lacordaire, 1865 vs Strongylopterini Lacordaire, 1865 vs Sympiezoscelini Lacordaire, 1865) not determined, current usage maintained.
Strongyloptérides Lacordaire, 1865: 73 [stem: *Strongylopter-*]. Type genus: *Strongylopterus* Schönherr, 1837. Comment: original vernacular name available (Art. 11.7.2): first used in latinized form and generally accepted as in Hustache (1936: 60, as Strongylopteridina [incorrect stem formation]).
Sympiézoscélides Lacordaire, 1865: 138 [stem: *Sympiezoscel-*]. Type genus: *Sympiezoscelus* G. R. Waterhouse, 1853. Comment: original vernacular name available (Art. 11.7.2): first used in latinized form and generally accepted as in Pascoe (1874: 96, as Sympiezoscelides [treated as Latin]).

### Tribe Sophrorhinini

Lacordaire, 1865

Sophrorhinides Lacordaire, 1865: 81 [stem: *Sophrorhin-*]. Type genus: *Sophrorhinus* Rouzet, 1855. Comment: original vernacular name available (Art. 11.7.2): first used in latinized form by C. O. Waterhouse (1879a: 310, as Sophororhinae [incorrect stem formation]), generally accepted as in Alonso-Zarazaga and Lyal (1999: 137, as Sophororhinini).

### Tribe Torneumatini

Bedel, 1884

Torneumatini Bedel, 1884b: 136, in note [stem: *Torneumat-*]. Type genus: *Torneuma* Wollaston, 1860.

### Subfamily Cyclominae

Schönherr, 1826

Cyclomides Schönherr, 1826: 185 [stem: *Cyclom-*]. Type genus: *Cyclomus* Schönherr, 1826 [syn. of *Epicthonius* Schönherr, 1826]. Comment: the classification of this subfamily follows Oberprieler (2010).

### Tribe Amycterini

Waterhouse, 1854

Amycteridae G. R. Waterhouse, 1854: 75, in note [stem: *Amycter-*]. Type genus: *Amycterus* Schönherr, 1823.
Euomides Lacordaire, 1863: 315 [stem: *Euom-*]. Type genus: *Euomus* Schönherr, 1847. Comment: original vernacular name available (Art. 11.7.2): first used in latinized form by W. J. MacLeay (1866: 319, as Euomidae), generally accepted as in Schenkling and G. A. K. Marshall (1931a: 27, as Euomini).
Psaliduridae Pierce, 1914: 350 [stem: *Phalidur-*]. Type genus: *Phalidura* Fischer von Waldheim, 1823 [as *Psalidura*, incorrect subsequent spelling of type genus name, not in prevailing usage; syn. of *Amycterus* Schönherr, 1823]. Comment: incorrect original stem formation, not in prevailing usage.
*Acantholophini Schenkling and G. A. K. Marshall, 1931a: Amyct. 6 [stem: *Acantholoph-*]. Type genus: *Acantholophus* Boisduval, 1835. Comment: unavailable family-group name, proposed after 1930 without description or bibliographic reference to such a description (Art. 13.1).

### Tribe Aterpini

Lacordaire, 1863

Aterpides Lacordaire, 1863: 410 [stem: *Aterp-*]. Type genus: *Aterpus* Schönherr, 1826 [syn. of *Aades* Schönherr, 1823]. Comment: precedence (Aterpini Lacordaire, 1863 vs Rhadinosomini Lacordaire, 1863) given to taxon originally proposed at the higher rank (Art. 24.1).

### Subtribe Aterpina

Lacordaire, 1863 nomen protectum

Heliomeneidae Gistel, 1848: [8] [stem: *Heliomen-*]. Type genus: *Heliomene* Gistel, 1848 [syn. of *Chrysolopus* Germar, 1817]. Comment: as pointed out by Alonso-Zarazaga and Lyal (2002: 22) this is the oldest available name for the subtribe name; Heliomeneini Gistel, 1848 was recently considered a *nomen oblitum* by Colonnelli (2003: 7) however the necessary supporting references were not provided; we hereby consider Heliomeneini Gistel, 1848 as a *nomen oblitum* (see supporting references in Appendix 1); incorrect original stem formation, not in prevailing usage.
Aterpides Lacordaire, 1863: 410 [stem: *Aterp-*]. Type genus: *Aterpus* Schönherr, 1826 [syn. of *Aades* Schönherr, 1823]. Comment: *nomen protectum* (see Appendix 1); original vernacular name available (Art. 11.7.2): first used in latinized form by Pascoe (1870b: 436, as Aterpinae), generally accepted as in Alonso-Zarazaga and Lyal (1999: 140, as Aterpini).
*Pélororhinides Lacordaire, 1863: 415 [stem: *Pelororhin-*]. Type genus: *Pelororhinus* Schönherr, 1834. Comment: original vernacular name unavailable (Art. 11.7.2): subsequently used in latinized form, e.g., Heyne and Taschenberg (1907: 228, as Pelororhinini), Morrone (1997: 101, as Pelorhirhini [incorrect stem formation]), but not generally accepted as valid; Pelorhinidae [incorrect stem formation] was used as valid by Ienistea (1986: 33) but it was not attributed to Lacordaire (1863); Ienistea’s name is also unavailable, it was proposed after 1930 without description or bibliographic reference to such a description (Art. 13.1).
Lophotides Jekel, 1865: 546 [stem: *Lophot-*]. Type genus: *Lophotus* Schönherr, 1834 [preoccupied genus name, not *Lophotus* Giorna, 1809 [Pisces]; syn. of *Aegorhinus* Erichson, 1834]. Comment: permanently invalid (Art. 39): based on preoccupied type genus.
Rhinarides Jekel, 1865: 546 [stem: *Rhinari-*]. Type genus: *Rhinaria* Kirby, 1819. Comment: incorrect original stem formation, not in prevailing usage.

### Subtribe Rhadinosomina

Lacordaire, 1863

Rhadinosomides Lacordaire, 1863: 63 [stem: *Rhadinosom-*]. Type genus: *Rhadinosomus* Schönherr, 1840. Comment: original vernacular name available (Art. 11.7.2): first used in latinized form by Pascoe (1875: 55, as Rhadinosominae), generally accepted as in Alonso-Zarazaga and Lyal (1999: 141, as Rhadinosomina).
Rhadinosomini Pierce, 1913: 405, in key [stem: *Rhadinosom-*]. Type genus: *Rhadinosomus* Schönherr, 1840. Comment: family-group name proposed as new without reference to Rhadinosomides Lacordaire, 1863.

### Tribe Cyclomini

Schönherr, 1826

Cyclomides Schönherr, 1826: 185 [stem: *Cyclom-*]. Type genus: *Cyclomus* Schönherr, 1826 [syn. of *Epicthonius* Schönherr, 1826].
Somatodides Lacordaire, 1863: 319 [stem: *Somatod-*]. Type genus: *Somatodes* Schönherr, 1840 [placed on the Official List of Generic Names in Zoology (ICZN 1994c)]. Comment: original vernacular name available (Art. 11.7.2): first used in latinized form by Pascoe (1870b: 436, as Somatodinae), generally accepted as in Alonso-Zarazaga and Lyal (1999: 144, as Somatodini); Somatodinae Lacordaire, 1863 placed on the Official List of Family-Group Names in Zoology (ICZN 1994c).

### Tribe Dichotrachelini

Hoffmann, 1957

Dichotrachelini Hoffmann, 1957a: 60, in note [stem: *Dichotrachel-*]. Type genus: *Dichotrachelus* Stierlin, 1853.

### Tribe Hipporhinini

Lacordaire, 1863

Hipporhinides Lacordaire, 1863: 323 [stem: *Hipporhin-*]. Type genus: *Hipporhinus* Schönherr, 1823. Comment: original vernacular name available (Art. 11.7.2): first used in latinized form and generally accepted as in Pascoe (1870b: 436, as Hipporhininae).
Gronopini Bedel, 1884a: 68, in key [stem: *Gronop-*]. Type genus: *Gronops* Schönherr, 1823.
Gronopina Louw and Oberprieler, 1998: 24 [stem: *Gronop-*]. Type genus: *Gronops* Schönherr, 1823. Comment: family-group name proposed as new without reference to Gronopini Bedel, 1884.

### Tribe Listroderini

LeConte, 1876

Listroderi J. L. LeConte, 1876: 124 [stem: *Listroder-*]. Type genus: *Listroderes* Schönherr, 1826.
Palaechtini C. Brinck, 1948: 43 [stem: *Palaechth-*]. Type genus: *Palaechthus* C. O. Waterhouse, 1884 [as *Palaechtus*, incorrect subsequent spelling of type genus name, not in prevailing usage]. Comment: incorrect original stem formation, not in prevailing usage.

### Tribe Notiomimetini

Wollaston, 1873

Notiomimetides Wollaston, 1873: 440 [stem: *Notiomimet-*]. Type genus: *Notiomimetes* Wollaston, 1873.
Apheli Csiki, 1936: 109 [stem: *Aphel-*]. Type genus: *Aphela* Pascoe, 1865. Comment: description by indication (distinguishing characters given in Wollaston (1873: 440)).

### Tribe Rhythirrinini

Lacordaire, 1863

Rhytirhinides Lacordaire, 1863: 296 [stem: *Rhythirrin-*]. Type genus: *Rhythirrinus* Schönherr, 1823 [as *Rhytirhinus*, incorrect subsequent spelling of type genus name, not in prevailing usage]. Comment: original vernacular name available (Art. 11.7.2): first used in latinized form by Seidlitz (1875 [Gatt.]: 112, as Rhytirhinini [incorrect stem formation]), generally accepted as in Alonso-Zarazaga and Lyal (1999: 141, as Rhythirrinini); incorrect original stem formation, not in prevailing usage; First Reviser found (Rhythirrinini Lacordaire, 1863 vs Rhyparosomini Lacordaire, 1863 vs Eupagini Lacordaire, 1863) is Kuschel (1971: 251).
Rhyparosomides Lacordaire, 1863: 327 [stem: *Rhyparosom-*]. Type genus: *Rhyparosomus* Schönherr, 1842. Comment: original vernacular name available (Art. 11.7.2): first used in latinized form by Stein (1868: 100, as Rhyparosomini), generally accepted as in Heyne and Taschenberg (1907: 228, as Rhyparosomini).
Eupagides Lacordaire, 1863: 328 [stem: *Eupag-*]. Type genus: *Eupages* Schönherr, 1834. Comment: original vernacular name available (Art. 11.7.2): first used in latinized form by Péringuey (1888: 168, as Eupagidae), generally accepted as in Schenkling and G. A. K. Marshall (1929: 47, as Eupagini).
Rhytidorhinides Wollaston, 1865: 307 [stem: *Rhytidorhin-*]. Type genus: *Rhytidorhinus* Wollaston, 1864 [preoccupied genus name, not *Rhytidorhinus* Agassiz, 1846 [Coleoptera: Curculionidae: Cyclominae]; syn. of *Rhythirrinus* Schönherr, 1823]. Comment: permanently invalid (Art. 39): based on preoccupied type genus.

### Subfamily Entiminae

Schönherr, 1823

Entimides Schönherr, 1823: column 1138 [stem: *Entim-*]. Type genus: *Entimus* Germar, 1817. Comment: First Revisers found (Entiminae Schönherr, 1823 vs Lordopinae Schönherr, 1823 vs Polydrusinae Schönherr, 1823) are Alonso-Zarazaga and Lyal (1999: 144).

### Tribe Agraphini

Horn, 1876

Agraphi G. H. Horn, 1876: 58 [stem: *Agraph-*]. Type genus: *Agraphus* Say, 1831.

### Tribe Alophini

LeConte, 1874

Alophini J. L. LeConte, 1874b: 461 [stem: *Aloph-*]. Type genus: *Alophus* Schönherr, 1826 [syn. of *Graptus* Schönherr, 1823].

### Tribe Anomophthalmini

Morrone, 1998

Anomophthalmina Morrone, 1998: 86 [stem: *Anomophthalm-*]. Type genus: *Anomophthalmus* Fairmaire, 1884.

### Tribe Anypotactini

Champion, 1911

Anypotactina Champion, 1911: 215 [stem: *Anypotact-*]. Type genus: *Anypotactus* Schönherr, 1840.

### Tribe Blosyrini

Lacordaire, 1863

Blosyrides Lacordaire, 1863: 27 [stem: *Blosyr-*]. Type genus: *Blosyrus* Schönherr, 1823. Comment: original vernacular name available (Art. 11.7.2): first used in latinized form by Péringuey (1888: 144, as Blosyridae), generally accepted as in Alonso-Zarazaga and Lyal (1999: 146, as Blosyrini).

### Tribe Brachyderini

Schönherr, 1826

Brachyderides Schönherr, 1826: 94 [stem: *Brachyder-*]. Type genus: *Brachyderes* Schönherr, 1823 [placed on the Official List of Generic Names in Zoology (ICZN 1987a)]. Comment: name placed on the Official List of Family-Group Names in Zoology (ICZN 1987a, as Brachyderinae Schönherr, 1837).
Thylacitidae Kirby, 1837: 202 [stem: *Thylacit-*]. Type genus: *Thylacites* Germar, 1817 [placed on the Official Index of Invalid and Rejected Generic Names in Zoology (ICZN 1987a)]. Comment: placed on the Official Index of Rejected and Invalid Family-Group Names in Zoology (ICZN 1987a).
Strophosomidae Gistel, 1848: [8] [stem: *Strophosom-*]. Type genus: *Strophosomum* Gistel, 1856 [syn. of *Strophosoma* Billberg, 1820].

### Tribe Celeuthetini

Lacordaire, 1863

Céleuthétides Lacordaire, 1863: 145 [stem: *Celeuthet-*]. Type genus: *Celeuthetes* Schönherr, 1842.

### Subtribe Celeuthetina

Lacordaire, 1863

Céleuthétides Lacordaire, 1863: 145 [stem: *Celeuthet-*]. Type genus: *Celeuthetes* Schönherr, 1842. Comment: original vernacular name available (Art. 11.7.2): first used in latinized form by Faust (1891: 166, as Celeuthetinae), generally accepted as in Alonso-Zarazaga and Lyal (1999: 147, as Celeuthetini).
Coptorrhynchina Voss, 1940: 279 [stem: *Coptorhynch-*]. Type genus: *Coptorhynchus* sensu Faust, 1897 [not *Coptorhynchus* Guérin-Méneville, 1841; syn. of *Resites* Alonso-Zarazaga and Lyal, 1999]. Comment: incorrect original stem formation, not in prevailing usage; based on a misidentified type genus.

### Subtribe Isopterina

Morimoto and Kojima, 2001

Isopterina Morimoto and Kojima, 2001: 274 [stem: *Isopter-*]. Type genus: *Isopterus* Faust, 1895.

### Tribe Cneorhinini

Lacordaire, 1863

Cnéorhinides Lacordaire, 1863: 31 [stem: *Cneorhin-*]. Type genus: *Cneorhinus* Schönherr, 1823. Comment: original vernacular name available (Art. 11.7.2): first used in latinized form by Seidlitz (1875 [Gatt.]: 111, as Cneorhinini), generally accepted as in Alonso-Zarazaga and Lyal (1999: 148, as Cneorhinini).
Philopedini Bedel, 1883: 32, in key [stem: *Philoped-*]. Type genus: *Philopedon* Schönherr, 1826.
Dermatodini Emden, 1936: 76, in key [stem: *Dermatod-*]. Type genus: *Dermatodes* Schönherr, 1840.
Stigmatrachelini Richard, 1983: 8 [stem: *Stigmatrachel-*]. Type genus: *Stigmatrachelus* Schönherr, 1840.

### Tribe Cratopodini

Hustache, 1919

Cratopini Hustache, 1919: 473 [stem: *Cratopod-*]. Type genus: *Cratopus* Schönherr, 1823. Comment: incorrect original stem formation, not in prevailing usage.

### Tribe Cylydrorhinini

Lacordaire, 1863

Cylindrorhinides Lacordaire, 1863: 339 [stem: *Cylydrorhin-*]. Type genus: *Cylydrorhinus* Guérin-Méneville, 1838 [as *Cylindrorhinus*, incorrect subsequent spelling of type genus name, not in prevailing usage]. Comment: original vernacular name available (Art. 11.7.2): first used in latinized form by Pascoe (1870b: 436, as Cylindrorhininae [incorrect stem formation]), generally accepted as in Alonso-Zarazaga and Lyal (1999: 150, as Cylydrorhinini); incorrect original stem formation, not in prevailing usage.

### Tribe Cyphicerini

Lacordaire, 1863

Cyphicérides Lacordaire, 1863: 220 [stem: *Cyphicer-*]. Type genus: *Cyphicerus* Schönherr, 1823. Comment: First Reviser (Cyphicerini Lacordaire, 1863 vs Phytoscaphini Lacordaire, 1863) not determined, current usage maintained.

### Subtribe Acanthotrachelina

Marshall, 1944

Acanthotrachelini G. A. K. Marshall, 1944: 76, in key [stem: *Acanthotrachel-*]. Type genus: *Acanthotrachelus* Schönherr, 1842.

### Subtribe Cyphicerina

Lacordaire, 1863

Cyphicérides Lacordaire, 1863: 220 [stem: *Cyphicer-*]. Type genus: *Cyphicerus* Schönherr, 1823. Comment: original vernacular name available (Art. 11.7.2): first used in latinized form by Faust (1888: 284, as Cyphicerinae), generally accepted as in Alonso-Zarazaga and Lyal (1999: 151, as Cyphicerini).
Corigetini Faust, 1885: 167, in key [stem: *Coriget-*]. Type genus: *Corigetus* Desbrochers des Loges, 1872.

### Subtribe Mylacorrhinina

Reitter, 1913

Mylacorrhynchina Reitter, 1913b: 12, in key [stem: *Mylacorrhin-*]. Type genus: *Mylacorrhina* Reitter, 1913 [syn. of *Altonomus* Desbrochers des Loges, 1907]. Comment: incorrect original stem formation, not in prevailing usage.

### Subtribe Myllocerina

Pierce, 1913

Myllocerini Pierce, 1913: 421, in key [stem: *Myllocer-*]. Type genus: *Myllocerus* Schönherr, 1826.
Ptochini Reitter, 1913b: 8, in key [stem: *Ptoch-*]. Type genus: *Ptochus* Schönherr, 1826 [placed on the Official List of Generic Names in Zoology (ICZN 1990d)].

### Subtribe Phytoscaphina

Lacordaire, 1863

Phytoscaphides Lacordaire, 1863: 229 [stem: *Phytoscaph-*]. Type genus: *Phytoscaphus* Schönherr, 1826. Comment: original vernacular name available (Art. 11.7.2): first used in latinized form by J. L. LeConte (1869: 382, as Phytoscaphi), generally accepted as in Alonso-Zarazaga and Lyal (1999: 154, as Phytoscaphina).
*Oxyophthalmina Voss, 1933a: 30 [stem: *Oxyophthalm-*]. Type genus: *Oxyophthalmus* Hochhuth, 1847. Comment: unavailable family-group name, proposed after 1930 without description or bibliographic reference to such a description (Art. 13.1).

### Tribe Ectemnorhinini

Lacordaire, 1863

Ectemnorhinides Lacordaire, 1863: 562 [stem: *Ectemnorhin-*]. Type genus: *Ectemnorhinus* G. R. Waterhouse, 1853. Comment: original vernacular name available (Art. 11.7.2): first used in latinized form by Pascoe (1870b: 436, as Ectemnorhinae), generally accepted as in Alonso-Zarazaga and Lyal (1999: 155, as Ectemnorhinini).
Canonopsini Dreux and Voisin, 1989: 112 [stem: *Canonopse-*]. Type genus: *Canonopsis* C. O. Waterhouse, 1875. Comment: incorrect original stem formation, not in prevailing usage.

### Tribe Elytrurini

Marshall, 1956

Elytrurini G. A. K. Marshall, 1956: 5 [stem: *Elytrur-*]. Type genus: *Elytrurus* Boisduval, 1835.

### Tribe Embrithini

Marshall, 1942

Embrithini G. A. K. Marshall, 1942: 3 [stem: *Embrith-*]. Type genus: *Embrithes* Schönherr, 1842.

### Tribe Entimini

Schönherr, 1823

Entimides Schönherr, 1823: column 1138 [stem: *Entim-*]. Type genus: *Entimus* Germar, 1817.

### Tribe Episomini

Lacordaire, 1863

Episomides Lacordaire, 1863: 175 [stem: *Episom-*]. Type genus: *Episomus* Schönherr, 1823. Comment: original vernacular name available (Art. 11.7.2): first used in latinized form by Faust (1894: 185, as Episomini), generally accepted as in Alonso-Zarazaga and Lyal (1999: 156, as Episomini).
Episomini Pierce, 1913: 421, in key [stem: *Episom-*]. Type genus: *Episomus* Schönherr, 1823. Comment: family-group name proposed as new without reference to Episomides Lacordaire, 1863.

### Tribe Eudiagogini

LeConte, 1874

Promécopides Lacordaire, 1863: 384 [stem: *Promecop-*]. Type genus: *Promecops* Schönherr, 1823 [preoccupied genus name, not *Promecops* Sahlberg, 1823 [Coleoptera: Curculionidae: Entiminae: Eudiagogini]; syn. of *Promecops* Sahlberg, 1823]. Comment: original vernacular name available (Art. 11.7.2): first used in latinized form and generally accepted as in Pascoe (1870b: 436, as Promecopinae); permanently invalid (Art. 39): based on preoccupied type genus.
Eudiagogini J. L. LeConte, 1874b: 454 [stem: *Eudiagog-*]. Type genus: *Eudiagogus* Schönherr, 1840. Comment: First Reviser (Eudiagogini J. L. LeConte, 1874 vs Bathyrini J. L. LeConte, 1874) not determined, current usage maintained.
Bathyrini J. L. LeConte, 1874b: 461 [stem: *Bathyrin-*]. Type genus: *Bathyris* J. L. LeConte, 1874 [syn. of *Colecerus* Schönherr, 1840]. Comment: incorrect original stem formation, not in prevailing usage.
Coleocerini Jekel, 1875: 144 [stem: *Colecer-*]. Type genus: *Colecerus* Schönherr, 1840 [as *Coleocerus*, incorrect subsequent spelling of type genus name, not in prevailing usage]. Comment: incorrect original stem formation, not in prevailing usage.

### Tribe Eupholini

Günther, 1943

*Eupholini Schenkling and G. A. K. Marshall, 1931b: Lept. 62 [stem: *Euphol-*]. Type genus: *Eupholus* Guérin-Méneville, 1838 [preoccupied genus name, not *Eupholus* Boisduval, 1835 [Coleoptera: Curculionidae: Entiminae]; syn. of *Eupholus* Boisduval, 1835]. Comment: unavailable family-group name, proposed after 1930 without description or bibliographic reference to such a description (Art. 13.1).
Eupholini Günther, 1943: 19 [stem: *Euphol-*]. Type genus: *Eupholus* Guérin-Méneville, 1838 [preoccupied genus name, not *Eupholus* Boisduval, 1835 [Coleoptera: Curculionidae: Entiminae]; syn. of *Eupholus* Boisduval, 1835]. Comment: although this name should be treated as permanently invalid because it is based on a preoccupied type genus (Art. 39), an application will be submitted to the Commission by MAAZ and CHCL to conserve Eupholini Günther, 1943 and designate *Eupholus* Boisduval, 1835 as its type genus.
Eupholini Alonso-Zarazaga and Lyal, 1999: 157 [stem: *Euphol-*]. Type genus: *Eupholus* Boisduval, 1835. Comment: proposed as a new taxon; junior homonym of Eupholini Günther, 1943.

### Tribe Eustylini

Lacordaire, 1863

Eustylides Lacordaire, 1863: 205 [stem: *Eustyl-*]. Type genus: *Eustylus* Schönherr, 1842. Comment: original vernacular name available (Art. 11.7.2): first used in latinized form by Faust (1886b: 338, as Eustylinorum), generally accepted as in Alonso-Zarazaga and Lyal (1999: 157, as Eustylini).
Exophthalmini G. H. Horn, 1876: 100 [stem: *Exophthalm-*]. Type genus: *Exophthalmus* Schönherr, 1823.
Eustylini Pierce, 1913: 421, in key [stem: *Eustyl-*]. Type genus: *Eustylus* Schönherr, 1842. Comment: family-group name proposed as new without reference to Eustylides Lacordaire, 1863.
Compsi Pierce, 1913: 406, in key [stem: *Comps-*]. Type genus: *Compsus* Schönherr, 1823. Comment: the younger name Compsina Martins and Galileo, 2007 (type genus *Compsa* Perty, 1832) in Cerambycidae is available; this case is to be referred to the Commission to remove the homonymy (Art. 55.3.1).
Exophthalmodina Voss, 1954: 199, in key [stem: *Exophthalmod-*]. Type genus: *Exophthalmodes* Pierce, 1916 [syn. of *Exophthalmus* Schönherr, 1823].

### Tribe Geonemini

Gistel, 1848

Geonemidae Gistel, 1848: [8] [stem: *Geonem-*]. Type genus: *Geonemus* Schönherr, 1833 [placed on the Official List of Generic Names in Zoology (ICZN 1988d)].
Barynotides Lacordaire, 1863: 37 [stem: *Barynot-*]. Type genus: *Barynotus* Germar, 1817. Comment: original vernacular name available (Art. 11.7.2): first used in latinized form by G. H. Horn (1876: 22, as Barynoti), generally accepted as in Dalla Torre et al. (1936: 32, as Barynotini).
Trigonoscutae J. L. LeConte, 1874b: 456 [stem: *Trigonoscut-*]. Type genus: *Trigonoscuta* Motschulsky, 1853.
Epicaeri G. H. Horn, 1876: 18 [stem: *Epicaer-*]. Type genus: *Epicaerus* Schönherr, 1834.
Calyptilli G. H. Horn, 1876: 26 [stem: *Calyptill-*]. Type genus: *Calyptillus* G. H. Horn, 1876.
Omilei G. H. Horn, 1876: 101 [stem: *Omile-*]. Type genus: *Omileus* G. H. Horn, 1876.
Trigonoscutini Pierce, 1913: 405, in key [stem: *Trigonoscut-*]. Type genus: *Trigonoscuta* Motschulsky, 1853. Comment: family-group name proposed as new without reference to Trigonoscutae J. L. LeConte, 1874.
Menoetiini Pierce, 1913: 373, in key [stem: *Menoeti-*]. Type genus: *Menoetius* Dejean, 1821 [placed on the Official Index of Rejected and Invalid Generic Names in Zoology (ICZN 1987f)]. Comment: permanently invalid (Art. 39): based on suppressed type genus.
Calyptillini Pierce, 1913: 405, in key [stem: *Calyptill-*]. Type genus: *Calyptillus* G. H. Horn, 1876. Comment: family-group name proposed as new without reference to Calyptilli G. H. Horn, 1876.

### Tribe Holcorhinini

Desbrochers des Loges, 1898

Holcorhinidae Desbrochers des Loges, 1898: 5 [stem: *Holcorhin-*]. Type genus: *Holcorhinus* Schönherr, 1826.
Cyclopterini Reitter, 1913b: 9, in key [stem: *Cyclopter-*]. Type genus: *Cyclopterus* Marseul, 1871 [preoccupied genus name, not *Cyclopterus* Linnaeus, 1758 [Pisces]; syn. of *Nucterocephalus* Desbrochers des Loges, 1897]. Comment: permanently invalid (Art. 39): based on preoccupied type genus; Cyclopteridae Bonaparte, 1831 (type genus *Cyclopterus* Linnaeus, 1758) is available in Pisces.

### Tribe Hormorini

Horn, 1876

Hormori G. H. Horn, 1876: 23 [stem: *Hormor-*]. Type genus: *Hormorus* G. H. Horn, 1876.

### Tribe Laparocerini

Lacordaire, 1863

Laparocérides Lacordaire, 1863: 196 [stem: *Laparocer-*]. Type genus: *Laparocerus* Schönherr, 1834. Comment: original vernacular name available (Art. 11.7.2): first used in latinized form by Heyne and Taschenberg (1907: 226, as Laparocerini), generally accepted as in Alonso-Zarazaga and Lyal (1999: 160, as Laparocerini).
Laparocerini Pierce, 1913: 421, in key [stem: *Laparocer-*]. Type genus: *Laparocerus* Schönherr, 1834. Comment: family-group name proposed as new without reference to Laparocérides Lacordaire, 1863.

### Tribe Leptostethini

Lacordaire, 1863

Leptostéthides Lacordaire, 1863: 258 [stem: *Leptosteth-*]. Type genus: *Leptostethus* G. R. Waterhouse, 1853. Comment: original vernacular name available (Art. 11.7.2): first used in latinized form by Heyne and Taschenberg (1907: 226, as Leptostethini), generally accepted as in Alonso-Zarazaga and Lyal (1999: 161, as Leptostethini).

### Tribe Lordopini

Schönherr, 1823

Lordopides Schönherr, 1823: column 1142 [stem: *Lordop-*]. Type genus: *Lordops* Schönherr, 1823.
Hypsonotidae Jekel, 1853: I.II.3 [stem: *Hypsonot-*]. Type genus: *Hypsonotus* Germar, 1824.
Alocorhini Jekel, 1856: 9bis, in key [stem: *Alocorhin-*]. Type genus: *Alocorhinus* Sahlberg, 1823.
Elytroxysi Jekel, 1856: 9bis, in key [stem: *Elytroxe-*]. Type genus: *Elytroxys* Jekel, 1856. Comment: incorrect original stem formation, not in prevailing usage.
Eurylobi Jekel, 1856: 9bis, in key [stem: *Eurylob-*]. Type genus: *Eurylobus* Schönherr, 1826.
Merodonti Jekel, 1856: 9bis, in key [stem: *Merodont-*]. Type genus: *Merodontus* Jekel, 1856.
Tomorhini Jekel, 1856: 9bis, in key [stem: *Tomorhin-*]. Type genus: *Tomorhinus* Jekel, 1856.

### Tribe Mesostylini

Reitter, 1913

Mesostylini Reitter, 1913b: 8, in key [stem: *Mesostyl-*]. Type genus: *Mesostylus* Faust, 1894.

### Tribe Myorhinini

Marseul, 1863

Myorhinidae Marseul, 1863: 223 [stem: *Myorhin-*]. Type genus: *Myorhinus* Schönherr, 1826 [syn. of *Apsis* Germar, 1820]. Comment: original vernacular name available (Art. 11.7.2): first used in latinized form by Kiesenwetter (1864: 265, as Myorhinidae), generally accepted as in Alonso-Zarazaga and Lyal (1999: 162, as Myorhinini).

### Tribe Nastini

Reitter, 1913

Nastini Reitter, 1913b: 9, in key [stem: *Nast-*]. Type genus: *Nastus* Schönherr, 1842.

### Tribe Naupactini

Gistel, 1848 nomen protectum

Iphiides Schönherr, 1823: column 1139 [stem: *Iphi-*]. Type genus: *Iphius* Schönherr, 1823 [syn. of *Alceis* Billberg, 1820]. Comment: *nomen oblitum* (see Appendix 1).
Naupactidae Gistel, 1848: [8] [stem: *Naupact-*]. Type genus: *Naupactus* Dejean, 1821. Comment: *nomen protectum* (see Appendix 1).
Cyphides Lacordaire, 1863: 107 [stem: *Cyph-*]. Type genus: *Cyphus* Germar, 1824 [syn. of *Cyrtomon* Schönherr, 1823]. Comment: original vernacular name available (Art. 11.7.2): first used in latinized form by J. L. LeConte (1874b: 458, as Cyphi), generally accepted as in J. L. LeConte and G. H. Horn (1883: 453, as Cyphi).
Macrostyles J. L. LeConte, 1874b: 457 [stem: *Macrostyl-*]. Type genus: *Macrostylus* Boheman, 1840.
Symmathetes J. L. LeConte, 1874b: 458 [stem: *Symmathet-*]. Type genus: *Symmathetes* Schönherr, 1847 [syn. of *Pantomorus* Schönherr, 1840].
Artipi G. H. Horn, 1876: 91 [stem: *Artipod-*]. Type genus: *Artipus* Schönherr, 1823 [preoccupied genus name, not *Artipus* Sahlberg, 1823 [Coleoptera: Curculionidae: Entiminae: Naupactini]; syn. of *Artipus* Sahlberg, 1823]. Comment: permanently invalid (Art. 39): based on preoccupied type genus.
Platyomina Champion, 1911: 282 [stem: *Platyom-*]. Type genus: *Platyomus* Schönherr, 1823 [preoccupied genus name, not *Platyomus* Sahlberg, 1823 [Coleoptera: Curculionidae: Entiminae: Naupactini]; syn. of *Platyomus* Sahlberg, 1823]. Comment: permanently invalid (Art. 39): based on preoccupied type genus.
Alceidini Pierce, 1913: 404, in key [stem: *Alceent-*]. Type genus: *Alceis* Billberg, 1820. Comment: incorrect original stem formation, not in prevailing usage.
Glaphyrometopi Pierce, 1913: 406, in key [stem: *Glaphyrometop-*]. Type genus: *Glaphyrometopus* Pierce, 1913.
Pseudocyphi Pierce, 1913: 406, in key [stem: *Pseudocyph-*]. Type genus: *Pseudocyphus* Schaeffer, 1905 [syn. of *Platyomus* Sahlberg, 1823].
Neocyphini Hustache, 1919: 476 [stem: *Neocyph-*]. Type genus: *Neocyphus* Bedel, 1883 [syn. of *Cyrtomon* Schönherr, 1823].
Platyomina Voss, 1954: 199, in key [stem: *Platyom-*]. Type genus: *Platyomus* Sahlberg, 1823. Comment: family-group name proposed as new without reference to Platyomina Champion, 1911.
Canephorotomina Voss, 1954: 206, in key [stem: *Canephorotom-*]. Type genus: *Canephorotomus* Voss, 1954 [syn. of *Amitrus* Schönherr, 1840].
Pantomorina Voss, 1954: 207, in key [stem: *Pantomor-*]. Type genus: *Pantomorus* Schönherr, 1840.
Plectrophorina Voss, 1954: 207, in key [stem: *Plectrophor-*]. Type genus: *Plectrophorus* Schönherr, 1826 [preoccupied genus name, not *Plectrophorus* Férussac, 1819 [Mollusca]; syn. of *Plectrophoroides* Wibmer and O’Brien, 1986]. Comment: permanently invalid (Art. 39): based on preoccupied type genus.

### Tribe Nothognathini

Marshall, 1916

Nothognathides G. A. K. Marshall, 1916: 204, in key [stem: *Nothognath-*]. Type genus: *Nothognathus* G. A. K. Marshall, 1916.

### Tribe Omiini

Shuckard, 1839

Omiadae Shuckard, 1839b: 60 [stem: *Omi-*]. Type genus: *Omias* Germar, 1817. Comment: senior homonym of Omiini Beck, 1996 in Lepidoptera (type genus *Omia* Hübner, 1821); this case is to be referred to the Commission to remove the homonymy (Art. 55.3.1).
Mylacini Reitter, 1913b: 9, in key [stem: *Mylac-*]. Type genus: *Mylacus* Boheman, 1843 [syn. of *Omias* Germar, 1817].

### Tribe Oosomini

Lacordaire, 1863

*Oosomides Schönherr, 1823: column 1145 [stem: *Oosom-*]. Type genus: *Oosomus* Schönherr, 1826. Comment: family-group name unavailable (Art. 11.7.1.1): not based on an available genus name at the time.
Oosomides Lacordaire, 1863: 164 [stem: *Oosom-*]. Type genus: *Oosomus* Schönherr, 1826. Comment: original vernacular name available (Art. 11.7.2): first used in latinized form by Kolbe (1891: 26, as Oosominae), generally accepted as in Alonso-Zarazaga and Lyal (1999: 166, as Oosomini).
Oosomini Pierce, 1913: 421, in key [stem: *Oosom-*]. Type genus: *Oosomus* Schönherr, 1826. Comment: family-group name proposed as new without reference to Oosomides Lacordaire, 1863.

### Tribe Ophryastini

Lacordaire, 1863

Ophryastides Lacordaire, 1863: 256 [stem: *Ophryast-*]. Type genus: *Ophryastes* Germar, 1829. Comment: original vernacular name available (Art. 11.7.2): first used in latinized form by J. L. LeConte (1874b: 454, as Ophryastini), generally accepted as in Alonso-Zarazaga and Lyal (1999: 167, as Ophryastini).

### Tribe Ophtalmorrhynchini

Hoffmann, 1965

Ophtalmorynchini Hoffmann, 1965: 1411 [stem: *Ophtalmorrhynch-*]. Type genus: *Ophtalmorrhynchus* Hoffmann, 1965. Comment: incorrect original stem formation, not in prevailing usage.

### Tribe Otiorhynchini

Schönherr, 1826

Loborhynchides Schönherr, 1823: column 1144 [stem: *Loborhynch-*]. Type genus: *Loborhynchus* Schönherr, 1823 [placed on the Official Index of Rejected and Invalid Generic Names in Zoology (ICZN 1972); see Lyal and Alonso-Zarazaga (2010) and Appendix 6; syn. of *Otiorhynchus* Germar, 1822]. Comment: Loborhynchinae Schönherr, 1823 placed on the Official Index of Rejected and Invalid Family-Group Names in Zoology (ICZN 1972).
Otiorhynchides Schönherr, 1826: 203 [stem: *Otiorhynch-*]. Type genus: *Otiorhynchus* Germar, 1822 [placed on the Official List of Generic Names in Zoology (ICZN 1972, as *Otiorhynchus* Germar, 1824); see Lyal and Alonso-Zarazaga (2010) and Appendix 6]. Comment: name placed on the Official List of Family-Group Names in Zoology (ICZN 1972, as Otiorhynchinae Schönherr, 1826).
Brachyrrhinidae Bedel, 1883: 30 [stem: *Brachyrhin-*]. Type genus: *Brachyrhinus* Latreille, 1802 [as *Brachyrrhinus*, incorrect original spelling of type genus name, not in prevailing usage; *Brachyrhinus* Latreille, 1802 placed on the Official Index of Rejected and Invalid Generic Names in Zoology (ICZN 1972); syn. of *Cryphiphorus* Stierlin, 1883]. Comment: name placed on the Official Index of Rejected and Invalid Family-Group Names in Zoology (ICZN 1972).

### Tribe Ottistirini

Heller, 1925

Ottistirini Heller, 1925a: 56, in key [stem: *Ottistir-*]. Type genus: *Ottistira* Pascoe, 1872.

### Tribe Pachyrhynchini

Schönherr, 1826

Somatodides Schönherr, 1823: column 1139 [stem: *Somatod-*]. Type genus: *Somatodes* Schönherr, 1823 [placed on the Official Index of Rejected and Invalid Generic Names in Zoology (ICZN 1994c); syn. of *Pachyrhynchus* Germar, 1824]. Comment: name placed on the Official Index of Rejected and Invalid Family-Group Names in Zoology as Somatodini Schönherr, 1823 (ICZN 1994c).
Pachyrhynchides Schönherr, 1826: 88 [stem: *Pachyrhynch-*]. Type genus: *Pachyrhynchus* Germar, 1824 [placed on the Official List of Generic Names in Zoology (ICZN 1970b)]. Comment: name placed on the Official List of Family-Group Names in Zoology as Pachyrhynchini Schönherr, 1826 (ICZN 1970b).

### Tribe Peritelini

Lacordaire, 1863

Péritélides Lacordaire, 1863: 178 [stem: *Peritel-*]. Type genus: *Peritelus* Germar, 1824. Comment: original vernacular name available (Art. 11.7.2): first used in latinized form by J. L. LeConte (1874b: 455, as Periteli), generally accepted as in Alonso-Zarazaga and Lyal (1999: 171, as Peritelini).
Simoini Pierce, 1913: 421, in key [stem: *Simon-*]. Type genus: *Simo* Dejean, 1821. Comment: incorrect original stem formation, not in prevailing usage.
Paraptochi Pierce, 1913: 423, in key [stem: *Paraptoch-*]. Type genus: *Paraptochus* Seidlitz, 1868.
Homorythmini Hoffmann, 1950: 154 [stem: *Homorhythm-*]. Type genus: *Homorhythmus* Bedel, 1883 [as *Homorythmus*, incorrect subsequent spelling of type genus name, not in prevailing usage; syn. of *Simo* Dejean, 1821]. Comment: incorrect original stem formation, not in prevailing usage.

### Tribe Phyllobiini

Schönherr, 1826

Phyllobides Schönherr, 1826: 178 [stem: *Phyllobi-*]. Type genus: *Phyllobius* Germar, 1824 [placed on the Official List of Generic Names in Zoology (ICZN 1981c)]. Comment: Phyllobiini Schönherr, 1826 placed on the Official List of Family-Group Names in Zoology (ICZN 1981c); incorrect original stem formation, not in prevailing usage.
Evotini J. L. LeConte, 1874b: 454 [stem: *Evot-*]. Type genus: *Evotus* J. L. LeConte, 1874.
Aphrasti J. L. LeConte, 1874b: 458 [stem: *Aphrast-*]. Type genus: *Aphrastus* Say, 1831.
Metacinopinae Reitter, 1913b: 6, in key [stem: *Metacinop-*]. Type genus: *Metacinops* Kraatz, 1862.

### Tribe Polycatini

Marshall, 1956

Polycatini G. A. K. Marshall, 1956: 4 [stem: *Polycat-*]. Type genus: *Polycatus* Heller, 1913.

### Tribe Polydrusini

Schönherr, 1823

Polydrosides Schönherr, 1823: column 1144 [stem: *Polydrus-*]. Type genus: *Polydrusus* Germar, 1817 [as *Polydrosus*, unjustified emendation of type genus name by Schönherr (1823), not in prevailing usage; syn. of *Polydrusus* Germar, 1817; *Polydrusus* Germar, 1817 placed on the Official List of Generic Names in Zoology (ICZN 1981c)]. Comment: placed on the Official List of Family-Group Names in Zoology (ICZN 1981c, as Polydrosini Schönherr, 1823), however, since this family-group name was based on an unjustified emendation of the type genus name, it was corrected (see Art. 32.5.3.2) to Polydrusini by Alonso-Zarazaga and Lyal (1999: 174).
Phyllomanisidae Gistel, 1848: [8] [stem: *Phylloman-*]. Type genus: *Phyllomanes* Gistel, 1848 [preoccupied genus name, not *Phyllomanes* Cabanis, 1847 [Aves]; syn. of *Polydrusus* Germar, 1817]. Comment: permanently invalid (Art. 39): based on preoccupied type genus; incorrect original stem formation, not in prevailing usage.
Liophloeidae Gistel, 1848: [8] [stem: *Liophloe-*]. Type genus: *Liophloeus* Germar, 1817.
Oligocyidae Gistel, 1856a: 373 [stem: *Oligoce-*]. Type genus: *Oligocys* Gistel, 1856 [syn. of *Liophloeus* Germar, 1817]. Comment: incorrect original stem formation, not in prevailing usage.
Scythropides Lacordaire, 1863: 380 [stem: *Scythrop-*]. Type genus: *Scythropus* Schönherr, 1826 [syn. of *Pachyrhinus* Schönherr, 1823]. Comment: original vernacular name available (Art. 11.7.2): first used in latinized form by Marseul (1863: 224, as Scytropidae [incorrect stem formation]), generally accepted as in Hustache (1919: 509, as Scythropini).
Auchmeresthinae Reitter, 1913b: 6, in key [stem: *Auchmeresthet-*]. Type genus: *Auchmeresthes* Kraatz, 1862. Comment: incorrect original stem formation, not in prevailing usage.

### Tribe Premnotrypini

Kuschel, 1956

Premnotrypini Kuschel, 1956: 187 [stem: *Premnotryp-*]. Type genus: *Premnotrypes* Pierce, 1914.

### †Tribe Pristorhynchini

Heer, 1847

Pristorhynchiden Heer, 1847: 190 [stem: *Pristorhynch-*]. Type genus: *Pristorhynchus* Heer, 1847. Comment: original vernacular name available (Art. 11.7.2): first used in latinized form by Scudder (1893: 29, as Pristorhynchini), generally accepted as in Alonso-Zarazaga and Lyal (1999: 176, as Pristorhynchini).

### Tribe Prypnini

Lacordaire, 1863

Prypnides Lacordaire, 1863: 135 [stem: *Prypn-*]. Type genus: *Prypnus* Schönherr, 1823. Comment: original vernacular name available (Art. 11.7.2): first used in latinized form by Heyne and Taschenberg (1907: 225, as Prypnini), generally accepted as in Alonso-Zarazaga and Lyal (1999: 176, as Prypnini).

### Tribe Psallidiini

Lacordaire, 1863

Psalidiides Lacordaire, 1863: 138 [stem: *Psallidi-*]. Type genus: *Psallidium* Herbst, 1795 [as *Psalidium*, incorrect subsequent spelling of type genus name, not in prevailing usage]. Comment: original vernacular name available (Art. 11.7.2): first used in latinized form by Everts (1903: 547, as Psalidiini [incorrect stem formation]), generally accepted as in Alonso-Zarazaga and Lyal (1999: 176, as Psallidiini); incorrect original stem formation, not in prevailing usage.

### Tribe Rhyncogonini

Sharp, 1919

Rhyncogonides Sharp, 1919a: 77 [stem: *Rhyncogon-*]. Type genus: *Rhyncogonus* Sharp, 1885.

### Tribe Sciaphilini

Sharp, 1891

Sciaphilina Sharp, 1891: 167 [stem: *Sciaphil-*]. Type genus: *Sciaphilus* Schönherr, 1823.
Sciaphilini Pierce, 1913: 405, in key [stem: *Sciaphil-*]. Type genus: *Sciaphilus* Schönherr, 1823. Comment: family-group name proposed as new without reference to Sciaphilina Sharp, 1891.

### Tribe Sitonini

Gistel, 1848

Sitonisidae Gistel, 1848: [8] [stem: *Siton-*]. Type genus: *Sitones* Schönherr, 1840 [syn. of *Sitona* Germar, 1817]. Comment: incorrect original stem formation, not in prevailing usage.

### Tribe Tanymecini

Lacordaire, 1863

Tanymécides Lacordaire, 1863: 82 [stem: *Tanymec-*]. Type genus: *Tanymecus* Germar, 1817.

### Subtribe Piazomiina

Reitter, 1913

Piazomiini Reitter, 1913b: 28, in key [stem: *Piazomi-*]. Type genus: *Piazomias* Schönherr, 1840.

### Subtribe Tainophthalmina

Desbrochers des Loges, 1873

Tainophthalmidae Desbrochers des Loges, 1873: 426 [stem: *Tainophthalm-*]. Type genus: *Tainophthalmus* Desbrochers des Loges, 1873.
Amomphi J. L. LeConte, 1874b: 455 [stem: *Amomph-*]. Type genus: *Amomphus* Schönherr, 1848 [syn. of *Aspidiotes* Schönherr, 1847].

### Subtribe Tanymecina

Lacordaire, 1863

Tanymécides Lacordaire, 1863: 82 [stem: *Tanymec-*]. Type genus: *Tanymecus* Germar, 1817. Comment: original vernacular name available (Art. 11.7.2): first used in latinized form by J. L. LeConte (1874b: 454, as Tanymecini), generally accepted as in Alonso-Zarazaga and Lyal (1999: 178, as Tanymecini).
Hadromerides Jekel, 1865: 545 [stem: *Hadromer-*]. Type genus: *Hadromerus* Schönherr, 1834 [preoccupied genus name, not *Hadromerus* Schönherr, 1823 [Coleoptera: Curculionidae: Entiminae]; syn. of *Hadromeropsis* Pierce, 1913]. Comment: permanently invalid (Art. 39): based on preoccupied type genus.
Siderodactylides Jekel, 1865: 551, in note [stem: *Siderodactyl-*]. Type genus: *Siderodactylus* Schönherr, 1834 [syn. of *Hadromerus* Schönherr, 1823].
Pachnaei J. L. LeConte, 1874b: 457 [stem: *Pachnae-*]. Type genus: *Pachnaeus* Schönherr, 1826.
Minyomeri G. H. Horn, 1876: 17 [stem: *Minyomer-*]. Type genus: *Minyomerus* G. H. Horn, 1876.
Pandeleteini Pierce, 1913: 399, in key [stem: *Pandeletei-*]. Type genus: *Pandeleteius* Schönherr, 1834. Comment: incorrect original stem formation, not in prevailing usage.

### Tribe Tanyrhynchini

Schönherr, 1826

Tanyrhynchides Schönherr, 1826: 212 [stem: *Tanyrhynch-*]. Type genus: *Tanyrhynchus* Schönherr, 1826.
Éremnides Lacordaire, 1863: 220 [stem: *Eremn-*]. Type genus: *Eremnus* Schönherr, 1823. Comment: original vernacular name available (Art. 11.7.2): first used in latinized form by J. L. LeConte (1869: 380, as Eremnini), generally accepted as in Schenkling and G. A. K. Marshall (1931b: Erem. 13, as Eremnini).

### Tribe Thecesternini

Lacordaire, 1863

Thécesternides Lacordaire, 1863: 306 [stem: *Thecestern-*]. Type genus: *Thecesternus* Say, 1831. Comment: original vernacular name available (Art. 11.7.2): first used in latinized form by Sharp (1891: 86, as Thecesterninae), generally accepted as in Alonso-Zarazaga and Lyal (1999: 182, as Thecesternini).

### Tribe Trachyphloeini

Gistel, 1848

Trachyphloeidae Gistel, 1848: [7] [stem: *Trachyphloe-*]. Type genus: *Trachyphloeus* Germar, 1817.

### Subtribe Trachyphilina

Voss, 1948

Trachyphilina Voss, 1948: 73, in key [stem: *Trachyphil-*]. Type genus: *Trachyphilus* Faust, 1887.

### Subtribe Trachyphloeina

Gistel, 1848

Trachyphloeidae Gistel, 1848: [7] [stem: *Trachyphloe-*]. Type genus: *Trachyphloeus* Germar, 1817.
Phyllastolidae Gistel, 1856a: 372 [stem: *Phyllastol-*]. Type genus: *Phyllastolus* Gistel, 1856 [syn. of *Trachyphloeus* Germar, 1817].
Cathormiocerini Reitter, 1913b: 8, in key [stem: *Cathormiocer-*]. Type genus: *Cathormiocerus* Schönherr, 1842.
Trachyphloeini Pierce, 1913: 421, in key [stem: *Trachyphloe-*]. Type genus: *Trachyphloeus* Germar, 1817. Comment: family-group name proposed as new without reference to Trachyphloeidae Gistel, 1848.
Pseudocneorrhinini Kôno, 1930: 163, in key [stem: *Pseudocneorhin-*]. Type genus: *Pseudocneorhinus* Roelofs, 1873 [as *Pseudocneorrhinus*, incorrect subsequent spelling of type genus name, not in prevailing usage]. Comment: incorrect original stem formation, not in prevailing usage.
Callirhopalini Voss, 1956b: 23 [stem: *Callirhopal-*]. Type genus: *Callirhopalus* Hochhuth, 1851.

### Tribe Tropiphorini

Marseul, 1863

Tropiphoridae Marseul, 1863: 220 [stem: *Tropiphor-*]. Type genus: *Tropiphorus* Schönherr, 1842 [placed on the Official List of Generic Names in Zoology (ICZN 1988c)]. Comment: Tropiphorini, published on 15 June 1863, is given priority over Strangaliodini, Byrsopagini, Pantopoeini and Synaptonychini which were published by Lacordaire “before 10 August 1863”.
Leptopsides Lacordaire, 1863: 232 [stem: *Leptop-*]. Type genus: *Leptops* Schönherr, 1834 [preoccupied genus name, not *Leptops* Rafinesque, 1820 [Pisces]; syn. of *Leptopius* Oke, 1951]. Comment: original vernacular name available (Art. 11.7.2): first used in latinized form by Pascoe (1870a: 181, as Leptopinae), generally accepted as in Heyne and Taschenberg (1907: 226, as Leptopsini [incorrect stem formation]); permanently invalid (Art. 39): based on preoccupied type genus; incorrect original stem formation, not in prevailing usage.
Strangaliodides Lacordaire, 1863: 234 [stem: *Strangaliod-*]. Type genus: *Strangaliodes* Schönherr, 1842. Comment: original vernacular name available (Art. 11.7.2): first used in latinized form by G. H. Horn (1876: 37, as Strangaliodes [treated as Latin]), generally accepted as in Voss (1954: 237, as Strangaliodini).
Byrsopagides Lacordaire, 1863: 337 [stem: *Byrsopag-*]. Type genus: *Byrsopages* Schönherr, 1842. Comment: original vernacular name available (Art. 11.7.2): first used in latinized form by Heyne and Taschenberg (1907: 228, as Byrsopagini), generally accepted as in Schenkling and G. A. K. Marshall (1929: 57, as Byrsopagini).
Pantopéides Lacordaire, 1863: 346 [stem: *Pantopoe-*]. Type genus: *Pantopoeus* Schönherr, 1842 [syn. of *Perperus* Schönherr, 1842]. Comment: original vernacular name available (Art. 11.7.2): first used in latinized form and generally accepted as in Faust (1892a: 179, as Pantopeinarum [incorrect stem formation]); incorrect original stem formation, not in prevailing usage.
Synaptonycides Lacordaire, 1863: 372 [stem: *Synaptonych-*]. Type genus: *Synaptonyx* G. R. Waterhouse, 1853. Comment: original vernacular name available (Art. 11.7.2): first used in latinized form and generally accepted as in Wollaston (1877: 158, as Synaptonychides [treated as Latin]); incorrect original stem formation, not in prevailing usage.
Rhigopsini J. L. LeConte, 1874b: 454 [stem: *Rhigopse-*]. Type genus: *Rhigopsis* J. L. LeConte, 1874. Comment: incorrect original stem formation, not in prevailing usage, the comment stating the contrary in Alonso-Zarazaga and Lyal (2002: 28) is incorrect.
Dyslobini J. L. LeConte, 1874b: 454 [stem: *Dyslob-*]. Type genus: *Dyslobus* J. L. LeConte, 1869.
Phyxeles G. H. Horn, 1876: 56 [stem: *Phyxelid-*]. Type genus: *Phyxelis* Schönherr, 1842. Comment: incorrect original stem formation, not in prevailing usage.
Dirotognathini G. H. Horn, 1876: 79 [stem: *Dirotognath-*]. Type genus: *Dirotognathus* G. H. Horn, 1876.
Synirmini Bedel, 1883: 32, in key [stem: *Synirm-*]. Type genus: *Synirmus* Bedel, 1883 [syn. of *Tropiphorus* Schönherr, 1842].
Stenocorynini McKeown, 1939: 408 [stem: *Stenocoryn-*]. Type genus: *Stenocorynus* Schönherr, 1842. Comment: replacement name for Leptopsides Lacordaire, 1863 because of the homonymy of the type genus.
Leptopiinae Oke, 1951: 24 [stem: *Leptopi-*]. Type genus: *Leptopius* Oke, 1951. Comment: replacement name for Leptopsides Lacordaire, 1863 because of the homonymy of the type genus.
Leptosinae G. A. K. Marshall, 1952: 264 [stem: *Leptos-*]. Type genus: *Leptosus* G. A. K. Marshall, 1952 [syn. of *Leptopius* Oke, 1951]. Comment: replacement name for Leptopsides Lacordaire, 1863 because of the homonymy of the type genus.

### Tribe Typhlorhinini

Kuschel, 1954

Typhlorhinini Kuschel, 1954: 287 [stem: *Typhlorhin-*]. Type genus: *Typhlorhinus* Kuschel, 1954 [syn. of *Hapactorrhynchus* Richard, 1953].

### Subfamily Hyperinae

Marseul, 1863 (1848)

Hyperidae Marseul, 1863: 224 [stem: *Hyper-*]. Type genus: *Hypera* Germar, 1817. Comment: use of family-group name conserved over Phytonominae Gistel, 1848 (Art. 40.2).

### Tribe Cepurini

Capiomont, 1867

*Haplopides Lacordaire, 1863: 394 [stem: *Haplopod-*]. Type genus: *Haplopus* Schönherr, 1840 [preoccupied genus name, not *Haplopus* Burmeister, 1838 [Orthoptera]; syn. of *Haplopodus* G. A. K. Marshall, 1946]. Comment: original vernacular name unavailable (Art. 11.7.2): subsequently used in latinized form, e.g., Heyne and Taschenberg (1907: 228, as Haplopini) but not generally accepted as valid; Haplopidae was used as valid by Ienistea (1986: 33) but it was not attributed to Lacordaire (1863); Ienistea’s name is also unavailable, it was proposed after 1930 without description or bibliographic reference to such a description (Art. 13.1); if found to be available then permanently invalid (Art. 39): based on preoccupied type genus; incorrect original stem formation, not in prevailing usage.
Cépurides Capiomont, 1867: 438 [stem: *Cepur-*]. Type genus: *Cepurus* Schönherr, 1834. Comment: original vernacular name available (Art. 11.7.2): first used in latinized form by Csiki (1934: 3, as Cepurini), generally accepted as in Alonso-Zarazaga and Lyal (1999: 189, as Cepurini).

### Tribe Hyperini

Marseul, 1863 (1848)

Phytonomidae Gistel, 1848: [8] [stem: *Phytonom-*]. Type genus: *Phytonomus* Schönherr, 1823 [syn. of *Hypera* Germar, 1817]. Comment: use of the younger names Hyperinae/-ini Marseul, 1863 are conserved over this name (Art. 40.2).
Hyperidae Marseul, 1863: 224 [stem: *Hyper-*]. Type genus: *Hypera* Germar, 1817. Comment: published 15 June 1863; use of family-group name conserved over Phytonomini Gistel, 1848 (Art. 40.2); this family-group name was also used in the same year by Lacordaire (1863 [before 10 August]: 395, as Hypérides).
Coniatina Legalov, 2007: 401 [stem: *Coniat-*]. Type genus: *Coniatus* Germar, 1817.
Macrotarrhusina Legalov, 2007: 401 [stem: *Macrotarrh-*]. Type genus: *Macrotarrhus* Bedel, 1906. Comment: incorrect original stem formation, not in prevailing usage.

### Subfamily Lixinae

Schönherr, 1823

Lixides Schönherr, 1823: column 1146 [stem: *Lix-*]. Type genus: *Lixus* Fabricius, 1801.

### Tribe Cleonini

Schönherr, 1826 nomen protectum

Geomorides Schönherr, 1823: column 1141 [stem: *Geomor-*]. Type genus: *Geomorus* Schönherr, 1823 [syn. of *Cleonis* Dejean, 1821]. Comment: as pointed out by Alonso-Zarazaga and Lyal (1999: 190) this is the oldest available name for the tribe name, however, it has not been used as valid after 1899 and therefore we treat it here as a *nomen oblitum* (see Appendix 1).
Cleonides Schönherr, 1826: 145 [stem: *Cleon-*]. Type genus: *Cleonus* Schönherr, 1826 [syn. of *Cleonis* Dejean, 1821]. Comment: *nomen protectum* (see Appendix 1).
Cleonidae Kirby, 1837: 198 [stem: *Cleon-*]. Type genus: *Cleonis* Dejean, 1821.
Xerobiadae Gistel, 1856a: 373 [stem: *Xerobi-*]. Type genus: *Xerobia* Gistel, 1856 [syn. of *Cleonis* Dejean, 1821]. Comment: incorrect original stem formation, not in prevailing usage.
*Bothynodérides Chevrolat, 1872: 16 [stem: *Bothynoder-*]. Type genus: *Bothynoderes* sensu Chevrolat, 1872 [not *Bothynoderes* Schönherr, 1823; syn. of *Asproparthenis* Gozis, 1886]. Comment: original vernacular name unavailable (Art. 11.7.2): not subsequently latinized.
*Conorhynchides Chevrolat, 1872: 17 [stem: *Conorhynch-*]. Type genus: *Conorhynchus* Motschulsky, 1860. Comment: original vernacular name unavailable (Art. 11.7.2): subsequently used in latinized form, e.g., Handlirsch (1925: 687 [as Cneorhynchides; *lapsus calami* for Conorhynchides [treated as Latin]), but not generally accepted as valid; Conorhynchinae Gill, 1861 (type genus *Conorhynchus* Gill, 1861, unjustified emendation of *Conorynchus* Nozeman, 1758 and junior homonym of *Conorhynchus* Motschulsky, 1860) is available in Pisces; the Pisces family-group name is permanently invalid since it it based on a preoccupied type genus (Art. 39).
*Cossinodérides Chevrolat, 1872: 18 [stem: *Cossinoder-*]. Type genus: *Cossinoderus* Chevrolat, 1872 [syn. of *Porocleonus* Motschulsky, 1860]. Comment: original vernacular name unavailable (Art. 11.7.2): not subsequently latinized.
Epirhynchini Petri, 1914: 3, in key [stem: *Epirrhynch-*]. Type genus: *Epirrhynchus* Schönherr, 1823 [as *Epirhynchus*, incorrect subsequent spelling of type genus name, not in prevailing usage]. Comment: incorrect original stem formation, not in prevailing usage.

### Tribe Lixini

Schönherr, 1823

Lixides Schönherr, 1823: column 1146 [stem: *Lix-*]. Type genus: *Lixus* Fabricius, 1801.
*Lariniden Suffrian, 1848: 62 [stem: *Larin-*]. Type genus: *Larinus* Germar, 1824 [preoccupied genus name, not *Larinus* Dejean, 1821 [Coleoptera: Curculionidae: Lixinae: Lixini]; syn. of *Larinus* Dejean, 1821]. Comment: original vernacular name unavailable (Art. 11.7.2): subsequently used in latinized form but not generally attributed to Suffrian (1848); if found to be available then permanently invalid (Art. 39): based on preoccupied type genus.
Larinidae Gistel, 1848: [7] [stem: *Larin-*]. Type genus: *Larinus* Germar, 1824 [preoccupied genus name, not *Larinus* Dejean, 1821 [Coleoptera: Curculionidae: Lixinae: Lixini]; syn. of *Larinus* Dejean, 1821]. Comment: permanently invalid (Art. 39): based on preoccupied type genus.
Phyllonomeidae Gistel, 1856a: 372 [stem: *Phyllonome-*]. Type genus: *Phyllonomeus* Gistel, 1856.
Larinini Petri, 1914: 4, in key [stem: *Larin-*]. Type genus: *Larinus* Germar, 1824 [preoccupied genus name, not *Larinus* Dejean, 1821 [Coleoptera: Curculionidae: Lixinae: Lixini]; syn. of *Larinus* Dejean, 1821]. Comment: family-group name proposed as new without reference to Larinidae Gistel, 1848; permanently invalid (Art. 39): based on preoccupied type genus.

### Tribe Rhinocyllini

Lacordaire, 1863

Rhinocyllides Lacordaire, 1863: 433 [stem: *Rhinocyll-*]. Type genus: *Rhinocyllus* Germar, 1817. Comment: original vernacular name available (Art. 11.7.2): first used in latinized form by Faust (1904: 182, as Rhinocyllinae), generally accepted as in Alonso-Zarazaga and Lyal (1999: 193, as Rhinocyllini).

### Subfamily Mesoptiliinae

Lacordaire, 1863

Mésoptilides Lacordaire, 1863: 563 [stem: *Mesoptili-*]. Type genus: *Mesoptilius* Labram and Imhoff, 1845.

### Tribe Carciliini

Pierce, 1916

Carciliinae Pierce, 1916: 465 [stem: *Carcili-*]. Type genus: *Carcilia* Roelofs, 1874.

### Tribe Laemosaccini

Lacordaire, 1865

Lémosacides Lacordaire, 1865: 12 [stem: *Laemosacc-*]. Type genus: *Laemosaccus* Schönherr, 1823. Comment: original vernacular name available (Art. 11.7.2): first used in latinized form by Pascoe (1870b: 437, as Laemosaccinae), generally accepted as in Alonso-Zarazaga and Lyal (1999: 194, as Laemosaccini); incorrect original stem formation, not in prevailing usage.

### Tribe Magdalidini

Pascoe, 1870 nomen protectum

Thamnophilides Schönherr, 1823: column 1136 [stem: *Thamnophil-*]. Type genus: *Thamnophilus* Schönherr, 1823 [preoccupied genus name, not *Thamnophilus* Vieillot, 1816 [Aves]; syn. of *Magdalis* Germar, 1817]. Comment: permanently invalid (Art. 39): based on preoccupied type genus.
Scardamyctisidae Gistel, 1848: [7] [stem: *Scardamyct-*]. Type genus: *Scardamyctes* Gistel, 1848 [syn. of *Magdalis* Germar, 1817]. Comment: Scardamyctini Gistel, 1848 was recently considered a *nomen oblitum* by Colonnelli (2003: 7) however the necessary supporting references were not provided; we hereby consider Scardamyctini Gistel, 1848 as a *nomen oblitum* (see supporting references in Appendix 1); incorrect original stem formation, not in prevailing usage.
Magdalinidae Gistel, 1856a: 371 [stem: *Magdalin-*]. Type genus: *Magdalinus* Germar, 1843 [placed on the Official Index of Rejected and Invalid Generic Names in Zoology by the Commission (ICZN 1955a); syn. of *Magdalis* Germar, 1817]. Comment: permanently invalid (Art. 39): based on suppressed type genus.
Magdalinae Pascoe, 1870b: 436 [stem: *Magdalid-*]. Type genus: *Magdalis* Germar, 1817 [placed on the Official List of Generic Names in Zoology (ICZN 1955a)]. Comment: *nomen protectum* (see Appendix 1); incorrect original stem formation, not in prevailing usage.
Rhininae Pierce, 1916: 465 [stem: *Rhin-*]. Type genus: *Rhina* Latreille, 1802 [preoccupied genus name, not *Rhina* Schneider, 1801 [Pisces]; syn. of *Panus* Schönherr, 1823]. Comment: permanently invalid (Art. 39): based on preoccupied type genus.

### Tribe Mesoptiliini

Lacordaire, 1863

Mésoptilides Lacordaire, 1863: 563 [stem: *Mesoptili-*]. Type genus: *Mesoptilius* Labram and Imhoff, 1845. Comment: original vernacular name available (Art. 11.7.2): first used in latinized form by Pascoe (1870b: 436, as Mesoptilinae [incorrect stem formation]), generally accepted as in Alonso-Zarazaga and Lyal (1999: 193, as Mesoptiliini); incorrect original stem formation, not in prevailing usage.
Cnemidophorini Hustache, 1937: 199, in key [stem: *Cnemidophor-*]. Type genus: *Cnemidophorus* Schönherr, 1835 [preoccupied genus name, not *Cnemidophorus* Wagler, 1830 [Reptilia]; syn. of *Cnemidontus* Schenkling, 1935]. Comment: permanently invalid (Art. 39): based on preoccupied type genus.
Cnemidontini Kuschel, 1955: 271 [stem: *Cnemidont-*]. Type genus: *Cnemidontus* Schenkling, 1935. Comment: replacement name for Cnemidophorini Hustache, 1937 because of the homonymy of the type genus.

### Subfamily Molytinae

Schönherr, 1823

Molytides Schönherr, 1823: column 1142 [stem: *Molyt-*]. Type genus: *Molytes* Schönherr, 1823 [syn. of *Liparus* A. G. Olivier, 1807].

### Tribe Anoplini

Bedel, 1884

Anoplini Bedel, 1884a: 67, in key [stem: *Anopl-*]. Type genus: *Anoplus* Germar, 1820.

### Tribe Amalactini

Lacordaire, 1863

Amalactides Lacordaire, 1863: 506 [stem: *Amalact-*]. Type genus: *Amalactus* Schönherr, 1835. Comment: original vernacular name available (Art. 11.7.2): first used in latinized form by Pascoe (1870b: 436, as Amalactinae), generally accepted as in Alonso-Zarazaga and Lyal (1999: 196, as Amalactini).

### Tribe Aminyopini

Voss, 1956

Aminyopini Voss, 1956b: 30, in key [stem: *Aminyop-*]. Type genus: *Aminyops* Voss, 1956.
Niphadini Voss, 1963: 5 [stem: *Niphad-*]. Type genus: *Niphades* Pascoe, 1871.
Niphadonothina Voss, 1965: 344, in key [stem: *Niphadonoth-*]. Type genus: *Niphadonothus* Voss, 1965.

### Tribe Amorphocerini

Voss, 1939

Amorphocerini Voss, 1939: 65 [stem: *Amorphocer-*]. Type genus: *Amorphocerus* Schönherr, 1826.

### Tribe Anchonini

Imhoff, 1856

Anchonidae Imhoff, 1856: [2] 213 [stem: *Anchon-*]. Type genus: *Anchonus* Schönherr, 1825.

### Tribe Brachyceropseini

Aurivillius, 1926

Brachyceropseinae Aurivillius, 1926b: 2 [stem: *Brachyceropse-*]. Type genus: *Brachyceropsis* Aurivillius, 1926. Comment: Alonso-Zarazaga and Lyal (2002: 18) incorrectly changed their previous opinion in Alonso-Zarazaga and Lyal (1999: 103) regarding the correct stem based on this genus name, the correct stem is *Brachyceropse*-.

### Tribe Cholini

Schönherr, 1825

Cholides Schönherr, 1825: column 584 [stem: *Chol-*]. Type genus: *Cholus* Germar, 1824 [placed on the Official List of Generic Names in Zoology (ICZN 1987d)].

### Subtribe Cholina

Schönherr, 1825

Cholides Schönherr, 1825: column 584 [stem: *Chol-*]. Type genus: *Cholus* Germar, 1824 [placed on the Official List of Generic Names in Zoology (ICZN 1987d)].
Amerininae Pierce, 1919: 30, in key [stem: *Amerin-*]. Type genus: *Ameris* Dejean, 1821.

### Subtribe Cholomina

Vaurie, 1974

Cholomini Vaurie, 1974: 4 [stem: *Cholom-*]. Type genus: *Cholomus* Roelofs, 1880.

### Subtribe Rhinastina

Vaurie, 1973

Rhinastini Vaurie, 1973: 4, in key [stem: *Rhinast-*]. Type genus: *Rhinastus* Schönherr, 1825.

### Tribe Cleogonini

Gistel, 1848

Cleogonidae Gistel, 1848: [7] [stem: *Cleogon-*]. Type genus: *Cleogonus* Schönherr, 1825.

### Tribe Conotrachelini

Jekel, 1865

Conotrachelides Jekel, 1865: 550 [stem: *Conotrachel-*]. Type genus: *Conotrachelus* Dejean, 1835.
Echinaspini Blatchley, 1922: 121 [stem: *Echinaspid-*]. Type genus: *Echinaspis* Blatchley, 1922 [preoccupied genus name, not *Echinaspis* Haeckel, 1881 [Protista]; syn. of *Microhyus* J. L. LeConte, 1876]. Comment: permanently invalid (Art. 39): based on preoccupied type genus; incorrect original stem formation, not in prevailing usage.

### Tribe Cycloterini

Lacordaire, 1863

Cyclotérides Lacordaire, 1863: 365 [stem: *Cycloter-*]. Type genus: *Cycloteres* Schönherr, 1843.

### Subtribe Cycloterina

Lacordaire, 1863

Cyclotérides Lacordaire, 1863: 365 [stem: *Cycloter-*]. Type genus: *Cycloteres* Schönherr, 1843. Comment: original vernacular name available (Art. 11.7.2): first used in latinized form by Heyne and Taschenberg (1907: 228, as Cycloterini), generally accepted as in Alonso-Zarazaga and Lyal (1999: 200, as Cycloterini).

### Subtribe Thrombosternina

Voss, 1965

Thrombosternina Voss, 1965: 344, in key [stem: *Thrombostern-*]. Type genus: *Thrombosternus* G. A. K. Marshall, 1955.

### Tribe Dinomorphini

Lacordaire, 1863

Dinomorphides Lacordaire, 1863: 291 [stem: *Dinomorph-*]. Type genus: *Dinomorphus* Perty, 1832. Comment: original vernacular name available (Art. 11.7.2): first used in latinized form by Pascoe (1870b: 436, as Dinomorphinae), generally accepted as in Alonso-Zarazaga and Lyal (1999: 201, as Dinomorphini).

### Tribe Emphyastini

Lacordaire, 1863

Emphiastides Lacordaire, 1863: 510 [stem: *Emphyast-*]. Type genus: *Emphyastes* Mannerheim, 1852 [as *Emphiastes*, incorrect subsequent spelling of type genus name, not in prevailing usage]. Comment: original vernacular name available (Art. 11.7.2): first used in latinized form by J. L. LeConte (1876: 137, as Emphyastini), generally accepted as in Alonso-Zarazaga and Lyal (1999: 143, as Emphyastina); incorrect original stem formation, not in prevailing usage.
Phycocoetes J. L. LeConte, 1876: 189 [stem: *Phycocoet-*]. Type genus: *Phycocoetes* J. L. LeConte, 1876 [preoccupied genus name, not *Phycocoetes* Agassiz, 1846 [Pisces]; syn. of *Thalasselephas* Egorov and Korotyaev, 1976]. Comment: permanently invalid (Art. 39): based on preoccupied type genus.
Thalasselephantini Alonso-Zarazaga and Lyal, 1999: 208 [stem: *Thalasselephant-*]. Type genus: *Thalasselephas* Egorov and Korotyaev, 1976. Comment: replacement name for Phycocoetini J. L. LeConte, 1876 because of the homonymy of the type genus.

### Tribe Euderini

Lacordaire, 1865

Eudérides Lacordaire, 1865: 18 [stem: *Euder-*]. Type genus: *Euderes* Schönherr, 1825. Comment: original vernacular name available (Art. 11.7.2): first used in latinized form by Pascoe (1870b: 437, as Euderinae), generally accepted as in Alonso-Zarazaga and Lyal (1999: 201, as Euderini); the junior homonym Euderini Erdös, 1956 (type genus *Euderus* Haliday, 1844) is available in Hymenoptera: Eulophidae; the name Entiinae Hedqvist, 1974 is now used as valid in Hymenoptera instead of Euderini Erdös, 1956 (see Hansson and Straka 2009: 272).

### Tribe Galloisiini

Morimoto, 1962

Galloisiinae Morimoto, 1962b: 375 [stem: *Galloisi-*]. Type genus: *Galloisia* Hustache, 1920.

### Tribe Guioperini

Lacordaire, 1865

Guiopérides Lacordaire, 1865: 78 [stem: *Guioper-*]. Type genus: *Guioperus* Perty, 1832. Comment: original vernacular name available (Art. 11.7.2): first used in latinized form by Heyne and Taschenberg (1907: 232, as Guioperini), generally accepted as in Alonso-Zarazaga and Lyal (1999: 201, as Guioperini).

### Tribe Hylobiini

Kirby, 1837

Hylobidae Kirby, 1837: 196 [stem: *Hylobi-*]. Type genus: *Hylobius* Germar, 1817.

### Subtribe Epistrophina

Marshall, 1932

Epistrophina G. A. K. Marshall, 1932: 346, in key [stem: *Epistroph-*]. Type genus: *Epistrophus* Kirsch, 1868 [preoccupied genus name, not *Epistrophus* Gistel, 1834 [Coleoptera: Elateridae]; *Epistrophus* Kirsch, 1868 is currently considered a valid genus name]. Comment: as pointed out by Alonso-Zarazaga and Lyal (1999: 202) this family-group name should be treated as permanently invalid (Art. 39) because it is based on a preoccupied type genus, however, an application will be submitted by MAAZ and CHCL to the Commission for the conservation of the genus *Epistrophus* Kirsch, 1868 over its unused senior homonym; meanwhile current usage is maintained.

### Subtribe Hylobiina

Kirby, 1837

Hylobidae Kirby, 1837: 196 [stem: *Hylobi-*]. Type genus: *Hylobius* Germar, 1817. Comment: incorrect original stem formation, not in prevailing usage.
Heilipinae Faust, 1892b: 202, in key [stem: *Heilipod-*]. Type genus: *Heilipus* Germar, 1824. Comment: incorrect original stem formation, not in prevailing usage.
Syphorbina G. A. K. Marshall, 1932: 346, in key [stem: *Syphorb-*]. Type genus: *Syphorbus* Pascoe, 1881.

### Tribe Ithyporini

Lacordaire, 1865

Ithyporides Lacordaire, 1865: 50 [stem: *Ithypor-*]. Type genus: *Ithyporus* Schönherr, 1836. Comment: precedence (Ithyporini Lacordaire, 1865 vs Sclerocardiini Lacordaire, 1865) given to taxon originally proposed at the higher rank (Art. 24.1).

### Subtribe Colobodina

Voss, 1958

Colobodini Voss, 1958: 50, in key [stem: *Colobod-*]. Type genus: *Colobodes* Schönherr, 1837.

### Subtribe Ithyporina

Lacordaire, 1865

Ithyporides Lacordaire, 1865: 50 [stem: *Ithypor-*]. Type genus: *Ithyporus* Schönherr, 1836. Comment: original vernacular name available (Art. 11.7.2): first used in latinized form by Kirsch (1870: 192, as Ithyporidae), generally accepted as in Alonso-Zarazaga and Lyal (1999: 203, as Ithyporini).

### Subtribe Sclerocardiina

Lacordaire, 1865

Sclérocardiides Lacordaire, 1865: 317 [stem: *Sclerocardi-*]. Type genus: *Sclerocardius* Schönherr, 1847. Comment: original vernacular name available (Art. 11.7.2): first used in latinized form by Heyne and Taschenberg (1907: 233, as Sclerocardini [incorrect stem formation]), generally accepted as in Alonso-Zarazaga and Lyal (1999: 204, as Sclerocardiina).

### Tribe Itini

Reitter, 1913

Itini Reitter, 1913b: 58, in key [stem: *It-*]. Type genus: *Ita* Tournier, 1878.

### Tribe Juanorhinini

Aurivillius, 1931

Juanorhinini Aurivillius, 1931: 465 [stem: *Juanorhin-*]. Type genus: *Juanorhinus* Aurivillius, 1926.

### Tribe Lepyrini

Kirby, 1837

Lepyridae Kirby, 1837: 197 [stem: *Lepyr-*]. Type genus: *Lepyrus* Germar, 1817.

### Tribe Lithinini

Lacordaire, 1863

Lithinides Lacordaire, 1863: 349 [stem: *Lithin-*]. Type genus: *Lithinus* Klug, 1833.

### Subtribe Lithinina

Lacordaire, 1863

Lithinides Lacordaire, 1863: 349 [stem: *Lithin-*]. Type genus: *Lithinus* Klug, 1833. Comment: original vernacular name available (Art. 11.7.2): first used in latinized form by Pascoe (1870b: 436, as Lithininae), generally accepted as in Alonso-Zarazaga and Lyal (1999: 204, as Lithinini).

### Subtribe Rhytidophloeina

Voss, 1963

Rhytidophloeini Voss, 1963: 3 [stem: *Rhytidophloe-*]. Type genus: *Rhytidophloeus* Schönherr, 1842.

### Tribe Lymantini

Lacordaire, 1865

Lymantides Lacordaire, 1865: 328 [stem: *Lymant-*]. Type genus: *Lymantes* Schönherr, 1837. Comment: original vernacular name available (Art. 11.7.2): first used in latinized form by Heyne and Taschenberg (1907: 234, as Lymantini), generally accepted as in Alonso-Zarazaga and Lyal (1999: 205, as Lymantini).
Ithaurinae Kuschel, 1959b: 509 [stem: *Ithaur-*]. Type genus: *Ithaura* Pascoe, 1871.
Caecossonina Osella, 1980: 369 [stem: *Caecosson-*]. Type genus: *Caecossonus* Gilbert, 1955.

### Tribe Mecysolobini

Reitter, 1913

Alcidides Jekel, 1865: 547 [stem: *Alcid-*]. Type genus: *Alcides* Schönherr, 1825 [preoccupied genus name, not *Alcides* Hübner, 1822 [Lepidoptera]; syn. of *Sternuchopsis* Heller, 1918]. Comment: permanently invalid (Art. 39): based on preoccupied type genus.
Mecyslobini Reitter, 1913b: 33, in key [stem: *Mecysolob-*]. Type genus: *Mecysolobus* Reitter, 1905 [as *Mecyslobus*, incorrect subsequent spelling of type genus name, not in prevailing usage]. Comment: incorrect original stem formation, not in prevailing usage.
Alcidodinae G. A. K. Marshall, 1939: 582 [stem: *Alcidod-*]. Type genus: *Alcidodes* G. A. K. Marshall, 1939 [syn. of *Sternuchopsis* Heller, 1918]. Comment: replacement name for Alcidinae Jekel, 1865 because of the homonymy of the type genus.

### Tribe Metatygini

Pascoe, 1888

Metatyginae Pascoe, 1888: 409 [stem: *Metatyg-*]. Type genus: *Metatyges* Pascoe, 1865 [syn. of *Omophorus* Schönherr, 1835].
Omophorinae G. A. K. Marshall, 1917: 195 [stem: *Omophor-*]. Type genus: *Omophorus* Schönherr, 1835.
Metatyginae Pierce, 1919: 30, in key [stem: *Metatyg-*]. Type genus: *Metatyges* Pascoe, 1865 [syn. of *Omophorus* Schönherr, 1835]. Comment: family-group name proposed as new without reference to Metatyginae Pascoe, 1888.
Sternechosomini Voss, 1958: 43, in key [stem: *Sternechosom-*]. Type genus: *Sternechosomus* Voss, 1958.

### Tribe Molytini

Schönherr, 1823

Molytides Schönherr, 1823: column 1142 [stem: *Molyt-*]. Type genus: *Molytes* Schönherr, 1823 [syn. of *Liparus* A. G. Olivier, 1807].

### Subtribe Leiosomatina

Reitter, 1913

Liosomina Reitter, 1913b: 52, in key [stem: *Leiosomat-*]. Type genus: *Leiosoma* Stephens, 1829 [as *Liosoma*, unjustified emendation of type genus name by Agassiz (1846b: 204), not in prevailing usage]. Comment: incorrect original stem formation, not in prevailing usage.

### Subtribe Molytina

Schönherr, 1823

Molytides Schönherr, 1823: column 1142 [stem: *Molyt-*]. Type genus: *Molytes* Schönherr, 1823 [syn. of *Liparus* A. G. Olivier, 1807].
Liparides Latreille, 1828: 597 [stem: *Lipar-*]. Type genus: *Liparus* A. G. Olivier, 1807.

### Subtribe Plinthina

Lacordaire, 1863

Plinthides Lacordaire, 1863: 359 [stem: *Plinth-*]. Type genus: *Plinthus* sensu Westwood, 1838 [not *Plinthus* Germar, 1817; syn. of *Mitoplinthus* Reitter, 1897 but see Alonso-Zarazaga and Lyal (2010) and Appendix 6]. Comment: based on a misidentified type genus; an application was recently submitted to the Commission by Alonso-Zarazaga and Lyal (2010) to keep the name Plinthina in its usual sense, with *Plinthus* Germar, 1817 as its type genus (see Appendix 6).
Minyopidae Marseul, 1863: 221 [stem: *Minyop-*]. Type genus: *Minyops* Schönherr, 1823.

### Subtribe Typoderina

Voss, 1965

Typoderina Voss, 1965: 343, in key [stem: *Typoder-*]. Type genus: *Typoderus* G. A. K. Marshall, 1953.

### Tribe Nettarhinini

Lacordaire, 1865

Nettarhinides Lacordaire, 1865: 76 [stem: *Nettarhin-*]. Type genus: *Nettarhinus* Schönherr, 1826. Comment: original vernacular name available (Art. 11.7.2): first used in latinized form by Hustache (1936: 62, as Nettarhinina), generally accepted as in Alonso-Zarazaga and Lyal (1999: 206, as Nettarhinini).

### Tribe Pacholenini

Lacordaire, 1863

Pacholénides Lacordaire, 1863: 443 [stem: *Pacholen-*]. Type genus: *Pacholenus* Schönherr, 1826. Comment: original vernacular name available (Art. 11.7.2): first used in latinized form by Heyne and Taschenberg (1907: 229, as Pacholenini), generally accepted as in Alonso-Zarazaga and Lyal (1999: 206, as Pacholenini).

### Tribe Paipalesomini

Marshall, 1932

Paipalesomini G. A. K. Marshall, 1932: 345, in key [stem: *Paipalesom-*]. Type genus: *Paipalesomus* Schönherr, 1847 [syn. of *Peribleptus* Schönherr, 1843].

### Tribe Petalochilini

Lacordaire, 1863

Pétalochilides Lacordaire, 1863: 517 [stem: *Petalochil-*]. Type genus: *Petalochilus* Schönherr, 1836. Comment: original vernacular name available (Art. 11.7.2): first used in latinized form by Pascoe (1870b: 436, as Petalochilinae), generally accepted as in Alonso-Zarazaga and Lyal (1999: 206, as Petalochilini).
Épipédides Lacordaire, 1865: 186 [stem: *Epiped-*]. Type genus: *Epipedus* Schönherr, 1842 [preoccupied genus name, not *Epipedus* Spinola, 1837 [Hemiptera]; syn. of *Epipedophyes* G. A. K. Marshall, 1946]. Comment: original vernacular name available (Art. 11.7.2): first used in latinized form and generally accepted as in Pascoe (1870b: 437, as Epipedinae); permanently invalid (Art. 39): based on preoccupied type genus.
Hormopini J. L. LeConte, 1876: 320 [stem: *Hormop-*]. Type genus: *Hormops* J. L. LeConte, 1876.
Schoenherriellinae Viana, 1952: 231 [stem: *Schoenherriell-*]. Type genus: *Schoenherriella* Viana, 1952 [syn. of *Epipedophyes* G. A. K. Marshall, 1946]. Comment: replacement name for Epipédides Lacordaire, 1865 because of the homonymy of the type genus.
Epipedophyinae Kuschel, 1955: 270 [stem: *Epipedophy-*]. Type genus: *Epipedophyes* G. A. K. Marshall, 1946. Comment: replacement name for Epipédides Lacordaire, 1865 because of the homonymy of the type genus.

### Tribe Phoenicobatini

Champion, 1914

Phoenicobatina Champion, 1914: 416 [stem: *Phoenicobat-*]. Type genus: *Phoenicobates* Champion, 1914.

### Tribe Phrynixini

Kuschel, 1964

Phrynixinae Kuschel, 1964: 472 [stem: *Phrynix-*]. Type genus: *Phrynixus* Pascoe, 1875.

### Tribe Pissodini

Gistel, 1848

Pissodisidae Gistel, 1848: [7] [stem: *Pissod-*]. Type genus: *Pissodes* Germar, 1817.

### Subtribe Cotasteromimina

Morimoto, 1962

Cotasteromimini Morimoto, 1962a: 61, in key [stem: *Cotasteromim-*]. Type genus: *Cotasteromimus* Chûjô and Voss, 1960.

### Subtribe Orthorhinina

Jekel, 1865

Orthorhinides Jekel, 1865: 548 [stem: *Orthorhin-*]. Type genus: *Orthorhinus* Schönherr, 1825.

### Subtribe Pissodina

Gistel, 1848

Pissodisidae Gistel, 1848: [7] [stem: *Pissod-*]. Type genus: *Pissodes* Germar, 1817. Comment: incorrect original stem formation, not in prevailing usage.
Angianides Sharp, 1919b: 151 [stem: *Angian-*]. Type genus: *Angianus* Sharp, 1919 [syn. of *Vanapa* Pouillaude, 1915].

### Tribe Sternechini

Lacordaire, 1863

Sternéchides Lacordaire, 1863: 447 [stem: *Sternech-*]. Type genus: *Sternechus* Schönherr, 1826. Comment: original vernacular name available (Art. 11.7.2): first used in latinized form by Champion (1902: 113, as Sternechina), generally accepted as in Alonso-Zarazaga and Lyal (1999: 208, as Sternechini).

### Tribe Styanacini

Chûjô and Voss, 1960

Styanacinae Chûjô and Voss, 1960: 2 [stem: *Styanac-*]. Type genus: *Styanax* Pascoe, 1871.

### Tribe Trachodini

Gistel, 1848

Trachodisidae Gistel, 1848: [7] [stem: *Trachod-*]. Type genus: *Trachodes* Germar, 1824. Comment: incorrect original stem formation, not in prevailing usage.
Blastophiladae Gistel, 1856a: 370 [stem: *Blastophil-*]. Type genus: *Blastophila* Gistel, 1856 [syn. of *Trachodes* Germar, 1824]. Comment: incorrect original stem formation, not in prevailing usage.
Acicnémides Lacordaire, 1865: 31 [stem: *Acicnemid-*]. Type genus: *Acicnemis* Fairmaire, 1849. Comment: original vernacular name available (Art. 11.7.2): first used in latinized form by Klima (1935: Acic. 1, as Acicnemidinae), generally accepted as in Alonso-Zarazaga and Lyal (1999: 196, as Acicnemidini); incorrect original stem formation, not in prevailing usage.

### Tribe Trigonocolini

Lacordaire, 1863

Trigonocolides Lacordaire, 1863: 592 [stem: *Trigonocol-*]. Type genus: *Trigonocolus* Lacordaire, 1863. Comment: original vernacular name available (Art. 11.7.2): first used in latinized form by Pascoe (1870b: 436, as Trigonocolinae), generally accepted as in Alonso-Zarazaga and Lyal (1999: 208, as Trigonocolini).
Megarhininae Faust, 1888: 284 [stem: *Megarhin-*]. Type genus: *Megarhinus* Schönherr, 1835 [preoccupied genus name, not *Megarhinus* Rafinesque, 1820 [Pisces]; syn. of *Trigonocolus* Lacordaire, 1863]. Comment: permanently invalid (Art. 39): based on preoccupied type genus.

### Tribe Trypetidini

Lacordaire, 1865

Trypétides Lacordaire, 1865: 177 [stem: *Trypetid-*]. Type genus: *Trypetes* Schönherr, 1836 [placed on the Official List of Generic Names in Zoology (ICZN 1974a)]. Comment: original vernacular name available (Art. 11.7.2): first used in latinized form by Pascoe (1870b: 437, as Trypetinae [incorrect stem formation]), generally accepted as in Alonso-Zarazaga and Lyal (1999: 209, as Trypetidini); incorrect original stem formation, not in prevailing usage; stem based on *Trypetes* Schönherr, 1836 ruled to be *Trypetid*- and Trypetidinae placed on the Official List of Family-Group Names in Zoology (ICZN 1974a, as Trypetidinae Pierce, 1919).
Trypetesidae Heller, 1916: 348, in note [stem: *Trypetes-*]. Type genus: *Trypetes* Schönherr, 1836 [placed on the Official List of Generic Names in Zoology (ICZN 1974a)]. Comment: emendation for Trypetidae Lacordaire, 1865 to avoid homonymy with Trypetidae Loew, 1861 (type genus *Trypeta* Meigen, 1803) in Diptera; corrected spelling not adopted since it creates homonymy problem with Trypetesidae Stebbing, 1910 (type genus *Trypetesa* Norman, 1903) in Crustacea.

### Subfamily Orobitidinae

Thomson, 1859

Orobitina C. G. Thomson, 1859: 138 [stem: *Orobitid-*]. Type genus: *Orobitis* Germar, 1817. Comment: incorrect original stem formation, not in prevailing usage; correction of stem by Alonso-Zarazaga and Lyal (1999: 210).

### Subfamily Xiphaspidinae

Marshall, 1920

Xiphaspidinae G. A. K. Marshall, 1920: 393 [stem: *Xiphaspid-*]. Type genus: *Xiphaspis* G. A. K. Marshall, 1920.

### Subfamily Scolytinae

Latreille, 1804

Scolitarii Latreille, 1804c: 156 [stem: *Scolyt-*]. Type genus: *Scolytus* Geoffroy, 1762 [placed on the Official List of Generic Names in Zoology (ICZN 1963b)]. Comment: name placed on the Official List of Family-Group Names in Zoology (ICZN 1963b, as Scolytidae Westwood, 1838).

### Tribe Amphiscolytini

Mandelshtam and Beaver, 2003

Amphiscolytini Mandelshtam and Beaver, 2003: 2 [stem: *Amphiscolyt-*]. Type genus: *Amphiscolytus* Mandelshtam and Beaver, 2003.

### Tribe Bothrosternini

Blandford, 1896

Bothrosternini Blandford, 1896: 120 [stem: *Bothrostern-*]. Type genus: *Bothrosternus* Eichhoff, 1868.

### Tribe Cactopinini

Chamberlin, 1939

Cactopinae Chamberlin, 1939: 243 [stem: *Cactopin-*]. Type genus: *Cactopinus* Schwartz, 1899. Comment: incorrect original stem formation, not in prevailing usage; correction of stem by Wood (1978: 113).

### Tribe Carphodicticini

Wood, 1971

Carphodicticini Wood, 1971: 19 [stem: *Carphodictic-*]. Type genus: *Carphodicticus* Wood, 1971.

### Tribe Coptonotini

Chapuis, 1869

Coptonotidae Chapuis, 1869: 11 [stem: *Coptonot-*]. Type genus: *Coptonotus* Chapuis, 1869.

### Tribe Corthylini

LeConte, 1876

Corthyli J. L. LeConte, 1876: 347 [stem: *Corthyl-*]. Type genus: *Corthylus* Erichson, 1836.

### Subtribe Corthylina

LeConte, 1876

Corthyli J. L. LeConte, 1876: 347 [stem: *Corthyl-*]. Type genus: *Corthylus* Erichson, 1836.
Amphicranidae Eichhoff, 1878: 460 [stem: *Amphicran-*]. Type genus: *Amphicranus* Erichson, 1836.
Xyleboripina Reitter, 1913c: 102 [stem: *Xyleborip-*]. Type genus: *Xyleborips* Reitter, 1913. Comment: incorrectly spelled Xyloboripina on page 31 of the same work.
Gnathotrichina Balachowsky, 1949: 241 [stem: *Gnathotrich-*]. Type genus: *Gnathotrichus* Eichhoff, 1869.

### Subtribe Pityophthorina

Eichhoff, 1878

Pityophthoridae Eichhoff, 1878: 173 [stem: *Pityophthor-*]. Type genus: *Pityophthorus* Eichhoff, 1864. Comment: First Revisers found (Pityophthorina Eichhoff, 1878 vs Araptina Eichhoff, 1878) are Wood and Bright (1992: 949).
Araptidae Eichhoff, 1878: 305 [stem: *Arapt-*]. Type genus: *Araptus* Eichhoff, 1872.

### Tribe Cryphalini

Lindemann, 1877

Cryphaloideae Lindemann, 1877: 165 [stem: *Cryphal-*]. Type genus: *Cryphalus* Erichson, 1836.
Trypophloeinae Nüsslin, 1911: 375 [stem: *Trypophloe-*]. Type genus: *Trypophloeus* Fairmaire, 1868.
Ernoporinae Nüsslin, 1911: 375 [stem: *Ernopor-*]. Type genus: *Ernoporus* C. G. Thomson, 1859.
*Eidopherinae Murayama, 1954: 200 [stem: *Eidophel-*]. Type genus: *Eidophelus* Eichhoff, 1875. Comment: unavailable (Art. 13.1): proposed after 1930 without description or bibliographic reference to such a description; incorrect original stem formation, not in prevailing usage; correction of stem by Wood (1978: 114).

### Tribe Crypturgini

LeConte, 1876

Crypturgi J. L. LeConte, 1876: 387 [stem: *Crypturg-*]. Type genus: *Crypturgus* Erichson, 1836.

### †Tribe Cylindrobrotini

Kirejtshuk, Azar, Beaver, Mandelshtam and Nel, 2009

Cylindrobrotini Kirejtshuk et al., 2009: 103 [stem: *Cylindrobrot-*]. Type genus: *Cylindrobrotus* Kirejtshuk et al., 2009.

### Tribe Diamerini

Hagedorn, 1909

Diamerinae Hagedorn, 1909: 163 [stem: *Diamer-*]. Type genus: *Diamerus* Erichson, 1836.
Strombophorini Schedl, 1959: 16 [stem: *Strombophor-*]. Type genus: *Strombophorus* Hagedorn, 1909. Comment: name proposed after 1930 without description or bibliographic reference to such a description (Art. 13.1), however available because it was used as valid before 2000 as in Ferreira (1966: 664, as Strombophorini) and was not rejected by an author who, between 1961 and 1999, applied Article 13 of the then current edition of the Code (see Art. 13.2.1).
Sphaerotrypini Murayama, 1963: 66 [stem: *Sphaerotryp-*]. Type genus: *Sphaerotrypes* Blandford, 1894.

### Tribe Dryocoetini

Lindemann, 1877

Dryocoetoideae Lindemann, 1877: 165 [stem: *Dryocoet-*]. Type genus: *Dryocoetes* Eichhoff, 1864 [placed on the Official List of Generic Names in Zoology (ICZN 1979b)].
Thamnurginae Nüsslin, 1911: 377 [stem: *Thamnurg-*]. Type genus: *Thamnurgus* Eichhoff, 1864.
Taphrorychini Reitter, 1913c: 29 [stem: *Taphrorych-*]. Type genus: *Taphrorychus* Eichhoff, 1878.

### Tribe Hexacolini

Eichhoff, 1878

Ctenophoridae Chapuis, 1869: 49 [stem: *Ctenophor-*]. Type genus: *Ctenophorus* Chapuis, 1869 [preoccupied genus name, not *Ctenophorus* Fitzinger, 1843 [Reptilia]; syn. of *Scolytodes* Ferrari, 1867]. Comment: permanently invalid (Art. 39): based on preoccupied type genus.
Hexacolidae Eichhoff, 1878: 306 [stem: *Hexacol-*]. Type genus: *Hexacolus* Eichhoff, 1868. Comment: First Revisers found (Problechilini Eichhoff, 1878 vs Hexacolini Eichhoff, 1878) are Alonso-Zarazaga and Lyal (2009: 58).
Problechilidae Eichhoff, 1878: 167 [stem: *Problechil-*]. Type genus: *Problechilus* Eichhoff, 1878.
Erineophilides Hopkins, 1902: 37 [stem: *Erineophil-*]. Type genus: *Erineophilus* Hopkins, 1902.

### Tribe Hylastini

LeConte, 1876

Hylastes J. L. LeConte, 1876: 387 [stem: *Hylast-*]. Type genus: *Hylastes* Erichson, 1836.
Hylurgopina Balachowsky, 1949: 122 [stem: *Hylurgop-*]. Type genus: *Hylurgops* J. L. LeConte, 1876.

### Tribe Hylesinini

Erichson, 1836

Hylesinen Erichson, 1836: 46 [stem: *Hylesin-*]. Type genus: *Hylesinus* Fabricius, 1801. Comment: original vernacular name available (Art. 11.7.2): first used in latinized form by Shuckard (1839b: 63, as Hylesinidae), generally accepted as in Alonso-Zarazaga and Lyal (2009: 63, as Hylesinini); incorrect original stem formation, not in prevailing usage.
Phloeotrupides Lacordaire, 1865: 370 [stem: *Phloeotrup-*]. Type genus: *Phloeotrupes* Erichson, 1836. Comment: original vernacular name available (Art. 11.7.2): first used in latinized form and generally accepted as in Chapuis (1869: 11, as Phloeotrupidae).
Phloeobori Blandford, 1893: 426 [stem: *Phloeobor-*]. Type genus: *Phloeoborus* Erichson, 1836.
Dactylipalpi Blandford, 1893: 426 [stem: *Dactylipalp-*]. Type genus: *Dactylipalpus* Chapuis, 1869.
Alniphagini Murayama, 1963: 29 [stem: *Alniphag-*]. Type genus: *Alniphagus* Swaine, 1918.

### Tribe Hylurgini

Gistel, 1848

Hylurgidae Gistel, 1848: [6] [stem: *Hylurg-*]. Type genus: *Hylurgus* Latreille, 1806. Comment: status resurrected by Alonso-Zarazaga and Lyal (2009: 69).
Dendroctonides Nüsslin, 1912: 278 [stem: *Dendrocton-*]. Type genus: *Dendroctonus* Erichson, 1836 [placed on the Official List of Generic Names in Zoology (ICZN 1963a)].
Xylechinides Nüsslin, 1912: 281 [stem: *Xylechin-*]. Type genus: *Xylechinus* Chapuis, 1869.
Tomicini Wood, 1978: 118, in key [stem: *Tomic-*]. Type genus: *Tomicus* Latreille, 1802 [placed on the Official List of Generic Names in Zoology (ICZN 1963a)]. Comment: the older name Tomicidae Shuckard, 1839 was based on misidentified type genus (see Alonso-Zarazaga and Lyal 2009); an application will be submitted by MAAZ and CHCL to the Commission to suppress Tomicidae Shuckard, 1839 for the Principles of Priority and Homonymy (Art. 65.2.1).

### Tribe Hyorrhynchini

Hopkins, 1915

Hyorrhynchinae Hopkins, 1915: 225, in key [stem: *Hyorrhynch-*]. Type genus: *Hyorrhynchus* Blandford, 1894.
*Sueinae Murayama, 1959: 26 [stem: *Sue-*]. Type genus: *Sueus* Murayama, 1951. Comment: unavailable (Art. 13.1): proposed after 1930 without description or bibliographic reference to such a description.
Hyorrhynchini Murayama, 1963: 62 [stem: *Hyorrhynch-*]. Type genus: *Hyorrhynchus* Blandford, 1894. Comment: family-group name proposed as new without reference to Hyorrhynchinae Hopkins, 1915.
Sueinae Murayama, 1963: 4 [stem: *Sue-*]. Type genus: *Sueus* Murayama, 1951.

### Tribe Hypoborini

Nüsslin, 1911

Hypoborinae Nüsslin, 1911: 376 [stem: *Hypobor-*]. Type genus: *Hypoborus* Erichson, 1836.
Chaetophloeini Schedl, 1966: 361 [stem: *Chaetophloe-*]. Type genus: *Chaetophloeus* J. L. LeConte, 1876.

### Tribe Ipini

Bedel, 1888

Tomicidae Shuckard, 1839b: 64 [stem: *Tomic-*]. Type genus: *Tomicus* sensu Shuckard, 1839 [not *Tomicus* Latreille, 1802; syn. of *Ips* DeGeer, 1775]. Comment: based on a misidentified type genus (see Alonso-Zarazaga and Lyal 2009); see also Tomicini Wood, 1978; an application will be submitted by MAAZ and CHCL to the Commission to suppress Tomicidae Shuckard, 1839 for the Principles of Priority and Homonymy (Art. 65.2.1).
Ipini Bedel, 1888: 386 [stem: *Ip-*]. Type genus: *Ips* DeGeer, 1775. Comment: an application needs to be submitted to the Commission to suppress Ipini Latreille, 1802 (based on the misidentified type genus *Ips* sensu Latreille, 1802) for the Principles of Priority and Homonymy (Art. 65.2.1) to conserve this name as valid.
Pityogenina Balachowsky, 1949: 244 [stem: *Pityogen-*]. Type genus: *Pityogenes* Bedel, 1888.

### Tribe Micracidini

LeConte, 1876

Micracides J. L. LeConte, 1876: 367 [stem: *Micracid-*]. Type genus: *Micracis* J. L. LeConte, 1868. Comment: incorrect original stem formation, not in prevailing usage; stem corrected by Alonso-Zarazaga and Lyal (2009: 81).
Hylocuridae Eichhoff, 1878: 298 [stem: *Hylocur-*]. Type genus: *Hylocurus* Eichhoff, 1872.

### Tribe Phloeosinini

Nüsslin, 1912

Phloeosinides Nüsslin, 1912: 289 [stem: *Phloeosin-*]. Type genus: *Phloeosinus* Chapuis, 1869 [placed on the Official List of Generic Names in Zoology (ICZN 1981a)].
*Dendrosinini Nunberg, 1967: 314 [stem: *Dendrosin-*]. Type genus: *Dendrosinus* Chapuis, 1869. Comment: unavailable (Art. 13.1): proposed after 1930 without description or bibliographic reference to such a description.

### Tribe Phloeotribini

Chapuis, 1869

Phloeotribidae Chapuis, 1869: 42 [stem: *Phloeotrib-*]. Type genus: *Phloeotribus* Latreille, 1797 [placed on the Official List of Generic Names in Zoology (ICZN 1979a)].
Phthorophloeides Nüsslin, 1912: 289 [stem: *Phthorophloe-*]. Type genus: *Phthorophloeus* Rey, 1885.

### Tribe Phrixosomatini

Wood, 1978

Phrixosomini Wood, 1978: 118, in key [stem: *Phrixosomat-*]. Type genus: *Phrixosoma* Blandford, 1897. Comment: incorrect original stem formation, not in prevailing usage; correction of stem by Alonso-Zarazaga and Lyal (2009: 91).

### Tribe Polygraphini

Chapuis, 1869

Polygraphidae Chapuis, 1869: 48 [stem: *Polygraph-*]. Type genus: *Polygraphus* Erichson, 1836.
Carphoborinae Nüsslin, 1911: 376 [stem: *Carphobor-*]. Type genus: *Carphoborus* Eichhoff, 1864.

### Tribe Premnobiini

Browne, 1962

Premnobiini Browne, 1962: 80 [stem: *Premnobi-*]. Type genus: *Premnobius* Eichhoff, 1878. Comment: family-group name available (Art. 13.1.2): description by indication (distinguishing characters given in Browne (1961)).

### Tribe Scolytini

Latreille, 1804

Scolitarii Latreille, 1804c: 156 [stem: *Scolyt-*]. Type genus: *Scolytus* Geoffroy, 1762 [placed on the Official List of Generic Names in Zoology (ICZN 1963b)]. Comment: name placed on the Official List of Family-Group Names in Zoology (ICZN 1963b, as Scolytidae Westwood, 1838); incorrect original stem formation, not in prevailing usage.
Eccoptogastridae Gistel, 1848: [6] [stem: *Eccoptogastr-*]. Type genus: *Ekkoptogaster* Herbst, 1793 [as *Eccoptogaster*, incorrect subsequent spelling of type genus name by Gyllenhal (1813), not in prevailing usage; *Ekkoptogaster* Herbst, 1793 and *Eccoptogaster* Erichson, 1836 placed on the Official Index of Rejected and Invalid Genus-Group Names in Zoology (ICZN 1963b); syn. of *Scolytus* Geoffroy, 1762]. Comment: permanently invalid (Art. 39): based on suppressed type genus; Eccoptogasterinae Trédl, 1907 and Eccoptogasterini Reitter, 1906 placed on the Index of Rejected and Invalid Family-Group names in Zoology (ICZN 1963b).
Camptocérides Lacordaire, 1865: 366 [stem: *Camptocer-*]. Type genus: *Camptocerus* Dejean, 1821. Comment: original vernacular name available (Art. 11.7.2): first used in latinized form and generally accepted as in Chapuis (1869: 49, as Camptoceridae).
Minulini Reitter, 1913c: 12 [stem: *Minul-*]. Type genus: *Minulus* Eggers, 1912.

### Tribe Scolytoplatypodini

Blandford, 1893

Scolytoplatypini Blandford, 1893: 428 [stem: *Scolytoplatypod-*]. Type genus: *Scolytoplatypus* C. Schaufuss, 1891. Comment: incorrect original stem formation, not in prevailing usage; correction of stem by Blanford (1895: 84).
Taeniocerini Blandford, 1893: 428 [stem: *Taeniocer-*]. Type genus: *Taeniocerus* Blandford, 1893 [preoccupied genus name, not *Taeniocerus* Kaup, 1871 [Coleoptera: Passalidae]; syn. of *Scolytoplatypus* C. Schaufuss, 1891]. Comment: permanently invalid (Art. 39): based on preoccupied type genus.
Spongocerinae Hagedorn, 1909: 163 [stem: *Spongocer-*]. Type genus: *Spongocerus* Blandford, 1893.

### Tribe Xyleborini

LeConte, 1876

Xylebori J. L. LeConte, 1876: 358 [stem: *Xylebor-*]. Type genus: *Xyleborus* Eichhoff, 1864 [placed on the Official List of Generic Names in Zoology (ICZN 1968a)].
Webbinae Hopkins, 1915: 224, in key [stem: *Webbi-*]. Type genus: *Webbia* Hopkins, 1915. Comment: incorrect original stem formation, not in prevailing usage.
*Eccoptopterina Browne, 1961: 49 [stem: *Eccoptopter-*]. Type genus: *Eccoptopterus* Motschulsky, 1863. Comment: unavailable (Art. 13.1): proposed after 1930 without description or bibliographic reference to such a description.

### Tribe Xyloctonini

Eichhoff, 1878

Xyloctonidae Eichhoff, 1878: 171 [stem: *Xylocton-*]. Type genus: *Xyloctonus* Eichhoff, 1872.

### Tribe Xyloterini

LeConte, 1876

Xyloteri J. L. LeConte, 1876: 356 [stem: *Xyloter-*]. Type genus: *Xyloterus* Erichson, 1836.
*Trypodendrinae Trédl, 1907: 70 [stem: *Trypodendr-*]. Type genus: *Trypodendron* Stephens, 1830. Comment: family-group name unavailable (Art. 11.7.1.1): not based on a genus used as valid at the time.
Trypodendrina Nunberg, 1954: 16 [stem: *Trypodendr-*]. Type genus: *Trypodendron* Stephens, 1830.

### Subfamily Platypodinae

Shuckard, 1839

Platypodidae Shuckard, 1839b: 64 [stem: *Platypod-*]. Type genus: *Platypus* Herbst, 1793.

### Tribe Mecopelmini

Thompson, 1992

*Mecopelmini Wood, 1966: 45 [stem: *Mecopelm-*]. Type genus: *Mecopelmus* Blackman, 1944. Comment: unavailable (Art. 13.1): proposed after 1930 without description or bibliographic reference to such a description.
Mecopelminae Thompson, 1992: 873, in key [stem: *Mecopelm-*]. Type genus: *Mecopelmus* Blackman, 1944.

### Tribe Platypodini

Shuckard, 1839

Platypodidae Shuckard, 1839b: 64 [stem: *Platypod-*]. Type genus: *Platypus* Herbst, 1793.
Crossotarsariae H. Strohmeyer, 1914: 19 [stem: *Crossotars-*]. Type genus: *Crossotarsus* Chapuis, 1865.

### Tribe Schedlariini

Wood and Bright, 1992

Chapuisiides Blandford, 1895: 89 [stem: *Chapuisi-*]. Type genus: *Chapuisia* Dugès, 1886 [preoccupied genus name, not *Chapuisia* Duvivier, 1885 [Coleoptera: Chrysomelidae]; syn. of *Schedlarius* Wood, 1957]. Comment: permanently invalid (Art. 39): based on preoccupied type genus.
Schedlarini Wood and Bright, 1992: 1087 [stem: *Schedlari-*]. Type genus: *Schedlarius* Wood, 1957. Comment: replacement name for Chapuisiides Blandford, 1895 because of the homonymy of the type genus; incorrect original stem formation, not in prevailing usage; correction of stem and transfer to Platypodinae by Alonso-Zarazaga and Lyal (2009: 17).

### Tribe Tesserocerini

Strohmeyer, 1914

Tesserocerinae H. Strohmeyer, 1914: 19 [stem: *Tesserocer-*]. Type genus: *Tesserocerus* Saunders, 1836. Comment: precedence (Tesserocerini H. Strohmeyer, 1914 vs Diapodini Strohmeyer, 1914) given to taxon originally proposed at the higher rank (Art. 24.1).

### Subtribe Diapodina

Strohmeyer, 1914

Diapodariae H. Strohmeyer, 1914: 19 [stem: *Diapod-*]. Type genus: *Diapus* Chapuis, 1865.
Genyocerinae Hopkins, 1915: 225, in key [stem: *Genyocer-*]. Type genus: *Genyocerus* Motschulsky, 1858.

### Subtribe Tesserocerina

Strohmeyer, 1914

Tesserocerinae H. Strohmeyer, 1914: 19 [stem: *Tesserocer-*]. Type genus: *Tesserocerus* Saunders, 1836. Comment: precedence (Tesserocerina H. Strohmeyer, 1914 vs Cenocephalina Strohmeyer, 1914) given to taxon originally proposed at the higher rank (Art. 24.1).
Symmerariae H. Strohmeyer, 1914: 19 [stem: *Symmer-*]. Type genus: *Symmerus* Chapuis, 1865 [preoccupied genus name, not *Symmerus* Walker, 1848 [Diptera]; syn. of *Chaetastus* Nunberg, 1953]. Comment: permanently invalid (Art. 39): based on preoccupied type genus.
Cenocephalariae H. Strohmeyer, 1914: 19 [stem: *Cenocephal-*]. Type genus: *Cenocephalus* Chapuis, 1865.
Platytarsilidae Schedl, 1939: 387 [stem: *Platytarsul-*]. Type genus: *Platytarsulus* Schedl, 1935. Comment: incorrect original stem formation, not in prevailing usage; correction of stem by Wood and Bright (1992: 1084).
Periomatini Schedl, 1939: 397, in key [stem: *Periommat-*]. Type genus: *Periommatus* Chapuis, 1865 [as *Periomatus*, incorrect subsequent spelling of type genus name, not in prevailing usage]. Comment: incorrect original stem formation, not in prevailing usage; correction of stem by Schedl (1972: 249).
Platypicerinae Nunberg, 1953: 44 [stem: *Platypicer-*]. Type genus: *Platypicerus* Nunberg, 1953.

### Coleoptera incertae sedis

Homoeoplastidae Gistel, 1856a: 360. Type genus: *Homoeoplastus* Gistel, 1856 [Gistel includes three species in his new genus, none of which are described in his work nor attributed to authors as having been described previously; we therefore consider *Homoeoplastus* unavailable]. Comment: family-group name unavailable (Art. 11.7.1.1): not based on an available genus name.
Plocasteidae Gistel, 1856a: 365. Type genus: *Plocastes* Gistel, 1856 [the only species originally included in *Plocastes* is *scaraboides*; Gistel does not mention the author of the species name, nor does he describe the taxon, we therefore consider *Plocastes* as unavailable]. Comment: family-group name unavailable (Art. 11.7.1.1): not based on an available genus name.
Serratopalpidae Gistel, 1856a: 384. Type genus: *Serratopalpus* Gistel, 1856 [*Serratopalpus helwigii* was the only species originally included in *Serratopalpus*; this new species was not described in Gistel’s original paper therefore the genus name is considered unavailable]. Comment: family-group name unavailable (Art. 11.7.1.1): not based on an available genus name.

## Supplementary Material

XML Treatments for this article (over 3000)

## Appendix 1

Conservation of younger names using Reversal of Precedence (Art. 23.9). Cases listed in alphabetical order by family.

### Carabidae

Supporting references for the conservation of Perigonini Horn, 1881 over Trechicini Bates, 1873 (Art. 23.9.2). The taxon name Trechicini Bates, 1873 has not been used as valid after 1899 to our knowledge.

Assmann T (1999) A new anophthalmic genus of Perigonini from the Iberian Peninsula (Insecta, Coleoptera, Carabidae). Spixiana 22 (3): 255–262.

Ball GE, Bousquet Y (2000) Carabidae Latreille, 1810 [pp. 32–132]. In: Arnett RH, Thomas MC (Eds) American beetles. Volume 1. Archostemata, Myxophaga, Adephaga, Polyphaga: Staphyliniformia. CRC Press, Boca Raton, xv + 443 pp.

Basilewsky P (1989) Révision des Perigonini d’Afrique (Coleoptera Carabidae). Revue de Zoologie Africaine 103 (4): 413–452.

Bousquet Y (2003) Tribe Perigonini G.H. Horn, 1881 [pp. 448–449]. In: Löbl I, Smetana A (Eds) Catalogue of Palaearctic Coleoptera. Volume 1. Archostemata - Myxophaga - Adephaga. Apollo Books, Stenstrup, 819 pp.

Bousquet Y, Larochelle A (1993) Catalogue of the Geadephaga (Coleoptera: Trachypachidae, Rhysodidae, Carabidae including Cicindelini) of America north of Mexico. Memoirs of the Entomological Society of Canada 167: 1–397.

Dajoz R (2002) Les coléoptères carabidés et ténébrionidés: écologie et biologie. Tec et Doc, Paris, xi + 522 pp.

Darlington PJ, Jr. (1968) The carabid beetles of New Guinea Part III. Harpalinae (continued): Perigonini to Pseudomorphini. Bulletin of the Museum of Comparative Zoology at Harvard 137 (1): 1–253.

Erwin TL (1985) The taxon pulse: a general pattern of lineage radiation and extinction among carabid beetles [pp. 437–472]. In: Ball GE (Ed) Taxonomy, phylogeny and zoogeography of beetles and ants. Series Entomologica Volume 33. Junk, Dordrecht, xiii + 514 pp. + 2 pls.

Erwin TL, Sims LL (1984) Carabid beetles of the West Indies (Insecta: Coleoptera): a synopsis of the genera and checklist of tribes of Caraboidea, and of the West Indian species. Quaestiones Entomologicae 20 (4): 351–466 + 1 pl.

Hůrka K (1996) Carabidae of the Czech and Slovak republics. Kabourek, Zlin, 565 pp.

Kryzhanovskij OL, Belousov IA, Kabak II, Kataev BM, Makarov KV, Shilenkov VG (1995) A checklist of the ground beetles of Russia and adjacent lands (Insecta, Coleoptera, Carabidae). Pensoft Series Faunistica No. 3. Pensoft Publishers, Sofia and Moscow, 271 pp.

Lafer GS (1989) I. Podotryad Archostemata; II. Podtryad Adephaga [pp. 66–259]. Tom III Zhestkokrylye, ili zhuki Chast 1. Nauka, Leningrad, 572 pp. [in Russian].

Larochelle A, Larivière M-C (2001) Carabidae (Insecta: Coleoptera): catalogue. Fauna of New Zealand. Ko te Aitanga Pepeke o Aotearoa. Number 43, Manaaki Whenua Press, Lincoln, 285 pp.

Larochelle A, Larivière M-C (2007) Carabidae (Insecta: Coleoptera): synopsis of supraspecific taxa. Fauna of New Zealand. Ko te Aitanga Pepeke o Aotearoa. Number 60, Manaaki Whenua Press, Lincoln, 188 pp.

Lindroth CH (1968) The ground-beetles (Carabidae, excl. Cicindelinae) of Canada and Alaska. Part 5. Opuscula Entomologica Supplementum 33: 649–944.

Lorenz W (2005) Systematic list of extant ground beetles of the world (Insecta Coleoptera «Geadephaga»: Trachypachidae and Carabidae incl. Paussinae, Cicindelinae, Rhysodinae). Second Edition. W. Lorenz, Tutzing, [ii] + 530 pp.

Martínez C (2005) Introducción a los escarabajos Carabidae (Coleoptera) de Colombia. Instituto de Investigación de Recursos Biológicos Alexander von Humboldt, Bogota, 546 pp.

Moore BP, Weir TA, Pyke JE (1987) Carabidae [pp. 23–320]. In: Lawrence JF, Moore BP, Pyke JE, Weir TA (Eds) Zoological catalogue of Australia. Volume 4. Coleoptera: Archostemata, Myxophaga and Adephaga. Australian Government Publishing Service, Canberra, viii + 444 pp.

Moret P (2008) Four new species of *Diploharpus* Chaudoir, 1850 from Ecuador (Coleoptera, Carabidae, Perigonini). Memoirs on Biodiversity 1: 201–208.

Nelson RE (1991) First records of *Perigona pallipennis* (LeC.) and *Perigona nigriceps* (Dej.) (Coleoptera: Carabidae: Perigonini) from Maine: easternmost records for the genus in North America. The Coleopterists Bulletin 45 (3): 284–285.

Perrault GG (1985) Etudes sur la faune des Carabidae de Guyane. 2. Une nouvelle espèce de *Perigona* microphthalme (Coleoptera, Carabidae, Perigonini). L’Entomologiste 41: 61–64.

Perrault GG (1988) Notes sur la tribu des Perigonini (Coleoptera Carabidae) avec les descriptions de deux sous-genres et d’une espèce. Entomologische Blätter für Biologie und Systematik der Käfer 84 (1/2): 11–16.

Perrault GG (1992a) Notes sur les types des espèces néotropicales du genre *Perigona* décrites par Bates (Coleoptera, Carabidae, Perigonini). Nouvelle Revue d’Entomologie Nouvelle Série 8 [1991] (4): 398.

Perrault GG (1992b) Etudes sur la faune des Carabidae de Guyane. III. Deux espèces nouvelles de *Diploharpus* (Perigonini). L’Entomologiste 48 (2): 99–103.

Reichardt H (1977) A synopsis of the genera of Neotropical Carabidae (Insecta: Coleoptera). Quaestiones Entomologicae 13: 346–493.

### Carabidae

Supporting references for the conservation of Anisodactylina Lacordaire, 1854 over Eurytrichina LeConte, 1848 (Art. 23.9.2). The taxon name Eurytrichina LeConte, 1848 has not been used as valid after 1899 to our knowledge.

Ball GE, Bousquet Y (2000) Carabidae Latreille, 1810 [pp. 32–132]. In: Arnett RH, Thomas MC (Eds) American beetles. Volume 1. Archostemata, Myxophaga, Adephaga, Polyphaga: Staphyliniformia. CRC Press, Boca Raton, xv + 443 pp.

Bousquet Y, Larochelle A (1993) Catalogue of the Geadephaga (Coleoptera: Trachypachidae, Rhysodidae, Carabidae including Cicindelini) of America north of Mexico. Memoirs of the Entomological Society of Canada 167: 1–397.

Bousquet Y, Tchang J-P (1992) Anisodactyline larvae (Coleoptera: Carabidae: Harpalini): descriptions of genus-group taxa of eastern Canada and phylogenetic remarks. The Canadian Entomologist 124 (5): 751–783.

Habu A (1973) Carabidae: Harpalini (Insecta: Coleoptera). Fauna Japonica, Keigaku Publishing Co., Tokyo, xiii + 430 pp. + 24 pls.

Hůrka K (1996) Carabidae of the Czech and Slovak republics. Kabourek, Zlin, 565 pp.

Ito N (1997) Some new taxa of the subtribe Anisodactylina from Asia with a key to the Asian genera (Coleoptera, Carabidae, Harpalini). Japanese Journal of Entomology 65 (4): 816–825.

Ito N (2003a) Subtribe Anisodactylina Lacordaire, 1854 [pp. 360–363]. In: Löbl I, Smetana A (Eds) Catalogue of Palaearctic Coleoptera. Volume 1. Archostemata - Myxophaga - Adephaga. Apollo Books, Stenstrup, 819 pp.

Ito N (2003b) Notes on species of the harpaline subtribe Anisodactylina (Coleoptera, Carabidae) from China. Special Bulletin of the Japanese Society of Coleopterology 6: 79–86.

Kaiser M (2004) Faunistik und Biogeographie der Anisodactylinae und Harpalinae Westfalens (Coleoptera: Carabidae). Abhandlungen aus dem Westfälischen Museum für Naturkunde 66 (3): 1–155.

Kataev BM (2003) A new genus and species of the subtribe Anisodactylina from south-western Australia (Coleoptera: Carabidae: Harpalini). Acta Zoologica Academiae Scientiarum Hungaricae 48 (3): 173–179.

Kataev BM (2002) On some new and little-known species of the Anisodactylina and Harpalina (the Selenophori group) from East Asia and Oriental region (Coleoptera: Carabidae: Harpalini). O nekotorykh novykh i maloizvestnykh vidakh Anisodactylina i Harpalina (gruppa Selenophori) iz vostochnoy Azii i Oriental’nogo regiona (Coleoptera: Carabidae: Harpalini). Russian Entomological Journal 11 (3): 241–252.

Kataev BM, Wrase DW (2001) A new genus and a new species of the subtribe Anisodactylina from Vietnam and remarks on the taxonomic position of *Hiekea picipes* N. Ito 1997 (Coleoptera: Carabidae: Harpalini). Linzer Biologische Beiträge 33: 637–646.

Kryzhanovskij OL, Belousov IA, Kabak II, Kataev BM, Makarov KV, Shilenkov VG (1995) A checklist of the ground beetles of Russia and adjacent lands (Insecta, Coleoptera, Carabidae). Pensoft Series Faunistica No. 3. Pensoft Publishers, Sofia and Moscow, 271 pp.

Larochelle A, Larivière M-C (2001) Carabidae (Insecta: Coleoptera): catalogue. Fauna of New Zealand. Ko te Aitanga Pepeke o Aotearoa. Number 43, Manaaki Whenua Press, Lincoln, 285 pp.

Larochelle A, Larivière M-C (2005) Harpalini (Insecta: Coleoptera: Carabidae: Harpalinae). Fauna of New Zealand. Ko te Aitanga Pepeke o Aotearoa. Number 53, Manaaki Whenua Press, Lincoln, 160 pp.

Larochelle A, Larivière M-C (2007) Carabidae - (Insecta: Coleoptera): synopsis of supraspecific taxa. Fauna of New Zealand. Ko te Aitanga Pepeke o Aotearoa. Number 60, Manaaki Whenua Press, Lincoln, 188 pp.

Lindroth CH (1968) The ground-beetles (Carabidae, excl. Cicindelinae) of Canada and Alaska. Part 5. Opuscula Entomologica Supplementum 33: 649–944.

Machado A (1992) Monografia de los carábidos de las Islas Canarias (Insecta, Coleoptera). Instituto de Estudios Canarios, La Laguna, 734pp.

Martínez C (2005) Introducción a los escarabajos Carabidae (Coleoptera) de Colombia. Instituto de Investigación de Recursos Biológicos Alexander von Humboldt, Bogota, 546 pp.

Martinez-Navarro EM, Galian J, Serrano J (2003) Phylogenetic relationships among subtribes of Harpalini Bonelli 1810 (Coleoptera, Carabidae), and *Harpalus* Latreille 1802 and some related western Palearctic taxa. A preliminary cladistic analysis using morphological and karyotypic characters. Acta Entomológica Ibérica e Macaronésica 1: 59–65.

Martinez-Navarro EM, Galian J, Serrano J (2005) Phylogeny and molecular evolution of the tribe Harpalini (Coleoptera, Carabidae) inferred from mitochondrial cytochrome-oxidase I. Molecular Phylogenetics and Evolution 35: 127–146.

Noonan GR (1973) The anisodactylines (Insecta: Coleoptera: Carabidae: Harpalini): classification, evolution, and zoogeography. Quaestiones Entomologicae 9 (4): 266–480.

Noonan GR (1974) *Allendia*, a new South American genus with notes on its evolutionary relationships to other genera of Anisodactylina (Coleoptera: Carabidae: Harpalini). The Coleopterists Bulletin 28 (4): 219–227.

Noonan GR (1976) Synopsis of the supra-specific taxa of the tribe Harpalini (Coleoptera: Carabidae). Quaestiones Entomologicae 12 (1): 3–87.

Reichardt H (1977) A synopsis of the genera of Neotropical Carabidae (Insecta: Coleoptera). Quaestiones Entomologicae 13 (4): 346–493.

### Cucujidae

Supporting references for the conservation of *Pediacus* Shuckard, 1839 over *Biophloeus* Dejean, 1835 (Art. 23.9.2). The genus name *Biophloeus* Dejean, 1835 has not been used as valid after 1899 to our knowledge.

Alexander KNA (1983) Some records of *Pediacus dermestoides* (Fab.) (Col., Cucujidae) from central southern England. Entomologist’s Monthly Magazine 119: 219.

Alexander KNA (1987) *Pediacus dermestoides* (Fab.) (Col., Cucujidae) new to Cornwall, and some comments on its apparent increase in abundance in southern Britain. Entomologist’s Monthly Magazine 123: 162.

Beutel RG, Pollock DA (2000) Larval head morphology of *Phycosecis litoralis* (Pascoe) (Coleoptera: Phycosecidae) with phylogenetic implications. Invertebrate Taxonomy 14 (6): 825–835.

Böhme J (2005) Band K. Katalog (Faunistische Übersicht). 2. Auflage. Die Käfer Mitteleuropas, Elsevier GmbH, Spektrum A. V., Munich, xii + 515 pp.

Bousquet Y (1991) Family Cucujidae: flat bark beetles [pp. 219–220]. In: Bousquet Y (Ed) Checklist of the beetles of Canada and Alaska. Research Branch Publication 1861/E, Ottawa, vi + 430 pp.

Burakowski B, Mroczkowski M, Stefańska J (1986) Chrząszcze Coleoptera. Cucujoidea, część 1. Katalog Fauny Polski. Część XXIII, tom 12 [Nr 43]. Panstwowe Wydawnictwo Naukowe, Warszawa, 266 pp.

Cooter J (1977) *Pediacus depressus* (Herbst, 1797) (Col.: Cucujidae) new to Scotland. The Entomologist’s Record and Journal of Variation 89 (12): 339–340.

Crowson RA (1973) Further observations on Phloeostichidae and Cavognathidae, with definitions of new genera from Australia and New Zealand. The Coleopterists Bulletin 27 (2): 54–62.

Grosso-Silva JM (2002) Registos interessantes de coleópteros (Insecta, Coleoptera) para Portugal (3ª nota). Primeiro registo ibérico de *Pediacus dermestoides* (Fabricius, 1792) (Cucujidae). Boletín de la Sociedad Entomológica Aragonesa 31: 49–54.

Háva J (2000) Faunistic records from the Czech Republic - 95. Coleoptera. Klapalekiana 36 (1/3): 36.

Jansen RP, van de Sande C (2008) The genus *Pediacus* in The Netherlands (Coleoptera: Cucujidae). Entomologische Berichten 68 (1): 17–20.

Lawrence JF (1991) Cucujidae (Cucujoidea) (including Catogenidae, Laemophloeidae, Passandridae, Silvanidae) [pp. 463–488]. In: Stehr FW (Ed) Immature Insects. Volume 2. Kendall / Hunt Publishing Company, Dubuque, xvi + 975 pp.

Lawrence JF, Britton EB (1994) Australian Beetles. Melbourne University Press, Carlton, x + 192 pp. + 16 pls.

Lawrence JF, Newton AF, Jr. (1995) Families and subfamilies of Coleoptera (with selected genera, notes, references and data on family-group names) [pp. 779–1006]. In: Pakaluk J, Ślipiński SA (Eds) Biology, phylogeny, and classification of Coleoptera: papers celebrating the 80th birthday of Roy A Crowson Volume 2. Muzeum i Institut Zologii PAN, Warszawa, x + 1092 pp. in 2 vols.

Majka CG (2008) The flat bark beetles (Coleoptera, Silvanidae, Cucujidae, Laemophloeidae) of Atlantic Canada. ZooKeys 2: 221–238.

Martínez de Murguía L, Lapaza J, Salaberria E, Méndez M, Molino-Olmedo F (2004) Coleópteros saproxílicos (Insecta: Coleoptera) de un hayedo acidófilo en regeneración del norte peninsular. Munibe (Sciencias Naturales) 55: 167–182.

Muona J, Rutanen I (1994) The short-term impact of fire on the beetle fauna in boreal coniferous forest. Annales Zoologici Fennici 31: 109–121.

Nakane T, Ohbayashi K, Nomura S, Kurosawa Y (1984) Iconographia Insectorum Japonicorum, Colore Naturali Edita. Volume 2 (Coleoptera). Hokuryukan, Tokyo, 461 pp.

Nikitskij NB, Belov VV (1979) Novyy I maloizvestnyy vidy Clavicornia (Coleoptera) iz Talysha. New and poorly known species of Clavicornia (Coleoptera) from Talysh. Zoologicheskii Zhurnal 58: 849–854.

Sasaji H (1983) Contribution to the taxonomy of the superfamily Cucujoidea (Coleoptera) of Japan and her adjacent districts, 1. Memoir of the Faculty of Education Fukui University (Series II: Natural Science) 33: 17–52.

Thomas MC (1988) Generic key to the known larvae of the Cucujidae, Passandridae, and Silvanidae of America North of Mexico (Coleoptera). Insecta Mundi 2 (2): 81–89.

Thomas MC (2002) 82. Cucujidae Latreille 1802 [pp. 329–330]. In: Arnett RH, Jr., Thomas MC, Skelley PE, Frank JH (Eds) American beetles. Volume 2. Polyphaga: Scarabaeoidea through Curculionoidea. CRC Press, New York, xiv + 861 pp.

Thomas MC (2004) A revision of *Pediacus* Shuckard (Coleoptera: Cucujidae) for America north of Mexico, with notes on other species. Insecta Mundi 17 (3/4): 157–177.

Vavra J (1993) Nové druhy brouků (Coleoptera) pro území Slovenska. New species of beetles (Coleoptera) for the territory of Slovakia. Klapalekiana 29 (1/2): 61–62.

Węgrzynowicz P (2007) Family Cucujidae Latreille, 1802 [pp. 502–503]. In: Löbl I, Smetana A (Eds) Catalogue of Palaearctic Coleoptera. Volume 4. Apollo Books, Stenstrup, 935 pp.

### Curculionidae

Supporting references for the conservation of Smicronychini Seidlitz, 1891 over Desmorini LeConte, 1876 (Art. 23.9.2). The taxon name Desmorini LeConte, 1876 has not been used as valid after 1899 to our knowledge.

Alonso-Zarazaga MA (2002) Lista preliminar de los Coleoptera Curculionoidea del área ibero-balear, con descripción de *Melicius* gen. nov. y nuevas citas. Boletín de la Sociedad Entomológica Aragonesa 31: 9–33.

Alonso-Zarazaga MA, Ugarte San Vicente I, Coello P (2009) Nuevos datos sobre la distribución de *Sharpia rubida* (Rosenhauer, 1856) (Coleoptera, Curculionidae, Smicronychini). Boletín de la Sociedad Entomológica Aragonesa 44: 573–574.

Anderson DM, Cox ML (1997) *Smicronyx* species (Coleoptera: Curculionidae), economically important seed predators of witchweeds (*Striga* spp.) (Scrophulariaceae) in sub-Saharan Africa. Bulletin of Entomological Research 87 (1): 3–17.

Anderson RS (2002) IV. Curculioninae Latreille, 1802 [pp. 732–740]. In: Arnett RH, Jr., Thomas MC, Skelley PE, Frank JH (Eds) American beetles. Volume 2. Polyphaga: Scarabaeoidea through Curculionoidea CRC Press, Boca Raton, xiv + 861 pp.

Angelov PA (1980) Coleoptera, Curculionidae. IV čast Calandrinae 2. [Coleoptera, Curculionidae. Part 4. Calandrinae. 2]. Fauna na B’lgariya 10. B’lgarska Akademia na Naukite, Sofia, 301 pp. [in Bulgarian].

Arnett RH, Jr. (2000) American insects. A handbook of the insects of America north of Mexico. Second edition. CRC Press, Boca Raton, xvii + 1003 pp.

Balsbaugh EU, Aarhus DG (1990) Checklist and new state records of Curculionidae (broad sense) (Coleoptera) for North Dakota. Journal of the Kansas Entomological Society 63(2): 227–236.

Bolu H, Legalov AA (2008) On the Curculionoidea (Coleoptera) fauna of almond (*Amygdalus communis* L.) orchards in south-eastern and eastern Anatolia in Turkey. Baltic Journal of Coleopterology 8(1): 75–85.

Braunert C (2009) Verzeichnis der Rüsselkäfer Luxemburgs (Coleoptera, Curculionoidea) mit Ausnahme der Borkenkäfer (Scolytinae) und Kernkäfer (Platypodinae). Bulletin de la Société des Naturalistes Luxembourgeois 110: 125–142.

Davis SR (2007) Nectarivory in a weevil, *Smicronyx squalidus* (Coleoptera: Curculionidae: Curculioninae), on *Desmanthus* (Fabaceae). Polish Journal of Entomology 76 (3): 221–224.

Franz NM (2006) Towards a phylogenetic system of derelomine flower weevils (Coleoptera: Curculionidae). Systematic Entomology 31 (2): 220–287.

Hoffmann A (1968) The scientific results of the Hungarian Soil Zoological Expedition to the Brazzaville-Congo. 32. Espèces de la famille Curculionidae (Coleoptera). Opuscula Zoologica 8(1): 11–29.

Hyder DE, Oseto CY (2005) Structure of the stridulatory apparatus and analysis of the sound produced by *Smicronyx fulvus* and *Smicronyx sordidus* (Coleoptera, Curculionidae, Erirrhininae, Smicronychini). Journal of Morphology 201(1): 69–84.

Li S, Sun L, Oseto CY, Ferris VR (2007) Phylogenetic analyses and a method for rapid molecular diagnosis of two sunflower seed weevils (Coleoptera: Curculionidae). Annals of the Entomological Society of America 100(5): 649–654.

Lyal CHC, Douglas DA, Hine SJ (2006) Morphology and systematic significance of sclerolepidia in the weevils (Coleoptera: Curculionoidea). Systematics and Biodiversity 4(2): 203–241.

Majka C, Anderson RS, McAlpine DF, Webster RP (2007) The weevils (Coleoptera: Curculionoidea) of the Maritime Provinces of Canada, I: New records from New Brunswick. The Canadian Entomologist 139(3): 378–396.

Marvaldi AE, Lanteri AA (2005) Key to higher taxa of South American weevils based on adult characters (Coleoptera, Curculionoidea). Revista Chilena de Historia Natural 78(1): 65–87.

Marvaldi AE, Morrone JJ (2000) Phylogenetic systematics of weevils (Coleoptera: Curculionoidea): a reappraisal based on larval and adult morphology. Insect Systematics & Evolution 31 (1): 43–58.

Morrone JJ (2000) Mexican weevils (Coleoptera: Curculionoidea): a preliminary key to families and subfamilies. Acta Zoologica Mexicana (Nueva Serie) 80: 131–141.

Pešic S (2003) Balkan weevils (Curculionoidea) in The Natural History Museum London (Reitter’s collection). Kragujevac Journal of Science 25: 163–172.

Piper GL, Mulford BL (1980) Life history observations on *Smicronyx commixtus* Dietz (Coleoptera: Curculionidae) in southeastern Washington. The Coleopterists Bulletin 34(3): 295–298.

Roseland CR, Bates MB, Oseto CY (1990) Role of a male-produced pheromone of the red sunflower seed weevil (Coleoptera: Curculionidae) in host finding. Environmental Entomology 19(6): 1675–1680.

Salas-Araiza MD, O’Brien CW, Romero-Napoles J (2001) Curculionoidea (Insecta: Coleoptera) from the state of Guanajuato, Mexico. Insecta Mundi 15 (1): 45–57.

Tanner VM (1966) Rhynchophora beetles of the Nevada Test Site. Brigham Young University Science Bulletin (Biological Series) 8(2): 1–35.

Wanat M, Mokrzycki T (2005) A new checklist of the weevils of Poland (Coleoptera: Curculionoidea). Genus 16: 69–117.

### Curculionidae

Supporting references for the conservation of Bagoinae C.G. Thomson, 1859 over Lyprinae Gistel 1848 (Art. 23.9.2). The taxon name Lyprinae Gistel 1848 has not been used as valid after 1899 to our knowledge. Note: Lyprinae Gistel, 1848 was recently considered a *nomen oblitum* by Colonnelli (2003: 7), however the necessary supporting references were not provided.

Alonso-Zarazaga MA, Lyal CHC (1999) A world catalogue of families and genera of Curculionoidea (Insecta: Coleoptera) (excepting Scolytidae and Platypodidae). Entomopraxis, S.C.P., Barcelona, 315 pp.

Alonso-Zarazaga MA, Lyal CHC (2002) Addenda and corrigenda to ‘A world catalogue of families and genera of Curculionoidea (Insecta: Coleoptera)’. Zootaxa 63: 1–37.

Amann E, Brandstetter CM, Kapp A (1994) Käfer am Wasser. (Gattungsschlussel der (semi-) aquatischen Käfer Mitteleuropas). Erster Vorarlberger Coleopterologischer Verein, Burs, i, 1–38

Anderson RS (2002) V. Bagoinae Thomson 1859 [p. 740]. In: Arnett RH, Jr., Thomas MC, Skelley PE, Frank JH (Eds) American beetles. Volume 2. Polyphaga: Scarabaeoidea through Curculionoidea. CRC Press, Boca Raton.

Angelov P (1989) Eine neue *Bagous*-Art aus Bulgarien (Insecta, Coleoptera, Curculionidae: Bagoinae). Reichenbachia 27 (1): 69–70.

Caldara R, O’Brien CW (1998) Systematics and evolution of weevils of the genus *Bagous*. VI. Taxonomic treatment of the species of the western Palearctic Region (Coleoptera Curculionidae). Memorie della Società Entomologica Italiana 76 [1997]: 131–347.

Colonnelli E (2003) A revised checklist of Italian Curculionoidea (Coleoptera). Zootaxa 337: 1–142.

DeLoach CJ (1976) Identification and biological notes on the species of *Neochetina* that attack Pontederiaceae in Argentina (Coleoptera: Curculionidae: Bagoini). The Coleopterists Bulletin 29 [1975] (4): 257–265.

Dieckmann L (1983) Beiträge zur Insektenfauna der DDR: Coleoptera - Curculionidae (Tanymecinae, Leptopiinae, Cleoninae, Tanyrhynchinae, Cossoninae, Raymondionyminae, Bagoinae, Tanysphyrinae). Beiträge zur Entomologie 33 (2): 257–381.

Dieckmann L (1990) Revision der mitteleuropäischen Arten der *Bagous collignensis*-Gruppe (Insecta, Coleoptera, Curculionidae: Bagoinae). Reichenbachia 27 (2): 141–145.

Freude H, Harde KW, Lohse GA (1983) Die Käfer Mitteleuropas. Band 11. Goecke & Evers, Krefeld, 342 pp.

Kissinger DG (1966) *Cyrtobagous* Hustache, a genus of weevils new to the United States fauna (Coleoptera: Curculionidae: Bagoini). The Coleopterists’ Bulletin 20 (4): 125–127.

Menet D (1997) Les curculionides du nord de la France. 7ème partie: sous-familles des Pissodinae, Anoplinae, Tanysphyrinae, Bagoinae et Cossoninae. Bulletin de la Société Entomologique du Nord de la France 285: 3–10.

Morris MG (1985) *Bagous brevis* Gyllenhal new to Ireland from the Burren, Co. Clare, with a brief review of the Irish Bagoini (Coleoptera: Curculionidae). Irish Naturalists’ Journal 21: 400–403.

O’Brien CW (1976) A taxonomic revision of the New World subaquatic genus *Neochetina* (Coleoptera: Curculionidae: Bagoini). Annals of the Entomological Society of America 69 (2): 165–174.

O’Brien CW, Askevold IS (1992) Systematics and evolution of weevils of the genus *Bagous* Germar (Coleoptera: Curculionidae), 1. Species of Australia. Transactions of the American Entomological Society 118 (4): 331–452.

O’Brien CW, Marshall GB (1979) U.S. *Bagous*, bionomic notes, a new species, and a new name (Bagoini, Erirhininae, Curculionidae, Coleoptera). Southwestern Entomologist 4 (2): 141–149.

Pajni HR, Kamal Tewari P (1986) On a new species of genus *Hydronomidius* (Bagoinae: Curculionidae) from India. Journal of the Bombay Natural History Society 82 [1985] (3): 610–613.

Pesarini C (1978) Tabelle per la determinazione dei generi di curculionidi italiani (Coleoptera). Informatore del Giovane Entomologo 19: 1–8.

Smreczyński S (1972) Ryjkowce-Curculionidae. Podrodzina Curculioninae. Klucze do Oznaczania Owadów Polski [Nr 77]. Część XIX. Chrząszcze – Coleoptera, Zeszyt 98d. Panstwowe Wydawnictwo Naukowe, Warszawa, 195 pp.

Sprick P (2000) Eignung einer Insektengruppe für die Fauna-Flora-Habitat-Richtlinie der EU (92/43/EWG, 21. Mai 1992) am Beispiel der Rüsselkäfer-Unterfamilie Bagoinae (Col., Curculionidae) (Beiträge zur Ökologie phytophager Käfer III). Insecta, Zeitschrift für Entomologie und Naturschutz 6: 61–96.

Sprick P (2001) Suitability of an insect group for the habitats directive of the EU: the weevil subfamily Bagoinae (Col., Curculionidae). Contributions to the ecology of phytophagous beetles VII. [pp. 7–40] In: Stüben PE (Ed) Studies on taxonomy, biology and ecology of Curculionoidea. Snudebiller 2. Curculio-Institut, Mönchengladbach [CD ROM].

Stresemann E, Hannemann H-J, Klausnitzer B, Senglaub K (1989) Exkursionsfauna für die Gebiete der DDR und der BRD. Band 2/1. Wirbellose: Insekten - Erster Teil. 8 Aufl. Volk und Wissen Volkseigener Verlag, Berlin, 504 pp.

Warner RE (1970) *Neochetina eichhorniae*, a new species of weevil from waterhyacinth, and biological notes on it and *N. bruchi* (Coleoptera: Curculionidae: Bagoini). Proceedings of the Entomological Society of Washington 72 (4): 487–496.

Zimmerman EC (1994) Australian weevils (Coleoptera: Curculionoidea). Volume 1. Orthoceri Anthribidae to Attelabidae, the primitive weevils. CSIRO, Melbourne, i-xxxii, pp. 1–741.

### Curculionidae

Supporting references for the conservation of Aterpina Lacordaire, 1863 over Heliomenina Gistel, 1848 (Art. 23.9.2). The taxon name Heliomenina Gistel, 1848 has not been used as valid after 1899 to our knowledge.

Alonso-Zarazaga MA, Lyal CHC (1999) A world catalogue of families and genera of Curculionoidea (Insecta: Coleoptera) (excepting Scolytidae and Platypodidae). Entomopraxis, S.C.P., Barcelona, 315 pp.

Barratt BIP, Evan AA, Ferguson CM, Barker GM, McNeill MR, Phillips CB (1997) Laboratory nontarget host range of the introduced parasitoids *Microctonus aethiopoides* and *M. hyperodae* (Hymenoptera: Braconidae) compared with field parasitism in New Zealand. Environmental Entomology 26(3): 694–702.

Elgueta M (1975) Una nueva especie de Aterpinae (Coleoptera. Curculionidae). Revista Chilena de Entomología 8 [1974]: 133–134.

Elgueta M (2000) Dos especies nuevas de *Aegorhinus* (Coleoptera: Curculionidae: Aterpini) de Chile. Two new *Aegorhinus* species (Coleoptera: Curculionidae: Aterpinae) from Chile. Acta Entomológica Chilena 24: 7–18.

Escalante T, Linaje M, Illoldi-Rangel P, Rivas M, Estrada P, Neira F, Morrone JJ (2009) Ecological niche models and patterns of richness and endemism of the southern Andean genus *Eurymetopum* (Coleoptera, Cleridae). Revista Brasileira de Entomologia 53(3): 379–385.

Hawkeswood TJ (1991) Review of the history, biology and host plants of the Australian weevil *Chrysolophus spectabilis* (Fabricius) (Coleoptera: Curculionidae: Aterpinae). Spixiana 14(1): 17–25.

Hunsdoerfer AK, Rheinheimer J, Wink M (2009) Towards the phylogeny of the Curculionoidea (Coleoptera): reconstructions from mitochondrial and nuclear ribosomal DNA sequences. Zoologischer Anzeiger 248 (1): 9–31.

Kuschel G (1982) Apionidae and Curculionidae (Coleoptera) from the Poor Knights Islands, New Zealand. Journal of the Royal Society of New Zealand 12(3): 273–282.

Leschen RAB, Lawrence JF, Kuschel G, Thorpe S, Wang Q (2003) Coleoptera genera of New Zealand. New Zealand Entomologist 26: 15–28.

Lyal CHC, Douglas DA, Hine SJ (2006) Morphology and systematic significance of sclerolepidia in the weevils (Coleoptera: Curculionoidea). Systematics and Biodiversity 4(2): 203–241.

Marvaldi AE, Morrone JJ (1999) Immature stages of *Rhyparonotus altarensis* (Olliff) (Coleoptera: Curculionidae: Molytinae), with comments on larval characters in Anchonini and Molytinae. Journal of the New York Entomological Society 106 [1998] (2/3): 95–104.

Marvaldi AE, Morrone JJ (2000) Phylogenetic systematics of weevils (Coleoptera: Curculionoidea): a reappraisal based on larval and adult morphology. Insect Systematics & Evolution 31 (1): 43–58.

May BM (1993) Larvae of Curculionoidea (Insecta: Coleoptera): a systematic overview. Fauna of New Zealand . Ko te Aitanga Pepeke o Aotearoa. Number 28, Manaaki Whenua Press, Lincoln, 225 pp.

Morrone JJ (1998) On Udvardy’s Insulantarctica province: a test from the weevils (Coleoptera: Curculionoidea). Journal of Biogeography 25 (5): 947–955.

Morrone JJ (2000) Mexican weevils (Coleoptera: Curculionoidea): a preliminary key to families and subfamilies. Acta Zoologica Mexicana (Nueva Serie) 80: 131–141.

Morrone JJ (2002) Checklist of the species of Cyclominae (Coleoptera: Curculionidae) occurring in America south of the United States. Revista de la Sociedad Entomológica Argentina 61 (1/2): 11–24.

Morrone JJ, Marvaldi AE (1998) *Listroderes abditus* or *Antarctobius abditus*?: a simultaneous analysis of larval and adult characters (Coleoptera: Curculionidae). European Journal of Entomology 95 (3): 429–436.

Morrone JJ, Roig-Junent S (2000) Synopsis and cladistics of the American Aterpini (Coleoptera: Curculionidae, Cyclominae). Entomologica Scandinavica 30 [1999] (4): 417–434.

Oberprieler RG, Marvaldi AE, Anderson RS (2007) Weevils, weevils, weevils everywhere. Zootaxa 1668: 491–520.

Parra LB, Mutis AT, Aguilera AP, Rebolledo RR, Quiroz AC (2009) Estado del conocimiento sobre el cabrito del frambueso (CF), *Aegorhinus superciliosus* (Guérin) (Coleoptera: Curculionidae). Idesia 27(1): 57–65.

Pérez-Schultheiss J, Arriagada AM, Baessolo L (2009) Aterpini (Coleoptera: Curculionidae) del Parque Nacional Isla Guamblín, Archipiélago de los Chonos, Aysén, Chile. Boletín de la Sociedad Entomológica Aragonesa 45: 249–252.

Posadas P, Morrone JJ (2003) Biogeografía histórica de la familia Curculionidae (Coleoptera) en las subregiones Subantártica y Chilena Central. Revista de la Sociedad Entomológica Argentina 62(1/2): 75–84.

Richardson BJ, Oberprieler RG (2007) The diversity of Linnaean communities: a way of detecting invertebrate groups at risk of extinction. Journal of Insect Conservation 11(3): 287–297.

Setliff GP (2007) Annotated checklist of weevils from the Papuan region (Coleoptera, Curculionoidea). Zootaxa 1536: 1–296.

Vergara OE, Jerez V, Parra LE (2006) Diversidad y patrones de distribución de coleópteros en la Región del Biobío, Chile: una aproximación preliminar para la conservación de la diversidad. Revista Chilena de Historia Natural 79: 369–388.

### Curculionidae

Supporting references for the conservation of Naupactini Gistel, 1848 over Iphiini Schönherr, 1823 (Art. 23.9.2). The taxon name Iphiini Schönherr, 1823 has not been used as valid after 1899 to our knowledge.

Bordon C (1991) El género *Macrostylus* Boheman (Col. Curc. Brachyderinae, Naupactini) en Venezuela. Acta Biologica Venezuelica 13: 1–50.

Chen Y (1991) [The Chinese *Mesagroicus* Schönherr (Coleoptera: Curculionidae, Naupactini)]. Acta Entomologica Sinica 34 (4): 468–469 [in Chinese].

Coscarón M, del C, Díaz NB, Lanteri AA, Loiacono MS (1991) Importancia taxonómica del revestimiento tegumentario en la tribu Naupactini. I. Género *Cyrtomon* y taxa afines (Coleoptera: Curculionidae). Neotropica 37 (No. 97): 31–54.

Díaz NB, Loiácono MS, Coscarón M, del C, Lanteri AA (1990) Importancia taxonómica de las piezas bucales en la tribu Naupactini. 1. Género *Cyrtomon* Schoenherr y taxa afines (Coleoptera, Curculionidae). Revista Brasileira de Entomologia 34 (4): 861–876.

Díaz NB, Loiácono MS, Lanteri AA, Coscarón M, del C (1991) Importancia taxonómica de la piezas bucales en la tribu Naupactini. II. Las especies del género *Cyrtomon* Schoenherr (Coleoptera: Curculionidae). Neotropica 36 (No. 96) [1990]: 93–99.

Ferragu M, Richard R (1986) Cinq curculionides nouveaux de Madagascar, des Mascareignes et d’Afrique australe (Coleoptera). Revue Française d’Entomologie (Nouvelle Série) 8 (4): 149–155.

Gomez CA, Lanteri AA (2006) Primer registro de *Naupactus ruizi* (Coleoptera: Curculionoidea) asociado con *Pinus ponderosa* (Gymnospermae: Pinaceae) en Patagonia. Revista de la Sociedad Entomológica Argentina 65: 107–109.

Guadalupe del Río M, Lanteri AA (2007) Taxonomic revision of *Melanocyphus* Jekel (Coleoptera: Curculionidae). Studies on Neotropical Fauna and Environment 42: 127–132.

Guadalupe del Río M, Lanteri AA (2008) *Thoraconaupactus*, a new Brazilian genus of broad nosed weevil (Coleoptera: Curculionidae: Entiminae) associated with *Leucaena* (Fabaceae). Entomological News 118 [2007] (5): 459–469.

Guadalupe del Río M, Lanteri AA, Guedes JVC (2006) Taxonomic revision and cladistic analysis of *Teratopactus* Heller (Coleoptera: Curculionidae). Invertebrate Systematics 20 (5): 585–602.

Guedes JVC, Lanteri AA, Parra JRP (2005) Chave de identificação, ocorrência e distribuição dos curculionídeos-das-raízes dos citros em São Paulo e Minas Gerais. Neotropical Entomology 34 (4): 577–584.

Lanteri AA (1981) Estudio comparativo de las estructuras genitales en la tribu Naupactini. I. Los caracteres a nivel genérico de *Naupactus* Schönherr, *Teratopactus* Heller y *Trichonaupactus* Hustache (Coleoptera: Curculionidae). Revista de la Sociedad Entomológica Argentina 40 (1/4): 273–278.

Lanteri AA (1989) Estudio sistemático de los géneros *Trichocyphus* Heller y *Mendozella* Hustache (Coleoptera: Curculionidae). Boletín de la Sociedad de Biología de Concepción 60: 139–147.

Lanteri AA (1990) Revisión sistemática del género *Priocyphus* Hustache 1939 y creación de los géneros *Priocyphopsis* y *Lamprocyphopsis* (Coleoptera, Curculionidae). Revista Brasileira de Entomologia 34 (2): 403–422.

Lanteri AA (1992) Systematics, cladistics and biogeography of a new weevil genus, *Galapaganus* (Coleoptera: Curculionidae) from the Galápagos Islands, and coasts of Ecuador and Perú. Transactions of the American Entomological Society 118 (3): 227–267.

Lanteri AA (1995) Systematic revision of *Ericydeus* Pascoe (Coleoptera: Curculionidae). Entomologica Scandinavica 26 (4): 393–424.

Lanteri AA (2004) New taxonomic and biogeographic information on *Galapaganus femoratus* species group (Coleoptera: Curculionidae: Entiminae). Transactions of the American Entomological Society 130 (2/3): 177–192.

Lanteri AA, Guadalupe del Río M (2003) Revision of the genus *Briarius* (Fischer de Waldheim) (Coleoptera: Curculionidae). Insect Systematics & Evolution 34 (3): 281–294.

Lanteri AA, Guadalupe del Río M (2005) Taxonomic revision of *Thoracocyphus* Emden (Coleoptera: Curculionidae). Insect Systematics & Evolution 35 (4): 449–456.

Lanteri AA, Guadalupe del Río M (2005) Taxonomy of the monotypic genus *Trichaptus* Pascoe (Coleoptera: Curculionidae: Entiminae), a potential weevil mimic of Mutillidae. The Coleopterists Bulletin 59 (1): 47–54.

Lanteri AA, Guadalupe del Río M (2006) Taxonomic revision of the genus *Cyphopsis* Roelofs (Coleoptera, Curculionidae). Deutsche Entomologische Zeitschrift 53 (2): 275–281.

Lanteri AA, Guadalupe del Río M (2006) Taxonomic revision of the monotypic genus *Acyphus* Heller (Coleoptera: Curculionidae) with comments on infraspecific variation. Zootaxa 1312: 59–68.

Lanteri AA, Guedes JVC, Parra JRP (2002) Weevils injurious for roots of citrus in São Paulo state, Brazil. Neotropical Entomology 31 (4): 561–569.

Lanteri AA, Morrone JJ (1991) Cladistic analysis of *Priocyphis* Hustache and related genera (Coleoptera: Curculionidae). Proceedings of the Entomological Society of Washington 93 (2): 278–287.

Lanteri AA, Normark BB (1995) Parthenogenesis in the tribe Naupactini (Coleoptera: Curculionidae). Annals of the Entomological Society of America 88 (6): 722–731.

Marvaldi AE, Loiacono MS (1994) First instar larvae in the tribe Naupactini (Coleoptera, Curculionidae). Revista Brasileira de Entomologia 38 (2): 453–466.

Normark BB (1996) Phylogeny and evolution of parthenogenetic weevils of the *Aramigus tessellatus* species complex (Coleoptera: Curculionidae: Naupactini): evidence from mitochondrial DNA sequences. Evolution 50 (2): 734–745.

Pesarini C (1977) Tabelle per la determinazione dei generi di curculionidi italiani (Coleoptera). 2. L’Informatore del Giovane Entomologo 18: 9–16.

Wibmer GJ, O’Brien CW (1986) Annotated checklist of the weevils (Curculionidae sensu lato) of South America (Coleoptera: Curculionoidea). Memoirs of the American Entomological Institute No. 39: i-xvi, 1–563.

### Curculionidae

Supporting references for the conservation of Cleonini Schönherr, 1826 over Geomorini Schönherr, 1823 (Art. 23.9.2). The taxon name Geomorini Schönherr, 1823 has not been used as valid after 1899 to our knowledge.

Alonso Zarazaga MA, Lyal CHC (2009) On the nomenclature of some genera in the Cleonini (Coleoptera, Curculionidae, Lixinae). Graellsia 65(2): 241–242.

Alonso-Zarazaga MA, Lyal CHC (1999) A world catalogue of families and genera of Curculionoidea (Insecta: Coleoptera) (excepting Scolytidae and Platypodidae). Entomopraxis, S.C.P., Barcelona, 315 pp.

Anderson RS (1988) Systematics, phylogeny and biogeography of New World weevils traditionally of the tribe Cleonini (Coleoptera: Curculionidae; Cleoninae). Quaestiones Entomologicae 23 [1987] (4): 431–709.

Anderson RS (1989) New synonymy in North American *Stephanocleonus* Motschulsky (Coleoptera: Curculionidae). The Coleopterists Bulletin 43 (1): 93.

Arzanov YG (2006) *Borisocleonus* gen. n. – novyy rod dolgonosikov triby Cleonini (Coleoptera: Curculionidae: Lixinae). *Borisocleonus* gen. n. - a new genus of weevils from the tribus of Cleonini (Coleoptera: Curculionidae: Lixinae). Russian Entomological Journal 15 (1): 63–66.

Aslam NA (1963) On the genera of Indo-Pakistan Cleoninae and Hylobiinae (Coleoptera: Curculionidae). Bulletin of the British Museum (Natural History) (Entomology) 13 (3): 47–66.

Balsbaugh EU, Aarhus DG (1990) Checklist and new state records of Curculionidae (broad sense) (Coleoptera) for North Dakota. Journal of the Kansas Entomological Society 63(2): 227–236.

Barratt BIP, Evan AA, Ferguson CM, Barker GM, McNeill MR, Phillips CB (1997) Laboratory nontarget host range of the introduced parasitoids *Microctonus aethiopoides* and *M. hyperodae* (Hymenoptera: Braconidae) compared with field parasitism in New Zealand. Environmental Entomology 26(3): 694–702.

Brun LA, Sheppard AW, Carrara A (1994) Host range of *Pachycerus cordiger* (= *P. scabrosus*) (Col.: Curculionidae). Entomophaga 38[1993] (4): 537–539.

Dieckmann L (1983) Beiträge zur Insektenfauna der DDR: Coleoptera Curculionidae (Tanymecinae, Leptopiinae, Cleoninae, Tanyrhynchinae, Cossoninae, Raymondionyminae, Bagoinae, Tanysphyrinae). Beiträge zur Entomologie 33 (2): 257–381.

Gratshev VG, Zherichin VV (2003) The fossil record of weevils and related beetle families (Coleoptera, Curculionoidea). Acta Zoologica Cracoviensia 46 (Supplement. Fossil Insects): 129–138.

Holecová M, Rozek M, Karagyan G (2000) Karyological notes on three weevil species from Armenia (Coleoptera, Curculionidae, Cleoninae). Folia Biologica (Cracow) 48(1–2): 25–27.

Hunsdoerfer AK, Rheinheimer J, Wink M (2009) Towards the phylogeny of the Curculionoidea (Coleoptera): reconstructions from mitochondrial and nuclear ribosomal DNA sequences. Zoologischer Anzeiger 248 (3): 9–31.

Kingsolver JM (1962) Notes on fossil Cleoninae (Coleoptera: Curculionidae). Psyche 69 (1): 47–49.

Louw S (1993) Breeding populations of *Lixus carinerostris* Boheman and *Calodemas prolixus* Faust (Coleoptera: Curculionidae) co-existing on Mesembryanthemaceae. The Coleopterists Bulletin 47(4): 335–339.

Meregalli M (2002) Notes on the genus *Pachycerus* Schoeherr 1823 with description of a new species from Somalia (Coleoptera Curculionidae Lixinae). Tropical Zoology 15 (2): 233–242.

Meregalli M (2004) *Pseudeumecops* n. gen., a new genus of Cleonini from East Africa (Coleoptera, Curculionidae, Lixinae). Italian Journal of Zoology 71 (2): 153–163.

Meregalli M (2008) Taxonomic relationships between *Pachycerus* and *Rhabdorrhynchus* (Coleoptera: Curculionidae: Lixinae), with descriptions of two new species of *Rhabdorrhynchus* from the Arabian Peninsula. Zoological Journal of the Linnean Society 152(1): 25–37.

Meregalli M (2009) Revision of the Indo-African *Pachycerus* Schoenherr, 1823, with a description of four new species (Coleoptera: Curculionidae: Lixinae). Zoological Journal of the Linnean Society 157(2): 295–325.

Perrin HM, Meregalli M (2008) Désignation de lectotypes des espèces de Cleonini, décrites par Gebler et Chevrolat, dans les collections du MNHN à Paris (Coleoptera, Curculionidae, Lixinae). Revue Française d’Entomologie (Nouvelle Série) 29 [2007] (4): 129–148.

Takenouchi Y (1973) A revised study of the chromosomes of twenty-four species of Japanese weevils (Coleoptera: Curculionidae). Genetica 44(4): 621–632.

Volovnik SV (1989) On distribution and ecology of some species of cleonine weevils (Coleoptera: Curculionidae) I. Tribe Cleonini. Entomological Review 68(3): 138–144.[publ. originally in *Entomologicheskoe Obozrenie* 68 (1): 86–92]

Volovnik SV (2005) On parasites and predators of Cleoninae weevils (Col. Curculionidae) in Ukrainian steppe. Anzeiger für Schädlingskunde 67(4): 77–79.

Volovnik SV (2007) On distribution and ecology of some species of cleonines (Coleoptera, Curculionidae): IV. Genus *Lixus* F., subgenus *Eulixus* Reitt. Entomological Review 87 (7): 840–847. [publ. originally in *Entomologicheskoe Obozrenie* 86 (3): 521–531]

Wheeler AG, Jr., Whitehead DR (1985) *Larinus planus* (F.) in North America (Coleoptera: Curculionidae: Cleoninae) and comments on biological control of Canada thistle. Proceedings of the Entomological Society of Washington 87(4): 751–758.

### Curculionidae

Supporting references for the conservation of Magdalidini Pascoe, 1870 over Scardamyctini Gistel, 1848 (Art. 23.9.2). The taxon name Scardamyctini Gistel, 1848 has not been used as valid after 1899 to our knowledge.

Alonso-Zarazaga MA (2002) Lista preliminar de los Coleoptera Curculionoidea del área ibero-balear, con descripción de *Melicius* gen. nov. y nuevas citas. Boletín de la Sociedad Entomológica Aragonesa 31: 9–33.

Alonso-Zarazaga MA, Lyal CHC (1999) A world catalogue of families and genera of Curculionoidea (Insecta: Coleoptera) (excepting Scolytidae and Platypodidae). Entomopraxis, S.C.P., Barcelona, 315 pp.

Anderson RS (1997) Weevils (Coleoptera: Curculionoidea, excluding Scolytinae and Platypodinae) of the Yukon [pp. 523–562]. In: Danks HV, Downes JA (Eds) Insects of the Yukon. Biological Survey of Canada (Terrestrial Arthropods), Ottawa, 1034 pp.

Anderson RS (2002) XV. Mesoptiliinae Lacordaire, 1863 [p. 786]. In: Arnett RH, Jr., Thomas MC, Skelley PE, Frank JH (Eds) American Beetles. Volume 2. Polyphaga: Scarabaeoidea through Curculionoidea CRC Press, Boca Raton, xiv + 861 pp.

Balsbaugh EU, Aarhus DG (1990) Checklist and new state records of Curculionidae (broad sense) (Coleoptera) for North Dakota. Journal of the Kansas Entomological Society 63(2): 227–236.

Braunert C (2009) Verzeichnis der Rüsselkäfer Luxemburgs (Coleoptera, Curculionoidea) mit Ausnahme der Borkenkäfer (Scolytinae) und Kernkäfer (Platypodinae). Bulletin de la Société des Naturalistes Luxembourgeois 110: 125–142.

Bright DE, Bouchard P (2008) Coleoptera, Curculionidae, Entiminae. Weevils of Canada and Alaska. Volume 2. The Insects and Arachnids of Canada. Part 25, NRC Research Press, Ottawa, xiii + 327 pp.

Burrini AG, Magnano L, Magnano AR, Scala C, Baccetti B (1988) Spermatozoa and phylogeny of Curculionoidea (Coleoptera). International Journal of Insect Morphology and Embryology 17(1): 1–50.

Crowson RA (1984) The associations of Coleoptera with Ascomycetes [pp. 256–285]. In: Wheeler Q, Blackwell M (Eds) Fungus-insect relationships: perspectives in ecology and evolution. Columbia University Press, New York, xiii + 514 pp.

Elgueta M, Arriagada SG (1989) Estado actual del conocimiento de los Coleópteros de Chile (Insecta: Coleoptera). Revista Chilena de Entomología 17: 5–60.

Hollis D (Ed), (1980) Animal identification: a reference guide. Volume 3. Insects. London, British Museum (Natural History) and John Wiley, viii + 160 pp.

Hunsdoerfer AK, Rheinheimer J, Wink M (2009) Towards the phylogeny of the Curculionoidea (Coleoptera): reconstructions from mitochondrial and nuclear ribosomal DNA sequences. Zoologischer Anzeiger 248 (1): 9–31.

Kojima H, Morimoto K (2004) An online checklist and database of the Japanese weevils (Insecta: Coleoptera: Curculionoidea) (excepting Scolytidae and Platypodidae). Bulletin of the Kyushu University Museum 2: 33–147.

Kuschel G (2003) Nemonychidae, Belidae, Brentidae (Insecta: Coleoptera: Curculionoidea). Fauna of New Zealand. Ko te Aitanga Pepeke o Aotearoa. Number 45, Manaaki Whenua Press, Lincoln, 97 pp.

Lu X, Legalov AA, Zhang R (2005) A new subgenus and two new species of *Magdalis* Germar from northern China (Coleoptera: Curculionidae: Magdalinae). The Coleopterists Bulletin 59(3): 369–378.

Majka C, Anderson RS, McAlpine DF, Webster RP (2007) The weevils (Coleoptera: Curculionoidea) of the Maritime Provinces of Canada, I: New records from New Brunswick. The Canadian Entomologist 139(3): 378–396.

Mann F. G. (1960) Regiones biogeográficas de Chile. Investigaciones Zoológicas Chilenas 6: 15–49.

McKenna DD, Sequeira AS, Marvaldi AE, Farrell BD (2009) Temporal lags and overlap in the diversification of weevils and flowering plants. Proceedings of the National Academy of Sciences of the United States of America 106(17): 7083–7088.

Morris MG (1993) A check list of the weevils of Ireland (Coleoptera: Curculionoidea). Entomologist’s Gazette 44 (4): 289–296.

Morris MG (2002) True weevils (part I): Coleoptera: Curculionidae (subfamilies Raymondionyminae to Smicronychinae). Handbooks for the Identification of British Insects 5(17b): 1–149.

Morrone JJ, Muñiz R, Asiain J, Márquez J (2002) Lista de las especies de Curculionoidea (Insecta: Coleoptera) depositadas en la colección del Museo de Zoología ‘Alfonso L. Herrera,’ Facultad de Ciencias, UNAM (MZFC). Acta Zoologica Mexicana (Nueva serie) 87: 147–165.

O’Brien CW, Wibmer GJ (1982) Annotated checklist of the weevils (Curculionidae sensu lato) of North America, Central America, and the West Indies (Coleoptera: Curculionoidea). Memoirs of the American Entomological Institute 34: i-ix, 1–382.

Pešic S (2003) Balkan weevils (Curculionoidea) in The Natural History Museum London: world part. Kragujevac Journal of Science 25: 139–162.

Ribera I (1999) Evolución, filogenia y clasificación de los Coleoptera (Arthropoda: Hexapoda). Boletín de la Sociedad Entomológica Aragonesa 26(Evolución y filogenia de Arthropoda): 435–458.

Wanat M, Mokrzycki T (2005) A new checklist of the weevils of Poland (Coleoptera: Curculionoidea). Genus 16 (1): 69–117.

### Elateridae

Supporting references for the conservation of Agrypninae/-ini Candèze, 1857 over Adelocerinae/-ini Gistel, 1848 and Pangaurinae/-ini Gistel, 1856 (Art. 23.9.2). The taxon name Pangaurinae/-ini Gistel, 1856 has not been used as valid after 1899 to our knowledge. The taxon name Adelocerinae/-ini has been used as valid after 1899 but attributed to Buysson, 1893 who proposed the name as new, without reference to Gistel’s name.

Casari SA (2002) Review of the genus *Chalcolepidius* Eschscholtz, 1829 (Coleoptera, Elateridae, Agrypninae). Revista Brasileira de Entomologia 46 (3): 263–428.

Casari SA (2004) New species of *Alaus* Eschscholtz, 1829 (Coleoptera: Elateridae, Agrypninae, Hemirhipini). Annales de la Société Entomologique de France (Nouvelle Série) 39 [2003] (4): 315–333.

Casari SA (2006) Larva, pupa and adult of *Aeolus cinctus* Candèze (Coleoptera, Elateridae, Agrypninae). Revista Brasileira de Entomologia 50 (3): 347–351.

Casari SA (2008) Cladistic analysis of Hemirhipini with establishment of *Propalaus* gen. nov. (Coleoptera, Elateridae, Agrypninae). Papéis Avulsos de Zoologia 48 (16): 139–180.

Chakraborty P, Chakraborty S (2000) Agrypninae (Coleoptera: Elateridae) of India: a taxonomic review and checklist. Records of the Zoological Survey of India 98: 71–83.

Chassain J (2005) *Lacon giuglarisi*, espèce nouvelle pour la fauna de Guyane française (Coleoptera, Elateridae, Agrypninae). L’Entomologiste 61 (3): 131–134.

Chassain J (2009) Description d’une nouvelle espèce guyanaise et brésilienne d’élatéride du genre *Coctilelater* (Costa, 1975) (Coleoptera Elateridae Agrypninae Pyrophorini). L’Entomologiste 65 (1): 35–38.

Cobos A (1966) Nuevo género y especie de Agrypninae (Col. Elateridae). Annales de la Société Entomologique de France (Nouvelle Série) 2 (3): 651–653.

Costa C, Cesari-Chen SA (1984) Larvae of Neotropical Coleoptera. VIII. Elateridae: Agrypninae, Elaterinae and Physorhininae. Revista Brasileira de Entomologia 28 (3): 315–328.

Costa C, Rosa SP, Chassain J (2009) A taxonomic revision and revalidation of *Nycterilampus* Montrouzier (Coleoptera: Elateridae, Agrypninae). Zootaxa 2017: 47–57.

Hoffman RL (2007) The distribution of *Conoderus scissus* (Schaeffer) with notes on some taxonomic characters (Coleoptera: Elateridae: Agrypninae). Banisteria 30: 32–34.

Jiang S-H, Chen X-Q, Wu S-J, Meng Z-Y, Li G-J (2009) Molecular phylogenetic analysis of Elateridae (Insecta: Coleoptera) based on 28S rDNA gene fragments. Acta Entomologica Sinica 52 (1): 74–83 [in Chinese, English summary].

Kishii T (1995) A study on the elaterid-beetles of Shibata Collection from Taiwan, I (Coleoptera, Elateridae). On the subfamilies Oxynopterinae and Agrypninae. The Entomological Review of Japan 50 (1): 1–14, pls. 1–4.

Lee S-H, Woo K-S, Lee YI (1987) Taxonomic study on the subfamily Agrypninae (Coleoptera: Elateridae) in Korea. Korean Journal of Plant Protection 26: 1–7.

Ohira H (2002) Notes on the morphological structure of *Agrypnus* species from Japan (I). (Coleoptera: Elateridae, Agrypninae, *Agrypnus*). Miscellaneous Reports of the Hiwa Museum for Natural History 41: 53–67.

Ohira H (2004) Notes on the morphological structure of *Agrypnus* species from Japan (III). (Coleoptera: Elateridae, Agrypninae, *Agrypnus*, *Colaulon* group). Miscellaneous Reports of the Hiwa Museum for Natural History 43: 67–90.

Platia G (2008) Description of three wingless species of *Rismethus* Fleutiaux, 1947 (Coleoptera, Elateridae: Agrypnini) from Western Malaysia. Folia Entomologica Hungarica 69: 179–184.

Rosa SP (2004) Nova espécie do gênero *Ptesimopsia* Costa (Coleoptera, Elateridae, Agrypninae). Revista Brasileira de Entomologia 48 (2): 227–228.

Rosa SP (2004) Revisão do gênero *Opselater* Costa (Coleoptera, Elateridae, Agrypninae). Revista Brasileira de Entomologia 48 (2): 203–219.

Rosa SP (2009) Description of *Coctilelater minimus* sp. nov. (Coleoptera, Elateridae, Agrypninae). Revista Brasileira de Entomologia 53 (1): 36–37.

Smith JW, Enns WR (1977) The click beetle subfamilies Agrypninae, Pyrophorinae, and Melanotinae (Coleoptera: Elateridae) in Missouri - part I. Journal of the Kansas Entomological Society 50 (3): 436–468.

Smith JW, Enns WR (1978) The click beetle subfamilies Agrypninae, Pyrophorinae, and Melanotinae (Coleoptera: Elateridae) in Missouri - part II. Journal of the Kansas Entomological Society 51 (1): 42–74.

Stibick JNL (1979) Classification of the Elateridae (Coleoptera). Relationships and classification of the subfamilies and tribes. Pacific Insects 20 (2/3): 145–186.

Tarnawski D, Buchholz L (2008) Sprezykowate -Elateridae. Część ogolna oraz podrodziny: Agrypninae, Negastriinae i Diminae. Klucze do Oznaczania Owadów Polski [Nr. 172]. Część XIX. Chrząszcze - Coleoptera. Zeszyt 34a. Polskie Towarzystwo Entomologiczne, Warszawa, 125 pp.

Vats LK, Vasu V (1993) Taxonomic importance of internal organs of reproduction in Agrypninae (Coleoptera: Elateridae). Journal of Entomological Research 17 (1): 65–70.

### Elateridae

Supporting references for the conservation of Prosternini Gistel, 1856 over Diacanthini Gistel, 1848 (Art. 23.9.2). The taxon name Diacanthini Gistel, 1848 has not been used as valid after 1899 to our knowledge.

Barševskis A (2005) Catalogue of click-beetles (Coleoptera: Elateridae) of Latvia. Proceedings on Taxonomy and Faunistics of Beetles (Coleoptera) dedicated to the 100th birthday of the Latvian entomologist Mihails Stiprais (1905–1990). Pp. 7–28.

Bishop DJ, Majka CG, Bondrup-Nielsen S, Peck SB (2009) Deadwood and saproxylic beetle diversity in naturally disturbed and managed spruce forests in Nova Scotia. ZooKeys 22: 309–340.

Casari SA (2008) A phylogenetic study of the subtribe Dicrepidiina (Elateridae, Elaterinae, Ampedini). Revista Brasileira de Entomologia 52 (2): 182–260.

Johnson PJ (1995) A new genus of Conoderini, with new generic classifications for *Ctenicera sleeperi* Becker and *Ctenicera pilatei* (Champion), and a new species from Jamaica (Coleoptera: Elateridae). The Coleopterists Bulletin 49 (1): 59–71.

Johnson PJ (2002a) 58. Elateridae Leach 1815 [pp. 160–173]. In: Arnett RH, Jr., Thomas MC, Skelley PE, Frank JH (Eds) American beetles. Volume 2. Polyphaga: Scarabaeoidea through Curculionoidea. CRC Press, Boca Raton, xiv + 861 pp.

Johnson PJ (2002b) Lectotype designations for Elateridae (Coleoptera) described by George C. Champion in the Biologia Centrali-Americana. Dugesiana 9 (1): 15–46.

Kalniņš M, Juceviča E, Karpa A, Salmane I, A. P, Telnov D (2007) Bezmugurkaulnieki [pp. 106–151]. In: Pilāts V (Ed) Biologiskā Daudzveidība Gaujas Nacionālajā Parkā Biodiversity in Gauja National Park. Gaujas nacionālā parka administrācija, Sigulda.

Kishii T (1997) A study on the elaterid-beetles of Shibata collection from Taiwan, VI. (Coleoptera: Elateridae). On the subfamily Denticollinae: Tribe Prosternini. The Entomological Review of Japan 52 (1): 15–18.

Kishii T (2004) Two new species of the subfamily Dendrometrinae (Coleoptera: Elateridae) from Japan. The Entomological Review of Japan 59 (1): 61–66.

Kishii T, Jiang S-H (1996) Notes on the Chinese Elateridae (3) (Coleoptera). Entomological Review of Japan 51 (2): 97–102.

Kishii T, Jiang S-H (1999) Notes on the Chinese Elateridae (Coleoptera) (4). Entomological Review of Japan 54 (1): 11–19.

Lundberg B (1995) Catalogus Coleopterorum Sueciae. Naturhistoriska Riksmuseet, Stockholm [unpaginated].

Majka CG, Johnson PJ (2008) The Elateridae (Coleoptera) of the Maritime Provinces of Canada: faunal composition, new records, and taxonomic changes. Zootaxa 1811: 1–33.

Ohira H (2007) Notes on the morphological structure of *Actenicerus* species (Coleoptera: Elateridae, Dendrometrinae, Prosternini) from Japan (8). Nanki Seibutu 49: 131–134.

Ohira H (2008) Notes on the morphological structure of *Actenicerus* species (Coleoptera: Elateridae, Dendrometrinae, Prosternini) from Japan (9). Nanki Seibutu 50: 79–81.

Recalde JI, Sánchez-Ruiz A (2003) Elateridae (Coleoptera) forestales de Navarra (III). Acerca de la presencia del género *Paraphotistus* Kishii, 1966 en la Península Ibérica. Boletín de la Sociedad Entomológica Aragonesa 32: 95–97.

Sánchez-Ruiz A (1996) Catalogo bibliográfico de las especies de la familia Elateridae (Coleoptera) de la Península Iberica e Islas Baleares. Documentos Fauna Ibérica, 2. Museo Nacional de Ciencias Naturales, CSIC, Madrid, 265 pp.

Sánchez-Ruiz A (1998) Familia Elateridae (Insecta: Coleoptera). Catalogus de la Entomofauna Aragonesa 17: 13–17.

Sánchez-Ruiz A (1999) Los Elateridae (Coleoptera) de Los Monegros. Boletín de la Sociedad Entomológica Aragonesa 24 [1998]: 167–168.

Sánchez-Ruiz A, Muñoz J, Blasco-Zumeta J (1998) Nuevos datos para la fauna de Elateridae (Coleoptera) de Aragón. Boletín de la Sociedad Entomológica Aragonesa 22: 13–15.

Schneider MC, Rosa SP, Almeida MC, Costa C, Cella DM (2007) Strategies of karyotype differentiation in Elateridae (Coleoptera, Polyphaga). Micron 38 (6): 590–598.

Silfverberg H (1992) Enumeratio Coleopterorum Fennoscandiae, Daniae et Baltiae. Helsingfors Entomologiska Bytesförening, Helsinki, v + 94 pp.

Telnov D (2004) Check-list of Latvian beetles (Insecta: Coleoptera). Second edition. Compendium of Latvian Coleoptera 1. Entomological Society of Latvia, Rïga, 113 pp.

Telnov D, Barsevskis A, Savich F, Kovalevsky F, Berdnikov S, Doronin M, Cibulskis R, Ratniece D (1997) Check-list of Latvian beetles (Insecta: Coleoptera). Mitteilungen des Internationalen Entomologischen Vereins, Supplement 5: 1–141.

Zapata de la Vega JL, Sánchez-Ruiz A (2002) El género *Selatosomus* Stephens, 1830 en la península Ibérica (Coleoptera: Elateridae: Dendrometrinae: Prosternini). Boletín de la Sociedad Entomológica Aragonesa 30: 101–110.

### Monotomidae

Supporting references for the conservation of *Monotoma* Herbst, 1793 over *Monotoma* Panzer, 1792 (Art. 23.9.2). The taxon name *Monotoma* Panzer, 1792 has not been used as valid after 1899 to our knowledge.

Arnett RH, Jr. (1962b) Part V Suborder Polyphaga (cont.) series Cucujiformia (cont.) Tenebrionoidea, Cucujoidea [pp. (2 unn. +) 645–850]. In: The beetles of the United States (a manual for identification) [original loose-leaf edition]. Catholic University of America Press, Washington D.C., xii + 1112 pp. [+ unn. pp.].

Arnett RH, Jr. (2000) American insects. A handbook of the insects of America north of Mexico. Second edition. CRC Press, Boca Raton, xvii + 1003 pp.

Bousquet Y (1991) Family Rhizophagidae rhizophagid beetles [pp. 218–219]. In: Bousquet Y (Ed) Checklist of beetles of Canada and Alaska. Agriculture Canada, Publication 1861/R, 430 pp.

Bousquet Y (2002) Family 79. Monotomidae Laporte 1840 [pp. 319–321]. In: Arnett RH, Thomas MC, Skelley PE, Frank JH (Eds) American beetles. Volume 2. Polyphaga: Scarabaeoidea through Curculionoidea. CRC Press, Boca Raton, xiv + 861 pp.

Bousquet Y (2010) Monotomidae Laporte, 1840 [pp. 319–324]. In: Part 38. Coleoptera, beetles. Volume 2: Morphology and systematics (Elateroidea, Bostrichiformia, Cucujiformia partim). In: Leschen RAB, Beutel RG, Lawrence JF, Ślipiński SA (Eds) Handbook of zoology. Volume IV. Arthropoda: Insecta. W. de Gruyter, New York and Berlin, xiii + 786 pp.

Bousquet Y, Laplante S (2000) Taxonomic review of the Canadian species of the genus *Monotoma* Herbst (Coleoptera: Monotomidae). Proceedings of the Entomological Society of Ontario 130 [1999]: 67–96.

De Marzo L (2005) Una peculiarità anatomica del dotto eiaculatore in *Monotoma* Herbst, 1793 (Insecta Coleoptera Rhizophagidae). Naturalista Siciliano (4) 29 (1/2): 67–70.

Downie NM, Arnett RH, Jr. (1996) The beetles of northeastern North America. Volume II: Polyphaga: Series Bostrichiformia through Curculionoidea. Sandhill Crane Press, Gainesville, pp. i-x, 891–1721.

Hansen M, Kristensen S (1991) To nye danske biller af slaegten *Monotoma* Herbst (Coleoptera, Monotomidae). Entomologiske Meddelelser 59 (2): 41–44.

Holzschuh C, Lohse GA (1981) Eine neue Art der Gattung *Monotoma* Herbst aus Mitteleuropa: *Monotoma gotzi* n. sp. Entomologische Blätter für Biologie und Systematik der Käfer 77 (3): 175–177.

Jelínek J (2007) Family Monotomidae Laporte, 1840 [pp. 491–495]. In: Löbl I, Smetana A (Eds) Catalogue of Palaearctic Coleoptera. Volume 4. Elateroidea - Derodontoidea - Bostrichoidea - Lymexyloidea - Cleroidea - Cucujoidea. Apollo Books, Stenstrup, 935 pp.

Johnson C, Otero JC (2010) Description of a new *Monotoma* (*Monotomina*) species (Coleoptera: Monotomidae) from the Iberian Peninsula. Entomologica Fennica 21 (2): 104–106.

Klimaszewski J, Watt JC (1997) Coleoptera: family-group review and keys to identification. Fauna of New Zealand. Ko te Aitanga Pepeke o Aotearoa. Number 37. Manaaki Whenua Press, Lincoln, 197 pp.

Kuschel G (1979) The genera *Monotoma* Herbst (Rhizophagidae) and *Anommaus* Wesmael (Cerylidae) in New Zealand (Coleoptera). New Zealand Entomologist 7 (1): 44–48.

Majka CG, Bousquet Y (2010) Monotomidae (Coleoptera) of the Maritime Provinces of Canada. Journal of the Acadian Entomological Society 6: 1–8.

Milander G (1992) On the Monotomidae (Coleoptera) of Estonia. Izvestiya Akademii Nauk Estonskoi SSR Seriya Biologicheskaya 41: 66–71.

Nikitsky NB (1986) Zhestkokrylye podsemeystv Monotominae i Thioninae (Coleoptera, Rhizophagidae) dalnego Vostoka SSSR [Beetles of the subfamilies Monotominae and Thioninae (Coleoptera, Rhizophagidae) in the Soviet Far East]. Zoologicheskii Zhurnal 65 (11): 1622–1630 [in Russian].

Pal TK (2000). On five monotomid species deposited in the Museum of Natural History of the Humboldt-University in Berlin (Coleoptera, Cucujoidea). Mitteilungen aus dem Museum für Naturkunde in Berlin, Zoologische Reihe 76 (1): 113–119.

Peacock ER (1977) Vol. V, Part 5(a). Coleoptera Rhizophagidae. Handbooks for the identification of British insects. Royal Entomological Society of London, London, pp. 1–19.

Peck SB (2006) The beetles of the Galápagos Islands, Ecuador: evolution, ecology, and diversity (Insecta: Coleoptera). NRC Research Press, Ottawa, xiii + 313 pp.

Peck SB, Thomas MC (1998) A distributional checklist of the beetles (Coleoptera) of Florida. Arthropods of Florida and neighboring land areas. Volume 16, Florida Department of Agriculture and Consumer Services, Gainesville, viii + 180 pp.

Sen Gupta T (1977) A new genus and species of Rhizophagidae (Clavicornia: Coleoptera) from Sikkim. Oriental Insects 11 (4): 531–536.

Sen Gupta T (1988) Review of the genera of the family Rhizophagidae (Clavicornia: Coleoptera) of the world. Memoirs of the Zoological Survey of India 17: 1–58, pls. i-xxiv.

Silfverberg H (1992) Enumeratio Coleopterorum Fennoscandiae, Daniae et Baltiae. Helsingfors Entomologiska Bytesförening, Helsinki, v + 94 pp.

Ślipiński SA (1981) Klucze do oznaczania owadów Polski [Nr. 119]. Część XIX. Chrząszcze - Coleoptera. Zeszyt 63. Monotomidae. Polskie Towarzystwo Entomologiczne, Warszawa, 11 pp.

Vorst O (2010). Monotomidae [pp. 124–125]. In: Vorst O (Ed) Catalogus van de Nederlandse kevers. Nederlandse Entomologische Vereniging, Amsterdam, 317 pp.

### Oedemeridae

Supporting references for the conservation of Calopodinae A. Costa, 1852 over Sparedrinae Gistel, 1848 (Art. 23.9.2). The taxon name Sparedrinae Gistel, 1848 has not been used as valid after 1899 to our knowledge.

Arnett RH, Jr. (1962) Part V. Suborder Polyphaga (cont.). Series Cucujiformia (Cont.). Tenebrionoidea, Cucujoidea [pp. (2 unn. +) 645–850]. In: The beetles of the United States (a manual for identification) [original loose-leaf edition]. The Catholic University of America Press, Washington D.C., xii + 1112 pp. [+ unn. pp.].

Arnett RH, Jr. (1993) American insects. A handbook of the insects of America north of Mexico. The Sandhill Crane Press, Inc., Gainesville, xiii + 850 pp.

Arnett RH, Jr. (2000) American insects. A handbook of the insects of America north of Mexico. Second edition. CRC Press, Boca Raton, xvii + 1003 pp.

Bologna MA (2005) *Zonitis fernancastroi*, a new species for the Italian fauna, and additional records of Meloidae and Oedemeridae (Coleoptera). Bollettino della Società Entomologica Italiana 137 (2): 107–114.

Campbell JM (1991) Family Oedemeridae false blister beetles [p. 266]. In: Bousquet Y (Ed) Checklist of beetles of Canada and Alaska. Agriculture Canada Research Branch Publication 1861/E, vi + 430 pp.

Crowson RA (1967) The natural classification of the families of Coleoptera. E.W. Classey Ltd., Middlesex (England), [1] + 214 pp. [Note: this is a reprint].

Gómez de Dios MÁ, Verdugo Páez A (2008) Nuevo registro de *Sparedrus lencinae* Vázquez, 1988 (Oedemeridae: Calopodinae) de la Comunidad Autónoma de Andalucía (España). Boletín de la Sociedad Entomológica Aragonesa 42: 461–462.

Hatch MH (1965) The beetles of the Pacific Northwest. Part IV: Macrodactyles, Palpicornes, and Heteromera. University of Washington Press, Seattle, viii + 268 pp.

Hudson L (1975) A systematic revision of the New Zealand Oedemeridae (Coleoptera, Insecta). Journal of the Royal Society of New Zealand 5 (3): 227–274.

Hůrka K (2005) Brouci České a Slovenské republiky. Käfer der Tschechischen und Slowakischen Republik. Vit Kabourek, Zlín, 390 pp.

Kaszab Z (1969) 70. Familie: Oedemeridae [pp. 79–92]. In: Freude H, Harde KW, Lohse GA (Eds) Die Käfer mitteleuropas. Band 8. Teredilia, Heteromera, Lamellicornia. Goecke & Evers, Krefeld, 388 pp.

Klausnitzer B (1999) 112. Familie: Oedemeridae [pp. 310–324]. In: Klausnitzer B (Ed) Die Larven der Käfer mitteleuropas. 5 Band. Polyphaga Teil 4. Goecke & Evers, Krefeld, 336 pp.

Kriska NL (2002) 109. Oedemeridae Latreille 1810 [pp. 514–519]. In: Arnett RH, Jr., Thomas MC, Skelley PE, Frank JH (Eds) American beetles. Volume 2. Polyphaga: Scarabaeoidea through Curculionoidea. CRC Press, Boca Raton, xiv + 861 pp.

Kubisz D (2006) Oedemeridae and Scraptiidae of Poland (Coleoptera, Tenebrionoidea). Monografie Faunistyczne 24: 1–165.

Lawrence JL (2005) *Dasytomima*, a new genus of Australian Oedemeridae and its relationship to *Polypria* Chevrolat (Coleoptera: Tenebrionoidea). Annales Zoologici (Warszawa) 55 (4): 663–676.

Lundberg B (1995) Catalogus Coleopterorum Sueciae. Naturhistoriska Riksmuseet, Stockholm [unpaginated].

Švihla V (1986) Revision of the generic classification of the Old World Oedemeridae (Coleoptera). Sborník Národního Muzea v Praze (Řada B - Přírodní Vědy) 41 [1985] (3/4): 141–238.

Švihla V (1993) Oedemeridae [pp. 112–113]. In: Jelínek J (Ed) Check-list of Czechoslovak insects IV (Coleoptera). Seznam československých brouků. Folia Heyrovskyana Supplementum 1, 172 pp.

Švihla V (2008) Family Oedemeridae Latreille, 1810 [pp. 353–369]. In: Löbl I, Smetana A (Eds) Catalogue of Palaearctic Coleoptera. Volume 5. Tenebrionoidea. Apollo Books, Strenstrup, 670 pp.

Telnov D (2004) Check-list of Latvian beetles (Insecta: Coleoptera). Second edition. Compendium of Latvian Coleoptera 1. Entomological Society of Latvia, Rïga, 113 pp.

Telnov D, Barsevskis A, Savich F, Kovalevsky F, Berdnikov S, Doronin M, Cibulskis R, Ratniece D (1997) Check-list of Latvian beetles (Insecta: Coleoptera). Mitteilungen des Internationalen Entomologischen Vereins, Supplement 5: 1–141.

Vázquez XA (1988) A new species of Oedemeridae from the Iberian Peninsula (Coleoptera). Nouvelle Revue d’Entomologie (Nouvelle série) 5 (3): 259–261.

Vázquez XA (1993) Coleoptera, Oedemeridae, Pyrochroidae, Pythidae, Mycteridae. Fauna Iberica, Vol 5, Museo Nacional de Ciencias Naturales, Madrid, 181 pp.

Vázquez XA (2002) European Fauna of Oedemeridae. Argania editio S.C.P., Barcelona, 179 pp.

Zahradník J (1985) Käfer Mittel- und Nordwesteuropas. Ein Bestimmungsbuch für Biologen und Naturfreunde. Paul Parey, Hamburg, 498 pp.

### Scarabaeidae

Supporting references for the conservation of *Pachypus* Dejean, 1821 over *Pachypus* Billberg, 1820 (Art. 23.9.2). The genus name *Pachypus* Billberg, 1820 has not been used as valid after 1899 to our knowledge.

Ahrens D (2006) The phylogeny of Sericini and their position within the Scarabaeidae based on morphological characters (Coleoptera: Scarabaeidae). Systematic Entomology 31 (1): 113–144.

Arnone M, Sparacio I (1990) Il *Pachypus caesus* Erichson 1840: brevi note sulla biologia e la distribuzione in Sicilia (Coleoptera Scarabaeoidea). Naturalista Siciliano (4) 14 (1): 63–71.

Ballerio A, Tacchetti M (1990) *Pachypus candidae* (Petagna 1786) (Coleoptera Pachipodidae). Bollettino della Società Entomologica Italiana 121 [1989] (3): 234.

Baraud J (1977) Coléoptères Scarabaeoidea. Faune de l’Europe occidentale: Belgique, France, Grande-Bretagne, Italie, Péninsule Ibérique. Nouvelle Revue d’Entomologie Supplement 7 (1): 1–352.

Baraud J (1980) Nouveaux coléoptères Scarabaeoidea d’Afrique du nord. Nouvelle Revue d’Entomologie 10 (3): 279–284.

Baraud J (1992) Coléoptères Scarabaeoidea d’Europe. Faune de France 78. Société Linnéenne de Lyon, Lyon, ix + 856 pp. + 11 pls.

Bidault J (1999) Voyage entomologique en Haute Corse (Coleoptera, Carabidae, Cerambycidae, Scarabaeidae). Revue de l’Association Roussillonnaise d’Entomologie 8: 21–23.

Cantot P, Cantot C, Cantot S (1988) Sur deux espèces de coléoptères capturées en Corse (Scarabaeidae & Cerambycidae). L’ Entomologiste 44 (6): 308.

Crovetti A (1970) Contributi alla conoscenza dei Coleotteri Scarabeidi. 1. Il genere *Pachypus* Serville (Coleoptera, Scarabaeidae, Pachypodinae). Bollettino di Zoologia Agraria e Bachicoltura (Ser. 2) 9 [1968–69]: 134–188.

Crowson RA (1995) Some interesting evolutionary parallels in Coleoptera [pp. 63–85]. In: Pakaluk J, Ślipiński SA (Eds). Biology, phylogeny and classification of Coleoptera: Papers celebrating the 80th birthday of Roy A Crowson. Muzeum i Instytut Zoologii PAN, Warszawa, x + 1092 pp. in 2 vols.

Dupuis F (2005) L’abdomen et les genitalia des femelles de coléoptères Scarabaeoidea (Insecta, Coleoptera). Zoosystema 27 (4): 733–823.

Evans AV, Bellamy CL (1996) An inordinate foundness for beetles. Nevraumont Publishing Company, New York, 208 pp.

Evans G (1975) The life of beetles. George Allen & Unwin Ltd, London, 232 pp.

Král D, Löbl I (2006) Subfamily Pachypodinae Erichson, 1840 [p. 180]. In: Löbl I, Smetana A (Eds). Catalogue of Palaearctic Coleoptera. Volume 3. Scarabaeoidea - Scirtoidea - Dascilloidea - Buprestoidea - Byrrhoidea. Apollo Books, Stenstrup, 690 pp.

Kukalová-Peck J, Lawrence JF (2004) Relationships among coleopteran suborders and major endoneopteran lineages: evidence from hind wing characters. European Journal of Entomology 101 (1): 95–144.

Lapiana F, Sparacio I (2006) I Coleotteri Lamellicorni delle Madonie (Sicilia) (Insecta Coleoptera Lucanoidea et Scarabaeoidea). Naturalista Siciliano (4) 30 (2): 227–292.

Lawrence JF, Newton AF, Jr. (1995) Families and subfamilies of Coleoptera (with selected genera, notes, references and data on family-group names) [pp. 779–1006]. In: Pakaluk J, Ślipiński SA (Eds). Biology, phylogeny and classification of Coleoptera: Papers celebrating the 80th birthday of Roy A Crowson Vol II. Muzeum i Instytut Zoologii PAN, Warszawa, x + 1092 pp. in 2 vols.

Meinecke C-C (1975) Riechsensillen und Systematik der Lamellicornia (Insecta, Coleoptera). Zoomorphologie 82 (1): 1–42.

Ocampo FC, Ruiz-Manzanos E, Marvaldi AE (2010) Systematic revision, cladistics and biogeography of the genus *Neogutierrezia* Martínez (Coleoptera: Scarabaeidae) and its phylogenetic placement in Rutelinae based on structural alignment of 28S rDNA sequences. Invertebrate Systematics 24 (1): 81–111.

Paulian R (1988) Biologie des Coléoptères. Lechevalier, Paris, xi + 719 pp.

Pesarini C (2004) Insetti della fauna Italiana Coleotteri Lamellicorni. Natura (Milan) 93 (2): 1–130.

Sabella G, Sparacio I (2004) Il ruolo dei Parchi Siciliani nella conservazione di taxa di insetti di particolare interesse naturalistico (Insecta Coleoptera et Lepidoptera Rhopalocera). Naturalista Siciliano (4) 28 (1): 477–508.

Scholtz CH, Grebennokov V (2005) 13. Scarabaeoidea Latreille, 1802 [pp. 367–425]. In: Beutel RG, Leschen RAB (Eds). Handbook of zoology. A natural history of the phyla of the animal kingdom. Volume IV - Arthropoda: Insecta. Walter De Gruyter, Berlin, xi + 567 pp.

Smith ABT (2006) A review of the family-group names for the superfamily Scarabaeoidea (Coleoptera) with corrections to nomenclature and a current classification. Coleopterists Society Monograph 5: 144–204.

Sparacio I (2008) Una nuova specie di *Pachypus* Dejean di Sardegna (Coleoptera, Scarabaeoidea, Pachypodidae). Doriana 8 (360): 1–13.

### Scarabaeidae

Supporting references for the conservation of *Sparrmannia* Laporte, 1840 over *Leocaeta* Dejean, 1833 and *Cephalotrichia* Hope, 1837 (Art. 23.9.2). The generic names *Leocaeta* Dejean, 1833 and *Cephalotrichia* Hope, 1837 have not been used as valid after 1899 to our knowledge.

Ahrens D (2006) The phylogeny of Sericini and their position within the Scarabaeidae based on morphological characters (Coleoptera: Scarabaeidae). Systematic Entomology 31 (1): 113–144.

Browne DJ, Scholtz CH (1995) Phylogeny of the families of Scarabaeoidea (Coleoptera) based on characters of the hindwing articulation, hindwing base and wing venation. Systematic Entomology 20 (3): 145–173.

Browne DJ, Scholtz CH (1996) The morphology of the hind wing articulation and wing base of the Scarabaeoidea (Coleoptera) with some phylogenetic implications. Bonner Zoologische Monographien 40: 1–200.

Browne DJ, Scholtz CH (1998) Evolution of the scarab hindwing articulation and wing base: a contribution toward the phylogeny of the Scarabaeidae (Scarabaeoidea: Coleoptera). Systematic Entomology 23 (4): 307–326.

Chown SL, Nicolson SW (2004) Insect physiological ecology: mechanisms and patterns. Oxford University Press, Oxford, ix + 243 pp.

Chown SL, Scholtz CH (1993) Temperature regulation in the nocturnal melolonthine *Sparrmannia flava*. Journal of Thermal Biology 18 (1): 25–33.

Coca-Abia M (2007) Phylogenetic relationships of the subfamily Melolonthinae (Coleoptera, Scarabaeidae). Insect Systematics & Evolution 38 (4): 447–472.

d’Hotman D, Scholtz CH (1991) Comparative morphology of the male genitalia of derived groups of Scarabaeoidea (Coleoptera). Elytron 4 [1990]: 3–39.

Evans AV (1988) Three new species of *Glyptoglossa* Brenske (Coleoptera: Scarabaeidae: Melolonthinae). Journal of the Entomological Society of Southern Africa 51 (1): 87–96.

Evans AV (1989) Revision of the genus *Sparrmannia* Laporte (Coleoptera: Melolonthidae: Melolonthinae). Journal of the Entomological Society of Southern Africa 52 (1): 11–44.

Ferreira MC (1966) Catálogo dos Coleópteros de Moçambique (Conclusão). Revista de Entomologia de Moçambique 6 [1963] (2): [1] + 533–1008 + 1 map.

Hardy, A.R. (1978) Placement of the genus *Benedictia* Sanderson (Coleoptera: Scarabaeidae). The Coleopterists Bulletin 32 (1): 67–70.

Katovich K (2008) A generic-level phylogenetic review of the Macrodactylini (Coleoptera: Scarabaeidae: Melolonthinae). Insecta Mundi 23: 1–78.

Lacroix M (2004) Contribution à la connaissance des Pachydeminae africains, IV. Nouvelles espèces (Coleoptera, Melolonthidae). Coléoptères 10 (9): 107–118.

Lacroix M (2007) Pachydeminae du monde: genera et catalogue (Coleoptera, Melolonthidae). Collection Hannetons, Paris, 450 pp.

Nel A, Scholtz CH (1990) Comparative morphology of the mouthparts of adult Scarabaeoidea. Entomology Memoir, Department of Agricultural Development, Republic of South Africa 80: 1–84.

Paulian R (1993) Les Coléoptères: à la conquête de la terre. Sociéte Nouvelle des Éditions Boubée, Paris, 241 + [4] pp.

Picker M, Griffiths C, Weaving A (2002) Field guide to insects of South Africa. Struik Publishers, Cape Town, 440 pp.

Sanmartín I (2003) Dispersal vs. vicariance in the Mediterranean: historical biogeography of the Palearctic Pachydeminae (Coleoptera, Scarabaeoidea). Journal of Biogeography 30 (12): 1883–1897.

Sanmartín I, Martín-Piera F (2003) First phylogenetic analysis of the subfamily Pachydeminae (Coleoptera, Scarabaeoidea, Melolonthidae): the Palearctic Pachydeminae. Journal of Zoological Systematics and Evolutionary Research 41 (1): 2–46.

Scholtz CH (1988) Biology of *Sparrmannia flava* Arrow (Coleoptera: Scarabaeidae: Melolonthinae). The Coleopterists Bulletin 42 (1): 57–62.

Scholtz CH, Chown SL (1995) The evolution of habitat use and diet in the Scarabaeoidea: a phylogenetic approach [pp. 355–374]. In: Pakaluk J, Ślipiński SA (Eds) Biology, phylogeny and classification of Coleoptera: Papers celebrating the 80th birthday of Roy A Crowson. Volume one. Muzeum i Instytut Zoologii PAN, Warszawa, x + 1092 pp. in 2 vols.

Scholtz CH, Grebennikov VV (2005) Scarabaeiformia Crowson, 1960. Chapter 12 [pp. 345–366]. In: Beutel RG, Leschen RAB (Eds). Handbook of zoology. A natural history of the phyla of the animal kingdom. Volume IV - Arthropoda: Insecta. Walter De Gruyter, Berlin, xi + 567 pp.

Scholtz CH, Holm E (1985) Insects of southern Africa. Butterworths, Durban, 502 p. + xii plates.

Smith ABT (2006) A review of the family-group names for the superfamily Scarabaeoidea (Coleoptera) with corrections to nomenclature and a current classification. Coleopterists Society Monograph 5: 144–204.

### Tenebrionidae

Supporting references for the conservation of Adesmiini Lacordaire, 1859 over Macropodini Agassiz, 1846 (Art. 23.9.2). The taxon name Macropodini Agassiz, 1846 has not been used as valid after 1899 to our knowledge and is a junior homonym of the well-established name Macropodidae Gray, 1821 [Mammalia].

Alfieri A (1976) The Coleoptera of Egypt. Mémoires de la Société Entomologique d’Égypte 5: 1–361.

Ardoin P (1972) Liste des espèces de Tenebrionidae (Coleoptera) récoltées au Sudan par les expéditions finlandaises (1962–1964) (Zoological contribution from the Finnish expeditions to the Sudan no. 27). Commentationes Biologicae 49: 1–20.

Bouchard P, Lawrence JF, Davies AE, Newton AF (2005) Synoptic classification of the world Tenebrionidae (Insecta: Coleoptera) with a review of family-group names. Annales Zoologici (Warszawa) 55 (4): 499–530.

Cloudsley Thompson JL (2001) Thermal and water relations of desert beetles. Naturwissenschaften 88: 447–460.

Doyen JT (1994) Cladistic relationships among Pimeliine Tenebrionidae (Coleoptera). Journal of the New York Entomological Society 101 [1993] (4): 443–514.

Duncan FD (2003) The role of the subelytral cavity in respiration in a tenebrionid beetle, *Onymacris multistriata* (Tenebrionidae: Adesmiini). Journal of Insect Physiology 49: 339–346.

Ferreira MC (1965) Catálogo dos Coleópteros de Moçambique. Revista de Entomologia de Moçambique 6 [1963] (1): 1–531 + [1, corrigenda].

Ferreira MC (1967) Catálogo dos Coleópteros de Angola. Revista de Entomologia de Moçambique 8 [1965] (2): 415–1317.

Girard C (1967) Coléoptères ténébrionidés Erodiini, Tentyriini et Adesmiini récoltés par J. Mateu dans le massif de l’Ennedi. Bulletin de l’Institut Français de l’Afrique Noire (Série A) 29 (3): 945–953.

Hanrahan SA, Nicolson SW (1984) The effect of dehydration on the Malpighian tubules of *Onymacris plana* (Coleoptera: Adesmiini). Elektronmikroskopievereniging van Suidelike Afrika Verrigtings 14: 107–108.

Hauffe HC, Pietruszka RD, Seely MK (1988) Observations on the behaviour of *Onymacris laeviceps* Gebien (Coleoptera: Tenebrionidae: Adesmiini) in the central Namib Desert dunes. Journal of the Entomological Society of Southern Africa 51: 183–192.

Kaszab Z (1981) Insects of Saudi Arabia. Coleoptera: fam. Tenebrionidae (part 2). Fauna of Saudi Arabia 3: 276–401.

Koch C (1962) The Tenebrionidae of Southern Africa. XXXI. Comprehensive notes on the tenebrionid fauna of the Namib desert. Annals of the Transvaal Museum 24 (2/3): 61–106.

Kwieton E (1981) Esquisse entomogéographique de l’Algérie et de l’histoire du désert Saharien, à la base des coléoptères Tenebrionidae. Anais da Faculdade de Ciências Universidade do Porto 62: 189–237.

Kwieton E (1982) Revue critique des systèmes récents de la famille des Tenebrionidae (Col.). Sborník Národního Muzea v Praze (Řada B - Přírodní Vědy) 38 (1/2): 79–100.

McClain E, Praetorius RL, Hanrahan SA, Seely MK (1984) Dynamics of the wax bloom of a seasonal Namib Desert tenebrionid, *Cauricara phalangium* (Coleoptera: Adesmiini). Oecologia 63 (3): 314–319.

Penrith M-L (1979) Revision of the western southern African Adesmiini (Coleoptera: Tenebrionidae). Cimbebasia (Series A) 5: 1–94.

Penrith M-L (1986) Relationships in the tribe Adesmiini (Coleoptera: Tenebrionidae) and a revision of the genus *Stenodesia* Reitter. Annals of the Transvaal Museum 34 (13): 275–302.

Roer H (1981) Weitere Untersuchungen zur Anpassung des Namibwüstenkäfers *Onymacris r. rugatipennis* (Haag 1875, Col.: Tenebrionidae, Adesmiini) am das Trockenflussbett des Kuiseb in Südwestafrika. Mitteilungen der Deutschen Gesellschaft für Allgemeine und Angewandte Entomologie 3 (1/3): 218–222.

Roer H (1983) Aktionsraum und Anpassungsphanomene des Dunenkafers *Onymacris laeviceps* Gebien (Col.: Tenebrionidae, Adesmiini) in der Namibwüste. Bonner Zoologische Beiträge 34 (1/3): 357–369.

Roer H (1984) Dispersion dynamics of desert beetles of the genus *Onymacris* (Col., Tenebrionidae, Adesmiini) in the Namib [p. 302]. XVII International Congress of Entomology. Abstract Volume, 954 pp.

Roer H (1985) Dispersion dynamics of tenebrionids of the genus *Onymacris* (Col.: Tenebrionidae, Adesmiini) in the Namib Desert. Journal SWA Scientific Society 39: 65–69.

Roer H (1986) Zur Anpassung des Schwarzkäfers *Onymacris unguicularis* (Haag) (Col.: Tenebrionidae, Adesmiini) an die Nebelzone der Namibwüste. Bonner Zoologische Beiträge 37 (2): 143–154.

Skopin NG (1978) Tenebrionidae [pp. 223–266]. In: Klausnitzer B (Ed) Ordnung Coleoptera (Larven). W. Junk, The Hague, vi + 378 pp.

Veiga Ferreira G, da (1966) Catálogo dos tipos de insectos existentes no Museu Dr. Álvaro de Castro. Revista de Entomologia de Moçambique 7 [1964] (1): 197–216.

### Tenebrionidae

Supporting references for the conservation of Bolitophagini Kirby, 1837 over Eledonini Billberg, 1820 (Art. 23.9.2). The taxon name Eledonini Billberg, 1820 has not been used as valid in Tenebrionidae after 1899 to our knowledge. Furthermore, conservation of Bolitophagini avoids homonymy with Eledoninae used as valid in cephalopods.

Aalbu RL (2006) 2006, where are we at: assessing the currents state of Tenebrionidae systematics on a global scale (Coleoptera: Tenebrionidae). Cahiers Scientifiques. Centre de Conservation et d’Étude des Collections (Lyon) 10: 55–70.

Aalbu RL, Flores GE, Triplehorn CA (2002) Tenebrionidae [pp. 499–512]. In: Bousquets JL, Morrone JJ (Eds) Biodiversidad, taxonomía y biogeografía de arthropódos de México: Hacia una síntesis de su conocimiento Volumen III. Universidad Nacional Autonóma de México, México, x + 690 pp.

Aalbu RL, Triplehorn CA, Campbell JM, Brown KW, Somerby RE, Thomas DB (2002) 106. Tenebrionidae Latreille 1802 [pp. 463–509]. In: Arnett RHJ, Thomas MC, Skelley PE, Frank JH (Eds) American beetles. Volume 2. Polyphaga: Scarabaeoidea through Curculionoidea. CRC Press, Boca Raton, xiv + 861 pp.

Bouchard P, Lawrence JF, Davies AE, Newton AF (2005) Synoptic classification of the world Tenebrionidae (Insecta: Coleoptera) with a review of family-group names. Annales Zoologici (Warsaw) 55 (4): 499–530.

Downie NM, Arnett RH, Jr. (1996) The beetles of northeastern North America. Volume 2: Polyphaga: Series Bostrichiformia through Curculionoidea. Sandhill Crane Press, Gainesville, pp. i-x, 891–1721.

Doyen JT, Matthews EG, Lawrence JF (1990) Classification and annotated checklist of the Australian genera of Tenebrionidae (Coleoptera). Invertebrate Taxonomy 3 [1989] (3): 229–260.

Doyen JT, Poinar GO, Jr (1994) Tenebrionidae from Dominican amber (Coleoptera). Entomologica Scandinavica 25 (1): 27–51.

Endrödy-Younga S (1998) *Afrobyrsax capensis* spec. nov., with a tropical West African relationship (Coleoptera: Tenebrionidae: Bolitophagini). Annals of the Transvaal Museum 36 (30): 421–423.

Español F (1985) Los Bolitophaginae de la fauna española (Col., Tenebrionidae). Publicaciones del Departamento de Zoología, Universidad de Barcelona 11: 61–64.

Hornig U (1993) Das Typenmaterial der Tenebrionidae im Staatlichen Museum für Tierkunde Dresden. Teil 1: Tribus Platyscelini, Praocini, Pedinini, Opatrini, Phaleriini, Crypticini, Bolitophagini, Rhipidandrini, Diaperini, Gnathidiini, Leiochrini et Phrenapatini (Insecta, Coleoptera). Entomologische Abhandlungen (Dresden) 55 (2): 153–161.

Jung BH, Kim SY, Kim JI (2007) Taxonomic review of the tribe Bolitophagini in Korea (Coleoptera: Tenebrionidae: Tenebrioninae). Entomological Research 37 (3): 190–196.

Kompantseva TV (1994) Mycetophilous tenebrionid fauna of Bolitophagini and Diaperini (Coleoptera, Tenebrionidae) from Middle Asia. Byulleten’ Moskovskogo Obshchestva Ispytatelei Prirody Otdel Biologicheskii 99: 44–47.

Kompantseva TV (1996) Larva of *Rhipidandrus crenipennis* (Motschulsky, 1858), and the position of the genus in Bolitophagini (Coleoptera Tenebrionidae). Lichinka *Rhipidandrus crenipennis* (Motschulsky, 1858), I polozhenie roda v tribe Bolitophagini (Coleoptera Tenebrionidae). Russian Entomological Journal 4 (1/4) [1995]: 55–59.

Löbl I, Ando K, Bouchard P, Iwan D, Lillig M, Masumoto K, Merkl O, Nabozhenko M, Novák V, Petterson R, Schawaller W, Soldati F (2008) Family Tenebrionidae Latreille, 1802 [pp. 105–352]. In: Löbl I, Smetana A (Eds) Catalogue of Palaearctic Coleoptera. Volume 5. Tenebrionoidea. Apollo Books, Stenstrup, 670 pp.

Masumoto K (2006) Tenebrionid beetles (Coleoptera) from the Palau Islands collected by Keiichi Takahashi. Entomological Review of Japan 61 (2): 143–161.

Masumoto K, Akita K (2001) Two new tenebrionid species from Japan. Special Publication of the Japanese Coleopterist Society 1: 247–250.

Matthews EG, Bouchard P (2008) Tenebrionid beetles of Australia: descriptions of tribes, keys to genera, catalogue of species. Australian Biological Resources Study, Canberra, viii + 398 pp.

Miyatake M (1964) Notes on the tribe Bolitophagini of Japan, with the descriptions of four new genera and two new species (Coleoptera: Tenebrionidae). Transactions of the Shikoku Entomological Society 8 (2): 59–84.

Miyatake M (1970) A revision of the genus *Byrsax* Pascoe of Japan, with some notes on the Japanese Bolitophagini (Coleoptera: Tenebrionidae). Transactions of the Shikoku Entomological Society 10 (3/4): 116–126.

Schawaller W (2006) A new species of *Cryphaeus* and new records of other fungus-adapted tenebrionids from South Africa (Coleoptera: Tenebrionidae). Annals of the Transvaal Museum 43: 69–74.

Soldati F, Soldati L, Thieren Y (2009) Discovery of *Eledonoprius serrifrons* (Reitter, 1890) in Corsica, a new species for the fauna of France (Coleoptera, Tenebrionidae, Bolitophagini). Rutilans 12: 33–36.

Steiner WE, Jr. (2004) New distribution records for *Eleates depressus* (Randall) in the south-eastern United States (Coleoptera: Tenebrionidae; Bolitophaginae) and notes on these occurrences. Banisteria 24: 52–53.

Tezcan S, Karsavuran Y, Pehlivan E, Keskin B, Ferrer J (2004) Contributions to the knowledge of the Tenebrionidae (Coleoptera) from Turkey Part I. Lagriinae, Pimeliinae, Bolitophaginae, Diaperinae. Turkiye Entomoloji Dergisi 28: 99–114.

Tovar AC, Sáez Bolaño J, Baena M (2008) Nuevas citas de Bolitophagini Kirby, 1837 (Coleoptera, Tenebrionidae) de España. Boletín de la Sociedad Entomológica Aragonesa 42: 361–365.

### Throscidae

Supporting references for the conservation of Throscidae Laporte, 1840 over Stereolidae Rafinesque, 1815 (Art. 23.9.2). The taxon name Stereolidae Rafinesque, 1815 has not been used as valid after 1899 to our knowledge.

Allen AA (2001) The identity of *Trixagus elateroides* (Throscidae) of British authors. Coleopterist 10: 37.

Burakowski B (2000) Redescription of *Aulonothroscus laticollis* (Rybinski, 1897) (Coleoptera: Throscidae). Annales Zoologici (Warszawa) 50: 27–34.

Franz H, von (1982) Revision der *Throscus*-Arten (Coleoptera) der Makaronesischen Inseln. Zeitschrift der Arbeitsgemeinschaft Österreichischer Entomologen 34 (1/2): 49–58.

Gruszka A, Tarnawszki D (1994) *Trixagus elateroides* (Heer) (Coleoptera, Throscidae), *Isorhipis melasoides* (Cast.) and *Dirhagus pygmaeus* (F.) (Coleoptera, Eucnemidae) - new records from Poland. Wiadomosci Entomologiczne 13: 256.

Hornig U (2003) Faunistic records from the Czech Republic - 164. Coleoptera: Throscidae. Klapalekiana 39 (1/3): 129.

Hornig U (2005) Fauna der Throscidae der Oberlaustiz (Col.). Entomologische Nachrichten und Berichte 49 (2): 123–126.

Johnson PJ (1997) New distribution records for three Throscidae (Coleoptera) in South Dakota. Prairie Naturalist 29: 51–52.

Johnson PJ (2002) 57. Throscidae Laporte 1840 [pp. 158–159]. In: Arnett RH, Jr., Thomas MC, Skelley PE, Frank JH (Eds) American beetles. Volume 2. Polyphaga: Scarabaeoidea through Curculionoidea. CRC Press, Boca Raton, xiv + 861 pp.

Kistner DH, Abdel-Galil FA (1986) A new genus and species of termitophilous Throscidae from South Africa (Coleoptera). Sociobiology 12 (2): 305–313.

Leseigneur L (1995) Statut actuel des genres *Trixagus* Kugelann, 1794, et *Throscus* Latreille, 1796. Désignation des lectotypes des espèces paléarctiques de H. de Bonvouloir (Coleoptera, Throscidae). Bulletin de la Société Entomologique de France 100 (4): 347–359.

Leseigneur L (1996) *Trixagus atticus* Reitter et *T. minutus* Rey, deux espèces méconnues d’Europe occidentale présentes en France (Coleoptera Throscidae). Bulletin Mensuel de la Société Linnéenne de Lyon 65: 181–192.

Leseigneur L (1997) Réhabilitation de *Trixagus gracilis* Wollaston (Coleoptera, Throscidae). Bulletin de la Société Entomologique de France 102 (2): 137–142.

Leseigneur L (2005a) Description de *Trixagus meybohmi* n. sp. et note sur la morphologie des *Trixagus* du groupe *carinifrons* (Coleoptera, Throscidae). Bulletin de la Société Entomologique de France 110 (1): 89–96.

Leseigneur L (2005b) *Trixagus angelinii* n. sp. (Coleoptera, Throscidae). Bulletin de la Société Entomologique de France 110 (2): 181–184.

Leseigneur L, Gueorguiev B (2006) A contribution to the study of Bulgarian Throscidae (Coleoptera: Elateriformia). Historia Naturalis Bulgarica 17: 43–46.

Muona J (1993) Eucnemidae and Throscidae from Baltic amber (Coleoptera). Entomologische Blätter für Biologie und Systematik der Käfer 89: 15–45.

Muona J (1995) The phylogeny of Elateroidea (Coleoptera), or which tree is best today? Cladistics 11 (4): 317–341.

Muona J (2002) *Trixagus leseigneuri* n. sp. (Coleoptera, Throscidae). Bulletin de la Société Entomologique de France 107 (2): 187–190.

Nikitsky NB, Semenov VB (2001) To the knowledge of the beetles (Coleoptera) of the Moscow region. Byulleten’ Moskovskogo Obshchestva Ispytatelei Prirody Otdel Biologicheskii 106: 38–49 [in Russian].

Van-Meer C (1998) *Aulonothroscus laticollis* (Rybinski) (Coleoptera, Throscidae), une espèce nouvelle pour la faune d’Europe occidentale. Bulletin de la Société Linnéenne de Bordeaux 26: 181–183.

Wedmann S (1994) Fossile Vertreter der Eucnemidae und Throscidae (Insecta: Coleoptera) aus der mitteleozanen Messel-Formation. Courier Forschungsinstitut Senckenberg 170: 65–73.

Yensen E (1975) A review of the genus *Pactopus* Leconte (Coleoptera: Throscidae). The Coleopterists Bulletin 29 (2): 87–91.

Yensen E (1975) A revision of the North American species of *Trixagus* Kugelann (Coleoptera: Throscidae). Transactions of the American Entomological Society 101 (1): 125–166.

Yensen E (1981) A new species of *Trixagus* from Panama and a key to New World *Trixagus* (Coleoptera: Throscidae). The Coleopterists Bulletin 34 [1980] (3): 257–261.

Yoshizawa N (2003) Notes on the *Drapetes torigaii* Nakane (Coleoptera, Throscidae) in Nagano, Japan. Gekkan-Mushi 391: 14–15.

### Trogossitidae

Supporting references for the conservation of Lophocaterini Crowson, 1964 over Lycoptini Casey, 1890 (Art. 23.9.2). The taxon name Lycoptini Casey, 1890 has not been used as valid after 1899 to our knowledge.

Beutel RG, Leschen RAB (2005) Classification [pp. 11–16]. In: Beutel RG, Leschen RAB (Eds). Handbook of zoology. A natural history of the phyla of the animal kingdom. Volume IV - Arthropoda: Insecta. Walter De Gruyter, Berlin, xi + 567 pp.

Crowson RA (1964) A review of the classification of Cleroidea (Coleoptera), with descriptions of two new genera of Peltidae and of several new larval types. Transactions of the Royal Entomological Society of London 116 (12): 275–327.

Crowson RA (1970) Further observations on Cleroidea (Coleoptera). Proceedings of the Royal Entomological Society of London (Series B) 39 (1/2): 1–20.

Klausnitzer B (1996) Die Larven der Käfer Mitteleuropas. 3. Band. Polyphaga. Teil 2. Goecke & Evers, Krefeld, 335 pp.

Klimaszewski J, Watt JC (1997) Coleoptera: family-group review and keys to identification. Fauna of New Zealand. Ko te Aitanga Pepeke o Aotearoa. Number 37, Manaaki Whenua Press, Lincoln, 199 pp.

Kolibáč J (2006) A review of the Trogossitidae. Part 2: larval morphology, phylogeny and taxonomy (Coleoptera, Cleroidea). Entomologica Basiliensia et Collectionis Frey 28: 105–153.

Kolibáč J (2007) Family Trogossitidae Latreille, 1802 [pp. 364–366]. In: Löbl I, Smetana A (Eds) Catalogue of Palaearctic Coleoptera. Volume 4. Elateroidea - Derodontoidea - Bostrichoidea - Lymexyloidea - Cleroidea - Cucujoidea. Apollo Books, Stenstrup, 935 pp.

Lafer GS (1992) 55. Sem. Peltidae (Lophocateridae) [pp. 82–86]. In: Ler PA (Ed) Opredelitel’ nasekomykh Dal’nego Vostoka SSSR Tom 3 - Zheskokrylye, ili zhuki Chast 2 [Keys to the identification of insects of the Soviet Far East 3 - Coleoptera, or beetles Part 2]. Nauka, Saint-Petersburg, 704 pp. [in Russian].

Lawrence JF, Britton EB (1991) Coleoptera (beetles) [pp. 543–683]. In: The insects of Australia: a textbook for student and research workers. Second edition. Volume II. Melbourne University Press, Carlton, pp. i-vi + 543–1137.

Lawrence JF, Newton AFJ (1995) Families and subfamilies of Coleoptera (with selected genera, notes, references and data on family-group names) [pp. 779–1006]. In: Pakaluk J, Ślipiński SA (Eds) Biology, phylogeny, and classification of Coleoptera: papers celebrating the 80th birthday of Roy A Crowson. Museum i Instytut Zoologii PAN, Warszawa, x + 1092 pp. in 2 vols.

Leschen RAB (2000) Beetles feeding on bugs (Coleoptera, Hemiptera): repeated shifts from mycophagous ancestors. Invertebrate Taxonomy 14 (6): 917–929.

Leschen RAB (2002) 72. Trogossitidae Latreille, 1802 [pp. 263–266]. In: Arnett RH, Jr., Thomas MC, Skelley PE, Frank JH (Eds) American beetles. Volume 2. Polyphaga: Scarabaeoidea through Curculionoidea. CRC Press, Boca Raton, xiv + 861 pp.

Leschen RAB, Lawrence JF, Kuschel G, Thorpe S, Wang Q (2003) Coleoptera genera of New Zealand. New Zealand Entomologist 26: 15–28.

Leschen RAB, Lawrence JF, Ślipiński SA (2005) Classification of basal Cucujoidea (Coleoptera: Polyphaga): cladistic analysis, keys and review of new families. Invertebrate Systematics 19 (1): 17–73.

Lucht W (1998) 32.c Familie: Lophocateridae [pp. 207–208]. In: Lucht W, Klausnitzer B (Eds) Die Käfer Mitteleuropas. 4. Supplementband. Goecke & Evers, Krefeld, 398 pp.

Lundberg B (1995) Catalogus Coleopterorum Sueciae. Naturhistoriska Riksmuseet, Stockholm [unpaginated].

Machado A, Oromí P (2000) Elenco de los Coleópteros de las Islas Canarias. Instituto de Estudios Canarios, La Laguna, 306 + [2] pp.

McKenna DD, Farrell BD (2009) Beetles (Coleoptera) [pp. 278–289]. In: Hedges SB, Kumar S (Eds) The timetree of life. Oxford University Press Inc., New York.

Peck SB (2005) A checklist of the beetles of Cuba with data on distributions and bionomics (Insecta: Coleoptera). Arthropods of Florida and neighboring land areas. Volume 18, Florida Department of Agriculture and Consumer Services, Gainesville, vi + 241 pp.

Peck SB, Thomas MC (1998) A distributional checklist of the beetles (Coleoptera) of Florida. Arthropods of Florida and neighboring land areas. Volume 16, Florida Department of Agriculture and Consumer Services, Gainesville, viii + 180 pp.

Ribera I (1999) Evolución, filogenia y clasificación de los Coleoptera (Arthropoda: Hexapoda). Boletín de la Sociedad Entomológica Aragonesa 26: 435–458.

Silfverberg H (1992) Enumeratio Coleopterorum Fennoscandiae, Daniae et Baltiae. Helsingfors Entomologiska Bytesförening, Helsinki, v + 94 pp.

Ślipiński SA (1992) Larinotinae - a new subfamily of Trogossitidae (Coleoptera), with notes on the constitution of Trogossitidae and related families of Cleroidea. Revue Suisse de Zoologie 99 (2): 439–463.

Tait SM, Dahlsten DL, Gill RJ, Doyen JT (1990) Life history of the incense cedar scale, *Xylococculus macrocarpae* (Homoptera: Margarodidae), on incense cedar in California with a description of the larvae of one of its common predators, *Eronyxa expansus* van Dyke (Coleoptera: Trogositidae). Hilgardia 58 (2): 1-19.

Telnov D (2004) Check-list of Latvian beetles (Insecta: Coleoptera). Second edition. Compendum of Latvian Coleoptera 1. Entomological Society of Latvia, Rïga, 113 pp.

Telnov D, Barsevskis A, Savich F, Kovalevsky F, Berdnikov S, Doronin M, Cibulskis R, Ratniece D (1997) Check-list of Latvian beetles (Insecta: Coleoptera). Mitteilungen des Internationalen Entomologischen Vereins, Supplement 5: 1-141.

## Appendix 2

Coleoptera family-group name changes required based on the Principle of Priority. The action that has been taken to fix the problem, or recommendation for future work, is mentioned for each case. Cases listed in alphabetical order by family.

**Table:**

Family	Change from:	Change to:	Action	Reference
Aderidae	Aderidae/-ini Csiki, 1909	Euglenesidae/-ini Seidlitz, 1875	Younger name given precedence over older name and placed on the Official List of Family-Group Names in Zoology	ICZN (1989e)
Anthribidae	Anthribidae Billberg, 1820	Choragidae Kirby, 1819	Younger name given precedence over older name and placed on the Official List of Family-Group Names in Zoology	ICZN (1994b)
Brentidae	Acratina Alonso-Zarazaga et al., 1999	Nemocephalina Lacordaire, 1865	An application to the Commission is needed to suppress the older name because it is based on a misidentified type genus (Art. 65.2.1)	
Brentidae	Pseudoceocephalina Kleine, 1922	Uropterina Jakobson, 1911	An application to the Commission is needed to conserve usage of the well-established name Pseudoceocephalina Kleine, 1922	
Brentidae	Pseudoceocephalina Kleine, 1922	Ceocephalina Lacordaire, 1865	An application to the Commission is needed to suppress the older name because it is based on a misidentified type genus (Art. 65.2.1)	
Buprestidae	Chalcophorina Lacordaire, 1857	Anaglyptina Gistel, 1848	Use of younger name conserved (Art. 40.2)	this paper
Callirhipidae	Callirhipidae Emden, 1924	Zenoidae LeConte, 1866	An application to the Commission is needed to conserve usage of the well-established name Callirhipidae Emden, 1924	
Cantharidae	Cantharidae/-inae/-ini Imhoff, 1856	Telephoridae/-nae/-ini Leach, 1815	Use of younger name conserved (Art. 40.2)	Lawrence and Newton (1995)
Carabidae	Anisodactylina Lacordaire, 1854	Eurytrichina LeConte, 1847	Use of younger name conserved (Art. 23.9.2)	this paper
Carabidae	Harpalinae Bonelli, 1810	Graphipterinae Latreille, 1802	Use of younger name conserved (Art. 35.5)	this paper
Carabidae	Hexagoniini Horn, 1881	Trigonodactylini Brullé, 1834	Use of younger name conserved (Art. 40.2)	this paper
Carabidae	Iresiina Rivalier, 1971	Euprosopina Horn, 1893	An application to the Commission is needed to conserve usage of the well-established name Iresiina Rivalier, 1971	
Carabidae	Iresiina Rivalier, 1971	Eucalliina Horn, 1893	An application to the Commission is needed to conserve usage of the well-established name Iresiina Rivalier, 1971	
Carabidae	Iresiina Rivalier, 1971	Distipsiderina Horn, 1893	An application to the Commission is needed to conserve usage of the well-established name Iresiina Rivalier, 1971	
Carabidae	Perigonini Horn, 1881	Trechicini Bates, 1873	Use of younger name conserved (Art. 23.9.2)	this paper
Carabidae	Salcediini/-ina Alluaud, 1930	Zelmini/-ina Andrewes, 1929	Use of younger name conserved (Art. 40.2)	this paper
Cerambycidae	Eurypodini Gahan, 1906	Zaracini Lacordaire, 1868	Use of younger name conserved (Art. 40.2)	this paper
Cerambycidae	Hemilophini Thomson, 1868	Amphionychini Thomson, 1860	Older name treated as a nomen oblitum	Bousquet et al. (2009)
Cerambycidae	Macronini Lacordaire, 1868	Enchopterini Thomson, 1861	An application to the Commission is needed to conserve usage of the well-established name Macronini Lacordaire, 1868	
Cerambycidae	Pseudocephalini Aurivillius, 1912	Ametrocephalini Thomson, 1861	Use of younger name conserved (Art. 40.2)	Bousquet et al. (2009)
Cerambycidae	Stenoderini Pascoe, 1867	Syllitini Thomson, 1864	An application to the Commission is needed to conserve usage of the well-established name Stenoderini Pascoe, 1867	
Chrysomelidae	Amblycerina Bridwell, 1932	Spermophagina Crotch, 1873	An application to the Commission is needed to suppress the older name because it is based on a misidentified type genus (Art. 65.2.1)	
Chrysomelidae	Basiprionotini Gressitt, 1952	Priopterini Spaeth, 1914	Use of younger name conserved (Art. 40.2)	this paper
Chrysomelidae	Bromiini Baly, 1865	Adoxiini Baly, 1863	Use of younger name conserved (Art. 40.2)	this paper
Chrysomelidae	Bromiini Baly, 1865	Heteraspini Baly, 1863	An application to the Commission is needed to conserve usage of the well-established name Bromiini Baly, 1865	
Chrysomelidae	Dorynotini Monrós and Viana, 1949	Batonotini Spaeth, 1923	Use of younger name conserved (Art. 40.2)	this paper
Chrysomelidae	Hemisphaerotini Monrós and Viana, 1951	Porphyraspidini Spaeth, 1929	Use of younger name conserved (Art. 40.2)	this paper
Chrysomelidae	Nothosacanthini Gressitt, 1952	Hoplonotini Spaeth, 1929	Use of younger name conserved (Art. 40.2)	this paper
Chrysomelidae	Oediopalpini Monrós and Viana, 1947	Amplipalpini Weise, 1910	Use of younger name conserved (Art. 40.2)	this paper
Chrysomelidae	Oidini Laboissière, 1921	Adoriini Chapuis, 1875	Use of younger name conserved (Art. 40.2)	this paper
Chrysomelidae	Omocerini Hincks, 1952	Tauromini Spaeth, 1923	Use of younger name conserved (Art. 40.2)	this paper
Chrysomelidae	Sceloenoplini Uhmann, 1930	Cephalodontini Gestro, 1906	An application to the Commission is needed to conserve usage of the well-established name Sceloenoplini Uhmann, 1930	
Cryptophagidae	Ipidae/-inae/-ini Latreille, 1802	Cryptophagidae/-inae/-ini Kirby, 1826	An application to the Commission is needed to suppress the older name because it is based on a misidentified type genus (Art. 65.2.1)	
Curculionidae	Aterpini/-ina Lacordaire, 1863	Heliomeneini/-ina Gistel, 1848	Use of younger name conserved (Art. 23.9.2)	this paper
Curculionidae	Bagoinae Thomson, 1859	Lyprinae Gistel, 1848	Use of younger name conserved (Art. 23.9.2)	this paper
Curculionidae	Cleonini Schönherr, 1826	Geomorini Schönherr, 1823	Use of younger name conserved (Art. 23.9.2)	this paper
Curculionidae	Hyperinae/-ini Lacordaire, 1863	Phytonominae/-ini Gistel, 1848	Use of younger name conserved (Art. 40.2)	this paper
Curculionidae	Magdalidini Pascoe, 1870	Scardamyctini Gistel, 1848	Use of younger name conserved (Art. 23.9.2)	this paper
Curculionidae	Mesoptiliinae Lacordaire, 1863	Scardamyctinae Gistel, 1848	Use of younger name conserved (Art. 35.5)	this paper
Curculionidae	Naupactini Gistel, 1848	Iphiini Schönherr, 1823	Use of younger name conserved (Art. 23.9.2)	this paper
Curculionidae	Smicronychini Seidlitz, 1891	Desmorini LeConte, 1876	Use of younger name conserved (Art. 23.9.2)	this paper
Curculionidae	Tonesiina Alonso-Zarazaga and Lyal	Lyteriina Lacordaire, 1865	An application to the Commission is needed to suppress the older name because it is based on a misidentified type genus (Art. 65.2.1)	
Curculionidae	Zygobaridina Pierce, 1907	Centrinina Jekel, 1865	Use of younger name conserved because the older name is a junior homonym	Alonso-Zarazaga and Lyal (1999)
Dascilloidea	Dascilloidea/-idae/-inae/-ini Guérin-Méneville, 1843	Atopoidea/-idae/-inae/-ini Laporte, 1834	Use of younger name conserved (Art. 40.2)	Lawrence and Newton (1995)
Dascilloidea	Dascilloidea Guérin-Méneville, 1843	Rhipiceroidea Latreille, 1834	Use of younger name conserved because relationships between Dascillidae and Rhipiceridae are uncertain	
Derodontoidea	Derodontoidea LeConte, 1861	Nosodendroidea Erichson, 1846	Use of younger name conserved (Art. 35.5)	this paper
Dryopidae	Dryopidae Billberg, 1820	Parnidae Leach, 1817	Use of younger name conserved (Art. 40.2)	Lawrence and Newton (1995)
Elateridae	Agrypninae/-ini Candèze, 1857	Adelocerinae/-ini Gistel, 1848	Use of younger name conserved (Art. 23.9.2)	this paper
Elateridae	Agrypninae/-ini Candèze, 1857	Pangaurinae/-ini Gistel, 1856	Use of younger name conserved (Art. 23.9.2)	this paper
Elateridae	Agrypninae Candèze, 1857	Oophorinae Gistel, 1848	Use of younger name conserved (Art. 35.5)	this paper
Elateridae	Denticollinae/-ini/-ina Stein and Weise, 1877	Campylinae/-ini/-ina Gistel, 1848	Use of younger name conserved (Art. 40.2)	this paper
Elateridae	Hypnoidini Schwarz, 1906	Cryptohypnini Candèze, 1860	Use of younger name conserved (Art. 40.2)	this paper
Elateridae	Melanotini Candèe, 1859	Cratonychini Gistel, 1848	Use of younger name conserved (Art. 40.2)	Sánchez-Ruiz (1996)
Elateridae	Prosternini Gistel, 1856	Diacanthini Gistel, 1848	Use of younger name conserved (Art. 23.9.2)	this paper
Elateridae	Thylacosterninae Fleutiaux, 1920	Pterotarsinae Fleutiaux, 1902	An application to the Commission will be submitted by J. Muona and H. Silfverberg (pers. comm. 2010) to conserve usage of the well-established name Thylacosterninae Fleutiaux, 1920	
Elateroidea/-idae	Elateroidea/-idae Leach, 1815	Cebrionoidea/-idae Latreille, 1802	An application to the Commission will be sent to conserve usage of the well-established names Elateroidea/-idae Leach, 1815	P. J. Johnson (in Lawrence and Newton 1995, pers. comm. 2009)
Erotylidae	Encaustini Crotch, 1876	Engidini MacLeay, 1825	An application to the Commission is needed to suppress the older name because it is based on a misidentified type genus (Art. 65.2.1)	
Eucnemidae	Eucnemidae Eschscholtz, 1829	Melasidae Fleming, 1821	An application to the Commission has been sent to conserve usage of the well-established name Eucnemidae Fleming, 1821	see Appendix 6
Gyrinidae	Enhydrini Régimbart, 1882	Dineutini Desmarest, 1851	Use of younger name conserved (Art. 35.5)	this paper
Hydrophilidae	Coelostomatini Heyden, 1891	Cyclonotini Horn, 1890	Use of younger name conserved (Art. 40.2)	Hansen (1991)
Hygrobiidae	Hygrobiidae Régimbart, 1879	Pelobiinae Erichson, 1837	Use of younger name conserved (Art. 40.2)	Lawrence and Newton (1995)
Latridiidae	Latridiidae Erichson, 1842	Corticariidae Curtis, 1829	An application to the Commission was submitted by Bousquet et al. (2010) to conserve usage of the well-established name Latridiidae Erichson, 1842	
Leiodidae	Catopocerinae/-ini Hatch, 1927	Pinodytinae/-ini Horn, 1880	Use of younger name conserved (Art. 40.2)	Newton and Thayer (1992)
Leiodidae	Coloninae Horn, 1880	Myloechinae Thomson, 1859	Use of younger name conserved (Art. 40.2)	Newton and Thayer (1992)
Leiodidae	Leptodirini/-ina Lacordaire, 1854	Stagobiini/-ina Schiødte, 1849	Use of younger name conserved (Art. 40.2)	Newton and Thayer (1992)
Lepiceroidea	Lepiceroidea/-idae Hinton, 1936	Cyathoceroidea/-idae Sharp, 1882	Use of younger name conserved (Art. 40.2)	Lawrence and Newton (1995)
Lymexyloidea	Lymexyloidea/-idae Fleming, 1821	Hylecoetoidea/-idae Germar, 1818	Use of younger name conserved (Art. 35.5)	this paper
Melandryidae	Osphyinae Mulsant, 1856	Nothinae Shuckard, 1839	Use of younger name conserved (Art. 40.2)	Lawrence and Newton (1995)
Meloidae	Epicautini Parker and Böving, 1924	Macrobasini LeConte, 1862	An application to the Commission is needed to conserve usage of the well-established name Epicautini Parker and Böving, 1924	
Meloidae	Epicautini Parker and Böving, 1924	Apterospastini Wellman, 1910	An application to the Commission is needed to conserve usage of the well-established name Epicautini Parker and Böving, 1924	
Meloidae	Lyttini Solier, 1851	Cantharini Latreille, 1802	An application to the Commission is needed to suppress the older name because it is based on a misidentified type genus (Art. 65.2.1)	
Meloidae	Meloidae Gyllenhal, 1810	Horiidae Latreille, 1802	Younger name given precedence over older name and placed on the Official List of Family-Group Names in Zoology	ICZN (1999c)
Meloidae	Meloidae/-inae Gyllenhal, 1810	Cantharidae/-inae Latreille, 1802	An application to the Commission is needed to suppress the older name because it is based on a misidentified type genus (Art. 65.2.1)	
Meloidae	Nemognathinae Laporte, 1840	Horiinae Latreille, 1802	Younger name given precedence over older name and placed on the Official List of Family-Group Names in Zoology	ICZN (1999c)
Nemonychidae	Nemonychidae Bedel, 1882	Cimberididae Gozis, 1882	Younger name given precedence over older name and placed on the Official List of Family-Group Names in Zoology	ICZN (2005c)
Oedemeridae	Calopodinae Costa, 1852	Sparedrinae Gistel, 1848	Use of younger name conserved (Art. 23.9.2)	this paper
Psephenidae	Placonychinae Horn, 1880	Eubrianacinae Jakobson, 1913	Use of younger name conserved (Art. 40.2)	Lawrence and Newton (1995)
Ptiliidae	Acrotrichinae Reitter, 1909	Cleopteriinae Gistel, 1856	Use of younger name conserved (Art. 40.2)	Newton and Thayer (1992)
Ptiliidae	Ptinellini Reitter, 1906	Neuglenini Reitter, 1891	Use of younger name conserved (Art. 40.2)	this paper
Ptilodactylidae	Anchytarsinae Champion, 1897	Coloboderinae Erichson, 1847	An application to the Commission is needed to conserve usage of the well-established name Anchytarsinae Champion, 1897	
Ripiphoridae	Macrosiagonini Heyden, 1908	Ripiphorini Laporte, 1840	An application to the Commission is needed to suppress the older name because it is based on a misidentified type genus (Art. 65.2.1)	
Ripiphoridae	Ripiphoridae/-inae/-ini Gemminger, 1870	Myoditidae/-inae/-ini Gerstaecker, 1855	Use of younger name conserved (Art. 40.2)	Lawrence and Newton (1995)
Scarabaeidae	Anomalini/-ina Streubel, 1839	Euchlorini/-ina Hope, 1839	Use of younger name conserved (Art. 23.9.2)	Smith (2006)
Scarabaeidae	Cremastocheilini Burmeister and Schaum, 1841	Macromini Burmeister and Schaum, 1840	Use of younger name conserved (Art. 35.5)	Smith (2006)
Scarabaeidae	Eupariini Schmidt, 1910	Ataeniini Harold, 1868	Use of younger name conserved (Art. 23.9.2)	Smith (2006)
Scarabaeidae	Heterosternina Bates, 1888	Macropnina Horn, 1867	Use of younger name conserved (Art. 23.9.2)	Smith (2006)
Scarabaeidae	Oniticellini Kolbe, 1905	Drepanocerina van Lansberge, 1875	Use of younger name conserved (Art. 35.5)	Smith (2006)
Schizophoroidea	Schizophoroidea Ponomarenko, 1968	Schizocoleoidea Rohdendorf, 1961	Use of younger name conserved (Art. 35.5)	this paper
Sphindidae	Sphindidae/-inae Jacquelin Du Val, 1860	Aspidiphoridae/-inae Kiesenwetter, 1877 (1859)	Ruled under the plenary power that the younger name is to be given precedence over the older name	ICZN (1997)
Staphylinidae	Astenina Hatch, 1857	Suniina Sharp, 1886	An application to the Commission is needed to suppress the older name because it is based on a misidentified type genus (Art. 65.2.1)	
Staphylinidae	Athetini/-ina Casey, 1910	Callicerini Jakobson, 1908	An application to the Commission was submitted by Gusarov to conserve usage of the well-established names Athetini/-ina Casey, 1910	
Staphylinidae	Dolicaonina Casey, 1905	Gnathymenina Solier, 1849	An application to the Commission is needed to conserve usage of the well-established name Dolicaonina Casey, 1905	
Staphylinidae	Eusphalerini Hatch, 1957	Anthobiini Portevin, 1929	An application to the Commission is needed to suppress the older name because it is based on a misidentified type genus (Art. 65.2.1)	
Staphylinidae	Goniaceritae/-ini Reitter, 1882	Goniastitae/-ini Schaufuss, 1872	Use of younger name conserved (Art. 40.2)	Newton and Thayer (1992)
Staphylinidae	Xantholinini Erichson, 1839	Agrodini Nordmann, 1837	Younger name given precedence over older names and placed on the Official List of Family-Group Names in Zoology	ICZN (1996c)
Staphylinidae	Xantholinini Erichson, 1839	Gyrohypnini Kirby, 1837	Younger name given precedence over older names and placed on the Official List of Family-Group Names in Zoology	ICZN (1996c)
Staphylinidae	Xanthopygina Sharp, 1884	Platycnemina Nordmann 1837	Younger name given precedence over older name and placed on the Official List of Family-Group Names in Zoology	ICZN (1996c)
Tenebrionidae	Adelinina LeConte, 1862	Alphitophagina Gistel, 1856	An application to the Commission is needed to conserve usage of the well-established name Adelinina LeConte, 1862	
Tenebrionidae	Adesmiini Lacordaire, 1859	Megageniini Solier, 1851	Use of younger name conserved (Art. 23.9.2)	Bouchard et al. (2007)
Tenebrionidae	Adesmiini Lacordaire, 1859	Macropodini Agassiz, 1846	Use of younger name conserved (Art. 23.9.2)	this paper
Tenebrionidae	Alleculinae Laporte, 1841	Cteniopodinae Solier, 1835	Use of younger name conserved (Art. 35.5)	Bouchard et al. (2005)
Tenebrionidae	Alleculinae/-ini Laporte, 1840	Xystropodinae/-ini Solier, 1835	Use of younger name conserved (Art. 35.5)	Bouchard et al. (2005)
Tenebrionidae	Bolitophagini Kirby, 1837	Eledonini Billberg, 1820	Use of younger name conserved (Art. 23.9.2)	this paper
Tenebrionidae	Cryptoglossini LeConte, 1862	Centriopterini Lacordaire, 1859	Use of younger name conserved (Art. 23.9.2)	Aalbu (2006)
Tenebrionidae	Epitragini Blanchard, 1845	Lygophilini Rafinesque, 1815	Use of younger name conserved (Art. 23.9.2)	Bouchard et al. (2007)
Tenebrionidae	Erodiini Billberg, 1820	Cephacerini Rafinesque, 1815	Use of younger name conserved (Art. 23.9.2)	Bouchard et al. (2007)
Tenebrionidae	Lagriinae Latreille, 1825	Cossyphinae Latreille, 1802	Use of younger name conserved because placement of older taxon uncertain	Bouchard et al. (2005)
Tenebrionidae	Lagriinae/-ini/-ina Latreille, 1825	Lachninae/-ini/-ina Billberg, 1820	Use of younger name conserved (Art. 40.2)	Bouchard et al. (2005)
Tenebrionidae	Melanimini Seidlitz, 1894	Microzoini Mulsant, 1854	Use of younger name conserved (Art. 40.2)	Bouchard et al. (2005)
Tenebrionidae	Pycnocerini Lacordaire, 1859	Chiroscelini Laporte, 1840	Use of younger name conserved (Art. 23.9.2)	Bouchard et al. (2005)
Tenebrionidae	Stenosini Lacordaire, 1859	Tagenini Solier, 1834	Use of younger name conserved (Art. 40.2)	Bouchard et al. (2005)
Tenebrionidae	Talanini Champion, 1887	Dignamptini LeConte and Horn, 1883	Use of younger name conserved (Art. 40.2)	Bouchard et al. (2005)
Throscidae	Throscidae Laporte, 1840	Stereolidae Rafinesque, 1815	Use of younger name conserved (Art. 23.9.2)	this paper
Trogossitidae	Lophocaterini Crowson, 1964	Lycoptini Casey, 1890	Use of younger name conserved (Art. 23.9.2)	this paper
Zopheridae	Zopheridae Solier, 1834	Colydiidae Billberg, 1820	Use of younger name conserved (Art. 35.5)	this paper

## Appendix 3

Coleoptera family-group name changes required based on the Principle of Homonymy. The action that has been taken to fix the problem, or recommendation for future work, is mentioned for each case. Cases listed in alphabetical order by family.

**Table:**

Family	Coleoptera name:	Homonym:	Type genera	Action	Reference
Anthicidae	Notoxinae Stephens, 1829	Notoxini Sturm, 1826 [Coleoptera: Cleridae]	same, older name based on misidentified type genus	An application to the Commission is needed to suppress the older name because it is based on a misidentified type genus (Art. 65.2.1)	
Anthicidae	Steropinae Jacquelin du Val, 1863	Steropinae Dana, 1854 [Copepoda]	different	An application to the Commission is needed to remove the homonymy (Art. 55.3.1)	
Anthribidae	Apolectini Lacordaire, 1865	Apolectidae Jordan, 1923	different	An application to the Commission is needed to remove the homonymy (Art. 55.3.1)	
Anthribidae	Eupariini Valentine, 1960	Eupariina Schmidt, 1910 [Coleoptera: Scarabaeidae]	different	An application to the Commission is needed to remove the homonymy (Art. 55.3.1)	
Attelabidae	Anisonychina Legalov, 2003	Anisonychidae Burmeister, 1844 [Coleoptera: Scarabaeidae]	different	An application to the Commission is needed to remove the homonymy (Art. 55.3.1)	
Brachyceridae	Aonychini Zimmerman, 1993	Aonychini Davis, 1978 [Mammalia]	different	An application to the Commission is needed to remove the homonymy (Art. 55.3.1)	
Brachyceridae	Brotheini Marshall, 1907	Brotheinae Simon, 1879 [Arachnida]	different	An application to the Commission is needed to remove the homonymy (Art. 55.3.1)	
Buprestidae	Anaglyptina Gistel, 1848	Anaglyptini Lacordaire, 1868 [Coleoptera: Cerambycidae]	different	Older name treated as a nomen oblitum (Art. 23.9.2)	Bousquet et al. (2009)
Cantharidae	Cantharidae Imhoff, 1856	Cantharidae Latreille, 1802	same, older name based on misidentified type genus	An application to the Commission is needed to suppress the older name because it is based on a misidentified type genus (Art. 65.2.1)	
Carabidae	Agonidae Kirby, 1837	Agonidae Swainson, 1839 [Pisces]	different	Stem of beetle name emended and Agoniumidae Kirby, 1837 placed on the Official List of Family-Group Names in Zoology	ICZN (1996d)
Carabidae	Amblytelini Blackburn, 1892	Amblytelinae Viereck, 1918 [Hymenoptera]	different	An application to the Commission is needed to remove the homonymy (Art. 55.3.1)	
Carabidae	Arthropterini Wasmann, 1928	Arthropteridae Jordan, 1923 [Pisces]	same, Coleoptera genus older	An application to the Commission is needed to conserve the Coleoptera name	
Carabidae	Bradybaenina Csiki, 1932	Bradybaenidae Pilsbry, 1939 [Mollusca]	different	An application to the Commission is needed to remove the homonymy (Art. 55.3.1)	
Carabidae	Colpodini Chaudoir, 1872	Colpodidae Poche, 1913 [Protista]	different	An application to the Commission is needed to remove the homonymy (Art. 55.3.1)	
Carabidae	Granigerina Antoine, 1959 [Carabidae: Harpalinae]	Granigerini Bedel, 1900 [Carabidae: Trechinae]	same, older name based on misidentified type genus	An application to the Commission is needed to suppress the older name because it is based on a misidentified type genus (Art. 65.2.1)	
Carabidae	Nomiini Gozis, 1875	Nomiidae Robertson, 1904 [Hymenoptera]	different	An application was submitted to the Commission to emend the stem of the beetle family-group name	Engel and Bouchard (2009)
Cerambycidae	Anaglyptini Lacordaire, 1868	Anaglyptina Gistel, 1848 [Coleoptera: Buprestidae]	different	Older name treated as a nomen oblitum (Art. 23.9.2)	Bousquet et al. (2009)
Cerambycidae	Compsina Martins and Galileo, 2007	Compsini Pierce, 1913 [Coleoptera: Curculionidae]	different	An application to the Commission is needed to remove the homonymy (Art. 55.3.1)	
Cerambycidae	Dryobiini Arnett, 1962	Dryobiini Gistel, 1856 [Coleoptera: Ptinidae]	different	Older name treated as a nomen oblitum	Bousquet et al. (2009)
Cerambycidae	Lissonotini Swainson, 1840	Lissonotini Förster, 1869 [Hymenoptera]	different	An application to the Commission is needed to remove the homonymy (Art. 55.3.1)	
Cerambycidae	Platysternini Lacordaire, 1872	Platysternidae Gray, 1869 [Reptilia]	different	An application to the Commission is needed to remove the homonymy (Art. 55.3.1)	
Cerambycidae	Polyarthrini Gounelle, 1911	Polyarthridae Daday, 1893 [Rotifera]	different	An application to the Commission is needed to remove the homonymy (Art. 55.3.1)	
Cerambycidae	Sphaeriini Lacordaire, 1868	Sphaeriidae Deshayes, 1855 [Mollusca]	different	An application to the Commission is needed to remove the homonymy (Art. 55.3.1)	
Cerambycidae	Sphaeriini Lacordaire, 1868	Sphaeriidae Erichson, 1845 [Coleoptera: Myxophaga]	different	Stem of older beetle name emended and Sphaeriusidae Erichson, 1845 placed on the Official List of Family-Group Names in Zoology	ICZN (2000)
Cerambycidae	Stenocorini Thomson, 1861 [Cerambycidae: Lepturinae]	Stenocorini Hope, 1834 [Cerambycidae: Cerambycinae]	same, older name based on misidentified type genus	An application to the Commission is needed to suppress the older name because it is based on a misidentified type genus (Art. 65.2.1)	
Cerambycidae	Stenoderini Pascoe, 1867	Stenoderini Selander, 1991 [Coleoptera: Meloidae]	different	An application to the Commission is needed to remove the homonymy (Art. 55.3.1)	
Cerambycidae	Stenodontini Lameere, 1903	Stenodontina Schmiedeknecht, 1903 [Hymenoptera]	different	An application to the Commission is needed to remove the homonymy (Art. 55.3.1)	
Cerambycidae	Toxotini LeConte and Horn, 1883	Toxotidae Günther, 1860 [Pisces]	different	An application to the Commission is needed to remove the homonymy (Art. 55.3.1)	
Chaetosomatidae	Chaetosomatidae Crowson, 1952	Chaetosomatidae Claus, 1872 [Nematoda]	same, Coleoptera genus older	An application to the Commission was submitted by YB and PB to conserve the Coleoptera name	
Chrysomelidae	Carpophagini Chapuis, 1874	Carpophaginae Selby, 1835 [Aves]	different	An application to the Commission is needed to remove the homonymy (Art. 55.3.1)	
Chrysomelidae	Chalepini Weise, 1910 [Chrysomelidae: Cassidinae]	Chalepidae Burmeister, 1847 [Scarabaeidae: Dynastinae]	same, Scarabaeidae name older	An application to the Commission is needed to conserve usage of the well established Chrysomelidae name	
Chrysomelidae	Oreinini Bechyné, 1958	Oreinini Bleeker, 1863 [Pisces]	different	An application to the Commission is needed to remove the homonymy (Art. 55.3.1)	
Chrysomelidae	Megacerini Bridwell, 1946	Megacerini Viret, 1961 [Mammalia]	different	An application to the Commission is needed to remove the homonymy (Art. 55.3.1)	
Chrysomelidae	Spermophagina Borowiec, 1987 [Chrysomelidae: Bruchinae: Amblycerini: Spermophagina]	Spermophagina Crotch, 1873 [Chrysomelidae: Bruchinae: Amblycerini: Amblycerina]	same, older name based on misidentified type genus	An application to the Commission is needed to suppress the older name because it is based on a misidentified type genus (Art. 65.2.1)	
Chrysomelidae	Spilophorini Chapuis, 1875 [Chrysomelidae: Cassidinae]	Spilophorina Krikken, 1984 [Coleoptera: Scarabaeidae]	different	An application to the Commission is needed to remove the homonymy (Art. 55.3.1)	
Cleridae	Trichodini Portevin, 1931	Trichodina Maitland, 1851[Protozoa]	different	An application to the Commission is needed to remove the homonymy (Art. 55.3.1)	
Cryptophagidae	Ipini Latreille, 1802 [Cryptophagidae: Cryptophaginae: Cryptophagini]	Ipini Bedel, 1888 [Curculionidae: Scolytinae]	same, older name based on misidentified type genus	An application to the Commission is needed to suppress the older name because it is based on a misidentified type genus (Art. 65.2.1)	
Curculionidae	Centrinina Jekel, 1865	Centrininae Swainson, 1839 [Pisces]	different	The younger name Zygobaridina is used as valid instead of Centrinina	Alonso-Zarazaga and Lyal (1999)
Curculionidae	Compsini Pierce, 1913	Compsina Martins and Galileo, 2007 [Coleoptera: Cerambycidae]	different	An application to the Commission is needed to remove the homonymy (Art. 55.3.1)	
Curculionidae	Conoderinae Schönherr, 1833	Conoderini Fleutiaux, 1919 [Coleoptera: Elateridae]	different	An application to the Commission is needed to remove the homonymy (Art. 55.3.1)	
Curculionidae	Euderini Lacordaire, 1865	Euderini Erdös, 1956 [Hymenoptera]	different	A junior synonym is used as valid instead of Euderini in Hymenoptera	Hansson and Straka (2009)
Curculionidae	Haplonychini Lacordaire, 1865	Haplonychidae Burmeister, 1855 [Coleoptera: Scarabaeidae]	different	An application to the Commission is needed to remove the homonymy (Art. 55.3.1)	
Curculionidae	Ipini Bedel, 1888 [Curculionidae: Scolytinae]	Ipini Latreille, 1802 [Cryptophagidae: Cryptophaginae: Cryptophagini]	same, older name based on misidentified type genus	An application to the Commission is needed to suppress the older name because it is based on a misidentified type genus (Art. 65.2.1)	
Curculionidae	Omiini Shuckard, 1839	Omiini Beck, 1996 [Lepidoptera]	different	An application to the Commission is needed to remove the homonymy (Art. 55.3.1)	
Curculionidae	Phaenomerina Faust, 1898	Phaenomerini Erichson, 1847 [Coleoptera: Scarabaeidae]	different	Stem of Scarabaeidae name emended and Phaenomerididae (with incorrect author and year “Ohaus, 1913”) placed on the Official List of Family-Group Names in Zoology	ICZN (1962)
Curculionidae	Rhynchaenini Blanchard, 1853	Rhynchaenini Latreille, 1828	same, older name based on misidentified type genus	An application to the Commission is needed to suppress the older name because it is based on a misidentified type genus (Art. 65.2.1)	
Curculionidae	Tomicini Wood, 1978 [Curculionidae: Scolytinae: Hylurgini]	Tomicini Shuckard, 1839 [Curculionidae: Scolytinae: Ipini]	same, older name based on misidentified type genus	An application to be submitted to the Commission by MAAZ and CHCL to suppress the older name because it is based on a misidentified type genus (Art. 65.2.1)	
Curculionidae	Trypetini Lacordaire, 1865	Trypetidae Loew, 1861[Diptera]	different	Stem of beetle name emended and Trypetidinae (with incorrect author and year “Pierce, 1919”) placed on the Official List of Family-Group Names in Zoology	ICZN (1974a)
Elateridae	Conoderini Fleutiaux, 1919	Conoderinae Schönherr, 1833 [Coleoptera: Curculionidae]	different	An application to the Commission is needed to remove the homonymy (Art. 55.3.1)	
Elateridae	Drasteriini Houlbert, 1912	Drasteriini Wiltshire, 1976 [Lepidoptera]	different	An application to the Commission is needed to remove the homonymy (Art. 55.3.1)	
Elateridae	Ludiini Candèze, 1857	Ludiini Aurivilius, 1904 [Lepidoptera]	different	The Lepidoptera name was replaced by Micragonini Cockerell, 1914	Oberprieler (1997)
Elateroidea	Cantharidae Imhoff, 1856	Cantharidae Latreille, 1802	same, older name based on misidentified type genus	An application to the Commission is needed to suppress the older name because it is based on a misidentified type genus (Art. 65.2.1)	
Elmidae	Larini LeConte, 1861	Laridae Rafinesque-Schmaltz, 1815 [Aves]	different	Stem of beetle name emended and Laraini LeConte, 1861 placed on the Official List of Family-Group Names in Zoology	ICZN (1988g)
Gyrinidae	Enhydrini Régimbart, 1882	Enhydrini Gray, 1825	different	An application was recently submitted to the Commission by Özdikmen and Darilmaz (2010) to remove the homonymy	
Helotidae	Helotidae Chapuis, 1876	Helotidae Adams et al., 1854 [Pisces]	different	An application to the Commission is needed to remove the homonymy (Art. 55.3.1)	
Hydrophilidae	Hydrobiina Mulsant, 1844	Hydrobiidae Troschel 1857 [Mollusca]	different	Stem of beetle name emended and Hydrobiusina Mulsant, 1844 placed on the Official List of Family-Group Names in Zoology	ICZN (2003a)
Lampyridae	Photinini LeConte, 1881	Photininae Giglio-Tos, 1915 [Mantodea]	different	An application to the Commission was submitted by Svenson and Branham (2007) to remove the homonymy (Art. 55.3.1)	
Leiodidae	Anisotomidae Reitter, 1884 [Leiodinae: Agathidiini]	Anisotomidae Erichson, 1845 [Leiodinae: Leiodini]	same, older name based on misidentified type genus	An application to the Commission is needed to suppress the older name because it is based on a misidentified type genus (Art. 65.2.1)	
Leiodidae	Triarthrini Jeannel, 1962	Triarthridae Ulrich, 1930 [Trilobita]	different	An application to the Commission is needed to remove the homonymy (Art. 55.3.1)	
Meloidae	Lydina Kaszab, 1959	Lydidae Newman, 1834 [Hymenoptera]	different	An application to the Commission is needed to remove the homonymy (Art. 55.3.1)	
Meloidae	Stenoderini Selander, 1991	Stenoderini Pascoe, 1867 [Coleoptera: Cerambycidae]	different	An application to the Commission is needed to remove the homonymy (Art. 55.3.1)	
Meloidae	Zonitina Mulsant, 1857	Zonitidae Mörch, 1864 [Mollusca]	different	Stem of beetle name emended and Zonitidinae Mulsant, 1857 placed on the Official List of Family-Group Names in Zoology	ICZN (1999c)
Plastoceridae	Plastoceridae Crowson, 1972	Plastocerini LeConte, 1861 [Coleoptera: Elateridae]	same, older name based on misidentified type genus	An application to the Commission is needed to suppress the older name because it is based on a misidentified type genus (Art. 65.2.1)	
Prionoceridae	Prionoceridae Lacordaire, 1857	Prionocerini Savchenko, 1966 [Diptera]	different	An application to the Commission is needed to remove the homonymy (Art. 55.3.1)	
Ptinidae	Dryobiini Gistel, 1856	Dryobiini Arnett, 1962 [Coleoptera: Cerambycidae]	different	Older name treated as a nomen oblitum (Art. 23.9.2)	Bousquet et al. (2009)
Ripiphoridae	Ripiphoridae/-inae/-ini Gemminger, 1870 [Ripiphoridae: Ripiphorinae: Ripiphorini]	Ripiphoridae/-inae/-ini Laporte, 1840 [Ripiphoridae: Ripiphorinae: Macrosiagonini]	same, older name based on misidentified type genus	An application to the Commission is needed to suppress the older name because it is based on a misidentified type genus (Art. 65.2.1)	
Salpingidae	Elacatidae Cockerell, 1906	Elacatinae Gill, 1861 [Pisces]	different	An application to the Commission is needed to remove the homonymy (Art. 55.3.1)	
Scarabaeidae	Anisonychidae Burmeister, 1844	Anisonychini Legalov, 2003 [Coleoptera: Attelabidae]	different	An application to the Commission is needed to remove the homonymy (Art. 55.3.1)	
Scarabaeidae	Chalepidae Burmeister, 1847 [Scarabaeidae: Dynastinae]	Chalepini Weise, 1910 [Chrysomelidae: Cassidinae]	same, Scarabaeidae name older	An application to the Commission is needed to conserve usage of the well established name in Chrysomelidae	
Scarabaeidae	Cryptodontina Lacordaire, 1856	Cryptodontidae Dall, 1895 [Mollusca]	different	An application to the Commission is needed to remove the homonymy (Art. 55.3.1)	
Scarabaeidae	Euchroeina Paulian and Descarpentries, 1982	Euchroeidae Dahlbom, 1854 [Hymenoptera]	different	An application to the Commission is needed to remove the homonymy (Art. 55.3.1)	
Scarabaeidae	Eupariina Schmidt, 1910	Eupariini Valentine, 1960 [Coleoptera: Anthribidae]	different	An application to the Commission is needed to remove the homonymy (Art. 55.3.1)	
Scarabaeidae	Haplonychidae Burmeister, 1855	Haplonychini Lacordaire, 1865 [Coleoptera: Curculionidae]	different	An application to the Commission is needed to remove the homonymy (Art. 55.3.1)	
Scarabaeidae	Macrophyllina Burmeister, 1855	Macrophyllina Gray, 1866 [Mammalia]	different	An application to the Commission is needed to remove the homonymy (Art. 55.3.1)	
Scarabaeidae	Phaenomerini Erichson, 1847	Phaenomerina Faust, 1898 [Coleoptera: Curculionidae]	different	Stem of Scarabaeidae name emended and Phaenomeridini Ohaus, 1913 [in error for Erichson, 1847] placed on the Official List of Family-Group Names in Zoology	ICZN (1962)
Scarabaeidae	Spilophorina Krikken, 1984	Spilophorini Chapuis, 1875 [Coleoptera: Chrysomelidae]	different	An application to the Commission is needed to remove the homonymy (Art. 55.3.1)	
Scarabaeidae	Trichiini Fleming, 1821	Trichiinae Lozek, 1956 [Mollusca]	different	The mollusc name was placed on the Official Index of Rejected and Invalid Family-Group Names in Zoology	ICZN (2004c)
Scarabaeidae	Trigonostomina Ohaus, 1912	Trigonostomidae Graff, 1905 [Platyhelminthes]	different	Stem of beetle name emended and Trigonostomusina Ohaus, 1912 placed on the Official List of Family-Group Names in Zoology	ICZN (2009b)
Sphaeriusidae	Sphaeriidae Erichson, 1845	Sphaeriini Lacordaire, 1868 [Coleoptera: Cerambycidae] and Sphaeriidae Deshayes, 1855 [Mollusca]	different	Stem of older beetle name emended and Sphaeriusidae Erichson, 1845 placed on the Official List of Family-Group Names in Zoology	ICZN (2000)
Staphylinidae	Callicerina Jakobson, 1908	Callicerina Rondani, 1856 [Diptera]	different	An application to the Commission was recently submitted by Gusarov to suppress the Coleoptera name	
Staphylinidae	Metopiini Raffray, 1904	Metopiinae Foerster, 1869 [Hymenoptera]	different	Stem of beetle name emended and Metopiasini Raffray, 1904 placed on the Official List of Family-Group Names in Zoology	ICZN (1994d)
Staphylinidae	Tachinidae Fleming, 1821	Tachinidae Robineau-Desvoidy, 1830 [Diptera]	different	Stem of beetle name emended and Tachinusidae Fleming, 1821 placed on the Official List of Family-Group Names in Zoology	ICZN (1993f)
Staphylinidae	Toxoderini Bernhauer and Schubert, 1911	Toxoderini Saussure, 1869 [Mantodea]	different	An application to the Commission is needed to remove the homonymy (Art. 55.3.1)	
Tenebrionidae	Adeliini Kirby, 1828	Adeliini Viereck, 1918 [Hymenoptera]	different	An application to the Commission is needed to remove the homonymy (Art. 55.3.1)	
Tenebrionidae	Lachnini Billberg, 1820	Lachnini Herrich-Schaeffer 1854 [Hemiptera]	different	An application to the Commission is needed to remove the homonymy (Art. 55.3.1)	
Tenebrionidae	Macropodini Agassiz, 1846	Macropodidae Gray, 1821 [Mammalia]	different	Beetle name treated as a nomen oblitum (Art. 23.9.2)	this paper
Tenebrionidae	Pentaphyllini Mulsant, 1854	Pentaphyllinae Schindewolf, 1942 [Anthozoa]	different	An application to the Commission is needed to remove the homonymy (Art. 55.3.1)	

## Appendix 4

Coleoptera names as they appear on the *Official List of Family-Group Names in Zoology*. Cases listed in alphabetical order by family. Names on the *Official List* with the symbol “°” differ from those presented in our catalogue (spelling, authorship and/or year of publication).

**Table:**

Family	Name on Official List	Opinion(s) or Direction	Reference
Aderidae	°Aderidae Winkler, 1927	1549	ICZN 1989e
Aderidae	Euglenesidae Seidlitz, 1875	1549	ICZN 1989e
Anthribidae	Anthribidae Billberg, 1820	1756	ICZN 1994b
Anthribidae	Choragidae Kirby, 1819	1756	ICZN 1994b
Brentidae	°Eurhinini Lacordaire, 1866	1352	ICZN 1985e
Brentidae	Eurhynchinae Lacordaire, 1863	1352	ICZN 1985e
Buprestidae	Tracheidae Laporte, 1835	2222	ICZN 2009a
Carabidae	Agonumidae Kirby, 1837	1855	ICZN 1996d
Carabidae	Dromiusidae Bonelli, 1810	2149	ICZN 2006b
Carabidae	°Galeritini Lacordaire, 1854	862	ICZN 1968b
Cerylonidae	Cerylonidae Billberg, 1820	1811	ICZN 1995c
Chrysomelidae	Bruchidae Latreille, 1802	1809	ICZN 1995a
Chrysomelidae	°Cassidinae Stephens, 1831	1023	ICZN 1974b
Chrysomelidae	°Uroplatini Leng, 1920	1359	ICZN 1985f
Curculionidae	°Brachyderinae Schönherr, 1837	1440	ICZN 1987a
Curculionidae	Cryptorhynchinae Schönherr, 1825	808	ICZN 1967b
Curculionidae	Otiorhynchinae Schönherr, 1826	982	ICZN 1972
Curculionidae	Pachyrhynchini Schönherr, 1826	928	ICZN 1970b
Curculionidae	Phaenomerina Faust, 1898	621	ICZN 1962
Curculionidae	Phyllobiini Schönherr, 1826	1179	ICZN 1981c
Curculionidae	°Polydrosini Schönherr, 1823	1179	ICZN 1981c
Curculionidae	°Scolytidae Westwood, 1838	683	ICZN 1963b
Curculionidae	Somatodinae Lacordaire, 1863	1770	ICZN 1994c
Curculionidae	°Trypetidinae Pierce, 1919	1005	ICZN 1974a
Dytiscidae	°Dytiscidae Leach, 1817	619	ICZN 1961e
Elmidae	Elmidae Curtis, 1830	1812	ICZN 1995d
Elmidae	Laraini LeConte, 1861	1515	ICZN 1988g
Geotrupidae	°Geotrupini Latreille, 1806	Direction 28	ICZN 1955c
Hydrophilidae	Hydrobiusina Mulsant, 1844	2034	ICZN 2003a
Kateretidae	Brachypterinae Erichson, [1845]	1916	ICZN 1999a
Kateretidae	°Kateretidae Erichson in Agassiz, [1846]	1916	ICZN 1999a
Meloidae	Horiidae Latreille, 1802	1918	ICZN 1999b
Meloidae	Meloidae Gyllenhal, 1810	1918	ICZN 1999b
Meloidae	Nemognathinae Castelnau, 1840	1918	ICZN 1999b
Meloidae	Zonitidinae Mulsant, 1857	1918	ICZN 1999b
Nemonychidae	Cimberididae Gozis, 1882	2111	ICZN 2005c
Nemonychidae	Nemonychidae Bedel, 1882	2111	ICZN 2005c
Orsodacnidae	Aulacoscelidinae Chapuis, 1874	2242	ICZN 2010a
Ptinidae	Ptinidae Latreille, 1802	1809	ICZN 1995a
Scarabaeidae	°Phaenomerididae Ohaus, 1913	621	ICZN 1962
Scarabaeidae	Trigonostomusina Ohaus, 1912	2229	ICZN 2009b
Sphaeriusidae	Microsporidae Crotch, 1873	1331, 1957	ICZN 1985d, 2000
Sphaeriusidae	Sphaeriusidae Erichson, 1845	1957	ICZN 2000
Sphindidae	Aspidiphoridae Kiesenwetter, 1877	1862	ICZN 1997
Sphindidae	°Sphindidae Jacquelin du Val, [1861]	1862	ICZN 1997
Staphylinidae	Agrodini Nordmann, 1837	1851	ICZN 1996c
Staphylinidae	Bolitocharini Thomson, 1859	599	ICZN 1961a
Staphylinidae	Gyrohypnini Kirby, 1837	1851	ICZN 1996c
Staphylinidae	Metopiasini Raffray, 1904	1772	ICZN 1994d
Staphylinidae	Oxypodides Thomson, 1859	463	ICZN 1957
Staphylinidae	Platycnemini Nordmann, 1837	1851	ICZN 1996c
Staphylinidae	Quediini Kraatz, [1857]	1851	ICZN 1996c
Staphylinidae	°Staphylinidae Latreille, [1803-1804]	546	ICZN 1959a
Staphylinidae	Tachinusidae Fleming, 1821	1743	ICZN 1993f
Staphylinidae	Tachyporidae MacLeay, 1825	1743	ICZN 1993f
Staphylinidae	Xantholinini Erichson, 1839	1851	ICZN 1996c
Zopheridae	°Colydiidae Erichson, 1842	1811	ICZN 1995c
Zopheridae	Orthocerini Blanchard, 1845 (1820)	1811	ICZN 1995c
Zopheridae	Rhopalocerini Reitter, 1911	1397	ICZN 1986b

## Appendix 5

Coleoptera type genus names as they appear on the *Official List of Generic Names in Zoology*. Cases listed in alphabetical order by family.

**Table:**

Family	Name on Official List	Opinion(s)	Reference(s)
Aderidae	*Aderus* Stephens, 1829	1549	ICZN 1989e
Aderidae	*Euglenes* Westwood, 1830	1549	ICZN 1989e
Anthribidae	*Anthribus* Geoffroy, 1762	1754	ICZN 1994a
Anthribidae	*Mecocerus* Schönherr, 1833	982	ICZN 1972
Anthribidae	*Choragus* Kirby, 1819	1756	ICZN 1994b
Attelabidae	*Attelabus* Linnaeus, 1758	1239	ICZN 1983a
Bostrichidae	*Bostrichus* Geoffroy, 1762	1754	ICZN 1994a
Brentidae	*Eurhynchus* Kirby, in Kirby & Spence, 1828	1352	ICZN 1985
Brentidae	*Nanophyes* Schönherr, 1838	1526	ICZN 1989b
Buprestidae	*Acmaeodera* Eschscholtz, 1829	2100	ICZN 2005b
Buprestidae	*Actenodes* Dejean, 1833	2008	ICZN 2002b
Buprestidae	*Anthaxia* Eschscholtz, 1829	2009	ICZN 2002c
Buprestidae	*Astraeus* Laporte & Gory, 1837	795	ICZN 1966
Buprestidae	*Buprestis* Linnaeus, 1758	1784	ICZN 1994e
Buprestidae	*Chrysobothris* Eschscholtz, 1829	1784	ICZN 1994e
Buprestidae	*Chrysodema* Laporte & Gory, 1835	2076	ICZN 2004b
Buprestidae	*Dicerca* Eschscholtz, 1829	1784	ICZN 1994e
Buprestidae	*Iridotaenia* Deyrolle, 1864	2076	ICZN 2004b
Buprestidae	*Melanophila* Eschscholtz, 1829	1826	ICZN 1996b
Buprestidae	*Nascio* Gory & Laporte, 1838	2008	ICZN 2002b
Buprestidae	*Poecilonota* Eschscholtz, 1829	1825	ICZN 1996a
Buprestidae	*Trachys* Fabricius, 1801	2222	ICZN 2009a
Byrrhidae	*Byrrhus* Linnaeus, 1767	1754	ICZN 1994a
Byrrhidae	*Simplocaria* Stephens, 1829	1323	ICZN 1985c
Carabidae	*Agonum* Bonelli, 1810	1855	ICZN 1996d
Carabidae	*Carabus* Linnaeus, 1758	243	ICZN 1950
Carabidae	*Dromius* Bonelli, 1810	2149	ICZN 2006b
Carabidae	*Galerita* Fabricius, 1801	862	ICZN 1968b
Carabidae	*Glyptus* Brullé, 1835	1299	ICZN 1985a
Carabidae	*Ophonus* Dejean, 1821	1598	ICZN 1990c
Carabidae	*Tachys* Dejean, 1821	1598	ICZN 1990c
Cerambycidae	*Ceroplesis* Audinet-Serville, 1835	1407	ICZN 1986
Cerambycidae	*Phymatodes* Mulsant, 1839	1525	ICZN 1989a
Cerambycidae	*Prionus* Geoffroy, 1762	1754	ICZN 1994a
Cerambycidae	*Saperda* Fabricius, 1775	1155	ICZN 1980
Cerambycidae	*Stenocorus* Geoffroy, 1762	1754	ICZN 1994a
Cerambycidae	*Tetropium* Kirby, 1837	1473	ICZN 1988b
Cerylonidae	*Cerylon* Latreille, 1802	1811	ICZN 1995c
Chrysomelidae	*Altica* Geoffroy, 1762	1754	ICZN 1994a
Chrysomelidae	*Bruchus* Linnaeus, 1767	1809	ICZN 1995a
Chrysomelidae	*Cassida* Linnaeus, 1758	1023	ICZN 1974b
Chrysomelidae	Chrysolina Motschulsky, 1860	1279	ICZN 1984c
Chrysomelidae	*Chrysomela* Linnaeus, 1758	1279	ICZN 1984c
Chrysomelidae	*Crioceris* Geoffroy, 1762	908, 1754	ICZN 1970a, 1994a
Chrysomelidae	*Cryptocephalus* Geoffroy, 1762	1754	ICZN 1994a
Chrysomelidae	*Galeruca* Geoffroy, 1762	1754	ICZN 1994a
Chrysomelidae	*Lema* Fabricius, 1798	908	ICZN 1970a
Chrysomelidae	*Luperus* Geoffroy, 1762	1273	ICZN 1984a
Chrysomelidae	*Uroplata* Chevrolat, 1835	1359	ICZN 1985
Cleridae	*Clerus* Geoffroy, 1762	1273	ICZN 1984a
Cleridae	*Korynetes* Herbst, (1792)	604	ICZN 1961c
Cleridae	*Necrobia* Olivier, 1795	604	ICZN 1961c
Cleridae	*Notoxus* Geoffroy, 1762	1754	ICZN 1994a
Cleridae	*Trichodes* Herbst, 1792	1273	ICZN 1984a
Coprinisphaeridae	*Coprinisphaera* Sauer, 1955	2211	ICZN 2008b
Cryptophagidae	*Cryptophagus* Herbst, 1792	1810	ICZN 1995b
Cucujidae	*Cucujus* Fabricius, 1775	1754	ICZN 1994a
Curculionidae	*Brachyderes* Schönherr, 1823	1440	ICZN 1987
Curculionidae	*Ceutorhynchus* Germar, 1824	1529	ICZN 1989c
Curculionidae	*Cholus* Germar, 1824	1449	ICZN 1987d
Curculionidae	*Coeliodes* Schönherr, 1837	1529	ICZN 1989c
Curculionidae	*Cryptorhynchus* Illiger, 1807	808	ICZN 1967b
Curculionidae	*Dendroctonus* Erichson, 1836	670	ICZN 1963a
Curculionidae	*Dryocoetes* Eichhoff, 1864	1145	ICZN 1979b
Curculionidae	*Eurhinus* Illiger, 1807	1352	ICZN 1985
Curculionidae	*Geonemus* Schönherr, 1833	1493	ICZN 1988d
Curculionidae	*Magdalis* Germar, 1817	345	ICZN 1955a
Curculionidae	*Mononychus* Germar, 1824	1529	ICZN 1989c
Curculionidae	*Otiorhynchus* Germar, 1824	982	ICZN 1972
Curculionidae	*Pachyrhynchus* Germar, 1824	928	ICZN 1970b
Curculionidae	*Phaenomerus* Schönherr, 1836	621	ICZN 1962
Curculionidae	*Phloeosinus* Chapuis, 1869	1167	ICZN 1981a
Curculionidae	*Phloeotribus* Latreille, 1796	1144	ICZN 1979a
Curculionidae	*Phyllobius* Germar, 1824	1179	ICZN 1981c
Curculionidae	*Phytobius* Schönherr, 1833	1968	ICZN 2001
Curculionidae	*Polydrusus* Germar, 1817	1179	ICZN 1981c
Curculionidae	*Ptochus* Schönherr, 1826	1616	ICZN 1990d
Curculionidae	*Rhinoncus* Schönherr, 1825	1529	ICZN 1989c
Curculionidae	*Rhyncolus* Germar, 1817	1655	ICZN 1991b
Curculionidae	*Scolytus* Geoffroy, 1762	683	ICZN 1963b
Curculionidae	*Somatodes* Schönherr, 1840	1770	ICZN 1994c
Curculionidae	*Tomicus* Latreille, [1802]	670	ICZN 1963a
Curculionidae	*Tropiphorus* Schönherr, 1842	1474	ICZN 1988c
Curculionidae	*Trypetes* Schönherr, 1836	1005	ICZN 1974a
Curculionidae	*Xyleborus* Eichhoff, 1864	848	ICZN 1968a
Curculionidae	*Zygops* Schönherr, 1825	1450	ICZN 1987e
Cyclaxyridae	*Cyclaxyra* Broun, 1893	1472	ICZN 1988a
Dermestidae	*Anthrenus* Geoffroy, 1762	1754	ICZN 1994a
Dryophthoridae	*Dryophthorus* Germar, 1824	1448	ICZN 1987c
Dryophthoridae	*Rhinostomus* Rafinesque, 1815	345	ICZN 1955a
Dryophthoridae	*Sitophilus* Schönherr, 1838	572	ICZN 1959b
Dryophthoridae	*Sphenophorus* Schönherr, 1838	572	ICZN 1959b
Dytiscidae	*Acilius* Leach, 1817	619	ICZN 1961e
Dytiscidae	*Dytiscus* Linnaeus, 1758	619	ICZN 1961e
Dytiscidae	*Graphoderus* Dejean, 1833	618	ICZN 1961d
Dytiscidae	*Vatellus* Aubé, [1837]	1681	ICZN 1992
Elateridae	*Drasterius* Eschscholtz, 1829	1441	ICZN 1987b
Elmidae	*Elmis* Latreille, 1802	1812	ICZN 1995d
Elmidae	*Lara* Le Conte, 1852	1515	ICZN 1988g
Erotylidae	*Tritoma* Fabricius, 1775	1754	ICZN 1994a
Geotrupidae	*Athyreus* MacLeay, 1819	1299	ICZN 1985a
Geotrupidae	*Bolboceras* Kirby, 1819	2138	ICZN 2006a
Geotrupidae	*Geotrupes* Latreille, 1796	346	ICZN 1955b
Geotrupidae	*Odonteus* Samouelle, 1819	2138	ICZN 2006a
Gyrinidae	*Enhydrus* Laporte, 1834	710	ICZN 1964
Gyrinidae	*Gyrinus* Geoffroy, 1762	1754	ICZN 1994a
Hydraenidae	*Ochthebius* Leach, 1815	1631	ICZN 1991a
Hydrophilidae	*Berosus* Leach, 1817	1577	ICZN 1990a
Hydrophilidae	*Georissus* Latreille, 1809	1891	ICZN 1998
Hydrophilidae	*Helochares* Mulsant, 1844	710	ICZN 1964
Hydrophilidae	*Helophorus* Fabricius, 1775	1724	ICZN 1993b
Hydrophilidae	*Hydrobius* Leach, 1815	1577	ICZN 1990a
Hydrophilidae	*Hydrophilus* Geoffroy, 1762	1754	ICZN 1994a
Hydrophilidae	*Megasternum* Mulsant, 1844	1178	ICZN 1981b
Hygrobiidae	*Hygrobia* Latreille, 1804	280	ICZN 1954
Kateretidae	*Brachypterus* Kugelann, 1794	1916	ICZN 1999a
Kateretidae	*Kateretes* Herbst, 1793	1916	ICZN 1999a
Lampyridae	*Lampyris* Geoffroy, 1762	1273	ICZN 1984a
Leiodidae	*Colon* Herbst, 1797	1810	ICZN 1995b
Lucanidae	*Platycerus* Geoffroy, 1762	1754	ICZN 1994a
Megalopodidae	*Zeugophora* Kunze, 1818	1382	ICZN 1986
Meloidae	*Cerocoma* Geoffroy, 1762	1754	ICZN 1994a
Meloidae	*Horia* Fabricius, 1787	1918	ICZN 1999b
Meloidae	*Meloe* Linnaeus, 1758	1918	ICZN 1999b
Meloidae	*Mylabris* Fabricius, 1775	1809	ICZN 1995a
Meloidae	*Nemognatha* Illiger, 1807	1918	ICZN 1999b
Meloidae	*Zonitis* Fabricius, 1775	1918	ICZN 1999b
Monotomidae	*Rhizophagus* Herbst, 1793	1810	ICZN 1995b
Nemonychidae	*Cimberis* Gozis, 1881	2111	ICZN 2005c
Nemonychidae	*Nemonyx* Redtenbacher, 1845	2111	ICZN 2005c
Oedemeridae	*Oncomera* Stephens, 1829	1506	ICZN 1988f
Omalisidae	*Omalisus* Geoffroy, 1762	1754	ICZN 1994a
Orsodacnidae	*Orsodacne* Latreille, 1802	1989	ICZN 2002a
Ptiliidae	*Nephanes* Thomson, 1859	1307	ICZN 1985b
Ptiliidae	*Ptenidium* Erichson, 1845	1277	ICZN 1984b
Ptiliidae	*Ptilium* Gyllenhal, 1827	1277	ICZN 1984b
Ptiliidae	*Ptinella* Motschulsky, 1844	1307	ICZN 1985b
Ptinidae	*Anobium* Fabricius, 1775	1062	ICZN 1976
Ptinidae	*Dorcatoma* Herbst, 1792	1810	ICZN 1995b
Ptinidae	*Ptilinus* Geoffroy, 1762	1754	ICZN 1994a
Ptinidae	*Ptinus* Linnaeus, 1767	1809	ICZN 1995a
Ptinidae	*Xyletinus* Latreille, 1809	966	ICZN 1971
Pyrochroidae	*Pyrochroa* Geoffroy, 1762	1754	ICZN 1994a
Scarabaeidae	*Ataenius* Harold, 1867	2241	ICZN 2010a
Scarabaeidae	*Anomala* Samouelle, 1819	1546	ICZN 1989d
Scarabaeidae	*Bothynus* Hope, 1837	2199	ICZN 2008a
Scarabaeidae	*Copris* Geoffroy, 1762	1754	ICZN 1994a
Scarabaeidae	*Gymnetis* MacLeay, 1819	806	ICZN 1967a
Scarabaeidae	*Melolontha* Fabricius, 1775	1754	ICZN 1994a
Scarabaeidae	*Osmoderma* Lepeletier & Serville, 1828	2186	ICZN 2007
Scarabaeidae	*Pelidnota* MacLeay, 1819	2054	ICZN 2003b
Scarabaeidae	*Pentodon* Hope, 1837	2054	ICZN 2003b
Scarabaeidae	*Phaenomeris* Hope, 1833	621	ICZN 1962
Scarabaeidae	*Podalgus* Burmeister, 1847	2066	ICZN 2004a
Scarabaeidae	*Stethaspis* Hope, 1837	1244	ICZN 1983b
Scarabaeidae	*Trichius* Fabricius, 1775	2079	ICZN 2004c
Scarabaeidae	*Trigonostomus* Brenske, 1893	2229	ICZN 2009b
Schizopodidae	*Schizopus* LeConte, 1858	1727	ICZN 1993d
Scraptiidae	*Anaspis* Geoffroy, 1762	1273	ICZN 1984a
Sphaeriusidae	*Microsporus* Kolenati, 1846	1331	ICZN 1985d
Sphaeriusidae	*Sphaerius* Waltl, 1838	1331, 1957	ICZN 1985d, 2000
Sphindidae	*Aspidiphorus* Ziegler in Dejean, 1821	1862	ICZN 1997
Sphindidae	*Sphindus* Megerle in Dejean, 1821	1862	ICZN 1997
Staphylinidae	*Tachinus* Gravenhorst, 1802	1743	ICZN 1993f
Staphylinidae	*Acrotona* Thomson, 1859	600	ICZN 1961b
Staphylinidae	*Agrodes* Nordmann, 1837	1851	ICZN 1996c
Staphylinidae	*Anthophagus* Gravenhorst, 1802	2084	ICZN 2004d
Staphylinidae	*Atheta* Thomson, 1858	600	ICZN 1961b
Staphylinidae	*Baeocera* Erichson, 1845	1221	ICZN 1982
Staphylinidae	*Bolitochara* Mannerheim, 1831	599	ICZN 1961a
Staphylinidae	*Bryaxis* Kugelann, 1794	887	ICZN 1969b
Staphylinidae	*Bythinus* Leach, 1817	887	ICZN 1969b
Staphylinidae	*Coprophilus* Latreille, 1829	1722	ICZN 1993a
Staphylinidae	*Coryphium* Stephens, 1834	1597	ICZN 1990b
Staphylinidae	*Creophilus* Samouelle, 1819	546	ICZN 1959a
Staphylinidae	*Geostiba* Thomson, 1858	2098	ICZN 2005a
Staphylinidae	*Gyrohypnus* Samouelle, 1819	1250	ICZN 1983c
Staphylinidae	*Ischnopoda* Stephens, 1835	600	ICZN 1961b
Staphylinidae	*Leptusa* Kraatz, 1856	2113	ICZN 2005d
Staphylinidae	*Lesteva* Latreille, 1797	2084	ICZN 2004d
Staphylinidae	*Metopias* Gory, 1832	1772	ICZN 1994d
Staphylinidae	*Mycetoporus* Mannerheim, 1831	1726	ICZN 1993c
Staphylinidae	*Othius* Stephens, 1829	1250	ICZN 1983c
Staphylinidae	*Oxypoda* Mannerheim, 1831	463	ICZN 1957
Staphylinidae	*Platycnemus* Nordmann, 1837	1851	ICZN 1996c
Staphylinidae	*Proteinus* Latreille, 1796	876	ICZN 1969a
Staphylinidae	*Quedius* Stephens, 1829	1851	ICZN 1996c
Staphylinidae	*Staphylinus* Linnaeus, 1758	546	ICZN 1959a
Staphylinidae	*Tachyporus* Gravenhorst, 1802	1743	ICZN 1993f
Staphylinidae	*Tachyusa* Erichson, 1837	600	ICZN 1961b
Staphylinidae	*Xantholinus* Dejean, 1821	1250	ICZN 1983c
Staphylinidae	*Zyras* Stephens, 1835	599	ICZN 1961a
Tenebrionidae	*Alphitobius* Stephens, 1829	1039	ICZN 1975
Tenebrionidae	*Diaperis* Geoffroy, 1762	1754	ICZN 1994a
Tenebrionidae	*Gnatocerus* Thunberg, 1814	1039	ICZN 1975
Tenebrionidae	*Helops* Fabricius, 1775	2237	ICZN 2009d
Tenebrionidae	*Phaleria* Latreille, [1802]	1039	ICZN 1975
Tenebrionidae	*Platyscelis* Latreille, 1818	1729	ICZN 1993e
Tenebrionidae	*Tentyria* Latreille, 1802	2244	ICZN 2010c
Tenebrionidae	*Tribolium* MacLeay, 1825	1495	ICZN 1988e
Tenebrionidae	*Uloma* Dejean, 1821	1039	ICZN 1975
Trogossitidae	*Peltis* Kugelann, 1792	1754	ICZN 1994a
Zopheridae	*Colydium* Fabricius, 1792	1811	ICZN 1995c
Zopheridae	*Orthocerus* Latreille, 1796	1811	ICZN 1995c
Zopheridae	*Rhopalocerus* W. Redtenbacher, 1842	1397	ICZN 1986

## Appendix 6

Summary of cases involving Coleoptera family-group names and/or their type genera awaiting a ruling by the Commission. Cases listed in alphabetical order by family.

### Carabidae

Engel and Bouchard (2009: Case 3484) reported that the Coleoptera name Nomiidae Gozis, 1875 and the Hymenoptera name Nomiidae Robertson, 1904 are homonyms based on similar but not identical type genera. Since the Hymenoptera name is currently used as valid and the Coleoptera name is not, the Commission was asked to emend the latter to Nomiusidae Gozis, 1875.

### Chaetosomatidae

Bousquet and Bouchard (2010) submitted an application to the Commission (Case 3513) to conserve usage of Chaetosomatidae Crowson, 1952 over Chaetosomatidae Claus, 1872 [Nematoda]. In addition, the Commission was asked to conserve usage of *Chaetosoma* Westwood, 1851 and *Apodasya* Pascoe, 1863, both threatened by the discovery of the older name *Chaetosoma* Chevrolat, 1843.

### Chrysomelidae

An application to conserve *Eupales* Lefèvre, 1885 and Eupalini Verma et al. 2005 was recently submitted by Jolivet and Verma (Bulletin of Zoological Nomenclature 2009: 204; see notice of “New applications to the Commission”). This Case (number 3498) has not been published yet.

Moseyko et al. (2010) submitted an application to the Commission (Case 3519) to conserve usage of *Eumolpus* Weber, 1801 and *Bromius* Chevrolat, 1836.

### Curculionidae

An application to conserve usage of Plinthini Lacordaire, 1863 and to designate *Plinthus* Germar, 1817 as its type genus was recently submitted by Alonso-Zarazaga and Lyal (2010: Case 3530).

An application to emend the entries for *Otiorhynchus* and *Loborhynchus* in the Official List of Generic Names in Zoology was recently submitted to the Commission by Lyal and Alonso-Zarazaga (2010: Case 3529).

### Eucnemidae

Muona (1994; see notice of “New applications to the Commission”) submitted an application to conserve usage of Eucnemidae Eschscholtz, 1829 over Melasidae Fleming, 1821. This Case (number 2938) was not subsequently published in the Bulletin of Zoological Nomenclatureand is apparently not closed (ICZN secretariat pers. comm. 2010). The status of this application is still uncertain.

### Gyrinidae

The name Enhydrini Régimbart, 1882 is a junior homonym of the mammalian name Enhydrini Gray, 1825. An application was recently submitted to the Commission by Özdikmen and Darilmaz (2010: Case 3514) to remove the homonymy by changing the stem of the beetle name to Enhydrus-.

### Lampyridae

Svenson and Branham (2007: Case 3402) submitted an application to remove the homonymy between Photinini LeConte, 1881 and Photininae Giglio-Tos, 1915 [Mantodea]. Both family-group names are correctly formed and are based on similar but not identical type genera. The Commission was asked to emend the Mantodea name to Photinaini Giglio-Tos, 1915.

### Latridiidae

Bousquet et al. (2010) submitted an application to the Commission (Case 3517) to give precedence to Latridiidae Erichson, 1842 over Corticariidae Curtis, 1829 whenever their type genera are placed in the same family-group taxon and to conserve the current usage of *Corticaria* Marsham, threatened by the discovery of an overlooked type species designation.

### Staphylinidae

An application to conserve Athetini Casey, 1910 and Athetina Casey, 2010 was recently submitted by Gusarov (Bulletin of Zoological Nomenclature 2010: 270; see notice of “New applications to the Commission”). This Case (number 3537) has not been published yet.
